# Ferroptosis-associated molecular classification characterized by distinct tumor microenvironment profiles in colorectal cancer

**DOI:** 10.7150/ijbs.69808

**Published:** 2022-02-07

**Authors:** Wenqin Luo, Weixing Dai, Qingguo Li, Shaobo Mo, Lingyu Han, Xiuying Xiao, Ruiqi Gu, Wenqiang Xiang, Li Ye, Renjie Wang, Ye Xu, Sanjun Cai, Guoxiang Cai

**Affiliations:** 1 Department of Colorectal Surgery, Fudan University Shanghai Cancer Center, Shanghai, China.; 2 Department of Oncology, Shanghai Medical College, Fudan University, Shanghai, China.; 3 State Key Laboratory of Oncogenes and Related Genes, Shanghai Cancer Institute, Department of Oncology, Renji Hospital, School of Medicine, Shanghai Jiao Tong University, Shanghai, China.

**Keywords:** Colorectal cancer, Ferroptosis, Tumor microenvironment, Immune activation, Stromal cells infiltration, Myofibroblast, Immunotherapy.

## Abstract

Ferroptosis is a non-apoptotic form of cell death recognized in recent years. Nonetheless, the potential role of ferroptosis-associated genes in immune regulation and tumor microenvironment formation remains unknown. In this study, we characterized the ferroptosis-associated patterns of colorectal cancer through integrative analyses of multiple datasets with transcriptomics, genomics, and single-cell transcriptome profiling. Three distinct ferroptosis-associated clusters (FAC1, FAC2 and FAC3) were identified from 1251 CRC bulk samples, which were associated with different clinical outcomes and biological pathways. The TME characterization revealed that the three patterns were highly consistent with known immune profiles: immune-desert (FAC1), immune-inflamed (FAC2) and immune-excluded (FAC3), respectively. Ferroptosis-associated immune and stromal-activated genes were obtained and characterized by corresponding function in CRC tumorigenesis. Further single-cell analyses identified the ferroptosis-associated immune responding tumor cells and ferroptosis-associated stromal cells infiltration pattern. Based on the Fersig score, which was extracted from the ferroptosis phenotype-related signature, patients with lower Fersig score were characterized by prolonged survival time and effective immune responses. Collectively, we uncovered the ferroptosis-associated patterns associated with TME diversity and immune response phenotype. The Fersig we constructed could be the potential therapeutic target genes to improve the efficacy of patients' immunotherapy. The Fersig scoring scheme could enhance the understanding of TME infiltration associated with ferroptosis and prediction of immunotherapy efficacy.

## Introduction

Colorectal cancer (CRC) is one of the most common malignancies worldwide, which is the main cause of cancer death 1. Once diagnosed with CRC at an advanced stage, patients would have extremely poor prognosis 2. Thus, more valuable prognostic markers and potential therapeutic targets are required to help patients benefit from more aggressive therapy.

Ferroptosis is an iron- and lipid reactive oxygen species (ROS)-dependent form of programmed cell death 3, 4, which is involved in multiple diseases, including neurological disorders and diverse cancers. Despite recent advances in cancer therapeutics, some patients remain resistant to therapy because of anti-apoptosis. Thus, the induction of ferroptosis may be an effective intervention strategy to the treatment of cancers 5-7. Recently, various molecules have been implicated in ferroptosis during pathogenesis of CRC. RSL3 inhibited GPX4 to induce ferroptosis in CRC 8. Knockout of SLC7A11 induced ferroptotic cell death to reduce chemotherapy resistance 9. Chen et al. reported that combination of β-elemene, a ferroptosis inducer, with anti-EGFR treatment could trigger ferroptosis to produce anti-tumor effects in CRC patients with RAS mutation 10, 11. These studies highlighted the promising strategy of inducing ferroptosis in cancer therapy. However, there is a lack of comprehensive understanding of ferroptosis-associated genes in pathogenesis of CRC. In this study, we aimed to integrate transcriptomics and genomic data for systematical analysis of ferroptosis-associated genes to enhance our understanding of ferroptosis in CRC tumorigenesis.

Increasing evidence indicated that the tumor microenvironment (TME) contexture (including infiltrating immune cells, stromal cells, and secreted cytokines et al) played an important role in the tumorigenesis 12, 13. In CRC, disease relapse and mortality could be predicted by the enrichment level of lymphocyte populations (cytotoxic CD8^+^ T cells) at the center or margin of tumor 14. Recently, immunotherapy has been recognized as a revolution in cancer treatments, which can modulate immune responses against tumors. Immune-checkpoint blockade (ICB) therapy, represented by CTLA-4 and PD-1, has achieved a marked durable response in CRC patients 15. In CRC, deficient mismatch repair or high microsatellite instability (dMMR/MSI-H) is a well-established biomarker for the potential response to ICB immunotherapy 16. However, only a small proportion of dMMR/MSI-H patients showed positive responses to immunotherapy, thus there is an urgent clinical need to find more precise and reliable biomarkers to identify patients who may benefit from immunotherapy, and monitor their therapeutic responses 17. Given that there are classes of TME contexture associated with ICB responsiveness, comprehensively understanding the microenvironment infiltration patterns can facilitate the management of ICB therapy.

Recent studies revealed the correlation between TME cells infiltration and ferroptosis. For example, M2-polarized tumor-associated macrophages (TAMs) can act as “iron-donators” to promote cancer progression 18. Ferroportin (FPN-1), as an iron exporter, can reduce the cellular iron of M2-polarized TAMs, thereby providing iron for tumor cells 19. Wang's group demonstrated that the treatment of PD-L1 inhibitors could increase lipid ROS in CD45-IDB cells and suppress tumor progression 20. Further understanding the interactions between TME cells infiltration and ferroptosis can provide new angles for cancer therapy, such as ferroptosis-based immunotherapy 21.

In this study, we integrated TCGA (COREAD), GEO (GSE14333 22, GSE37892 23 and GSE39582 24), and single-cell RNA (scRNA) sequencing (GSE144735 25) databases to evaluate the association between ferroptosis patterns and TME cell-infiltrating characteristics. We identified three distinct patterns using unsupervised clustering, which were closely linked to three previously reported immune phenotypes: immune-inflamed, immune-excluded, and immune-desert 26. We also identified the concrete genes associated with different TME infiltration phenotypes, quantified the ferroptosis patterns of individual tumors, and predicted their correlation with the response to immunotherapy. These findings will help understanding ferroptosis in shaping diverse TME profiles and provide new angles for the therapeutic intervention in CRC.

## Results

### Ferroptosis-associated molecular classification in colorectal cancer

**Figure 1:**
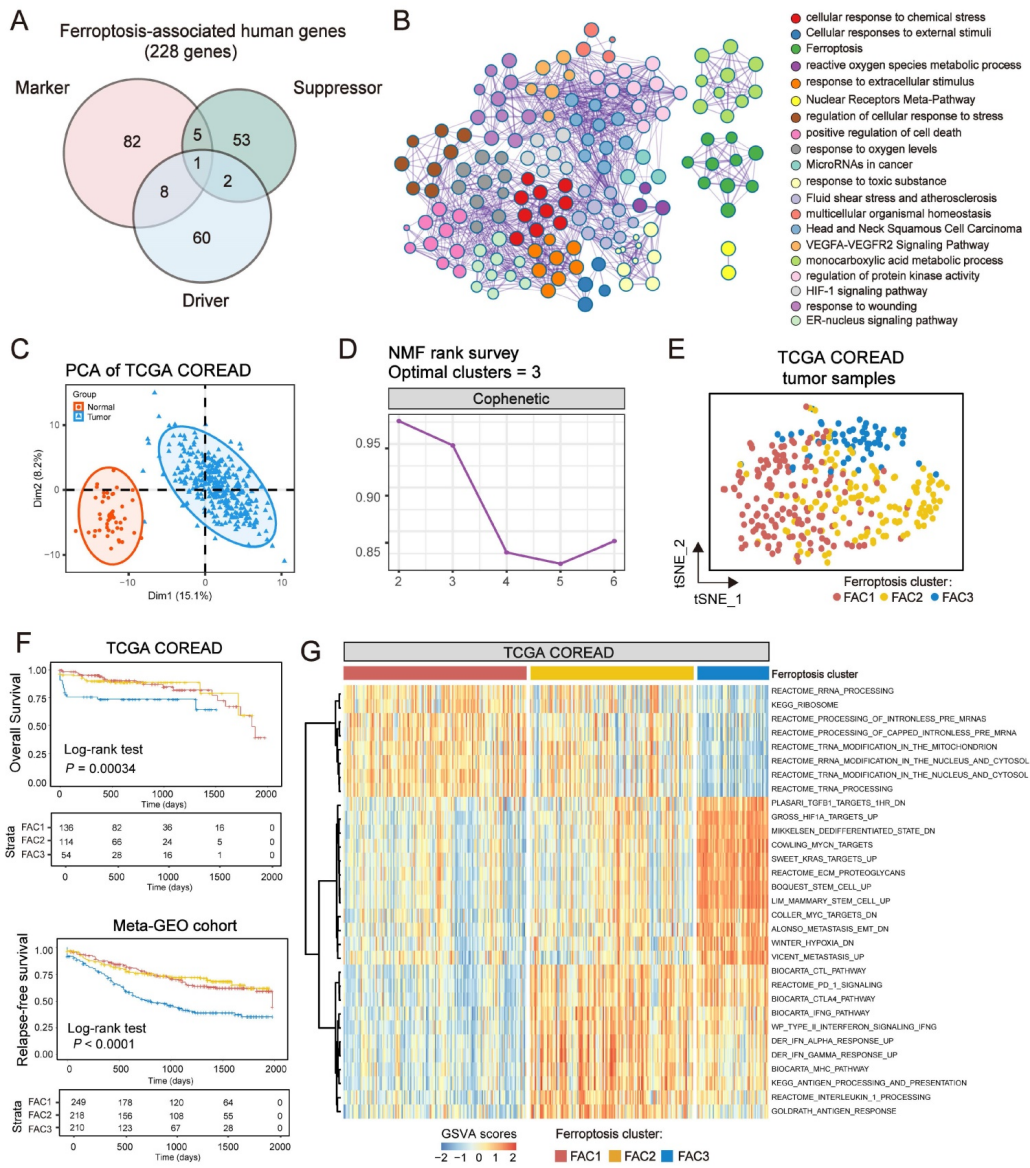
** Ferroptosis-associated molecular classification in colorectal cancer. (A)** Venn diagram shows three subtypes of ferroptosis-associated human genes, including 71 drivers, 61 suppressors and 96 markers.** (B)** Metascape enrichment network visualization shows the clusters of enriched GO terms of ferroptosis-associated genes. Cluster annotations are shown in the color code.** (C)** Principal component analysis of ferroptosis-associated genes to distinguish tumors from normal patients. All samples: n=382; tumor: n=338; normal: n=44. **(D)** NMF rank survey was shown. The optimal number of clusters: rank=3. **(E)** t-SNE visualization of three NMF clusters in tumors of TCGA-COREAD cohort, n=338. Dots are colored according to clusters. **(F)** Kaplan-Meier curves for overall survival of three NMF clusters in TCGA and for relapse-free survival of three clusters in meta-GEO cohort. P value was determined by the log-rank test. Patients' survival days within 2000 days are shown. **(G)** Pathway activities of three clusters using GSVA analyses.

The analytical process in this study is illustrated in Figure S1. We investigated the roles of 259 ferroptosis-associated genes derived from FerrDb database 27 in CRC, including 108 drivers, 69 suppressors and 111 markers. We retained totally 228 human genes for analyses (Figure 1A). As expected, gene ontology (GO) enrichment analyses showed that these genes were characterized by the biological processes of ferroptosis and cell death (Figure 1B). Principal component analysis (PCA) (Figure 1C) and hierarchical clustering of gene expression (Figure S2C) based on paired tumor-normal samples showed that ferroptosis-associated genes could distinguish CRC samples from normal samples, suggesting that ferroptosis might play a significant role in regulating CRC tumorigenesis. We next utilized consensus clustering analysis of the non-negative matrix factorization (NMF) algorithm^28^ to stratify tumor samples based on the expression of the 228 genes (Figure S2A). Accordingly, we identified three distinct clusters and referred to these clusters as ferroptosis-associated clusters (FAC), including 148 cases in FAC1, 132 cases in FAC2 and 58 cases in FAC3 (Figure 1D-F, Figure S2B, Table S1), among which FAC3 had the worst prognosis in TCGA-COREAD cohort (Figure 1F, overall survival (OS) within 2000 days, P = 0.00034; Figure S2B, overall survival (OS) of all samples, P = 0.0092, log-rank test). In addition, we combined three GEO datasets with available clinical data (GSE39582, GSE14333 and GSE37892) into a meta-GEO cohort and obtained similar results of classification and prognosis (Figure 1F, relapse-free survival (RFS) within 2000 days, P < 0.0001; Figure S2B, relapse-free survival (RFS) of all samples, P < 0.0001, log-rank test).

To understand the biological discrepancy among three distinct clusters, we performed gene set variation analysis (GSVA) 29 on tumor samples of TCGA (Figure 1G). The results showed that FAC1 was enriched in pathways related to RNA modification and processing. Interestingly, FAC2 was remarkably enriched in immune activation and immunotherapy-related pathways, such as antigen response, interferon gamma/alpha response, PD-1 and CTLA4 signaling. However, FAC3 presented enrichment pathways mainly correlated with tumor-specific and stromal pathways such as TGF-β, stem cell and epithelial-mesenchymal transition (EMT), supporting its worse prognosis.

**Figure 2:**
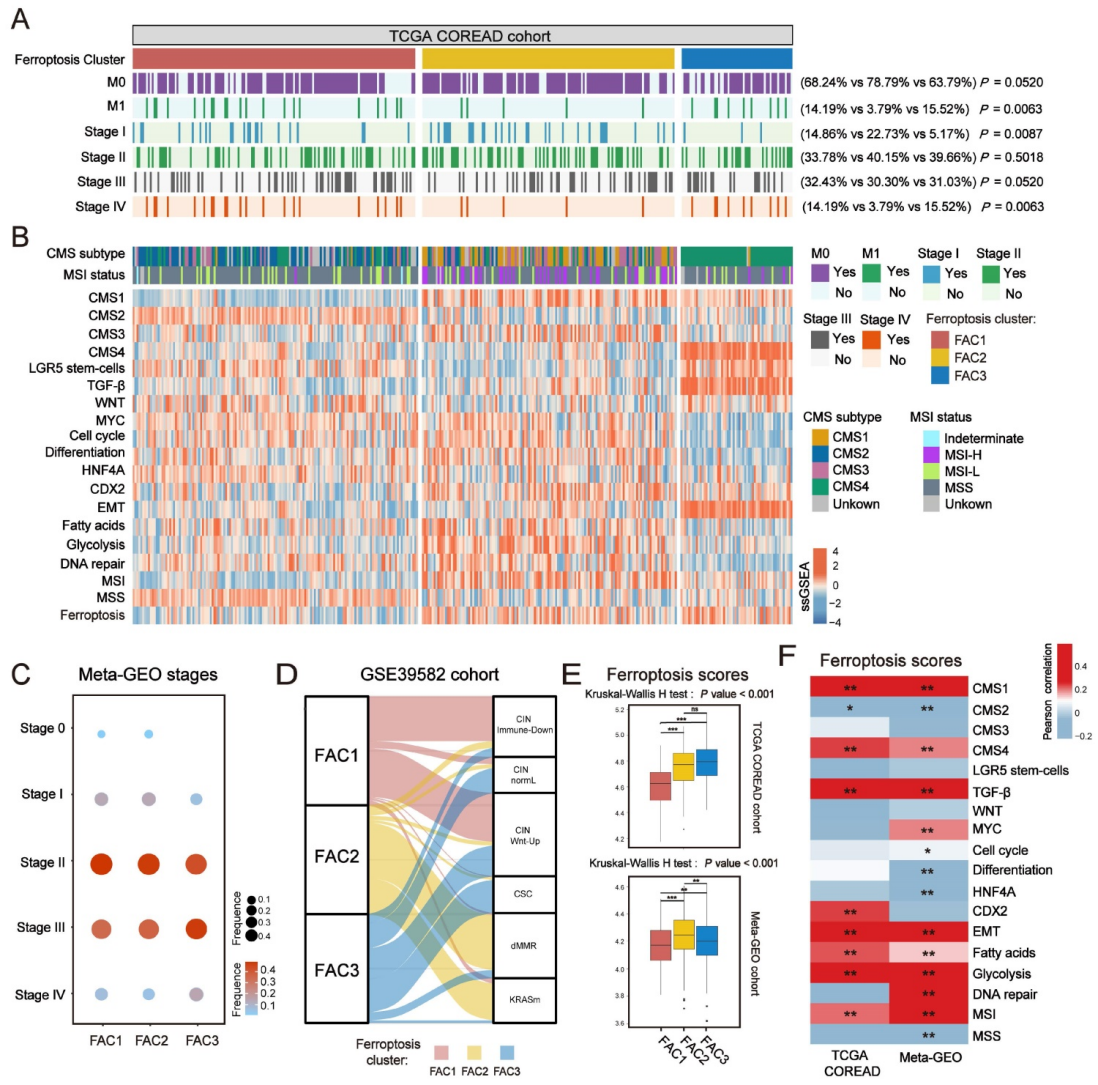
** Clinical characteristics and biological molecular changes underlying three clusters in CRC. (A)** Heatmap shows clinical characteristics of NMF clusters in TCGA cohort. P value was determined by Pearson's chi-square test. **(B)** Heatmap shows molecular characteristics of NMF clusters in TCGA cohort. **(C)** Dot plot shows the proportions of different stages among NMF clusters of meta-GEO cohort. Both color and size represent the fraction of stages in different clusters.** (D)** Alluvial diagram of clusters in groups with different molecular subtypes (CIN_Immune-Down_, CIN_WntUp_, CIN_normaL_, CSC, dMMR, and KRASm). **(E)** Barplot shows ssGSEA score of ferroptosis-associated genes in the NMF clusters. The statistical difference of three clusters was compared by the Kruskal-Wallis H test. *P < 0.05; **P < 0.01; ***P < 0.001. The difference of two clusters was compared by the wilcox test. *P < 0.05; **P < 0.01; ***P < 0.001. **(F)** Correlations between ferroptosis-associated ssGSEA score and biological gene signatures correlated with CMS subtypes using Pearson analysis.

In terms of clinical characteristics (Figure 2A), most patients in FAC3 had advanced stage tumor (M1 and stage IV) (15.52% in FAC-3 patients, *P* = 0.0063, Pearson's chi-square test), only few patients were with early-stage tumor (stage I) (5.17% in FAC3, *P* = 0.0087, Pearson's chi-square test). The lowest proportion of advanced stage tumors (M1 and stage IV) (3.79% in FAC2 patients, *P* = 0.0063, Pearson's chi-square test), but highest proportion of early-stage tumors (stage I) (22.73% in FAC2 patients, *P* = 0.0087, Pearson's chi-square test) were observed in FAC2 samples. Similar results in meta-GEO cohort indicated that the less patients with early-stage CRC (Stage 0-I) were in FAC1 and FAC2, the more patients with advanced stage (Stage IV) would be in FAC3 (Figure 2C).

### Biological molecular changes underlying different clusters in CRC

To further investigate molecular changes underlying these clusters, we utilized a consensus molecular subtypes (CMS) classifier, referred to as CMScaller^30^, to stratify TCGA tumor samples. We found that nearly all patients in FAC3 were enriched in CMS4 subtype, while FAC2 contained most of the CMS1 patients (Figure 2B). For microsatellite instability (MSI) status, patients in FAC2 encompassed the majority of MSI-H tumors, reminiscent of CMS1 characteristics 31. We next applied single-sample gene set enrichment (ssGSEA) analysis 29 using signatures extracted from CMScaller package (Figure 2B and Figure S3A-B). The results showed that FAC3 presented high enrichment of gene signatures associated with CMS4-mesenchymal, such as LGR5^+^ stem cells, TGF-β signaling, and EMT process. Moreover, we found that CMS3-metabolic characteristics such as fatty acids and glycolysis signature were enriched in FAC2, conforming to the biological features of ferroptosis 32. Patients in FAC1 were mainly enriched in CMS2-canonical characteristics, marked by WNT signaling activation. We next turned our attention to GSE39582 dataset with six molecular subtypes (CIN_Immune-Down_, CIN_WntUp_, CIN_normaL_, CSC, dMMR, and KRASm) 24. In this dataset, dMMR tumor was associated with immune activation. CIN_WntUp_ tumor was associated with upregulation of WNT signaling. CIN_normal_ and CSC were associated with EMT, stem cells and stromal pathways. Consistent with previous findings, patients of CSC and CIN_normal_ subtype were mainly clustered into FAC3. CIN_WntUp_ and CIN_Immune-Down_ subtypes were predominantly clustered into FAC1. dMMR subtype was concentrated within FAC2 patients (Figure 2D). All these findings indicated that marked differences in the intrinsic biological underpinnings of ferroptosis-associated classification in CRC.

Given that our classification was based on the expression of ferroptosis-associated genes, we then calculated the ssGSEA score using the gene set with 228 genes, which we named as ferroptosis score. This analysis revealed that ferroptosis score was significantly upregulated in FAC2 and FAC3 (Figure 2B and 2E). Moreover, we found ferroptosis had positive correlation with both CMS1 (MSI signature included) and CMS4-characteristics (TGF-β signaling and EMT signature included) (Figure 2F and Figure S3C). Therefore, we postulated that ferroptosis-associated genes may regulate CRC tumorigenesis mainly through immune activation and stromal pathways activation.

### Single-cell transcriptome profiling of CRC cells based on ferroptosis-associated molecular classification

**Figure 3:**
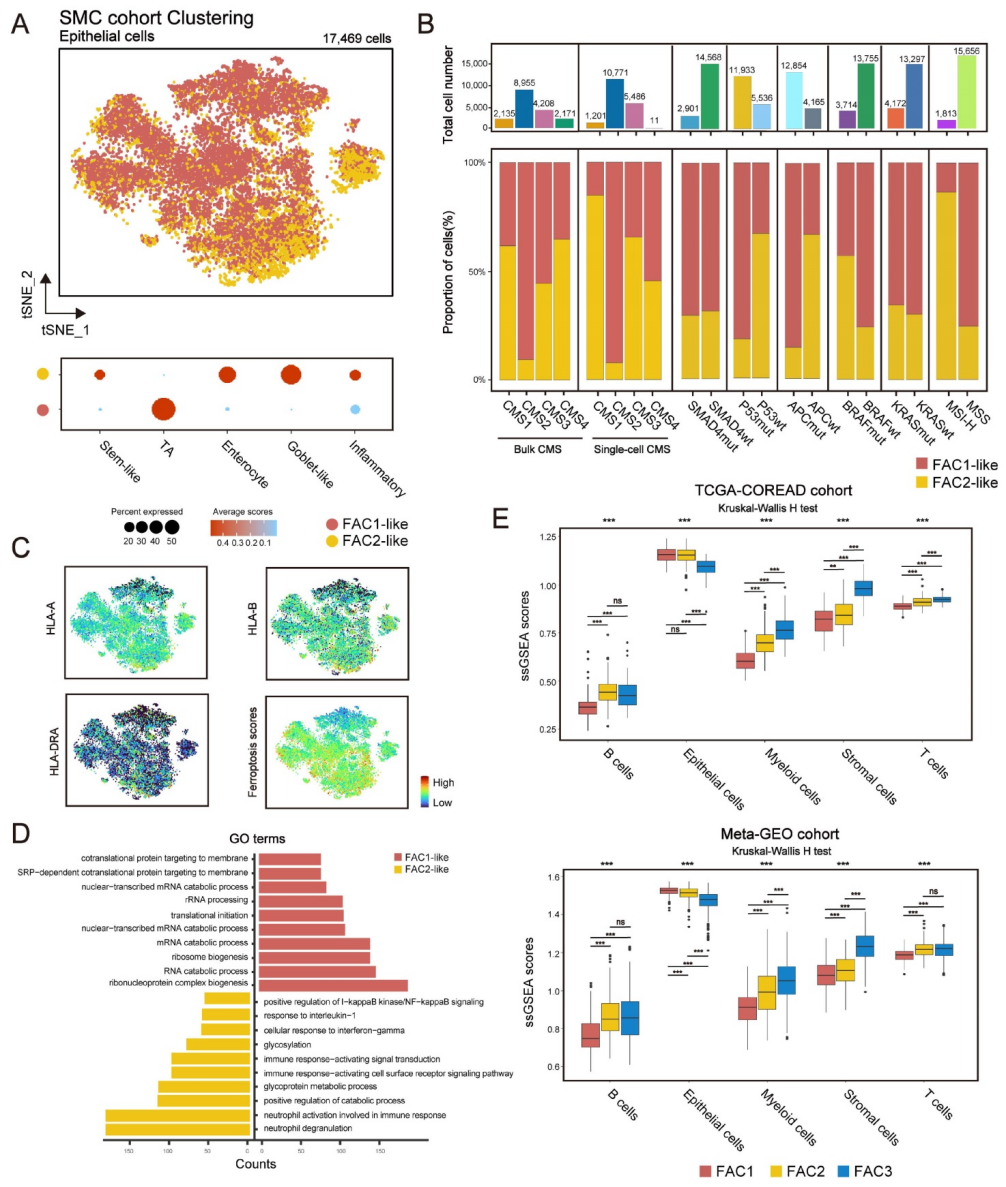
** Single-cell transcriptome profiling of CRC cells based on ferroptosis-associated molecular classification. (A)** t-SNE visualization of 17,469 tumor epithelial cells from SMC single-cell cohort (top panel). Cells are colored according to clusters. Dot plot for the score of gene signatures associated with CRC cellular phenotype and responses to therapy in each cell type. Color represents the mean score in each cell cluster, and size indicates the fraction of cells expressing gene score (bottom panel). **(B)** Barplot shows the proportion of different molecular characteristics in CRC tumor cells. Bars are colored according to clusters.** (C)** t-SNE visualization of MHC-I (HLA-A, HLA-B), MHC-II (HLA-DRA) molecular expression and the score of ferroptosis-associated genes.** (D)** GO analysis of differential expressed genes between FAC1-like and FAC2-like CRC tumor cells. **(E)** ssGSEA score of 5 cell types (epithelial cells, T cells, B cells, Myeloid cells and stromal cells) among three clusters in TCGA and meta-GEO cohorts. The statistical difference of three clusters was compared by the Kruskal-Wallis H test. *P < 0.05; **P < 0.01; ***P < 0.001. The difference of two clusters was compared by the wilcox test. *P < 0.05; **P < 0.01; ***P < 0.001.

Since bulk-tissue contains signatures of distinct cell populations, we then analyzed single-cell RNA (scRNA) dataset (GSE144735) 25 to investigate whether our classification could be precisely reflected in CRC tumor single cells. The dataset, Lee *et al*. (GSE144735), contained the SMC and KUL3 cohorts with scRNA expression data. We firstly isolated 17,469 tumor epithelial cells from the SMC cohort, and then scored them using differentially expressed genes (DEGs) of FAC1-3 bulks (Figure 3A). We found that CRC cells could only be assigned into FAC1 and FAC2-like populations, of which FAC2-like tumor cells showed upregulated inflammatory signature (Figure 3A), marked by high expression of chemokines and interferon-related genes as previously described 33. For CMS classification (Figure 3B), FAC1-like tumor cells were mainly enriched in CMS2 subtype, while FAC2-like tumor cells were predominantly enriched in CMS1 subtype through both bulk and single-cell level classification. For mutation type, FAC2-like tumor cells were mainly clustered into MSI-H and BRAF mutation groups, which has also been reported as CMS1 characteristics 34. In contrast, APC mutation, referred to as characteristics of CIN subtype (CIN_WntUp_) in GSE39582 dataset, was mainly enriched in FAC1-like tumor cells.

Considering the immune activation in FAC2 bulk samples, we checked the expression of MHC-I (HLA-A and HLA-B) and MHC-II (HLA-DRA) molecules, and found their upregulation in FAC2-like tumor cells (Figure 3C). These results indicated that FAC2-like cells displayed immune response 35, as confirmed by gene ontology (GO) analysis (Figure 3D). In contrast, FAC1-like displayed enrichment of RNA processing (Figure 3D), in line with the description in Figure 1G. Finally, examination of ferroptosis-associated gene score (Figure 3C) showed that ferroptosis had positive correlation with immune responding cells. Similar results were obtained in the KUL3 cohort (Figure S4A-D). Overall, FAC1 and FAC2-like tumor cells presented similar molecular patterns with previous analyses of TCGA and GEO cohort. Notably, FAC3-like tumor single cells were not detected in SMC and KUL3 cohorts.

Lee *et al*. has indicated that single-cell representation of CMS classification included few CMS4-like tumor cells 25. Just as shown in Figure 3B, although CMS4 bulk subtype was detected, few CMS4-like tumor cells were presented at single-cell level, even for those from CMS4 bulks. Lee *et al*. verified that remarkable stromal cells infiltration led to scarce CMS4-like tumor cells in CMS4 tissues. Based on this finding, we estimated the influences of single-cell types on our bulk classification (Figure 3E). Here we demonstrated that stromal cell types (fibroblasts and endothelial cells included) were highly enriched in FAC3 tumors, while epithelial cells were enriched in FAC1-2 tumors. Subsequent ESTIMATE algorithm 36 confirmed low tumor purity and remarkable stromal infiltration in FAC3 tumors (Figure S4E). Therefore, FAC3, marked by remarkable stromal infiltration, couldn't be reflected in tumor cells at single cell level.

### Distinct tumor microenvironment infiltration in three tumor clusters

**Figure 4:**
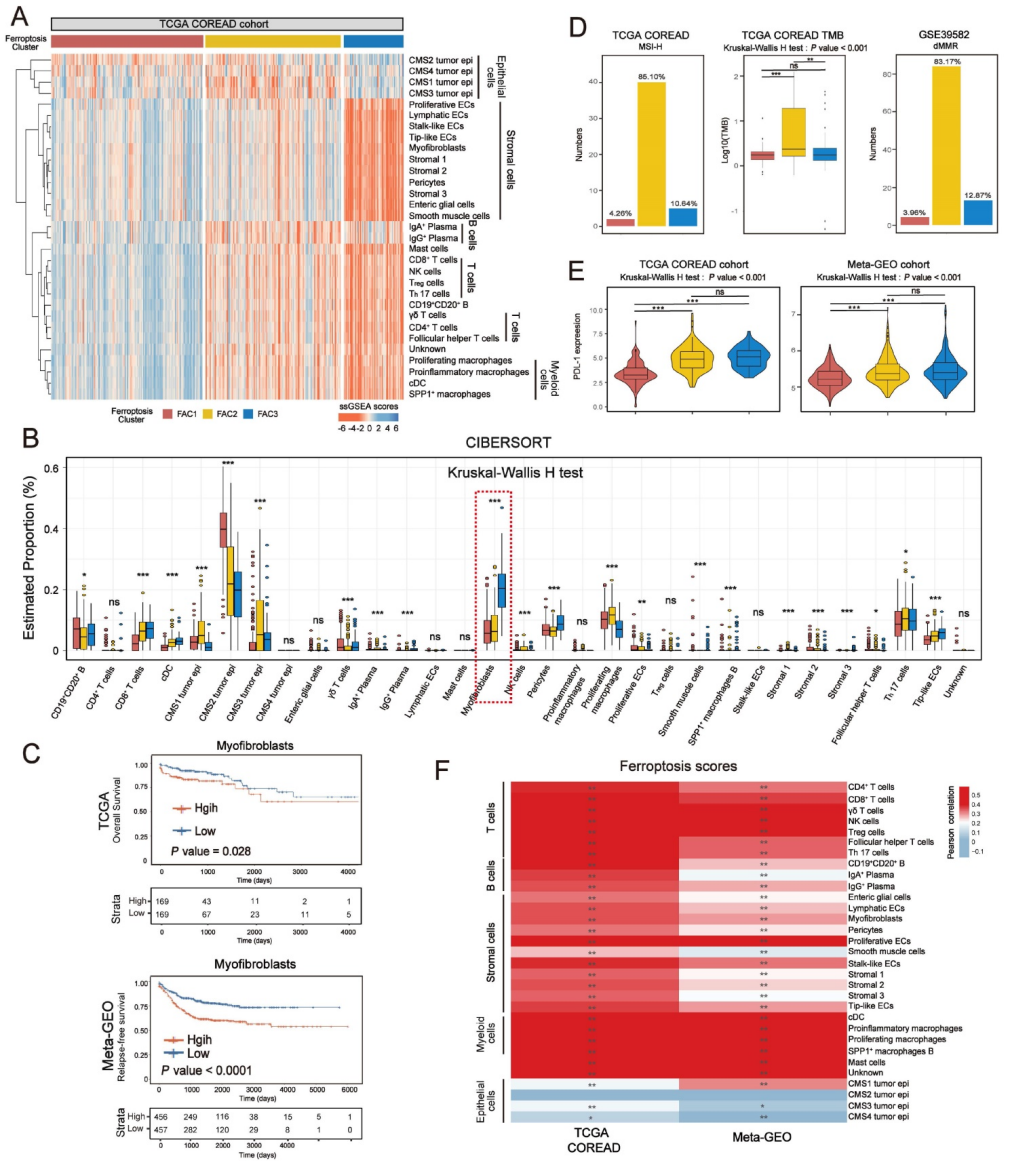
** Distinct tumor microenvironment infiltration in three tumor clusters. (A)** Heatmap shows the ssGSEA score of 31 cell subtypes in three ferroptosis-associated clusters. **(B)** Relative abundance of 31 cell subtypes of three clusters in TCGA cohort. The statistical difference of three clusters was compared by the Kruskal-Wallis H test. *P < 0.05; **P < 0.01; ***P < 0.001. **(C)** Kaplan-Meier curves for overall survival of TCGA and for relapse-free survival of meta-GEO cohort. The high and low groups were divided by the median value of the ssGSEA score of DEGs of myofibroblasts. P value was determined by the log-rank test.** (D)** Proportion of patients with MSI-H in the NMF clusters of TCGA cohort. TMB level of the NMF clusters in TCGA cohort. Proportion of patients with dMMR in the NMF clusters of meta-GEO cohort. The statistical difference of three clusters was compared by the Kruskal-Wallis H test. *P < 0.05; **P < 0.01; ***P < 0.001. The difference of two clusters was compared by the wilcox test. *P < 0.05; **P < 0.01; ***P < 0.001. **(E)** Expression of PD-L1 among three clusters of TCGA and meta-GEO cohorts. The statistical difference of three clusters was compared by the Kruskal-Wallis H test. *P < 0.05; **P < 0.01; ***P < 0.001. The difference of two clusters was compared by the wilcox test. *P < 0.05; **P < 0.01; ***P < 0.001. **(F)** Correlations between ssGSEA score of ferroptosis-associated and DEGs of 31 cell subtypes of CRC using Pearson analysis.

In Figure 3E, tumor microenvironment (TME) infiltration displayed significant differences among three ferroptosis-associated clusters. To further characterize their microenvironment heterogeneity, we performed ssGSEA and CIBERSORT analyses using 31 cell types detected in single-cell research 25 (Figure 4A-B; Figure S5A). For immune infiltration, ssGSEA score of 14 cell subtypes was upregulated in both FAC2 and FAC3 (Figure 4A and Figure S5A). We next examined the functional status of CD8^+^ T cells (Figure S5B) and found that FAC2 had the highest CD8^+^ T cytotoxicity score, indicating greater functionality 37. We also evaluated immune cell infiltration using cell types from the study of Charoentong 38 (Figure S5C) and found that FAC2 was rich in adaptive immune cells such as activated CD4^+^ and CD8^+^ T cells, while FAC3 was enriched in innate immune cells, such as natural killer cells, MDSC and regulatory T cells, recognized as the major components of immune suppressive TME 39. Nearly all stromal cell types were mainly enriched in FAC3 (Figure 4A and S5A), among which myofibroblasts were the most abundant (Figure 4B). Subsequent survival analysis showed that myofibroblasts could predict a poor prognosis (Figure 4C; overall survival (OS) in TCGA, P = 0.028; relapse-free survival (RFS) in meta-GEO, P < 0.0001; log-rank test). Previous findings in Figure 2B showed that FAC3 tumors displayed stromal pathways activation such as TGF-β and EMT signaling. Given that stromal myofibroblasts can suppress immune microenvironment by TGF-β production 25, and TGF-β can promote angiogenesis, metastasis, tumor cell EMT and fibroblast activation 40, we further evaluated the stromal-related signature enrichment using gene sets (angiogenesis, EMT2, EMT3 and pan-fibroblast TGF-β included) derived from Mariathasan *et al*. 40, and then confirmed their activation in FAC3 tumors (Figure S5D). Moreover, previous studies indicated that immune cells could be retained in the stroma surrounding tumor cell nests rather than penetrating their parenchyma, so that the antitumor immunity could be rendered ineffective 26. Therefore, although FAC3 tumors had an abundance of immune cells, FAC3 tumors were recognized as immune-excluded phenotype 41, 42.

Previous studies have indicated that MSI/dMMR status and tumor mutation burden (TMB) level could predict the response to immunotherapy 43. We next checked them in our classification and identified their high level (MSI-H/dMMR and TMB-H) in FAC2 (Figure 4D). Given that PD-L1 expression could predict the response to anti-PD-1/L1 treatment 44, we compared the PD-L1 expression among the three clusters, and then observed the higher expression of PDL-1 in FAC2 and FAC3 (Figure 4E). Based on previous findings, FAC1 subtype was recognized as immune-deserted characterized by a paucity of immune and stromal cells. FAC2 subtype was recognized as immune-inflamed characterized by adaptive immune cell infiltration and immune activation, which predicted a positive response to immunotherapy, such as anti-PD-L1/PD-1 therapy. However, FAC3 subtype was recognized as immune-excluded characterized by innate immune cell infiltration and remarkable stromal cell infiltration. Therefore, FAC3 subtype, though with upregulated PD-L1 expression, might show ineffective response to anti-PD-L1/PD-1 therapy (Figure 4E). Correlation between detailed cell types and ferroptosis-associated gene score demonstrated the strong influences of ferroptosis on CRC stromal and immune infiltration (Figure 4F).

### Identifying specific ferroptosis-associated genes correlated with immune activation

**Figure 5:**
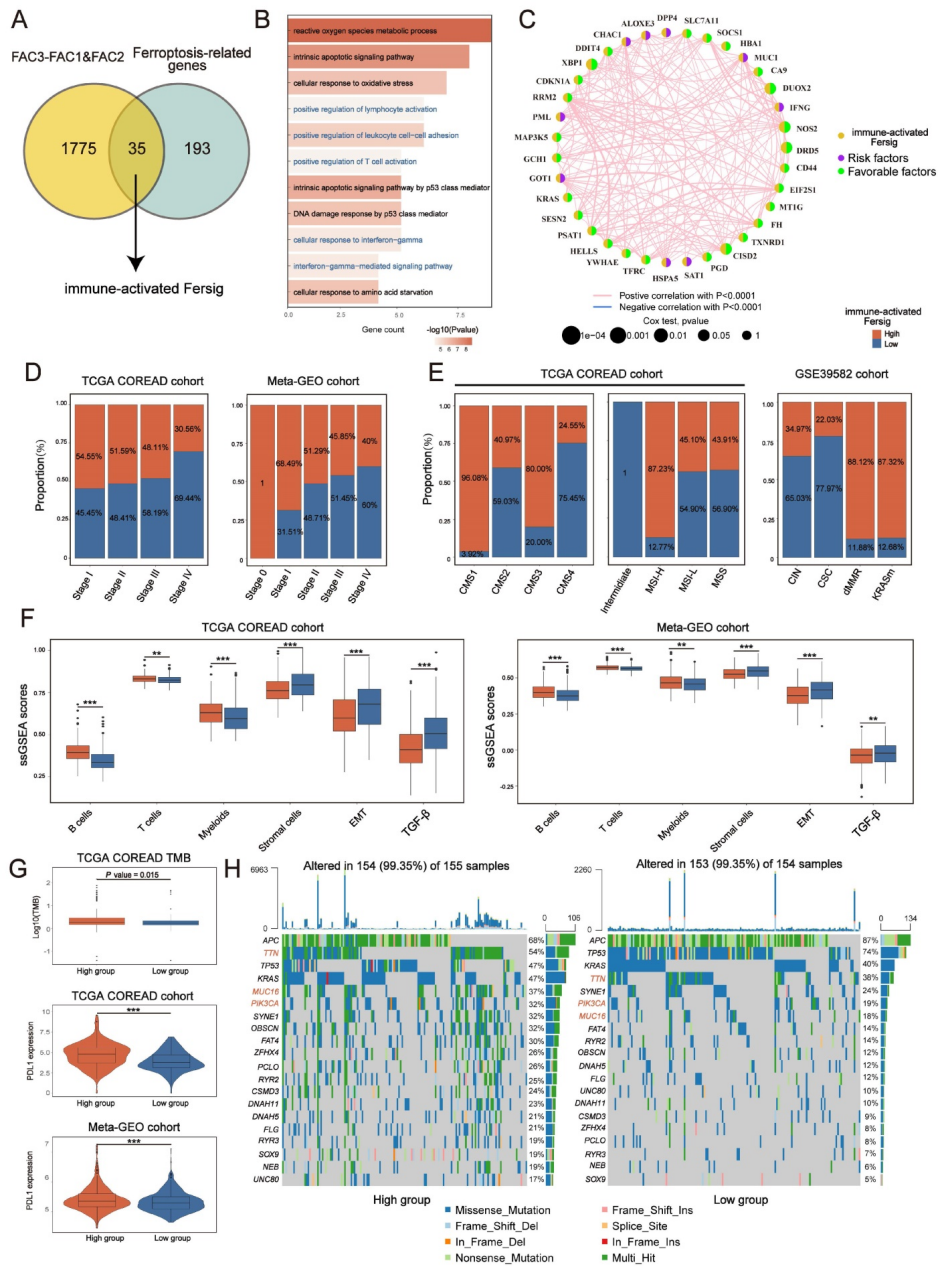
** Identifying specific ferroptosis-associated genes correlated with immune activation. (A)** Venn diagram shows shared genes between DEGs upregulated in FAC2 and ferroptosis-associated genes, which obtained 35 genes termed immune-activated Fersig. **(B)** GO analysis of immune-activated Fersig. **(C)** Correlation between 35 genes. The size of each gene represents survival impact (log-rank test P values indicated)**.** Favorable factors for overall survival are indicated in green, and risk factors are indicated in purple. The thickness of the line represents the strength of correlation estimated by Pearson correlation analysis. Positive correlation is indicated in pink and negative correlation in blue. **(D)** The high and low groups were divided by the median value of the ssGSEA score of immune-activated Fersig. Barplots show the proportion of clinical characteristics between high and low groups of immune-activated Fersig. **(E)** Barplots show the proportion of molecular characteristics between high and low groups of immune-activated Fersig.** (F)** ssGSEA score of signatures of TME cell types, EMT and TGF-β between high and low groups in TCGA and meta-GEO cohorts. The difference of two clusters was compared through the wilcox test. *P < 0.05; **P < 0.01; ***P < 0.001.** (G)** Comparison of TMB and PD-L1 expression between high and low groups. The difference of two clusters was compared through the wilcox test. *P < 0.05; **P < 0.01; ***P < 0.001. **(H)** Oncoplots show genetic alterations of common mutant genes in CRC between high and low groups. The number on the right indicates the mutation frequency in each regulator. Each column representes individual patient. High group: n=155; low group: n=154.

We assessed the transcriptional signature of ferroptosis correlated with immune activation. By overlapping ferroptosis-associated genes with DEGs upregulated in FAC2 (Figure 5A), we obtained 35 genes related to immune activation, such as T cells activation and interferon gamma response (Figure 5B), and then recognized them as immune-activated Fersig. All these genes displayed positive correlation (Figure 5C), implying their similar functional category.

Analysis of copy number variation (CNV) showed a widespread CNV alteration (Figure S6A) among them. Therefore, we investigated their influence on clinical and molecular patterns using ssGSEA score. We found that patients with high immune-activated Fersig expression displayed a paucity of advanced stage (stage IV) (30.56% vs 69.44% in TCGA; 40% vs 60% in meta-GEO) (Figure 5D). Moreover, patients with high immune-activated Fersig expression presented immune-activated characteristics, such as remarkable enrichment of CMS1 (96.08% in TCGA), MSI-H (87.23% in TCGA), dMMR subtypes (88.12% in GSE39582) (Figure 5E), and significant immune cells infiltration (Figure 5F). We observed that stromal cell infiltration and stromal pathways were downregulated in high immune-activated Fersig score group (Figure 5F). Somatic mutation analysis showed that high immune-activated Fersig score group was associated with higher mutation rates of common mutant genes in CRC, such as TTN (54% vs 38%), MUC16 (37% vs 18%) and PIK3CA (32% vs 19%) (Figure 5H). Given that the high immune-activated Fersig score group displayed a higher level of PD-L1 expression and TMB quantification (Figure 5G), we postulated that immune-activated Fersig presented a predictive advantage in response to immunotherapy.

To find the potential genes in regulating immune activation for future study, we performed univariate Cox analyses of immune-activated Fersig on both TCGA (based on overall survival (OS)) and meta-GEO (based on relapse-free survival (RFS)) cohort (Figure S6B). The results showed that 11 genes were associated with better prognosis, among which DUOX2, XBP1, EIF2S1, SOCS1, DRD5, NOS2 and PSAT1 have been reported to be correlated with the promotion of ferroptosis, whereas RRM2, TFRC, CISD2 and GCH1 have been reported to be associated with the inhibition of ferroptosis. These 11 genes were highly expressed in FAC2 group, confirming their main role in immune activation (Figure S6C). Subsequent correlation analysis showed that XBP1, CISD2, EIF2S1, RRM2, GCH1, SOCS1, NOS2 and PSAT1 displayed positive correlation with CD8^+^ T cytotoxicity score and negative correlation with myofibroblasts score (Figure S6D), suggesting these genes could trigger immune activation. The correlation between their regulation of ferroptosis and immune activation in CRC need to be explored in future studies.

### Identifying specific ferroptosis-associated genes correlated with stromal activation

**Figure 6:**
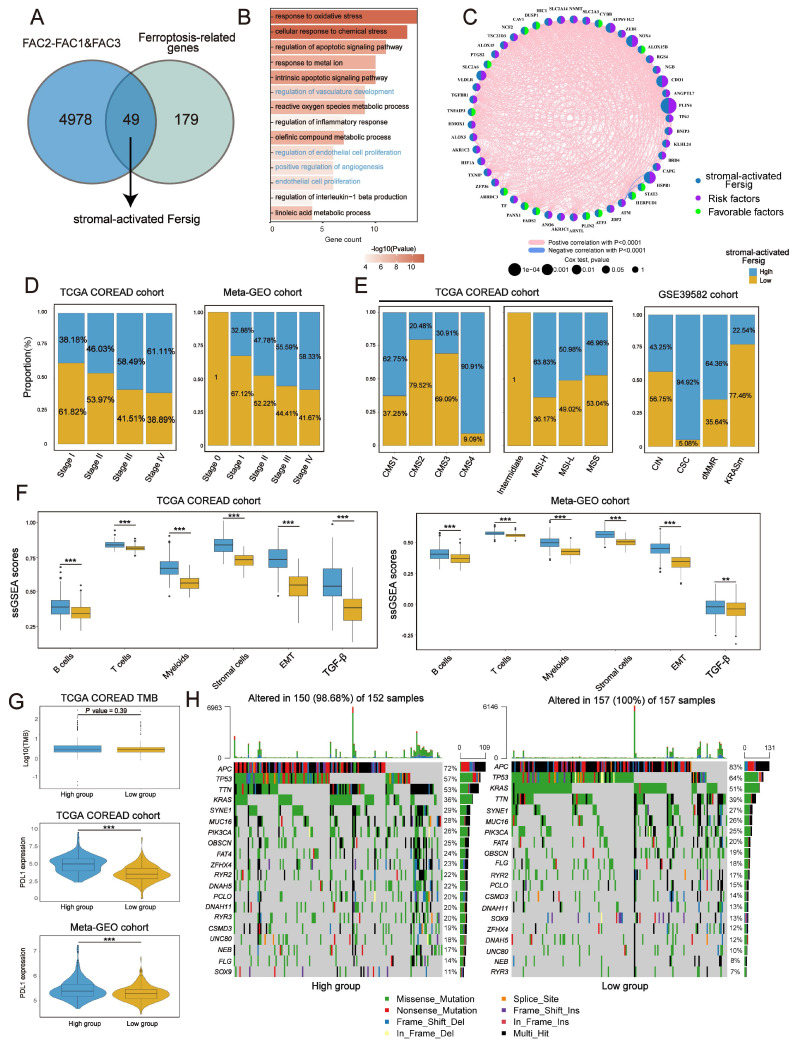
** Identifying specific ferroptosis-associated genes correlated with stromal activation. (A)** Venn diagram shows shared genes between DEGs upregulated in FAC3 and ferroptosis-associated genes, which obtained 49 genes termed stromal-activated Fersig. **(B)** GO analysis of stromal-activated Fersig. **(C)** Correlation between 49 genes. The size of each gene represents survival impact (log-rank test P values indicated). Favorable factors for overall survival are indicated in green, and risk factors are indicated in purple. The thickness of the line represents the strength of correlation estimated by Pearson correlation analysis. Positive correlation is indicated in pink and negative correlation in blue. **(D)** The high and low groups were divided by the median value of the ssGSEA score of stromal-activated Fersig. Barplots show the proportion of clinical characteristics between high and low groups of stromal-activated Fersig. **(E)** Barplots show the proportion of molecular characteristics between high and low groups of stromal-activated Fersig.** (F)** ssGSEA score of signatures of TME cell types, EMT and TGF-β between high and low groups in TCGA and meta-GEO cohort. The difference of two clusters was compared through the wilcox test. *P < 0.05; **P < 0.01; ***P < 0.001.** (G)** Comparison of TMB and PD-L1 expression between high and low groups. The difference of two clusters was compared by the wilcox test. *P < 0.05; **P < 0.01; ***P < 0.001. **(H)** Oncoplots show genetic alterations of common mutant genes in CRC between high and low groups. The number on the right indicates the mutation frequency in each regulator. Each column representes individual patient. High group: n=155; low group: n=154.

Identical analyses were performed to obtain a stromal-activated Fersig, including 49 genes, characterized by stromal processes enrichment such as endothelial cell proliferation (Figure 6A-C). Prevalent CNV alteration also indicated their influence on CRC progression (Figure S7A). Contrary to immune-activated Fersig, patients with high stromal-activated Fersig expression were enriched in more advanced stage (stage IV) (61.11% vs 38.89% in TCGA; 58.33% vs 38.89% in meta-GEO) (Figure 6D). For molecular characteristics, high stromal-activated Fersig score group was prominently enriched in stromal subtypes, including CMS4 (90.91% in TCGA) and CSC (94.92% in GSE39582). We also observed the immune cell infiltration and higher expression of PD-L1 in high stromal-activated Fersig score group (Figure 6E-G). However, high stromal-activated Fersig score group displayed remarkable stromal cell infiltration, EMT and TGF-β pathways activation, and showed no significant differences in somatic mutation and TMB (Figure 6F-H). Therefore, stromal-activated Fersig may promote CRC progression and predict an ineffective response to immunotherapy.

Next, univariate Cox analyses of stromal-activated Fersig on both TCGA (based on overall survival (OS)) and meta-GEO cohort (based on relapse-free survival (RFS)) obtained 16 genes associated with significant worse prognosis. Among them, NOX4, ALOX15, CDO1, ATF3, TXNIP, PLIN4, HIC1 and ATP6V1G2 have been reported to be correlated with the promotion of ferroptosis, whereas DUSP1, RGS4, SLC2A3, SLC2A6, SLC2A14, AKR1C2, HSPB1 and ZFP36 have been reported to be associated with the inhibition of ferroptosis (Figure S7B). These 16 genes were highly expressed in FAC3 group, confirming their main role in stromal infiltration (Figure S7C). Subsequent correlation analysis showed that PLIN4, RGS4, SLC2A3, SLC2A14, HIC1, DUSP1, CDO1, NOX4 and SLC2A6 displayed negative correlation with CD8^+^ T cytotoxicity score and positive correlation with myofibroblasts as well as EMT gene score (Figure S7D), suggesting these genes could contribute to the suppressive tumor microenvironment. The correlation between their regulation of ferroptosis and the suppressive tumor microenvironment in CRC need to be explored in future studies.

### Examining expression of immune-activated and stromal-activated Fersig at single-cell level

**Figure 7:**
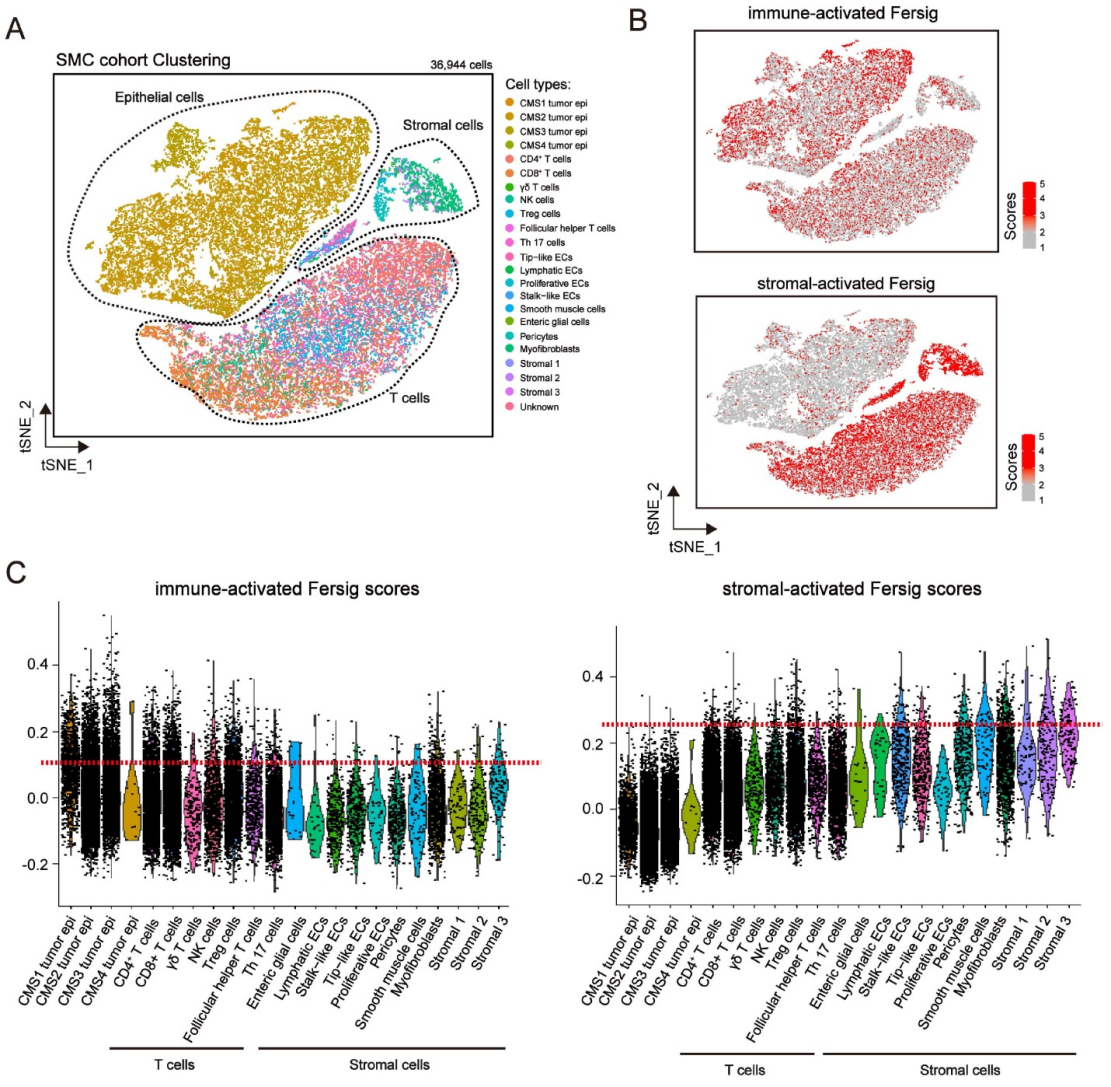
** Examining expression of immune-activated and stromal-activated Fersig at single-cell level. (A)** t-SNE visualization of 36,944 single cells of CRC. Cells are colored according to cell types. **(B)** t-SNE visualization of score of immune-activated Fersig and stromal-activated Fersig.** (C)** Violin plots shows the score of immune-activated Fersig and stromal-activated Fersig among different cell subtypes.

We explored the expression level of immune-activated and stromal-activated Fersig at single-cell level (Figure 7A and S8A). The results showed that immune-activated ferroptosis-associated genes were mainly expressed in tumor cells and T cells (Figure 7B-7C and Figure S8B-S8C). However, stromal-activated ferroptosis-associated genes were predominantly expressed in stromal cells (Figure 7B-7C and Figure S8B-S8C). Consistent with the previous mapping FAC1-3 subtypes on CRC tumor cells, the expression of stromal-activated genes in CRC tumor epithelial cells were lower than the immune-activated genes. These analyses further confirmed the definition of immune-activated and stromal-activated genes.

### Ferroptosis phenotype-related DEGs in colorectal cancer

**Figure 8:**
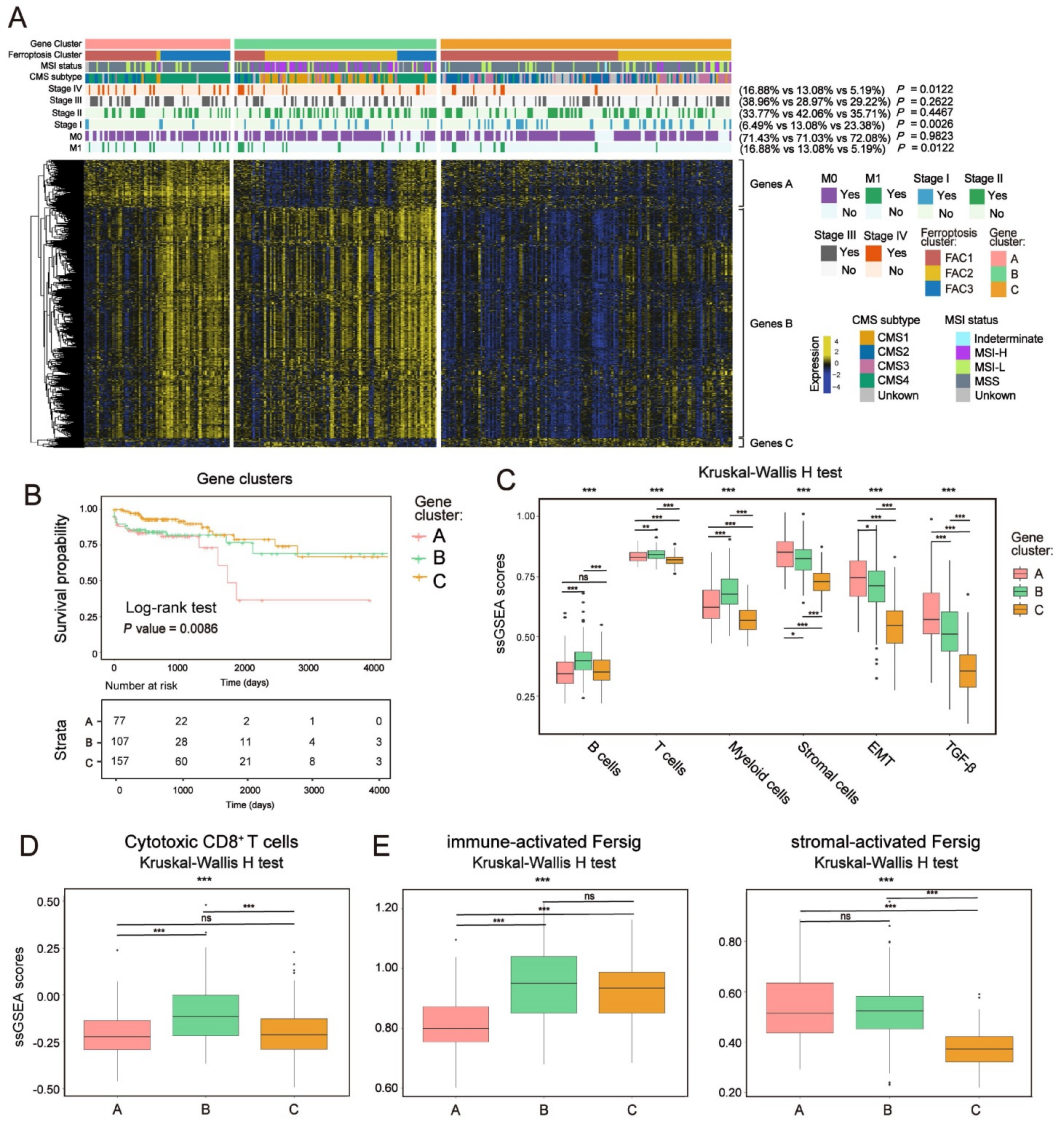
** Ferroptosis phenotype-related DEGs in colorectal cancer. (A)** NMF clustering of TCGA tumor samples using ferroptosis phenotype-related signature**.** Clinical and molecular characteristics are shown on the top. The difference of three gene clusters was compared through the Pearson's chi-square test.** (B)** Kaplan-Meier curves for overall survival of three gene clusters in TCGA. P value was determined by the log-rank test.** (C)** ssGSEA score of signatures of TME cell types, EMT and TGF-β among three gene clusters in TCGA. The statistical difference of three clusters was compared by the Kruskal-Wallis H test. *P < 0.05; **P < 0.01; ***P < 0.001. The difference of two clusters was compared by the wilcox test. *P < 0.05; **P < 0.01; ***P < 0.001. **(D-F)** ssGSEA score of signatures of cytotoxic CD8^+^ T cells, immune-activated and stromal-activated ferroptosis-associated genes among three gene clusters. The statistical difference of three clusters was compared by the Kruskal-Wallis H test. *P < 0.05; **P < 0.01; ***P < 0.001. The difference of two clusters was compared by the wilcox test. *P < 0.05; **P < 0.01; ***P < 0.001.

To further confirm the underlying molecular and clinical patterns determined by ferroptosis-associated genes, we overlapped 854 DEGs among the three ferroptosis-associated clusters and recognized them as ferroptosis phenotype-related signature (Figure S9A). We next performed unsupervised consensus clustering analyses based on a univariate Cox regression analysis of the 854 DEGs (Method section). Both in TCGA and meta-GEO cohorts, this analysis divided patients into three ferroptosis phenotype-related signature groups that had different clinicopathologic subgroups, which were defined as gene-cluster A, B and C (Figure 8A; Figure S9B and Figure S10A; Table S5). By hierarchical clustering and GO analysis (Figure 8A and Figure S9C), the 854 DEGs were further grouped into signature genes A, B and C (Table S5). Genes A were associated with stromal biological processes such as muscle system process and TGF-β signaling. Genes B were associated with immune cells activation. Genes C were enriched in catabolic process and RNA processing. We observed that gene-cluster A presented worst prognosis (Figure 8B; overall survival (OS) in TCGA, P = 0.0086; Figure S10A, relapse-free survival (RFS) in meta-GEO, P = 0.03, log-rank test) with significant enrichment of advanced stage patients (M1 and stage IV) (16.88% in gene-cluster A, *P* = 0.0122, Pearson's chi-square test) (Figure 8A). We also found that gene-cluster A contained most of the previous FAC3 tumors. Moreover, it was also characterized by CMS4 subtype enrichment (Figure 8A), remarkable stromal cells infiltration (especially myofibroblasts infiltration) (Figure S9E) and stromal-related pathways activation (TGF-β, EMT and angiogenesis included) (Figure 8C, S9D and S10B). Gene-cluster B had most of the FAC2 tumors, characterized by CMS1 subtype enrichment, strong immune cells infiltration (Figure 8A, 8C, S9E and S10B) and especially highest enrichment of cytotoxic CD8^+^ T cells (Figure 8D and S10C). Gene-cluster C possessed most of the FAC1 tumors, and displayed the paucity of TME cells (Figure 8A, 8C, S9E and S10B). Subsequent analysis showed that our immune-activated Fersig tended to be upregulated in gene-cluster B, while stromal-activated Fersig tended to be upregulated in gene-cluster A (Figure 8E and S10D). This result was consistent with previous findings that ferroptosis played a main role in both immune and stromal infiltration. Taken together, based on ferroptosis-associated genes regulation, CRC could be divided into three stable distinct phenotypes, including immune-deserted, immune-inflamed, and immune-excluded phenotypes.

### Further construction of Fersig score and exploration of its role in predicting immunotherapeutic benefits

**Figure 9:**
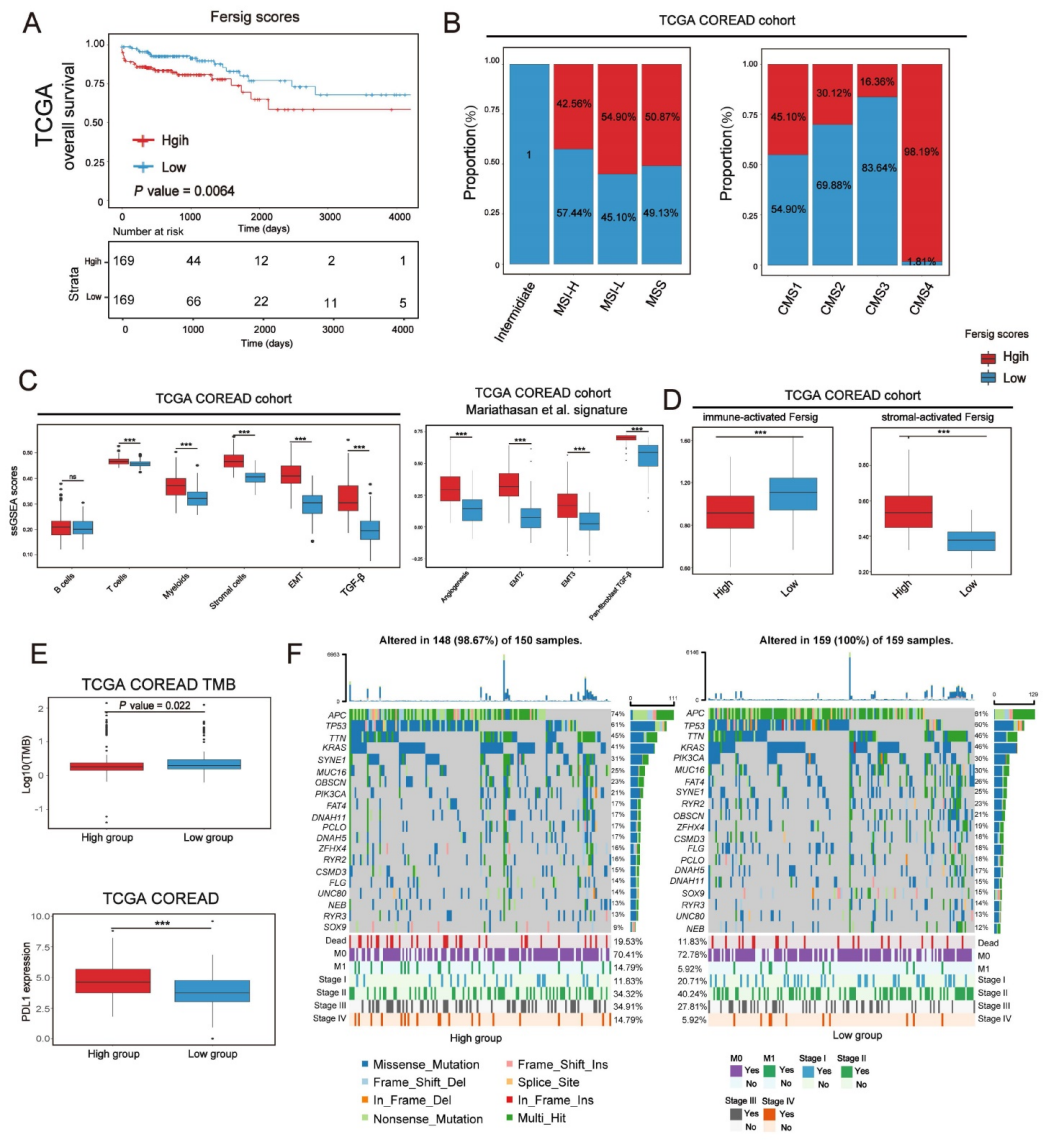
** Further construction of Fersig score. (A)** Kaplan-Meier curves for overall survival of TCGA. The high and low groups were divided by the median value of the PCA score of Fersig. P value was determined by the log-rank test. **(B)** Barplots show the proportion of molecular characteristics between high and low groups of Fersig. **(C)** ssGSEA score of signatures of TME cell types, EMT, TGF-β and other stromal-related signatures between high and low groups in TCGA cohort. The difference of two clusters was compared by the wilcox test. *P < 0.05; **P < 0.01; ***P < 0.001. **(D)** ssGSEA score of immune-activated and stromal-activated Fersig between high and low groups in TCGA cohort. The difference of two clusters was compared by the wilcox test. *P < 0.05; **P < 0.01; ***P < 0.001. **(E)** Comparison of TMB and PD-L1 expression between high and low groups. The difference of two clusters was compared by the wilcox test. *P < 0.05; **P < 0.01; ***P < 0.001.** (F)** Oncoplots show genetic alterations of common mutant genes in CRC between high and low groups. The number on the right indicates the mutation frequency in each regulator. Each column representes individual patient. High group: n=150; low group: n=159.

We have characterized the role of ferroptosis-associated genes in prognosis, immune and stromal infiltration modulation based on patient population. After that, we developed a scoring scheme termed Fersig score (Methods section), which is based on ferroptosis phenotype-related signature, to quantify ferroptosis regulation in individual CRC patient. Since the regulation of ferroptosis focused on immune and stromal infiltration, we calculated the Fersig score based on immune-related genes B and stromal-related genes A in Figure 8A. In TCGA and GEO cohort, we observed that the high Fersig score group had the worse prognosis (Figure 9A and S11A; overall survival (OS) in TCGA, P = 0.0064; relapse-free survival (RFS) in meta-GEO, P = 0.0021; log-rank test). For molecular characteristics, we found that nearly all CMS4 (98.19% in TCGA) and CSC subtype (94.92% in GSE39582) were clustered into the high Fersig score group (Figure 9B and S11B). Subsequent TME infiltration analysis showed that although the high Fersig score group displayed immune cell infiltration (Figure 9C and S11C, S11E), it presented stromal-related signaling activation and remarkable stromal cell infiltration (Figure 9C, S11C and S11E), especially myofibroblasts infiltration (Figure S11E). We then demonstrated that the high Fersig score group upregulated the expression of stromal-activated Fersig and downregulated the expression of immune-activated Fersig (Figure 9D and S11D), indicating the high Fersig score group was like an immune-excluded phenotype. Finally, we turned our attention to the somatic mutation patterns and found that the high Fersig score group had no advantage in gene mutation and downregulated the TMB (Figure 9E). Moreover, high Fersig score group was enriched in more death (Figure 9F), and advanced stage (M1 and stage IV) of CRC patients (Figure 9F and S11B)), consistent with the worse prognosis in the high Fersig score group. Therefore, although PD-L1 was upregulated (Figure 9E), the high Fersig score group predicted an ineffective response to immunotherapy due to its remarkable stromal cell infiltration.

**Figure 10:**
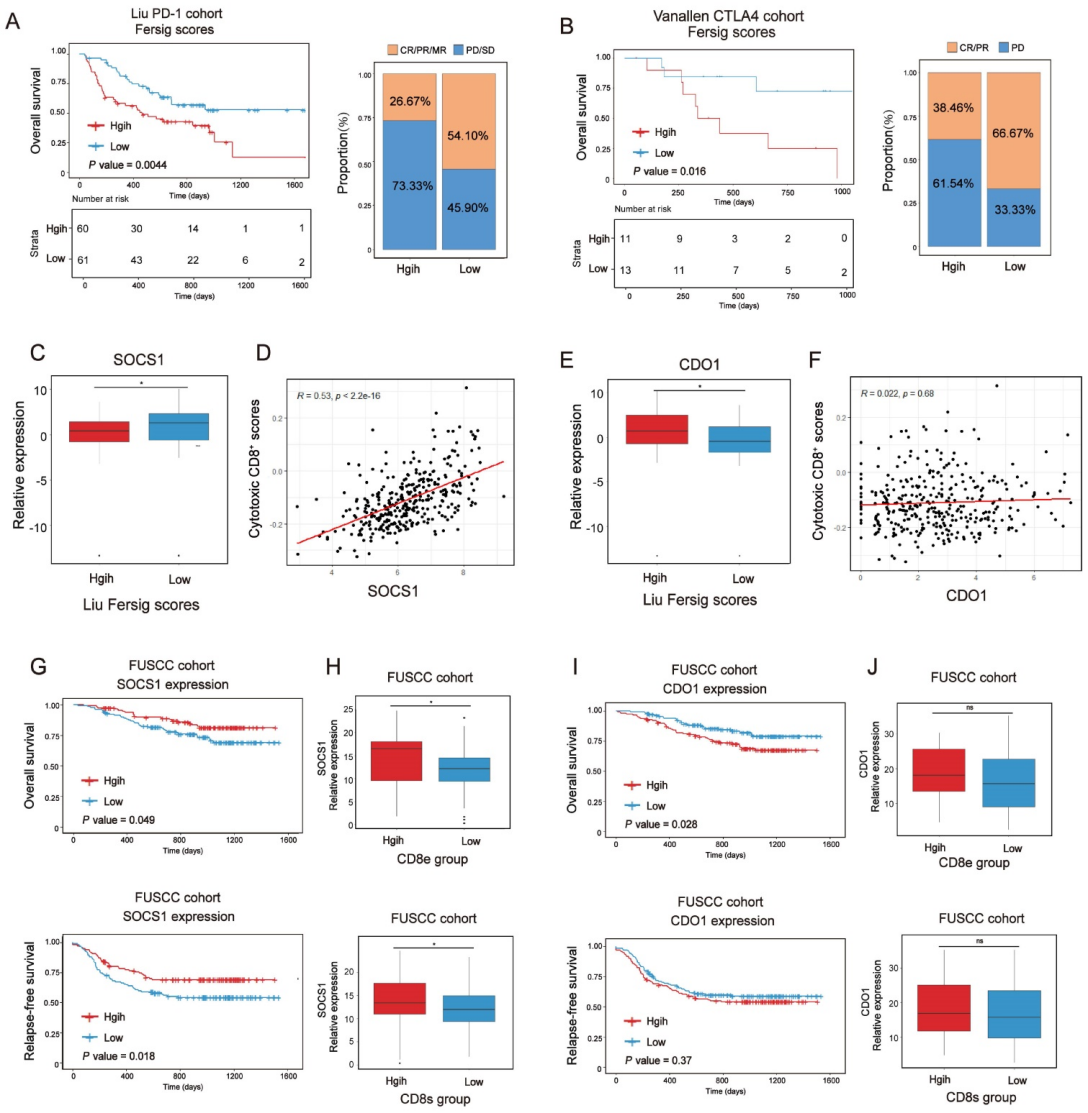
** Exploration of Fersig score' role in predicting immunotherapeutic benefits. (A)** Kaplan-Meier curves for high and low Fersig score patient groups in the Liu et al.'s cohort. Log-rank test, P = 0.0044. The fraction of patients with clinical response to anti-PD-1 immunotherapy in low or high Fersig score groups of Liu et al.'s cohort. **(B)** Kaplan-Meier curves for high and low Fersig score patient groups in the Vanallen et al.'s cohort. Log-rank test, P = 0.016. The fraction of patients with clinical response to anti-CTLA-4 immunotherapy in low or high Fersig score groups of Vanallen et al.'s cohort. **(C, E)** Barplots show the expression of SOCS1/CDO1 in high and low Fersig score patient groups of the Liu et al.'s cohort. (**D, F**) Dotplots show the correlation between the expression of SOCS1/CDO1 and cytotoxic CD8+ T score**. (G, J)** The high and low groups were divided by the median value of gene expression. Kaplan-Meier curves for high and low patient groups in FUSCC TMA cohort. P value was determined by the log-rank test.** (H,J)** The gene expression of SOCS1 and CDO1 in CD8e or CD8s high and low groups. The difference of two groups was compared by the wilcox test. *P < 0.05; **P < 0.01; ***P < 0.001.

Immunotherapies represented by PD-1/CTLA-4 inhibitors have been widely used in cancer therapy 45. We next investigated whether our Fersig scoring algorithm could predict effective response to these two immune checkpoint blockade therapies. In both anti-PD-1 cohort (Liu *et al*. study) 46 and anti-CTLA-4 cohort (Vanallen *et al.* study)^47^, patients with low Fersig score exhibited significantly better prognosis (Figure 10A, P = 0.0044; Figure 10B, P = 0.016; log-rank test). Furthermore, patients with low Fersig score displayed more effective clinical response to anti-PD-1/CTLA-4 immunotherapies, compared to those with high Fersig score (response rate of anti-PD-1 cohort: 54.10% vs. 26.67%, Figure 10A; response rate of anti-CTLA-4 cohort: 66.67% vs. 38.46%, Figure 10B). Taken together, the Fersig score we constructed could be used to predict the prognosis of patients and their response to immunotherapies.

We previously identified immune-activated and stromal-activated ferroptosis-associated genes. To further understand their correlation with immunotherapies, we compared the expression level of genes with significant prognosis between low and high Fersig score groups of the anti-PD-1 cohort (Liu et al. study) (Figure S12A-S12B). The results showed that immune-activated genes with better prognosis, such as SOCS1, were upregulated in the low Fersig score group (Figure 10C), while stromal-activated genes with worse prognosis, such as CDO1, were upregulated in the high Fersig score group (Figure 10E). Subsequent correlation analyses showed that the expression of SOCS1 was positively correlated with the infiltration of cytotoxic CD8^+^ T cells, while the expression of CDO1 didn't show significant correlation (Figure 10D and 10F). We also utilized tissue microarray (TMA), containing 308 patients, as a patient cohort to explored the correlation between SOCS1/CDO1 and the prognosis of patients. The results showed that SOCS1 could predict the better prognosis, while CDO1 may predict the worse prognosis (Figure 10G and10I). As our team previously reported^48^, the cancer tissues on our TMA could be divided into high or low intraepithelial or stromal CD8^+^ TILs groups. We found that high intraepithelial or stromal CD8^+^ TILs groups (CD8e or CD8s) were positively correlated with the expression of SOCS1, while CDO1 didn't show significant association with high CD8e or CD8s groups (Figure 10H and 10J; Figure S12C-S12D). These data indicated the considerable immunotherapeutic benefit of CRC with high SOCS1 expression. Overall, immune-activated, and stromal-activated ferroptosis-associated genes we constructed could be the target genes to enhance the outcomes of patients' immunotherapy.

## Discussion

Evidences have showed that ferroptosis could regulate the antitumor immunity by their interaction with different immune cell types, which is potentially recognized as a concept of immunogenic death (ICD) 49, 50. Although various studies have revealed the regulation of ferroptosis-associated genes in TME, a landscape of TME characteristics mediated by ferroptosis-associated genes have not been comprehensively recognized. Therefore, characterizing ferroptosis-associated phenotypes in TME would promote our understanding of how ferroptosis impacts tumor immunity, which may be applied to the existing immunotherapies to enhance their efficacy.

In this study, we identified three distinct ferroptosis-associated molecular patterns characterized by different TME phenotypes, which were related to diverse antitumor immunity in CRC patients. The ferroptosis-associated cluster FAC1 was characterized by paucity of TME cells infiltration, corresponding to the immune-desert phenotype. The FAC2 was characterized by immune activation, consistent with the immune-inflamed and CMS1-like phenotype. The FAC3 was characterized by remarkable stromal cells infiltration, together with EMT, TGF-β signaling pathway activation, identical to the immune-excluded and CMS4-like phenotype. We also performed analyses of scRNA databases to confirmed the biological characteristics of FAC1 and FAC2 on CRC tumor single cells, and highlighted the remarkable infiltration of stromal cells in FAC3 patients. Given that baseline levels of tumor-infiltrating immune, stromal cells and inflammatory cytokines secretion have been showed to predict the immune response 51. We also identified that FAC2 was significantly associated with elevated cytotoxic CD8^+^ T cells infiltration and PD-L1 expression, supporting its predictive advantage in response to immunotherapy. However, FAC3 patients presented enrichment of myofibroblasts, which was confirmed to be associated with worse prognosis in CRC patients. Previous studies reported that stromal myofibroblasts contributed to an immune suppressive microenvironment by TGF-β production 25, 52. Although FAC3 patients upregulated the expression of PD-L1, we postulated that CRC patients with FAC3 pattern couldn't effectively respond to immunotherapy because the TGF-β pathways and myofibroblasts impeded the penetration of cytotoxic CD8^+^ T cells into the tumor parenchyma. Therefore, therapies targeting TGF-β might restore the effective response to immunotherapy in CRC patients with FAC3 pattern.

Next, we identified two subsets of ferroptosis-associated genes, recognized as immune-activated and stromal-activated genes. By comprehensive analyses of clinical characteristics, TME infiltration, stromal-related pathways enrichment and TMB level, we found that patients with upregulated expression of immune-activated genes exhibited clinical advantages, immune activation, and the high level of TMB, implying their effective response to immunotherapy. Conversely, patients with upregulated expression of stromal-activated genes displayed clinical disadvantages, stromal-related pathways activation and remarkable stromal cells infiltration, implying their ineffective response to immunotherapy. Notably, consistent with the expression patterns of PD-L1 in FAC2 and FAC3 groups, both patients with upregulation of immune-activated or stromal-activated genes highly expressed PD-L1. Previous studies indicated that the prognostic of PD-L1 expression in CRC is contradictory 53-55. Blocking PD-L1/PD-1 interaction can prolong tumor suppression or stabilize the progression of cancers 56. Based on our findings of two phenotypes with distinct TME infiltration in patients with upregulation of PD-L1, we postulated that we should determine the response of CRC patients to immunotherapy mainly based on TME cells infiltration, especially in patients with high expression of PD-L1. In our study, we found that the two subsets of immune-activated and stromal-activated ferroptosis-associated genes could distinguish better or worse response to immunotherapy in CRC patients with high expression of PD-L1.

We also identified the potential ferroptosis-associated genes of immune-activated and stromal-activated subsets based on their significant influence on the prognosis of CRC. In immune-activated subset, XBP1, CISD2, EIF2S1, RRM2, GCH1, SOCS1, NOS2 and PSAT1 displayed positive correlation with prognosis and cytotoxic CD8^+^ T cells infiltration, implying their advantages in immunotherapy. However, these genes have been reported as drivers or suppressors in ferroptosis. Similarly, in stromal-activated subset, PLIN4, RGS4, SLC2A3, SLC2A14, HIC1, DUSP1, CDO1, NOX4 and SLC2A6 exhibited negative correlation with CD8^+^ T cytotoxicity score and positive correlation with myofibroblasts as well as EMT signature score, implying their disadvantages in immunotherapy. They also contained drivers or suppressors of ferroptosis. Therefore, the role of these genes in ferroptosis for CRC should be further explored. Overall, these findings suggested that we could apply the intervention on these target genes to enhance the outcomes of patients' immunotherapy.

Further unsupervised consensus clustering analyses based on ferroptosis phenotype-related signature confirmed the stable TME infiltration patterns in CRC patients, and highlighted the main role of ferroptosis-related process in immune activation and stromal cells infiltration. We also constructed a scoring scheme termed the Fersig score, and verified its positive correlation with stromal-related pathways activation and stromal cells infiltration. Given the absence of CRC immunotherapy dataset, we analyzed different immunotherapy regimens (anti-PD-1/CTLA-4) across different datasets, and verified that low level of Fersig score could predict better response to immunotherapies. As a PCA algorithm, this scoring scheme showed an advantage of focusing the score on the well-correlated genes, and it could downregulate contributions of anti-correlated genes, which could improve the accuracy and efficiency of its prognostic and immunotherapy prediction. In clinical practice, the Fersig scoring scheme could be used to comprehensively investigate the ferroptosis-regulated patterns and corresponding TME infiltration characterization in individual patient. It could not only evaluate patients' clinicopathological features such as stages, molecular subtypes and TMB, but also identify the immune phenotypes of tumors. The Fersig scoring scheme may guide the more effective clinical practice to improve patients' response to immunotherapies.

In conclusion, we comprehensively explored the ferroptosis regulation patterns among 1,251 CRC tumor bulk samples and 29 single-cell samples, and identified their correlation with TME cell-infiltrating characteristics. These integrated analyses highlighted the main role of ferroptosis in immune activation and stromal cells infiltration during CRC development, which will contribute to understanding the TME infiltration based on ferroptosis and provide an interesting insight into immunotherapeutic efficacy.

## Methods

### Immunohistochemistry (IHC)

Cancer tissue microarray including 308 CRC cases were obtained from the Fudan University Shanghai Cancer Center (FUSCC). This study was approved by the Ethical Committee of the FUSCC and all participants signed written informed consent in accordance with the regulations of the Institutional Review Boards of the FUSCC. Immunohistochemically staining was performed according to standard protocol. Briefly, paraffin-embedded samples were cut into 4 μm sections and placed on polysine coated slides. Paraffin sections were baked overnight at 58°C, dewaxed in xylene, rehydrated through a graded series of ethanol, quenched for endogenous peroxidase activity in 0.3% hydrogen peroxide for 15 mins. Antigen retrieval was performed by high-pressure cooking in citrate buffer (pH=6.0) for about 20 mins, then allowed to cool to room temperature, blocking the nonspecific antibody binding sites in 5% normal goat serum for 2 hrs. Sections were incubated at 37°C for 1.5 hrs with rabbit polyclonal antibody against SOCS1 (1:100, Affinity, AF5378), and CDO1 (1:100, Proteintech, 12589-1-AP).

### Preparation of bulk RNA expression datasets

Colorectal cancer databases (gene expression data and clinical data included) were obtained from the GEO database (http://www.ncbi.nlm.nih.gov/geo/) and TCGA (https://xenabrowser.net /datapages/TCGA-COREAD). For GEO database, a total of 913 patients from GSE39582, GSE14333, and GSE37892 were identified and fully studied in this study. Combat method was used to remove the batch effects among three different GEO datasets. We finally combined three GEO datasets as a meta-GEO cohort.

For TCGA database, RNA expression data of a total 382 patients (338 tumor and 44 normal) were downloaded from the UCSC Xena (https://xenabrowser.net/datapages/TCGA-COREAD). The somatic mutation data of TCGA-COREAD were curated from https://portal.gdc.cancer.gov/repository. The copy number variation data was curated from the UCSC Xena database. The description of patients with bulk RNA expression data was shown in Table S1.

### Preparation of single-cell RNA expression datasets

scRNA data was downloaded from the GEO database (http://www.ncbi.nlm.nih.gov/geo/). Two cohorts of GSE144735 25 were identified and fully studied in this study.

We performed Seurat-based analysis to preprocess the single-cell data. The method of quality control (including the threshold of number of detected genes per cell and the percentage of mitochondrial genes expressed) followed Lee *et al*.'s study 25. For batch correcting, we applied Harmony integration as previously described 57. The basic information for single-cell datasets of all samples were shown in Table S2.

### Ferroptosis-associated genes used for analysis

259 ferroptosis-associated genes were obtained from FerrDb database (http://www.zhounan.org/ferrdb/), including 108 drivers, 69 suppressors and 111 markers. The biological information of these genes was shown in Table S3. Only human genes were analyzed in our study. There are totally 71 drivers, 61 suppressors and 96 markers.

### Consensus molecular clustering by NMF

To obtain consensus clustering of colorectal cancer samples, we performed consensus clustering with NMF to identify distinct classification based on the expression of ferroptosis-associated human genes. The optimal number of clusters was selected according to cophenetic coefficients (Fig S1A). When rank=3, the consensus matrix heatmap still kept sharp boundaries, indicating its stable clustering. Therefore, rank=3 was finally selected as optimal number of clusters. The NMF clusters of TCGA and meta-GEO were shown in Table S1.

### Gene set variation analysis (GSVA) and single-sample gene set enrichment (ssGSEA) analysis

In Figure 1C, we quantified the pathway activities of tumor samples by applying GSVA analysis. The pathway signatures derived from the Hallmark gene sets and C2 curated gene sets (download from MSigDB database v7.4). The significantly expressed pathways in three distinct clusters were shown by heatmap.

The gene signatures in Figure 2B were obtained from CMScaller package, and the gene sets in Figure 8C were constructed by Mariathasan *et al*.. The signatures named EMT and TGF-β through our study were obtained from CMScaller package.

Gene signatures of single cell types used for calculating ssGSEA score were obtained from DEGs of cell types detected in Lee *et al*.'s study 25. The expression difference on a natural log scale was set at least 0.25.

Cytotoxic marker genes were CST7, GZMA, GZMB, IFNG, NKG7 and PRF1. MHC-I marker genes were HLA-A, HLA-B and HLA-C. MHC-II marker genes were HLA-DMA, HLA-DOA, HLA-DRA, HLA-DOB, HLA-DMB, HLA-DPA1, HLA-DPB1, HLA-DQB1, HLA-DQB2, HLA-DPA1, HLA-DPB2, HLA-DQB1 and HLA-DRB.

### CMS classification for bulk RNA-seq

We utilized CMScaller to stratify TCGA-COREAD tumor samples. The CMS subtypes annotation were shown in Table S1.

### Mapping ferroptosis-associated molecular classification of bulk samples onto CRC tumor cells at single-cell level

We first identified the specific genes among three NMF clusters of TCGA-COREAD cohort using FindAllMarkers function. All genes were probed and the expression difference on a natural log scale was set at least 0.5. Then we subset tumor cells from SMC and KUL3 cohorts in Lee *et al*.'s (GSE144735) study and scored them with the specific genes of NMF clusters using AddModuleScore function. We finally annotated each cell type based on their maximum gene score. The annotation of single cell was shown in Table S4.

### Molecular characteristics of single cells identification

The molecular characteristics of each sample in SMC and KUL3 cohorts have been described in Lee *et al*.'s (GSE144735) study, including CMS subtypes at bulk and single-cell levels, SMAD4 mutation, P53 mutation, Braf mutation, KRAS mutation and MSI status. The molecular types of each CRC tumor cell were annotated using their sample characteristics.

### TME infiltration evaluation using ssGSEA and CIBERSORT

In order to precisely quantify the abundance of cell types in CRC tumor, we utilized 5 cell types (epithelial cells, T cells, B cells, Stromal cells and Myeloid cells included) and 31 cell subtypes detected in a single-cell CRC study (GSE144735), and calculated these cell type scores in TCGA and GEO bulk samples using ssGESA analysis. We further calculated relative abundance of each cell type using the deconvolution approach CIBERSORT. To confirm the stable TME infiltration patterns of NMF clusters, we also evaluated immune cell infiltration with cell types from the study of Charoentong 38 using ssGESA analysis.

### Somatic mutation analyses

The somatic mutation data of TCGA-COREAD were curated from https://portal.gdc.cancer.gov/repository. Varscan file format was selected for analyses. The copy number variation data was curated from UCSC Xena database. We identified significantly mutated genes and calculated TMB using 'maftool'.

### Constructing immune-activated and stromal-activated Fersig

We utilized 'limma' package to calculate differentially expressed genes (DEGs) between FAC2 and the other two clusters, recognized as DEGs upregulated in FAC2. Then we overlapped DEGs of FAC2 and ferroptosis-associated genes, and obtained 35 genes termed immune-activated subset. We obtained stromal-activated subset by identical analyses.

In Figure 5 and 6, we divided bulk samples into high and low groups by setting the median value of ssGSEA score of immune-activated and stromal-activated genes as the threshold.

### Defining cell score

We used the AddModuleScore function in the Seurat R package to evaluate the score of immune-activated and stromal-activated genes on single cells in Figure 7 and S7.

### Collection of genomic and clinical information of the ICI-based cohort

Two immunotherapeutic cohorts were included in our study: metastatic melanoma treated with anti-PD-1^46^ or anti-CTLA-4 mcAb^47^. The gene expression profiles of pre-therapy biopsy samples were curated and transformed into the TPM format for further analysis.

### Construction of the Fersig score

We developed a Fersig scoring scheme to quantify the ferroptosis-associated genes expression level of individual patients by using principal component analysis (PCA) as previously described 58. Firstly, each DEG among NMF clusters FAC1-3 was calculated across all samples in the TCGA cohort. The overlapping DEGs identified from different clusters were selected and used to perform prognostic analysis using a univariate Cox regression model. NMF unsupervised clustering method using prognostic DEGs was employed to classify patients into three gene clusters in the TCGA and meta-GEO cohort. Then we performed PCA analysis in bulk samples using the DEGs correlated to immune and stromal activation respectively, and principal component 1 (PC1) was extracted to serve as the signature score. After obtaining the prognostic value of each PC1 of immune-related and stromal-related DEGs, we applied a method similar to GGI 59 to define the Fersig sore of each patient:

Fersig score= ΣPC1_i_-ΣPC1_j_

where i is the signature score of clusters whose Cox coefficient is positive, and j is the expression of genes whose Cox coefficient is negative. The Fersig score of each database was shown in Table S6.

### Statistical analyses

Statistical analysis was performed using R (version 4.0.0) and GraphPad Prism (version 7.04). The Kruskal-Wallis H test, Pearson's chi-square test, wilcox test and logrank test were used in this study. Detailed descriptions of statistical tests are specified in the figure legends and in the results.

## Supplementary Material

Supplementary figures and tables.

## Abbreviations

CRC: Colorectal cancer
scRNA: single-cell RNA
TMB: Tumor mutation burden
CNV: Copy number variation
EMT: Epithelial-mesenchymal transition
GEO: Gene-expression omnibus
TCGA: The cancer genome atlas
GSVA: Gene set variation analysis
HR: Hazard ratios
ICB: Immunological checkpoint blockade
MSI: Microsatellite instability
dMMR: deficient mismatch repair
DEGs: Differentially expressed genes
ssGSEA: single-sample gene-set enrichment analysis
TGF-β: Transforming growth factor beta
TME: Tumor microenvironment
