# Supplementary material for: Ferroptosis-associated molecular classification characterized by distinct tumor microenvironment profiles in colorectal cancer
Source: Int J Biol Sci. 2022 Feb 7;18(5):1773–94. doi: 10.7150/ijbs.69808 (PMC8935236; doi:10.7150/ijbs.69808)
Supplement: Supplementary file 1 — Supplementary figures and tables. [file ijbsv18p1773s1.pdf]

Supplemental file 1

Ferroptosis-associated molecular classification characterized by distinct tumor microenvironment profiles in colorectal cancer

Authors:

Wenqin Luo[1,2†], Weixing Dai[1,2†], Qingguo Li[1,2†], Shaobo Mo[1,2†], Lingyu Han[1,2], Xiuying Xiao[3], Ruiqi Gu[1,2], Wenqiang Xiang[1,2], Li Ye[1,2], Renjie Wang[1,2], Ye Xu[1,2], Sanjun Cai[1,2], Guoxiang Cai[1,2*]

[1] Department of Colorectal Surgery, Fudan University Shanghai Cancer Center, Shanghai, China.

[2] Department of Oncology, Shanghai Medical College, Fudan University, Shanghai, China.

[3] State Key Laboratory of Oncogenes and Related Genes, Shanghai Cancer Institute, Department of Oncology, Renji Hospital, School of Medicine, Shanghai Jiao Tong University, Shanghai, China.

[†] Wenqin Luo, Weixing Dai, Qingguo Li and Shaobo Mo contributed equally to this work.

Correspondence:

Guoxiang Cai, MD, PhD.

Department of Colorectal Surgery, Fudan University Shanghai Cancer Center Department of Oncology, Shanghai Medical College, Fudan University

270 Dong'an Road, Shanghai, 200032, China

E-mail: gxcaifuscc@163.com

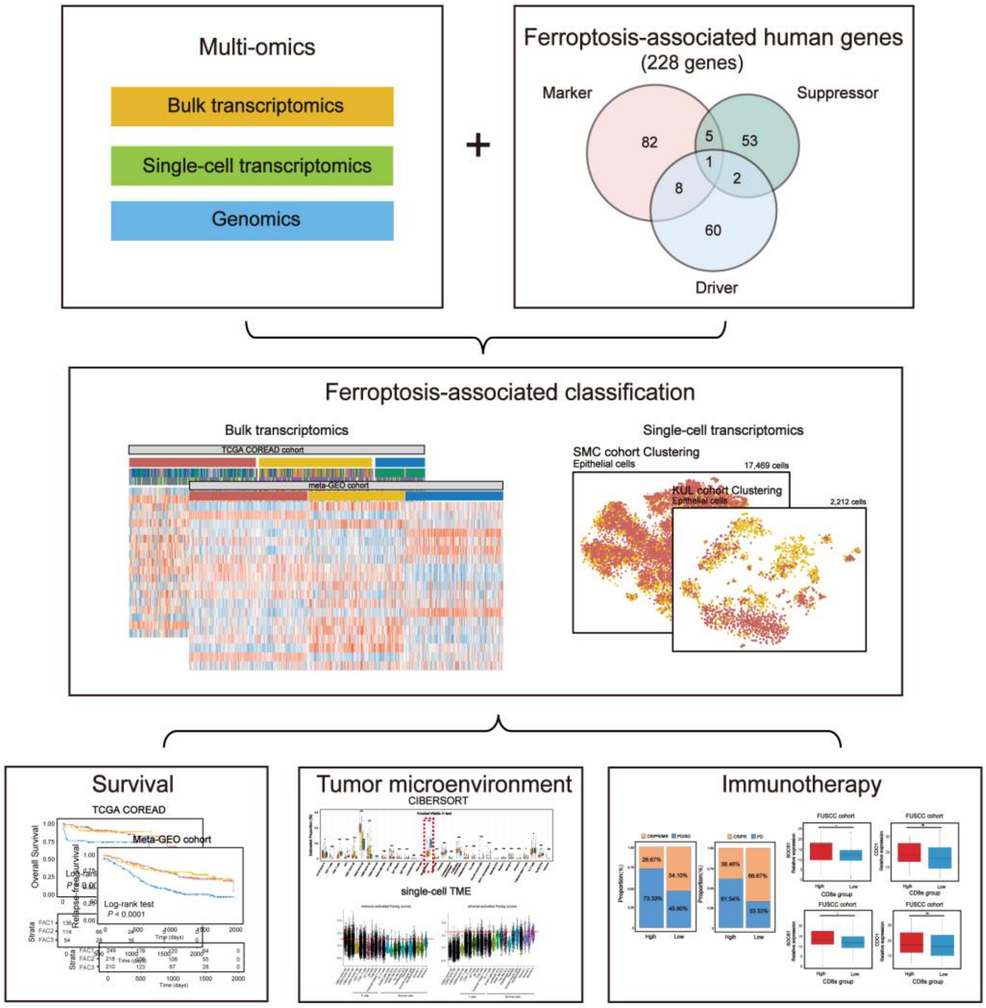

Figure S1. The entire analytical process of the study.

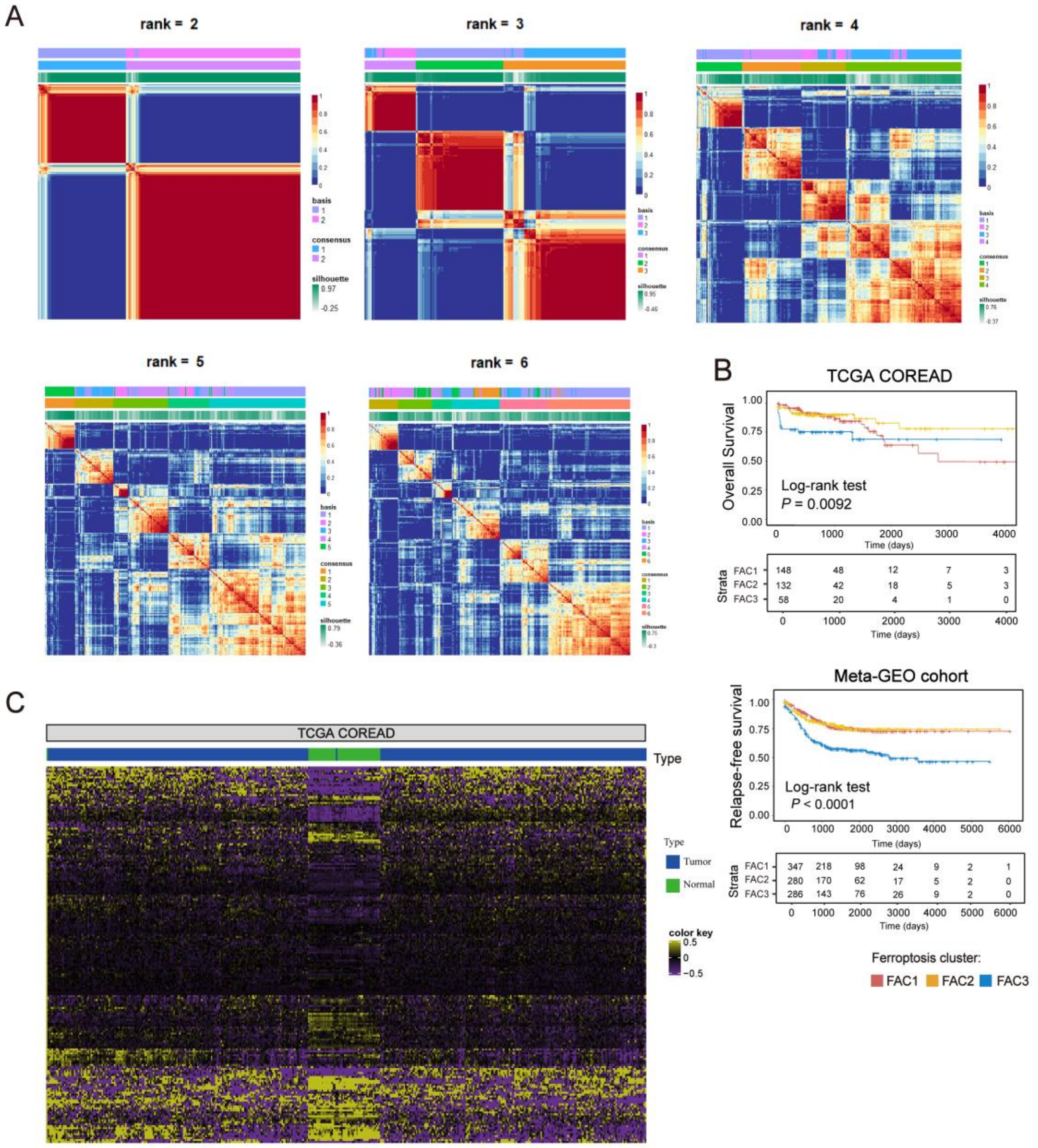

Figure S2. Ferroptosis-associated molecular classification in colorectal cancer, related to Figure 1

(A) Heatmap representation of NMF clustering for ferroptosis-associated genes in TCGA cohort with cluster numbers from 2 to 6. **(B)** Kaplan-Meier curves for overall survival of three NMF clusters in TCGA and for relapse-free survival of three clusters in meta-GEO cohort. The P value was calculated by the log-rank test. **(C)** Heatmap shows expression

of ferroptosis-associated genes in tumor and normal samples.

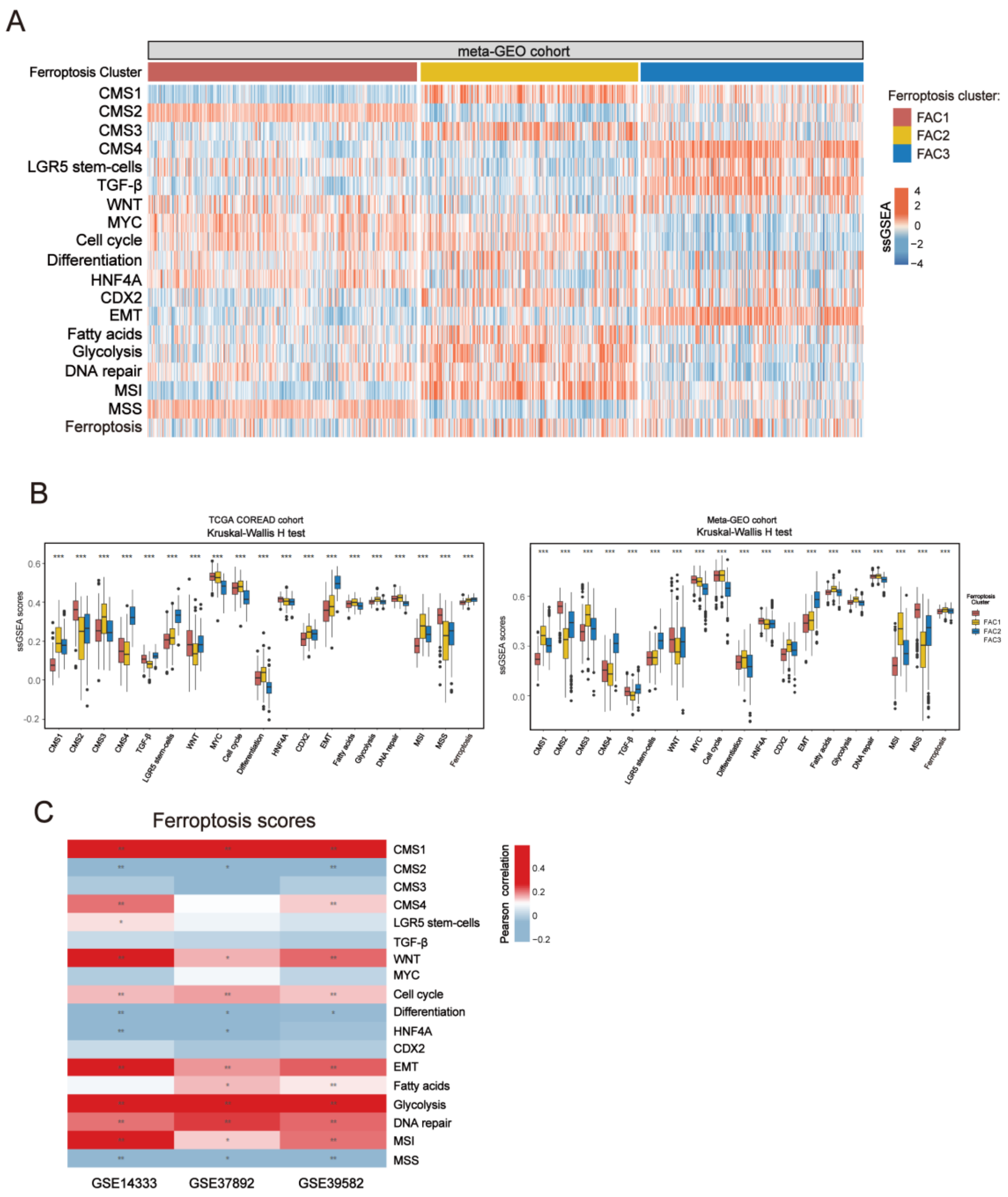

Figure S3. Clinical characteristics and biological molecular changes underlying three clusters in CRC, related to Figure 2

(A) Heatmap shows molecular characteristics of three clusters in meta-GEO cohort. **(B)** Quantification of gene signatures related to CMS

subtypes among three clusters of TCGA and meta-GEO cohort. The statistical difference of three clusters was compared through the Kruskal-Wallis H test. *P < 0.05; **P < 0.01; ***P < 0.001. **(C)** Heatmap shows correlation between ferroptosis score and gene signatures.

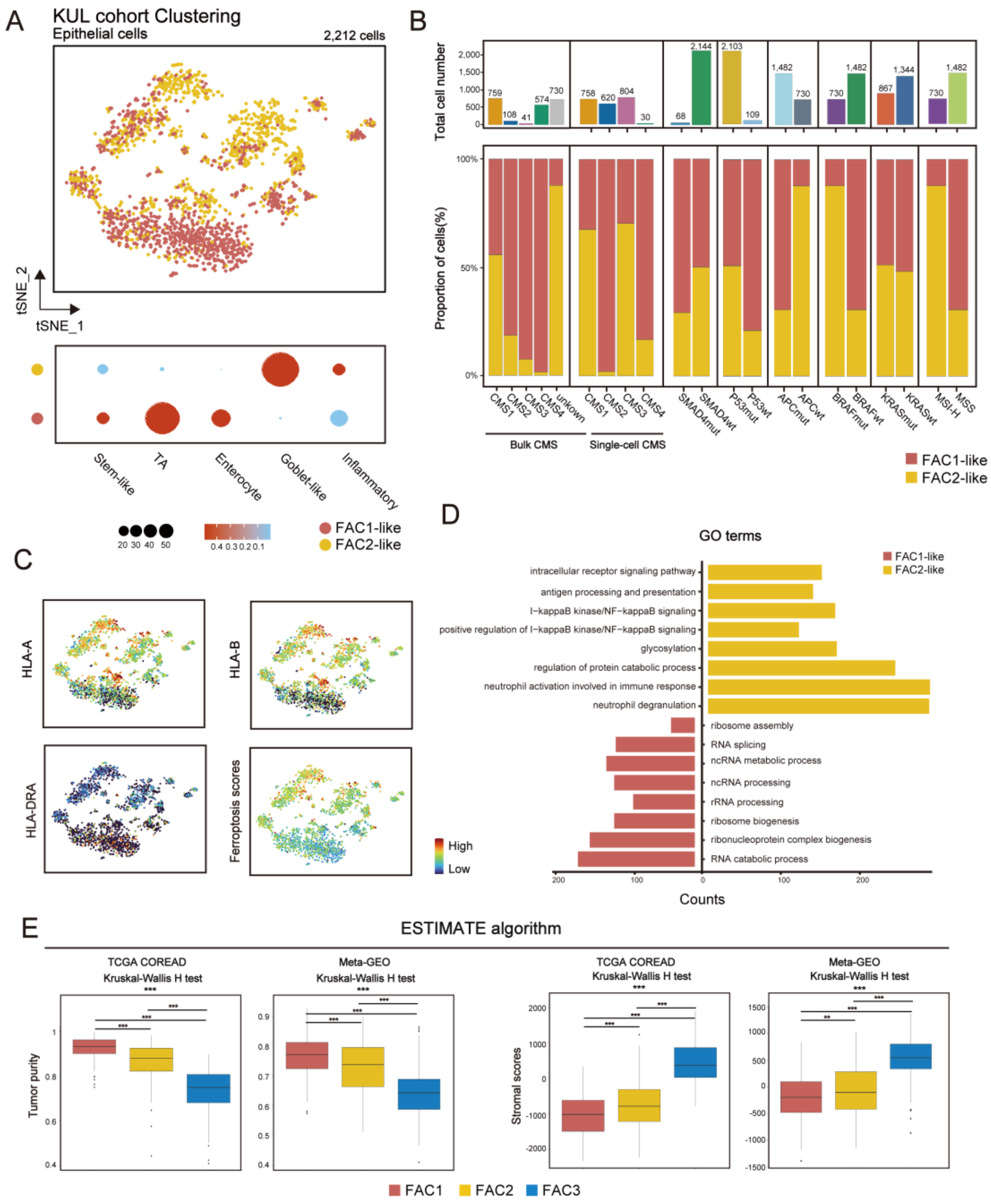

Figure S4. Single-cell transcriptome profiling of CRC cells based on

ferroptosis-associated molecular classification, related to Figure 3

(A) t-SNE visualization of 2,212 tumor epithelial cells from KUL single-cell cohort (top panel). Cells are colored according to clusters. Dot plot for the score of gene signatures associated with CRC cellular phenotype and responses to therapy in each cell type. Color represents the mean score in each cell cluster, and size indicates the fraction of cells expressing gene score (bottom panel). **(B)** Barplot shows the proportion of different molecular characteristics in CRC tumor cells. Bars are colored according to clusters. **(C)** t-SNE visualization of MHC-I (HLA-A, HLA-B), MHC-II (HLA-DRA) molecular expression and the score of ferroptosis-associated genes. (D) GO analysis of differential expressed genes between FAC1-like and FAC2-like CRC tumor cells. **(E)** Tumor purity and stromal score of three ferroptosis-associated clusters in TCGA and meta-GEO cohort. The statistical difference of three clusters was compared through the Kruskal-Wallis H test. *P < 0.05; **P < 0.01; ***P < 0.001. The difference of two clusters was compared through the wilcox test. *P < 0.05; **P < 0.01; ***P < 0.001.

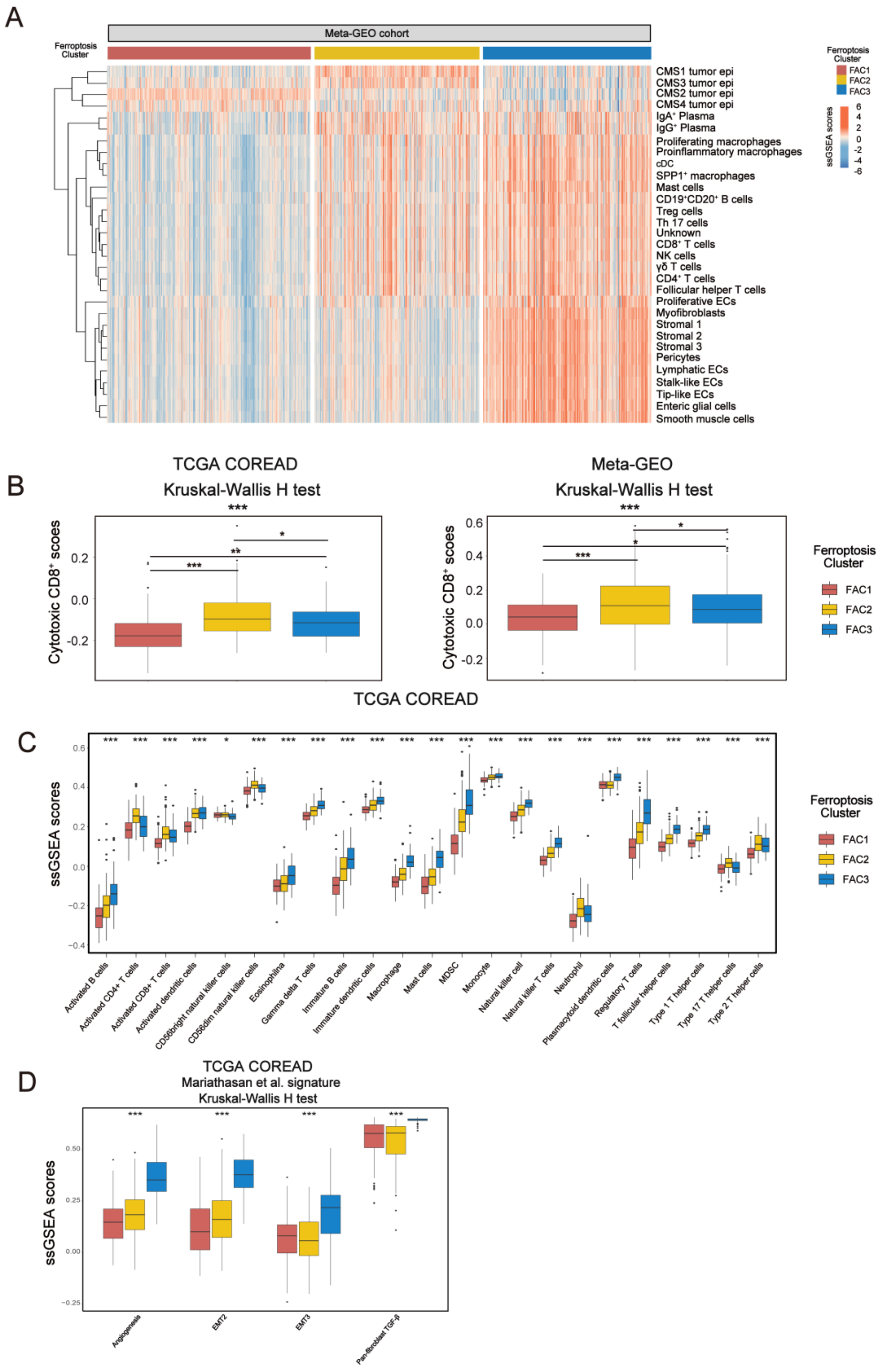

Figure S5. Distinct tumor microenvironment infiltration in three tumor clusters, related to Figure 4

(A) Heatmap shows the ssGSEA score of 31 cell subtypes in three ferroptosis-associated clusters in meta-GEO cohort. **(B-D)** ssGSEA score of corresponding signatures among three clusters in TCGA and meta-GEO cohort. The statistical difference of three clusters was compared through the Kruskal-Wallis H test. *P < 0.05; **P < 0.01; ***P < 0.001.

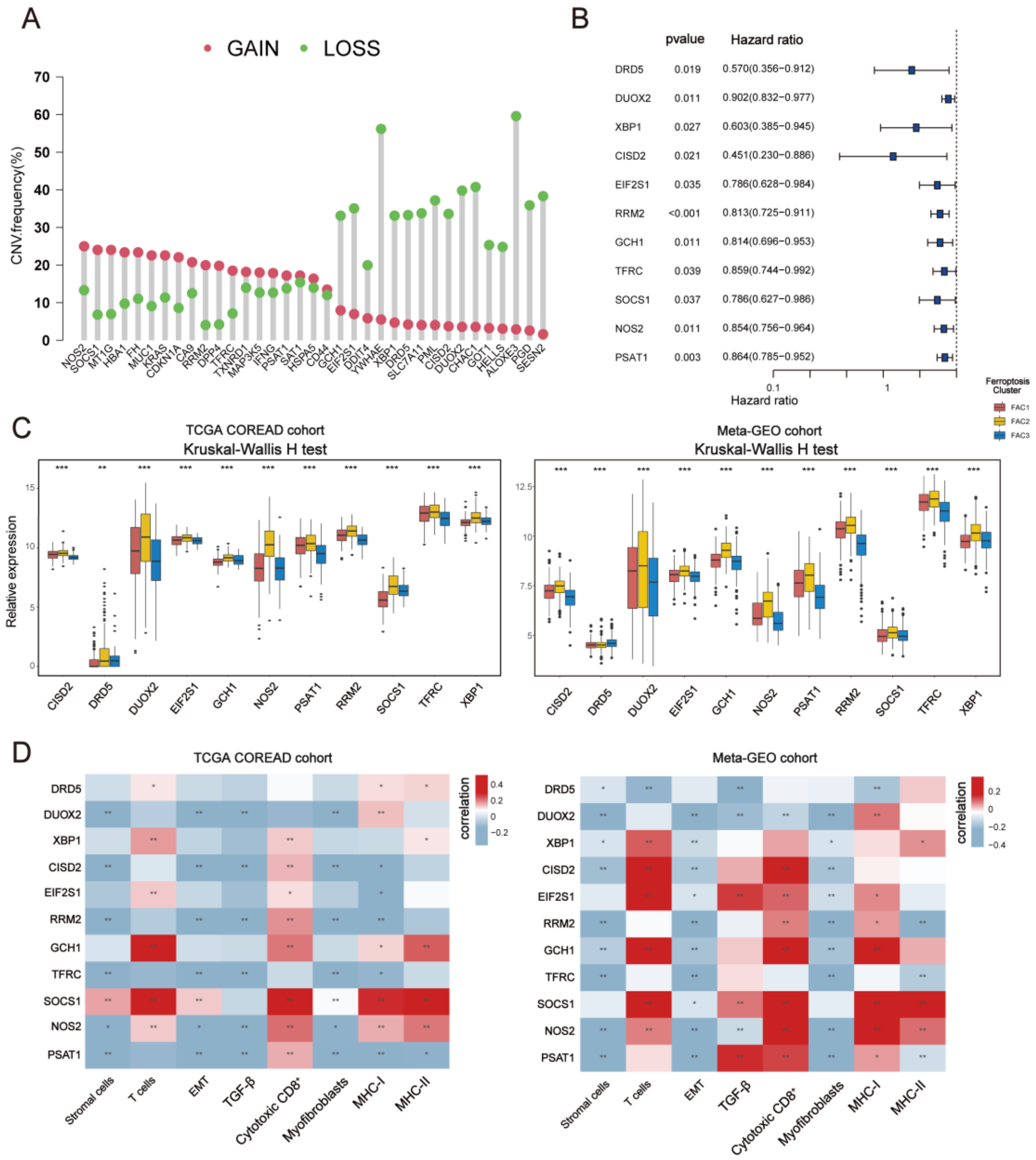

Figure S6. Identifying specific ferroptosis-associated genes correlated with immune activation, related to Figure 5

(A) CNV frequency of immune-activated Fersig in CRC tumors. **(B)** Univariate analysis of immune-activated Fersig in CRC patients. **(C)** Expression of immune-activated Fersig among three clusters. The statistical difference of three clusters was compared through the Kruskal-Wallis H test. *P < 0.05; **P < 0.01; ***P < 0.001. **(D)** Correlation between immune-activated Fersig and different gene signatures, including stromal cells, T cells, cytotoxic $CD8^+$ T, myofibroblasts, EMT, TGF-β, MHC-I and MHC-II.

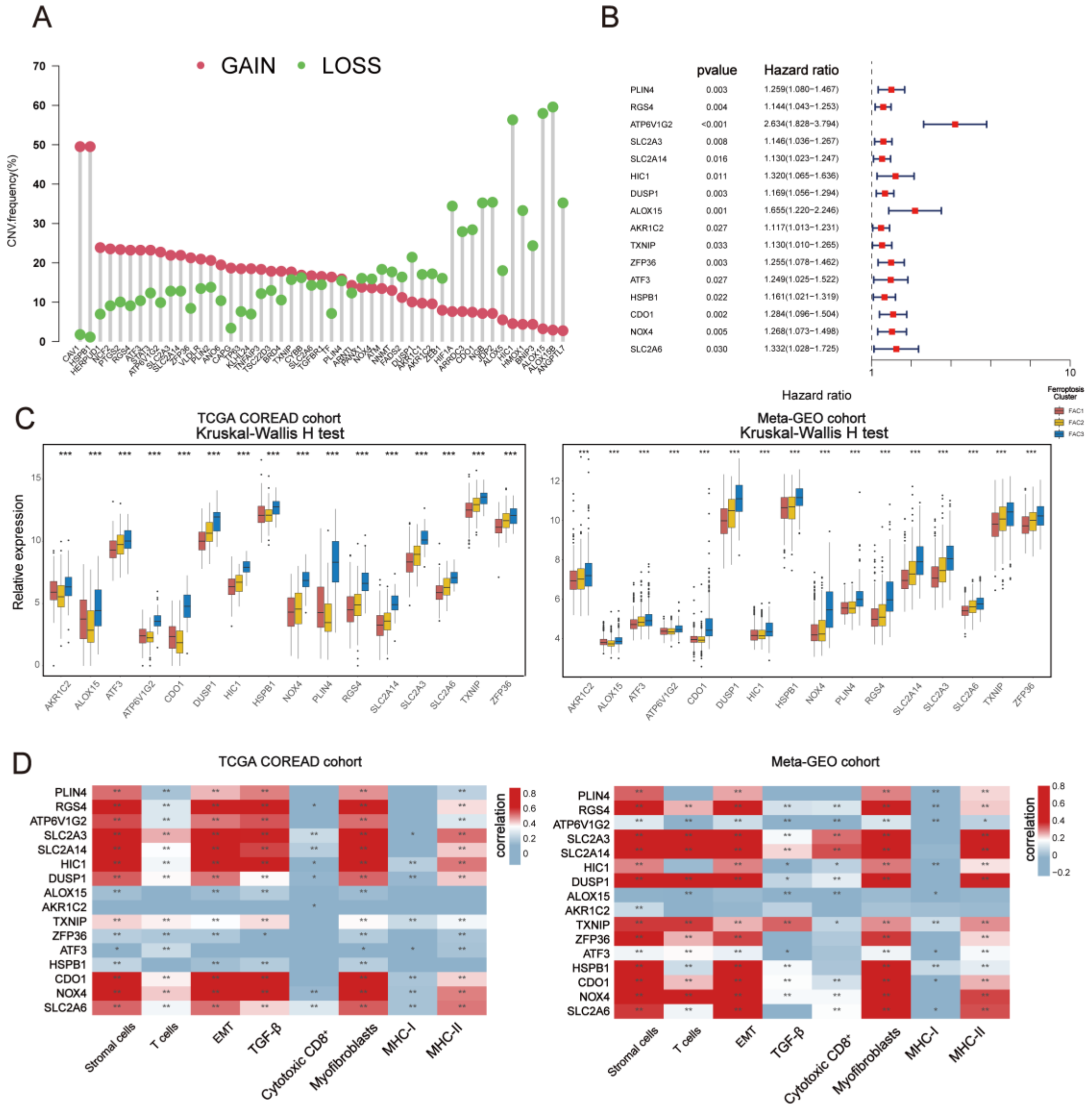

Figure S7. Identifying specific ferroptosis-associated genes correlated with stromal activation, related to Figure 6

(A) CNV frequency of stromal-activated Fersig in CRC tumors. **(B)** Univariate analysis of stromal-activated Fersig in CRC patients. **(C)** Expression of stromal-activated Fersig among three clusters. The statistical difference of three clusters was compared through the Kruskal-Wallis H test. *P < 0.05; **P < 0.01; ***P < 0.001. **(D)** Correlation between stromal-activated Fersig and different gene

signatures, including stromal cells, T cells, cytotoxic CD8$^+$ T, myofibroblasts, EMT, TGF-β, MHC-I and MHC-II.

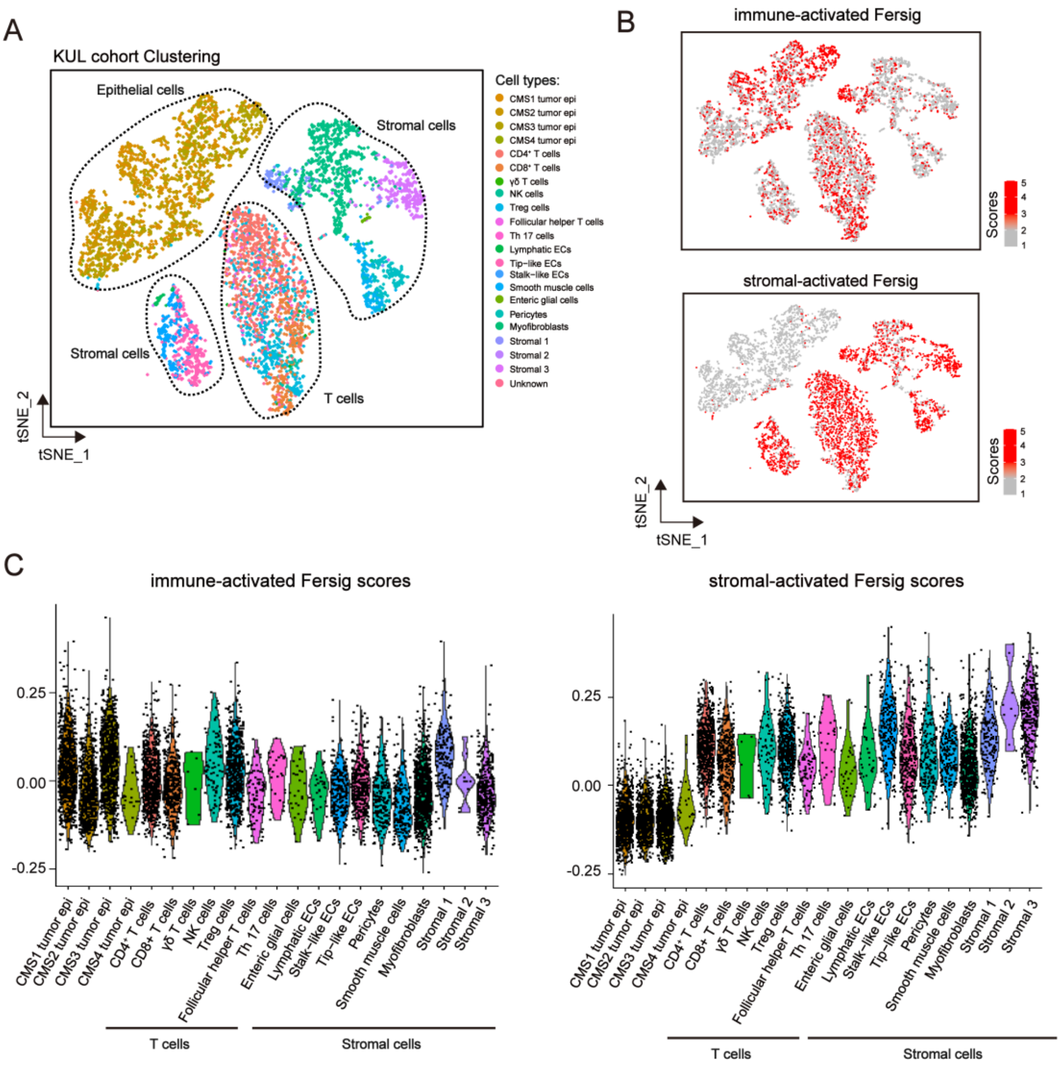

Figure S8. Examining expression of immune-activated and stromal-activated Fersig at single-cell level, related to Figure 7

(A) t-SNE visualization of 6,470 single cells of CRC in KUL cohort. Cells are colored according to cell types. **(B)** t-SNE visualization of score of immune-activated Fersig and stromal-activated Fersig. **(C)** Violin plots shows the score of immune-activated Fersig and stromal-activated Fersig

among different cell subtypes.

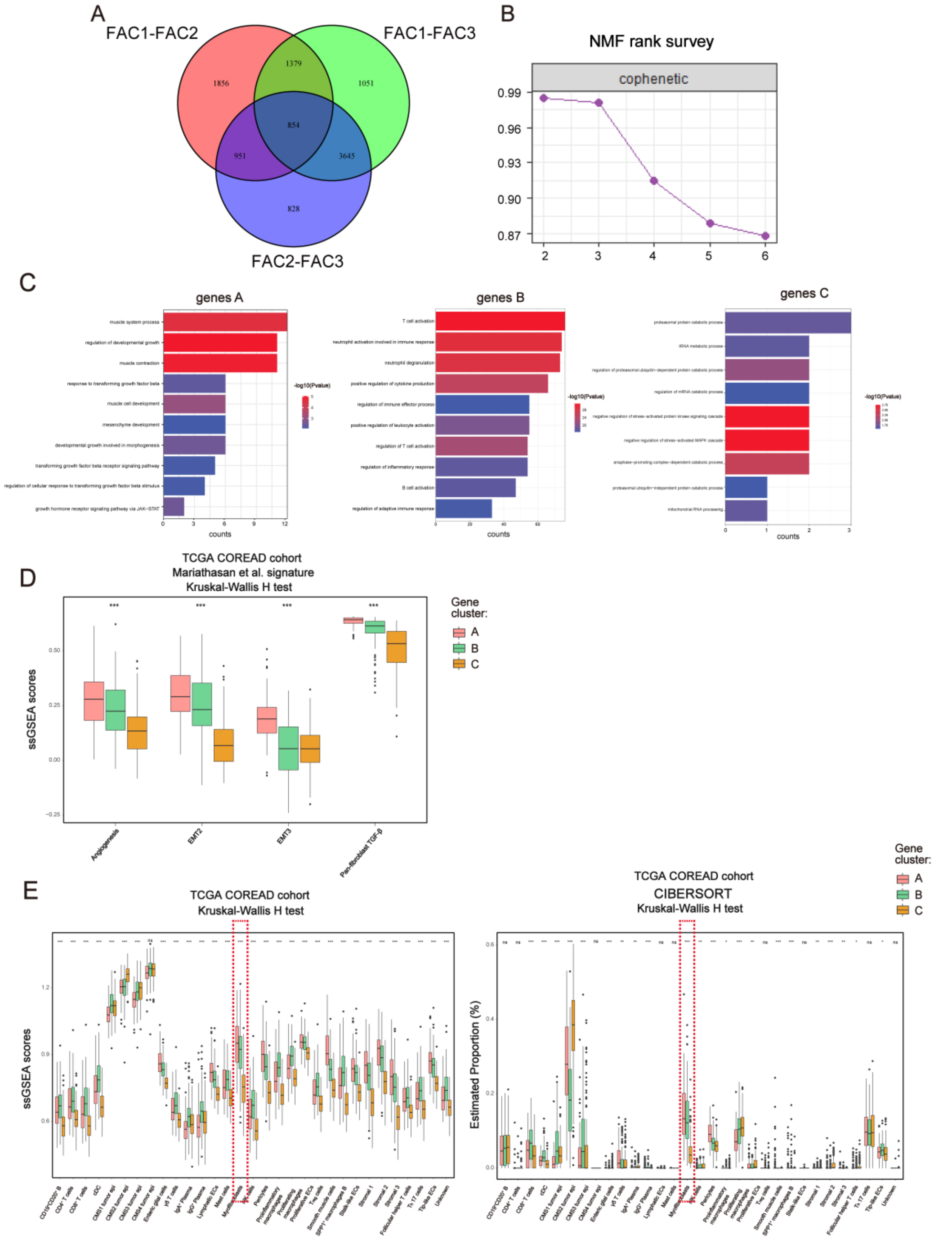

Figure S9. Ferroptosis phenotype-related DEGs in colorectal cancer, related to Figure 8

(A) Venn diagram shows shared genes between DEGs among three ferroptosis-associated clusters. **(B)** NMF rank survey was shown. The optimal number of clusters: k=3. **(C)** GO analyses of genes A-B. **(D-E)** ssGSEA score of corresponding signatures among three gene clusters.

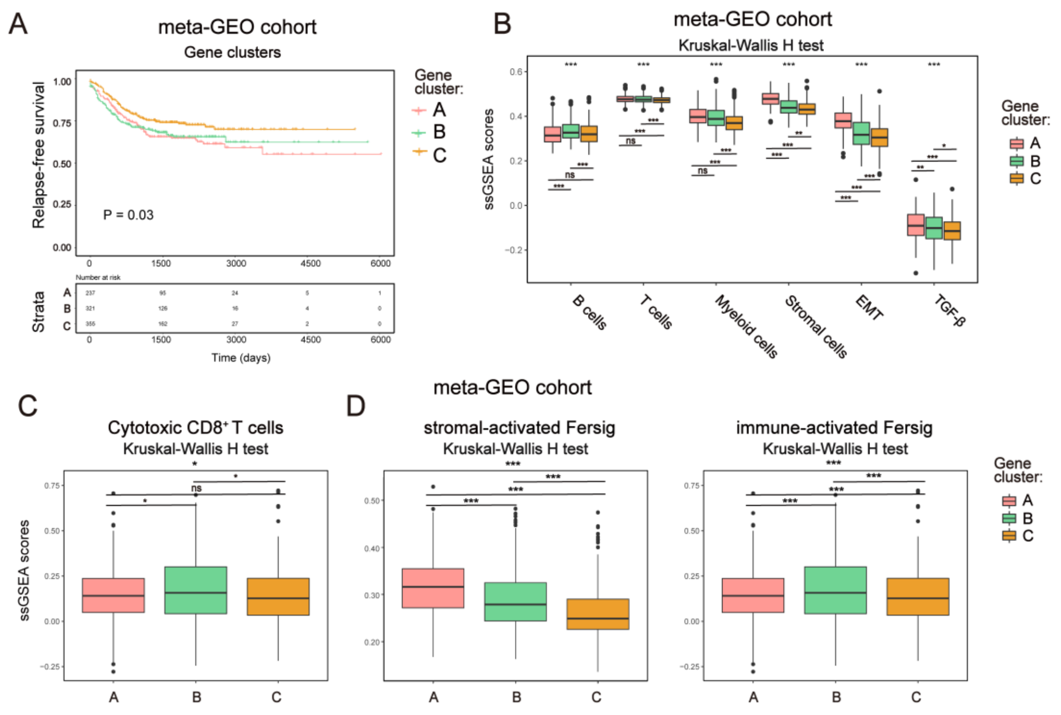

Figure S10. Ferroptosis phenotype-related DEGs in colorectal cancer, related to Figure 8

(A) Kaplan-Meier curves for overall survival of three gene clusters in meta-GEO cohort. The P value was calculated by the log-rank test. **(B)** ssGSEA score of signatures of TME cell types, EMT and TGF-β among three gene clusters in TCGA. The statistical difference of three clusters was compared through the Kruskal-Wallis H test. *P < 0.05; **P < 0.01; ***P < 0.001. The difference of two clusters was compared through the

wilcox test. *P < 0.05; **P < 0.01; ***P < 0.001. **(C-D)** ssGSEA score of signatures of cytotoxic CD8+ T cells, immune-activated and stromal-activated ferroptosis-associated genes among three gene clusters. The statistical difference of three clusters was compared through the Kruskal-Wallis H test. *P < 0.05; **P < 0.01; ***P < 0.001. The difference of two clusters was compared through the wilcox test. *P < 0.05; **P < 0.01; ***P < 0.001.

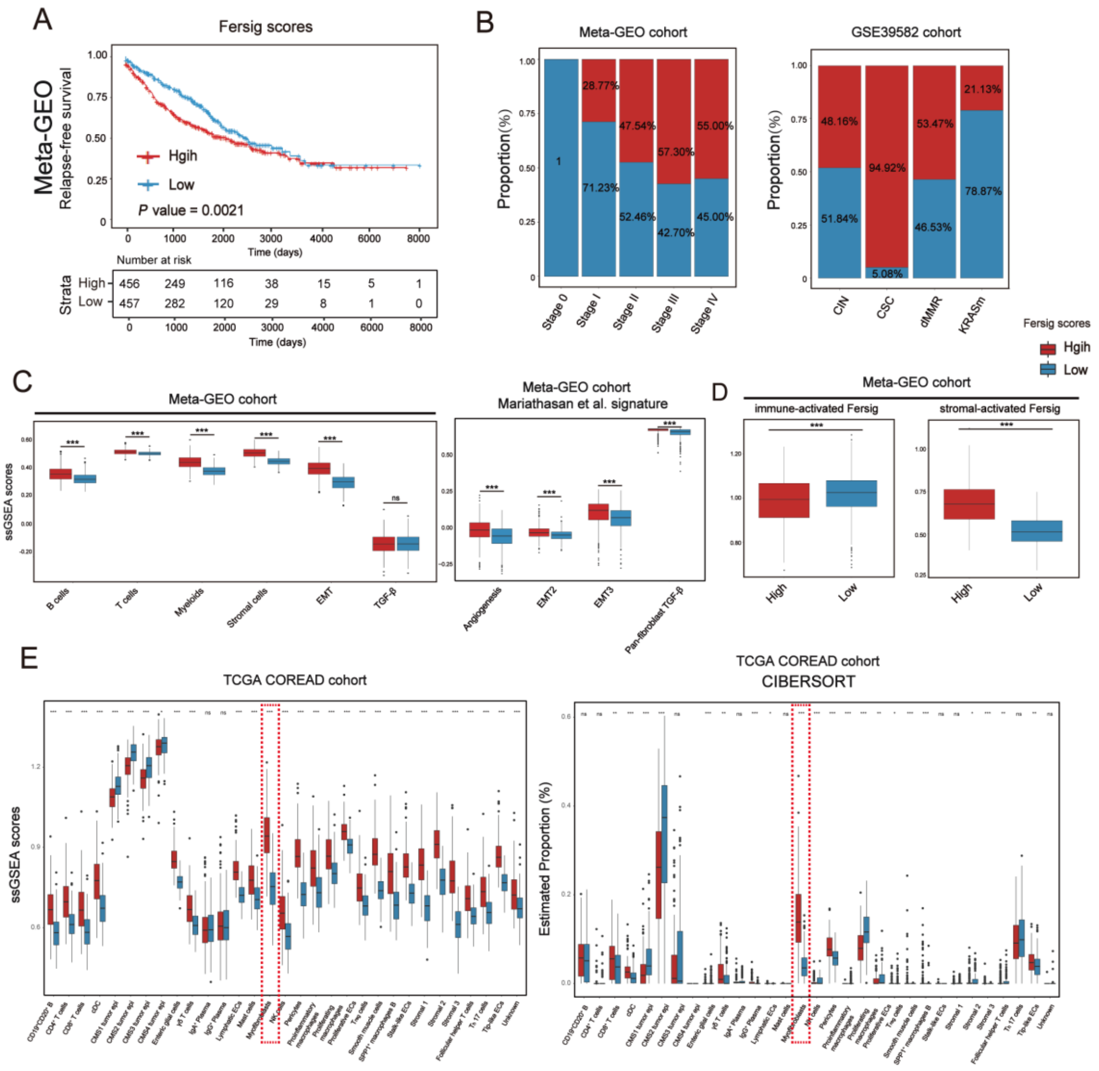

Figure S11. Further construction of Fersig score, related to Figure 9

(A) Kaplan-Meier curves for relapse-free survival of meta-GEO. The high and low groups were divided by the median value of the PCA score of Fersig. The P value was calculated by the log-rank test. **(B)** Barplots show the proportion of molecular characteristics between high and low groups of Fersig. **(C)** ssGSEA score of signatures of TME cell types, EMT, TGF-β and other stromal-related signatures between high and low groups in meta-GEO cohort. The difference of two clusters was compared through the wilcox test. *P < 0.05; **P < 0.01; ***P < 0.001. **(D)** ssGSEA score of immune-activated and stromal-activated Fersig between high and low groups in meta-GEO cohort. The difference of two clusters was compared through the wilcox test. *P < 0.05; **P < 0.01; ***P < 0.001. **(E)** ssGSEA score of 31 cell subtypes between high and low groups in meta-GEO cohort. The difference of two clusters was compared through the wilcox test. *P < 0.05; **P < 0.01; ***P < 0.001.

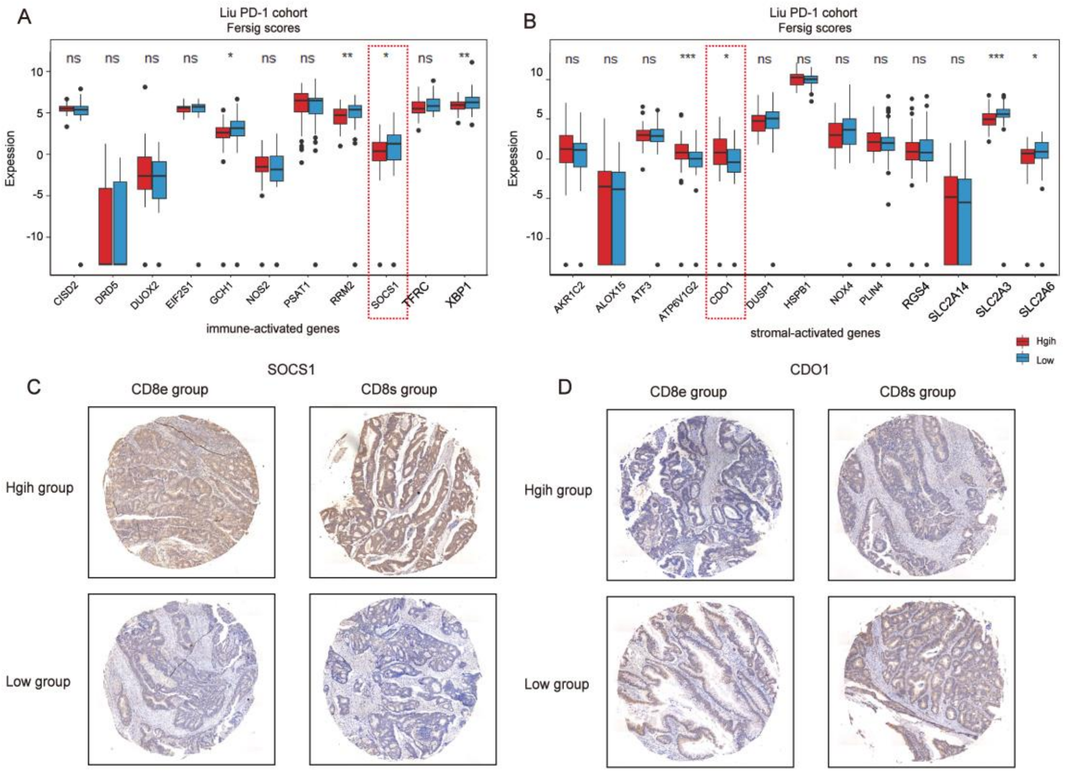

Figure S12. Expression of immune-activated and stromal-activated Fersig with significant prognosis in the Liu et al. cohort.

(A-B) Barplots show the expression of immune-activated and stromal-activated Fersig with significant prognosis in the Liu et al. cohort. The difference of two clusters was compared through the wilcox test. *P < 0.05; **P < 0.01; ***P < 0.001.

B. Supplementary Tables

Supplementary Table S1. The description of patients with bulk RNA expression data, including clinical characteristics and NMF clusters.

Please see separated Excel spreadsheet file.

Supplementary Table S2. The basic information for single-cell datasets.

Please see separated Excel spreadsheet file.

Supplementary Table S3. The biological information of ferroptosis-associated genes.

Please see separated Excel spreadsheet file.

Supplementary Table S4. The cell type annotation of single cells.

Please see separated Excel spreadsheet file.

Supplementary Table S5. Gene clusters in TCGA and meta-GEO cohorts, and genes A-C.

Please see separated Excel spreadsheet file.

Supplementary Table S6. The Fersig score of each database.

Please see separated Excel spreadsheet file.

Table S1. Basic information of bulk databases (TCGA, GSE14333, GSE39582 and (

samples	MSI status	Metastasis status	Stage
TCGA.3L.AA1B.01	MSS	M0	Stage I
TCGA.4N.A93T.01	MSS	M0	Stage III
TCGA.4T.AA8H.01	Indeterminate	MX	Stage II
TCGA.5M.AATE.01	MSS	M0	Stage II
TCGA.A6.5656.01	NA	M0	Stage I
TCGA.A6.5659.01	NA	M0	Stage I
TCGA.A6.5660.01	MSS	M0	Stage III
TCGA.A6.5662.01	MSS	M1	Stage IV
TCGA.A6.5666.01	MSI-L	M0	Stage II
TCGA.A6.5667.01	MSS	MX	Stage III
TCGA.A6.6140.01	MSS	M0	Stage II
TCGA.A6.6648.01	MSS	M1a	Stage IV
TCGA.A6.6652.01	MSS	M1	Stage IV
TCGA.A6.A56B.01	MSS	M0	Stage III
TCGA.AA.3496.01	MSI-L	M0	Stage II
TCGA.AA.3509.01	MSS	M0	Stage II
TCGA.AA.3511.01	MSS	M0	Stage II
TCGA.AA.3660.01	MSS	M0	Stage II
TCGA.AA.3662.01	MSS	M1	Stage IV
TCGA.AA.3675.01	MSS	M0	Stage II
TCGA.AA.A01X.01	MSS	M0	Stage III
TCGA.AD.6888.01	MSS	M0	Stage III
TCGA.AD.6890.01	MSS	MX	NA
TCGA.AD.6965.01	MSS	M0	Stage III
TCGA.AD.A5EK.01	MSS	MX	Stage I
TCGA.AF.3911.01	MSI-L	MX	Stage III
TCGA.AF.4110.01	MSS	MX	Stage IV
TCGA.AF.6136.01	MSS	MX	Stage III
TCGA.AF.6672.01	MSS	MX	Stage IV
TCGA.AF.A56L.01	MSS	M0	Stage III
TCGA.AF.A56N.01	MSS	M0	Stage II
TCGA.AG.3732.01	MSS	M0	Stage I
TCGA.AH.6544.01	MSS	M1	NA
TCGA.AH.6549.01	MSI-L	MX	NA
TCGA.AH.6643.01	MSS	M0	Stage III
TCGA.AH.6897.01	MSI-L	M0	Stage I
TCGA.AH.6903.01	MSS	M0	Stage III
TCGA.AM.5820.01	MSS	M1	Stage IV
TCGA.AY.A54L.01	MSI-L	M0	Stage I
TCGA.AY.A69D.01	MSI-L	M0	Stage II
TCGA.AY.A71X.01	MSS	M0	Stage I
TCGA.AY.A8YK.01	MSS	M1	Stage IV
TCGA.AZ.4684.01	MSI-L	M1	Stage IV
TCGA.CA.5255.01	MSS	M0	Stage II
TCGA.CA.5256.01	MSS	M0	Stage II
TCGA.CA.5797.01	MSS	M0	Stage II
TCGA.CA.6715.01	MSS	M0	Stage III
TCGA.CA.6716.01	MSS	M0	Stage II
TCGA.CI.6619.01	MSS	M1	Stage IV
TCGA.CI.6620.01	MSS	M1	Stage IV
TCGA.CI.6622.01	MSS	M0	Stage II
TCGA.CK.5912.01	MSS	MX	Stage I
TCGA.CK.5915.01	MSS	MX	Stage I
TCGA.CL.4957.01	MSS	M0	NA
TCGA.CL.5917.01	MSS	MX	Stage III
TCGA.CL.5918.01	MSS	MX	Stage II

TCGA.CM.4747.01	MSI-L	M1a	Stage IV
TCGA.CM.5344.01	MSS	M0	Stage III
TCGA.CM.5864.01	MSS	M0	Stage I
TCGA.CM.5868.01	MSS	M1a	Stage IV
TCGA.CM.6161.01	MSI-L	M0	Stage I
TCGA.CM.6163.01	MSS	M0	Stage I
TCGA.CM.6164.01	MSS	M0	Stage II
TCGA.CM.6165.01	MSS	M0	Stage II
TCGA.CM.6166.01	MSS	M0	Stage I
TCGA.CM.6170.01	MSS	M0	Stage I
TCGA.CM.6172.01	MSS	M0	Stage III
TCGA.CM.6676.01	MSI-L	M0	Stage I
TCGA.CM.6678.01	MSS	M1a	Stage IV
TCGA.D5.5537.01	MSS	MX	Stage II
TCGA.D5.5540.01	MSI-L	M0	Stage II
TCGA.D5.5541.01	MSS	M0	Stage III
TCGA.D5.6532.01	MSI-L	M0	Stage II
TCGA.D5.6533.01	MSS	M0	NA
TCGA.D5.6538.01	MSI-L	M0	Stage III
TCGA.D5.6922.01	MSS	M0	Stage III
TCGA.D5.6923.01	MSS	M0	Stage I
TCGA.D5.6924.01	MSS	M0	Stage II
TCGA.D5.6926.01	MSS	M0	Stage III
TCGA.D5.6929.01	MSI-L	M1	Stage IV
TCGA.D5.6932.01	MSS	M0	Stage II
TCGA.DC.4745.01	MSS	M0	Stage III
TCGA.DC.4749.01	MSS	M0	Stage I
TCGA.DC.5869.01	MSS	M0	Stage III
TCGA.DC.6154.01	MSI-H	M1	Stage IV
TCGA.DC.6155.01	MSS	M0	Stage III
TCGA.DC.6157.01	MSS	M0	Stage I
TCGA.DC.6681.01	MSS	M1a	Stage IV
TCGA.DC.6682.01	MSS	M0	Stage II
TCGA.DC.6683.01	MSS	M0	Stage III
TCGA.DM.A1D0.01	MSS	M0	Stage II
TCGA.DM.A1D4.01	MSI-L	M0	Stage II
TCGA.DM.A1D6.01	MSS	M0	Stage II
TCGA.DM.A1D7.01	MSS	M0	Stage II
TCGA.DM.A1D8.01	MSS		NA
TCGA.DM.A1D9.01	MSS	M0	Stage II
TCGA.DM.A1HA.01	MSS	M0	Stage III
TCGA.DM.A282.01	MSS	M0	Stage II
TCGA.DM.A288.01	MSS	M0	Stage III
TCGA.DM.A28A.01	MSS	M0	Stage III
TCGA.DM.A28C.01	MSS	M0	Stage II
TCGA.DM.A28E.01	MSS	M0	Stage II
TCGA.DM.A28F.01	MSS	M0	Stage III
TCGA.DM.A28G.01	MSS	M0	Stage II
TCGA.DM.A28H.01	MSI-L	M0	Stage III
TCGA.DY.A0XA.01	MSS	M0	Stage II
TCGA.DY.A1DD.01	MSI-L	M0	Stage III
TCGA.DY.A1DF.01	MSI-L	M0	Stage III
TCGA.DY.A1H8.01	MSS	M0	Stage III
TCGA.EF.5830.01	MSS	M0	Stage II
TCGA.EF.5831.01	MSS	M0	Stage II
TCGA.EI.6508.01	MSS	M0	Stage III
TCGA.EI.6509.01	MSS	M0	Stage III
TCGA.EI.6512.01	MSS	M0	Stage III

TCGA.EI.6513.01	MSS	M0	Stage III
TCGA.EI.6514.01	MSS	M0	Stage II
TCGA.EI.6881.01	MSS	M0	Stage III
TCGA.EI.6883.01	MSS	M0	Stage II
TCGA.EI.7002.01	MSI-L	M1	Stage IV
TCGA.F4.6463.01	MSS	M0	Stage II
TCGA.F4.6806.01	MSS	M0	Stage I
TCGA.F4.6808.01	MSS	M0	Stage I
TCGA.F4.6854.01	MSS	M0	Stage II
TCGA.F5.6571.01	MSI-L	M0	Stage II
TCGA.F5.6814.01	MSS	M0	Stage II
TCGA.F5.6861.01	MSS	M0	Stage II
TCGA.F5.6863.01	MSI-L	M0	Stage III
TCGA.F5.6864.01	MSI-L	M0	Stage III
TCGA.G4.6303.01	MSI-L	M1	Stage IV
TCGA.G4.6304.01	MSI-H	M0	Stage II
TCGA.G4.6307.01	MSS	M0	Stage III
TCGA.G4.6310.01	MSS	M0	Stage III
TCGA.G4.6315.01	MSS	M1	Stage IV
TCGA.G4.6317.01	MSI-L	MX	Stage III
TCGA.G5.6235.01	MSS	MX	Stage III
TCGA.G5.6641.01	MSS	MX	Stage III
TCGA.NH.A50T.01	MSS	MX	Stage II
TCGA.NH.A6GA.01	MSS	MX	Stage III
TCGA.NH.A6GC.01	MSS	M1b	Stage IV
TCGA.NH.A8F7.01	Indeterminate	MX	Stage II
TCGA.NH.A8F8.01	MSS	M1	Stage IV
TCGA.QG.A5YV.01	MSS	MX	Stage III
TCGA.QG.A5YX.01	MSI-L	MX	Stage II
TCGA.QG.A5Z1.01	MSI-L	MX	Stage III
TCGA.QL.A97D.01	MSS	MX	Stage I
TCGA.RU.A8FL.01	MSS	MX	Stage III
TCGA.SS.A7HO.01	MSS	M0	Stage II
TCGA.T9.A92H.01	MSS	M0	Stage II

GSE37892)

OS (days)	Dead	Sample type	NMF clusters	CMS subtypes
475	0	Tumor	CC1	CMS2
146	0	Tumor	CC1	NA
385	0	Tumor	CC1	CMS3
1200	0	Tumor	CC1	CMS2
1001	0	Tumor	CC1	CMS2
926	0	Tumor	CC1	CMS2
888	0	Tumor	CC1	CMS2
718	0	Tumor	CC1	CMS4
995	0	Tumor	CC1	CMS2
887	0	Tumor	CC1	CMS4
734	0	Tumor	CC1	CMS2
766	0	Tumor	CC1	CMS2
751	0	Tumor	CC1	CMS2
1595	1	Tumor	CC1	CMS4
31	0	Tumor	CC1	CMS4
1915	0	Tumor	CC1	CMS2
212	0	Tumor	CC1	CMS4
2375	0	Tumor	CC1	CMS2
184	0	Tumor	CC1	CMS2
1431	0	Tumor	CC1	CMS3
791	0	Tumor	CC1	CMS2
155	1	Tumor	CC1	CMS2
746	0	Tumor	CC1	CMS2
805	0	Tumor	CC1	CMS2
500	0	Tumor	CC1	NA
1148	0	Tumor	CC1	CMS2
912	0	Tumor	CC1	CMS4
741	0	Tumor	CC1	CMS3
748	0	Tumor	CC1	CMS3
2007	0	Tumor	CC1	CMS2
360	0	Tumor	CC1	CMS2
1003	0	Tumor	CC1	CMS3
1173	0	Tumor	CC1	CMS3
532	0	Tumor	CC1	CMS4
21	1	Tumor	CC1	CMS4
804	0	Tumor	CC1	NA
592	0	Tumor	CC1	CMS3
14	0	Tumor	CC1	NA
525	0	Tumor	CC1	CMS3
543	0	Tumor	CC1	CMS3
588	0	Tumor	CC1	CMS3
573	0	Tumor	CC1	CMS2
1977	0	Tumor	CC1	CMS4
376	0	Tumor	CC1	CMS1
379	0	Tumor	CC1	CMS2
383	0	Tumor	CC1	CMS2
383	0	Tumor	CC1	CMS2
371	0	Tumor	CC1	CMS2
184	0	Tumor	CC1	CMS4
1009	0	Tumor	CC1	NA
1362	0	Tumor	CC1	CMS2
1466	1	Tumor	CC1	NA
0	0	Tumor	CC1	CMS2
425	1	Tumor	CC1	CMS2
2376	0	Tumor	CC1	CMS4
218	0	Tumor	CC1	CMS2

761	0 Tumor	CC1	NA
670	0 Tumor	CC1	CMS4
457	0 Tumor	CC1	CMS2
518	0 Tumor	CC1	CMS4
457	0 Tumor	CC1	CMS2
427	0 Tumor	CC1	NA
883	0 Tumor	CC1	CMS2
488	0 Tumor	CC1	CMS4
669	0 Tumor	CC1	CMS2
457	0 Tumor	CC1	CMS2
335	0 Tumor	CC1	CMS3
337	0 Tumor	CC1	CMS2
335	0 Tumor	CC1	CMS3
456	1 Tumor	CC1	CMS2
1706	0 Tumor	CC1	NA
1701	0 Tumor	CC1	CMS2
555	0 Tumor	CC1	CMS2
775	0 Tumor	CC1	CMS2
521	0 Tumor	CC1	CMS2
308	0 Tumor	CC1	CMS4
378	0 Tumor	CC1	CMS4
435	0 Tumor	CC1	CMS4
275	0 Tumor	CC1	CMS4
408	0 Tumor	CC1	CMS4
346	0 Tumor	CC1	CMS4
639	0 Tumor	CC1	NA
762	0 Tumor	CC1	CMS2
943	0 Tumor	CC1	CMS2
365	0 Tumor	CC1	CMS2
425	0 Tumor	CC1	NA
1581	0 Tumor	CC1	CMS2
790	0 Tumor	CC1	CMS4
762	0 Tumor	CC1	NA
762	0 Tumor	CC1	CMS2
3974	0 Tumor	CC1	CMS2
2821	1 Tumor	CC1	NA
1518	1 Tumor	CC1	CMS3
405	1 Tumor	CC1	NA
383	1 Tumor	CC1	NA
4270	0 Tumor	CC1	NA
4000	0 Tumor	CC1	NA
4233	0 Tumor	CC1	CMS2
427	1 Tumor	CC1	NA
805	1 Tumor	CC1	CMS2
2475	1 Tumor	CC1	NA
3648	0 Tumor	CC1	NA
1094	1 Tumor	CC1	CMS2
1849	1 Tumor	CC1	CMS3
3561	0 Tumor	CC1	NA
3846	0 Tumor	CC1	CMS2
1741	1 Tumor	CC1	CMS2
734	1 Tumor	CC1	CMS4
992	1 Tumor	CC1	NA
106	0 Tumor	CC1	CMS2
127	0 Tumor	CC1	CMS4
636	0 Tumor	CC1	CMS3
517	0 Tumor	CC1	CMS4
538	0 Tumor	CC1	CMS1

497	0	Tumor	CC1	CMS2
496	0	Tumor	CC1	CMS4
499	0	Tumor	CC1	CMS2
350	0	Tumor	CC1	CMS2
364	0	Tumor	CC1	CMS2
1087	0	Tumor	CC1	CMS4
1260	0	Tumor	CC1	CMS3
1024	0	Tumor	CC1	CMS2
16	0	Tumor	CC1	CMS2
1288	0	Tumor	CC1	CMS4
1131	0	Tumor	CC1	CMS1
1160	0	Tumor	CC1	CMS2
23	1	Tumor	CC1	CMS4
14	0	Tumor	CC1	CMS4
1882	1	Tumor	CC1	CMS4
1631	0	Tumor	CC1	NA
1674	0	Tumor	CC1	CMS2
1935	0	Tumor	CC1	CMS4
1883	0	Tumor	CC1	CMS2
1095	0	Tumor	CC1	CMS2
1696	0	Tumor	CC1	CMS2
804	0	Tumor	CC1	CMS3
553	0	Tumor	CC1	CMS2
182	1	Tumor	CC1	CMS1
389	0	Tumor	CC1	CMS3
543	0	Tumor	CC1	CMS2
167	1	Tumor	CC1	CMS4
1301	0	Tumor	CC1	CMS2
1003	0	Tumor	CC1	CMS3
202	1	Tumor	CC1	CMS4
666	0	Tumor	CC1	NA
1177	0	Tumor	CC1	NA
1829	0	Tumor	CC1	NA
362	0	Tumor	CC1	CMS2

Table S1. Basic information of bulk databases (TCGA, GSE14333, GSE39582 and GSE378

samples	Stage	RFS (days)	RFS.event	Sample type
GSM358341	stage I	109.2	0	Tumor
GSM358342	stage I	435.9	1	Tumor
GSM358343	stage I	494.1	0	Tumor
GSM358344	stage I	592.5	0	Tumor
GSM358345	stage I	600.6	0	Tumor
GSM358346	stage I	718.8	0	Tumor
GSM358347	stage I	810.6	1	Tumor
GSM358348	stage I	868.8	0	Tumor
GSM358349	stage I	1313.7	0	Tumor
GSM358350	stage I	1567.2	0	Tumor
GSM358351	stage I	1653.9	0	Tumor
GSM358352	stage I	1733.7	0	Tumor
GSM358353	stage I	1785.9	0	Tumor
GSM358354	stage I	1923	0	Tumor
GSM358355	stage I	1931.1	0	Tumor
GSM358356	stage I	1976.4	0	Tumor
GSM358357	stage I	2015.7	0	Tumor
GSM358358	stage I	2034.6	0	Tumor
GSM358359	stage I	2055.3	0	Tumor
GSM358360	stage I	2282.1	0	Tumor
GSM358361	stage I	2468.7	0	Tumor
GSM358362	stage I	2508	0	Tumor
GSM358363	stage I	2511.9	0	Tumor
GSM358364	stage I	2523.9	0	Tumor
GSM358365	stage I	3276.3	0	Tumor
GSM358366	stage I	3576.3	0	Tumor
GSM358367	stage I	3681.6	0	Tumor
GSM358368	stage I	4276.5	0	Tumor
GSM358370	stage I	684	0	Tumor
GSM358371	stage I	432	0	Tumor
GSM358373	stage I	1632	0	Tumor
GSM358374	stage I	1935	0	Tumor
GSM358375	stage I	54	0	Tumor
GSM358376	stage I	1260	0	Tumor
GSM358377	stage I	1695	0	Tumor
GSM358378	stage I	1089	0	Tumor
GSM358380	stage I	1326	0	Tumor
GSM358381	stage I	1563	0	Tumor
GSM358382	stage I	900	0	Tumor
GSM358383	stage I	1317	0	Tumor
GSM358384	stage I	2424	0	Tumor
GSM358385	stage II	67.8	0	Tumor
GSM358386	stage II	180.3	1	Tumor
GSM358387	stage II	298.8	1	Tumor
GSM358388	stage II	423.9	1	Tumor
GSM358389	stage II	427.8	1	Tumor
GSM358390	stage II	451.5	0	Tumor
GSM358391	stage II	471.3	0	Tumor
GSM358392	stage II	538.5	0	Tumor
GSM358393	stage II	545.4	0	Tumor
GSM358394	stage II	580.8	1	Tumor
GSM358395	stage II	660.6	0	Tumor
GSM358396	stage II	671.4	1	Tumor
GSM358397	stage II	671.4	1	Tumor
GSM358398	stage II	725.7	0	Tumor
GSM358399	stage II	756.3	1	Tumor

GSM358400	stage II	757.2	0	Tumor
GSM358401	stage II	768.3	0	Tumor
GSM358402	stage II	795.9	0	Tumor
GSM358403	stage II	799.8	0	Tumor
GSM358404	stage II	804.6	0	Tumor
GSM358405	stage II	814.5	0	Tumor
GSM358406	stage II	829.2	1	Tumor
GSM358407	stage II	858.9	0	Tumor
GSM358408	stage II	865.8	0	Tumor
GSM358409	stage II	876.6	0	Tumor
GSM358410	stage II	931.8	0	Tumor
GSM358411	stage II	996	0	Tumor
GSM358412	stage II	1010.7	0	Tumor
GSM358413	stage II	1077	0	Tumor
GSM358414	stage II	1102.5	0	Tumor
GSM358415	stage II	1119.3	1	Tumor
GSM358416	stage II	1119.3	0	Tumor
GSM358417	stage II	1177.5	0	Tumor
GSM358418	stage II	1349.1	0	Tumor
GSM358419	stage II	1401.3	0	Tumor
GSM358420	stage II	1435.8	0	Tumor
GSM358421	stage II	1495.2	0	Tumor
GSM358422	stage II	1564.2	0	Tumor
GSM358423	stage II	1575	0	Tumor
GSM358424	stage II	1659.9	0	Tumor
GSM358425	stage II	1677.6	0	Tumor
GSM358426	stage II	1740.6	0	Tumor
GSM358427	stage II	1788	0	Tumor
GSM358428	stage II	1798.8	0	Tumor
GSM358429	stage II	1897.5	0	Tumor
GSM358430	stage II	1945.8	0	Tumor
GSM358431	stage II	2119.5	0	Tumor
GSM358432	stage II	2281.2	0	Tumor
GSM358433	stage II	2373.9	0	Tumor
GSM358434	stage II	2407.5	0	Tumor
GSM358435	stage II	2558.4	0	Tumor
GSM358436	stage II	2592.9	0	Tumor
GSM358437	stage II	2669.7	0	Tumor
GSM358438	stage II	2809.8	1	Tumor
GSM358439	stage II	3208.2	0	Tumor
GSM358440	stage II	3323.7	0	Tumor
GSM358441	stage II	3557.4	0	Tumor
GSM358442	stage II	363	0	Tumor
GSM358443	stage II	888	0	Tumor
GSM358444	stage II	1110	0	Tumor
GSM358445	stage II	855	0	Tumor
GSM358446	stage II	1689	0	Tumor
GSM358447	stage II	1422	0	Tumor
GSM358448	stage II	405	0	Tumor
GSM358449	stage II	1704	0	Tumor
GSM358450	stage II	1584	0	Tumor
GSM358451	stage II	1359	0	Tumor
GSM358452	stage II	1671	0	Tumor
GSM358453	stage II	1200	0	Tumor
GSM358454	stage II	426	1	Tumor
GSM358455	stage II	123	0	Tumor
GSM358456	stage II	1785	0	Tumor
GSM358457	stage II	1287	0	Tumor

GSM358458	stage II	978	1	Tumor
GSM358459	stage II	939	0	Tumor
GSM358460	stage II	1017	0	Tumor
GSM358461	stage II	1431	0	Tumor
GSM358462	stage II	1146	0	Tumor
GSM358463	stage II	1332	0	Tumor
GSM358464	stage II	1344	0	Tumor
GSM358465	stage II	1710	0	Tumor
GSM358466	stage II	1212	0	Tumor
GSM358467	stage II	156	0	Tumor
GSM358468	stage II	348	0	Tumor
GSM358469	stage II	1752	0	Tumor
GSM358470	stage II	1086	0	Tumor
GSM358471	stage II	1362	0	Tumor
GSM358472	stage II	1527	0	Tumor
GSM358473	stage II	1677	0	Tumor
GSM358474	stage II	429	1	Tumor
GSM358475	stage II	1014	0	Tumor
GSM358476	stage II	1140	0	Tumor
GSM358477	stage II	2226	0	Tumor
GSM358478	stage II	2547	0	Tumor
GSM358479	stage III	27.6	0	Tumor
GSM358480	stage III	47.1	1	Tumor
GSM358481	stage III	99.6	1	Tumor
GSM358482	stage III	127.2	0	Tumor
GSM358483	stage III	156.6	0	Tumor
GSM358484	stage III	160.5	1	Tumor
GSM358485	stage III	246.3	1	Tumor
GSM358486	stage III	249.3	1	Tumor
GSM358487	stage III	257.4	1	Tumor
GSM358488	stage III	286.8	1	Tumor
GSM358489	stage III	357	1	Tumor
GSM358490	stage III	441.6	1	Tumor
GSM358491	stage III	482.1	1	Tumor
GSM358492	stage III	567.9	1	Tumor
GSM358493	stage III	568.8	0	Tumor
GSM358494	stage III	599.4	0	Tumor
GSM358495	stage III	613.2	0	Tumor
GSM358496	stage III	631.2	1	Tumor
GSM358497	stage III	638.1	1	Tumor
GSM358498	stage III	667.5	0	Tumor
GSM358499	stage III	700.2	1	Tumor
GSM358500	stage III	723.9	1	Tumor
GSM358501	stage III	745.5	1	Tumor
GSM358502	stage III	787.8	0	Tumor
GSM358503	stage III	807.6	0	Tumor
GSM358504	stage III	827.4	1	Tumor
GSM358505	stage III	832.2	1	Tumor
GSM358506	stage III	881.7	1	Tumor
GSM358507	stage III	940.8	0	Tumor
GSM358508	stage III	957.6	0	Tumor
GSM358509	stage III	1025.7	1	Tumor
GSM358510	stage III	1107.6	1	Tumor
GSM358511	stage III	1107.6	0	Tumor
GSM358512	stage III	1142.1	0	Tumor
GSM358513	stage III	1161.6	0	Tumor
GSM358514	stage III	1231.8	1	Tumor
GSM358515	stage III	1340.1	0	Tumor

GSM358516	stage III	1342.2	0 Tumor
GSM358517	stage III	1434	0 Tumor
GSM358518	stage III	1473.3	0 Tumor
GSM358519	stage III	1512.9	0 Tumor
GSM358520	stage III	1517.7	0 Tumor
GSM358521	stage III	1585.8	0 Tumor
GSM358522	stage III	1647	0 Tumor
GSM358523	stage III	1753.5	0 Tumor
GSM358524	stage III	1772.1	0 Tumor
GSM358525	stage III	1780.2	0 Tumor
GSM358526	stage III	1929.9	0 Tumor
GSM358527	stage III	1947.9	0 Tumor
GSM358528	stage III	1966.5	0 Tumor
GSM358529	stage III	2162.7	0 Tumor
GSM358530	stage III	2230.8	0 Tumor
GSM358531	stage III	2235.9	0 Tumor
GSM358532	stage III	2383.8	1 Tumor
GSM358533	stage III	2568.3	0 Tumor
GSM358534	stage III	2575.2	1 Tumor
GSM358535	stage III	2688.6	0 Tumor
GSM358536	stage III	2852.1	0 Tumor
GSM358537	stage III	2985.3	0 Tumor
GSM358538	stage III	3155.1	0 Tumor
GSM358539	stage III	3369.9	0 Tumor
GSM358540	stage III	927	0 Tumor
GSM358541	stage III	720	0 Tumor
GSM358542	stage III	948	0 Tumor
GSM358543	stage III	1488	0 Tumor
GSM358544	stage III	456	1 Tumor
GSM358545	stage III	726	0 Tumor
GSM358546	stage III	2073	0 Tumor
GSM358547	stage III	2043	0 Tumor
GSM358548	stage III	309	1 Tumor
GSM358549	stage III	1776	0 Tumor
GSM358550	stage III	1842	0 Tumor
GSM358551	stage III	450	0 Tumor
GSM358552	stage III	1734	0 Tumor
GSM358553	stage III	564	0 Tumor
GSM358554	stage III	135	1 Tumor
GSM358555	stage III	1143	1 Tumor
GSM358556	stage III	1212	0 Tumor
GSM358557	stage III	171	1 Tumor
GSM358558	stage III	306	0 Tumor
GSM358559	stage III	2541	0 Tumor
GSM358560	stage III	507	0 Tumor
GSM358561	stage III	285	1 Tumor
GSM358562	stage III	2061	0 Tumor
GSM358563	stage III	1785	0 Tumor
GSM358564	stage III	561	0 Tumor
GSM358565	stage III	2124	0 Tumor
GSM358566	stage III	133.2	1 Tumor
GSM358567	stage III	1200	1 Tumor
GSM358568	stage III	2187	1 Tumor
GSM358569	stage III	540	1 Tumor

392)

NMF clusters
CC2
CC3
CC1
CC3
CC1
CC1
CC3
CC1
CC1
CC1
CC3
CC3
CC2
CC2
CC2
CC1
CC2
CC2
CC3
CC2
CC2
CC2
CC1
CC3
CC3
CC2
CC3
CC3
CC2
CC1
CC1
CC2
CC1
CC2
CC2
CC1
CC1
CC2
CC1
CC3
CC3
CC2
CC1
CC2
CC3
CC3
CC3
CC2
CC3
CC1
CC1
CC2
CC1
CC1
CC2
CC1

CC3
CC1
CC1
CC3
CC2
CC3
CC3
CC1
CC1
CC3
CC2
CC3
CC2
CC3
CC2
CC3
CC3
CC3
CC2
CC3
CC1
CC3
CC2
CC2
CC2
CC3
CC3
CC2
CC3
CC2
CC1
CC1
CC1
CC2
CC1
CC3
CC3
CC2
CC3
CC1
CC2
CC1
CC2
CC1
CC2
CC2
CC3
CC2
CC1
CC1
CC2
CC2
CC2
CC2
CC2
CC1
CC1
CC2

CC2
CC2
CC1
CC1
CC3
CC2
CC2
CC2
CC3
CC3
CC1
CC2
CC3
CC1
CC3
CC2
CC1
CC3
CC2
CC2
CC3
CC2
CC2
CC2
CC2
CC1
CC2
CC3
CC3
CC2
CC1
CC2
CC1
CC3
CC3
CC3
CC3
CC1
CC3
CC3
CC2
CC3
CC3
CC3
CC2
CC2
CC2
CC1
CC1
CC3
CC1
CC3
CC3
CC1
CC3
CC3
CC3
CC3

CC1
CC2
CC1
CC2
CC2
CC1
CC1
CC1
CC3
CC3
CC1
CC2
CC1
CC1
CC3
CC1
CC3
CC1
CC3
CC3
CC2
CC1
CC3
CC1
CC1
CC3
CC1
CC2
CC1
CC2
CC2
CC1
CC2
CC2
CC1
CC2
CC1
CC3
CC2
CC1
CC1
CC1
CC3
CC2
CC1
CC1
CC1
CC2
CC2
CC1
CC1
CC3
CC3
CC1

Table S1. Basic information of bulk databases (TCGA, GSE14333, GSE39582 and GSE37[illegible]

samples	Stage	RFS (days)	RFS.event	Sample type
GSM971957	stage IV	0	1	Tumor
GSM971958	stage IV	0	1	Tumor
GSM971959	stage II	120	1	Tumor
GSM971960	stage I	2220	0	Tumor
GSM971961	stage IV	30	1	Tumor
GSM971962	stage III	2190	0	Tumor
GSM971963	stage II	510	1	Tumor
GSM971964	stage III	1050	0	Tumor
GSM971965	stage II	990	0	Tumor
GSM971966	stage III	1890	0	Tumor
GSM971968	stage II	2790	1	Tumor
GSM971969	stage I	1890	0	Tumor
GSM971970	stage I	2580	0	Tumor
GSM971971	stage IV	1950	0	Tumor
GSM971972	stage I	2250	0	Tumor
GSM971973	stage II	1380	0	Tumor
GSM971974	stage IV	1680	0	Tumor
GSM971975	stage II	1380	0	Tumor
GSM971976	stage II	1080	0	Tumor
GSM971977	stage III	1470	0	Tumor
GSM971978	stage I	3270	0	Tumor
GSM971979	stage II	1590	0	Tumor
GSM971980	stage III	1770	0	Tumor
GSM971981	stage II	1440	0	Tumor
GSM971982	stage III	1260	0	Tumor
GSM971983	stage III	2610	0	Tumor
GSM971984	stage II	150	1	Tumor
GSM971985	stage 0	210	0	Tumor
GSM971986	stage II	2040	0	Tumor
GSM971987	stage II	2040	0	Tumor
GSM971988	stage IV	450	1	Tumor
GSM971989	stage I	480	1	Tumor
GSM971990	stage I	360	0	Tumor
GSM971991	stage II	1650	0	Tumor
GSM971992	stage II	600	0	Tumor
GSM971993	stage II	1680	0	Tumor
GSM971994	stage II	1290	0	Tumor
GSM971995	stage II	750	0	Tumor
GSM971996	stage III	2280	0	Tumor
GSM971997	stage III	2670	0	Tumor
GSM971998	stage III	540	1	Tumor
GSM971999	stage III	30	0	Tumor
GSM972000	stage III	270	1	Tumor
GSM972001	stage III	2190	0	Tumor
GSM972002	stage III	750	1	Tumor
GSM972003	stage IV	0	1	Tumor
GSM972004	stage IV	2670	0	Tumor
GSM972005	stage IV	0	1	Tumor
GSM972006	stage IV	0	1	Tumor
GSM972007	stage IV	60	0	Tumor
GSM972008	stage IV	60	1	Tumor
GSM972009	stage IV	0	1	Tumor
GSM972010	stage IV	300	1	Tumor
GSM972011	stage IV	540	1	Tumor
GSM972012	stage IV	3030	0	Tumor
GSM972013	stage IV	1290	0	Tumor

GSM972014	stage IV	0	1	Tumor
GSM972015	stage II	2730	0	Tumor
GSM972016	stage II	420	1	Tumor
GSM972017	stage III	1290	0	Tumor
GSM972018	stage III	3510	0	Tumor
GSM972019	stage III	630	1	Tumor
GSM972020	stage III	270	1	Tumor
GSM972021	stage III	150	1	Tumor
GSM972022	stage III	2460	0	Tumor
GSM972023	stage III	180	1	Tumor
GSM972024	stage III	1110	1	Tumor
GSM972025	stage III	1590	0	Tumor
GSM972026	stage III	960	1	Tumor
GSM972027	stage III	2820	0	Tumor
GSM972028	stage III	2340	0	Tumor
GSM972029	stage III	1080	0	Tumor
GSM972030	stage III	1560	0	Tumor
GSM972031	stage II	1650	0	Tumor
GSM972032	stage II	1800	0	Tumor
GSM972033	stage II	1500	0	Tumor
GSM972034	stage II	1500	0	Tumor
GSM972035	stage II	1170	0	Tumor
GSM972036	stage II	1500	0	Tumor
GSM972037	stage II	990	0	Tumor
GSM972038	stage II	30	0	Tumor
GSM972039	stage IV	0	1	Tumor
GSM972040	stage II	1890	0	Tumor
GSM972041	stage II	1650	0	Tumor
GSM972042	stage II	1530	0	Tumor
GSM972043	stage IV	0	1	Tumor
GSM972044	stage II	1260	0	Tumor
GSM972045	stage IV	0	1	Tumor
GSM972046	stage II	630	1	Tumor
GSM972047	stage II	990	0	Tumor
GSM972048	stage IV	0	1	Tumor
GSM972049	stage II	780	0	Tumor
GSM972050	stage II	570	0	Tumor
GSM972051	stage IV	0	1	Tumor
GSM972052	stage II	450	0	Tumor
GSM972053	stage II	870	0	Tumor
GSM972054	stage II	780	0	Tumor
GSM972055	stage II	2790	0	Tumor
GSM972056	stage II	2760	0	Tumor
GSM972057	stage II	780	0	Tumor
GSM972058	stage II	1800	0	Tumor
GSM972059	stage II	1770	0	Tumor
GSM972060	stage II	2460	0	Tumor
GSM972061	stage II	1290	0	Tumor
GSM972062	stage II	0	1	Tumor
GSM972063	stage II	2100	0	Tumor
GSM972064	stage IV	0	1	Tumor
GSM972065	stage II	1380	0	Tumor
GSM972066	stage IV	0	1	Tumor
GSM972067	stage II	5490	0	Tumor
GSM972068	stage II	90	1	Tumor
GSM972069	stage II	6030	0	Tumor
GSM972070	stage II	2040	0	Tumor
GSM972071	stage II	5490	0	Tumor

GSM972072	stage II	5160	0 Tumor
GSM972073	stage II	4890	0 Tumor
GSM972074	stage II	30	1 Tumor
GSM972075	stage II	1320	0 Tumor
GSM972076	stage II	4590	0 Tumor
GSM972077	stage II	780	1 Tumor
GSM972078	stage II	1620	1 Tumor
GSM972079	stage II	5040	0 Tumor
GSM972080	stage II	1590	1 Tumor
GSM972081	stage II	780	0 Tumor
GSM972082	stage II	3570	1 Tumor
GSM972083	stage II	3810	0 Tumor
GSM972084	stage II	3870	0 Tumor
GSM972085	stage II	2910	0 Tumor
GSM972086	stage II	1290	0 Tumor
GSM972087	stage II	240	1 Tumor
GSM972088	stage II	2370	0 Tumor
GSM972089	stage II	2610	0 Tumor
GSM972090	stage II	2070	0 Tumor
GSM972091	stage II	1350	1 Tumor
GSM972092	stage II	1410	0 Tumor
GSM972093	stage II	2220	0 Tumor
GSM972094	stage II	1830	0 Tumor
GSM972095	stage II	2010	0 Tumor
GSM972096	stage III	90	1 Tumor
GSM972097	stage III	360	1 Tumor
GSM972098	stage III	450	0 Tumor
GSM972099	stage III	450	0 Tumor
GSM972100	stage III	750	0 Tumor
GSM972101	stage III	630	1 Tumor
GSM972102	stage III	1380	0 Tumor
GSM972103	stage III	840	1 Tumor
GSM972104	stage III	1230	0 Tumor
GSM972105	stage III	960	0 Tumor
GSM972106	stage III	270	1 Tumor
GSM972107	stage III	1800	0 Tumor
GSM972108	stage III	3090	0 Tumor
GSM972109	stage III	1080	0 Tumor
GSM972110	stage III	120	1 Tumor
GSM972111	stage III	390	1 Tumor
GSM972112	stage III	960	0 Tumor
GSM972113	stage III	540	1 Tumor
GSM972114	stage III	1380	0 Tumor
GSM972115	stage III	330	1 Tumor
GSM972116	stage III	60	1 Tumor
GSM972117	stage III	330	1 Tumor
GSM972118	stage III	570	1 Tumor
GSM972119	stage III	750	1 Tumor
GSM972120	stage III	1080	0 Tumor
GSM972121	stage III	1050	0 Tumor
GSM972122	stage III	570	1 Tumor
GSM972123	stage II	2130	0 Tumor
GSM972124	stage II	2250	0 Tumor
GSM972125	stage II	420	1 Tumor
GSM972126	stage II	1890	0 Tumor
GSM972127	stage II	2550	0 Tumor
GSM972128	stage II	2790	0 Tumor
GSM972129	stage II	330	0 Tumor

GSM972130	stage II	1650	0 Tumor
GSM972131	stage II	2580	0 Tumor
GSM972132	stage II	1830	0 Tumor
GSM972133	stage III	810	1 Tumor
GSM972134	stage III	210	1 Tumor
GSM972135	stage II	120	1 Tumor
GSM972136	stage III	1830	1 Tumor
GSM972137	stage II	270	1 Tumor
GSM972138	stage II	1350	1 Tumor
GSM972139	stage III	1140	1 Tumor
GSM972140	stage II	1680	1 Tumor
GSM972141	stage III	450	1 Tumor
GSM972142	stage III	60	1 Tumor
GSM972143	stage II	4620	0 Tumor
GSM972144	stage II	3630	0 Tumor
GSM972145	stage III	4050	0 Tumor
GSM972146	stage III	1980	0 Tumor
GSM972147	stage II	4200	0 Tumor
GSM972148	stage III	3810	0 Tumor
GSM972149	stage II	3960	0 Tumor
GSM972150	stage III	3270	0 Tumor
GSM972151	stage III	2310	0 Tumor
GSM972152	stage III	1680	0 Tumor
GSM972153	stage II	1980	0 Tumor
GSM972154	stage III	90	1 Tumor
GSM972155	stage III	180	1 Tumor
GSM972156	stage III	3360	0 Tumor
GSM972157	stage III	3540	0 Tumor
GSM972158	stage III	3600	0 Tumor
GSM972159	stage III	2790	0 Tumor
GSM972160	stage II	1170	1 Tumor
GSM972161	stage II	270	1 Tumor
GSM972162	stage III	2310	0 Tumor
GSM972163	stage II	1230	0 Tumor
GSM972164	stage III	90	1 Tumor
GSM972165	stage II	1080	1 Tumor
GSM972166	stage III	90	1 Tumor
GSM972167	stage II	1080	1 Tumor
GSM972168	stage III	120	1 Tumor
GSM972169	stage II	4920	0 Tumor
GSM972170	stage III	2190	0 Tumor
GSM972171	stage III	3900	0 Tumor
GSM972172	stage II	120	1 Tumor
GSM972173	stage II	450	1 Tumor
GSM972174	stage III	2910	0 Tumor
GSM972175	stage III	840	1 Tumor
GSM972176	stage III	1050	1 Tumor
GSM972177	stage II	600	1 Tumor
GSM972178	stage III	630	1 Tumor
GSM972179	stage II	510	1 Tumor
GSM972180	stage II	570	1 Tumor
GSM972181	stage III	390	1 Tumor
GSM972182	stage II	690	1 Tumor
GSM972183	stage III	330	1 Tumor
GSM972184	stage II	120	1 Tumor
GSM972185	stage III	660	1 Tumor
GSM972186	stage III	420	1 Tumor
GSM972187	stage II	270	1 Tumor

GSM972188	stage III	330	1	Tumor
GSM972189	stage III	5760	0	Tumor
GSM972190	stage II	3240	0	Tumor
GSM972191	stage III	3660	0	Tumor
GSM972192	stage II	4740	0	Tumor
GSM972193	stage III	2190	0	Tumor
GSM972194	stage III	2490	0	Tumor
GSM972195	stage II	3600	0	Tumor
GSM972196	stage II	2190	0	Tumor
GSM972197	stage II	1500	0	Tumor
GSM972198	stage III	1710	0	Tumor
GSM972199	stage III	1980	0	Tumor
GSM972200	stage II	1620	0	Tumor
GSM972201	stage III	2010	0	Tumor
GSM972202	stage III	1590	0	Tumor
GSM972203	stage III	1620	0	Tumor
GSM972204	stage II	120	1	Tumor
GSM972205	stage II	210	1	Tumor
GSM972206	stage II	0	1	Tumor
GSM972207	stage III	1020	1	Tumor
GSM972208	stage IV	0	1	Tumor
GSM972209	stage IV	0	1	Tumor
GSM972210	stage IV	0	1	Tumor
GSM972211	stage III	1710	0	Tumor
GSM972212	stage IV	0	1	Tumor
GSM972213	stage IV	0	1	Tumor
GSM972214	stage IV	0	1	Tumor
GSM972215	stage III	1410	0	Tumor
GSM972216	stage IV	0	1	Tumor
GSM972217	stage IV	0	1	Tumor
GSM972218	stage IV	0	1	Tumor
GSM972219	stage IV	0	1	Tumor
GSM972220	stage III	30	0	Tumor
GSM972221	stage II	1950	0	Tumor
GSM972222	stage II	1710	0	Tumor
GSM972223	stage II	990	0	Tumor
GSM972224	stage III	600	1	Tumor
GSM972225	stage II	1110	0	Tumor
GSM972226	stage II	600	0	Tumor
GSM972227	stage IV	600	0	Tumor
GSM972228	stage II	30	0	Tumor
GSM972229	stage II	0	0	Tumor
GSM972230	stage III	810	0	Tumor
GSM972231	stage IV	420	0	Tumor
GSM972232	stage IV	630	0	Tumor
GSM972233	stage II	450	0	Tumor
GSM972234	stage IV	300	0	Tumor
GSM972235	stage III	210	0	Tumor
GSM972236	stage IV	360	0	Tumor
GSM972237	stage I	750	0	Tumor
GSM972238	stage III	570	0	Tumor
GSM972239	stage II	420	0	Tumor
GSM972240	stage IV	420	0	Tumor
GSM972241	stage II	510	0	Tumor
GSM972242	stage I	510	0	Tumor
GSM972243	stage II	60	0	Tumor
GSM972244	stage I	420	0	Tumor
GSM972245	stage IV	0	0	Tumor

GSM972246	stage III	0	0 Tumor
GSM972247	stage II	180	0 Tumor
GSM972248	stage II	420	0 Tumor
GSM972249	stage I	420	0 Tumor
GSM972250	stage III	360	0 Tumor
GSM972251	stage IV	210	0 Tumor
GSM972252	stage IV	480	0 Tumor
GSM972253	stage IV	480	0 Tumor
GSM972254	stage IV	270	1 Tumor
GSM972255	stage II	150	0 Tumor
GSM972256	stage IV	390	1 Tumor
GSM972257	stage II	270	1 Tumor
GSM972258	stage II	0	0 Tumor
GSM972259	stage II	870	0 Tumor
GSM972260	stage IV	390	0 Tumor
GSM972261	stage III	210	0 Tumor
GSM972262	stage II	150	0 Tumor
GSM972263	stage III	2280	0 Tumor
GSM972264	stage II	2190	0 Tumor
GSM972265	stage III	1800	0 Tumor
GSM972266	stage IV	600	0 Tumor
GSM972267	stage II	2010	0 Tumor
GSM972268	stage III	1440	0 Tumor
GSM972269	stage I	2370	0 Tumor
GSM972270	stage II	1950	0 Tumor
GSM972271	stage III	390	1 Tumor
GSM972272	stage IV	450	0 Tumor
GSM972273	stage 0	480	0 Tumor
GSM972274	stage III	1710	0 Tumor
GSM972275	stage II	90	1 Tumor
GSM972276	stage III	1290	0 Tumor
GSM972277	stage II	1530	0 Tumor
GSM972278	stage I	1350	0 Tumor
GSM972279	stage III	1560	0 Tumor
GSM972280	stage II	1380	0 Tumor
GSM972281	stage I	1680	0 Tumor
GSM972282	stage I	1530	0 Tumor
GSM972283	stage II	1530	0 Tumor
GSM972284	stage IV	810	0 Tumor
GSM972285	stage II	60	0 Tumor
GSM972286	stage II	1110	0 Tumor
GSM972287	stage II	1290	0 Tumor
GSM972288	stage IV	1050	0 Tumor
GSM972289	stage IV	510	0 Tumor
GSM972290	stage I	1170	0 Tumor
GSM972291	stage III	300	1 Tumor
GSM972292	stage II	750	0 Tumor
GSM972293	stage I	1500	0 Tumor
GSM972294	stage II	750	0 Tumor
GSM972295	stage III	1830	0 Tumor
GSM972296	stage 0	660	0 Tumor
GSM972297	stage I	1380	0 Tumor
GSM972298	stage II	270	0 Tumor
GSM972299	stage IV	1230	0 Tumor
GSM972300	stage 0	1230	0 Tumor
GSM972301	stage III	3150	0 Tumor
GSM972302	stage III	810	0 Tumor
GSM972303	stage I	570	0 Tumor

GSM972304	stage II	900	0 Tumor
GSM972305	stage III	930	0 Tumor
GSM972306	stage II	1680	0 Tumor
GSM972307	stage II	2460	0 Tumor
GSM972308	stage III	2760	0 Tumor
GSM972309	stage II	2850	0 Tumor
GSM972310	stage III	750	0 Tumor
GSM972311	stage II	3180	0 Tumor
GSM972312	stage II	2910	0 Tumor
GSM972313	stage I	2730	0 Tumor
GSM972314	stage III	1380	0 Tumor
GSM972315	stage I	2520	0 Tumor
GSM972316	stage II	2490	0 Tumor
GSM972317	stage II	150	1 Tumor
GSM972318	stage II	1080	0 Tumor
GSM972319	stage II	2340	0 Tumor
GSM972320	stage III	300	1 Tumor
GSM972321	stage III	270	0 Tumor
GSM972322	stage II	1980	1 Tumor
GSM972323	stage III	2400	0 Tumor
GSM972324	stage II	2460	0 Tumor
GSM972325	stage III	1590	1 Tumor
GSM972326	stage II	2550	0 Tumor
GSM972327	stage II	2490	0 Tumor
GSM972328	stage III	2340	1 Tumor
GSM972329	stage II	2340	0 Tumor
GSM972330	stage II	2580	0 Tumor
GSM972331	stage II	2250	0 Tumor
GSM972332	stage II	2610	0 Tumor
GSM972333	stage II	2400	0 Tumor
GSM972334	stage III	2310	0 Tumor
GSM972335	stage I	1380	0 Tumor
GSM972336	stage III	1500	0 Tumor
GSM972337	stage III	1230	0 Tumor
GSM972338	stage II	2220	0 Tumor
GSM972339	stage III	2280	0 Tumor
GSM972340	stage II	1440	1 Tumor
GSM972341	stage III	2070	0 Tumor
GSM972342	stage I	840	0 Tumor
GSM972343	stage II	1560	0 Tumor
GSM972344	stage II	2250	0 Tumor
GSM972345	stage III	1230	1 Tumor
GSM972346	stage III	2160	0 Tumor
GSM972347	stage II	210	0 Tumor
GSM972348	stage II	1860	0 Tumor
GSM972349	stage III	150	0 Tumor
GSM972351	stage II	720	0 Tumor
GSM972352	stage III	2220	1 Tumor
GSM972353	stage II	3990	0 Tumor
GSM972354	stage III	690	1 Tumor
GSM972355	stage II	510	1 Tumor
GSM972357	stage II	1260	0 Tumor
GSM972358	stage II	2430	0 Tumor
GSM972359	stage II	360	1 Tumor
GSM972360	stage III	4230	0 Tumor
GSM972361	stage III	540	1 Tumor
GSM972362	stage III	2940	0 Tumor
GSM972363	stage III	4200	0 Tumor

GSM972364	stage III	3090	0	Tumor
GSM972365	stage III	1410	0	Tumor
GSM972366	stage III	420	1	Tumor
GSM972367	stage III	2850	0	Tumor
GSM972368	stage III	2760	0	Tumor
GSM972369	stage III	540	1	Tumor
GSM972370	stage III	960	1	Tumor
GSM972371	stage III	2430	0	Tumor
GSM972372	stage III	990	1	Tumor
GSM972373	stage III	1110	1	Tumor
GSM972374	stage III	2700	0	Tumor
GSM972375	stage III	3510	0	Tumor
GSM972376	stage III	2610	0	Tumor
GSM972377	stage III	2460	0	Tumor
GSM972378	stage III	2580	0	Tumor
GSM972379	stage III	2850	0	Tumor
GSM972380	stage III	180	1	Tumor
GSM972381	stage II	810	1	Tumor
GSM972382	stage II	1110	1	Tumor
GSM972383	stage II	2760	0	Tumor
GSM972384	stage II	3180	0	Tumor
GSM972385	stage II	1890	0	Tumor
GSM972386	stage II	2700	0	Tumor
GSM972387	stage II	720	1	Tumor
GSM972388	stage II	2490	0	Tumor
GSM972389	stage II	2790	0	Tumor
GSM972390	stage III	180	1	Tumor
GSM972391	stage II	1290	1	Tumor
GSM972392	stage II	630	1	Tumor
GSM972393	stage II	150	1	Tumor
GSM972394	stage III	180	1	Tumor
GSM972395	stage III	4410	0	Tumor
GSM972396	stage II	3210	0	Tumor
GSM972397	stage II	3150	0	Tumor
GSM972398	stage II	1920	0	Tumor
GSM972399	stage III	2700	0	Tumor
GSM972400	stage IV	240	1	Tumor
GSM972401	stage II	0	0	Tumor
GSM972402	stage II	0	1	Tumor
GSM972403	stage II	1680	0	Tumor
GSM972404	stage II	1440	0	Tumor
GSM972405	stage II	2370	0	Tumor
GSM972406	stage II	3180	0	Tumor
GSM972407	stage II	3960	0	Tumor
GSM972408	stage II	960	0	Tumor
GSM972409	stage II	3120	0	Tumor
GSM972410	stage II	2730	0	Tumor
GSM972411	stage III	3750	0	Tumor
GSM972412	stage III	150	1	Tumor
GSM972413	stage III	480	1	Tumor
GSM972414	stage II	1590	0	Tumor
GSM972415	stage III	240	1	Tumor
GSM972416	stage II	150	1	Tumor
GSM972417	stage II	4380	0	Tumor
GSM972418	stage II	2100	0	Tumor
GSM972419	stage III	900	1	Tumor
GSM972420	stage III	180	1	Tumor
GSM972421	stage III	660	1	Tumor

GSM972422	stage II	390	1 Tumor
GSM972423	stage II	390	1 Tumor
GSM972424	stage II	3120	0 Tumor
GSM972425	stage III	1770	0 Tumor
GSM972426	stage II	2430	0 Tumor
GSM972427	stage II	1590	0 Tumor
GSM972428	stage III	1650	0 Tumor
GSM972429	stage III	1860	0 Tumor
GSM972430	stage II	1590	0 Tumor
GSM972431	stage III	1620	0 Tumor
GSM972432	stage III	570	1 Tumor
GSM972433	stage III	90	1 Tumor
GSM972434	stage II	570	1 Tumor
GSM972435	stage III	420	1 Tumor
GSM972436	stage II	60	1 Tumor
GSM972437	stage IV	0	1 Tumor
GSM972438	stage IV	0	1 Tumor
GSM972439	stage IV	0	1 Tumor
GSM972440	stage II	1560	0 Tumor
GSM972441	stage IV	0	1 Tumor
GSM972442	stage IV	0	1 Tumor
GSM972443	stage II	1440	0 Tumor
GSM972444	stage III	150	1 Tumor
GSM972445	stage II	1050	0 Tumor
GSM972447	stage II	930	0 Tumor
GSM972449	stage III	570	1 Tumor
GSM972450	stage II	570	1 Tumor
GSM972451	stage II	2910	0 Tumor
GSM972452	stage III	2970	0 Tumor
GSM972453	stage III	3000	0 Tumor
GSM972454	stage I	2250	0 Tumor
GSM972455	stage III	2220	0 Tumor
GSM972456	stage II	0	0 Tumor
GSM972457	stage III	1020	1 Tumor
GSM972458	stage II	510	0 Tumor
GSM972459	stage II	360	0 Tumor
GSM972460	stage II	2880	0 Tumor
GSM972461	stage II	2880	0 Tumor
GSM972462	stage I	2640	0 Tumor
GSM972464	stage I	2580	0 Tumor
GSM972465	stage II	2520	0 Tumor
GSM972466	stage III	2250	0 Tumor
GSM972467	stage III	2160	0 Tumor
GSM972468	stage II	720	0 Tumor
GSM972469	stage I	2580	0 Tumor
GSM972470	stage II	2550	0 Tumor
GSM972472	stage II	2400	0 Tumor
GSM972473	stage II	1350	1 Tumor
GSM972474	stage III	270	1 Tumor
GSM972475	stage II	2610	0 Tumor
GSM972476	stage III	1920	0 Tumor
GSM972477	stage II	2430	0 Tumor
GSM972478	stage II	2070	0 Tumor
GSM972479	stage III	90	0 Tumor
GSM972480	stage II	2070	0 Tumor
GSM972481	stage III	1950	0 Tumor
GSM972482	stage II	1260	0 Tumor
GSM972483	stage II	690	1 Tumor

GSM972484	stage II	2280	0 Tumor
GSM972485	stage II	2250	0 Tumor
GSM972486	stage II	990	0 Tumor
GSM972487	stage II	2010	0 Tumor
GSM972488	stage II	1470	0 Tumor
GSM972489	stage II	2220	0 Tumor
GSM972490	stage II	2220	0 Tumor
GSM972491	stage II	2100	0 Tumor
GSM972492	stage I	2040	0 Tumor
GSM972493	stage II	1770	0 Tumor
GSM972494	stage I	1710	0 Tumor
GSM972495	stage III	540	1 Tumor
GSM972496	stage I	4260	0 Tumor
GSM972497	stage III	30	0 Tumor
GSM972498	stage III	570	1 Tumor
GSM972499	stage III	2160	0 Tumor
GSM972501	stage III	3180	0 Tumor
GSM972502	stage II	3930	0 Tumor
GSM972503	stage II	4380	0 Tumor
GSM972504	stage III	720	1 Tumor
GSM972505	stage II	4320	0 Tumor
GSM972506	stage II	4230	0 Tumor
GSM972507	stage III	1260	0 Tumor
GSM972508	stage I	4020	0 Tumor
GSM972509	stage I	420	0 Tumor
GSM972510	stage II	2580	0 Tumor
GSM972511	stage III	1410	0 Tumor
GSM972512	stage III	120	1 Tumor
GSM972513	stage III	390	1 Tumor
GSM972515	stage II	3540	0 Tumor
GSM972516	stage II	1980	0 Tumor
GSM972517	stage II	960	1 Tumor
GSM972518	stage III	450	1 Tumor
GSM972519	stage II	330	1 Tumor
GSM972520	stage III	1860	0 Tumor
GSM972521	stage II	2280	0 Tumor
GSM972522	stage II	2970	0 Tumor

892)

Subtype	NMF clusters
CINWntUp	CC2
CSC	CC3
KRASm	CC2
CINImmuneDown	CC1
CSC	CC3
CSC	CC3
dMMR	CC2
CINImmuneDown	CC2
CINImmuneDown	CC3
CINWntUp	CC1
CSC	CC3
dMMR	CC2
KRASm	CC2
dMMR	CC2
CINImmuneDown	CC2
CINWntUp	CC2
KRASm	CC2
CINImmuneDown	CC1
CINWntUp	CC1
dMMR	CC2
dMMR	CC2
dMMR	CC2
dMMR	CC2
dMMR	CC2
dMMR	CC2
dMMR	CC2
dMMR	CC2
KRASm	CC2
CINWntUp	CC3
CINnormL	CC3
dMMR	CC2
KRASm	CC2
KRASm	CC2
CINWntUp	CC1
CINImmuneDown	CC1
KRASm	CC2
CINWntUp	CC1
CINnormL	CC2
CSC	CC3
dMMR	CC2
CINnormL	CC2
CINnormL	CC2
CINWntUp	CC1
CINImmuneDown	CC1
KRASm	CC2
CINImmuneDown	CC2
CINImmuneDown	CC1
KRASm	CC1
CINWntUp	CC3
CSC	CC3
CSC	CC3
CINnormL	CC3
CINWntUp	CC3
CSC	CC3
KRASm	CC2
CINWntUp	CC1

CINWntUp	CC1
dMMR	CC2
dMMR	CC2
CINImmuneDown	CC3
CINWntUp	CC3
CSC	CC3
CINnormL	CC3
dMMR	CC3
CINImmuneDown	CC3
KRASm	CC3
CINImmuneDown	CC1
CINImmuneDown	CC1
CSC	CC3
CINWntUp	CC3
CINnormL	CC3
CINnormL	CC3
dMMR	CC3
CINWntUp	CC1
CINWntUp	CC1
CINImmuneDown	CC1
CINImmuneDown	CC1
CINImmuneDown	CC1
KRASm	CC2
KRASm	CC2
CINWntUp	CC1
CSC	CC3
CINWntUp	CC1
CINWntUp	CC1
KRASm	CC2
CINImmuneDown	CC1
KRASm	CC2
KRASm	CC2
CINWntUp	CC3
CINWntUp	CC1
CINImmuneDown	CC1
KRASm	CC2
KRASm	CC2
CSC	CC3
CINWntUp	CC1
CINWntUp	CC1
CSC	CC3
dMMR	CC2
dMMR	CC2
CINImmuneDown	CC1
dMMR	CC2
dMMR	CC3
CINWntUp	CC1
CINImmuneDown	CC3
CINnormL	CC3
CINImmuneDown	CC3
CSC	CC3
CINImmuneDown	CC3
CINImmuneDown	CC1
CINWntUp	CC3
CSC	CC3
CINWntUp	CC1
CINnormL	CC3
CINWntUp	CC1

CSC	CC2
CSC	CC3
CINImmuneDown	CC1
dMMR	CC2
dMMR	CC2
CINnormL	CC3
CINWntUp	CC3
dMMR	CC3
dMMR	CC2
CINImmuneDown	CC2
dMMR	CC3
CINWntUp	CC3
CINnormL	CC3
CINImmuneDown	CC3
CINImmuneDown	CC1
CINImmuneDown	CC1
CINnormL	CC3
CSC	CC3
CINImmuneDown	CC1
KRASm	CC2
CINWntUp	CC3
CINWntUp	CC1
CINWntUp	CC3
CINWntUp	CC1
CINImmuneDown	CC1
CSC	CC3
KRASm	CC2
CINWntUp	CC1
KRASm	CC2
CINnormL	CC3
CINnormL	CC3
dMMR	CC2
CSC	CC3
CINWntUp	CC3
CINWntUp	CC1
CINnormL	CC3
CINWntUp	CC1
dMMR	CC2
KRASm	CC2
KRASm	CC2
CSC	CC3
CSC	CC3
KRASm	CC2
CINnormL	CC3
CINnormL	CC3
dMMR	CC2
CINImmuneDown	CC2
CINWntUp	CC1
CINWntUp	CC2
KRASm	CC2
CINWntUp	CC1
CINImmuneDown	CC1
CINnormL	CC1
CINnormL	CC3
CINWntUp	CC1
CINnormL	CC3
CINWntUp	CC3
dMMR	CC2

CINWntUp	CC1
CSC	CC3
CINWntUp	CC1
CINnormL	CC3
CINWntUp	CC3
KRASm	CC2
CINImmuneDown	CC1
CINImmuneDown	CC1
CINImmuneDown	CC1
CINWntUp	CC1
CSC	CC3
CINWntUp	CC3
CINWntUp	CC1
CINWntUp	CC3
CINImmuneDown	CC1
CINnormL	CC3
dMMR	CC3
CINWntUp	CC1
CINWntUp	CC1
CINImmuneDown	CC3
CINnormL	CC3
KRASm	CC1
CINImmuneDown	CC1
CINImmuneDown	CC1
CINImmuneDown	CC1
CINImmuneDown	CC2
CINnormL	CC1
CINWntUp	CC3
CSC	CC3
KRASm	CC2
CINWntUp	CC3
CINImmuneDown	CC3
CINWntUp	CC3
CINnormL	CC3
CINWntUp	CC1
CINnormL	CC3
CINImmuneDown	CC1
CINWntUp	CC1
dMMR	CC2
CINImmuneDown	CC1
CINWntUp	CC3
dMMR	CC3
dMMR	CC3
CINImmuneDown	CC1
CINnormL	CC3
CINImmuneDown	CC1
CINWntUp	CC3
CINnormL	CC3
KRASm	CC3
CINWntUp	CC3
dMMR	CC2
CSC	CC3
CINImmuneDown	CC3
KRASm	CC2
CSC	CC3
CINImmuneDown	CC3
CSC	CC3
dMMR	CC3

CINImmuneDown	CC3
dMMR	CC2
CINWntUp	CC1
KRASm	CC2
dMMR	CC2
CINImmuneDown	CC1
CINImmuneDown	CC1
KRASm	CC2
dMMR	CC2
KRASm	CC2
CINImmuneDown	CC1
CINImmuneDown	CC1
CINnormL	CC1
CINWntUp	CC3
CINImmuneDown	CC1
CINImmuneDown	CC1
CINWntUp	CC3
CINWntUp	CC3
CINnormL	CC3
CINWntUp	CC3
CINWntUp	CC3
CINWntUp	CC3
CSC	CC3
CINWntUp	CC1
KRASm	CC2
CINWntUp	CC3
dMMR	CC2
CINWntUp	CC2
CINImmuneDown	CC1
CINWntUp	CC1
KRASm	CC2
CINWntUp	CC1
dMMR	CC2
dMMR	CC2
dMMR	CC2
dMMR	CC2
dMMR	CC2
dMMR	CC1
KRASm	CC2
KRASm	CC2
KRASm	CC2
dMMR	CC2
CINImmuneDown	CC1
CSC	CC3
CSC	CC3
CINWntUp	CC1
CSC	CC3
CINWntUp	CC1
CINWntUp	CC1
CINWntUp	CC1
CINWntUp	CC1
CSC	CC2
CSC	CC3
dMMR	CC2
CINWntUp	CC2
CINImmuneDown	CC1
CINnormL	CC3
CINImmuneDown	CC1

CINWntUp	CC1
CINImmuneDown	CC1
CINImmuneDown	CC1
CINImmuneDown	CC1
CINWntUp	CC1
CINWntUp	CC1
CINWntUp	CC1
CINImmuneDown	CC1
CINWntUp	CC3
CINWntUp	CC3
CINImmuneDown	CC1
CINImmuneDown	CC1
CINImmuneDown	CC1
KRASm	CC3
CINImmuneDown	CC3
dMMR	CC2
CSC	CC3
dMMR	CC2
dMMR	CC2
dMMR	CC2
dMMR	CC1
CINImmuneDown	CC1
KRASm	CC2
KRASm	CC3
dMMR	CC2
CINWntUp	CC1
CINImmuneDown	CC1
KRASm	CC1
dMMR	CC2
CSC	CC3
dMMR	CC2
dMMR	CC2
dMMR	CC2
CSC	CC2
dMMR	CC2
CINWntUp	CC2
dMMR	CC1
dMMR	CC2
CINWntUp	CC1
dMMR	CC2
dMMR	CC2
dMMR	CC2
CINWntUp	CC1
CINImmuneDown	CC1
CINWntUp	CC1
CSC	CC1
dMMR	CC2
dMMR	CC2
dMMR	CC2
KRASm	CC1
KRASm	CC2
dMMR	CC3
dMMR	CC2
CINWntUp	CC1
KRASm	CC1
CINnormL	CC2
CINImmuneDown	CC1
CINImmuneDown	CC1

KRASm	CC2
dMMR	CC2
CINImmuneDown	CC1
CINImmuneDown	CC1
dMMR	CC2
CINImmuneDown	CC1
CINImmuneDown	CC2
CINWntUp	CC3
CINWntUp	CC1
CINWntUp	CC1
dMMR	CC2
KRASm	CC2
dMMR	CC2
dMMR	CC2
CINImmuneDown	CC1
CINImmuneDown	CC1
CINnormL	CC1
CINnormL	CC1
CINImmuneDown	CC1
CINWntUp	CC1
CINWntUp	CC1
KRASm	CC2
KRASm	CC1
dMMR	CC2
CINWntUp	CC1
CINnormL	CC1
dMMR	CC2
CINWntUp	CC1
dMMR	CC2
CINImmuneDown	CC1
dMMR	CC2
CINImmuneDown	CC1
CINnormL	CC1
dMMR	CC2
CINnormL	CC1
CINImmuneDown	CC1
CINWntUp	CC1
CINWntUp	CC1
CINImmuneDown	CC1
CINWntUp	CC1
CINWntUp	CC1
CSC	CC1
dMMR	CC2
CINWntUp	CC1
CINImmuneDown	CC1
CSC	CC3
CINnormL	CC1
CINWntUp	CC3
dMMR	CC2
dMMR	CC1
CINWntUp	CC3
CINnormL	CC3
CINWntUp	CC1
CINnormL	CC3
CINWntUp	CC1
CSC	CC3
CINImmuneDown	CC1
CINWntUp	CC1

KRASm	CC2
CSC	CC3
CINnormL	CC3
CSC	CC3
CINImmuneDown	CC1
CINWntUp	CC3
CINImmuneDown	CC1
CINWntUp	CC1
CINImmuneDown	CC1
CINWntUp	CC1
CINnormL	CC1
CSC	CC3
CINWntUp	CC1
CINWntUp	CC3
CINWntUp	CC1
KRASm	CC1
CSC	CC3
CINWntUp	CC1
CINWntUp	CC1
CINnormL	CC1
CINWntUp	CC1
CSC	CC3
CINImmuneDown	CC1
CINImmuneDown	CC1
CINImmuneDown	CC1
KRASm	CC2
KRASm	CC2
dMMR	CC2
CINImmuneDown	CC1
CINWntUp	CC3
CINnormL	CC3
CINWntUp	CC1
KRASm	CC1
CINImmuneDown	CC1
CINImmuneDown	CC1
CINWntUp	CC1
CINnormL	CC2
CSC	CC3
CINWntUp	CC3
dMMR	CC2
CINImmuneDown	CC1
CINWntUp	CC1
dMMR	CC2
CINWntUp	CC3
CINWntUp	CC3
CSC	CC3
CINWntUp	CC3
CINImmuneDown	CC1
CSC	CC3
CSC	CC3
CINImmuneDown	CC1
CINImmuneDown	CC1
CINWntUp	CC3
CINWntUp	CC3
CINWntUp	CC1
CINWntUp	CC3
dMMR	CC3
CINWntUp	CC3

CSC	CC3
CINnormL	CC3
CINnormL	CC3
dMMR	CC3
CINnormL	CC3
CINWntUp	CC3
CINWntUp	CC3
KRASm	CC2
dMMR	CC2
CINWntUp	CC3
CINnormL	CC3
CSC	CC3
CINImmuneDown	CC2
CINImmuneDown	CC3
CINImmuneDown	CC1
CINWntUp	CC3
CSC	CC3
CINnormL	CC3
CSC	CC3
CINnormL	CC3
CINImmuneDown	CC2
KRASm	CC2
CSC	CC2
dMMR	CC2
dMMR	CC2
CINnormL	CC2
CINImmuneDown	CC1
CINImmuneDown	CC1
dMMR	CC2
KRASm	CC2
CINnormL	CC1
dMMR	CC2
CINWntUp	CC1
KRASm	CC1
dMMR	CC2
KRASm	CC2
CINWntUp	CC1
CINImmuneDown	CC1
CINWntUp	CC1
CINWntUp	CC1
dMMR	CC2
CINImmuneDown	CC1
CSC	CC3
CINWntUp	CC1
CINWntUp	CC1
dMMR	CC2
KRASm	CC2
KRASm	CC2
CINnormL	CC1
KRASm	CC2
CSC	CC3
KRASm	CC2
CINnormL	CC1
CSC	CC3
KRASm	CC2
CINWntUp	CC1
dMMR	CC2
CINWntUp	CC1

CINWntUp	CC1
CINWntUp	CC1
CINWntUp	CC1
KRASm	CC2
KRASm	CC2
KRASm	CC2
CINImmuneDown	CC1
CINWntUp	CC1
CINWntUp	CC1
CINWntUp	CC1
dMMR	CC2
CINWntUp	CC1
KRASm	CC1
CINWntUp	CC1
CINImmuneDown	CC1
dMMR	CC2
CINWntUp	CC1
KRASm	CC2
CINnormL	CC3
CINImmuneDown	CC3
CINImmuneDown	CC2
CINnormL	CC3
dMMR	CC2
CINImmuneDown	CC1
CINImmuneDown	CC1
KRASm	CC1
CINWntUp	CC1
KRASm	CC2
CINWntUp	CC1
CINImmuneDown	CC1
CINImmuneDown	CC1
CINWntUp	CC1
CINnormL	CC3
CINnormL	CC3
dMMR	CC2
CSC	CC3
dMMR	CC3

Table S1. Basic information of bulk databases (TCGA, GSE14333, GSE39582 a

samples	Stage	RFS (days)	RFS.event
GSM929508	stage II	438.9999999	1
GSM929511	stage III	858	1
GSM929622	stage III	78	1
GSM929546	stage II	263	1
GSM929623	stage III	350.0000001	1
GSM929539	stage II	383.0000001	1
GSM929614	stage III	561	1
GSM929496	stage III	165	1
GSM929574	stage III	645	1
GSM929590	stage III	551.0000001	1
GSM929586	stage II	750.9999999	1
GSM929585	stage II	840.9999999	1
GSM929612	stage III	993	1
GSM929609	stage III	204	1
GSM929497	stage III	209	1
GSM929593	stage III	438	1
GSM929591	stage II	519	1
GSM929551	stage II	1387	1
GSM929587	stage II	1150	1
GSM929523	stage III	300.9999999	1
GSM929519	stage III	779.0000001	1
GSM929524	stage III	591	1
GSM929525	stage III	111	1
GSM929599	stage III	1015	1
GSM929617	stage III	336.9999999	1
GSM929576	stage III	137	1
GSM929572	stage III	366	1
GSM929499	stage III	965.0000001	1
GSM929504	stage III	965.0000001	1
GSM929610	stage III	1156	1
GSM929498	stage III	1140	1
GSM929618	stage III	608.0000001	1
GSM929575	stage III	549.9999999	1
GSM929577	stage III	426	1
GSM929502	stage III	199	1
GSM929503	stage III	200	1
GSM929500	stage III	1721	1
GSM929494	stage II	1705	0
GSM929495	stage II	1211	0
GSM929501	stage III	1757	0
GSM929505	stage II	2773	0
GSM929506	stage II	1259	0
GSM929507	stage II	2600	0
GSM929509	stage II	2308	0
GSM929510	stage II	357.9999999	0
GSM929512	stage III	981.9999999	0
GSM929513	stage III	1259	0
GSM929514	stage II	2168	0
GSM929515	stage II	1941	0
GSM929516	stage II	1695	0
GSM929517	stage II	1399	0
GSM929518	stage III	461.0000001	0
GSM929520	stage III	1107	0
GSM929521	stage III	1100	0
GSM929522	stage III	1083	0
GSM929526	stage III	459	0

GSM929527	stage III	774.9999999	0
GSM929528	stage II	1621	0
GSM929529	stage II	1096	0
GSM929530	stage II	1240	0
GSM929531	stage II	1355	0
GSM929532	stage II	1348	0
GSM929533	stage II	1369	0
GSM929534	stage II	1390	0
GSM929535	stage II	1326	0
GSM929536	stage II	1736	0
GSM929537	stage II	1349	0
GSM929538	stage II	1285	0
GSM929540	stage II	1948	0
GSM929541	stage II	1700	0
GSM929542	stage II	1553	0
GSM929543	stage II	1282	0
GSM929544	stage II	1006	0
GSM929545	stage II	1323	0
GSM929547	stage II	1456	0
GSM929548	stage II	2429	0
GSM929549	stage II	2654	0
GSM929550	stage II	2124	0
GSM929552	stage II	1444	0
GSM929553	stage II	2274	0
GSM929554	stage II	1878	0
GSM929555	stage II	2059	0
GSM929556	stage II	468.9999999	0
GSM929557	stage II	899.0000001	0
GSM929558	stage II	1788	0
GSM929559	stage II	17.00000001	0
GSM929560	stage II	1032	0
GSM929561	stage II	39	0
GSM929562	stage II	804	0
GSM929563	stage II	816	0
GSM929564	stage II	590.0000001	0
GSM929565	stage II	1693	0
GSM929566	stage II	1834	0
GSM929567	stage II	1522	0
GSM929568	stage II	1545	0
GSM929569	stage II	2101	0
GSM929570	stage II	1210	0
GSM929571	stage II	1534	0
GSM929573	stage III	1430	0
GSM929578	stage III	999	0
GSM929579	stage II	1685	0
GSM929580	stage II	1663	0
GSM929581	stage II	1680	0
GSM929582	stage II	1939	0
GSM929583	stage II	1771	0
GSM929584	stage II	1779	0
GSM929588	stage II	2155	0
GSM929589	stage III	1904	0
GSM929592	stage III	1432	0
GSM929594	stage III	1820	0
GSM929595	stage III	1804	0
GSM929596	stage III	3137.000001	0
GSM929597	stage III	1787	0
GSM929598	stage III	1668	0

GSM929600	stage III	2163	0
GSM929601	stage III	1853	0
GSM929602	stage II	1096	0
GSM929603	stage III	1496	0
GSM929604	stage III	2818	0
GSM929605	stage III	1407	0
GSM929606	stage III	1099	0
GSM929607	stage III	2864	0
GSM929608	stage III	1149	0
GSM929611	stage III	1327	0
GSM929613	stage II	1416	0
GSM929615	stage III	2135	0
GSM929616	stage II	160	0
GSM929619	stage II	1887	0
GSM929620	stage II	2624	0
GSM929621	stage II	2860	0

nd GSE37892)

Sample type	NMF clusters
Tumor	CC3
Tumor	CC2
Tumor	CC3
Tumor	CC1
Tumor	CC3
Tumor	CC3
Tumor	CC3
Tumor	CC2
Tumor	CC2
Tumor	CC3
Tumor	CC1
Tumor	CC1
Tumor	CC1
Tumor	CC3
Tumor	CC2
Tumor	CC3
Tumor	CC3
Tumor	CC2
Tumor	CC1
Tumor	CC3
Tumor	CC1
Tumor	CC1
Tumor	CC2
Tumor	CC2
Tumor	CC2
Tumor	CC2
Tumor	CC3
Tumor	CC3
Tumor	CC3
Tumor	CC1
Tumor	CC1
Tumor	CC2
Tumor	CC3
Tumor	CC2
Tumor	CC2
Tumor	CC3
Tumor	CC2
Tumor	CC1
Tumor	CC1
Tumor	CC3
Tumor	CC2
Tumor	CC1
Tumor	CC3
Tumor	CC2
Tumor	CC2
Tumor	CC3
Tumor	CC3
Tumor	CC1
Tumor	CC1
Tumor	CC1
Tumor	CC2
Tumor	CC2
Tumor	CC2
Tumor	CC2
Tumor	CC2
Tumor	CC1

Tumor	CC2
Tumor	CC1
Tumor	CC1
Tumor	CC3
Tumor	CC2
Tumor	CC2
Tumor	CC1
Tumor	CC1
Tumor	CC1
Tumor	CC1
Tumor	CC1
Tumor	CC1
Tumor	CC1
Tumor	CC1
Tumor	CC2
Tumor	CC2
Tumor	CC1
Tumor	CC1
Tumor	CC1
Tumor	CC3
Tumor	CC3
Tumor	CC1
Tumor	CC3
Tumor	CC3
Tumor	CC3
Tumor	CC3
Tumor	CC1
Tumor	CC1
Tumor	CC1
Tumor	CC3
Tumor	CC2
Tumor	CC1
Tumor	CC3
Tumor	CC2
Tumor	CC2
Tumor	CC1
Tumor	CC1
Tumor	CC1
Tumor	CC2
Tumor	CC3
Tumor	CC1
Tumor	CC2
Tumor	CC3
Tumor	CC3
Tumor	CC1
Tumor	CC2
Tumor	CC1
Tumor	CC3
Tumor	CC1
Tumor	CC1
Tumor	CC1
Tumor	CC2
Tumor	CC3
Tumor	CC3
Tumor	CC1
Tumor	CC1
Tumor	CC2
Tumor	CC1

Tumor	CC3
Tumor	CC3
Tumor	CC1
Tumor	CC1
Tumor	CC2
Tumor	CC2
Tumor	CC3
Tumor	CC1
Tumor	CC1
Tumor	CC1
Tumor	CC1
Tumor	CC1
Tumor	CC3
Tumor	CC1
Tumor	CC3
Tumor	CC3

Table S2. Basic information of single-cell databases (GSE144735) (SMC and KU

Tumor	Sample type	Gender	Age	TNM stage	Stage
SMC01-T	Colorectal cancer	F	64	T3 N0 M0	IIA
SMC02-T	Colorectal cancer	M	66	T3 N1b M0	IIIB
SMC03-T	Colorectal cancer	F	83	T4b N2a M0	IIIC
SMC04-T	Colorectal cancer	M	69	T3 N1b M0	IIIB
SMC05-T	Colorectal cancer	F	58	T3 N0 M0	IIA
SMC06-T	Colorectal cancer	M	46	T3 N1b M0	IIIB
SMC07-T	Colorectal cancer	F	67	T2 N0 M0	I
SMC08-T	Colorectal cancer	M	68	T3 N1b M0	IIIB
SMC09-T	Colorectal cancer	M	75	T3 N0 M0	IIA
SMC10-T	Colorectal cancer	F	77	T3 N0 M0	IIA
SMC11-T	Colorectal cancer	F	38	T2 N1a M0	IIIA
SMC14-T	Colorectal cancer	M	77	T4a N1b M0	IIIB
SMC15-T	Colorectal cancer	M	56	T3 N0 M0	IIA
SMC16-T	Colorectal cancer	M	59	T4a N1b M0	IIIB
SMC17-T	Colorectal cancer	M	47	T4a N1b M0	IIIB
SMC18-T	Colorectal cancer	F	63	T3 N0 M0	IIA
SMC19-T	Colorectal cancer	F	80	T3 N1 M0	IIIB
SMC20-T	Colorectal cancer	F	65	T4a N1c M0	IIIB
SMC21-T	Colorectal cancer	M	51	T3 N1 M1a	IVA
SMC22-T	Colorectal cancer	M	76	T3 N1b M0	IIIB
SMC23-T	Colorectal cancer	F	67	T3 N1b M0	IIIB
SMC24-T	Colorectal cancer	F	48	T1 N0 M0	I
SMC25-T	Colorectal cancer	F	57	T3 N2b M1a	IVA

JL cohorts)

Anatomic regio	Left/Right-sided	MSI
rectum	left	MSS
rectum	left	MSS
hepatic flexure	right	MSI-H
sigmoid	left	MSS
ascending	right	MSS
hepatic flexure	right	MSI-H
ascending	right	MSS
sigmoid	left	MSS
sigmoid	left	MSS
ascending	right	MSI-H
sigmoid	left	MSS
rectosigmoid	left	MSS
sigmoid	left	MSS
ascending	right	MSS
hepatic flexure	right	MSS
sigmoid	left	MSS
ascending	right	MSS
ascending	right	MSS
rectum	left	MSS
sigmoid	left	MSS
ascending	right	MSS
ascending	right	MSI-H
sigmoid	left	MSS

Pathological subtype	Bulk CMS subtypes
Adenocarcinoma, well differentiated	CMS3
Adenocarcinoma, well differentiated	CMS4
Adenocarcinoma, poorly differentiated	CMS1
Adenocarcinoma, moderately differentiated	CMS4
Adenocarcinoma, well differentiated with mucin production (<10%)	CMS3
Adenocarcinoma, well differentiated	CMS1
Adenocarcinoma, well differentiated	CMS2
Adenocarcinoma, moderately differentiated	CMS1
Adenocarcinoma, well differentiated	CMS2
Adenocarcinoma, moderately differentiated	CMS1
Adenocarcinoma, well differentiated	CMS2
Adenocarcinoma, moderately differentiated	CMS4
Adenocarcinoma, well differentiated	CMS1
Adenocarcinoma, moderately differentiated	CMS3
Mucinous adenocarcinoma	CMS4
Adenocarcinoma, moderately differentiated	CMS2
Adenocarcinoma, moderately differentiated	CMS3
Mucinous adenocarcinoma	CMS4
Adenocarcinoma, moderately differentiated	CMS2
Adenocarcinoma, moderately differentiated	CMS2
Adenocarcinoma, moderately differentiated	CMS2
Adenocarcinoma, well differentiated with mucin production (10%)	CMS4
Adenocarcinoma, moderately differentiated	CMS2

KRAS	BRAF	TP53	APC	SMAD4
Mutant	Wildtype	Mutant	Mutant	Wildtype
Wildtype	Wildtype	Mutant	Mutant	Mutant
Wildtype	Mutant	Wildtype	Mutant	Wildtype
Mutant	Wildtype	Mutant	Mutant	Wildtype
Mutant	Wildtype	Wildtype	Mutant	Wildtype
Mutant	Wildtype	Wildtype	Wildtype	Wildtype
Mutant	Wildtype	Mutant	Mutant	Wildtype
Wildtype	Wildtype	Mutant	Mutant	Wildtype
Wildtype	Wildtype	Mutant	Mutant	Wildtype
Wildtype	Mutant	Wildtype	Wildtype	Wildtype
Wildtype	Wildtype	Mutant	Mutant	Wildtype
Mutant	Wildtype	Wildtype	Wildtype	Wildtype
Wildtype	Wildtype	Wildtype	Mutant	Wildtype
Wildtype	Wildtype	Wildtype	Wildtype	Wildtype
Wildtype	Mutant	Wildtype	Wildtype	Mutant
Wildtype	Wildtype	Mutant	Mutant	Wildtype
Mutant	Wildtype	Mutant	Mutant	Wildtype
Mutant	Wildtype	Mutant	Wildtype	Wildtype
Wildtype	Wildtype	Mutant	Mutant	Mutant
Wildtype	Mutant	Mutant	Mutant	Wildtype
Mutant	Wildtype	Mutant	Mutant	Wildtype
Mutant	Wildtype	Wildtype	Mutant	Mutant
Wildtype	Wildtype	Mutant	Mutant	Wildtype

Table S2. Basic information of single-cell databases (GSE144735) (SMC

Patient	Core	Sample type	Gender	Age
KUL01	KUL01-T	Colorectal cancer	F	81
KUL19	KUL19-T	Colorectal cancer	F	86
KUL21	KUL21-T	Colorectal cancer	F	50
KUL28	KUL28-T	Colorectal cancer	M	52
KUL30	KUL30-T	Colorectal cancer	M	84
KUL31	KUL31-T	Colorectal cancer	M	85

and KUL cohorts)

TNM stage	Stage	Anatomic region	Left/Right-sided	MSI
pT4aN0	IIB	caecum	right	MSI-H
pT3N1b	IIIB	rectosigmoid	left	MSS
pT4aN1aM1a	IVA	sigmoid	left	MSS
pT3N0L1	IIA	sigmoid	left	MSS
pT3N0L1	IIA	ascending	right	MSS
pT1N0	I	sigmoid	left	MSS

Pathological subtype
Global moderately differentiated adenocarcinoma with mixed glandulair, mucinous growth patern, mode
Moderately differentiated adenocarcinoma NST
Moderately differentiated adenocarcinoma
Moderately differentiated adenocarcinoma
Moderately differentiated adenocarcinoma
Well differentiated adenocarcinoma NST

Bulk CMS subtypes	TP53	APC	KRAS	BRAF	SMAD4
Unknown	Mutant	Wildtype	Wildtype	Mutant	Wildtype
CMS4	Mutant	Mutant	Wildtype	Wildtype	Wildtype
CMS1	Mutant	Mutant	Mutant	Wildtype	Wildtype
CMS2	Mutant	Mutant	Wildtype	Wildtype	Wildtype
CMS2	Wildtype	Mutant	Mutant	Wildtype	Mutant
CMS3	Wildtype	Mutant	Mutant	Wildtype	Wildtype

Table S3. Bi[illegible]

Symbol
RPL8
IREB2
ATP5MC3
CS
EMC2
ACSF2
NOX1
CYBB
NOX3
NOX4
NOX5
DUOX1
DUOX2
G6PD
PGD
VDAC2
TP53
ACSL4
LPCAT3
NRAS
KRAS
HRAS
CARS1
KEAP1
HMOX1
ATG5
ATG7
NCOA4
ALOX12
ALOX12B
ALOX15
ALOX15B
ALOXE3
PHKG2
SAT1
EGFR
MAPK3
MAPK1
ZEB1
DPP4
CDKN2A
PEBP1
SOCS1
CDO1
MYB
CHAC1
LINC00472
PRKAA2
PRKAA1
ELAVL1
BAP1
ABCC1
MIR6852
ACVR1B
TGFBR1

IFNG
ANO6
HMGB1
TNFAIP3
ATF3
ATM
YY1AP1
EGLN2
MIOX
TAZ
MTDH
IDH1
FBXW7
PANX1
DNAJB6
LONP1
PTGS2
DUSP1
NOS2
NCF2
MT3
UBC
ALB
TXNRD1
SRXN1
GPX2
BNIP3
OXSR1
SELENOS
ANGPTL7
CHAC1
SLC7A11
DDIT4
LOC284561
ASNS
TSC22D3
DDIT3
JDP2
SESN2
SLC1A4
PCK2
TXNIP
VLDLR
GPT2
PSAT1
LURAP1L
SLC7A5
HERPUD1
XBP1
ATF3
SLC3A2
CBS
ATF4
ZNF419
KLHL24
TRIB3
ZFP69B
ATP6V1G2

VEGFA
GDF15
TUBE1
ARRDC3
CEBPG
SNORA16A
RGS4
BLOC1S5-TXI
LOC390705
EIF2S1
HSD17B11
AGPAT3
SETD1B
HMOX1
TF
FTL
RPL8
ATP5MC3
TFRC
MAFG
FTH1
DRD5
DRD4
MAP3K5
MAPK14
SLC2A1
SLC2A3
SLC2A6
SLC2A8
SLC2A12
GLUT13
SLC2A14
EIF2AK4
ALOX5
ALOX12
ALOX15
HMGB1
ELAVL1
HBA1
NNMT
PLIN4
HIC1
STMN1
RRM2
CAPG
HNF4A
NGB
YWHAE
GABPB1
AURKA
MIR4715
RIPK1
PRDX1
MIR30B
SLC7A11
GPX4
AKR1C1
AKR1C2

AKR1C3
RB1
HSPB1
HSF1
NFE2L2
SQSTM1
NQO1
HMOX1
FTH1
MUC1
MT1G
SLC40A1
CISD1
HSPA5
ATF4
TP53
HELLS
SCD
FADS2
SRC
STAT3
PML
NFS1
TP63
CDKN1A
MIR137
VDAC2
FH
CISD2
MIR9-1
MIR9-2
MIR9-3
CBS
ISCU
ACSL3
OTUB1
CD44
LINC00336
BRD4
PRDX6
MIR17
SESN2
NF2
ARNTL
HIF1A
JUN
CA9
TMBIM4
PLIN2
AIFM2
LAMP2
ZFP36
PROM2
CHMP5
CHMP6
CAV1
GCH1

ological information of ferroptosis-associated genes (human genes)

Name
Ribosomal protein L8
Iron response element binding protein 2
ATP synthase membrane subunit c locus 3
Citrate synthase
ER membrane protein complex subunit 2
Acyl-CoA synthetase family member 2
Nicotinamide adenine dinucleotide phosphate (NADPH) oxidase (NOX) 1
Cytochrome b-245 beta chain
Nicotinamide adenine dinucleotide phosphate (NADPH) oxidase (NOX) 3
Nicotinamide adenine dinucleotide phosphate (NADPH) oxidase (NOX) 4
Nicotinamide adenine dinucleotide phosphate (NADPH) oxidase (NOX) 5
Dual oxidase 1
Dual oxidase 2
Glucose-6-phosphate dehydrogenase
Phosphoglycerate dehydrogenase
Valtage-dependent anion channels 2
Tumor protein p53
Acyl-CoA synthetase long chain family member 4
Lysophosphatidylcholine acyltransferase 3
NRAS proto-oncogene, GTPase
KRAS proto-oncogene, GTPase
HRas proto-oncogene, GTPase
Cysteinyl-tRNA synthetase 1
Kelch like ECH associated protein 1
Heme oxygenase 1
Autophagy related 5
Autophagy related 7
Nuclear receptor coactivator 4
Arachidonate 12-lipoxygenase, 12S type
Arachidonate 12-lipoxygenase, 12R type
Arachidonate 15-lipoxygenase
Arachidonate 15-lipoxygenase type B
Arachidonate lipoxygenase 3
Phosphorylase kinase catalytic subunit gamma 2
Spermidine/spermine N1-acetyltransferase 1
Epidermal growth factor receptor
Mitogen-activated protein kinase 3
Mitogen-activated protein kinase 1
Zinc finger E-box binding homeobox 1
Dipeptidyl peptidase 4
Cyclin dependent kinase inhibitor 2A
Phosphatidylethanolamine binding protein 1
Suppressor of cytokine signaling 1
Cysteine dioxygenase type 1
MYB proto-oncogene, transcription factor
ChaC glutathione specific gamma-glutamylcyclotransferase 1
Long intergenic non-protein coding RNA 472
Protein kinase AMP-activated catalytic subunit alpha 2
Protein kinase AMP-activated catalytic subunit alpha 1
ELAV like RNA binding protein 1
BRCA1 associated protein 1
ATP binding cassette subfamily C member 1
microRNA 6852
Activin A receptor type 1B
Transforming growth factor beta receptor 1

Interferon gamma
Anoctamin 6
High mobility group box 1
TNF alpha induced protein 3
Activating transcription factor 3
ATM serine/threonine kinase
YY1 associated protein 1
Egl-9 family hypoxia inducible factor 2
Myo-inositol oxygenase
Tafazzin
Metadherin
Isocitrate dehydrogenase (NADP(+)) 1
F-box and WD repeat domain containing 7
Pannexin 1
DnaJ heat shock protein family (Hsp40) member B6
Lon peptidase 1, mitochondrial
Prostaglandin-endoperoxide synthase 2
Dual specificity phosphatase 1
Nitric oxide synthase 2
Neutrophil cytosolic factor 2
Metallothionein 3
Ubiquitin C
Albumin
Thioredoxin reductase 1
Sulfiredoxin 1
Glutathione peroxidase 2
BCL2 interacting protein 3
Oxidative stress responsive kinase 1
Selenoprotein S
Angiopoietin like 7
ChaC glutathione specific gamma-glutamylcyclotransferase 1
Solute carrier family 7 member 11
DNA damage inducible transcript 4
_NA_
Asparagine synthetase (glutamine-hydrolyzing)
TSC22 domain family member 3
DNA damage inducible transcript 3
Jun dimerization protein 2
Sestrin 2
Solute carrier family 1 member 4
Phosphoenolpyruvate carboxykinase 2, mitochondrial
Thioredoxin interacting protein
Very low density lipoprotein receptor
Glutamic--pyruvic transaminase 2
Phosphoserine aminotransferase 1
Leucine rich adaptor protein 1 like
Solute carrier family 7 member 5
Homocysteine inducible ER protein with ubiquitin like domain 1
X-box binding protein 1
Activating transcription factor 3
Solute carrier family 3 member 2
Cystathionine beta-synthase
Activating transcription factor 4
Zinc finger protein 419
Kelch like family member 24
Tribbles pseudokinase 3
ZFP69 zinc finger protein B
ATPase H+ transporting V1 subunit G2

Vascular endothelial growth factor A
Growth differentiation factor 15
Tubulin epsilon 1
Arrestin domain containing 3
CCAAT enhancer binding protein gamma
Small nucleolar RNA, H/ACA box 16A
Regulator of G protein signaling 4
BLOC1S5-TXNDC5 readthrough (NMD candidate)
_NA_
Eukaryotic translation initiation factor 2 subunit 1
Hydroxysteroid 17-beta dehydrogenase 11
1-acylglycerol-3-phosphate O-acyltransferase 3
SET domain containing 1B, histone lysine methyltransferase
Heme oxygenase 1
Transferrin
Ferritin light chain
Ribosomal protein L8
ATP synthase membrane subunit c locus 3
Transferrin receptor
MAF bZIP transcription factor G
Ferritin heavy chain 1
Dopamine receptor D5
Dopamine receptor D4
Mitogen-activated protein kinase kinase kinase 5
Mitogen-activated protein kinase 14
Solute carrier family 2 member 1
Solute carrier family 2 member 3
Solute carrier family 2 member 6
Solute carrier family 2 member 8
Solute carrier family 2 member 12
_NA_
Solute carrier family 2 member 14
Eukaryotic translation initiation factor 2 alpha kinase 4
Arachidonate 5-lipoxygenase
Arachidonate 12-lipoxygenase, 12S type
Arachidonate 15-lipoxygenase
High mobility group box 1
ELAV like RNA binding protein 1
Hemoglobin subunit alpha 1
Nicotinamide N-methyltransferase
Perilipin 4
HIC ZBTB transcriptional repressor 1
Stathmin 1
Ribonucleotide reductase regulatory subunit M2
Capping actin protein, gelsolin like
Hepatocyte nuclear factor 4 alpha
Neuroglobin
Tyrosine 3-monooxygenase/tryptophan 5-monooxygenase activation protein epsilon
GA binding protein transcription factor subunit beta 1
Aurora kinase A
microRNA 4715
Receptor interacting serine/threonine kinase 1
Peroxiredoxin 1
microRNA 30b
Solute carrier family 7 member 11
Glutathione peroxidase 4
Aldo-keto reductase family 1 member C1
Aldo-keto reductase family 1 member C2

Aldo-keto reductase family 1 member C3
RB transcriptional corepressor 1
Heat shock protein family B (small) member 1
Heat shock transcription factor 1
Nuclear factor, erythroid 2 like 2
Sequestosome 1
NAD(P)H quinone dehydrogenase 1
Heme oxygenase 1
Ferritin heavy chain 1
Mucin 1, cell surface associated
Metallothionein 1G
Solute carrier family 40 member 1
CDGSH iron sulfur domain 1
Heat shock protein family A (Hsp70) member 5
Activating transcription factor 4
Tumor protein p53
Helicase, lymphoid specific
Stearoyl-CoA desaturase
Fatty acid desaturase 2
SRC proto-oncogene, non-receptor tyrosine kinase
Signal transducer and activator of transcription 3
Promyelocytic leukemia
NFS1 cysteine desulfurase
Tumor protein p63
Cyclin dependent kinase inhibitor 1A
microRNA 137
Voltage dependent anion channel 2
Fumarate hydratase
CDGSH iron sulfur domain 2
microRNA 9-1
microRNA 9-2
microRNA 9-3
Cystathionine beta-synthase
Iron-sulfur cluster assembly enzyme
Acyl-CoA synthetase long chain family member 3
OTU deubiquitinase, ubiquitin aldehyde binding 1
CD44 molecule (Indian blood group)
Long intergenic non-protein coding RNA 336
Bromodomain containing 4
Peroxiredoxin 6
microRNA 17
Sestrin 2
Neurofibromin 2
Aryl hydrocarbon receptor nuclear translocator like
Hypoxia inducible factor 1 subunit alpha
Jun proto-oncogene, AP-1 transcription factor subunit
Carbonic anhydrase 9
Transmembrane BAX inhibitor motif containing 4
Perilipin 2
Apoptosis inducing factor mitochondria associated 2
Lysosomal associated membrane protein 2
ZFP36 ring finger protein
Prominin 2
Charged multivesicular body protein 5
Charged multivesicular body protein 6
Caveolin 1
GTP cyclohydrolase 1

HGNC_ID
HGNC:10368
HGNC:6115
HGNC:843
HGNC:2422
HGNC:28963
HGNC:26101
HGNC:7889
HGNC:2578
HGNC:7890
HGNC:7891
HGNC:14874
HGNC:3062
HGNC:13273
HGNC:4057
HGNC:8891
HGNC:12672
HGNC:11998
HGNC:3571
HGNC:30244
HGNC:7989
HGNC:6407
HGNC:5173
HGNC:1493
HGNC:23177
HGNC:5013
HGNC:589
HGNC:16935
HGNC:7671
HGNC:429
HGNC:430
HGNC:433
HGNC:434
HGNC:13743
HGNC:8931
HGNC:10540
HGNC:3236
HGNC:6877
HGNC:6871
HGNC:11642
HGNC:3009
HGNC:1787
HGNC:8630
HGNC:19383
HGNC:1795
HGNC:7545
HGNC:28680
HGNC:21380
HGNC:9377
HGNC:9376
HGNC:3312
HGNC:950
HGNC:51
HGNC:49993
HGNC:172
HGNC:11772

HGNC:5438
HGNC:25240
HGNC:4983
HGNC:11896
HGNC:785
HGNC:795
HGNC:30935
HGNC:14660
HGNC:14522
HGNC:11577
HGNC:29608
HGNC:5382
HGNC:16712
HGNC:8599
HGNC:14888
HGNC:9479
HGNC:9605
HGNC:3064
HGNC:7873
HGNC:7661
HGNC:7408
HGNC:12468
HGNC:399
HGNC:12437
HGNC:16132
HGNC:4554
HGNC:1084
HGNC:8508
HGNC:30396
HGNC:24078
HGNC:28680
HGNC:11059
HGNC:24944
_NA_
HGNC:753
HGNC:3051
HGNC:2726
HGNC:17546
HGNC:20746
HGNC:10942
HGNC:8725
HGNC:16952
HGNC:12698
HGNC:18062
HGNC:19129
HGNC:31452
HGNC:11063
HGNC:13744
HGNC:12801
HGNC:785
HGNC:11026
HGNC:1550
HGNC:786
HGNC:20648
HGNC:25947
HGNC:16228
HGNC:28053
HGNC:862

HGNC:12680
HGNC:30142
HGNC:20775
HGNC:29263
HGNC:1837
HGNC:32605
HGNC:10000
HGNC:42001
_NA_
HGNC:3265
HGNC:22960
HGNC:326
HGNC:29187
HGNC:5013
HGNC:11740
HGNC:3999
HGNC:10368
HGNC:843
HGNC:11763
HGNC:6781
HGNC:3976
HGNC:3026
HGNC:3025
HGNC:6857
HGNC:6876
HGNC:11005
HGNC:11007
HGNC:11011
HGNC:13812
HGNC:18067
_NA_
HGNC:18301
HGNC:19687
HGNC:435
HGNC:429
HGNC:433
HGNC:4983
HGNC:3312
HGNC:4823
HGNC:7861
HGNC:29393
HGNC:4909
HGNC:6510
HGNC:10452
HGNC:1474
HGNC:5024
HGNC:14077
HGNC:12851
HGNC:4074
HGNC:11393
HGNC:41666
HGNC:10019
HGNC:9352
HGNC:31625
HGNC:11059
HGNC:4556
HGNC:384
HGNC:385

HGNC:386
HGNC:9884
HGNC:5246
HGNC:5224
HGNC:7782
HGNC:11280
HGNC:2874
HGNC:5013
HGNC:3976
HGNC:7508
HGNC:7399
HGNC:10909
HGNC:30880
HGNC:5238
HGNC:786
HGNC:11998
HGNC:4861
HGNC:10571
HGNC:3575
HGNC:11283
HGNC:11364
HGNC:9113
HGNC:15910
HGNC:15979
HGNC:1784
HGNC:31523
HGNC:12672
HGNC:3700
HGNC:24212
HGNC:31641
HGNC:31642
HGNC:31646
HGNC:1550
HGNC:29882
HGNC:3570
HGNC:23077
HGNC:1681
HGNC:33813
HGNC:13575
HGNC:16753
HGNC:31547
HGNC:20746
HGNC:7773
HGNC:701
HGNC:4910
HGNC:6204
HGNC:1383
HGNC:24257
HGNC:248
HGNC:21411
HGNC:6501
HGNC:12862
HGNC:20685
HGNC:26942
HGNC:25675
HGNC:1527
HGNC:4193

Evidence
Required in erastin-induced ferroptosis. Silencing of it conferred against erastin-induced ferroptosis.
Required in erastin-induced ferroptosis. Silencing of it conferred against erastin-induced ferroptosis.
Required in erastin-induced ferroptosis. Silencing of it conferred against erastin-induced ferroptosis.
Required in erastin-induced ferroptosis. Silencing of it conferred against erastin-induced ferroptosis.
Required in erastin-induced ferroptosis. Silencing of it conferred against erastin-induced ferroptosis.
Required in erastin-induced ferroptosis. Silencing of it conferred against erastin-induced ferroptosis.
Suppresses erastin-induced ferroptosis when inhibited by inhibitor.
Suppresses erastin-induced ferroptosis when inhibited by inhibitor.
Suppresses erastin-induced ferroptosis when inhibited by inhibitor.
Suppresses erastin-induced ferroptosis when inhibited by inhibitor.
Suppresses erastin-induced ferroptosis when inhibited by inhibitor.
Suppresses erastin-induced ferroptosis when inhibited by inhibitor.
Suppresses erastin-induced ferroptosis when inhibited by inhibitor.
Required in erastin-induced ferroptosis.
Required in erastin-induced ferroptosis.
Required in erastin-induced ferroptosis.
Inhibits cystine uptake and sensitizes cells to ferroptosis. Erastin induced high levels of cell death in p53+
Deletion of this gene likely suppress ferroptosis by limiting the membrane-resident pool of oxidation-se
Deletion of this gene likely suppress ferroptosis by limiting the membrane-resident pool of oxidation-se
NRAS12V mutant protects RMS13 cells from ferroptotic cell death.
KRAS12V mutant protects RMS13 cells from ferroptotic cell death.
HRAS12V mutant protects RMS13 cells from ferroptotic cell death.
Required for ferroptosis in diverse cell contexts. Knockdown of CARS inhibited erastin-induced death by
Knockdown of Keap1 reversed loss of p62-increased degradation of NRF2 in ferroptosis. Keap1 knockdc
Zinc protoporphyrin IX, a HO-1 inhibitor, prevented Erastin-triggered ferroptotic cancer cell death. Over
Knockout or knockdown limited erastin-induced ferroptosis.
Knockout or knockdown limited erastin-induced ferroptosis.
Inhibition suppressed ferroptosis, and overexpression promoted ferroptosis.
Silencing ALOX genes made cells resistant to ferroptosis.
Silencing ALOX genes made cells resistant to ferroptosis.
Silencing ALOX genes made cells resistant to ferroptosis.
Silencing ALOX genes made cells resistant to ferroptosis. Erastin-induced cell death was rescued by silen
Silencing ALOX genes made cells resistant to ferroptosis. Erastin-induced cell death was rescued by silen
U-2-OS cells became resistant to erastin upon PHKG2 silencing. Erastin-treated HT-1080 cells were resc
p53-mediated activation of SAT1 contributes to ferroptotic cell death in the presence of ROS stress. Kno
Cell death in activated EGFR mutant cells occurs by ferroptosis. Inhibiting EGFR and MAPK signaling resc
Inhibiting EGFR and MAPK signaling rescued cell viability following cystine withdrawal.
Inhibiting EGFR and MAPK signaling rescued cell viability following cystine withdrawal.
Knockout of ZEB1 prevents cell death induced by GPX4 inhibition.
Required for ferroptosis in TP53-deficient CRC cells.
Combination of ARF induction and ROS treatment induced ferroptotic cell death. Knockdown of endoge
Elevated levels of PEBP1 resulted in increased sensitivity of HK2 cells to RSL3 whereas lowered contents c
Expression of SOCS1 sensitized cells to ferroptosis inducer. This effect of SOCS1 was efficiently blocked b
CDO1 suppression contributes to ferroptosis resistance.
Erastin-induced ferroptosis was restrained when c-Myb was suppressed.
CHAC1 degradation of GSH might enhance cystine-starvation-induced cell death.
Increases erastin-induced growth inhibition, whereas depletion of P53RRA decreased erastin-induced gr
Inhibition of PRKAA/AMPKalpha diminishes ferroptosis.
Inhibition of PRKAA/AMPKalpha diminishes ferroptosis.
ELAVL1 siRNA led to ferroptosis resistance, whereas ELAVL1 plasmid contributed to classical ferroptotic e
Suppresses SLC7A11-mediated cystine uptake and promotes ferroptosis. BAP1 mutants lose their abilitie
Accelerates ferroptosis. Disruption of MRP1 inhibited ferroptosis potently.
Promotes ferroptosis. Binds to LINC0033 and serves as a negative upstream regulator of CBS-mediated f
Inhibition attenuated erastin-induced ferroptosis.
Inhibition attenuated erastin-induced ferroptosis.

Interferon gamma released from CD8+ T cells downregulates the expression of SLC3A2 and SLC7A11, ar
Essential for ferroptosis. Inhibition blocked ferroptotic cell death induced by RSL3/erastin.
Required for erastin-induced ferroptosis. Knockdown of HMGB1 decreased erastin-induced cell death.
Overexpression increased ROS generation and enhanced erastin-induced ferroptosis, whereas knockdov
Promotes ferroptosis induced by erastin.
Essential for ferroptosis. Genetic knockdown and chemical inhibition of ATM both suppress ferroptotic ce
Makes cells more sensitive to ferroptosis. Cells lacking YAP were no longer sensitised to ferroptosis.
Inhibiting EGLN2 activation diminished ferroptotic tumor cell death.
Overexpression exacerbates cell death, knockdown inhibits ferroptosis.
TAZ removal confers ferroptosis resistance, whereas overexpression of TAZS89A sensitizes cells to ferrop
Can enhance sensitivity to inducers of ferroptosis. Enhances the vulnerability of cancer cells to ferroptosi
Deletion of the mutant IDH1 allele or pharmacological inhibition of mutant IDH1 confers resistance to er
FBXW7 plasmid induces ferroptosis.
Deletion protects against ferroptotic cell death. Silenced Panx1 expression significantly attenuated ferrop
Promotes ferroptosis in esophageal squamous cell carcinoma.
Inhibition of LONP1 negatively regulates erastin-induced cell death.
Simply a downstream marker of ferroptosis. The most upregulated gene in BJeLR cells upon treatment w
Expression was upregulated during ferroptosis induced by erastin or RSL3.
Expression was upregulated during ferroptosis induced by erastin or RSL3.
Expression was upregulated during ferroptosis induced by erastin or RSL3.
Expression was upregulated during ferroptosis induced by erastin or RSL3.
Expression was upregulated during ferroptosis induced by erastin or RSL3.
Expression was upregulated during ferroptosis induced by erastin or RSL3.
Expression was upregulated during ferroptosis induced by erastin or RSL3.
Expression was upregulated during ferroptosis induced by erastin or RSL3.
Expression was upregulated during ferroptosis induced by erastin or RSL3.
Expression was upregulated during ferroptosis induced by erastin or RSL3.
Expression was upregulated during ferroptosis induced by erastin or RSL3.
Expression was upregulated during ferroptosis induced by erastin or RSL3.
Expression was downregulated during ferroptosis induced by erastin or RSL3.
Up-regulated in erastin-treated samples. A useful pharmacodynamic marker of system Xc- inhibition.
Similar to erastin treatment, silencing of this gene inhibits glutamate release. Erastin specifically inhibits S
Up-regulated (>= 2 fold) in erastin-treated samples.
Up-regulated (>= 2 fold) in erastin-treated samples.
Up-regulated (>= 2 fold) in erastin-treated samples.
Up-regulated (>= 2 fold) in erastin-treated samples.
Up-regulated (>= 2 fold) in erastin-treated samples.
Up-regulated (>= 2 fold) in erastin-treated samples.
Up-regulated (>= 2 fold) in erastin-treated samples.
Up-regulated (>= 2 fold) in erastin-treated samples.
Up-regulated (>= 2 fold) in erastin-treated samples.
Up-regulated (>= 2 fold) in erastin-treated samples.
Up-regulated (>= 2 fold) in erastin-treated samples.
Up-regulated (>= 2 fold) in erastin-treated samples.
Up-regulated (>= 2 fold) in erastin-treated samples.
Up-regulated (>= 2 fold) in erastin-treated samples.
Up-regulated (>= 2 fold) in erastin-treated samples.
Up-regulated (>= 2 fold) in erastin-treated samples.
Up-regulated (>= 2 fold) in erastin-treated samples.
Up-regulated (>= 2 fold) in erastin-treated samples.
Up-regulated (>= 2 fold) in erastin-treated samples.
Up-regulated (>= 2 fold) in erastin-treated samples.
Up-regulated (>= 2 fold) in erastin-treated samples.
Up-regulated (>= 2 fold) in erastin-treated samples.
Up-regulated (>= 2 fold) in erastin-treated samples.
Up-regulated (>= 2 fold) in erastin-treated samples.
Up-regulated (>= 2 fold) in erastin-treated samples.
Up-regulated (>= 2 fold) in erastin-treated samples.

Up-regulated (>= 2 fold) in erastin-treated samples.
Up-regulated (>= 2 fold) in erastin-treated samples.
Up-regulated (>= 2 fold) in erastin-treated samples.
Up-regulated (>= 2 fold) in erastin-treated samples.
Up-regulated (>= 2 fold) in erastin-treated samples.
Down-regulated (>= 2 fold) in erastin-treated samples.
Down-regulated (>= 2 fold) in erastin-treated samples.
Down-regulated (>= 2 fold) in erastin-treated samples.
Down-regulated (>= 2 fold) in erastin-treated samples.
Phosphorylated in erastin-treated sample.
Enriched in RSL3-resistant cells.
Enriched in RSL3-resistant cells.
Enriched in GPX4 inhibitor ML162-resistant cells.
Its expression increased in response to artesunate-induced ferroptosis, indicating activation of ROS-mec
Its expression is decreased in patients.
Its expression is decreased in patients.
Significantly reduced in tumor tissues
Significantly reduced in tumor tissues
Expression of this gene is increased in patients.
The interaction between NRF2 and MafG was increased in response to erastin and sorafenib.
An increase of endogenous FTH1 level during ferroptosis. Degradation of FTH1 protein upon ferroptosis
Ferroptotic erastin induces DRD5 gene expression in ferroptosis.
Antiferroptotic dopamine suppressed dopamine receptor D4 protein degradation. Ferroptotic erastin pro
Cold stress evokes ferroptosis, and the ASK1-p38 pathway is activated downstream of lipid peroxide, lea
Cold stress evokes ferroptosis, and the ASK1-p38 pathway is activated downstream of lipid peroxide, lea
Increased at LSH overexpression. Decreased at LSH knockdown. LSH can inhibit ferroptosis.
Decreased at LSH knockdown. LSH can inhibit ferroptosis.
Increased at LSH overexpression. Decreased at LSH knockdown. LSH can inhibit ferroptosis.
Decreased at LSH knockdown. LSH can inhibit ferroptosis.
Increased at LSH overexpression. Decreased at LSH knockdown. LSH can inhibit ferroptosis.
Increased at LSH overexpression. Decreased at LSH knockdown. LSH can inhibit ferroptosis.
Decreased at LSH knockdown. LSH can inhibit ferroptosis.
CHAC1 degradation of GSH enhances cystine-starvation-induced ferroptosis through the activated GCN
Overexpression sensitizes cells to ferroptosis.
Overexpression sensitizes cells to ferroptosis.
Overexpression sensitizes cells to ferroptosis.
Associated with ferroptotic cell death. Ferroptosis activators induce HMGB1 release.
Binds to and increases the expression of the negative ferroptosis regulator LINC00336.
Upregulated in cells treated with ferroptosis inducer erastin. Stimulates ferroptosis possibly in a GSH-dep
Upregulated in cells treated with ferroptosis inducer erastin. Stimulates ferroptosis possibly in a GSH-dep
Upregulated in cells treated with ferroptosis inducer erastin. Stimulates ferroptosis possibly in a GSH-dep
Essential for stimulation of pro-ferroptotic gene transcription upon ferroptosis induction.
Downregulated in cells treated with ferroptosis inducer erastin. Suppresses ferroptosis possibly in a GSH-
Downregulated in cells treated with ferroptosis inducer erastin. Suppresses ferroptosis possibly in a GSH-
Downregulated in cells treated with ferroptosis inducer erastin. Suppresses ferroptosis possibly in a GSH-
Essential for stimulation of anti-ferroptotic gene transcription.
Human neuroglobin (hNgb)-EGFP-expressing SH-SY5Y cells to be significantly more resistant to ferropto
Required by RSL3 (a ferroptosis inducer) to inactivate GPX4 (a ferroptosis inhibitor).
Downregulated by lncRNA GABPB1-AS1 upon erastin treatment.
Inhibition of AURKA or reconstitution of miR-4715-3p inhibited GPX4 and induced cell death, suggesting
Inhibition of AURKA or reconstitution of miR-4715-3p inhibited GPX4 and induced cell death, suggesting
Reduced expression results in resistance to ferroptosis.
Necessary to ferroptosis-related lipid peroxidation.
Upregulation of miR-30b-5p in preeclampsia models plays a pivotal role in ferroptosis.
Silencing of SLC7A11 sensitized HT-1080 cells to erastin-induced death, whereas transfection of HT-108
RNAi-mediated GPX4 knockdown induces ferroptosis.
Up-regulated in DU-145 erastin-resistant clones. Participate in the detoxification of toxic lipid metabolite
Up-regulated in DU-145 erastin-resistant clones. Participate in the detoxification of toxic lipid metabolite

Up-regulated in DU-145 erastin-resistant clones. Participate in the detoxification of toxic lipid metabolit
Rb knock-down cells exposed to sorafenib encounter ferroptosis. Lack of Rb sensitized HCC cells to the i
Knockdown of HSF1 and HSPB1 enhances erastin-induced ferroptosis, whereas heat shock pretreatment
Knockdown of HSF1 and HSPB1 enhances erastin-induced ferroptosis, whereas heat shock pretreatment
NRF2 plays a central role in protecting hepatocellular carcinoma (HCC) cells against ferroptosis
The interaction between p62 and Keap1 increased following erastin and sorafenib treatment. Knockdow
Knockdown of p62, quinone oxidoreductase-1, heme oxygenase-1, and ferritin heavy chain-1 by RNA ir
Knockdown of p62, quinone oxidoreductase-1, heme oxygenase-1, and ferritin heavy chain-1 by RNA ir
Knockdown of p62, quinone oxidoreductase-1, heme oxygenase-1, and ferritin heavy chain-1 by RNA ir
MUC1-C (C-terminal subunit) blocks erastin-induced ferroptosis and induces increases in GSH.
A negative regulator of ferroptosis in HCC cells. Knockdown of MT-1G by RNA interference increases glu
Involved in siramesine and lapatinib-induced ferroptotic cell death. Its expression is decreased after trea
Genetic inhibition of CISD1 contributes to erastin-induced ferroptosis. Stabilization of the iron sulfur clus
Negatively regulates ferroptosis. Suppression of HSPA5 expression increased erastin-induced death. Ove
Inhibition of ATF4 expression increased erastin-induced cell death. ATF4 results in the induction of HSPA
Inhibits ferroptosis in human colorectal cancer (CRC) cells. Loss of TP53 restored erastin sensitivity. Inhibi
LSH inhibits ferroptosis by decreasing the intracellular levels of iron and lipid ROS.
Depletion of the SCD1 and FADS2 metabolic genes induces ferroptosis.
Depletion of the SCD1 and FADS2 metabolic genes induces ferroptosis.
Src-STAT3 activation renders the cell unable to undergo to ferroptosis. Src inhibition decreased cell viab
Src-STAT3 activation renders the cell unable to undergo to ferroptosis.
PML expression turned cells highly resistant to ferroptosis.
Suppression of NFS1 cooperates with inhibition of cysteine transport to trigger ferroptosis in vitro and sl
Delta Np63 alpha can inhibit ferroptosis independent of p53. Overexpression protects cells from ferropto
Required to to suppress ferroptosis.
Suppresses ferroptosis both in vitro and in vivo.
Overexpression could partially protect cells from ferroptosis.
FH inactivation (FH-/-) proves synthetic lethal with inducers of ferroptosis. FH-/- sensitizes cells to mult
Overexpression conferred resistance to ferroptosis. Inhibition blocked resistance to ferroptotic cell death
Overexpression of miR-9 suppressed GOT1, which subsequently reduced ferroptosis. Suppression of miR
Overexpression of miR-9 suppressed GOT1, which subsequently reduced ferroptosis. Suppression of miR
Overexpression of miR-9 suppressed GOT1, which subsequently reduced ferroptosis. Suppression of miR
Inhibition triggers ferroptosis in hepatocellular carcinoma.
Over expression significantly attenuated DHA induced ferroptosis.
Required for exogenous monounsaturated fatty acids to protect cells against ferroptosis. Negatively corr
Inactivation promotes ferroptosis by down-regulating SLC7A11 levels. Overexpression is critical for tumc
Knockdown sensitizes cells to ferroptosis.
Overexpression inhibits ferroptosis. Knockdown promotes ferroptosis.
Inhibition induces ferroptosis.
A negative regulator of ferroptotic cell death.
Protects endothelial HUVEC cells from erastin-induced ferroptosis. Overexpression significantly reduced
Has cytoprotective effect against ferroptosis. In cells expressing Sesn2, erastin-induced cell death, ROS fo
Genetic inactivation of NF2 rendered cancer cells more sensitive to ferroptosis. Mediates cell density-dep
Degration of the protein is critical for ferroptosis. Blocking ARNTL degradation diminished ferroptotic tu
Destabilizing HIF1A facilitated ferroptotic tumor cell death.
O-GlcNAcylated c-Jun represents an obstructive factor to ferroptosis.
Inhibition induces ferroptosis.
Protects against ferroptosis in HCC cells. Inhibition increased ferroptotic cel death.
An indispensable gene and protein in the suppression of ferroptosis caused by abnormal lipometabolism
A glutathione-independent ferroptosis suppressor. Pharmacological targeting of FSP1 strongly synergize
Knockdown promoted ferroptosis.
ZFP36 plasmid impaired FBXW7 plasmid-induced HSC ferroptosis. Overexpression of Zfp36 impaired era
Induced by ferroptotic stress and promotes resistance to ferroptotic cell death. Facilitates ferroptosis resi
Ferroptosis activators increase ESCRT-III subunits (e.g., CHMP5 and CHMP6). Knockdown of CHMP5 or C
Ferroptosis activators increase ESCRT-III subunits (e.g., CHMP5 and CHMP6). Knockdown of CHMP5 or C
Cav-1 deficiency aggravated ferroptosis. Short hairpin RNA of Cav-1 promoted ferroptosis, which was ar
Gch1 overexpression and its downstream metabolites BH4/BH2 rescue from ferroptosis. Inhibition of GC

Test method
shRNA screening, qPCR, gene silencing
shRNA screening, qPCR, gene silencing
shRNA screening, qPCR, gene silencing
shRNA screening, qPCR, gene silencing
shRNA screening, qPCR, gene silencing
shRNA screening, qPCR, gene silencing
Inhibition test by diphenylene iodonium (DPI) and GKT137831.
Inhibition test by diphenylene iodonium (DPI).
Inhibition test by diphenylene iodonium (DPI).
Inhibition test by diphenylene iodonium (DPI) and GKT137831.
Inhibition test by diphenylene iodonium (DPI).
Inhibition test by diphenylene iodonium (DPI).
Inhibition test by diphenylene iodonium (DPI).
shRNA silencing, RT-qPCR
shRNA silencing, RT-qPCR
shRNA silencing, RT-qPCR
Cell death kinetics, shRNA, western blotting, RT-PCR, ChIP assay, cystine uptake assay
Retroviral-mediated insertional mutagenesis and sequencing
Retroviral-mediated insertional mutagenesis and sequencing
Cell viability assessed by MTT assay, western blot
Cell viability assessed by MTT assay, western blot
Cell viability assessed by MTT assay, western blot
siRNA screen, shRNA, western blot, cell viability
Western blot, shRNA knockdown, cell viability
Cell viability assay, qRT-PCR, western blot
shRNA
shRNA
shRNA, gene transfection
qPCR
qPCR
qPCR
qPCR, siRNA
qPCR, siRNA
shRNA suppressor screen, qPCR, cellular iron staining, cell viability
qRT-PCR, siRNA, CRISPR-cas9, western blot
Fluorescence-activated cell sorting (FACS) analysis, cell viability
Immunoblots, lucifer yellow infiltration, cell viability, fluorescence-activated cell sorting (FACS)
Immunoblots, lucifer yellow infiltration, cell viability, fluorescence-activated cell sorting (FACS)
sgRNA, cell viability
Cell death, RNAi, western blot, enzyme activity
RNAi, immunoblot, phase-contrast image, cell death quantification
Western blot, siRNA, cell death assay
Cell death assay, cell viability assay, qPCR
siRNA, western blot, MTT assay
Western blot, siRNA, qRT-PCR, MTT assay
Western blot, real-time PCR, cell viability assay, siRNA, GSH assay kit
Bisulfite sequencing, RNA pull-down, mass spectrometry, RNA immunoprecipitation, qRT-PCF
siRNA, western blot, cell viability assay, GSH assay, lipid peroxidation assay, iron assay
siRNA, western blot, cell viability assay, GSH assay, lipid peroxidation assay, iron assay
Immunohistochemistry, hydroxyproline assay, immunofluorescence, cell viability assay, westeri
Flow cytometry, cell viability assay, cell death assay, western blot, gene knockout, lipid peroxid
Western blot, GSH assay, dead cell counts, gene transfection, CRISPR/Cas9, cell viability assay,
qRT-PCR, gene knockdown, luciferase assay, gene overexpression, MTT assay, colony-formati
Western blot, siRNA, glutathione assay, RT-PCR, cell viability assay
Western blot, siRNA, glutathione assay, RT-PCR, cell viability assay

BODIPY-C11, liperfluo staining, cell death measurement, FACS, cell proliferation and viability a
Immunocytochemistry, RT-PCR, TUNEL Assay, flow Cytometry, LDH assay, gene knockout, siRN
Gene transfection, RNAi, cell viability assay, ELISA, LDH assay, flow cytometry, qPCR, western b
Lentivirus transduction, CCK-8 assay, DYE670 staining assay, ROS staining assay, RT-qPCR, we
Cell viability assay, transfection, lentiviral infection, qRT-PCR, western blot
siRNA, western blot, nuclear/cytosol fraction, cell viability, cell death, GSH assay, kinome scree
Cell death, cell viability, lipid peroxidation, immunoblotting, CRISPR/Cas9, ChIP assay, qRT-PC
Cytotoxicity assays, western blot, immunoprecipitation, RNA interference, gene transfection, q
MTT assay, measurement of GPX4 activity, labile iron pool assay, evaluation of cell death and
Cell viability, cell death, western blot, siRNA, qRT-PCR, microarray, immunofluorescence staini
Cell viability, qRT-PCR, western blotting, GSH assay, GPx4 activity assay, tumor xenograft mod
Cell transfection, cell viability, GSH assay, lipid ROS assay
Cell viability, lipid peroxidation, iron, GSH and ROS assays, western blot, RT-PCR, RNA-seq
Cell viability, cell death, qRT-PCR, western blot, lipid peroxidation detection, shRNA
Western blot, immunohistochemistry,lentivirus infection, colony-forming, RT?qPCR, CCK-8, tra
Cell viability, western blot, qRT-PCR, lipid peroxidation assay, glutathione assay
RT-qPCR, cell viability
RT-qPCR, cell viability
RT-qPCR, cell viability
RT-qPCR, cell viability
RT-qPCR, cell viability
RT-qPCR, cell viability
RT-qPCR, cell viability
RT-qPCR, cell viability
RT-qPCR, cell viability
RT-qPCR, cell viability
RT-qPCR, cell viability
RT-qPCR, cell viability
RT-qPCR, cell viability
RT-qPCR, cell viability
RNA-seq, RT-qPCR
RNA silencing, RT-qPCR
RNA-seq
RNA-seq
RNA-seq
RNA-seq
RNA-seq
RNA-seq
RNA-seq
RNA-seq
RNA-seq
RNA-seq
RNA-seq
RNA-seq
RNA-seq
RNA-seq
RNA-seq
RNA-seq
RNA-seq
RNA-seq
RNA-seq
RNA-seq
RNA-seq
RNA-seq
RNA-seq
RNA-seq
RNA-seq
RNA-seq

RNA-seq
RNA-seq
RNA-seq
RNA-seq
RNA-seq
RNA-seq
RNA-seq
RNA-seq
RNA-seq
Western blot
Gene trap insertion
Gene trap insertion
Gene trap insertion
Western blotting
Gene expression analysis
Gene expression analysis
Gene expression analysis
Gene expression analysis
Gene expression analysis
Western blot
Immunoblotting, q-PCR
Western blot, Q-PCR
Western blot, Q-PCR
LDH assay, immunoblot, RNAi, CCK-8
LDH assay, immunoblot, RNAi, CCK-8
ChIP analysis, RT-qPCR
RT-qPCR
RT-qPCR
RT-qPCR
RT-qPCR
RT-qPCR
RT-qPCR
Western blot, real-time PCR, cell viability assay, siRNA, GSH assay kit
Western blot, mass spectrometry, cell viability assay, gene overexpression
Western blot, mass spectrometry, cell viability assay, gene overexpression
Western blot, mass spectrometry, cell viability assay, gene overexpression
ELISA, western blot, RNAi
RNA pulldown, mass spectrometry, qRT-PCR, gene overexpression, gene knockdown, western
Tandem mass tags, RNA-seq, DNase-seq, qPCR, Western blot, IHC
Tandem mass tags, RNA-seq, DNase-seq, qPCR, Western blot, IHC
Tandem mass tags, RNA-seq, DNase-seq, qPCR, Western blot, IHC
Immunohistochemistry, immunofluorescence, western blot, qPCR, metabolites examination, Cl
Tandem mass tags, RNA-seq, DNase-seq, qPCR, Western blot, IHC
Tandem mass tags, RNA-seq, DNase-seq, qPCR, Western blot, IHC
Tandem mass tags, RNA-seq, DNase-seq, qPCR, Western blot, IHC
Immunohistochemistry, immunofluorescence, western blot, qPCR, metabolites examination, Cl
Cell death, cell viability, lipid peroxidation assay, RT-qPCR
Mass spectrometry, siRNA, western blot
RT-qPCR, western blot, siRNA, cell viability, lipid peroxidation assay
qRT-PCR, western blot, lentiviral infection, CellTiter-Glo luminescence assay
qRT-PCR, western blot, lentiviral infection, CellTiter-Glo luminescence assay
Cell viability, western blot, RNA-seq, GSH assay, qRT-PCR, immunofluorescence imaging
Western blot, siRNA, flow cytometry, cell viability, RT-PCR
GSH assay, MDA measurement, western blot, immunohistochemistry, microarray, RT-qPCR, lal
RT-qPCR, siRNA, gene transfection, cell viability
Affinity-based chemoproteomics, western blotting, and RNAi
RNA-seq, glutamate release assay, cell growth, flow cytometry
RNA-seq, glutamate release assay, cell growth, flow cytometry

RNA-seq, glutamate release assay, cell growth, flow cytometry
RNA interference, cell viability assay, oxidative stress measurement
RNAi, RT-qPCR, western blot, cell viability
RNAi, RT-qPCR, western blot, cell viability
Cell Viability Analysis, western blot, RNAi, qRT-PCR
Immunoprecipitation assay, shRNA knockdown, iron assay, lipid peroxidation assay, glutathior
qRT-PCR, RNA interference
qRT-PCR, RNA interference
qRT-PCR, RNA interference
shRNA, GSH level measurement, cell death test
RNA interference, cell viability assay, colony formation assay, western blot, qRT-PCR, glutathio
Western blot, densitometry quantification, siRNA, gene transfection, cell death test
Cytotoxicity assays, western blot, q-PCR, RNAi
Cell viability assay, western blot, shRNA, gene transfection
CCK8 cell viability assay, clonogenic cell survival assay, western blot, q-PCR, RNAi
RNAi, live-cell imaging, cell viability, qPCR, western blot
Plate-colony formation assay; measurement of total ROS, Lipid ROS, and intracellular iron
shRNA, RT-qPCR, MTT assay, total iron detection, lipid ROS detection
shRNA, RT-qPCR, MTT assay, total iron detection, lipid ROS detection
Immunoblotting, RNAi, qPCR, western blot, cell viability
Immunoblotting, RNAi, qPCR, western blot, cell viability
Cell death assessment
RNAi, cell viability assay,flow cytometry
siRNA, cell death quantification
CRISPR, shRNA, immunoblotting, cell-cycle profile, cell death quantification
Immunoblotting, luciferase reporter assay, qRT-PCR, cell viability assay, colony formation assa
Western blot, cell viability assay
CRISPR/Cas9, sgRNA, cell viability assay, immunoblotting, qPCR,
Cell viability and death assay, GSH and ROS levels and lipid peroxidation assays, RNAi, gene tr
Immunoblotting, luciferase reporter assay, qPCR, cell viability assay, MDA assay, iron assay, glu
Immunoblotting, luciferase reporter assay, qPCR, cell viability assay, MDA assay, iron assay, glu
Immunoblotting, luciferase reporter assay, qPCR, cell viability assay, MDA assay, iron assay, glu
Cell viability, western blotting, LC/ESI-MS/MS, flow cytometry, xenograft mice
Cell viability, cell cycle, ROS determination, western blot, immunofluorescence, ectopic express
Dead cell count, gene knockout, confocal imaging, thin-layer chromatography, BODIPY 493/5
CRISPR/Cas9, RNAi, cell death, western blot, qRT-PCR
Western blot, siRNA, cell death
qRT-PCR, MTT assay, colony-formation assay, lipid ROS assays, iron assay, immunoblotting
Cell proliferation assay, migration and invasion assays, RT-PCR, western blot, immunohistoche
Western blot, flow cytometry, SRB assay, gene transfection, shRNA, LOOH quantification
Lentivirus transduction, CCK-8 assay, DYE670 staining assay, ROS staining assay, RT-qPCR, we
MTT assay, RT-PCR, luciferase assay, immunoblot, siRNA, Lipid peroxidation assay, GSH measu
Cell death, cell viability, lipid peroxidation, immunoblotting, CRISPR/Cas9, ChIP assay, qRT-PC
Cytotoxicity assays, western blot, immunoprecipitation, RNA interference, gene transfection, q
Cytotoxicity assays, western blot, immunoprecipitation, RNA interference, gene transfection, q
Immunofluorescence, western blotting, immunohistochemistry, cell viability, colony formation,
qRT-PCR, western blot, cell viability assay, siRNA, ROS detection
Cell viability and clonogenic survival assay, immunofluorescence, western blot, RNAi, gene trar
Cell proliferation and clonogenic assay, cell survival analysis, xenografts tumor, RNA-seq, RT?c
Expression cloning, cell death, cell viability, LDH assay, western blot, LC-MS, NADH consumpti
Cell viability, western blot, ROS induction, glutathione assay, lipid peroxidation assay
Cell viability, lipid peroxidation, iron, GSH and ROS assays, western blot, RT-PCR, RNA-seq
RNA-seq, western blot, qPCR, siRNA, cell count, cell death, iron assay
RNAi, western blot, iron, malondialdehyde and cytotoxicity assays
RNAi, western blot, iron, malondialdehyde and cytotoxicity assays
Immunofluorescence, western blot, ROS detection
CRISPR activation screening, qRT-PCR, cell viability, cell counts, western blot

Test in
Human
Human
Human
Human
Human
Human
Human
Human
Human
Human
Human
Human
Human
Human
Human
Human
Human, mice
Human
Human
Human
Human
Human
Human, rat
Human, mice
Human, mice
Human, mice
Human, mice
Human
Human
Human
Human
Human
Human
Human
Human, mice
Human
Human
Human
Human
Human, mice
Human, mice
Human, mice
Human
Human
Human
Human
Human
Human
Human
Human, mice
Human
Human
Human
Human
Human

Human, mice
Human, mice
Human, mice
Human
Human
Human
Human, mice
Human, mice
Human, mice
Human, mice
Human, mice
Human
Human, mice
Human, mice
Human, mice
Human
Human
Human
Human
Human
Human
Human
Human
Human
Human
Human
Human
Human
Human
Human
Human
Human
Human
Human
Human
Human
Human
Human
Human
Human
Human
Human
Human
Human
Human
Human
Human
Human
Human
Human
Human
Human
Human
Human
Human
Human
Human
Human

Human
Human
Human
Human
Human
Human
Human
Human
Human
Human
Human
Human
Human
Human
Human
Human
Human
Human
Human
Human, mice
Human
Human
Human
Human
Human
Human
Human
Human
Human
Human
Human
Human
Human
Human
Human
Human
Human, mice
Human
Human
Human
Human
Human
Human
Human
Human
Human
Human
Human
Human
Human
Human
Human
Human
Human, rat
Human
Human, mice
Human
Human

Human
Human, mice
Human, mice
Human, mice
Human, mice
Human, mice
Human, mice
Human, mice
Human, mice
Human
Human, mice
Human
Human
Human, mice
Human
Human, mice
Human, mice
Human
Human
Human
Human
Human
Human
Human
Human
Human, mice
Human
Human
Human
Human
Human
Human
Human, mice
Human
Human
Human, mice
Human
Human
Human
Human
Human
Human, mice
Human, mice
Human, mice
Human, mice
Human, mice
Human
Human, mice
Human, mice
Human, mice
Human
Human, mice
Human
Human, mice
Human, mice
Human, mice
Human

Test setting
NRAS mutant HT-1080 fibrosarcoma cells; KRAS mutant Calu-1 non small cell lung cancer cell
NRAS mutant HT-1080 fibrosarcoma cells; KRAS mutant Calu-1 non small cell lung cancer cell
NRAS mutant HT-1080 fibrosarcoma cells; KRAS mutant Calu-1 non small cell lung cancer cell
NRAS mutant HT-1080 fibrosarcoma cells; KRAS mutant Calu-1 non small cell lung cancer cell
NRAS mutant HT-1080 fibrosarcoma cells; KRAS mutant Calu-1 non small cell lung cancer cell
NRAS mutant HT-1080 fibrosarcoma cells; KRAS mutant Calu-1 non small cell lung cancer cell
KRAS mutant Calu-1 non small cell lung cancer cell
KRAS mutant Calu-1 non small cell lung cancer cell
KRAS mutant Calu-1 non small cell lung cancer cell
KRAS mutant Calu-1 non small cell lung cancer cell
KRAS mutant Calu-1 non small cell lung cancer cell
KRAS mutant Calu-1 non small cell lung cancer cell
KRAS mutant Calu-1 non small cell lung cancer cell
KRAS mutant Calu-1 non small cell lung cancer cell
KRAS mutant Calu-1 non small cell lung cancer cell
KRAS mutant Calu-1 non small cell lung cancer cell
H1299, U2OS and MCF7 cells, mouse embryonic fibroblasts
Chronic myeloid leukemia cell line KBM7
Chronic myeloid leukemia cell line KBM7
RMS13 cells
RMS13 cells
RMS13 cells
HT-1080 fibrosarcoma cells,BJeLR fibroblast cells, Panc1 cells, A673 (Ewing's sarcoma) and 143B (osteosa
HepG2, Hepa1-6, Hep3B, and SNU-182 cells
HT-1080 fibrosarcoma cells, mice lung fibroblastic cells
Mouse embryonic fibroblasts, human pancreatic cancer cell lines (PANC1 and PANC2.03), and the huma
Mouse embryonic fibroblasts, human pancreatic cancer cell lines (PANC1 and PANC2.03), and the huma
PANC1 or HT-1080 cells
G-401 cells
G-401 cells
G-401 cells
G-401, BJeLR and HT-1080 cells
G-401, BJeLR and HT-1080 cells
U-2-OS, HT-1080 cells
H1299, U2OS cells, mouse embryonic fibroblasts, mouse xenograft
Human mammary epithelial) cells
Human mammary epithelial) cells
Human mammary epithelial) cells
KP4 pancreatic cancer cells
TP53-/- HCT116 cells, athymic nude mice
H1299, SaoS2, U2OS cells, mouse embryonic fibroblasts
HK2, HAEC and HT22 cells
U2OS or IMR90 cells
Gastric cancer cells AGS, BGC823, athymic nude mice
Gastric cancer cells AGS, BGC823, MKN45, SGC7901, and MGC803
MDA-MB-231, Hs 578T, and HCC 1937 cells
Lung cancer A549, SPCA1, and H522 cells
HCT116 and CX-1 cells
HCT116 and CX-1 cells
Human liver specimens and hepatic stellate cell; C57BL/6 mice; mouse hepatocyte and hepatic stellate ce
HEK-293T, Caki1, 786-O, 769-P, ACHN and NCI-H226 cell lines; RCC4, UMRC2, SLR20, and UMRC6 cell
HAP1, H1299, U-2 OS cells
A549 and SPC-A-1 cells
HK-2 cells
HK-2 cells

HT-1080, A375, B16-F0, ID8 cells, female NSG and C57BL/6 mice
A549, Cal27, HT29 and MG-63 cells, Cx3cr1-Cre mice
HL-60 cell, male NOD/SCID mice
HUVEC cells
HT1080 cells, retinal pigment epithelial cells
MDA-MB-231, RCC4, 293T, and HT-1080 cell lines
Mouse embryonic fibroblasts, NF639 cells, human epithelial tumour cells, human mesothelioma cells, xer
Calu-1, THP1, HT1080, and HL-60 cells, athymic nude mice
HK-2 cells, C57BL/6J mice
RCC4, 786O cells, JAX NOD.CB17-PrkdcSCID-J mice
Cell lines A549, H1975, DMS53, DMS273, KLE, AN3CA, RL95, Hec1A, Ishikawa, MDA-MB-231, MCF-7, He
HEK293T, HT-1080 and KYSE-170 cells
Hepatic stellate cells, C57BL/6 mice
HK-2 cells, C57BL/6 mice
Cell lines TE-1, Eca9706, Eca109, KYSE150, and KYSE450, BALB/c athymic nude mice
PANC1, BxPC3 cells
BJeLR cells
BJeLR cells
BJeLR cells
BJeLR cells
BJeLR cells
BJeLR cells
BJeLR cells
BJeLR cells
BJeLR cells
BJeLR cells
BJeLR cells
BJeLR cells
BJeLR cells
BJeLR cells
HT-1080 and Calu-1 cells
HT-1080 cells
HT-1080 cells
HT-1080 cells
HT-1080 cells
HT-1080 cells
HT-1080 cells
HT-1080 cells
HT-1080 cells
HT-1080 cells
HT-1080 cells
HT-1080 cells
HT-1080 cells
HT-1080 cells
HT-1080 cells
HT-1080 cells
HT-1080 cells
HT-1080 cells
HT-1080 cells
HT-1080 cells
HT-1080 cells
HT-1080 cells
HT-1080 cells
HT-1080 cells
HT-1080 cells
HT-1080 cells
HT-1080 cells
HT-1080 cells

HT-1080 cells
HT-1080 cells
HT-1080 cells
HT-1080 cells
HT-1080 cells
HT-1080 cells
HT-1080 cells
HT-1080 cells
HT-1080 cells
HT-1080 cells
Chronic myeloid leukemia cell line KBM7
Chronic myeloid leukemia cell line KBM7
Chronic myeloid leukemia cell line KBM7
Pancreatic adenocarcinoma cell line Panc-1 cells
Patient PDAC tissues
Patient PDAC tissues
Patient PDAC tissues
Patient PDAC tissues
Patient PDAC tissues
HepG2, Hepa1-6, Hep3B, and SNU-182 cells
HT1080 cells
PANC1 cells
PANC1 cells
A549 cells
A549 cells
H358, A549 cells
H358, A549 cells
H358, A549 cells
H358, A549 cells
H358, A549 cells
H358, A549 cells
H358, A549 cells
MDA-MB-231, Hs 578T, and HCC 1937 cells
HEK293 cells
HEK293 cells
HEK293 cells
HT1080 and PANC1 cells, mouse embryonic fibroblasts
H358, PC9, SPC-A-1, and A549 cells
HepG2, Bel-7402 cells
HepG2, Bel-7402 cells
HepG2, Bel-7402 cells
HepG2, Bel-7402 cells
HepG2, Bel-7402 cells
HepG2, Bel-7402 cells
HepG2, Bel-7402 cells
HepG2, Bel-7402 cells
SH-SY5Y cells
HEK293T cells
HepG2, Huh7, and Hep3B cells
OE33, STKM2, MKN45 cells
OE33, STKM2, MKN45 cells
Patient-derived fibroblasts
Primary corneal endothelial cells, B4G12 and HT1080 cells
Trophoblasts, preeclampsia model, HTR-8/SVneo and TEV-1 cells
HT-1080 cells
HRAS mutant BJeLR-engineered tumor cells, HT-1080 cells, xenograft mouse model (athymic nude mice
DU-145 prostate cancer cells
DU-145 prostate cancer cells

DU-145 prostate cancer cells
HCC cell lines Huh7 and PLC/PRF5; Balb/c nude mice received tumour xenografts derived from HCC cells
HeLa and U2OS cells, human xenograft mouse tumor derived from HeLa (#CCL-2) cells.
HeLa and U2OS cells, human xenograft mouse tumor derived from HeLa (#CCL-2) cells.
HepG2, Hepa1-6, Hep3B, and SNU-182 cells, C57BL/6 mice
HepG2, Hepa1-6, Hep3B, and SNU-182 cells
HepG2, Hepa1-6, Hep3B, and SNU-182 cells
HepG2, Hepa1-6, Hep3B, and SNU-182 cells
HepG2, Hepa1-6, Hep3B, and SNU-182 cells
MDA-MB-468, BT-20 cells
HCC cells (HepaG2, Hep3B, and Huh7 cells), nude mice.
MDA MB 231 and SKBr3 cells
HepG2 and Hep3B cells
Pancreatic ductal adenocarcinoma cell lines (PANC1, CFPAC1, MiaPaCa2), nude mice, B6 mice
Pancreatic ductal adenocarcinoma cells (e.g., PANC1, CFPAC1, MiaPaCa2, and Panc2.03)
HCT116, athymic nude mice
Lung cancer H358 and PC9 cells, SCID Mice
A549 cancer cells
A549 cancer cells
MCF-10A and SUM-159 cells
MCF-10A and SUM-159 cells
IMR90 cells
MDA-MB-231 cells, NOD.CB17 Scid/J mice
ME-180 cells
HT-1080 cells
Melanoma cell lines A375 and G-361, C57BL/6 mice
HT1080 cells
UOK262, HT1080, HK2, A498 cells
Head and neck cancer cells
A375 and G-361 cells
A375 and G-361 cells
A375 and G-361 cells
HepG2 cells, female ICR mice
HL60, KG1, THP-1 cells
HEK293, HT-1080 cells
H1299, SK-N-BE(2)C, T24, UM-UC-3, SW780 cells, nude mice
H1299 cells
A549 and SPC-A-1 cells
MDA-MB-231, Hs578T, H1299, A549 and MCF-10A cells, female athymic BALB/c nude mice
H1299 cells
HUVEC cells
HepG2 and AML-12 cell lines, mouse embryonic fibroblasts cells, ICR mice
Mouse embryonic fibroblasts, NF639 cells, human epithelial tumour cells, human mesothelioma cells, xer
Calu-1, THP1, HT1080, and HL-60 cells, athymic nude mice
Calu-1, THP1, HT1080, and HL-60 cells, athymic nude mice
Bel-7402, SMMC-7721 cells, athymic nude mice
Mesothelioma cell lines ACC-Meso-1, NCI-H2373 and NCI-H2052, mesothelial cell line MeT-5A
Hep G2, Huh-7, SMMC-7721 and PLC/PRF/5 cells, nude mice
SGC7901 and MGC803 cells, nude mice
MCF-7, HT1080, Pfa1 cells
ARPE-19, hfRPE cells
Hepatic stellate cells, C57BL/6 mice
MCF10A, Hs578t and MDA-MB-231 cells
PANC1 and HepG2 cells, athymic nude or B6 mice
PANC1 and HepG2 cells, athymic nude or B6 mice
LO2 cells, mice model
HT-1080,786-O, A-498, Caki-1, AU565, DU4475, MCF-7 cells

Pathway	Confidence
RPL8 :+: Ferroptosis	Validated
IREB2 :+: Ferroptosis	Validated
ATP5MC3 :+: Ferroptosis	Validated
CS :+: Ferroptosis	Validated
EMC2 :+: Ferroptosis	Validated
ACSF2 :+: Ferroptosis	Validated
NOX1 :+: Ferroptosis	Deduced
CYBB :+: Ferroptosis	Deduced
NOX3 :+: Ferroptosis	Deduced
NOX4 :+: Ferroptosis	Deduced
NOX5 :+: Ferroptosis	Deduced
DUOX1 :+: Ferroptosis	Deduced
DUOX2 :+: Ferroptosis	Deduced
G6PD :+: Ferroptosis	Validated
PGD :+: Ferroptosis	Validated
VDAC2 :+: Ferroptosis	Validated
TP53 :-: SLC7A11, SLC7A11 :+: Cystine,	Validated
ACSL4 :+: Ferroptosis	Predicted
LPCAT3 :+: Ferroptosis	Predicted
NRAS :+: Ferroptosis	Deduced
KRAS :+: Ferroptosis	Deduced
HRAS :+: Ferroptosis	Deduced
CARS1 :-: Transsulfuration pathway, Tra	Validated
KEAP1 :-: NFE2L2, NFE2L2 :-: Ferroptos	Validated
HMOX1 :+: Lipid ROS, Lipid ROS :+: Fe	Validated
ATG5 :+: Ferroptosis	Validated
ATG7 :+: Ferroptosis	Validated
NCOA4 :-: FTH1, FTH1 :-: Ferroptosis	Validated
ALOX12 :+: PUFAs peroxidation, PUFAs	Validated
ALOX12B :+: PUFAs peroxidation, PUF	Validated
ALOX15 :+: PUFAs peroxidation, PUFAs	Validated
ALOX15B :+: PUFAs peroxidation, PUF	Validated
ALOXE3 :+: PUFAs peroxidation, PUFAs	Validated
PHKG2 :+: PUFAs peroxidation, PUFAs	Validated
SAT1 :+: ALOX15, ALOX15 :+: Lipid RO	Validated
EGFR :+: MAPK, MAPK :+: Ferroptosis	Validated
MAPK :-: GPX4, GPX4 :-: Lipid ROS, MA	Validated
MAPK :-: GPX4, GPX4 :-: Lipid ROS, MA	Validated
ZEB1 :+: Lipid ROS, Lipid ROS :+: Ferro	Validated
DPP4 :+: Lipid ROS, Lipid ROS :+: Ferro	Validated
CDKN2A :-: NFE2L2, NFE2L2 :+: SLC7A	Validated
(PEBP1/15LO) :+: Ferroptosis	Validated
SOCS1 :+: p53, p53 :-: SLC7A11, SLC7A	Validated
CDO1 :-: GPX4, GPX4 :-: Ferroptosis	Validated
MYB :+: CDO1, CDO1 :-: GPX4, GPX4 :-	Validated
CHAC1 :-: GSH, GSH :-: Ferroptosis	Validated
LINC00472 :+: p53, p53 :+: Ferroptosis	Validated
PRKAA2 :+: (BECN1/SLC7A11), (BECN1	Validated
PRKAA1 :+: (BECN1/SLC7A11), (BECN1	Validated
ELAVL1 :+: Ferroptosis	Validated
BAP1 :-: SLC7A11, SLC7A11 :-: Lipid RC	Validated
ABCC1 :-: GSH, GSH :-: Ferroptosis	Validated
MIR6852 :-: CBS, CBS :-: Ferroptosis	Validated
ACVR1B :-: NFE2L2, NFE2L2 :-: Ferropt	Validated
TGFBR1 :-: NFE2L2, NFE2L2 :-: Ferropt	Validated

IFNG :-: SLC3A2, IFNG :-: SLC7A11, SLC	Validated
ANO6 :+: Ferroptosis	Validated
HMGB1 :+: Ferroptosis	Validated
TNFAIP3 :+: ACSL4, ACSL4 :+: Lipid RO	Validated
ATF3 :-: SLC7A11, SLC7A11 :+: System	Validated
ATM :-: MTF1, MTF1 :+: FTL, MTF1 :+: F	Validated
YAP :+: ACSL4, ACSL4 :+: Ferroptosis, Y	Validated
EGLN2 :-: HIF1A, HIF1A :-: Lipid ROS, L	Validated
MIOX :-: GPX4, GPX4 :-: Ferroptosis, M	Validated
TAZ :+: EMP1, EMP1 :+: NOX4, NOX4 :	Validated
MTDH :-: Cysteine, Cystein :-: Ferroptos	Validated
IDH1 :-: GPX4, GPX4 :-: Lipid ROS, Lipic	Validated
FBXW7 :+: Ferroptosis	Deduced
PANX1 :-: HMOX1, HMOX1 :-: Ferropto	Validated
DNAJB6 :-: GPX4, GPX4 :-: Ferroptosis	Predicted
LONP1 :-: Nrf2/Keap1 pathway, Nrf2/K	Validated
Validated	_NA_
Deduced	Inferred as ferroptotic marker
Deduced	Inferred as ferroptotic marker
Deduced	Inferred as ferroptotic marker
Deduced	Inferred as ferroptotic marker
Deduced	Inferred as ferroptotic marker
Deduced	Inferred as ferroptotic marker
Deduced	Inferred as ferroptotic marker
Deduced	Inferred as ferroptotic marker
Deduced	Inferred as ferroptotic marker
Deduced	Inferred as ferroptotic marker
Deduced	Inferred as ferroptotic marker
Deduced	Inferred as ferroptotic marker
Deduced	Inferred as ferroptotic marker
Validated	Further study needed to conf
Deduced	Further study needed to conf
Screened	Further study needed to conf
Screened	Further study needed to conf
Screened	Further study needed to conf
Screened	Further study needed to conf
Screened	Further study needed to conf
Screened	Further study needed to conf
Screened	Further study needed to conf
Screened	Further study needed to conf
Screened	Further study needed to conf
Screened	Further study needed to conf
Screened	Further study needed to conf
Screened	Further study needed to conf
Screened	Further study needed to conf
Screened	Further study needed to conf
Screened	Further study needed to conf
Screened	Further study needed to conf
Screened	Further study needed to conf
Screened	Further study needed to conf
Screened	Further study needed to conf
Screened	Further study needed to conf
Screened	Further study needed to conf
Screened	Further study needed to conf
Screened	Further study needed to conf
Screened	Further study needed to conf
Screened	Further study needed to conf
Screened	Further study needed to conf

Screened	Further study needed to conf
Screened	Further study needed to conf
Screened	Further study needed to conf
Screened	Further study needed to conf
Screened	Further study needed to conf
Screened	Further study needed to conf
Screened	Further study needed to conf
Screened	Further study needed to conf
Screened	Further study needed to conf
Deduced	_NA_
Screened	_NA_
Screened	_NA_
Screened	_NA_
Deduced	_NA_
Deduced	_NA_
Deduced	_NA_
Deduced	_NA_
Deduced	_NA_
Deduced	_NA_
Deduced	_NA_
Validated	An error may exist in the orig
Deduced	_NA_
Deduced	_NA_
Deduced	_NA_
Deduced	_NA_
Deduced	_NA_
Deduced	_NA_
Deduced	_NA_
Deduced	_NA_
Deduced	_NA_
Deduced	_NA_
Deduced	_NA_
Deduced	_NA_
Deduced	Not essential but may play a
Deduced	Not essential but may play a
Deduced	Not essential but may play a
Deduced	_NA_
Deduced	_NA_
Deduced	_NA_
Deduced	_NA_
Deduced	_NA_
Deduced	_NA_
Deduced	_NA_
Deduced	_NA_
Deduced	_NA_
Deduced	_NA_
Deduced	_NA_
Deduced	_NA_
Deduced	_NA_
Predicted	_NA_
Predicted	_NA_
Deduced	_NA_
Deduced	_NA_
Deduced	_NA_
SLC7A11 :+: Cystine, Cystine :-: Ferropt	Validated
GPX4 :-: Lipid ROS, Lipid ROS :+: Ferro	Validated
AKR1C1 :-: Ferroptosis	Validated
AKR1C2 :-: Ferroptosis	Validated

AKR1C3 :-: Ferroptosis	Validated
RB1 :-: Ferroptosis	Validated
HSPB1 :-: Ferroptosis	Validated
HSF1 :-: Ferroptosis	Validated
NFE2L2 :+: (NQO1/HMOX1/FTH1), (NQ	Validated
SQSTM1 :-: KEAP1, KEAP1 :-: NFE2L2, N	Validated
NQO1 :-: Ferroptosis	Validated
HMOX1 :-: Ferroptosis	Validated
FTH1 :-: Ferroptosis	Validated
MUC1 :+: System Xc-, System Xc- :+: G	Validated
MT1G :-: Ferroptosis	Validated
SLC40A1 :-: Lipid ROS, Lipid ROS :+: Fe	Validated
CISD1 :-: Mitochondrial lipid ROS, Mito	Validated
HSPA5 :+: GPX4, GPX4 :-: Lipid ROS, Li	Validated
ATF4 :+: HSPA5, HSPA5 :+: GPX4, GPX4	Validated
TP53 :+: Nucleus DPP4, Nucleus DPP4 :	Validated
HELLS :+: GLUT1, HELLS :+: SCD, HELLS	Validated
SCD :-: Ferroptosis	Validated
FADS2 :-: Ferroptosis	Validated
SRC :+: STAT3, STAT3 :-: ACSL4, ACSL4	Validated
STAT3 :-: ACSL4, ACSL4 :+: Ferroptosis	Validated
PML :-: Ferroptosis	Validated
NFS1 :-: TFRC, TFRC :+: Ferroptosis	Validated
TP63 :-: Ferroptosis	Validated
CDKN1A :+: GSH, GSH :-: Lipid ROS, Li	Validated
MIR137 :-: SLC1A5, SLC1A5 :+: Glutami	Validated
VDAC2 :-: Ferroptosis	Validated
FH :+: GPX4, GPX4 :-: Ferroptosis	Validated
CISD2 :-: Lipid ROS, Lipid ROS :+: Ferro	Validated
MIR9 :-: GOT1, GOT1 :+: alpha KG, alph	Validated
MIR9 :-: GOT1, GOT1 :+: alpha KG, alph	Validated
MIR9 :-: GOT1, GOT1 :+: alpha KG, alph	Validated
CBS :-: Ferroptosis	Validated
ISCU :+: GSH, GSH :-: Ferroptosis	Validated
ACSL3 :-: Ferroptosis	Validated
OTUB1 :+: SLC7A11, SLC7A11 :-: Ferro	Validated
CD44 :+: SLC7A11, SLC7A11 :-: Ferropt	Validated
(LINC00336/ELAVL1) :-: Ferroptosis	Validated
BRD4 :-: Ferritinophagy, Ferritinophagy	Validated
PRDX6 :-: Lipid ROS, Lipid ROS :+: Ferr	Validated
MIR17 :-: A20, A20 :+: ACSL4, ACSL4 :+	Validated
SESN2 :-: Lipid ROS, Lipid ROS :+: Ferro	Validated
NF2 :-: YAP, YAP :+: Ferroptosis	Validated
ARNTL :-: EGLN2, EGLN2 :-: HIF1A, HIF	Validated
HIF1A :-: Lipid ROS, Lipid ROS :+: Ferr	Validated
JUN :+: GSH, GSH :-: Ferroptosis, JUN :	Deduced
CA9 :-: Ferroptosis	Validated
S1R :-: Ferroptosis	Validated
PLIN2 :-: Ferroptosis	Screened
FPS1 :+: CoQ10, CoQ10 :-: Lipid ROS, L	Validated
LAMP2 :+: Cysteine, Cysteine :+: GSH, (	Validated
ZFP36 :-: ATG16L1, ATG16L1 :+: Ferrop	Validated
Prominin2 :+: Ferritin-containing MVB,	Validated
CHMP5 :-: Ferroptosis	Validated
CHMP6 :-: Ferroptosis	Validated
CAV-1 :+: xCT, xCT :+: GPX4, GPX4 :-: F	Validated
GCH1 :+: Phospholipid, Phospholipid :-	Validated

Caution
_NA_
_NA_
_NA_
_NA_
_NA_
_NA_
The presented inhibition study is not sufficient to confirm its role in ferroptosis.
The presented inhibition study is not sufficient to confirm its role in ferroptosis.
The presented inhibition study is not sufficient to confirm its role in ferroptosis.
The presented inhibition study is not sufficient to confirm its role in ferroptosis.
The presented inhibition study is not sufficient to confirm its role in ferroptosis.
The presented inhibition study is not sufficient to confirm its role in ferroptosis.
The presented inhibition study is not sufficient to confirm its role in ferroptosis.
_NA_
_NA_
_NA_
The acetylation-defective mutant (p53[3KR]) also retains the ability to induce ferroptosis upon reactive o
Author prediction based on gene function.
Author prediction based on gene function.
Inferred as a promoter because RAS mutant is resistant to ferroptosis.
Inferred as a promoter because RAS mutant is resistant to ferroptosis.
Inferred as a promoter because RAS mutant is resistant to ferroptosis.
_NA_
_NA_
Other articles (PMID 26403645, 28515173) suggest it a suppressor.
_NA_
_NA_
_NA_
_NA_
_NA_
_NA_
_NA_
_NA_
_NA_
Activated by p53.
_NA_
_NA_
_NA_
_NA_
_NA_
_NA_
_NA_
_NA_
_NA_
_NA_
_NA_
Also triggers apoptosis.
_NA_
_NA_
_NA_
_NA_
_NA_
_NA_
_NA_
_NA_

_NA_
_NA_
_NA_
_NA_
_NA_
_NA_
_NA_
_NA_
_NA_
_NA_
_NA_
_NA_
_NA_
_NA_
_NA_
_NA_
Prostaglandin G/H synthase 2
Dual specificity protein phosphatase 1
Nitric oxide synthase
Neutrophil cytosol factor 2
Metallothionein-3
Polyubiquitin-C
Serum albumin
Thioredoxin reductase 1
Sulfiredoxin-1
Glutathione peroxidase 2
BCL2/adenovirus E1B 19 kDa protein-interacting protein 3
Serine/threonine-protein kinase OSR1
Selenoprotein S
Angiopoietin-related protein 7
Glutathione-specific gamma-glutamylcyclotransferase 1
Cystine/glutamate transporter
DNA damage-inducible transcript 4 protein
_NA_
Asparagine synthetase [glutamine-hydrolyzing]
TSC22 domain family protein 3
DDIT3 upstream open reading frame protein
Jun dimerization protein 2
Sestrin-2
Neutral amino acid transporter A
Phosphoenolpyruvate carboxykinase [GTP]
Thioredoxin-interacting protein
Very low-density lipoprotein receptor
Alanine aminotransferase 2
Phosphoserine aminotransferase
Leucine rich adaptor protein 1-like
Large neutral amino acids transporter small subunit 1
Homocysteine-responsive endoplasmic reticulum-resident ubiquitin-like domain member 1 protein
X-box-binding protein 1
Cyclic AMP-dependent transcription factor ATF-3
4F2 cell-surface antigen heavy chain
Cystathionine beta-synthase
Cyclic AMP-dependent transcription factor ATF-4
Zinc finger protein 419
Kelch-like protein 24
Tribbles homolog 3
Zinc finger protein 69 homolog B
V-type proton ATPase subunit G 2

Vascular endothelial growth factor A
Growth/differentiation factor 15
Tubulin epsilon chain
Arrestin domain-containing protein 3
CCAAT/enhancer-binding protein gamma
_NA_
Regulator of G-protein signaling 4
_NA_
_NA_
Eukaryotic translation initiation factor 2 subunit 1
Estradiol 17-beta-dehydrogenase 11
1-acyl-sn-glycerol-3-phosphate acyltransferase gamma
Histone-lysine N-methyltransferase SETD1B
Heme oxygenase 1
Serotransferrin
Ferritin light chain
60S ribosomal protein L8
ATP synthase F(0) complex subunit C3
Transferrin receptor protein 1
Transcription factor MafG
Ferritin heavy chain
D(1B) dopamine receptor
D(4) dopamine receptor
Mitogen-activated protein kinase kinase kinase 5
Mitogen-activated protein kinase 14
Solute carrier family 2
Solute carrier family 2
Solute carrier family 2
Solute carrier family 2
Solute carrier family 2
_NA_
Solute carrier family 2
eIF-2-alpha kinase GCN2
Arachidonate 5-lipoxygenase
Arachidonate 12-lipoxygenase
Arachidonate 15-lipoxygenase
High mobility group protein B1
ELAV-like protein 1
Hemoglobin subunit alpha
Nicotinamide N-methyltransferase
Perilipin-4
Hypermethylated in cancer 1 protein
Stathmin
Ribonucleoside-diphosphate reductase subunit M2
Macrophage-capping protein
Hepatocyte nuclear factor 4-alpha
Neuroglobin
14-3-3 protein epsilon
GA-binding protein subunit beta-1
Aurora kinase A
_NA_
Receptor-interacting serine/threonine-protein kinase 1
Peroxiredoxin-1
_NA_
_NA_
_NA_
_NA_
_NA_

_NA_
_NA_
_NA_
_NA_
_NA_
_NA_
_NA_
Article (PMID 26405158) suggests it a driver.
_NA_
_NA_
_NA_
_NA_
_NA_
_NA_
_NA_
Promotes ferroptosis via a transcription-dependent mechanism in non-CRC cells.
_NA_
_NA_
_NA_
_NA_
_NA_
_NA_
_NA_
_NA_
_NA_
_NA_
_NA_
_NA_
_NA_
_NA_
_NA_
_NA_
_NA_
_NA_
_NA_
_NA_
_NA_
_NA_
_NA_
_NA_
_NA_
_NA_
_NA_
_NA_
_NA_
_NA_
_NA_
_NA_
_NA_
_NA_
_NA_
_NA_
_NA_
_NA_
_NA_
_NA_
_NA_

Protein encoded
60S ribosomal protein L8
Iron-responsive element-binding protein 2
ATP synthase F(0) complex subunit C3
Citrate synthase
ER membrane protein complex subunit 2
Medium-chain acyl-CoA ligase ACSF2
NADPH oxidase 1
Cytochrome b-245 heavy chain
NADPH oxidase 3
NADPH oxidase 4
NADPH oxidase 5
Dual oxidase 1
Dual oxidase 2
Glucose-6-phosphate 1-dehydrogenase
6-phosphogluconate dehydrogenase
Voltage-dependent anion-selective channel protein 2
Cellular tumor antigen p53
Long-chain-fatty-acid--CoA ligase 4
Lysophospholipid acyltransferase 5
GTPase NRas
GTPase KRas
GTPase HRas
Cysteine--tRNA ligase
Kelch-like ECH-associated protein 1
Heme oxygenase 1
Autophagy protein 5
Ubiquitin-like modifier-activating enzyme ATG7
Nuclear receptor coactivator 4
Arachidonate 12-lipoxygenase
Arachidonate 12-lipoxygenase
Arachidonate 15-lipoxygenase
Arachidonate 15-lipoxygenase B
Hydroperoxide isomerase ALOXE3
Phosphorylase b kinase gamma catalytic chain
Diamine acetyltransferase 1
Epidermal growth factor receptor
Mitogen-activated protein kinase 3
Mitogen-activated protein kinase 1
Zinc finger E-box-binding homeobox 1
Dipeptidyl peptidase 4
Cyclin-dependent kinase inhibitor 2A
Phosphatidylethanolamine-binding protein 1
Suppressor of cytokine signaling 1
Cysteine dioxygenase type 1
Transcriptional activator Myb
Glutathione-specific gamma-glutamylcyclotransferase 1
Putative uncharacterized protein encoded by LINC00472
5'-AMP-activated protein kinase catalytic subunit alpha-2
5'-AMP-activated protein kinase catalytic subunit alpha-1
ELAV-like protein 1
Ubiquitin carboxyl-terminal hydrolase BAP1
Multidrug resistance-associated protein 1
_NA_
Activin receptor type-1B
TGF-beta receptor type-1

Interferon gamma
Anoctamin-6
High mobility group protein B1
Tumor necrosis factor alpha-induced protein 3
Cyclic AMP-dependent transcription factor ATF-3
Serine-protein kinase ATM
YY1-associated protein 1
Egl nine homolog 2
Inositol oxygenase
Tafazzin
Protein LYRIC
Isocitrate dehydrogenase [NADP] cytoplasmic
F-box/WD repeat-containing protein 7
Pannexin-1
DnaJ homolog subfamily B member 6
Lon protease homolog, mitochondrial
P35354 (PGH2_HUMAN)
P28562 (DUS1_HUMAN)
P35228 (NOS2_HUMAN)
P19878 (NCF2_HUMAN)
P25713 (MT3_HUMAN)
P0CG48 (UBC_HUMAN)
P02768 (ALBU_HUMAN)
Q16881 (TRXR1_HUMAN)
Q9BYN0 (SRXN1_HUMAN)
P18283 (GPX2_HUMAN)
Q12983 (BNIP3_HUMAN)
O95747 (OXSR1_HUMAN)
Q9BQE4 (SELS_HUMAN)
O43827 (ANGL7_HUMAN)
Q9BUX1 (CHAC1_HUMAN)
Q9UPY5 (XCT_HUMAN)
Q9NX09 (DDIT4_HUMAN)
_NA_
P08243 (ASNS_HUMAN)
Q99576 (T22D3_HUMAN)
P0DPQ6 (DT3UO_HUMAN)
Q8WYK2 (JDP2_HUMAN)
P58004 (SESN2_HUMAN)
P43007 (SATT_HUMAN)
Q16822 (PCKGM_HUMAN)
Q9H3M7 (TXNIP_HUMAN)
P98155 (VLDLR_HUMAN)
Q8TD30 (ALAT2_HUMAN)
Q9Y617 (SERC_HUMAN)
Q8IV03 (LUR1L_HUMAN)
Q01650 (LAT1_HUMAN)
Q15011 (HERP1_HUMAN)
P17861 (XBP1_HUMAN)
P18847 (ATF3_HUMAN)
P08195 (4F2_HUMAN)
P35520 (CBS_HUMAN)
P18848 (ATF4_HUMAN)
Q96HQ0 (ZN419_HUMAN)
Q6TFL4 (KLH24_HUMAN)
Q96RU7 (TRIB3_HUMAN)
Q9UJL9 (ZF69B_HUMAN)
O95670 (VATG2_HUMAN)

P15692 (VEGFA_HUMAN)
Q99988 (GDF15_HUMAN)
Q9UJT0 (TBE_HUMAN)
Q96B67 (ARRD3_HUMAN)
P53567 (CEBPG_HUMAN)
_NA_
P49798 (RGS4_HUMAN)
_NA_
_NA_
P05198 (IF2A_HUMAN)
Q8NBQ5 (DHB11_HUMAN)
Q9NRZ7 (PLCC_HUMAN)
Q9UPS6 (SET1B_HUMAN)
P09601 (HMOX1_HUMAN)
P02787 (TRFE_HUMAN)
P02792 (FRIL_HUMAN)
P62917 (RL8_HUMAN)
P48201 (AT5G3_HUMAN)
P02786 (TFR1_HUMAN)
O15525 (MAFG_HUMAN)
P02794 (FRIH_HUMAN)
P21918 (DRD5_HUMAN)
P21917 (DRD4_HUMAN)
Q99683 (M3K5_HUMAN)
Q16539 (MK14_HUMAN)
P11166 (GTR1_HUMAN)
P11169 (GTR3_HUMAN)
Q9UGQ3 (GTR6_HUMAN)
Q9NY64 (GTR8_HUMAN)
Q8TD20 (GTR12_HUMAN)
_NA_
Q8TDB8 (GTR14_HUMAN)
Q9P2K8 (E2AK4_HUMAN)
P09917 (LOX5_HUMAN)
P18054 (LOX12_HUMAN)
P16050 (LOX15_HUMAN)
P09429 (HMGB1_HUMAN)
Q15717 (ELAV1_HUMAN)
P69905 (HBA_HUMAN)
P40261 (NNMT_HUMAN)
Q96Q06 (PLIN4_HUMAN)
Q14526 (HIC1_HUMAN)
P16949 (STMN1_HUMAN)
P31350 (RIR2_HUMAN)
P40121 (CAPG_HUMAN)
P41235 (HNF4A_HUMAN)
Q9NPG2 (NGB_HUMAN)
P62258 (1433E_HUMAN)
Q06547 (GABP1_HUMAN)
O14965 (AURKA_HUMAN)
_NA_
Q13546 (RIPK1_HUMAN)
Q06830 (PRDX1_HUMAN)
_NA_
Cystine/glutamate transporter
Phospholipid hydroperoxide glutathione peroxidase
Aldo-keto reductase family 1 member C1
Aldo-keto reductase family 1 member C2

Aldo-keto reductase family 1 member C3
Retinoblastoma-associated protein
Heat shock protein beta-1
Heat shock factor protein 1
Nuclear factor erythroid 2-related factor 2
Sequestosome-1
NAD(P)H dehydrogenase [quinone] 1
Heme oxygenase 1
Ferritin heavy chain
Mucin-1
Metallothionein-1G
Solute carrier family 40 member 1
CDGSH iron-sulfur domain-containing protein 1
Endoplasmic reticulum chaperone BiP
Cyclic AMP-dependent transcription factor ATF-4
Cellular tumor antigen p53
Lymphoid-specific helicase
Acyl-CoA desaturase
Acyl-CoA 6-desaturase
Proto-oncogene tyrosine-protein kinase Src
Signal transducer and activator of transcription 3
Protein PML
Cysteine desulfurase
Tumor protein 63
Cyclin-dependent kinase inhibitor 1
_NA_
Voltage-dependent anion-selective channel protein 2
Fumarate hydratase
CDGSH iron-sulfur domain-containing protein 2
_NA_
_NA_
_NA_
Cystathionine beta-synthase
Iron-sulfur cluster assembly enzyme ISCU
Long-chain-fatty-acid--CoA ligase 3
Ubiquitin thioesterase OTUB1
CD44 antigen
Putative uncharacterized protein encoded by LINC00336
Bromodomain-containing protein 4
Peroxiredoxin-6
_NA_
Sestrin-2
Merlin
Aryl hydrocarbon receptor nuclear translocator-like protein 1
Hypoxia-inducible factor 1-alpha
Transcription factor AP-1
Carbonic anhydrase 9
Protein lifeguard 4
Perilipin-2
Apoptosis-inducing factor 2
Lysosome-associated membrane glycoprotein 2
mRNA decay activator protein ZFP36
Prominin-2
Charged multivesicular body protein 5
Charged multivesicular body protein 6
Caveolin-1
GTP cyclohydrolase 1

UniProtKB	PMID	Remark
P62917 (RL8_HUMAN)	22632970	_NA_
P48200 (IREB2_HUMAN)	22632970	_NA_
P48201 (AT5G3_HUMAN)	22632970	ATP5G3 in article
O75390 (CISY_HUMAN)	22632970	_NA_
Q15006 (EMC2_HUMAN)	22632970	TTC35 in article
Q96CM8 (ACSF2_HUMAN)	22632970	_NA_
Q9Y5S8 (NOX1_HUMAN)	22632970	_NA_
P04839 (CY24B_HUMAN)	22632970	NOX2 in article
Q9HBY0 (NOX3_HUMAN)	22632970	_NA_
Q9NPH5 (NOX4_HUMAN)	22632970	_NA_
Q96PH1 (NOX5_HUMAN)	22632970	_NA_
Q9NRD9 (DUOX1_HUMAN)	22632970	_NA_
Q9NRD8 (DUOX2_HUMAN)	22632970	_NA_
P11413 (G6PD_HUMAN)	22632970	_NA_
P52209 (6PGD_HUMAN)	22632970	_NA_
P45880 (VDAC2_HUMAN)	22632970	_NA_
P04637 (P53_HUMAN)	25799988	_NA_
O60488 (ACSL4_HUMAN)	25965523	_NA_
Q6P1A2 (MBOA5_HUMAN)	25965523	_NA_
P01111 (RASN_HUMAN)	26157704	_NA_
P01116 (RASK_HUMAN)	26157704	_NA_
P01112 (RASH_HUMAN)	26157704	_NA_
P49589 (SYCC_HUMAN)	26184909	CARS in article
Q14145 (KEAP1_HUMAN)	26403645	_NA_
P09601 (HMOX1_HUMAN)	26405158	HO-1 in article
Q9H1Y0 (ATG5_HUMAN)	27245739	_NA_
O95352 (ATG7_HUMAN)	27245739	_NA_
Q13772 (NCOA4_HUMAN)	27245739	_NA_
P18054 (LOX12_HUMAN)	27506793	_NA_
O75342 (LX12B_HUMAN)	27506793	_NA_
P16050 (LOX15_HUMAN)	27506793	_NA_
O15296 (LX15B_HUMAN)	27506793	_NA_
Q9BYJ1 (LOXE3_HUMAN)	27506793	_NA_
P15735 (PHKG2_HUMAN)	27506793	_NA_
P21673 (SAT1_HUMAN)	27698118	_NA_
P00533 (EGFR_HUMAN)	28297659	_NA_
P27361 (MK03_HUMAN)	28297659	ERK1 in article
P28482 (MK01_HUMAN)	28297659	ERK2 in article
P37275 (ZEB1_HUMAN)	28678785	_NA_
P27487 (DPP4_HUMAN)	28813679	_NA_
P42771 (CDN2A_HUMAN)	28985506	ARF in article
P30086 (PEBP1_HUMAN)	29053969	_NA_
O15524 (SOCS1_HUMAN)	29081404	_NA_
Q16878 (CDO1_HUMAN)	29144989	_NA_
P10242 (MYB_HUMAN)	29144989	C-Myb in article
Q9BUX1 (CHAC1_HUMAN)	29383104	_NA_
Q9H8W2 (CF155_HUMAN)	29588351	P53RRA in article
P54646 (AAPK2_HUMAN)	30057310	PRKAA in article
Q13131 (AAPK1_HUMAN)	30057310	AMPK alpha in article
Q15717 (ELAV1_HUMAN)	30081711	_NA_
Q92560 (BAP1_HUMAN)	30202049	_NA_
P33527 (MRP1_HUMAN)	30726737	MRP1 in article
_NA_	30787392	_NA_
P36896 (ACV1B_HUMAN)	30804470	ALK4 in article
P36897 (TGFR1_HUMAN)	30804470	ALK5 in article

P01579 (IFNG_HUMAN)			31043744	IFNgamma in article
Q4KMQ2 (ANO6_HUMAN)			31060306	_NA_
P09429 (HMGB1_HUMAN)			31105999	_NA_
P21580 (TNAP3_HUMAN)			31160087	Zinc lipoprotein A20 in article
P18847 (ATF3_HUMAN)			31273299	_NA_
Q13315 (ATM_HUMAN)			31320750	_NA_
Q9H869 (YYAP1_HUMAN)			31341276	YAP in article
Q96KS0 (EGLN2_HUMAN)			31355331	_NA_
Q9UGB7 (MIOX_HUMAN)			31437128	_NA_
Q16635 (TAZ_HUMAN)			31484063	_NA_
Q86UE4 (LYRIC_HUMAN)			31527591	_NA_
O75874 (IDHC_HUMAN)			31591388	_NA_
Q969H0 (FBXW7_HUMAN)			31679460	_NA_
Q96RD7 (PANX1_HUMAN)			31694915	_NA_
O75190 (DNJB6_HUMAN)			31701262	_NA_
P36776 (LONM_HUMAN)			31822343	_NA_
	24439385	May promote ferroptosis.		marker
	24439385	May promote ferroptosis.		marker
	24439385	May promote ferroptosis.		marker
	24439385	May promote ferroptosis.		marker
	24439385	May promote ferroptosis.		marker
	24439385	May promote ferroptosis.		marker
	24439385	May promote ferroptosis.		marker
	24439385	May promote ferroptosis.		marker
	24439385	May promote ferroptosis.		marker
	24439385	May promote ferroptosis.		marker
	24439385	May promote ferroptosis.		marker
	24439385	May promote ferroptosis.		marker
	24439385	SELS in article. May promote		marker
	24439385	May promote ferroptosis.		marker
	24844246	_NA_		marker
	24844246	May inhibit ferroptosis.		marker
	24844246	May promote ferroptosis.		marker
	24844246	Not found in HGNC. May pro		marker
	24844246	May promote ferroptosis.		marker
	24844246	May promote ferroptosis.		marker
	24844246	May promote ferroptosis.		marker
	24844246	May promote ferroptosis.		marker
	24844246	May promote ferroptosis.		marker
	24844246	May promote ferroptosis.		marker
	24844246	May promote ferroptosis.		marker
	24844246	May promote ferroptosis.		marker
	24844246	May promote ferroptosis.		marker
	24844246	May promote ferroptosis.		marker
	24844246	May promote ferroptosis.		marker
	24844246	C9ORF150 in article. May pro		marker
	24844246	May promote ferroptosis.		marker
	24844246	May promote ferroptosis.		marker
	24844246	May promote ferroptosis.		marker
	24844246	May promote ferroptosis.		marker
	24844246	May promote ferroptosis.		marker
	24844246	May promote ferroptosis.		marker
	24844246	May promote ferroptosis.		marker
	24844246	May promote ferroptosis.		marker
	24844246	May promote ferroptosis.		marker
	24844246	May promote ferroptosis.		marker
	24844246	ZNF643 in article. May promo		marker
	24844246	May promote ferroptosis.		marker

	24844246	May promote ferroptosis.	marker
	24844246	May promote ferroptosis.	marker
	24844246	May promote ferroptosis.	marker
	24844246	May promote ferroptosis.	marker
	24844246	May promote ferroptosis.	marker
	24844246	May inhibit ferroptosis.	marker
	24844246	May inhibit ferroptosis.	marker
	24844246	MUTED-TXNDC5 in article. M	marker
	24844246	Not found in HGNC. May inh	marker
	24844246	eIF2alpha in article. May pro	marker
	25965523	May promote ferroptosis.	marker
	25965523	May promote ferroptosis.	marker
	25965523	May promote ferroptosis.	marker
	26097885	HO-1 in article. May promote	marker
	26097885	May promote ferroptosis.	marker
	26097885	May promote ferroptosis.	marker
	26097885	May promote ferroptosis.	marker
	26097885	ATP5G3 in article. May prom	marker
	26097885	May inhibit ferroptosis.	marker
	26403645	May inhibit ferroptosis.	marker
	27514700	_NA_	marker
	27793671	May promote ferroptosis.	marker
	27793671	May inhibit ferroptosis.	marker
	28887319	ASK1 in article. May promote	marker
	28887319	p38 in article. May promote f	marker
	28900510	GLUT1 in article. May inhibit	marker
	28900510	GLUT3 in article. May inhibit	marker
	28900511	GLUT6 in article. May inhibit	marker
	28900512	GLUT8 in article. May inhibit	marker
	28900513	GLUT12 in article. May inhibit	marker
	28900514	Not found in HGNC. May inh	marker
	28900515	GLUT14 in article. May inhibit	marker
	29383104	GCN2 in article. May promote	marker
	29632885	5-LOX in article	marker
	29632885	p12-LOX in article	marker
	29632885	15-LOX-1 in article	marker
	30686534	May promote ferroptosis.	marker
	30787392	May inhibit ferroptosis.	marker
	31108460	May promote ferroptosis.	marker
	31108460	May promote ferroptosis.	marker
	31108460	May promote ferroptosis.	marker
	31108460	May promote ferroptosis.	marker
	31108460	May inhibit ferroptosis.	marker
	31108460	May inhibit ferroptosis.	marker
	31108460	May inhibit ferroptosis.	marker
	31108460	May inhibit ferroptosis.	marker
	31405213	May inhibit ferroptosis.	marker
	31581313	14-3-3epsilon in article. May	marker
	31700067	May inhibit ferroptosis.	marker
	31740746	May inhibit ferroptosis.	marker
	31740746	miR-4715-3p in article. May	marker
	31827280	May promote ferroptosis.	marker
	31901729	May promote ferroptosis.	marker
	31926626	miR-30b-5p in article. May p	marker
Q9UPY5 (XCT_HUMAN)		22632970	_NA_
P36969 (GPX4_HUMAN)		24439385	_NA_
Q04828 (AK1C1_HUMAN)		24844246	_NA_
P52895 (AK1C2_HUMAN)		24844246	_NA_

P42330 (AK1C3_HUMAN)	24844246 _NA_
P06400 (RB_HUMAN)	25444922 _NA_
P04792 (HSPB1_HUMAN)	25728673 _NA_
Q00613 (HSF1_HUMAN)	25728673 _NA_
Q16236 (NF2L2_HUMAN)	26403645 NRF2 in article
Q13501 (SQSTM_HUMAN)	26403645 p62 in article.
P15559 (NQO1_HUMAN)	26403645 _NA_
P09601 (HMOX1_HUMAN)	26403645 HO1 in article
P02794 (FRIH_HUMAN)	26403645 _NA_
P15941 (MUC1_HUMAN)	26930718 _NA_
P13640 (MT1G_HUMAN)	27015352 MT-1G in article
Q9NP59 (S40A1_HUMAN)	27441659 Ferroportin-1 (FPN) in article
Q9NZ45 (CISD1_HUMAN)	27510639 _NA_
P11021 (BIP_HUMAN)	28130223 _NA_
P18848 (ATF4_HUMAN)	28130223 _NA_
P04637 (P53_HUMAN)	28813679 _NA_
Q9NRZ9 (HELLS_HUMAN)	28900510 LSH in article
O00767 (ACOD_HUMAN)	28900510 SCD1 in article
O95864 (FADS2_HUMAN)	28900510 _NA_
P12931 (SRC_HUMAN)	28972104 _NA_
P40763 (STAT3_HUMAN)	28972104 _NA_
P29590 (PML_HUMAN)	29081404 _NA_
Q9Y697 (NFS1_HUMAN)	29168506 _NA_
Q9H3D4 (P63_HUMAN)	29212036 Delta Np63 alpha in article
P38936 (CDN1A_HUMAN)	29346757 Encoding p21
_NA_	29348676 _NA_
P45880 (VDAC2_HUMAN)	29569437 _NA_
P07954 (FUMH_HUMAN)	29917289 _NA_
Q8N5K1 (CISD2_HUMAN)	29928961 _NA_
_NA_	30035324 miR-9 in article
_NA_	30035324 miR-9 in article
_NA_	30035324 miR-9 in article
P35520 (CBS_HUMAN)	30258181 _NA_
Q9H1K1 (ISCU_HUMAN)	30557609 _NA_
O95573 (ACSL3_HUMAN)	30686757 _NA_
Q96FW1 (OTUB1_HUMAN)	30709928 _NA_
P16070 (CD44_HUMAN)	30709928 _NA_
Q6ZUF6 (NC336_HUMAN)	30787392 _NA_
O60885 (BRD4_HUMAN)	30988278 _NA_
P30041 (PRDX6_HUMAN)	31036877 _NA_
_NA_	31160087 miR-17-92 in article
P58004 (SESN2_HUMAN)	31323261 Sesn2 in article
P35240 (MERL_HUMAN)	31341276 Also known as merlin
O00327 (BMAL1_HUMAN)	31355331 _NA_
Q16665 (HIF1A_HUMAN)	31355331 _NA_
P05412 (JUN_HUMAN)	31394193 c-Jun in article
Q16790 (CAH9_HUMAN)	31442913 _NA_
Q9HC24 (LFG4_HUMAN)	31507082 S1R in article
Q99541 (PLIN2_HUMAN)	31520166 Also known as ADRP
Q9BRQ8 (AIFM2_HUMAN)	31634899 FSP1 in article
P13473 (LAMP2_HUMAN)	31672277 _NA_
P26651 (TTP_HUMAN)	31679460 _NA_
Q8N271 (PROM2_HUMAN)	31735663 Prominin2 in article
Q9NZZ3 (CHMP5_HUMAN)	31761326 _NA_
Q96FZ7 (CHMP6_HUMAN)	31761326 _NA_
Q03135 (CAV1_HUMAN)	31877357 Cav-1 in article
P30793 (GCH1_HUMAN)	31989025 _NA_

Subtypes
driver
driver
driver
driver
driver
driver
driver
driver
driver
driver
driver
driver
driver
driver
driver
driver
driver
driver
driver
driver
driver
driver
driver
driver
driver
driver
driver
driver
driver
driver
driver
driver
driver
driver
driver
driver
driver
driver
driver
driver
driver
driver
driver
driver
driver
driver
driver
driver
driver
driver
driver
driver
driver
driver
driver

driver
driver
driver
driver
driver
driver
driver
driver
driver
driver
driver
driver
driver
driver
driver
driver
marker
marker
marker
marker
marker
marker
marker
marker
marker
marker
marker
marker
marker
marker
marker
marker
marker
marker
marker
marker
marker
marker
marker
marker
marker
marker
marker
marker
marker
marker
marker
marker
marker
marker
marker
marker
marker
marker
marker
marker
marker
marker

marker
marker
marker
marker
marker
marker
marker
marker
marker
marker
marker
marker
marker
marker
marker
marker
marker
marker
marker
marker
marker
marker
marker
marker
marker
marker
marker
marker
marker
marker
marker
marker
marker
marker
marker
marker
marker
marker
marker
marker
marker
marker
marker
marker
marker
marker
marker
marker
marker
marker
marker
marker
marker
marker
suppressor
suppressor
suppressor
suppressor

suppressor
suppressor
suppressor
suppressor
suppressor
suppressor
suppressor
suppressor
suppressor
suppressor
suppressor
suppressor
suppressor
suppressor
suppressor
suppressor
suppressor
suppressor
suppressor
suppressor
suppressor
suppressor
suppressor
suppressor
suppressor
suppressor
suppressor
suppressor
suppressor
suppressor
suppressor
suppressor
suppressor
suppressor
suppressor
suppressor
suppressor
suppressor
suppressor
suppressor
suppressor
suppressor
suppressor
suppressor
suppressor
suppressor
suppressor
suppressor
suppressor
suppressor
suppressor
suppressor
suppressor
suppressor
suppressor
suppressor
suppressor

Table S4. The annotation of single cells

cell name	annotation
SMC01.T.AAACCTGCATACGCCG	CC1
SMC01.T.AAACCTGGTCGCATAT	CC1
SMC01.T.AAACCTGTCCCTTGCA	CC1
SMC01.T.AAACGGGAGGGAAACA	CC1
SMC01.T.AAACGGGGTATAGGTA	CC2
SMC01.T.AAAGATGAGGCCGAAT	CC1
SMC01.T.AAAGATGCATGGATGG	CC1
SMC01.T.AAAGATGTCACGACTA	CC2
SMC01.T.AAAGATGTCCGTTGCT	CC1
SMC01.T.AAAGCAACAGTCGATT	CC1
SMC01.T.AAAGTAGAGAGGTACC	CC1
SMC01.T.AAAGTAGAGGGCTTGA	CC2
SMC01.T.AAAGTAGCAAGGACAC	CC1
SMC01.T.AAAGTAGCAAGTAGTA	CC1
SMC01.T.AAAGTAGTCAGCATGT	CC1
SMC01.T.AAAGTAGTCTAACTGG	CC1
SMC01.T.AAAGTAGTCTCGCATC	CC1
SMC01.T.AAATGCCAGGATCGCA	CC1
SMC01.T.AAATGCCTCATTTGGG	CC1
SMC01.T.AACACGTTCTGGCGAC	CC1
SMC01.T.AACCATGCATCACGTA	CC1
SMC01.T.AACCATGCATCGACGC	CC1
SMC01.T.AACCATGGTATAGTAG	CC1
SMC01.T.AACCATGTCACAGTAC	CC1
SMC01.T.AACCATGTCCTTGACC	CC1
SMC01.T.AACCGCGAGTAGCGGT	CC1
SMC01.T.AACCGCGCAGCTCGAC	CC2
SMC01.T.AACCGCGTCTAACCGA	CC2
SMC01.T.AACCGCGTCTTTACGT	CC1
SMC01.T.AACGTTGGTAAGGGAA	CC1
SMC01.T.AACGTTGGTGCGGTAA	CC2
SMC01.T.AACTCAGGTTCCGGCA	CC1
SMC01.T.AACTCCCGTTTGTTGG	CC1
SMC01.T.AACTCCCTCTGCAGTA	CC1
SMC01.T.AACTCTTAGTCCGTAT	CC1
SMC01.T.AACTCTTCACAACGCC	CC1
SMC01.T.AACTCTTGTTCGTCTC	CC1
SMC01.T.AACTCTTGTTGCGTTA	CC1
SMC01.T.AACTCTTTCTGGTATG	CC1
SMC01.T.AACTTTCAGGCTAGCA	CC1
SMC01.T.AACTTTCGTACTCGCG	CC1
SMC01.T.AACTTTCGTTCGGGCT	CC1
SMC01.T.AAGACCTAGGTAAACT	CC1
SMC01.T.AAGACCTCAGTGGAGT	CC2
SMC01.T.AAGCCGCTCCAAACTG	CC1
SMC01.T.AAGGAGCAGCGTTCCG	CC1
SMC01.T.AAGGAGCCACATGGGA	CC1
SMC01.T.AAGGAGCCACTGAAGG	CC2
SMC01.T.AAGGAGCCATCGGACC	CC1
SMC01.T.AAGGAGCTCAGGCAAG	CC1
SMC01.T.AAGGAGCTCTAGAGTC	CC1
SMC01.T.AAGGCAGCAAAGGCGT	CC1
SMC01.T.AAGGTTCCACGTCTCT	CC1
SMC01.T.AAGGTTCTCCAATGGT	CC1
SMC01.T.AAGTCTGAGTAAGTAC	CC2
SMC01.T.AAGTCTGAGTGGTCCC	CC1

SMC01.T.AATCCAGAGTGCGTGA	CC1
SMC01.T.AATCCAGGTTTGTTGG	CC2
SMC01.T.AATCCAGTCCGCGGTA	CC1
SMC01.T.AATCGGTAGTGGAGTC	CC2
SMC01.T.AATCGGTAGTGGCACA	CC1
SMC01.T.AATCGGTCAATCGAAA	CC1
SMC01.T.AATCGGTGTCCAACTA	CC1
SMC01.T.ACACCAAGTACAGTTC	CC2
SMC01.T.ACACCAATCTCATTCA	CC1
SMC01.T.ACACTGACATTCTCAT	CC2
SMC01.T.ACACTGATCCTCCTAG	CC1
SMC01.T.ACAGCCGGTCTAGCCG	CC1
SMC01.T.ACAGCCGTCCCTAATT	CC1
SMC01.T.ACAGCTAGTTTGGCGC	CC1
SMC01.T.ACAGCTATCTGATTCT	CC1
SMC01.T.ACATACGAGTTGAGAT	CC2
SMC01.T.ACATACGCACGAAACG	CC1
SMC01.T.ACATACGTCTATCCCG	CC1
SMC01.T.ACATCAGGTAGCGTGA	CC1
SMC01.T.ACATCAGTCAGAGACG	CC1
SMC01.T.ACCAGTAAGATCGGGT	CC1
SMC01.T.ACCAGTATCGTCCAGG	CC1
SMC01.T.ACCCACTGTACTTAGC	CC1
SMC01.T.ACCGTAATCCCTGACT	CC1
SMC01.T.ACCTTTAAGTCGATAA	CC1
SMC01.T.ACCTTTATCCCACTTG	CC1
SMC01.T.ACCTTTATCGGTTCGG	CC1
SMC01.T.ACGAGCCCACATTCGA	CC1
SMC01.T.ACGAGGACAATCAGAA	CC1
SMC01.T.ACGAGGAGTCTCTCTG	CC2
SMC01.T.ACGATACGTAGGGTAC	CC1
SMC01.T.ACGCAGCAGGTTACCT	CC1
SMC01.T.ACGCAGCGTGTAAGTA	CC2
SMC01.T.ACGCAGCTCTACCTGC	CC1
SMC01.T.ACGCCAGCACTGCCAG	CC1
SMC01.T.ACGCCGAAGCTGCCCA	CC2
SMC01.T.ACGCCGAGTAACGTTC	CC1
SMC01.T.ACGCCGAGTCGTTGTA	CC2
SMC01.T.ACGCCGATCTAACTGG	CC1
SMC01.T.ACGGAGAAGGATGGAA	CC2
SMC01.T.ACGGAGACACGGTGTC	CC1
SMC01.T.ACGGCCAGTCATGCCG	CC1
SMC01.T.ACGGGCTAGGTGCAAC	CC1
SMC01.T.ACGGGCTAGTTCGCGC	CC1
SMC01.T.ACGGGTCAGATCGATA	CC1
SMC01.T.ACGGGTCAGTGTCCAT	CC1
SMC01.T.ACTATCTCACCTCGGA	CC1
SMC01.T.ACTATCTCATATGGTC	CC1
SMC01.T.ACTATCTGTATAGTAG	CC2
SMC01.T.ACTATCTTCACTTCAT	CC1
SMC01.T.ACTGAACAGGTGCTTT	CC1
SMC01.T.ACTGAACCACAAGACG	CC2
SMC01.T.ACTGAGTGTTGACGTT	CC1
SMC01.T.ACTGAGTTCAATACCG	CC1
SMC01.T.ACTGATGAGGTTACCT	CC1
SMC01.T.ACTGATGTCAGCTCGG	CC2
SMC01.T.ACTGATGTCCGCATCT	CC1
SMC01.T.ACTGCTCAGCTCAACT	CC1

SMC01.T.ACTGCTCGTTAGAACA	CC1
SMC01.T.ACTGTCCAGCTGAAAT	CC1
SMC01.T.ACTGTCCAGTCCCACG	CC1
SMC01.T.ACTGTCCGTTTGTGTG	CC2
SMC01.T.ACTGTCCTCAATACCG	CC2
SMC01.T.ACTGTCCTCACGATGT	CC1
SMC01.T.ACTGTCCTCCAGGGCT	CC1
SMC01.T.ACTGTCCTCGCCAGCA	CC1
SMC01.T.ACTTACTGTTACGACT	CC1
SMC01.T.ACTTACTGTTAGGGTG	CC2
SMC01.T.ACTTGTTAGACGCACA	CC1
SMC01.T.ACTTGTTAGGCCATAG	CC1
SMC01.T.ACTTGTTCATATGAGA	CC1
SMC01.T.ACTTTCAGTAGGCATG	CC1
SMC01.T.ACTTTCATCCTTTCTC	CC1
SMC01.T.AGACGTTGTGGCTCCA	CC1
SMC01.T.AGAGCGACACCCTATC	CC1
SMC01.T.AGAGCGACATGGATGG	CC1
SMC01.T.AGAGCGAGTCGCGTGT	CC1
SMC01.T.AGAGCTTAGAGCCTAG	CC1
SMC01.T.AGAGCTTAGGTGCTTT	CC1
SMC01.T.AGAGCTTAGTTTAGGA	CC1
SMC01.T.AGAGTGGAGACACTAA	CC2
SMC01.T.AGAGTGGTCTGTGCAA	CC2
SMC01.T.AGATCTGAGCCTATGT	CC1
SMC01.T.AGATCTGCATCAGTCA	CC1
SMC01.T.AGATCTGGTGATGTCT	CC2
SMC01.T.AGATTGCAGTGTGAAT	CC1
SMC01.T.AGCAGCCGTAGATTAG	CC2
SMC01.T.AGCAGCCGTATGAAAC	CC1
SMC01.T.AGCAGCCTCAAAGTAG	CC1
SMC01.T.AGCAGCCTCTAGAGTC	CC1
SMC01.T.AGCATACAGATGGCGT	CC1
SMC01.T.AGCATACAGCAGGTCA	CC1
SMC01.T.AGCATACCACCGGAAA	CC1
SMC01.T.AGCATACTCCACGTGG	CC1
SMC01.T.AGCATACTCCGCGCAA	CC1
SMC01.T.AGCATACTCTTACCTA	CC2
SMC01.T.AGCCTAACAAGTTCTG	CC2
SMC01.T.AGCCTAACAGTTCCCT	CC1
SMC01.T.AGCCTAATCAGCGATT	CC1
SMC01.T.AGCGTATAGAGCTGCA	CC1
SMC01.T.AGCGTATCAGGTGGAT	CC2
SMC01.T.AGCGTCGAGATTACCC	CC1
SMC01.T.AGCGTCGGTGTGGCTC	CC1
SMC01.T.AGCTCCTTCACATGCA	CC1
SMC01.T.AGCTCTCAGCGATAGC	CC1
SMC01.T.AGCTCTCAGGTGACCA	CC1
SMC01.T.AGCTCTCCAACGATGG	CC1
SMC01.T.AGCTTGATCACTCCTG	CC1
SMC01.T.AGGCCACAGCTGCCCA	CC1
SMC01.T.AGGCCACGTTAAGAAC	CC2
SMC01.T.AGGCCACTCCGCTGTT	CC1
SMC01.T.AGGCCGTGTGGTGTAG	CC1
SMC01.T.AGGCCGTTCAGAAATG	CC1
SMC01.T.AGGCCGTTCATTTGGG	CC1
SMC01.T.AGGGAGTAGTGACATA	CC1
SMC01.T.AGGGAGTCAATCACAC	CC1

SMC01.T.AGGGATGGTTCCACGG	CC1
SMC01.T.AGGGATGTCACCTTAT	CC2
SMC01.T.AGGGTGAGTCGCCATG	CC1
SMC01.T.AGGGTGATCACGAAGG	CC1
SMC01.T.AGGTCATAGCATCATC	CC1
SMC01.T.AGGTCATTCCCGACTT	CC1
SMC01.T.AGTAGTCTCTCAAGTG	CC1
SMC01.T.AGTCTTTTCTATCCTA	CC1
SMC01.T.AGTGAGGAGTGTCCCG	CC1
SMC01.T.AGTGAGGGTGCGGTAA	CC1
SMC01.T.AGTGGGAAGTTACGGG	CC2
SMC01.T.AGTGGGAGTCGGCTCA	CC1
SMC01.T.ATAACGCAGCCCAATT	CC1
SMC01.T.ATAACGCCATCCCACT	CC1
SMC01.T.ATAACGCGTTTGACTG	CC2
SMC01.T.ATAAGAGCAAATTGCC	CC1
SMC01.T.ATAAGAGTCAAGAAGT	CC1
SMC01.T.ATAGACCAGCAGGCTA	CC1
SMC01.T.ATAGACCTCTTTACGT	CC1
SMC01.T.ATCACGACATTAGGCT	CC1
SMC01.T.ATCATCTCATCTATGG	CC2
SMC01.T.ATCATGGCAACGATCT	CC1
SMC01.T.ATCATGGGTACGCTGC	CC1
SMC01.T.ATCATGGTCGGTCTAA	CC1
SMC01.T.ATCCACCTCTGCGGCA	CC1
SMC01.T.ATCCGAATCCGCATCT	CC1
SMC01.T.ATCGAGTCATCGGAAG	CC1
SMC01.T.ATCTACTAGGCATTGG	CC2
SMC01.T.ATCTACTGTATTCGTG	CC2
SMC01.T.ATCTACTTCTGGCGAC	CC1
SMC01.T.ATCTGCCCAAATACAG	CC1
SMC01.T.ATGCGATGTTGGTTTG	CC1
SMC01.T.ATGCGATTCCATGAGT	CC2
SMC01.T.ATGGGAGAGTTCCACA	CC1
SMC01.T.ATGGGAGCACGAGGTA	CC1
SMC01.T.ATGGGAGTCCAACCAA	CC1
SMC01.T.ATGTGTGAGGCAAAGA	CC1
SMC01.T.ATGTGTGCAAAGAATC	CC1
SMC01.T.ATGTGTGTCCGCATCT	CC1
SMC01.T.ATTACTCCATGGGACA	CC1
SMC01.T.ATTATCCAGGGCTTCC	CC1
SMC01.T.ATTATCCGTTCGGGCT	CC1
SMC01.T.ATTATCCTCAGCATGT	CC1
SMC01.T.ATTCTACCACGTGAGA	CC1
SMC01.T.ATTCTACGTAAGCACG	CC1
SMC01.T.ATTCTACGTCAAAGCG	CC1
SMC01.T.ATTCTACTCTTATCTG	CC1
SMC01.T.ATTGGACAGATGTGTA	CC2
SMC01.T.ATTGGACGTGGTAACG	CC1
SMC01.T.ATTGGTGCAATGGACG	CC1
SMC01.T.ATTGGTGTCACCACCT	CC2
SMC01.T.ATTTCTGGTAGCAAAT	CC1
SMC01.T.CAACCTCAGCGACGTA	CC1
SMC01.T.CAACCTCCATCACAAC	CC1
SMC01.T.CAACCTCGTCAGAATA	CC1
SMC01.T.CAACCTCTCGCCGTGA	CC1
SMC01.T.CAACTAGAGACTTGAA	CC2
SMC01.T.CAACTAGAGGCGATAC	CC1

SMC01.T.CAACTAGAGTGCAAGC	CC1
SMC01.T.CAACTAGGTCCTGCTT	CC1
SMC01.T.CAACTAGGTCTAGCGC	CC1
SMC01.T.CAACTAGTCGTTTGCC	CC1
SMC01.T.CAAGATCCAGCATACT	CC2
SMC01.T.CAAGATCCATGAACCT	CC1
SMC01.T.CAAGGCCCAAGGTTCT	CC2
SMC01.T.CAAGGCCCACGCCAGT	CC1
SMC01.T.CAAGTTGGTCATGCAT	CC1
SMC01.T.CAAGTTGGTGATGTGG	CC1
SMC01.T.CAAGTTGGTGCACGAA	CC1
SMC01.T.CAAGTTGTCTACCAGA	CC1
SMC01.T.CAAGTTGTCTTCGAGA	CC1
SMC01.T.CACAAACGTCTCAACA	CC1
SMC01.T.CACACAACAATCTGCA	CC1
SMC01.T.CACACAACACACTGCG	CC1
SMC01.T.CACACAATCAGTTTGG	CC1
SMC01.T.CACACAATCATTCACT	CC1
SMC01.T.CACACCTCATGAAGTA	CC2
SMC01.T.CACACCTCATGTAGTC	CC1
SMC01.T.CACACCTTCAGTCAGT	CC2
SMC01.T.CACACTCAGGATGGAA	CC1
SMC01.T.CACACTCGTAAGGATT	CC1
SMC01.T.CACACTCGTAGAGTGC	CC1
SMC01.T.CACACTCGTTATCGGT	CC1
SMC01.T.CACAGGCCATCCAACA	CC1
SMC01.T.CACAGGCTCACTTCAT	CC1
SMC01.T.CACAGTAAGTACGATA	CC1
SMC01.T.CACAGTAGTCGCCATG	CC1
SMC01.T.CACAGTATCGCCATAA	CC1
SMC01.T.CACATAGAGGCCCTTG	CC1
SMC01.T.CACATAGAGTCCAGGA	CC1
SMC01.T.CACATAGCAAGACACG	CC1
SMC01.T.CACATAGTCTTAACCT	CC1
SMC01.T.CACATTTAGACCTAGG	CC2
SMC01.T.CACATTTAGCAGGCTA	CC1
SMC01.T.CACATTTAGTTGTCGT	CC1
SMC01.T.CACCACTCAGCTGTTA	CC1
SMC01.T.CACCACTCAGGACGTA	CC1
SMC01.T.CACCAGGAGCGATTCT	CC1
SMC01.T.CACCAGGGTGGTCTCG	CC1
SMC01.T.CACCAGGTCAAAGTAG	CC1
SMC01.T.CACCTTGGTTCGCGAC	CC1
SMC01.T.CACTCCACACCAGTTA	CC1
SMC01.T.CACTCCAGTCTAGCGC	CC1
SMC01.T.CAGAATCCAAGACGTG	CC1
SMC01.T.CAGAATCCAGCTGTTA	CC1
SMC01.T.CAGAATCGTCGATTGT	CC2
SMC01.T.CAGAGAGCAAACCTAC	CC1
SMC01.T.CAGATCAAGCACGCCT	CC1
SMC01.T.CAGATCACAGCTGGCT	CC1
SMC01.T.CAGATCAGTCTAGGTT	CC1
SMC01.T.CAGATCATCATTATCC	CC1
SMC01.T.CAGCAGCGTCAAAGCG	CC1
SMC01.T.CAGCAGCGTGAAGGCT	CC1
SMC01.T.CAGCAGCGTTGAGGTG	CC1
SMC01.T.CAGCATAAGATGCGAC	CC1
SMC01.T.CAGCCGAAGCTGAACG	CC1

SMC01.T.CAGCCGATCGATAGAA	CC1
SMC01.T.CAGCGACAGAGTCGGT	CC1
SMC01.T.CAGCGACAGGCCCGTT	CC1
SMC01.T.CAGCGACAGGGCTTGA	CC2
SMC01.T.CAGCTAAGTCTGCAAT	CC1
SMC01.T.CAGCTAATCAGTGCAT	CC1
SMC01.T.CAGCTGGAGCGCTTAT	CC1
SMC01.T.CAGCTGGCACATCCAA	CC1
SMC01.T.CAGCTGGCAGACGCCT	CC1
SMC01.T.CAGCTGGGTCACTTCC	CC2
SMC01.T.CAGCTGGTCAAGGTAA	CC1
SMC01.T.CAGCTGGTCCGTAGGC	CC1
SMC01.T.CAGGTGCAGGTAGCTG	CC1
SMC01.T.CAGGTGCCACTAGTAC	CC1
SMC01.T.CAGGTGCCAGATGGGT	CC1
SMC01.T.CAGTAACGTTAAAGTG	CC1
SMC01.T.CAGTCCTAGTCTCGGC	CC1
SMC01.T.CAGTCCTCACCTATCC	CC1
SMC01.T.CAGTCCTTCATCGCTC	CC1
SMC01.T.CATATTCAGGCATGGT	CC2
SMC01.T.CATATTCAGTGATCGG	CC1
SMC01.T.CATATTCCATACCATG	CC1
SMC01.T.CATATTCGTACCCAAT	CC1
SMC01.T.CATATTCGTAGGGTAC	CC1
SMC01.T.CATATTCGTGTCAATC	CC1
SMC01.T.CATCAAGAGCCGGTAA	CC1
SMC01.T.CATCAAGCACAAGACG	CC1
SMC01.T.CATCAAGCAGTATCTG	CC1
SMC01.T.CATCAAGCATGCCTTC	CC1
SMC01.T.CATCAAGGTCTTGATG	CC1
SMC01.T.CATCAAGTCACAAACC	CC1
SMC01.T.CATCAGAGTTTCGCTC	CC1
SMC01.T.CATCCACGTATAGGTA	CC1
SMC01.T.CATCCACTCACGATGT	CC1
SMC01.T.CATCGAAAGGAGTCTG	CC1
SMC01.T.CATCGAAGTTAAGATG	CC1
SMC01.T.CATCGAATCATGTGGT	CC1
SMC01.T.CATCGAATCTTGTATC	CC2
SMC01.T.CATCGGGAGACTGGGT	CC1
SMC01.T.CATGACAAGGGTTTCT	CC1
SMC01.T.CATGACACAGCCACCA	CC1
SMC01.T.CATGGCGTCCCAACGG	CC1
SMC01.T.CATTATCTCGCCAGCA	CC1
SMC01.T.CATTATCTCGGTCCGA	CC1
SMC01.T.CATTCGCTCGCTGATA	CC1
SMC01.T.CCAATCCTCTTGAGAC	CC1
SMC01.T.CCACCTAAGAACAACT	CC1
SMC01.T.CCACCTACAGGGATTG	CC1
SMC01.T.CCACCTAGTGTGCCTG	CC1
SMC01.T.CCACCTATCAGGTTCA	CC1
SMC01.T.CCACCTATCATCTGCC	CC1
SMC01.T.CCACGGAAGCGCTTAT	CC1
SMC01.T.CCAGCGAAGAATGTGT	CC1
SMC01.T.CCAGCGAGTCAGATAA	CC2
SMC01.T.CCATGTCAGCAGGCTA	CC1
SMC01.T.CCATGTCGTGAAGGCT	CC1
SMC01.T.CCATTCGGTCTGCGGT	CC1
SMC01.T.CCCAATCAGTGTACTC	CC1

SMC01.T.CCCAATCAGTGTGGCA	CC1
SMC01.T.CCCATACAGGATCGCA	CC1
SMC01.T.CCCATACAGGCGTACA	CC1
SMC01.T.CCCATACAGGGTCGAT	CC1
SMC01.T.CCCATACCACCGTTGG	CC1
SMC01.T.CCCATACTCAGCGACC	CC1
SMC01.T.CCCATACTCCAGAGGA	CC1
SMC01.T.CCCTCCTAGCGTGAAC	CC1
SMC01.T.CCCTCCTGTGCTGTAT	CC1
SMC01.T.CCGGGATAGGTAGCTG	CC1
SMC01.T.CCGGGATCAATAACGA	CC1
SMC01.T.CCGGGATGTAAACACA	CC2
SMC01.T.CCGGGATGTCCTCTTG	CC1
SMC01.T.CCGGGATTCATGCAAC	CC1
SMC01.T.CCGGGATTCCGTAGTA	CC1
SMC01.T.CCGGTAGCAAAGAATC	CC1
SMC01.T.CCGGTAGCACGACTCG	CC2
SMC01.T.CCGGTAGGTAGCGATG	CC1
SMC01.T.CCGGTAGTCATACGGT	CC1
SMC01.T.CCGTACTTCGTGTAGT	CC1
SMC01.T.CCGTGGAAGTGTGGCA	CC1
SMC01.T.CCGTTCAGTCTAGTCA	CC1
SMC01.T.CCGTTCAGTGTGGCTC	CC1
SMC01.T.CCGTTCATCATAAAGG	CC2
SMC01.T.CCTACACCAATAAGCA	CC1
SMC01.T.CCTACACGTGGGTCAA	CC2
SMC01.T.CCTACACGTTCTCATT	CC1
SMC01.T.CCTACCAGTCCAGTGC	CC1
SMC01.T.CCTACCATCAACGGGA	CC1
SMC01.T.CCTAGCTAGGATGTAT	CC1
SMC01.T.CCTAGCTCACCAGGCT	CC1
SMC01.T.CCTAGCTTCGCCAGCA	CC1
SMC01.T.CCTATTAAGAGTAATC	CC1
SMC01.T.CCTATTATCAGCTTAG	CC1
SMC01.T.CCTCAGTAGCCCGAAA	CC1
SMC01.T.CCTCAGTGTTATCGGT	CC2
SMC01.T.CCTCAGTTCACGACTA	CC1
SMC01.T.CCTCTGAGTATTACCG	CC1
SMC01.T.CCTCTGAGTGACGGTA	CC2
SMC01.T.CCTTACGAGGCCGAAT	CC1
SMC01.T.CCTTCGACAGGTGGAT	CC1
SMC01.T.CCTTCGAGTGCAACGA	CC1
SMC01.T.CCTTCGAGTTGGAGGT	CC1
SMC01.T.CCTTTCTAGAAACCTA	CC1
SMC01.T.CCTTTCTTCACCAGGC	CC1
SMC01.T.CCTTTCTTCGGCTACG	CC1
SMC01.T.CGAACATTCGAACTGT	CC1
SMC01.T.CGAACATTCTGATTCT	CC1
SMC01.T.CGAATGTGTGACAAAT	CC1
SMC01.T.CGACCTTGTGTCTGAT	CC1
SMC01.T.CGACCTTTCGGTGTCG	CC2
SMC01.T.CGACCTTTCTTCAACT	CC1
SMC01.T.CGACTTCCACTAAGTC	CC1
SMC01.T.CGACTTCGTGCATCTA	CC1
SMC01.T.CGACTTCTCAATAAGG	CC1
SMC01.T.CGACTTCTCTGTCCGT	CC1
SMC01.T.CGACTTCTCTTCTGGC	CC1
SMC01.T.CGAGAAGAGTGTTAGA	CC1

SMC01.T.CGAGAAGGTCATGCAT	CC1
SMC01.T.CGAGCACGTCGAACAG	CC1
SMC01.T.CGAGCACTCCGATATG	CC2
SMC01.T.CGAGCCAGTCTGGTCG	CC2
SMC01.T.CGAGCCATCTGTCAAG	CC1
SMC01.T.CGATCGGAGCTGAAAT	CC1
SMC01.T.CGATCGGGTCTGCAAT	CC1
SMC01.T.CGATCGGGTGAGGCTA	CC2
SMC01.T.CGATCGGTCCCTTGCA	CC1
SMC01.T.CGATGGCAGACAGAGA	CC1
SMC01.T.CGATGGCCAAGCCTAT	CC1
SMC01.T.CGATGTAAGGACATTA	CC1
SMC01.T.CGATTGACATCTGGTA	CC2
SMC01.T.CGATTGAGTCACTGGC	CC1
SMC01.T.CGATTGATCTCTGTCG	CC1
SMC01.T.CGCCAAGAGCATGGCA	CC1
SMC01.T.CGCCAAGAGTGGTAAT	CC2
SMC01.T.CGCGGTACATGTTGAC	CC1
SMC01.T.CGCGGTATCCCGGATG	CC2
SMC01.T.CGCGTTTCACAGTCGC	CC1
SMC01.T.CGCGTTTGTGCAGACA	CC1
SMC01.T.CGCGTTTGTTCGTCTC	CC1
SMC01.T.CGCGTTTTCGCGTTTC	CC1
SMC01.T.CGCTATCCAATGGACG	CC2
SMC01.T.CGCTATCTCTATGTGG	CC1
SMC01.T.CGCTGGATCCAAATGC	CC1
SMC01.T.CGCTGGATCGTGGTCG	CC2
SMC01.T.CGCTTCAAGCATCATC	CC1
SMC01.T.CGCTTCAAGGGCTTGA	CC1
SMC01.T.CGGACACGTCGACTGC	CC1
SMC01.T.CGGACGTAGAGGTTAT	CC1
SMC01.T.CGGACGTTCCCTTGTG	CC1
SMC01.T.CGGACTGAGTGGAGTC	CC1
SMC01.T.CGGACTGGTACACCGC	CC1
SMC01.T.CGGACTGGTTGATTGC	CC1
SMC01.T.CGGAGCTAGGGCTCTC	CC1
SMC01.T.CGGAGTCGTCGAGATG	CC1
SMC01.T.CGGAGTCTCCGAAGAG	CC1
SMC01.T.CGGCTAGAGCAACGGT	CC1
SMC01.T.CGGCTAGAGCAGACTG	CC1
SMC01.T.CGGCTAGGTCCAGTTA	CC2
SMC01.T.CGGGTCAAGTTAACGA	CC1
SMC01.T.CGGGTCAGTGAACCTT	CC2
SMC01.T.CGGGTCAGTGCGCTTG	CC1
SMC01.T.CGGGTCATCTGACCTC	CC1
SMC01.T.CGGTTAACAATAGCAA	CC1
SMC01.T.CGTAGCGAGACGCTTT	CC1
SMC01.T.CGTAGCGCATCGGGTC	CC1
SMC01.T.CGTAGCGGTGGGTCAA	CC1
SMC01.T.CGTCACTCAAGGTTTC	CC1
SMC01.T.CGTCACTCAGGGTATG	CC1
SMC01.T.CGTCACTCAGGTTTCA	CC1
SMC01.T.CGTCAGGTCTGAGTGT	CC1
SMC01.T.CGTCAGGTCTTGTACT	CC1
SMC01.T.CGTCCATCAATCTACG	CC1
SMC01.T.CGTCCATGTACGACCC	CC1
SMC01.T.CGTCTACGTTGGAGGT	CC1
SMC01.T.CGTGAGCCACTCGACG	CC1

SMC01.T.CGTGAGCGTTCGTGAT	CC1
SMC01.T.CGTGTAAGTTGGACCC	CC1
SMC01.T.CGTGTAATCCTACAGA	CC1
SMC01.T.CGTGTCTAGAAGGTGA	CC1
SMC01.T.CGTGTCTTCTTGCATT	CC2
SMC01.T.CGTTAGAGTGGTCCGT	CC2
SMC01.T.CGTTAGAGTTCGGCAC	CC1
SMC01.T.CGTTAGATCTCTGCTG	CC1
SMC01.T.CGTTCTGCATGTTGAC	CC1
SMC01.T.CGTTGGGAGGCGTACA	CC1
SMC01.T.CTAACTTAGAATGTTG	CC1
SMC01.T.CTAAGACAGGGTTTCT	CC1
SMC01.T.CTAAGACCACTTCTGC	CC1
SMC01.T.CTAAGACTCTGATTCT	CC1
SMC01.T.CTAATGGAGCTACCGC	CC1
SMC01.T.CTAATGGCATACTCTT	CC1
SMC01.T.CTACACCAGAGAGCTC	CC1
SMC01.T.CTACACCGTTCTGAAC	CC1
SMC01.T.CTACACCTCCTTGACC	CC1
SMC01.T.CTACATTCATGACATC	CC1
SMC01.T.CTACATTGTACCATCA	CC2
SMC01.T.CTACCCATCCCGGATG	CC1
SMC01.T.CTACCCATCGTATCAG	CC1
SMC01.T.CTACCCATCTGGCGAC	CC1
SMC01.T.CTACGTCCATTCCTCG	CC1
SMC01.T.CTACGTCGTCGGCATC	CC1
SMC01.T.CTACGTCGTGTATGGG	CC2
SMC01.T.CTAGAGTAGCGCTCCA	CC1
SMC01.T.CTAGAGTAGGATCGCA	CC1
SMC01.T.CTAGAGTCACATCCAA	CC1
SMC01.T.CTAGCCTAGGAGCGTT	CC1
SMC01.T.CTAGCCTCAAATACAG	CC1
SMC01.T.CTAGCCTTCGTTTAGG	CC1
SMC01.T.CTAGTGAGTAAGGGCT	CC2
SMC01.T.CTCACACTCACGAAGG	CC1
SMC01.T.CTCAGAAAGCTCTCGG	CC1
SMC01.T.CTCAGAACATCCCATC	CC1
SMC01.T.CTCAGAAGTTAAAGTG	CC1
SMC01.T.CTCATTACATGCCCGA	CC1
SMC01.T.CTCCTAGAGAACAACT	CC1
SMC01.T.CTCCTAGAGTCTCAAC	CC1
SMC01.T.CTCCTAGCACAGATTC	CC1
SMC01.T.CTCCTAGCAGGTTTCA	CC1
SMC01.T.CTCCTAGCATAAAGGT	CC2
SMC01.T.CTCCTAGTCTCCAGGG	CC1
SMC01.T.CTCGAAAAGAGACTAT	CC1
SMC01.T.CTCGAAACATCGATGT	CC1
SMC01.T.CTCGAAAGTGCACGAA	CC1
SMC01.T.CTCGAAATCTAACTGG	CC1
SMC01.T.CTCGAGGGTAGCACGA	CC2
SMC01.T.CTCGGAGCAGACTCGC	CC1
SMC01.T.CTCGGGACACTAGTAC	CC1
SMC01.T.CTCGTACAGGTGCACA	CC1
SMC01.T.CTCGTACGTCCTGCTT	CC1
SMC01.T.CTCGTACTCTCGTATT	CC1
SMC01.T.CTCGTCAAGCTCTCGG	CC1
SMC01.T.CTCGTCACACCAGCAC	CC1
SMC01.T.CTCGTCAGTCGAAAGC	CC1

SMC01.T.CTCGTCATCAGCGATT	CC1
SMC01.T.CTCTAATGTGCATCTA	CC1
SMC01.T.CTCTAATTCATGCAAC	CC1
SMC01.T.CTCTAATTCTTGCCGT	CC1
SMC01.T.CTCTACGGTGACGGTA	CC1
SMC01.T.CTCTGGTGTCCCTTGT	CC1
SMC01.T.CTCTGGTTCCATGAAC	CC1
SMC01.T.CTGAAACAGCATGGCA	CC1
SMC01.T.CTGAAACCATCTGGTA	CC1
SMC01.T.CTGAAACGTTCAGTAC	CC1
SMC01.T.CTGAAACGTTTGACTG	CC1
SMC01.T.CTGAAGTGTTCAGACT	CC1
SMC01.T.CTGAAGTTCAATCACG	CC1
SMC01.T.CTGATAGCACGTTGGC	CC2
SMC01.T.CTGATAGGTCTGGTCG	CC1
SMC01.T.CTGATCCCACAAGTAA	CC1
SMC01.T.CTGCCTAAGAAGGTGA	CC1
SMC01.T.CTGCCTACATCAGTCA	CC1
SMC01.T.CTGCCTAGTCCGAAGA	CC1
SMC01.T.CTGCCTATCTCACATT	CC1
SMC01.T.CTGCGGACACAGGAGT	CC1
SMC01.T.CTGCGGAGTGAGCGAT	CC1
SMC01.T.CTGCTGTAGGACAGCT	CC2
SMC01.T.CTGCTGTCAAGGACAC	CC1
SMC01.T.CTGCTGTCATGGTTGT	CC1
SMC01.T.CTGGTCTCAGATCTGT	CC1
SMC01.T.CTGGTCTGTTGATTGC	CC2
SMC01.T.CTGGTCTTCCAGAAGG	CC1
SMC01.T.CTGTGCTGTTGCGTTA	CC1
SMC01.T.CTGTTTAAGCGTCTAT	CC1
SMC01.T.CTTAACTAGGAGTCTG	CC1
SMC01.T.CTTAACTAGTAAGTAC	CC2
SMC01.T.CTTAACTCACGGTAAG	CC1
SMC01.T.CTTAACTTCATGTCTT	CC1
SMC01.T.CTTAACTTCCAAGTAC	CC1
SMC01.T.CTTACCGAGAGACTAT	CC1
SMC01.T.CTTACCGTCTTGCATT	CC1
SMC01.T.CTTAGGAGTCGAGATG	CC1
SMC01.T.CTTCTCTAGCCTTGAT	CC1
SMC01.T.CTTCTCTAGCGGATCA	CC1
SMC01.T.CTTCTCTCAAGGTGTG	CC2
SMC01.T.CTTGGCTTCAGCCTAA	CC1
SMC01.T.CTTTGCGCATATACCG	CC1
SMC01.T.CTTTGCGGTGATGTCT	CC1
SMC01.T.CTTTGCGTCTTCGGTC	CC2
SMC01.T.GAAACTCAGACGCACA	CC1
SMC01.T.GAAACTCAGATCACGG	CC1
SMC01.T.GAAACTCCAGAAGCAC	CC1
SMC01.T.GAAACTCTCCTGTAGA	CC1
SMC01.T.GAAACTCTCGCCGTGA	CC1
SMC01.T.GAAATGAGTCCAACTA	CC1
SMC01.T.GAACATCAGATCCCAT	CC1
SMC01.T.GAACATCGTAGGACAC	CC1
SMC01.T.GAACCTAAGACCGGAT	CC1
SMC01.T.GAACCTATCATGTCCC	CC1
SMC01.T.GAACGGACAATAGCAA	CC1
SMC01.T.GAACGGAGTTACAGAA	CC1
SMC01.T.GAAGCAGCAGTATGCT	CC1

SMC01.T.GAAGCAGTCACCAGGC	CC1
SMC01.T.GAAGCAGTCGGAAACG	CC1
SMC01.T.GAATAAGAGAAGGTTT	CC1
SMC01.T.GAATGAAAGCTAACTC	CC2
SMC01.T.GAATGAATCTCTAAGG	CC1
SMC01.T.GACACGCAGACAGACC	CC2
SMC01.T.GACACGCTCAATCACG	CC2
SMC01.T.GACAGAGAGGATGGAA	CC1
SMC01.T.GACAGAGGTGCCTGCA	CC2
SMC01.T.GACCAATGTCAAAGCG	CC1
SMC01.T.GACCAATGTTTCCACC	CC1
SMC01.T.GACCAATTCGGCGGTT	CC1
SMC01.T.GACCTGGGTCTAACGT	CC1
SMC01.T.GACGCGTAGTATCTCG	CC1
SMC01.T.GACGCGTTCGCACTCT	CC1
SMC01.T.GACGGCTAGCGAGAAA	CC1
SMC01.T.GACGGCTAGGACGAAA	CC1
SMC01.T.GACGGCTAGTGCAAGC	CC1
SMC01.T.GACGGCTAGTGCCATT	CC1
SMC01.T.GACGGCTCATACTACG	CC1
SMC01.T.GACGTGCAGAGCTATA	CC1
SMC01.T.GACGTGCGTAGGAGTC	CC1
SMC01.T.GACGTGCGTCTCCACT	CC1
SMC01.T.GACGTTACACGACTCG	CC1
SMC01.T.GACGTTACATTGTGCA	CC1
SMC01.T.GACTAACCAAAGAATC	CC1
SMC01.T.GACTAACGTTCCACGG	CC2
SMC01.T.GACTACAAGGATTCGG	CC1
SMC01.T.GACTACACACTTCTGC	CC1
SMC01.T.GACTACAGTCACCCAG	CC1
SMC01.T.GACTACAGTTAAGGGC	CC1
SMC01.T.GACTACAGTTGCCTCT	CC2
SMC01.T.GACTGCGCAATGGAAT	CC1
SMC01.T.GACTGCGCATTAACCG	CC1
SMC01.T.GACTGCGGTAAGCACG	CC1
SMC01.T.GAGCAGACACAGCCCA	CC1
SMC01.T.GAGCAGATCGTCTGAA	CC1
SMC01.T.GAGCAGATCTTGTCAT	CC2
SMC01.T.GAGTCCGAGCAGGCTA	CC1
SMC01.T.GAGTCCGGTCAGAGGT	CC1
SMC01.T.GATCAGTCATCCGTGG	CC1
SMC01.T.GATCAGTGTATAGGGC	CC1
SMC01.T.GATCAGTTCATGTCTT	CC1
SMC01.T.GATCGATAGCATGGCA	CC1
SMC01.T.GATCGATAGCTAACAA	CC1
SMC01.T.GATCGATCACCAGGCT	CC1
SMC01.T.GATCGATCACCAGGTC	CC1
SMC01.T.GATCGATGTCTCTTAT	CC1
SMC01.T.GATCGATTCTGTGCAA	CC1
SMC01.T.GATCGCGCACCAGGTC	CC1
SMC01.T.GATCGCGCATCTACGA	CC1
SMC01.T.GATCGCGGTAGCCTCG	CC2
SMC01.T.GATCTAGAGCTCTCGG	CC1
SMC01.T.GATCTAGGTGACTCAT	CC2
SMC01.T.GATGAAAAGTGGAGTC	CC2
SMC01.T.GATGAAACAAAGGAAG	CC1
SMC01.T.GATGAAAGTCGCGTGT	CC1
SMC01.T.GATGAGGAGGACCACA	CC1

SMC01.T.GATGAGGAGGCATGTG	CC1
SMC01.T.GATGAGGCACCCAGTG	CC1
SMC01.T.GATGAGGCACGAGGTA	CC1
SMC01.T.GATGAGGGTGTTTGTG	CC1
SMC01.T.GATGCTAAGCGCCTCA	CC1
SMC01.T.GATGCTATCTTCTGGC	CC1
SMC01.T.GATTCAGAGGTGTGGT	CC1
SMC01.T.GATTCAGCAGTTAACC	CC1
SMC01.T.GATTCAGGTTAGGGTG	CC1
SMC01.T.GATTCAGTCTGGAGCC	CC1
SMC01.T.GCAAACTAGACTGTAA	CC1
SMC01.T.GCAAACTAGTCCAGGA	CC2
SMC01.T.GCAAACTCAGCGTCCA	CC2
SMC01.T.GCACATACAACTGCGC	CC1
SMC01.T.GCACATAGTCAACATC	CC1
SMC01.T.GCACATAGTCCTCCAT	CC1
SMC01.T.GCACATAGTTGGAGGT	CC1
SMC01.T.GCACTCTAGAAACCGC	CC2
SMC01.T.GCACTCTAGATATGGT	CC1
SMC01.T.GCACTCTCACGAGAGT	CC1
SMC01.T.GCAGCCAAGTAGGTGC	CC2
SMC01.T.GCAGCCACAATAGAGT	CC1
SMC01.T.GCAGCCAGTAAGGATT	CC1
SMC01.T.GCAGCCATCTGATACG	CC1
SMC01.T.GCAGCCATCTGATTCT	CC1
SMC01.T.GCAGTTACATGCGCAC	CC1
SMC01.T.GCAGTTATCAGAGACG	CC1
SMC01.T.GCAGTTATCCAAACTG	CC1
SMC01.T.GCATACAAGCACCGCT	CC1
SMC01.T.GCATACAAGCGGCTTC	CC1
SMC01.T.GCATACAAGGAGTAGA	CC2
SMC01.T.GCATACAAGTGCCAGA	CC2
SMC01.T.GCATACACAAAGTCAA	CC1
SMC01.T.GCATACACACTGTGTA	CC1
SMC01.T.GCATACACATAGTAAG	CC1
SMC01.T.GCATGATCAATTCCTT	CC1
SMC01.T.GCATGATTCCTCAATT	CC1
SMC01.T.GCATGCGAGCGATAGC	CC2
SMC01.T.GCATGCGAGGCATGTG	CC1
SMC01.T.GCATGCGTCAGGCCCA	CC1
SMC01.T.GCATGTAAGAGGTAGA	CC1
SMC01.T.GCATGTACAGGGAGAG	CC1
SMC01.T.GCATGTACATCGGTTA	CC1
SMC01.T.GCATGTAGTCATCGGC	CC2
SMC01.T.GCCAAATAGTCCGTAT	CC1
SMC01.T.GCCAAATCAGTATGCT	CC1
SMC01.T.GCGACCAAGCATCATC	CC1
SMC01.T.GCGACCACATAGAAAC	CC1
SMC01.T.GCGACCAGTGCGGTAA	CC1
SMC01.T.GCGAGAACAAGAGTCG	CC1
SMC01.T.GCGAGAATCACCAGGC	CC1
SMC01.T.GCGCAACAGACCTAGG	CC2
SMC01.T.GCGCAACAGTTCGCGC	CC2
SMC01.T.GCGCAACGTGGTGTAG	CC1
SMC01.T.GCGCAGTAGCTGCCCA	CC1
SMC01.T.GCGCAGTGTACAGACG	CC1
SMC01.T.GCGCAGTGTCAACATC	CC1
SMC01.T.GCGCCAAGTCCAGTAT	CC2

SMC01.T.GCGCCAAGTGGTCTCG	CC1
SMC01.T.GCGGGTTAGTACCGGA	CC1
SMC01.T.GCTCCTAGTTGCCTCT	CC2
SMC01.T.GCTCTGTGTTCCCGAG	CC1
SMC01.T.GCTCTGTTCCTGTACC	CC1
SMC01.T.GCTGCAGAGATGCGAC	CC1
SMC01.T.GCTGCAGAGTTGTCGT	CC2
SMC01.T.GCTGCAGCAGGGTTAG	CC1
SMC01.T.GCTGCTTGTTCCACTC	CC1
SMC01.T.GCTGGGTAGTCTCGGC	CC1
SMC01.T.GCTGGGTTCAAGATCC	CC1
SMC01.T.GCTGGGTTCTTAGCCC	CC1
SMC01.T.GCTTGAAGTGTGAATA	CC1
SMC01.T.GGAAAGCCAAATCCGT	CC1
SMC01.T.GGAAAGCTCCAGTAGT	CC1
SMC01.T.GGAACTTGTAAATACG	CC1
SMC01.T.GGAATAACAGTATGCT	CC1
SMC01.T.GGAATAATCCCTAACC	CC1
SMC01.T.GGACAAGAGCTGGAAC	CC1
SMC01.T.GGACAAGCATTCACTT	CC1
SMC01.T.GGACAAGGTGTGAATA	CC2
SMC01.T.GGACAAGTCGGTGTTA	CC1
SMC01.T.GGACAGACATAGACTC	CC1
SMC01.T.GGACAGAGTCTCAACA	CC1
SMC01.T.GGACATTAGCAGCGTA	CC1
SMC01.T.GGACATTTCATCGATG	CC2
SMC01.T.GGACATTTCGTGGTCG	CC1
SMC01.T.GGAGCAACAAGGTGTG	CC1
SMC01.T.GGAGCAACACAGCCCA	CC1
SMC01.T.GGAGCAACATGGATGG	CC1
SMC01.T.GGAGCAATCTGCAGTA	CC1
SMC01.T.GGATGTTCAATAAGCA	CC2
SMC01.T.GGATTACAGTAGGTGC	CC1
SMC01.T.GGATTACTCAACCATG	CC1
SMC01.T.GGATTACTCTGCGACG	CC1
SMC01.T.GGATTACTCTTGCCGT	CC1
SMC01.T.GGCAATTGTAGAGTGC	CC1
SMC01.T.GGCCGATCAAAGAATC	CC1
SMC01.T.GGCCGATCATGTCTCC	CC1
SMC01.T.GGCGACTCAGATCCAT	CC1
SMC01.T.GGCGACTTCACGCGGT	CC1
SMC01.T.GGCGTGTCAAGCGATG	CC1
SMC01.T.GGCGTGTGTCCAGTGC	CC1
SMC01.T.GGCGTGTTCAGCGATT	CC1
SMC01.T.GGCGTGTTCCGTAGTA	CC1
SMC01.T.GGCGTGTTCGTTTAGG	CC1
SMC01.T.GGCGTGTTCTGCAAGT	CC1
SMC01.T.GGCTCGATCAGCTGGC	CC1
SMC01.T.GGGAATGCACTCAGGC	CC1
SMC01.T.GGGAATGGTCATATGC	CC1
SMC01.T.GGGACCTAGAGTGAGA	CC1
SMC01.T.GGGACCTCACGAGAGT	CC1
SMC01.T.GGGAGATCATACCATG	CC1
SMC01.T.GGGAGATGTCAGCTAT	CC1
SMC01.T.GGGAGATTCCACGACG	CC1
SMC01.T.GGGATGACAAACTGTC	CC2
SMC01.T.GGGATGAGTTCCGTCT	CC1
SMC01.T.GGGATGATCTGAAAGA	CC1

SMC01.T.GGGCATCCACGGACAA	CC2
SMC01.T.GGGCATCCAGTCCTTC	CC1
SMC01.T.GGGCATCTCTAACTGG	CC2
SMC01.T.GGGTCTGAGGTCGGAT	CC2
SMC01.T.GGGTCTGCAAGGACAC	CC1
SMC01.T.GGGTCTGCATGCCTAA	CC1
SMC01.T.GGGTCTGGTAGATTAG	CC1
SMC01.T.GGGTTGCAGTACGCGA	CC1
SMC01.T.GGGTTGCAGTCAATAG	CC1
SMC01.T.GGGTTGCAGTTCGATC	CC1
SMC01.T.GGGTTGCGTGCACGAA	CC1
SMC01.T.GGTATTGCACGGATAG	CC1
SMC01.T.GGTATTGCATTTCACT	CC1
SMC01.T.GGTATTGGTGTGGCTC	CC1
SMC01.T.GGTGAAGTCATTATCC	CC1
SMC01.T.GGTGCGTCAGCATACT	CC1
SMC01.T.GGTGCGTCATAGGATA	CC1
SMC01.T.GGTGCGTCATCCAACA	CC1
SMC01.T.GGTGCGTGTCACCCAG	CC1
SMC01.T.GGTGCGTGTTACGTCA	CC1
SMC01.T.GGTGCGTTCAGTTGAC	CC1
SMC01.T.GGTGTTAAGTCTTGCA	CC1
SMC01.T.GGTGTTACAAGACGTG	CC1
SMC01.T.GTAACGTGTTGATTCG	CC1
SMC01.T.GTAACTGCATGACATC	CC1
SMC01.T.GTAACTGTCGAGAGCA	CC1
SMC01.T.GTACGTAAGAATCTCC	CC1
SMC01.T.GTACTCCCAGCGAACA	CC1
SMC01.T.GTACTCCCAGGCTCAC	CC1
SMC01.T.GTACTTTAGCCAGGAT	CC1
SMC01.T.GTACTTTAGTCGATAA	CC2
SMC01.T.GTACTTTTCGCCAAAT	CC2
SMC01.T.GTAGGCCAGTGACTCT	CC1
SMC01.T.GTAGGCCCATTGGCGC	CC1
SMC01.T.GTAGTCAAGAATTCCC	CC1
SMC01.T.GTATCTTAGCCAACAG	CC2
SMC01.T.GTATCTTTCTTTCCTC	CC1
SMC01.T.GTATTCTCATTCACTT	CC1
SMC01.T.GTCAAGTCAACTGCGC	CC1
SMC01.T.GTCACAAAGACGCTTT	CC1
SMC01.T.GTCACAATCAAGGTAA	CC1
SMC01.T.GTCACAATCAGTTCGA	CC1
SMC01.T.GTCATTTAGTTGTAGA	CC2
SMC01.T.GTCATTTCACCTCGGA	CC2
SMC01.T.GTCATTTCATCCTAGA	CC1
SMC01.T.GTCCTCACACTGAAGG	CC1
SMC01.T.GTCCTCAGTCTGATTG	CC2
SMC01.T.GTCCTCATCAAGCCTA	CC1
SMC01.T.GTCCTCATCCAGAGGA	CC1
SMC01.T.GTCTCGTAGATGAGAG	CC1
SMC01.T.GTCTCGTGTGTTCGAT	CC1
SMC01.T.GTCTCGTGTTTCCACC	CC1
SMC01.T.GTCTTCGCAAAGCAAT	CC2
SMC01.T.GTCTTCGCACGAAATA	CC2
SMC01.T.GTCTTCGCATCACGAT	CC2
SMC01.T.GTCTTCGGTCCTCTTG	CC1
SMC01.T.GTCTTCGGTCGTTGTA	CC1
SMC01.T.GTGAAGGCAATGTAAG	CC1

SMC01.T.GTGAAGGGTCTTCTCG	CC1
SMC01.T.GTGCAGCAGAGTCTGG	CC1
SMC01.T.GTGCAGCCAAGTCTGT	CC1
SMC01.T.GTGCAGCTCCGAGCCA	CC1
SMC01.T.GTGCATAAGCTAACAA	CC1
SMC01.T.GTGCATACACACCGCA	CC1
SMC01.T.GTGCATATCTCCTATA	CC1
SMC01.T.GTGCGGTAGAAGGTGA	CC1
SMC01.T.GTGCGGTAGCAAATCA	CC2
SMC01.T.GTGCGGTCATCTATGG	CC1
SMC01.T.GTGCGGTGTCCGTGAC	CC1
SMC01.T.GTGCGGTTCATGTAGC	CC1
SMC01.T.GTGCTTCTCTGCCCTA	CC1
SMC01.T.GTGGGTCAGCAAATCA	CC1
SMC01.T.GTGGGTCGTCCGTCAG	CC1
SMC01.T.GTGGGTCTCAGCTCGG	CC1
SMC01.T.GTGTGCGCACGACTCG	CC1
SMC01.T.GTGTGCGGTGTCCTCT	CC1
SMC01.T.GTGTTAGGTTACGACT	CC1
SMC01.T.GTGTTAGGTTCATGGT	CC1
SMC01.T.GTGTTAGTCCGAGCCA	CC1
SMC01.T.GTTAAGCAGGCTACGA	CC1
SMC01.T.GTTAAGCCATCCGTGG	CC1
SMC01.T.GTTAAGCGTCCATCCT	CC2
SMC01.T.GTTAAGCTCGATCCCT	CC1
SMC01.T.GTTACAGGTAATTGGA	CC1
SMC01.T.GTTACAGGTGTCAATC	CC1
SMC01.T.GTTACAGTCCCTGACT	CC1
SMC01.T.GTTCATTGTCTAGCGC	CC1
SMC01.T.GTTCATTTCCTGCTTG	CC1
SMC01.T.GTTCATTTCTTAGAGC	CC1
SMC01.T.GTTCTCGGTGTGCGTC	CC1
SMC01.T.GTTCTCGTCAGTTGAC	CC1
SMC01.T.GTTTCTAAGGCAGGTT	CC1
SMC01.T.GTTTCTAAGGCTCAGA	CC1
SMC01.T.TAAACCGTCTACTATC	CC1
SMC01.T.TAAGAGACAATCGAAA	CC1
SMC01.T.TAAGAGACATCGGACC	CC1
SMC01.T.TAAGCGTAGTACACCT	CC1
SMC01.T.TAAGCGTCATCGATTG	CC1
SMC01.T.TAAGCGTGTATAAACG	CC1
SMC01.T.TAAGCGTGTCTAGAGG	CC1
SMC01.T.TAAGCGTGTGAGTGAC	CC1
SMC01.T.TACACGAAGAGACTTA	CC1
SMC01.T.TACACGAAGCCCAACC	CC1
SMC01.T.TACACGAGTCGCGGTT	CC2
SMC01.T.TACACGATCCACGTTC	CC2
SMC01.T.TACAGTGCACAAGTAA	CC1
SMC01.T.TACAGTGTCTGGCGAC	CC1
SMC01.T.TACCTATAGTGCTGCC	CC1
SMC01.T.TACCTATCACGGTGTC	CC1
SMC01.T.TACCTATGTCCGAGTC	CC1
SMC01.T.TACCTTACACCAACCG	CC1
SMC01.T.TACCTTATCGCCAGCA	CC1
SMC01.T.TACGGATAGTGGTAAT	CC1
SMC01.T.TACGGGCAGAGGACGG	CC1
SMC01.T.TACGGGCGTACAGCAG	CC2
SMC01.T.TACGGGCGTCAAAGAT	CC2

SMC01.T.TACGGGCGTCCCTTGT	CC1
SMC01.T.TACGGGCTCGGCGCTA	CC2
SMC01.T.TACGGTACATCGATTG	CC1
SMC01.T.TACGGTATCTCTAAGG	CC2
SMC01.T.TACTCATCAGGTCTCG	CC1
SMC01.T.TACTCGCTCACTTCAT	CC2
SMC01.T.TACTTACAGCGTGAAC	CC1
SMC01.T.TACTTACAGCTTCGCG	CC1
SMC01.T.TACTTACGTGTTAAGA	CC1
SMC01.T.TACTTGTAGGCATGGT	CC1
SMC01.T.TACTTGTTCATTGCGA	CC1
SMC01.T.TACTTGTTCCCTAATT	CC1
SMC01.T.TAGACCACATGTCTCC	CC2
SMC01.T.TAGACCATCGCCGTGA	CC1
SMC01.T.TAGGCATAGGTCATCT	CC1
SMC01.T.TAGGCATGTTGGTAAA	CC1
SMC01.T.TAGGCATTCCGTCAAA	CC1
SMC01.T.TAGTGGTCAAGCCGCT	CC1
SMC01.T.TAGTTGGAGGCCCTCA	CC1
SMC01.T.TAGTTGGCAAACGCGA	CC1
SMC01.T.TAGTTGGTCCCAGGTG	CC1
SMC01.T.TATCTCAAGGTGGGTT	CC1
SMC01.T.TATGCCCCATGGGACA	CC2
SMC01.T.TATTACCAGAAGCCCA	CC2
SMC01.T.TATTACCAGTGAAGTT	CC1
SMC01.T.TATTACCCACAAGTAA	CC1
SMC01.T.TATTACCCACTAAGTC	CC2
SMC01.T.TATTACCGTCCAAGTT	CC1
SMC01.T.TCAACGACAGGCTCAC	CC1
SMC01.T.TCAACGAGTTATGCGT	CC1
SMC01.T.TCAATCTCACACGCTG	CC1
SMC01.T.TCAATCTCATGGTTGT	CC1
SMC01.T.TCAATCTGTCATCGGC	CC2
SMC01.T.TCACAAGTCAGCAACT	CC1
SMC01.T.TCACGAAAGAGTTGGC	CC1
SMC01.T.TCACGAAAGTATTGGA	CC1
SMC01.T.TCAGATGAGAGTACCG	CC1
SMC01.T.TCAGATGCACAGGCCT	CC1
SMC01.T.TCAGCAACATGCCTAA	CC1
SMC01.T.TCAGGATTCTCTTGAT	CC1
SMC01.T.TCAGGATTCTGCAGTA	CC1
SMC01.T.TCAGGTACATGCATGT	CC1
SMC01.T.TCAGGTAGTTATCGGT	CC1
SMC01.T.TCATTACAGCTCCTTC	CC1
SMC01.T.TCATTACTCCGAGCCA	CC1
SMC01.T.TCATTTGAGGGTGTGT	CC1
SMC01.T.TCATTTGTCGCACTCT	CC1
SMC01.T.TCCACACGTTAAAGAC	CC1
SMC01.T.TCCCGATCATGAGCGA	CC1
SMC01.T.TCCCGATTCGCCAAAT	CC1
SMC01.T.TCCCGATTCTCGTATT	CC1
SMC01.T.TCGAGGCAGAAACCAT	CC1
SMC01.T.TCGAGGCCAGTATAAG	CC2
SMC01.T.TCGAGGCTCACAAACC	CC1
SMC01.T.TCGCGAGCAAAGTCAA	CC1
SMC01.T.TCGCGAGCAATCTGCA	CC1
SMC01.T.TCGCGAGCAATGGACG	CC2
SMC01.T.TCGCGAGCATAGTAAG	CC1

SMC01.T.TCGCGAGGTCAACTGT	CC2
SMC01.T.TCGCGAGGTCGACTAT	CC1
SMC01.T.TCGCGTTAGTTGTAGA	CC1
SMC01.T.TCGCGTTCACGAAGCA	CC1
SMC01.T.TCGGGACGTAGTAGTA	CC2
SMC01.T.TCGGGACGTTATGTGC	CC1
SMC01.T.TCGGTAAAGCTTTGGT	CC1
SMC01.T.TCGGTAACAAAGAATC	CC1
SMC01.T.TCGGTAACAAAGCAAT	CC2
SMC01.T.TCGTACCAGCACCGTC	CC1
SMC01.T.TCGTACCCACCAGGCT	CC1
SMC01.T.TCGTACCTCAGTGTTG	CC1
SMC01.T.TCGTAGAAGCTGAAAT	CC2
SMC01.T.TCGTAGAAGTTCCACA	CC1
SMC01.T.TCGTAGACATTGGTAC	CC1
SMC01.T.TCGTAGATCCGCGCAA	CC1
SMC01.T.TCTATTGAGTTTGCGT	CC1
SMC01.T.TCTATTGCAAGTAATG	CC1
SMC01.T.TCTATTGGTAGGAGTC	CC1
SMC01.T.TCTATTGGTCCGTGAC	CC1
SMC01.T.TCTCATAAGCGTTCCG	CC1
SMC01.T.TCTCATATCTCGCTTG	CC1
SMC01.T.TCTCTAAGTTAGATGA	CC1
SMC01.T.TCTCTAATCAGGTTCA	CC2
SMC01.T.TCTCTAATCCGTACAA	CC2
SMC01.T.TCTGAGAAGGAGTTGC	CC1
SMC01.T.TCTGGAAAGACAGACC	CC1
SMC01.T.TCTGGAACACCGCTAG	CC1
SMC01.T.TCTTCGGGTGGTCCGT	CC1
SMC01.T.TCTTCGGTCAGCTTAG	CC2
SMC01.T.TCTTCGGTCTCCAGGG	CC2
SMC01.T.TCTTCGGTCTGCAAGT	CC1
SMC01.T.TCTTTCCCATTGGCGC	CC1
SMC01.T.TCTTTCCGTTTGCATG	CC1
SMC01.T.TCTTTCCTCCACGACG	CC2
SMC01.T.TGAAAGACAAGTAATG	CC1
SMC01.T.TGAAAGACACCCATTC	CC1
SMC01.T.TGACAACGTAGGGTAC	CC1
SMC01.T.TGACAACGTGGTAACG	CC1
SMC01.T.TGACGGCGTTCGTGAT	CC1
SMC01.T.TGACTAGAGTGTCCCG	CC1
SMC01.T.TGACTAGCAAGCCTAT	CC1
SMC01.T.TGACTAGTCTCCTATA	CC2
SMC01.T.TGACTTTAGACAATAC	CC1
SMC01.T.TGACTTTAGTGAAGAG	CC1
SMC01.T.TGACTTTCATCCGGGT	CC2
SMC01.T.TGAGAGGCAGATAATG	CC1
SMC01.T.TGAGAGGCAGATCTGT	CC1
SMC01.T.TGAGAGGTCTTCAACT	CC1
SMC01.T.TGAGCATTCCGTAGTA	CC1
SMC01.T.TGAGCATTCGGCGCAT	CC1
SMC01.T.TGAGCCGAGACACGAC	CC1
SMC01.T.TGAGCCGAGCTCAACT	CC1
SMC01.T.TGAGCCGCATTTGCCC	CC1
SMC01.T.TGAGGGAAGGGATACC	CC1
SMC01.T.TGAGGGACATTCCTGC	CC2
SMC01.T.TGAGGGATCTTGCCGT	CC1
SMC01.T.TGAGGGATCTTTAGGG	CC1

SMC01.T.TGATTTCAGCAGACTG	CC2
SMC01.T.TGATTTCGTAGCACGA	CC1
SMC01.T.TGCACCTAGTCTCCTC	CC1
SMC01.T.TGCACCTCAGCTTCGG	CC1
SMC01.T.TGCACCTTCGCAAGCC	CC1
SMC01.T.TGCCAAAAGCGTCAAG	CC1
SMC01.T.TGCCCATTCGGTTCGG	CC1
SMC01.T.TGCCCTAAGTTACCCA	CC1
SMC01.T.TGCCCTAGTATAGGGC	CC1
SMC01.T.TGCCCTATCGCGCCAA	CC2
SMC01.T.TGCGCAGCAGTATGCT	CC1
SMC01.T.TGCGCAGTCAGTTAGC	CC1
SMC01.T.TGCGGGTAGACCACGA	CC1
SMC01.T.TGCGGGTAGGGTTTCT	CC1
SMC01.T.TGCGGGTCAGCTGCTG	CC1
SMC01.T.TGCGGGTCATATACGC	CC1
SMC01.T.TGCGTGGAGATTACCC	CC2
SMC01.T.TGCGTGGTCCATGCTC	CC1
SMC01.T.TGCTACCAGGCTACGA	CC1
SMC01.T.TGCTACCGTAAACCTC	CC1
SMC01.T.TGCTGCTAGAGAGCTC	CC1
SMC01.T.TGCTGCTCATGCCCGA	CC2
SMC01.T.TGGACGCAGGATGCGT	CC2
SMC01.T.TGGACGCCACAGAGGT	CC1
SMC01.T.TGGACGCGTATAGGGC	CC2
SMC01.T.TGGACGCTCTGGTATG	CC1
SMC01.T.TGGCCAGAGTTGAGAT	CC1
SMC01.T.TGGCGCAAGGAACTGC	CC1
SMC01.T.TGGCGCAAGTCGTTTG	CC1
SMC01.T.TGGCGCATCTGTGCAA	CC1
SMC01.T.TGGCTGGAGCTAGGCA	CC1
SMC01.T.TGGCTGGGTAATTGGA	CC1
SMC01.T.TGGCTGGGTGTAACGG	CC1
SMC01.T.TGGGAAGAGGCGTACA	CC1
SMC01.T.TGGGAAGGTTCAGGCC	CC1
SMC01.T.TGGGAAGTCGCCAGCA	CC1
SMC01.T.TGGGAAGTCTGCTGCT	CC1
SMC01.T.TGGGCGTCAGCTATTG	CC1
SMC01.T.TGGGCGTTCTCTTGAT	CC1
SMC01.T.TGGTTAGAGGCTAGGT	CC1
SMC01.T.TGGTTCCCATGACATC	CC1
SMC01.T.TGTATTCCACATAACC	CC1
SMC01.T.TGTATTCCATCGACGC	CC1
SMC01.T.TGTCCCAAGAGTACAT	CC1
SMC01.T.TGTCCCACAAGGTTTC	CC1
SMC01.T.TGTGGTACAGGACGTA	CC2
SMC01.T.TGTGGTAGTATGGTTC	CC1
SMC01.T.TGTGTTTAGTGCTGCC	CC1
SMC01.T.TGTGTTTCAGGCTCAC	CC1
SMC01.T.TGTGTTTTCACCGGGT	CC1
SMC01.T.TTAACTCAGACTCGGA	CC1
SMC01.T.TTAACTCCACGGCCAT	CC1
SMC01.T.TTAGGACCAACGCACC	CC1
SMC01.T.TTAGGACTCAGCATGT	CC1
SMC01.T.TTAGGACTCCTCCTAG	CC1
SMC01.T.TTAGGACTCTAACTCT	CC1
SMC01.T.TTAGGCAAGGAACTGC	CC1
SMC01.T.TTAGGCATCCGCAGTG	CC1

SMC01.T.TTAGTTCCACGGCCAT	CC1
SMC01.T.TTAGTTCGTGTCCTCT	CC1
SMC01.T.TTAGTTCTCGGCATCG	CC1
SMC01.T.TTATGCTTCAGCAACT	CC1
SMC01.T.TTATGCTTCGGCGCAT	CC2
SMC01.T.TTCGAAGCACAGGAGT	CC1
SMC01.T.TTCGAAGTCTAACCGA	CC1
SMC01.T.TTCGGTCAGAAACCAT	CC1
SMC01.T.TTCGGTCGTACCATCA	CC2
SMC01.T.TTCGGTCTCTTACCGC	CC1
SMC01.T.TTCTACAAGGCTACGA	CC1
SMC01.T.TTCTACACATGCCTTC	CC1
SMC01.T.TTCTACATCGGATGGA	CC2
SMC01.T.TTCTACATCTTGACGA	CC2
SMC01.T.TTCTCAAGTCTACCTC	CC1
SMC01.T.TTCTCAATCAACCAAC	CC1
SMC01.T.TTCTCCTTCGGACAAG	CC1
SMC01.T.TTCTTAGAGCCTTGAT	CC1
SMC01.T.TTGAACGAGTGTGGCA	CC1
SMC01.T.TTGAACGGTCCGAAGA	CC2
SMC01.T.TTGACTTAGGGTCTCC	CC2
SMC01.T.TTGACTTGTCTCATCC	CC2
SMC01.T.TTGCCGTAGATGCGAC	CC2
SMC01.T.TTGCCGTGTCGTGGCT	CC1
SMC01.T.TTGCGTCAGTGGTAGC	CC1
SMC01.T.TTGCGTCCAACAACCT	CC1
SMC01.T.TTGCGTCGTACGCACC	CC1
SMC01.T.TTGCGTCGTTGCGCAC	CC1
SMC01.T.TTGCGTCTCACTTATC	CC1
SMC01.T.TTGCGTCTCCCAAGAT	CC2
SMC01.T.TTGCGTCTCCTCAACC	CC2
SMC01.T.TTGGAACGTGGGTATG	CC1
SMC01.T.TTGGAACGTTATTCTC	CC1
SMC01.T.TTGGCAAAGCGTGTCC	CC1
SMC01.T.TTGGCAAAGGTAGCTG	CC2
SMC01.T.TTGTAGGAGGTGACCA	CC1
SMC01.T.TTGTAGGCAATGACCT	CC1
SMC01.T.TTTACTGCAAGTTCTG	CC1
SMC01.T.TTTACTGCAGTTCCCT	CC1
SMC01.T.TTTCCTCAGAAACGAG	CC1
SMC01.T.TTTCCTCGTAGCGTCC	CC1
SMC01.T.TTTCCTCGTAGTGAAT	CC1
SMC01.T.TTTCCTCGTCGTCTTC	CC1
SMC01.T.TTTGCGCCATGCCCGA	CC1
SMC01.T.TTTGCGCTCCTAGTGA	CC1
SMC01.T.TTTGCGCTCGTTTATC	CC1
SMC01.T.TTTGGTTAGGCCGAAT	CC1
SMC01.T.TTTGGTTGTCTAAAGA	CC2
SMC01.T.TTTGTCAAGACATAAC	CC1
SMC01.T.TTTGTCACAGGGTATG	CC1
SMC01.T.TTTGTCAGTCCGACGT	CC1
SMC02.T.AAACGGGTCTGCCAGG	CC1
SMC02.T.AAAGCAAAGCTACCTA	CC1
SMC02.T.AACACGTGTGGAAAGA	CC1
SMC02.T.AACACGTTCTTAGCCC	CC1
SMC02.T.AACTCCCAGTCTCGGC	CC1
SMC02.T.AACTCTTAGACTAAGT	CC1
SMC02.T.AACTTTCGTTCGCTAA	CC1

SMC02.T.AAGACCTGTGACCAAG	CC2
SMC02.T.AAGGAGCAGATATGGT	CC1
SMC02.T.AAGGCAGAGAGTAATC	CC1
SMC02.T.AAGGCAGGTGTTAAGA	CC1
SMC02.T.AAGGCAGGTTGTACAC	CC1
SMC02.T.AATCCAGCAGGATCGA	CC1
SMC02.T.AATCGGTCACATCCGG	CC1
SMC02.T.ACAGCCGGTCCTCCAT	CC1
SMC02.T.ACAGCTACAAACCTAC	CC1
SMC02.T.ACAGCTACATTGTGCA	CC1
SMC02.T.ACCAGTAAGTGGTCCC	CC1
SMC02.T.ACCGTAATCGGTTCGG	CC1
SMC02.T.ACCTTTAAGATATGGT	CC1
SMC02.T.ACGCAGCCAGGACCCT	CC1
SMC02.T.ACGGAGAAGGCCATAG	CC1
SMC02.T.ACGGAGATCATGCTCC	CC1
SMC02.T.ACGGCCAAGCTGTTCA	CC2
SMC02.T.ACGGGCTTCCGCATAA	CC1
SMC02.T.ACGTCAAAGAGCCTAG	CC1
SMC02.T.ACTATCTCAAATTGCC	CC1
SMC02.T.ACTGAACTCAATAAGG	CC1
SMC02.T.ACTGCTCCAATAGAGT	CC1
SMC02.T.ACTGCTCGTCACAAGG	CC1
SMC02.T.ACTTACTCAGTATAAG	CC1
SMC02.T.AGAATAGGTTTGTTTC	CC1
SMC02.T.AGACGTTTCGACCAGC	CC1
SMC02.T.AGAGCGAAGTAGTGCG	CC1
SMC02.T.AGAGCGACAAGCCTAT	CC1
SMC02.T.AGATTGCTCACCCTCA	CC1
SMC02.T.AGCAGCCAGCCAACAG	CC1
SMC02.T.AGCATACCACATCCGG	CC1
SMC02.T.AGCATACCATATGAGA	CC1
SMC02.T.AGCATACGTATAATGG	CC1
SMC02.T.AGCATACTCACGAAGG	CC1
SMC02.T.AGCGTATGTGTTCGAT	CC1
SMC02.T.AGCTCTCTCCAAAGTC	CC1
SMC02.T.AGGCCGTAGCAGCCTC	CC1
SMC02.T.AGGGAGTTCGAGCCCA	CC1
SMC02.T.AGGGATGAGCATGGCA	CC1
SMC02.T.AGGTCATCAAAGGAAG	CC1
SMC02.T.AGGTCATTCGTCTGCT	CC1
SMC02.T.AGTCTTTGTCACACGC	CC1
SMC02.T.AGTGAGGAGAGACTTA	CC1
SMC02.T.AGTGAGGAGATCTGAA	CC1
SMC02.T.AGTTGGTGTGAGCGAT	CC1
SMC02.T.ATAACGCAGTACGCCC	CC1
SMC02.T.ATAACGCCAGTTAACC	CC1
SMC02.T.ATCACGACACCAGGTC	CC1
SMC02.T.ATCATCTTCCAAACTG	CC1
SMC02.T.ATCCACCAGAGGGCTT	CC1
SMC02.T.ATCCACCGTTGAGGTG	CC1
SMC02.T.ATCTGCCCAGCGAACA	CC1
SMC02.T.ATGAGGGAGGCCGAAT	CC1
SMC02.T.ATTATCCTCTCTAAGG	CC1
SMC02.T.ATTGGACTCGCTTAGA	CC1
SMC02.T.ATTGGTGGTCGAGATG	CC1
SMC02.T.ATTTCTGAGTTACGGG	CC1
SMC02.T.CAACCTCAGGGCATGT	CC2

SMC02.T.CAACTAGCAAAGCGGT	CC1
SMC02.T.CAACTAGCATTCTTAC	CC1
SMC02.T.CAAGGCCGTTCCACGG	CC1
SMC02.T.CAAGTTGCATCCCATC	CC1
SMC02.T.CACACAATCCTTTCGG	CC1
SMC02.T.CACACAATCTGCTGCT	CC1
SMC02.T.CACACCTGTGTTGGGA	CC1
SMC02.T.CACAGTAGTCTGCGGT	CC1
SMC02.T.CACATAGTCCTCAATT	CC1
SMC02.T.CACATTTCAACAACCT	CC1
SMC02.T.CACATTTGTTTGGGCC	CC1
SMC02.T.CACCACTAGCCAGTTT	CC1
SMC02.T.CACCTTGTCCAGAGGA	CC1
SMC02.T.CAGAATCTCTTTAGTC	CC1
SMC02.T.CAGAGAGAGCCCTAAT	CC1
SMC02.T.CAGAGAGTCATAGCAC	CC1
SMC02.T.CAGATCAAGGGTCTCC	CC1
SMC02.T.CAGATCATCAACACCA	CC1
SMC02.T.CAGCATAGTTGCTCCT	CC1
SMC02.T.CAGCCGAGTGGTCCGT	CC1
SMC02.T.CAGCCGATCCGCATAA	CC2
SMC02.T.CAGCTAAAGTACACCT	CC1
SMC02.T.CAGCTGGCAAGTCTAC	CC1
SMC02.T.CAGGTGCAGCTAGCCC	CC1
SMC02.T.CAGTAACGTCGTGGCT	CC1
SMC02.T.CAGTCCTAGATGGCGT	CC1
SMC02.T.CAGTCCTTCCAATGGT	CC1
SMC02.T.CATCAAGTCGGATGTT	CC1
SMC02.T.CATCAGAGTGTGGCTC	CC1
SMC02.T.CATCCACGTCTGATCA	CC1
SMC02.T.CATCCACGTTACGGAG	CC1
SMC02.T.CATCGAAAGACTAGGC	CC1
SMC02.T.CATGCCTGTCTAAAGA	CC1
SMC02.T.CATTATCGTATGAAAC	CC1
SMC02.T.CATTCGCAGCCGATTT	CC1
SMC02.T.CATTCGCGTTGCCTCT	CC1
SMC02.T.CCACCTAGTGGACGAT	CC2
SMC02.T.CCACTACGTCCGAATT	CC1
SMC02.T.CCATGTCTCTACTTAC	CC1
SMC02.T.CCCAATCTCGAATCCA	CC1
SMC02.T.CCCAGTTGTCAAAGCG	CC1
SMC02.T.CCCATACCAGGTCGTC	CC1
SMC02.T.CCCTCCTTCCAGGGCT	CC1
SMC02.T.CCGTTCACAACTGCGC	CC1
SMC02.T.CCTACACCAATGCCAT	CC1
SMC02.T.CCTATTAGTCAGGACA	CC1
SMC02.T.CCTCAGTCAGACGCCT	CC1
SMC02.T.CCTCTGACATTCCTGC	CC1
SMC02.T.CCTCTGAGTAGGACAC	CC1
SMC02.T.CCTCTGATCAACCATG	CC1
SMC02.T.CCTTCCCGTGCACTTA	CC1
SMC02.T.CCTTCGACACGGTTTA	CC1
SMC02.T.CCTTCGACATATACCG	CC1
SMC02.T.CGAATGTGTAAGAGGA	CC1
SMC02.T.CGACCTTAGATGTAAC	CC1
SMC02.T.CGACTTCCAACCGCCA	CC1
SMC02.T.CGACTTCCAATCGGTT	CC1
SMC02.T.CGAGAAGAGACTGTAA	CC1

SMC02.T.CGATCGGGTTATCGGT	CC1
SMC02.T.CGATGGCCACAGACTT	CC1
SMC02.T.CGATGGCCAGCTGCTG	CC1
SMC02.T.CGATGGCTCCGCGGTA	CC1
SMC02.T.CGCGGTACAAACGCGA	CC1
SMC02.T.CGCGGTATCCGGCACA	CC1
SMC02.T.CGCGTTTTCTCGTTTA	CC1
SMC02.T.CGCTATCGTTTCCACC	CC1
SMC02.T.CGGACACGTCGAATCT	CC1
SMC02.T.CGGACGTAGGATGCGT	CC1
SMC02.T.CGGAGCTAGGTGGGTT	CC1
SMC02.T.CGGAGCTGTCCGTGAC	CC1
SMC02.T.CGGAGTCTCTGCGTAA	CC1
SMC02.T.CGGGTCAAGGCATGGT	CC1
SMC02.T.CGTAGCGCACATTAGC	CC1
SMC02.T.CGTAGCGGTCTTTCAT	CC1
SMC02.T.CGTAGGCTCAACTCTT	CC1
SMC02.T.CGTCACTCAAGTCATC	CC1
SMC02.T.CGTCCATGTTCGAATC	CC1
SMC02.T.CGTGTAAGTAAACGCG	CC1
SMC02.T.CGTGTAAGTACTTCTT	CC1
SMC02.T.CGTGTCTGTGAAGGCT	CC1
SMC02.T.CGTGTCTGTGCCTGTG	CC1
SMC02.T.CGTGTCTGTGTTAAGA	CC1
SMC02.T.CGTTCTGTCAGTGTTG	CC1
SMC02.T.CTAACTTCATGCATGT	CC1
SMC02.T.CTAACTTGTGACTCAT	CC1
SMC02.T.CTAACTTTCCTGCCAT	CC1
SMC02.T.CTAAGACGTACTCTCC	CC1
SMC02.T.CTAAGACTCACGCATA	CC1
SMC02.T.CTACCCAAGGTGATTA	CC1
SMC02.T.CTACCCATCTTCTGGC	CC1
SMC02.T.CTACGTCAGCCGTCGT	CC1
SMC02.T.CTCAGAAAGAACTCGG	CC1
SMC02.T.CTCAGAAGTTCCTCCA	CC1
SMC02.T.CTCAGAATCAGTTAGC	CC1
SMC02.T.CTCATTACAAATCCGT	CC1
SMC02.T.CTCCTAGGTACCGCTG	CC1
SMC02.T.CTCGGGAAGCCATCGC	CC1
SMC02.T.CTCGGGATCGTGGTCG	CC1
SMC02.T.CTCGTCATCCAGGGCT	CC1
SMC02.T.CTCTACGAGGCTACGA	CC1
SMC02.T.CTGATCCGTCGAAAGC	CC1
SMC02.T.CTGATCCTCAGATAAG	CC1
SMC02.T.CTGCCTAGTGACTACT	CC1
SMC02.T.CTGCTGTTCTAGCACA	CC1
SMC02.T.CTGCTGTTCTCTGCTG	CC1
SMC02.T.CTGGTCTCAATCTACG	CC1
SMC02.T.CTGTGCTCACTGTGTA	CC1
SMC02.T.CTTAACTCAGCTGCTG	CC1
SMC02.T.CTTAGGAAGGCGACAT	CC1
SMC02.T.CTTAGGAGTCTGCCAG	CC1
SMC02.T.CTTGGCTTCAACACTG	CC1
SMC02.T.CTTTGCGAGCGAGAAA	CC1
SMC02.T.GAAACTCAGTCCTCCT	CC1
SMC02.T.GAACATCAGACTCGGA	CC1
SMC02.T.GAAGCAGCATGAGCGA	CC1
SMC02.T.GAATGAAAGCGTTGCC	CC1

SMC02.T.GAATGAACATTCTTAC	CC1
SMC02.T.GACACGCGTGTGGCTC	CC1
SMC02.T.GACACGCTCACCCTCA	CC1
SMC02.T.GACCAATGTACCGGCT	CC1
SMC02.T.GACCTGGTCCACGACG	CC1
SMC02.T.GACGCGTCAATCCGAT	CC1
SMC02.T.GACGTGCCACCAGATT	CC1
SMC02.T.GACGTGCTCTGAGGGA	CC1
SMC02.T.GACGTTAGTCGGCTCA	CC1
SMC02.T.GACTACAAGTAGCCGA	CC1
SMC02.T.GACTACAGTGCGGTAA	CC1
SMC02.T.GACTGCGTCTGGCGAC	CC1
SMC02.T.GATCGTAGTGTGCCTG	CC1
SMC02.T.GATCTAGTCCACGAAT	CC1
SMC02.T.GATCTAGTCTCTTGAT	CC1
SMC02.T.GATGAAAGTTCTCATT	CC1
SMC02.T.GATGAAATCGCCATAA	CC1
SMC02.T.GATTCAGGTCGAGTTT	CC1
SMC02.T.GCAAACTAGAAGGTTT	CC2
SMC02.T.GCACATACATAGTAAG	CC1
SMC02.T.GCAGCCAAGTTCGCAT	CC1
SMC02.T.GCAGCCACAAAGTCAA	CC1
SMC02.T.GCAGCCACACTTGGAT	CC1
SMC02.T.GCAGCCAGTAGAGTGC	CC1
SMC02.T.GCAGTTACAACACCTA	CC1
SMC02.T.GCATGATAGTGGGATC	CC1
SMC02.T.GCATGATGTGTTTGGT	CC1
SMC02.T.GCATGCGAGACCGGAT	CC1
SMC02.T.GCATGTAGTAACGCGA	CC1
SMC02.T.GCCTCTAGTAGGCATG	CC1
SMC02.T.GCGACCAAGATGCCAG	CC1
SMC02.T.GCGCCAATCTTAGAGC	CC1
SMC02.T.GCGGGTTTCCTTGACC	CC1
SMC02.T.GCGGGTTTCTAACTTC	CC1
SMC02.T.GCTCCTACACTATCTT	CC1
SMC02.T.GCTCCTAGTGTGGTTT	CC1
SMC02.T.GCTCTGTGTACTCAAC	CC1
SMC02.T.GCTGCAGGTAGCTCCG	CC1
SMC02.T.GCTGGGTGTGCCTGTG	CC1
SMC02.T.GCTTCCAAGATGTCGG	CC1
SMC02.T.GGAAAGCGTCGCGAAA	CC1
SMC02.T.GGAACTTGTGGACGAT	CC1
SMC02.T.GGAATAAAGGGAACGG	CC1
SMC02.T.GGAATAAGTACTTCTT	CC1
SMC02.T.GGACGTCTCCAGAAGG	CC1
SMC02.T.GGAGCAAGTGGACGAT	CC1
SMC02.T.GGATGTTTCCCACTTG	CC1
SMC02.T.GGCGACTCACGGTGTC	CC1
SMC02.T.GGCGACTTCTCCGGTT	CC1
SMC02.T.GGCTGGTTCGTTACAG	CC1
SMC02.T.GGGCACTAGCTCCCAG	CC1
SMC02.T.GGGCATCAGAGTCTGG	CC1
SMC02.T.GGGTCTGAGAGTACAT	CC1
SMC02.T.GGGTCTGAGTCCATAC	CC1
SMC02.T.GGTGAAGTCTCGGACG	CC1
SMC02.T.GGTGCGTAGATGTTAG	CC1
SMC02.T.GGTGCGTTCGTTGCCT	CC1
SMC02.T.GGTGTTAAGTCCTCCT	CC1

SMC02.T.GGTGTTATCCGAGCCA	CC1
SMC02.T.GTACGTACATGGGACA	CC1
SMC02.T.GTATCTTGTTTGTTGG	CC1
SMC02.T.GTATTCTCATCGACGC	CC1
SMC02.T.GTATTCTTCGCTAGCG	CC1
SMC02.T.GTCAAGTGTAGCAAAT	CC1
SMC02.T.GTCAAGTGTCAGAAGC	CC1
SMC02.T.GTCAAGTGTCGTCTTC	CC1
SMC02.T.GTCACAATCATTTGGG	CC1
SMC02.T.GTCGGGTCATACTACG	CC1
SMC02.T.GTCGGGTTCTATCCCG	CC1
SMC02.T.GTCGTAAGTCTAGGTT	CC1
SMC02.T.GTCTCGTAGAGATGAG	CC1
SMC02.T.GTCTCGTGTATATGAG	CC1
SMC02.T.GTCTCGTTCACGCGGT	CC1
SMC02.T.GTCTCGTTCGGAGCAA	CC1
SMC02.T.GTCTTCGAGCGTTTAC	CC1
SMC02.T.GTGAAGGCATGCCACG	CC1
SMC02.T.GTGAAGGTCATCGCTC	CC1
SMC02.T.GTGCAGCGTCGAATCT	CC1
SMC02.T.GTGCATAGTAGCAAAT	CC1
SMC02.T.GTGCTTCTCAACACCA	CC1
SMC02.T.GTGTGCGAGGCAATTA	CC1
SMC02.T.GTGTGCGGTAGATTAG	CC1
SMC02.T.GTGTTAGAGCCACGCT	CC1
SMC02.T.GTTAAGCTCGACAGCC	CC1
SMC02.T.GTTAAGCTCTCAAACG	CC2
SMC02.T.GTTCGGGGTCGGCATC	CC1
SMC02.T.GTTCTCGAGACTAGGC	CC1
SMC02.T.TAAACCGGTGTAACGG	CC1
SMC02.T.TAAGAGAAGACAGAGA	CC1
SMC02.T.TAAGAGAAGTACGTTC	CC2
SMC02.T.TACACGATCGCGTTTC	CC1
SMC02.T.TACAGTGCACGACGAA	CC1
SMC02.T.TACGGATTCCATGAGT	CC1
SMC02.T.TACTTACTCCCGGATG	CC1
SMC02.T.TACTTGTCACAAGCCC	CC1
SMC02.T.TAGACCAAGATGGGTC	CC1
SMC02.T.TAGACCAAGCGAAGGG	CC1
SMC02.T.TAGACCACAACTTGAC	CC1
SMC02.T.TAGCCGGTCCTATTCA	CC1
SMC02.T.TAGGCATAGAGCCTAG	CC1
SMC02.T.TAGTGGTGTGACTACT	CC1
SMC02.T.TATCAGGGTCCCTTGT	CC1
SMC02.T.TATCTCAGTCAAGCGA	CC1
SMC02.T.TATGCCCCACGGTTTA	CC1
SMC02.T.TATTACCTCCGAGCCA	CC1
SMC02.T.TCAACGAGTGACAAAT	CC1
SMC02.T.TCAATCTTCTCCCTGA	CC1
SMC02.T.TCACAAGCATACCATG	CC1
SMC02.T.TCAGGTACACAAGACG	CC1
SMC02.T.TCATTACAGCGTAGTG	CC1
SMC02.T.TCATTTGAGTCTCAAC	CC1
SMC02.T.TCCCGATAGAAGGTGA	CC1
SMC02.T.TCGAGGCAGTGAAGAG	CC1
SMC02.T.TCGAGGCTCCAAACAC	CC1
SMC02.T.TCGAGGCTCCCTGACT	CC1
SMC02.T.TCGGGACTCGTTACAG	CC1

SMC02.T.TCGTAGAGTTAAGGGC	CC1
SMC02.T.TCTATTGTCCGTAGGC	CC1
SMC02.T.TCTCATACAAACCCAT	CC1
SMC02.T.TCTCTAACACGCCAGT	CC1
SMC02.T.TCTCTAATCGCAAGCC	CC1
SMC02.T.TCTGAGAAGTCAAGCG	CC1
SMC02.T.TCTGAGACATGTCCTC	CC2
SMC02.T.TCTTCGGCATTAGGCT	CC1
SMC02.T.TCTTTCCCATCATCCC	CC1
SMC02.T.TGAAAGAAGTAGTGCG	CC1
SMC02.T.TGACGGCTCGCATGAT	CC1
SMC02.T.TGACTAGGTTCCACAA	CC1
SMC02.T.TGACTTTAGATTACCC	CC1
SMC02.T.TGACTTTGTCTCACCT	CC1
SMC02.T.TGAGCCGAGAGGTACC	CC1
SMC02.T.TGAGCCGGTACCGTTA	CC1
SMC02.T.TGAGCCGTCAGAGGTG	CC1
SMC02.T.TGAGCCGTCTGCTGCT	CC1
SMC02.T.TGAGGGATCCGCTGTT	CC1
SMC02.T.TGATTTCCACGTCAGC	CC1
SMC02.T.TGATTTCTCCTTTACA	CC1
SMC02.T.TGCACCTTCAATCACG	CC1
SMC02.T.TGCCAAAGTTCAGCGC	CC1
SMC02.T.TGCCAAATCAGTGCAT	CC1
SMC02.T.TGCCCATCATGCAATC	CC1
SMC02.T.TGCCCATGTCACCTAA	CC1
SMC02.T.TGCCCATTCAGTCAGT	CC1
SMC02.T.TGCGCAGCAGTGAGTG	CC2
SMC02.T.TGCGGGTCACCTATCC	CC1
SMC02.T.TGCGGGTTCGTGGACC	CC1
SMC02.T.TGCGTGGGTCTTCAAG	CC1
SMC02.T.TGCGTGGTCTGCTTGC	CC1
SMC02.T.TGCTACCCATACGCTA	CC1
SMC02.T.TGGACGCCAATAGAGT	CC1
SMC02.T.TGGCGCAAGGACATTA	CC1
SMC02.T.TGGCGCAAGTCACGCC	CC1
SMC02.T.TGGCTGGAGACATAAC	CC1
SMC02.T.TGGGCGTTCTGATACG	CC1
SMC02.T.TGGTTAGCAAGCGTAG	CC1
SMC02.T.TGTATTCAGGATTCGG	CC1
SMC02.T.TGTTCCGGTTCGTCTC	CC1
SMC02.T.TTAGGACTCAAAGTAG	CC1
SMC02.T.TTAGGCAAGTCAAGCG	CC1
SMC02.T.TTAGGCAGTACCAGTT	CC1
SMC02.T.TTGACTTCACAACTGT	CC1
SMC02.T.TTGACTTGTGGTCTCG	CC1
SMC02.T.TTGCCGTTCACTATTC	CC1
SMC02.T.TTGGCAAAGTACGTTC	CC1
SMC02.T.TTGGCAATCAAGGTAA	CC1
SMC02.T.TTGGCAATCCAAAGTC	CC1
SMC02.T.TTTACTGAGACACGAC	CC1
SMC02.T.TTTACTGTCTGCAAGT	CC1
SMC02.T.TTTCCTCGTATAAACG	CC1
SMC02.T.TTTGCGCCAGATCCAT	CC1
SMC02.T.TTTGTCACAGTATAAG	CC1
SMC03.T.AAACCTGGTTGCCTCT	CC1
SMC03.T.AAACCTGTCAGTTTGG	CC2
SMC03.T.AAACGGGAGATGGGTC	CC2

SMC03.T.AAACGGGCATTACCTT	CC2
SMC03.T.AAAGATGAGATCCGAG	CC2
SMC03.T.AAAGCAAAGATCACGG	CC2
SMC03.T.AAAGCAAGTAGGCTGA	CC2
SMC03.T.AAAGTAGAGGCCCTCA	CC2
SMC03.T.AAATGCCAGATGTCGG	CC2
SMC03.T.AAATGCCCAACAACCT	CC2
SMC03.T.AACACGTAGTGTGAAT	CC2
SMC03.T.AACACGTGTCTCACCT	CC2
SMC03.T.AACACGTTCACGATGT	CC2
SMC03.T.AACCATGGTAGGCATG	CC2
SMC03.T.AACCGCGCATTCGACA	CC2
SMC03.T.AACTCAGTCCGCATCT	CC2
SMC03.T.AACTCCCGTCACAAGG	CC2
SMC03.T.AACTCTTTCCTGTAGA	CC2
SMC03.T.AACTGGTCAGTCGTGC	CC2
SMC03.T.AACTGGTGTCTCCACT	CC2
SMC03.T.AACTTTCAGAGTACCG	CC2
SMC03.T.AACTTTCGTCTGATCA	CC2
SMC03.T.AACTTTCTCAGTACGT	CC2
SMC03.T.AACTTTCTCGGCTTGG	CC2
SMC03.T.AAGACCTGTCGTGGCT	CC2
SMC03.T.AAGACCTGTGTTCGAT	CC2
SMC03.T.AAGGAGCAGGGCTTCC	CC2
SMC03.T.AAGGAGCCAGTAAGCG	CC2
SMC03.T.AAGGAGCTCCACGTTC	CC2
SMC03.T.AAGGAGCTCGTACGGC	CC2
SMC03.T.AAGGCAGAGCTGTCTA	CC2
SMC03.T.AAGGTTCAGGTACTCT	CC2
SMC03.T.AAGGTTCCATGTCCTC	CC2
SMC03.T.AATCCAGAGCCAGTAG	CC2
SMC03.T.AATCCAGAGTACGTAA	CC2
SMC03.T.AATCCAGGTTTGTGTG	CC2
SMC03.T.AATCCAGTCCTTTCTC	CC2
SMC03.T.AATCGGTTCGCCAGCA	CC2
SMC03.T.AATCGGTTCTATGTGG	CC1
SMC03.T.ACACCAATCGCTTGTC	CC2
SMC03.T.ACACCCTAGTGCTGCC	CC2
SMC03.T.ACAGCCGCAACTTGAC	CC2
SMC03.T.ACAGCCGCACCACGTG	CC1
SMC03.T.ACAGCTAAGCTAACAA	CC2
SMC03.T.ACAGCTAAGTCAAGGC	CC2
SMC03.T.ACATACGGTAGCTCCG	CC2
SMC03.T.ACATACGTCAGAAATG	CC1
SMC03.T.ACATCAGTCATAAAGG	CC2
SMC03.T.ACATGGTAGAGGTTGC	CC2
SMC03.T.ACCAGTATCTACGAGT	CC2
SMC03.T.ACCCACTAGAGTCGGT	CC2
SMC03.T.ACCCACTAGCCACTAT	CC2
SMC03.T.ACCGTAACACCTATCC	CC2
SMC03.T.ACCGTAAGTGTCCTCT	CC2
SMC03.T.ACCGTAATCTGTCCGT	CC2
SMC03.T.ACGAGCCGTCTGGTCG	CC2
SMC03.T.ACGAGGACACATAACC	CC2
SMC03.T.ACGATGTAGGATGGAA	CC2
SMC03.T.ACGCAGCAGCCCAACC	CC2
SMC03.T.ACGCAGCCAAGTCTGT	CC2
SMC03.T.ACGCCAGTCAAAGTAG	CC1

SMC03.T.ACGCCGACAGGATTGG	CC2
SMC03.T.ACGGAGATCAATCACG	CC2
SMC03.T.ACGGCCAAGCCAGTTT	CC2
SMC03.T.ACGGCCATCATTGCGA	CC2
SMC03.T.ACTATCTAGGAGCGAG	CC2
SMC03.T.ACTATCTAGGATGGAA	CC2
SMC03.T.ACTGAGTCAAGTTGTC	CC2
SMC03.T.ACTGAGTTCAGTCAGT	CC2
SMC03.T.ACTGATGCAGTTCATG	CC2
SMC03.T.ACTGATGTCCACGTTC	CC2
SMC03.T.ACTGTCCAGAGCTATA	CC2
SMC03.T.ACTGTCCAGGGATCTG	CC2
SMC03.T.ACTGTCCTCTGATTCT	CC2
SMC03.T.ACTGTCCTCTTGTATC	CC2
SMC03.T.ACTTACTAGGCGCTCT	CC2
SMC03.T.ACTTACTAGTACGCGA	CC2
SMC03.T.ACTTACTAGTCCCACG	CC2
SMC03.T.ACTTACTCAATAAGCA	CC2
SMC03.T.ACTTACTTCACATAGC	CC2
SMC03.T.ACTTACTTCCGCATAA	CC2
SMC03.T.ACTTGTTGTTACGACT	CC2
SMC03.T.ACTTGTTGTTGCCTCT	CC2
SMC03.T.ACTTGTTTCAGGCGAA	CC2
SMC03.T.ACTTTCATCCACGCAG	CC2
SMC03.T.ACTTTCATCTAACGGT	CC2
SMC03.T.AGAATAGGTCAATGTC	CC2
SMC03.T.AGAATAGTCGGGAGTA	CC2
SMC03.T.AGACGTTAGATATACG	CC2
SMC03.T.AGACGTTAGCTGGAAC	CC2
SMC03.T.AGACGTTCAATAAGCA	CC2
SMC03.T.AGAGCGAGTATTCTCT	CC2
SMC03.T.AGAGCGAGTCTGCCAG	CC2
SMC03.T.AGAGCGATCTTGAGGT	CC2
SMC03.T.AGAGTGGCACCAGTTA	CC2
SMC03.T.AGAGTGGCATCAGTCA	CC2
SMC03.T.AGAGTGGTCGGCTACG	CC2
SMC03.T.AGAGTGGTCTGACCTC	CC2
SMC03.T.AGATCTGAGATCCGAG	CC2
SMC03.T.AGATTGCTCTGTTTGT	CC2
SMC03.T.AGCAGCCCAATAAGCA	CC2
SMC03.T.AGCATACAGGTGCAAC	CC2
SMC03.T.AGCATACCAAGCCATT	CC2
SMC03.T.AGCATACGTCTAGGTT	CC2
SMC03.T.AGCCTAAAGCCAACAG	CC2
SMC03.T.AGCGGTCAGACACTAA	CC2
SMC03.T.AGCGGTCAGCTAACTC	CC2
SMC03.T.AGCGTATAGGCCATAG	CC2
SMC03.T.AGCGTATCACGACGAA	CC2
SMC03.T.AGCGTCGCAAGCTGTT	CC2
SMC03.T.AGCGTCGCAGTAAGAT	CC2
SMC03.T.AGCTCCTTCTATCCTA	CC2
SMC03.T.AGCTCTCAGTGAACGC	CC2
SMC03.T.AGCTCTCGTCCTCCAT	CC2
SMC03.T.AGCTTGAGTCTCTTTA	CC2
SMC03.T.AGCTTGAGTGTAAGTA	CC2
SMC03.T.AGGCCACAGATAGCAT	CC1
SMC03.T.AGGCCACGTGTATGGG	CC2
SMC03.T.AGGCCGTGTGCGCTTG	CC2

SMC03.T.AGGCCGTGTTTGACTG	CC2
SMC03.T.AGGCCGTTCACATGCA	CC2
SMC03.T.AGGGAGTAGCTGTTCA	CC2
SMC03.T.AGGGAGTCAAGTACCT	CC2
SMC03.T.AGGGAGTGTAGCGTGA	CC2
SMC03.T.AGGGAGTGTTCGCGAC	CC1
SMC03.T.AGGGATGCACAGGTTT	CC2
SMC03.T.AGGGTGACAATGTAAG	CC2
SMC03.T.AGGGTGAGTCGACTGC	CC2
SMC03.T.AGGGTGAGTGCCTGCA	CC2
SMC03.T.AGGTCATCAACACGCC	CC2
SMC03.T.AGGTCATGTGATGTGG	CC2
SMC03.T.AGTAGTCAGGTAGCTG	CC2
SMC03.T.AGTAGTCGTCCGTTAA	CC2
SMC03.T.AGTAGTCTCATTGCGA	CC2
SMC03.T.AGTCTTTAGACCTTTG	CC2
SMC03.T.AGTCTTTAGTGTTTGC	CC2
SMC03.T.AGTGAGGCACTCAGGC	CC1
SMC03.T.AGTGAGGCACTGTGTA	CC2
SMC03.T.AGTGAGGCAGTTCCCT	CC2
SMC03.T.AGTGTCAGTTCGTGAT	CC2
SMC03.T.AGTTGGTCAAGTAATG	CC2
SMC03.T.AGTTGGTTCCATGCTC	CC2
SMC03.T.ATAAGAGTCAATAAGG	CC2
SMC03.T.ATAGACCCAGCGTCCA	CC2
SMC03.T.ATCACGAAGCGTGAAC	CC2
SMC03.T.ATCACGAGTAAGTGGC	CC2
SMC03.T.ATCATCTAGACTGTAA	CC2
SMC03.T.ATCATGGGTAAGGATT	CC2
SMC03.T.ATCCACCAGACACGAC	CC1
SMC03.T.ATCCACCCACAGGTTT	CC2
SMC03.T.ATCCGAAGTTAAGACA	CC2
SMC03.T.ATCGAGTTCCCAACGG	CC2
SMC03.T.ATCTACTAGGTTACCT	CC2
SMC03.T.ATCTACTGTCATGCCG	CC2
SMC03.T.ATCTGCCTCATCTGTT	CC2
SMC03.T.ATCTGCCTCGTGGGAA	CC2
SMC03.T.ATGAGGGAGCGAGAAA	CC2
SMC03.T.ATGAGGGTCATATCGG	CC2
SMC03.T.ATGCGATCATGGTCTA	CC2
SMC03.T.ATGGGAGGTAAGGGAA	CC2
SMC03.T.ATGTGTGAGTATGACA	CC2
SMC03.T.ATTACTCTCTAGAGTC	CC2
SMC03.T.ATTATCCGTACACCGC	CC2
SMC03.T.ATTATCCGTCGCGAAA	CC2
SMC03.T.ATTCTACAGTACGTAA	CC2
SMC03.T.ATTCTACTCGCATGAT	CC2
SMC03.T.ATTCTACTCGCCATAA	CC2
SMC03.T.ATTGGACCAGACAGGT	CC2
SMC03.T.ATTGGACCATTAGCCA	CC2
SMC03.T.ATTGGTGCACCCAGTG	CC2
SMC03.T.ATTGGTGGTTCCGTCT	CC2
SMC03.T.ATTGGTGGTTCGAATC	CC2
SMC03.T.ATTTCTGAGCGTCAAG	CC2
SMC03.T.ATTTCTGAGCTAAGAT	CC2
SMC03.T.CAACCAACAGGACGTA	CC2
SMC03.T.CAACCTCAGAATAGGG	CC2
SMC03.T.CAACCTCAGTCTCGGC	CC2

SMC03.T.CAACTAGAGATGTGGC	CC2
SMC03.T.CAACTAGCACATGTGT	CC2
SMC03.T.CAACTAGCACCTCGGA	CC2
SMC03.T.CAACTAGTCACTCTTA	CC2
SMC03.T.CAAGATCAGGGTTTCT	CC2
SMC03.T.CAAGATCCATGCCCGA	CC2
SMC03.T.CAAGATCTCATGTCCC	CC2
SMC03.T.CAAGTTGTCAGTTCGA	CC2
SMC03.T.CAAGTTGTCTGGAGCC	CC2
SMC03.T.CACAAACAGTTGTCGT	CC2
SMC03.T.CACAAACGTCATATCG	CC2
SMC03.T.CACACAATCCACGAAT	CC2
SMC03.T.CACACCTTCACCACCT	CC1
SMC03.T.CACACCTTCCTTTCTC	CC2
SMC03.T.CACACCTTCGGCTACG	CC2
SMC03.T.CACAGGCTCGTCGTTC	CC2
SMC03.T.CACATAGCAGACGCCT	CC2
SMC03.T.CACATAGGTCCTGCTT	CC2
SMC03.T.CACATTTTCCCTCAGT	CC2
SMC03.T.CACATTTTCCTAAGTG	CC2
SMC03.T.CACATTTTCGCTTAGA	CC2
SMC03.T.CACCACTGTACAGTTC	CC2
SMC03.T.CACCAGGGTAAACCTC	CC2
SMC03.T.CACCTTGCAGGGTTAG	CC2
SMC03.T.CACCTTGGTTATCCGA	CC2
SMC03.T.CACCTTGTCATATCGG	CC1
SMC03.T.CACCTTGTCTGGAGCC	CC2
SMC03.T.CACTCCACACAGGCCT	CC2
SMC03.T.CACTCCATCTGAGTGT	CC1
SMC03.T.CAGAATCAGAAACCTA	CC2
SMC03.T.CAGAATCCACTGTTAG	CC2
SMC03.T.CAGAGAGAGCACGCCT	CC2
SMC03.T.CAGAGAGAGCTAAGAT	CC2
SMC03.T.CAGAGAGCATAAGACA	CC2
SMC03.T.CAGAGAGTCCCAAGAT	CC2
SMC03.T.CAGATCAGTAATCACC	CC2
SMC03.T.CAGATCATCACTTCAT	CC1
SMC03.T.CAGCAGCAGACAAGCC	CC2
SMC03.T.CAGCAGCCATCCCATC	CC2
SMC03.T.CAGCAGCGTCCTGCTT	CC2
SMC03.T.CAGCAGCGTGCAACGA	CC2
SMC03.T.CAGCAGCTCGGCCGAT	CC2
SMC03.T.CAGCATAGTTACGACT	CC2
SMC03.T.CAGCCGACACCGGAAA	CC1
SMC03.T.CAGCGACAGCCCTAAT	CC2
SMC03.T.CAGCGACTCAGTGCAT	CC2
SMC03.T.CAGCGACTCGGTTAAC	CC2
SMC03.T.CAGCTAATCCATGAAC	CC2
SMC03.T.CAGGTGCCAATGCCAT	CC2
SMC03.T.CAGTAACAGAGTAATC	CC2
SMC03.T.CAGTAACGTACAGTGG	CC1
SMC03.T.CAGTCCTAGGTACTCT	CC2
SMC03.T.CAGTCCTGTTCCACTC	CC2
SMC03.T.CAGTCCTTCTTGAGGT	CC2
SMC03.T.CATATTCAGCGACGTA	CC2
SMC03.T.CATCAGAGTTATCCGA	CC2
SMC03.T.CATCCACTCTTATCTG	CC2
SMC03.T.CATCGGGAGTCGCCGT	CC2

SMC03.T.CATGACACATCGATGT	CC2
SMC03.T.CATGCCTAGTGCTGCC	CC2
SMC03.T.CATGCCTGTAAACGCG	CC2
SMC03.T.CATGCCTGTCCTCTTG	CC2
SMC03.T.CATGCCTTCCCTTGTG	CC2
SMC03.T.CATGCCTTCTGCTTGC	CC2
SMC03.T.CATGGCGCATCACGTA	CC2
SMC03.T.CATGGCGGTAGGAGTC	CC2
SMC03.T.CATGGCGGTTATCACG	CC2
SMC03.T.CATGGCGTCAAGCCTA	CC2
SMC03.T.CATTATCAGTGGTAGC	CC2
SMC03.T.CATTATCCATAGACTC	CC1
SMC03.T.CATTCGCAGAATTCCC	CC2
SMC03.T.CATTCGCGTCCGACGT	CC2
SMC03.T.CCACCTATCGCCTGAG	CC2
SMC03.T.CCACGGACATCTATGG	CC2
SMC03.T.CCACTACAGTTAGCGG	CC2
SMC03.T.CCACTACGTTTGGCGC	CC2
SMC03.T.CCAGCGAAGTTGTCGT	CC2
SMC03.T.CCATTCGCAGGTCTCG	CC2
SMC03.T.CCCAATCAGTACATGA	CC1
SMC03.T.CCCAATCTCACCATAG	CC2
SMC03.T.CCCAGTTCATCTACGA	CC2
SMC03.T.CCCAGTTGTCTCTCGT	CC2
SMC03.T.CCCATACCATCCTTGC	CC2
SMC03.T.CCCTCCTAGAGGGCTT	CC2
SMC03.T.CCCTCCTTCCGGCACA	CC2
SMC03.T.CCGGGATAGAGGGCTT	CC2
SMC03.T.CCGGGATAGGGAACGG	CC2
SMC03.T.CCGGGATCACATGTGT	CC2
SMC03.T.CCGGTAGGTACTTAGC	CC1
SMC03.T.CCGTACTAGCACAGGT	CC2
SMC03.T.CCGTACTGTGAAAGAG	CC2
SMC03.T.CCGTGGAAGCCACTAT	CC1
SMC03.T.CCGTGGAGTTACTGAC	CC2
SMC03.T.CCGTTCAAGAAAGTGG	CC1
SMC03.T.CCGTTCAAGATGAGAG	CC2
SMC03.T.CCGTTCAAGTATGACA	CC2
SMC03.T.CCTAAAGAGTACGACG	CC2
SMC03.T.CCTAAAGCACAGCGTC	CC2
SMC03.T.CCTAAAGTCCGCATCT	CC2
SMC03.T.CCTACACCATACTACG	CC1
SMC03.T.CCTACCACATGCCTTC	CC1
SMC03.T.CCTAGCTGTGGAAAGA	CC2
SMC03.T.CCTATTACAGGCTGAA	CC2
SMC03.T.CCTCAGTAGTCGATAA	CC2
SMC03.T.CCTCTGAAGCTGATAA	CC2
SMC03.T.CCTCTGAGTGTTGGGA	CC2
SMC03.T.CCTCTGATCTAGAGTC	CC2
SMC03.T.CCTTCCCCAGTAGAGC	CC2
SMC03.T.CCTTCGATCTCGTATT	CC2
SMC03.T.CCTTTCTAGAAACCGC	CC2
SMC03.T.CCTTTCTAGAAGAAGC	CC2
SMC03.T.CGAATGTAGCTAGTCT	CC1
SMC03.T.CGAATGTCAGCCAGAA	CC2
SMC03.T.CGACCTTCATGTCCTC	CC2
SMC03.T.CGACTTCAGACAATAC	CC2
SMC03.T.CGACTTCAGATCCGAG	CC2

SMC03.T.CGACTTCGTTCCATGA	CC2
SMC03.T.CGACTTCTCTCCCTGA	CC2
SMC03.T.CGAGAAGCAATGGTCT	CC2
SMC03.T.CGAGAAGCACATGACT	CC2
SMC03.T.CGAGAAGTCTTTACGT	CC2
SMC03.T.CGAGCACAGGAGCGAG	CC2
SMC03.T.CGAGCACGTAAGGGCT	CC2
SMC03.T.CGAGCACTCAGGTTCA	CC2
SMC03.T.CGATCGGTCAGTGCAT	CC2
SMC03.T.CGATGTACATAGTAAG	CC2
SMC03.T.CGATGTACATCGGACC	CC2
SMC03.T.CGATGTATCTTTAGGG	CC2
SMC03.T.CGATTGACACGGATAG	CC2
SMC03.T.CGATTGAGTCTTGATG	CC2
SMC03.T.CGATTGATCCTCATTA	CC2
SMC03.T.CGCCAAGAGATCGATA	CC2
SMC03.T.CGCGGTAAGATTACCC	CC2
SMC03.T.CGCGGTAAGTGTTTGC	CC2
SMC03.T.CGCGTTTAGGCTAGCA	CC2
SMC03.T.CGCGTTTCAGCATACT	CC2
SMC03.T.CGCGTTTCATTAGCCA	CC2
SMC03.T.CGCGTTTGTTAGATGA	CC2
SMC03.T.CGCTATCTCACCAGGC	CC2
SMC03.T.CGCTATCTCTGGTTCC	CC2
SMC03.T.CGCTGGAAGTAGCCGA	CC2
SMC03.T.CGCTGGAGTGGAAAGA	CC2
SMC03.T.CGCTTCAGTAGCGTAG	CC2
SMC03.T.CGCTTCAGTGGCGAAT	CC2
SMC03.T.CGCTTCATCCAAGTAC	CC2
SMC03.T.CGGACACGTAAGAGAG	CC2
SMC03.T.CGGACGTGTATTCGTG	CC2
SMC03.T.CGGACGTGTTCGTCTC	CC2
SMC03.T.CGGACGTTCCGTAGGC	CC2
SMC03.T.CGGACTGCATTCTCAT	CC2
SMC03.T.CGGACTGGTACTCGCG	CC2
SMC03.T.CGGAGCTTCGTACCGG	CC2
SMC03.T.CGGAGTCCACAGGCCT	CC2
SMC03.T.CGGAGTCGTGACGGTA	CC1
SMC03.T.CGGAGTCTCACTTACT	CC2
SMC03.T.CGGAGTCTCTCGTTTA	CC2
SMC03.T.CGGCTAGAGGGTTCCC	CC2
SMC03.T.CGGGTCATCAACACAC	CC2
SMC03.T.CGGTTAATCCCAACGG	CC2
SMC03.T.CGTAGCGGTAGGCTGA	CC2
SMC03.T.CGTAGCGTCCTAGAAC	CC2
SMC03.T.CGTAGGCAGAACTGTA	CC2
SMC03.T.CGTAGGCTCAACACGT	CC2
SMC03.T.CGTCAGGCAGTAGAGC	CC2
SMC03.T.CGTCCATCACATTTCT	CC2
SMC03.T.CGTCCATGTCTGATTG	CC2
SMC03.T.CGTCTACAGTCCGTAT	CC2
SMC03.T.CGTCTACAGTGGACGT	CC2
SMC03.T.CGTGAGCAGACTAAGT	CC2
SMC03.T.CGTGAGCAGGCAATTA	CC2
SMC03.T.CGTGAGCCACTGTCGG	CC1
SMC03.T.CGTGTAAAGAAGGGTA	CC2
SMC03.T.CGTGTAACACGAAAGC	CC2
SMC03.T.CGTGTAAGTCTCCACT	CC1

SMC03.T.CGTGTCTCAGTAAGAT	CC2
SMC03.T.CGTTAGAAGGTACTCT	CC2
SMC03.T.CGTTAGATCTTGTCAT	CC2
SMC03.T.CGTTCTGGTTTACTCT	CC2
SMC03.T.CGTTGGGCACCCTATC	CC2
SMC03.T.CGTTGGGCAGCGAACA	CC2
SMC03.T.CGTTGGGTCTTGCCGT	CC2
SMC03.T.CTAACTTTCCAGGGCT	CC2
SMC03.T.CTAAGACCAAGTCTGT	CC2
SMC03.T.CTAAGACCAGGATCGA	CC2
SMC03.T.CTAATGGAGCCTTGAT	CC2
SMC03.T.CTAATGGGTTCAGTAC	CC2
SMC03.T.CTACACCGTTACGTCA	CC2
SMC03.T.CTACACCTCTGCTGTC	CC2
SMC03.T.CTACCCAAGCACCGCT	CC2
SMC03.T.CTACCCAAGCTGAACG	CC2
SMC03.T.CTACCCAGTGAAATCA	CC2
SMC03.T.CTACGTCGTACAGTTC	CC2
SMC03.T.CTACGTCGTATTCTCT	CC2
SMC03.T.CTACGTCGTCGACTAT	CC2
SMC03.T.CTAGCCTAGGAGTTTA	CC2
SMC03.T.CTCACACGTCCAGTGC	CC2
SMC03.T.CTCAGAACAGTCGATT	CC2
SMC03.T.CTCAGAACATACTACG	CC2
SMC03.T.CTCATTAAGCAGCGTA	CC2
SMC03.T.CTCATTAAGGAGTTGC	CC2
SMC03.T.CTCATTATCTGTACGA	CC2
SMC03.T.CTCCTAGCATTTGCCC	CC2
SMC03.T.CTCCTAGGTATTCTCT	CC2
SMC03.T.CTCCTAGTCACCGTAA	CC1
SMC03.T.CTCGAAATCTCCAGGG	CC2
SMC03.T.CTCGAGGAGTGACATA	CC2
SMC03.T.CTCGAGGCAATAACGA	CC2
SMC03.T.CTCGAGGCATCCTAGA	CC1
SMC03.T.CTCGAGGTCACCTCGT	CC2
SMC03.T.CTCGGAGAGCCAGTAG	CC2
SMC03.T.CTCGGAGAGCGTCTAT	CC2
SMC03.T.CTCGGAGAGGGAGTAA	CC2
SMC03.T.CTCGGAGCACGGATAG	CC2
SMC03.T.CTCGTACGTGGTCTCG	CC2
SMC03.T.CTCGTCACACAGCGTC	CC2
SMC03.T.CTCTAATAGCAATCTC	CC2
SMC03.T.CTCTACGAGACTTGAA	CC2
SMC03.T.CTCTACGAGAGACGAA	CC2
SMC03.T.CTCTACGGTCCAGTAT	CC2
SMC03.T.CTCTACGTCCCAGGTG	CC2
SMC03.T.CTCTACGTCTCTGCTG	CC2
SMC03.T.CTGAAACAGACTTGAA	CC2
SMC03.T.CTGAAACTCTTAGCCC	CC2
SMC03.T.CTGAAGTAGCGAAGGG	CC2
SMC03.T.CTGAAGTAGTGACATA	CC2
SMC03.T.CTGAAGTGTCCAACTA	CC2
SMC03.T.CTGATAGAGTGGAGTC	CC2
SMC03.T.CTGATAGGTCTTCAAG	CC2
SMC03.T.CTGATCCAGCCAACAG	CC2
SMC03.T.CTGATCCGTACCAGTT	CC2
SMC03.T.CTGCGGAAGATGTGGC	CC1
SMC03.T.CTGCGGAGTAGCAAAT	CC2

SMC03.T.CTGCTGTGTACCATCA	CC2
SMC03.T.CTGGTCTTCTCAACTT	CC2
SMC03.T.CTGGTCTTCTGCAGTA	CC2
SMC03.T.CTGTGCTGTAGGGTAC	CC2
SMC03.T.CTGTGCTTCACAGGCC	CC2
SMC03.T.CTGTGCTTCGTCTGCT	CC2
SMC03.T.CTGTTTAAGGTGCTAG	CC2
SMC03.T.CTGTTTATCACGCGGT	CC2
SMC03.T.CTTACCGAGCGCTCCA	CC2
SMC03.T.CTTACCGGTGATAAAC	CC2
SMC03.T.CTTCTCTGTCGCATCG	CC2
SMC03.T.CTTCTCTTCATGCTCC	CC2
SMC03.T.CTTGGCTGTCAAAGAT	CC2
SMC03.T.CTTGGCTTCCGTCAAA	CC2
SMC03.T.GAAACTCAGAAAGTGG	CC2
SMC03.T.GAAACTCCACCTCGTT	CC1
SMC03.T.GAAACTCGTGTGCCTG	CC2
SMC03.T.GAAATGATCTTTAGTC	CC1
SMC03.T.GAACATCAGTATGACA	CC2
SMC03.T.GAACATCAGTTTGCGT	CC2
SMC03.T.GAAGCAGAGGATGGTC	CC2
SMC03.T.GAAGCAGCACGAAACG	CC2
SMC03.T.GAAGCAGCACTAGTAC	CC2
SMC03.T.GAATGAAAGTACGTTC	CC2
SMC03.T.GACACGCGTACACCGC	CC1
SMC03.T.GACACGCGTGTATGGG	CC2
SMC03.T.GACACGCGTTCTCATT	CC2
SMC03.T.GACCAATGTACGCACC	CC2
SMC03.T.GACCTGGCATCGGAAG	CC2
SMC03.T.GACGCGTTCCACGTGG	CC2
SMC03.T.GACGGCTAGAAGGGTA	CC2
SMC03.T.GACGGCTAGTTGAGTA	CC2
SMC03.T.GACGGCTCACAGACTT	CC2
SMC03.T.GACGTGCCATGCAATC	CC2
SMC03.T.GACGTTAAGGTAGCCA	CC2
SMC03.T.GACGTTATCCGCATAA	CC2
SMC03.T.GACTAACGTCGGCATC	CC2
SMC03.T.GACTACATCTTGTCAT	CC2
SMC03.T.GACTGCGGTTGGGACA	CC2
SMC03.T.GACTGCGTCAAGGCTT	CC2
SMC03.T.GAGCAGACATACCATG	CC2
SMC03.T.GAGGTGACATTCCTGC	CC2
SMC03.T.GAGGTGATCGTAGGAG	CC2
SMC03.T.GAGGTGATCTTTACAC	CC2
SMC03.T.GAGTCCGGTGTGAAAT	CC2
SMC03.T.GATCGATAGTGTACTC	CC2
SMC03.T.GATCGATCACGAAACG	CC2
SMC03.T.GATCGATGTCTCGTTC	CC1
SMC03.T.GATCGCGGTAAATGAC	CC2
SMC03.T.GATCGTATCTGAGGGA	CC2
SMC03.T.GATCTAGCATACTACG	CC2
SMC03.T.GATCTAGCATGCCCGA	CC2
SMC03.T.GATCTAGGTTCGGGCT	CC2
SMC03.T.GATGAAAGTTGGACCC	CC2
SMC03.T.GATGAGGGTTGGGACA	CC2
SMC03.T.GATGAGGTCATACGGT	CC2
SMC03.T.GATGCTACACATCCAA	CC2
SMC03.T.GATGCTACATAGTAAG	CC2

SMC03.T.GATTCAGTCCACGACG	CC2
SMC03.T.GCAAACTCACATCTTT	CC2
SMC03.T.GCAAACTCACCATGTA	CC2
SMC03.T.GCAAACTGTATAAACG	CC2
SMC03.T.GCAATCAAGATCCCGC	CC2
SMC03.T.GCAATCAAGCATGGCA	CC2
SMC03.T.GCAATCAGTTCAACCA	CC2
SMC03.T.GCACATATCGTACCGG	CC2
SMC03.T.GCACTCTTCGCATGAT	CC2
SMC03.T.GCAGCCAAGCTCCTCT	CC2
SMC03.T.GCAGCCACAAGTCTAC	CC2
SMC03.T.GCAGTTAGTACAGACG	CC2
SMC03.T.GCATACAAGAAACCAT	CC2
SMC03.T.GCATACAAGGCACATG	CC2
SMC03.T.GCATACACAACGCACC	CC2
SMC03.T.GCATGCGAGACTAGAT	CC1
SMC03.T.GCATGCGTCACCAGGC	CC2
SMC03.T.GCATGTACAAGTCATC	CC2
SMC03.T.GCCAAATAGAACTCGG	CC2
SMC03.T.GCCAAATGTGTTAAGA	CC2
SMC03.T.GCCAAATGTTCCACTC	CC2
SMC03.T.GCCTCTAAGTCTCCTC	CC2
SMC03.T.GCCTCTATCTATCGCC	CC2
SMC03.T.GCGACCAGTTATTCTC	CC2
SMC03.T.GCGACCATCACAATGC	CC2
SMC03.T.GCGACCATCAGCACAT	CC2
SMC03.T.GCGAGAATCAGCTTAG	CC2
SMC03.T.GCGAGAATCCAACCAA	CC2
SMC03.T.GCGCAACAGCTTTGGT	CC2
SMC03.T.GCGCAACCACACCGAC	CC2
SMC03.T.GCGCAACTCCAAACAC	CC2
SMC03.T.GCGCAGTTCATTATCC	CC2
SMC03.T.GCGGGTTCACCTTGTC	CC2
SMC03.T.GCGGGTTTCTTCCTTC	CC2
SMC03.T.GCTCCTAGTATAATGG	CC2
SMC03.T.GCTCTGTAGAAGGTTT	CC2
SMC03.T.GCTCTGTAGACTACAA	CC2
SMC03.T.GCTCTGTGTTCAACCA	CC2
SMC03.T.GCTGCGACAGCGTAAG	CC2
SMC03.T.GCTGCGAGTAAGAGGA	CC2
SMC03.T.GCTGGGTCAGTAAGCG	CC2
SMC03.T.GCTTCCAAGCAAATCA	CC2
SMC03.T.GGAAAGCCAAGCGATG	CC2
SMC03.T.GGAAAGCGTGACCAAG	CC2
SMC03.T.GGAAAGCTCATCGGAT	CC2
SMC03.T.GGAACTTAGGTGATAT	CC2
SMC03.T.GGAACTTGTCGCTTTC	CC2
SMC03.T.GGAATAACAATTCCTT	CC2
SMC03.T.GGAATAATCACGGTTA	CC2
SMC03.T.GGACATTTCAACACTG	CC2
SMC03.T.GGACGTCTCACTGGGC	CC2
SMC03.T.GGAGCAAAGATGCGAC	CC2
SMC03.T.GGAGCAAAGGTCGGAT	CC2
SMC03.T.GGATGTTCAATCAGAA	CC2
SMC03.T.GGATTACCAGTGACAG	CC2
SMC03.T.GGCAATTAGTGTACTC	CC2
SMC03.T.GGCAATTTCAAACCAC	CC2
SMC03.T.GGCGACTGTCGGGTCT	CC2

SMC03.T.GGCGACTTCCGTACAA	CC2
SMC03.T.GGCGTGTGTAAAGGAG	CC2
SMC03.T.GGCTCGAGTACTTAGC	CC2
SMC03.T.GGCTGGTCAAATACAG	CC2
SMC03.T.GGCTGGTTCTCCAACC	CC2
SMC03.T.GGGAATGAGTGACATA	CC2
SMC03.T.GGGAATGCAACAACCT	CC2
SMC03.T.GGGACCTTCACAATGC	CC2
SMC03.T.GGGCACTGTACCAGTT	CC2
SMC03.T.GGGCACTGTCGACTAT	CC2
SMC03.T.GGGCATCGTCGGATCC	CC2
SMC03.T.GGGCATCGTGGTACAG	CC2
SMC03.T.GGGCATCTCATATCGG	CC2
SMC03.T.GGGTCTGCAGCTGTGC	CC2
SMC03.T.GGGTTGCAGAAGGACA	CC2
SMC03.T.GGGTTGCAGCAGCGTA	CC2
SMC03.T.GGGTTGCAGTGATCGG	CC2
SMC03.T.GGGTTGCCAGCCTTTC	CC2
SMC03.T.GGTATTGAGATGAGAG	CC2
SMC03.T.GGTGAAGAGACATAAC	CC2
SMC03.T.GGTGAAGAGGACTGGT	CC1
SMC03.T.GGTGAAGAGTCGAGTG	CC1
SMC03.T.GGTGAAGGTTACGCGC	CC2
SMC03.T.GGTGCGTAGATTACCC	CC2
SMC03.T.GGTGCGTTCCATGAAC	CC2
SMC03.T.GGTGTTATCCCACTTG	CC1
SMC03.T.GGTGTTATCTCCGGTT	CC2
SMC03.T.GTAACGTCATTTCAGG	CC2
SMC03.T.GTAACGTTCGTGGTCG	CC2
SMC03.T.GTAACTGAGAATCTCC	CC2
SMC03.T.GTACGTACAGACGCTC	CC2
SMC03.T.GTACGTAGTATAATGG	CC2
SMC03.T.GTACGTATCCGTTGTC	CC2
SMC03.T.GTACTCCGTGAGGCTA	CC2
SMC03.T.GTACTCCTCAGTACGT	CC2
SMC03.T.GTACTTTTCAAGAAGT	CC2
SMC03.T.GTAGGCCAGGGTGTTG	CC2
SMC03.T.GTAGGCCTCCCTTGCA	CC1
SMC03.T.GTAGTCACACCTTGTC	CC2
SMC03.T.GTAGTCACACTGAAGG	CC2
SMC03.T.GTATCTTTCCTTGCCA	CC2
SMC03.T.GTATTCTCACTTGGAT	CC1
SMC03.T.GTATTCTGTAAATGAC	CC2
SMC03.T.GTATTCTTCTAACCGA	CC2
SMC03.T.GTCAAGTAGTGCAAGC	CC1
SMC03.T.GTCAAGTCAAGGGTCA	CC2
SMC03.T.GTCAAGTTCTATCCTA	CC2
SMC03.T.GTCACAACAGGTTTCA	CC2
SMC03.T.GTCACAAGTTGTCTTT	CC2
SMC03.T.GTCACGGCATTTCACT	CC2
SMC03.T.GTCATTTGTCTGATCA	CC1
SMC03.T.GTCCTCAAGGAATCGC	CC2
SMC03.T.GTCCTCAAGTTCGCGC	CC2
SMC03.T.GTCCTCACAGCAGTTT	CC2
SMC03.T.GTCGGGTGTGGTGTAG	CC2
SMC03.T.GTCGTAAAGAGGTTAT	CC2
SMC03.T.GTCGTAAAGTTATCGC	CC2
SMC03.T.GTCGTAACATACTACG	CC2

SMC03.T.GTCGTAAGTTCGTGAT	CC1
SMC03.T.GTCGTAATCCATGAGT	CC2
SMC03.T.GTCTCGTAGACCTTTG	CC2
SMC03.T.GTCTCGTGTTAAGATG	CC2
SMC03.T.GTCTCGTTCAAGGCTT	CC2
SMC03.T.GTCTCGTTCCGGGTGT	CC2
SMC03.T.GTCTTCGCACGGTAGA	CC2
SMC03.T.GTGAAGGTCACAACGT	CC2
SMC03.T.GTGCAGCAGGGATCTG	CC2
SMC03.T.GTGCAGCCAGACGTAG	CC2
SMC03.T.GTGCAGCCATTAGGCT	CC2
SMC03.T.GTGCATACAGTTCCCT	CC2
SMC03.T.GTGCGGTAGCACACAG	CC2
SMC03.T.GTGCGGTCAGTAGAGC	CC1
SMC03.T.GTGCTTCCAGAGCCAA	CC2
SMC03.T.GTGCTTCCAGCTGTAT	CC2
SMC03.T.GTTAAGCGTAGGCATG	CC2
SMC03.T.GTTACAGGTCGGGTCT	CC2
SMC03.T.GTTACAGGTGTCCTCT	CC2
SMC03.T.GTTACAGTCCGGGTGT	CC2
SMC03.T.GTTCATTAGAAACGAG	CC2
SMC03.T.GTTCGGGAGATATACG	CC2
SMC03.T.GTTCTCGCAGCTCGAC	CC1
SMC03.T.GTTCTCGGTAGCTGCC	CC2
SMC03.T.GTTTCTAAGAAACGCC	CC2
SMC03.T.GTTTCTAAGCGTTGCC	CC2
SMC03.T.GTTTCTATCCACGAAT	CC2
SMC03.T.GTTTCTATCCGAAGAG	CC2
SMC03.T.TAAACCGCACGAAAGC	CC2
SMC03.T.TAAACCGTCGGTGTTA	CC2
SMC03.T.TAAGAGACACTACAGT	CC2
SMC03.T.TAAGCGTAGCTAGTGG	CC2
SMC03.T.TAAGTGCAGTACACCT	CC2
SMC03.T.TAAGTGCCAATCTACG	CC2
SMC03.T.TAAGTGCTCACTATTC	CC2
SMC03.T.TACACGACACAGATTC	CC2
SMC03.T.TACAGTGAGAAACCTA	CC2
SMC03.T.TACAGTGCACAAGCCC	CC2
SMC03.T.TACCTATAGAGTAATC	CC2
SMC03.T.TACGGATAGTGTCCAT	CC2
SMC03.T.TACGGGCAGTTTCCTT	CC2
SMC03.T.TACGGGCTCCGCATCT	CC2
SMC03.T.TACGGTATCCCTGACT	CC2
SMC03.T.TACTCATAGCAGCGTA	CC2
SMC03.T.TACTCATCAGCTGTAT	CC2
SMC03.T.TACTCATGTCTCACCT	CC1
SMC03.T.TACTTACAGCGCTTAT	CC2
SMC03.T.TACTTACGTAAGGATT	CC2
SMC03.T.TACTTACTCTCTTATG	CC2
SMC03.T.TACTTGTTCCCTGACT	CC2
SMC03.T.TAGACCATCATATCGG	CC2
SMC03.T.TAGAGCTCAATGGAAT	CC2
SMC03.T.TAGCCGGAGGAATCGC	CC2
SMC03.T.TAGTTGGAGCGACGTA	CC2
SMC03.T.TAGTTGGCATGCTAGT	CC2
SMC03.T.TAGTTGGTCAAAGTAG	CC1
SMC03.T.TAGTTGGTCACCGTAA	CC2
SMC03.T.TATCAGGGTGTAATGA	CC2

SMC03.T.TATCAGGTCCTAGGGC	CC2
SMC03.T.TATGCCCAGACTTTCG	CC2
SMC03.T.TATGCCCTCGTTTAGG	CC2
SMC03.T.TATTACCCACACGCTG	CC2
SMC03.T.TCAACGATCCAAACTG	CC2
SMC03.T.TCAACGATCCGAACGC	CC2
SMC03.T.TCAACGATCTTAGCCC	CC2
SMC03.T.TCAATCTTCATCGGAT	CC2
SMC03.T.TCAATCTTCATCTGTT	CC2
SMC03.T.TCACAAGCACGAAACG	CC2
SMC03.T.TCACAAGTCCCAACGG	CC1
SMC03.T.TCACAAGTCGTGGGAA	CC2
SMC03.T.TCACGAACATAGACTC	CC1
SMC03.T.TCACGAATCACCAGGC	CC2
SMC03.T.TCAGCTCCAAGTTGTC	CC2
SMC03.T.TCAGCTCGTGCAGACA	CC2
SMC03.T.TCAGGTAAGATGTGTA	CC2
SMC03.T.TCAGGTACACAAGACG	CC2
SMC03.T.TCATTACTCGGCATCG	CC2
SMC03.T.TCATTTGTCAGAGACG	CC2
SMC03.T.TCATTTGTCCGCTGTT	CC2
SMC03.T.TCCACACGTATGAAAC	CC1
SMC03.T.TCCCGATAGTGGTCCC	CC1
SMC03.T.TCGAGGCGTGTGACGA	CC2
SMC03.T.TCGCGAGTCCGTACAA	CC2
SMC03.T.TCGCGTTAGTATCTCG	CC2
SMC03.T.TCGGGACCAATGGACG	CC2
SMC03.T.TCGGGACTCCACGACG	CC2
SMC03.T.TCGGGACTCGGAATCT	CC2
SMC03.T.TCGGTAAAGCTCCTCT	CC2
SMC03.T.TCGGTAAGTGCAGGTA	CC2
SMC03.T.TCGTACCCAAGGTTCT	CC2
SMC03.T.TCGTACCCAATGGATA	CC2
SMC03.T.TCGTAGAAGACACGAC	CC2
SMC03.T.TCTCATAAGGGCTCTC	CC2
SMC03.T.TCTCATACAGTTCATG	CC2
SMC03.T.TCTCTAAAGGTGTTAA	CC2
SMC03.T.TCTCTAATCAACACTG	CC2
SMC03.T.TCTGGAAAGCTCCCAG	CC2
SMC03.T.TCTGGAAGTTACCGAT	CC2
SMC03.T.TCTGGAAGTTTAGGAA	CC2
SMC03.T.TCTGGAATCGCAAACT	CC2
SMC03.T.TCTTTCCAGATAGTCA	CC2
SMC03.T.TCTTTCCCAAAGCGGT	CC2
SMC03.T.TCTTTCCCAATGGTCT	CC2
SMC03.T.TCTTTCCCACGTCAGC	CC2
SMC03.T.TGAAAGACAAATTGCC	CC2
SMC03.T.TGAAAGAGTCAACATC	CC2
SMC03.T.TGAAAGAGTTACGGAG	CC2
SMC03.T.TGACAACAGAAGGCCT	CC2
SMC03.T.TGACGGCAGAGCAATT	CC2
SMC03.T.TGACGGCCAAACTGCT	CC2
SMC03.T.TGACGGCCAGGTTTCA	CC1
SMC03.T.TGACGGCGTTTGACAC	CC2
SMC03.T.TGACGGCTCCCAAGTA	CC2
SMC03.T.TGACTAGAGCGTTCCG	CC2
SMC03.T.TGACTAGGTCTAACGT	CC2
SMC03.T.TGACTAGTCATCGCTC	CC2

SMC03.T.TGACTAGTCTGCAGTA	CC2
SMC03.T.TGACTTTAGTCTCCTC	CC2
SMC03.T.TGACTTTAGTCTCGGC	CC2
SMC03.T.TGACTTTCAGTTAACC	CC2
SMC03.T.TGAGAGGAGATGTCGG	CC2
SMC03.T.TGAGAGGGTGGCCCTA	CC2
SMC03.T.TGAGAGGTCAAACAAG	CC1
SMC03.T.TGAGCCGGTTACGTCA	CC2
SMC03.T.TGAGCCGTCGTGGTCG	CC2
SMC03.T.TGAGGGAAGACAGACC	CC2
SMC03.T.TGAGGGAGTCTTCTCG	CC2
SMC03.T.TGATTTCAGGCCATAG	CC2
SMC03.T.TGATTTCCACAGGCCT	CC2
SMC03.T.TGATTTCCATATGGTC	CC2
SMC03.T.TGATTTCGTACGCACC	CC2
SMC03.T.TGCACCTGTTTGACTG	CC2
SMC03.T.TGCACCTTCGATAGAA	CC2
SMC03.T.TGCACCTTCTAAGCCA	CC2
SMC03.T.TGCCAAAAGCAATATG	CC2
SMC03.T.TGCCCATAGTACGATA	CC2
SMC03.T.TGCCCATCAGTCGTGC	CC2
SMC03.T.TGCCCATTCACAGTAC	CC2
SMC03.T.TGCGCAGGTACCCAAT	CC2
SMC03.T.TGCTACCTCCTGTACC	CC2
SMC03.T.TGGCCAGTCAAACCAC	CC2
SMC03.T.TGGCGCAAGCTGAACG	CC2
SMC03.T.TGGCTGGAGACCCACC	CC2
SMC03.T.TGGCTGGAGCTAACTC	CC2
SMC03.T.TGGGAAGCAACTGGCC	CC2
SMC03.T.TGGGCGTAGCGCTCCA	CC2
SMC03.T.TGGGCGTTCTTGTTTG	CC2
SMC03.T.TGGTTAGGTATAGTAG	CC2
SMC03.T.TGGTTAGGTCTTTCAT	CC1
SMC03.T.TGGTTCCGTGATAAGT	CC1
SMC03.T.TGTATTCAGGTGCTTT	CC2
SMC03.T.TGTATTCCACAGGTTT	CC2
SMC03.T.TGTATTCGTGTTTGTG	CC2
SMC03.T.TGTCCCACAGGTTTCA	CC2
SMC03.T.TGTCCCACATAAAGGT	CC2
SMC03.T.TGTCCCAGTCACAAGG	CC2
SMC03.T.TGTCCCAGTTCATGGT	CC2
SMC03.T.TGTGTTTCAGAGTGTG	CC2
SMC03.T.TGTGTTTCATCCCATC	CC1
SMC03.T.TGTGTTTTCCGTAGGC	CC2
SMC03.T.TGTGTTTTCGAATGCT	CC2
SMC03.T.TGTTCCGGTCGTTGTA	CC2
SMC03.T.TGTTCCGGTTGGAGGT	CC2
SMC03.T.TGTTCCGGTTTAGCTG	CC2
SMC03.T.TGTTCCGTCCGCATCT	CC2
SMC03.T.TTAACTCAGTAATCCC	CC2
SMC03.T.TTAACTCCAGATTGCT	CC2
SMC03.T.TTAACTCGTGGCGAAT	CC2
SMC03.T.TTAGGACCACGTCAGC	CC2
SMC03.T.TTAGGACTCCAGTAGT	CC2
SMC03.T.TTAGGCAAGACCTTTG	CC2
SMC03.T.TTAGGCAAGTGTGGCA	CC1
SMC03.T.TTAGTTCGTAACGTTC	CC2
SMC03.T.TTATGCTCACGTGAGA	CC2

SMC03.T.TTCCCAGCACCGGAAA	CC2
SMC03.T.TTCCCAGCACGGCTAC	CC2
SMC03.T.TTCCCAGGTTAAGAAC	CC1
SMC03.T.TTCCCAGTCACCAGGC	CC2
SMC03.T.TTCCCAGTCCGTCAAA	CC2
SMC03.T.TTCGAAGCAACCGCCA	CC2
SMC03.T.TTCGAAGTCCGTCAAA	CC2
SMC03.T.TTCGGTCAGCGATCCC	CC2
SMC03.T.TTCGGTCCAGGAACGT	CC2
SMC03.T.TTCGGTCCATGGGACA	CC2
SMC03.T.TTCGGTCGTTCGGCAC	CC2
SMC03.T.TTCTACAGTAGCTCCG	CC2
SMC03.T.TTCTCAACAGTTTACG	CC2
SMC03.T.TTCTCCTCACAAGACG	CC2
SMC03.T.TTCTCCTCATTCTCAT	CC2
SMC03.T.TTCTTAGAGCTGTTCA	CC2
SMC03.T.TTGAACGAGCGACGTA	CC2
SMC03.T.TTGAACGCACCGAATT	CC2
SMC03.T.TTGAACGCATGACGGA	CC2
SMC03.T.TTGCCGTCATCCGCGA	CC2
SMC03.T.TTGCGTCCACTTGGAT	CC2
SMC03.T.TTGCGTCTCATAGCAC	CC2
SMC03.T.TTGGAACAGCTAAGAT	CC2
SMC03.T.TTGGCAAAGCGTTGCC	CC2
SMC03.T.TTGGCAACAAGTAATG	CC2
SMC03.T.TTGGCAACAGACAAAT	CC2
SMC03.T.TTGTAGGCACCTATCC	CC2
SMC03.T.TTGTAGGTCAAACCAC	CC2
SMC03.T.TTGTAGGTCATCGCTC	CC2
SMC03.T.TTGTAGGTCCAAACAC	CC2
SMC03.T.TTGTAGGTCTTTACGT	CC2
SMC03.T.TTTATGCCAGCTGGCT	CC2
SMC03.T.TTTATGCGTAGATTAG	CC2
SMC03.T.TTTCCTCTCCAAGCCG	CC2
SMC03.T.TTTGCGCGTGTTGAGG	CC2
SMC03.T.TTTGGTTAGCGTTGCC	CC2
SMC03.T.TTTGTCAAGACCGGAT	CC2
SMC03.T.TTTGTCAAGAGACGAA	CC2
SMC03.T.TTTGTCAAGAGGGCTT	CC2
SMC04.T.AAACCTGCAGGTCTCG	CC1
SMC04.T.AAACCTGGTGGACGAT	CC1
SMC04.T.AAACCTGTCTGTGCAA	CC1
SMC04.T.AAACGGGCACAGACAG	CC1
SMC04.T.AAAGTAGAGCTAACAA	CC1
SMC04.T.AAAGTAGAGTACTTGC	CC1
SMC04.T.AAAGTAGCAAAGGCGT	CC1
SMC04.T.AACACGTCAGGAATCG	CC1
SMC04.T.AACCATGCAGATCGGA	CC1
SMC04.T.AACCATGGTTGGAGGT	CC1
SMC04.T.AACTCAGCAGTAGAGC	CC1
SMC04.T.AACTCTTGTTTGTGTG	CC1
SMC04.T.AACTGGTTCGTGGGAA	CC1
SMC04.T.AAGGTTCTCAAGAAGT	CC1
SMC04.T.AATCCAGGTCCAGTTA	CC1
SMC04.T.AATCCAGGTTCCATGA	CC1
SMC04.T.AATCCAGTCCCAACGG	CC2
SMC04.T.ACACTGAAGAATTCCC	CC1
SMC04.T.ACACTGATCGTCCAGG	CC1

SMC04.T.ACATCAGGTAAACCTC	CC1
SMC04.T.ACATGGTAGATCCCGC	CC1
SMC04.T.ACCAGTAAGATGAGAG	CC2
SMC04.T.ACCAGTAAGTTAAGTG	CC1
SMC04.T.ACGAGCCGTTAGAACA	CC1
SMC04.T.ACGAGCCTCCATGAGT	CC1
SMC04.T.ACGATACAGACTGTAA	CC2
SMC04.T.ACGATACCATTGGCGC	CC1
SMC04.T.ACGCCGATCAACACTG	CC1
SMC04.T.ACGGAGAGTTACCAGT	CC1
SMC04.T.ACGGCCAAGCGTTTAC	CC1
SMC04.T.ACGGCCATCGGACAAG	CC1
SMC04.T.ACTGATGTCTCTTGAT	CC1
SMC04.T.ACTGTCCAGGCTATCT	CC1
SMC04.T.ACTGTCCGTTCGCGAC	CC2
SMC04.T.AGACGTTAGATCACGG	CC1
SMC04.T.AGACGTTAGGAGTTGC	CC1
SMC04.T.AGAGCTTGTAAGCACG	CC1
SMC04.T.AGAGCTTTCTGGGCCA	CC1
SMC04.T.AGAGTGGGTCTGCAAT	CC1
SMC04.T.AGCAGCCTCCCTCAGT	CC1
SMC04.T.AGCATACGTCCATGAT	CC1
SMC04.T.AGCCTAATCGTTGACA	CC1
SMC04.T.AGCGTATCAAGGACTG	CC2
SMC04.T.AGCTCTCGTACGACCC	CC1
SMC04.T.AGCTCTCGTTGATTCG	CC2
SMC04.T.AGGCCACAGGGTATCG	CC1
SMC04.T.AGGCCACTCGCTTAGA	CC1
SMC04.T.AGGCCGTTCCACGTGG	CC1
SMC04.T.AGGCCGTTCTCTAGGA	CC1
SMC04.T.AGGGTGAAGCCTTGAT	CC1
SMC04.T.AGTGTCACAGGACCCT	CC1
SMC04.T.AGTGTCAGTGACAAAT	CC1
SMC04.T.AGTTGGTGTCATATCG	CC1
SMC04.T.ATAAGAGAGGCCCGTT	CC1
SMC04.T.ATCACGATCAGGCGAA	CC1
SMC04.T.ATCATGGCACCAGCAC	CC1
SMC04.T.ATCCACCGTCACAAGG	CC1
SMC04.T.ATCTACTGTTTGGCGC	CC1
SMC04.T.ATCTGCCCAGGAATGC	CC1
SMC04.T.ATGCGATAGACCGGAT	CC1
SMC04.T.ATGGGAGGTCCAGTTA	CC1
SMC04.T.ATGTGTGGTGATGTCT	CC1
SMC04.T.ATTATCCCACCGATAT	CC1
SMC04.T.ATTATCCTCCAAAGTC	CC1
SMC04.T.ATTATCCTCGTGACAT	CC1
SMC04.T.ATTCTACGTAGCGTGA	CC1
SMC04.T.CAACCAAGTCAGTGGA	CC1
SMC04.T.CAACTAGTCAGGATCT	CC1
SMC04.T.CAAGATCCAAGGTTTC	CC1
SMC04.T.CAAGATCTCTGTCTAT	CC1
SMC04.T.CACAAACAGCCGTCGT	CC1
SMC04.T.CACAGGCCAGGACCCT	CC1
SMC04.T.CACAGGCTCACGACTA	CC1
SMC04.T.CACTCCAAGTAGGCCA	CC1
SMC04.T.CACTCCAGTGATGTGG	CC1
SMC04.T.CAGAGAGAGCTGTTCA	CC1
SMC04.T.CAGGTGCGTTATGTGC	CC1

SMC04.T.CAGGTGCTCTAACTCT	CC1
SMC04.T.CATATTCCAGACAAAT	CC1
SMC04.T.CATATTCGTCACAAGG	CC2
SMC04.T.CATCGAAAGTTAGCGG	CC1
SMC04.T.CATCGAACAGACGTAG	CC1
SMC04.T.CATCGAAGTAGCCTCG	CC1
SMC04.T.CATCGGGTCCGAATGT	CC1
SMC04.T.CATGCCTCAAGGACAC	CC1
SMC04.T.CATGCCTCACACCGCA	CC1
SMC04.T.CATTATCGTTATCCGA	CC1
SMC04.T.CCACGGACAATGTTGC	CC1
SMC04.T.CCACGGACACCAGGTC	CC1
SMC04.T.CCAGCGACATCGTCGG	CC1
SMC04.T.CCATGTCAGCAGGCTA	CC1
SMC04.T.CCATTCGCAAAGTCAA	CC1
SMC04.T.CCCAATCCAGACGCTC	CC1
SMC04.T.CCCAATCGTAAGGGAA	CC1
SMC04.T.CCCATACAGCACCGCT	CC1
SMC04.T.CCCATACAGTGTTAGA	CC1
SMC04.T.CCCTCCTGTGCACTTA	CC1
SMC04.T.CCGTACTAGTGTACGG	CC1
SMC04.T.CCGTTCAAGCCACCTG	CC2
SMC04.T.CCTACCAAGGCAGTCA	CC1
SMC04.T.CCTAGCTCATTTCAGG	CC1
SMC04.T.CCTAGCTTCACCCTCA	CC1
SMC04.T.CCTTCGACAAACGTGG	CC2
SMC04.T.CCTTCGAGTAAATGAC	CC2
SMC04.T.CCTTTCTAGGTGATTA	CC1
SMC04.T.CCTTTCTGTACCATCA	CC1
SMC04.T.CGACCTTGTTATTCTC	CC1
SMC04.T.CGACCTTTCGGAGCAA	CC1
SMC04.T.CGAGAAGGTGTGACGA	CC1
SMC04.T.CGAGAAGTCGTTTGCC	CC1
SMC04.T.CGAGCACCACGAAACG	CC1
SMC04.T.CGATCGGCAGATCTGT	CC1
SMC04.T.CGATCGGGTTAAAGAC	CC2
SMC04.T.CGCCAAGAGACTGTAA	CC1
SMC04.T.CGCGGTATCAGGTTCA	CC1
SMC04.T.CGCTGGACAAGCCTAT	CC1
SMC04.T.CGGACACAGAGCTTCT	CC1
SMC04.T.CGGACACTCCGCATCT	CC1
SMC04.T.CGGACACTCGGATGTT	CC1
SMC04.T.CGGACGTTCAGGCAAG	CC1
SMC04.T.CGGCTAGAGATCCTGT	CC1
SMC04.T.CGGGTCACACAGGAGT	CC1
SMC04.T.CGGGTCAGTTGTGGCC	CC1
SMC04.T.CGGGTCATCACCTCGT	CC1
SMC04.T.CGTAGCGCATGCCCGA	CC1
SMC04.T.CGTAGCGGTCACTGGC	CC1
SMC04.T.CGTGTAAAGGAATGGA	CC1
SMC04.T.CGTGTAATCCGCGCAA	CC1
SMC04.T.CGTTGGGAGAAGAAGC	CC1
SMC04.T.CGTTGGGAGACTGTAA	CC1
SMC04.T.CTAACTTAGCCAGTAG	CC1
SMC04.T.CTAACTTGTCACACGC	CC1
SMC04.T.CTAATGGCAGGTGCCT	CC1
SMC04.T.CTACGTCCAGTCTTCC	CC1
SMC04.T.CTACGTCGTTTGTGTG	CC1

SMC04.T.CTAGTGAAGAATCTCC	CC1
SMC04.T.CTAGTGATCGCACTCT	CC1
SMC04.T.CTCATTACACTAAGTC	CC1
SMC04.T.CTCGGGACATTCCTCG	CC1
SMC04.T.CTCGTCATCAGAGACG	CC1
SMC04.T.CTCTACGGTCTAGAGG	CC1
SMC04.T.CTGAAACCAGCCTTGG	CC1
SMC04.T.CTGATAGGTTTGTTTC	CC1
SMC04.T.CTGCCTACACGCGAAA	CC1
SMC04.T.CTGCCTATCACAATGC	CC1
SMC04.T.CTGCGGACAATCTACG	CC1
SMC04.T.CTGCTGTTCACAGGCC	CC1
SMC04.T.CTGCTGTTCACTTACT	CC2
SMC04.T.CTGGTCTCAAAGAATC	CC1
SMC04.T.CTGTGCTCATATGAGA	CC1
SMC04.T.CTGTGCTGTGATAAAC	CC1
SMC04.T.CTGTTTATCGAGAACG	CC1
SMC04.T.CTTAACTTCTCCAGGG	CC1
SMC04.T.CTTACCGCACTTAACG	CC1
SMC04.T.CTTAGGAAGTAGGTGC	CC1
SMC04.T.CTTAGGACAAGGTTCT	CC1
SMC04.T.CTTTGCGAGCAACGGT	CC1
SMC04.T.CTTTGCGGTCATCGGC	CC1
SMC04.T.GAAACTCCACCTCGTT	CC1
SMC04.T.GAACGGAGTTTGGGCC	CC1
SMC04.T.GAATAAGAGGTGATAT	CC1
SMC04.T.GACAGAGCATGAAGTA	CC1
SMC04.T.GACAGAGGTGCACCAC	CC1
SMC04.T.GACCAATTCAGTTGAC	CC1
SMC04.T.GACCTGGTCAATCACG	CC1
SMC04.T.GACGTGCGTTCCTCCA	CC1
SMC04.T.GACGTGCGTTCGCTAA	CC1
SMC04.T.GACTGCGAGAAGAAGC	CC1
SMC04.T.GAGGTGACACCAGGTC	CC1
SMC04.T.GAGTCCGTCTCGCATC	CC1
SMC04.T.GATCGCGTCCAACCAA	CC1
SMC04.T.GATGAAAAGATCGGGT	CC1
SMC04.T.GATGAAACAAGTTCTG	CC1
SMC04.T.GATGCTAGTGGTCCGT	CC1
SMC04.T.GATGCTATCGCCCTTA	CC1
SMC04.T.GCAAACTCACGGCGTT	CC1
SMC04.T.GCAATCAGTAAGGGCT	CC1
SMC04.T.GCACTCTGTACAAGTA	CC1
SMC04.T.GCAGCCACACCGTTGG	CC1
SMC04.T.GCAGCCAGTGGCGAAT	CC1
SMC04.T.GCGAGAAAGCGATGAC	CC1
SMC04.T.GCGAGAAGTAGCGTCC	CC1
SMC04.T.GCGCAACAGAGTGACC	CC1
SMC04.T.GCGCAACTCAGGCAAG	CC1
SMC04.T.GCGCAGTCACAGACTT	CC1
SMC04.T.GCGCAGTCACCGTTGG	CC1
SMC04.T.GCGCAGTTCATCTGTT	CC1
SMC04.T.GCGCGATTCCTTGCCA	CC1
SMC04.T.GCGGGTTGTAGGCATG	CC2
SMC04.T.GCGGGTTTCACATGCA	CC1
SMC04.T.GCTGCAGAGAAACCTA	CC1
SMC04.T.GCTGGGTGTGCCTGTG	CC1
SMC04.T.GCTTCCACATCCTAGA	CC1

SMC04.T.GCTTGAATCAGTGCAT	CC1
SMC04.T.GGAAAGCAGATCCCGC	CC1
SMC04.T.GGAAAGCTCGGTCTAA	CC1
SMC04.T.GGAACTTTCTGGAGCC	CC1
SMC04.T.GGAATAAGTCGCGTGT	CC1
SMC04.T.GGACGTCCACAAGCCC	CC1
SMC04.T.GGACGTCGTTATTCTC	CC2
SMC04.T.GGACGTCTCCAAATGC	CC1
SMC04.T.GGACGTCTCGTCTGAA	CC1
SMC04.T.GGAGCAAGTTTGTGTG	CC1
SMC04.T.GGATGTTAGAGGGCTT	CC1
SMC04.T.GGCGACTTCATCACCC	CC1
SMC04.T.GGCGTGTTCTAAGCCA	CC1
SMC04.T.GGCTCGACAACGATCT	CC1
SMC04.T.GGCTCGACACAGGCCT	CC1
SMC04.T.GGGTCTGTCGCTTGTC	CC1
SMC04.T.GGTATTGGTACCGGCT	CC1
SMC04.T.GTAACGTCAGCCTTGG	CC2
SMC04.T.GTACTTTAGCAACGGT	CC1
SMC04.T.GTACTTTTCCAGATCA	CC1
SMC04.T.GTACTTTTCTTCCTTC	CC1
SMC04.T.GTAGGCCAGATCCCGC	CC1
SMC04.T.GTAGTCATCACTTATC	CC1
SMC04.T.GTATTCTCATTAGGCT	CC1
SMC04.T.GTCAAGTCAAGCTGAG	CC1
SMC04.T.GTCGGGTAGTACGCGA	CC1
SMC04.T.GTCGGGTGTACTTAGC	CC1
SMC04.T.GTCGTAATCCCATTAT	CC1
SMC04.T.GTCTCGTTCCTCAATT	CC1
SMC04.T.GTCTTCGGTTACCAGT	CC1
SMC04.T.GTCTTCGTCAAACCGT	CC1
SMC04.T.GTCTTCGTCCAGATCA	CC1
SMC04.T.GTGCAGCTCACAGTAC	CC1
SMC04.T.GTGCTTCCAAGTTAAG	CC1
SMC04.T.GTGTGCGGTCAGTGGA	CC1
SMC04.T.GTTACAGAGCACCGCT	CC1
SMC04.T.GTTACAGGTAGCTCCG	CC1
SMC04.T.GTTCATTAGAGAGCTC	CC1
SMC04.T.GTTCGGGCAGACGCAA	CC1
SMC04.T.TAAGAGAGTCAGTGGA	CC1
SMC04.T.TACACGACAACTGCGC	CC1
SMC04.T.TACACGATCAGTTTGG	CC1
SMC04.T.TACCTTAAGATCTGCT	CC1
SMC04.T.TACCTTATCCGCGCAA	CC1
SMC04.T.TACCTTATCGCGCCAA	CC1
SMC04.T.TACGGTACACACAGAG	CC1
SMC04.T.TACGGTAGTTAGGGTG	CC1
SMC04.T.TACTCGCAGGCAGTCA	CC2
SMC04.T.TAGACCACACCGAAAG	CC1
SMC04.T.TAGCCGGCATTAACCG	CC1
SMC04.T.TAGCCGGGTTACGACT	CC2
SMC04.T.TAGGCATGTAGCGCAA	CC1
SMC04.T.TATGCCCGTCTAGTCA	CC1
SMC04.T.TCAACGAGTAAGGGCT	CC1
SMC04.T.TCACAAGTCTAACTCT	CC1
SMC04.T.TCAGATGGTCGAACAG	CC1
SMC04.T.TCAGATGTCCGTAGTA	CC1
SMC04.T.TCAGCTCCACCACCAG	CC1

SMC04.T.TCAGCTCCAGTATCTG	CC1
SMC04.T.TCGAGGCTCATCGATG	CC2
SMC04.T.TCGAGGCTCGCTTGTC	CC1
SMC04.T.TCGCGAGAGCGCCTTG	CC1
SMC04.T.TCGCGAGGTTTGGCGC	CC1
SMC04.T.TCGCGTTGTACCATCA	CC1
SMC04.T.TCGCGTTTCCGTCAAA	CC1
SMC04.T.TCGTACCAGGACGAAA	CC1
SMC04.T.TCGTACCCATCACGTA	CC1
SMC04.T.TCGTAGATCCACGAAT	CC2
SMC04.T.TCTCATACAGAGCCAA	CC1
SMC04.T.TCTCATAGTGGTCCGT	CC1
SMC04.T.TCTTCGGAGAGCTATA	CC1
SMC04.T.TCTTCGGCATCCGCGA	CC1
SMC04.T.TGAGCATAGACCACGA	CC1
SMC04.T.TGATTTCTCCTCGCAT	CC1
SMC04.T.TGCACCTCAATGGACG	CC2
SMC04.T.TGCACCTTCAATAAGG	CC1
SMC04.T.TGCACCTTCTTTCCTC	CC1
SMC04.T.TGCCAAAAGGCGATAC	CC2
SMC04.T.TGCCAAATCCGAACGC	CC1
SMC04.T.TGCCCATTCGTCGTTC	CC1
SMC04.T.TGCGCAGTCGCTTGTC	CC1
SMC04.T.TGCGGGTCATCGATTG	CC1
SMC04.T.TGCGTGGGTCGGCACT	CC1
SMC04.T.TGCTACCGTGTGACGA	CC1
SMC04.T.TGCTGCTCATCATCCC	CC1
SMC04.T.TGGCCAGTCCTTTACA	CC1
SMC04.T.TGGCTGGCAGCTGTTA	CC1
SMC04.T.TGGGAAGAGGACAGCT	CC1
SMC04.T.TGGGCGTAGCGACGTA	CC1
SMC04.T.TGGTTAGAGTTCCACA	CC1
SMC04.T.TGTCCCAGTCAATGTC	CC2
SMC04.T.TGTCCCAGTGAGGGAG	CC1
SMC04.T.TGTGTTTAGATATACG	CC1
SMC04.T.TGTGTTTAGGTCGGAT	CC1
SMC04.T.TGTGTTTAGTGGTAAT	CC1
SMC04.T.TGTTCCGAGCTAGTTC	CC1
SMC04.T.TGTTCCGAGTAAGTAC	CC2
SMC04.T.TGTTCCGGTCGCGAAA	CC1
SMC04.T.TTAACTCTCAGTGTTG	CC1
SMC04.T.TTAACTCTCTAGAGTC	CC1
SMC04.T.TTATGCTAGAGACTTA	CC1
SMC04.T.TTCGAAGTCTGGTATG	CC1
SMC04.T.TTCGGTCAGCGTGAGT	CC1
SMC04.T.TTCGGTCGTCTCCATC	CC1
SMC04.T.TTCTCAACAATCCAAC	CC1
SMC04.T.TTGAACGGTATAAACG	CC1
SMC04.T.TTGAACGTCGTATCAG	CC1
SMC04.T.TTGACTTCAGGGTACA	CC1
SMC04.T.TTGTAGGCAATGTAAG	CC2
SMC04.T.TTTACTGAGCCAGAAC	CC1
SMC04.T.TTTACTGGTGCCTGGT	CC1
SMC04.T.TTTATGCAGCTCCCAG	CC1
SMC04.T.TTTATGCTCCACGTGG	CC1
SMC04.T.TTTCCTCGTGCTCTTC	CC1
SMC05.T.CCACTACCATTGTGCA	CC2
SMC05.T.CCGTACTAGTATGACA	CC2

SMC05.T.CCGTTCAAGTCGTACT	CC1
SMC05.T.CGATCGGTCCAACCAA	CC2
SMC05.T.CGTCAGGTCGCGCCAA	CC2
SMC05.T.CTTAACTAGAGGTACC	CC2
SMC05.T.GAGGTGAAGAGGTTAT	CC2
SMC05.T.GTCATTTGTTTACTCT	CC2
SMC05.T.GTCTCGTGTTACGGAG	CC2
SMC05.T.GTGTGCGGTAACGACG	CC2
SMC05.T.TCGCGTTTCCGCAGTG	CC1
SMC05.T.TCGTAGAGTGTGAAAT	CC2
SMC05.T.TCTATTGTCGACAGCC	CC2
SMC06.T.AAAGTAGGTTCCCTTG	CC1
SMC06.T.AACACGTAGCTAACTC	CC1
SMC06.T.AACTCTTGTGAGCGAT	CC1
SMC06.T.AATCGGTTCAATACCG	CC2
SMC06.T.ACACCAAAGGAACTGC	CC1
SMC06.T.ACAGCCGGTATTCTCT	CC2
SMC06.T.ACAGCTATCAAGAAGT	CC1
SMC06.T.ACATACGAGACCTAGG	CC2
SMC06.T.ACATCAGAGTGTGAAT	CC1
SMC06.T.ACCCACTCAGCCACCA	CC1
SMC06.T.ACGATACAGATCACGG	CC1
SMC06.T.ACGATACAGTAGGCCA	CC2
SMC06.T.ACGGGCTGTTAAGGGC	CC2
SMC06.T.ACGTCAAAGCGTGAAC	CC1
SMC06.T.ACTGAGTTCTCTAAGG	CC2
SMC06.T.ACTGATGAGTGTCCAT	CC2
SMC06.T.ACTGATGGTAAACGCG	CC1
SMC06.T.ACTTTCAGTCTCTTAT	CC2
SMC06.T.AGAATAGCACATCCGG	CC1
SMC06.T.AGAGCGATCGCCAGCA	CC1
SMC06.T.AGAGTGGAGCAGGTCA	CC2
SMC06.T.AGATTGCAGGAGTCTG	CC2
SMC06.T.AGGGTGAGTGCTGTAT	CC2
SMC06.T.AGGGTGAGTTCAGCGC	CC1
SMC06.T.AGGTCCGGTCTAGCCG	CC1
SMC06.T.AGTAGTCAGTCCTCCT	CC1
SMC06.T.AGTCTTTGTTAGAACA	CC2
SMC06.T.AGTCTTTTCCATTCTA	CC2
SMC06.T.ATAACGCTCTTAACCT	CC2
SMC06.T.ATAGACCAGCTCCTCT	CC1
SMC06.T.ATAGACCCAAGGTGTG	CC1
SMC06.T.ATCATGGAGTGCAAGC	CC1
SMC06.T.ATCCACCAGACAGACC	CC1
SMC06.T.ATCCACCAGATGGCGT	CC2
SMC06.T.ATCCACCGTTACGTCA	CC1
SMC06.T.ATCCGAAGTTACGTCA	CC1
SMC06.T.ATCGAGTGTAAGGGCT	CC1
SMC06.T.ATCGAGTTCAGTGTTG	CC1
SMC06.T.ATGAGGGAGTCCATAC	CC2
SMC06.T.ATGGGAGAGGTACTCT	CC2
SMC06.T.ATTGGTGTCTCGTTTA	CC1
SMC06.T.ATTTCTGTCATCTGCC	CC2
SMC06.T.CAACCTCGTAATCACC	CC1
SMC06.T.CACAAACCAAGCCATT	CC2
SMC06.T.CACACAATCCACTGGG	CC2
SMC06.T.CACACCTAGATCCGAG	CC2
SMC06.T.CACACTCCAGATGGCA	CC2

SMC06.T.CACACTCCAGGACCCT	CC1
SMC06.T.CACATTTGTCCAGTGC	CC2
SMC06.T.CACCTTGCAAGCCTAT	CC2
SMC06.T.CACTCCACAGTCTTCC	CC2
SMC06.T.CAGATCACAATGTAAG	CC2
SMC06.T.CAGCCGATCTCCCTGA	CC2
SMC06.T.CAGGTGCGTAGCTCCG	CC2
SMC06.T.CATATTCGTAAAGGAG	CC1
SMC06.T.CATCAAGCAGCTTCGG	CC1
SMC06.T.CATCAAGGTGGCTCCA	CC2
SMC06.T.CATCAGAAGTACTTGC	CC1
SMC06.T.CATCGAAAGGAATCGC	CC1
SMC06.T.CATGACATCAAGGCTT	CC1
SMC06.T.CATGCCTTCCCGACTT	CC1
SMC06.T.CATGGCGGTATCTGCA	CC1
SMC06.T.CATTATCCATGACGGA	CC2
SMC06.T.CCACGGAGTATAGGTA	CC1
SMC06.T.CCACTACTCCTCCTAG	CC1
SMC06.T.CCCATACAGGCTAGAC	CC2
SMC06.T.CCCTCCTGTCAAACTC	CC2
SMC06.T.CCGGTAGGTCCAGTGC	CC1
SMC06.T.CCTAAAGAGGCAGGTT	CC1
SMC06.T.CCTACCAAGGGCTCTC	CC1
SMC06.T.CCTTCCCCAAGCCGCT	CC1
SMC06.T.CCTTTCTTCTCTGTCG	CC2
SMC06.T.CGAATGTAGAGTAATC	CC1
SMC06.T.CGACCTTGTCGATTGT	CC1
SMC06.T.CGACCTTGTTGGTAAA	CC1
SMC06.T.CGAGAAGCAGTCTTCC	CC1
SMC06.T.CGAGCACTCCTTTCGG	CC2
SMC06.T.CGCCAAGCAGCGATCC	CC2
SMC06.T.CGCGGTATCATGTAGC	CC1
SMC06.T.CGGCTAGCACACATGT	CC2
SMC06.T.CGTCAGGGTCGAATCT	CC2
SMC06.T.CGTCCATCACCATCCT	CC2
SMC06.T.CGTGTCTCATGCCACG	CC2
SMC06.T.CGTTAGACATGTAAGA	CC2
SMC06.T.CGTTCTGGTACACCGC	CC2
SMC06.T.CGTTGGGCAGATTGCT	CC2
SMC06.T.CTACACCTCTCCCTGA	CC2
SMC06.T.CTCATTAAGAGGTACC	CC2
SMC06.T.CTCTAATCAATGCCAT	CC2
SMC06.T.CTCTAATCACGCTTTC	CC1
SMC06.T.CTCTAATTCCGCTGTT	CC2
SMC06.T.CTGAAACGTCGAACAG	CC1
SMC06.T.CTGAAGTGTTTGCATG	CC1
SMC06.T.CTGATCCGTGTTAAGA	CC1
SMC06.T.CTGCTGTTCAACCATG	CC2
SMC06.T.CTGCTGTTCATACGGT	CC1
SMC06.T.CTGCTGTTCTGGCGAC	CC1
SMC06.T.CTGTGCTAGGATGCGT	CC2
SMC06.T.CTTACCGAGTGATCGG	CC2
SMC06.T.CTTAGGAGTCAAGCGA	CC1
SMC06.T.CTTCTCTAGTTCGATC	CC2
SMC06.T.CTTGGCTCACGGCCAT	CC2
SMC06.T.CTTTGCGGTAGCGCAA	CC2
SMC06.T.GACACGCTCTTTCCTC	CC1
SMC06.T.GACAGAGGTGTGGTTT	CC2

SMC06.T.GACCTGGCAGCTGTTA	CC2
SMC06.T.GACGCGTCAGAAGCAC	CC2
SMC06.T.GACGTGCAGTACGTAA	CC2
SMC06.T.GACTACAAGCGATAGC	CC2
SMC06.T.GAGCAGACATGCTGGC	CC2
SMC06.T.GATCAGTGTCTTGCGG	CC1
SMC06.T.GATCTAGGTAGCGCAA	CC1
SMC06.T.GATGAGGGTGATGTGG	CC2
SMC06.T.GATGAGGTCCAGGGCT	CC1
SMC06.T.GATTCAGGTATATGAG	CC2
SMC06.T.GCCAAATAGAACTGTA	CC2
SMC06.T.GCTGCAGCAGCCTGTG	CC1
SMC06.T.GCTGCGAAGACAAAGG	CC2
SMC06.T.GCTGCTTGTTGGTAAA	CC1
SMC06.T.GCTTCCACATAGACTC	CC2
SMC06.T.GGACGTCCATCTGGTA	CC2
SMC06.T.GGAGCAAGTATTCTCT	CC1
SMC06.T.GGATGTTCACATCCAA	CC1
SMC06.T.GGCCGATAGTGGTAGC	CC2
SMC06.T.GGCGACTAGAGCCCAA	CC2
SMC06.T.GGCGACTCAGCCTTTC	CC2
SMC06.T.GGGCATCGTAAACACA	CC1
SMC06.T.GGGTCTGAGAGGTTAT	CC2
SMC06.T.GTAACGTCAATGAAAC	CC2
SMC06.T.GTACTCCAGAAACCTA	CC1
SMC06.T.GTACTCCAGTTACCCA	CC2
SMC06.T.GTAGTCATCGGTCTAA	CC2
SMC06.T.GTCCTCACAACGCACC	CC1
SMC06.T.GTCTCGTAGACTAGGC	CC2
SMC06.T.GTCTCGTTCGCACTCT	CC1
SMC06.T.GTGCAGCAGCCCGAAA	CC1
SMC06.T.GTGCATAGTCTGATCA	CC1
SMC06.T.GTGCTTCTCACCAGGC	CC1
SMC06.T.GTGTTAGGTAGCTTGT	CC1
SMC06.T.GTTAAGCAGCTAACAA	CC2
SMC06.T.GTTCTCGGTATTACCG	CC1
SMC06.T.TAAACCGCATACTACG	CC2
SMC06.T.TAAGAGACACGAAGCA	CC2
SMC06.T.TAAGCGTGTTGACGTT	CC1
SMC06.T.TACCTTACACGGCGTT	CC2
SMC06.T.TACTCGCAGGCTAGAC	CC2
SMC06.T.TACTTACAGCGGCTTC	CC1
SMC06.T.TAGTTGGTCTTCATGT	CC1
SMC06.T.TATCTCACAAGCCGCT	CC1
SMC06.T.TCAACGAGTAAAGTCA	CC1
SMC06.T.TCACGAAAGACAGACC	CC2
SMC06.T.TCAGATGCACTCAGGC	CC1
SMC06.T.TCAGGTAAGAATTGTG	CC2
SMC06.T.TCAGGTAAGTGGAGTC	CC1
SMC06.T.TCAGGTACATTACGAC	CC1
SMC06.T.TCATTACTCTTGAGAC	CC2
SMC06.T.TCATTTGCAGGGCATA	CC2
SMC06.T.TCCCGATAGCCAGTAG	CC2
SMC06.T.TCGCGAGAGTTTGCGT	CC1
SMC06.T.TCTCTAAAGATCACGG	CC2
SMC06.T.TCTGGAACAAGTAGTA	CC1
SMC06.T.TCTGGAAGTCCGTGAC	CC2
SMC06.T.TCTGGAAGTGACTCAT	CC1

SMC06.T.TCTGGAAGTTATCGGT	CC1
SMC06.T.TGACTAGAGCTGCCCA	CC2
SMC06.T.TGACTTTTCCGAATGT	CC1
SMC06.T.TGAGAGGTCGACGGAA	CC2
SMC06.T.TGAGGGAGTCGAGATG	CC1
SMC06.T.TGAGGGATCTAACTCT	CC1
SMC06.T.TGAGGGATCTTCGAGA	CC2
SMC06.T.TGCCCTACACCCATTC	CC2
SMC06.T.TGCCCTAGTCATGCAT	CC1
SMC06.T.TGCGTGGCAGGTGGAT	CC1
SMC06.T.TGGACGCGTGTGGCTC	CC2
SMC06.T.TGGCGCACATCTACGA	CC2
SMC06.T.TGTCCCATCGCAGGCT	CC2
SMC06.T.TGTGGTAGTCATATGC	CC1
SMC06.T.TTAACTCCATGAAGTA	CC1
SMC06.T.TTAGGACTCGACAGCC	CC1
SMC06.T.TTAGGCAGTTGGGACA	CC1
SMC06.T.TTAGTTCAGGCTAGAC	CC2
SMC06.T.TTATGCTTCCAGTATG	CC1
SMC06.T.TTCTCAACAAGGACTG	CC1
SMC06.T.TTGCCGTAGGCGACAT	CC1
SMC06.T.TTGCCGTGTTAGATGA	CC1
SMC06.T.TTGCGTCAGGGCACTA	CC2
SMC06.T.TTGGAACCAGCCTATA	CC1
SMC06.T.TTGGAACCATAGGATA	CC2
SMC06.T.TTGGAACCATTCACTT	CC1
SMC07.T.AAAGATGCATTCCTGC	CC1
SMC07.T.AAATGCCGTGGTCCGT	CC2
SMC07.T.AAATGCCTCCCTTGCA	CC1
SMC07.T.AAATGCCTCTGGAGCC	CC1
SMC07.T.AACACGTAGGAACTGC	CC1
SMC07.T.AACACGTGTCTGATTG	CC1
SMC07.T.AACCATGAGCGTCAAG	CC1
SMC07.T.AACCGCGAGCCGATTT	CC1
SMC07.T.AACGTTGCACGGTAAG	CC1
SMC07.T.AACTCAGGTGTCGCTG	CC1
SMC07.T.AACTCAGTCACCCTCA	CC1
SMC07.T.AACTCTTGTGGTCCGT	CC2
SMC07.T.AACTTTCTCGCAAGCC	CC1
SMC07.T.AAGACCTCATCTCCCA	CC1
SMC07.T.AAGCCGCCAGCTGCTG	CC1
SMC07.T.AAGCCGCGTGTTTGGT	CC1
SMC07.T.AAGGAGCGTTGGGACA	CC1
SMC07.T.AAGGTTCAGTCCCACG	CC1
SMC07.T.AAGGTTCCAATCACAC	CC1
SMC07.T.AAGGTTCGTCGCTTCT	CC1
SMC07.T.AATCCAGGTGGACGAT	CC1
SMC07.T.ACACCCTGTCAATACC	CC1
SMC07.T.ACAGCTATCAGCTCGG	CC1
SMC07.T.ACATCAGGTCGAGATG	CC1
SMC07.T.ACCAGTAGTCCCTACT	CC1
SMC07.T.ACCTTTAAGCGCCTCA	CC1
SMC07.T.ACGAGCCCACGCTTTC	CC1
SMC07.T.ACGATGTCAAAGGTGC	CC1
SMC07.T.ACGCAGCCATGGATGG	CC1
SMC07.T.ACGCCAGAGTGGTCCC	CC1
SMC07.T.ACGCCAGTCGCTAGCG	CC1
SMC07.T.ACGCCGAAGCTAGGCA	CC1

SMC07.T.ACGGGCTCACCCTATC	CC1
SMC07.T.ACGTCAAGTGCAGGTA	CC2
SMC07.T.ACTATCTCAAGAAGAG	CC1
SMC07.T.ACTGAACAGGCTAGCA	CC1
SMC07.T.ACTGAACCACAGGAGT	CC1
SMC07.T.ACTGAACGTCGCATCG	CC1
SMC07.T.ACTGATGAGTCCGTAT	CC1
SMC07.T.ACTGTCCAGCCCAGCT	CC1
SMC07.T.ACTGTCCCAGCGAACA	CC2
SMC07.T.ACTGTCCGTGATGATA	CC2
SMC07.T.ACTGTCCGTGTGGTTT	CC1
SMC07.T.ACTTACTTCTCTAGGA	CC1
SMC07.T.AGAATAGAGGGTCGAT	CC1
SMC07.T.AGAGTGGTCTTATCTG	CC1
SMC07.T.AGATCTGAGTTGTCGT	CC1
SMC07.T.AGATCTGCAGTCCTTC	CC1
SMC07.T.AGATTGCAGCACACAG	CC1
SMC07.T.AGCAGCCTCAAGCCTA	CC1
SMC07.T.AGCCTAAGTCGGATCC	CC1
SMC07.T.AGCCTAATCCTGCAGG	CC1
SMC07.T.AGCGGTCCAAGGTTTC	CC1
SMC07.T.AGCGTATAGTTGTAGA	CC1
SMC07.T.AGCGTATCATTCACTT	CC1
SMC07.T.AGCGTCGTCTACTTAC	CC1
SMC07.T.AGCTCCTAGTACGACG	CC1
SMC07.T.AGCTCCTAGTGTACGG	CC1
SMC07.T.AGCTCTCCAGTAGAGC	CC1
SMC07.T.AGGCCACGTGATGTGG	CC1
SMC07.T.AGGCCGTAGAGTGACC	CC1
SMC07.T.AGGCCGTCACACCGCA	CC1
SMC07.T.AGGCCGTTCGGTGTTA	CC1
SMC07.T.AGGTCATTCACATAGC	CC1
SMC07.T.AGGTCATTCGCGATCG	CC1
SMC07.T.AGTAGTCCAGGGCATA	CC1
SMC07.T.AGTCTTTAGCGATTCT	CC1
SMC07.T.AGTCTTTGTAGCACGA	CC1
SMC07.T.AGTGAGGTCAACTCTT	CC1
SMC07.T.AGTGGGAAGCTACCGC	CC1
SMC07.T.AGTTGGTCATGAAGTA	CC1
SMC07.T.ATAGACCTCTGATACG	CC1
SMC07.T.ATCATGGAGTGTACGG	CC1
SMC07.T.ATCATGGGTCTGGTCG	CC1
SMC07.T.ATCCGAATCAGCAACT	CC1
SMC07.T.ATCTACTCATGCCACG	CC1
SMC07.T.ATCTACTGTTAAGATG	CC1
SMC07.T.ATGAGGGGTATAAACG	CC1
SMC07.T.ATGCGATTCCACGAAT	CC1
SMC07.T.ATGGGAGGTGACTACT	CC1
SMC07.T.ATGGGAGTCCTGCAGG	CC1
SMC07.T.ATGGGAGTCTGCCCTA	CC1
SMC07.T.ATGTGTGAGATCCCGC	CC1
SMC07.T.ATGTGTGAGATCCGAG	CC1
SMC07.T.ATGTGTGTCGGCCGAT	CC1
SMC07.T.ATTCTACCAAGAAAGG	CC1
SMC07.T.ATTCTACCACGCGAAA	CC1
SMC07.T.ATTGGACAGCGCCTCA	CC1
SMC07.T.ATTGGACTCTGCAGTA	CC1
SMC07.T.ATTTCTGGTAGCAAAT	CC1

SMC07.T.CAACCAAAGTACGACG	CC1
SMC07.T.CAAGAAATCCCATTAT	CC1
SMC07.T.CAAGGCCGTCTCGTTC	CC1
SMC07.T.CAAGGCCTCGAACGGA	CC1
SMC07.T.CAAGTTGGTTTGTGTG	CC1
SMC07.T.CACAAACAGACGCACA	CC1
SMC07.T.CACAAACCACTTCTGC	CC1
SMC07.T.CACAAACTCTAACTGG	CC1
SMC07.T.CACACAACATTAGGCT	CC1
SMC07.T.CACACCTAGCTAAACA	CC1
SMC07.T.CACACCTGTAATCGTC	CC1
SMC07.T.CACACCTGTCAGAGGT	CC1
SMC07.T.CACACTCGTTTCGCTC	CC1
SMC07.T.CACACTCTCTTTACGT	CC2
SMC07.T.CACAGGCGTATATGGA	CC1
SMC07.T.CACAGGCGTTCGAATC	CC1
SMC07.T.CACAGTAAGCGTGAAC	CC1
SMC07.T.CACATAGTCCATTCTA	CC1
SMC07.T.CACATAGTCCTAAGTG	CC1
SMC07.T.CACATTTTCCTAGGGC	CC1
SMC07.T.CACCACTAGCTGTTCA	CC1
SMC07.T.CACCAGGAGCGTGTCC	CC1
SMC07.T.CACCAGGGTGACTCAT	CC1
SMC07.T.CACCAGGTCCAGGGCT	CC1
SMC07.T.CACTCCATCTCACATT	CC1
SMC07.T.CAGAATCGTAGCGCAA	CC1
SMC07.T.CAGAATCGTCACTTCC	CC1
SMC07.T.CAGAATCGTGCAACGA	CC1
SMC07.T.CAGAGAGTCTGTTGAG	CC1
SMC07.T.CAGATCAAGTCGTTTG	CC1
SMC07.T.CAGATCACACCTTGTC	CC1
SMC07.T.CAGATCAGTAAAGTCA	CC1
SMC07.T.CAGATCAGTCTCCCTA	CC2
SMC07.T.CAGATCAGTGCACTTA	CC1
SMC07.T.CAGCAGCGTACCGGCT	CC1
SMC07.T.CAGCATATCATCGATG	CC2
SMC07.T.CAGCCGAAGTAGATGT	CC1
SMC07.T.CAGCTAACATCTCGCT	CC1
SMC07.T.CAGCTAATCACCATAG	CC2
SMC07.T.CAGCTGGTCCGCAGTG	CC1
SMC07.T.CAGGTGCCACATAACC	CC1
SMC07.T.CAGGTGCGTTCGTGAT	CC1
SMC07.T.CAGTAACTCAAGGCTT	CC1
SMC07.T.CAGTAACTCTGGAGCC	CC1
SMC07.T.CAGTCCTCATATACGC	CC1
SMC07.T.CATATTCGTCAACTGT	CC1
SMC07.T.CATATTCTCGGTTAAC	CC1
SMC07.T.CATATTCTCTAACCGA	CC1
SMC07.T.CATCAAGGTTACGTCA	CC1
SMC07.T.CATCCACAGATTACCC	CC1
SMC07.T.CATCGAAAGCCACGCT	CC1
SMC07.T.CATCGGGAGCCCAGCT	CC1
SMC07.T.CATGACACAGTCGTGC	CC1
SMC07.T.CATGCCTTCTATCGCC	CC1
SMC07.T.CATTATCGTGGAAAGA	CC1
SMC07.T.CATTCGCCAGATCTGT	CC1
SMC07.T.CATTCGCGTCCGAATT	CC2
SMC07.T.CCAATCCAGGTCGGAT	CC1

SMC07.T.CCACGGACAAAGAATC	CC2
SMC07.T.CCACGGAGTTTAAGCC	CC1
SMC07.T.CCACGGATCTCCGGTT	CC1
SMC07.T.CCACTACCAAGCTGAG	CC1
SMC07.T.CCAGCGATCTACTTAC	CC1
SMC07.T.CCATGTCGTGTGAATA	CC1
SMC07.T.CCATTCGAGCAGGTCA	CC1
SMC07.T.CCCAATCGTAGCTTGT	CC1
SMC07.T.CCCAGTTAGATCTGCT	CC1
SMC07.T.CCCAGTTTCCTTCAAT	CC1
SMC07.T.CCCATACTCTTGTACT	CC1
SMC07.T.CCCTCCTGTCTTGCGG	CC1
SMC07.T.CCGGTAGAGGGCTTCC	CC1
SMC07.T.CCGTGGAGTCACCTAA	CC1
SMC07.T.CCGTGGATCATTGCCC	CC1
SMC07.T.CCGTTCATCAAGGTAA	CC1
SMC07.T.CCTAAAGAGCCACGCT	CC1
SMC07.T.CCTACACGTCTAAAGA	CC1
SMC07.T.CCTACCACAAACTGCT	CC1
SMC07.T.CCTACCAGTTCGTGAT	CC1
SMC07.T.CCTAGCTAGGACCACA	CC1
SMC07.T.CCTAGCTGTGGCCCTA	CC1
SMC07.T.CCTATTATCATCTGCC	CC1
SMC07.T.CCTTCCCAGAGTACAT	CC1
SMC07.T.CCTTCCCTCTTGAGAC	CC1
SMC07.T.CCTTCGATCGGAGGTA	CC1
SMC07.T.CCTTTCTTCACATACG	CC1
SMC07.T.CGAACATGTCTAGTCA	CC1
SMC07.T.CGAACATGTTCGGGCT	CC1
SMC07.T.CGACTTCAGTAGATGT	CC1
SMC07.T.CGAGAAGCACCAGGCT	CC1
SMC07.T.CGAGAAGTCTTCGAGA	CC1
SMC07.T.CGAGCACTCAAAGACA	CC1
SMC07.T.CGAGCCAGTCGGATCC	CC1
SMC07.T.CGATCGGAGTCTTGCA	CC1
SMC07.T.CGATCGGCATGGTCTA	CC1
SMC07.T.CGATGGCAGACAGGCT	CC1
SMC07.T.CGATGGCCATTGCGGC	CC1
SMC07.T.CGATGGCGTGAGCGAT	CC1
SMC07.T.CGATGTAAGCACGCCT	CC1
SMC07.T.CGATGTAGTGGGTATG	CC1
SMC07.T.CGCCAAGTCTCAAACG	CC1
SMC07.T.CGCGGTACAAGCCGTC	CC1
SMC07.T.CGCGGTACATTGGTAC	CC1
SMC07.T.CGCTATCGTTAAAGAC	CC1
SMC07.T.CGCTATCTCCCATTAT	CC1
SMC07.T.CGCTTCACATGATCCA	CC1
SMC07.T.CGCTTCATCTTGCAAG	CC2
SMC07.T.CGGACGTAGAGGTACC	CC1
SMC07.T.CGGACGTTCTTAGAGC	CC1
SMC07.T.CGGAGCTGTAAACCTC	CC2
SMC07.T.CGGAGTCCAGCCTATA	CC1
SMC07.T.CGGAGTCGTACACCGC	CC1
SMC07.T.CGGCTAGAGTGTGGCA	CC1
SMC07.T.CGGCTAGTCTACTTAC	CC2
SMC07.T.CGGGTCAGTAATCACC	CC1
SMC07.T.CGGTTAACAGGTGCCT	CC1
SMC07.T.CGGTTAAGTTACGTCA	CC1

SMC07.T.CGGTTAATCAAACCGT	CC1
SMC07.T.CGTCACTGTATTCTCT	CC1
SMC07.T.CGTCACTTCGATCCCT	CC2
SMC07.T.CGTCAGGCAGCCTGTG	CC1
SMC07.T.CGTCTACTCACATAGC	CC1
SMC07.T.CGTGAGCAGGACACCA	CC1
SMC07.T.CGTGTAAGTTTAGCTG	CC1
SMC07.T.CGTGTCTCATCTGGTA	CC1
SMC07.T.CGTTAGAAGTACGACG	CC1
SMC07.T.CTAACTTTCTAACCGA	CC1
SMC07.T.CTAAGACTCTCGAGTA	CC1
SMC07.T.CTAATGGGTCGGGTCT	CC1
SMC07.T.CTACACCAGAGTCGGT	CC1
SMC07.T.CTACACCAGCAAATCA	CC2
SMC07.T.CTACACCAGCCGGTAA	CC1
SMC07.T.CTACATTGTACTCGCG	CC1
SMC07.T.CTACCCATCGATGAGG	CC1
SMC07.T.CTACGTCTCAGTGTTG	CC1
SMC07.T.CTACGTCTCGCAGGCT	CC2
SMC07.T.CTAGAGTGTCTACCTC	CC1
SMC07.T.CTAGCCTTCTGAGGGA	CC1
SMC07.T.CTCACACAGCTAGTTC	CC1
SMC07.T.CTCACACTCAGCGACC	CC1
SMC07.T.CTCAGAAAGCGAAGGG	CC1
SMC07.T.CTCAGAACACATTCGA	CC1
SMC07.T.CTCATTAGTCACACGC	CC2
SMC07.T.CTCATTATCCTGCCAT	CC1
SMC07.T.CTCGAAAAGCTGAACG	CC1
SMC07.T.CTCGAAAGTATGAAAC	CC1
SMC07.T.CTCGAAATCAAGGCTT	CC1
SMC07.T.CTCGGAGAGCCATCGC	CC1
SMC07.T.CTCGGAGCATTCGACA	CC1
SMC07.T.CTCGGGAGTCACTGGC	CC1
SMC07.T.CTCGGGATCGGCGCAT	CC1
SMC07.T.CTCGTACTCGTTTATC	CC1
SMC07.T.CTCTAATGTTATGCGT	CC1
SMC07.T.CTCTACGCATGGTCTA	CC1
SMC07.T.CTCTGGTCAATGGTCT	CC1
SMC07.T.CTGAAACCAAGGGTCA	CC1
SMC07.T.CTGATAGGTTGTGGCC	CC1
SMC07.T.CTGATCCCAACTGCGC	CC1
SMC07.T.CTGATCCGTCCGTGAC	CC1
SMC07.T.CTGATCCTCTTCGGTC	CC1
SMC07.T.CTGCTGTGTCCATGAT	CC1
SMC07.T.CTGCTGTGTTGTCGCG	CC1
SMC07.T.CTGTTTACACATTTCT	CC1
SMC07.T.CTTAACTCAGCCAGAA	CC1
SMC07.T.CTTAACTCATAGGATA	CC1
SMC07.T.CTTAACTGTATTCGTG	CC1
SMC07.T.CTTAACTGTTTGACTG	CC1
SMC07.T.CTTCTCTTCGTAGGTT	CC1
SMC07.T.CTTGGCTAGTGGAGTC	CC1
SMC07.T.CTTGGCTGTCATCCCT	CC1
SMC07.T.CTTGGCTTCATCACCC	CC1
SMC07.T.CTTGGCTTCGATCCCT	CC1
SMC07.T.CTTTGCGTCTTACCTA	CC1
SMC07.T.CTTTGCGTCTTAGAGC	CC1
SMC07.T.GAACATCGTATTCGTG	CC1

SMC07.T.GAACATCGTTTGACTG	CC1
SMC07.T.GAACCTAAGCACACAG	CC1
SMC07.T.GAAGCAGAGAAACCGC	CC1
SMC07.T.GAAGCAGCAACAACCT	CC1
SMC07.T.GAAGCAGCAGAAGCAC	CC1
SMC07.T.GAATGAAAGTGTACGG	CC1
SMC07.T.GAATGAACAGACGTAG	CC1
SMC07.T.GAATGAACATCGATGT	CC1
SMC07.T.GAATGAACATGGTTGT	CC1
SMC07.T.GAATGAAGTTGCGTTA	CC1
SMC07.T.GACACGCAGTTAGGTA	CC1
SMC07.T.GACACGCGTTCCCGAG	CC1
SMC07.T.GACCAATCAGGGTTAG	CC1
SMC07.T.GACCTGGGTTCCTCCA	CC1
SMC07.T.GACGCGTGTCGCGTGT	CC1
SMC07.T.GACGCGTGTCGTTGTA	CC1
SMC07.T.GACGCGTGTGTTGAGG	CC1
SMC07.T.GACGTGCCAGCGATCC	CC1
SMC07.T.GACGTTACAATAGCAA	CC1
SMC07.T.GACTACATCCTAGAAC	CC1
SMC07.T.GAGCAGACACCATCCT	CC1
SMC07.T.GAGCAGAGTCCTCTTG	CC1
SMC07.T.GAGGTGACAAGTTGTC	CC1
SMC07.T.GAGTCCGAGAGTACAT	CC1
SMC07.T.GAGTCCGGTTTACTCT	CC1
SMC07.T.GATCAGTAGTCCCACG	CC1
SMC07.T.GATCAGTTCTTCAACT	CC1
SMC07.T.GATCGATAGATGTTAG	CC1
SMC07.T.GATCGATAGGGTCGAT	CC1
SMC07.T.GATCGCGAGCAACGGT	CC1
SMC07.T.GATCGCGAGTGTCCCG	CC1
SMC07.T.GATCGCGGTCCGCTGA	CC1
SMC07.T.GATCTAGAGAGTCTGG	CC1
SMC07.T.GATCTAGGTGAAAGAG	CC1
SMC07.T.GATGAAAAGGCTCTTA	CC2
SMC07.T.GATGAAACACTCAGGC	CC1
SMC07.T.GATGAAATCAACCATG	CC2
SMC07.T.GATGAAATCACTGGGC	CC1
SMC07.T.GATGAGGAGACAGACC	CC1
SMC07.T.GATGCTAGTACCCAAT	CC1
SMC07.T.GCACATAAGCAGGCTA	CC1
SMC07.T.GCACATAGTCATTAGC	CC1
SMC07.T.GCACATAGTCGCTTTC	CC1
SMC07.T.GCACATATCTAACCGA	CC1
SMC07.T.GCACTCTTCCCGGATG	CC1
SMC07.T.GCAGCCAGTCAACTGT	CC1
SMC07.T.GCAGCCATCAGTGCAT	CC1
SMC07.T.GCAGTTACACCAGTTA	CC1
SMC07.T.GCATACAAGACGCAAC	CC1
SMC07.T.GCATACACACGCGAAA	CC1
SMC07.T.GCATGCGAGTTACCCA	CC1
SMC07.T.GCCTCTATCCGTCATC	CC1
SMC07.T.GCGACCAAGTCTCCTC	CC1
SMC07.T.GCGACCATCGTGGTCG	CC1
SMC07.T.GCGAGAACAAGCCATT	CC1
SMC07.T.GCGAGAACAGACAAAT	CC1
SMC07.T.GCGCAACAGTGGTAGC	CC1
SMC07.T.GCGCAACGTAGCTTGT	CC2

SMC07.T.GCGCAACGTTCAGACT	CC1
SMC07.T.GCGCAGTAGAAGCCCA	CC1
SMC07.T.GCGCAGTAGCAGCCTC	CC1
SMC07.T.GCGCCAATCTCGATGA	CC1
SMC07.T.GCGCCAATCTGCAGTA	CC1
SMC07.T.GCTCCTAAGCGTCAAG	CC1
SMC07.T.GCTCTGTAGTGCGATG	CC1
SMC07.T.GCTGCGACAAGCGCTC	CC1
SMC07.T.GCTGCGACAGGTCGTC	CC1
SMC07.T.GCTGCGAGTTTCGCTC	CC1
SMC07.T.GCTGCTTAGCTGAACG	CC1
SMC07.T.GCTGGGTGTCTAGAGG	CC1
SMC07.T.GCTGGGTGTTCCGGCA	CC1
SMC07.T.GCTTCCATCGGACAAG	CC1
SMC07.T.GCTTGAACAGACGCCT	CC1
SMC07.T.GCTTGAATCTTCAACT	CC1
SMC07.T.GGAAAGCTCATCGGAT	CC1
SMC07.T.GGAATAAAGCCGGTAA	CC1
SMC07.T.GGAATAAGTTCTGTTT	CC1
SMC07.T.GGACATTTCCGGCACA	CC1
SMC07.T.GGACGTCCAGACGCCT	CC1
SMC07.T.GGATGTTAGGAATGGA	CC1
SMC07.T.GGATTACAGAGCTGCA	CC1
SMC07.T.GGATTACGTCGTTGTA	CC1
SMC07.T.GGATTACGTTTAGGAA	CC1
SMC07.T.GGCCGATAGAAGGTTT	CC1
SMC07.T.GGCGACTCACCTCGTT	CC2
SMC07.T.GGCTCGACATACAGCT	CC1
SMC07.T.GGCTCGATCGGCATCG	CC1
SMC07.T.GGGAATGAGCGTTTAC	CC1
SMC07.T.GGGAATGAGGTGATTA	CC1
SMC07.T.GGGACCTCAATAACGA	CC1
SMC07.T.GGGAGATGTCTGATTG	CC1
SMC07.T.GGGAGATTCTTACCGC	CC1
SMC07.T.GGGCACTAGCCCAACC	CC1
SMC07.T.GGGCACTCAGCTCGCA	CC1
SMC07.T.GGGCACTTCAACACTG	CC1
SMC07.T.GGGCACTTCGTCCAGG	CC1
SMC07.T.GGGCACTTCTTTACGT	CC1
SMC07.T.GGGCATCAGTATCTCG	CC1
SMC07.T.GGGCATCCATCCGCGA	CC1
SMC07.T.GGGTTGCAGCTAACAA	CC1
SMC07.T.GGGTTGCAGGATATAC	CC1
SMC07.T.GGGTTGCAGTTACCCA	CC1
SMC07.T.GGTATTGAGTCTCCTC	CC1
SMC07.T.GGTGAAGAGAATTGTG	CC2
SMC07.T.GGTGAAGTCGGCCGAT	CC1
SMC07.T.GGTGCGTGTACAAGTA	CC1
SMC07.T.GGTGTTAAGACACTAA	CC1
SMC07.T.GGTGTTAAGGGAAACA	CC1
SMC07.T.GGTGTTACATGCAATC	CC1
SMC07.T.GGTGTTAGTGTTGGGA	CC1
SMC07.T.GTAACGTCATATGAGA	CC1
SMC07.T.GTACGTAGTGTGAAAT	CC1
SMC07.T.GTAGGCCAGGTGATAT	CC1
SMC07.T.GTAGGCCAGTACTTGC	CC1
SMC07.T.GTAGGCCCATTCTTAC	CC1
SMC07.T.GTAGTCAGTTTAAGCC	CC2

SMC07.T.GTAGTCATCGCCAAAT	CC1
SMC07.T.GTATCTTGTTTGTTGG	CC1
SMC07.T.GTATTCTAGCGTGAGT	CC1
SMC07.T.GTATTCTAGTTCCACA	CC1
SMC07.T.GTCAAGTGTTGTCGCG	CC1
SMC07.T.GTCACAAGTCAAAGAT	CC1
SMC07.T.GTCATTTAGATCCCGC	CC1
SMC07.T.GTCATTTTCTCGTTTA	CC1
SMC07.T.GTCGTAACAGGTCCAC	CC1
SMC07.T.GTCGTAAGTACGAAAT	CC1
SMC07.T.GTCGTAATCACAGGCC	CC1
SMC07.T.GTCTTCGCAAGCGATG	CC1
SMC07.T.GTGAAGGGTGGTCCGT	CC1
SMC07.T.GTGCAGCGTTATTCTC	CC1
SMC07.T.GTGCAGCTCTTGTCAT	CC1
SMC07.T.GTGCATACAATCTACG	CC1
SMC07.T.GTGCATAGTTAGGGTG	CC1
SMC07.T.GTGCATATCAGATAAG	CC1
SMC07.T.GTGCATATCCAAGCCG	CC1
SMC07.T.GTGCATATCGGATGTT	CC1
SMC07.T.GTGCTTCAGAGTACCG	CC1
SMC07.T.GTGCTTCAGATCGATA	CC1
SMC07.T.GTGCTTCAGTTAGCGG	CC1
SMC07.T.GTGCTTCTCGCATGGC	CC1
SMC07.T.GTGGGTCGTGTAACGG	CC1
SMC07.T.GTGTTAGTCGGCCGAT	CC1
SMC07.T.GTGTTAGTCGTTTGCC	CC1
SMC07.T.GTTACAGGTCCAGTAT	CC1
SMC07.T.GTTCATTGTCAGCTAT	CC1
SMC07.T.GTTCATTTCAATCTCT	CC1
SMC07.T.GTTCATTTCTCTAGGA	CC2
SMC07.T.GTTCGGGAGGAACTGC	CC1
SMC07.T.GTTCGGGCAAAGGCGT	CC2
SMC07.T.GTTCGGGGTGTGACGA	CC1
SMC07.T.GTTTCTAAGCTAGGCA	CC1
SMC07.T.GTTTCTACAGCTGGCT	CC1
SMC07.T.GTTTCTACATCCTTGC	CC1
SMC07.T.GTTTCTAGTGTCAATC	CC1
SMC07.T.TAAACCGCAGGATCGA	CC1
SMC07.T.TAAACCGTCAGGCCCA	CC2
SMC07.T.TAAGAGAAGGTAGCCA	CC1
SMC07.T.TAAGCGTGTTACGACT	CC1
SMC07.T.TACAGTGAGCCGTCGT	CC1
SMC07.T.TACAGTGAGTCTCAAC	CC1
SMC07.T.TACAGTGAGTGTCCCG	CC1
SMC07.T.TACCTATAGATCTGAA	CC1
SMC07.T.TACCTATAGGTGATTA	CC2
SMC07.T.TACGGATGTCGAACAG	CC1
SMC07.T.TACGGATTCGTTTATC	CC1
SMC07.T.TACTCATCAAGTACCT	CC1
SMC07.T.TACTCATGTACCGCTG	CC2
SMC07.T.TACTCATTCTTCGGTC	CC2
SMC07.T.TACTCGCAGTAGCGGT	CC1
SMC07.T.TACTTACAGGACAGCT	CC1
SMC07.T.TACTTACGTGTGACGA	CC1
SMC07.T.TACTTGTAGCACGCCT	CC1
SMC07.T.TAGCCGGAGATGGCGT	CC1
SMC07.T.TAGCCGGAGGCGACAT	CC1

SMC07.T.TAGCCGGTCTGGGCCA	CC1
SMC07.T.TATCTCAGTGAGGCTA	CC1
SMC07.T.TATCTCATCATCTGTT	CC1
SMC07.T.TCAACGAGTTGCCTCT	CC1
SMC07.T.TCACAAGCAACCGCCA	CC1
SMC07.T.TCACGAAAGACCACGA	CC1
SMC07.T.TCAGCTCCAGGGATTG	CC1
SMC07.T.TCAGCTCGTTCCACAA	CC1
SMC07.T.TCAGCTCTCATGCATG	CC1
SMC07.T.TCAGGATAGAATTGTG	CC1
SMC07.T.TCAGGATGTTCAGACT	CC1
SMC07.T.TCAGGTAAGCACAGGT	CC1
SMC07.T.TCAGGTAGTGTCGCTG	CC1
SMC07.T.TCCACACAGAATCTCC	CC1
SMC07.T.TCCACACGTATGGTTC	CC1
SMC07.T.TCCCGATAGTACGCGA	CC1
SMC07.T.TCCCGATCAACGCACC	CC1
SMC07.T.TCGAGGCGTCGAGATG	CC1
SMC07.T.TCGAGGCGTGCGGTAA	CC1
SMC07.T.TCGAGGCTCGCCAAAT	CC1
SMC07.T.TCGCGAGCAAGCCATT	CC1
SMC07.T.TCGCGAGTCCGATATG	CC1
SMC07.T.TCGCGTTGTCTACCTC	CC1
SMC07.T.TCGCGTTGTGACTCAT	CC1
SMC07.T.TCGGGACCACGACTCG	CC1
SMC07.T.TCGGTAAAGCTGAAAT	CC1
SMC07.T.TCGTACCGTCTAGCCG	CC1
SMC07.T.TCGTAGACAGCTGGCT	CC1
SMC07.T.TCTATTGAGACAAGCC	CC1
SMC07.T.TCTATTGCAGAAGCAC	CC1
SMC07.T.TCTCATAAGCTATGCT	CC1
SMC07.T.TCTCATACAACGATCT	CC1
SMC07.T.TCTCTAACAAACAACA	CC1
SMC07.T.TCTCTAATCCGCGCAA	CC1
SMC07.T.TCTTCGGGTAAACGCG	CC2
SMC07.T.TCTTCGGTCGGCCGAT	CC1
SMC07.T.TCTTCGGTCTTCCTTC	CC1
SMC07.T.TCTTTCCAGCATCATC	CC2
SMC07.T.TCTTTCCAGTGTGAAT	CC1
SMC07.T.TCTTTCCTCTGGTTCC	CC1
SMC07.T.TGAAAGATCCAAATGC	CC1
SMC07.T.TGACGGCAGAAGGGTA	CC1
SMC07.T.TGACGGCAGATATGGT	CC1
SMC07.T.TGACGGCCAAGCCCAC	CC1
SMC07.T.TGACTAGCATTACGAC	CC1
SMC07.T.TGACTTTAGGGTATCG	CC2
SMC07.T.TGACTTTGTCTTCTCG	CC1
SMC07.T.TGAGCCGGTCACAAGG	CC1
SMC07.T.TGCCCATCATGGGACA	CC1
SMC07.T.TGCCCTAAGCTAGCCC	CC1
SMC07.T.TGCCCTAGTAAATACG	CC2
SMC07.T.TGCCCTAGTCGTTGTA	CC1
SMC07.T.TGCCCTAGTCTCTTTA	CC1
SMC07.T.TGCCCTAGTCTTGATG	CC1
SMC07.T.TGCGCAGCAGGCAGTA	CC1
SMC07.T.TGCGGGTGTCGCATAT	CC1
SMC07.T.TGCGTGGCACTCTGTC	CC2
SMC07.T.TGCGTGGTCTCATTCA	CC1

SMC07.T.TGCTACCTCAGTTTGG	CC1
SMC07.T.TGCTACCTCTTAACCT	CC1
SMC07.T.TGCTGCTCACGGATAG	CC1
SMC07.T.TGGACGCAGACAAGCC	CC1
SMC07.T.TGGACGCAGTCCATAC	CC1
SMC07.T.TGGACGCGTGAGTATA	CC1
SMC07.T.TGGCCAGCACAAGACG	CC1
SMC07.T.TGGCCAGTCGGGAGTA	CC1
SMC07.T.TGGCGCACACGACTCG	CC1
SMC07.T.TGGCTGGGTAGCCTCG	CC1
SMC07.T.TGGCTGGTCTGATTCT	CC1
SMC07.T.TGGGAAGAGGCTCAGA	CC1
SMC07.T.TGGGCGTAGTATCTCG	CC1
SMC07.T.TGGTTAGGTAGCTTGT	CC1
SMC07.T.TGGTTCCCAGCGTTCG	CC1
SMC07.T.TGGTTCCTCCCGGATG	CC1
SMC07.T.TGTATTCAGGATCGCA	CC1
SMC07.T.TGTGGTAAGCGATAGC	CC1
SMC07.T.TGTGTTTCACGTCTCT	CC1
SMC07.T.TGTGTTTTCGTCTGCT	CC1
SMC07.T.TGTTCCGTCGGACAAG	CC1
SMC07.T.TGTTCCGTCTGCGGCA	CC1
SMC07.T.TTAGGACCATATGAGA	CC1
SMC07.T.TTAGGCAAGGCTCAGA	CC1
SMC07.T.TTAGGCACAGACGTAG	CC1
SMC07.T.TTAGGCAGTTAAGATG	CC1
SMC07.T.TTAGTTCAGGCAGGTT	CC1
SMC07.T.TTAGTTCGTGTGACCC	CC1
SMC07.T.TTCCCAGCACTTCGAA	CC1
SMC07.T.TTCGAAGCAGCATACT	CC2
SMC07.T.TTCGGTCGTAAATGAC	CC1
SMC07.T.TTCTACAAGTTAGCGG	CC1
SMC07.T.TTCTTAGAGAGGACGG	CC1
SMC07.T.TTGAACGGTATGAATG	CC1
SMC07.T.TTGACTTAGTGGGTTG	CC1
SMC07.T.TTGACTTCACTTACGA	CC1
SMC07.T.TTGCCGTCAGGTCTCG	CC1
SMC07.T.TTGCGTCGTAGCGCTC	CC1
SMC07.T.TTGGAACCAAGAGGCT	CC1
SMC07.T.TTGGCAAGTAGCTGCC	CC1
SMC07.T.TTGGCAATCTAACTTC	CC1
SMC07.T.TTGTAGGAGGAGCGTT	CC1
SMC07.T.TTTGCGCAGAGTAATC	CC1
SMC07.T.TTTGCGCTCGGTGTTA	CC1
SMC07.T.TTTGGTTGTGACTCAT	CC2
SMC07.T.TTTGTCAAGTCGCCGT	CC1
SMC08.T.AAACGGGCATGCCTTC	CC1
SMC08.T.AAACGGGTCAGTTTGG	CC1
SMC08.T.AAAGATGTCCTAGGGC	CC1
SMC08.T.AAAGCAAAGTATTGGA	CC1
SMC08.T.AAAGCAACAATGGACG	CC1
SMC08.T.AAAGCAATCCTAGGGC	CC1
SMC08.T.AAATGCCTCTTTAGTC	CC1
SMC08.T.AACACGTCACCCATTC	CC1
SMC08.T.AACCGCGCAAGCGTAG	CC1
SMC08.T.AACGTTGAGATCACGG	CC1
SMC08.T.AACTCAGGTCCGAGTC	CC1
SMC08.T.AACTCCCCACAGGCCT	CC1

SMC08.T.AACTCCCGTCCGTGAC	CC1
SMC08.T.AACTCTTGTACCGGCT	CC1
SMC08.T.AACTGGTAGGCCCGTT	CC1
SMC08.T.AACTGGTTCCAAAGTC	CC1
SMC08.T.AAGACCTCAAGCTGGA	CC1
SMC08.T.AAGGAGCCACGTGAGA	CC1
SMC08.T.AAGGCAGAGCTCCCAG	CC1
SMC08.T.AAGGCAGGTAGCTCCG	CC1
SMC08.T.AAGTCTGAGCGATGAC	CC1
SMC08.T.AATCCAGAGATAGTCA	CC1
SMC08.T.AATCCAGTCGGCGGTT	CC1
SMC08.T.AATCGGTCATATACCG	CC1
SMC08.T.ACACCAACATGAGCGA	CC1
SMC08.T.ACACCGGCAGGATTGG	CC1
SMC08.T.ACACCGGGTGTGCCTG	CC1
SMC08.T.ACACTGAAGTCCATAC	CC1
SMC08.T.ACAGCCGAGTTGTAGA	CC1
SMC08.T.ACAGCTACACATCTTT	CC1
SMC08.T.ACATACGGTATCTGCA	CC1
SMC08.T.ACATACGTCACCTTAT	CC1
SMC08.T.ACATGGTAGTGTACCT	CC1
SMC08.T.ACATGGTTCCAGAGGA	CC1
SMC08.T.ACCGTAAAGTTTCCTT	CC1
SMC08.T.ACCTTTACAAAGCAAT	CC1
SMC08.T.ACGATACTCTAACCGA	CC1
SMC08.T.ACGATGTAGGATTCGG	CC1
SMC08.T.ACGATGTGTCATTAGC	CC1
SMC08.T.ACGCCAGTCGCCTGAG	CC1
SMC08.T.ACGCCGAGTAGCACGA	CC1
SMC08.T.ACGGGCTAGCGCTCCA	CC1
SMC08.T.ACGGGCTTCGCCATAA	CC1
SMC08.T.ACGGGTCCATGTCTCC	CC1
SMC08.T.ACTATCTAGGAGTACC	CC1
SMC08.T.ACTGAGTGTTATCGGT	CC1
SMC08.T.ACTGTCCTCTTGACGA	CC1
SMC08.T.ACTTGTTCACCTTGTC	CC1
SMC08.T.ACTTTCATCATTATCC	CC1
SMC08.T.AGAGCGAAGGCATGGT	CC1
SMC08.T.AGAGCGAGTGCAGTAG	CC1
SMC08.T.AGAGCTTCAATCAGAA	CC1
SMC08.T.AGATTGCAGGGAGTAA	CC1
SMC08.T.AGATTGCTCCTTGCCA	CC1
SMC08.T.AGATTGCTCTCTAAGG	CC1
SMC08.T.AGCAGCCAGCACAGGT	CC1
SMC08.T.AGCATACTCATCTGCC	CC1
SMC08.T.AGCCTAACATTTCACT	CC1
SMC08.T.AGCTCTCTCAGCCTAA	CC1
SMC08.T.AGGGAGTAGAGCAATT	CC1
SMC08.T.AGGGTGATCCATGAGT	CC1
SMC08.T.AGGTCATCACGAGAGT	CC1
SMC08.T.AGTCTTTAGGGTATCG	CC1
SMC08.T.AGTGGGAGTTGCGCAC	CC1
SMC08.T.AGTGTCATCCTCAACC	CC1
SMC08.T.AGTTGGTCATACTCTT	CC1
SMC08.T.AGTTGGTGTTCCACAA	CC1
SMC08.T.ATAACGCGTCACTGGC	CC1
SMC08.T.ATAACGCTCCGCTGTT	CC1
SMC08.T.ATAGACCAGACCCACC	CC1

SMC08.T.ATAGACCTCTAACTCT	CC1
SMC08.T.ATCCACCCAAACTGCT	CC1
SMC08.T.ATCCGAATCTGCTGCT	CC1
SMC08.T.ATCGAGTCATTACCTT	CC1
SMC08.T.ATCTACTCACGCCAGT	CC1
SMC08.T.ATCTGCCAGGCGATAC	CC1
SMC08.T.ATTACTCAGGTCATCT	CC1
SMC08.T.ATTACTCTCTGTGCAA	CC1
SMC08.T.ATTGGACAGGAGCGAG	CC1
SMC08.T.ATTTCTGTCCTAGTGA	CC1
SMC08.T.CAACTAGGTATTCGTG	CC1
SMC08.T.CAACTAGTCGTAGGAG	CC1
SMC08.T.CAAGAAACAATGTTGC	CC1
SMC08.T.CAAGATCTCGTGTAGT	CC1
SMC08.T.CACAAACCAATAGCGG	CC1
SMC08.T.CACAAACGTGTGAATA	CC1
SMC08.T.CACACTCCAATGGAGC	CC1
SMC08.T.CACAGGCAGCAATCTC	CC1
SMC08.T.CACATAGCATGACGGA	CC1
SMC08.T.CACCACTAGAGGTACC	CC1
SMC08.T.CAGCATAGTCGAAAGC	CC1
SMC08.T.CAGCCGAGTACATGTC	CC1
SMC08.T.CAGCTAAGTAGGAGTC	CC1
SMC08.T.CAGCTAATCTCGGACG	CC1
SMC08.T.CAGCTGGAGAGTGAGA	CC1
SMC08.T.CAGGTGCTCGGTTCGG	CC1
SMC08.T.CAGTCCTGTAGGCTGA	CC1
SMC08.T.CATCAAGGTGGTCTCG	CC1
SMC08.T.CATCCACAGTGAATTG	CC1
SMC08.T.CATCCACTCTTGAGAC	CC1
SMC08.T.CATCGAAGTACATCCA	CC1
SMC08.T.CATGACAAGCGACGTA	CC1
SMC08.T.CATGACATCTCATTCA	CC1
SMC08.T.CATTCGCTCTCTGTCG	CC1
SMC08.T.CCATGTCAGTACATGA	CC1
SMC08.T.CCCAATCTCACGCATA	CC1
SMC08.T.CCCATACTCAGAAATG	CC1
SMC08.T.CCGGGATTCTGTCCGT	CC1
SMC08.T.CCGGTAGCAAGAAAGG	CC1
SMC08.T.CCGGTAGTCCGCATAA	CC1
SMC08.T.CCTACACGTTGATTCG	CC1
SMC08.T.CCTACCATCGTCTGAA	CC1
SMC08.T.CCTAGCTAGCTGGAAC	CC1
SMC08.T.CCTAGCTGTTCAGTAC	CC1
SMC08.T.CCTAGCTTCAGCATGT	CC1
SMC08.T.CCTCTGAGTCTTGCGG	CC1
SMC08.T.CCTTCGATCGCATGGC	CC1
SMC08.T.CCTTTCTAGGACCACA	CC1
SMC08.T.CGAACATCATAGTAAG	CC1
SMC08.T.CGACTTCCAGTGACAG	CC1
SMC08.T.CGACTTCTCCACTGGG	CC1
SMC08.T.CGAGCACGTTCTCATT	CC1
SMC08.T.CGATGTATCATTATCC	CC1
SMC08.T.CGATTGATCCCACTTG	CC1
SMC08.T.CGCCAAGCAGCCAGAA	CC1
SMC08.T.CGCTATCAGACTTGAA	CC1
SMC08.T.CGCTATCTCCATGCTC	CC1
SMC08.T.CGCTGGAAGAAGAAGC	CC1

SMC08.T.CGCTGGATCACTCCTG	CC1
SMC08.T.CGGACTGAGCCGTCGT	CC1
SMC08.T.CGGAGCTTCAACTCTT	CC1
SMC08.T.CGGAGCTTCGTGGTCG	CC1
SMC08.T.CGGAGTCAGTAACCCT	CC1
SMC08.T.CGGCTAGGTGCTAGCC	CC1
SMC08.T.CGGCTAGTCAGTTCGA	CC1
SMC08.T.CGGGTCAAGCAGGCTA	CC1
SMC08.T.CGGGTCACAGTCGATT	CC1
SMC08.T.CGGTTAAGTTGCCTCT	CC1
SMC08.T.CGTCTACGTTGTCTTT	CC1
SMC08.T.CGTGAGCGTCCGTGAC	CC1
SMC08.T.CGTTCTGCACCAGCAC	CC1
SMC08.T.CGTTCTGGTTAAGTAG	CC1
SMC08.T.CTAAGACAGTCGTTTG	CC1
SMC08.T.CTAAGACCACGCATCG	CC1
SMC08.T.CTAATGGGTAGCGATG	CC1
SMC08.T.CTACACCCAAAGCGGT	CC1
SMC08.T.CTACACCGTCCAGTTA	CC1
SMC08.T.CTACATTGTCGACTAT	CC1
SMC08.T.CTACGTCTCCTAAGTG	CC1
SMC08.T.CTCGAAAGTCTTCTCG	CC1
SMC08.T.CTCGGGAAGTATCTCG	CC1
SMC08.T.CTCGTCATCTTGACGA	CC1
SMC08.T.CTGAAGTAGCGATAGC	CC1
SMC08.T.CTGAAGTCAGCTGTGC	CC1
SMC08.T.CTGATAGAGATCCTGT	CC1
SMC08.T.CTGCCTAGTCTTGATG	CC1
SMC08.T.CTGTGCTAGAGTGACC	CC1
SMC08.T.CTGTGCTAGTGAATTG	CC1
SMC08.T.CTGTGCTGTTCATGGT	CC1
SMC08.T.CTGTTTAAGACTTGAA	CC1
SMC08.T.CTTAGGAAGCCAGTAG	CC1
SMC08.T.CTTGGCTAGGGAACGG	CC1
SMC08.T.GAAATGACAAGTTAAG	CC1
SMC08.T.GAACCTAAGGAGTCTG	CC1
SMC08.T.GAAGCAGGTAAGAGAG	CC1
SMC08.T.GACAGAGCAGGACCCT	CC1
SMC08.T.GACCAATGTCTAGGTT	CC1
SMC08.T.GACCAATTCACCTCGT	CC2
SMC08.T.GACCAATTCCGCAAGC	CC1
SMC08.T.GACCTGGAGGATATAC	CC1
SMC08.T.GACGCGTAGGAGTTGC	CC1
SMC08.T.GACGCGTGTCGACTGC	CC1
SMC08.T.GACGTGCAGACTAAGT	CC1
SMC08.T.GACGTTAAGTCGTTTG	CC1
SMC08.T.GACGTTACATAAAGGT	CC1
SMC08.T.GACTACAGTGATGTGG	CC1
SMC08.T.GACTGCGAGCGTAGTG	CC1
SMC08.T.GAGCAGAAGTCAAGGC	CC1
SMC08.T.GAGGTGACAGTAAGCG	CC1
SMC08.T.GAGGTGACAGTCACTA	CC1
SMC08.T.GAGGTGAGTCATGCCG	CC1
SMC08.T.GAGTCCGTCATATCGG	CC1
SMC08.T.GATCGATCACTGTGTA	CC1
SMC08.T.GATCGATTCTGGTATG	CC1
SMC08.T.GATCGCGTCAGAGACG	CC1
SMC08.T.GATCGTACATCCGTGG	CC1

SMC08.T.GATCTAGGTCGAATCT	CC1
SMC08.T.GATCTAGGTCGGGTCT	CC1
SMC08.T.GATGAGGGTGTGTGCC	CC1
SMC08.T.GATGAGGTCACTATTC	CC1
SMC08.T.GATGAGGTCGGCTTGG	CC1
SMC08.T.GATGAGGTCTACCTGC	CC1
SMC08.T.GATGCTATCGTTTAGG	CC1
SMC08.T.GATTCAGAGACTAGGC	CC1
SMC08.T.GCACATACACTAGTAC	CC1
SMC08.T.GCACATACATAAAGGT	CC1
SMC08.T.GCACATATCACCTTAT	CC1
SMC08.T.GCACTCTAGTAGCGGT	CC1
SMC08.T.GCAGCCACAAACAACA	CC1
SMC08.T.GCATACACAACCGCCA	CC1
SMC08.T.GCGAGAAAGCTAGTTC	CC1
SMC08.T.GCGCAACCATCACGTA	CC1
SMC08.T.GCGCAGTGTTAAAGTG	CC1
SMC08.T.GCGCCAAGTTACTGAC	CC1
SMC08.T.GCGCGATTCACATGCA	CC1
SMC08.T.GCGGGTTGTGACGGTA	CC1
SMC08.T.GCTCTGTGTCTTCGTC	CC1
SMC08.T.GCTGCGACAATAGCGG	CC1
SMC08.T.GCTTCCACAGTCGTGC	CC1
SMC08.T.GGACATTAGGACACCA	CC1
SMC08.T.GGACATTCAAGAGGCT	CC1
SMC08.T.GGACATTCAATTCCTT	CC1
SMC08.T.GGACATTGTATCAGTC	CC1
SMC08.T.GGACATTGTTAAGTAG	CC1
SMC08.T.GGACATTTCGAGCCCA	CC1
SMC08.T.GGAGCAAAGGTTCCTA	CC1
SMC08.T.GGATGTTCACAGACAG	CC1
SMC08.T.GGATTACGTCGAATCT	CC1
SMC08.T.GGCTCGACAAGGCTCC	CC1
SMC08.T.GGCTCGAGTTCCAACA	CC1
SMC08.T.GGGACCTAGAGCTGGT	CC1
SMC08.T.GGGCACTAGACTTGAA	CC1
SMC08.T.GGGCACTTCTCTTATG	CC1
SMC08.T.GGTATTGAGACAGGCT	CC1
SMC08.T.GGTGTTAGTGCACTTA	CC1
SMC08.T.GTAACGTTCCTTGGTC	CC1
SMC08.T.GTACGTATCATGTAGC	CC1
SMC08.T.GTACTCCAGAGGACGG	CC1
SMC08.T.GTACTTTGTTCTGTTT	CC1
SMC08.T.GTCACAAAGCTGTCTA	CC1
SMC08.T.GTCACAAAGGACATTA	CC1
SMC08.T.GTCACGGGTTCAACCA	CC1
SMC08.T.GTCATTTCAAGGTGTG	CC1
SMC08.T.GTCATTTTCCTTGCCA	CC1
SMC08.T.GTCCTCAGTAAACCTC	CC1
SMC08.T.GTCGGGTGTGACGGTA	CC1
SMC08.T.GTGCAGCCACGGTAAG	CC1
SMC08.T.GTGCGGTTCGCGCCAA	CC1
SMC08.T.GTGCTTCAGGTGCTAG	CC1
SMC08.T.GTGTGCGCATTGAGCT	CC1
SMC08.T.GTGTTAGAGTGTCCAT	CC1
SMC08.T.GTTAAGCAGAAGCCCA	CC1
SMC08.T.GTTCATTCACGGATAG	CC1
SMC08.T.GTTCGGGGTCGATTGT	CC1

SMC08.T.GTTCTCGCAGCCTATA	CC1
SMC08.T.GTTCTCGCAGTCAGCC	CC1
SMC08.T.GTTTCTAAGACCTTTG	CC1
SMC08.T.TAAGAGAGTGCGAAAC	CC1
SMC08.T.TAAGAGATCGGTGTCG	CC1
SMC08.T.TACCTATGTCAGAATA	CC1
SMC08.T.TACGGGCAGAGGGCTT	CC1
SMC08.T.TACGGGCCACCCAGTG	CC1
SMC08.T.TACTCATTCTCTGCTG	CC1
SMC08.T.TACTTACTCCAAGTAC	CC1
SMC08.T.TAGAGCTGTTGATTGC	CC1
SMC08.T.TAGGCATAGATGCGAC	CC1
SMC08.T.TAGGCATGTGTTTGTG	CC1
SMC08.T.TAGTGGTTCTGATTCT	CC1
SMC08.T.TAGTTGGAGGACGAAA	CC1
SMC08.T.TATCAGGCATGGTCTA	CC1
SMC08.T.TATTACCAGGGTTCCC	CC1
SMC08.T.TATTACCTCAACACCA	CC1
SMC08.T.TCAACGAAGACCGGAT	CC1
SMC08.T.TCAATCTAGACCTAGG	CC1
SMC08.T.TCAATCTGTTGGTAAA	CC1
SMC08.T.TCAATCTTCAGCACAT	CC1
SMC08.T.TCACAAGGTGTGAATA	CC1
SMC08.T.TCACGAACAAGAAAGG	CC1
SMC08.T.TCAGATGCAACTGCTA	CC1
SMC08.T.TCAGATGCAATAAGCA	CC1
SMC08.T.TCAGGATCAATGGTCT	CC1
SMC08.T.TCAGGTAAGGGCACTA	CC1
SMC08.T.TCAGGTAAGGTAAACT	CC1
SMC08.T.TCATTTGGTCTAGAGG	CC1
SMC08.T.TCCACACTCGTTGCCT	CC1
SMC08.T.TCGCGAGCACCTCGTT	CC1
SMC08.T.TCGCGTTTCTTCTGGC	CC1
SMC08.T.TCTATTGCAATCCGAT	CC1
SMC08.T.TCTATTGCACCTTGTC	CC1
SMC08.T.TCTCATAAGCTCCCAG	CC1
SMC08.T.TCTTTCCGTACCGCTG	CC1
SMC08.T.TCTTTCCGTCTCCATC	CC1
SMC08.T.TCTTTCCTCTTCGAGA	CC1
SMC08.T.TGACGGCCACAACTGT	CC1
SMC08.T.TGACTTTAGACTGTAA	CC1
SMC08.T.TGACTTTCACATCCAA	CC1
SMC08.T.TGATTTCAGCTGCCCA	CC1
SMC08.T.TGCCCATAGCGGCTTC	CC1
SMC08.T.TGCCCATAGTGCGTGA	CC1
SMC08.T.TGCGCAGGTCGGATCC	CC1
SMC08.T.TGCGTGGTCCTGCAGG	CC1
SMC08.T.TGCGTGGTCTGGTATG	CC1
SMC08.T.TGCTACCCACTCTGTC	CC1
SMC08.T.TGCTACCGTCGTGGCT	CC1
SMC08.T.TGCTACCTCGCTAGCG	CC1
SMC08.T.TGGACGCCAAGTTCTG	CC1
SMC08.T.TGGCCAGAGCCTTGAT	CC1
SMC08.T.TGGCGCAAGGGCATGT	CC1
SMC08.T.TGGCTGGCAGCCACCA	CC1
SMC08.T.TGGGAAGAGTCGCCGT	CC1
SMC08.T.TGGGAAGTCTCAACTT	CC1
SMC08.T.TGGTTAGCATGTCCTC	CC1

SMC08.T.TGGTTAGGTGTCCTCT	CC1
SMC08.T.TGGTTAGTCCCTAACC	CC1
SMC08.T.TGGTTCCCAAGCCGCT	CC1
SMC08.T.TGTGGTATCCTTCAAT	CC1
SMC08.T.TGTGTTTCAAGTTAAG	CC1
SMC08.T.TTAACTCGTACCCAAT	CC1
SMC08.T.TTAGTTCAGCTACCGC	CC1
SMC08.T.TTCGAAGGTTGGTAAA	CC1
SMC08.T.TTCTACAAGTTCGATC	CC1
SMC08.T.TTCTCAAGTAAGAGAG	CC1
SMC08.T.TTCTTAGAGTGTCTCA	CC1
SMC08.T.TTCTTAGTCGCGGATC	CC1
SMC08.T.TTGAACGTCGTCTGAA	CC1
SMC08.T.TTGGAACAGAGTAAGG	CC1
SMC08.T.TTGGCAAAGATCCTGT	CC1
SMC08.T.TTTACTGCAATCCAAC	CC1
SMC08.T.TTTACTGGTTAGATGA	CC1
SMC08.T.TTTGCGCAGACTACAA	CC1
SMC09.T.AAACCTGAGAAACCTA	CC2
SMC09.T.AAACCTGAGCCAGTAG	CC2
SMC09.T.AAACCTGAGGCAGGTT	CC1
SMC09.T.AAACCTGCACTTGGAT	CC1
SMC09.T.AAACCTGGTGAAAGAG	CC1
SMC09.T.AAACCTGTCCACGAAT	CC1
SMC09.T.AAACCTGTCGCTTGTC	CC1
SMC09.T.AAACCTGTCTCCCTGA	CC1
SMC09.T.AAACGGGAGTGAAGTT	CC1
SMC09.T.AAACGGGTCGAGAGCA	CC1
SMC09.T.AAAGATGAGAATAGGG	CC1
SMC09.T.AAAGATGAGAGCCTAG	CC1
SMC09.T.AAAGATGAGGGCTTCC	CC1
SMC09.T.AAAGATGAGGGCTTGA	CC1
SMC09.T.AAAGATGCAACAACCT	CC1
SMC09.T.AAAGATGTCTTTACAC	CC2
SMC09.T.AAAGCAAAGCCAGGAT	CC2
SMC09.T.AAAGCAACATGCCCGA	CC1
SMC09.T.AAAGCAAGTTTGGGCC	CC1
SMC09.T.AAAGCAATCTCTTATG	CC1
SMC09.T.AAAGTAGAGCTCCCAG	CC1
SMC09.T.AAAGTAGAGTACCGGA	CC2
SMC09.T.AAAGTAGCAAATTGCC	CC1
SMC09.T.AAAGTAGCACAGGCCT	CC1
SMC09.T.AAAGTAGGTACTTAGC	CC1
SMC09.T.AAAGTAGGTTATGTGC	CC1
SMC09.T.AAAGTAGGTTTGTTTC	CC1
SMC09.T.AAAGTAGTCACAATGC	CC1
SMC09.T.AAAGTAGTCTAACGGT	CC1
SMC09.T.AAATGCCAGCCTCGTG	CC1
SMC09.T.AAATGCCCACATGACT	CC1
SMC09.T.AAATGCCCAGCTGTAT	CC1
SMC09.T.AACACGTCAATGGACG	CC1
SMC09.T.AACACGTGTCTCATCC	CC1
SMC09.T.AACACGTTCTCGCTTG	CC1
SMC09.T.AACCATGCAAGAGGCT	CC1
SMC09.T.AACCATGCAGGGTTAG	CC1
SMC09.T.AACCATGGTTCACGGC	CC1
SMC09.T.AACCATGTCCTTTCTC	CC1
SMC09.T.AACCATGTCTGGTTCC	CC2

SMC09.T.AACCGCGGTAAGAGAG	CC1
SMC09.T.AACGTTGCATACTACG	CC2
SMC09.T.AACGTTGCATTAGCCA	CC1
SMC09.T.AACGTTGGTAATCGTC	CC1
SMC09.T.AACGTTGGTCGCGAAA	CC1
SMC09.T.AACGTTGTCAAGGTAA	CC1
SMC09.T.AACTCAGCACTTACGA	CC1
SMC09.T.AACTCAGCAGACGTAG	CC2
SMC09.T.AACTCAGGTCATATGC	CC1
SMC09.T.AACTCAGGTGGAAAGA	CC1
SMC09.T.AACTCAGTCGGTTAAC	CC1
SMC09.T.AACTCCCGTTTAAGCC	CC1
SMC09.T.AACTCTTAGCGATGAC	CC1
SMC09.T.AACTCTTGTAAGTAGT	CC1
SMC09.T.AACTCTTGTTAAAGAC	CC1
SMC09.T.AACTCTTTCAGCTGGC	CC2
SMC09.T.AACTGGTAGGAACTGC	CC1
SMC09.T.AACTGGTCAGCGTAAG	CC1
SMC09.T.AACTGGTCATCACGAT	CC1
SMC09.T.AACTGGTGTCCGACGT	CC1
SMC09.T.AACTTTCTCGAATGGG	CC1
SMC09.T.AACTTTCTCTCCGGTT	CC2
SMC09.T.AAGACCTGTCTGGAGA	CC1
SMC09.T.AAGCCGCCAGCATGAG	CC1
SMC09.T.AAGGAGCAGAGCTATA	CC1
SMC09.T.AAGGAGCAGGGTTTCT	CC1
SMC09.T.AAGGAGCGTTGTCTTT	CC1
SMC09.T.AAGGAGCTCCTTGGTC	CC1
SMC09.T.AAGGCAGAGCGATCCC	CC1
SMC09.T.AAGGCAGAGCGTTGCC	CC1
SMC09.T.AAGGCAGCAATAACGA	CC1
SMC09.T.AAGGCAGCAATACGCT	CC1
SMC09.T.AAGGCAGCACAACTGT	CC1
SMC09.T.AAGGCAGGTAATTGGA	CC1
SMC09.T.AAGGTTCAGCACCGTC	CC1
SMC09.T.AAGGTTCAGTACATGA	CC2
SMC09.T.AAGGTTCGTTTGGCGC	CC2
SMC09.T.AAGGTTCTCAAGATCC	CC1
SMC09.T.AAGGTTCTCGGAGGTA	CC1
SMC09.T.AAGTCTGCAGAAGCAC	CC1
SMC09.T.AAGTCTGGTCCCGACA	CC1
SMC09.T.AAGTCTGGTTTAAGCC	CC1
SMC09.T.AAGTCTGTCCTAGTGA	CC1
SMC09.T.AATCCAGAGAATTCCC	CC1
SMC09.T.AATCCAGCATTGTGCA	CC1
SMC09.T.AATCCAGGTGATGTGG	CC1
SMC09.T.ACACCAAAGCACCGCT	CC2
SMC09.T.ACACCAAAGCTAGTCT	CC1
SMC09.T.ACACCAACAATCGAAA	CC1
SMC09.T.ACACCAAGTTAGTGGG	CC1
SMC09.T.ACACCAATCAACACAC	CC1
SMC09.T.ACACCAATCCACTGGG	CC1
SMC09.T.ACACCCTCAGATCTGT	CC1
SMC09.T.ACACCCTGTCAGGACA	CC1
SMC09.T.ACACCCTTCTCCGGTT	CC1
SMC09.T.ACACCGGAGCACCGCT	CC1
SMC09.T.ACACCGGAGTCTCGGC	CC1
SMC09.T.ACACCGGGTTAAAGAC	CC1

SMC09.T.ACACCGGTCGCGTTTC	CC1
SMC09.T.ACACTGACACGACTCG	CC2
SMC09.T.ACACTGATCGCCCTTA	CC1
SMC09.T.ACACTGATCTCTTGAT	CC1
SMC09.T.ACAGCCGAGAAGATTC	CC1
SMC09.T.ACAGCCGGTCAGCTAT	CC1
SMC09.T.ACAGCCGTCCAGTAGT	CC1
SMC09.T.ACAGCTAAGCCAGTAG	CC2
SMC09.T.ACATACGAGATCCCAT	CC1
SMC09.T.ACATACGAGATCGATA	CC1
SMC09.T.ACATACGCAAGAAGAG	CC1
SMC09.T.ACATACGCAAGCCGCT	CC1
SMC09.T.ACATACGCAGACGCCT	CC1
SMC09.T.ACATACGCATCTCGCT	CC1
SMC09.T.ACATACGCATGACGGA	CC1
SMC09.T.ACATACGTCACCTCGT	CC2
SMC09.T.ACATACGTCTTATCTG	CC1
SMC09.T.ACATCAGGTCGGCACT	CC1
SMC09.T.ACATGGTCAAAGTCAA	CC1
SMC09.T.ACATGGTCAACGATGG	CC1
SMC09.T.ACATGGTCAGTATAAG	CC1
SMC09.T.ACATGGTCATGTAGTC	CC1
SMC09.T.ACCAGTAAGAGGGATA	CC1
SMC09.T.ACCAGTAAGGCACATG	CC1
SMC09.T.ACCAGTACACTACAGT	CC1
SMC09.T.ACCAGTACAGCTCGAC	CC1
SMC09.T.ACCAGTACATAACCTG	CC1
SMC09.T.ACCAGTAGTAGCTGCC	CC1
SMC09.T.ACCCACTAGCTGGAAC	CC2
SMC09.T.ACCCACTAGGACAGCT	CC1
SMC09.T.ACCCACTCACCATGTA	CC1
SMC09.T.ACCCACTCACCTGGTG	CC1
SMC09.T.ACCCACTGTCGATTGT	CC1
SMC09.T.ACCGTAAGTGCACCAC	CC1
SMC09.T.ACCGTAATCGCAAACT	CC1
SMC09.T.ACCTTTAAGGATGCGT	CC1
SMC09.T.ACCTTTAAGTTGAGAT	CC1
SMC09.T.ACCTTTACACATGTGT	CC1
SMC09.T.ACCTTTAGTCATATCG	CC1
SMC09.T.ACCTTTAGTTATCACG	CC1
SMC09.T.ACCTTTATCTCATTCA	CC1
SMC09.T.ACGAGCCGTATCGCAT	CC1
SMC09.T.ACGAGCCTCATTATCC	CC1
SMC09.T.ACGAGGATCAAAGACA	CC1
SMC09.T.ACGAGGATCCGCGGTA	CC1
SMC09.T.ACGAGGATCGGTGTTA	CC1
SMC09.T.ACGATACAGCCAGTTT	CC1
SMC09.T.ACGATACAGGCTACGA	CC2
SMC09.T.ACGATACGTCACTGGC	CC1
SMC09.T.ACGATACGTCGTGGCT	CC1
SMC09.T.ACGATACGTTCTGAAC	CC1
SMC09.T.ACGATACTCCTTTACA	CC1
SMC09.T.ACGATGTGTCGGCTCA	CC2
SMC09.T.ACGCAGCAGACGCTTT	CC1
SMC09.T.ACGCAGCGTCTCTTAT	CC1
SMC09.T.ACGCCAGAGAACTCGG	CC1
SMC09.T.ACGCCAGCATCCCACT	CC1
SMC09.T.ACGCCAGTCTCGGACG	CC2

SMC09.T.ACGCCGACATGCCCGA	CC1
SMC09.T.ACGCCGATCATTGCGA	CC1
SMC09.T.ACGGAGAAGAGGTTAT	CC1
SMC09.T.ACGGAGACAGTCGTGC	CC2
SMC09.T.ACGGAGATCTTGAGAC	CC1
SMC09.T.ACGGCCACAATGGAAT	CC1
SMC09.T.ACGGCCACACGGCGTT	CC1
SMC09.T.ACGGCCAGTCCTCCAT	CC1
SMC09.T.ACGGCCAGTTAAAGTG	CC1
SMC09.T.ACGGCCATCGGCGCAT	CC1
SMC09.T.ACGGCCATCTACGAGT	CC1
SMC09.T.ACGGGTCAGTTCGCGC	CC1
SMC09.T.ACGGGTCTCCTCATTA	CC1
SMC09.T.ACGTCAAAGCGGATCA	CC1
SMC09.T.ACGTCAACACAAGACG	CC1
SMC09.T.ACGTCAACACCTTGTC	CC1
SMC09.T.ACGTCAACAGCTATTG	CC1
SMC09.T.ACTGAACAGACTCGGA	CC1
SMC09.T.ACTGAACAGCCACGTC	CC2
SMC09.T.ACTGAACCAAACCTAC	CC1
SMC09.T.ACTGAACTCGCTAGCG	CC1
SMC09.T.ACTGAGTCAGACAAGC	CC1
SMC09.T.ACTGAGTCAGGTCCAC	CC1
SMC09.T.ACTGAGTCATATGGTC	CC1
SMC09.T.ACTGAGTGTCTCCACT	CC2
SMC09.T.ACTGATGCAGCCACCA	CC1
SMC09.T.ACTGATGGTTCAACCA	CC1
SMC09.T.ACTGCTCAGGGAACGG	CC2
SMC09.T.ACTGCTCCACTGAAGG	CC1
SMC09.T.ACTGCTCGTGTTTGGT	CC1
SMC09.T.ACTGCTCGTTCCGTCT	CC1
SMC09.T.ACTGCTCTCCGCTGTT	CC1
SMC09.T.ACTGCTCTCTGCTGTC	CC2
SMC09.T.ACTGTCCGTGACGCCT	CC1
SMC09.T.ACTTACTAGACGACGT	CC1
SMC09.T.ACTTACTAGCCCAATT	CC1
SMC09.T.ACTTACTCAAAGGCGT	CC1
SMC09.T.ACTTACTCATAAAGGT	CC1
SMC09.T.ACTTACTTCGAGGTAG	CC2
SMC09.T.ACTTGTTAGCAATATG	CC1
SMC09.T.ACTTGTTAGTCCGGTC	CC1
SMC09.T.ACTTGTTCAAGTAGTA	CC1
SMC09.T.ACTTGTTCAATGTAAG	CC1
SMC09.T.ACTTGTTGTAAATGTG	CC1
SMC09.T.ACTTGTTGTCAGCTAT	CC1
SMC09.T.ACTTGTTTCATCGGAT	CC2
SMC09.T.ACTTGTTTCATGCATG	CC1
SMC09.T.ACTTTCAGTAGCGCTC	CC1
SMC09.T.ACTTTCATCTCAAACG	CC1
SMC09.T.AGAATAGAGACCTTTG	CC1
SMC09.T.AGAATAGAGGGCTTCC	CC1
SMC09.T.AGAATAGGTAAAGTCA	CC1
SMC09.T.AGAATAGTCGAGAGCA	CC1
SMC09.T.AGAATAGTCGGACAAG	CC1
SMC09.T.AGAATAGTCTTTAGGG	CC1
SMC09.T.AGACGTTAGGCCGAAT	CC1
SMC09.T.AGACGTTCATGAGCGA	CC1
SMC09.T.AGACGTTGTTCGTCTC	CC2

SMC09.T.AGAGCGAAGCTTTGGT	CC1
SMC09.T.AGAGCGAAGTCTCCTC	CC1
SMC09.T.AGAGCGACAACTGCGC	CC1
SMC09.T.AGAGCGACAGCATACT	CC1
SMC09.T.AGAGCGAGTAGGCTGA	CC1
SMC09.T.AGAGCGAGTCCTCCAT	CC1
SMC09.T.AGAGCGATCCGCGTTT	CC1
SMC09.T.AGAGCTTAGTGGAGTC	CC1
SMC09.T.AGAGCTTCAATAGCAA	CC1
SMC09.T.AGAGCTTCAATGTAAG	CC1
SMC09.T.AGAGCTTCACATCTTT	CC1
SMC09.T.AGAGCTTGTCAATGTC	CC2
SMC09.T.AGAGCTTGTCTTGATG	CC1
SMC09.T.AGAGCTTTCCTTTACA	CC1
SMC09.T.AGAGTGGAGGCTAGGT	CC1
SMC09.T.AGAGTGGCAGTATCTG	CC1
SMC09.T.AGAGTGGGTAAGCACG	CC1
SMC09.T.AGAGTGGTCGCCTGAG	CC1
SMC09.T.AGATCTGTCAACACGT	CC1
SMC09.T.AGATCTGTCAGGTAAA	CC2
SMC09.T.AGATCTGTCCAAGCCG	CC1
SMC09.T.AGATTGCAGAAGGTGA	CC1
SMC09.T.AGATTGCAGTCCCACG	CC1
SMC09.T.AGATTGCCAAGCGTAG	CC1
SMC09.T.AGATTGCCAGCATGAG	CC1
SMC09.T.AGATTGCCAGGGAGAG	CC1
SMC09.T.AGATTGCGTTCCCGAG	CC1
SMC09.T.AGCAGCCAGTGACATA	CC1
SMC09.T.AGCAGCCCACACGCTG	CC2
SMC09.T.AGCATACCATTATCTC	CC1
SMC09.T.AGCATACGTAAGGGCT	CC2
SMC09.T.AGCATACGTGCCTGGT	CC1
SMC09.T.AGCCTAACACTTAACG	CC2
SMC09.T.AGCCTAACAGATGGGT	CC1
SMC09.T.AGCCTAACAGCAGTTT	CC1
SMC09.T.AGCCTAACATATGGTC	CC1
SMC09.T.AGCCTAAGTGTTTGTG	CC1
SMC09.T.AGCCTAATCCAAACAC	CC1
SMC09.T.AGCCTAATCGGCTACG	CC1
SMC09.T.AGCGGTCCAGCTATTG	CC1
SMC09.T.AGCGGTCTCGGAAACG	CC2
SMC09.T.AGCGTATAGTACATGA	CC1
SMC09.T.AGCGTATCAGAAGCAC	CC1
SMC09.T.AGCGTATCAGTCTTCC	CC1
SMC09.T.AGCGTATCATCAGTCA	CC1
SMC09.T.AGCGTATTCCTGCCAT	CC1
SMC09.T.AGCGTCGAGATATGGT	CC1
SMC09.T.AGCGTCGTCATAGCAC	CC1
SMC09.T.AGCGTCGTCCAAGCCG	CC1
SMC09.T.AGCTCCTAGACCTTTG	CC1
SMC09.T.AGCTCCTGTAAGGGAA	CC1
SMC09.T.AGCTCCTGTCTTGTCC	CC1
SMC09.T.AGCTCCTTCATCTGTT	CC1
SMC09.T.AGCTCCTTCGGCGCTA	CC1
SMC09.T.AGCTCTCGTAAGAGGA	CC1
SMC09.T.AGCTCTCGTCATATGC	CC1
SMC09.T.AGCTTGAAGTGGAGTC	CC1
SMC09.T.AGCTTGACATGACGGA	CC1

SMC09.T.AGCTTGACATGCCCGA	CC1
SMC09.T.AGCTTGATCGAATCCA	CC1
SMC09.T.AGCTTGATCTAACTCT	CC1
SMC09.T.AGGCCACAGATCCGAG	CC1
SMC09.T.AGGCCACCATATGAGA	CC1
SMC09.T.AGGCCACGTTGCTCCT	CC1
SMC09.T.AGGCCACTCTGAGTGT	CC1
SMC09.T.AGGCCGTCACTACAGT	CC1
SMC09.T.AGGCCGTCAGTCTTCC	CC1
SMC09.T.AGGCCGTCAGTGAGTG	CC1
SMC09.T.AGGCCGTCATGTTGAC	CC1
SMC09.T.AGGCCGTGTATGAATG	CC1
SMC09.T.AGGCCGTGTCTAGGTT	CC1
SMC09.T.AGGCCGTGTTGGTAAA	CC1
SMC09.T.AGGCCGTTCTCCGGTT	CC1
SMC09.T.AGGCCGTTCTGTCAAG	CC2
SMC09.T.AGGGAGTCACGTCAGC	CC1
SMC09.T.AGGGAGTCAGCCAGAA	CC1
SMC09.T.AGGGATGAGAAGGACA	CC1
SMC09.T.AGGGATGAGATTACCC	CC1
SMC09.T.AGGGATGAGGTAAACT	CC1
SMC09.T.AGGGATGTCGAATCCA	CC1
SMC09.T.AGGGTGACAGGAATCG	CC1
SMC09.T.AGGGTGAGTGCCTGCA	CC1
SMC09.T.AGGTCATAGACATAAC	CC1
SMC09.T.AGGTCATCAAGCGCTC	CC1
SMC09.T.AGGTCATGTTCAGCGC	CC1
SMC09.T.AGGTCATTCCCTAACC	CC1
SMC09.T.AGGTCATTCGTCGTTC	CC1
SMC09.T.AGGTCCGGTGGTTTCA	CC1
SMC09.T.AGGTCCGTCAATACCG	CC1
SMC09.T.AGGTCCGTCGAGCCCA	CC1
SMC09.T.AGGTCCGTCTAAGCCA	CC1
SMC09.T.AGTAGTCTCGCTGATA	CC1
SMC09.T.AGTAGTCTCGGCGCTA	CC1
SMC09.T.AGTAGTCTCGTAGGAG	CC1
SMC09.T.AGTCTTTCAAAGGCGT	CC1
SMC09.T.AGTGAGGAGGCAAAGA	CC1
SMC09.T.AGTGAGGCATGTTCCC	CC1
SMC09.T.AGTGGGAGTGCTAGCC	CC1
SMC09.T.AGTGGGAGTTCTGGTA	CC1
SMC09.T.AGTGGGAGTTGGTAAA	CC1
SMC09.T.AGTGGGATCTACGAGT	CC1
SMC09.T.AGTGTCAAGGGCTTGA	CC1
SMC09.T.AGTGTCACAAGGTGTG	CC1
SMC09.T.AGTGTCACATCCAACA	CC1
SMC09.T.AGTGTCAGTGCTAGCC	CC1
SMC09.T.AGTGTCATCTGAGGGA	CC1
SMC09.T.AGTTGGTAGGTGCTAG	CC1
SMC09.T.AGTTGGTCAGCTGCAC	CC1
SMC09.T.AGTTGGTGTGGTTTCA	CC1
SMC09.T.AGTTGGTTCCTGTAGA	CC1
SMC09.T.ATAACGCCAATCGAAA	CC1
SMC09.T.ATAACGCGTTCGTTGA	CC1
SMC09.T.ATAACGCTCTCAACTT	CC1
SMC09.T.ATAACGCTCTGTCCGT	CC1
SMC09.T.ATAACGCTCTGTCTAT	CC1
SMC09.T.ATAAGAGAGATGTGGC	CC1

SMC09.T.ATAAGAGGTCCGAACC	CC1
SMC09.T.ATAGACCCAGTAACGG	CC1
SMC09.T.ATAGACCGTATTCTCT	CC1
SMC09.T.ATAGACCTCCGAACGC	CC1
SMC09.T.ATAGACCTCTTGTTTG	CC1
SMC09.T.ATCACGAAGTACGCCC	CC1
SMC09.T.ATCACGACAGGAATGC	CC1
SMC09.T.ATCACGAGTCGGGTCT	CC1
SMC09.T.ATCATCTAGAAGAAGC	CC2
SMC09.T.ATCATCTCAAACTGTC	CC1
SMC09.T.ATCATCTCATATGGTC	CC1
SMC09.T.ATCATCTCATCTCCCA	CC1
SMC09.T.ATCATCTCATGTCCTC	CC1
SMC09.T.ATCATCTGTTTGGGCC	CC1
SMC09.T.ATCATCTTCCACTGGG	CC1
SMC09.T.ATCATGGCAAGGCTCC	CC2
SMC09.T.ATCCACCCAAGACGTG	CC2
SMC09.T.ATCCACCCAGCTGTAT	CC1
SMC09.T.ATCCACCGTCAACATC	CC1
SMC09.T.ATCCACCGTGGTCCGT	CC2
SMC09.T.ATCCACCTCCACGCAG	CC1
SMC09.T.ATCCACCTCGATGAGG	CC1
SMC09.T.ATCCGAACACTCGACG	CC2
SMC09.T.ATCCGAATCACCCTCA	CC1
SMC09.T.ATCCGAATCCAGGGCT	CC1
SMC09.T.ATCGAGTCAGTACACT	CC1
SMC09.T.ATCGAGTGTGAGGGAG	CC1
SMC09.T.ATCGAGTGTGTGAATA	CC1
SMC09.T.ATCGAGTTCGCACTCT	CC2
SMC09.T.ATCTACTCAAGAGGCT	CC1
SMC09.T.ATCTACTGTGGTCTCG	CC1
SMC09.T.ATCTGCCAGAGTACCG	CC1
SMC09.T.ATCTGCCGTACATGTC	CC2
SMC09.T.ATGAGGGAGAGTAAGG	CC1
SMC09.T.ATGAGGGAGTACGTAA	CC1
SMC09.T.ATGAGGGAGTGCGATG	CC1
SMC09.T.ATGAGGGCAGCAGTTT	CC1
SMC09.T.ATGAGGGTCCAAGCCG	CC1
SMC09.T.ATGCGATAGCCCGAAA	CC1
SMC09.T.ATGCGATAGTGGGTTG	CC1
SMC09.T.ATGCGATCATACCATG	CC1
SMC09.T.ATGCGATGTCTGCCAG	CC1
SMC09.T.ATGCGATGTTAGGGTG	CC2
SMC09.T.ATGCGATGTTGGTGGA	CC1
SMC09.T.ATGGGAGAGACTTGAA	CC1
SMC09.T.ATGGGAGGTCTCTCGT	CC1
SMC09.T.ATGGGAGGTGGCGAAT	CC1
SMC09.T.ATGGGAGTCATATCGG	CC1
SMC09.T.ATGTGTGAGCAATATG	CC1
SMC09.T.ATGTGTGTCCACGAAT	CC1
SMC09.T.ATTACTCCAGTGAGTG	CC1
SMC09.T.ATTACTCCATTTCACT	CC1
SMC09.T.ATTATCCAGAGCTGCA	CC1
SMC09.T.ATTATCCAGAGCTTCT	CC2
SMC09.T.ATTATCCAGGCAGGTT	CC2
SMC09.T.ATTATCCAGTGCGATG	CC1
SMC09.T.ATTATCCCAGGACCCT	CC1
SMC09.T.ATTATCCTCTGCCCTA	CC1

SMC09.T.ATTCTACAGAGACTTA	CC1
SMC09.T.ATTCTACAGCCGTCGT	CC1
SMC09.T.ATTCTACGTCGCGGTT	CC1
SMC09.T.ATTCTACGTTGGTAAA	CC1
SMC09.T.ATTGGACAGACCCACC	CC1
SMC09.T.ATTGGACCACAACTGT	CC1
SMC09.T.ATTGGACCAGACAAAT	CC1
SMC09.T.ATTGGACGTCGGCATC	CC1
SMC09.T.ATTGGTGAGATCCTGT	CC1
SMC09.T.ATTGGTGAGCTAGTCT	CC1
SMC09.T.ATTGGTGCACCAGCAC	CC1
SMC09.T.ATTGGTGGTATGCTTG	CC1
SMC09.T.ATTGGTGTCCAAAGTC	CC1
SMC09.T.ATTGGTGTCTTACCTA	CC1
SMC09.T.ATTTCTGCAATGAATG	CC1
SMC09.T.ATTTCTGGTCACAAGG	CC1
SMC09.T.ATTTCTGTCATGTCCC	CC1
SMC09.T.ATTTCTGTCCTGCCAT	CC1
SMC09.T.CAACCAAAGGACAGCT	CC1
SMC09.T.CAACCAACATGGTTGT	CC1
SMC09.T.CAACCAACATTCCTGC	CC1
SMC09.T.CAACCTCCATTCTCAT	CC1
SMC09.T.CAACCTCGTACTTAGC	CC1
SMC09.T.CAACCTCTCAGTGTTG	CC1
SMC09.T.CAACCTCTCGGAAATA	CC2
SMC09.T.CAACTAGCACGGTTTA	CC1
SMC09.T.CAACTAGCACTACAGT	CC1
SMC09.T.CAACTAGCAGACGTAG	CC1
SMC09.T.CAACTAGGTGGACGAT	CC1
SMC09.T.CAACTAGTCTTGCAAG	CC1
SMC09.T.CAAGAAAGTGCGGTAA	CC1
SMC09.T.CAAGATCCAAGCTGTT	CC1
SMC09.T.CAAGATCGTCTAGAGG	CC1
SMC09.T.CAAGATCGTGTGGTTT	CC1
SMC09.T.CAAGGCCAGATCCTGT	CC1
SMC09.T.CAAGGCCTCGAATGCT	CC1
SMC09.T.CACAAACCAATGCCAT	CC1
SMC09.T.CACAAACTCACGATGT	CC1
SMC09.T.CACAAACTCGGTCTAA	CC1
SMC09.T.CACACAAGTCTTCAAG	CC1
SMC09.T.CACACCTAGGTGCTAG	CC1
SMC09.T.CACACCTCAAAGGCGT	CC1
SMC09.T.CACACCTCAATCTACG	CC1
SMC09.T.CACACCTCACTAAGTC	CC2
SMC09.T.CACACCTTCAACACTG	CC1
SMC09.T.CACACTCAGCCGATTT	CC1
SMC09.T.CACACTCCACGTCAGC	CC1
SMC09.T.CACACTCCAGACGCCT	CC1
SMC09.T.CACAGGCGTAAGAGGA	CC1
SMC09.T.CACAGTAAGAATGTGT	CC1
SMC09.T.CACAGTAAGATGTCGG	CC1
SMC09.T.CACAGTAAGGTTCCTA	CC1
SMC09.T.CACAGTACAAGCGTAG	CC1
SMC09.T.CACAGTACACTCGACG	CC2
SMC09.T.CACAGTACATCTATGG	CC1
SMC09.T.CACAGTAGTACTCGCG	CC1
SMC09.T.CACAGTATCCTGCCAT	CC1
SMC09.T.CACAGTATCGGTTCGG	CC1

SMC09.T.CACATAGCAGTAAGCG	CC1
SMC09.T.CACATAGCATGAACCT	CC1
SMC09.T.CACATAGGTCATACTG	CC1
SMC09.T.CACATAGTCTGTCAAG	CC1
SMC09.T.CACATTTAGCTAACAA	CC1
SMC09.T.CACATTTGTAGTACCT	CC1
SMC09.T.CACATTTGTCATTAGC	CC1
SMC09.T.CACATTTGTTTACTCT	CC1
SMC09.T.CACATTTTCGGAAATA	CC1
SMC09.T.CACCACTAGAGGTACC	CC1
SMC09.T.CACCACTGTACAAGTA	CC1
SMC09.T.CACCAGGAGAAGAAGC	CC1
SMC09.T.CACCAGGCAAGGACTG	CC1
SMC09.T.CACCAGGGTCCAGTAT	CC1
SMC09.T.CACCAGGGTTCGTTGA	CC1
SMC09.T.CACCAGGTCACTATTC	CC1
SMC09.T.CACCAGGTCGGAAATA	CC1
SMC09.T.CACCTTGCATTTCAGG	CC1
SMC09.T.CACCTTGGTCCTGCTT	CC1
SMC09.T.CACCTTGTCGCGTTTC	CC1
SMC09.T.CACTCCAAGTAGATGT	CC1
SMC09.T.CACTCCAGTAAGGGCT	CC1
SMC09.T.CACTCCAGTCAAACTC	CC1
SMC09.T.CACTCCAGTCAACATC	CC2
SMC09.T.CAGAATCAGTGGACGT	CC1
SMC09.T.CAGAATCCACAACGTT	CC1
SMC09.T.CAGAATCCATCAGTAC	CC1
SMC09.T.CAGAATCGTGCCTGTG	CC1
SMC09.T.CAGAATCTCATGTCCC	CC1
SMC09.T.CAGAATCTCGGATGGA	CC1
SMC09.T.CAGAGAGCAGTCGATT	CC2
SMC09.T.CAGAGAGGTAGGAGTC	CC1
SMC09.T.CAGAGAGGTCTACCTC	CC2
SMC09.T.CAGATCAAGGGTGTGT	CC1
SMC09.T.CAGCAGCAGCCTCGTG	CC1
SMC09.T.CAGCAGCGTCAAACTC	CC1
SMC09.T.CAGCAGCGTCCGTGAC	CC2
SMC09.T.CAGCAGCGTGACCAAG	CC1
SMC09.T.CAGCATACATATGGTC	CC1
SMC09.T.CAGCATATCAAACGGG	CC1
SMC09.T.CAGCCGAAGTACTTGC	CC2
SMC09.T.CAGCCGACACTAAGTC	CC1
SMC09.T.CAGCCGAGTACAGTTC	CC2
SMC09.T.CAGCCGAGTTCGTGAT	CC1
SMC09.T.CAGCCGATCAGCACAT	CC2
SMC09.T.CAGCGACCATGGGAAC	CC1
SMC09.T.CAGCGACGTAAGCACG	CC1
SMC09.T.CAGCGACTCCTAGAAC	CC1
SMC09.T.CAGCGACTCTGCCAGG	CC1
SMC09.T.CAGCTAAAGACAGGCT	CC1
SMC09.T.CAGCTAACACTGTGTA	CC2
SMC09.T.CAGCTAAGTAGGCATG	CC1
SMC09.T.CAGCTAATCAGCTCTC	CC1
SMC09.T.CAGCTAATCGAACGGA	CC1
SMC09.T.CAGCTGGGTGTTTGGT	CC1
SMC09.T.CAGCTGGTCTTGCATT	CC1
SMC09.T.CAGGTGCAGCTAACAA	CC1
SMC09.T.CAGGTGCAGGTGCACA	CC1

SMC09.T.CAGGTGCAGTACGATA	CC1
SMC09.T.CAGGTGCCACGGTAGA	CC1
SMC09.T.CAGGTGCGTAAACACA	CC1
SMC09.T.CAGTAACCAGTATGCT	CC1
SMC09.T.CAGTAACGTCGGATCC	CC1
SMC09.T.CAGTAACGTGCAACGA	CC1
SMC09.T.CAGTAACTCCGTTGTC	CC1
SMC09.T.CAGTCCTAGGCTATCT	CC2
SMC09.T.CAGTCCTCACTGCCAG	CC1
SMC09.T.CAGTCCTGTAGAAAGG	CC1
SMC09.T.CAGTCCTGTTGTTTGG	CC1
SMC09.T.CAGTCCTTCAAGAAGT	CC1
SMC09.T.CAGTCCTTCGGATGGA	CC1
SMC09.T.CATATGGAGTAGTGCG	CC1
SMC09.T.CATATGGAGTGGGATC	CC1
SMC09.T.CATATGGTCGAACTGT	CC1
SMC09.T.CATATGGTCGCGGATC	CC2
SMC09.T.CATATGGTCGTCCGTT	CC1
SMC09.T.CATATTCAGCTGTTCA	CC1
SMC09.T.CATATTCCAATGCCAT	CC1
SMC09.T.CATATTCGTGGCTCCA	CC1
SMC09.T.CATATTCTCAGCTGGC	CC1
SMC09.T.CATCAAGAGAAACCGC	CC1
SMC09.T.CATCAAGCACGAAACG	CC1
SMC09.T.CATCAAGCACGTCAGC	CC1
SMC09.T.CATCAAGTCTCAAACG	CC2
SMC09.T.CATCAGACAAGGACAC	CC2
SMC09.T.CATCAGAGTAGGACAC	CC2
SMC09.T.CATCAGAGTCAAAGCG	CC1
SMC09.T.CATCAGAGTTGCCTCT	CC1
SMC09.T.CATCAGATCAAACCGT	CC1
SMC09.T.CATCCACAGGTAGCTG	CC1
SMC09.T.CATCCACCATGTCCTC	CC1
SMC09.T.CATCGAAAGGATTCGG	CC1
SMC09.T.CATCGAACATACTCTT	CC1
SMC09.T.CATCGAAGTTAAGAAC	CC1
SMC09.T.CATCGAATCAGCGACC	CC1
SMC09.T.CATCGAATCTCAACTT	CC1
SMC09.T.CATCGGGCACGCTTTC	CC1
SMC09.T.CATCGGGCAGCCACCA	CC2
SMC09.T.CATCGGGCATGAACCT	CC1
SMC09.T.CATCGGGGTCTAACGT	CC1
SMC09.T.CATCGGGGTGCTCTTC	CC1
SMC09.T.CATCGGGTCACTTACT	CC1
SMC09.T.CATGACAAGACTAGAT	CC1
SMC09.T.CATGCCTAGAGTCGGT	CC1
SMC09.T.CATGCCTAGCAATCTC	CC2
SMC09.T.CATGCCTAGCCGTCGT	CC2
SMC09.T.CATGCCTGTGCAGTAG	CC1
SMC09.T.CATGCCTGTTGAGTTC	CC2
SMC09.T.CATGGCGCAAACTGCT	CC1
SMC09.T.CATGGCGCAGAGTGTG	CC1
SMC09.T.CATGGCGCAGCTGGCT	CC1
SMC09.T.CATGGCGCATACTACG	CC1
SMC09.T.CATGGCGGTCTTGCGG	CC1
SMC09.T.CATTATCAGGACTGGT	CC2
SMC09.T.CATTATCCAGATGGGT	CC1
SMC09.T.CATTATCCAGGACGTA	CC1

SMC09.T.CATTATCGTACTTGAC	CC1
SMC09.T.CATTATCGTTGGAGGT	CC1
SMC09.T.CATTCGCCATCCGTGG	CC2
SMC09.T.CATTCGCGTAGGACAC	CC1
SMC09.T.CATTCGCGTTCTGTTT	CC1
SMC09.T.CATTCGCTCGGATGTT	CC1
SMC09.T.CCAATCCAGGATGGTC	CC2
SMC09.T.CCAATCCAGGCCGAAT	CC1
SMC09.T.CCAATCCCACGAGGTA	CC2
SMC09.T.CCAATCCGTATGGTTC	CC2
SMC09.T.CCAATCCGTCTAGGTT	CC1
SMC09.T.CCAATCCGTCTAGTCA	CC1
SMC09.T.CCACCTAAGGTCATCT	CC1
SMC09.T.CCACGGAAGGGTTCCC	CC1
SMC09.T.CCACGGACACTACAGT	CC1
SMC09.T.CCACGGATCTACGAGT	CC1
SMC09.T.CCACTACCAAGGACAC	CC1
SMC09.T.CCACTACTCAGCATGT	CC1
SMC09.T.CCACTACTCGCCCTTA	CC1
SMC09.T.CCAGCGAAGAAACGCC	CC1
SMC09.T.CCAGCGACACCACGTG	CC1
SMC09.T.CCAGCGACACCATCCT	CC1
SMC09.T.CCAGCGAGTATGGTTC	CC1
SMC09.T.CCAGCGAGTCATCGGC	CC2
SMC09.T.CCAGCGATCCGCATCT	CC1
SMC09.T.CCAGCGATCGCCATAA	CC1
SMC09.T.CCAGCGATCTGTCTCG	CC1
SMC09.T.CCATGTCAGGACTGGT	CC1
SMC09.T.CCATGTCGTCCGAATT	CC1
SMC09.T.CCATGTCTCTGAGGGA	CC1
SMC09.T.CCATTCGAGAAGGTGA	CC1
SMC09.T.CCATTCGGTAGCACGA	CC1
SMC09.T.CCATTCGGTGCGCTTG	CC1
SMC09.T.CCATTCGGTTCAACCA	CC1
SMC09.T.CCCAATCCATGACATC	CC1
SMC09.T.CCCAATCGTAGCCTCG	CC1
SMC09.T.CCCAGTTAGGGTGTTG	CC2
SMC09.T.CCCAGTTTCCCAGGTG	CC1
SMC09.T.CCCATACGTCGGCATC	CC1
SMC09.T.CCCATACTCACCCGAG	CC2
SMC09.T.CCCTCCTCAGCTCGAC	CC1
SMC09.T.CCCTCCTGTCAAGCGA	CC1
SMC09.T.CCCTCCTGTGTCGCTG	CC1
SMC09.T.CCGGGATGTGACTACT	CC1
SMC09.T.CCGGTAGCATGTCGAT	CC1
SMC09.T.CCGGTAGGTCAGCTAT	CC1
SMC09.T.CCGGTAGTCACGAAGG	CC2
SMC09.T.CCGGTAGTCCTACAGA	CC1
SMC09.T.CCGTACTAGTAGGCCA	CC1
SMC09.T.CCGTACTCAATCACAC	CC2
SMC09.T.CCGTGGAAGAGATGAG	CC1
SMC09.T.CCGTGGAAGTTGTCGT	CC1
SMC09.T.CCGTGGAGTCCATCCT	CC1
SMC09.T.CCGTTCACACCTTGTC	CC1
SMC09.T.CCGTTCAGTCAAGCGA	CC1
SMC09.T.CCGTTCAGTGTATGGG	CC1
SMC09.T.CCTAAAGCAATGAATG	CC1
SMC09.T.CCTAAAGCAATGGACG	CC1

SMC09.T.CCTACACAGAGCTATA	CC1
SMC09.T.CCTACACAGCAATCTC	CC1
SMC09.T.CCTACACAGCACCGTC	CC1
SMC09.T.CCTACACAGTAGATGT	CC1
SMC09.T.CCTACACCAAACCTAC	CC1
SMC09.T.CCTACACGTTATTCTC	CC1
SMC09.T.CCTACACGTTGCCTCT	CC1
SMC09.T.CCTACACTCCAAGTAC	CC1
SMC09.T.CCTAGCTAGATATGCA	CC1
SMC09.T.CCTAGCTCATGGAATA	CC1
SMC09.T.CCTAGCTTCACTTACT	CC1
SMC09.T.CCTATTATCGGGAGTA	CC1
SMC09.T.CCTCAGTAGGATGGAA	CC1
SMC09.T.CCTCAGTAGGTGATTA	CC1
SMC09.T.CCTCAGTAGTCTCCTC	CC1
SMC09.T.CCTCAGTCAAGAAGAG	CC1
SMC09.T.CCTCAGTGTCATCGGC	CC1
SMC09.T.CCTCTGAGTGTTGGGA	CC1
SMC09.T.CCTTACGAGTCATGCT	CC1
SMC09.T.CCTTACGGTAAACCTC	CC1
SMC09.T.CCTTACGTCATAGCAC	CC2
SMC09.T.CCTTACGTCGTCTGCT	CC2
SMC09.T.CCTTCCCGTAAGGGCT	CC1
SMC09.T.CCTTCCCTCCTAGAAC	CC1
SMC09.T.CCTTCGAAGTTCCACA	CC1
SMC09.T.CCTTCGACACGGCGTT	CC1
SMC09.T.CCTTCGAGTATGGTTC	CC1
SMC09.T.CCTTCGATCTTTAGGG	CC1
SMC09.T.CCTTTCTAGCTGTCTA	CC1
SMC09.T.CCTTTCTAGTAACCCT	CC1
SMC09.T.CCTTTCTCAAGCCATT	CC1
SMC09.T.CCTTTCTCATCACAAC	CC1
SMC09.T.CCTTTCTGTGAGGCTA	CC1
SMC09.T.CCTTTCTTCGGATGGA	CC2
SMC09.T.CGAACATAGGATGGTC	CC1
SMC09.T.CGAACATTCCGCATCT	CC1
SMC09.T.CGACCTTAGTGTCCAT	CC2
SMC09.T.CGACCTTGTAAATGTG	CC1
SMC09.T.CGACCTTGTAGCTGCC	CC1
SMC09.T.CGACCTTGTCATATCG	CC2
SMC09.T.CGACCTTTCTTGTATC	CC1
SMC09.T.CGACTTCCATGGTAGG	CC1
SMC09.T.CGAGAAGAGCGTTGCC	CC1
SMC09.T.CGAGAAGAGTCATCCA	CC1
SMC09.T.CGAGAAGAGTGTTTGC	CC1
SMC09.T.CGAGAAGCATTCCTGC	CC1
SMC09.T.CGAGAAGGTTCCTCCA	CC1
SMC09.T.CGAGAAGGTTTGGGCC	CC1
SMC09.T.CGAGCACAGGACAGCT	CC1
SMC09.T.CGAGCACGTAAGGGAA	CC1
SMC09.T.CGAGCACGTACATGTC	CC1
SMC09.T.CGAGCACGTACGCACC	CC1
SMC09.T.CGAGCACGTGAGTGAC	CC1
SMC09.T.CGAGCACTCAGAGACG	CC2
SMC09.T.CGAGCACTCGGAATCT	CC2
SMC09.T.CGAGCACTCTGAGTGT	CC1
SMC09.T.CGAGCACTCTGCTTGC	CC1
SMC09.T.CGAGCCAAGATCACGG	CC1

SMC09.T.CGAGCCAGTTCCACAA	CC1
SMC09.T.CGAGCCATCCAAAGTC	CC1
SMC09.T.CGATCGGCAATAACGA	CC1
SMC09.T.CGATGGCCACAGAGGT	CC2
SMC09.T.CGATGGCTCGTCCGTT	CC2
SMC09.T.CGATGTACACGAAACG	CC1
SMC09.T.CGATGTATCTATCGCC	CC1
SMC09.T.CGATTGAAGATCCGAG	CC1
SMC09.T.CGATTGACAATAGCAA	CC1
SMC09.T.CGATTGACACCCATTC	CC1
SMC09.T.CGATTGAGTACATGTC	CC1
SMC09.T.CGATTGAGTTCTGGTA	CC2
SMC09.T.CGATTGATCACTCCTG	CC1
SMC09.T.CGCCAAGAGACGCTTT	CC1
SMC09.T.CGCCAAGAGAGTAATC	CC1
SMC09.T.CGCCAAGCACAAGACG	CC2
SMC09.T.CGCCAAGCAGATCTGT	CC1
SMC09.T.CGCCAAGCATACCATG	CC1
SMC09.T.CGCCAAGCATACTACG	CC1
SMC09.T.CGCCAAGGTAAGGATT	CC1
SMC09.T.CGCCAAGGTAGCGTAG	CC1
SMC09.T.CGCCAAGTCCCTCAGT	CC1
SMC09.T.CGCCAAGTCTTCGGTC	CC1
SMC09.T.CGCGGTAAGACTTGAA	CC1
SMC09.T.CGCGGTAGTCATATCG	CC1
SMC09.T.CGCGTTTAGTGGTCCC	CC1
SMC09.T.CGCGTTTTCTACGAGT	CC1
SMC09.T.CGCTATCAGATCTGAA	CC1
SMC09.T.CGCTATCGTCATATCG	CC1
SMC09.T.CGCTATCTCCGAATGT	CC1
SMC09.T.CGCTGGAAGCAATCTC	CC1
SMC09.T.CGCTGGAGTCCATCCT	CC1
SMC09.T.CGCTGGAGTGAAAGAG	CC1
SMC09.T.CGCTGGATCGGTGTTA	CC1
SMC09.T.CGCTGGATCTTGGGTA	CC1
SMC09.T.CGCTTCAAGCCCAGCT	CC1
SMC09.T.CGCTTCACATCCGTGG	CC1
SMC09.T.CGCTTCAGTTGGAGGT	CC1
SMC09.T.CGGACACAGAGCCTAG	CC1
SMC09.T.CGGACACAGATGCCTT	CC1
SMC09.T.CGGACACAGGCAAAGA	CC1
SMC09.T.CGGACACCATCCGTGG	CC1
SMC09.T.CGGACACCATGGTCAT	CC2
SMC09.T.CGGACACGTGTTGAGG	CC1
SMC09.T.CGGACGTAGAGGTTAT	CC1
SMC09.T.CGGACGTAGGTGTGGT	CC1
SMC09.T.CGGACGTCACGTCTCT	CC1
SMC09.T.CGGACGTCAGGTCCAC	CC1
SMC09.T.CGGACGTGTGGTTTCA	CC1
SMC09.T.CGGAGCTAGAGTCTGG	CC1
SMC09.T.CGGAGCTAGCTGCAAG	CC1
SMC09.T.CGGAGCTGTCGCTTCT	CC1
SMC09.T.CGGAGCTGTCTTCGTC	CC1
SMC09.T.CGGAGCTGTGTGAATA	CC1
SMC09.T.CGGAGCTGTTTGTTGG	CC1
SMC09.T.CGGAGCTTCACCGGGT	CC2
SMC09.T.CGGAGCTTCGCAAGCC	CC1
SMC09.T.CGGAGTCAGTACGCCC	CC1

SMC09.T.CGGAGTCCATCCGGGT	CC1
SMC09.T.CGGAGTCTCAGTGTTG	CC1
SMC09.T.CGGCTAGAGCACGCCT	CC1
SMC09.T.CGGCTAGAGCGTGAAC	CC1
SMC09.T.CGGCTAGAGTTGCAGG	CC1
SMC09.T.CGGCTAGCAATCAGAA	CC1
SMC09.T.CGGCTAGCAGACTCGC	CC1
SMC09.T.CGGCTAGGTGTGACGA	CC1
SMC09.T.CGGGTCAAGCCACGCT	CC1
SMC09.T.CGGGTCAAGGTTCCTA	CC2
SMC09.T.CGGGTCACACAGAGGT	CC1
SMC09.T.CGGGTCACACTGTGTA	CC2
SMC09.T.CGGGTCAGTGTAATGA	CC1
SMC09.T.CGGGTCATCCGGGTGT	CC1
SMC09.T.CGGGTCATCTTGTTTG	CC1
SMC09.T.CGGTTAACACATGGGA	CC1
SMC09.T.CGGTTAACACCAGATT	CC1
SMC09.T.CGGTTAACAGCGTAAG	CC1
SMC09.T.CGGTTAACAGTAAGAT	CC1
SMC09.T.CGGTTAACATGATCCA	CC1
SMC09.T.CGGTTAAGTGTAAGTA	CC1
SMC09.T.CGGTTAAGTTCCGGCA	CC1
SMC09.T.CGGTTAATCCCAACGG	CC1
SMC09.T.CGTAGCGAGGCGCTCT	CC1
SMC09.T.CGTAGGCAGATGTGGC	CC1
SMC09.T.CGTAGGCAGTTCGCAT	CC1
SMC09.T.CGTAGGCCACAGTCGC	CC1
SMC09.T.CGTAGGCTCGGTGTTA	CC1
SMC09.T.CGTCACTAGTGAAGTT	CC1
SMC09.T.CGTCACTCAGATGGGT	CC1
SMC09.T.CGTCACTGTCAGATAA	CC1
SMC09.T.CGTCACTTCGATAGAA	CC1
SMC09.T.CGTCACTTCGATCCCT	CC1
SMC09.T.CGTCACTTCTACCTGC	CC1
SMC09.T.CGTCAGGAGGCATGTG	CC1
SMC09.T.CGTCAGGGTATAAACG	CC1
SMC09.T.CGTCAGGTCGCGGATC	CC1
SMC09.T.CGTCCATAGGAGTCTG	CC1
SMC09.T.CGTCCATGTCTTTCAT	CC1
SMC09.T.CGTCCATTCGTACCGG	CC1
SMC09.T.CGTCCATTCGTTACGA	CC1
SMC09.T.CGTCTACCAAACCCAT	CC1
SMC09.T.CGTCTACTCACCACCT	CC2
SMC09.T.CGTGAGCAGATATACG	CC1
SMC09.T.CGTGAGCGTAGTGAAT	CC1
SMC09.T.CGTGAGCTCCATTCTA	CC1
SMC09.T.CGTGAGCTCTCTGTCG	CC2
SMC09.T.CGTGTAACATTCTTAC	CC1
SMC09.T.CGTGTAAGTCCGACGT	CC1
SMC09.T.CGTGTAAGTGAGGGTT	CC1
SMC09.T.CGTGTAAGTTACGCGC	CC1
SMC09.T.CGTGTCTTCAGCGATT	CC1
SMC09.T.CGTTAGAAGGTCATCT	CC1
SMC09.T.CGTTAGACAGGGTATG	CC1
SMC09.T.CGTTAGAGTCTTCGTC	CC1
SMC09.T.CGTTCTGAGGGTTCCC	CC1
SMC09.T.CGTTCTGCATGCCCGA	CC1
SMC09.T.CGTTCTGTCTAACCGA	CC1

SMC09.T.CGTTCTGTCTGCCCTA	CC1
SMC09.T.CGTTGGGCACGGCCAT	CC1
SMC09.T.CGTTGGGCAGATGGGT	CC1
SMC09.T.CGTTGGGGTCCGAATT	CC1
SMC09.T.CGTTGGGTCTGCGTAA	CC2
SMC09.T.CTAACTTAGAGACGAA	CC1
SMC09.T.CTAACTTTCATCTGTT	CC1
SMC09.T.CTAAGACGTTCACGGC	CC2
SMC09.T.CTAAGACTCAGTGCAT	CC1
SMC09.T.CTAAGACTCGCGCCAA	CC2
SMC09.T.CTAATGGAGAACTGTA	CC1
SMC09.T.CTAATGGCAAAGCAAT	CC2
SMC09.T.CTAATGGTCAAGAAGT	CC1
SMC09.T.CTAATGGTCCAATGGT	CC2
SMC09.T.CTAATGGTCCCATTAT	CC1
SMC09.T.CTACACCGTGTCGCTG	CC1
SMC09.T.CTACATTCATTCCTGC	CC1
SMC09.T.CTACATTGTCGCATCG	CC1
SMC09.T.CTACATTGTCTAAAGA	CC1
SMC09.T.CTACATTGTGACGGTA	CC1
SMC09.T.CTACCCACAATCCAAC	CC1
SMC09.T.CTACCCACAGGAACGT	CC1
SMC09.T.CTACCCAGTCCGACGT	CC1
SMC09.T.CTACCCAGTCGGCATC	CC1
SMC09.T.CTACCCAGTCTAAACC	CC1
SMC09.T.CTACCCATCACCACCT	CC1
SMC09.T.CTACCCATCCGAATGT	CC1
SMC09.T.CTACGTCCACAGTCGC	CC1
SMC09.T.CTACGTCGTTAGTGGG	CC1
SMC09.T.CTACGTCTCCCACTTG	CC1
SMC09.T.CTACGTCTCTTATCTG	CC1
SMC09.T.CTAGAGTAGGCCCTTG	CC1
SMC09.T.CTAGAGTCAGGGAGAG	CC2
SMC09.T.CTAGAGTCATAACCTG	CC1
SMC09.T.CTAGAGTGTTAAGATG	CC1
SMC09.T.CTAGAGTTCTGTTTGT	CC1
SMC09.T.CTAGCCTAGGACTGGT	CC1
SMC09.T.CTAGCCTGTGCACGAA	CC1
SMC09.T.CTAGCCTGTGGGTCAA	CC1
SMC09.T.CTAGCCTTCCCACTTG	CC1
SMC09.T.CTAGCCTTCTACGAGT	CC1
SMC09.T.CTAGTGACAATAAGCA	CC1
SMC09.T.CTAGTGACATGGTCTA	CC1
SMC09.T.CTAGTGATCATCTGTT	CC1
SMC09.T.CTCACACAGCAATATG	CC1
SMC09.T.CTCACACAGCAGACTG	CC1
SMC09.T.CTCACACAGCCTCGTG	CC2
SMC09.T.CTCACACAGTACACCT	CC1
SMC09.T.CTCACACCAAGCTGAG	CC1
SMC09.T.CTCACACTCATATCGG	CC1
SMC09.T.CTCACACTCCGAAGAG	CC1
SMC09.T.CTCACACTCTTCGGTC	CC1
SMC09.T.CTCAGAAAGCTGCCCA	CC1
SMC09.T.CTCAGAAGTTACAGAA	CC1
SMC09.T.CTCAGAAGTTATGTGC	CC1
SMC09.T.CTCAGAAGTTCCCTTG	CC1
SMC09.T.CTCATTAAGTCATCCA	CC1
SMC09.T.CTCATTACATTGAGCT	CC1

SMC09.T.CTCATTAGTCCAGTTA	CC1
SMC09.T.CTCATTAGTGCACGAA	CC1
SMC09.T.CTCATTATCGCGGATC	CC1
SMC09.T.CTCCTAGAGCGCTCCA	CC2
SMC09.T.CTCCTAGAGTCCCACG	CC1
SMC09.T.CTCCTAGAGTCCGGTC	CC1
SMC09.T.CTCCTAGCAACACCTA	CC1
SMC09.T.CTCCTAGCAGCGTCCA	CC2
SMC09.T.CTCCTAGGTTCAGTAC	CC1
SMC09.T.CTCCTAGTCCGCGTTT	CC1
SMC09.T.CTCGAAACAGGTGGAT	CC1
SMC09.T.CTCGAAAGTAAGTGTA	CC2
SMC09.T.CTCGAAAGTCAAAGCG	CC1
SMC09.T.CTCGAGGGTCAAAGCG	CC1
SMC09.T.CTCGAGGGTCTTCGTC	CC1
SMC09.T.CTCGAGGGTTAAGGGC	CC1
SMC09.T.CTCGAGGTCCACGAAT	CC1
SMC09.T.CTCGAGGTCCAGTAGT	CC1
SMC09.T.CTCGGAGAGCTAGGCA	CC1
SMC09.T.CTCGGAGAGTGTACCT	CC1
SMC09.T.CTCGGAGCAAGCGTAG	CC1
SMC09.T.CTCGGAGCACTGCCAG	CC1
SMC09.T.CTCGGAGCATGGGAAC	CC1
SMC09.T.CTCGGAGGTAAGGGAA	CC1
SMC09.T.CTCGGAGGTCCTCTTG	CC1
SMC09.T.CTCGGAGGTTTGTGTG	CC1
SMC09.T.CTCGGAGTCTGTCCGT	CC1
SMC09.T.CTCGGGACACCCATGG	CC2
SMC09.T.CTCGGGACATAGAAAC	CC1
SMC09.T.CTCGTACCACATCCAA	CC1
SMC09.T.CTCGTACGTGCAACTT	CC1
SMC09.T.CTCGTACTCATGTCTT	CC1
SMC09.T.CTCGTACTCCGCATAA	CC2
SMC09.T.CTCGTCAAGCTGTTCA	CC1
SMC09.T.CTCGTCAAGGCGATAC	CC1
SMC09.T.CTCGTCAGTCCGTTAA	CC1
SMC09.T.CTCGTCAGTTACGACT	CC1
SMC09.T.CTCGTCAGTTGCCTCT	CC1
SMC09.T.CTCGTCATCACATAGC	CC1
SMC09.T.CTCGTCATCCTCATTA	CC1
SMC09.T.CTCGTCATCGGCTTGG	CC1
SMC09.T.CTCGTCATCTAACTGG	CC1
SMC09.T.CTCGTCATCTGAGTGT	CC2
SMC09.T.CTCTAATAGAGCTGGT	CC1
SMC09.T.CTCTAATAGTTCGATC	CC1
SMC09.T.CTCTAATGTCAATACC	CC1
SMC09.T.CTCTAATGTCTGATCA	CC1
SMC09.T.CTCTAATGTTGGGACA	CC1
SMC09.T.CTCTAATTCCTTAATC	CC1
SMC09.T.CTCTAATTCGATCCCT	CC1
SMC09.T.CTCTACGCAATAGAGT	CC1
SMC09.T.CTCTACGGTGACTCAT	CC1
SMC09.T.CTCTACGGTGTGTGCC	CC1
SMC09.T.CTCTACGTCACAATGC	CC1
SMC09.T.CTCTACGTCCGTACAA	CC1
SMC09.T.CTCTGGTAGTCCAGGA	CC1
SMC09.T.CTCTGGTTCGTCACGG	CC1
SMC09.T.CTCTGGTTCTCCAGGG	CC1

SMC09.T.CTGAAACAGGAGTTTA	CC1
SMC09.T.CTGAAACCATAGACTC	CC1
SMC09.T.CTGAAACTCACAATGC	CC1
SMC09.T.CTGAAGTAGCCGTCGT	CC1
SMC09.T.CTGAAGTCACAGACAG	CC1
SMC09.T.CTGAAGTGTACCAGTT	CC1
SMC09.T.CTGAAGTTCTCCAGGG	CC1
SMC09.T.CTGATAGAGCAGCCTC	CC1
SMC09.T.CTGATAGCATTAGGCT	CC1
SMC09.T.CTGATAGGTTCGTGAT	CC1
SMC09.T.CTGATAGTCCAAACAC	CC1
SMC09.T.CTGATCCAGAAACCGC	CC1
SMC09.T.CTGATCCGTAGAAAGG	CC2
SMC09.T.CTGATCCGTGTATGGG	CC1
SMC09.T.CTGATCCGTTGTCGCG	CC1
SMC09.T.CTGATCCTCATAGCAC	CC1
SMC09.T.CTGCCTAAGTGCCATT	CC2
SMC09.T.CTGCCTACAGCATGAG	CC1
SMC09.T.CTGCCTATCGTAGATC	CC1
SMC09.T.CTGCGGAAGATCACGG	CC1
SMC09.T.CTGCGGAAGCAGCCTC	CC2
SMC09.T.CTGCGGACATATGAGA	CC1
SMC09.T.CTGCGGACATCGATGT	CC1
SMC09.T.CTGCTGTAGTAATCCC	CC1
SMC09.T.CTGCTGTCAATGGTCT	CC2
SMC09.T.CTGCTGTGTCCGTTAA	CC1
SMC09.T.CTGGTCTCAAACCTAC	CC1
SMC09.T.CTGGTCTCATGGTCTA	CC2
SMC09.T.CTGGTCTGTCATGCCG	CC1
SMC09.T.CTGGTCTTCACCTTAT	CC1
SMC09.T.CTGGTCTTCCCTTGCA	CC1
SMC09.T.CTGTGCTAGGAATTAC	CC1
SMC09.T.CTGTGCTTCAACACGT	CC1
SMC09.T.CTGTTTAAGATGTAAC	CC1
SMC09.T.CTGTTTAAGCATCATC	CC1
SMC09.T.CTGTTTAAGGGATCTG	CC1
SMC09.T.CTGTTTACATGGAATA	CC2
SMC09.T.CTTAACTTCGTAGGTT	CC1
SMC09.T.CTTAACTTCTGCCCTA	CC1
SMC09.T.CTTACCGAGACCTTTG	CC1
SMC09.T.CTTACCGGTAAGAGGA	CC1
SMC09.T.CTTAGGAAGCTCAACT	CC1
SMC09.T.CTTAGGACATATACGC	CC1
SMC09.T.CTTAGGACATATGGTC	CC1
SMC09.T.CTTAGGAGTACTCTCC	CC1
SMC09.T.CTTCTCTAGCACGCCT	CC1
SMC09.T.CTTCTCTCAAGCGATG	CC1
SMC09.T.CTTCTCTCAGTAACGG	CC1
SMC09.T.CTTCTCTTCTTAGCCC	CC1
SMC09.T.CTTGGCTTCCACGTGG	CC1
SMC09.T.CTTGGCTTCTGCGACG	CC1
SMC09.T.CTTTGCGAGACAATAC	CC1
SMC09.T.CTTTGCGGTGCAGTAG	CC1
SMC09.T.CTTTGCGGTGTGACGA	CC2
SMC09.T.CTTTGCGGTTGCTCCT	CC1
SMC09.T.CTTTGCGTCAGCAACT	CC1
SMC09.T.CTTTGCGTCGAGGTAG	CC1
SMC09.T.GAAACTCAGATAGTCA	CC1

SMC09.T.GAAACTCAGTCCGGTC	CC1
SMC09.T.GAAACTCTCAGCTGGC	CC1
SMC09.T.GAAACTCTCATCGATG	CC1
SMC09.T.GAAACTCTCTCTGTCG	CC1
SMC09.T.GAAATGAAGACCACGA	CC1
SMC09.T.GAAATGAAGTACGATA	CC1
SMC09.T.GAAATGATCTTCTGGC	CC1
SMC09.T.GAACATCCAGGATCGA	CC1
SMC09.T.GAACATCTCTTGCCGT	CC1
SMC09.T.GAACATCTCTTTACGT	CC1
SMC09.T.GAACCTAAGAAGATTC	CC1
SMC09.T.GAACCTACAATACGCT	CC1
SMC09.T.GAACCTAGTTAAGGGC	CC1
SMC09.T.GAACCTATCGCTTAGA	CC1
SMC09.T.GAACGGAAGACTACAA	CC1
SMC09.T.GAACGGACAATGGATA	CC1
SMC09.T.GAACGGACACGAGGTA	CC1
SMC09.T.GAACGGATCATGCTCC	CC1
SMC09.T.GAAGCAGAGATGGCGT	CC1
SMC09.T.GAAGCAGAGCAATCTC	CC1
SMC09.T.GAAGCAGGTAACGCGA	CC1
SMC09.T.GAAGCAGTCCGTCAAA	CC1
SMC09.T.GAAGCAGTCCTCAACC	CC2
SMC09.T.GAATAAGAGAACAACT	CC1
SMC09.T.GAATAAGAGACTGGGT	CC1
SMC09.T.GAATAAGAGCTTTGGT	CC1
SMC09.T.GAATAAGAGGTTCCTA	CC1
SMC09.T.GAATAAGTCATACGGT	CC1
SMC09.T.GAATGAACAATCGGTT	CC1
SMC09.T.GAATGAACAGGTTTCA	CC1
SMC09.T.GAATGAAGTTCCACAA	CC1
SMC09.T.GACAGAGAGAGTTGGC	CC1
SMC09.T.GACAGAGAGCTAAACA	CC1
SMC09.T.GACAGAGGTACTCAAC	CC2
SMC09.T.GACAGAGGTCATACTG	CC1
SMC09.T.GACAGAGTCAAGATCC	CC1
SMC09.T.GACCAATAGGTGGGTT	CC1
SMC09.T.GACCAATAGGTTCCTA	CC1
SMC09.T.GACCAATGTCTCTCTG	CC1
SMC09.T.GACCAATTCCGCAGTG	CC1
SMC09.T.GACCAATTCTATCCTA	CC2
SMC09.T.GACCAATTCTGAAAGA	CC1
SMC09.T.GACCTGGGTTCCATGA	CC1
SMC09.T.GACCTGGTCTGAGTGT	CC1
SMC09.T.GACGCGTCACCCATGG	CC1
SMC09.T.GACGCGTTCTGGAGCC	CC1
SMC09.T.GACGGCTGTACAGCAG	CC1
SMC09.T.GACGGCTGTCCAGTTA	CC1
SMC09.T.GACGGCTGTGCAACGA	CC1
SMC09.T.GACGGCTGTGTTTGTG	CC2
SMC09.T.GACGGCTTCACATACG	CC2
SMC09.T.GACGGCTTCTCCAGGG	CC1
SMC09.T.GACGTGCTCAACTCTT	CC1
SMC09.T.GACGTTACACCACCAG	CC1
SMC09.T.GACGTTAGTGTTCTTT	CC1
SMC09.T.GACTAACCATGCATGT	CC1
SMC09.T.GACTAACGTTACCAGT	CC1
SMC09.T.GACTAACGTTTGACAC	CC1

SMC09.T.GACTAACTCCAATGGT	CC1
SMC09.T.GACTAACTCCCTAATT	CC2
SMC09.T.GACTACAAGCTAGGCA	CC2
SMC09.T.GACTACACACCACCAG	CC2
SMC09.T.GACTACACAGTGGGAT	CC1
SMC09.T.GACTACAGTAAGTGTA	CC1
SMC09.T.GACTACAGTGCACCAC	CC1
SMC09.T.GACTGCGCAAGCGCTC	CC1
SMC09.T.GACTGCGGTCTGCAAT	CC1
SMC09.T.GACTGCGTCTCTGTCG	CC1
SMC09.T.GAGCAGACAGCGTCCA	CC1
SMC09.T.GAGCAGACATAGAAAC	CC1
SMC09.T.GAGCAGACATGAGCGA	CC1
SMC09.T.GAGCAGAGTGTTGGGA	CC1
SMC09.T.GAGGTGACACACAGAG	CC1
SMC09.T.GAGGTGAGTACGACCC	CC1
SMC09.T.GAGGTGATCACCCGAG	CC1
SMC09.T.GAGGTGATCCTATGTT	CC2
SMC09.T.GAGGTGATCTTCCTTC	CC1
SMC09.T.GAGTCCGAGTCCAGGA	CC1
SMC09.T.GAGTCCGCACTCGACG	CC1
SMC09.T.GAGTCCGGTTACGCGC	CC2
SMC09.T.GAGTCCGGTTGGAGGT	CC1
SMC09.T.GAGTCCGTCGTAGATC	CC1
SMC09.T.GATCAGTAGTGAATTG	CC1
SMC09.T.GATCAGTCATGAACCT	CC1
SMC09.T.GATCAGTGTGTGGCTC	CC2
SMC09.T.GATCAGTGTTCTGTTT	CC1
SMC09.T.GATCGATAGAGTCGGT	CC2
SMC09.T.GATCGATCAATGGTCT	CC2
SMC09.T.GATCGATCATGTTCCC	CC1
SMC09.T.GATCGATGTAAGAGAG	CC2
SMC09.T.GATCGATTCGCGGATC	CC2
SMC09.T.GATCGCGAGCAGATCG	CC2
SMC09.T.GATCGCGAGGCTATCT	CC1
SMC09.T.GATCGCGTCCTCTAGC	CC1
SMC09.T.GATCGTAAGACTAGAT	CC1
SMC09.T.GATCGTAAGCGGCTTC	CC1
SMC09.T.GATCGTAAGGGAGTAA	CC1
SMC09.T.GATCTAGCATGAACCT	CC1
SMC09.T.GATCTAGGTACCCAAT	CC1
SMC09.T.GATCTAGTCGCCTGAG	CC1
SMC09.T.GATGAAAAGGTCATCT	CC1
SMC09.T.GATGAAACATGCAATC	CC1
SMC09.T.GATGAAAGTCCGCTGA	CC1
SMC09.T.GATGAAATCCTGTACC	CC1
SMC09.T.GATGAGGCATCGGACC	CC1
SMC09.T.GATGAGGGTCAGGACA	CC1
SMC09.T.GATGAGGTCCTACAGA	CC1
SMC09.T.GATGCTAAGATGGGTC	CC1
SMC09.T.GATGCTAAGTCGATAA	CC2
SMC09.T.GATGCTACAGGACCCT	CC1
SMC09.T.GATGCTACAGTCGATT	CC1
SMC09.T.GATGCTATCAGTTAGC	CC1
SMC09.T.GATGCTATCCCAGGTG	CC1
SMC09.T.GATGCTATCGGCGGTT	CC1
SMC09.T.GATTCAGAGATATGCA	CC2
SMC09.T.GATTCAGAGCGTTTAC	CC1

SMC09.T.GATTCAGCAATGGAAT	CC1
SMC09.T.GATTCAGGTCTCAACA	CC1
SMC09.T.GATTCAGGTTAAGGGC	CC1
SMC09.T.GCAAACTGTATTAGCC	CC1
SMC09.T.GCAATCAAGCGCTCCA	CC1
SMC09.T.GCAATCACAATACGCT	CC2
SMC09.T.GCAATCACATTTGCTT	CC1
SMC09.T.GCACATAGTCTTTCAT	CC1
SMC09.T.GCACATAGTGCCTGCA	CC1
SMC09.T.GCACTCTAGATGCCTT	CC1
SMC09.T.GCACTCTAGCGTGAAC	CC1
SMC09.T.GCAGCCAAGCACAGGT	CC1
SMC09.T.GCAGCCACAAGCGATG	CC1
SMC09.T.GCAGCCAGTAATTGGA	CC1
SMC09.T.GCAGCCAGTGACTACT	CC1
SMC09.T.GCAGCCAGTTATCGGT	CC1
SMC09.T.GCAGCCAGTTCAGACT	CC1
SMC09.T.GCAGCCATCTTGTACT	CC1
SMC09.T.GCAGTTAAGAGCTATA	CC1
SMC09.T.GCAGTTAAGGAGTAGA	CC1
SMC09.T.GCAGTTAGTTCGTCTC	CC1
SMC09.T.GCATACAAGGCTCAGA	CC1
SMC09.T.GCATACACAAACTGCT	CC2
SMC09.T.GCATACACACATTTCT	CC1
SMC09.T.GCATACACAGGATCGA	CC1
SMC09.T.GCATACACATAAGACA	CC1
SMC09.T.GCATACAGTCGAATCT	CC1
SMC09.T.GCATACATCAGCTGGC	CC1
SMC09.T.GCATACATCCGCGCAA	CC1
SMC09.T.GCATGATGTCAGTGGA	CC1
SMC09.T.GCATGCGGTCTCAACA	CC1
SMC09.T.GCATGCGGTGTTGAGG	CC1
SMC09.T.GCATGCGTCTCCCTGA	CC1
SMC09.T.GCATGCGTCTTGCAAG	CC2
SMC09.T.GCATGTAAGAGTAATC	CC1
SMC09.T.GCATGTAAGGCACATG	CC1
SMC09.T.GCATGTACACTAAGTC	CC1
SMC09.T.GCCAAATGTGAAAGAG	CC1
SMC09.T.GCCAAATGTGCTCTTC	CC1
SMC09.T.GCCAAATTCCTATGTT	CC1
SMC09.T.GCCAAATTCTCTTATG	CC1
SMC09.T.GCCAAATTCTGGTATG	CC1
SMC09.T.GCCTCTAAGAGTTGGC	CC1
SMC09.T.GCCTCTACAATAACGA	CC1
SMC09.T.GCCTCTACAGTAAGCG	CC1
SMC09.T.GCCTCTACATCCCATC	CC1
SMC09.T.GCGACCAAGGATTCGG	CC1
SMC09.T.GCGACCAGTAAACGCG	CC1
SMC09.T.GCGACCATCAGTTTGG	CC1
SMC09.T.GCGACCATCCAGGGCT	CC1
SMC09.T.GCGAGAAAGCCAGTAG	CC1
SMC09.T.GCGAGAACAAGGCTCC	CC1
SMC09.T.GCGAGAACATCTGGTA	CC1
SMC09.T.GCGAGAAGTCGACTAT	CC2
SMC09.T.GCGAGAAGTCGCATCG	CC1
SMC09.T.GCGAGAATCCTTCAAT	CC1
SMC09.T.GCGCAACTCAATAAGG	CC1
SMC09.T.GCGCAACTCGTTACGA	CC1

SMC09.T.GCGCAGTAGAGACGAA	CC1
SMC09.T.GCGCAGTCACGACTCG	CC1
SMC09.T.GCGCAGTCACGGACAA	CC1
SMC09.T.GCGCAGTCATTACGAC	CC1
SMC09.T.GCGCAGTGTTGCGCAC	CC1
SMC09.T.GCGCAGTTCAATAAGG	CC1
SMC09.T.GCGCAGTTCTTGCAAG	CC1
SMC09.T.GCGCCAAAGTGGGCTA	CC1
SMC09.T.GCGCCAAAGTTCGATC	CC1
SMC09.T.GCGCCAACATGACGGA	CC1
SMC09.T.GCGCCAAGTGTCAATC	CC1
SMC09.T.GCGCCAAGTTACGTCA	CC1
SMC09.T.GCGCGATTCATAAAGG	CC2
SMC09.T.GCGCGATTCCCTCTTT	CC1
SMC09.T.GCGCGATTCGTCACGG	CC1
SMC09.T.GCGGGTTCAGTCGTGC	CC1
SMC09.T.GCGGGTTGTCCGAAGA	CC1
SMC09.T.GCTCCTACACATTAGC	CC1
SMC09.T.GCTCCTAGTCGCCATG	CC1
SMC09.T.GCTCCTATCAAACAAG	CC1
SMC09.T.GCTCCTATCATCATTC	CC1
SMC09.T.GCTCCTATCCCATTAT	CC1
SMC09.T.GCTCCTATCTCGATGA	CC2
SMC09.T.GCTCTGTGTACATGTC	CC1
SMC09.T.GCTGCAGAGATCACGG	CC2
SMC09.T.GCTGCAGAGCTCCTCT	CC1
SMC09.T.GCTGCAGCATCTGGTA	CC1
SMC09.T.GCTGCAGTCTGGTATG	CC2
SMC09.T.GCTGCGAAGATGCGAC	CC1
SMC09.T.GCTGCGACACGAGAGT	CC1
SMC09.T.GCTGCGAGTATAGGTA	CC1
SMC09.T.GCTGCGATCTGTCTAT	CC2
SMC09.T.GCTGCTTCACAGGCCT	CC1
SMC09.T.GCTGCTTTCACTTCAT	CC1
SMC09.T.GCTGGGTAGGTTACCT	CC1
SMC09.T.GCTGGGTGTAAACACA	CC1
SMC09.T.GCTGGGTGTAAGTAGT	CC1
SMC09.T.GCTGGGTGTTAAAGTG	CC1
SMC09.T.GCTGGGTTCAGCTCTC	CC1
SMC09.T.GCTTCCACATGCCACG	CC1
SMC09.T.GCTTCCATCGGCTTGG	CC1
SMC09.T.GCTTGAACACATAACC	CC2
SMC09.T.GCTTGAAGTATATCCG	CC2
SMC09.T.GCTTGAAGTCTAAACC	CC1
SMC09.T.GCTTGAAGTGACAAAT	CC1
SMC09.T.GCTTGAATCAACCATG	CC1
SMC09.T.GGAAAGCAGGCTATCT	CC1
SMC09.T.GGAAAGCGTTACAGAA	CC1
SMC09.T.GGAAAGCTCTGCGACG	CC1
SMC09.T.GGAACTTAGCTGCAAG	CC1
SMC09.T.GGAACTTAGGGAACGG	CC1
SMC09.T.GGAACTTAGTTGAGAT	CC1
SMC09.T.GGAACTTGTGCCTTGG	CC1
SMC09.T.GGAATAAAGACCACGA	CC1
SMC09.T.GGAATAAAGCTCTCGG	CC2
SMC09.T.GGAATAACATTCACTT	CC1
SMC09.T.GGAATAAGTTAAAGTG	CC1
SMC09.T.GGAATAAGTTAAGATG	CC1

SMC09.T.GGAATAAGTTACGTCA	CC1
SMC09.T.GGAATAAGTTGGGACA	CC1
SMC09.T.GGACAAGAGAGCCTAG	CC1
SMC09.T.GGACAAGAGCGCCTCA	CC1
SMC09.T.GGACAAGGTTGTGGAG	CC1
SMC09.T.GGACAAGTCACCCGAG	CC1
SMC09.T.GGACAAGTCCTTTACA	CC1
SMC09.T.GGACAGAAGCAACGGT	CC2
SMC09.T.GGACAGAAGCTGAAAT	CC1
SMC09.T.GGACAGACAATGTTGC	CC1
SMC09.T.GGACAGAGTGCGAAAC	CC1
SMC09.T.GGACAGAGTTTGTTGG	CC1
SMC09.T.GGACAGATCGAATGGG	CC1
SMC09.T.GGACATTCAGATGAGC	CC2
SMC09.T.GGACATTCAGCTGCTG	CC1
SMC09.T.GGACATTCATCGGAAG	CC1
SMC09.T.GGACATTGTACAGTTC	CC1
SMC09.T.GGACGTCCAAGTTGTC	CC2
SMC09.T.GGACGTCGTCGACTGC	CC1
SMC09.T.GGACGTCGTTCGGCAC	CC1
SMC09.T.GGAGCAAAGAACAACT	CC1
SMC09.T.GGAGCAAAGCCCAGCT	CC1
SMC09.T.GGAGCAATCAAAGTAG	CC1
SMC09.T.GGATGTTAGTGGTAAT	CC2
SMC09.T.GGATGTTCACCGATAT	CC2
SMC09.T.GGATGTTCACGTCTCT	CC1
SMC09.T.GGATGTTGTGCAGTAG	CC1
SMC09.T.GGATGTTGTGTAACGG	CC1
SMC09.T.GGATGTTTCCTACAGA	CC1
SMC09.T.GGATGTTTCTACTATC	CC1
SMC09.T.GGATTACCATCACCCT	CC1
SMC09.T.GGATTACGTCCGAACC	CC1
SMC09.T.GGATTACGTCGAGTTT	CC1
SMC09.T.GGCAATTCAGCTTAAC	CC1
SMC09.T.GGCAATTCATTGTGCA	CC1
SMC09.T.GGCAATTTCTAAGCCA	CC1
SMC09.T.GGCCGATCACCACCAG	CC1
SMC09.T.GGCCGATGTTCAGCGC	CC1
SMC09.T.GGCGACTAGGCCCTTG	CC1
SMC09.T.GGCGACTAGTGTACTC	CC2
SMC09.T.GGCGACTAGTTCCACA	CC1
SMC09.T.GGCGACTCATGACATC	CC2
SMC09.T.GGCGACTGTAAGTAGT	CC1
SMC09.T.GGCGACTGTACCGCTG	CC1
SMC09.T.GGCGACTGTATATGGA	CC1
SMC09.T.GGCGACTTCATAGCAC	CC1
SMC09.T.GGCGTGTAGTGGAGAA	CC1
SMC09.T.GGCGTGTAGTGGTAAT	CC1
SMC09.T.GGCGTGTGTAGGACAC	CC1
SMC09.T.GGCTCGACACGACTCG	CC1
SMC09.T.GGCTCGACAGTCGATT	CC1
SMC09.T.GGCTCGACATACTCTT	CC1
SMC09.T.GGCTGGTGTCACCCAG	CC2
SMC09.T.GGCTGGTGTCCTCTTG	CC1
SMC09.T.GGCTGGTTCGTCTGCT	CC1
SMC09.T.GGCTGGTTCTTGTATC	CC2
SMC09.T.GGGAATGGTATCGCAT	CC2
SMC09.T.GGGAATGTCAACGAAA	CC1

SMC09.T.GGGACCTAGACCACGA	CC1
SMC09.T.GGGACCTAGCGCCTCA	CC1
SMC09.T.GGGACCTGTAAATACG	CC1
SMC09.T.GGGACCTTCATACGGT	CC1
SMC09.T.GGGAGATCAAACAACA	CC1
SMC09.T.GGGAGATCAAGCCCAC	CC1
SMC09.T.GGGAGATCATGACGGA	CC1
SMC09.T.GGGAGATCATGGTAGG	CC1
SMC09.T.GGGATGAAGTGATCGG	CC2
SMC09.T.GGGATGACACGTCAGC	CC1
SMC09.T.GGGATGAGTATATCCG	CC1
SMC09.T.GGGATGATCACCAGGC	CC1
SMC09.T.GGGATGATCCTAGTGA	CC1
SMC09.T.GGGATGATCGGCCGAT	CC1
SMC09.T.GGGCACTAGCCACTAT	CC1
SMC09.T.GGGCACTAGTTACGGG	CC1
SMC09.T.GGGCACTCACAGGAGT	CC1
SMC09.T.GGGCACTCACTACAGT	CC1
SMC09.T.GGGCACTGTTCGCGAC	CC1
SMC09.T.GGGCACTTCCAGTATG	CC1
SMC09.T.GGGCACTTCCATTCTA	CC1
SMC09.T.GGGCATCCACTACAGT	CC2
SMC09.T.GGGCATCGTACTCGCG	CC1
SMC09.T.GGGTCTGCAGATGGCA	CC1
SMC09.T.GGGTCTGCAGCCAATT	CC1
SMC09.T.GGGTCTGGTTACGGAG	CC1
SMC09.T.GGGTCTGTCTCCGGTT	CC1
SMC09.T.GGGTTGCAGAACAATC	CC2
SMC09.T.GGGTTGCAGTTAACGA	CC1
SMC09.T.GGGTTGCGTGATGTCT	CC1
SMC09.T.GGGTTGCTCCAAACTG	CC1
SMC09.T.GGGTTGCTCGCGTTTC	CC1
SMC09.T.GGTATTGAGAATCTCC	CC1
SMC09.T.GGTATTGAGAGACGAA	CC1
SMC09.T.GGTATTGCAGTCAGCC	CC2
SMC09.T.GGTATTGGTACTTCTT	CC1
SMC09.T.GGTATTGTCAGCTGGC	CC1
SMC09.T.GGTGAAGCACTATCTT	CC1
SMC09.T.GGTGAAGGTCTGATCA	CC1
SMC09.T.GGTGAAGTCAGTTTGG	CC1
SMC09.T.GGTGAAGTCGGTTCGG	CC1
SMC09.T.GGTGCGTGTAGCTGCC	CC1
SMC09.T.GGTGCGTGTTAGATGA	CC2
SMC09.T.GGTGCGTTCGCGATCG	CC1
SMC09.T.GGTGTTACATTACGAC	CC2
SMC09.T.GGTGTTAGTAACGACG	CC1
SMC09.T.GGTGTTAGTGCACCAC	CC2
SMC09.T.GGTGTTATCCGCGTTT	CC1
SMC09.T.GTAACGTAGAGTAATC	CC1
SMC09.T.GTAACGTAGGTGATTA	CC1
SMC09.T.GTAACGTAGTACGATA	CC1
SMC09.T.GTAACGTGTTCCTCCA	CC2
SMC09.T.GTAACTGAGAGATGAG	CC1
SMC09.T.GTAACTGCACGAAAGC	CC1
SMC09.T.GTAACTGTCCTTGACC	CC1
SMC09.T.GTACGTAAGGTAAACT	CC1
SMC09.T.GTACTCCAGACAATAC	CC1
SMC09.T.GTACTCCAGACTGTAA	CC1

SMC09.T.GTACTCCTCAGCTCGG	CC2
SMC09.T.GTACTCCTCATCTGCC	CC1
SMC09.T.GTACTTTTCCAGAAGG	CC1
SMC09.T.GTAGGCCAGCGTCTAT	CC1
SMC09.T.GTAGGCCAGGACAGAA	CC1
SMC09.T.GTAGGCCAGTTAGCGG	CC1
SMC09.T.GTAGGCCCAGGAATCG	CC1
SMC09.T.GTAGGCCTCTATCCCG	CC1
SMC09.T.GTAGTCAAGCTATGCT	CC1
SMC09.T.GTAGTCACACGCCAGT	CC1
SMC09.T.GTAGTCAGTGCCTTGG	CC1
SMC09.T.GTAGTCAGTTCCCGAG	CC1
SMC09.T.GTATCTTCAGCGTAAG	CC1
SMC09.T.GTATCTTGTAGGCATG	CC2
SMC09.T.GTATCTTGTGTATGGG	CC2
SMC09.T.GTATTCTAGGACGAAA	CC1
SMC09.T.GTATTCTCATTATCTC	CC1
SMC09.T.GTATTCTGTATCTGCA	CC1
SMC09.T.GTCAAGTAGCGATATA	CC1
SMC09.T.GTCAAGTCACCAGTTA	CC1
SMC09.T.GTCAAGTCATTACCTT	CC1
SMC09.T.GTCAAGTGTAGAAGGA	CC1
SMC09.T.GTCAAGTGTGTGTGCC	CC1
SMC09.T.GTCACAACACGAGAGT	CC1
SMC09.T.GTCACAAGTACGACCC	CC1
SMC09.T.GTCACAATCCTCATTA	CC1
SMC09.T.GTCACGGAGGACAGCT	CC1
SMC09.T.GTCACGGAGGCATTGG	CC1
SMC09.T.GTCACGGAGGTAGCCA	CC1
SMC09.T.GTCACGGCACTATCTT	CC1
SMC09.T.GTCACGGGTAGCGCAA	CC1
SMC09.T.GTCACGGTCTCGGACG	CC1
SMC09.T.GTCATTTAGACTCGGA	CC2
SMC09.T.GTCCTCAAGAAGATTC	CC1
SMC09.T.GTCCTCAAGACGCTTT	CC1
SMC09.T.GTCCTCAGTACCATCA	CC1
SMC09.T.GTCCTCAGTTCGGCAC	CC2
SMC09.T.GTCCTCATCGTCGTTC	CC1
SMC09.T.GTCGGGTAGTCGATAA	CC2
SMC09.T.GTCGGGTAGTGGGATC	CC1
SMC09.T.GTCGGGTGTACTTGAC	CC1
SMC09.T.GTCGGGTGTCCCTACT	CC2
SMC09.T.GTCGGGTGTGCAGACA	CC1
SMC09.T.GTCGTAACATTACCTT	CC1
SMC09.T.GTCGTAATCATAGCAC	CC1
SMC09.T.GTCTCGTAGTGGGTTG	CC1
SMC09.T.GTCTCGTGTTAAAGAC	CC1
SMC09.T.GTCTTCGAGGGAACGG	CC1
SMC09.T.GTCTTCGCACTTCGAA	CC1
SMC09.T.GTCTTCGCATCGATTG	CC1
SMC09.T.GTCTTCGTCTTGACGA	CC1
SMC09.T.GTGAAGGAGTGGGTTG	CC1
SMC09.T.GTGAAGGGTACAGACG	CC1
SMC09.T.GTGAAGGGTGGTCTCG	CC1
SMC09.T.GTGAAGGGTTATCCGA	CC1
SMC09.T.GTGAAGGGTTCTGGTA	CC1
SMC09.T.GTGCAGCCAGAGCCAA	CC1
SMC09.T.GTGCAGCGTAGGACAC	CC1

SMC09.T.GTGCAGCGTCATCCCT	CC1
SMC09.T.GTGCAGCGTCTCCACT	CC1
SMC09.T.GTGCAGCTCGAGAACG	CC1
SMC09.T.GTGCATACAGGATCGA	CC1
SMC09.T.GTGCATATCCACGAAT	CC1
SMC09.T.GTGCGGTAGATCCCAT	CC1
SMC09.T.GTGCGGTAGGACGAAA	CC1
SMC09.T.GTGCGGTCACATGACT	CC1
SMC09.T.GTGCGGTTCAGTCAGT	CC1
SMC09.T.GTGCTTCAGAATGTTG	CC1
SMC09.T.GTGCTTCAGTGTTGAA	CC1
SMC09.T.GTGCTTCCAATCTACG	CC1
SMC09.T.GTGCTTCGTTGTCGCG	CC1
SMC09.T.GTGCTTCTCTCTAAGG	CC1
SMC09.T.GTGGGTCCATTTGCTT	CC1
SMC09.T.GTGGGTCGTAGAGCTG	CC1
SMC09.T.GTGGGTCGTCGAAAGC	CC1
SMC09.T.GTGTGCGAGAGAACAG	CC1
SMC09.T.GTGTGCGAGTCCCACG	CC1
SMC09.T.GTGTGCGCAGGCGATA	CC1
SMC09.T.GTGTGCGTCAGTCAGT	CC2
SMC09.T.GTGTGCGTCATCACCC	CC1
SMC09.T.GTGTGCGTCATGTCTT	CC2
SMC09.T.GTGTGCGTCGTGACAT	CC1
SMC09.T.GTGTTAGAGTACATGA	CC1
SMC09.T.GTGTTAGCATACTACG	CC1
SMC09.T.GTTAAGCAGATCGGGT	CC2
SMC09.T.GTTAAGCCACCACCAG	CC1
SMC09.T.GTTAAGCGTTCAGGCC	CC1
SMC09.T.GTTAAGCTCTCAAACG	CC1
SMC09.T.GTTACAGAGAAGAAGC	CC1
SMC09.T.GTTACAGAGGGAACGG	CC1
SMC09.T.GTTACAGCAGGCTCAC	CC1
SMC09.T.GTTACAGGTATAGTAG	CC1
SMC09.T.GTTACAGGTGATAAGT	CC1
SMC09.T.GTTCATTCACAGGCCT	CC1
SMC09.T.GTTCATTCATGCCTAA	CC1
SMC09.T.GTTCATTTCCTAGAAC	CC2
SMC09.T.GTTCATTTCGCAAGCC	CC1
SMC09.T.GTTCGGGAGAAACCTA	CC1
SMC09.T.GTTCGGGAGGGTGTTG	CC1
SMC09.T.GTTCGGGCACGGTAAG	CC2
SMC09.T.GTTCGGGGTGTTTGTG	CC1
SMC09.T.GTTCGGGTCCGCGGTA	CC1
SMC09.T.GTTCTCGAGCCACTAT	CC1
SMC09.T.GTTCTCGCACTTCTGC	CC1
SMC09.T.GTTCTCGCATAGGATA	CC2
SMC09.T.GTTCTCGGTTCAGACT	CC1
SMC09.T.GTTCTCGTCATCTGCC	CC1
SMC09.T.GTTCTCGTCGGCTTGG	CC1
SMC09.T.GTTTCTAAGCGTGAGT	CC1
SMC09.T.GTTTCTAAGGCCCGTT	CC1
SMC09.T.GTTTCTACAAAGCAAT	CC1
SMC09.T.GTTTCTACATTGGGCC	CC1
SMC09.T.GTTTCTAGTAGGCATG	CC1
SMC09.T.GTTTCTAGTCGAACAG	CC2
SMC09.T.GTTTCTAGTGCTTCTC	CC1
SMC09.T.GTTTCTAGTGGCTCCA	CC1

SMC09.T.GTTTCTATCTTAACCT	CC1
SMC09.T.TAAACCGAGACAAAGG	CC1
SMC09.T.TAAACCGGTGCCTTGG	CC1
SMC09.T.TAAACCGGTGTGCCTG	CC2
SMC09.T.TAAGAGAAGTCATCCA	CC2
SMC09.T.TAAGAGACATAAAGGT	CC1
SMC09.T.TAAGAGACATACGCTA	CC1
SMC09.T.TAAGAGAGTAATCGTC	CC1
SMC09.T.TAAGAGAGTCATATCG	CC1
SMC09.T.TAAGAGATCAAACCGT	CC1
SMC09.T.TAAGAGATCCTTGCCA	CC1
SMC09.T.TAAGAGATCGTATCAG	CC1
SMC09.T.TAAGCGTAGGCTCTTA	CC1
SMC09.T.TAAGCGTCAAGCGTAG	CC1
SMC09.T.TAAGCGTTCAATAAGG	CC1
SMC09.T.TAAGTGCAGGTGCAAC	CC1
SMC09.T.TAAGTGCCACGGCCAT	CC2
SMC09.T.TAAGTGCCATCACCCT	CC2
SMC09.T.TACACGAAGGTGCTTT	CC1
SMC09.T.TACACGAGTCCGTTAA	CC2
SMC09.T.TACACGATCCGCGTTT	CC1
SMC09.T.TACACGATCTAACTGG	CC1
SMC09.T.TACACGATCTACTCAT	CC1
SMC09.T.TACACGATCTTGAGGT	CC1
SMC09.T.TACAGTGGTAAATGTG	CC1
SMC09.T.TACCTATAGCGATCCC	CC1
SMC09.T.TACCTATAGGTGATTA	CC1
SMC09.T.TACCTATCAATGGATA	CC1
SMC09.T.TACCTATCACTTCGAA	CC1
SMC09.T.TACCTATGTCAGATAA	CC1
SMC09.T.TACCTATGTTAGGGTG	CC1
SMC09.T.TACCTATTCGGCATCG	CC1
SMC09.T.TACCTATTCTTGAGAC	CC1
SMC09.T.TACCTTAAGGGTATCG	CC1
SMC09.T.TACCTTACACAAGTAA	CC1
SMC09.T.TACCTTACATCGGTTA	CC1
SMC09.T.TACGGATAGCTGAACG	CC1
SMC09.T.TACGGATAGGCACATG	CC1
SMC09.T.TACGGATAGGTTACCT	CC1
SMC09.T.TACGGGCAGTATTGGA	CC1
SMC09.T.TACGGGCCACGAAACG	CC1
SMC09.T.TACGGGCTCTTGAGAC	CC2
SMC09.T.TACGGTACACAGAGGT	CC1
SMC09.T.TACGGTACATAGAAAC	CC1
SMC09.T.TACGGTAGTAAGGGAA	CC1
SMC09.T.TACTCATCATTCACTT	CC1
SMC09.T.TACTCGCAGCCACCTG	CC1
SMC09.T.TACTCGCCAAATTGCC	CC1
SMC09.T.TACTCGCGTATGGTTC	CC1
SMC09.T.TACTCGCGTCACACGC	CC1
SMC09.T.TACTCGCGTCATGCCG	CC1
SMC09.T.TACTCGCTCCGAGCCA	CC1
SMC09.T.TACTTACGTTTCCACC	CC1
SMC09.T.TACTTGTAGAGCTTCT	CC2
SMC09.T.TACTTGTAGCCAGGAT	CC1
SMC09.T.TACTTGTCAAAGAATC	CC1
SMC09.T.TACTTGTGTATGGTTC	CC2
SMC09.T.TACTTGTTCCACTCCA	CC1

SMC09.T.TACTTGTTCCTAGGGC	CC1
SMC09.T.TAGACCAAGATCCGAG	CC1
SMC09.T.TAGACCAAGCCATCGC	CC1
SMC09.T.TAGACCAAGGCCCTTG	CC2
SMC09.T.TAGACCAAGTCCGGTC	CC1
SMC09.T.TAGACCACACCATGTA	CC1
SMC09.T.TAGACCACATTAGCCA	CC1
SMC09.T.TAGACCAGTAGAGTGC	CC1
SMC09.T.TAGACCATCTCAAGTG	CC2
SMC09.T.TAGAGCTAGGATGTAT	CC1
SMC09.T.TAGAGCTCAATAGAGT	CC1
SMC09.T.TAGAGCTCAGGACGTA	CC1
SMC09.T.TAGAGCTTCTTACCGC	CC1
SMC09.T.TAGAGCTTCTTGTCAT	CC1
SMC09.T.TAGCCGGCAGATCCAT	CC1
SMC09.T.TAGGCATCACTACAGT	CC1
SMC09.T.TAGGCATGTATAGGGC	CC1
SMC09.T.TAGGCATTCAAGGTAA	CC1
SMC09.T.TAGGCATTCCTGCTTG	CC1
SMC09.T.TAGTGGTAGTAGGCCA	CC1
SMC09.T.TAGTGGTTCTGCTGTC	CC1
SMC09.T.TAGTTGGAGCCGTCGT	CC1
SMC09.T.TAGTTGGCAGATAATG	CC1
SMC09.T.TAGTTGGTCATATCGG	CC1
SMC09.T.TATCAGGCACAGATTC	CC1
SMC09.T.TATCTCAAGAGACGAA	CC1
SMC09.T.TATCTCAAGCCAGTTT	CC2
SMC09.T.TATCTCACAATCAGAA	CC1
SMC09.T.TATCTCATCCTACAGA	CC1
SMC09.T.TATGCCCTCAGGCGAA	CC1
SMC09.T.TATGCCCTCTTCATGT	CC1
SMC09.T.TATTACCAGCCGGTAA	CC1
SMC09.T.TATTACCAGGCCATAG	CC1
SMC09.T.TATTACCCAATGGATA	CC1
SMC09.T.TATTACCGTAAAGGAG	CC1
SMC09.T.TATTACCGTAAATGTG	CC1
SMC09.T.TATTACCGTACTTAGC	CC1
SMC09.T.TATTACCGTTGAGTTC	CC1
SMC09.T.TATTACCTCAAGAAGT	CC1
SMC09.T.TCAACGACATCCGCGA	CC1
SMC09.T.TCAACGAGTACGAAAT	CC2
SMC09.T.TCAACGAGTGTCGCTG	CC1
SMC09.T.TCAACGATCCTAAGTG	CC1
SMC09.T.TCAATCTAGCGACGTA	CC1
SMC09.T.TCAATCTCATAGGATA	CC1
SMC09.T.TCAATCTCATGCCACG	CC1
SMC09.T.TCAATCTGTAGCGTCC	CC1
SMC09.T.TCAATCTTCATCATTC	CC2
SMC09.T.TCAATCTTCCCTCAGT	CC1
SMC09.T.TCAATCTTCTGCAAGT	CC1
SMC09.T.TCACAAGAGAAGGGTA	CC1
SMC09.T.TCACAAGAGGCAGTCA	CC1
SMC09.T.TCACAAGAGTCCCACG	CC2
SMC09.T.TCACAAGCATTATCTC	CC1
SMC09.T.TCACAAGTCACCTCGT	CC1
SMC09.T.TCACGAAAGGGCATGT	CC1
SMC09.T.TCACGAAAGTACTTGC	CC1
SMC09.T.TCACGAACACATAACC	CC1

SMC09.T.TCACGAATCCTTGACC	CC1
SMC09.T.TCAGATGCAGCCACCA	CC1
SMC09.T.TCAGATGGTTCCAACA	CC1
SMC09.T.TCAGATGTCTGAAAGA	CC2
SMC09.T.TCAGCAAAGTGAAGAG	CC2
SMC09.T.TCAGCAACAGTGGAGT	CC1
SMC09.T.TCAGCAAGTGATAAAC	CC1
SMC09.T.TCAGCAAGTTCGGCAC	CC1
SMC09.T.TCAGCAATCAGTACGT	CC1
SMC09.T.TCAGCTCAGGCTACGA	CC1
SMC09.T.TCAGCTCCAGACAGGT	CC1
SMC09.T.TCAGCTCGTCAACTGT	CC1
SMC09.T.TCAGCTCTCTGTCTAT	CC1
SMC09.T.TCAGGATCAGGCTGAA	CC1
SMC09.T.TCAGGTAAGGAACTGC	CC1
SMC09.T.TCAGGTACAAGCTGAG	CC1
SMC09.T.TCAGGTACACAGAGGT	CC1
SMC09.T.TCAGGTACATGGTTGT	CC1
SMC09.T.TCAGGTAGTGACCAAG	CC1
SMC09.T.TCAGGTAGTGCGCTTG	CC1
SMC09.T.TCAGGTATCAGGCCCA	CC1
SMC09.T.TCATTACCACAACGCC	CC1
SMC09.T.TCATTACCACGAGGTA	CC1
SMC09.T.TCATTACGTAGGACAC	CC1
SMC09.T.TCATTACTCAAACCGT	CC1
SMC09.T.TCATTACTCATGCAAC	CC1
SMC09.T.TCATTACTCCCGGATG	CC1
SMC09.T.TCATTACTCCGCGCAA	CC1
SMC09.T.TCATTTGAGAAACCAT	CC1
SMC09.T.TCATTTGAGATCGATA	CC1
SMC09.T.TCATTTGCAGTCTTCC	CC1
SMC09.T.TCATTTGTCGTTTATC	CC1
SMC09.T.TCCACACCAGTGGAGT	CC1
SMC09.T.TCCACACGTAGAGTGC	CC1
SMC09.T.TCCACACGTCTCCACT	CC1
SMC09.T.TCCCGATAGCCAGTAG	CC1
SMC09.T.TCCCGATAGGAGCGAG	CC2
SMC09.T.TCCCGATCAGATGGCA	CC1
SMC09.T.TCCCGATGTCTCCCTA	CC2
SMC09.T.TCCCGATTCAGTTAGC	CC2
SMC09.T.TCCCGATTCCATTCTA	CC1
SMC09.T.TCCCGATTCTGCGACG	CC1
SMC09.T.TCGAGGCAGAAGGTTT	CC1
SMC09.T.TCGAGGCCACCATCCT	CC1
SMC09.T.TCGAGGCGTTCGTTGA	CC1
SMC09.T.TCGAGGCTCAGAAATG	CC1
SMC09.T.TCGCGAGCACCGCTAG	CC1
SMC09.T.TCGCGAGCATGCAACT	CC1
SMC09.T.TCGCGAGGTAGCTAAA	CC1
SMC09.T.TCGCGAGGTGGTCTCG	CC1
SMC09.T.TCGCGAGTCAGCTCGG	CC1
SMC09.T.TCGCGAGTCTTGTACT	CC2
SMC09.T.TCGCGTTGTTGTGGCC	CC1
SMC09.T.TCGCGTTTCCGTAGGC	CC1
SMC09.T.TCGCGTTTCTGTCTCG	CC1
SMC09.T.TCGGGACCAATGAATG	CC1
SMC09.T.TCGGGACCAGTATCTG	CC1
SMC09.T.TCGGGACGTCCAAGTT	CC2

SMC09.T.TCGGGACTCACTCCTG	CC1
SMC09.T.TCGGGACTCGCCAAAT	CC1
SMC09.T.TCGGTAAAGTAGGCCA	CC1
SMC09.T.TCGGTAACAGCTCGCA	CC1
SMC09.T.TCGGTAAGTGGCCCTA	CC1
SMC09.T.TCGGTAATCATCGCTC	CC1
SMC09.T.TCGGTAATCGGAAATA	CC1
SMC09.T.TCGGTAATCTGTGCAA	CC1
SMC09.T.TCGTACCAGAGCCCAA	CC2
SMC09.T.TCGTACCAGTATCGAA	CC1
SMC09.T.TCGTACCCATCCGTGG	CC1
SMC09.T.TCGTACCGTTGCGCAC	CC1
SMC09.T.TCGTACCTCTACTTAC	CC1
SMC09.T.TCGTAGAAGACTAAGT	CC1
SMC09.T.TCGTAGACACACAGAG	CC1
SMC09.T.TCGTAGACATTATCTC	CC1
SMC09.T.TCGTAGATCTCGGACG	CC2
SMC09.T.TCTATTGCATACCATG	CC1
SMC09.T.TCTATTGCATATGAGA	CC2
SMC09.T.TCTATTGGTGGTTTCA	CC1
SMC09.T.TCTATTGTCTGCTGTC	CC1
SMC09.T.TCTCATAGTTACTGAC	CC1
SMC09.T.TCTGAGACACCTCGGA	CC1
SMC09.T.TCTGAGAGTTAAGAAC	CC2
SMC09.T.TCTGAGAGTTGGACCC	CC1
SMC09.T.TCTGAGATCTGATACG	CC1
SMC09.T.TCTGAGATCTTGTTTG	CC2
SMC09.T.TCTGGAAAGCTAGTTC	CC1
SMC09.T.TCTGGAAAGGTTCCTA	CC1
SMC09.T.TCTGGAACAAGCCGCT	CC1
SMC09.T.TCTGGAACAGTTCATG	CC1
SMC09.T.TCTGGAAGTCTTGCGG	CC1
SMC09.T.TCTGGAAGTTGTGGAG	CC1
SMC09.T.TCTTCGGAGAGGGATA	CC2
SMC09.T.TCTTCGGCAGATCTGT	CC1
SMC09.T.TCTTCGGTCAACACGT	CC1
SMC09.T.TCTTCGGTCCAAAGTC	CC1
SMC09.T.TGAAAGACAATCGAAA	CC1
SMC09.T.TGAAAGAGTAACGACG	CC1
SMC09.T.TGACAACAGTTTGCGT	CC1
SMC09.T.TGACAACCACATGTGT	CC1
SMC09.T.TGACAACCAGCTTCGG	CC1
SMC09.T.TGACAACTCGCGGATC	CC1
SMC09.T.TGACGGCAGAGTACAT	CC1
SMC09.T.TGACGGCCAGCCAGAA	CC1
SMC09.T.TGACGGCTCGAATGGG	CC1
SMC09.T.TGACGGCTCGCCATAA	CC1
SMC09.T.TGACGGCTCGTCACGG	CC1
SMC09.T.TGACTAGAGCCCGAAA	CC2
SMC09.T.TGACTAGCACGAAGCA	CC2
SMC09.T.TGACTAGGTACCTACA	CC1
SMC09.T.TGACTAGGTAGCACGA	CC1
SMC09.T.TGACTAGTCGTACGGC	CC1
SMC09.T.TGACTTTCACTAGTAC	CC2
SMC09.T.TGACTTTGTCCGTCAG	CC1
SMC09.T.TGACTTTTCCAACCAA	CC1
SMC09.T.TGAGAGGAGGATGTAT	CC1
SMC09.T.TGAGAGGCAAGGCTCC	CC1

SMC09.T.TGAGAGGCAGCGAACA	CC1
SMC09.T.TGAGAGGGTGGTGTAG	CC1
SMC09.T.TGAGAGGGTTGCGTTA	CC1
SMC09.T.TGAGCATAGAAACGAG	CC2
SMC09.T.TGAGCATAGAGACGAA	CC1
SMC09.T.TGAGCATAGCTCAACT	CC1
SMC09.T.TGAGCATCAAAGCGGT	CC1
SMC09.T.TGAGCATCACGAGGTA	CC1
SMC09.T.TGAGCCGCACAAGTAA	CC2
SMC09.T.TGAGCCGGTACAGCAG	CC1
SMC09.T.TGAGCCGGTATTCTCT	CC1
SMC09.T.TGAGCCGTCGGATGGA	CC1
SMC09.T.TGAGGGAGTCGCATCG	CC1
SMC09.T.TGATTTCAGGCTCAGA	CC1
SMC09.T.TGATTTCCACGTCAGC	CC1
SMC09.T.TGATTTCCAGCTGTGC	CC1
SMC09.T.TGATTTCGTAGCGATG	CC1
SMC09.T.TGATTTCGTCAGAAGC	CC1
SMC09.T.TGATTTCTCACCCGAG	CC1
SMC09.T.TGCACCTAGCAGGCTA	CC1
SMC09.T.TGCACCTGTCACCCAG	CC1
SMC09.T.TGCACCTGTCAGGACA	CC1
SMC09.T.TGCCAAATCATCTGCC	CC1
SMC09.T.TGCCCATCACTTAAGC	CC1
SMC09.T.TGCCCATCATTATCTC	CC1
SMC09.T.TGCCCATCATTCTCAT	CC1
SMC09.T.TGCCCTAAGTGGCACA	CC1
SMC09.T.TGCCCTAAGTGTTGAA	CC1
SMC09.T.TGCCCTACAATTGCTG	CC1
SMC09.T.TGCCCTACACGGTAGA	CC2
SMC09.T.TGCCCTAGTAAAGTCA	CC1
SMC09.T.TGCCCTAGTTGGTTTG	CC1
SMC09.T.TGCCCTATCCCTCAGT	CC1
SMC09.T.TGCCCTATCCGCGCAA	CC1
SMC09.T.TGCGCAGAGCCACGTC	CC1
SMC09.T.TGCGCAGCACATGACT	CC1
SMC09.T.TGCGCAGCACGACTCG	CC1
SMC09.T.TGCGCAGGTATGAAAC	CC1
SMC09.T.TGCGGGTAGACGCTTT	CC1
SMC09.T.TGCGGGTCACTTAACG	CC1
SMC09.T.TGCGGGTTCTTCAACT	CC2
SMC09.T.TGCGTGGAGTGTCTCA	CC1
SMC09.T.TGCGTGGTCCTGCTTG	CC1
SMC09.T.TGCTACCCAGACGCTC	CC1
SMC09.T.TGCTACCTCGGTCCGA	CC2
SMC09.T.TGCTACCTCGTTGACA	CC1
SMC09.T.TGCTGCTAGCTAAACA	CC1
SMC09.T.TGCTGCTAGGGATACC	CC1
SMC09.T.TGCTGCTCACCGAATT	CC1
SMC09.T.TGCTGCTTCGGTCTAA	CC2
SMC09.T.TGCTGCTTCTGCCCTA	CC1
SMC09.T.TGGACGCGTAAACCTC	CC1
SMC09.T.TGGACGCGTCTCATCC	CC1
SMC09.T.TGGACGCTCACGCATA	CC1
SMC09.T.TGGACGCTCTGGTATG	CC2
SMC09.T.TGGCCAGAGATGCGAC	CC1
SMC09.T.TGGCCAGAGCTAGTGG	CC1
SMC09.T.TGGCGCACAACTTGAC	CC1

SMC09.T.TGGCGCACACAGATTC	CC1
SMC09.T.TGGCGCACATCACGTA	CC1
SMC09.T.TGGCGCATCTTGCAAG	CC1
SMC09.T.TGGCTGGGTAAAGGAG	CC1
SMC09.T.TGGCTGGGTCGGATCC	CC2
SMC09.T.TGGCTGGGTCTAGAGG	CC1
SMC09.T.TGGCTGGTCGCAGGCT	CC2
SMC09.T.TGGGAAGGTCCGAACC	CC1
SMC09.T.TGGGAAGGTTAAGAAC	CC1
SMC09.T.TGGGAAGTCATTGCGA	CC1
SMC09.T.TGGGAAGTCCAAATGC	CC1
SMC09.T.TGGGCGTAGACGCTTT	CC1
SMC09.T.TGGGCGTAGCCGATTT	CC1
SMC09.T.TGGGCGTAGTTCCACA	CC1
SMC09.T.TGGGCGTCAAGGGTCA	CC1
SMC09.T.TGGGCGTGTACATGTC	CC1
SMC09.T.TGGGCGTGTTTGTTTC	CC1
SMC09.T.TGGTTAGAGACTCGGA	CC1
SMC09.T.TGGTTAGAGAGACTAT	CC1
SMC09.T.TGGTTAGAGCCAGAAC	CC1
SMC09.T.TGGTTAGAGCCAGTTT	CC1
SMC09.T.TGGTTAGAGGGTGTTG	CC1
SMC09.T.TGGTTAGCACGGTAAG	CC1
SMC09.T.TGGTTAGCAGCTCGAC	CC1
SMC09.T.TGGTTAGGTATTCTCT	CC1
SMC09.T.TGGTTCCGTTAAGACA	CC1
SMC09.T.TGGTTCCTCCAGTATG	CC1
SMC09.T.TGGTTCCTCTTTACAC	CC1
SMC09.T.TGTATTCAGAGCAATT	CC1
SMC09.T.TGTATTCAGCTTATCG	CC1
SMC09.T.TGTATTCCACCAGCAC	CC2
SMC09.T.TGTATTCTCATCTGCC	CC1
SMC09.T.TGTATTCTCATTGCGA	CC1
SMC09.T.TGTATTCTCCAGGGCT	CC1
SMC09.T.TGTCCCACACCACGTG	CC1
SMC09.T.TGTCCCACACGAAACG	CC1
SMC09.T.TGTCCCAGTGAGGGAG	CC1
SMC09.T.TGTCCCATCCGCAGTG	CC1
SMC09.T.TGTCCCATCTTGTATC	CC1
SMC09.T.TGTGGTACATTCTTAC	CC1
SMC09.T.TGTGTTTAGACAGGCT	CC1
SMC09.T.TGTGTTTAGGATGTAT	CC1
SMC09.T.TGTTCCGAGGACATTA	CC1
SMC09.T.TTAACTCAGCCACTAT	CC1
SMC09.T.TTAACTCAGCCTATGT	CC1
SMC09.T.TTAACTCGTAGCTAAA	CC1
SMC09.T.TTAGGACCAGATCCAT	CC1
SMC09.T.TTAGGACCATCGGACC	CC1
SMC09.T.TTAGGACCATGATCCA	CC1
SMC09.T.TTAGGACGTAGATTAG	CC1
SMC09.T.TTAGGCAAGAAACGCC	CC1
SMC09.T.TTAGGCACAAGACACG	CC1
SMC09.T.TTAGGCATCTCAAGTG	CC2
SMC09.T.TTAGTTCAGAAGGCCT	CC1
SMC09.T.TTAGTTCAGATCACGG	CC1
SMC09.T.TTAGTTCAGCGATTCT	CC2
SMC09.T.TTAGTTCAGCGGATCA	CC1
SMC09.T.TTAGTTCCATACCATG	CC1

SMC09.T.TTAGTTCGTCAAACTC	CC1
SMC09.T.TTAGTTCTCAAACCAC	CC1
SMC09.T.TTAGTTCTCGCAAACT	CC1
SMC09.T.TTAGTTCTCTGCGTAA	CC1
SMC09.T.TTATGCTAGAGGGATA	CC1
SMC09.T.TTATGCTAGCTAAACA	CC1
SMC09.T.TTCCCAGCAGGAATGC	CC1
SMC09.T.TTCCCAGCAGGTTTCA	CC1
SMC09.T.TTCCCAGGTATATGAG	CC1
SMC09.T.TTCCCAGTCAACCATG	CC1
SMC09.T.TTCCCAGTCTGGAGCC	CC1
SMC09.T.TTCGAAGAGACGCAAC	CC1
SMC09.T.TTCGAAGGTCACCTAA	CC1
SMC09.T.TTCGAAGTCAGATAAG	CC1
SMC09.T.TTCGGTCAGCTTTGGT	CC1
SMC09.T.TTCGGTCGTTACGCGC	CC1
SMC09.T.TTCTACAAGAAACGAG	CC1
SMC09.T.TTCTACAGTAGCGTCC	CC1
SMC09.T.TTCTACATCAAGAAGT	CC2
SMC09.T.TTCTCAAGTGCGCTTG	CC1
SMC09.T.TTCTCAATCAACACGT	CC1
SMC09.T.TTCTCAATCATGCTCC	CC1
SMC09.T.TTCTCAATCGCGCCAA	CC1
SMC09.T.TTCTCCTAGGCCATAG	CC1
SMC09.T.TTCTCCTCACATGTGT	CC1
SMC09.T.TTCTCCTCACTAGTAC	CC1
SMC09.T.TTCTCCTCATACCATG	CC2
SMC09.T.TTCTCCTCATGCCACG	CC2
SMC09.T.TTCTCCTGTTTAGGAA	CC2
SMC09.T.TTCTCCTTCGCAAGCC	CC1
SMC09.T.TTCTTAGAGGGATCTG	CC1
SMC09.T.TTCTTAGGTCTAAACC	CC1
SMC09.T.TTCTTAGTCGCTGATA	CC1
SMC09.T.TTGAACGCATCCTAGA	CC1
SMC09.T.TTGAACGGTATGAAAC	CC1
SMC09.T.TTGAACGGTCAGAAGC	CC1
SMC09.T.TTGAACGGTCCGAGTC	CC1
SMC09.T.TTGACTTCAACACGCC	CC1
SMC09.T.TTGACTTCACAAGTAA	CC1
SMC09.T.TTGCCGTAGAGGGCTT	CC1
SMC09.T.TTGCCGTAGCCCAGCT	CC1
SMC09.T.TTGCCGTGTTCTCATT	CC1
SMC09.T.TTGCGTCAGACGCACA	CC1
SMC09.T.TTGCGTCCAACCGCCA	CC1
SMC09.T.TTGCGTCCACCAGATT	CC1
SMC09.T.TTGCGTCCATCGTCGG	CC1
SMC09.T.TTGCGTCGTACGACCC	CC1
SMC09.T.TTGCGTCGTGAAAGAG	CC2
SMC09.T.TTGCGTCGTGATAAAC	CC1
SMC09.T.TTGCGTCGTTCAGACT	CC1
SMC09.T.TTGCGTCTCGACCAGC	CC2
SMC09.T.TTGCGTCTCTCTGTCG	CC1
SMC09.T.TTGGAACAGTACGTAA	CC1
SMC09.T.TTGGAACAGTGAATTG	CC1
SMC09.T.TTGGAACCAGACACTT	CC1
SMC09.T.TTGGAACGTTACGCGC	CC1
SMC09.T.TTGGAACGTTAGGGTG	CC1
SMC09.T.TTGGAACGTTGACGTT	CC1

SMC09.T.TTGGAACTCAACGGGA	CC1
SMC09.T.TTGGAACTCAATACCG	CC1
SMC09.T.TTGGAACTCCAGAAGG	CC1
SMC09.T.TTGGCAACAGTAAGAT	CC1
SMC09.T.TTGGCAAGTCGGCTCA	CC1
SMC09.T.TTGGCAATCTGTACGA	CC2
SMC09.T.TTGTAGGAGATATGGT	CC1
SMC09.T.TTGTAGGAGATCCCAT	CC1
SMC09.T.TTGTAGGCAATGGAGC	CC1
SMC09.T.TTGTAGGGTGTTTGGT	CC1
SMC09.T.TTGTAGGTCGGTTAAC	CC2
SMC09.T.TTTACTGAGGCATGTG	CC1
SMC09.T.TTTACTGCACCAGCAC	CC1
SMC09.T.TTTACTGCACTCTGTC	CC1
SMC09.T.TTTACTGCAGTCTTCC	CC1
SMC09.T.TTTACTGGTCTCACCT	CC1
SMC09.T.TTTACTGGTGTCAATC	CC2
SMC09.T.TTTACTGTCACGATGT	CC1
SMC09.T.TTTACTGTCCCGGATG	CC1
SMC09.T.TTTATGCAGCCAGTTT	CC1
SMC09.T.TTTATGCAGCGTAATA	CC1
SMC09.T.TTTATGCAGTGTGAAT	CC2
SMC09.T.TTTATGCAGTTGTCGT	CC2
SMC09.T.TTTATGCGTACTCGCG	CC1
SMC09.T.TTTATGCGTCAGGACA	CC1
SMC09.T.TTTATGCGTGGTGTAG	CC1
SMC09.T.TTTATGCTCAACACAC	CC1
SMC09.T.TTTATGCTCCCAAGTA	CC1
SMC09.T.TTTATGCTCCTAGAAC	CC1
SMC09.T.TTTCCTCAGGTTCCTA	CC1
SMC09.T.TTTCCTCGTCCAAGTT	CC1
SMC09.T.TTTCCTCGTTACGCGC	CC2
SMC09.T.TTTCCTCTCAATAAGG	CC1
SMC09.T.TTTGCGCGTTGAGGTG	CC1
SMC09.T.TTTGCGCTCAGCCTAA	CC1
SMC09.T.TTTGCGCTCATTCACT	CC1
SMC09.T.TTTGCGCTCTGTCAAG	CC1
SMC09.T.TTTGGTTAGGAATCGC	CC1
SMC09.T.TTTGGTTAGTTGAGAT	CC1
SMC09.T.TTTGGTTCATCGGACC	CC1
SMC09.T.TTTGGTTGTCTTCTCG	CC1
SMC09.T.TTTGGTTTCATATCGG	CC1
SMC09.T.TTTGGTTTCTTTACGT	CC1
SMC09.T.TTTGTCAAGATCACGG	CC1
SMC09.T.TTTGTCAAGTGGAGTC	CC1
SMC09.T.TTTGTCACATAAGACA	CC1
SMC09.T.TTTGTCACATCGACGC	CC1
SMC09.T.TTTGTCAGTTAGTGGG	CC2
SMC09.T.TTTGTCAGTTCAGTAC	CC1
SMC10.T.AAACCTGGTCACACGC	CC2
SMC10.T.AAACGGGGTACAGTGG	CC2
SMC10.T.AAAGATGCATATGGTC	CC2
SMC10.T.AAAGCAAAGCTTCGCG	CC2
SMC10.T.AAAGCAATCGCCCTTA	CC2
SMC10.T.AAAGTAGGTCTCAACA	CC2
SMC10.T.AAATGCCAGAAACGCC	CC1
SMC10.T.AACACGTCATGGTTGT	CC2
SMC10.T.AACACGTGTGAACCTT	CC2

SMC10.T.AACACGTTCAACTCTT	CC2
SMC10.T.AACACGTTCCAAGCCG	CC1
SMC10.T.AACCATGCAGCTCCGA	CC1
SMC10.T.AACCATGTCGTTACAG	CC1
SMC10.T.AACCGCGAGCTGATAA	CC2
SMC10.T.AACGTTGGTCGTCTTC	CC2
SMC10.T.AACTCTTGTGCGAAAC	CC2
SMC10.T.AACTCTTTCCTGCTTG	CC2
SMC10.T.AACTTTCAGGCCCGTT	CC2
SMC10.T.AACTTTCCACTGAAGG	CC1
SMC10.T.AAGACCTCAGATGGGT	CC2
SMC10.T.AAGCCGCAGCAGACTG	CC2
SMC10.T.AAGCCGCAGGGAACGG	CC2
SMC10.T.AAGCCGCTCAGTACGT	CC2
SMC10.T.AAGGAGCGTTTGACAC	CC2
SMC10.T.AAGGAGCTCTCAACTT	CC2
SMC10.T.AAGGTTCCACCGATAT	CC2
SMC10.T.AAGGTTCCAGCCTTTC	CC2
SMC10.T.AAGTCTGAGGCATGTG	CC2
SMC10.T.AAGTCTGGTCAAACTC	CC1
SMC10.T.AAGTCTGTCACGCATA	CC2
SMC10.T.ACACTGAAGCCACCTG	CC2
SMC10.T.ACACTGACACAGGTTT	CC2
SMC10.T.ACATACGAGGTGATAT	CC2
SMC10.T.ACATCAGCAGGTGGAT	CC2
SMC10.T.ACCAGTAAGAGTTGGC	CC1
SMC10.T.ACCAGTAGTTCGGCAC	CC2
SMC10.T.ACCAGTAGTTGCGCAC	CC1
SMC10.T.ACCAGTATCAAACGGG	CC2
SMC10.T.ACCAGTATCCGATATG	CC2
SMC10.T.ACCCACTGTATGAAAC	CC1
SMC10.T.ACCGTAATCCACGTGG	CC2
SMC10.T.ACCTTTATCTGCAAGT	CC2
SMC10.T.ACGAGCCGTGGTTTCA	CC2
SMC10.T.ACGATACCAGGTGCCT	CC2
SMC10.T.ACGATACTCAGAGCTT	CC2
SMC10.T.ACGCAGCTCAACACAC	CC1
SMC10.T.ACGCCAGAGCGACGTA	CC1
SMC10.T.ACGGAGAAGTTAGGTA	CC2
SMC10.T.ACGGAGAGTACGCTGC	CC2
SMC10.T.ACGGGCTAGAGTGACC	CC2
SMC10.T.ACGGGTCAGGATGTAT	CC2
SMC10.T.ACGGGTCCATTGTGCA	CC2
SMC10.T.ACGGGTCTCCCAGGTG	CC2
SMC10.T.ACGTCAAGTTGGACCC	CC2
SMC10.T.ACTATCTCATGACGGA	CC2
SMC10.T.ACTGAGTAGCGTGAGT	CC2
SMC10.T.ACTGAGTTCATTGCCC	CC2
SMC10.T.ACTGATGAGAGTGAGA	CC2
SMC10.T.ACTGATGGTCAACATC	CC2
SMC10.T.ACTGCTCCATCAGTCA	CC2
SMC10.T.ACTGCTCCATGCCACG	CC2
SMC10.T.ACTGCTCTCTTCATGT	CC1
SMC10.T.ACTGTCCCACATGTGT	CC2
SMC10.T.ACTTACTAGTAGCCGA	CC1
SMC10.T.ACTTGTTTCGGCTTGG	CC2
SMC10.T.AGAATAGAGGTTACCT	CC2
SMC10.T.AGAATAGTCCTGTAGA	CC1

SMC10.T.AGACGTTGTCTCATCC	CC2
SMC10.T.AGAGCGACACATTCGA	CC2
SMC10.T.AGAGCGACAGTGACAG	CC2
SMC10.T.AGAGCGATCAGATAAG	CC2
SMC10.T.AGAGTGGCACACATGT	CC2
SMC10.T.AGAGTGGGTATGAAAC	CC2
SMC10.T.AGAGTGGGTCTAACGT	CC2
SMC10.T.AGAGTGGTCTCGGACG	CC2
SMC10.T.AGATTGCAGCTCAACT	CC2
SMC10.T.AGCAGCCGTAATCGTC	CC2
SMC10.T.AGCATACTCAAGGCTT	CC2
SMC10.T.AGCGTCGAGCTCCTTC	CC1
SMC10.T.AGCTCCTGTTGCGTTA	CC2
SMC10.T.AGCTCTCGTACCGAGA	CC2
SMC10.T.AGCTTGAAGATGTTAG	CC2
SMC10.T.AGCTTGAGTGGTTTCA	CC2
SMC10.T.AGGGATGCATCATCCC	CC2
SMC10.T.AGGGATGGTCCAGTAT	CC2
SMC10.T.AGGTCATGTAAGTGGC	CC2
SMC10.T.AGTAGTCTCACAGGCC	CC2
SMC10.T.AGTAGTCTCGTAGATC	CC2
SMC10.T.AGTCTTTAGATATGCA	CC2
SMC10.T.AGTCTTTCATGTTCCC	CC1
SMC10.T.AGTCTTTTCGGTCTAA	CC2
SMC10.T.AGTGAGGTCTCAACTT	CC2
SMC10.T.AGTGGGATCACAGGCC	CC1
SMC10.T.AGTGTCAAGTCCATAC	CC2
SMC10.T.ATAACGCGTATCGCAT	CC2
SMC10.T.ATAAGAGCAGAGCCAA	CC1
SMC10.T.ATAGACCCACGGATAG	CC2
SMC10.T.ATAGACCGTCAGGACA	CC1
SMC10.T.ATCCACCGTAGCGTCC	CC1
SMC10.T.ATCCGAAAGTTCCACA	CC2
SMC10.T.ATCCGAATCCCTTGTG	CC2
SMC10.T.ATCTACTAGGACACCA	CC2
SMC10.T.ATCTACTCACATGACT	CC2
SMC10.T.ATCTGCCAGGGATCTG	CC1
SMC10.T.ATGAGGGGTGTCCTCT	CC1
SMC10.T.ATGCGATCAGGATTGG	CC2
SMC10.T.ATGGGAGCATGTAAGA	CC1
SMC10.T.ATGTGTGCAAGGCTCC	CC2
SMC10.T.ATGTGTGTCGCCTGTT	CC2
SMC10.T.ATTACTCAGGGTTCCC	CC2
SMC10.T.ATTGGACAGTTACCCA	CC2
SMC10.T.ATTGGACTCTCTGTCG	CC2
SMC10.T.ATTTCTGGTCGGCACT	CC2
SMC10.T.CAACCTCCATCGATGT	CC1
SMC10.T.CAAGAAAAGTTTCCTT	CC2
SMC10.T.CAAGATCTCATGTGGT	CC2
SMC10.T.CAAGTTGCAGCGTTCG	CC2
SMC10.T.CACAAACGTCGCCATG	CC2
SMC10.T.CACAAACTCCGTTGTC	CC2
SMC10.T.CACACTCCAAGCGATG	CC2
SMC10.T.CACAGGCGTCGCTTTC	CC2
SMC10.T.CACAGGCGTTAGGGTG	CC1
SMC10.T.CACATTTAGCGTGAGT	CC2
SMC10.T.CACCACTAGGCCCTCA	CC1
SMC10.T.CACCAGGAGAGTGAGA	CC2

SMC10.T.CACCAGGAGTATCGAA	CC2
SMC10.T.CACCTTGTCGGTTCGG	CC1
SMC10.T.CACTCCAAGCCCAGCT	CC1
SMC10.T.CACTCCAAGGATGGAA	CC2
SMC10.T.CACTCCATCGCGATCG	CC2
SMC10.T.CAGATCAAGCCGCCTA	CC2
SMC10.T.CAGATCACACAGACAG	CC1
SMC10.T.CAGATCAGTCCGCTGA	CC1
SMC10.T.CAGCGACGTAAGGGAA	CC1
SMC10.T.CAGCTAAGTGCTGTAT	CC2
SMC10.T.CAGGTGCGTAGCAAAT	CC2
SMC10.T.CAGGTGCTCAAGGTAA	CC1
SMC10.T.CAGGTGCTCTAGCACA	CC2
SMC10.T.CAGGTGCTCTGTCAAG	CC2
SMC10.T.CAGTAACAGACTACAA	CC2
SMC10.T.CAGTAACAGTGTGAAT	CC2
SMC10.T.CAGTAACGTCAATGTC	CC2
SMC10.T.CAGTAACGTTATGTGC	CC2
SMC10.T.CAGTCCTGTTACTGAC	CC1
SMC10.T.CATATTCTCACGCGGT	CC2
SMC10.T.CATCAAGCAACTGGCC	CC2
SMC10.T.CATCAAGTCCAGAGGA	CC2
SMC10.T.CATCAGAAGTGGTAAT	CC2
SMC10.T.CATCAGACATCCTTGC	CC2
SMC10.T.CATCCACGTGGAAAGA	CC2
SMC10.T.CATCCACTCCCGACTT	CC2
SMC10.T.CATCGGGTCAACCAAC	CC2
SMC10.T.CATGACAAGAAGGGTA	CC2
SMC10.T.CATGACAAGGGAAACA	CC2
SMC10.T.CATGACAGTCATACTG	CC1
SMC10.T.CATGACATCCATGCTC	CC2
SMC10.T.CATGACATCCGTCATC	CC2
SMC10.T.CATGGCGTCGGACAAG	CC2
SMC10.T.CATTCGCTCAGGCGAA	CC2
SMC10.T.CATTCGCTCCACGACG	CC2
SMC10.T.CCAATCCTCACGATGT	CC2
SMC10.T.CCACCTAAGTAGCCGA	CC2
SMC10.T.CCACCTATCATTGCCC	CC2
SMC10.T.CCACGGAAGACAATAC	CC1
SMC10.T.CCACGGAAGCGATAGC	CC2
SMC10.T.CCACTACTCATTGCGA	CC2
SMC10.T.CCACTACTCGTTTATC	CC2
SMC10.T.CCATGTCCAAATTGCC	CC1
SMC10.T.CCATTCGTCCATGAGT	CC2
SMC10.T.CCCAATCAGATAGGAG	CC2
SMC10.T.CCCAGTTAGTAGCCGA	CC2
SMC10.T.CCCAGTTTCTCTAAGG	CC2
SMC10.T.CCCATACTCATAAAGG	CC2
SMC10.T.CCCATACTCCTCAATT	CC1
SMC10.T.CCCATACTCTGCAGTA	CC2
SMC10.T.CCGGTAGAGATGCCAG	CC2
SMC10.T.CCGTACTGTTCAACCA	CC2
SMC10.T.CCGTGGACAACGATCT	CC2
SMC10.T.CCGTGGATCCATGCTC	CC2
SMC10.T.CCGTTCACATCTGGTA	CC2
SMC10.T.CCGTTCAGTGGTACAG	CC2
SMC10.T.CCTACACCAAAGCGGT	CC2
SMC10.T.CCTACACTCAGCTCTC	CC2

SMC10.T.CCTAGCTAGGCATTGG	CC1
SMC10.T.CCTAGCTGTGAAGGCT	CC2
SMC10.T.CCTATTACAGCCTTGG	CC2
SMC10.T.CCTTACGTCACTCCTG	CC2
SMC10.T.CGAACATCAGCTGTTA	CC2
SMC10.T.CGAACATGTCAATGTC	CC1
SMC10.T.CGAACATGTGGCCCTA	CC2
SMC10.T.CGAATGTTCAACCATG	CC2
SMC10.T.CGACCTTCATAGGATA	CC1
SMC10.T.CGACCTTTCACAGGCC	CC2
SMC10.T.CGATCGGAGGTGGGTT	CC2
SMC10.T.CGATCGGGTAAGAGAG	CC2
SMC10.T.CGATGGCCATACTACG	CC2
SMC10.T.CGATTGACAGAGTGTG	CC2
SMC10.T.CGATTGAGTAGGACAC	CC2
SMC10.T.CGCGGTAGTCTCAACA	CC2
SMC10.T.CGCGGTATCGCCAGCA	CC2
SMC10.T.CGCGTTTCATGACATC	CC2
SMC10.T.CGCTATCAGAATTGTG	CC2
SMC10.T.CGCTGGACACCAGCAC	CC2
SMC10.T.CGCTTCAAGGCTATCT	CC2
SMC10.T.CGCTTCAGTCTGCCAG	CC2
SMC10.T.CGCTTCAGTGTTGGGA	CC2
SMC10.T.CGGACACAGGGTCTCC	CC2
SMC10.T.CGGACACCAGCATACT	CC2
SMC10.T.CGGACACCATGCAATC	CC2
SMC10.T.CGGACACGTCACTGGC	CC2
SMC10.T.CGGACACGTTAGAACA	CC2
SMC10.T.CGGACGTTCGGCGCAT	CC2
SMC10.T.CGGAGCTCATTGTGCA	CC2
SMC10.T.CGGAGTCAGCGAGAAA	CC2
SMC10.T.CGGCTAGGTCTCAACA	CC2
SMC10.T.CGTAGCGCAGCTCGAC	CC2
SMC10.T.CGTAGGCAGCACCGCT	CC1
SMC10.T.CGTCACTAGGATGGTC	CC2
SMC10.T.CGTCAGGTCGCAAGCC	CC2
SMC10.T.CGTCCATAGCCACGTC	CC2
SMC10.T.CGTCCATCAATGAAAC	CC1
SMC10.T.CGTGTAAGTGGGTCAA	CC2
SMC10.T.CGTGTCTGTTAAAGTG	CC2
SMC10.T.CGTGTCTTCGTACGGC	CC2
SMC10.T.CGTTAGAGTGAGTATA	CC2
SMC10.T.CGTTAGAGTGGACGAT	CC2
SMC10.T.CGTTCTGAGCCTTGAT	CC2
SMC10.T.CGTTGGGGTGTAATGA	CC2
SMC10.T.CTAACTTGTCTAGAGG	CC1
SMC10.T.CTAAGACCAGCATGAG	CC2
SMC10.T.CTAAGACTCACCTCGT	CC2
SMC10.T.CTAATGGCACCGAAAG	CC2
SMC10.T.CTAATGGTCAAACCGT	CC2
SMC10.T.CTACATTCAGCCTATA	CC1
SMC10.T.CTAGCCTAGACAAAGG	CC2
SMC10.T.CTAGCCTGTGTGTGCC	CC2
SMC10.T.CTAGTGAGTAGGGTAC	CC1
SMC10.T.CTCATTAGTCTAGTGT	CC2
SMC10.T.CTCCTAGGTGGCCCTA	CC2
SMC10.T.CTCCTAGTCTACTCAT	CC2
SMC10.T.CTCGAAAAGCGTGAAC	CC2

SMC10.T.CTCGGAGCAGTACACT	CC1
SMC10.T.CTCGGAGCATAGACTC	CC2
SMC10.T.CTCGTACCACATAACC	CC1
SMC10.T.CTCGTACTCGGATGTT	CC2
SMC10.T.CTCGTCACACCGTTGG	CC2
SMC10.T.CTCTGGTTCGTTACGA	CC1
SMC10.T.CTCTGGTTCTAAGCCA	CC2
SMC10.T.CTGAAACTCATACGGT	CC2
SMC10.T.CTGATAGCAATGCCAT	CC2
SMC10.T.CTGATAGGTACGCTGC	CC2
SMC10.T.CTGCCTACACATCTTT	CC2
SMC10.T.CTGCCTATCTAACGGT	CC2
SMC10.T.CTGGTCTAGACAATAC	CC2
SMC10.T.CTGTGCTAGATCACGG	CC2
SMC10.T.CTGTGCTAGCCAACAG	CC2
SMC10.T.CTGTGCTCATGATCCA	CC2
SMC10.T.CTGTGCTGTGAACCTT	CC2
SMC10.T.CTGTGCTTCGAGGTAG	CC2
SMC10.T.CTTAACTTCAGGCGAA	CC2
SMC10.T.CTTAACTTCGTAGGAG	CC2
SMC10.T.CTTACCGCAGTAGAGC	CC2
SMC10.T.CTTCTCTGTCGACTAT	CC2
SMC10.T.CTTCTCTTCCCATTAT	CC2
SMC10.T.CTTGGCTGTGGTCTCG	CC1
SMC10.T.GAAACTCAGCTAGCCC	CC2
SMC10.T.GAAACTCTCCCACTTG	CC2
SMC10.T.GAAATGAGTATGCTTG	CC2
SMC10.T.GAACATCTCCAATGGT	CC2
SMC10.T.GAACGGAAGGAGTTGC	CC2
SMC10.T.GAACGGATCACTATTC	CC2
SMC10.T.GACACGCAGGCAGTCA	CC2
SMC10.T.GACACGCCAGCGTAAG	CC2
SMC10.T.GACAGAGTCCAAACAC	CC2
SMC10.T.GACCAATAGATCTGCT	CC2
SMC10.T.GACCAATTCGTCACGG	CC1
SMC10.T.GACGGCTTCAGAGACG	CC2
SMC10.T.GACGTTACACAACTGT	CC2
SMC10.T.GACGTTATCTGAGTGT	CC2
SMC10.T.GACTAACGTCTAGAGG	CC2
SMC10.T.GACTAACTCCACGAAT	CC1
SMC10.T.GACTACATCGGTTAAC	CC2
SMC10.T.GAGCAGAAGCTGCGAA	CC2
SMC10.T.GATCAGTTCCAGTATG	CC2
SMC10.T.GATCGATCAGCTGGCT	CC2
SMC10.T.GATCGATGTACTTCTT	CC2
SMC10.T.GATCGCGAGCCACGTC	CC2
SMC10.T.GATCGCGTCACTTACT	CC2
SMC10.T.GATCGCGTCCCTGACT	CC2
SMC10.T.GATGAAACAAGACGTG	CC2
SMC10.T.GATGAGGGTGGGTATG	CC2
SMC10.T.GATGCTAGTACGCACC	CC2
SMC10.T.GCACATAAGCTGAACG	CC2
SMC10.T.GCACATAAGGAATGGA	CC2
SMC10.T.GCAGCCAAGCTGTTCA	CC2
SMC10.T.GCAGCCACAATGGACG	CC2
SMC10.T.GCAGCCACATGACGGA	CC2
SMC10.T.GCAGTTAGTCTGCCAG	CC2
SMC10.T.GCAGTTATCAGGATCT	CC2

SMC10.T.GCATACAAGTGGTAGC	CC2
SMC10.T.GCATGATGTACCCAAT	CC2
SMC10.T.GCATGTACAAGGTGTG	CC2
SMC10.T.GCCAAATCATCTCCCA	CC2
SMC10.T.GCCAAATGTGCGAAAC	CC2
SMC10.T.GCCAAATTCGGAAACG	CC2
SMC10.T.GCCTCTATCGAGAGCA	CC2
SMC10.T.GCGCAACGTGCGAAAC	CC1
SMC10.T.GCGCAACTCCAGGGCT	CC2
SMC10.T.GCGCAGTCAAGGACTG	CC2
SMC10.T.GCGCAGTGTTTGACTG	CC2
SMC10.T.GCGCAGTTCGGAAACG	CC2
SMC10.T.GCGCCAACAACTGGCC	CC2
SMC10.T.GCGCCAACAGCCTGTG	CC1
SMC10.T.GCGCCAACATCTCCCA	CC2
SMC10.T.GCGCGATAGCATGGCA	CC1
SMC10.T.GCGGGTTAGCAAATCA	CC2
SMC10.T.GCTCCTATCCCTCTTT	CC2
SMC10.T.GCTCTGTTCGCGATCG	CC2
SMC10.T.GCTGCAGTCTGGTTCC	CC2
SMC10.T.GCTGCTTAGGCATTGG	CC2
SMC10.T.GCTGGGTAGAGTCGGT	CC2
SMC10.T.GCTGGGTCATACGCCG	CC2
SMC10.T.GCTTCCAGTACTCAAC	CC2
SMC10.T.GGAAAGCAGGTAGCCA	CC2
SMC10.T.GGAAAGCAGTCTCCTC	CC2
SMC10.T.GGAAAGCGTTGTGGCC	CC2
SMC10.T.GGAATAACACGGTAAG	CC2
SMC10.T.GGACGTCCACTGAAGG	CC2
SMC10.T.GGAGCAATCATTGCCC	CC1
SMC10.T.GGATGTTTCATAAAGG	CC1
SMC10.T.GGATGTTTCGGCCGAT	CC1
SMC10.T.GGATTACGTCCGCTGA	CC1
SMC10.T.GGCAATTAGTCCAGGA	CC2
SMC10.T.GGCAATTCATGCCTTC	CC2
SMC10.T.GGCCGATAGCCAGGAT	CC2
SMC10.T.GGCGACTAGAACAATC	CC2
SMC10.T.GGCGTGTAGGACGAAA	CC2
SMC10.T.GGCTCGAAGTACATGA	CC1
SMC10.T.GGCTGGTGTGCAGTAG	CC2
SMC10.T.GGGAATGAGTAGCGGT	CC2
SMC10.T.GGGAATGAGTGCGATG	CC2
SMC10.T.GGGAATGGTAGCAAAT	CC2
SMC10.T.GGGAGATTCGATGAGG	CC2
SMC10.T.GGGATGATCCCTCTTT	CC2
SMC10.T.GGGCACTCATGAAGTA	CC2
SMC10.T.GGGCACTTCGCATGAT	CC2
SMC10.T.GGGCATCAGTATTGGA	CC2
SMC10.T.GGTATTGCACTTGGAT	CC2
SMC10.T.GGTGTTAAGAATGTTG	CC2
SMC10.T.GGTGTTAAGTCCAGGA	CC2
SMC10.T.GGTGTTAGTCACCCAG	CC1
SMC10.T.GTAACTGGTCTTCGTC	CC2
SMC10.T.GTACTCCAGTAGATGT	CC2
SMC10.T.GTACTCCTCTACTCAT	CC2
SMC10.T.GTACTTTTCACTCTTA	CC2
SMC10.T.GTAGGCCAGTACGTTC	CC2
SMC10.T.GTATCTTCACCAGGCT	CC2

SMC10.T.GTATCTTGTCGCATAT	CC1
SMC10.T.GTATCTTTCAGTGTTG	CC1
SMC10.T.GTATTCTGTATTCTCT	CC2
SMC10.T.GTCACAAAGTGCGATG	CC2
SMC10.T.GTCACAACACGTAAGG	CC2
SMC10.T.GTCACAAGTTATGTGC	CC2
SMC10.T.GTCACAAGTTCTGTTT	CC2
SMC10.T.GTCACGGAGCTAACAA	CC2
SMC10.T.GTCATTTGTTTAAGCC	CC1
SMC10.T.GTCGGGTAGCTCCTTC	CC1
SMC10.T.GTCGGGTTCAAGGCTT	CC2
SMC10.T.GTCGGGTTCCTACAGA	CC2
SMC10.T.GTCGTAAAGCTTCGCG	CC2
SMC10.T.GTCTCGTTCAATAAGG	CC2
SMC10.T.GTCTCGTTCCGTAGGC	CC2
SMC10.T.GTCTTCGGTCTCTCTG	CC1
SMC10.T.GTGAAGGTCTTAGCCC	CC2
SMC10.T.GTGCAGCCAAGTCATC	CC2
SMC10.T.GTGCATATCAGTTGAC	CC2
SMC10.T.GTGCGGTTCCCTGACT	CC2
SMC10.T.GTGGGTCCATACTACG	CC2
SMC10.T.GTGGGTCGTAGTACCT	CC2
SMC10.T.GTGTGCGAGTGTTGAA	CC2
SMC10.T.GTGTGCGGTAGCGCTC	CC2
SMC10.T.GTGTGCGGTAGGCATG	CC2
SMC10.T.GTGTGCGGTCCCGACA	CC2
SMC10.T.GTGTGCGGTGATGTCT	CC2
SMC10.T.GTTAAGCCACATGGGA	CC2
SMC10.T.GTTAAGCTCTGTGCAA	CC2
SMC10.T.GTTACAGGTCAGAAGC	CC2
SMC10.T.GTTACAGGTCCGAATT	CC2
SMC10.T.GTTACAGGTTTGGCGC	CC2
SMC10.T.GTTCATTCAGTAGAGC	CC2
SMC10.T.GTTCGGGGTTAAGGGC	CC2
SMC10.T.GTTCTCGAGACTGTAA	CC2
SMC10.T.GTTTCTACATACTACG	CC1
SMC10.T.GTTTCTATCATGTGGT	CC2
SMC10.T.TAAACCGCAGCGTTCG	CC2
SMC10.T.TAAACCGTCATTTGGG	CC1
SMC10.T.TAAGAGATCCTGCCAT	CC2
SMC10.T.TACGGATAGTACGTTC	CC2
SMC10.T.TACGGGCTCACAAACC	CC2
SMC10.T.TACTCATCAAGAAGAG	CC1
SMC10.T.TACTCATCAGGCTGAA	CC2
SMC10.T.TACTCATGTAGCGATG	CC1
SMC10.T.TACTTGTAGATCACGG	CC2
SMC10.T.TAGACCAGTAGCGATG	CC2
SMC10.T.TAGCCGGAGCATGGCA	CC2
SMC10.T.TAGTTGGCACACGCTG	CC2
SMC10.T.TAGTTGGGTGATGTCT	CC2
SMC10.T.TATCAGGCAATCTGCA	CC2
SMC10.T.TATCTCAAGTCGATAA	CC1
SMC10.T.TATGCCCTCATCTGCC	CC2
SMC10.T.TATTACCAGAGGACGG	CC2
SMC10.T.TATTACCGTAGAGCTG	CC2
SMC10.T.TATTACCGTCTCCACT	CC2
SMC10.T.TATTACCGTGATAAAC	CC2
SMC10.T.TCAATCTAGCATGGCA	CC1

SMC10.T.TCAATCTCACCTGGTG	CC2
SMC10.T.TCACAAGGTTGCGCAC	CC2
SMC10.T.TCAGATGCACTGTCGG	CC2
SMC10.T.TCAGATGGTGCGATAG	CC2
SMC10.T.TCAGCAAAGATATGGT	CC2
SMC10.T.TCAGCAAGTAACGACG	CC2
SMC10.T.TCAGCTCTCACGAAGG	CC1
SMC10.T.TCAGGTATCGCTGATA	CC2
SMC10.T.TCATTACTCCTGCCAT	CC2
SMC10.T.TCATTTGGTAAACGCG	CC2
SMC10.T.TCCCGATAGACTGGGT	CC2
SMC10.T.TCCCGATTCTGCTTGC	CC2
SMC10.T.TCGCGTTTCGGTTCGG	CC2
SMC10.T.TCGGGACAGCATGGCA	CC1
SMC10.T.TCGGGACTCGTGTAGT	CC2
SMC10.T.TCGGTAACACCAGCAC	CC2
SMC10.T.TCGTACCGTGCAACTT	CC2
SMC10.T.TCTATTGAGACAGGCT	CC2
SMC10.T.TCTATTGAGAGGGATA	CC1
SMC10.T.TCTCTAACATCCTAGA	CC2
SMC10.T.TCTGAGAAGGCTAGGT	CC1
SMC10.T.TCTGAGAGTGTATGGG	CC2
SMC10.T.TCTGGAACAGTTAACC	CC1
SMC10.T.TCTGGAACATTACGAC	CC2
SMC10.T.TCTTTCCGTACTTCTT	CC2
SMC10.T.TCTTTCCTCCTTAATC	CC2
SMC10.T.TGAAAGATCTTACCTA	CC2
SMC10.T.TGACGGCCAGTTTACG	CC2
SMC10.T.TGACGGCGTCCAAGTT	CC2
SMC10.T.TGACGGCTCTAACTGG	CC1
SMC10.T.TGACTAGGTCGACTGC	CC2
SMC10.T.TGACTTTAGGGAGTAA	CC2
SMC10.T.TGACTTTTCTTGTACT	CC2
SMC10.T.TGAGAGGCATGGATGG	CC2
SMC10.T.TGAGAGGTCAACCATG	CC2
SMC10.T.TGAGCCGCACGGCGTT	CC2
SMC10.T.TGAGCCGTCCTCAATT	CC2
SMC10.T.TGAGCCGTCGAGGTAG	CC2
SMC10.T.TGATTTCCACAAGCCC	CC2
SMC10.T.TGATTTCCATCGGTTA	CC2
SMC10.T.TGCACCTCAGTGAGTG	CC1
SMC10.T.TGCCAAATCTCAAGTG	CC2
SMC10.T.TGCCCATCAAGTAATG	CC2
SMC10.T.TGCGCAGAGTCATGCT	CC2
SMC10.T.TGCGGGTAGCCACCTG	CC2
SMC10.T.TGCGTGGAGCGAAGGG	CC2
SMC10.T.TGCTACCTCAGCTTAG	CC2
SMC10.T.TGCTGCTTCCATGAGT	CC2
SMC10.T.TGGACGCGTTCCTCCA	CC2
SMC10.T.TGGCCAGAGAAGGGTA	CC2
SMC10.T.TGGCCAGTCATGTAGC	CC2
SMC10.T.TGGCGCACAACTTGAC	CC2
SMC10.T.TGGCGCACAGATCCAT	CC2
SMC10.T.TGGCGCATCCAATGGT	CC2
SMC10.T.TGGCTGGTCTATCGCC	CC2
SMC10.T.TGGGCGTCATCACAAC	CC1
SMC10.T.TGGGCGTGTGAGGGTT	CC2
SMC10.T.TGGTTCCGTAGCGCAA	CC2

SMC10.T.TGTCCCATCGCTAGCG	CC2
SMC10.T.TGTGGTATCGTCGTTC	CC2
SMC10.T.TGTGTTTGTAGAGCTG	CC2
SMC10.T.TTAACTCCATGGGACA	CC2
SMC10.T.TTAGGCACACACTGCG	CC2
SMC10.T.TTAGGCAGTGATGTGG	CC2
SMC10.T.TTAGTTCCAGGGTTAG	CC2
SMC10.T.TTATGCTTCACGAAGG	CC2
SMC10.T.TTCCCAGAGGGATCTG	CC2
SMC10.T.TTCGAAGCAGCCAGAA	CC2
SMC10.T.TTCGGTCAGACCTTTG	CC2
SMC10.T.TTCGGTCCACATCTTT	CC2
SMC10.T.TTCGGTCCAGCAGTTT	CC2
SMC10.T.TTCGGTCCAGCCTATA	CC1
SMC10.T.TTCTACAAGCCAGGAT	CC2
SMC10.T.TTCTACAGTAAGTAGT	CC2
SMC10.T.TTCTCAAAGCGCTCCA	CC2
SMC10.T.TTCTCAATCTATCCTA	CC2
SMC10.T.TTCTCCTCACGACTCG	CC1
SMC10.T.TTCTCCTGTGGTCTCG	CC2
SMC10.T.TTCTCCTTCGGCTTGG	CC2
SMC10.T.TTCTTAGTCGAATGGG	CC2
SMC10.T.TTGAACGCAGCCAGAA	CC2
SMC10.T.TTGACTTCAGCCAGAA	CC1
SMC10.T.TTGACTTGTTAAGATG	CC2
SMC10.T.TTGCGTCTCTCCGGTT	CC2
SMC10.T.TTGTAGGAGATGCCTT	CC2
SMC10.T.TTGTAGGCAGTGACAG	CC2
SMC10.T.TTGTAGGGTCTAAACC	CC2
SMC10.T.TTTACTGTCCTTTACA	CC2
SMC10.T.TTTCCTCAGCCCAACC	CC2
SMC10.T.TTTCCTCCAATGTTGC	CC2
SMC10.T.TTTGCGCAGCTAGGCA	CC1
SMC10.T.TTTGCGCAGTGTACCT	CC2
SMC10.T.TTTGCGCGTATAGGGC	CC2
SMC10.T.TTTGGTTAGGTACTCT	CC2
SMC10.T.TTTGGTTAGTGTGAAT	CC2
SMC10.T.TTTGGTTTCCCAGGTG	CC2
SMC11.T.AAACCTGGTACCAGTT	CC1
SMC11.T.AAACGGGAGATCCGAG	CC1
SMC11.T.AAACGGGGTTATTCTC	CC1
SMC11.T.AAAGATGCAATCAGAA	CC1
SMC11.T.AAAGATGTCTACCAGA	CC1
SMC11.T.AAAGCAATCTGTCAAG	CC1
SMC11.T.AAAGTAGCACAACGCC	CC1
SMC11.T.AACCATGGTCACCCAG	CC1
SMC11.T.AACGTTGAGCCAGTTT	CC1
SMC11.T.AACGTTGCACCGAAAG	CC1
SMC11.T.AACGTTGGTGCAGACA	CC1
SMC11.T.AACTCAGTCTTAACCT	CC1
SMC11.T.AACTGGTTCTGCGGCA	CC1
SMC11.T.AAGCCGCAGCTGTTCA	CC1
SMC11.T.AAGGAGCTCGTAGATC	CC1
SMC11.T.AAGGTTCAGCCGCCTA	CC1
SMC11.T.AAGTCTGAGACCCACC	CC1
SMC11.T.AATCCAGCATAACCTG	CC1
SMC11.T.AATCGGTGTAAGGGCT	CC1
SMC11.T.AATCGGTGTTCTGGTA	CC1

SMC11.T.ACACCCTCATCAGTCA	CC1
SMC11.T.ACACCGGAGACCACGA	CC1
SMC11.T.ACACCGGCAACAACCT	CC2
SMC11.T.ACACTGAAGTGTACCT	CC1
SMC11.T.ACACTGAGTTGGACCC	CC1
SMC11.T.ACAGCCGAGAACTGTA	CC1
SMC11.T.ACAGCCGAGATGCCAG	CC1
SMC11.T.ACATCAGAGGAGCGTT	CC1
SMC11.T.ACATGGTAGACTGTAA	CC1
SMC11.T.ACCAGTAGTCTCCCTA	CC1
SMC11.T.ACCAGTAGTTCTGAAC	CC1
SMC11.T.ACCCACTCACCAGGCT	CC1
SMC11.T.ACGCCAGAGAGGACGG	CC1
SMC11.T.ACGCCAGTCTGTTTGT	CC1
SMC11.T.ACGGAGATCCAATGGT	CC1
SMC11.T.ACGGGTCCACTAAGTC	CC1
SMC11.T.ACGTCAACACTTACGA	CC1
SMC11.T.ACTATCTTCTGCCAGG	CC1
SMC11.T.ACTGAACAGCCACTAT	CC1
SMC11.T.ACTGAGTAGTTAGCGG	CC1
SMC11.T.ACTGATGAGTATCTCG	CC1
SMC11.T.ACTGCTCCAGTAAGAT	CC1
SMC11.T.ACTGCTCGTGTTAAGA	CC1
SMC11.T.ACTGTCCCATTCCTCG	CC1
SMC11.T.ACTTACTCATCTACGA	CC2
SMC11.T.ACTTACTTCGCGATCG	CC1
SMC11.T.ACTTGTTCAAGGTGTG	CC1
SMC11.T.ACTTGTTCACAGGTTT	CC1
SMC11.T.ACTTGTTGTAGCGCTC	CC1
SMC11.T.ACTTGTTTCCGGGTGT	CC1
SMC11.T.AGAATAGAGCTAAACA	CC1
SMC11.T.AGAGTGGAGTTCGCGC	CC1
SMC11.T.AGAGTGGTCGGTGTTA	CC1
SMC11.T.AGCATACAGCGTAATA	CC1
SMC11.T.AGCATACCAGGGTACA	CC1
SMC11.T.AGCCTAACACGTCAGC	CC1
SMC11.T.AGCCTAATCCAAATGC	CC1
SMC11.T.AGCTCCTGTTGTGGCC	CC1
SMC11.T.AGGCCGTCATGGTAGG	CC1
SMC11.T.AGGCCGTCATTCGACA	CC1
SMC11.T.AGGCCGTTCACCACCT	CC1
SMC11.T.AGTAGTCAGCAGGCTA	CC1
SMC11.T.AGTGGGATCAGTTTGG	CC1
SMC11.T.AGTGTCATCTCGAGTA	CC1
SMC11.T.AGTTGGTAGATTACCC	CC1
SMC11.T.AGTTGGTAGGGCTTGA	CC1
SMC11.T.ATAGACCAGCAATCTC	CC1
SMC11.T.ATAGACCCATCGGAAG	CC1
SMC11.T.ATCACGACATTGGTAC	CC1
SMC11.T.ATCACGATCTATCCCG	CC1
SMC11.T.ATCCACCAGCTGCGAA	CC1
SMC11.T.ATCCACCTCATCGGAT	CC1
SMC11.T.ATCTGCCAGACCGGAT	CC1
SMC11.T.ATCTGCCAGTCTCCTC	CC1
SMC11.T.ATCTGCCCAGTGGAGT	CC1
SMC11.T.ATGAGGGCAAGTAGTA	CC1
SMC11.T.ATGAGGGCATATGCTG	CC1
SMC11.T.ATGGGAGTCTCGTTTA	CC1

SMC11.T.ATTATCCCACGTGAGA	CC1
SMC11.T.CAACCTCTCAATCACG	CC1
SMC11.T.CAACTAGGTATAAACG	CC1
SMC11.T.CAAGATCGTGTTCTTT	CC1
SMC11.T.CAAGGCCCATCGATGT	CC1
SMC11.T.CAAGTTGAGACGCTTT	CC1
SMC11.T.CACAGGCGTCCATCCT	CC1
SMC11.T.CACATAGAGACACTAA	CC1
SMC11.T.CACATAGTCGCAGGCT	CC1
SMC11.T.CACCACTGTCAACTGT	CC1
SMC11.T.CACCAGGCATGCTGGC	CC1
SMC11.T.CACCTTGGTGCTAGCC	CC1
SMC11.T.CAGAGAGAGGATGGAA	CC1
SMC11.T.CAGATCAAGCTAGGCA	CC1
SMC11.T.CAGCCGAGTCTGCGGT	CC1
SMC11.T.CAGCGACTCCTATTCA	CC1
SMC11.T.CAGCTAAGTAGTACCT	CC1
SMC11.T.CAGGTGCGTACTCGCG	CC1
SMC11.T.CAGTCCTGTCTTTCAT	CC1
SMC11.T.CATATTCAGATCCGAG	CC1
SMC11.T.CATCAAGCAATCGAAA	CC1
SMC11.T.CATCAAGCACCTCGTT	CC1
SMC11.T.CATCAGATCCGTCATC	CC1
SMC11.T.CATCCACAGACCGGAT	CC1
SMC11.T.CATCCACAGCTCCTCT	CC1
SMC11.T.CATCGGGCAAGCTGTT	CC1
SMC11.T.CATTATCCACGGTTTA	CC1
SMC11.T.CATTATCGTCGACTAT	CC1
SMC11.T.CATTATCTCAAACCGT	CC2
SMC11.T.CATTCGCGTCTCCACT	CC1
SMC11.T.CCAATCCTCATCGGAT	CC1
SMC11.T.CCACCTATCTCAAACG	CC1
SMC11.T.CCAGCGACATTTGCCC	CC1
SMC11.T.CCATGTCGTGCTAGCC	CC1
SMC11.T.CCATGTCGTTTACTCT	CC1
SMC11.T.CCATTCGTCCATGAAC	CC1
SMC11.T.CCCTCCTCACACATGT	CC1
SMC11.T.CCGGGATGTGACGGTA	CC1
SMC11.T.CCGGTAGTCGATGAGG	CC1
SMC11.T.CCGGTAGTCTCATTCA	CC1
SMC11.T.CCGTACTTCGCGTTTC	CC1
SMC11.T.CCGTGGAAGCCGTCGT	CC1
SMC11.T.CCGTTCAAGACTTTCG	CC1
SMC11.T.CCTCAGTAGATAGGAG	CC1
SMC11.T.CCTCAGTAGCGTTGCC	CC1
SMC11.T.CCTCAGTCATCGATGT	CC1
SMC11.T.CCTTCGAAGTGGTAGC	CC1
SMC11.T.CGAATGTGTTAAGGGC	CC1
SMC11.T.CGAATGTTCAGAGGTG	CC1
SMC11.T.CGACCTTGTCTAGGTT	CC1
SMC11.T.CGAGCACGTGCAACTT	CC1
SMC11.T.CGATCGGAGGGTATCG	CC1
SMC11.T.CGATCGGCATGTCCTC	CC1
SMC11.T.CGCGGTAGTAGCTTGT	CC1
SMC11.T.CGCTATCTCACCGTAA	CC1
SMC11.T.CGGACGTAGCGAAGGG	CC1
SMC11.T.CGGAGCTTCGGTGTTA	CC1
SMC11.T.CGGAGTCGTTATCGGT	CC1

SMC11.T.CGGCTAGCATCACGAT	CC1
SMC11.T.CGGGTCAGTCCGTTAA	CC1
SMC11.T.CGGGTCAGTCCTAGCG	CC1
SMC11.T.CGGGTCATCTGGTATG	CC1
SMC11.T.CGTAGCGGTTACTGAC	CC1
SMC11.T.CGTAGGCAGGATGTAT	CC1
SMC11.T.CGTAGGCGTTGCTCCT	CC1
SMC11.T.CGTAGGCTCTGATTCT	CC1
SMC11.T.CGTCAGGAGTGGTCCC	CC1
SMC11.T.CGTCCATCAAGCGAGT	CC1
SMC11.T.CGTCCATGTACACCGC	CC1
SMC11.T.CGTTGGGAGCAATCTC	CC1
SMC11.T.CTAAGACGTTTGTTTC	CC1
SMC11.T.CTACACCCAAGGACAC	CC1
SMC11.T.CTAGAGTTCCATTCTA	CC1
SMC11.T.CTAGCCTAGCCCTAAT	CC1
SMC11.T.CTCGAAAAGTGATCGG	CC1
SMC11.T.CTCTAATCAAACCCAT	CC1
SMC11.T.CTCTAATTCGGATGTT	CC1
SMC11.T.CTCTACGAGCTCCCAG	CC1
SMC11.T.CTGATCCTCTCTAGGA	CC1
SMC11.T.CTGCGGAAGCTCAACT	CC1
SMC11.T.CTGGTCTGTCGTGGCT	CC1
SMC11.T.CTGGTCTTCTTTAGTC	CC1
SMC11.T.CTGTGCTAGACAAAGG	CC1
SMC11.T.CTGTTTATCACATACG	CC1
SMC11.T.CTTCTCTGTAACGACG	CC1
SMC11.T.CTTGGCTTCGTATCAG	CC1
SMC11.T.CTTTGCGGTCTTGCGG	CC1
SMC11.T.GAAACTCGTCCAGTGC	CC1
SMC11.T.GAACCTAGTCGATTGT	CC1
SMC11.T.GAACCTATCCCGACTT	CC1
SMC11.T.GAACGGAGTGTCAATC	CC1
SMC11.T.GAATAAGAGGGTTTCT	CC1
SMC11.T.GAATGAATCAACGGGA	CC1
SMC11.T.GACAGAGAGCGTCTAT	CC1
SMC11.T.GACAGAGCAGCAGTTT	CC1
SMC11.T.GACAGAGTCCAGTATG	CC1
SMC11.T.GACCAATGTCCGTTAA	CC1
SMC11.T.GACCTGGAGTTCCACA	CC1
SMC11.T.GACTGCGCACCTGGTG	CC1
SMC11.T.GAGGTGAGTATATGAG	CC1
SMC11.T.GAGTCCGGTGCACCAC	CC1
SMC11.T.GAGTCCGTCGCAAACT	CC1
SMC11.T.GAGTCCGTCTTGTCAT	CC1
SMC11.T.GATCAGTCAGGTCTCG	CC1
SMC11.T.GATCGATGTTAAGATG	CC1
SMC11.T.GATCTAGCAGCGTCCA	CC1
SMC11.T.GATGAAACAATAGCAA	CC1
SMC11.T.GATGAAATCACAGGCC	CC1
SMC11.T.GATGCTACACTCGACG	CC1
SMC11.T.GATTCAGTCTGAAAGA	CC1
SMC11.T.GCAAACTCAGTCAGAG	CC1
SMC11.T.GCAATCAAGGACTGGT	CC1
SMC11.T.GCAATCACACCATCCT	CC1
SMC11.T.GCACATAGTAGTACCT	CC1
SMC11.T.GCACATATCCGTCATC	CC1
SMC11.T.GCACTCTCATGGTAGG	CC1

SMC11.T.GCAGTTACATTTGCCC	CC1
SMC11.T.GCAGTTATCCGAAGAG	CC1
SMC11.T.GCATGCGAGCGTAGTG	CC1
SMC11.T.GCATGCGGTCGAACAG	CC1
SMC11.T.GCCAAATCAGTATAAG	CC1
SMC11.T.GCGCCAAGTACTTGAC	CC1
SMC11.T.GCTCTGTCAATGGTCT	CC1
SMC11.T.GCTGCAGCATGCAACT	CC1
SMC11.T.GCTGCAGGTATCACCA	CC1
SMC11.T.GCTGCTTGTCAACTGT	CC1
SMC11.T.GCTGGGTCATATACCG	CC1
SMC11.T.GCTGGGTGTCCCTTGT	CC1
SMC11.T.GCTTCCAAGATCGATA	CC1
SMC11.T.GCTTCCATCTTGAGAC	CC1
SMC11.T.GGAACTTAGTGACATA	CC1
SMC11.T.GGACAGAGTGGCGAAT	CC1
SMC11.T.GGACAGATCCTTGGTC	CC1
SMC11.T.GGACATTAGTAGGCCA	CC1
SMC11.T.GGACATTGTCTTGTCC	CC1
SMC11.T.GGAGCAAGTTAAGACA	CC1
SMC11.T.GGATGTTTCCGAGCCA	CC1
SMC11.T.GGATGTTTCTGGAGCC	CC1
SMC11.T.GGCAATTAGAGGGCTT	CC2
SMC11.T.GGCGACTAGCGCCTTG	CC1
SMC11.T.GGCGACTTCAGATAAG	CC1
SMC11.T.GGCGTGTAGCCACCTG	CC1
SMC11.T.GGCTGGTGTGAAATCA	CC1
SMC11.T.GGGACCTTCACAATGC	CC1
SMC11.T.GGGATGAGTGTGGTTT	CC2
SMC11.T.GGGCACTGTGGCTCCA	CC1
SMC11.T.GGGTCTGGTCTAGTCA	CC1
SMC11.T.GGTGAAGCACCTGGTG	CC1
SMC11.T.GGTGAAGTCGTCCGTT	CC1
SMC11.T.GTACGTACAATGGTCT	CC1
SMC11.T.GTACTCCCACTGAAGG	CC1
SMC11.T.GTACTCCCAGTATAAG	CC1
SMC11.T.GTAGTCAGTCCCTTGT	CC1
SMC11.T.GTATTCTCATTCTTAC	CC1
SMC11.T.GTATTCTTCAATACCG	CC1
SMC11.T.GTCAAGTCATACCATG	CC1
SMC11.T.GTCATTTCATCCGGGT	CC1
SMC11.T.GTCCTCACATAACCTG	CC1
SMC11.T.GTCCTCATCAGAAATG	CC1
SMC11.T.GTCGGGTAGTGGAGAA	CC1
SMC11.T.GTCGTAAGTCCGAGTC	CC1
SMC11.T.GTCTCGTGTAGCTTGT	CC1
SMC11.T.GTGCATAGTATGAAAC	CC1
SMC11.T.GTGCTTCTCCCAAGTA	CC1
SMC11.T.GTGGGTCTCTGGTATG	CC1
SMC11.T.GTGTTAGCAGACACTT	CC1
SMC11.T.GTTAAGCGTCTGCCAG	CC1
SMC11.T.GTTAAGCGTCTTGATG	CC1
SMC11.T.GTTAAGCGTGCGCTTG	CC1
SMC11.T.GTTCGGGCATTGGCGC	CC1
SMC11.T.TAAACCGGTAGCACGA	CC1
SMC11.T.TAAGTGCGTCTTCAAG	CC1
SMC11.T.TAAGTGCTCAAAGTAG	CC1
SMC11.T.TAAGTGCTCTAACCGA	CC1

SMC11.T.TACACGAAGCTCCTCT	CC1
SMC11.T.TACAGTGCATAAGACA	CC1
SMC11.T.TACCTATCACGTCAGC	CC1
SMC11.T.TACCTATGTCGCTTCT	CC1
SMC11.T.TACGGGCAGCCTATGT	CC1
SMC11.T.TACGGGCCAAATCCGT	CC1
SMC11.T.TACTTACCATCGATTG	CC1
SMC11.T.TAGAGCTAGTAGATGT	CC1
SMC11.T.TAGAGCTAGTGGTAAT	CC1
SMC11.T.TAGCCGGAGTCAAGCG	CC1
SMC11.T.TAGCCGGTCTCCCTGA	CC1
SMC11.T.TAGTTGGGTGAAGGCT	CC1
SMC11.T.TATCAGGGTGGGTATG	CC1
SMC11.T.TATTACCGTCCGCTGA	CC1
SMC11.T.TCAACGAAGAAACGCC	CC1
SMC11.T.TCACGAATCGCAAACT	CC1
SMC11.T.TCAGATGTCTACCAGA	CC1
SMC11.T.TCAGCTCGTTAAAGAC	CC1
SMC11.T.TCAGGATTCTTACCTA	CC1
SMC11.T.TCATTACCAAATTGCC	CC1
SMC11.T.TCCCGATCACTATCTT	CC1
SMC11.T.TCCCGATTCCCATTTA	CC1
SMC11.T.TCGAGGCAGCTGCGAA	CC1
SMC11.T.TCGAGGCGTAAATACG	CC1
SMC11.T.TCGCGAGGTCCTGCTT	CC1
SMC11.T.TCGGTAATCACGGTTA	CC1
SMC11.T.TCGTACCAGAGGTACC	CC1
SMC11.T.TCGTAGAGTGGGTCAA	CC1
SMC11.T.TCGTAGATCCTCAATT	CC1
SMC11.T.TCTCTAACAGATCCAT	CC1
SMC11.T.TCTCTAATCACAATGC	CC1
SMC11.T.TGACGGCGTCAGCTAT	CC1
SMC11.T.TGACTTTAGCGGATCA	CC1
SMC11.T.TGAGGGAGTATGCTTG	CC1
SMC11.T.TGATTTCGTACTTCTT	CC1
SMC11.T.TGCGCAGTCTGCAAGT	CC1
SMC11.T.TGCGGGTTCGATGAGG	CC1
SMC11.T.TGCTACCAGACCTTTG	CC1
SMC11.T.TGCTGCTCAACCGCCA	CC1
SMC11.T.TGCTGCTCATGGGACA	CC1
SMC11.T.TGGCCAGGTATATGAG	CC1
SMC11.T.TGGCTGGAGCTAACAA	CC1
SMC11.T.TGGCTGGAGTTTCCTT	CC1
SMC11.T.TGGGCGTTCACAGTAC	CC1
SMC11.T.TGGTTAGCACCGAATT	CC1
SMC11.T.TGGTTAGCATTCACTT	CC1
SMC11.T.TGGTTAGTCGATCCCT	CC1
SMC11.T.TGTATTCGTATGAATG	CC1
SMC11.T.TGTGGTACATTGTGCA	CC1
SMC11.T.TGTTCCGAGTTGAGTA	CC1
SMC11.T.TGTTCCGTCGGTCCGA	CC1
SMC11.T.TTAACTCCACGGTAGA	CC1
SMC11.T.TTAGGACTCCTGTAGA	CC1
SMC11.T.TTAGGCACATAAGACA	CC1
SMC11.T.TTAGGCACATTCCTCG	CC1
SMC11.T.TTATGCTCACACCGCA	CC1
SMC11.T.TTATGCTTCTAACTCT	CC1
SMC11.T.TTCGAAGCAAGGACTG	CC1

SMC11.T.TTCGGTCAGGCTCTTA	CC1
SMC11.T.TTCGGTCCATCACGTA	CC1
SMC11.T.TTCTACATCTCTGAGA	CC1
SMC11.T.TTCTCAAAGCATCATC	CC1
SMC11.T.TTCTCAACACCATGTA	CC1
SMC11.T.TTCTCAAGTAATCGTC	CC1
SMC11.T.TTCTCCTGTACTCTCC	CC1
SMC11.T.TTCTTAGGTAGTACCT	CC1
SMC11.T.TTGACTTAGGCGCTCT	CC1
SMC11.T.TTGCCGTCAGCTTCGG	CC1
SMC11.T.TTGGAACGTCGTGGCT	CC1
SMC11.T.TTGGCAACACCGCTAG	CC1
SMC11.T.TTGTAGGTCCATGAGT	CC1
SMC11.T.TTTACTGAGCCGCCTA	CC1
SMC11.T.TTTCCTCGTCAGCTAT	CC1
SMC11.T.TTTCCTCTCCAGTATG	CC1
SMC14.T.AAACCTGCAGCTTCGG	CC1
SMC14.T.AACTCAGAGGAGTAGA	CC2
SMC14.T.AACTCCCTCCATGAGT	CC1
SMC14.T.AAGGTTCGTGAAGGCT	CC1
SMC14.T.ACACCGGGTCTAAAGA	CC1
SMC14.T.ACAGCTATCCATTCTA	CC1
SMC14.T.ACATGGTAGCTTTGGT	CC1
SMC14.T.ACGCCAGAGTATCGAA	CC1
SMC14.T.ACGGGCTCAGGATTGG	CC1
SMC14.T.ACTATCTAGTGTCCCG	CC2
SMC14.T.ACTGATGGTAGCGTAG	CC1
SMC14.T.ACTGCTCCAGCAGTTT	CC1
SMC14.T.ACTGTCCAGTGCAAGC	CC1
SMC14.T.ACTTTCACAGACAGGT	CC1
SMC14.T.ACTTTCACATCAGTAC	CC1
SMC14.T.AGAATAGTCTTTACAC	CC1
SMC14.T.AGACGTTTCAGGTTCA	CC1
SMC14.T.AGAGCTTGTAAACCTC	CC1
SMC14.T.AGCGGTCTCTGAGTGT	CC1
SMC14.T.AGGCCACGTTCTCATT	CC1
SMC14.T.AGGGAGTAGAGTAATC	CC2
SMC14.T.AGGGATGGTAGCGTGA	CC1
SMC14.T.AGGGTGATCAGAAATG	CC2
SMC14.T.AGGGTGATCTAACGGT	CC1
SMC14.T.ATAACGCCAGTCTTCC	CC1
SMC14.T.ATAAGAGTCGCCGTGA	CC1
SMC14.T.ATCATGGTCTAACTGG	CC1
SMC14.T.ATCCACCAGTTCGCGC	CC1
SMC14.T.ATCTACTAGGATCGCA	CC2
SMC14.T.ATGGGAGGTGGTCTCG	CC1
SMC14.T.ATTGGACAGGAGCGAG	CC1
SMC14.T.CAACCAAAGTTACGGG	CC1
SMC14.T.CAACCTCGTCCATGAT	CC1
SMC14.T.CACACCTAGAAAGTGG	CC1
SMC14.T.CACAGGCCAGACACTT	CC2
SMC14.T.CACAGTAAGATGTAAC	CC1
SMC14.T.CACCTTGAGTGTGGCA	CC2
SMC14.T.CACCTTGTCACCCGAG	CC1
SMC14.T.CAGAATCGTACGCACC	CC1
SMC14.T.CAGATCACACATGACT	CC2
SMC14.T.CAGCCGACATGCCTAA	CC1
SMC14.T.CAGGTGCCATTCGACA	CC1

SMC14.T.CAGTAACAGAGCAATT	CC2
SMC14.T.CAGTCCTAGGACAGAA	CC2
SMC14.T.CAGTCCTCAAACTGTC	CC1
SMC14.T.CATATGGAGATGTGGC	CC2
SMC14.T.CATCCACAGTACGTAA	CC2
SMC14.T.CATCCACGTTTAGCTG	CC2
SMC14.T.CATGGCGAGAGCTGGT	CC1
SMC14.T.CATTATCGTCGAAAGC	CC1
SMC14.T.CCACCTAGTTAAGGGC	CC1
SMC14.T.CCACCTATCACCTCGT	CC1
SMC14.T.CCATGTCCATGGGAAC	CC1
SMC14.T.CCATGTCTCAACACAC	CC1
SMC14.T.CCCAATCCACTATCTT	CC1
SMC14.T.CCCAATCTCCTCTAGC	CC1
SMC14.T.CCCTCCTGTAGCGTCC	CC1
SMC14.T.CCGTACTCAGATCCAT	CC1
SMC14.T.CCGTTCAGTAGAAAGG	CC1
SMC14.T.CCTAAAGAGATCCCGC	CC2
SMC14.T.CCTAAAGTCGACAGCC	CC2
SMC14.T.CCTACACGTCTTCAAG	CC1
SMC14.T.CCTAGCTAGGCAAAGA	CC1
SMC14.T.CCTAGCTAGTAGGTGC	CC1
SMC14.T.CCTAGCTGTGACGCCT	CC2
SMC14.T.CCTATTATCTCTTATG	CC1
SMC14.T.CCTCAGTAGCGATCCC	CC1
SMC14.T.CCTTCCCCATGCCACG	CC1
SMC14.T.CCTTCGAAGCGTGAAC	CC1
SMC14.T.CGAATGTTCACTCCTG	CC1
SMC14.T.CGAGCACGTCCAGTGC	CC2
SMC14.T.CGATTGAGTCAATGTC	CC2
SMC14.T.CGGACGTGTGCCTTGG	CC1
SMC14.T.CGGAGCTGTAGCAAAT	CC1
SMC14.T.CGGAGCTGTTATGTGC	CC1
SMC14.T.CGGAGCTGTTGCCTCT	CC1
SMC14.T.CGTGAGCAGCTAACAA	CC2
SMC14.T.CGTTAGACATGTCTCC	CC1
SMC14.T.CGTTCTGAGCGTGAAC	CC1
SMC14.T.CGTTCTGAGTGGTAAT	CC1
SMC14.T.CTACACCAGTTAAGTG	CC1
SMC14.T.CTACGTCTCGGCTTGG	CC2
SMC14.T.CTCGAAACAGCTATTG	CC1
SMC14.T.CTCGGGACACATGACT	CC2
SMC14.T.CTCTACGAGGACACCA	CC1
SMC14.T.CTCTGGTAGTGGACGT	CC1
SMC14.T.CTGATCCCAACTGCGC	CC2
SMC14.T.CTGTTTATCTTGGGTA	CC1
SMC14.T.CTTACCGAGCCTTGAT	CC1
SMC14.T.CTTCTCTCAGTATCTG	CC1
SMC14.T.GAAACTCCAGTGAGTG	CC1
SMC14.T.GAACATCGTTAAAGAC	CC2
SMC14.T.GAAGCAGCAATGACCT	CC1
SMC14.T.GAATAAGAGAAACCGC	CC2
SMC14.T.GACACGCTCCCACTTG	CC2
SMC14.T.GACTGCGCACGAGGTA	CC2
SMC14.T.GAGGTGAAGCGTGTCC	CC2
SMC14.T.GAGGTGATCTTGTACT	CC1
SMC14.T.GCAAACTGTCTAGCCG	CC2
SMC14.T.GCACTCTCACTCTGTC	CC2

SMC14.T.GCAGCCAAGCGTAATA	CC2
SMC14.T.GCAGCCATCAGTGCAT	CC1
SMC14.T.GCAGTTATCAGAGACG	CC2
SMC14.T.GCATACACACGTCAGC	CC2
SMC14.T.GCATGTATCGACGGAA	CC1
SMC14.T.GCGACCAAGCTATGCT	CC1
SMC14.T.GCGACCATCTGTTTGT	CC2
SMC14.T.GCGAGAATCAGAGACG	CC1
SMC14.T.GCGAGAATCAGGCGAA	CC2
SMC14.T.GCTCTGTTCATCTGCC	CC1
SMC14.T.GCTGCTTAGTGACTCT	CC2
SMC14.T.GCTTCCAAGAGCAATT	CC1
SMC14.T.GGAGCAAGTCTCCCTA	CC1
SMC14.T.GGATGTTTCCGTCATC	CC1
SMC14.T.GGCAATTAGCTTTGGT	CC2
SMC14.T.GGCGTGTGTGAGGCTA	CC1
SMC14.T.GGGCACTTCGCCATAA	CC1
SMC14.T.GGGTTGCAGCCCAACC	CC1
SMC14.T.GTACGTACATGGTCTA	CC1
SMC14.T.GTAGGCCTCAGATAAG	CC1
SMC14.T.GTCAAGTCAAGCCTAT	CC1
SMC14.T.GTCAAGTCAGCCAATT	CC1
SMC14.T.GTCACAATCACAAACC	CC1
SMC14.T.GTCACAATCCCAAGTA	CC1
SMC14.T.GTCATTTGTACCGTTA	CC1
SMC14.T.GTCTTCGAGCTTATCG	CC2
SMC14.T.GTGCATAGTCTCCCTA	CC1
SMC14.T.GTGGGTCAGCAATATG	CC2
SMC14.T.GTTCGGGGTGGTCCGT	CC1
SMC14.T.TAAGAGAAGACCACGA	CC1
SMC14.T.TAAGTGCCAAAGTGCG	CC1
SMC14.T.TACACGAAGACCGGAT	CC1
SMC14.T.TACCTTAGTTGTGGCC	CC2
SMC14.T.TACGGTAAGCTAGCCC	CC2
SMC14.T.TACTCGCAGCTACCTA	CC2
SMC14.T.TACTTGTAGATGTCGG	CC1
SMC14.T.TACTTGTTCATCTGCC	CC1
SMC14.T.TACTTGTTCTAAGCCA	CC2
SMC14.T.TAGAGCTCATGCAACT	CC2
SMC14.T.TATCAGGTCGAATGCT	CC1
SMC14.T.TCAGGTACAACGATCT	CC1
SMC14.T.TCGGTAAAGATCCTGT	CC2
SMC14.T.TCGTACCGTAAATACG	CC1
SMC14.T.TCTATTGTCTTGTCAT	CC1
SMC14.T.TCTCATATCAGCACAT	CC2
SMC14.T.TCTCTAAGTCAATACC	CC2
SMC14.T.TCTTTCCCAGGACGTA	CC2
SMC14.T.TGACTAGGTATCTGCA	CC2
SMC14.T.TGACTAGGTTGTGGCC	CC2
SMC14.T.TGACTTTAGCTTTGGT	CC2
SMC14.T.TGATTTCGTCCATGAT	CC1
SMC14.T.TGCACCTAGTACTTGC	CC1
SMC14.T.TGCCCTACACAGACTT	CC1
SMC14.T.TGCGGGTAGTGAACGC	CC2
SMC14.T.TGCTACCGTTACCAGT	CC1
SMC14.T.TGCTACCGTTCGGGCT	CC2
SMC14.T.TGCTGCTGTCCAGTGC	CC2
SMC14.T.TGCTGCTGTGACGCCT	CC2

SMC14.T.TGGCCAGTCGCAAACT	CC1
SMC14.T.TGGCTGGAGATCCTGT	CC1
SMC14.T.TGGTTAGCAGCCTATA	CC1
SMC14.T.TGTGTTTCACGAGGTA	CC2
SMC14.T.TTAACTCCAGCCTTGG	CC1
SMC14.T.TTAGGCAAGCCACTAT	CC2
SMC14.T.TTGGAACCACTGTTAG	CC1
SMC14.T.TTTGCGCAGGCATTGG	CC2
SMC15.T.AAACGGGCAAGACGTG	CC1
SMC15.T.AAAGATGCAGTCGATT	CC1
SMC15.T.AAAGATGTCCTCATTA	CC1
SMC15.T.AAAGCAAAGTACATGA	CC1
SMC15.T.AAAGCAAGTGCTAGCC	CC1
SMC15.T.AAAGCAATCCGTAGGC	CC2
SMC15.T.AAATGCCTCTATCGCC	CC1
SMC15.T.AACACGTCAATGCCAT	CC1
SMC15.T.AACGTTGAGCGATATA	CC2
SMC15.T.AACGTTGGTAAGAGAG	CC2
SMC15.T.AACGTTGTCAGCCTAA	CC1
SMC15.T.AACTCAGGTCTCTCTG	CC2
SMC15.T.AACTTTCTCATGCATG	CC1
SMC15.T.AAGGAGCGTTCACGGC	CC1
SMC15.T.AATCCAGTCGAATGCT	CC1
SMC15.T.ACACCAACACCGATAT	CC1
SMC15.T.ACACCAAGTGCGAAAC	CC1
SMC15.T.ACACCCTGTTACGCGC	CC1
SMC15.T.ACACCCTTCAACTCTT	CC1
SMC15.T.ACACCGGAGCCGATTT	CC1
SMC15.T.ACACCGGTCAAAGACA	CC1
SMC15.T.ACAGCTAGTGCTAGCC	CC1
SMC15.T.ACAGCTATCTTCCTTC	CC1
SMC15.T.ACATCAGGTCACCTAA	CC2
SMC15.T.ACATCAGGTGCCTGCA	CC2
SMC15.T.ACATGGTAGATCTGCT	CC1
SMC15.T.ACCGTAATCAAAGTAG	CC1
SMC15.T.ACCTTTACACTGTTAG	CC1
SMC15.T.ACGAGGAAGATGGCGT	CC2
SMC15.T.ACGCAGCAGGAGTAGA	CC1
SMC15.T.ACGGCCACAGGGTACA	CC2
SMC15.T.ACGGGTCAGCGATGAC	CC1
SMC15.T.ACTATCTCAGACAGGT	CC1
SMC15.T.ACTATCTGTCAACTGT	CC2
SMC15.T.ACTGAGTGTGAACCTT	CC1
SMC15.T.ACTGTCCAGAGGACGG	CC1
SMC15.T.ACTGTCCGTACCGCTG	CC1
SMC15.T.ACTTGTTCAAGTCTAC	CC1
SMC15.T.ACTTGTTTCGTAGGAG	CC1
SMC15.T.AGAATAGTCCGAAGAG	CC1
SMC15.T.AGAATAGTCTGGTATG	CC1
SMC15.T.AGACGTTCAAGTTGTC	CC2
SMC15.T.AGACGTTCATGAAGTA	CC1
SMC15.T.AGACGTTGTCTGCAAT	CC1
SMC15.T.AGAGCTTCAGCTTCGG	CC1
SMC15.T.AGATCTGAGCGACGTA	CC2
SMC15.T.AGATTGCAGGAATCGC	CC1
SMC15.T.AGATTGCCATGGGAAC	CC1
SMC15.T.AGCAGCCTCCGGCACA	CC1
SMC15.T.AGCGTATTCTGCTTGC	CC1

SMC15.T.AGCTCCTCATCACGAT	CC1
SMC15.T.AGCTCCTGTCTCCATC	CC1
SMC15.T.AGCTCCTTCTCGAGTA	CC1
SMC15.T.AGGCCACTCAAACCGT	CC1
SMC15.T.AGGCCGTGTCACCTAA	CC1
SMC15.T.AGGGATGAGAACAACT	CC1
SMC15.T.AGGGATGAGGGTTTCT	CC2
SMC15.T.AGGTCCGCAAGGTTTC	CC1
SMC15.T.AGTCTTTAGAACAACT	CC2
SMC15.T.AGTGGGAAGTATCTCG	CC1
SMC15.T.ATAAGAGAGATCTGCT	CC1
SMC15.T.ATAGACCTCAATCACG	CC1
SMC15.T.ATCATCTTCGGAGCAA	CC1
SMC15.T.ATCGAGTGTCTTCAAG	CC1
SMC15.T.ATGAGGGCAAGAGTCG	CC1
SMC15.T.ATGCGATAGACGCTTT	CC1
SMC15.T.ATGCGATCACGCGAAA	CC1
SMC15.T.ATGCGATTCTAAGCCA	CC2
SMC15.T.ATGTGTGTCTCATTCA	CC1
SMC15.T.ATTACTCGTAAGAGGA	CC1
SMC15.T.ATTGGTGAGTCGTTTG	CC1
SMC15.T.ATTTCTGTCTCCTATA	CC2
SMC15.T.CAACCTCTCTGTTGAG	CC2
SMC15.T.CAACTAGCAATGAATG	CC1
SMC15.T.CAAGAAAGTCTCACCT	CC1
SMC15.T.CAAGGCCTCAAGAAGT	CC1
SMC15.T.CAAGGCCTCAGTCAGT	CC1
SMC15.T.CACAAACAGAGCTGCA	CC1
SMC15.T.CACAAACCAAACCCAT	CC1
SMC15.T.CACACAACAAAGGTGC	CC2
SMC15.T.CACACTCGTTAGATGA	CC1
SMC15.T.CACAGGCCATCCTAGA	CC1
SMC15.T.CACATAGCATTGTGCA	CC2
SMC15.T.CACATAGTCACCCTCA	CC2
SMC15.T.CACCACTGTCGCTTTC	CC1
SMC15.T.CACCAGGCACAGCGTC	CC1
SMC15.T.CACCAGGGTAGCCTCG	CC1
SMC15.T.CAGAGAGCAAGGACAC	CC1
SMC15.T.CAGAGAGCAATGCCAT	CC1
SMC15.T.CAGAGAGGTTGAACTC	CC1
SMC15.T.CAGAGAGTCGAATGCT	CC1
SMC15.T.CAGATCACATGCATGT	CC1
SMC15.T.CAGCAGCCATGAACCT	CC1
SMC15.T.CAGCAGCGTATGAAAC	CC2
SMC15.T.CAGCCGACAGTCCTTC	CC1
SMC15.T.CAGCGACAGTACGCCC	CC2
SMC15.T.CAGCGACGTACGCTGC	CC1
SMC15.T.CAGCGACGTTACCAGT	CC1
SMC15.T.CAGGTGCGTCAGAAGC	CC1
SMC15.T.CAGTCCTTCTCGCATC	CC1
SMC15.T.CATATGGGTGGCCCTA	CC1
SMC15.T.CATCAAGAGTCATGCT	CC1
SMC15.T.CATCAGACACTGTCGG	CC1
SMC15.T.CATCGAAAGCCAGAAC	CC1
SMC15.T.CATCGGGTCGAGAGCA	CC1
SMC15.T.CATGACAAGTGCGTGA	CC1
SMC15.T.CATGACACATTCTTAC	CC1
SMC15.T.CATGCCTGTCGATTGT	CC2

SMC15.T.CATTATCCACAGACTT	CC2
SMC15.T.CATTATCTCCACGACG	CC1
SMC15.T.CATTCGCGTGTGCGTC	CC1
SMC15.T.CCACCTAGTATTCTCT	CC1
SMC15.T.CCCATACTCGTCCGTT	CC1
SMC15.T.CCGTACTTCATGCAAC	CC1
SMC15.T.CCGTGGAAGGGTTCCC	CC1
SMC15.T.CCGTTCACATGTCCTC	CC1
SMC15.T.CCTAGCTCATATGGTC	CC1
SMC15.T.CCTAGCTTCGATAGAA	CC1
SMC15.T.CCTATTAGTCTAACGT	CC1
SMC15.T.CCTCAGTGTGATGATA	CC1
SMC15.T.CCTCTGACATTTCAGG	CC1
SMC15.T.CCTTACGAGCCCGAAA	CC1
SMC15.T.CCTTACGTCCTAGGGC	CC1
SMC15.T.CCTTCCCTCCGATATG	CC2
SMC15.T.CGAACATAGGTGCACA	CC2
SMC15.T.CGAACATCAAGACGTG	CC1
SMC15.T.CGAACATCATGTCGAT	CC1
SMC15.T.CGAATGTGTTGATTGC	CC1
SMC15.T.CGAATGTTCGGTTCGG	CC1
SMC15.T.CGACCTTCATGTAGTC	CC1
SMC15.T.CGACCTTGTTAAGACA	CC1
SMC15.T.CGAGCACCAGACGCTC	CC1
SMC15.T.CGATCGGCATATACGC	CC1
SMC15.T.CGATGTAGTGAGGGAG	CC1
SMC15.T.CGATGTATCATGTGGT	CC1
SMC15.T.CGATTGACAGCTTAAC	CC1
SMC15.T.CGCCAAGAGTTGTCGT	CC1
SMC15.T.CGCGTTTGTAAGGGCT	CC1
SMC15.T.CGCTTCAAGTGCCAGA	CC1
SMC15.T.CGGACACAGAAGGTGA	CC1
SMC15.T.CGGACGTTCTGGGCCA	CC1
SMC15.T.CGGAGCTGTATCTGCA	CC2
SMC15.T.CGGTTAATCCCATTAT	CC1
SMC15.T.CGTAGCGGTCATACTG	CC1
SMC15.T.CGTAGGCGTCTAACGT	CC1
SMC15.T.CGTCAGGCACCAGGCT	CC1
SMC15.T.CGTCAGGTCATACGGT	CC1
SMC15.T.CGTCCATCAAGTCTAC	CC1
SMC15.T.CGTCTACTCCTCGCAT	CC1
SMC15.T.CGTGAGCCAATGGACG	CC2
SMC15.T.CGTGAGCGTCTCTCGT	CC1
SMC15.T.CGTGTCTGTGTAAGTA	CC1
SMC15.T.CGTGTCTTCTATCCCG	CC1
SMC15.T.CGTTGGGCACCGAAAG	CC1
SMC15.T.CTAACTTGTTTGACAC	CC1
SMC15.T.CTAACTTGTTTGTTGG	CC2
SMC15.T.CTAAGACCACGGTAGA	CC1
SMC15.T.CTAATGGAGACGCTTT	CC1
SMC15.T.CTACCCATCTTTAGGG	CC1
SMC15.T.CTAGCCTCATGTCGAT	CC1
SMC15.T.CTAGCCTTCCTATGTT	CC1
SMC15.T.CTAGTGAAGACCTTTG	CC1
SMC15.T.CTCATTAGTTCCACAA	CC1
SMC15.T.CTCGAGGAGAACTCGG	CC2
SMC15.T.CTCGAGGCAACACGCC	CC1
SMC15.T.CTCTAATAGCGCCTCA	CC1

SMC15.T.CTCTAATAGGAATGGA	CC1
SMC15.T.CTCTAATCAACAACCT	CC1
SMC15.T.CTCTACGTCTCGCTTG	CC1
SMC15.T.CTGAAGTGTCTAGCCG	CC1
SMC15.T.CTGATAGGTCTCTTTA	CC2
SMC15.T.CTGATAGTCGGTTCGG	CC2
SMC15.T.CTGATCCCAAGTCTGT	CC1
SMC15.T.CTGATCCCAATGGAGC	CC1
SMC15.T.CTGATCCCATCACGTA	CC1
SMC15.T.CTGATCCCATGTCCTC	CC1
SMC15.T.CTGCGGAGTCCGAAGA	CC1
SMC15.T.CTGCTGTAGCGTGTCC	CC1
SMC15.T.CTGTGCTAGATGTTAG	CC1
SMC15.T.CTTAACTCATACGCTA	CC1
SMC15.T.CTTACCGCATGCATGT	CC1
SMC15.T.GAAACTCAGCCCGAAA	CC1
SMC15.T.GAAACTCAGCGTCAAG	CC1
SMC15.T.GAAACTCTCGGTCCGA	CC1
SMC15.T.GAAACTCTCGTAGGAG	CC1
SMC15.T.GAAATGACAGCGTAAG	CC1
SMC15.T.GAACATCGTACCTACA	CC1
SMC15.T.GAACCTATCGAATGCT	CC1
SMC15.T.GAAGCAGGTTTGACTG	CC1
SMC15.T.GACCAATCAGCTCGCA	CC1
SMC15.T.GACCTGGCAAACCCAT	CC1
SMC15.T.GACCTGGGTGTTTGTG	CC1
SMC15.T.GACGCGTTCATTATCC	CC1
SMC15.T.GAGCAGAAGACAGACC	CC1
SMC15.T.GAGTCCGGTTTGACAC	CC1
SMC15.T.GATCAGTAGCAGCGTA	CC1
SMC15.T.GATCGATAGCCATCGC	CC1
SMC15.T.GATCGTAAGTGTACTC	CC1
SMC15.T.GATGCTAGTCCTCCAT	CC2
SMC15.T.GCAGTTACAGGAATCG	CC2
SMC15.T.GCATACACACGTCTCT	CC1
SMC15.T.GCATGTACACATTTCT	CC2
SMC15.T.GCGGGTTGTAGCTTGT	CC1
SMC15.T.GCTCCTAAGCGGCTTC	CC1
SMC15.T.GCTCCTAAGTGTTGAA	CC1
SMC15.T.GCTCTGTCACAGTCGC	CC2
SMC15.T.GCTCTGTGTATAGGGC	CC1
SMC15.T.GCTGCAGCAAGAGTCG	CC2
SMC15.T.GCTGCAGTCTTCATGT	CC1
SMC15.T.GCTGCGACACTCAGGC	CC1
SMC15.T.GCTGCTTAGCGATAGC	CC1
SMC15.T.GCTGCTTTCAGTTAGC	CC1
SMC15.T.GCTTCCAAGTGAATTG	CC1
SMC15.T.GGAAAGCTCACCACCT	CC1
SMC15.T.GGACGTCCAGACGCCT	CC1
SMC15.T.GGAGCAACATCGATTG	CC1
SMC15.T.GGAGCAAGTGCACGAA	CC1
SMC15.T.GGATTACCACAACTGT	CC1
SMC15.T.GGCCGATGTTTGTTTC	CC1
SMC15.T.GGCGACTAGGTGCACA	CC1
SMC15.T.GGGACCTAGGGTGTGT	CC1
SMC15.T.GGGATGAGTTGGTTTG	CC1
SMC15.T.GGGATGATCTATCCCG	CC2
SMC15.T.GGGCACTAGATGGCGT	CC2

SMC15.T.GGGCACTAGATGTTAG	CC1
SMC15.T.GGGCATCAGACTAGAT	CC1
SMC15.T.GGGCATCAGCAGATCG	CC1
SMC15.T.GGGTCTGCAAACAACA	CC1
SMC15.T.GGGTTGCCAGCTATTG	CC1
SMC15.T.GGGTTGCGTGTAACGG	CC1
SMC15.T.GTACTCCGTCGGATCC	CC1
SMC15.T.GTACTTTAGTAAGTAC	CC1
SMC15.T.GTAGTCAGTGCACGAA	CC1
SMC15.T.GTATCTTAGCTAGTGG	CC1
SMC15.T.GTATTCTGTACTCAAC	CC1
SMC15.T.GTCAAGTGTTCACGGC	CC2
SMC15.T.GTCAAGTTCACATGCA	CC1
SMC15.T.GTCACGGGTACTCTCC	CC1
SMC15.T.GTCACGGGTGTGAAAT	CC1
SMC15.T.GTCGGGTAGGCTAGGT	CC1
SMC15.T.GTCGGGTAGTTTCCTT	CC1
SMC15.T.GTCGGGTGTGATGTCT	CC1
SMC15.T.GTCTTCGCACAACTGT	CC1
SMC15.T.GTCTTCGGTAGAGTGC	CC1
SMC15.T.GTCTTCGGTCCTGCTT	CC1
SMC15.T.GTGCAGCAGCCGTCGT	CC2
SMC15.T.GTGCAGCGTCCTCTTG	CC1
SMC15.T.GTGCATAGTCGCCATG	CC1
SMC15.T.GTGCATAGTGTCGCTG	CC1
SMC15.T.GTGTTAGTCTCCTATA	CC1
SMC15.T.GTTAAGCCATGCAACT	CC2
SMC15.T.GTTCATTGTGGTGTAG	CC2
SMC15.T.GTTCGGGCAGGTCTCG	CC2
SMC15.T.TAAACCGCAATCGAAA	CC1
SMC15.T.TAAGAGACAACGATGG	CC1
SMC15.T.TACACGAAGGCATTGG	CC1
SMC15.T.TACCTTAGTTCGTGAT	CC1
SMC15.T.TACCTTATCACCACCT	CC1
SMC15.T.TACGGTAAGTATTGGA	CC1
SMC15.T.TACTCGCAGGAGTTGC	CC2
SMC15.T.TACTCGCTCAGGTTCA	CC1
SMC15.T.TACTTACTCAACACTG	CC1
SMC15.T.TACTTACTCAACGGGA	CC1
SMC15.T.TAGACCATCCTAGAAC	CC1
SMC15.T.TAGAGCTGTTACGTCA	CC1
SMC15.T.TAGGCATAGCCGATTT	CC2
SMC15.T.TATCAGGTCCTCGCAT	CC1
SMC15.T.TATCTCATCAACGCTA	CC1
SMC15.T.TATGCCCCACATCTTT	CC1
SMC15.T.TATTACCTCATCGCTC	CC1
SMC15.T.TCAACGATCTCCAGGG	CC1
SMC15.T.TCAATCTTCGCAAGCC	CC2
SMC15.T.TCACAAGAGGAGTTTA	CC1
SMC15.T.TCACGAACACATGGGA	CC2
SMC15.T.TCAGATGAGAGATGAG	CC2
SMC15.T.TCAGATGAGTTAGGTA	CC1
SMC15.T.TCAGGATGTGACGCCT	CC1
SMC15.T.TCAGGTACACTTCGAA	CC1
SMC15.T.TCAGGTAGTAGAGTGC	CC1
SMC15.T.TCAGGTATCGGTGTTA	CC1
SMC15.T.TCATTACAGTGGACGT	CC2
SMC15.T.TCATTTGAGAGTACAT	CC2

SMC15.T.TCATTTGCATGGGACA	CC2
SMC15.T.TCCACACCACATCCAA	CC1
SMC15.T.TCGCGAGCATACCATG	CC1
SMC15.T.TCGTAGAAGCGATAGC	CC1
SMC15.T.TCTCTAATCCCTAACC	CC1
SMC15.T.TCTCTAATCGACGGAA	CC1
SMC15.T.TCTTTCCTCAGCGATT	CC1
SMC15.T.TGACAACCAGTGACAG	CC1
SMC15.T.TGACGGCAGGTGCAAC	CC1
SMC15.T.TGACGGCCAAAGCGGT	CC1
SMC15.T.TGAGCCGGTCAGCTAT	CC1
SMC15.T.TGCACCTAGGAGTCTG	CC1
SMC15.T.TGCCCATAGATCCCGC	CC1
SMC15.T.TGCCCTACAGCTCGAC	CC1
SMC15.T.TGCGGGTCAGGAATGC	CC1
SMC15.T.TGCGGGTCAGTATCTG	CC1
SMC15.T.TGCGGGTTCACCATAG	CC1
SMC15.T.TGCGTGGCAGTATCTG	CC1
SMC15.T.TGCGTGGGTACGACCC	CC1
SMC15.T.TGCTGCTAGTATCTCG	CC1
SMC15.T.TGGCCAGGTTCCCGAG	CC1
SMC15.T.TGGCTGGCACCAACCG	CC1
SMC15.T.TGGTTCCGTCCGAGTC	CC1
SMC15.T.TGTATTCAGCACCGCT	CC2
SMC15.T.TGTCCCAGTCGCATAT	CC1
SMC15.T.TGTGTTTAGCTAGTCT	CC1
SMC15.T.TGTGTTTGTCATTAGC	CC1
SMC15.T.TGTTCCGAGCTAACTC	CC1
SMC15.T.TTCCCAGAGTACGTAA	CC1
SMC15.T.TTCGGTCCAGTTCCCT	CC2
SMC15.T.TTCGGTCGTACTTCTT	CC1
SMC15.T.TTGCGTCCAGCTATTG	CC1
SMC15.T.TTGCGTCCATTAGCCA	CC1
SMC15.T.TTGGCAACAGCTCCGA	CC1
SMC15.T.TTGGCAATCGGCTACG	CC2
SMC15.T.TTGGCAATCGTTTATC	CC1
SMC15.T.TTTATGCGTCTCTCGT	CC1
SMC16.T.AAACCTGGTTCGCGAC	CC1
SMC16.T.AAACCTGTCAGCTTAG	CC1
SMC16.T.AAACGGGAGTAGCGGT	CC2
SMC16.T.AAACGGGCAGACAGGT	CC2
SMC16.T.AAACGGGCAGACGCAA	CC2
SMC16.T.AAACGGGCAGCCTTTC	CC1
SMC16.T.AAACGGGCAGCTCGAC	CC1
SMC16.T.AAACGGGGTCTAGGTT	CC2
SMC16.T.AAACGGGTCGCATGGC	CC1
SMC16.T.AAACGGGTCTGCAAGT	CC2
SMC16.T.AAAGATGAGACGCACA	CC2
SMC16.T.AAAGATGAGAGAGCTC	CC2
SMC16.T.AAAGATGCAAGGCTCC	CC2
SMC16.T.AAAGATGCACAGACAG	CC1
SMC16.T.AAAGATGCACTCGACG	CC2
SMC16.T.AAAGATGGTAGCGTGA	CC1
SMC16.T.AAAGATGGTCAATACC	CC1
SMC16.T.AAAGCAAAGAGACTTA	CC1
SMC16.T.AAAGCAAAGAGCTATA	CC2
SMC16.T.AAAGCAAAGTGGAGTC	CC2
SMC16.T.AAAGCAAAGTGTACGG	CC2

SMC16.T.AAAGCAACACATTTCT	CC1
SMC16.T.AAAGCAAGTCTTGCGG	CC2
SMC16.T.AAAGCAAGTGGCTCCA	CC2
SMC16.T.AAAGCAAGTTTACTCT	CC2
SMC16.T.AAAGCAATCCACGTTC	CC1
SMC16.T.AAAGCAATCGGCGGTT	CC2
SMC16.T.AAAGTAGCACAAGCCC	CC2
SMC16.T.AAAGTAGCACTACAGT	CC2
SMC16.T.AAAGTAGGTACCTACA	CC2
SMC16.T.AAAGTAGGTCCTAGCG	CC2
SMC16.T.AAAGTAGTCTGCGTAA	CC2
SMC16.T.AAATGCCCACCAGATT	CC2
SMC16.T.AAATGCCCACTGTGTA	CC2
SMC16.T.AAATGCCGTACAGTTC	CC1
SMC16.T.AAATGCCGTCTCTCGT	CC2
SMC16.T.AAATGCCTCGCTGATA	CC2
SMC16.T.AAATGCCTCTTAACCT	CC1
SMC16.T.AACACGTAGGCGATAC	CC2
SMC16.T.AACACGTAGGTGCTTT	CC2
SMC16.T.AACACGTAGTACGCCC	CC2
SMC16.T.AACACGTCAATAGCAA	CC1
SMC16.T.AACACGTGTTGGGACA	CC1
SMC16.T.AACACGTTCACAATGC	CC1
SMC16.T.AACACGTTCTCCCTGA	CC2
SMC16.T.AACACGTTCTTAGAGC	CC2
SMC16.T.AACCATGCACGGTTTA	CC2
SMC16.T.AACCATGCAGCTTCGG	CC2
SMC16.T.AACCATGGTCTCGTTC	CC1
SMC16.T.AACCATGGTCTTCTCG	CC2
SMC16.T.AACCATGGTTGTGGAG	CC1
SMC16.T.AACCATGTCGCTGATA	CC2
SMC16.T.AACCATGTCTGCTGCT	CC1
SMC16.T.AACCGCGAGAAACGAG	CC2
SMC16.T.AACCGCGAGACTCGGA	CC2
SMC16.T.AACCGCGGTACAGTGG	CC2
SMC16.T.AACCGCGGTATCGCAT	CC2
SMC16.T.AACGTTGAGCATCATC	CC2
SMC16.T.AACGTTGAGCCGTCGT	CC2
SMC16.T.AACGTTGCAAGGTTCT	CC1
SMC16.T.AACGTTGCACTATCTT	CC2
SMC16.T.AACGTTGGTCATCGGC	CC2
SMC16.T.AACGTTGTCTCATTCA	CC2
SMC16.T.AACTCAGAGAGACGAA	CC1
SMC16.T.AACTCAGAGCATCATC	CC2
SMC16.T.AACTCAGCACCGCTAG	CC1
SMC16.T.AACTCAGCATTCGACA	CC1
SMC16.T.AACTCAGTCAACACAC	CC2
SMC16.T.AACTCAGTCATTGCCC	CC2
SMC16.T.AACTCCCAGTTGTAGA	CC2
SMC16.T.AACTCCCTCCGCAGTG	CC1
SMC16.T.AACTCCCTCGCATGAT	CC1
SMC16.T.AACTCCCTCTGGTTCC	CC1
SMC16.T.AACTCTTAGTTAGCGG	CC2
SMC16.T.AACTCTTGTACTCGCG	CC1
SMC16.T.AACTCTTGTAGGCATG	CC1
SMC16.T.AACTCTTGTCTCTTTA	CC1
SMC16.T.AACTCTTGTTACGGAG	CC2
SMC16.T.AACTGGTAGGTGCTTT	CC1

SMC16.T.AACTGGTCATGAACCT	CC2
SMC16.T.AACTGGTGTCATATGC	CC2
SMC16.T.AACTTTCAGAATTGTG	CC1
SMC16.T.AACTTTCCAAGGTTTC	CC2
SMC16.T.AACTTTCTCCGTTGCT	CC2
SMC16.T.AAGACCTAGGACATTA	CC2
SMC16.T.AAGACCTAGGGCTTCC	CC2
SMC16.T.AAGACCTAGTGCGATG	CC2
SMC16.T.AAGACCTCACGAGAGT	CC2
SMC16.T.AAGACCTCAGTAAGCG	CC2
SMC16.T.AAGACCTGTCTCTCTG	CC2
SMC16.T.AAGACCTGTTACGGAG	CC2
SMC16.T.AAGCCGCAGTGCAAGC	CC2
SMC16.T.AAGCCGCCACAACTGT	CC2
SMC16.T.AAGCCGCCACAGACAG	CC1
SMC16.T.AAGCCGCGTCGACTGC	CC2
SMC16.T.AAGCCGCGTTGCGTTA	CC1
SMC16.T.AAGGAGCAGCACGCCT	CC2
SMC16.T.AAGGAGCCAAGCCGTC	CC1
SMC16.T.AAGGAGCCAATCACAC	CC2
SMC16.T.AAGGAGCCATCATCCC	CC2
SMC16.T.AAGGAGCCATGGTCTA	CC2
SMC16.T.AAGGAGCGTACGCTGC	CC1
SMC16.T.AAGGAGCGTATAGGTA	CC2
SMC16.T.AAGGAGCTCATATCGG	CC2
SMC16.T.AAGGAGCTCCGCAGTG	CC2
SMC16.T.AAGGAGCTCCTATGTT	CC1
SMC16.T.AAGGAGCTCCTCAATT	CC2
SMC16.T.AAGGAGCTCGCCAAAT	CC2
SMC16.T.AAGGCAGAGAAACGCC	CC1
SMC16.T.AAGGCAGAGTACGTTC	CC2
SMC16.T.AAGGCAGCACACAGAG	CC2
SMC16.T.AAGGCAGCACGGCCAT	CC1
SMC16.T.AAGGCAGCAGCCAATT	CC1
SMC16.T.AAGGCAGTCACTTCAT	CC2
SMC16.T.AAGGCAGTCAGGATCT	CC2
SMC16.T.AAGGCAGTCCGAAGAG	CC1
SMC16.T.AAGGTTCAGAACAATC	CC2
SMC16.T.AAGGTTCAGAGCTGGT	CC1
SMC16.T.AAGGTTCTCCATTCTA	CC1
SMC16.T.AAGTCTGAGAGACTAT	CC2
SMC16.T.AAGTCTGCATGGTAGG	CC2
SMC16.T.AATCCAGAGACTTTCG	CC1
SMC16.T.AATCCAGAGCCCAATT	CC2
SMC16.T.AATCCAGCAAAGGTGC	CC1
SMC16.T.AATCCAGCACCGCTAG	CC2
SMC16.T.AATCCAGCAGTATAAG	CC2
SMC16.T.AATCCAGGTCAGATAA	CC1
SMC16.T.AATCCAGGTGATGATA	CC1
SMC16.T.AATCCAGTCAAACCGT	CC2
SMC16.T.AATCCAGTCTTGTATC	CC2
SMC16.T.AATCGGTAGCCCAACC	CC1
SMC16.T.AATCGGTAGTAACCCT	CC2
SMC16.T.AATCGGTCAGCTTCGG	CC1
SMC16.T.AATCGGTTCATTGCCC	CC1
SMC16.T.AATCGGTTCCTTGGTC	CC1
SMC16.T.ACACCAAAGCTGGAAC	CC1
SMC16.T.ACACCAACAGCCTTGG	CC1

SMC16.T.ACACCAACATTCTTAC	CC1
SMC16.T.ACACCAATCATGGTCA	CC2
SMC16.T.ACACCCTAGCTGATAA	CC1
SMC16.T.ACACCCTCACATTAGC	CC1
SMC16.T.ACACCCTCACGAAATA	CC1
SMC16.T.ACACCCTGTCCGACGT	CC1
SMC16.T.ACACCCTTCCACGTTC	CC2
SMC16.T.ACACCCTTCCTTAATC	CC2
SMC16.T.ACACCCTTCGATCCCT	CC2
SMC16.T.ACACCCTTCTATCGCC	CC2
SMC16.T.ACACCCTTCTTCCTTC	CC1
SMC16.T.ACACCGGGTGCGCTTG	CC2
SMC16.T.ACACCGGGTTTGCATG	CC2
SMC16.T.ACACCGGTCTCGCTTG	CC1
SMC16.T.ACACTGAAGAACTCGG	CC1
SMC16.T.ACACTGAGTTCTGTTT	CC2
SMC16.T.ACACTGATCATAAAGG	CC2
SMC16.T.ACACTGATCTCGTATT	CC2
SMC16.T.ACACTGATCTCTTATG	CC2
SMC16.T.ACACTGATCTTCTGGC	CC2
SMC16.T.ACAGCCGAGTCAAGGC	CC2
SMC16.T.ACAGCCGAGTCGATAA	CC1
SMC16.T.ACAGCCGGTACTTGAC	CC1
SMC16.T.ACAGCCGGTAGCGATG	CC2
SMC16.T.ACAGCCGGTCCATGAT	CC2
SMC16.T.ACAGCCGTCCAGTAGT	CC2
SMC16.T.ACAGCTAAGAGCAATT	CC2
SMC16.T.ACAGCTAAGGGATACC	CC1
SMC16.T.ACAGCTACACCCATTC	CC2
SMC16.T.ACAGCTACAGGCTCAC	CC2
SMC16.T.ACAGCTACAGGTTTCA	CC2
SMC16.T.ACAGCTAGTCAAAGCG	CC1
SMC16.T.ACAGCTAGTCTCTTAT	CC1
SMC16.T.ACAGCTATCGGACAAG	CC2
SMC16.T.ACAGCTATCGTACGGC	CC1
SMC16.T.ACAGCTATCTGGGCCA	CC2
SMC16.T.ACATACGAGAGGTTGC	CC1
SMC16.T.ACATACGAGCCACCTG	CC1
SMC16.T.ACATACGAGTACGCGA	CC2
SMC16.T.ACATACGAGTGAACGC	CC1
SMC16.T.ACATACGAGTTCGATC	CC2
SMC16.T.ACATACGCACGAGGTA	CC2
SMC16.T.ACATACGCAGTTAACC	CC1
SMC16.T.ACATACGTCAAAGACA	CC1
SMC16.T.ACATACGTCAGAGACG	CC2
SMC16.T.ACATACGTCAGAGGTG	CC2
SMC16.T.ACATCAGAGATAGCAT	CC2
SMC16.T.ACATCAGAGCTATGCT	CC1
SMC16.T.ACATCAGAGGACATTA	CC2
SMC16.T.ACATCAGGTCGCTTTC	CC2
SMC16.T.ACATCAGGTTGGTGGA	CC2
SMC16.T.ACATCAGTCTCAACTT	CC2
SMC16.T.ACATGGTAGCAACGGT	CC2
SMC16.T.ACATGGTAGGAGCGTT	CC2
SMC16.T.ACATGGTCAATAGCGG	CC2
SMC16.T.ACATGGTCATGTCGAT	CC2
SMC16.T.ACATGGTTCAATCACG	CC2
SMC16.T.ACCAGTAAGCACAGGT	CC1

SMC16.T.ACCAGTAAGTCGATAA	CC1
SMC16.T.ACCAGTACAATCTGCA	CC2
SMC16.T.ACCAGTACAGACTCGC	CC1
SMC16.T.ACCAGTAGTAATCGTC	CC2
SMC16.T.ACCAGTAGTCCGAACC	CC2
SMC16.T.ACCAGTATCTTTCCTC	CC2
SMC16.T.ACCCACTAGTCAAGGC	CC1
SMC16.T.ACCCACTCAAGAGTCG	CC2
SMC16.T.ACCCACTCAGACGTAG	CC1
SMC16.T.ACCCACTTCAACACTG	CC1
SMC16.T.ACCCACTTCCGCGGTA	CC2
SMC16.T.ACCCACTTCGACCAGC	CC2
SMC16.T.ACCGTAAAGCATGGCA	CC1
SMC16.T.ACCGTAAAGCTGATAA	CC2
SMC16.T.ACCGTAAGTGTGACCC	CC1
SMC16.T.ACCGTAAGTTTGGGCC	CC1
SMC16.T.ACCGTAATCCAAAGTC	CC2
SMC16.T.ACCTTTAAGATGGCGT	CC1
SMC16.T.ACCTTTAAGCGTGAGT	CC2
SMC16.T.ACCTTTACAATCGAAA	CC2
SMC16.T.ACGAGCCAGTATCGAA	CC2
SMC16.T.ACGAGCCCAGTGGGAT	CC2
SMC16.T.ACGAGCCGTAAACCTC	CC2
SMC16.T.ACGAGCCGTTTCCACC	CC2
SMC16.T.ACGAGCCTCGTTACGA	CC1
SMC16.T.ACGAGGAAGAATCTCC	CC2
SMC16.T.ACGAGGAAGGCTAGGT	CC1
SMC16.T.ACGAGGAAGGTTACCT	CC2
SMC16.T.ACGAGGAAGTGCGATG	CC2
SMC16.T.ACGAGGACACAGTCGC	CC1
SMC16.T.ACGAGGAGTATCTGCA	CC1
SMC16.T.ACGAGGAGTTGACGTT	CC1
SMC16.T.ACGATACAGACGCAAC	CC1
SMC16.T.ACGATACGTACAAGTA	CC2
SMC16.T.ACGATACGTATTCGTG	CC2
SMC16.T.ACGATACGTGTTCTTT	CC2
SMC16.T.ACGATGTAGCGATTCT	CC1
SMC16.T.ACGATGTAGTGGGATC	CC1
SMC16.T.ACGATGTCAAAGCGGT	CC2
SMC16.T.ACGATGTCAGCGATCC	CC1
SMC16.T.ACGATGTGTAAGTTCC	CC2
SMC16.T.ACGATGTGTATTAGCC	CC1
SMC16.T.ACGATGTGTCCATCCT	CC2
SMC16.T.ACGATGTGTCGAAAGC	CC2
SMC16.T.ACGATGTGTCGTTGTA	CC1
SMC16.T.ACGATGTGTGTATGGG	CC2
SMC16.T.ACGATGTTCATGCATG	CC1
SMC16.T.ACGATGTTCTCGTTTA	CC1
SMC16.T.ACGCAGCAGATGCGAC	CC1
SMC16.T.ACGCAGCAGCGCCTCA	CC1
SMC16.T.ACGCAGCCATGGGACA	CC2
SMC16.T.ACGCAGCCATTTGCTT	CC1
SMC16.T.ACGCAGCGTACTCAAC	CC1
SMC16.T.ACGCAGCGTCATCGGC	CC1
SMC16.T.ACGCAGCTCATACGGT	CC2
SMC16.T.ACGCCAGAGTACGCCC	CC2
SMC16.T.ACGCCAGAGTCAATAG	CC1
SMC16.T.ACGCCAGCACCCTATC	CC1

SMC16.T.ACGCCAGCACTACAGT	CC2
SMC16.T.ACGCCAGCATCACGAT	CC1
SMC16.T.ACGCCAGGTCTTGTCC	CC2
SMC16.T.ACGCCAGGTTCTGTTT	CC2
SMC16.T.ACGCCAGTCCATGCTC	CC2
SMC16.T.ACGCCGAAGACTAAGT	CC2
SMC16.T.ACGCCGAAGATGTAAC	CC1
SMC16.T.ACGCCGAAGCCTCGTG	CC1
SMC16.T.ACGCCGAAGTCTCCTC	CC1
SMC16.T.ACGCCGACATGAACCT	CC1
SMC16.T.ACGCCGAGTCGAGTTT	CC1
SMC16.T.ACGCCGATCCACTCCA	CC2
SMC16.T.ACGCCGATCTGAGTGT	CC2
SMC16.T.ACGGAGACACACATGT	CC1
SMC16.T.ACGGAGACAGCTGGCT	CC2
SMC16.T.ACGGAGACATGGGAAC	CC2
SMC16.T.ACGGAGAGTAAAGGAG	CC2
SMC16.T.ACGGAGAGTCTTGTCC	CC2
SMC16.T.ACGGAGAGTTGCGTTA	CC1
SMC16.T.ACGGAGATCACTTACT	CC1
SMC16.T.ACGGAGATCTACTTAC	CC1
SMC16.T.ACGGCCAAGGCGACAT	CC1
SMC16.T.ACGGCCAAGTGCTGCC	CC1
SMC16.T.ACGGCCACACTCTGTC	CC1
SMC16.T.ACGGCCACAGACGCCT	CC2
SMC16.T.ACGGCCAGTAAACACA	CC1
SMC16.T.ACGGCCATCAACGAAA	CC1
SMC16.T.ACGGGCTAGAGCTGGT	CC2
SMC16.T.ACGGGCTAGTGAACGC	CC1
SMC16.T.ACGGGCTCACATCCAA	CC2
SMC16.T.ACGGGCTCAGCCTTTC	CC1
SMC16.T.ACGGGCTCAGCGTCCA	CC2
SMC16.T.ACGGGCTCAGGTCCAC	CC1
SMC16.T.ACGGGCTCATGAGCGA	CC2
SMC16.T.ACGGGCTGTTCGTGAT	CC2
SMC16.T.ACGGGCTTCACCTTAT	CC1
SMC16.T.ACGGGCTTCGCGCCAA	CC2
SMC16.T.ACGGGCTTCTGTCTAT	CC1
SMC16.T.ACGGGCTTCTTGAGGT	CC2
SMC16.T.ACGGGTCAGAAGGTTT	CC2
SMC16.T.ACGGGTCAGATATGGT	CC1
SMC16.T.ACGGGTCAGTTGCAGG	CC1
SMC16.T.ACGGGTCTCGAGAACG	CC2
SMC16.T.ACGTCAAAGAGCTGGT	CC1
SMC16.T.ACGTCAAAGGTAGCTG	CC2
SMC16.T.ACGTCAACAACGATGG	CC2
SMC16.T.ACGTCAACAAGGTGTG	CC1
SMC16.T.ACGTCAACATCCAACA	CC1
SMC16.T.ACGTCAAGTTATCGGT	CC2
SMC16.T.ACGTCAATCCATGAAC	CC1
SMC16.T.ACGTCAATCCGCAGTG	CC2
SMC16.T.ACGTCAATCGATGAGG	CC2
SMC16.T.ACTATCTCAAGCCGCT	CC2
SMC16.T.ACTATCTCAGTCACTA	CC1
SMC16.T.ACTATCTTCAACTCTT	CC2
SMC16.T.ACTGAACAGAAGGACA	CC2
SMC16.T.ACTGAACAGGTAGCCA	CC1
SMC16.T.ACTGAACAGTGTACTC	CC2

SMC16.T.ACTGAACGTAGAGTGC	CC1
SMC16.T.ACTGAACGTCAGGACA	CC1
SMC16.T.ACTGAACGTGTAACGG	CC2
SMC16.T.ACTGAACGTTAAGACA	CC1
SMC16.T.ACTGAGTAGAAACCGC	CC1
SMC16.T.ACTGAGTAGCTGCCCA	CC1
SMC16.T.ACTGAGTAGTCATGCT	CC1
SMC16.T.ACTGAGTGTACCATCA	CC1
SMC16.T.ACTGAGTGTATGAAAC	CC2
SMC16.T.ACTGATGAGACCGGAT	CC2
SMC16.T.ACTGATGAGCTTTGGT	CC1
SMC16.T.ACTGATGGTGTGGTTT	CC2
SMC16.T.ACTGATGTCAAGAAGT	CC2
SMC16.T.ACTGATGTCGTCGTTC	CC2
SMC16.T.ACTGCTCAGACAGAGA	CC1
SMC16.T.ACTGCTCAGAGTCGGT	CC1
SMC16.T.ACTGCTCCAGTTCATG	CC1
SMC16.T.ACTGCTCGTCGACTGC	CC2
SMC16.T.ACTGCTCGTTCCAACA	CC2
SMC16.T.ACTGTCCCAGGCGATA	CC1
SMC16.T.ACTGTCCGTGTTAAGA	CC2
SMC16.T.ACTGTCCGTTCGGGCT	CC1
SMC16.T.ACTGTCCTCTTAGAGC	CC1
SMC16.T.ACTTACTAGACAAAGG	CC1
SMC16.T.ACTTACTGTAAACGCG	CC2
SMC16.T.ACTTACTTCTCACATT	CC2
SMC16.T.ACTTGTTCAAGCCGTC	CC2
SMC16.T.ACTTGTTGTTCTCATT	CC1
SMC16.T.ACTTGTTTCTCTGAGA	CC1
SMC16.T.ACTTTCAAGGCATTGG	CC2
SMC16.T.ACTTTCAAGTACTTGC	CC2
SMC16.T.ACTTTCACAAGTTGTC	CC1
SMC16.T.ACTTTCAGTCATGCCG	CC1
SMC16.T.ACTTTCAGTCGGCATC	CC1
SMC16.T.ACTTTCAGTTCTGAAC	CC2
SMC16.T.AGAATAGCACCGAATT	CC2
SMC16.T.AGAATAGCATACTCTT	CC2
SMC16.T.AGAATAGGTGTAAGTA	CC2
SMC16.T.AGAATAGTCAAAGTAG	CC2
SMC16.T.AGAATAGTCGAGCCCA	CC2
SMC16.T.AGAATAGTCTACTCAT	CC2
SMC16.T.AGAATAGTCTTGCCGT	CC2
SMC16.T.AGACGTTCAAGCGAGT	CC2
SMC16.T.AGACGTTCAGATGGGT	CC1
SMC16.T.AGACGTTGTAGGAGTC	CC2
SMC16.T.AGACGTTGTTGTCTTT	CC1
SMC16.T.AGAGCGAAGGTGGGTT	CC2
SMC16.T.AGAGCGAAGTGAATTG	CC2
SMC16.T.AGAGCGACACTGTGTA	CC1
SMC16.T.AGAGCGACAGATGGCA	CC1
SMC16.T.AGAGCGACATAGGATA	CC1
SMC16.T.AGAGCGACATAGTAAG	CC2
SMC16.T.AGAGCGAGTCCGACGT	CC2
SMC16.T.AGAGCGATCACAACGT	CC2
SMC16.T.AGAGCGATCTGTTGAG	CC1
SMC16.T.AGAGCTTAGGAGCGAG	CC2
SMC16.T.AGAGCTTAGTAAGTAC	CC1
SMC16.T.AGAGCTTCAAACAACA	CC2

SMC16.T.AGAGCTTCAATGACCT	CC2
SMC16.T.AGAGCTTCACCAGGTC	CC2
SMC16.T.AGAGCTTCACGTCAGC	CC2
SMC16.T.AGAGCTTGTTGCGTTA	CC2
SMC16.T.AGAGCTTTCGATAGAA	CC1
SMC16.T.AGAGTGGAGAAACGAG	CC1
SMC16.T.AGAGTGGAGACTAAGT	CC2
SMC16.T.AGAGTGGAGAGTAATC	CC1
SMC16.T.AGAGTGGAGCGACGTA	CC2
SMC16.T.AGAGTGGGTGCGAAAC	CC1
SMC16.T.AGAGTGGGTGTGGTTT	CC2
SMC16.T.AGAGTGGGTTCGAATC	CC1
SMC16.T.AGATCTGAGAACTGTA	CC2
SMC16.T.AGATCTGGTTATCGGT	CC2
SMC16.T.AGATCTGTCCAAGTAC	CC2
SMC16.T.AGATCTGTCGCCATAA	CC1
SMC16.T.AGATTGCCATTATCTC	CC1
SMC16.T.AGATTGCGTTGGTAAA	CC2
SMC16.T.AGATTGCTCCCTCAGT	CC2
SMC16.T.AGCAGCCAGAAGGGTA	CC2
SMC16.T.AGCAGCCGTAGTACCT	CC2
SMC16.T.AGCAGCCGTCAGAATA	CC2
SMC16.T.AGCAGCCGTCTTGCGG	CC1
SMC16.T.AGCATACCAACTGGCC	CC1
SMC16.T.AGCATACTCCTACAGA	CC2
SMC16.T.AGCATACTCTGCGGCA	CC1
SMC16.T.AGCCTAATCCTATTCA	CC2
SMC16.T.AGCCTAATCGAGCCCA	CC2
SMC16.T.AGCGGTCAGAGGTAGA	CC2
SMC16.T.AGCGGTCAGAGGTTGC	CC1
SMC16.T.AGCGGTCAGGTGCACA	CC2
SMC16.T.AGCGGTCCAAGTCTGT	CC2
SMC16.T.AGCGGTCGTCGTGGCT	CC1
SMC16.T.AGCGGTCGTGGTACAG	CC2
SMC16.T.AGCGGTCTCAGTTAGC	CC1
SMC16.T.AGCGTATAGTGCGATG	CC1
SMC16.T.AGCGTATGTACCAGTT	CC1
SMC16.T.AGCGTATGTCCAGTTA	CC1
SMC16.T.AGCGTATGTTCTCATT	CC1
SMC16.T.AGCGTCGAGGGCACTA	CC1
SMC16.T.AGCGTCGGTGTCTGAT	CC1
SMC16.T.AGCGTCGTCCATGAGT	CC1
SMC16.T.AGCGTCGTCTGCGGCA	CC1
SMC16.T.AGCTCCTAGCGTTGCC	CC2
SMC16.T.AGCTCCTGTCGCGAAA	CC1
SMC16.T.AGCTCCTTCGGCATCG	CC2
SMC16.T.AGCTCTCAGTTCGCAT	CC1
SMC16.T.AGCTCTCCAGCTGGCT	CC2
SMC16.T.AGCTCTCTCAAAGTAG	CC2
SMC16.T.AGCTCTCTCACGGTTA	CC2
SMC16.T.AGCTCTCTCATGTAGC	CC2
SMC16.T.AGCTTGAAGGCTAGAC	CC1
SMC16.T.AGCTTGACACCTCGGA	CC1
SMC16.T.AGCTTGAGTGACTCAT	CC2
SMC16.T.AGCTTGAGTGTTAAGA	CC2
SMC16.T.AGCTTGATCTTCTGGC	CC2
SMC16.T.AGGCCACAGGCGATAC	CC2
SMC16.T.AGGCCACAGTAGCGGT	CC1

SMC16.T.AGGCCACCACAGGAGT	CC1
SMC16.T.AGGCCACCAGGGTACA	CC1
SMC16.T.AGGCCACCATCCTTGC	CC2
SMC16.T.AGGCCACGTAAACCTC	CC2
SMC16.T.AGGCCACGTGAAAGAG	CC2
SMC16.T.AGGCCACGTGTAAGTA	CC1
SMC16.T.AGGCCACTCCTGTAGA	CC1
SMC16.T.AGGCCGTCAGGCGATA	CC1
SMC16.T.AGGCCGTTCATCTGTT	CC2
SMC16.T.AGGCCGTTCTGTTGAG	CC1
SMC16.T.AGGGAGTCACGAAATA	CC2
SMC16.T.AGGGAGTCATCCAACA	CC1
SMC16.T.AGGGAGTGTAACGCGA	CC2
SMC16.T.AGGGATGGTCAAAGAT	CC1
SMC16.T.AGGGATGGTCGCATCG	CC1
SMC16.T.AGGGATGGTGCAGACA	CC1
SMC16.T.AGGGATGTCAAGGTAA	CC2
SMC16.T.AGGGTGAAGAATTCCC	CC2
SMC16.T.AGGGTGACAGCGTCCA	CC2
SMC16.T.AGGGTGACATGTCGAT	CC1
SMC16.T.AGGGTGAGTCAGATAA	CC1
SMC16.T.AGGGTGATCCTAGAAC	CC2
SMC16.T.AGGTCATAGATGTGGC	CC1
SMC16.T.AGGTCATAGGATATAC	CC2
SMC16.T.AGGTCATAGGCTCATT	CC2
SMC16.T.AGGTCATAGGGTGTTG	CC2
SMC16.T.AGGTCATCACACTGCG	CC2
SMC16.T.AGGTCATCACAGCCCA	CC1
SMC16.T.AGGTCATCATGCCCGA	CC1
SMC16.T.AGGTCATGTATTACCG	CC1
SMC16.T.AGGTCATTCATGCTCC	CC1
SMC16.T.AGGTCATTCGCCCTTA	CC2
SMC16.T.AGGTCATTCTTTACAC	CC2
SMC16.T.AGGTCCGAGAGCCTAG	CC2
SMC16.T.AGGTCCGAGCGATCCC	CC2
SMC16.T.AGGTCCGAGGACGAAA	CC2
SMC16.T.AGGTCCGAGTAGATGT	CC1
SMC16.T.AGGTCCGCAGCATACT	CC1
SMC16.T.AGGTCCGGTCACTGGC	CC2
SMC16.T.AGGTCCGGTGCACTTA	CC1
SMC16.T.AGGTCCGGTTGTTTGG	CC2
SMC16.T.AGGTCCGTCTTGAGGT	CC1
SMC16.T.AGTAGTCAGAACTCGG	CC2
SMC16.T.AGTAGTCGTAGAGGAA	CC2
SMC16.T.AGTAGTCGTCAAGCGA	CC1
SMC16.T.AGTAGTCGTCTCTTTA	CC1
SMC16.T.AGTCTTTAGAGCTATA	CC2
SMC16.T.AGTCTTTAGGGTTCCC	CC1
SMC16.T.AGTCTTTCAGACGTAG	CC2
SMC16.T.AGTCTTTTCATGCTCC	CC1
SMC16.T.AGTGAGGAGGTCATCT	CC2
SMC16.T.AGTGAGGCAAAGCGGT	CC2
SMC16.T.AGTGAGGTCCTCAACC	CC1
SMC16.T.AGTGGGAAGAGCCCAA	CC1
SMC16.T.AGTGGGAAGTGGAGTC	CC1
SMC16.T.AGTGGGACAATGGACG	CC1
SMC16.T.AGTGGGACATTAGGCT	CC2
SMC16.T.AGTGGGAGTAGCTGCC	CC2

SMC16.T.AGTGGGAGTGCAGTAG	CC1
SMC16.T.AGTGGGATCACGAAGG	CC1
SMC16.T.AGTGGGATCTGAAAGA	CC1
SMC16.T.AGTGTCAAGGCTCAGA	CC1
SMC16.T.AGTGTCAGTCTAGCGC	CC2
SMC16.T.AGTGTCAGTCTGCCAG	CC2
SMC16.T.AGTGTCATCGGAAATA	CC2
SMC16.T.AGTGTCATCTTCATGT	CC2
SMC16.T.AGTTGGTCACAGCCCA	CC1
SMC16.T.AGTTGGTCACAGCGTC	CC1
SMC16.T.ATAACGCAGCTGGAAC	CC2
SMC16.T.ATAACGCAGGACATTA	CC1
SMC16.T.ATAACGCAGGAGCGTT	CC1
SMC16.T.ATAACGCGTGTTCGAT	CC1
SMC16.T.ATAACGCGTTCAGGCC	CC2
SMC16.T.ATAACGCTCTACTCAT	CC1
SMC16.T.ATAAGAGAGAGGTACC	CC2
SMC16.T.ATAAGAGAGGCATGTG	CC2
SMC16.T.ATAAGAGCACCCATTC	CC2
SMC16.T.ATAAGAGCACGTCTCT	CC2
SMC16.T.ATAAGAGGTCCAAGTT	CC1
SMC16.T.ATAGACCAGATGGGTC	CC2
SMC16.T.ATAGACCAGCCGATTT	CC1
SMC16.T.ATAGACCAGTAGGTGC	CC1
SMC16.T.ATAGACCCAGGTCGTC	CC2
SMC16.T.ATCACGACATTGGGCC	CC2
SMC16.T.ATCACGAGTTATCGGT	CC1
SMC16.T.ATCACGATCAACACGT	CC2
SMC16.T.ATCACGATCAGCGATT	CC2
SMC16.T.ATCACGATCCAGTATG	CC2
SMC16.T.ATCACGATCTTACCGC	CC2
SMC16.T.ATCATCTAGCCAACAG	CC2
SMC16.T.ATCATCTAGTGCGATG	CC2
SMC16.T.ATCATCTGTATAGTAG	CC2
SMC16.T.ATCATCTTCGTAGGAG	CC2
SMC16.T.ATCATGGGTATAGTAG	CC2
SMC16.T.ATCATGGTCAGCTCGG	CC1
SMC16.T.ATCCACCAGACAAGCC	CC2
SMC16.T.ATCCACCAGAGGACGG	CC1
SMC16.T.ATCCACCAGATCCTGT	CC1
SMC16.T.ATCCACCAGGGCTTCC	CC2
SMC16.T.ATCCACCCACAGATTC	CC1
SMC16.T.ATCCACCGTGCCTGCA	CC1
SMC16.T.ATCCACCGTGTATGGG	CC2
SMC16.T.ATCCGAACACCATCCT	CC2
SMC16.T.ATCCGAACAGGCTCAC	CC1
SMC16.T.ATCCGAAGTCTTCAAG	CC1
SMC16.T.ATCCGAATCACAGGCC	CC1
SMC16.T.ATCCGAATCACCAGGC	CC1
SMC16.T.ATCCGAATCCTCCTAG	CC1
SMC16.T.ATCCGAATCGTCCGTT	CC2
SMC16.T.ATCGAGTAGATCGATA	CC2
SMC16.T.ATCGAGTAGTGTACCT	CC1
SMC16.T.ATCGAGTCAGCTCCGA	CC1
SMC16.T.ATCGAGTCAGTAGAGC	CC2
SMC16.T.ATCGAGTGTTCGGCAC	CC2
SMC16.T.ATCTACTCACAAGACG	CC1
SMC16.T.ATCTACTCACCAGATT	CC2

SMC16.T.ATCTACTGTTCGTTGA	CC2
SMC16.T.ATCTGCCAGTTCCACA	CC1
SMC16.T.ATCTGCCCAAGTTAAG	CC1
SMC16.T.ATCTGCCCAGCGTCCA	CC1
SMC16.T.ATCTGCCCATATACGC	CC2
SMC16.T.ATCTGCCGTGTGCCTG	CC1
SMC16.T.ATCTGCCGTTCACGGC	CC1
SMC16.T.ATCTGCCTCAGTTTGG	CC2
SMC16.T.ATCTGCCTCCAATGGT	CC2
SMC16.T.ATCTGCCTCTACGAGT	CC2
SMC16.T.ATCTGCCTCTTTAGGG	CC2
SMC16.T.ATGAGGGTCGAACGGA	CC1
SMC16.T.ATGAGGGTCTCCCTGA	CC2
SMC16.T.ATGCGATAGTGGACGT	CC1
SMC16.T.ATGCGATCAATCAGAA	CC2
SMC16.T.ATGCGATCATGCTGGC	CC2
SMC16.T.ATGCGATGTACATCCA	CC1
SMC16.T.ATGCGATGTTAGGGTG	CC1
SMC16.T.ATGCGATTCTGTGCAA	CC1
SMC16.T.ATGCGATTCTTTCCTC	CC1
SMC16.T.ATGGGAGAGTGTTAGA	CC1
SMC16.T.ATGGGAGGTAGAGTGC	CC2
SMC16.T.ATGGGAGGTCTCAACA	CC2
SMC16.T.ATGGGAGTCCGCGTTT	CC2
SMC16.T.ATGGGAGTCTCTTGAT	CC1
SMC16.T.ATGTGTGAGTCTCCTC	CC1
SMC16.T.ATGTGTGCAGATTGCT	CC1
SMC16.T.ATGTGTGTCGGACAAG	CC1
SMC16.T.ATGTGTGTCTCTGCTG	CC1
SMC16.T.ATTACTCAGCGTGAAC	CC2
SMC16.T.ATTACTCCAAACGCGA	CC1
SMC16.T.ATTACTCCAAAGAATC	CC1
SMC16.T.ATTACTCCAAGCCTAT	CC2
SMC16.T.ATTACTCCAGATGAGC	CC1
SMC16.T.ATTACTCCAGTACACT	CC2
SMC16.T.ATTACTCCATGGTCAT	CC2
SMC16.T.ATTACTCGTGCAGTAG	CC2
SMC16.T.ATTACTCGTGCTGTAT	CC2
SMC16.T.ATTACTCTCAACACAC	CC2
SMC16.T.ATTACTCTCACGCATA	CC2
SMC16.T.ATTACTCTCACTTACT	CC1
SMC16.T.ATTATCCAGGATGGTC	CC2
SMC16.T.ATTATCCGTAGAAGGA	CC1
SMC16.T.ATTATCCGTAGCGTCC	CC1
SMC16.T.ATTATCCGTTGTGGAG	CC2
SMC16.T.ATTATCCTCAGTACGT	CC1
SMC16.T.ATTATCCTCGTCTGAA	CC2
SMC16.T.ATTCTACAGCCCAGCT	CC1
SMC16.T.ATTCTACCACTTCGAA	CC2
SMC16.T.ATTCTACGTTAGAACA	CC2
SMC16.T.ATTCTACTCACATGCA	CC1
SMC16.T.ATTCTACTCAGAGCTT	CC2
SMC16.T.ATTCTACTCCAAGTAC	CC2
SMC16.T.ATTGGACCACATTTCT	CC2
SMC16.T.ATTGGACGTGATGTGG	CC1
SMC16.T.ATTGGACGTTCACCTC	CC1
SMC16.T.ATTGGACTCACCCTCA	CC2
SMC16.T.ATTGGTGAGCACAGGT	CC2

SMC16.T.ATTGGTGAGGCACATG	CC2
SMC16.T.ATTGGTGCAAGCCTAT	CC2
SMC16.T.ATTGGTGGTAAGTGGC	CC2
SMC16.T.ATTGGTGGTTGGTAAA	CC2
SMC16.T.ATTGGTGGTTTGTGTG	CC2
SMC16.T.ATTGGTGTCAGTTGAC	CC2
SMC16.T.ATTTCTGAGGATCGCA	CC1
SMC16.T.ATTTCTGAGGCCATAG	CC1
SMC16.T.ATTTCTGCAGTCAGAG	CC2
SMC16.T.ATTTCTGCAGTTAACC	CC1
SMC16.T.ATTTCTGGTCAAACTC	CC2
SMC16.T.ATTTCTGGTCTAGTGT	CC1
SMC16.T.ATTTCTGGTGCGAAAC	CC1
SMC16.T.ATTTCTGTCAATCACG	CC1
SMC16.T.CAACCAAAGAAGGCCT	CC1
SMC16.T.CAACCAAAGTGGGATC	CC1
SMC16.T.CAACCAAGTGGCTCCA	CC1
SMC16.T.CAACCTCCACCATCCT	CC2
SMC16.T.CAACCTCCAGCTGCAC	CC2
SMC16.T.CAACCTCGTCACACGC	CC2
SMC16.T.CAACCTCGTTAAGAAC	CC1
SMC16.T.CAACCTCTCTGCAAGT	CC2
SMC16.T.CAACTAGCACCGCTAG	CC1
SMC16.T.CAACTAGCACTTACGA	CC1
SMC16.T.CAACTAGGTAGTAGTA	CC2
SMC16.T.CAACTAGGTGGCTCCA	CC2
SMC16.T.CAACTAGTCCTTGACC	CC1
SMC16.T.CAAGAAAAGAGGTACC	CC2
SMC16.T.CAAGAAAAGAGTAATC	CC1
SMC16.T.CAAGAAACACAACGTT	CC1
SMC16.T.CAAGAAAGTCAGAATA	CC2
SMC16.T.CAAGAAAGTTGTACAC	CC1
SMC16.T.CAAGAAATCGTCTGCT	CC2
SMC16.T.CAAGATCCAAGGCTCC	CC2
SMC16.T.CAAGATCCACAAGTAA	CC2
SMC16.T.CAAGATCCACGAAACG	CC2
SMC16.T.CAAGATCGTAACGACG	CC1
SMC16.T.CAAGATCGTGTATGGG	CC1
SMC16.T.CAAGATCGTTCAGGCC	CC2
SMC16.T.CAAGGCCAGATGGCGT	CC2
SMC16.T.CAAGGCCCAAACCCAT	CC1
SMC16.T.CAAGGCCCACGCATCG	CC2
SMC16.T.CAAGGCCCACTAAGTC	CC2
SMC16.T.CAAGGCCGTATCGCAT	CC2
SMC16.T.CAAGGCCGTTCCACTC	CC2
SMC16.T.CAAGGCCTCCAGAGGA	CC2
SMC16.T.CAAGTTGAGACACTAA	CC1
SMC16.T.CAAGTTGAGAGCCCAA	CC1
SMC16.T.CAAGTTGAGTGACTCT	CC1
SMC16.T.CAAGTTGCAAAGGAAG	CC1
SMC16.T.CAAGTTGCATCCTAGA	CC1
SMC16.T.CAAGTTGGTTCGGCAC	CC2
SMC16.T.CAAGTTGTCCGCAAGC	CC1
SMC16.T.CAAGTTGTCTGAGTGT	CC1
SMC16.T.CAAGTTGTCTGCGACG	CC2
SMC16.T.CAAGTTGTCTTCATGT	CC2
SMC16.T.CACAAACAGGTGGGTT	CC1
SMC16.T.CACAAACAGTAGGCCA	CC1

SMC16.T.CACAAACCAATAAGCA	CC2
SMC16.T.CACAAACCAGGCAGTA	CC2
SMC16.T.CACAAACCATTTCACT	CC1
SMC16.T.CACAAACGTAATCACC	CC1
SMC16.T.CACAAACTCCGTCATC	CC2
SMC16.T.CACACAAAGACAGAGA	CC1
SMC16.T.CACACAACACGCATCG	CC2
SMC16.T.CACACAACACTTAACG	CC2
SMC16.T.CACACAACATCACAAC	CC1
SMC16.T.CACACAAGTAATCGTC	CC1
SMC16.T.CACACAATCGTCTGAA	CC1
SMC16.T.CACACCTAGTCCGTAT	CC1
SMC16.T.CACACCTCAGCAGTTT	CC1
SMC16.T.CACACCTCATGCGCAC	CC1
SMC16.T.CACACCTCATGGTCAT	CC2
SMC16.T.CACACCTGTTGTGGAG	CC1
SMC16.T.CACACTCGTTGTGGCC	CC1
SMC16.T.CACACTCTCCCGGATG	CC2
SMC16.T.CACACTCTCGAACTGT	CC2
SMC16.T.CACACTCTCTAAGCCA	CC1
SMC16.T.CACAGGCAGAAGGTGA	CC2
SMC16.T.CACAGGCAGCACCGCT	CC1
SMC16.T.CACAGGCAGGTGCTAG	CC1
SMC16.T.CACAGGCCACCATCCT	CC1
SMC16.T.CACAGGCCATGACATC	CC1
SMC16.T.CACAGGCCATGCCACG	CC2
SMC16.T.CACAGGCGTTGAACTC	CC2
SMC16.T.CACAGGCGTTTAGGAA	CC2
SMC16.T.CACAGGCTCACCGTAA	CC2
SMC16.T.CACAGGCTCACTCCTG	CC1
SMC16.T.CACAGTAAGCCGCCTA	CC2
SMC16.T.CACAGTAAGTGAAGTT	CC1
SMC16.T.CACAGTACACAACGTT	CC2
SMC16.T.CACAGTACACATCCGG	CC2
SMC16.T.CACAGTAGTAATCACC	CC2
SMC16.T.CACAGTAGTAGAGCTG	CC2
SMC16.T.CACAGTAGTCTAGTGT	CC2
SMC16.T.CACAGTATCCACGACG	CC2
SMC16.T.CACAGTATCCCTCAGT	CC2
SMC16.T.CACATAGAGGAGTTGC	CC2
SMC16.T.CACATAGAGGATTCGG	CC1
SMC16.T.CACATAGAGGGTCGAT	CC1
SMC16.T.CACATAGAGGTAAACT	CC2
SMC16.T.CACATAGCAATACGCT	CC2
SMC16.T.CACATAGGTATATGGA	CC1
SMC16.T.CACATAGGTCTAAAGA	CC2
SMC16.T.CACATAGGTGTTTGTG	CC1
SMC16.T.CACATAGGTTTGACTG	CC2
SMC16.T.CACATTTAGGGATACC	CC2
SMC16.T.CACATTTCACGGATAG	CC2
SMC16.T.CACATTTCACGGTAGA	CC1
SMC16.T.CACATTTGTTACTGAC	CC2
SMC16.T.CACATTTTCAGGTAAA	CC2
SMC16.T.CACCACTAGAGGGCTT	CC1
SMC16.T.CACCACTAGATGGCGT	CC1
SMC16.T.CACCACTAGGCATGGT	CC1
SMC16.T.CACCACTCAGGCGATA	CC2
SMC16.T.CACCACTGTATAGTAG	CC1

SMC16.T.CACCAGGAGCTACCTA	CC1
SMC16.T.CACCAGGGTGAGCGAT	CC1
SMC16.T.CACCTTGAGAACTCGG	CC2
SMC16.T.CACCTTGAGCAAATCA	CC2
SMC16.T.CACCTTGCAAGTCTGT	CC1
SMC16.T.CACCTTGCACGCCAGT	CC2
SMC16.T.CACCTTGCAGGAACGT	CC1
SMC16.T.CACCTTGCATGGGACA	CC2
SMC16.T.CACCTTGTCAAACCGT	CC2
SMC16.T.CACTCCAAGATAGGAG	CC2
SMC16.T.CACTCCAAGTTGAGAT	CC2
SMC16.T.CACTCCACAAGACGTG	CC1
SMC16.T.CACTCCACAAGAGTCG	CC1
SMC16.T.CACTCCATCTGGAGCC	CC1
SMC16.T.CAGAATCGTCCTAGCG	CC2
SMC16.T.CAGAATCGTTTGTGTG	CC1
SMC16.T.CAGAATCTCACATACG	CC2
SMC16.T.CAGAATCTCATGCAAC	CC1
SMC16.T.CAGAGAGAGTGAAGAG	CC1
SMC16.T.CAGAGAGCAATGGACG	CC2
SMC16.T.CAGAGAGCATATGGTC	CC1
SMC16.T.CAGAGAGGTAGATTAG	CC2
SMC16.T.CAGAGAGGTGGTTTCA	CC2
SMC16.T.CAGAGAGGTTTGACTG	CC2
SMC16.T.CAGAGAGTCCATTCTA	CC2
SMC16.T.CAGAGAGTCTTGCAAG	CC1
SMC16.T.CAGATCAAGATATACG	CC1
SMC16.T.CAGCAGCAGGGTGTTG	CC2
SMC16.T.CAGCAGCCAAGCGTAG	CC1
SMC16.T.CAGCAGCCATCATCCC	CC2
SMC16.T.CAGCAGCGTTGCGCAC	CC1
SMC16.T.CAGCATAGTACAAGTA	CC1
SMC16.T.CAGCATAGTGACGGTA	CC2
SMC16.T.CAGCATATCGTTGACA	CC1
SMC16.T.CAGCCGAAGGAGCGTT	CC1
SMC16.T.CAGCCGACAGCGTTCG	CC1
SMC16.T.CAGCCGAGTCCTAGCG	CC2
SMC16.T.CAGCCGAGTGCGATAG	CC2
SMC16.T.CAGCCGATCACCGTAA	CC1
SMC16.T.CAGCCGATCATATCGG	CC2
SMC16.T.CAGCGACCATCACGAT	CC1
SMC16.T.CAGCGACGTACGAAAT	CC1
SMC16.T.CAGCGACGTCAGAGGT	CC2
SMC16.T.CAGCGACTCCCTTGTG	CC2
SMC16.T.CAGCTAACAATAAGCA	CC1
SMC16.T.CAGCTAAGTCACAAGG	CC2
SMC16.T.CAGCTAAGTTATCCGA	CC2
SMC16.T.CAGCTGGAGCGAAGGG	CC1
SMC16.T.CAGCTGGAGTCGCCGT	CC2
SMC16.T.CAGCTGGAGTTCGCAT	CC2
SMC16.T.CAGCTGGGTAAGGGCT	CC1
SMC16.T.CAGCTGGGTGATGTCT	CC1
SMC16.T.CAGCTGGTCTAACTTC	CC2
SMC16.T.CAGGTGCAGCGTTGCC	CC2
SMC16.T.CAGGTGCCAAGCGATG	CC2
SMC16.T.CAGGTGCCAGTAAGAT	CC2
SMC16.T.CAGGTGCCATCTACGA	CC2
SMC16.T.CAGGTGCGTGACGCCT	CC2

SMC16.T.CAGGTGCTCACTTACT	CC2
SMC16.T.CAGGTGCTCTGCTTGC	CC2
SMC16.T.CAGTAACTCATTGCCC	CC1
SMC16.T.CAGTAACTCCACTCCA	CC1
SMC16.T.CAGTAACTCCGTTGCT	CC1
SMC16.T.CAGTCCTCAGCTTAAC	CC2
SMC16.T.CAGTCCTCATAAAGGT	CC2
SMC16.T.CAGTCCTGTTGGTGGA	CC1
SMC16.T.CAGTCCTTCGCTTGTC	CC2
SMC16.T.CAGTCCTTCGTCTGCT	CC2
SMC16.T.CATATGGAGGTGACCA	CC2
SMC16.T.CATATGGAGTGTTAGA	CC2
SMC16.T.CATATGGTCTGAGGGA	CC2
SMC16.T.CATATTCAGGGTGTGT	CC2
SMC16.T.CATATTCCATGCCTAA	CC1
SMC16.T.CATATTCGTACTTCTT	CC2
SMC16.T.CATATTCTCCAGAGGA	CC2
SMC16.T.CATCAAGAGCACGCCT	CC2
SMC16.T.CATCAAGAGCCTCGTG	CC2
SMC16.T.CATCAAGCACCCTATC	CC1
SMC16.T.CATCAAGTCCTTAATC	CC2
SMC16.T.CATCAGAAGAGTACAT	CC2
SMC16.T.CATCAGAAGGTTCCTA	CC2
SMC16.T.CATCAGAAGTAGTGCG	CC1
SMC16.T.CATCAGACAGGATTGG	CC1
SMC16.T.CATCAGATCGGAGCAA	CC2
SMC16.T.CATCAGATCGGGAGTA	CC1
SMC16.T.CATCAGATCGTCACGG	CC2
SMC16.T.CATCAGATCTCACATT	CC1
SMC16.T.CATCAGATCTGAGTGT	CC2
SMC16.T.CATCCACGTCGGCTCA	CC2
SMC16.T.CATCCACGTTATCGGT	CC1
SMC16.T.CATCCACTCCGCAGTG	CC2
SMC16.T.CATCGAAAGGATGGAA	CC2
SMC16.T.CATCGAAAGTGTACGG	CC1
SMC16.T.CATCGAACAAAGCGGT	CC1
SMC16.T.CATCGGGCACAGAGGT	CC1
SMC16.T.CATCGGGGTAAGCACG	CC2
SMC16.T.CATCGGGGTAGAGTGC	CC1
SMC16.T.CATCGGGGTAGGGACT	CC2
SMC16.T.CATCGGGGTATAGGGC	CC2
SMC16.T.CATCGGGGTATGCTTG	CC1
SMC16.T.CATCGGGGTGTGACCC	CC1
SMC16.T.CATCGGGTCATCGCTC	CC1
SMC16.T.CATGACAAGAGCCTAG	CC2
SMC16.T.CATGACAAGCTTTGGT	CC2
SMC16.T.CATGACAAGTACATGA	CC2
SMC16.T.CATGACAGTGAGCGAT	CC2
SMC16.T.CATGACAGTTATCACG	CC2
SMC16.T.CATGACAGTTCGTGAT	CC2
SMC16.T.CATGACAGTTTGTGTG	CC1
SMC16.T.CATGACATCTGGTATG	CC2
SMC16.T.CATGCCTAGCTGCAAG	CC2
SMC16.T.CATGCCTAGGCAATTA	CC1
SMC16.T.CATGCCTAGTACGACG	CC1
SMC16.T.CATGCCTAGTGGTAGC	CC2
SMC16.T.CATGCCTAGTTGTAGA	CC2
SMC16.T.CATGCCTGTACGCACC	CC1

SMC16.T.CATGGCGAGGACTGGT	CC1
SMC16.T.CATGGCGAGGATGCGT	CC1
SMC16.T.CATGGCGAGGATGTAT	CC1
SMC16.T.CATGGCGAGGCAAAGA	CC2
SMC16.T.CATGGCGAGGCTAGCA	CC2
SMC16.T.CATGGCGCAACGATGG	CC2
SMC16.T.CATGGCGTCAAGAAGT	CC2
SMC16.T.CATGGCGTCGTAGGTT	CC1
SMC16.T.CATGGCGTCGTCCAGG	CC1
SMC16.T.CATTATCAGTTGTCGT	CC2
SMC16.T.CATTATCCAATAGCGG	CC1
SMC16.T.CATTATCCATGCCACG	CC2
SMC16.T.CATTATCGTAGCTGCC	CC1
SMC16.T.CATTATCGTTTCCACC	CC2
SMC16.T.CATTATCTCTTGAGAC	CC1
SMC16.T.CATTCGCGTAGGGTAC	CC2
SMC16.T.CATTCGCTCAGCAACT	CC2
SMC16.T.CATTCGCTCTGGCGTG	CC2
SMC16.T.CCAATCCAGGCTAGAC	CC1
SMC16.T.CCAATCCAGTCAAGGC	CC2
SMC16.T.CCAATCCGTCCTAGCG	CC2
SMC16.T.CCAATCCGTTCAGACT	CC1
SMC16.T.CCAATCCTCAATCACG	CC1
SMC16.T.CCAATCCTCATACGGT	CC1
SMC16.T.CCAATCCTCTGACCTC	CC1
SMC16.T.CCACCTAAGAGATGAG	CC2
SMC16.T.CCACCTAAGTACCGGA	CC1
SMC16.T.CCACCTAGTCAGTGGA	CC2
SMC16.T.CCACCTAGTCCCTTGT	CC1
SMC16.T.CCACCTATCATGGTCA	CC2
SMC16.T.CCACCTATCCTGTACC	CC1
SMC16.T.CCACCTATCCTTCAAT	CC2
SMC16.T.CCACGGAAGAGAGCTC	CC2
SMC16.T.CCACGGAAGGCATGGT	CC2
SMC16.T.CCACGGACAAGGTGTG	CC1
SMC16.T.CCACGGAGTTAGATGA	CC1
SMC16.T.CCACGGATCATCGATG	CC1
SMC16.T.CCACGGATCATCGCTC	CC2
SMC16.T.CCACTACCACGTTGGC	CC2
SMC16.T.CCACTACCAGACAGGT	CC2
SMC16.T.CCAGCGAGTACTCGCG	CC1
SMC16.T.CCAGCGAGTGTCTGAT	CC1
SMC16.T.CCAGCGATCGACAGCC	CC2
SMC16.T.CCATGTCCAAGCGCTC	CC2
SMC16.T.CCATGTCGTCTAGTCA	CC2
SMC16.T.CCATGTCTCCCTGACT	CC1
SMC16.T.CCATGTCTCGGAAATA	CC2
SMC16.T.CCATTCGAGCGACGTA	CC1
SMC16.T.CCATTCGCATTGGTAC	CC2
SMC16.T.CCATTCGGTTAGAACA	CC1
SMC16.T.CCATTCGGTTCCACAA	CC2
SMC16.T.CCATTCGGTTCTGGTA	CC1
SMC16.T.CCATTCGTCTGGTGTA	CC2
SMC16.T.CCCAATCAGTGTTTGC	CC1
SMC16.T.CCCAATCGTAATTGGA	CC2
SMC16.T.CCCAATCGTCCAGTTA	CC2
SMC16.T.CCCAATCGTCTAGGTT	CC2
SMC16.T.CCCAATCGTGTGGTTT	CC2

SMC16.T.CCCAATCTCATCGCTC	CC1
SMC16.T.CCCAGTTAGACTTTCG	CC1
SMC16.T.CCCAGTTAGATATACG	CC2
SMC16.T.CCCAGTTAGTGAAGTT	CC2
SMC16.T.CCCAGTTCAATGAATG	CC1
SMC16.T.CCCAGTTTCGCTTAGA	CC1
SMC16.T.CCCATACAGCGATGAC	CC2
SMC16.T.CCCATACAGGATTCGG	CC1
SMC16.T.CCCATACCAAATCCGT	CC2
SMC16.T.CCCTCCTAGCGATGAC	CC2
SMC16.T.CCCTCCTAGGGATGGG	CC1
SMC16.T.CCCTCCTCAAGGGTCA	CC1
SMC16.T.CCCTCCTCAGCTCGCA	CC1
SMC16.T.CCCTCCTTCTGCCAGG	CC2
SMC16.T.CCCTCCTTCTTCGAGA	CC2
SMC16.T.CCGGGATAGAGCTGGT	CC1
SMC16.T.CCGGGATCAGATGGGT	CC1
SMC16.T.CCGGGATCATACGCCG	CC1
SMC16.T.CCGGGATGTGGTTTCA	CC1
SMC16.T.CCGGGATTCAATACCG	CC2
SMC16.T.CCGGTAGAGCGAAGGG	CC1
SMC16.T.CCGGTAGAGTGTCCCG	CC2
SMC16.T.CCGGTAGGTGGTCCGT	CC2
SMC16.T.CCGGTAGTCACGATGT	CC2
SMC16.T.CCGGTAGTCAGTTGAC	CC2
SMC16.T.CCGGTAGTCTGTCCGT	CC1
SMC16.T.CCGTACTCAATTGCTG	CC1
SMC16.T.CCGTACTCACCAGTTA	CC1
SMC16.T.CCGTGGAGTACCCAAT	CC1
SMC16.T.CCGTGGATCAAGAAGT	CC2
SMC16.T.CCGTTCAAGAGAGCTC	CC2
SMC16.T.CCGTTCAAGGAGTACC	CC2
SMC16.T.CCGTTCACAATAACGA	CC2
SMC16.T.CCGTTCAGTCTCTCGT	CC2
SMC16.T.CCGTTCAGTGGTCCGT	CC2
SMC16.T.CCGTTCATCTCGAGTA	CC2
SMC16.T.CCTAAAGCATCGGGTC	CC2
SMC16.T.CCTAAAGGTATTCGTG	CC2
SMC16.T.CCTAAAGGTCAAAGAT	CC2
SMC16.T.CCTAAAGGTCAGAGGT	CC1
SMC16.T.CCTAAAGGTTGGTTTG	CC1
SMC16.T.CCTAAAGTCTTACCGC	CC1
SMC16.T.CCTACACAGCTGCAAG	CC1
SMC16.T.CCTACACAGGATTCGG	CC2
SMC16.T.CCTACACCAATCTGCA	CC2
SMC16.T.CCTACACGTTCCGGCA	CC2
SMC16.T.CCTACACGTTGGTGGA	CC2
SMC16.T.CCTACCAAGATCTGCT	CC2
SMC16.T.CCTACCAAGGTGCAAC	CC2
SMC16.T.CCTACCACAAGAGTCG	CC2
SMC16.T.CCTACCACAGGTGCCT	CC1
SMC16.T.CCTACCATCAGCTGGC	CC1
SMC16.T.CCTACCATCCTACAGA	CC2
SMC16.T.CCTACCATCGCCTGAG	CC2
SMC16.T.CCTAGCTCACAACGCC	CC2
SMC16.T.CCTATTAAGCAGGTCA	CC1
SMC16.T.CCTATTAAGCGGCTTC	CC2
SMC16.T.CCTATTAAGTACGTTC	CC1

SMC16.T.CCTATTACAGCCTTTC	CC2
SMC16.T.CCTATTACAGGAATGC	CC1
SMC16.T.CCTATTAGTGCTGTAT	CC2
SMC16.T.CCTATTAGTTGGACCC	CC2
SMC16.T.CCTATTATCAACCATG	CC1
SMC16.T.CCTATTATCCTTTACA	CC1
SMC16.T.CCTCAGTAGAAACGCC	CC2
SMC16.T.CCTCAGTGTACAGTGG	CC2
SMC16.T.CCTCAGTGTCGAACAG	CC1
SMC16.T.CCTCAGTGTTAGTGGG	CC1
SMC16.T.CCTCTGAAGAAGCCCA	CC1
SMC16.T.CCTCTGAAGTAGGCCA	CC1
SMC16.T.CCTCTGACACATCCAA	CC1
SMC16.T.CCTCTGACATGGTAGG	CC2
SMC16.T.CCTCTGAGTACGCACC	CC1
SMC16.T.CCTTACGAGGCATGGT	CC1
SMC16.T.CCTTACGAGTCCGGTC	CC1
SMC16.T.CCTTACGCATCCAACA	CC1
SMC16.T.CCTTACGGTCAATGTC	CC2
SMC16.T.CCTTACGGTCTTCTCG	CC1
SMC16.T.CCTTACGTCAATCTCT	CC2
SMC16.T.CCTTCCCAGACACGAC	CC2
SMC16.T.CCTTCCCAGGATGCGT	CC1
SMC16.T.CCTTCCCCACCATGTA	CC2
SMC16.T.CCTTCCCGTCAAAGAT	CC2
SMC16.T.CCTTCCCGTGAAAGAG	CC1
SMC16.T.CCTTCCCGTTCGGGCT	CC2
SMC16.T.CCTTCCCTCCTTTACA	CC2
SMC16.T.CCTTCCCTCTGGTGTA	CC1
SMC16.T.CCTTCGAAGTACTTGC	CC2
SMC16.T.CCTTCGAGTAGAGGAA	CC2
SMC16.T.CCTTCGATCGGTGTCG	CC1
SMC16.T.CCTTTCTCAGACACTT	CC1
SMC16.T.CCTTTCTTCAAAGTAG	CC2
SMC16.T.CCTTTCTTCCTTAATC	CC2
SMC16.T.CCTTTCTTCTCGGACG	CC1
SMC16.T.CGAACATAGCCAGTTT	CC2
SMC16.T.CGAACATCAGCCTTGG	CC2
SMC16.T.CGAACATCAGGGATTG	CC2
SMC16.T.CGAACATCATGTAAGA	CC2
SMC16.T.CGAACATGTAATAGCA	CC2
SMC16.T.CGAACATTCACCTTAT	CC1
SMC16.T.CGAACATTCTGTCAAG	CC2
SMC16.T.CGAATGTAGAGTAAGG	CC2
SMC16.T.CGAATGTAGGGAGTAA	CC2
SMC16.T.CGAATGTCATGTAAGA	CC2
SMC16.T.CGAATGTGTGCCTGTG	CC2
SMC16.T.CGAATGTGTGTCGCTG	CC2
SMC16.T.CGAATGTGTTTGACTG	CC1
SMC16.T.CGAATGTTCGAGGTAG	CC2
SMC16.T.CGACCTTAGAAACCAT	CC2
SMC16.T.CGACCTTCAGACAAAT	CC1
SMC16.T.CGACCTTCATACAGCT	CC1
SMC16.T.CGACTTCAGGCATGGT	CC1
SMC16.T.CGACTTCAGGCATGTG	CC2
SMC16.T.CGACTTCAGTTTCCTT	CC1
SMC16.T.CGACTTCCACATGTGT	CC1
SMC16.T.CGACTTCCAGTAAGAT	CC2

SMC16.T.CGACTTCTCCTAGTGA	CC2
SMC16.T.CGAGAAGAGATGGCGT	CC2
SMC16.T.CGAGAAGCAAGCCCAC	CC1
SMC16.T.CGAGAAGGTCATGCAT	CC1
SMC16.T.CGAGAAGGTCCAAGTT	CC1
SMC16.T.CGAGAAGTCAAAGACA	CC2
SMC16.T.CGAGAAGTCGGCCGAT	CC1
SMC16.T.CGAGCACAGGACAGCT	CC1
SMC16.T.CGAGCACGTTACCAGT	CC1
SMC16.T.CGAGCACTCCTACAGA	CC2
SMC16.T.CGAGCCAAGCGTAGTG	CC1
SMC16.T.CGAGCCAAGTAACCCT	CC2
SMC16.T.CGAGCCAAGTCAAGGC	CC1
SMC16.T.CGAGCCAAGTTACGGG	CC1
SMC16.T.CGAGCCAGTAGCTAAA	CC1
SMC16.T.CGAGCCAGTGAGTGAC	CC1
SMC16.T.CGAGCCATCCAACCAA	CC1
SMC16.T.CGAGCCATCTGGGCCA	CC1
SMC16.T.CGAGCCATCTTGACGA	CC2
SMC16.T.CGATCGGCATCAGTCA	CC1
SMC16.T.CGATCGGCATTAGCCA	CC2
SMC16.T.CGATCGGGTGTGTGCC	CC1
SMC16.T.CGATCGGTCCTTGCCA	CC1
SMC16.T.CGATGGCCACTCGACG	CC2
SMC16.T.CGATGGCGTACCATCA	CC2
SMC16.T.CGATGGCGTATAGGGC	CC2
SMC16.T.CGATGGCTCTGCTGTC	CC2
SMC16.T.CGATGTAAGAGCTGGT	CC2
SMC16.T.CGATGTAGTCGAGATG	CC2
SMC16.T.CGATGTATCACTTACT	CC2
SMC16.T.CGATGTATCATATCGG	CC2
SMC16.T.CGATGTATCCACGAAT	CC1
SMC16.T.CGATGTATCTGAAAGA	CC2
SMC16.T.CGATTGAAGGAGTTGC	CC1
SMC16.T.CGATTGAAGGCTAGAC	CC2
SMC16.T.CGATTGACATGGTCAT	CC2
SMC16.T.CGATTGACATGTTCCC	CC2
SMC16.T.CGATTGAGTAAGGGAA	CC2
SMC16.T.CGATTGAGTACTTCTT	CC1
SMC16.T.CGATTGAGTTGCTCCT	CC2
SMC16.T.CGATTGATCACGGTTA	CC1
SMC16.T.CGATTGATCTTAGAGC	CC2
SMC16.T.CGCCAAGAGCAATATG	CC2
SMC16.T.CGCCAAGCACAAGACG	CC1
SMC16.T.CGCCAAGCAGCTTCGG	CC2
SMC16.T.CGCCAAGCATCGACGC	CC2
SMC16.T.CGCCAAGGTACTTCTT	CC2
SMC16.T.CGCCAAGGTCAAACTC	CC2
SMC16.T.CGCCAAGGTCCTGCTT	CC2
SMC16.T.CGCCAAGGTTCGCTAA	CC2
SMC16.T.CGCCAAGTCGAGCCCA	CC2
SMC16.T.CGCGGTACATCTATGG	CC2
SMC16.T.CGCGGTAGTAAGTAGT	CC2
SMC16.T.CGCGGTAGTAGGCTGA	CC2
SMC16.T.CGCGGTAGTCGTGGCT	CC2
SMC16.T.CGCGGTATCTTGCAAG	CC2
SMC16.T.CGCGTTTAGACACGAC	CC1
SMC16.T.CGCGTTTAGTCAAGCG	CC1

SMC16.T.CGCGTTTAGTTAGCGG	CC1
SMC16.T.CGCGTTTCAATGGAGC	CC2
SMC16.T.CGCGTTTCACCATCCT	CC2
SMC16.T.CGCGTTTCATTGGCGC	CC1
SMC16.T.CGCGTTTGTCTTGTCC	CC2
SMC16.T.CGCGTTTGTTCGCTAA	CC2
SMC16.T.CGCGTTTTCAGCTGGC	CC2
SMC16.T.CGCGTTTTCTGAGTGT	CC1
SMC16.T.CGCTATCAGAGCTTCT	CC2
SMC16.T.CGCTATCAGGGCTCTC	CC2
SMC16.T.CGCTATCAGTGACTCT	CC2
SMC16.T.CGCTATCCATCCGTGG	CC2
SMC16.T.CGCTATCGTACGACCC	CC2
SMC16.T.CGCTATCGTCGCCATG	CC2
SMC16.T.CGCTATCGTCTGATTG	CC2
SMC16.T.CGCTATCTCGCGATCG	CC2
SMC16.T.CGCTATCTCTGAAAGA	CC2
SMC16.T.CGCTGGACAAGTCTGT	CC1
SMC16.T.CGCTGGACAGACGCTC	CC1
SMC16.T.CGCTGGACAGTATCTG	CC1
SMC16.T.CGCTGGACATCCCATC	CC1
SMC16.T.CGCTGGATCACCATAG	CC1
SMC16.T.CGCTGGATCCCATTAT	CC2
SMC16.T.CGCTGGATCCTTGCCA	CC1
SMC16.T.CGCTTCAAGACATAAC	CC2
SMC16.T.CGCTTCACACGACTCG	CC2
SMC16.T.CGCTTCATCAGTACGT	CC1
SMC16.T.CGGACACAGATCACGG	CC1
SMC16.T.CGGACACAGCCAGTAG	CC2
SMC16.T.CGGACACAGGACATTA	CC2
SMC16.T.CGGACACCACGTAAGG	CC2
SMC16.T.CGGACACCACTATCTT	CC1
SMC16.T.CGGACACCATGCTGGC	CC2
SMC16.T.CGGACACCATTCCTCG	CC1
SMC16.T.CGGACGTCACCTCGTT	CC1
SMC16.T.CGGACGTCAGGAACGT	CC2
SMC16.T.CGGACGTCAGGACCCT	CC1
SMC16.T.CGGACGTGTTAGAACA	CC1
SMC16.T.CGGACGTTCAGTGTTG	CC1
SMC16.T.CGGACGTTCATTTGGG	CC2
SMC16.T.CGGACGTTCTAACCGA	CC2
SMC16.T.CGGACGTTCTCTAAGG	CC1
SMC16.T.CGGACTGAGCCACCTG	CC1
SMC16.T.CGGACTGAGCTAACTC	CC1
SMC16.T.CGGACTGAGTGAATTG	CC2
SMC16.T.CGGACTGCAGCTGTAT	CC2
SMC16.T.CGGACTGTCGTTGCCT	CC2
SMC16.T.CGGAGCTAGACAAGCC	CC2
SMC16.T.CGGAGCTAGCCAGAAC	CC1
SMC16.T.CGGAGCTAGCTTCGCG	CC2
SMC16.T.CGGAGCTAGGCTACGA	CC2
SMC16.T.CGGAGCTAGTGCCAGA	CC2
SMC16.T.CGGAGCTCACACGCTG	CC2
SMC16.T.CGGAGCTCATGGTTGT	CC2
SMC16.T.CGGAGCTGTACCGCTG	CC2
SMC16.T.CGGAGCTGTCTCTTTA	CC2
SMC16.T.CGGAGCTGTTATCGGT	CC1
SMC16.T.CGGAGCTTCATGTCTT	CC1

SMC16.T.CGGAGTCAGAGGTTAT	CC2
SMC16.T.CGGAGTCAGTAGGTGC	CC2
SMC16.T.CGGAGTCCACGAAAGC	CC1
SMC16.T.CGGAGTCGTGCCTTGG	CC2
SMC16.T.CGGAGTCTCCGCGGTA	CC2
SMC16.T.CGGAGTCTCGATGAGG	CC2
SMC16.T.CGGAGTCTCGTCTGAA	CC2
SMC16.T.CGGAGTCTCTAACTCT	CC2
SMC16.T.CGGCTAGAGTTCGCAT	CC1
SMC16.T.CGGCTAGCAAGCGATG	CC1
SMC16.T.CGGCTAGCAATCTGCA	CC1
SMC16.T.CGGCTAGGTCATACTG	CC1
SMC16.T.CGGCTAGGTCTGGAGA	CC1
SMC16.T.CGGCTAGTCCTCCTAG	CC2
SMC16.T.CGGGTCAAGTGCTGCC	CC2
SMC16.T.CGGGTCAAGTGGGCTA	CC1
SMC16.T.CGGGTCAAGTGTACGG	CC2
SMC16.T.CGGGTCAGTCTAAAGA	CC2
SMC16.T.CGGGTCATCTAGAGTC	CC1
SMC16.T.CGGGTCATCTTATCTG	CC2
SMC16.T.CGGTTAAAGAGACTTA	CC1
SMC16.T.CGGTTAAAGCTGCAAG	CC2
SMC16.T.CGGTTAAAGGTAAACT	CC2
SMC16.T.CGGTTAACAGTATAAG	CC1
SMC16.T.CGGTTAACATAAAGGT	CC2
SMC16.T.CGGTTAAGTCGACTGC	CC2
SMC16.T.CGGTTAAGTTGTGGAG	CC1
SMC16.T.CGTAGCGAGAGGTTGC	CC1
SMC16.T.CGTAGCGCAGGAATCG	CC1
SMC16.T.CGTAGCGGTAACGCGA	CC2
SMC16.T.CGTAGCGGTACTTAGC	CC1
SMC16.T.CGTAGCGGTCACCTAA	CC2
SMC16.T.CGTAGCGTCGTTTGCC	CC1
SMC16.T.CGTAGGCAGCAGATCG	CC1
SMC16.T.CGTAGGCAGGCTATCT	CC1
SMC16.T.CGTAGGCCATGCAATC	CC1
SMC16.T.CGTAGGCGTACCGTTA	CC2
SMC16.T.CGTAGGCGTCTGGTCG	CC2
SMC16.T.CGTAGGCTCAAGCCTA	CC2
SMC16.T.CGTCACTCAGCGAACA	CC1
SMC16.T.CGTCACTTCATGTCCC	CC1
SMC16.T.CGTCACTTCTATCGCC	CC2
SMC16.T.CGTCAGGAGACTTGAA	CC2
SMC16.T.CGTCAGGAGCGATTCT	CC2
SMC16.T.CGTCAGGGTTGTCGCG	CC2
SMC16.T.CGTCAGGTCAAGCCTA	CC1
SMC16.T.CGTCAGGTCCTGCCAT	CC1
SMC16.T.CGTCCATAGCTACCGC	CC1
SMC16.T.CGTCCATAGGAGCGAG	CC1
SMC16.T.CGTCCATAGTCCATAC	CC2
SMC16.T.CGTCCATCAAGACGTG	CC2
SMC16.T.CGTCCATCACCTATCC	CC2
SMC16.T.CGTCCATTCAACACTG	CC2
SMC16.T.CGTCCATTCCACGACG	CC2
SMC16.T.CGTCTACAGCAGGTCA	CC1
SMC16.T.CGTCTACAGTTCGCGC	CC2
SMC16.T.CGTCTACGTGCCTGTG	CC2
SMC16.T.CGTGAGCCAATGGATA	CC2

SMC16.T.CGTGAGCCACAACGCC	CC1
SMC16.T.CGTGAGCGTACACCGC	CC2
SMC16.T.CGTGAGCGTACGACCC	CC1
SMC16.T.CGTGAGCGTTCCGGCA	CC2
SMC16.T.CGTGAGCTCAGGCGAA	CC2
SMC16.T.CGTGTAACACAGGAGT	CC1
SMC16.T.CGTGTAACACTTACGA	CC2
SMC16.T.CGTGTAATCACCAGGC	CC2
SMC16.T.CGTGTCTAGTGGAGTC	CC2
SMC16.T.CGTGTCTCAGTCGTGC	CC2
SMC16.T.CGTGTCTCATTCCTCG	CC1
SMC16.T.CGTGTCTGTTACGACT	CC2
SMC16.T.CGTGTCTTCCTTGCCA	CC1
SMC16.T.CGTTAGAAGCGATATA	CC1
SMC16.T.CGTTAGACAGCGTAAG	CC1
SMC16.T.CGTTAGAGTCCAGTTA	CC1
SMC16.T.CGTTAGATCATCACCC	CC2
SMC16.T.CGTTAGATCTCAAACG	CC1
SMC16.T.CGTTCTGAGCGATGAC	CC2
SMC16.T.CGTTCTGAGCGTCTAT	CC1
SMC16.T.CGTTCTGCAGCTGCAC	CC2
SMC16.T.CGTTCTGCAGTCAGCC	CC1
SMC16.T.CGTTCTGGTACAGACG	CC1
SMC16.T.CTAACTTAGCTGCGAA	CC2
SMC16.T.CTAACTTGTGATGATA	CC2
SMC16.T.CTAACTTGTGTGAAAT	CC2
SMC16.T.CTAACTTTCACGCGGT	CC2
SMC16.T.CTAAGACAGAGACTAT	CC2
SMC16.T.CTAAGACAGATGCCTT	CC2
SMC16.T.CTAAGACAGGCGCTCT	CC2
SMC16.T.CTAAGACCAATCGGTT	CC2
SMC16.T.CTAAGACGTGCAGGTA	CC1
SMC16.T.CTAAGACTCGCGATCG	CC2
SMC16.T.CTAATGGAGGCCCTCA	CC2
SMC16.T.CTAATGGAGGGTTCCC	CC2
SMC16.T.CTAATGGCACAAGTAA	CC2
SMC16.T.CTAATGGCAGACGCCT	CC2
SMC16.T.CTAATGGGTAGCGCAA	CC1
SMC16.T.CTAATGGGTAGGCTGA	CC2
SMC16.T.CTAATGGGTGGGTATG	CC2
SMC16.T.CTAATGGTCGCTGATA	CC2
SMC16.T.CTAATGGTCGGATGGA	CC1
SMC16.T.CTACACCAGGCCCGTT	CC1
SMC16.T.CTACACCAGTAGCGGT	CC2
SMC16.T.CTACACCAGTATTGGA	CC1
SMC16.T.CTACACCAGTTAAGTG	CC1
SMC16.T.CTACACCCAGTCAGAG	CC1
SMC16.T.CTACACCGTGAGGCTA	CC2
SMC16.T.CTACACCGTGTGCCTG	CC2
SMC16.T.CTACACCTCATACGGT	CC2
SMC16.T.CTACATTCAGCTGGCT	CC1
SMC16.T.CTACATTCATGCTAGT	CC2
SMC16.T.CTACATTGTATGAATG	CC2
SMC16.T.CTACATTGTCCGTTAA	CC2
SMC16.T.CTACATTGTTACGACT	CC2
SMC16.T.CTACATTTCCATGAGT	CC1
SMC16.T.CTACATTTCTGTTGAG	CC2
SMC16.T.CTACCCAAGAACAACT	CC2

SMC16.T.CTACCCAAGTATCTCG	CC2
SMC16.T.CTACCCACAAAGTGCG	CC1
SMC16.T.CTACCCACACACCGAC	CC2
SMC16.T.CTACCCACAGACGCTC	CC2
SMC16.T.CTACCCAGTAGCCTCG	CC1
SMC16.T.CTACCCAGTATTACCG	CC2
SMC16.T.CTACCCAGTCCCTACT	CC2
SMC16.T.CTACCCATCACGAAGG	CC1
SMC16.T.CTACGTCAGGGTGTTG	CC2
SMC16.T.CTACGTCAGGTGTGGT	CC2
SMC16.T.CTACGTCCACGCTTTC	CC2
SMC16.T.CTACGTCCATTTGCCC	CC2
SMC16.T.CTACGTCGTTCGTGAT	CC2
SMC16.T.CTACGTCGTTGATTCG	CC2
SMC16.T.CTAGAGTAGATGTGGC	CC1
SMC16.T.CTAGAGTAGGATTCGG	CC1
SMC16.T.CTAGAGTAGGGTGTTG	CC1
SMC16.T.CTAGAGTAGTGTTGAA	CC2
SMC16.T.CTAGAGTGTAAATGAC	CC2
SMC16.T.CTAGAGTGTCGGGTCT	CC2
SMC16.T.CTAGAGTGTTGATTCG	CC2
SMC16.T.CTAGAGTGTTGTTTGG	CC1
SMC16.T.CTAGAGTTCTCCTATA	CC2
SMC16.T.CTAGCCTAGACGCACA	CC2
SMC16.T.CTAGCCTAGTGCGATG	CC1
SMC16.T.CTAGCCTCAACGATGG	CC2
SMC16.T.CTAGCCTGTTCAGTAC	CC2
SMC16.T.CTAGTGAAGAAGGGTA	CC1
SMC16.T.CTAGTGAAGATCACGG	CC2
SMC16.T.CTAGTGAGTCAATACC	CC1
SMC16.T.CTAGTGAGTCATATGC	CC2
SMC16.T.CTAGTGAGTCTGCGGT	CC2
SMC16.T.CTCACACAGTGAACAT	CC1
SMC16.T.CTCACACCATTTCAGG	CC2
SMC16.T.CTCACACGTATGGTTC	CC2
SMC16.T.CTCACACGTGTAAGTA	CC1
SMC16.T.CTCACACTCAGTTGAC	CC1
SMC16.T.CTCACACTCGAATCCA	CC2
SMC16.T.CTCAGAAAGCGGATCA	CC2
SMC16.T.CTCAGAACAGTGGGAT	CC2
SMC16.T.CTCAGAAGTGCACGAA	CC1
SMC16.T.CTCAGAATCCTGCAGG	CC1
SMC16.T.CTCATTAAGTACATGA	CC1
SMC16.T.CTCATTAGTAAACCTC	CC2
SMC16.T.CTCATTAGTCCAAGTT	CC1
SMC16.T.CTCATTAGTCTAACGT	CC2
SMC16.T.CTCATTAGTCTAGCCG	CC2
SMC16.T.CTCATTAGTCTAGCGC	CC2
SMC16.T.CTCATTAGTGTATGGG	CC2
SMC16.T.CTCATTAGTTATGCGT	CC2
SMC16.T.CTCATTATCGTACCGG	CC1
SMC16.T.CTCCTAGAGCTGATAA	CC2
SMC16.T.CTCCTAGGTCTAAAGA	CC1
SMC16.T.CTCCTAGTCAAAGTAG	CC2
SMC16.T.CTCCTAGTCCGGGTGT	CC2
SMC16.T.CTCGAGGAGGGTGTGT	CC1
SMC16.T.CTCGAGGGTCACTTCC	CC1
SMC16.T.CTCGAGGTCGCATGAT	CC1

SMC16.T.CTCGGAGAGACGCACA	CC1
SMC16.T.CTCGGAGAGACTGTAA	CC2
SMC16.T.CTCGGAGCACTGAAGG	CC2
SMC16.T.CTCGGAGCATGGTCAT	CC2
SMC16.T.CTCGGGACAGGATTGG	CC1
SMC16.T.CTCGGGACAGGTGGAT	CC1
SMC16.T.CTCGGGACATCACGTA	CC2
SMC16.T.CTCGGGAGTCGTGGCT	CC1
SMC16.T.CTCGTACAGCTAGCCC	CC2
SMC16.T.CTCGTACAGGTGTGGT	CC1
SMC16.T.CTCGTACCAGGCAGTA	CC2
SMC16.T.CTCGTACTCTTGACGA	CC1
SMC16.T.CTCGTCAGTAGAGGAA	CC2
SMC16.T.CTCGTCAGTCCCGACA	CC1
SMC16.T.CTCGTCAGTGACTCAT	CC1
SMC16.T.CTCGTCATCAGCACAT	CC2
SMC16.T.CTCGTCATCGAGGTAG	CC1
SMC16.T.CTCGTCATCGTGGTCG	CC2
SMC16.T.CTCGTCATCTGGTGTA	CC1
SMC16.T.CTCTAATAGATGCCTT	CC2
SMC16.T.CTCTAATAGGTGGGTT	CC1
SMC16.T.CTCTAATCAGGTGGAT	CC2
SMC16.T.CTCTAATGTACAGCAG	CC2
SMC16.T.CTCTAATGTACCGCTG	CC1
SMC16.T.CTCTAATGTCTCCCTA	CC2
SMC16.T.CTCTAATTCAACCAAC	CC2
SMC16.T.CTCTAATTCAGTTCGA	CC2
SMC16.T.CTCTAATTCTGCCAGG	CC2
SMC16.T.CTCTACGCAAGACACG	CC2
SMC16.T.CTCTACGTCGTGGTCG	CC1
SMC16.T.CTCTGGTAGGTGATTA	CC2
SMC16.T.CTCTGGTCAGGATCGA	CC2
SMC16.T.CTCTGGTGTCCATGAT	CC2
SMC16.T.CTCTGGTTCGCCATAA	CC2
SMC16.T.CTGAAACAGAACAACT	CC2
SMC16.T.CTGAAACAGGGTGTGT	CC2
SMC16.T.CTGAAACAGGTCGGAT	CC2
SMC16.T.CTGAAACAGGTGCTAG	CC2
SMC16.T.CTGAAACCAAAGAATC	CC2
SMC16.T.CTGAAACGTACTCAAC	CC2
SMC16.T.CTGAAGTAGTCATCCA	CC2
SMC16.T.CTGAAGTGTAGCGCTC	CC1
SMC16.T.CTGAAGTTCAAGGCTT	CC1
SMC16.T.CTGAAGTTCGTCTGCT	CC2
SMC16.T.CTGATAGAGAGACTTA	CC1
SMC16.T.CTGATAGAGTGAACGC	CC2
SMC16.T.CTGATAGCATTGGTAC	CC2
SMC16.T.CTGATAGGTAAACGCG	CC1
SMC16.T.CTGATAGTCACCTTAT	CC1
SMC16.T.CTGATAGTCACGGTTA	CC2
SMC16.T.CTGATAGTCCACGCAG	CC1
SMC16.T.CTGATAGTCGAACGGA	CC1
SMC16.T.CTGATAGTCTATCCTA	CC2
SMC16.T.CTGATCCAGTCCCACG	CC2
SMC16.T.CTGATCCCACAAGCCC	CC2
SMC16.T.CTGATCCCACCCTATC	CC1
SMC16.T.CTGATCCGTAGGACAC	CC2
SMC16.T.CTGATCCGTGTGCGTC	CC1

SMC16.T.CTGATCCTCACATGCA	CC1
SMC16.T.CTGATCCTCACTGGGC	CC1
SMC16.T.CTGATCCTCCAGAAGG	CC1
SMC16.T.CTGATCCTCTTTACGT	CC2
SMC16.T.CTGCCTAAGATAGCAT	CC2
SMC16.T.CTGCCTAAGGCAAAGA	CC1
SMC16.T.CTGCCTACATTGAGCT	CC1
SMC16.T.CTGCCTATCAGGATCT	CC1
SMC16.T.CTGCGGAAGAGGACGG	CC2
SMC16.T.CTGCGGAAGGGTATCG	CC2
SMC16.T.CTGCGGATCGCAAACT	CC1
SMC16.T.CTGCTGTAGTGAACGC	CC1
SMC16.T.CTGCTGTCAAGCCGCT	CC2
SMC16.T.CTGCTGTCACAGCGTC	CC2
SMC16.T.CTGCTGTGTCGGCTCA	CC2
SMC16.T.CTGCTGTTCACTCCTG	CC1
SMC16.T.CTGGTCTCACCGAAAG	CC2
SMC16.T.CTGGTCTCAGCGTTCG	CC2
SMC16.T.CTGGTCTCATCTCCCA	CC1
SMC16.T.CTGGTCTTCATCACCC	CC1
SMC16.T.CTGTGCTCACATTCGA	CC2
SMC16.T.CTGTGCTGTAAGTGTA	CC1
SMC16.T.CTGTGCTGTGAAAGAG	CC1
SMC16.T.CTGTGCTTCAGCACAT	CC1
SMC16.T.CTGTGCTTCCTCGCAT	CC1
SMC16.T.CTGTGCTTCGGAGGTA	CC1
SMC16.T.CTGTGCTTCGTGGACC	CC2
SMC16.T.CTGTGCTTCGTTGACA	CC1
SMC16.T.CTGTGCTTCGTTTAGG	CC2
SMC16.T.CTGTTTAAGCAATCTC	CC2
SMC16.T.CTGTTTATCAGAAATG	CC1
SMC16.T.CTGTTTATCATACGGT	CC2
SMC16.T.CTTAACTCATTACCTT	CC2
SMC16.T.CTTAACTGTTTAGGAA	CC1
SMC16.T.CTTAACTTCACATAGC	CC2
SMC16.T.CTTAACTTCCATGCTC	CC2
SMC16.T.CTTACCGCATCCTAGA	CC1
SMC16.T.CTTACCGCATTCCTGC	CC2
SMC16.T.CTTAGGACAATAGAGT	CC2
SMC16.T.CTTAGGAGTTATCGGT	CC2
SMC16.T.CTTCTCTAGCCAGTAG	CC1
SMC16.T.CTTCTCTCAAAGGTGC	CC1
SMC16.T.CTTCTCTCAAGAGGCT	CC1
SMC16.T.CTTCTCTCACAAGACG	CC2
SMC16.T.CTTCTCTCACAAGCCC	CC1
SMC16.T.CTTCTCTCAGGTTTCA	CC1
SMC16.T.CTTCTCTCATCTACGA	CC1
SMC16.T.CTTCTCTGTGGCCCTA	CC2
SMC16.T.CTTCTCTGTTATCCGA	CC1
SMC16.T.CTTGGCTAGACCCACC	CC2
SMC16.T.CTTGGCTAGCGGATCA	CC1
SMC16.T.CTTGGCTAGTAGCCGA	CC1
SMC16.T.CTTGGCTAGTCAAGGC	CC1
SMC16.T.CTTGGCTAGTCATCCA	CC2
SMC16.T.CTTGGCTAGTGTACTC	CC2
SMC16.T.CTTGGCTAGTTGTCGT	CC1
SMC16.T.CTTGGCTCAAGAAAGG	CC2
SMC16.T.CTTGGCTCACAGACAG	CC2

SMC16.T.CTTGGCTCAGGCTGAA	CC1
SMC16.T.CTTGGCTCATGCTAGT	CC2
SMC16.T.CTTGGCTGTCAACTGT	CC1
SMC16.T.CTTTGCGAGCTGCGAA	CC1
SMC16.T.CTTTGCGAGGCTCATT	CC1
SMC16.T.CTTTGCGCAAGCGCTC	CC1
SMC16.T.CTTTGCGCATTCTTAC	CC1
SMC16.T.CTTTGCGGTCCAGTGC	CC2
SMC16.T.CTTTGCGTCAAACCGT	CC2
SMC16.T.CTTTGCGTCCTATGTT	CC1
SMC16.T.CTTTGCGTCCTGCCAT	CC1
SMC16.T.GAAACTCAGTAGGTGC	CC1
SMC16.T.GAAACTCCAGCAGTTT	CC2
SMC16.T.GAAACTCCAGTCTTCC	CC2
SMC16.T.GAAACTCGTCCAACTA	CC1
SMC16.T.GAAACTCGTCCGAACC	CC1
SMC16.T.GAAACTCTCCGCGCAA	CC1
SMC16.T.GAAACTCTCCGTCAAA	CC2
SMC16.T.GAAATGACAAGACGTG	CC1
SMC16.T.GAACATCAGTGCTGCC	CC2
SMC16.T.GAACATCCAAATACAG	CC2
SMC16.T.GAACATCCACCTCGTT	CC2
SMC16.T.GAACATCCACTGAAGG	CC1
SMC16.T.GAACATCTCCACGACG	CC1
SMC16.T.GAACATCTCTGACCTC	CC2
SMC16.T.GAACCTAAGCCCAATT	CC2
SMC16.T.GAACCTAAGTAGCCGA	CC2
SMC16.T.GAACCTACACGGTAGA	CC1
SMC16.T.GAACCTACACGGTGTC	CC1
SMC16.T.GAACCTAGTCATCCCT	CC1
SMC16.T.GAACCTAGTGCAGGTA	CC2
SMC16.T.GAACCTAGTGCGAAAC	CC2
SMC16.T.GAACCTAGTGGCGAAT	CC2
SMC16.T.GAACCTAGTTACGACT	CC2
SMC16.T.GAACCTATCTGTCCGT	CC2
SMC16.T.GAACGGACAAGTCTAC	CC2
SMC16.T.GAACGGACAGACACTT	CC2
SMC16.T.GAACGGAGTCCATGAT	CC2
SMC16.T.GAACGGAGTGAGGGAG	CC2
SMC16.T.GAACGGAGTGCGCTTG	CC1
SMC16.T.GAACGGAGTTAAAGAC	CC2
SMC16.T.GAAGCAGAGACACGAC	CC1
SMC16.T.GAAGCAGAGAGGTACC	CC1
SMC16.T.GAAGCAGAGGCTATCT	CC2
SMC16.T.GAAGCAGAGGGTTCCC	CC1
SMC16.T.GAAGCAGGTCAAGCGA	CC2
SMC16.T.GAAGCAGGTTCGGGCT	CC1
SMC16.T.GAATAAGAGTGTTGAA	CC2
SMC16.T.GAATAAGAGTTGCAGG	CC2
SMC16.T.GAATAAGCAAGGCTCC	CC1
SMC16.T.GAATAAGCAAGTCTAC	CC1
SMC16.T.GAATAAGGTAATCGTC	CC1
SMC16.T.GAATAAGGTTAAGACA	CC2
SMC16.T.GAATAAGTCACATGCA	CC1
SMC16.T.GAATAAGTCATTATCC	CC1
SMC16.T.GAATGAAAGAGGTTAT	CC1
SMC16.T.GAATGAAAGTACGACG	CC1
SMC16.T.GAATGAACAGACAGGT	CC1

SMC16.T.GAATGAACAGATGGCA	CC2
SMC16.T.GAATGAACAGCTTCGG	CC1
SMC16.T.GAATGAACATGATCCA	CC2
SMC16.T.GAATGAAGTCAGAAGC	CC2
SMC16.T.GAATGAAGTGCCTTGG	CC2
SMC16.T.GAATGAATCCAACCAA	CC2
SMC16.T.GAATGAATCTGGTATG	CC1
SMC16.T.GACACGCAGGCATGTG	CC2
SMC16.T.GACACGCAGGGAACGG	CC2
SMC16.T.GACACGCCAGCTGTGC	CC2
SMC16.T.GACACGCGTACCGTAT	CC1
SMC16.T.GACACGCGTATGCTTG	CC2
SMC16.T.GACACGCGTCGGCTCA	CC2
SMC16.T.GACACGCGTGATGCCC	CC2
SMC16.T.GACACGCGTTGTCTTT	CC1
SMC16.T.GACACGCTCACATAGC	CC1
SMC16.T.GACACGCTCACCAGGC	CC2
SMC16.T.GACAGAGAGAAGAAGC	CC2
SMC16.T.GACAGAGAGCCGATTT	CC2
SMC16.T.GACAGAGCATAACCTG	CC2
SMC16.T.GACAGAGCATGCCTAA	CC1
SMC16.T.GACAGAGGTCTAACGT	CC2
SMC16.T.GACAGAGGTGCAGACA	CC1
SMC16.T.GACAGAGGTTCGTTGA	CC1
SMC16.T.GACCAATCACAGACTT	CC2
SMC16.T.GACCAATCATGTAGTC	CC2
SMC16.T.GACCAATTCACCTCGT	CC2
SMC16.T.GACCAATTCTTCTGGC	CC2
SMC16.T.GACCAATTCTTTCCTC	CC2
SMC16.T.GACCTGGCAAGTAATG	CC2
SMC16.T.GACCTGGCATACGCCG	CC2
SMC16.T.GACCTGGTCGGTGTTA	CC1
SMC16.T.GACCTGGTCTTGCCGT	CC2
SMC16.T.GACGCGTAGCACAGGT	CC2
SMC16.T.GACGCGTCAAGTTGTC	CC1
SMC16.T.GACGCGTCATTAACCG	CC1
SMC16.T.GACGCGTGTGCTTCTC	CC2
SMC16.T.GACGCGTGTGGACGAT	CC2
SMC16.T.GACGCGTTCACCATAG	CC2
SMC16.T.GACGCGTTCCCGACTT	CC1
SMC16.T.GACGGCTAGGGTGTTG	CC2
SMC16.T.GACGGCTCACACGCTG	CC1
SMC16.T.GACGGCTCATGCCTTC	CC2
SMC16.T.GACGGCTTCCAACCAA	CC1
SMC16.T.GACGGCTTCGAGCCCA	CC1
SMC16.T.GACGGCTTCTACCAGA	CC1
SMC16.T.GACGTGCAGCCTCGTG	CC1
SMC16.T.GACGTGCCAGATAATG	CC1
SMC16.T.GACGTGCCATCTCGCT	CC2
SMC16.T.GACGTGCGTAGAAGGA	CC2
SMC16.T.GACGTGCGTGCGATAG	CC2
SMC16.T.GACGTGCTCAGAGACG	CC1
SMC16.T.GACGTTAAGCCCTAAT	CC1
SMC16.T.GACGTTAAGGATGCGT	CC1
SMC16.T.GACGTTACAATAGCAA	CC2
SMC16.T.GACGTTACACAGACAG	CC1
SMC16.T.GACGTTACACATGACT	CC1
SMC16.T.GACGTTAGTTACGGAG	CC2

SMC16.T.GACGTTATCTGTTGAG	CC1
SMC16.T.GACTAACAGATCCCGC	CC1
SMC16.T.GACTAACAGCGAGAAA	CC2
SMC16.T.GACTAACTCAGCCTAA	CC1
SMC16.T.GACTAACTCGTGGTCG	CC1
SMC16.T.GACTAACTCTGTTTGT	CC2
SMC16.T.GACTACACACCAGCAC	CC2
SMC16.T.GACTACACATGCCTAA	CC1
SMC16.T.GACTACAGTACTTAGC	CC2
SMC16.T.GACTACAGTCAGTGGA	CC2
SMC16.T.GACTACATCAGAAATG	CC1
SMC16.T.GACTACATCTATCCTA	CC1
SMC16.T.GACTACATCTTCCTTC	CC1
SMC16.T.GACTGCGAGCCACGTC	CC1
SMC16.T.GACTGCGAGTAGGTGC	CC2
SMC16.T.GACTGCGAGTCCAGGA	CC1
SMC16.T.GACTGCGCAATCGAAA	CC1
SMC16.T.GACTGCGCAATGACCT	CC1
SMC16.T.GACTGCGGTCGCATCG	CC1
SMC16.T.GACTGCGGTGAGGGTT	CC1
SMC16.T.GACTGCGGTGGACGAT	CC2
SMC16.T.GACTGCGTCCAGATCA	CC2
SMC16.T.GACTGCGTCGCGGATC	CC2
SMC16.T.GAGCAGACACTCAGGC	CC2
SMC16.T.GAGCAGATCCTATGTT	CC2
SMC16.T.GAGCAGATCTAACGGT	CC1
SMC16.T.GAGGTGAAGAGTGAGA	CC2
SMC16.T.GAGGTGAAGCACGCCT	CC2
SMC16.T.GAGGTGAAGTGTGGCA	CC2
SMC16.T.GAGGTGACAGCCAGAA	CC2
SMC16.T.GAGGTGACAGGGTTAG	CC2
SMC16.T.GAGGTGACATAGGATA	CC2
SMC16.T.GAGGTGAGTAAGTGTA	CC2
SMC16.T.GAGTCCGCAGTCACTA	CC2
SMC16.T.GAGTCCGTCACATGCA	CC2
SMC16.T.GAGTCCGTCCCGACTT	CC1
SMC16.T.GAGTCCGTCTCGTATT	CC1
SMC16.T.GATCAGTGTTAGGGTG	CC1
SMC16.T.GATCAGTTCTCGCTTG	CC1
SMC16.T.GATCGATAGGATGGTC	CC2
SMC16.T.GATCGATCAACTGGCC	CC2
SMC16.T.GATCGATCACATTTCT	CC2
SMC16.T.GATCGATGTCTAACGT	CC2
SMC16.T.GATCGATGTCTAGAGG	CC1
SMC16.T.GATCGATGTTATGTGC	CC1
SMC16.T.GATCGATGTTGTACAC	CC2
SMC16.T.GATCGCGAGAAACCAT	CC1
SMC16.T.GATCGCGAGACCTTTG	CC2
SMC16.T.GATCGCGCACGAAGCA	CC1
SMC16.T.GATCGCGCACGAGGTA	CC1
SMC16.T.GATCGCGCAGCATACT	CC1
SMC16.T.GATCGCGGTCATATCG	CC2
SMC16.T.GATCGCGGTCGAGATG	CC2
SMC16.T.GATCGTAAGTCAATAG	CC1
SMC16.T.GATCGTAAGTTGTAGA	CC2
SMC16.T.GATCGTACAAAGCGGT	CC1
SMC16.T.GATCGTACAGCTCGAC	CC1
SMC16.T.GATCGTAGTATGGTTC	CC2

SMC16.T.GATCGTAGTGAAGGCT	CC1
SMC16.T.GATCTAGAGCAACGGT	CC2
SMC16.T.GATCTAGAGCAGGTCA	CC2
SMC16.T.GATCTAGCAAATCCGT	CC1
SMC16.T.GATCTAGCAATAAGCA	CC2
SMC16.T.GATGAAAAGTACGTTC	CC2
SMC16.T.GATGAAACAAAGCAAT	CC1
SMC16.T.GATGAAACATCTATGG	CC2
SMC16.T.GATGAAATCAAACGGG	CC1
SMC16.T.GATGAAATCGCCGTGA	CC2
SMC16.T.GATGAGGAGCGTCTAT	CC2
SMC16.T.GATGAGGAGCGTGAAC	CC2
SMC16.T.GATGAGGCACGAAATA	CC2
SMC16.T.GATGAGGCATTATCTC	CC2
SMC16.T.GATGAGGGTCGCTTTC	CC2
SMC16.T.GATGAGGTCGCCTGTT	CC1
SMC16.T.GATGAGGTCGTGGACC	CC1
SMC16.T.GATGCTAAGACAAGCC	CC2
SMC16.T.GATGCTAAGTCGTACT	CC2
SMC16.T.GATGCTAGTACAAGTA	CC1
SMC16.T.GATGCTAGTCCAGTGC	CC1
SMC16.T.GATGCTAGTTAAAGAC	CC1
SMC16.T.GATTCAGAGCGCTTAT	CC1
SMC16.T.GATTCAGCATTGTGCA	CC2
SMC16.T.GATTCAGGTAAGGATT	CC2
SMC16.T.GATTCAGGTCTAAACC	CC2
SMC16.T.GATTCAGGTGCTAGCC	CC2
SMC16.T.GATTCAGTCAGCTTAG	CC2
SMC16.T.GATTCAGTCATCTGTT	CC2
SMC16.T.GATTCAGTCGATGAGG	CC1
SMC16.T.GATTCAGTCTAAGCCA	CC2
SMC16.T.GCAAACTAGAAGATTC	CC2
SMC16.T.GCAAACTAGGGTCTCC	CC1
SMC16.T.GCAAACTCAGAGTGTG	CC1
SMC16.T.GCAAACTGTAAACCTC	CC1
SMC16.T.GCAAACTGTCTTCAAG	CC1
SMC16.T.GCAATCACATGCTAGT	CC2
SMC16.T.GCAATCAGTAAGGATT	CC2
SMC16.T.GCAATCAGTACCCAAT	CC2
SMC16.T.GCAATCAGTAGAGGAA	CC1
SMC16.T.GCAATCAGTGTTAAGA	CC2
SMC16.T.GCACATAAGCCACGTC	CC2
SMC16.T.GCACATACAAGCCCAC	CC1
SMC16.T.GCACATACAGACGTAG	CC2
SMC16.T.GCACATATCGCTGATA	CC2
SMC16.T.GCACATATCGGATGGA	CC1
SMC16.T.GCACTCTCAGAGTGTG	CC2
SMC16.T.GCACTCTCAGATGGGT	CC2
SMC16.T.GCACTCTGTCACACGC	CC2
SMC16.T.GCACTCTGTGGTCCGT	CC1
SMC16.T.GCACTCTTCGTTACGA	CC1
SMC16.T.GCAGCCACAAAGTGCG	CC2
SMC16.T.GCAGCCACAATAGCAA	CC2
SMC16.T.GCAGCCACAATGCCAT	CC1
SMC16.T.GCAGCCACACCCATGG	CC1
SMC16.T.GCAGCCACAGGACCCT	CC1
SMC16.T.GCAGCCACAGGATTGG	CC2
SMC16.T.GCAGCCACAGTCGATT	CC2

SMC16.T.GCAGCCAGTAACGTTC	CC1
SMC16.T.GCAGCCAGTCACTGGC	CC1
SMC16.T.GCAGCCAGTCCTGCTT	CC1
SMC16.T.GCAGCCATCACATACG	CC1
SMC16.T.GCAGCCATCTGAGGGA	CC2
SMC16.T.GCAGTTAAGTAGATGT	CC1
SMC16.T.GCAGTTAGTCACACGC	CC1
SMC16.T.GCAGTTATCCTAAGTG	CC2
SMC16.T.GCATACAAGTCGTACT	CC1
SMC16.T.GCATACACAATGAAAC	CC2
SMC16.T.GCATACACATTCTTAC	CC1
SMC16.T.GCATACAGTTCCGTCT	CC1
SMC16.T.GCATGATAGCCAGTTT	CC1
SMC16.T.GCATGATCAAGAGGCT	CC2
SMC16.T.GCATGATCACAACGCC	CC2
SMC16.T.GCATGATGTCTCGTTC	CC2
SMC16.T.GCATGATTCACAAACC	CC1
SMC16.T.GCATGATTCACTTATC	CC1
SMC16.T.GCATGCGAGAGTCTGG	CC1
SMC16.T.GCATGCGTCTCTGAGA	CC1
SMC16.T.GCATGTAAGCGATATA	CC1
SMC16.T.GCATGTACATCTGGTA	CC1
SMC16.T.GCATGTAGTCGCGGTT	CC2
SMC16.T.GCCAAATAGGCTCTTA	CC2
SMC16.T.GCCAAATCAGCTCCGA	CC2
SMC16.T.GCCAAATGTTACCGAT	CC2
SMC16.T.GCCAAATTCACTCCTG	CC2
SMC16.T.GCCAAATTCAGCGATT	CC2
SMC16.T.GCCTCTAAGCACAGGT	CC2
SMC16.T.GCCTCTACAAATCCGT	CC1
SMC16.T.GCCTCTACACGAAATA	CC1
SMC16.T.GCCTCTACAGACGCCT	CC2
SMC16.T.GCCTCTACAGCCTTTC	CC2
SMC16.T.GCCTCTACATGCCACG	CC1
SMC16.T.GCCTCTACATGGGAAC	CC1
SMC16.T.GCCTCTAGTGCCTGCA	CC2
SMC16.T.GCCTCTATCACCCTCA	CC2
SMC16.T.GCCTCTATCAGCTGGC	CC1
SMC16.T.GCGACCAAGGTGCACA	CC1
SMC16.T.GCGACCAAGTGCAAGC	CC2
SMC16.T.GCGACCACAGTCGTGC	CC1
SMC16.T.GCGACCACATCCTAGA	CC1
SMC16.T.GCGACCACATTCCTGC	CC1
SMC16.T.GCGACCAGTAGCGATG	CC1
SMC16.T.GCGACCAGTAGCTCCG	CC1
SMC16.T.GCGACCATCCGTTGCT	CC1
SMC16.T.GCGACCATCGCCTGAG	CC1
SMC16.T.GCGAGAAAGTCATGCT	CC2
SMC16.T.GCGAGAAAGTTATCGC	CC1
SMC16.T.GCGAGAACAGATCTGT	CC1
SMC16.T.GCGAGAACAGGAATCG	CC2
SMC16.T.GCGAGAAGTTGTACAC	CC2
SMC16.T.GCGAGAATCCCTCAGT	CC1
SMC16.T.GCGCAACAGGCGATAC	CC1
SMC16.T.GCGCAACAGGTGGGTT	CC2
SMC16.T.GCGCAACCAATAGAGT	CC1
SMC16.T.GCGCAACGTAAGTAGT	CC1
SMC16.T.GCGCAACTCAACGCTA	CC2

SMC16.T.GCGCAACTCAAGAAGT	CC1
SMC16.T.GCGCAGTAGACCTAGG	CC1
SMC16.T.GCGCAGTCACCGAATT	CC1
SMC16.T.GCGCAGTCAGCTTAAC	CC2
SMC16.T.GCGCAGTCATACTCTT	CC2
SMC16.T.GCGCAGTGTCTGATTG	CC2
SMC16.T.GCGCAGTGTTGTTTGG	CC1
SMC16.T.GCGCAGTTCCCAAGTA	CC2
SMC16.T.GCGCAGTTCCCTCAGT	CC1
SMC16.T.GCGCAGTTCCGTAGGC	CC2
SMC16.T.GCGCAGTTCGATGAGG	CC1
SMC16.T.GCGCAGTTCTCCGGTT	CC2
SMC16.T.GCGCCAAAGGCTCAGA	CC2
SMC16.T.GCGCCAAAGTCTCCTC	CC1
SMC16.T.GCGCCAACACACGCTG	CC1
SMC16.T.GCGCCAATCATCATTC	CC2
SMC16.T.GCGCGATAGCTAGCCC	CC2
SMC16.T.GCGCGATCAGCCTGTG	CC1
SMC16.T.GCGCGATGTGAGGGTT	CC2
SMC16.T.GCGCGATTCAGGTAAA	CC1
SMC16.T.GCGCGATTCCAGAAGG	CC1
SMC16.T.GCGGGTTAGATATGCA	CC2
SMC16.T.GCGGGTTGTCCGTCAG	CC1
SMC16.T.GCGGGTTGTGACCAAG	CC1
SMC16.T.GCGGGTTGTTAGGGTG	CC2
SMC16.T.GCGGGTTTCGAATGGG	CC2
SMC16.T.GCGGGTTTCGTACGGC	CC1
SMC16.T.GCTCCTAAGTTTCCTT	CC1
SMC16.T.GCTCCTACAAACCTAC	CC2
SMC16.T.GCTCCTACAGACTCGC	CC1
SMC16.T.GCTCCTAGTCTGCCAG	CC2
SMC16.T.GCTCCTAGTTCCAACA	CC2
SMC16.T.GCTCCTATCAGTTCGA	CC1
SMC16.T.GCTCCTATCGCCGTGA	CC2
SMC16.T.GCTCTGTAGGGTTTCT	CC1
SMC16.T.GCTCTGTCACCCTATC	CC1
SMC16.T.GCTCTGTCAGCTTAAC	CC2
SMC16.T.GCTCTGTCATGTTCCC	CC1
SMC16.T.GCTCTGTGTGGGTCAA	CC1
SMC16.T.GCTGCAGAGAAGGTGA	CC2
SMC16.T.GCTGCAGCAAAGCAAT	CC1
SMC16.T.GCTGCAGGTCGAATCT	CC1
SMC16.T.GCTGCGAAGAATGTGT	CC1
SMC16.T.GCTGCGATCGGCGCTA	CC2
SMC16.T.GCTGCTTAGGGCTTCC	CC2
SMC16.T.GCTGCTTAGTGATCGG	CC1
SMC16.T.GCTGCTTCAAACAACA	CC2
SMC16.T.GCTGCTTCATCACGAT	CC2
SMC16.T.GCTGCTTGTTACGCGC	CC2
SMC16.T.GCTGCTTTCAGATAAG	CC2
SMC16.T.GCTGCTTTCCTATGTT	CC2
SMC16.T.GCTGCTTTCGGCTACG	CC2
SMC16.T.GCTGCTTTCTCCGGTT	CC1
SMC16.T.GCTGGGTCACACGCTG	CC2
SMC16.T.GCTGGGTCACATCTTT	CC1
SMC16.T.GCTGGGTGTGCGCTTG	CC2
SMC16.T.GCTGGGTGTTATTCTC	CC2
SMC16.T.GCTGGGTTCCTAGGGC	CC2

SMC16.T.GCTTCCAAGGCATGTG	CC1
SMC16.T.GCTTCCACACGTGAGA	CC2
SMC16.T.GCTTCCACAGCTCGAC	CC2
SMC16.T.GCTTCCATCATATCGG	CC1
SMC16.T.GCTTGAAAGAAACGCC	CC1
SMC16.T.GCTTGAACATCGACGC	CC2
SMC16.T.GCTTGAAGTAGCAAAT	CC1
SMC16.T.GCTTGAAGTGACGCCT	CC2
SMC16.T.GGAAAGCAGGACCACA	CC1
SMC16.T.GGAAAGCAGGACGAAA	CC1
SMC16.T.GGAAAGCGTAGAGGAA	CC1
SMC16.T.GGAAAGCGTCGTCTTC	CC1
SMC16.T.GGAAAGCGTGCGCTTG	CC1
SMC16.T.GGAAAGCGTTTACTCT	CC2
SMC16.T.GGAAAGCTCTGCAAGT	CC1
SMC16.T.GGAACTTAGTTCGCGC	CC2
SMC16.T.GGAACTTCAAAGCAAT	CC1
SMC16.T.GGAACTTTCAATCTCT	CC2
SMC16.T.GGAATAAAGCCTTGAT	CC2
SMC16.T.GGAATAACATGCCTAA	CC2
SMC16.T.GGAATAAGTCCTGCTT	CC2
SMC16.T.GGAATAAGTTACGTCA	CC1
SMC16.T.GGAATAAGTTGAGTTC	CC2
SMC16.T.GGACAAGAGCGATAGC	CC1
SMC16.T.GGACAAGCATGCCTAA	CC2
SMC16.T.GGACAAGTCAGTTAGC	CC1
SMC16.T.GGACAGAAGTGTTGAA	CC1
SMC16.T.GGACAGACAAGGTGTG	CC2
SMC16.T.GGACAGAGTCTCTCTG	CC2
SMC16.T.GGACAGAGTGCCTGTG	CC2
SMC16.T.GGACAGAGTGTAACGG	CC2
SMC16.T.GGACATTAGGGATACC	CC1
SMC16.T.GGACATTAGGGTCTCC	CC2
SMC16.T.GGACATTAGTCCGGTC	CC2
SMC16.T.GGACATTAGTGAAGTT	CC1
SMC16.T.GGACATTCACTAAGTC	CC2
SMC16.T.GGACATTGTCGCGTGT	CC2
SMC16.T.GGACATTGTGCGCTTG	CC1
SMC16.T.GGACATTTCAGGCCCA	CC1
SMC16.T.GGACATTTCCTGTAGA	CC2
SMC16.T.GGACGTCAGCGATATA	CC2
SMC16.T.GGACGTCAGGTGCACA	CC2
SMC16.T.GGACGTCTCATCGCTC	CC1
SMC16.T.GGACGTCTCTGGCGAC	CC1
SMC16.T.GGAGCAACAGGACCCT	CC2
SMC16.T.GGAGCAAGTTCTGGTA	CC2
SMC16.T.GGAGCAATCAACACCA	CC2
SMC16.T.GGAGCAATCCAAGCCG	CC1
SMC16.T.GGAGCAATCCCGACTT	CC1
SMC16.T.GGAGCAATCCGCGGTA	CC1
SMC16.T.GGAGCAATCTTTAGTC	CC1
SMC16.T.GGATGTTAGAGTCTGG	CC2
SMC16.T.GGATGTTAGCTCCTTC	CC2
SMC16.T.GGATGTTCAATGAATG	CC2
SMC16.T.GGATGTTCACAACTGT	CC2
SMC16.T.GGATGTTCATGTTCCC	CC1
SMC16.T.GGATGTTGTCTGGAGA	CC2
SMC16.T.GGATGTTGTGGTTTCA	CC2

SMC16.T.GGATGTTGTTATCGGT	CC2
SMC16.T.GGATGTTTCTCCAGGG	CC1
SMC16.T.GGATTACAGCGTCTAT	CC1
SMC16.T.GGATTACGTCGGCACT	CC1
SMC16.T.GGATTACTCGCGTTTC	CC1
SMC16.T.GGCAATTAGACTGGGT	CC2
SMC16.T.GGCAATTAGGATGGAA	CC2
SMC16.T.GGCAATTCATAGACTC	CC2
SMC16.T.GGCAATTGTGTGTGCC	CC2
SMC16.T.GGCAATTGTTCGTCTC	CC2
SMC16.T.GGCAATTTCCTATTCA	CC1
SMC16.T.GGCAATTTCGAGAACG	CC1
SMC16.T.GGCAATTTCTTCAACT	CC1
SMC16.T.GGCCGATAGCCGATTT	CC1
SMC16.T.GGCCGATCAAGTCTAC	CC1
SMC16.T.GGCCGATCAAGTTCTG	CC2
SMC16.T.GGCCGATCAGTGGAGT	CC2
SMC16.T.GGCCGATGTCGCATCG	CC1
SMC16.T.GGCGACTAGAAAGTGG	CC1
SMC16.T.GGCGACTAGAATCTCC	CC2
SMC16.T.GGCGACTAGACAAAGG	CC2
SMC16.T.GGCGACTAGCCTATGT	CC2
SMC16.T.GGCGACTGTCCGAAGA	CC1
SMC16.T.GGCGACTGTCGTGGCT	CC1
SMC16.T.GGCGACTTCTAACTTC	CC2
SMC16.T.GGCGACTTCTGCCAGG	CC1
SMC16.T.GGCGTGTCACTACAGT	CC1
SMC16.T.GGCGTGTCAGCGTCCA	CC1
SMC16.T.GGCGTGTCAGTCCTTC	CC2
SMC16.T.GGCGTGTGTAAGAGGA	CC2
SMC16.T.GGCGTGTGTATAGGGC	CC2
SMC16.T.GGCTCGAAGCTAAGAT	CC2
SMC16.T.GGCTCGAAGTCTCAAC	CC2
SMC16.T.GGCTCGACACTATCTT	CC2
SMC16.T.GGCTCGACACTTAAGC	CC1
SMC16.T.GGCTCGACATCCGCGA	CC2
SMC16.T.GGCTCGACATCCTAGA	CC2
SMC16.T.GGCTCGAGTACAGACG	CC2
SMC16.T.GGCTCGAGTGAGCGAT	CC1
SMC16.T.GGCTCGAGTTACGGAG	CC1
SMC16.T.GGCTCGATCACAGGCC	CC1
SMC16.T.GGCTCGATCCTCTAGC	CC2
SMC16.T.GGCTCGATCGATAGAA	CC1
SMC16.T.GGCTCGATCGCAAACT	CC2
SMC16.T.GGCTGGTAGGGATCTG	CC1
SMC16.T.GGCTGGTCAAAGCAAT	CC2
SMC16.T.GGCTGGTCATTAGGCT	CC1
SMC16.T.GGCTGGTTCAACACAC	CC1
SMC16.T.GGGAATGAGAACAATC	CC2
SMC16.T.GGGAATGAGATCGATA	CC2
SMC16.T.GGGAATGGTCAGAAGC	CC1
SMC16.T.GGGAATGTCAGCGATT	CC1
SMC16.T.GGGAATGTCGCCAAAT	CC2
SMC16.T.GGGACCTAGGCTCTTA	CC2
SMC16.T.GGGACCTAGGTGTTAA	CC2
SMC16.T.GGGACCTCAGTGGAGT	CC2
SMC16.T.GGGACCTGTATAGGTA	CC2
SMC16.T.GGGACCTGTCCGAACC	CC2

SMC16.T.GGGACCTGTGTGAATA	CC2
SMC16.T.GGGACCTGTTGCGCAC	CC1
SMC16.T.GGGAGATAGAGTCTGG	CC2
SMC16.T.GGGAGATCACAGAGGT	CC1
SMC16.T.GGGAGATCATAGGATA	CC2
SMC16.T.GGGAGATGTTCAGACT	CC2
SMC16.T.GGGAGATTCACTATTC	CC1
SMC16.T.GGGAGATTCAGTTTGG	CC1
SMC16.T.GGGAGATTCGCATGAT	CC1
SMC16.T.GGGATGAAGTCAAGGC	CC2
SMC16.T.GGGATGACAATCTGCA	CC1
SMC16.T.GGGATGATCCAAGTAC	CC2
SMC16.T.GGGATGATCCCTAACC	CC2
SMC16.T.GGGATGATCCTCGCAT	CC2
SMC16.T.GGGATGATCGCGTAGC	CC1
SMC16.T.GGGATGATCTCGTATT	CC2
SMC16.T.GGGATGATCTTTAGGG	CC1
SMC16.T.GGGCACTAGACTCGGA	CC1
SMC16.T.GGGCACTAGCGTGAAC	CC1
SMC16.T.GGGCACTAGGGTTTCT	CC2
SMC16.T.GGGCACTAGGTGTTAA	CC2
SMC16.T.GGGCACTCACTCGACG	CC2
SMC16.T.GGGCACTCAGACACTT	CC2
SMC16.T.GGGCACTCAGGGCATA	CC1
SMC16.T.GGGCACTCATACTCTT	CC2
SMC16.T.GGGCACTGTGTGGCTC	CC2
SMC16.T.GGGCACTGTTCCACTC	CC2
SMC16.T.GGGCACTTCACTCCTG	CC1
SMC16.T.GGGCACTTCGGCTACG	CC1
SMC16.T.GGGCATCCACAACTGT	CC1
SMC16.T.GGGCATCCATGAAGTA	CC1
SMC16.T.GGGCATCTCAGTTCGA	CC2
SMC16.T.GGGCATCTCTTGTACT	CC2
SMC16.T.GGGTCTGAGGCGTACA	CC2
SMC16.T.GGGTCTGAGGCTCTTA	CC2
SMC16.T.GGGTCTGAGTGTACTC	CC2
SMC16.T.GGGTCTGGTCAATGTC	CC2
SMC16.T.GGGTCTGTCATTATCC	CC2
SMC16.T.GGGTCTGTCGAATGCT	CC1
SMC16.T.GGGTCTGTCTGAGTGT	CC1
SMC16.T.GGGTTGCAGTTAACGA	CC1
SMC16.T.GGGTTGCGTAGGGACT	CC1
SMC16.T.GGTATTGAGACTAAGT	CC1
SMC16.T.GGTATTGCAAAGAATC	CC2
SMC16.T.GGTATTGCACCGATAT	CC1
SMC16.T.GGTATTGCACCGCTAG	CC1
SMC16.T.GGTATTGCACGGTGTC	CC2
SMC16.T.GGTATTGGTAGCGCTC	CC1
SMC16.T.GGTATTGGTCGGCTCA	CC2
SMC16.T.GGTATTGTCAGAGCTT	CC1
SMC16.T.GGTATTGTCCTTTCGG	CC1
SMC16.T.GGTGAAGAGAGTGACC	CC2
SMC16.T.GGTGAAGAGCGTAATA	CC1
SMC16.T.GGTGAAGAGCTGTCTA	CC2
SMC16.T.GGTGAAGTCCTAGAAC	CC1
SMC16.T.GGTGCGTCACATCTTT	CC1
SMC16.T.GGTGCGTCATGTCTCC	CC2
SMC16.T.GGTGCGTGTCACCTAA	CC2

SMC16.T.GGTGCGTGTCGGCACT	CC2
SMC16.T.GGTGCGTGTTCGTCTC	CC1
SMC16.T.GGTGTTAAGACTACAA	CC1
SMC16.T.GGTGTTAAGTACGCCC	CC2
SMC16.T.GGTGTTACAAGCGCTC	CC2
SMC16.T.GGTGTTACACCACCAG	CC2
SMC16.T.GGTGTTACACGCGAAA	CC1
SMC16.T.GTAACGTAGTATCTCG	CC1
SMC16.T.GTAACGTCAGGCTCAC	CC2
SMC16.T.GTAACGTTCCGTACAA	CC2
SMC16.T.GTAACGTTCTCGTATT	CC1
SMC16.T.GTAACTGAGGGTGTGT	CC1
SMC16.T.GTAACTGCAGATCCAT	CC2
SMC16.T.GTAACTGCATTGCGGC	CC2
SMC16.T.GTAACTGGTACAGACG	CC2
SMC16.T.GTAACTGGTATAGGGC	CC2
SMC16.T.GTAACTGGTTAAGATG	CC1
SMC16.T.GTAACTGGTTGTACAC	CC2
SMC16.T.GTAACTGTCAACACTG	CC1
SMC16.T.GTACGTAAGCCAGTAG	CC1
SMC16.T.GTACGTAAGGTGTTAA	CC1
SMC16.T.GTACGTACAAACGCGA	CC2
SMC16.T.GTACGTACACAGGTTT	CC2
SMC16.T.GTACGTACACCTTGTC	CC1
SMC16.T.GTACGTACACTGAAGG	CC2
SMC16.T.GTACGTAGTGACGGTA	CC1
SMC16.T.GTACTCCCACACGCTG	CC1
SMC16.T.GTACTCCCACGTGAGA	CC1
SMC16.T.GTACTCCCAGGGATTG	CC2
SMC16.T.GTACTCCTCTAACTCT	CC2
SMC16.T.GTACTTTAGTGAATTG	CC1
SMC16.T.GTACTTTCACGAAGCA	CC2
SMC16.T.GTACTTTGTGGACGAT	CC2
SMC16.T.GTACTTTTCAACACAC	CC2
SMC16.T.GTAGGCCAGGCTCTTA	CC1
SMC16.T.GTAGGCCCACTTGGAT	CC1
SMC16.T.GTAGGCCGTCGGCATC	CC2
SMC16.T.GTAGGCCTCGCAAACT	CC2
SMC16.T.GTAGGCCTCGCCTGTT	CC2
SMC16.T.GTAGTCAAGAGTGAGA	CC1
SMC16.T.GTAGTCAGTTAGATGA	CC1
SMC16.T.GTAGTCAGTTTGGCGC	CC1
SMC16.T.GTAGTCATCAACGGCC	CC1
SMC16.T.GTAGTCATCCGATATG	CC2
SMC16.T.GTATCTTAGGCTCATT	CC2
SMC16.T.GTATCTTAGGTGCTAG	CC2
SMC16.T.GTATCTTCAGATCCAT	CC1
SMC16.T.GTATTCTAGTATCGAA	CC2
SMC16.T.GTATTCTTCCAGATCA	CC2
SMC16.T.GTATTCTTCGTCACGG	CC2
SMC16.T.GTCAAGTAGCTAGCCC	CC2
SMC16.T.GTCAAGTCACCTTGTC	CC2
SMC16.T.GTCAAGTCACTTACGA	CC2
SMC16.T.GTCAAGTCATCCGTGG	CC2
SMC16.T.GTCAAGTGTGATAAAC	CC2
SMC16.T.GTCACAATCATGTCTT	CC1
SMC16.T.GTCACAATCCACTGGG	CC1
SMC16.T.GTCACAATCGCAGGCT	CC1

SMC16.T.GTCACAATCTCGAGTA	CC1
SMC16.T.GTCACAATCTTGACGA	CC2
SMC16.T.GTCACGGAGAATTCCC	CC1
SMC16.T.GTCACGGAGCTGTTCA	CC1
SMC16.T.GTCACGGCAAGGACTG	CC2
SMC16.T.GTCACGGCACGTCAGC	CC2
SMC16.T.GTCACGGGTGTGTGCC	CC2
SMC16.T.GTCACGGGTGTTCTTT	CC2
SMC16.T.GTCACGGGTTGATTGC	CC2
SMC16.T.GTCATTTAGTATCGAA	CC2
SMC16.T.GTCATTTCACGTCAGC	CC2
SMC16.T.GTCATTTCACTGCCAG	CC2
SMC16.T.GTCATTTGTCTTCGTC	CC2
SMC16.T.GTCATTTGTTATGCGT	CC2
SMC16.T.GTCATTTTCCCTCTTT	CC2
SMC16.T.GTCATTTTCCTTGGTC	CC2
SMC16.T.GTCCTCAAGGAATCGC	CC2
SMC16.T.GTCCTCACAAACCTAC	CC1
SMC16.T.GTCCTCACACATTAGC	CC2
SMC16.T.GTCCTCACATTGGGCC	CC1
SMC16.T.GTCCTCAGTCCCTTGT	CC1
SMC16.T.GTCGGGTCAACGATCT	CC2
SMC16.T.GTCGGGTCATGCCTTC	CC2
SMC16.T.GTCGGGTTCACGATGT	CC1
SMC16.T.GTCGGGTTCCATGAGT	CC2
SMC16.T.GTCGTAAAGAAGGCCT	CC1
SMC16.T.GTCGTAAAGCTACCGC	CC1
SMC16.T.GTCGTAACACATCTTT	CC1
SMC16.T.GTCGTAACATAACCTG	CC2
SMC16.T.GTCGTAAGTCAGAGGT	CC2
SMC16.T.GTCGTAAGTGTGTGCC	CC1
SMC16.T.GTCGTAAGTGTTGAGG	CC1
SMC16.T.GTCTCGTAGGCCCTTG	CC1
SMC16.T.GTCTCGTCAATCTACG	CC1
SMC16.T.GTCTCGTCAATGGACG	CC1
SMC16.T.GTCTCGTCATCATCCC	CC2
SMC16.T.GTCTCGTTCCCTCAGT	CC2
SMC16.T.GTCTTCGAGAAGGTGA	CC2
SMC16.T.GTCTTCGAGCACCGTC	CC1
SMC16.T.GTCTTCGAGCTCTCGG	CC2
SMC16.T.GTCTTCGAGGCTCAGA	CC2
SMC16.T.GTCTTCGCAGTATAAG	CC2
SMC16.T.GTCTTCGCATACTACG	CC1
SMC16.T.GTCTTCGGTAAATGAC	CC2
SMC16.T.GTCTTCGGTACTTGAC	CC1
SMC16.T.GTCTTCGGTGAAATCA	CC2
SMC16.T.GTCTTCGGTGGACGAT	CC2
SMC16.T.GTCTTCGGTTCGCTAA	CC2
SMC16.T.GTGAAGGCAACACCTA	CC1
SMC16.T.GTGAAGGCAACTTGAC	CC1
SMC16.T.GTGAAGGCACCTGGTG	CC1
SMC16.T.GTGAAGGCAGAGCCAA	CC1
SMC16.T.GTGAAGGGTCATGCCG	CC2
SMC16.T.GTGAAGGTCACATGCA	CC1
SMC16.T.GTGAAGGTCTCCCTGA	CC2
SMC16.T.GTGCAGCAGGCCCTTG	CC1
SMC16.T.GTGCAGCCAAGTTCTG	CC2
SMC16.T.GTGCAGCCAGGTCCAC	CC2

SMC16.T.GTGCAGCGTCACACGC	CC2
SMC16.T.GTGCAGCTCTATCCTA	CC2
SMC16.T.GTGCATAAGAAGGACA	CC1
SMC16.T.GTGCATAAGAGGTTAT	CC2
SMC16.T.GTGCATAAGCGTTTAC	CC2
SMC16.T.GTGCATAAGTGCGTGA	CC1
SMC16.T.GTGCATAGTAGAAAGG	CC2
SMC16.T.GTGCATAGTAGGCATG	CC1
SMC16.T.GTGCATAGTCGCGGTT	CC2
SMC16.T.GTGCATAGTGATGTCT	CC1
SMC16.T.GTGCATATCCGTTGCT	CC1
SMC16.T.GTGCATATCGCTAGCG	CC1
SMC16.T.GTGCGGTAGCTCCCAG	CC2
SMC16.T.GTGCGGTAGGAGTAGA	CC1
SMC16.T.GTGCGGTAGGTAGCTG	CC1
SMC16.T.GTGCGGTCACAGCGTC	CC1
SMC16.T.GTGCGGTTCGCACTCT	CC1
SMC16.T.GTGCGGTTCTTGTATC	CC2
SMC16.T.GTGCGGTTCTTTAGTC	CC2
SMC16.T.GTGCTTCAGAGTCTGG	CC2
SMC16.T.GTGCTTCCAAAGGTGC	CC1
SMC16.T.GTGCTTCCATGAACCT	CC1
SMC16.T.GTGCTTCCATGCTGGC	CC2
SMC16.T.GTGCTTCGTGGCGAAT	CC2
SMC16.T.GTGGGTCAGCTAGTGG	CC2
SMC16.T.GTGGGTCCACATGACT	CC1
SMC16.T.GTGGGTCCATGTCGAT	CC1
SMC16.T.GTGGGTCGTGCACTTA	CC1
SMC16.T.GTGGGTCGTGGGTCAA	CC1
SMC16.T.GTGGGTCGTGTGCGTC	CC2
SMC16.T.GTGGGTCTCCCAAGAT	CC2
SMC16.T.GTGGGTCTCGCATGAT	CC2
SMC16.T.GTGTGCGAGCCCAACC	CC2
SMC16.T.GTGTGCGAGTGGGCTA	CC2
SMC16.T.GTGTGCGCAAGGTTTC	CC2
SMC16.T.GTGTGCGCACATAACC	CC2
SMC16.T.GTGTGCGCACTTACGA	CC1
SMC16.T.GTGTGCGCAGTTCCCT	CC2
SMC16.T.GTGTGCGGTACCGCTG	CC2
SMC16.T.GTGTGCGGTTGGTGGA	CC1
SMC16.T.GTGTGCGTCAGTTCGA	CC1
SMC16.T.GTGTGCGTCTTCGAGA	CC1
SMC16.T.GTGTTAGAGAGGACGG	CC1
SMC16.T.GTGTTAGAGGGTCTCC	CC2
SMC16.T.GTGTTAGCACGCTTTC	CC1
SMC16.T.GTGTTAGTCGAGGTAG	CC2
SMC16.T.GTTAAGCCACTCAGGC	CC1
SMC16.T.GTTAAGCGTACTCGCG	CC1
SMC16.T.GTTAAGCGTGAGGGTT	CC1
SMC16.T.GTTAAGCTCATGCATG	CC2
SMC16.T.GTTACAGAGGTGTTAA	CC1
SMC16.T.GTTACAGAGTTCGCGC	CC1
SMC16.T.GTTACAGCACGGCGTT	CC1
SMC16.T.GTTACAGGTGGTAACG	CC2
SMC16.T.GTTACAGGTTTAGGAA	CC2
SMC16.T.GTTACAGTCATACGGT	CC1
SMC16.T.GTTACAGTCCAAGTAC	CC1
SMC16.T.GTTACAGTCCCAGGTG	CC2

SMC16.T.GTTCATTAGTCTCCTC	CC1
SMC16.T.GTTCATTAGTGGTAAT	CC1
SMC16.T.GTTCATTCATGATCCA	CC1
SMC16.T.GTTCATTCATTGCGGC	CC1
SMC16.T.GTTCATTGTACCTACA	CC2
SMC16.T.GTTCGGGAGATGTTAG	CC1
SMC16.T.GTTCGGGCACGTCAGC	CC1
SMC16.T.GTTCGGGCAGCTTAAC	CC1
SMC16.T.GTTCGGGGTCTCTCTG	CC2
SMC16.T.GTTCTCGAGTTGTAGA	CC1
SMC16.T.GTTCTCGCAACTGGCC	CC1
SMC16.T.GTTCTCGCACGGTAGA	CC2
SMC16.T.GTTCTCGCATCACGAT	CC1
SMC16.T.GTTCTCGGTACGACCC	CC1
SMC16.T.GTTCTCGGTATATGGA	CC2
SMC16.T.GTTCTCGGTCAATACC	CC2
SMC16.T.GTTTCTAAGACACTAA	CC2
SMC16.T.GTTTCTAAGACGCAAC	CC1
SMC16.T.GTTTCTAAGGGTTTCT	CC2
SMC16.T.GTTTCTACACACATGT	CC2
SMC16.T.GTTTCTAGTTCACGGC	CC2
SMC16.T.GTTTCTATCTCTGAGA	CC2
SMC16.T.TAAACCGAGCCGGTAA	CC1
SMC16.T.TAAACCGAGCGTTGCC	CC2
SMC16.T.TAAACCGAGGATGCGT	CC1
SMC16.T.TAAACCGAGGCATGGT	CC1
SMC16.T.TAAACCGCAATGGAGC	CC1
SMC16.T.TAAACCGGTCCGTGAC	CC1
SMC16.T.TAAACCGGTCTACCTC	CC2
SMC16.T.TAAACCGGTCTGATTG	CC1
SMC16.T.TAAGAGACAGGATCGA	CC2
SMC16.T.TAAGAGACATACTCTT	CC1
SMC16.T.TAAGAGAGTTCCTCCA	CC2
SMC16.T.TAAGAGATCTCCGGTT	CC1
SMC16.T.TAAGCGTAGGTGTGGT	CC2
SMC16.T.TAAGCGTCAGATGGGT	CC2
SMC16.T.TAAGCGTGTAGCGTAG	CC2
SMC16.T.TAAGCGTGTCCGTGAC	CC2
SMC16.T.TAAGCGTGTCTCACCT	CC2
SMC16.T.TAAGCGTTCCAGAGGA	CC2
SMC16.T.TAAGTGCGTGCAGGTA	CC1
SMC16.T.TAAGTGCTCAGCTCGG	CC2
SMC16.T.TACACGACACGAGAGT	CC1
SMC16.T.TACACGAGTGTGCGTC	CC2
SMC16.T.TACAGTGAGGCACATG	CC1
SMC16.T.TACAGTGAGGCCGAAT	CC1
SMC16.T.TACAGTGAGTCAAGGC	CC1
SMC16.T.TACAGTGCAACACCTA	CC2
SMC16.T.TACAGTGCACGGTGTC	CC1
SMC16.T.TACAGTGCAGATGGCA	CC1
SMC16.T.TACAGTGGTTCGCGAC	CC1
SMC16.T.TACAGTGTCTAGAGTC	CC1
SMC16.T.TACAGTGTCTCGGACG	CC2
SMC16.T.TACAGTGTCTGAGGGA	CC1
SMC16.T.TACCTATAGCATGGCA	CC2
SMC16.T.TACCTATCATAAAGGT	CC2
SMC16.T.TACCTATGTAGAAGGA	CC2
SMC16.T.TACCTATGTTACTGAC	CC2

SMC16.T.TACCTATTCATGTCTT	CC1
SMC16.T.TACCTATTCGTCACGG	CC1
SMC16.T.TACCTTACACAACGTT	CC1
SMC16.T.TACCTTACATTCCTGC	CC1
SMC16.T.TACCTTAGTAGAGCTG	CC2
SMC16.T.TACCTTAGTAGAGTGC	CC2
SMC16.T.TACGGATAGGATGCGT	CC2
SMC16.T.TACGGATAGTCCCACG	CC1
SMC16.T.TACGGATTCAGCTCGG	CC2
SMC16.T.TACGGATTCAGTTGAC	CC1
SMC16.T.TACGGATTCGAGCCCA	CC2
SMC16.T.TACGGATTCGGTGTCG	CC2
SMC16.T.TACGGGCAGACCGGAT	CC2
SMC16.T.TACGGGCAGTGTTTGC	CC2
SMC16.T.TACGGGCCAAGCGCTC	CC2
SMC16.T.TACGGGCCACATCTTT	CC2
SMC16.T.TACGGGCCAGGAACGT	CC2
SMC16.T.TACGGGCTCATGCATG	CC2
SMC16.T.TACGGGCTCTAACCGA	CC2
SMC16.T.TACGGTAAGCAGCGTA	CC1
SMC16.T.TACGGTAAGGAATGGA	CC1
SMC16.T.TACGGTAAGGTGCTTT	CC1
SMC16.T.TACGGTACACATTCGA	CC2
SMC16.T.TACGGTAGTAGAAAGG	CC1
SMC16.T.TACGGTATCACTGGGC	CC2
SMC16.T.TACGGTATCCTATGTT	CC1
SMC16.T.TACGGTATCGGCTTGG	CC1
SMC16.T.TACGGTATCTTGTCAT	CC2
SMC16.T.TACGGTATCTTTCCTC	CC2
SMC16.T.TACTCATAGAAACGAG	CC2
SMC16.T.TACTCATCAATCTACG	CC2
SMC16.T.TACTCATCACGGCTAC	CC2
SMC16.T.TACTCATGTGAGGGAG	CC2
SMC16.T.TACTCATGTGATAAAC	CC1
SMC16.T.TACTCATGTTGCTCCT	CC2
SMC16.T.TACTCGCAGTGGGATC	CC2
SMC16.T.TACTCGCCAGTTAACC	CC2
SMC16.T.TACTCGCGTTGCGTTA	CC2
SMC16.T.TACTTACAGGCGCTCT	CC1
SMC16.T.TACTTACAGGCTCTTA	CC1
SMC16.T.TACTTACAGGGAGTAA	CC1
SMC16.T.TACTTACAGTTAGCGG	CC2
SMC16.T.TACTTACCACGTAAGG	CC2
SMC16.T.TACTTACTCCTATGTT	CC1
SMC16.T.TACTTACTCTCAAACG	CC1
SMC16.T.TACTTGTCAAGTTAAG	CC2
SMC16.T.TACTTGTCACTATCTT	CC2
SMC16.T.TACTTGTCAGATCTGT	CC1
SMC16.T.TACTTGTCAGTCAGCC	CC2
SMC16.T.TACTTGTTCGTTGACA	CC1
SMC16.T.TACTTGTTCGTTTATC	CC1
SMC16.T.TAGACCACACTGCCAG	CC2
SMC16.T.TAGACCACATGCCCGA	CC2
SMC16.T.TAGACCAGTACAGTGG	CC1
SMC16.T.TAGAGCTAGACATAAC	CC2
SMC16.T.TAGAGCTAGGTGTTAA	CC1
SMC16.T.TAGAGCTGTGTTGGGA	CC1
SMC16.T.TAGAGCTGTTATTCTC	CC1

SMC16.T.TAGAGCTTCACAACGT	CC2
SMC16.T.TAGAGCTTCCGAAGAG	CC2
SMC16.T.TAGAGCTTCCTTTCGG	CC1
SMC16.T.TAGCCGGAGAAGGTGA	CC1
SMC16.T.TAGCCGGCACAGTCGC	CC2
SMC16.T.TAGCCGGTCGTTTATC	CC1
SMC16.T.TAGGCATAGGCCCGTT	CC1
SMC16.T.TAGGCATCATGTAGTC	CC2
SMC16.T.TAGGCATGTAAGGGAA	CC2
SMC16.T.TAGGCATTCGCCTGAG	CC2
SMC16.T.TAGGCATTCTAACTCT	CC1
SMC16.T.TAGTGGTAGGATGCGT	CC1
SMC16.T.TAGTGGTCATCCCACT	CC2
SMC16.T.TAGTGGTCATCCTTGC	CC2
SMC16.T.TAGTTGGAGGTGCAAC	CC2
SMC16.T.TAGTTGGCAAGCCCAC	CC2
SMC16.T.TAGTTGGCAGGGTACA	CC2
SMC16.T.TAGTTGGGTCTCAACA	CC1
SMC16.T.TAGTTGGTCCCACTTG	CC1
SMC16.T.TATCAGGAGCGTAGTG	CC1
SMC16.T.TATCAGGCACTGAAGG	CC1
SMC16.T.TATCAGGTCCCATTAT	CC2
SMC16.T.TATCAGGTCCGAATGT	CC2
SMC16.T.TATCAGGTCTGGCGAC	CC2
SMC16.T.TATCAGGTCTTGTCAT	CC2
SMC16.T.TATCTCACAAACGTGG	CC2
SMC16.T.TATCTCATCTCGGACG	CC2
SMC16.T.TATGCCCAGCTAGTTC	CC2
SMC16.T.TATGCCCAGTTCGCGC	CC1
SMC16.T.TATGCCCCAGTTCCCT	CC2
SMC16.T.TATGCCCTCACATACG	CC1
SMC16.T.TATGCCCTCGGTGTTA	CC1
SMC16.T.TATTACCCAAACCCAT	CC1
SMC16.T.TATTACCCACGAAATA	CC2
SMC16.T.TATTACCCAGAGTGTG	CC2
SMC16.T.TATTACCCAGTACACT	CC2
SMC16.T.TATTACCGTTCCCGAG	CC2
SMC16.T.TATTACCTCAGCATGT	CC1
SMC16.T.TCAACGAAGAGCTATA	CC1
SMC16.T.TCAACGAAGTTAACGA	CC1
SMC16.T.TCAACGACAATCTACG	CC1
SMC16.T.TCAACGACATTCTTAC	CC2
SMC16.T.TCAACGAGTGATGTCT	CC2
SMC16.T.TCAACGATCCATGAAC	CC1
SMC16.T.TCAACGATCGGTTAAC	CC2
SMC16.T.TCAATCTAGTGTCCAT	CC2
SMC16.T.TCAATCTCACGGACAA	CC1
SMC16.T.TCAATCTGTCGCTTCT	CC1
SMC16.T.TCAATCTTCCAAACTG	CC2
SMC16.T.TCAATCTTCTCTGAGA	CC2
SMC16.T.TCAATCTTCTGACCTC	CC2
SMC16.T.TCACAAGAGGGTGTGT	CC1
SMC16.T.TCACAAGCACCTCGGA	CC2
SMC16.T.TCACAAGGTAGATTAG	CC2
SMC16.T.TCACAAGGTATGAATG	CC1
SMC16.T.TCACGAAAGTACTTGC	CC1
SMC16.T.TCACGAAGTACGCTGC	CC1
SMC16.T.TCACGAAGTCTTGCGG	CC2

SMC16.T.TCACGAATCCCTAATT	CC2
SMC16.T.TCACGAATCGCTGATA	CC2
SMC16.T.TCAGATGAGCCCAATT	CC1
SMC16.T.TCAGATGAGCGTTCCG	CC1
SMC16.T.TCAGATGCAGCTTCGG	CC2
SMC16.T.TCAGATGGTAGAGGAA	CC2
SMC16.T.TCAGATGTCTAGCACA	CC1
SMC16.T.TCAGCAAAGAAACGCC	CC2
SMC16.T.TCAGCAACAAAGCGGT	CC1
SMC16.T.TCAGCAACAGGGCATA	CC1
SMC16.T.TCAGCAAGTATGAAAC	CC1
SMC16.T.TCAGCAATCTTGGGTA	CC2
SMC16.T.TCAGCTCGTGGAAAGA	CC2
SMC16.T.TCAGCTCTCAGCGACC	CC1
SMC16.T.TCAGCTCTCATTATCC	CC1
SMC16.T.TCAGCTCTCGAGAACG	CC1
SMC16.T.TCAGCTCTCTAACTGG	CC2
SMC16.T.TCAGCTCTCTTTAGTC	CC2
SMC16.T.TCAGGATAGATCGATA	CC2
SMC16.T.TCAGGATCAAGTACCT	CC1
SMC16.T.TCAGGATGTACCGCTG	CC2
SMC16.T.TCAGGATGTATCAGTC	CC2
SMC16.T.TCAGGATGTGTTCTTT	CC2
SMC16.T.TCAGGATTCAGTGCAT	CC1
SMC16.T.TCAGGTACAATAGCAA	CC2
SMC16.T.TCAGGTACACGGTAAG	CC1
SMC16.T.TCAGGTACATCAGTCA	CC2
SMC16.T.TCAGGTAGTAAACGCG	CC2
SMC16.T.TCAGGTAGTCGCATCG	CC2
SMC16.T.TCAGGTAGTGCCTGCA	CC2
SMC16.T.TCAGGTAGTTAAGTAG	CC1
SMC16.T.TCAGGTAGTTGGTTTG	CC2
SMC16.T.TCAGGTATCAGTTCGA	CC2
SMC16.T.TCATTACAGTCTCGGC	CC1
SMC16.T.TCATTACGTTCGAATC	CC2
SMC16.T.TCATTACTCCTTGCCA	CC2
SMC16.T.TCATTTGAGTAGATGT	CC2
SMC16.T.TCATTTGCAGTCTTCC	CC2
SMC16.T.TCATTTGGTAATCGTC	CC2
SMC16.T.TCATTTGGTGTATGGG	CC2
SMC16.T.TCATTTGTCATCATTC	CC1
SMC16.T.TCATTTGTCGGCTTGG	CC1
SMC16.T.TCATTTGTCGTGGGAA	CC1
SMC16.T.TCATTTGTCTTGTTTG	CC1
SMC16.T.TCCACACAGACCTTTG	CC1
SMC16.T.TCCACACAGATGTGGC	CC2
SMC16.T.TCCACACAGCCAGTTT	CC2
SMC16.T.TCCACACAGGGCTTCC	CC1
SMC16.T.TCCACACAGGTGATAT	CC2
SMC16.T.TCCACACCAGACTCGC	CC2
SMC16.T.TCCACACTCATGCATG	CC2
SMC16.T.TCCACACTCCAGTATG	CC2
SMC16.T.TCCCGATAGTTAACGA	CC1
SMC16.T.TCCCGATCAAAGCAAT	CC2
SMC16.T.TCCCGATGTCACCTAA	CC1
SMC16.T.TCGAGGCAGCATGGCA	CC1
SMC16.T.TCGAGGCAGTCTCGGC	CC1
SMC16.T.TCGAGGCCAAGCGCTC	CC2

SMC16.T.TCGAGGCGTTGGAGGT	CC1
SMC16.T.TCGCGAGAGCGGCTTC	CC2
SMC16.T.TCGCGAGGTAGAAAGG	CC2
SMC16.T.TCGCGAGGTCACCCAG	CC1
SMC16.T.TCGCGAGGTCCATCCT	CC1
SMC16.T.TCGCGAGGTCTAACGT	CC1
SMC16.T.TCGCGAGGTGGAAAGA	CC1
SMC16.T.TCGCGTTCAATAACGA	CC1
SMC16.T.TCGGGACAGGAATGGA	CC1
SMC16.T.TCGGGACCAAACTGTC	CC2
SMC16.T.TCGGGACCATAGAAAC	CC1
SMC16.T.TCGGGACGTTGCGCAC	CC1
SMC16.T.TCGGGACTCAGAGCTT	CC1
SMC16.T.TCGGGACTCAGAGGTG	CC1
SMC16.T.TCGGGACTCTGTACGA	CC2
SMC16.T.TCGGTAAAGTAGTGCG	CC1
SMC16.T.TCGGTAAAGTTAGCGG	CC1
SMC16.T.TCGGTAACATAGACTC	CC2
SMC16.T.TCGTACCAGACTAAGT	CC1
SMC16.T.TCGTACCAGAGAACAG	CC2
SMC16.T.TCGTACCAGGCCCTTG	CC1
SMC16.T.TCGTACCCAAGAAAGG	CC1
SMC16.T.TCGTACCCACATAACC	CC1
SMC16.T.TCGTACCTCGGCGCAT	CC1
SMC16.T.TCGTACCTCGTTTAGG	CC2
SMC16.T.TCGTAGAAGAACTGTA	CC2
SMC16.T.TCGTAGAAGTCATCCA	CC2
SMC16.T.TCGTAGACATGCATGT	CC2
SMC16.T.TCGTAGAGTTCAACCA	CC1
SMC16.T.TCGTAGAGTTTGGCGC	CC2
SMC16.T.TCGTAGATCACCCGAG	CC2
SMC16.T.TCGTAGATCAGCGATT	CC1
SMC16.T.TCGTAGATCTTGAGGT	CC2
SMC16.T.TCTATTGCACCAACCG	CC1
SMC16.T.TCTATTGCACTGTCGG	CC2
SMC16.T.TCTATTGGTAAGGGAA	CC2
SMC16.T.TCTATTGTCTTAACCT	CC1
SMC16.T.TCTCATAAGCTCCCAG	CC1
SMC16.T.TCTCATACACCCAGTG	CC1
SMC16.T.TCTCATACACTGAAGG	CC1
SMC16.T.TCTCATAGTACAGTGG	CC2
SMC16.T.TCTCATAGTCAGATAA	CC1
SMC16.T.TCTCATATCTACTATC	CC2
SMC16.T.TCTCTAAAGACTAGAT	CC2
SMC16.T.TCTCTAACACAGTCGC	CC2
SMC16.T.TCTCTAACATGGAATA	CC2
SMC16.T.TCTCTAAGTTTGTGTG	CC2
SMC16.T.TCTCTAATCGTAGGTT	CC2
SMC16.T.TCTGAGAAGCGTGAGT	CC2
SMC16.T.TCTGAGACAATCGGTT	CC2
SMC16.T.TCTGAGACAATGGAGC	CC2
SMC16.T.TCTGAGACACAGCCCA	CC1
SMC16.T.TCTGAGACAGTTAACC	CC1
SMC16.T.TCTGAGACATGCTGGC	CC1
SMC16.T.TCTGAGATCCGCGCAA	CC1
SMC16.T.TCTGAGATCTTCGAGA	CC1
SMC16.T.TCTGGAACATATACCG	CC2
SMC16.T.TCTGGAACATCCGTGG	CC1

SMC16.T.TCTTCGGAGGTGTTAA	CC1
SMC16.T.TCTTCGGAGTGAACAT	CC2
SMC16.T.TCTTCGGCAAAGAATC	CC1
SMC16.T.TCTTCGGCATGGGACA	CC2
SMC16.T.TCTTCGGGTATAAACG	CC1
SMC16.T.TCTTCGGGTCTCAACA	CC2
SMC16.T.TCTTCGGGTGTTCGAT	CC2
SMC16.T.TCTTCGGTCGGCCGAT	CC2
SMC16.T.TCTTTCCGTTCCGTCT	CC2
SMC16.T.TCTTTCCGTTTCCACC	CC2
SMC16.T.TCTTTCCTCCTTGACC	CC2
SMC16.T.TCTTTCCTCTGAGTGT	CC2
SMC16.T.TGAAAGAAGACGACGT	CC2
SMC16.T.TGAAAGAAGCGCCTTG	CC2
SMC16.T.TGAAAGACACCGAAAG	CC2
SMC16.T.TGAAAGATCCCTAATT	CC1
SMC16.T.TGAAAGATCGGCGCAT	CC1
SMC16.T.TGACAACCACATCTTT	CC2
SMC16.T.TGACAACTCATGTCCC	CC2
SMC16.T.TGACAACTCTTAGAGC	CC2
SMC16.T.TGACGGCAGAAGAAGC	CC1
SMC16.T.TGACGGCCACACCGCA	CC2
SMC16.T.TGACGGCGTGCACGAA	CC2
SMC16.T.TGACGGCGTTAAGATG	CC2
SMC16.T.TGACGGCTCACGAAGG	CC2
SMC16.T.TGACGGCTCATTCACT	CC2
SMC16.T.TGACTAGAGTCACGCC	CC1
SMC16.T.TGACTAGCATATGCTG	CC2
SMC16.T.TGACTAGCATCGGACC	CC1
SMC16.T.TGACTAGGTACAGCAG	CC1
SMC16.T.TGACTAGTCATCATTC	CC2
SMC16.T.TGACTAGTCATTTGGG	CC2
SMC16.T.TGACTAGTCTTGTCAT	CC1
SMC16.T.TGACTTTAGAGGGATA	CC1
SMC16.T.TGACTTTAGCTGCCCA	CC1
SMC16.T.TGACTTTAGGTAGCTG	CC1
SMC16.T.TGACTTTCAGCATGAG	CC2
SMC16.T.TGACTTTGTATAAACG	CC1
SMC16.T.TGAGAGGAGCGACGTA	CC2
SMC16.T.TGAGAGGGTACAGCAG	CC2
SMC16.T.TGAGCATAGCACGCCT	CC2
SMC16.T.TGAGCATAGGTAGCCA	CC1
SMC16.T.TGAGCATCAGATGAGC	CC2
SMC16.T.TGAGCATGTCCGAACC	CC1
SMC16.T.TGAGCATGTGACGCCT	CC1
SMC16.T.TGAGCCGAGCGTAATA	CC1
SMC16.T.TGAGCCGCAGGATTGG	CC1
SMC16.T.TGAGCCGCAGTCCTTC	CC2
SMC16.T.TGAGCCGCATAGAAAC	CC2
SMC16.T.TGAGCCGGTAGCGTAG	CC1
SMC16.T.TGAGCCGGTATTCGTG	CC2
SMC16.T.TGAGCCGGTGCCTGTG	CC2
SMC16.T.TGAGGGAAGAGAGCTC	CC2
SMC16.T.TGAGGGACACTAGTAC	CC1
SMC16.T.TGAGGGACAGTGACAG	CC2
SMC16.T.TGAGGGACATCCAACA	CC2
SMC16.T.TGAGGGAGTCTCGTTC	CC2
SMC16.T.TGAGGGAGTGAGCGAT	CC1

SMC16.T.TGAGGGAGTGGTGTAG	CC1
SMC16.T.TGAGGGAGTTCTCATT	CC2
SMC16.T.TGAGGGATCTTGTTTG	CC1
SMC16.T.TGATTTCAGTCCTCCT	CC2
SMC16.T.TGATTTCGTTGAACTC	CC2
SMC16.T.TGCACCTCACGCTTTC	CC1
SMC16.T.TGCACCTGTGAGGGTT	CC2
SMC16.T.TGCACCTTCACCGGGT	CC1
SMC16.T.TGCACCTTCGCGCCAA	CC1
SMC16.T.TGCCAAAAGAAGGTGA	CC2
SMC16.T.TGCCAAAAGGCTAGCA	CC1
SMC16.T.TGCCAAAAGTTGTCGT	CC1
SMC16.T.TGCCAAACAAAGTGCG	CC2
SMC16.T.TGCCAAACAGCAGTTT	CC2
SMC16.T.TGCCAAAGTTACGACT	CC2
SMC16.T.TGCCCATAGGCGCTCT	CC1
SMC16.T.TGCCCATGTCAAGCGA	CC1
SMC16.T.TGCCCATTCCAAACAC	CC2
SMC16.T.TGCCCATTCCCTAACC	CC2
SMC16.T.TGCCCATTCTGATTCT	CC2
SMC16.T.TGCCCTACAACGATGG	CC2
SMC16.T.TGCCCTAGTAACGCGA	CC1
SMC16.T.TGCCCTAGTCCGAACC	CC1
SMC16.T.TGCCCTAGTCGTCTTC	CC1
SMC16.T.TGCCCTAGTTAGGGTG	CC2
SMC16.T.TGCCCTAGTTGAACTC	CC2
SMC16.T.TGCCCTATCAACACTG	CC2
SMC16.T.TGCCCTATCACTTACT	CC1
SMC16.T.TGCGCAGAGCCCAATT	CC2
SMC16.T.TGCGCAGCAAAGCAAT	CC1
SMC16.T.TGCGCAGCACCGAAAG	CC2
SMC16.T.TGCGCAGGTAGCTAAA	CC2
SMC16.T.TGCGCAGTCACTCTTA	CC2
SMC16.T.TGCGCAGTCCGATATG	CC1
SMC16.T.TGCGGGTCACATCCAA	CC2
SMC16.T.TGCGGGTCACGCGAAA	CC1
SMC16.T.TGCGGGTCAGCGTAAG	CC1
SMC16.T.TGCGGGTCATTGAGCT	CC1
SMC16.T.TGCGGGTGTATATCCG	CC2
SMC16.T.TGCGGGTGTTCGCGAC	CC2
SMC16.T.TGCGGGTGTTTGACAC	CC2
SMC16.T.TGCGGGTTCAAGCCTA	CC2
SMC16.T.TGCGTGGAGCTCAACT	CC1
SMC16.T.TGCGTGGCATGCTGGC	CC2
SMC16.T.TGCTACCAGATCGGGT	CC1
SMC16.T.TGCTACCCAGGTCGTC	CC2
SMC16.T.TGCTACCCATCACAAC	CC2
SMC16.T.TGCTACCGTCCAACTA	CC1
SMC16.T.TGCTGCTCAAAGGCGT	CC2
SMC16.T.TGCTGCTCATCACCCT	CC2
SMC16.T.TGCTGCTTCCGTACAA	CC2
SMC16.T.TGGACGCAGTGGAGAA	CC2
SMC16.T.TGGACGCGTGTTCTTT	CC2
SMC16.T.TGGACGCGTTAAGGGC	CC2
SMC16.T.TGGCCAGAGATCTGAA	CC1
SMC16.T.TGGCCAGCAAGCTGTT	CC2
SMC16.T.TGGCCAGCACGCCAGT	CC1
SMC16.T.TGGCCAGGTTATTCTC	CC2

SMC16.T.TGGCCAGGTTCCACTC	CC1
SMC16.T.TGGCCAGTCAGAGCTT	CC1
SMC16.T.TGGCCAGTCCGGCACA	CC1
SMC16.T.TGGCGCAAGAAGGGTA	CC2
SMC16.T.TGGCGCAAGATGTGTA	CC2
SMC16.T.TGGCGCACAGTCGTGC	CC2
SMC16.T.TGGCGCAGTGTCAATC	CC2
SMC16.T.TGGCGCAGTTTGTGTG	CC1
SMC16.T.TGGCGCATCAGTTGAC	CC2
SMC16.T.TGGCGCATCCGTAGGC	CC1
SMC16.T.TGGCGCATCCTCCTAG	CC2
SMC16.T.TGGCGCATCTTTAGTC	CC2
SMC16.T.TGGCTGGAGCCAGTTT	CC1
SMC16.T.TGGCTGGAGTAGCCGA	CC2
SMC16.T.TGGCTGGAGTTATCGC	CC1
SMC16.T.TGGCTGGCACTGCCAG	CC2
SMC16.T.TGGCTGGTCAAGGTAA	CC1
SMC16.T.TGGCTGGTCAGCCTAA	CC2
SMC16.T.TGGCTGGTCATGCAAC	CC2
SMC16.T.TGGGAAGAGCTGGAAC	CC2
SMC16.T.TGGGAAGGTATTAGCC	CC2
SMC16.T.TGGGAAGGTCTAGGTT	CC2
SMC16.T.TGGGAAGGTCTTGCGG	CC1
SMC16.T.TGGGCGTAGACAGACC	CC2
SMC16.T.TGGGCGTAGCAACGGT	CC2
SMC16.T.TGGGCGTCACGGCGTT	CC2
SMC16.T.TGGGCGTCAGTCAGAG	CC1
SMC16.T.TGGGCGTGTCCGAAGA	CC2
SMC16.T.TGGGCGTTCCTGCTTG	CC2
SMC16.T.TGGGCGTTCTTCGGTC	CC1
SMC16.T.TGGTTAGAGTTAGGTA	CC2
SMC16.T.TGGTTAGGTAATAGCA	CC2
SMC16.T.TGGTTAGTCATGTCCC	CC2
SMC16.T.TGGTTAGTCGCCAAAT	CC2
SMC16.T.TGGTTCCAGGGAAACA	CC2
SMC16.T.TGGTTCCCAGACGTAG	CC1
SMC16.T.TGGTTCCGTGGTCTCG	CC1
SMC16.T.TGGTTCCTCCGATATG	CC2
SMC16.T.TGGTTCCTCTTGGGTA	CC1
SMC16.T.TGTATTCAGATCGATA	CC2
SMC16.T.TGTATTCGTACTTCTT	CC2
SMC16.T.TGTCCCAAGACACGAC	CC2
SMC16.T.TGTCCCAAGGCTAGCA	CC2
SMC16.T.TGTCCCAGTAAACGCG	CC2
SMC16.T.TGTCCCAGTTACCGAT	CC1
SMC16.T.TGTCCCATCCCTCTTT	CC2
SMC16.T.TGTCCCATCCCTGACT	CC1
SMC16.T.TGTCCCATCGCACTCT	CC2
SMC16.T.TGTGGTAAGGGCATGT	CC2
SMC16.T.TGTGGTAAGTCATGCT	CC2
SMC16.T.TGTGGTATCCACGCAG	CC1
SMC16.T.TGTGGTATCCTGCTTG	CC1
SMC16.T.TGTGGTATCCTTTCTC	CC2
SMC16.T.TGTGTTTGTCCGACGT	CC2
SMC16.T.TGTGTTTTCCAACCAA	CC2
SMC16.T.TGTGTTTTCCAAGCCG	CC2
SMC16.T.TGTTCCGAGTCCGGTC	CC2
SMC16.T.TGTTCCGAGTGTGAAT	CC2

SMC16.T.TGTTCCGCACCACCAG	CC2
SMC16.T.TGTTCCGCATTTCACT	CC2
SMC16.T.TGTTCCGGTCGGCTCA	CC2
SMC16.T.TTAACTCAGATCCTGT	CC1
SMC16.T.TTAACTCAGTTTAGGA	CC1
SMC16.T.TTAACTCCACAACTGT	CC1
SMC16.T.TTAACTCCAGTAAGCG	CC1
SMC16.T.TTAACTCTCCTGCTTG	CC2
SMC16.T.TTAACTCTCTAAGCCA	CC2
SMC16.T.TTAGGACAGACAGGCT	CC2
SMC16.T.TTAGGACCACGGATAG	CC1
SMC16.T.TTAGGACGTACTTAGC	CC2
SMC16.T.TTAGGACGTAGCTCCG	CC2
SMC16.T.TTAGGACGTCTGGAGA	CC2
SMC16.T.TTAGGCAAGGCATGTG	CC2
SMC16.T.TTAGGCACAAGACGTG	CC2
SMC16.T.TTAGGCACAGGTCTCG	CC1
SMC16.T.TTAGGCACATCTCCCA	CC1
SMC16.T.TTAGGCAGTCGAGTTT	CC2
SMC16.T.TTAGGCAGTCGCATCG	CC2
SMC16.T.TTAGGCATCAACGGCC	CC2
SMC16.T.TTAGGCATCACGAAGG	CC2
SMC16.T.TTAGGCATCAGTCCCT	CC2
SMC16.T.TTAGGCATCCTTCAAT	CC2
SMC16.T.TTAGTTCAGCTAGTGG	CC1
SMC16.T.TTAGTTCGTCCGAACC	CC2
SMC16.T.TTAGTTCTCAGGATCT	CC2
SMC16.T.TTAGTTCTCGAATGCT	CC2
SMC16.T.TTATGCTAGAGCTGCA	CC2
SMC16.T.TTATGCTAGGGATCTG	CC1
SMC16.T.TTCCCAGCAATGGATA	CC1
SMC16.T.TTCCCAGCAGATGGCA	CC2
SMC16.T.TTCCCAGGTACAGTTC	CC2
SMC16.T.TTCCCAGGTACGAAAT	CC1
SMC16.T.TTCCCAGGTATGCTTG	CC1
SMC16.T.TTCCCAGGTGTGACGA	CC2
SMC16.T.TTCCCAGTCTCCGGTT	CC1
SMC16.T.TTCCCAGTCTGGAGCC	CC1
SMC16.T.TTCGAAGAGGGAACGG	CC2
SMC16.T.TTCGAAGAGGGATACC	CC2
SMC16.T.TTCGAAGCACAAGTAA	CC2
SMC16.T.TTCGAAGCATGCATGT	CC1
SMC16.T.TTCGAAGCATTTCAGG	CC1
SMC16.T.TTCGAAGGTAAGTGGC	CC2
SMC16.T.TTCGAAGGTTGAGTTC	CC2
SMC16.T.TTCGAAGTCACCATAG	CC1
SMC16.T.TTCGAAGTCAGGTTCA	CC1
SMC16.T.TTCGAAGTCATCGGAT	CC1
SMC16.T.TTCGGTCAGGATATAC	CC1
SMC16.T.TTCGGTCCAAAGGCGT	CC2
SMC16.T.TTCGGTCCAACTGGCC	CC1
SMC16.T.TTCGGTCCAAGCCCAC	CC2
SMC16.T.TTCGGTCGTGTGGTTT	CC1
SMC16.T.TTCTACAAGCGTTGCC	CC2
SMC16.T.TTCTACAAGCTTTGGT	CC2
SMC16.T.TTCTACAAGTAGTGCG	CC2
SMC16.T.TTCTACACAGTCTTCC	CC2
SMC16.T.TTCTACATCCTAGAAC	CC1

SMC16.T.TTCTACATCCTCGCAT	CC2
SMC16.T.TTCTACATCGATCCCT	CC2
SMC16.T.TTCTCAAAGAACAACT	CC2
SMC16.T.TTCTCAAAGAGCTTCT	CC2
SMC16.T.TTCTCAACAATGAATG	CC2
SMC16.T.TTCTCAACAGAAGCAC	CC2
SMC16.T.TTCTCAACATTCACTT	CC2
SMC16.T.TTCTCAAGTCAAGCGA	CC2
SMC16.T.TTCTCAATCAGCGACC	CC2
SMC16.T.TTCTCAATCCGCGTTT	CC1
SMC16.T.TTCTCAATCGGAGGTA	CC2
SMC16.T.TTCTCAATCTTGTCAT	CC2
SMC16.T.TTCTCCTAGATGCGAC	CC1
SMC16.T.TTCTCCTAGGCATTGG	CC1
SMC16.T.TTCTCCTCAAGAAGAG	CC1
SMC16.T.TTCTCCTCACCCAGTG	CC1
SMC16.T.TTCTCCTTCCCAGGTG	CC1
SMC16.T.TTCTTAGCAGCCACCA	CC2
SMC16.T.TTCTTAGTCAACACAC	CC2
SMC16.T.TTCTTAGTCACAACGT	CC2
SMC16.T.TTCTTAGTCTCGTATT	CC1
SMC16.T.TTGAACGCAATACGCT	CC1
SMC16.T.TTGAACGTCTAGCACA	CC2
SMC16.T.TTGAACGTCTCTGTCG	CC2
SMC16.T.TTGACTTAGCGGATCA	CC1
SMC16.T.TTGACTTAGTGTCCCG	CC2
SMC16.T.TTGACTTCATGCAACT	CC1
SMC16.T.TTGACTTTCGTAGGTT	CC2
SMC16.T.TTGCCGTAGAGCTGGT	CC1
SMC16.T.TTGCCGTAGCACGCCT	CC2
SMC16.T.TTGCCGTCAACTGGCC	CC1
SMC16.T.TTGCCGTCAAGAAGAG	CC2
SMC16.T.TTGCCGTCAAGCGAGT	CC1
SMC16.T.TTGCCGTCAATGTAAG	CC2
SMC16.T.TTGCCGTCACACCGAC	CC1
SMC16.T.TTGCCGTGTCACTTCC	CC2
SMC16.T.TTGCCGTGTTACAGAA	CC2
SMC16.T.TTGCGTCAGAGAGCTC	CC2
SMC16.T.TTGCGTCAGCGAGAAA	CC2
SMC16.T.TTGCGTCCAAGAGGCT	CC2
SMC16.T.TTGCGTCCAAGCCGCT	CC2
SMC16.T.TTGCGTCCACACCGCA	CC1
SMC16.T.TTGCGTCCACTACAGT	CC1
SMC16.T.TTGCGTCCAGCATACT	CC2
SMC16.T.TTGCGTCCAGTGACAG	CC1
SMC16.T.TTGCGTCGTAGCGATG	CC2
SMC16.T.TTGCGTCGTTGTTTGG	CC2
SMC16.T.TTGGAACCAGTCGTGC	CC1
SMC16.T.TTGGAACGTGCAGGTA	CC2
SMC16.T.TTGGAACGTTCGAATC	CC1
SMC16.T.TTGGAACTCGAATGGG	CC2
SMC16.T.TTGGAACTCTAACGGT	CC2
SMC16.T.TTGGCAAAGTGGACGT	CC2
SMC16.T.TTGGCAACATGCGCAC	CC1
SMC16.T.TTGGCAAGTATATGAG	CC1
SMC16.T.TTGGCAAGTCTCTTTA	CC1
SMC16.T.TTGGCAAGTGTTTGGT	CC1
SMC16.T.TTGGCAATCATCACCC	CC1

SMC16.T.TTGGCAATCGGCGGTT	CC2
SMC16.T.TTGTAGGAGAATAGGG	CC1
SMC16.T.TTGTAGGCAAACAACA	CC2
SMC16.T.TTGTAGGCAATGGATA	CC2
SMC16.T.TTGTAGGGTCAGAAGC	CC1
SMC16.T.TTGTAGGGTCTTTCAT	CC2
SMC16.T.TTGTAGGGTTTAGCTG	CC2
SMC16.T.TTGTAGGTCCTTCAAT	CC2
SMC16.T.TTGTAGGTCGTCTGAA	CC2
SMC16.T.TTTACTGAGATGTGTA	CC1
SMC16.T.TTTACTGCACAAGACG	CC1
SMC16.T.TTTACTGGTGCTGTAT	CC1
SMC16.T.TTTACTGGTGGTCCGT	CC2
SMC16.T.TTTACTGGTGTGGTTT	CC1
SMC16.T.TTTATGCGTAAAGTCA	CC1
SMC16.T.TTTCCTCAGGCTATCT	CC1
SMC16.T.TTTCCTCAGGTGCTTT	CC2
SMC16.T.TTTCCTCAGTGCAAGC	CC1
SMC16.T.TTTCCTCCAGTCAGAG	CC1
SMC16.T.TTTCCTCGTGCAGTAG	CC2
SMC16.T.TTTCCTCTCATGTCTT	CC1
SMC16.T.TTTCCTCTCCGCATAA	CC2
SMC16.T.TTTGCGCTCAGATAAG	CC1
SMC16.T.TTTGCGCTCTCCCTGA	CC1
SMC16.T.TTTGGTTAGTGTTAGA	CC1
SMC16.T.TTTGGTTCAGGACCCT	CC2
SMC16.T.TTTGGTTCATGGTAGG	CC2
SMC16.T.TTTGGTTTCCAGAGGA	CC1
SMC16.T.TTTGGTTTCGTAGATC	CC2
SMC16.T.TTTGTCAGTACTCGCG	CC2
SMC16.T.TTTGTCATCTTTAGTC	CC2
SMC17.T.AAACCTGTCTCTGAGA	CC2
SMC17.T.AAAGATGGTACCGAGA	CC2
SMC17.T.AAAGCAAGTTATGCGT	CC2
SMC17.T.AAAGTAGCAGTAACGG	CC2
SMC17.T.AACACGTCATCCGGGT	CC2
SMC17.T.AACACGTTCCCTAATT	CC2
SMC17.T.AACCATGGTCGACTAT	CC2
SMC17.T.AACCATGTCCAAGTAC	CC2
SMC17.T.AACGTTGGTCCGTCAG	CC2
SMC17.T.AACTCTTTCTGCTGCT	CC1
SMC17.T.AACTGGTGTCTGGAGA	CC2
SMC17.T.AAGGAGCAGGCTAGCA	CC2
SMC17.T.AAGGAGCCATTAGGCT	CC2
SMC17.T.AAGGAGCGTCTGCCAG	CC2
SMC17.T.AAGGCAGTCCTATGTT	CC2
SMC17.T.AAGGTTCCAAGTCTGT	CC2
SMC17.T.AAGGTTCGTGGCAAAC	CC2
SMC17.T.AATCCAGAGAGCCCAA	CC2
SMC17.T.AATCGGTCAATGGAAT	CC2
SMC17.T.AATCGGTCAGGGTTAG	CC2
SMC17.T.ACACCCTTCATCGATG	CC2
SMC17.T.ACACCGGAGTATGACA	CC2
SMC17.T.ACAGCCGCACGAAACG	CC2
SMC17.T.ACAGCCGCAGCCTTGG	CC2
SMC17.T.ACAGCCGGTCCTCCAT	CC2
SMC17.T.ACAGCTACATCCGCGA	CC2
SMC17.T.ACATACGGTCTACCTC	CC2

SMC17.T.ACATCAGCACAGACTT	CC2
SMC17.T.ACATCAGCAGCTTCGG	CC2
SMC17.T.ACATCAGGTAGCGATG	CC2
SMC17.T.ACATCAGTCGCCATAA	CC2
SMC17.T.ACCAGTAGTCATATCG	CC2
SMC17.T.ACCAGTATCCACGTGG	CC2
SMC17.T.ACCCACTCATCTATGG	CC2
SMC17.T.ACCGTAATCCAACCAA	CC2
SMC17.T.ACCGTAATCTCTTATG	CC2
SMC17.T.ACCTTTATCTTGTATC	CC2
SMC17.T.ACGATACCAGGTTTCA	CC2
SMC17.T.ACGATGTAGATGCGAC	CC2
SMC17.T.ACGATGTGTATAAACG	CC2
SMC17.T.ACGCCAGTCCGTCATC	CC2
SMC17.T.ACGCCAGTCTCTTATG	CC2
SMC17.T.ACGCCGACAGGACCCT	CC2
SMC17.T.ACGCCGAGTGTGCCTG	CC2
SMC17.T.ACGGAGAGTCTGGTCG	CC2
SMC17.T.ACGGCCAAGAAGGGTA	CC2
SMC17.T.ACGGCCAGTACCGAGA	CC2
SMC17.T.ACGGGTCCACCATGTA	CC2
SMC17.T.ACGTCAACAAAGAATC	CC2
SMC17.T.ACTATCTCAACACCTA	CC2
SMC17.T.ACTATCTTCTGTGCAA	CC2
SMC17.T.ACTGAACCAGGATCGA	CC2
SMC17.T.ACTGAGTCAAGCTGAG	CC2
SMC17.T.ACTGAGTCACCGGAAA	CC2
SMC17.T.ACTGAGTTCTCGCATC	CC2
SMC17.T.ACTGATGCAAGAAGAG	CC2
SMC17.T.ACTGTCCTCCATGAGT	CC2
SMC17.T.ACTTACTAGGAGTTTA	CC2
SMC17.T.ACTTACTCAGATCCAT	CC2
SMC17.T.ACTTACTGTTAGATGA	CC2
SMC17.T.ACTTACTTCTACTATC	CC2
SMC17.T.ACTTTCAGTGTTTGTG	CC2
SMC17.T.AGAATAGCAGGTTTCA	CC2
SMC17.T.AGACGTTAGGTGCTAG	CC2
SMC17.T.AGACGTTCACATGACT	CC2
SMC17.T.AGACGTTGTTGGTTTG	CC2
SMC17.T.AGAGCGACAGTAGAGC	CC2
SMC17.T.AGAGTGGCACCGAATT	CC2
SMC17.T.AGATCTGAGCGTGAGT	CC2
SMC17.T.AGATTGCAGTCTTGCA	CC2
SMC17.T.AGCATACAGACTTGAA	CC2
SMC17.T.AGCGTATTCCGTAGTA	CC2
SMC17.T.AGCTCCTAGCTGAACG	CC2
SMC17.T.AGCTCCTTCTTTAGTC	CC2
SMC17.T.AGGCCGTCAGCTTCGG	CC2
SMC17.T.AGGGAGTCAAACGCGA	CC2
SMC17.T.AGGGAGTTCGAGAGCA	CC2
SMC17.T.AGGTCATGTCACCCAG	CC2
SMC17.T.AGGTCCGGTCTCCATC	CC2
SMC17.T.AGTCTTTAGCTGCAAG	CC2
SMC17.T.AGTCTTTCAAGAGTCG	CC2
SMC17.T.AGTGAGGAGGAGTCTG	CC2
SMC17.T.AGTGAGGCAACCGCCA	CC2
SMC17.T.AGTGAGGCAGACTCGC	CC2
SMC17.T.AGTGAGGTCAAGCCTA	CC2

SMC17.T.AGTGAGGTCGTGGGAA	CC2
SMC17.T.ATAAGAGCAGGCGATA	CC2
SMC17.T.ATAGACCGTACATCCA	CC2
SMC17.T.ATCACGATCTTGGGTA	CC2
SMC17.T.ATCATCTCACCAACCG	CC2
SMC17.T.ATCATCTCACGCGAAA	CC2
SMC17.T.ATCCGAACACTTAACG	CC2
SMC17.T.ATCCGAAGTACTCAAC	CC2
SMC17.T.ATCTACTCAATAGCGG	CC2
SMC17.T.ATCTACTGTCAAAGAT	CC2
SMC17.T.ATCTGCCAGTGTTGAA	CC2
SMC17.T.ATCTGCCTCTCGCATC	CC2
SMC17.T.ATGCGATTCACTCCTG	CC2
SMC17.T.ATGCGATTCCGCATCT	CC2
SMC17.T.ATGGGAGCAAGCTGTT	CC2
SMC17.T.ATGGGAGGTCCTCTTG	CC2
SMC17.T.ATTACTCCAGAGTGTG	CC2
SMC17.T.ATTACTCTCTGCTGTC	CC2
SMC17.T.ATTGGACGTAGCGCAA	CC2
SMC17.T.CAACCAAAGAGCTATA	CC2
SMC17.T.CAACCTCAGAGCTTCT	CC2
SMC17.T.CAACCTCCATAACCTG	CC2
SMC17.T.CAACTAGGTACCGTAT	CC2
SMC17.T.CAAGGCCAGTGCGATG	CC2
SMC17.T.CAAGTTGTCTGGTATG	CC2
SMC17.T.CACACAAAGACTGGGT	CC2
SMC17.T.CACACAACAATCTACG	CC2
SMC17.T.CACACAATCCCTAATT	CC2
SMC17.T.CACACAATCTTTACAC	CC2
SMC17.T.CACACTCTCCTACAGA	CC2
SMC17.T.CACAGGCCACGAAAGC	CC2
SMC17.T.CACATAGAGAACTGTA	CC2
SMC17.T.CACATAGAGTACTTGC	CC2
SMC17.T.CACATTTAGGTAGCCA	CC2
SMC17.T.CACCACTTCACCCGAG	CC2
SMC17.T.CACCACTTCTGCAAGT	CC2
SMC17.T.CACCACTTCTGCGTAA	CC2
SMC17.T.CACCAGGCAATGACCT	CC2
SMC17.T.CACCTTGCAGTATAAG	CC2
SMC17.T.CACTCCAAGAGCCTAG	CC2
SMC17.T.CAGAATCCAAGCGCTC	CC2
SMC17.T.CAGATCAGTTACCGAT	CC2
SMC17.T.CAGCAGCCATGTCCTC	CC2
SMC17.T.CAGCAGCGTACGAAAT	CC2
SMC17.T.CAGCATAAGTTTAGGA	CC2
SMC17.T.CAGCATAGTTGGAGGT	CC2
SMC17.T.CAGCCGAGTTTGACAC	CC2
SMC17.T.CAGCCGATCAATCTCT	CC2
SMC17.T.CAGCCGATCGTGTAGT	CC2
SMC17.T.CAGCTAAAGGATGGAA	CC2
SMC17.T.CAGGTGCCAGTACACT	CC2
SMC17.T.CAGGTGCGTTGGTGGA	CC2
SMC17.T.CAGTAACCAAATTGCC	CC2
SMC17.T.CAGTAACCACCTATCC	CC2
SMC17.T.CAGTAACGTTGCGCAC	CC2
SMC17.T.CAGTCCTAGCTCCTCT	CC2
SMC17.T.CAGTCCTTCTGTCTCG	CC2
SMC17.T.CATATGGAGAGGTAGA	CC2

SMC17.T.CATATGGCAGCGTCCA	CC2
SMC17.T.CATATGGTCCGTAGGC	CC2
SMC17.T.CATATTCGTGTTCGAT	CC2
SMC17.T.CATCAAGGTCGAATCT	CC2
SMC17.T.CATCAAGGTGGAAAGA	CC2
SMC17.T.CATCAAGTCACGAAGG	CC2
SMC17.T.CATCAGACAGCGTCCA	CC2
SMC17.T.CATCGAACAAAGTGCG	CC2
SMC17.T.CATCGAACATGGTCAT	CC2
SMC17.T.CATGACAAGTGACTCT	CC2
SMC17.T.CATGCCTAGGTTCCTA	CC2
SMC17.T.CATTATCCACTTAACG	CC2
SMC17.T.CCAATCCAGAACAACT	CC2
SMC17.T.CCAATCCGTCCTCCAT	CC2
SMC17.T.CCAATCCGTGCACTTA	CC2
SMC17.T.CCACCTACAGCCACCA	CC2
SMC17.T.CCACGGACAAAGTGCG	CC2
SMC17.T.CCACTACAGGGAAACA	CC2
SMC17.T.CCACTACCATTAACCG	CC2
SMC17.T.CCACTACTCGGACAAG	CC2
SMC17.T.CCAGCGAAGTACTTGC	CC2
SMC17.T.CCATTCGAGAGCCCAA	CC2
SMC17.T.CCATTCGGTCCTAGCG	CC2
SMC17.T.CCCAATCGTACCTACA	CC2
SMC17.T.CCCTCCTCAATGGATA	CC2
SMC17.T.CCCTCCTCACTTGGAT	CC2
SMC17.T.CCCTCCTCAGCTTAAC	CC2
SMC17.T.CCCTCCTCATTACCTT	CC2
SMC17.T.CCCTCCTGTCTCTTAT	CC2
SMC17.T.CCGGGATCAACGATGG	CC2
SMC17.T.CCGGGATGTCATGCCG	CC2
SMC17.T.CCGGGATTCATGTAGC	CC2
SMC17.T.CCGGTAGCATCCCACT	CC2
SMC17.T.CCGGTAGTCATCGGAT	CC2
SMC17.T.CCGTACTCAACACCTA	CC2
SMC17.T.CCGTACTCATGCCACG	CC2
SMC17.T.CCGTGGAGTACCGTTA	CC2
SMC17.T.CCTACACAGCTGATAA	CC2
SMC17.T.CCTAGCTGTTCGCTAA	CC2
SMC17.T.CCTCTGAGTGACTACT	CC2
SMC17.T.CCTTACGCATTGAGCT	CC2
SMC17.T.CCTTACGGTTATCACG	CC2
SMC17.T.CCTTCCCAGATCGGGT	CC2
SMC17.T.CCTTTCTAGTGTTTGC	CC2
SMC17.T.CGAACATAGGTGCTAG	CC2
SMC17.T.CGAACATCACCCATTC	CC2
SMC17.T.CGAATGTCAATAGCAA	CC2
SMC17.T.CGACCTTTCCTTTCTC	CC2
SMC17.T.CGACTTCCATTCCTCG	CC2
SMC17.T.CGAGAAGAGTACGTTC	CC2
SMC17.T.CGAGAAGTCGTACCGG	CC2
SMC17.T.CGAGCACAGGTGATAT	CC2
SMC17.T.CGAGCACGTACGAAAT	CC2
SMC17.T.CGAGCCAAGTGGTCCC	CC2
SMC17.T.CGATCGGCAGTAAGAT	CC2
SMC17.T.CGATGTACATCCAACA	CC2
SMC17.T.CGATGTAGTCGTCTTC	CC1
SMC17.T.CGATTGACAGAGTGTG	CC2

SMC17.T.CGCCAAGGTAGATTAG	CC2
SMC17.T.CGCGGTACAAGCCGTC	CC2
SMC17.T.CGCGGTACATTTCACT	CC2
SMC17.T.CGCGTTTTCAAGAAGT	CC2
SMC17.T.CGCTATCCACCCTATC	CC2
SMC17.T.CGCTGGATCCTGCCAT	CC2
SMC17.T.CGCTTCAGTCGGGTCT	CC2
SMC17.T.CGGACACAGGGTTCCC	CC2
SMC17.T.CGGACACTCAAGGTAA	CC2
SMC17.T.CGGACACTCCCTGACT	CC2
SMC17.T.CGGACGTCACATTAGC	CC2
SMC17.T.CGGAGCTCAAAGCAAT	CC2
SMC17.T.CGGAGCTGTGATAAGT	CC2
SMC17.T.CGGAGTCAGCCAGGAT	CC2
SMC17.T.CGGCTAGGTTATCACG	CC2
SMC17.T.CGGCTAGGTTCACGGC	CC2
SMC17.T.CGGGTCACATTTGCCC	CC2
SMC17.T.CGGTTAAAGAAACCGC	CC2
SMC17.T.CGTCAGGAGACAAAGG	CC2
SMC17.T.CGTCAGGGTCTCATCC	CC2
SMC17.T.CGTCAGGGTTCCATGA	CC2
SMC17.T.CGTCAGGTCTGTTTGT	CC2
SMC17.T.CGTCCATGTAGAAAGG	CC2
SMC17.T.CGTGAGCAGAGGGCTT	CC1
SMC17.T.CGTGAGCAGTGTTAGA	CC2
SMC17.T.CGTGTCTCAAAGGCGT	CC2
SMC17.T.CGTTAGAGTTGTTTGG	CC2
SMC17.T.CGTTCTGAGTGCGATG	CC2
SMC17.T.CGTTCTGGTCCAGTGC	CC2
SMC17.T.CGTTGGGTCGGATGTT	CC2
SMC17.T.CTAACTTCATACCATG	CC2
SMC17.T.CTAAGACCATATACGC	CC2
SMC17.T.CTAAGACTCTCAACTT	CC2
SMC17.T.CTAATGGTCTGTGCAA	CC2
SMC17.T.CTACACCAGAATAGGG	CC2
SMC17.T.CTACACCAGATGCCAG	CC2
SMC17.T.CTACACCAGCACACAG	CC2
SMC17.T.CTACACCGTCTCACCT	CC2
SMC17.T.CTACACCGTTAAGAAC	CC2
SMC17.T.CTACATTGTGATAAGT	CC2
SMC17.T.CTACCCATCACATAGC	CC2
SMC17.T.CTACGTCAGCAACGGT	CC2
SMC17.T.CTAGCCTGTGTCGCTG	CC2
SMC17.T.CTAGTGACACCATGTA	CC2
SMC17.T.CTAGTGAGTACCGCTG	CC2
SMC17.T.CTCACACCATCTCCCA	CC2
SMC17.T.CTCACACGTGACTCAT	CC2
SMC17.T.CTCAGAATCCCACTTG	CC2
SMC17.T.CTCCTAGTCCACGTTC	CC2
SMC17.T.CTCGAAAAGTCTCGGC	CC2
SMC17.T.CTCGGAGTCGTCCGTT	CC2
SMC17.T.CTCGGGAAGTTACGGG	CC2
SMC17.T.CTCGGGACACTGCCAG	CC2
SMC17.T.CTCGTACAGCGCCTCA	CC2
SMC17.T.CTCGTCAAGACCGGAT	CC2
SMC17.T.CTCGTCAGTACCGTTA	CC2
SMC17.T.CTCTAATAGCACCGTC	CC2
SMC17.T.CTCTAATTCAGAGACG	CC2

SMC17.T.CTCTACGCAATCTGCA	CC2
SMC17.T.CTGAAACCATATGGTC	CC2
SMC17.T.CTGAAGTTCAAACCAC	CC2
SMC17.T.CTGCCTAAGAGACTTA	CC2
SMC17.T.CTGCTGTCAATAGCGG	CC2
SMC17.T.CTGCTGTGTGTCGCTG	CC2
SMC17.T.CTGCTGTTCTCTTATG	CC2
SMC17.T.CTGGTCTCAGGAATGC	CC2
SMC17.T.CTGGTCTGTCACAAGG	CC2
SMC17.T.CTGGTCTTCACATAGC	CC2
SMC17.T.CTGTGCTAGACTAGGC	CC2
SMC17.T.CTGTTTATCTGATTCT	CC2
SMC17.T.CTGTTTATCTTTAGTC	CC2
SMC17.T.CTTAACTTCCTTCAAT	CC2
SMC17.T.CTTAGGACAGGGTTAG	CC2
SMC17.T.CTTGGCTAGATCGGGT	CC2
SMC17.T.CTTTGCGTCTTAGAGC	CC2
SMC17.T.GAAACTCTCGCCAAAT	CC2
SMC17.T.GAAACTCTCGGTGTCG	CC2
SMC17.T.GAAATGACACCATGTA	CC2
SMC17.T.GAAATGATCGTCCGTT	CC2
SMC17.T.GAACATCAGCTGCAAG	CC2
SMC17.T.GAACATCGTGCAACTT	CC2
SMC17.T.GAAGCAGGTGCTTCTC	CC2
SMC17.T.GAATAAGGTACTTCTT	CC2
SMC17.T.GAATAAGTCTCACATT	CC2
SMC17.T.GAATGAATCACTTACT	CC2
SMC17.T.GACACGCTCACAGTAC	CC2
SMC17.T.GACAGAGCAAACAACA	CC2
SMC17.T.GACCAATAGAACTGTA	CC2
SMC17.T.GACGCGTAGTAACCCT	CC2
SMC17.T.GACGCGTTCTTCCTTC	CC2
SMC17.T.GACGGCTAGCTAACTC	CC2
SMC17.T.GACGGCTTCGCACTCT	CC2
SMC17.T.GACGTTATCTAACTGG	CC2
SMC17.T.GACTAACAGCCACTAT	CC2
SMC17.T.GACTAACCACGCATCG	CC2
SMC17.T.GACTAACTCCACTCCA	CC2
SMC17.T.GACTACAAGGACATTA	CC2
SMC17.T.GACTACAGTCCAAGTT	CC2
SMC17.T.GACTGCGCAATCCAAC	CC2
SMC17.T.GAGCAGAGTCAGCTAT	CC2
SMC17.T.GAGCAGATCTCTGTCG	CC2
SMC17.T.GAGGTGAAGCCCAACC	CC2
SMC17.T.GAGGTGAAGTCCGTAT	CC2
SMC17.T.GAGTCCGGTAGGCATG	CC2
SMC17.T.GATCAGTGTACGCTGC	CC2
SMC17.T.GATCGATGTTTAAGCC	CC2
SMC17.T.GATCGTACAAACTGTC	CC2
SMC17.T.GATCTAGGTTGCCTCT	CC2
SMC17.T.GATGAGGCACTTACGA	CC2
SMC17.T.GATTCAGAGGCTAGAC	CC2
SMC17.T.GCAAACTGTGTTGGGA	CC2
SMC17.T.GCAGCCATCGCTGATA	CC2
SMC17.T.GCAGTTATCATATCGG	CC2
SMC17.T.GCAGTTATCCGGCACA	CC2
SMC17.T.GCATACAAGTAGTGCG	CC2
SMC17.T.GCATGATTCAATACCG	CC2

SMC17.T.GCATGCGTCCTGCTTG	CC2
SMC17.T.GCATGTAAGCTGAAAT	CC2
SMC17.T.GCCAAATCAAGCCCAC	CC2
SMC17.T.GCCAAATGTAGCTGCC	CC2
SMC17.T.GCCTCTATCTATGTGG	CC2
SMC17.T.GCGAGAACAGTTCCCT	CC2
SMC17.T.GCGCAGTAGGAATTAC	CC2
SMC17.T.GCGCAGTAGTGACTCT	CC2
SMC17.T.GCGCGATCACCACCAG	CC2
SMC17.T.GCTCCTACAAACGCGA	CC2
SMC17.T.GCTCCTAGTAATCGTC	CC2
SMC17.T.GCTCTGTGTAGCTAAA	CC2
SMC17.T.GCTGCGAAGTGACATA	CC2
SMC17.T.GCTGGGTGTTGGTGGA	CC2
SMC17.T.GCTTCCAAGTGTGGCA	CC2
SMC17.T.GCTTCCACAAAGTGCG	CC2
SMC17.T.GCTTCCAGTTGAGGTG	CC2
SMC17.T.GGAAAGCCATTACGAC	CC2
SMC17.T.GGAAAGCTCTGCTTGC	CC2
SMC17.T.GGAACTTAGAATGTTG	CC2
SMC17.T.GGAACTTCATGCCCGA	CC2
SMC17.T.GGAACTTTCAGAAATG	CC2
SMC17.T.GGAACTTTCCCGACTT	CC2
SMC17.T.GGACAAGCAATGCCAT	CC2
SMC17.T.GGACAAGTCCACGTGG	CC2
SMC17.T.GGACAGACACCTCGTT	CC2
SMC17.T.GGACATTTCTGTCTAT	CC2
SMC17.T.GGACGTCGTGTGGTTT	CC2
SMC17.T.GGAGCAAAGTGACTCT	CC2
SMC17.T.GGAGCAATCCCATTAT	CC2
SMC17.T.GGATGTTGTAAGTAGT	CC2
SMC17.T.GGATTACCAACACCTA	CC2
SMC17.T.GGCGACTTCTGGTATG	CC2
SMC17.T.GGCTCGACAGGTCGTC	CC2
SMC17.T.GGCTCGATCGGAGGTA	CC2
SMC17.T.GGCTCGATCTGCGGCA	CC2
SMC17.T.GGCTGGTGTTCCGGCA	CC2
SMC17.T.GGGAATGTCCTTGGTC	CC2
SMC17.T.GGGAATGTCTGCCAGG	CC2
SMC17.T.GGGAGATGTGGCGAAT	CC2
SMC17.T.GGGAGATTCACCGGGT	CC2
SMC17.T.GGGATGAGTTCAGTAC	CC2
SMC17.T.GGGCACTCAATACGCT	CC2
SMC17.T.GGGTCTGTCACCGGGT	CC2
SMC17.T.GGTGAAGTCGCGCCAA	CC2
SMC17.T.GGTGCGTCAATCTGCA	CC2
SMC17.T.GGTGTTAAGACAAAGG	CC2
SMC17.T.GGTGTTATCAGGCGAA	CC1
SMC17.T.GGTGTTATCCCTGACT	CC2
SMC17.T.GTAACGTAGAGACTTA	CC2
SMC17.T.GTACTCCAGTCCGTAT	CC2
SMC17.T.GTACTCCAGTGATCGG	CC2
SMC17.T.GTAGGCCTCATGTGGT	CC2
SMC17.T.GTAGGCCTCTTGTACT	CC2
SMC17.T.GTATCTTAGCCGCCTA	CC2
SMC17.T.GTATCTTCATATACCG	CC2
SMC17.T.GTATTCTAGCCGATTT	CC2
SMC17.T.GTATTCTTCCTGTAGA	CC2

SMC17.T.GTCAAGTAGCTGGAAC	CC2
SMC17.T.GTCAAGTCAGTCGTGC	CC2
SMC17.T.GTCAAGTTCAGCTCTC	CC2
SMC17.T.GTCAAGTTCTGCTGCT	CC2
SMC17.T.GTCACAAAGCAGGTCA	CC2
SMC17.T.GTCGGGTGTTAGATGA	CC2
SMC17.T.GTCGTAACATCGGGTC	CC2
SMC17.T.GTGCAGCCACAAGTAA	CC2
SMC17.T.GTGCAGCCACTTCGAA	CC2
SMC17.T.GTGCAGCTCGTAGATC	CC2
SMC17.T.GTGCGGTTCTGCGTAA	CC2
SMC17.T.GTGGGTCCACCATCCT	CC2
SMC17.T.GTGGGTCTCAAGAAGT	CC2
SMC17.T.GTGTGCGAGTAGGTGC	CC2
SMC17.T.GTGTGCGTCCCTGACT	CC2
SMC17.T.GTGTGCGTCGTACGGC	CC2
SMC17.T.GTGTTAGAGAGCTATA	CC2
SMC17.T.GTTACAGTCGTAGGTT	CC2
SMC17.T.GTTCGGGAGACACGAC	CC2
SMC17.T.GTTCGGGTCTGTTGAG	CC2
SMC17.T.GTTTCTAGTGTGAATA	CC2
SMC17.T.TAAACCGGTTGATTGC	CC2
SMC17.T.TAAGAGACATGCATGT	CC2
SMC17.T.TACCTATGTCCAACTA	CC2
SMC17.T.TACCTATGTCTAGCGC	CC2
SMC17.T.TACCTTAAGAAACCTA	CC2
SMC17.T.TACCTTATCGTTACAG	CC2
SMC17.T.TACGGGCGTAAGTGGC	CC2
SMC17.T.TACGGGCGTTACGTCA	CC2
SMC17.T.TACTCGCTCCTGTACC	CC2
SMC17.T.TACTCGCTCGGCTACG	CC2
SMC17.T.TACTTACAGCGTTGCC	CC2
SMC17.T.TACTTACTCACCTTAT	CC2
SMC17.T.TAGACCAAGCTCTCGG	CC2
SMC17.T.TAGAGCTGTATGGTTC	CC2
SMC17.T.TAGCCGGTCAACCAAC	CC2
SMC17.T.TAGTGGTTCTGTCAAG	CC2
SMC17.T.TAGTTGGAGGGCACTA	CC2
SMC17.T.TATGCCCCAAGTAGTA	CC2
SMC17.T.TATGCCCCACTGTCGG	CC2
SMC17.T.TATGCCCGTCGAACAG	CC2
SMC17.T.TATTACCGTGGTCTCG	CC2
SMC17.T.TCAACGAAGACCGGAT	CC2
SMC17.T.TCAACGAGTTGATTCG	CC2
SMC17.T.TCAATCTCATCCGCGA	CC2
SMC17.T.TCACAAGAGTTACGGG	CC2
SMC17.T.TCACGAAAGTACGATA	CC2
SMC17.T.TCAGATGGTGCAGGTA	CC2
SMC17.T.TCAGCTCGTTGATTGC	CC2
SMC17.T.TCAGGATCAAGTAATG	CC2
SMC17.T.TCAGGTAAGCCCAGCT	CC2
SMC17.T.TCAGGTAAGTATTGGA	CC2
SMC17.T.TCAGGTAGTAGAAGGA	CC2
SMC17.T.TCATTTGTCTAGCACA	CC2
SMC17.T.TCCACACGTCCGTCAG	CC2
SMC17.T.TCCCGATTCAACACCA	CC2
SMC17.T.TCCCGATTCGCTAGCG	CC2
SMC17.T.TCGAGGCGTCGAGATG	CC2

SMC17.T.TCGCGAGTCCTATTCA	CC2
SMC17.T.TCGCGTTAGACGCACA	CC2
SMC17.T.TCGGGACAGAACAACT	CC2
SMC17.T.TCGGGACAGTAGTGCG	CC2
SMC17.T.TCGTACCTCTCCAGGG	CC2
SMC17.T.TCTATTGTCGAACGGA	CC2
SMC17.T.TCTCATAAGTCCGGTC	CC2
SMC17.T.TCTCATATCCCTCAGT	CC2
SMC17.T.TCTTCGGAGTCATCCA	CC2
SMC17.T.TCTTTCCAGCGTGTCC	CC2
SMC17.T.TCTTTCCGTGACAAAT	CC2
SMC17.T.TGACGGCTCTCAACTT	CC2
SMC17.T.TGACTTTGTGTGGTTT	CC2
SMC17.T.TGAGAGGGTAGCCTAT	CC2
SMC17.T.TGAGAGGTCCAAGTAC	CC2
SMC17.T.TGAGCCGCAAGGACTG	CC2
SMC17.T.TGAGCCGCATCGGGTC	CC2
SMC17.T.TGAGGGAAGAGGTTGC	CC2
SMC17.T.TGAGGGATCTGATTCT	CC2
SMC17.T.TGATTTCAGGCACATG	CC2
SMC17.T.TGATTTCAGTTAACGA	CC2
SMC17.T.TGATTTCTCGCCAAAT	CC2
SMC17.T.TGCACCTTCAGGATCT	CC2
SMC17.T.TGCCAAACAGGTGCCT	CC2
SMC17.T.TGCCCATGTCTTCTCG	CC2
SMC17.T.TGCCCTAAGCCAGTTT	CC2
SMC17.T.TGCCCTAAGGTGTGGT	CC2
SMC17.T.TGCGGGTGTAGAAGGA	CC2
SMC17.T.TGCGGGTGTTTACTCT	CC2
SMC17.T.TGCTACCTCCACTGGG	CC2
SMC17.T.TGCTGCTTCCGCAGTG	CC2
SMC17.T.TGGCCAGCATCCCACT	CC2
SMC17.T.TGGCCAGTCTCGAGTA	CC2
SMC17.T.TGGCGCAAGCTTATCG	CC2
SMC17.T.TGGCTGGAGAACTGTA	CC2
SMC17.T.TGGTTCCAGTACGATA	CC2
SMC17.T.TGTATTCTCAACCAAC	CC2
SMC17.T.TGTCCCACAACTGGCC	CC2
SMC17.T.TGTCCCAGTTTCGCTC	CC2
SMC17.T.TGTGGTACAGCTGTGC	CC2
SMC17.T.TGTGGTACATGGAATA	CC2
SMC17.T.TGTGGTATCTATCGCC	CC2
SMC17.T.TGTGTTTAGTGCGATG	CC2
SMC17.T.TGTGTTTCACTACAGT	CC2
SMC17.T.TTAGGACGTTGCGCAC	CC2
SMC17.T.TTAGGCACACATCCGG	CC2
SMC17.T.TTAGGCAGTTCTGAAC	CC2
SMC17.T.TTATGCTCACATTAGC	CC2
SMC17.T.TTATGCTCAGTTCCCT	CC2
SMC17.T.TTATGCTCATCTGGTA	CC2
SMC17.T.TTATGCTCATGGTAGG	CC2
SMC17.T.TTATGCTTCAACGGCC	CC2
SMC17.T.TTCCCAGGTTGCCTCT	CC2
SMC17.T.TTCCCAGTCATTCACT	CC2
SMC17.T.TTCGAAGAGGATGGAA	CC2
SMC17.T.TTCTACAAGGGCTCTC	CC2
SMC17.T.TTCTCCTAGAAAGTGG	CC2
SMC17.T.TTCTTAGAGAGACGAA	CC2

SMC17.T.TTGACTTCAAGACACG	CC2
SMC17.T.TTGCCGTGTCCATGAT	CC2
SMC17.T.TTGCGTCAGTGGTAAT	CC2
SMC17.T.TTGCGTCCACCGATAT	CC2
SMC17.T.TTGGCAATCCGCAAGC	CC2
SMC17.T.TTGGCAATCGGAATCT	CC2
SMC17.T.TTTACTGTCACCCTCA	CC2
SMC17.T.TTTACTGTCATTGCGA	CC2
SMC17.T.TTTATGCAGCCGATTT	CC2
SMC17.T.TTTATGCGTAGCGCTC	CC2
SMC17.T.TTTCCTCGTCCGAATT	CC2
SMC17.T.TTTGCGCCAAGTTGTC	CC2
SMC17.T.TTTGCGCGTATAAACG	CC2
SMC17.T.TTTGCGCGTATAATGG	CC2
SMC18.T.AAACCTGAGCGCCTTG	CC1
SMC18.T.AAACCTGCAAATCCGT	CC1
SMC18.T.AAACCTGGTTACGGAG	CC1
SMC18.T.AAACCTGTCTCAACTT	CC1
SMC18.T.AAACGGGAGCCCGAAA	CC1
SMC18.T.AAACGGGCAATGGAGC	CC1
SMC18.T.AAACGGGCAGGATTGG	CC1
SMC18.T.AAACGGGCAGGTTTCA	CC1
SMC18.T.AAACGGGGTAGAAGGA	CC1
SMC18.T.AAACGGGGTGCGCTTG	CC1
SMC18.T.AAACGGGGTGTGCGTC	CC1
SMC18.T.AAAGATGAGTGTTAGA	CC1
SMC18.T.AAAGATGTCGCTGATA	CC1
SMC18.T.AAAGCAAAGCGTGAAC	CC1
SMC18.T.AAAGCAAAGTAGGTGC	CC1
SMC18.T.AAAGCAACACATTCGA	CC1
SMC18.T.AAAGCAAGTACCGGCT	CC1
SMC18.T.AAAGCAATCCTAAGTG	CC1
SMC18.T.AAAGTAGGTTCAGGCC	CC1
SMC18.T.AAAGTAGTCTCTTATG	CC1
SMC18.T.AAATGCCAGCTACCTA	CC1
SMC18.T.AAATGCCAGCTAGTCT	CC1
SMC18.T.AAATGCCCAGGAATGC	CC1
SMC18.T.AAATGCCGTCTTTCAT	CC1
SMC18.T.AACACGTAGTCAAGGC	CC1
SMC18.T.AACACGTCATTTCACT	CC1
SMC18.T.AACACGTGTACTTAGC	CC1
SMC18.T.AACACGTGTCTGATTG	CC1
SMC18.T.AACACGTTCCGCGGTA	CC1
SMC18.T.AACACGTTCTCTTATG	CC1
SMC18.T.AACCATGGTTTGTTTC	CC1
SMC18.T.AACCATGTCACATGCA	CC1
SMC18.T.AACCGCGAGACTACAA	CC1
SMC18.T.AACCGCGGTACCGCTG	CC1
SMC18.T.AACCGCGGTGATGTCT	CC1
SMC18.T.AACCGCGGTGTGAAAT	CC1
SMC18.T.AACCGCGTCAGCACAT	CC1
SMC18.T.AACGTTGAGACCTTTG	CC1
SMC18.T.AACGTTGCACATCCAA	CC1
SMC18.T.AACGTTGTCCCTCAGT	CC1
SMC18.T.AACTCAGTCTTCGGTC	CC1
SMC18.T.AACTCCCAGTGCTGCC	CC1
SMC18.T.AACTCCCGTCTAAAGA	CC1
SMC18.T.AACTCCCTCCCTCAGT	CC1

SMC18.T.AACTCCCTCTTCAACT	CC1
SMC18.T.AACTCTTCAAGCCCAC	CC1
SMC18.T.AACTCTTGTCATCGGC	CC1
SMC18.T.AACTCTTTCATCATTC	CC1
SMC18.T.AACTGGTAGAAGCCCA	CC1
SMC18.T.AACTGGTCAAGCCATT	CC1
SMC18.T.AACTGGTCATATGGTC	CC1
SMC18.T.AACTGGTTCAATCTCT	CC1
SMC18.T.AACTTTCCAAGTTAAG	CC1
SMC18.T.AACTTTCCACAGCCCA	CC1
SMC18.T.AAGACCTAGCCTATGT	CC1
SMC18.T.AAGACCTCAACTGCTA	CC1
SMC18.T.AAGCCGCAGTGTTAGA	CC1
SMC18.T.AAGCCGCGTCAAAGAT	CC1
SMC18.T.AAGCCGCTCTTCGGTC	CC1
SMC18.T.AAGCCGCTCTTTAGGG	CC1
SMC18.T.AAGGAGCAGTACGTAA	CC1
SMC18.T.AAGGAGCCACCTTGTC	CC1
SMC18.T.AAGGAGCCAGTTCCCT	CC1
SMC18.T.AAGGCAGAGACGCACA	CC1
SMC18.T.AAGGCAGAGTACTTGC	CC1
SMC18.T.AAGGCAGCAGCCAATT	CC1
SMC18.T.AAGGTTCAGCTTTGGT	CC1
SMC18.T.AAGGTTCAGGACAGCT	CC1
SMC18.T.AAGTCTGGTTTCGCTC	CC1
SMC18.T.AATCCAGCACCCATTC	CC1
SMC18.T.AATCCAGGTACAAGTA	CC1
SMC18.T.AATCCAGGTCCGAGTC	CC1
SMC18.T.AATCGGTAGATCTGAA	CC1
SMC18.T.AATCGGTAGATGGGTC	CC1
SMC18.T.AATCGGTAGGTACTCT	CC1
SMC18.T.AATCGGTCACAGATTC	CC1
SMC18.T.AATCGGTCACATCCAA	CC1
SMC18.T.AATCGGTCACCAGGCT	CC1
SMC18.T.AATCGGTCATCTACGA	CC1
SMC18.T.AATCGGTGTTGTTTGG	CC1
SMC18.T.AATCGGTTCCGCTGTT	CC1
SMC18.T.AATCGGTTCCTTGCCA	CC1
SMC18.T.ACACCAAAGCTAACTC	CC1
SMC18.T.ACACCAAAGGGTTCCC	CC1
SMC18.T.ACACCAACAATCCAAC	CC1
SMC18.T.ACACCCTAGGGATGGG	CC1
SMC18.T.ACACCCTCACTGTTAG	CC1
SMC18.T.ACACCCTCATCGGAAG	CC1
SMC18.T.ACACCCTCATGACATC	CC1
SMC18.T.ACACCCTTCTGTTGAG	CC1
SMC18.T.ACACCGGCAAATTGCC	CC1
SMC18.T.ACACCGGCACGAAACG	CC1
SMC18.T.ACACCGGCACGGCCAT	CC1
SMC18.T.ACACTGACAAACCCAT	CC1
SMC18.T.ACAGCCGAGGCTCAGA	CC1
SMC18.T.ACAGCCGCATCCCACT	CC1
SMC18.T.ACAGCCGCATGAACCT	CC1
SMC18.T.ACAGCCGGTCTCTCGT	CC1
SMC18.T.ACAGCCGGTGCAGACA	CC1
SMC18.T.ACAGCTAAGCAGCGTA	CC1
SMC18.T.ACAGCTAAGGATGCGT	CC1
SMC18.T.ACAGCTACAAAGTCAA	CC1

SMC18.T.ACAGCTACACGGCTAC	CC1
SMC18.T.ACAGCTAGTGTTTGGT	CC1
SMC18.T.ACAGCTAGTTCCCTTG	CC1
SMC18.T.ACATACGAGCCGATTT	CC1
SMC18.T.ACATACGCATTGGTAC	CC1
SMC18.T.ACATACGGTTGGTAAA	CC1
SMC18.T.ACATCAGAGGGCTCTC	CC1
SMC18.T.ACATCAGCAGCGAACA	CC1
SMC18.T.ACATCAGGTAGCGCTC	CC1
SMC18.T.ACATCAGTCATAACCG	CC1
SMC18.T.ACATGGTAGGCCGAAT	CC1
SMC18.T.ACATGGTCAAGGACAC	CC1
SMC18.T.ACATGGTGTCAATACC	CC1
SMC18.T.ACATGGTGTGTTCTTT	CC1
SMC18.T.ACATGGTTCGTTGCCT	CC1
SMC18.T.ACCAGTAAGAGCAATT	CC1
SMC18.T.ACCAGTAAGAGGTACC	CC1
SMC18.T.ACCAGTACAAGTTGTC	CC1
SMC18.T.ACCAGTACAATAGCGG	CC1
SMC18.T.ACCAGTATCTCATTCA	CC1
SMC18.T.ACCCACTAGCTCCTTC	CC1
SMC18.T.ACCCACTAGGCCCTTG	CC1
SMC18.T.ACCCACTCAGCGAACA	CC1
SMC18.T.ACCCACTCATGCCTTC	CC1
SMC18.T.ACCCACTGTACTTCTT	CC1
SMC18.T.ACCCACTGTTCCACGG	CC1
SMC18.T.ACCCACTTCGAGAACG	CC1
SMC18.T.ACCGTAAAGATCTGAA	CC1
SMC18.T.ACCGTAAAGTTAGCGG	CC1
SMC18.T.ACCGTAAAGTTTAGGA	CC1
SMC18.T.ACCGTAAGTAAGTGGC	CC1
SMC18.T.ACCGTAAGTTCCCGAG	CC1
SMC18.T.ACCGTAATCTTAGAGC	CC1
SMC18.T.ACCTTTAAGCCATCGC	CC1
SMC18.T.ACCTTTACAGGTGGAT	CC1
SMC18.T.ACCTTTACATTATCTC	CC1
SMC18.T.ACCTTTATCGTATCAG	CC1
SMC18.T.ACGAGCCCAAGTTCTG	CC1
SMC18.T.ACGAGCCGTCGCGTGT	CC1
SMC18.T.ACGAGCCTCTTTAGTC	CC1
SMC18.T.ACGAGGAAGCAGCGTA	CC1
SMC18.T.ACGAGGAGTCGAGATG	CC1
SMC18.T.ACGAGGATCACAGGCC	CC1
SMC18.T.ACGAGGATCGATAGAA	CC1
SMC18.T.ACGATACCAAACGTGG	CC1
SMC18.T.ACGATGTAGGCTCAGA	CC1
SMC18.T.ACGATGTGTATGCTTG	CC1
SMC18.T.ACGCAGCGTATAGGTA	CC1
SMC18.T.ACGCAGCTCAGTTCGA	CC1
SMC18.T.ACGCCAGAGGCTCAGA	CC1
SMC18.T.ACGCCGAAGCGTTGCC	CC1
SMC18.T.ACGCCGACACATTCGA	CC1
SMC18.T.ACGGAGACACCAGCAC	CC1
SMC18.T.ACGGAGAGTCTAACGT	CC1
SMC18.T.ACGGCCAAGCTTCGCG	CC1
SMC18.T.ACGGCCAAGGGTTTCT	CC1
SMC18.T.ACGGCCACACCCATGG	CC1
SMC18.T.ACGGGCTAGTGCCAGA	CC1

SMC18.T.ACGGGCTCAGTGACAG	CC1
SMC18.T.ACGGGCTCATCCTTGC	CC1
SMC18.T.ACGGGCTTCGGATGGA	CC1
SMC18.T.ACGGGTCCAAGTACCT	CC1
SMC18.T.ACGGGTCGTACATCCA	CC1
SMC18.T.ACGTCAAAGTGGTAGC	CC1
SMC18.T.ACGTCAACACGCATCG	CC1
SMC18.T.ACGTCAAGTAGGACAC	CC1
SMC18.T.ACGTCAAGTCGCATCG	CC1
SMC18.T.ACGTCAAGTTCCATGA	CC1
SMC18.T.ACTATCTCACGTAAGG	CC1
SMC18.T.ACTATCTCAGTGACAG	CC1
SMC18.T.ACTATCTGTCACCCAG	CC1
SMC18.T.ACTATCTTCAGCTCGG	CC1
SMC18.T.ACTATCTTCCTGCTTG	CC1
SMC18.T.ACTGAACAGCGATATA	CC1
SMC18.T.ACTGAACCAAATACAG	CC1
SMC18.T.ACTGAACCAGGAATGC	CC1
SMC18.T.ACTGAACGTCTCAACA	CC1
SMC18.T.ACTGAACTCGGTTAAC	CC1
SMC18.T.ACTGAGTGTAGAAGGA	CC1
SMC18.T.ACTGATGCATAAAGGT	CC1
SMC18.T.ACTGATGCATACAGCT	CC1
SMC18.T.ACTGATGGTGTTCTTT	CC1
SMC18.T.ACTGATGTCACTTCAT	CC1
SMC18.T.ACTGCTCGTATTACCG	CC1
SMC18.T.ACTGCTCGTCGTCTTC	CC1
SMC18.T.ACTGCTCGTGTGACCC	CC1
SMC18.T.ACTGCTCTCTCGTTTA	CC1
SMC18.T.ACTGTCCAGGAGTACC	CC1
SMC18.T.ACTGTCCCAGTATAAG	CC1
SMC18.T.ACTGTCCCATTTGCTT	CC1
SMC18.T.ACTTACTAGAGCTGCA	CC1
SMC18.T.ACTTACTCAAGAGGCT	CC1
SMC18.T.ACTTACTGTACCGTAT	CC1
SMC18.T.ACTTACTGTCTTGCGG	CC1
SMC18.T.ACTTACTGTTATCACG	CC1
SMC18.T.ACTTGTTAGTAAGTAC	CC1
SMC18.T.ACTTGTTGTAGTACCT	CC1
SMC18.T.ACTTGTTGTTAGTGGG	CC1
SMC18.T.ACTTTCAGTCCCTACT	CC1
SMC18.T.ACTTTCATCACCGTAA	CC1
SMC18.T.ACTTTCATCACGATGT	CC1
SMC18.T.AGAATAGAGCCCTAAT	CC1
SMC18.T.AGAATAGCACGGCTAC	CC1
SMC18.T.AGAATAGCAGTGGGAT	CC1
SMC18.T.AGAATAGTCCGCATCT	CC1
SMC18.T.AGACGTTCACTCGACG	CC1
SMC18.T.AGACGTTTCTTGAGAC	CC1
SMC18.T.AGAGCGACACAGACTT	CC1
SMC18.T.AGAGCTTGTGGGTCAA	CC1
SMC18.T.AGAGCTTGTTCCACTC	CC1
SMC18.T.AGAGCTTGTTGCTCCT	CC1
SMC18.T.AGAGCTTTCCACGAAT	CC1
SMC18.T.AGAGCTTTCCACGTTC	CC1
SMC18.T.AGAGCTTTCCTAGTGA	CC1
SMC18.T.AGAGTGGGTGCCTGTG	CC1
SMC18.T.AGAGTGGTCTCATTCA	CC1

SMC18.T.AGATCTGAGGTGATTA	CC1
SMC18.T.AGATCTGAGTGTCTCA	CC1
SMC18.T.AGATCTGCATTGCGGC	CC1
SMC18.T.AGATCTGGTAAACCTC	CC1
SMC18.T.AGATCTGGTAGCAAAT	CC1
SMC18.T.AGATCTGGTCTCAACA	CC1
SMC18.T.AGATCTGGTGACGGTA	CC1
SMC18.T.AGATCTGGTGAGTATA	CC1
SMC18.T.AGATTGCCAAGAGTCG	CC1
SMC18.T.AGATTGCTCAAACAAG	CC1
SMC18.T.AGATTGCTCGCCGTGA	CC1
SMC18.T.AGCAGCCAGTGCAAGC	CC1
SMC18.T.AGCATACGTCGCTTTC	CC1
SMC18.T.AGCATACTCCCTCTTT	CC1
SMC18.T.AGCCTAAAGAAGGTGA	CC1
SMC18.T.AGCCTAAAGCGGATCA	CC1
SMC18.T.AGCCTAACAAGCTGTT	CC1
SMC18.T.AGCCTAACAATGAATG	CC1
SMC18.T.AGCCTAACACAGCGTC	CC1
SMC18.T.AGCCTAATCACCCGAG	CC1
SMC18.T.AGCGGTCAGAAGGACA	CC1
SMC18.T.AGCGGTCCAGCTGGCT	CC1
SMC18.T.AGCGGTCTCAACACCA	CC1
SMC18.T.AGCGGTCTCCAGAAGG	CC1
SMC18.T.AGCGGTCTCGAATGGG	CC1
SMC18.T.AGCGGTCTCGTCTGAA	CC1
SMC18.T.AGCGTATGTCAGTGGA	CC1
SMC18.T.AGCGTATGTCCAAGTT	CC1
SMC18.T.AGCGTATTCGCTGATA	CC1
SMC18.T.AGCGTATTCTGGCGAC	CC1
SMC18.T.AGCGTCGCATACGCCG	CC1
SMC18.T.AGCGTCGCATGCCCGA	CC1
SMC18.T.AGCGTCGGTCTCCACT	CC1
SMC18.T.AGCGTCGGTGCTGTAT	CC1
SMC18.T.AGCTCCTGTTGAGGTG	CC1
SMC18.T.AGCTCCTTCAGTTCGA	CC1
SMC18.T.AGCTCTCCATGGGAAC	CC1
SMC18.T.AGCTCTCGTACCTACA	CC1
SMC18.T.AGCTTGAAGACTCGGA	CC1
SMC18.T.AGCTTGAGTTAGAACA	CC1
SMC18.T.AGCTTGAGTTCCACGG	CC1
SMC18.T.AGGCCACAGATGCCTT	CC1
SMC18.T.AGGCCACAGTTAGGTA	CC1
SMC18.T.AGGCCACGTAGATTAG	CC1
SMC18.T.AGGCCGTAGTGAACGC	CC1
SMC18.T.AGGCCGTCAGGCTGAA	CC1
SMC18.T.AGGCCGTGTAACGCGA	CC1
SMC18.T.AGGCCGTTCACATAGC	CC1
SMC18.T.AGGCCGTTCGGTTCGG	CC1
SMC18.T.AGGGAGTGTATCGCAT	CC1
SMC18.T.AGGGAGTGTCACTTCC	CC1
SMC18.T.AGGGAGTGTTGACGTT	CC1
SMC18.T.AGGGAGTTCACTTCAT	CC1
SMC18.T.AGGGATGAGTTGCAGG	CC1
SMC18.T.AGGGTGAGTGGACGAT	CC1
SMC18.T.AGGTCATAGGGATCTG	CC1
SMC18.T.AGGTCATAGTAGGTGC	CC1
SMC18.T.AGGTCATGTCGCTTCT	CC1

SMC18.T.AGGTCATTCAACGGCC	CC1
SMC18.T.AGGTCCGCATAGGATA	CC1
SMC18.T.AGGTCCGGTCTAGCGC	CC1
SMC18.T.AGGTCCGGTTTGTTGG	CC1
SMC18.T.AGTAGTCAGGCATTGG	CC1
SMC18.T.AGTAGTCAGTGGTCCC	CC1
SMC18.T.AGTAGTCCAACTGCTA	CC2
SMC18.T.AGTCTTTCACTTCGAA	CC1
SMC18.T.AGTCTTTCAGATTGCT	CC1
SMC18.T.AGTCTTTGTCAGTGGA	CC1
SMC18.T.AGTGAGGAGCAGATCG	CC1
SMC18.T.AGTGAGGCATGCCCGA	CC1
SMC18.T.AGTGTCACACGCGAAA	CC1
SMC18.T.AGTTGGTCATGGATGG	CC1
SMC18.T.AGTTGGTGTAGAAGGA	CC1
SMC18.T.ATAACGCTCAAAGACA	CC1
SMC18.T.ATAACGCTCTGATTCT	CC1
SMC18.T.ATAAGAGAGGGTATCG	CC1
SMC18.T.ATAAGAGCAAAGTGCG	CC1
SMC18.T.ATAAGAGGTGATGTCT	CC1
SMC18.T.ATAAGAGTCAGGCGAA	CC1
SMC18.T.ATAGACCAGCCAGTTT	CC1
SMC18.T.ATAGACCAGGGTGTTG	CC1
SMC18.T.ATAGACCTCGAATGCT	CC1
SMC18.T.ATCACGAAGACTGGGT	CC1
SMC18.T.ATCACGAAGTACCGGA	CC2
SMC18.T.ATCACGACAGCATGAG	CC1
SMC18.T.ATCACGATCTGTCAAG	CC1
SMC18.T.ATCATCTCAAGTCTAC	CC1
SMC18.T.ATCATCTGTACAGTTC	CC1
SMC18.T.ATCATGGAGATCCGAG	CC1
SMC18.T.ATCATGGAGCAGGTCA	CC1
SMC18.T.ATCATGGAGTACCGGA	CC1
SMC18.T.ATCATGGGTCTTCAAG	CC1
SMC18.T.ATCATGGGTCTTCGTC	CC1
SMC18.T.ATCCACCCAGCGATCC	CC1
SMC18.T.ATCCACCCATTAGGCT	CC1
SMC18.T.ATCCACCGTACAAGTA	CC1
SMC18.T.ATCCACCGTATAATGG	CC1
SMC18.T.ATCCACCTCAGGCCCA	CC1
SMC18.T.ATCCGAACACCGCTAG	CC1
SMC18.T.ATCCGAAGTCCGAATT	CC1
SMC18.T.ATCCGAAGTTAAGGGC	CC1
SMC18.T.ATCCGAATCTATCCCG	CC1
SMC18.T.ATCGAGTCAAATTGCC	CC1
SMC18.T.ATCGAGTCAGCTCCGA	CC1
SMC18.T.ATCGAGTCAGGTGCCT	CC1
SMC18.T.ATCGAGTGTCATCGGC	CC1
SMC18.T.ATCGAGTTCCGTCAAA	CC1
SMC18.T.ATCGAGTTCGTGGTCG	CC1
SMC18.T.ATCTGCCCAATCACAC	CC1
SMC18.T.ATCTGCCTCCCTCAGT	CC1
SMC18.T.ATGAGGGAGCTGCCCA	CC1
SMC18.T.ATGAGGGCACCAACCG	CC1
SMC18.T.ATGAGGGGTAGAGTGC	CC1
SMC18.T.ATGAGGGGTCCATGAT	CC1
SMC18.T.ATGAGGGGTCCGAGTC	CC1
SMC18.T.ATGAGGGGTGTGCCTG	CC1

SMC18.T.ATGAGGGTCACCACCT	CC1
SMC18.T.ATGCGATAGCAATCTC	CC1
SMC18.T.ATGCGATAGCGTCTAT	CC1
SMC18.T.ATGCGATAGCTCCTCT	CC1
SMC18.T.ATGCGATAGCTTATCG	CC1
SMC18.T.ATGCGATCATTGGGCC	CC1
SMC18.T.ATGCGATGTGGTGTAG	CC1
SMC18.T.ATGCGATTCAAGAAGT	CC1
SMC18.T.ATGGGAGAGCTGATAA	CC1
SMC18.T.ATGGGAGCACTGCCAG	CC1
SMC18.T.ATGGGAGCATAAGACA	CC1
SMC18.T.ATGGGAGGTCAAAGAT	CC1
SMC18.T.ATGGGAGGTTTGCATG	CC1
SMC18.T.ATGTGTGCATCACGTA	CC1
SMC18.T.ATGTGTGTCAGTTCGA	CC1
SMC18.T.ATGTGTGTCCGTAGTA	CC1
SMC18.T.ATTACTCAGCGCTTAT	CC1
SMC18.T.ATTACTCCACACTGCG	CC1
SMC18.T.ATTACTCGTACATGTC	CC1
SMC18.T.ATTACTCGTCGACTGC	CC1
SMC18.T.ATTACTCGTGGCAAAC	CC1
SMC18.T.ATTACTCTCACAAACC	CC1
SMC18.T.ATTATCCAGAGTGACC	CC1
SMC18.T.ATTATCCCACTCGACG	CC1
SMC18.T.ATTATCCCAGTCGTGC	CC1
SMC18.T.ATTATCCGTTGGGACA	CC1
SMC18.T.ATTCTACAGAGACTAT	CC1
SMC18.T.ATTCTACAGTCGATAA	CC1
SMC18.T.ATTCTACCACAGACAG	CC1
SMC18.T.ATTCTACTCCCACTTG	CC1
SMC18.T.ATTGGACCAAAGTCAA	CC1
SMC18.T.ATTGGACCAAGTCTGT	CC1
SMC18.T.ATTGGACGTCCAGTAT	CC1
SMC18.T.ATTGGACGTCGAACAG	CC1
SMC18.T.ATTGGACGTCGCGAAA	CC1
SMC18.T.ATTGGTGAGGTGCTTT	CC1
SMC18.T.ATTGGTGCATCGATTG	CC1
SMC18.T.ATTGGTGCATTGGCGC	CC1
SMC18.T.ATTGGTGGTGTTAAGA	CC1
SMC18.T.ATTTCTGAGCAATATG	CC1
SMC18.T.ATTTCTGGTCTGCCAG	CC1
SMC18.T.ATTTCTGGTGCCTGTG	CC1
SMC18.T.ATTTCTGTCAACCATG	CC1
SMC18.T.ATTTCTGTCGAGCCCA	CC1
SMC18.T.ATTTCTGTCGTTTGCC	CC1
SMC18.T.CAACCAAAGACTAGGC	CC1
SMC18.T.CAACCAAAGGGTGTTG	CC1
SMC18.T.CAACCAACAATAGAGT	CC1
SMC18.T.CAACCTCCAGACAGGT	CC1
SMC18.T.CAACCTCGTAACGACG	CC1
SMC18.T.CAACCTCGTCCGCTGA	CC1
SMC18.T.CAACCTCGTGACCAAG	CC1
SMC18.T.CAACTAGCAAACAACA	CC1
SMC18.T.CAACTAGTCACAAACC	CC1
SMC18.T.CAAGAAAGTAATTGGA	CC1
SMC18.T.CAAGAAAGTGGACGAT	CC1
SMC18.T.CAAGAAAGTTCGTGAT	CC1
SMC18.T.CAAGAAATCGGAGGTA	CC1

SMC18.T.CAAGATCCATGACATC	CC1
SMC18.T.CAAGATCTCCCGACTT	CC1
SMC18.T.CAAGATCTCTTCAACT	CC1
SMC18.T.CAAGGCCAGGCGATAC	CC1
SMC18.T.CAAGGCCCACTGAAGG	CC1
SMC18.T.CAAGGCCGTCACAAGG	CC1
SMC18.T.CAAGGCCTCCCTCTTT	CC1
SMC18.T.CAAGTTGAGAGACTAT	CC1
SMC18.T.CAAGTTGGTAAATGAC	CC1
SMC18.T.CACAAACCAAACAACA	CC1
SMC18.T.CACAAACGTACCGAGA	CC1
SMC18.T.CACAAACGTGTAATGA	CC1
SMC18.T.CACACAACAATCACAC	CC1
SMC18.T.CACACAACAGCCTTTC	CC1
SMC18.T.CACACAATCCTTCAAT	CC1
SMC18.T.CACACAATCGTCTGAA	CC1
SMC18.T.CACACCTAGAACTCGG	CC1
SMC18.T.CACACCTAGGCAAAGA	CC1
SMC18.T.CACACCTCACCACCAG	CC1
SMC18.T.CACACCTGTCTTCAAG	CC1
SMC18.T.CACACCTGTGATGTCT	CC1
SMC18.T.CACACCTTCTTACCTA	CC1
SMC18.T.CACACTCGTGGTTTCA	CC1
SMC18.T.CACACTCTCAGGATCT	CC1
SMC18.T.CACAGGCAGCCACTAT	CC1
SMC18.T.CACAGGCCATTAGCCA	CC1
SMC18.T.CACAGGCGTTAGAACA	CC1
SMC18.T.CACATAGAGTGGACGT	CC1
SMC18.T.CACATAGCACAAGCCC	CC2
SMC18.T.CACATAGCAGTCCTTC	CC1
SMC18.T.CACATAGGTGGTTTCA	CC1
SMC18.T.CACATAGTCGACGGAA	CC1
SMC18.T.CACATTTAGTGGTAGC	CC1
SMC18.T.CACATTTAGTTACGGG	CC1
SMC18.T.CACATTTCATGCAACT	CC1
SMC18.T.CACCACTAGCTAGTGG	CC1
SMC18.T.CACCACTGTCAATACC	CC1
SMC18.T.CACCAGGAGACTAAGT	CC1
SMC18.T.CACCAGGGTCTTCTCG	CC1
SMC18.T.CACCAGGGTTACGTCA	CC2
SMC18.T.CACCTTGCACGAGGTA	CC1
SMC18.T.CACCTTGCAGCTTCGG	CC1
SMC18.T.CACCTTGGTAGGACAC	CC1
SMC18.T.CACCTTGGTTATCCGA	CC1
SMC18.T.CACCTTGTCCCTCAGT	CC1
SMC18.T.CACTCCACACCAGGCT	CC1
SMC18.T.CACTCCAGTACTTAGC	CC1
SMC18.T.CAGAATCAGAGTACAT	CC1
SMC18.T.CAGAATCCATGTCGAT	CC1
SMC18.T.CAGAATCGTCGCGAAA	CC1
SMC18.T.CAGAATCGTTAGGGTG	CC1
SMC18.T.CAGAATCTCTTCAACT	CC1
SMC18.T.CAGAGAGAGCACGCCT	CC1
SMC18.T.CAGAGAGCACGTGAGA	CC1
SMC18.T.CAGATCAAGGAGTAGA	CC1
SMC18.T.CAGATCAAGGCTAGCA	CC1
SMC18.T.CAGATCAAGTTAACGA	CC1
SMC18.T.CAGATCACATTCCTGC	CC1

SMC18.T.CAGATCATCTAACTGG	CC1
SMC18.T.CAGCAGCCATCTACGA	CC1
SMC18.T.CAGCAGCGTATGAAAC	CC1
SMC18.T.CAGCAGCGTCCAGTGC	CC1
SMC18.T.CAGCAGCTCATCGATG	CC1
SMC18.T.CAGCATAAGTATCTCG	CC1
SMC18.T.CAGCATACACCCTATC	CC1
SMC18.T.CAGCATAGTGAGTGAC	CC1
SMC18.T.CAGCATAGTTGTCTTT	CC1
SMC18.T.CAGCATATCACCTCGT	CC1
SMC18.T.CAGCATATCATTGCCC	CC1
SMC18.T.CAGCATATCTGTCAAG	CC1
SMC18.T.CAGCCGAAGTGAACAT	CC1
SMC18.T.CAGCGACGTCAATGTC	CC1
SMC18.T.CAGCGACGTCCATGAT	CC1
SMC18.T.CAGCTAACAGTCCTTC	CC1
SMC18.T.CAGCTAAGTCCTCTTG	CC1
SMC18.T.CAGCTAATCTGGTATG	CC1
SMC18.T.CAGCTGGAGCTGTCTA	CC1
SMC18.T.CAGCTGGAGGCCGAAT	CC1
SMC18.T.CAGCTGGAGGCGATAC	CC1
SMC18.T.CAGCTGGCAGCTGGCT	CC1
SMC18.T.CAGGTGCAGGCCCTCA	CC1
SMC18.T.CAGGTGCCACTCAGGC	CC1
SMC18.T.CAGTAACAGCAGGCTA	CC1
SMC18.T.CAGTAACTCATTGCCC	CC1
SMC18.T.CAGTCCTAGCGATCCC	CC1
SMC18.T.CAGTCCTGTACACCGC	CC1
SMC18.T.CATATTCGTCCTCCAT	CC1
SMC18.T.CATCAGAAGCGCCTCA	CC1
SMC18.T.CATCAGACAGTTTACG	CC1
SMC18.T.CATCAGATCGCATGAT	CC1
SMC18.T.CATCAGATCTTTACAC	CC1
SMC18.T.CATCCACAGGGATGGG	CC1
SMC18.T.CATCCACCACAGAGGT	CC1
SMC18.T.CATCGAACAGCTTCGG	CC1
SMC18.T.CATCGAAGTAGCGATG	CC1
SMC18.T.CATCGAAGTGCAGGTA	CC1
SMC18.T.CATCGAATCGCCTGAG	CC1
SMC18.T.CATCGAATCGGTGTTA	CC1
SMC18.T.CATGACACAGTCACTA	CC1
SMC18.T.CATGACAGTCGAAAGC	CC1
SMC18.T.CATGACATCTGTCTAT	CC1
SMC18.T.CATGCCTAGTCAATAG	CC1
SMC18.T.CATGCCTGTAATTGGA	CC1
SMC18.T.CATGCCTGTATATCCG	CC1
SMC18.T.CATGCCTTCAAACCGT	CC1
SMC18.T.CATGCCTTCCTAGAAC	CC1
SMC18.T.CATGCCTTCTCTAGGA	CC1
SMC18.T.CATGCCTTCTGCTTGC	CC1
SMC18.T.CATGGCGTCATCATTC	CC1
SMC18.T.CATGGCGTCCAAAGTC	CC1
SMC18.T.CATTATCAGTCCGTAT	CC1
SMC18.T.CATTATCTCTCATTCA	CC1
SMC18.T.CATTCGCGTCTCTTAT	CC1
SMC18.T.CATTCGCTCTTTACGT	CC1
SMC18.T.CCAATCCCAAGTTAAG	CC1
SMC18.T.CCAATCCCATAGAAAC	CC1

SMC18.T.CCAATCCCATGCCACG	CC1
SMC18.T.CCAATCCTCCACTGGG	CC1
SMC18.T.CCACCTATCTTCATGT	CC1
SMC18.T.CCACTACAGATCGATA	CC1
SMC18.T.CCACTACGTAGAAAGG	CC1
SMC18.T.CCAGCGATCACTCTTA	CC1
SMC18.T.CCAGCGATCTGCTGTC	CC1
SMC18.T.CCATGTCAGTACGCCC	CC1
SMC18.T.CCATGTCGTACCAGTT	CC1
SMC18.T.CCATTCGAGAGATGAG	CC1
SMC18.T.CCATTCGCACGGTAAG	CC1
SMC18.T.CCATTCGGTCTTGTCC	CC1
SMC18.T.CCCAATCGTCTCCATC	CC1
SMC18.T.CCCAATCTCTAACTCT	CC1
SMC18.T.CCCAGTTCAAGTCTAC	CC1
SMC18.T.CCCAGTTCATTTGCCC	CC1
SMC18.T.CCCATACAGTTAAGTG	CC1
SMC18.T.CCCATACCAACCGCCA	CC1
SMC18.T.CCCATACGTCGTGGCT	CC1
SMC18.T.CCCATACTCGCTAGCG	CC1
SMC18.T.CCCTCCTCAAGTAATG	CC1
SMC18.T.CCCTCCTGTAATCACC	CC1
SMC18.T.CCCTCCTGTTCGGGCT	CC1
SMC18.T.CCCTCCTTCTGCAAGT	CC1
SMC18.T.CCGGGATGTCCGAACC	CC1
SMC18.T.CCGGGATGTTATGTGC	CC1
SMC18.T.CCGGTAGGTGATAAAC	CC1
SMC18.T.CCGGTAGTCACATAGC	CC1
SMC18.T.CCGGTAGTCTCCGGTT	CC1
SMC18.T.CCGTACTCACACTGCG	CC1
SMC18.T.CCGTGGAAGAGCAATT	CC1
SMC18.T.CCGTGGACATAAGACA	CC1
SMC18.T.CCGTGGACATGTAAGA	CC1
SMC18.T.CCGTGGACATGTCTCC	CC1
SMC18.T.CCGTGGAGTATCTGCA	CC1
SMC18.T.CCGTGGAGTCAACTGT	CC1
SMC18.T.CCGTTCAGTAGCGATG	CC1
SMC18.T.CCTAAAGCATCGACGC	CC1
SMC18.T.CCTAAAGCATTCGACA	CC1
SMC18.T.CCTACACAGGGCTTGA	CC1
SMC18.T.CCTACACCATCCCACT	CC1
SMC18.T.CCTACACGTCTGGAGA	CC1
SMC18.T.CCTACACTCTCTGCTG	CC1
SMC18.T.CCTACCAAGAAGGTGA	CC1
SMC18.T.CCTACCAAGAGCTATA	CC1
SMC18.T.CCTACCACACGGTTTA	CC1
SMC18.T.CCTACCACACGTCAGC	CC1
SMC18.T.CCTACCACACTTAACG	CC1
SMC18.T.CCTACCAGTCACAAGG	CC1
SMC18.T.CCTACCATCGCTAGCG	CC1
SMC18.T.CCTAGCTAGCTTCGCG	CC1
SMC18.T.CCTAGCTCAGCTGCAC	CC1
SMC18.T.CCTAGCTTCATAAAGG	CC1
SMC18.T.CCTAGCTTCCGCAAGC	CC1
SMC18.T.CCTATTAAGCCCAGCT	CC1
SMC18.T.CCTATTACAAGTAGTA	CC1
SMC18.T.CCTATTAGTATATGGA	CC1
SMC18.T.CCTATTAGTTCCAACA	CC1

SMC18.T.CCTATTATCAGTTTGG	CC1
SMC18.T.CCTATTATCGGAAACG	CC1
SMC18.T.CCTATTATCGGTTCGG	CC1
SMC18.T.CCTCAGTCAAGCGAGT	CC1
SMC18.T.CCTCAGTCATACCATG	CC1
SMC18.T.CCTCAGTCATGTAGTC	CC1
SMC18.T.CCTCAGTTCAAGAAGT	CC1
SMC18.T.CCTCAGTTCCTCGCAT	CC1
SMC18.T.CCTCTGAAGACAGGCT	CC1
SMC18.T.CCTTACGAGTTAGGTA	CC1
SMC18.T.CCTTCCCAGAGTAATC	CC1
SMC18.T.CCTTCCCAGCTTATCG	CC1
SMC18.T.CCTTCCCGTTCCATGA	CC1
SMC18.T.CCTTCGAAGACTTTCG	CC1
SMC18.T.CCTTCGAAGAGCCTAG	CC1
SMC18.T.CCTTCGAAGCTGAAAT	CC1
SMC18.T.CCTTCGATCAGTTTGG	CC1
SMC18.T.CCTTCGATCCCAACGG	CC1
SMC18.T.CCTTTCTGTACTTGAC	CC1
SMC18.T.CCTTTCTGTTCCGTCT	CC1
SMC18.T.CGAACATAGCGAGAAA	CC1
SMC18.T.CGAACATCATTATCTC	CC1
SMC18.T.CGAACATTCGGACAAG	CC1
SMC18.T.CGAATGTAGGCGTACA	CC1
SMC18.T.CGAATGTCATGTAAGA	CC1
SMC18.T.CGAATGTTCTCTAAGG	CC1
SMC18.T.CGACCTTCAAGGCTCC	CC1
SMC18.T.CGACCTTGTAGCACGA	CC1
SMC18.T.CGACTTCCAGGATCGA	CC1
SMC18.T.CGACTTCCATGAACCT	CC1
SMC18.T.CGACTTCGTGTTGAGG	CC1
SMC18.T.CGACTTCTCAGGTTCA	CC1
SMC18.T.CGACTTCTCGAGCCCA	CC1
SMC18.T.CGAGAAGAGTAGATGT	CC1
SMC18.T.CGAGAAGCAAGCGTAG	CC1
SMC18.T.CGAGAAGCAGTATCTG	CC1
SMC18.T.CGAGAAGTCCACGACG	CC1
SMC18.T.CGAGCACAGATGTGTA	CC1
SMC18.T.CGAGCACAGTGTACTC	CC1
SMC18.T.CGAGCACCAACGCACC	CC1
SMC18.T.CGAGCACCAGTTTACG	CC1
SMC18.T.CGAGCACTCTGGTTCC	CC1
SMC18.T.CGAGCCAGTAATCACC	CC1
SMC18.T.CGAGCCAGTCCGAATT	CC1
SMC18.T.CGAGCCATCCGCAGTG	CC1
SMC18.T.CGAGCCATCCTCATTA	CC1
SMC18.T.CGATCGGCACGAAATA	CC1
SMC18.T.CGATCGGCAGTAAGAT	CC1
SMC18.T.CGATCGGGTTCAGCGC	CC1
SMC18.T.CGATCGGTCAGTCAGT	CC1
SMC18.T.CGATGGCGTTGTGGCC	CC1
SMC18.T.CGATGTAAGTTGAGTA	CC1
SMC18.T.CGATGTATCGGGAGTA	CC1
SMC18.T.CGATGTATCTAACTCT	CC1
SMC18.T.CGATTGAAGCTGAAAT	CC1
SMC18.T.CGATTGACAGTAAGAT	CC1
SMC18.T.CGATTGACATCCAACA	CC1
SMC18.T.CGATTGAGTATCGCAT	CC1

SMC18.T.CGATTGAGTGGACGAT	CC1
SMC18.T.CGATTGATCCGTCAAA	CC1
SMC18.T.CGCCAAGAGTACGCGA	CC1
SMC18.T.CGCCAAGTCACCCGAG	CC1
SMC18.T.CGCGGTAGTGTCCTCT	CC1
SMC18.T.CGCGGTAGTTTACTCT	CC1
SMC18.T.CGCGGTATCACCGGGT	CC1
SMC18.T.CGCGGTATCATGCTCC	CC1
SMC18.T.CGCGTTTAGATGTGTA	CC1
SMC18.T.CGCGTTTCACTTAAGC	CC1
SMC18.T.CGCGTTTTCCTCCTAG	CC1
SMC18.T.CGCGTTTTCGTTTAGG	CC1
SMC18.T.CGCTATCCAGTCAGAG	CC1
SMC18.T.CGCTATCTCATTATCC	CC1
SMC18.T.CGCTATCTCGTGGACC	CC1
SMC18.T.CGCTGGAAGTGGGATC	CC1
SMC18.T.CGCTGGACAGCTATTG	CC1
SMC18.T.CGCTGGACAGTCGTGC	CC1
SMC18.T.CGCTGGAGTAGCTTGT	CC1
SMC18.T.CGCTGGATCCGTCAAA	CC1
SMC18.T.CGCTTCACAGTAAGCG	CC1
SMC18.T.CGGACACGTAAATGAC	CC1
SMC18.T.CGGACACGTAGAAGGA	CC1
SMC18.T.CGGACGTAGAAACCTA	CC1
SMC18.T.CGGACGTAGATCGGGT	CC1
SMC18.T.CGGACGTAGGCTACGA	CC1
SMC18.T.CGGACGTCACATTCGA	CC1
SMC18.T.CGGACGTCAGGTCCAC	CC1
SMC18.T.CGGACGTGTGGACGAT	CC1
SMC18.T.CGGACGTGTTCCACTC	CC1
SMC18.T.CGGACTGAGCTGAAAT	CC2
SMC18.T.CGGACTGCACGGCGTT	CC1
SMC18.T.CGGACTGCAGGGAGAG	CC1
SMC18.T.CGGAGCTAGGACAGAA	CC1
SMC18.T.CGGAGCTAGGAGCGTT	CC1
SMC18.T.CGGAGCTCATGCCTTC	CC1
SMC18.T.CGGAGTCAGACTCGGA	CC1
SMC18.T.CGGCTAGAGAATGTTG	CC1
SMC18.T.CGGCTAGGTAAGTAGT	CC1
SMC18.T.CGGCTAGGTATAGGTA	CC1
SMC18.T.CGGCTAGGTTCAGTAC	CC1
SMC18.T.CGGGTCATCCCTAACC	CC1
SMC18.T.CGGTTAACAAGGTGTG	CC1
SMC18.T.CGGTTAACATGGAATA	CC1
SMC18.T.CGGTTAATCCGCAAGC	CC1
SMC18.T.CGTAGCGCACTTAACG	CC1
SMC18.T.CGTAGCGTCAGCCTAA	CC1
SMC18.T.CGTAGGCTCATACGGT	CC1
SMC18.T.CGTAGGCTCGCTGATA	CC1
SMC18.T.CGTCACTAGGGAACGG	CC1
SMC18.T.CGTCACTCATGAGCGA	CC1
SMC18.T.CGTCACTGTGCGATAG	CC1
SMC18.T.CGTCAGGCACCATCCT	CC1
SMC18.T.CGTCAGGCAGCTGTAT	CC1
SMC18.T.CGTCAGGTCAGTCAGT	CC1
SMC18.T.CGTCCATAGTGGTAGC	CC1
SMC18.T.CGTCCATTCCAGTATG	CC1
SMC18.T.CGTCCATTCGGCATCG	CC1

SMC18.T.CGTCTACAGAGGGATA	CC1
SMC18.T.CGTCTACAGTAATCCC	CC1
SMC18.T.CGTCTACCACCGATAT	CC1
SMC18.T.CGTCTACGTTGCGCAC	CC1
SMC18.T.CGTCTACTCCTACAGA	CC1
SMC18.T.CGTGAGCGTAGGGTAC	CC1
SMC18.T.CGTGAGCGTCCCGACA	CC1
SMC18.T.CGTGAGCTCGCATGAT	CC1
SMC18.T.CGTGTAATCTAGAGTC	CC1
SMC18.T.CGTGTCTGTAAGCACG	CC1
SMC18.T.CGTTAGATCTTTCCTC	CC1
SMC18.T.CGTTGGGAGCGTCTAT	CC1
SMC18.T.CGTTGGGCACTAAGTC	CC1
SMC18.T.CGTTGGGGTCATCGGC	CC1
SMC18.T.CTAACTTAGAGCAATT	CC1
SMC18.T.CTAACTTCAAAGTGCG	CC1
SMC18.T.CTAACTTGTAGAGGAA	CC1
SMC18.T.CTAACTTGTCTAGTGT	CC1
SMC18.T.CTAACTTGTTGTCTTT	CC1
SMC18.T.CTAACTTTCCGTTGTC	CC1
SMC18.T.CTAAGACAGTTACCCA	CC1
SMC18.T.CTAAGACTCGAATGCT	CC1
SMC18.T.CTAATGGAGGCTCTTA	CC1
SMC18.T.CTAATGGGTGTTTGGT	CC1
SMC18.T.CTACACCCAGGTCGTC	CC1
SMC18.T.CTACACCCATGCTGGC	CC1
SMC18.T.CTACATTCAGCTGCAC	CC1
SMC18.T.CTACATTGTGTTGGGA	CC1
SMC18.T.CTACATTTCAGTGCAT	CC1
SMC18.T.CTACCCACAGCTTAAC	CC1
SMC18.T.CTAGAGTAGTGCCAGA	CC1
SMC18.T.CTAGAGTTCTGACCTC	CC1
SMC18.T.CTAGCCTTCAGAGGTG	CC1
SMC18.T.CTAGCCTTCATGTCTT	CC1
SMC18.T.CTAGCCTTCCATGCTC	CC1
SMC18.T.CTAGTGACACGAAACG	CC1
SMC18.T.CTAGTGACAGTGACAG	CC1
SMC18.T.CTAGTGATCTGATTCT	CC1
SMC18.T.CTCACACAGGTCGGAT	CC1
SMC18.T.CTCACACCACCCATTC	CC1
SMC18.T.CTCACACTCGTGGACC	CC1
SMC18.T.CTCAGAACAAGGTGTG	CC1
SMC18.T.CTCAGAACACCAGTTA	CC1
SMC18.T.CTCAGAACATCTCGCT	CC1
SMC18.T.CTCAGAATCGCTTAGA	CC1
SMC18.T.CTCATTAAGGCCATAG	CC1
SMC18.T.CTCATTACAATCTACG	CC1
SMC18.T.CTCCTAGCACAGACAG	CC1
SMC18.T.CTCCTAGCACGCATCG	CC1
SMC18.T.CTCCTAGGTCCTAGCG	CC1
SMC18.T.CTCCTAGGTTCCACGG	CC1
SMC18.T.CTCCTAGTCCCAAGAT	CC1
SMC18.T.CTCGAAATCATGCATG	CC1
SMC18.T.CTCGAAATCTGTCAAG	CC1
SMC18.T.CTCGAGGAGGGTTCCC	CC1
SMC18.T.CTCGAGGCAATCCGAT	CC1
SMC18.T.CTCGAGGCAGTGGGAT	CC1
SMC18.T.CTCGAGGGTACGCTGC	CC1

SMC18.T.CTCGGAGTCCACTGGG	CC1
SMC18.T.CTCGGGAAGGCGACAT	CC1
SMC18.T.CTCGGGAGTTAAAGTG	CC1
SMC18.T.CTCGGGATCATCACCC	CC1
SMC18.T.CTCGTACCACCTCGGA	CC1
SMC18.T.CTCGTACCATTATCTC	CC1
SMC18.T.CTCGTACGTAAGTAGT	CC1
SMC18.T.CTCGTACTCCCATTAT	CC1
SMC18.T.CTCGTCAAGTACGCCC	CC1
SMC18.T.CTCGTCAAGTGTTAGA	CC1
SMC18.T.CTCGTCACACAGACTT	CC1
SMC18.T.CTCGTCACATGACATC	CC1
SMC18.T.CTCTAATAGCAGATCG	CC1
SMC18.T.CTCTAATCACTACAGT	CC1
SMC18.T.CTCTAATTCGGCGCTA	CC1
SMC18.T.CTCTACGAGGCATGGT	CC1
SMC18.T.CTCTACGGTATATCCG	CC1
SMC18.T.CTCTACGGTTCCCGAG	CC1
SMC18.T.CTCTGGTAGAAACCAT	CC1
SMC18.T.CTCTGGTAGGCATGTG	CC1
SMC18.T.CTCTGGTCATGCATGT	CC1
SMC18.T.CTCTGGTGTTACTGAC	CC1
SMC18.T.CTCTGGTTCTTGTACT	CC1
SMC18.T.CTGAAACAGTTGCAGG	CC1
SMC18.T.CTGAAACCAGTGGGAT	CC1
SMC18.T.CTGAAACTCGGCGCTA	CC1
SMC18.T.CTGAAGTAGACACGAC	CC1
SMC18.T.CTGAAGTCACAGGTTT	CC1
SMC18.T.CTGAAGTTCCTAGGGC	CC1
SMC18.T.CTGAAGTTCTATCGCC	CC1
SMC18.T.CTGATAGCACGGTTTA	CC1
SMC18.T.CTGATAGGTATCGCAT	CC1
SMC18.T.CTGATAGGTTGCGTTA	CC1
SMC18.T.CTGATCCAGGTGATTA	CC1
SMC18.T.CTGCCTAGTGCTGTAT	CC1
SMC18.T.CTGCGGAAGGGTTCCC	CC1
SMC18.T.CTGCGGAAGTGACATA	CC1
SMC18.T.CTGCGGAAGTGCAAGC	CC1
SMC18.T.CTGCGGACAGTGACAG	CC1
SMC18.T.CTGCGGATCTAACGGT	CC1
SMC18.T.CTGCTGTCACCAGCAC	CC1
SMC18.T.CTGCTGTCATTTGCTT	CC1
SMC18.T.CTGCTGTTCAGGCGAA	CC1
SMC18.T.CTGGTCTAGTGTACCT	CC1
SMC18.T.CTGGTCTCACATAACC	CC1
SMC18.T.CTGGTCTCAGCGAACA	CC1
SMC18.T.CTGGTCTGTCTAACGT	CC1
SMC18.T.CTGTGCTTCAACACCA	CC1
SMC18.T.CTGTTTAGTAGTAGTA	CC1
SMC18.T.CTGTTTAGTTTGTTTC	CC1
SMC18.T.CTTAACTAGCGTTGCC	CC1
SMC18.T.CTTAACTAGCTCCTTC	CC1
SMC18.T.CTTAACTAGTAACCCT	CC1
SMC18.T.CTTAACTCAACACGCC	CC1
SMC18.T.CTTAACTCAAGCCATT	CC1
SMC18.T.CTTAACTGTGACCAAG	CC1
SMC18.T.CTTAACTTCGGTGTTA	CC1
SMC18.T.CTTACCGAGTGGGTTG	CC1

SMC18.T.CTTACCGGTGACTCAT	CC1
SMC18.T.CTTACCGGTTACGTCA	CC1
SMC18.T.CTTACCGTCCTAGAAC	CC1
SMC18.T.CTTAGGAAGAAACGAG	CC1
SMC18.T.CTTAGGACAATGGAAT	CC1
SMC18.T.CTTAGGACACAGCCCA	CC1
SMC18.T.CTTAGGAGTGCTTCTC	CC1
SMC18.T.CTTAGGATCCTTTCGG	CC1
SMC18.T.CTTAGGATCGGAAATA	CC1
SMC18.T.CTTCTCTAGAGTACAT	CC1
SMC18.T.CTTCTCTAGGCTCATT	CC1
SMC18.T.CTTCTCTAGTGACTCT	CC1
SMC18.T.CTTCTCTCAACGATCT	CC1
SMC18.T.CTTCTCTCAGACAAAT	CC1
SMC18.T.CTTCTCTCAGACAAGC	CC1
SMC18.T.CTTCTCTTCTACCTGC	CC1
SMC18.T.CTTGGCTAGCTAGCCC	CC1
SMC18.T.CTTGGCTGTACAAGTA	CC1
SMC18.T.CTTGGCTGTAGCACGA	CC1
SMC18.T.CTTGGCTGTTACGACT	CC1
SMC18.T.CTTGGCTTCAACGCTA	CC1
SMC18.T.CTTGGCTTCAATCACG	CC1
SMC18.T.CTTTGCGGTCCGCTGA	CC1
SMC18.T.CTTTGCGTCGTCCAGG	CC1
SMC18.T.GAAACTCAGCCTCGTG	CC1
SMC18.T.GAAACTCCATCGGTTA	CC1
SMC18.T.GAAACTCGTATAAACG	CC1
SMC18.T.GAAACTCGTTATCCGA	CC1
SMC18.T.GAAACTCTCCGCGCAA	CC1
SMC18.T.GAAATGAGTCCATGAT	CC1
SMC18.T.GAACATCAGACCTTTG	CC1
SMC18.T.GAACATCGTAGAGGAA	CC1
SMC18.T.GAACATCGTTTAGGAA	CC1
SMC18.T.GAACATCGTTTCCACC	CC1
SMC18.T.GAACATCTCATCGGAT	CC1
SMC18.T.GAACCTACAGATTGCT	CC1
SMC18.T.GAACCTATCAAAGACA	CC1
SMC18.T.GAACGGACAATCCGAT	CC1
SMC18.T.GAACGGAGTTGCGTTA	CC1
SMC18.T.GAAGCAGCAGGTTTCA	CC1
SMC18.T.GAAGCAGGTCGAGATG	CC1
SMC18.T.GAATAAGAGAAGATTC	CC1
SMC18.T.GAATAAGAGAGTAAGG	CC1
SMC18.T.GAATAAGTCTCCTATA	CC1
SMC18.T.GAATGAAGTTGGACCC	CC1
SMC18.T.GAATGAATCGTGACAT	CC1
SMC18.T.GACACGCAGCAACGGT	CC1
SMC18.T.GACACGCGTCAACTGT	CC2
SMC18.T.GACACGCGTCTCCACT	CC1
SMC18.T.GACACGCTCCCGGATG	CC1
SMC18.T.GACAGAGCAGCCAGAA	CC1
SMC18.T.GACAGAGGTACTTAGC	CC1
SMC18.T.GACAGAGGTGTTTGTG	CC1
SMC18.T.GACCAATAGTGTTGAA	CC1
SMC18.T.GACGCGTCACCAGTTA	CC1
SMC18.T.GACGCGTTCAATACCG	CC1
SMC18.T.GACGCGTTCTAACTGG	CC1
SMC18.T.GACGGCTGTCTAACGT	CC1

SMC18.T.GACGGCTGTTCTGGTA	CC1
SMC18.T.GACGTGCAGTTGCAGG	CC1
SMC18.T.GACGTGCCAGCTCGAC	CC1
SMC18.T.GACGTGCGTGAACCTT	CC1
SMC18.T.GACGTGCTCGGTGTTA	CC1
SMC18.T.GACGTTACAAGCTGTT	CC1
SMC18.T.GACGTTATCCGAGCCA	CC1
SMC18.T.GACGTTATCTATCCCG	CC1
SMC18.T.GACTACAAGTTGTAGA	CC1
SMC18.T.GACTACACAATGTAAG	CC1
SMC18.T.GACTACATCCGCAGTG	CC1
SMC18.T.GACTACATCGTGTAGT	CC1
SMC18.T.GACTGCGGTCGGGTCT	CC1
SMC18.T.GACTGCGTCTGGCGAC	CC1
SMC18.T.GAGCAGAAGCTCCCAG	CC1
SMC18.T.GAGCAGATCGGAGCAA	CC1
SMC18.T.GAGGTGAAGAAGATTC	CC1
SMC18.T.GAGGTGACAAGACACG	CC1
SMC18.T.GAGGTGAGTCCGTTAA	CC1
SMC18.T.GAGTCCGAGAGTAATC	CC1
SMC18.T.GAGTCCGCATGGGAAC	CC1
SMC18.T.GATCAGTAGGCAGTCA	CC1
SMC18.T.GATCAGTGTTAAGGGC	CC1
SMC18.T.GATCAGTGTTTGCATG	CC1
SMC18.T.GATCGATAGCTACCTA	CC1
SMC18.T.GATCGATCAAGGTTCT	CC1
SMC18.T.GATCGATTCGCAAACT	CC1
SMC18.T.GATCGCGGTGATGTCT	CC1
SMC18.T.GATCGCGTCAACACTG	CC1
SMC18.T.GATCGCGTCCTTCAAT	CC1
SMC18.T.GATCGTACACTGCCAG	CC1
SMC18.T.GATCGTAGTAATCGTC	CC1
SMC18.T.GATCTAGTCTGTCCGT	CC1
SMC18.T.GATGAAACATATGGTC	CC1
SMC18.T.GATGAAAGTAAAGTCA	CC1
SMC18.T.GATGAGGGTCACACGC	CC1
SMC18.T.GATGAGGTCCAAGTAC	CC1
SMC18.T.GATGAGGTCTGCTGTC	CC1
SMC18.T.GATGCTATCCCAACGG	CC1
SMC18.T.GATTCAGAGACTTGAA	CC1
SMC18.T.GATTCAGGTTATGCGT	CC1
SMC18.T.GATTCAGTCTTGTACT	CC1
SMC18.T.GCAAACTCAGCCTATA	CC1
SMC18.T.GCAATCAAGCACGCCT	CC1
SMC18.T.GCAATCACACCGCTAG	CC1
SMC18.T.GCAATCACAGCTGGCT	CC1
SMC18.T.GCAATCAGTTTGGCGC	CC1
SMC18.T.GCAATCATCGGAGGTA	CC1
SMC18.T.GCACATAGTCGCGGTT	CC1
SMC18.T.GCACATATCCCACTTG	CC1
SMC18.T.GCACTCTAGTCCCACG	CC1
SMC18.T.GCACTCTCATCCTTGC	CC1
SMC18.T.GCACTCTGTCATATCG	CC1
SMC18.T.GCAGCCACACCAGGCT	CC1
SMC18.T.GCAGCCACAGATGGCA	CC1
SMC18.T.GCAGCCAGTTAAGGGC	CC1
SMC18.T.GCAGCCATCAGCTGGC	CC1
SMC18.T.GCAGCCATCAGTTAGC	CC1

SMC18.T.GCAGTTAAGGCATTGG	CC1
SMC18.T.GCAGTTACATACCATG	CC1
SMC18.T.GCAGTTAGTAGCCTAT	CC1
SMC18.T.GCAGTTAGTCTTGTCC	CC1
SMC18.T.GCAGTTAGTTGCGCAC	CC1
SMC18.T.GCAGTTATCCTGCAGG	CC1
SMC18.T.GCAGTTATCGGATGTT	CC1
SMC18.T.GCATACAAGCACCGCT	CC1
SMC18.T.GCATACAAGTACGTAA	CC1
SMC18.T.GCATACACAAGTACCT	CC1
SMC18.T.GCATACAGTACTCGCG	CC1
SMC18.T.GCATACAGTATAATGG	CC1
SMC18.T.GCATGATAGTGCCATT	CC1
SMC18.T.GCATGATTCAGCCTAA	CC1
SMC18.T.GCATGATTCTCCAACC	CC1
SMC18.T.GCATGCGAGAAACCAT	CC1
SMC18.T.GCATGCGCAATAGCGG	CC1
SMC18.T.GCATGCGTCAGTTCGA	CC1
SMC18.T.GCATGTAAGCGCTCCA	CC1
SMC18.T.GCATGTACAAAGTGCG	CC1
SMC18.T.GCATGTACACAACGTT	CC1
SMC18.T.GCATGTATCCATTCTA	CC1
SMC18.T.GCATGTATCCTAGAAC	CC1
SMC18.T.GCCAAATGTTCCCGAG	CC1
SMC18.T.GCCTCTAGTAAAGTCA	CC1
SMC18.T.GCCTCTAGTCTCTTAT	CC1
SMC18.T.GCCTCTAGTTATGCGT	CC1
SMC18.T.GCGACCAGTCTGATTG	CC1
SMC18.T.GCGACCATCAACACCA	CC1
SMC18.T.GCGACCATCCAGGGCT	CC1
SMC18.T.GCGACCATCCGCGGTA	CC1
SMC18.T.GCGAGAAAGTTTAGGA	CC1
SMC18.T.GCGAGAATCGGCTTGG	CC1
SMC18.T.GCGCAACAGGCACATG	CC1
SMC18.T.GCGCAACTCATCTGTT	CC1
SMC18.T.GCGCAGTCACGCGAAA	CC1
SMC18.T.GCGCAGTCACTAAGTC	CC1
SMC18.T.GCGCAGTGTCCAGTGC	CC1
SMC18.T.GCGCAGTGTGACGCCT	CC1
SMC18.T.GCGCAGTGTGAGTATA	CC1
SMC18.T.GCGCCAAAGCGTTGCC	CC1
SMC18.T.GCGCCAACAATGACCT	CC1
SMC18.T.GCGCCAATCCAAATGC	CC1
SMC18.T.GCGCGATGTCCGTTAA	CC1
SMC18.T.GCGCGATGTCGGGTCT	CC1
SMC18.T.GCGGGTTCAGGTCTCG	CC1
SMC18.T.GCGGGTTCATTGTGCA	CC1
SMC18.T.GCGGGTTTCAGCTCTC	CC1
SMC18.T.GCGGGTTTCGTAGATC	CC1
SMC18.T.GCGGGTTTCTACTCAT	CC1
SMC18.T.GCTCCTAAGACTGTAA	CC1
SMC18.T.GCTCCTACAATGGAGC	CC1
SMC18.T.GCTCTGTAGCGTAATA	CC1
SMC18.T.GCTCTGTGTAACGTTC	CC1
SMC18.T.GCTCTGTTCCTCCTAG	CC1
SMC18.T.GCTGCAGCATTGGGCC	CC1
SMC18.T.GCTGCAGGTTATCCGA	CC1
SMC18.T.GCTGCGAAGCGCCTTG	CC1

SMC18.T.GCTGCGACAGAAGCAC	CC1
SMC18.T.GCTGCGATCGTGGACC	CC1
SMC18.T.GCTGCGATCTCGCATC	CC1
SMC18.T.GCTGGGTAGCGTTCCG	CC1
SMC18.T.GCTGGGTGTGTGAAAT	CC1
SMC18.T.GCTGGGTGTTTGACTG	CC1
SMC18.T.GCTTCCACAGTAAGCG	CC1
SMC18.T.GCTTGAAAGTCCCACG	CC1
SMC18.T.GCTTGAACAGGTTTCA	CC1
SMC18.T.GCTTGAATCACTATTC	CC1
SMC18.T.GCTTGAATCCAGGGCT	CC1
SMC18.T.GGAAAGCCAATAGCGG	CC1
SMC18.T.GGAAAGCGTCTAGTGT	CC1
SMC18.T.GGAAAGCTCGGATGGA	CC1
SMC18.T.GGAACTTAGCCCGAAA	CC1
SMC18.T.GGAACTTCAGTATCTG	CC1
SMC18.T.GGAACTTGTGTGACGA	CC1
SMC18.T.GGAATAAAGACGACGT	CC1
SMC18.T.GGAATAAAGCTCCTCT	CC1
SMC18.T.GGAATAAAGTAGATGT	CC1
SMC18.T.GGAATAAGTGCTTCTC	CC1
SMC18.T.GGACAAGAGAATTGTG	CC1
SMC18.T.GGACAAGAGCGATAGC	CC1
SMC18.T.GGACAAGCACAGACTT	CC1
SMC18.T.GGACAAGTCCAAGTAC	CC1
SMC18.T.GGACAGACAGACAAGC	CC1
SMC18.T.GGACAGAGTAGCGTGA	CC1
SMC18.T.GGACAGATCGCAAGCC	CC1
SMC18.T.GGACATTTCTTGTCAT	CC1
SMC18.T.GGACGTCAGCTAAACA	CC1
SMC18.T.GGACGTCCAAATCCGT	CC1
SMC18.T.GGACGTCCACCGAAAG	CC1
SMC18.T.GGACGTCCATCGACGC	CC1
SMC18.T.GGACGTCGTAGAGGAA	CC2
SMC18.T.GGACGTCTCACGATGT	CC1
SMC18.T.GGAGCAAAGTGTCCCG	CC1
SMC18.T.GGATGTTCAAGGACAC	CC1
SMC18.T.GGATGTTCAGTTTACG	CC1
SMC18.T.GGATGTTCATTTCAGG	CC1
SMC18.T.GGATTACCAATGGATA	CC1
SMC18.T.GGATTACGTTGGGACA	CC1
SMC18.T.GGCAATTCATACTCTT	CC1
SMC18.T.GGCCGATCAGCGATCC	CC1
SMC18.T.GGCCGATGTCCGCTGA	CC1
SMC18.T.GGCCGATGTCCGTGAC	CC1
SMC18.T.GGCCGATTCGTGACAT	CC1
SMC18.T.GGCGACTCAGGTGCCT	CC1
SMC18.T.GGCGTGTCAATGGAAT	CC1
SMC18.T.GGCGTGTCACGCGAAA	CC1
SMC18.T.GGCGTGTCAGGCTGAA	CC1
SMC18.T.GGCTCGAGTTCTGTTT	CC1
SMC18.T.GGCTGGTCAGAGTGTG	CC1
SMC18.T.GGCTGGTTCGGCTACG	CC1
SMC18.T.GGCTGGTTCGGTTCGG	CC1
SMC18.T.GGCTGGTTCGTTACGA	CC1
SMC18.T.GGGAATGAGAGCCTAG	CC1
SMC18.T.GGGAATGGTACCTACA	CC1
SMC18.T.GGGAATGTCGGCGCTA	CC1

SMC18.T.GGGAATGTCGTTTGCC	CC1
SMC18.T.GGGACCTAGCAGACTG	CC1
SMC18.T.GGGACCTCAAGCGATG	CC1
SMC18.T.GGGAGATCACTTCTGC	CC1
SMC18.T.GGGAGATGTACCAGTT	CC1
SMC18.T.GGGAGATTCATCGATG	CC1
SMC18.T.GGGATGAAGACAATAC	CC1
SMC18.T.GGGATGATCAACTCTT	CC1
SMC18.T.GGGATGATCGTTACGA	CC1
SMC18.T.GGGCACTAGATCGATA	CC1
SMC18.T.GGGCACTGTCTAGAGG	CC1
SMC18.T.GGGCATCAGGAATGGA	CC1
SMC18.T.GGGCATCCACGTAAGG	CC1
SMC18.T.GGGCATCGTTTCCACC	CC1
SMC18.T.GGGCATCTCAACACGT	CC1
SMC18.T.GGGTCTGCACAAGCCC	CC1
SMC18.T.GGGTCTGGTAGCAAAT	CC1
SMC18.T.GGGTCTGGTAGCGCAA	CC1
SMC18.T.GGGTCTGTCATCTGTT	CC1
SMC18.T.GGGTTGCCAAACTGTC	CC1
SMC18.T.GGGTTGCCATGCTAGT	CC1
SMC18.T.GGGTTGCCATGGTAGG	CC1
SMC18.T.GGGTTGCGTTGTTTGG	CC1
SMC18.T.GGTATTGAGAAGAAGC	CC1
SMC18.T.GGTATTGCATCCGTGG	CC1
SMC18.T.GGTATTGTCCACGAAT	CC1
SMC18.T.GGTATTGTCCAGAAGG	CC1
SMC18.T.GGTATTGTCCCAGGTG	CC1
SMC18.T.GGTATTGTCCGAAGAG	CC1
SMC18.T.GGTGAAGAGTTAGCGG	CC1
SMC18.T.GGTGAAGGTGGCGAAT	CC1
SMC18.T.GGTGAAGGTTCCTCCA	CC1
SMC18.T.GGTGCGTAGCAGCGTA	CC1
SMC18.T.GGTGTTAAGCCACGTC	CC1
SMC18.T.GGTGTTAAGCGTAGTG	CC1
SMC18.T.GGTGTTAAGTCTCAAC	CC1
SMC18.T.GGTGTTACATCTATGG	CC1
SMC18.T.GGTGTTAGTCTCACCT	CC1
SMC18.T.GGTGTTAGTGGTGTAG	CC1
SMC18.T.GTAACGTAGGCTAGAC	CC1
SMC18.T.GTAACGTGTAGCGTAG	CC1
SMC18.T.GTAACGTGTATCTGCA	CC1
SMC18.T.GTAACTGTCGAATCCA	CC1
SMC18.T.GTAACTGTCGCCTGTT	CC1
SMC18.T.GTAACTGTCGGAAACG	CC1
SMC18.T.GTAACTGTCGTGGGAA	CC1
SMC18.T.GTACGTAAGGCTAGGT	CC1
SMC18.T.GTACGTACATCACCCT	CC1
SMC18.T.GTACGTAGTCAGTGGA	CC1
SMC18.T.GTACGTAGTTGGTTTG	CC2
SMC18.T.GTACTCCAGGAGTACC	CC1
SMC18.T.GTACTCCCAAGTAGTA	CC1
SMC18.T.GTACTCCCAATTCCTT	CC1
SMC18.T.GTACTCCGTGGTAACG	CC1
SMC18.T.GTACTTTAGAGATGAG	CC1
SMC18.T.GTACTTTGTAGTACCT	CC1
SMC18.T.GTACTTTGTGTGAAAT	CC1
SMC18.T.GTAGGCCCACATGACT	CC1

SMC18.T.GTAGGCCCAGGTTTCA	CC1
SMC18.T.GTAGGCCTCCGCGTTT	CC1
SMC18.T.GTAGGCCTCTCATTCA	CC1
SMC18.T.GTAGTCAAGTACGATA	CC1
SMC18.T.GTATCTTGTCGGCACT	CC1
SMC18.T.GTATCTTTCCAGTAGT	CC1
SMC18.T.GTATCTTTCTATCGCC	CC1
SMC18.T.GTATTCTCAACACGCC	CC1
SMC18.T.GTATTCTCACACATGT	CC1
SMC18.T.GTATTCTGTTGTGGAG	CC1
SMC18.T.GTCAAGTCATAGACTC	CC1
SMC18.T.GTCAAGTGTCATATCG	CC1
SMC18.T.GTCAAGTGTTCACCTC	CC1
SMC18.T.GTCAAGTTCTGTTGAG	CC1
SMC18.T.GTCACAACACAGATTC	CC1
SMC18.T.GTCACAACAGCGAACA	CC1
SMC18.T.GTCACAACAGCTCCGA	CC1
SMC18.T.GTCACAATCCATGAAC	CC1
SMC18.T.GTCACAATCCGATATG	CC1
SMC18.T.GTCACAATCCTTAATC	CC1
SMC18.T.GTCACAATCTTATCTG	CC1
SMC18.T.GTCACGGCAAGTTAAG	CC1
SMC18.T.GTCACGGCAGATAATG	CC1
SMC18.T.GTCACGGCAGTTCCCT	CC1
SMC18.T.GTCACGGCATTCCTCG	CC1
SMC18.T.GTCACGGGTCGGGTCT	CC1
SMC18.T.GTCACGGGTTCTGTTT	CC1
SMC18.T.GTCACGGTCCACGACG	CC1
SMC18.T.GTCACGGTCGCCTGTT	CC1
SMC18.T.GTCACGGTCTGCTGCT	CC1
SMC18.T.GTCATTTCAGACGCTC	CC1
SMC18.T.GTCCTCAAGAAACGAG	CC1
SMC18.T.GTCCTCAAGCAAATCA	CC1
SMC18.T.GTCCTCAAGCGTTCCG	CC1
SMC18.T.GTCCTCAAGGTAGCCA	CC1
SMC18.T.GTCCTCACAAAGGCGT	CC1
SMC18.T.GTCCTCACAAGTCATC	CC1
SMC18.T.GTCCTCATCCATGCTC	CC1
SMC18.T.GTCGGGTCAGTCCTTC	CC1
SMC18.T.GTCGGGTGTAAATGTG	CC1
SMC18.T.GTCGGGTTCTTCCTTC	CC1
SMC18.T.GTCGTAAAGAGCTATA	CC1
SMC18.T.GTCGTAAGTTCACCTC	CC1
SMC18.T.GTCTCGTAGATGTGTA	CC1
SMC18.T.GTCTCGTAGCCCGAAA	CC1
SMC18.T.GTCTCGTCAATGGATA	CC1
SMC18.T.GTCTCGTGTCATGCAT	CC1
SMC18.T.GTCTCGTGTTATTCTC	CC1
SMC18.T.GTCTCGTTCTGGTGTA	CC1
SMC18.T.GTCTTCGAGCTTCGCG	CC1
SMC18.T.GTCTTCGCACAGACAG	CC1
SMC18.T.GTCTTCGTCCTGCCAT	CC1
SMC18.T.GTGAAGGTCATTGCCC	CC1
SMC18.T.GTGCAGCAGTTAACGA	CC1
SMC18.T.GTGCAGCCAATGTTGC	CC1
SMC18.T.GTGCAGCCAGTCGATT	CC1
SMC18.T.GTGCATAAGGCATGGT	CC1
SMC18.T.GTGCGGTAGCCAGAAC	CC1

SMC18.T.GTGCGGTAGGATGGTC	CC1
SMC18.T.GTGCGGTCAGCCAGAA	CC1
SMC18.T.GTGCGGTTCGAATGGG	CC1
SMC18.T.GTGCTTCCATGGGAAC	CC2
SMC18.T.GTGCTTCTCTAACCGA	CC1
SMC18.T.GTGCTTCTCTCGGACG	CC1
SMC18.T.GTGGGTCGTTGGTTTG	CC1
SMC18.T.GTGTGCGAGCCTTGAT	CC1
SMC18.T.GTGTTAGAGTCATGCT	CC1
SMC18.T.GTGTTAGAGTGGTAAT	CC1
SMC18.T.GTGTTAGAGTGTGGCA	CC1
SMC18.T.GTGTTAGCAGTCAGCC	CC1
SMC18.T.GTGTTAGGTTACAGAA	CC1
SMC18.T.GTTAAGCAGTACGTAA	CC1
SMC18.T.GTTAAGCAGTCCTCCT	CC1
SMC18.T.GTTAAGCCAGGACGTA	CC1
SMC18.T.GTTAAGCCATGGTTGT	CC1
SMC18.T.GTTAAGCGTAAACACA	CC1
SMC18.T.GTTAAGCTCCAGTAGT	CC1
SMC18.T.GTTACAGCAAATACAG	CC1
SMC18.T.GTTACAGGTACCGCTG	CC1
SMC18.T.GTTACAGTCGGGAGTA	CC1
SMC18.T.GTTCATTCAGGTTTCA	CC1
SMC18.T.GTTCATTGTTTGTTGG	CC1
SMC18.T.GTTCGGGAGCTAAACA	CC1
SMC18.T.GTTCGGGCAAGAGGCT	CC1
SMC18.T.GTTCGGGGTCTTGCGG	CC1
SMC18.T.GTTCTCGAGACGCACA	CC1
SMC18.T.GTTCTCGAGCCGATTT	CC1
SMC18.T.GTTCTCGTCCCTTGTG	CC1
SMC18.T.TAAACCGCACCACCAG	CC1
SMC18.T.TAAACCGCACCGGAAA	CC1
SMC18.T.TAAACCGTCCTGCCAT	CC1
SMC18.T.TAAGAGAAGCTGTTCA	CC1
SMC18.T.TAAGAGAGTCGAACAG	CC1
SMC18.T.TAAGAGAGTTTGTGTG	CC1
SMC18.T.TAAGCGTAGAAGGTGA	CC1
SMC18.T.TAAGCGTAGATGCGAC	CC1
SMC18.T.TAAGCGTCATGCAACT	CC1
SMC18.T.TAAGCGTGTGAGCGAT	CC1
SMC18.T.TAAGTGCCACGAGAGT	CC1
SMC18.T.TAAGTGCCATCCGCGA	CC1
SMC18.T.TAAGTGCGTAGCGCTC	CC1
SMC18.T.TAAGTGCTCCAAGCCG	CC1
SMC18.T.TACACGAAGAGTACAT	CC1
SMC18.T.TACACGATCATTTGGG	CC1
SMC18.T.TACAGTGAGATGTAAC	CC1
SMC18.T.TACAGTGCAAGTCTAC	CC1
SMC18.T.TACCTATAGAAGCCCA	CC1
SMC18.T.TACCTATAGCGATAGC	CC1
SMC18.T.TACCTATCACATCCGG	CC1
SMC18.T.TACCTATTCCTCGCAT	CC1
SMC18.T.TACCTTAAGATGCGAC	CC1
SMC18.T.TACCTTAAGCAATCTC	CC1
SMC18.T.TACCTTAAGCCACGCT	CC1
SMC18.T.TACCTTAAGGAGTCTG	CC1
SMC18.T.TACCTTAAGGGATGGG	CC1
SMC18.T.TACCTTAAGTACATGA	CC1

SMC18.T.TACCTTATCTTGACGA	CC1
SMC18.T.TACGGATAGGGCACTA	CC1
SMC18.T.TACGGATGTCCTGCTT	CC1
SMC18.T.TACGGATTCAAACCGT	CC1
SMC18.T.TACGGATTCTCGATGA	CC1
SMC18.T.TACGGGCAGAGGGCTT	CC1
SMC18.T.TACGGGCCACCTTGTC	CC1
SMC18.T.TACGGGCTCCTAGTGA	CC1
SMC18.T.TACGGTAAGAAACCTA	CC1
SMC18.T.TACGGTAAGCTCCTTC	CC1
SMC18.T.TACGGTACAAGCTGAG	CC1
SMC18.T.TACGGTAGTCTCACCT	CC1
SMC18.T.TACGGTAGTCTGATCA	CC1
SMC18.T.TACTCATTCGTGTAGT	CC1
SMC18.T.TACTCGCTCCGAATGT	CC1
SMC18.T.TACTTACCAGGAACGT	CC1
SMC18.T.TACTTACTCAGCGATT	CC1
SMC18.T.TACTTACTCGGTTAAC	CC1
SMC18.T.TACTTGTAGGATGGAA	CC1
SMC18.T.TACTTGTAGGCGATAC	CC1
SMC18.T.TAGACCAAGTGCGATG	CC1
SMC18.T.TAGACCAGTTCAACCA	CC1
SMC18.T.TAGAGCTAGTCATCCA	CC1
SMC18.T.TAGAGCTCATTCACTT	CC1
SMC18.T.TAGCCGGCAACGCACC	CC1
SMC18.T.TAGCCGGCAGTCACTA	CC1
SMC18.T.TAGCCGGGTATAATGG	CC1
SMC18.T.TAGCCGGGTATATGGA	CC1
SMC18.T.TAGCCGGTCCGAGCCA	CC1
SMC18.T.TAGCCGGTCGCGCCAA	CC1
SMC18.T.TAGGCATAGTAATCCC	CC1
SMC18.T.TAGGCATTCGTAGATC	CC1
SMC18.T.TAGGCATTCTCTGTCG	CC1
SMC18.T.TAGTGGTAGGTCATCT	CC1
SMC18.T.TAGTGGTCACTGTTAG	CC1
SMC18.T.TAGTGGTTCTGTTGAG	CC1
SMC18.T.TAGTTGGAGAAGGTTT	CC1
SMC18.T.TAGTTGGCATTGGTAC	CC1
SMC18.T.TATCAGGAGAGGTTGC	CC1
SMC18.T.TATCAGGAGCTGAACG	CC1
SMC18.T.TATCAGGTCAAACAAG	CC1
SMC18.T.TATCTCAAGATGCCAG	CC1
SMC18.T.TATCTCAAGATGGGTC	CC1
SMC18.T.TATCTCACACATGGGA	CC1
SMC18.T.TATCTCACACCGATAT	CC1
SMC18.T.TATCTCACATAGAAAC	CC1
SMC18.T.TATCTCATCAGCTCTC	CC1
SMC18.T.TATGCCCCATCACAAC	CC1
SMC18.T.TATGCCCCATGTCTCC	CC1
SMC18.T.TATGCCCGTTACGCGC	CC1
SMC18.T.TCAACGACACAGACAG	CC1
SMC18.T.TCAACGACACCTATCC	CC1
SMC18.T.TCAACGAGTAAGGATT	CC1
SMC18.T.TCAACGATCTGGTTCC	CC1
SMC18.T.TCAATCTAGTCTCGGC	CC1
SMC18.T.TCAATCTCAAGAGGCT	CC1
SMC18.T.TCAATCTTCAGATAAG	CC1
SMC18.T.TCACAAGAGAAACGCC	CC1

SMC18.T.TCACAAGTCAGTTTGG	CC1
SMC18.T.TCACAAGTCGGATGGA	CC1
SMC18.T.TCACAAGTCTCGAGTA	CC1
SMC18.T.TCACGAAAGATCCCAT	CC1
SMC18.T.TCACGAATCAGCAACT	CC1
SMC18.T.TCAGATGGTCCGTGAC	CC1
SMC18.T.TCAGCAATCACGGTTA	CC1
SMC18.T.TCAGCAATCTCCAGGG	CC1
SMC18.T.TCAGCTCAGATCTGCT	CC1
SMC18.T.TCAGCTCCAATTGCTG	CC1
SMC18.T.TCAGCTCCACCAGGTC	CC1
SMC18.T.TCAGCTCCACTGTGTA	CC1
SMC18.T.TCAGCTCGTCAACATC	CC1
SMC18.T.TCAGCTCGTCATATGC	CC1
SMC18.T.TCAGCTCTCCATGAAC	CC1
SMC18.T.TCAGCTCTCCATGAGT	CC1
SMC18.T.TCAGCTCTCGCCAAAT	CC1
SMC18.T.TCAGGATCAAGGTGTG	CC1
SMC18.T.TCAGGATGTCTTTCAT	CC1
SMC18.T.TCAGGATTCAACCAAC	CC1
SMC18.T.TCAGGATTCGAGAGCA	CC1
SMC18.T.TCAGGTAAGATCTGCT	CC1
SMC18.T.TCAGGTAAGTAGGCCA	CC1
SMC18.T.TCAGGTAGTATAATGG	CC1
SMC18.T.TCAGGTATCGCGATCG	CC1
SMC18.T.TCATTACAGAATTCCC	CC1
SMC18.T.TCATTACCAAGCCATT	CC1
SMC18.T.TCATTACTCGGGAGTA	CC1
SMC18.T.TCATTTGAGATAGCAT	CC1
SMC18.T.TCATTTGCAATAGCGG	CC1
SMC18.T.TCATTTGGTAAGGATT	CC1
SMC18.T.TCATTTGGTATAGGTA	CC1
SMC18.T.TCATTTGGTCCGTGAC	CC1
SMC18.T.TCCACACCACAGAGGT	CC1
SMC18.T.TCCACACCAGCTCGCA	CC1
SMC18.T.TCCACACCATCACCCT	CC1
SMC18.T.TCCACACTCGGTCCGA	CC1
SMC18.T.TCGAGGCCAGCAGTTT	CC1
SMC18.T.TCGAGGCCAGTCTTCC	CC1
SMC18.T.TCGAGGCGTCAGATAA	CC1
SMC18.T.TCGAGGCTCTCTTATG	CC1
SMC18.T.TCGCGAGAGCTGCAAG	CC1
SMC18.T.TCGCGAGAGTGCCATT	CC1
SMC18.T.TCGCGAGCAGACAAAT	CC1
SMC18.T.TCGCGAGGTTCGGGCT	CC1
SMC18.T.TCGCGTTCATAAGACA	CC1
SMC18.T.TCGCGTTGTCATCGGC	CC1
SMC18.T.TCGGGACCAAACGTGG	CC1
SMC18.T.TCGGGACCACTCAGGC	CC1
SMC18.T.TCGGGACGTGTCAATC	CC1
SMC18.T.TCGGTAACACGGTTTA	CC1
SMC18.T.TCGGTAAGTTAAAGTG	CC1
SMC18.T.TCGTACCAGCCCTAAT	CC1
SMC18.T.TCGTACCAGTTCGCGC	CC1
SMC18.T.TCGTACCCAAGCGAGT	CC1
SMC18.T.TCGTAGAAGATGGGTC	CC1
SMC18.T.TCGTAGAAGCCCAACC	CC1
SMC18.T.TCGTAGAAGGGTGTGT	CC1

SMC18.T.TCGTAGACAATTGCTG	CC1
SMC18.T.TCGTAGATCCCATTTA	CC1
SMC18.T.TCGTAGATCTTCGGTC	CC1
SMC18.T.TCTATTGAGAACAACT	CC1
SMC18.T.TCTATTGAGAGGACGG	CC1
SMC18.T.TCTATTGAGATTACCC	CC1
SMC18.T.TCTATTGAGCCTTGAT	CC1
SMC18.T.TCTATTGGTGTTCTTT	CC1
SMC18.T.TCTCATAGTTGTCTTT	CC1
SMC18.T.TCTCTAAGTGACTCAT	CC1
SMC18.T.TCTCTAAGTGATGTGG	CC1
SMC18.T.TCTGAGACATGGAATA	CC1
SMC18.T.TCTGGAACACCACCAG	CC1
SMC18.T.TCTGGAATCTCACATT	CC1
SMC18.T.TCTTCGGAGCGATATA	CC1
SMC18.T.TCTTCGGCAATAACGA	CC1
SMC18.T.TCTTCGGCACCAGGCT	CC1
SMC18.T.TCTTCGGGTAATCGTC	CC1
SMC18.T.TCTTCGGGTACAGACG	CC1
SMC18.T.TCTTTCCGTCCGACGT	CC1
SMC18.T.TCTTTCCGTCTAGTCA	CC1
SMC18.T.TCTTTCCTCGGCTTGG	CC1
SMC18.T.TCTTTCCTCTGGTTCC	CC1
SMC18.T.TGAAAGAAGGCTCTTA	CC1
SMC18.T.TGAAAGATCCACTGGG	CC1
SMC18.T.TGACAACAGGTGATAT	CC1
SMC18.T.TGACAACTCCCTTGTG	CC1
SMC18.T.TGACGGCCAAGTACCT	CC1
SMC18.T.TGACGGCCACGAGGTA	CC1
SMC18.T.TGACGGCGTCGCATAT	CC1
SMC18.T.TGACGGCGTGACCAAG	CC1
SMC18.T.TGACGGCGTGTTTGTG	CC1
SMC18.T.TGACGGCTCACGCGGT	CC1
SMC18.T.TGACTAGAGTTACGGG	CC1
SMC18.T.TGACTAGCATCTACGA	CC1
SMC18.T.TGACTTTCATCAGTAC	CC1
SMC18.T.TGAGAGGAGGCCGAAT	CC1
SMC18.T.TGAGAGGCAGGCTGAA	CC1
SMC18.T.TGAGCATAGACTAAGT	CC1
SMC18.T.TGAGCCGAGTACGTTC	CC2
SMC18.T.TGAGCCGGTACCTACA	CC1
SMC18.T.TGAGCCGGTGCCTGTG	CC1
SMC18.T.TGAGGGACATCGGAAG	CC1
SMC18.T.TGATTTCAGTGCTGCC	CC1
SMC18.T.TGATTTCGTAGAAAGG	CC1
SMC18.T.TGATTTCTCCCTTGTG	CC1
SMC18.T.TGATTTCTCGTGTAGT	CC1
SMC18.T.TGCACCTAGTGGGTTG	CC1
SMC18.T.TGCACCTCAACTGCTA	CC1
SMC18.T.TGCACCTCATTTGCTT	CC1
SMC18.T.TGCACCTGTGTGCGTC	CC1
SMC18.T.TGCACCTTCTCGGACG	CC1
SMC18.T.TGCCAAAAGATGCGAC	CC1
SMC18.T.TGCCCATCAAGCGCTC	CC1
SMC18.T.TGCCCATCACAACGTT	CC1
SMC18.T.TGCCCATGTTTACTCT	CC1
SMC18.T.TGCCCATTCCGCATCT	CC1
SMC18.T.TGCCCTAGTCAAAGCG	CC1

SMC18.T.TGCCCTATCAGAGACG	CC1
SMC18.T.TGCGCAGAGTAGGCCA	CC1
SMC18.T.TGCGCAGCAAGCGCTC	CC1
SMC18.T.TGCGCAGCAGTCGATT	CC1
SMC18.T.TGCGCAGCATCGGGTC	CC1
SMC18.T.TGCGCAGCATTGGGCC	CC1
SMC18.T.TGCGCAGGTGAAATCA	CC1
SMC18.T.TGCGCAGTCCAGGGCT	CC1
SMC18.T.TGCGCAGTCTGCCCTA	CC1
SMC18.T.TGCGGGTAGAATTCCC	CC1
SMC18.T.TGCGGGTCAGTTTACG	CC1
SMC18.T.TGCGTGGAGACATAAC	CC1
SMC18.T.TGCGTGGAGCGATCCC	CC1
SMC18.T.TGCGTGGCATTGGGCC	CC1
SMC18.T.TGCGTGGGTCTCTTAT	CC1
SMC18.T.TGCGTGGTCCGTAGGC	CC1
SMC18.T.TGCTACCAGTTCCACA	CC1
SMC18.T.TGCTACCGTACCTACA	CC1
SMC18.T.TGCTACCTCTGTCTCG	CC1
SMC18.T.TGCTGCTCAATCGAAA	CC1
SMC18.T.TGCTGCTTCGGTGTTA	CC1
SMC18.T.TGGACGCGTTCCCTTG	CC1
SMC18.T.TGGACGCTCAACACGT	CC1
SMC18.T.TGGACGCTCCAACCAA	CC1
SMC18.T.TGGACGCTCGAGAGCA	CC1
SMC18.T.TGGACGCTCTGATTCT	CC1
SMC18.T.TGGCCAGAGATCTGAA	CC1
SMC18.T.TGGCCAGAGTGCCAGA	CC1
SMC18.T.TGGCCAGCATCACCCT	CC1
SMC18.T.TGGCCAGTCTATCGCC	CC1
SMC18.T.TGGCGCAAGGTGACCA	CC1
SMC18.T.TGGCGCAGTCAATGTC	CC1
SMC18.T.TGGCGCATCTCTGTCG	CC1
SMC18.T.TGGGAAGGTCCGCTGA	CC1
SMC18.T.TGGGAAGTCAGCGACC	CC1
SMC18.T.TGGGCGTAGCGCCTCA	CC1
SMC18.T.TGGGCGTAGGACCACA	CC1
SMC18.T.TGGGCGTCACCATCCT	CC1
SMC18.T.TGGGCGTGTTCAGACT	CC1
SMC18.T.TGGGCGTTCAAGGCTT	CC1
SMC18.T.TGGTTAGAGAAGGACA	CC2
SMC18.T.TGGTTAGAGCGCCTCA	CC1
SMC18.T.TGGTTAGGTCCATGAT	CC1
SMC18.T.TGGTTAGGTCTCACCT	CC1
SMC18.T.TGGTTAGTCATGCATG	CC1
SMC18.T.TGGTTAGTCTGAGGGA	CC1
SMC18.T.TGGTTCCAGATAGCAT	CC1
SMC18.T.TGGTTCCAGCTGCCCA	CC1
SMC18.T.TGGTTCCAGTGTCCCG	CC1
SMC18.T.TGGTTCCAGTTACCCA	CC1
SMC18.T.TGGTTCCGTGTTCGAT	CC1
SMC18.T.TGTATTCAGTCTCGGC	CC1
SMC18.T.TGTATTCGTAGCCTAT	CC1
SMC18.T.TGTATTCGTGATAAAC	CC1
SMC18.T.TGTCCCAAGCCGCCTA	CC1
SMC18.T.TGTCCCAAGCGATCCC	CC1
SMC18.T.TGTCCCAAGGGCATGT	CC1
SMC18.T.TGTCCCACAGCTCCGA	CC1

SMC18.T.TGTCCCACATCAGTCA	CC1
SMC18.T.TGTCCCACATTCTCAT	CC1
SMC18.T.TGTCCCAGTGCGCTTG	CC1
SMC18.T.TGTGGTAGTAAATGAC	CC1
SMC18.T.TGTGTTTAGAGTACCG	CC1
SMC18.T.TGTGTTTAGTATCGAA	CC1
SMC18.T.TGTGTTTCAGCCTATA	CC1
SMC18.T.TGTGTTTGTAACGACG	CC1
SMC18.T.TGTGTTTGTAATCGTC	CC1
SMC18.T.TGTTCCGCACCATCCT	CC1
SMC18.T.TGTTCCGTCGTTGACA	CC1
SMC18.T.TTAACTCAGCCCAATT	CC1
SMC18.T.TTAACTCCACTGTTAG	CC1
SMC18.T.TTAACTCTCCTGCCAT	CC1
SMC18.T.TTAGGACAGAGTTGGC	CC1
SMC18.T.TTAGGACAGCTACCGC	CC1
SMC18.T.TTAGGCAAGGGTATCG	CC1
SMC18.T.TTAGGCACATCTCGCT	CC1
SMC18.T.TTAGGCAGTCCGACGT	CC1
SMC18.T.TTAGGCAGTCCGTGAC	CC1
SMC18.T.TTAGGCAGTTGATTGC	CC1
SMC18.T.TTAGGCATCACGCATA	CC1
SMC18.T.TTAGGCATCTCTAGGA	CC1
SMC18.T.TTAGTTCTCACAATGC	CC1
SMC18.T.TTATGCTAGCTGAAAT	CC1
SMC18.T.TTCCCAGGTCAGGACA	CC1
SMC18.T.TTCCCAGGTGCCTGCA	CC1
SMC18.T.TTCCCAGTCAAGCCTA	CC1
SMC18.T.TTCCCAGTCGTACGGC	CC1
SMC18.T.TTCGAAGAGAATAGGG	CC1
SMC18.T.TTCGAAGGTAGGCATG	CC1
SMC18.T.TTCGAAGTCTTACCTA	CC1
SMC18.T.TTCGGTCCAAGGACAC	CC1
SMC18.T.TTCGGTCCAGACGCAA	CC1
SMC18.T.TTCGGTCGTGAGGGTT	CC1
SMC18.T.TTCGGTCTCAGTCCCT	CC1
SMC18.T.TTCTACACACACGCTG	CC1
SMC18.T.TTCTACACACGCTTTC	CC1
SMC18.T.TTCTACAGTCTGCAAT	CC1
SMC18.T.TTCTACATCCTGTACC	CC1
SMC18.T.TTCTCAAAGAGGTTGC	CC1
SMC18.T.TTCTCCTCAAGAGGCT	CC1
SMC18.T.TTCTCCTCAGGTCCAC	CC1
SMC18.T.TTCTCCTGTCTCATCC	CC1
SMC18.T.TTCTTAGAGTCTCAAC	CC1
SMC18.T.TTGAACGAGGCTCAGA	CC1
SMC18.T.TTGAACGTCCCTAACC	CC1
SMC18.T.TTGACTTCAGGAACGT	CC1
SMC18.T.TTGACTTCAGTATCTG	CC1
SMC18.T.TTGACTTGTCATATGC	CC1
SMC18.T.TTGACTTTCACGAAGG	CC1
SMC18.T.TTGCCGTCATGCCCGA	CC1
SMC18.T.TTGCCGTTCCCAAGTA	CC1
SMC18.T.TTGCGTCCATCCGTGG	CC1
SMC18.T.TTGGAACAGTAAGTAC	CC1
SMC18.T.TTGGCAATCACTTCAT	CC1
SMC18.T.TTGTAGGTCACTCCTG	CC1
SMC18.T.TTTACTGAGCCGATTT	CC1

SMC18.T.TTTACTGCAGTTAACC	CC1
SMC18.T.TTTATGCGTTTAAGCC	CC1
SMC18.T.TTTATGCTCAACGCTA	CC1
SMC18.T.TTTCCTCAGCTAAGAT	CC1
SMC18.T.TTTCCTCCAAGTTGTC	CC1
SMC18.T.TTTCCTCCACCCTATC	CC1
SMC18.T.TTTCCTCCATGTTCCC	CC1
SMC18.T.TTTCCTCTCCAAACAC	CC1
SMC18.T.TTTGCGCGTCAAGCGA	CC1
SMC18.T.TTTGCGCGTCTAGTCA	CC1
SMC18.T.TTTGCGCTCCTATGTT	CC1
SMC18.T.TTTGGTTAGAGCCTAG	CC1
SMC18.T.TTTGGTTAGATGTGTA	CC1
SMC18.T.TTTGGTTCATGTTCCC	CC1
SMC18.T.TTTGGTTGTGATGTCT	CC1
SMC18.T.TTTGGTTGTGGCCCTA	CC1
SMC18.T.TTTGGTTTCCACGACG	CC1
SMC18.T.TTTGTCAAGACTGGGT	CC1
SMC18.T.TTTGTCAAGCGTAATA	CC1
SMC18.T.TTTGTCAAGGTGATTA	CC1
SMC18.T.TTTGTCACAACAACCT	CC1
SMC18.T.TTTGTCACACTTCGAA	CC1
SMC19.T.AAACGGGAGGGCTCTC	CC1
SMC19.T.AAACGGGGTGGTCTCG	CC1
SMC19.T.AAAGTAGAGTCGAGTG	CC1
SMC19.T.AAATGCCAGCCACCTG	CC2
SMC19.T.AAATGCCCAATAGCAA	CC1
SMC19.T.AAATGCCCAGTATGCT	CC2
SMC19.T.AAATGCCCATGTTCCC	CC1
SMC19.T.AAATGCCGTAGGCTGA	CC1
SMC19.T.AAATGCCTCAGTCCCT	CC1
SMC19.T.AACACGTAGCTGCAAG	CC2
SMC19.T.AACCATGTCGGTCTAA	CC2
SMC19.T.AACCGCGTCTCCTATA	CC1
SMC19.T.AACTCAGGTAGCGCAA	CC1
SMC19.T.AACTCTTTCTAACCGA	CC2
SMC19.T.AAGACCTTCTACCAGA	CC2
SMC19.T.AAGCCGCGTCAGGACA	CC2
SMC19.T.AAGGAGCCATAGGATA	CC2
SMC19.T.AAGGTTCAGGGAGTAA	CC2
SMC19.T.AAGTCTGCACAACTGT	CC2
SMC19.T.AAGTCTGTCGTCCAGG	CC2
SMC19.T.AATCCAGTCATTCACT	CC2
SMC19.T.AATCCAGTCTGCTTGC	CC1
SMC19.T.AATCGGTAGACGCACA	CC2
SMC19.T.ACACCCTCAATGACCT	CC1
SMC19.T.ACATACGAGGATTCGG	CC2
SMC19.T.ACATACGGTACCAGTT	CC2
SMC19.T.ACATCAGCATGTTCCC	CC1
SMC19.T.ACATGGTCAAAGAATC	CC2
SMC19.T.ACATGGTCAACAACCT	CC1
SMC19.T.ACGAGCCAGAGACGAA	CC2
SMC19.T.ACGAGGACAGTACACT	CC1
SMC19.T.ACGATACCAAGTCTAC	CC1
SMC19.T.ACGCAGCGTGCAGACA	CC1
SMC19.T.ACGCCAGCAATGGTCT	CC1
SMC19.T.ACGGAGAAGAGTGACC	CC2
SMC19.T.ACGGAGACAAACGCGA	CC2

SMC19.T.ACGGCCAGTGCGATAG	CC1
SMC19.T.ACGGGTCTCGACCAGC	CC1
SMC19.T.ACGTCAACACGCCAGT	CC1
SMC19.T.ACTGAGTGTCGCGAAA	CC2
SMC19.T.ACTGAGTGTCTAACGT	CC2
SMC19.T.ACTGCTCGTGAGGGAG	CC2
SMC19.T.ACTTTCACATCGGACC	CC2
SMC19.T.AGACGTTGTCCCTACT	CC2
SMC19.T.AGAGCGACAAGACGTG	CC2
SMC19.T.AGAGCTTTCCCTAACC	CC1
SMC19.T.AGAGTGGGTCCGTGAC	CC2
SMC19.T.AGATCTGGTCAATACC	CC1
SMC19.T.AGATTGCGTAAATACG	CC1
SMC19.T.AGCCTAACACTATCTT	CC1
SMC19.T.AGCTCCTAGGGTATCG	CC1
SMC19.T.AGCTCTCAGTAAGTAC	CC1
SMC19.T.AGCTCTCTCTTGCATT	CC1
SMC19.T.AGGCCACAGCAGGTCA	CC1
SMC19.T.AGGCCGTCAATCAGAA	CC1
SMC19.T.AGGCCGTTCACGAAGG	CC1
SMC19.T.AGGGATGGTTTAGCTG	CC1
SMC19.T.AGGGTGACAGTGACAG	CC1
SMC19.T.AGGTCATGTACTTGAC	CC2
SMC19.T.AGGTCCGAGTACGACG	CC1
SMC19.T.AGGTCCGCACGAAACG	CC2
SMC19.T.AGGTCCGGTGTGAATA	CC1
SMC19.T.AGTAGTCAGCTGATAA	CC2
SMC19.T.AGTCTTTGTTCCACTC	CC2
SMC19.T.AGTTGGTGTCTAGTCA	CC2
SMC19.T.ATAACGCCAAGTCTAC	CC1
SMC19.T.ATCGAGTAGCTAACAA	CC1
SMC19.T.ATCTACTGTAAGGGAA	CC2
SMC19.T.ATCTACTTCGCAAGCC	CC1
SMC19.T.ATCTACTTCTGCAAGT	CC2
SMC19.T.ATGCGATCACGCCAGT	CC1
SMC19.T.ATGCGATCACTAGTAC	CC2
SMC19.T.ATGCGATTCCAAACAC	CC2
SMC19.T.ATTACTCAGCACCGCT	CC1
SMC19.T.ATTACTCGTAGAAAGG	CC1
SMC19.T.ATTACTCGTATAGTAG	CC1
SMC19.T.ATTGGACAGGGTGTTG	CC1
SMC19.T.CAACCAACATCCGTGG	CC1
SMC19.T.CACAAACGTGGGTATG	CC1
SMC19.T.CACACAAAGCTCCTCT	CC2
SMC19.T.CACAGGCAGCTACCTA	CC1
SMC19.T.CACAGGCCAGTTTACG	CC1
SMC19.T.CACATAGTCCTGCAGG	CC2
SMC19.T.CACCACTTCTGATACG	CC2
SMC19.T.CACCAGGTCACTGGGC	CC1
SMC19.T.CAGCAGCAGGATATAC	CC2
SMC19.T.CAGCAGCAGTTCGCAT	CC2
SMC19.T.CAGCAGCGTCTGGAGA	CC1
SMC19.T.CAGGTGCCATGTTGAC	CC1
SMC19.T.CAGTAACGTGGTCCGT	CC1
SMC19.T.CAGTAACTCTTCAACT	CC1
SMC19.T.CATATTCCAACACGCC	CC2
SMC19.T.CATATTCCATGTTGAC	CC1
SMC19.T.CATATTCGTGGCTCCA	CC1

SMC19.T.CATATTCTCGTGACAT	CC1
SMC19.T.CATCAAGAGGGTGTTG	CC2
SMC19.T.CATCAAGGTCACCTAA	CC2
SMC19.T.CATCAGAAGAGTAAGG	CC1
SMC19.T.CATCAGATCTCCGGTT	CC2
SMC19.T.CATCCACTCCTGTAGA	CC1
SMC19.T.CATCGGGCAAAGTCAA	CC1
SMC19.T.CATCGGGCAGTAGAGC	CC2
SMC19.T.CATTCGCCAAGGTTCT	CC1
SMC19.T.CCACCTAGTGGACGAT	CC1
SMC19.T.CCACGGATCGCTAGCG	CC1
SMC19.T.CCACTACCAAACTGTC	CC1
SMC19.T.CCAGCGACAAGTTCTG	CC2
SMC19.T.CCAGCGAGTGTGACGA	CC2
SMC19.T.CCATGTCGTCTCTCGT	CC2
SMC19.T.CCCAGTTGTACAAGTA	CC1
SMC19.T.CCCATACAGCAAATCA	CC1
SMC19.T.CCCTCCTAGCTTCGCG	CC1
SMC19.T.CCGGGATGTTGCGCAC	CC2
SMC19.T.CCGTACTTCTGGGCCA	CC1
SMC19.T.CCGTGGAGTCTTGATG	CC1
SMC19.T.CCGTTCAAGATCCCAT	CC2
SMC19.T.CCTAGCTAGACAATAC	CC1
SMC19.T.CCTAGCTGTCCAGTTA	CC2
SMC19.T.CCTCTGAGTAGCGATG	CC2
SMC19.T.CCTTCCCGTAGCGCAA	CC1
SMC19.T.CCTTCGATCAGGCGAA	CC1
SMC19.T.CGAACATCAAGAGGCT	CC1
SMC19.T.CGACCTTAGACGCTTT	CC2
SMC19.T.CGACCTTAGGAATTAC	CC1
SMC19.T.CGAGAAGCATATACGC	CC1
SMC19.T.CGATCGGAGCTAGTTC	CC1
SMC19.T.CGATGGCTCAGCTCTC	CC1
SMC19.T.CGATGTAAGATGTAAC	CC2
SMC19.T.CGATTGAAGGAGTACC	CC1
SMC19.T.CGATTGAGTCTCTTAT	CC1
SMC19.T.CGCCAAGGTGCACGAA	CC1
SMC19.T.CGCGTTTCAATGAATG	CC1
SMC19.T.CGCTATCCACAGACTT	CC1
SMC19.T.CGCTATCGTAGCTCCG	CC2
SMC19.T.CGCTATCGTGTGGCTC	CC2
SMC19.T.CGCTTCATCCAGTAGT	CC1
SMC19.T.CGGAGTCCAGCGATCC	CC1
SMC19.T.CGTAGCGGTTACGCGC	CC1
SMC19.T.CGTCACTAGATAGCAT	CC2
SMC19.T.CGTGTCTTCATATCGG	CC2
SMC19.T.CGTTAGAAGTGAACAT	CC1
SMC19.T.CGTTCTGGTCAAAGCG	CC1
SMC19.T.CTAAGACAGAGACGAA	CC1
SMC19.T.CTAAGACAGTTACCCA	CC1
SMC19.T.CTAAGACGTTAAGACA	CC1
SMC19.T.CTAAGACTCTTGTACT	CC2
SMC19.T.CTACATTGTTGTGGAG	CC1
SMC19.T.CTACATTTCGCTGATA	CC1
SMC19.T.CTACGTCCACATGACT	CC1
SMC19.T.CTAGAGTAGCCTTGAT	CC1
SMC19.T.CTAGTGAGTTCATGGT	CC2
SMC19.T.CTCACACGTGTAACGG	CC1

SMC19.T.CTCACACGTTGTCGCG	CC2
SMC19.T.CTCAGAAGTGGAAAGA	CC1
SMC19.T.CTCATTATCTCAACTT	CC1
SMC19.T.CTCCTAGTCCTGCTTG	CC2
SMC19.T.CTCCTAGTCGTAGGTT	CC1
SMC19.T.CTCGAAACACTTAAGC	CC2
SMC19.T.CTCGAAAGTTTGGCGC	CC1
SMC19.T.CTCGAAATCACGCGGT	CC2
SMC19.T.CTCGAGGAGGGTTTCT	CC2
SMC19.T.CTCGAGGGTCTAGCGC	CC1
SMC19.T.CTCGGAGCAAACGCGA	CC2
SMC19.T.CTCGGAGCAAGCCGTC	CC1
SMC19.T.CTCGGAGGTGGACGAT	CC1
SMC19.T.CTCGGGAAGCTGTCTA	CC1
SMC19.T.CTCGGGAAGTTCGCGC	CC2
SMC19.T.CTCGTACAGAGTTGGC	CC2
SMC19.T.CTCGTACTCATGTCTT	CC2
SMC19.T.CTCTACGAGACACTAA	CC1
SMC19.T.CTCTGGTAGTACGTAA	CC1
SMC19.T.CTCTGGTTCACAGTAC	CC1
SMC19.T.CTGAAACTCTGGCGAC	CC1
SMC19.T.CTGAAGTAGTTACGGG	CC2
SMC19.T.CTGAAGTCACGTGAGA	CC2
SMC19.T.CTGCTGTAGCGATCCC	CC2
SMC19.T.CTGCTGTGTCAACATC	CC1
SMC19.T.CTGCTGTTCTTGTACT	CC1
SMC19.T.CTGTGCTCACGAGGTA	CC1
SMC19.T.CTGTGCTGTACTCAAC	CC1
SMC19.T.CTGTGCTGTAGCAAAT	CC1
SMC19.T.CTGTTTATCAGTTTGG	CC1
SMC19.T.CTTAACTGTTAAAGAC	CC1
SMC19.T.CTTACCGTCATTGCCC	CC2
SMC19.T.CTTCTCTGTTCGCGAC	CC1
SMC19.T.CTTCTCTTCTAACTCT	CC2
SMC19.T.CTTTGCGGTCAGAAGC	CC2
SMC19.T.CTTTGCGGTGTGACGA	CC1
SMC19.T.GAAACTCAGGCAAAGA	CC1
SMC19.T.GAAACTCTCCTTCAAT	CC1
SMC19.T.GAACCTATCATGTCTT	CC2
SMC19.T.GAAGCAGAGCCAACAG	CC1
SMC19.T.GAAGCAGTCCGCGGTA	CC2
SMC19.T.GAATGAAGTCGTGGCT	CC2
SMC19.T.GACAGAGGTTCACGGC	CC1
SMC19.T.GACCAATGTAGAAAGG	CC1
SMC19.T.GACCTGGCACGGCCAT	CC2
SMC19.T.GACCTGGCAGGAACGT	CC1
SMC19.T.GACCTGGTCCAGTAGT	CC1
SMC19.T.GACGGCTCAATGGATA	CC1
SMC19.T.GACGGCTCAGCCAGAA	CC2
SMC19.T.GACGGCTCAGCTCGCA	CC2
SMC19.T.GACGGCTTCCGCGCAA	CC2
SMC19.T.GACGTGCAGTTCGCGC	CC1
SMC19.T.GACGTGCGTCGCGTGT	CC1
SMC19.T.GACGTTAGTAAATACG	CC2
SMC19.T.GACGTTAGTTATGCGT	CC2
SMC19.T.GACTAACAGGACTGGT	CC2
SMC19.T.GAGGTGAAGCTAGTTC	CC2
SMC19.T.GAGGTGAGTGACGGTA	CC1

SMC19.T.GATCAGTAGTATCGAA	CC2
SMC19.T.GATCGATTCGCGATCG	CC2
SMC19.T.GATCGCGGTCATACTG	CC1
SMC19.T.GATCGTAGTAATCACC	CC1
SMC19.T.GATCTAGTCACGGTTA	CC1
SMC19.T.GATGAAACACCAGCAC	CC2
SMC19.T.GATGAAATCACAAACC	CC2
SMC19.T.GCAAACTCATGTCGAT	CC2
SMC19.T.GCAATCACATGAAGTA	CC1
SMC19.T.GCACATACAGCCTATA	CC2
SMC19.T.GCACTCTAGGCTAGAC	CC2
SMC19.T.GCACTCTGTCGAACAG	CC2
SMC19.T.GCATACAAGAATTGTG	CC1
SMC19.T.GCATGCGGTCCCGACA	CC1
SMC19.T.GCATGCGGTCTCACCT	CC1
SMC19.T.GCATGCGTCACGCGGT	CC2
SMC19.T.GCATGTACACCGCTAG	CC2
SMC19.T.GCATGTACACCTATCC	CC2
SMC19.T.GCCTCTAAGGCATGGT	CC1
SMC19.T.GCGACCACATGACGGA	CC2
SMC19.T.GCGCAGTAGGGCATGT	CC2
SMC19.T.GCGGGTTTCCGAACGC	CC1
SMC19.T.GCTCCTACAATTGCTG	CC2
SMC19.T.GCTGCAGAGATAGGAG	CC1
SMC19.T.GCTGGGTAGTCCGGTC	CC2
SMC19.T.GCTGGGTCACCGGAAA	CC1
SMC19.T.GCTGGGTGTGCAACGA	CC2
SMC19.T.GCTGGGTTCTATGTGG	CC2
SMC19.T.GCTTCCATCTAGAGTC	CC2
SMC19.T.GGAAAGCAGTTTCCTT	CC2
SMC19.T.GGAAAGCGTGCGAAAC	CC1
SMC19.T.GGACAAGAGCGATAGC	CC1
SMC19.T.GGACAGACACAAGTAA	CC1
SMC19.T.GGACAGACAGTGACAG	CC1
SMC19.T.GGACATTAGGTCGGAT	CC2
SMC19.T.GGACATTTCAGGTTCA	CC2
SMC19.T.GGAGCAACAATCTACG	CC1
SMC19.T.GGATGTTCATGTAAGA	CC2
SMC19.T.GGATGTTGTATGCTTG	CC1
SMC19.T.GGATGTTTCCAGAGGA	CC2
SMC19.T.GGATTACCAGCCTTTC	CC1
SMC19.T.GGCAATTAGCGCCTTG	CC1
SMC19.T.GGCAATTAGCTCAACT	CC2
SMC19.T.GGCCGATAGCGTTGCC	CC2
SMC19.T.GGCTCGAAGATGGGTC	CC1
SMC19.T.GGCTCGACATACGCCG	CC2
SMC19.T.GGGAATGGTTGCTCCT	CC2
SMC19.T.GGGACCTTCCTAGAAC	CC1
SMC19.T.GGGAGATGTGCAACTT	CC2
SMC19.T.GGGAGATGTTTGACAC	CC2
SMC19.T.GGGATGACACAGCCCA	CC1
SMC19.T.GGGCACTGTAGTACCT	CC2
SMC19.T.GGGCACTTCTCAAACG	CC1
SMC19.T.GGGCATCTCAGAGCTT	CC1
SMC19.T.GGGTCTGTCCGCGCAA	CC1
SMC19.T.GGGTTGCAGATGGCGT	CC1
SMC19.T.GGTATTGTCGCATGAT	CC1
SMC19.T.GTACGTATCCCGACTT	CC1

SMC19.T.GTACTCCCATCCCATC	CC1
SMC19.T.GTACTTTGTCGAACAG	CC2
SMC19.T.GTACTTTGTTAAAGTG	CC2
SMC19.T.GTAGGCCAGTCTCCTC	CC2
SMC19.T.GTATCTTTCACCTTAT	CC2
SMC19.T.GTCAAGTCATAGACTC	CC2
SMC19.T.GTCATTTCACTGTCGG	CC2
SMC19.T.GTCATTTTCGAGAGCA	CC2
SMC19.T.GTCCTCAAGCAGGTCA	CC1
SMC19.T.GTCCTCAGTGACTCAT	CC2
SMC19.T.GTCGGGTAGTGTACGG	CC1
SMC19.T.GTCGGGTTCGGGAGTA	CC2
SMC19.T.GTCGTAAAGTAGGCCA	CC1
SMC19.T.GTCGTAACAATGTAAG	CC1
SMC19.T.GTGAAGGAGTTGCAGG	CC1
SMC19.T.GTGAAGGCACAGCCCA	CC2
SMC19.T.GTGCAGCCACGAAGCA	CC1
SMC19.T.GTGCATAAGTGTACGG	CC2
SMC19.T.GTGCGGTTCGACCAGC	CC1
SMC19.T.GTGTTAGTCTCCCTGA	CC2
SMC19.T.GTTAAGCGTCGAATCT	CC2
SMC19.T.GTTCATTAGTTAGGTA	CC2
SMC19.T.GTTCATTGTGCACGAA	CC1
SMC19.T.GTTCGGGAGTTGTCGT	CC2
SMC19.T.GTTCGGGGTCTCCACT	CC1
SMC19.T.GTTCGGGGTTCCCTTG	CC1
SMC19.T.GTTTCTAGTGGTTTCA	CC2
SMC19.T.GTTTCTATCAACGAAA	CC1
SMC19.T.TAAGCGTCAGCCAGAA	CC2
SMC19.T.TAAGCGTCATATGGTC	CC2
SMC19.T.TAAGTGCAGTGAATTG	CC2
SMC19.T.TAAGTGCTCACCCTCA	CC1
SMC19.T.TACACGAAGGAGTACC	CC2
SMC19.T.TACACGACAAAGCGGT	CC2
SMC19.T.TACACGACATGGTAGG	CC1
SMC19.T.TACAGTGCACAAGACG	CC2
SMC19.T.TACGGATCAATGTTGC	CC1
SMC19.T.TACGGGCAGTTAACGA	CC1
SMC19.T.TACTCGCAGAACTGTA	CC2
SMC19.T.TACTTACAGCAATCTC	CC2
SMC19.T.TACTTGTAGCTCCTTC	CC1
SMC19.T.TAGACCACAAATCCGT	CC1
SMC19.T.TAGAGCTAGAATCTCC	CC2
SMC19.T.TAGCCGGGTGATAAGT	CC1
SMC19.T.TAGTGGTCACAGACAG	CC2
SMC19.T.TATCTCAAGCTCCTTC	CC2
SMC19.T.TATTACCAGATGCCTT	CC1
SMC19.T.TCACAAGGTGATGATA	CC1
SMC19.T.TCACGAACATCACGAT	CC2
SMC19.T.TCACGAAGTGGTCTCG	CC1
SMC19.T.TCAGATGGTGCTGTAT	CC1
SMC19.T.TCAGCAACATGTCTCC	CC1
SMC19.T.TCAGCTCAGAAGCCCA	CC1
SMC19.T.TCAGGATGTTCGTTGA	CC2
SMC19.T.TCATTTGGTCTTCAAG	CC1
SMC19.T.TCATTTGGTTTGACAC	CC2
SMC19.T.TCGAGGCGTAAGGATT	CC2
SMC19.T.TCGAGGCTCTTGAGGT	CC1

SMC19.T.TCGGGACAGCCACCTG	CC1
SMC19.T.TCGGTAATCCCTTGCA	CC1
SMC19.T.TCGTACCGTACCTACA	CC2
SMC19.T.TCTATTGCACTCAGGC	CC1
SMC19.T.TCTATTGTCGGTTAAC	CC2
SMC19.T.TCTCATACACAGACAG	CC1
SMC19.T.TCTCATAGTTGTTTGG	CC1
SMC19.T.TCTCTAAGTTCGGGCT	CC1
SMC19.T.TCTCTAATCGGAGCAA	CC2
SMC19.T.TCTGAGATCCCTTGTG	CC2
SMC19.T.TCTGGAATCTGGTGTA	CC1
SMC19.T.TGAAAGAGTTGAGGTG	CC2
SMC19.T.TGACAACGTGTAAGTA	CC1
SMC19.T.TGACGGCTCTGAGGGA	CC2
SMC19.T.TGAGCATGTATTCTCT	CC2
SMC19.T.TGAGCCGAGCAGGCTA	CC1
SMC19.T.TGAGCCGCAACGCACC	CC1
SMC19.T.TGCCCATTCTATCCTA	CC2
SMC19.T.TGCGGGTAGCACCGTC	CC2
SMC19.T.TGCGGGTTCACATAGC	CC1
SMC19.T.TGCGTGGCAGCTGTGC	CC1
SMC19.T.TGCTGCTGTCGATTGT	CC1
SMC19.T.TGCTGCTGTCTCTCGT	CC2
SMC19.T.TGCTGCTTCGCGCCAA	CC1
SMC19.T.TGGTTAGTCAGTTGAC	CC1
SMC19.T.TGGTTAGTCTGTCTCG	CC2
SMC19.T.TGGTTCCCACAGCCCA	CC1
SMC19.T.TGTATTCGTCACACGC	CC1
SMC19.T.TGTGGTATCACATGCA	CC1
SMC19.T.TTAACTCAGACAGGCT	CC1
SMC19.T.TTAGGACAGGGAACGG	CC2
SMC19.T.TTAGGCATCGCTAGCG	CC2
SMC19.T.TTATGCTAGATGTGGC	CC2
SMC19.T.TTATGCTGTAGAAAGG	CC2
SMC19.T.TTCGAAGCAAGTAATG	CC2
SMC19.T.TTCGAAGGTGGCAAAC	CC2
SMC19.T.TTCGAAGTCCGCAGTG	CC1
SMC19.T.TTCGGTCAGTACGTTC	CC1
SMC19.T.TTCGGTCCATCCGCGA	CC2
SMC19.T.TTCGGTCTCCAGATCA	CC2
SMC19.T.TTCTACACATCGGTTA	CC1
SMC19.T.TTCTTAGCACGTAAGG	CC1
SMC19.T.TTCTTAGCAGCGAACA	CC2
SMC19.T.TTGCCGTAGTTATCGC	CC1
SMC19.T.TTGGAACCAGCTATTG	CC1
SMC19.T.TTGGCAATCATGTAGC	CC1
SMC19.T.TTTACTGAGAGTACCG	CC1
SMC19.T.TTTACTGAGCAATCTC	CC1
SMC19.T.TTTACTGTCATGTCCC	CC2
SMC19.T.TTTATGCAGGCTAGCA	CC1
SMC19.T.TTTATGCAGGGTCGAT	CC1
SMC19.T.TTTATGCTCCGAACGC	CC1
SMC19.T.TTTGCGCCAAGACACG	CC2
SMC19.T.TTTGCGCGTTCCGTCT	CC1
SMC19.T.TTTGCGCTCTGCCAGG	CC2
SMC19.T.TTTGTCAGTTGATTGC	CC1
SMC20.T.AAACGGGGTAGCACGA	CC2
SMC20.T.AAAGATGGTTTGACTG	CC2

SMC20.T.AAAGCAACAAGACACG	CC1
SMC20.T.AAAGTAGGTCCAAGTT	CC2
SMC20.T.AAATGCCGTAGCCTAT	CC2
SMC20.T.AAATGCCGTCAACTGT	CC2
SMC20.T.AAATGCCGTTATCCGA	CC2
SMC20.T.AAATGCCTCACCTTAT	CC2
SMC20.T.AACACGTGTAGCCTAT	CC2
SMC20.T.AACACGTTCATGTGGT	CC2
SMC20.T.AACCGCGGTCACAAGG	CC2
SMC20.T.AACGTTGGTAAGGGCT	CC2
SMC20.T.AACTGGTCACTTCGAA	CC2
SMC20.T.AACTTTCCACGAAACG	CC2
SMC20.T.AAGACCTCATGTTGAC	CC2
SMC20.T.AAGCCGCGTTTGGCGC	CC2
SMC20.T.AAGGCAGAGCTACCGC	CC2
SMC20.T.AAGGCAGCACCCATTC	CC2
SMC20.T.ACACCAAAGAAGGTGA	CC2
SMC20.T.ACACCAAAGCCCTAAT	CC2
SMC20.T.ACACCAAAGTGGGTTG	CC2
SMC20.T.ACACCCTGTCCGAACC	CC2
SMC20.T.ACAGCCGCATTAACCG	CC2
SMC20.T.ACAGCCGGTCCAGTGC	CC2
SMC20.T.ACATACGTCCACGACG	CC2
SMC20.T.ACATGGTAGAACAACT	CC2
SMC20.T.ACATGGTGTTCTGTTT	CC2
SMC20.T.ACCAGTAAGAGATGAG	CC2
SMC20.T.ACCCACTAGCCGGTAA	CC2
SMC20.T.ACCCACTCACCCAGTG	CC1
SMC20.T.ACCTTTAAGATGCCTT	CC2
SMC20.T.ACCTTTACACATTCGA	CC2
SMC20.T.ACGAGCCGTACAGTTC	CC2
SMC20.T.ACGAGGAAGCTCAACT	CC2
SMC20.T.ACGATACCATCCTTGC	CC2
SMC20.T.ACGGAGAGTCATATGC	CC2
SMC20.T.ACGGAGATCTTAACCT	CC2
SMC20.T.ACGGCCAAGTCGATAA	CC2
SMC20.T.ACGTCAATCGCGCCAA	CC1
SMC20.T.ACTATCTGTGATGATA	CC2
SMC20.T.ACTATCTTCACTTCAT	CC2
SMC20.T.ACTGAGTCAAGAGGCT	CC2
SMC20.T.ACTGAGTGTTCGCGAC	CC2
SMC20.T.ACTTACTGTGTGAAAT	CC2
SMC20.T.ACTTGTTGTTCTGAAC	CC2
SMC20.T.ACTTTCAGTTATCGGT	CC2
SMC20.T.AGAATAGGTCATGCAT	CC2
SMC20.T.AGACGTTTCCTCCTAG	CC2
SMC20.T.AGAGCGAGTTCGTCTC	CC2
SMC20.T.AGAGCGATCATTATCC	CC2
SMC20.T.AGAGCTTGTGCAACTT	CC2
SMC20.T.AGAGTGGGTTCCACGG	CC2
SMC20.T.AGATCTGAGTTCGATC	CC2
SMC20.T.AGATCTGCAGCCACCA	CC2
SMC20.T.AGATCTGGTGTCCTCT	CC2
SMC20.T.AGATCTGGTTCCGGCA	CC2
SMC20.T.AGATTGCCATCGATTG	CC2
SMC20.T.AGCATACTCGCCAAAT	CC2
SMC20.T.AGCATACTCGCGTTTC	CC2
SMC20.T.AGCCTAAGTTATCACG	CC2

SMC20.T.AGCGGTCAGGTGCTAG	CC2
SMC20.T.AGCGGTCCAAACTGCT	CC2
SMC20.T.AGCGTATAGCAGATCG	CC2
SMC20.T.AGCGTATAGCCTTGAT	CC2
SMC20.T.AGCTCTCTCTGCAAGT	CC2
SMC20.T.AGCTCTCTCTTGTATC	CC2
SMC20.T.AGCTTGAGTCAATGTC	CC2
SMC20.T.AGCTTGAGTCATGCAT	CC1
SMC20.T.AGCTTGATCAGCACAT	CC2
SMC20.T.AGCTTGATCTCTGAGA	CC2
SMC20.T.AGGCCACAGCATCATC	CC2
SMC20.T.AGGCCACAGCTGTCTA	CC2
SMC20.T.AGGCCGTTCTGAGTGT	CC2
SMC20.T.AGGGAGTAGATATGCA	CC2
SMC20.T.AGGGAGTAGTAACCCT	CC2
SMC20.T.AGGGAGTCACGGATAG	CC2
SMC20.T.AGGGATGCAGGAACGT	CC2
SMC20.T.AGGGTGAAGAGCCCAA	CC2
SMC20.T.AGGGTGAAGCTCCTTC	CC2
SMC20.T.AGGGTGATCGGATGTT	CC2
SMC20.T.AGGTCATGTGGTCCGT	CC2
SMC20.T.AGGTCCGCAGGTGCCT	CC2
SMC20.T.AGGTCCGCATGGATGG	CC2
SMC20.T.AGTAGTCTCTTACCGC	CC2
SMC20.T.AGTCTTTCACATCCAA	CC2
SMC20.T.AGTCTTTTCAAAGTAG	CC2
SMC20.T.AGTGGGACAGATTGCT	CC2
SMC20.T.AGTGTCACAAACGCGA	CC2
SMC20.T.ATAACGCGTAGCCTCG	CC2
SMC20.T.ATAACGCTCTGTGCAA	CC2
SMC20.T.ATAAGAGAGAGGTAGA	CC2
SMC20.T.ATAAGAGAGTTTAGGA	CC2
SMC20.T.ATAAGAGGTAAAGGAG	CC2
SMC20.T.ATAAGAGGTTGTGGAG	CC2
SMC20.T.ATAAGAGTCAGATAAG	CC2
SMC20.T.ATAAGAGTCTGCCAGG	CC2
SMC20.T.ATAGACCTCCGTAGTA	CC2
SMC20.T.ATCACGATCGTTTATC	CC2
SMC20.T.ATCATCTCACTTCGAA	CC2
SMC20.T.ATCATCTTCATTGCGA	CC2
SMC20.T.ATCATCTTCCGTCAAA	CC2
SMC20.T.ATCCGAACACAGACAG	CC2
SMC20.T.ATCGAGTGTGCAGTAG	CC2
SMC20.T.ATCTACTAGTGTCTCA	CC2
SMC20.T.ATCTGCCGTGGTAACG	CC2
SMC20.T.ATGAGGGAGAGTACCG	CC2
SMC20.T.ATGGGAGAGGTGCTAG	CC2
SMC20.T.ATTACTCGTGATAAAC	CC2
SMC20.T.ATTCTACAGAGTGAGA	CC2
SMC20.T.ATTCTACCACAGGAGT	CC2
SMC20.T.ATTCTACGTTGACGTT	CC2
SMC20.T.ATTGGACCAAGGTTCT	CC2
SMC20.T.ATTTCTGGTATATGAG	CC2
SMC20.T.ATTTCTGGTTGACGTT	CC2
SMC20.T.CAACCAATCCTTAATC	CC2
SMC20.T.CAACCTCTCTTTAGTC	CC2
SMC20.T.CAACTAGGTAGAGTGC	CC2
SMC20.T.CAAGATCCAAGTTGTC	CC2

SMC20.T.CAAGGCCCAGTAAGAT	CC2
SMC20.T.CAAGGCCCATCACGAT	CC2
SMC20.T.CAAGTTGCAGCTGCTG	CC2
SMC20.T.CACACAACAAACCTAC	CC2
SMC20.T.CACACCTCACGAGAGT	CC2
SMC20.T.CACACTCCATCCTAGA	CC1
SMC20.T.CACACTCGTCTAGTGT	CC2
SMC20.T.CACAGTAAGTGCGTGA	CC2
SMC20.T.CACAGTACAGCTGCTG	CC2
SMC20.T.CACAGTAGTCCGACGT	CC2
SMC20.T.CACATAGAGATCCTGT	CC2
SMC20.T.CACATAGGTGTAATGA	CC1
SMC20.T.CACATTTAGACTGTAA	CC2
SMC20.T.CACCAGGCATCGGACC	CC2
SMC20.T.CACCAGGGTCGTGGCT	CC2
SMC20.T.CACCTTGGTTCTGGTA	CC2
SMC20.T.CACCTTGTCAACACAC	CC2
SMC20.T.CACTCCAGTTCTGGTA	CC2
SMC20.T.CAGAATCAGCTAAGAT	CC2
SMC20.T.CAGAATCTCCGGGTGT	CC2
SMC20.T.CAGAGAGAGTGGACGT	CC2
SMC20.T.CAGAGAGTCTACTCAT	CC2
SMC20.T.CAGATCACACCGAATT	CC2
SMC20.T.CAGCAGCGTAAACCTC	CC2
SMC20.T.CAGCATAGTCAGGACA	CC2
SMC20.T.CAGCTAAAGATCCGAG	CC2
SMC20.T.CAGCTAAAGGCAGTCA	CC2
SMC20.T.CAGCTAACAGATGAGC	CC2
SMC20.T.CAGCTAAGTATATCCG	CC2
SMC20.T.CAGGTGCTCCCTCTTT	CC2
SMC20.T.CAGTAACAGGCAATTA	CC2
SMC20.T.CAGTCCTCAGTCGTGC	CC2
SMC20.T.CAGTCCTGTCTTGATG	CC2
SMC20.T.CATCAAGCAGCTCGAC	CC2
SMC20.T.CATCAGATCCTGTAGA	CC2
SMC20.T.CATCCACCAATGTTGC	CC2
SMC20.T.CATCGAACAGACGCAA	CC2
SMC20.T.CATGCCTGTCTGATTG	CC2
SMC20.T.CATGCCTGTTACAGAA	CC2
SMC20.T.CATGCCTTCGTACGGC	CC2
SMC20.T.CCAATCCGTAGAAGGA	CC2
SMC20.T.CCAATCCGTTGGACCC	CC2
SMC20.T.CCACCTAGTACAAGTA	CC2
SMC20.T.CCACTACCACTAAGTC	CC2
SMC20.T.CCACTACCATCGTCGG	CC2
SMC20.T.CCAGCGAAGTGTACTC	CC2
SMC20.T.CCAGCGAGTAAATGTG	CC2
SMC20.T.CCAGCGAGTAGCTCCG	CC2
SMC20.T.CCATGTCGTCTCATCC	CC2
SMC20.T.CCCATACAGCTACCGC	CC2
SMC20.T.CCCATACCATTATCTC	CC2
SMC20.T.CCCATACGTCGGGTCT	CC2
SMC20.T.CCCTCCTAGATATACG	CC2
SMC20.T.CCGGGATCAGATAATG	CC2
SMC20.T.CCGGGATCAGTGGGAT	CC2
SMC20.T.CCGTACTAGTTGCAGG	CC2
SMC20.T.CCGTACTTCCAGAAGG	CC2
SMC20.T.CCGTGGAAGACCGGAT	CC2

SMC20.T.CCGTTCATCCGGGTGT	CC2
SMC20.T.CCTAAAGCACTACAGT	CC2
SMC20.T.CCTAAAGGTAAAGGAG	CC2
SMC20.T.CCTAAAGGTTTGTTTC	CC2
SMC20.T.CCTAAAGTCTACGAGT	CC2
SMC20.T.CCTACCAAGGATGGTC	CC2
SMC20.T.CCTACCACAGTTCATG	CC2
SMC20.T.CCTACCACATGTCCTC	CC2
SMC20.T.CCTACCATCTATCGCC	CC2
SMC20.T.CCTATTACAGACGCAA	CC2
SMC20.T.CCTATTACAGCTTCGG	CC2
SMC20.T.CCTCAGTAGGCGTACA	CC2
SMC20.T.CCTCAGTCACACCGAC	CC2
SMC20.T.CCTCAGTGTTATCGGT	CC2
SMC20.T.CCTTCGAGTCGGCACT	CC2
SMC20.T.CCTTCGATCCTGCTTG	CC2
SMC20.T.CCTTCGATCGGCTTGG	CC2
SMC20.T.CCTTTCTTCCTTTACA	CC2
SMC20.T.CGAACATCAGGCGATA	CC2
SMC20.T.CGAATGTAGCGATCCC	CC2
SMC20.T.CGAATGTAGGTAAACT	CC2
SMC20.T.CGAATGTCATTGGCGC	CC2
SMC20.T.CGACCTTCAGTTAACC	CC2
SMC20.T.CGAGAAGAGGACATTA	CC2
SMC20.T.CGAGCCAGTTCGTGAT	CC2
SMC20.T.CGAGCCATCTTCCTTC	CC2
SMC20.T.CGATCGGAGTTGCAGG	CC2
SMC20.T.CGATGGCGTACATCCA	CC2
SMC20.T.CGATGGCTCCCATTTA	CC2
SMC20.T.CGCCAAGAGATGTGGC	CC2
SMC20.T.CGCGGTACAATTGCTG	CC2
SMC20.T.CGCGGTAGTCGGCACT	CC1
SMC20.T.CGCGGTATCGGAGGTA	CC2
SMC20.T.CGCTATCGTAAGTGGC	CC2
SMC20.T.CGCTATCTCGGATGTT	CC2
SMC20.T.CGCTATCTCGTGGGAA	CC2
SMC20.T.CGCTGGACAGATAATG	CC2
SMC20.T.CGCTGGAGTTGCGTTA	CC2
SMC20.T.CGCTTCAAGTATTGGA	CC2
SMC20.T.CGGACGTAGATCCGAG	CC2
SMC20.T.CGGACTGCAGCGATCC	CC2
SMC20.T.CGGAGCTAGAATCTCC	CC2
SMC20.T.CGGAGTCAGAGACTAT	CC2
SMC20.T.CGGAGTCCATGAAGTA	CC2
SMC20.T.CGGCTAGTCCTAGAAC	CC2
SMC20.T.CGGGTCACATGACGGA	CC1
SMC20.T.CGGTTAACATTTCACT	CC2
SMC20.T.CGTAGCGAGCCAGGAT	CC2
SMC20.T.CGTCACTAGTCCGTAT	CC2
SMC20.T.CGTCTACGTTCGCTAA	CC2
SMC20.T.CGTGAGCAGAGACTTA	CC2
SMC20.T.CGTGTAAAGTAAGTAC	CC2
SMC20.T.CGTGTAAAGTTAGCGG	CC2
SMC20.T.CGTGTCTCACTTAAGC	CC2
SMC20.T.CGTGTCTGTTGCCTCT	CC2
SMC20.T.CTAAGACCAGATGGCA	CC2
SMC20.T.CTACATTGTTAGATGA	CC2
SMC20.T.CTACATTTCTGCGTAA	CC2

SMC20.T.CTACATTTCTTCAACT	CC2
SMC20.T.CTACGTCGTCATATCG	CC2
SMC20.T.CTACGTCGTGTGCGTC	CC2
SMC20.T.CTAGAGTGTCTTTCAT	CC2
SMC20.T.CTAGCCTAGACAGAGA	CC2
SMC20.T.CTAGCCTCATCAGTCA	CC2
SMC20.T.CTAGCCTTCGCCAGCA	CC2
SMC20.T.CTAGTGACATTACCTT	CC2
SMC20.T.CTCAGAATCCTTTACA	CC2
SMC20.T.CTCATTAGTCTGGAGA	CC2
SMC20.T.CTCCTAGGTTTGACAC	CC2
SMC20.T.CTCGAAAAGCAGATCG	CC1
SMC20.T.CTCGAAACAATAGCAA	CC2
SMC20.T.CTCGAAATCTTGGGTA	CC2
SMC20.T.CTCGAGGTCAGCCTAA	CC2
SMC20.T.CTCGAGGTCGCGTTTC	CC2
SMC20.T.CTCGGAGCACCATCCT	CC2
SMC20.T.CTCGGAGGTAGTACCT	CC2
SMC20.T.CTCGGAGTCTTTACGT	CC2
SMC20.T.CTCGGGAAGGACACCA	CC2
SMC20.T.CTCGGGAAGGTTACCT	CC2
SMC20.T.CTCGGGACAAGTCTGT	CC2
SMC20.T.CTCGGGAGTGATGTGG	CC2
SMC20.T.CTCGTACAGGGTGTGT	CC2
SMC20.T.CTCGTACCACATTAGC	CC2
SMC20.T.CTCGTACGTATTAGCC	CC2
SMC20.T.CTCGTCATCGGTTAAC	CC2
SMC20.T.CTCTACGAGCCTATGT	CC2
SMC20.T.CTCTACGAGGGTTCCC	CC2
SMC20.T.CTCTACGCAGACAGGT	CC2
SMC20.T.CTCTACGGTAAGAGGA	CC2
SMC20.T.CTCTACGTCCACTCCA	CC2
SMC20.T.CTCTGGTTCTAACTGG	CC2
SMC20.T.CTGAAACAGTATCTCG	CC2
SMC20.T.CTGAAACTCTTAACCT	CC2
SMC20.T.CTGAAGTTCGAACGGA	CC1
SMC20.T.CTGATAGTCATCGATG	CC1
SMC20.T.CTGATCCCAAGGGTCA	CC2
SMC20.T.CTGCTGTGTGAACCTT	CC2
SMC20.T.CTGCTGTTCATCACCC	CC2
SMC20.T.CTGGTCTGTTATGCGT	CC2
SMC20.T.CTGGTCTTCGCGTAGC	CC2
SMC20.T.CTGTGCTGTGTGGCTC	CC2
SMC20.T.CTGTTTATCGAACGGA	CC2
SMC20.T.CTTAACTCAGACGCCT	CC2
SMC20.T.CTTACCGGTTTAGGAA	CC2
SMC20.T.CTTCTCTCAGTACACT	CC2
SMC20.T.CTTCTCTTCACAGTAC	CC2
SMC20.T.CTTTGCGAGGTCATCT	CC2
SMC20.T.CTTTGCGTCAACACAC	CC2
SMC20.T.GAAATGACAAGCCTAT	CC2
SMC20.T.GAAATGACAGGCGATA	CC2
SMC20.T.GAAATGAGTGTGCGTC	CC2
SMC20.T.GAACATCAGGTTCCTA	CC2
SMC20.T.GAACATCGTAATCGTC	CC2
SMC20.T.GAACATCTCCACGCAG	CC2
SMC20.T.GAACCTATCATATCGG	CC2
SMC20.T.GACAGAGCAAGTAGTA	CC2

SMC20.T.GACAGAGTCACCCTCA	CC2
SMC20.T.GACGTTAGTCTAGAGG	CC2
SMC20.T.GACGTTATCCATGCTC	CC2
SMC20.T.GACTACACAAGAGGCT	CC2
SMC20.T.GACTGCGGTCCGAACC	CC1
SMC20.T.GAGTCCGCAGCTATTG	CC2
SMC20.T.GAGTCCGGTTAAGGGC	CC2
SMC20.T.GAGTCCGTCCGGGTGT	CC2
SMC20.T.GATCAGTAGCTGGAAC	CC2
SMC20.T.GATCAGTGTCAGAATA	CC2
SMC20.T.GATCAGTGTCGACTAT	CC2
SMC20.T.GATCAGTTCATACGGT	CC2
SMC20.T.GATCGTAAGCACACAG	CC2
SMC20.T.GATGAAAAGCGCTTAT	CC2
SMC20.T.GATGCTACAGGATTGG	CC2
SMC20.T.GCACATATCAAGATCC	CC2
SMC20.T.GCACTCTGTACCGAGA	CC2
SMC20.T.GCAGTTAAGTCGATAA	CC2
SMC20.T.GCAGTTAGTGCCTGTG	CC2
SMC20.T.GCATACAAGAACAACT	CC2
SMC20.T.GCATACAGTACTTGAC	CC2
SMC20.T.GCATACATCACGCGGT	CC2
SMC20.T.GCATGTACATACGCCG	CC2
SMC20.T.GCATGTATCGCGTTTC	CC2
SMC20.T.GCCAAATCATTAACCG	CC2
SMC20.T.GCCAAATGTTGTACAC	CC2
SMC20.T.GCGAGAAAGATGAGAG	CC2
SMC20.T.GCGCAACAGGAACTGC	CC2
SMC20.T.GCGCAACGTCTCAACA	CC2
SMC20.T.GCGCAACTCGTTTAGG	CC2
SMC20.T.GCGCAGTGTTGTCTTT	CC2
SMC20.T.GCGCCAACAGGGCATA	CC2
SMC20.T.GCGCGATAGGGTTCCC	CC2
SMC20.T.GCGCGATCAAGCGTAG	CC2
SMC20.T.GCGGGTTAGAGCTGGT	CC2
SMC20.T.GCGGGTTCATCACCCT	CC2
SMC20.T.GCTCCTAGTTCCCGAG	CC2
SMC20.T.GCTCTGTAGGATGTAT	CC2
SMC20.T.GCTGGGTCAGATCCAT	CC2
SMC20.T.GCTTCCATCATGCAAC	CC2
SMC20.T.GCTTGAACACATGACT	CC2
SMC20.T.GGAAAGCAGCCACGCT	CC2
SMC20.T.GGAATAAAGTGGGATC	CC2
SMC20.T.GGAATAACATGTTGAC	CC2
SMC20.T.GGAATAAGTAAGTTCC	CC2
SMC20.T.GGACAAGCAGAGTGTG	CC2
SMC20.T.GGACAAGGTGGCGAAT	CC2
SMC20.T.GGACAGAAGTGAACGC	CC1
SMC20.T.GGACAGATCACGATGT	CC2
SMC20.T.GGACATTAGGAGTACC	CC2
SMC20.T.GGACATTGTATAGTAG	CC2
SMC20.T.GGAGCAACATCCGTGG	CC2
SMC20.T.GGATGTTCAGGCGATA	CC1
SMC20.T.GGCCGATTCGTTACAG	CC2
SMC20.T.GGCCGATTCTCTTATG	CC2
SMC20.T.GGCGACTCACGTTGGC	CC2
SMC20.T.GGCGTGTTCAAACCGT	CC2
SMC20.T.GGGAATGAGAACTGTA	CC2

SMC20.T.GGGAATGGTTCCCTTG	CC2
SMC20.T.GGGACCTGTACCATCA	CC2
SMC20.T.GGGACCTGTATAGTAG	CC2
SMC20.T.GGGAGATCAGCTGTTA	CC2
SMC20.T.GGGAGATGTCTAACGT	CC2
SMC20.T.GGGATGATCTAACTGG	CC2
SMC20.T.GGGCACTAGAACTCGG	CC2
SMC20.T.GGGCACTGTGCGATAG	CC2
SMC20.T.GGGCACTGTGCTCTTC	CC2
SMC20.T.GGGCACTGTGGTGTAG	CC2
SMC20.T.GGGCATCGTCGAGATG	CC2
SMC20.T.GGGCATCGTGTGAATA	CC2
SMC20.T.GGGCATCTCCTTGCCA	CC2
SMC20.T.GGGTCTGTCGCCAGCA	CC2
SMC20.T.GGTGAAGGTTCATGGT	CC2
SMC20.T.GGTGCGTCAGGTGCCT	CC2
SMC20.T.GGTGCGTCATACCATG	CC2
SMC20.T.GGTGTTAAGCGATTCT	CC2
SMC20.T.GGTGTTAGTGCCTGGT	CC2
SMC20.T.GTAACTGCAAACTGTC	CC2
SMC20.T.GTAACTGTCACCACCT	CC2
SMC20.T.GTACGTAAGCGCTCCA	CC2
SMC20.T.GTACGTACAATGGTCT	CC2
SMC20.T.GTACTTTGTGAAGGCT	CC2
SMC20.T.GTAGTCAAGTGGTAGC	CC2
SMC20.T.GTATCTTGTGCACGAA	CC2
SMC20.T.GTATTCTCATGACGGA	CC2
SMC20.T.GTATTCTTCGGTCTAA	CC2
SMC20.T.GTCAAGTAGCTTATCG	CC2
SMC20.T.GTCACAATCGCGCCAA	CC2
SMC20.T.GTCACAATCTCTTATG	CC2
SMC20.T.GTCACGGGTACCCAAT	CC2
SMC20.T.GTCCTCAAGCGTAATA	CC2
SMC20.T.GTCCTCAGTTGGGACA	CC2
SMC20.T.GTCCTCATCTTTCCTC	CC2
SMC20.T.GTCGGGTAGACAAAGG	CC2
SMC20.T.GTCGGGTTCACCTTAT	CC2
SMC20.T.GTCGTAAGTGAAGGCT	CC2
SMC20.T.GTCTCGTAGCTGCGAA	CC2
SMC20.T.GTCTTCGGTGTATGGG	CC2
SMC20.T.GTCTTCGTCTTACCGC	CC2
SMC20.T.GTGAAGGGTCTTGTCC	CC2
SMC20.T.GTGAAGGTCACAAACC	CC2
SMC20.T.GTGCAGCGTCGACTGC	CC2
SMC20.T.GTGCTTCAGATGTGGC	CC2
SMC20.T.GTGTGCGCATGACATC	CC2
SMC20.T.GTGTTAGTCTACCTGC	CC2
SMC20.T.GTGTTAGTCTTGAGAC	CC2
SMC20.T.GTTACAGCACTCGACG	CC2
SMC20.T.GTTACAGTCGTCTGAA	CC2
SMC20.T.GTTCATTAGACGACGT	CC2
SMC20.T.GTTCATTAGATCTGAA	CC2
SMC20.T.GTTCATTAGTACATGA	CC2
SMC20.T.GTTCATTCAGCCAGAA	CC2
SMC20.T.GTTCATTCAGGGTATG	CC2
SMC20.T.GTTCATTCAGTAAGCG	CC2
SMC20.T.GTTCATTGTTCCGTCT	CC2
SMC20.T.GTTCTCGTCATACGGT	CC2

SMC20.T.GTTTCTATCAGTCAGT	CC2
SMC20.T.TAAACCGAGAAGCCCA	CC2
SMC20.T.TAAACCGGTGGTAACG	CC2
SMC20.T.TAAGAGACATACCATG	CC2
SMC20.T.TAAGCGTAGATACACA	CC2
SMC20.T.TAAGCGTAGTACGACG	CC2
SMC20.T.TAAGCGTTCCAAACTG	CC2
SMC20.T.TAAGTGCCATGTCTCC	CC2
SMC20.T.TACACGATCAATCTCT	CC2
SMC20.T.TACAGTGGTTTGGGCC	CC2
SMC20.T.TACGGATGTACATCCA	CC2
SMC20.T.TACGGTACATCGACGC	CC2
SMC20.T.TACTCATAGTACGTAA	CC2
SMC20.T.TACTCATCAAGTTGTC	CC2
SMC20.T.TACTCATCACTTAAGC	CC2
SMC20.T.TACTCATTCCCTTGTG	CC2
SMC20.T.TACTCGCAGTGCGATG	CC2
SMC20.T.TACTCGCCAAGTCTAC	CC2
SMC20.T.TACTCGCGTCCTCTTG	CC2
SMC20.T.TACTTACGTCCGTCAG	CC2
SMC20.T.TACTTGTCACATGTGT	CC2
SMC20.T.TAGACCAAGAGAACAG	CC2
SMC20.T.TAGACCAGTCCTCTTG	CC2
SMC20.T.TAGACCAGTGGTAACG	CC2
SMC20.T.TAGAGCTTCAGCAACT	CC2
SMC20.T.TAGAGCTTCCGCGCAA	CC2
SMC20.T.TAGAGCTTCGACGGAA	CC2
SMC20.T.TAGTGGTGTTCTGTTT	CC2
SMC20.T.TAGTTGGTCTGTTTGT	CC2
SMC20.T.TATCAGGAGTCTCAAC	CC2
SMC20.T.TATCTCAAGTGTTGAA	CC2
SMC20.T.TATCTCACAGGGAGAG	CC2
SMC20.T.TATCTCACATCCGGGT	CC2
SMC20.T.TATCTCAGTCCGACGT	CC2
SMC20.T.TCAATCTGTCTTTCAT	CC2
SMC20.T.TCACAAGCACATGACT	CC2
SMC20.T.TCACAAGCACCGAAAG	CC2
SMC20.T.TCAGCAATCTGCCCTA	CC2
SMC20.T.TCAGCTCAGCCCAACC	CC1
SMC20.T.TCAGGTAAGGTGTTAA	CC2
SMC20.T.TCATTACTCTCCAGGG	CC2
SMC20.T.TCATTTGAGGTCATCT	CC2
SMC20.T.TCCACACAGGGTGTGT	CC2
SMC20.T.TCCCGATCACTGTTAG	CC2
SMC20.T.TCCCGATGTTCTCATT	CC1
SMC20.T.TCGAGGCAGCTAGCCC	CC2
SMC20.T.TCGAGGCGTGTGAAAT	CC2
SMC20.T.TCGGGACCATGTCTCC	CC2
SMC20.T.TCGGGACGTCTTGCGG	CC2
SMC20.T.TCGGGACTCCCTTGTG	CC2
SMC20.T.TCGGTAAAGAACAACT	CC2
SMC20.T.TCGGTAAGTGAGTGAC	CC2
SMC20.T.TCGGTAATCGAACTGT	CC2
SMC20.T.TCGTAGACAGACGCCT	CC2
SMC20.T.TCTATTGAGGAGTTGC	CC2
SMC20.T.TCTCATATCTTCATGT	CC2
SMC20.T.TCTCTAAGTAAATGTG	CC2
SMC20.T.TCTCTAATCCAAACAC	CC2

SMC20.T.TCTTCGGGTCGAGTTT	CC2
SMC20.T.TCTTCGGTCTGAAAGA	CC2
SMC20.T.TCTTTCCTCGTACGGC	CC2
SMC20.T.TGAAAGACAAGAGTCG	CC2
SMC20.T.TGAAAGACAATGTTGC	CC2
SMC20.T.TGAAAGATCACGCATA	CC2
SMC20.T.TGACAACCAATCGGTT	CC2
SMC20.T.TGAGCCGGTAAATACG	CC2
SMC20.T.TGAGGGAGTCTTGTCC	CC2
SMC20.T.TGCCAAAGTCGCATAT	CC1
SMC20.T.TGCCAAAGTGAAGGCT	CC2
SMC20.T.TGCCAAATCACAAACC	CC2
SMC20.T.TGCCAAATCTAGCACA	CC2
SMC20.T.TGCCCATGTGTCAATC	CC1
SMC20.T.TGCCCTAGTGTGGCTC	CC2
SMC20.T.TGCGGGTGTCGGATCC	CC2
SMC20.T.TGCTACCGTTCCGGCA	CC2
SMC20.T.TGCTACCTCCTCCTAG	CC2
SMC20.T.TGGCCAGAGGATATAC	CC2
SMC20.T.TGGGCGTAGCTACCTA	CC2
SMC20.T.TGGGCGTTCATCTGTT	CC2
SMC20.T.TGTATTCTCATGGTCA	CC2
SMC20.T.TGTCCCAGTGAGCGAT	CC2
SMC20.T.TGTGGTAAGAAACCAT	CC2
SMC20.T.TGTGGTAAGCGTTGCC	CC2
SMC20.T.TGTGTTTAGAGGTAGA	CC2
SMC20.T.TGTGTTTCAAGCCTAT	CC2
SMC20.T.TGTGTTTGTCGCCATG	CC2
SMC20.T.TTAACTCCACGCTTTC	CC2
SMC20.T.TTAGGACTCGGGAGTA	CC2
SMC20.T.TTAGTTCGTTCCCTTG	CC2
SMC20.T.TTATGCTCAGAGTGTG	CC2
SMC20.T.TTATGCTCATCCGGGT	CC2
SMC20.T.TTATGCTGTGAGGGTT	CC2
SMC20.T.TTCCCAGTCTTGGGTA	CC2
SMC20.T.TTCGAAGCAGCAGTTT	CC2
SMC20.T.TTCGAAGTCCCTCAGT	CC1
SMC20.T.TTCGGTCCATGTCCTC	CC2
SMC20.T.TTCTACAGTATAAACG	CC2
SMC20.T.TTCTCCTGTTCCTCCA	CC2
SMC20.T.TTCTTAGGTACAGCAG	CC2
SMC20.T.TTGACTTAGTTGTAGA	CC2
SMC20.T.TTGCCGTAGCGTGAGT	CC2
SMC20.T.TTGCGTCAGTACACCT	CC2
SMC20.T.TTGCGTCTCGAATGGG	CC2
SMC20.T.TTGCGTCTCGTGACAT	CC2
SMC20.T.TTGGAACAGAGCTTCT	CC2
SMC20.T.TTGGAACGTTCATGGT	CC2
SMC20.T.TTTACTGAGGAGTAGA	CC2
SMC20.T.TTTACTGGTGCTTCTC	CC2
SMC20.T.TTTACTGTCGCCTGAG	CC2
SMC20.T.TTTATGCAGGAGCGAG	CC1
SMC20.T.TTTCCTCTCTGCCAGG	CC2
SMC20.T.TTTGCGCGTAATAGCA	CC2
SMC20.T.TTTGGTTAGGATCGCA	CC2
SMC20.T.TTTGGTTGTTGAACTC	CC2
SMC20.T.TTTGTCAAGGATTCGG	CC2
SMC20.T.TTTGTCAAGTCTCCTC	CC1

SMC21.T.AAACCTGAGCACAGGT	CC1
SMC21.T.AAACCTGAGCTGCCCA	CC1
SMC21.T.AAACCTGCAGGTCTCG	CC1
SMC21.T.AAACCTGCAGTGGAGT	CC1
SMC21.T.AAACCTGCATCTCCCA	CC1
SMC21.T.AAACCTGGTGCCTGGT	CC1
SMC21.T.AAACCTGGTGCGCTTG	CC1
SMC21.T.AAACGGGAGGTTCCTA	CC1
SMC21.T.AAACGGGCATCCCACT	CC1
SMC21.T.AAACGGGGTAGCTCCG	CC1
SMC21.T.AAACGGGTCTTGACGA	CC1
SMC21.T.AAAGATGAGCATCATC	CC1
SMC21.T.AAAGCAAAGTGTACGG	CC1
SMC21.T.AAAGCAAGTAGCTCCG	CC1
SMC21.T.AAAGCAAGTGGTGTAG	CC1
SMC21.T.AAAGTAGCAGCTCCGA	CC1
SMC21.T.AAAGTAGGTAGTGAAT	CC1
SMC21.T.AAAGTAGGTGTGGCTC	CC1
SMC21.T.AAATGCCCAATAACGA	CC1
SMC21.T.AAATGCCGTACTTCTT	CC1
SMC21.T.AACACGTCAACGCACC	CC1
SMC21.T.AACACGTCACATTCGA	CC1
SMC21.T.AACACGTGTAGTAGTA	CC1
SMC21.T.AACACGTTCAACGGCC	CC1
SMC21.T.AACCATGAGCCCAACC	CC1
SMC21.T.AACCATGAGTGGACGT	CC1
SMC21.T.AACCATGGTACATCCA	CC1
SMC21.T.AACCATGGTGTCAATC	CC1
SMC21.T.AACCATGGTTCGTTGA	CC1
SMC21.T.AACCATGTCATCATTC	CC1
SMC21.T.AACGTTGAGGATGGTC	CC1
SMC21.T.AACGTTGCAAGCCGCT	CC1
SMC21.T.AACGTTGCAGCTTCGG	CC1
SMC21.T.AACGTTGCAGTGGGAT	CC2
SMC21.T.AACGTTGCATGCCCGA	CC1
SMC21.T.AACGTTGGTCCATCCT	CC1
SMC21.T.AACGTTGGTTCAACCA	CC1
SMC21.T.AACGTTGTCCACGACG	CC1
SMC21.T.AACTCAGAGAGCTTCT	CC1
SMC21.T.AACTCAGAGCGCTCCA	CC1
SMC21.T.AACTCAGTCTTGCATT	CC1
SMC21.T.AACTCCCAGCGATCCC	CC1
SMC21.T.AACTCCCAGTCCGTAT	CC1
SMC21.T.AACTCCCTCAACGCTA	CC1
SMC21.T.AACTCCCTCACTCCTG	CC1
SMC21.T.AACTCCCTCATCGCTC	CC1
SMC21.T.AACTCCCTCCAAACAC	CC1
SMC21.T.AACTCTTCAAGTCTGT	CC1
SMC21.T.AACTCTTGTACATGTC	CC1
SMC21.T.AACTCTTGTCCGCTGA	CC1
SMC21.T.AACTGGTCAATCGGTT	CC1
SMC21.T.AACTTTCTCTGAGTGT	CC1
SMC21.T.AAGCCGCAGAACAATC	CC1
SMC21.T.AAGCCGCAGGACATTA	CC1
SMC21.T.AAGCCGCTCTCCGGTT	CC1
SMC21.T.AAGGAGCCAACAACCT	CC1
SMC21.T.AAGGAGCCACTGAAGG	CC1
SMC21.T.AAGGAGCGTAAGGGAA	CC1

SMC21.T.AAGGCAGAGACTTGAA	CC1
SMC21.T.AAGGCAGCAAGTCTAC	CC1
SMC21.T.AAGGCAGCACACATGT	CC1
SMC21.T.AAGGCAGGTCCAAGTT	CC1
SMC21.T.AAGGTTCAGAGTTGGC	CC1
SMC21.T.AAGGTTCAGGATTCGG	CC1
SMC21.T.AAGGTTCAGGCCATAG	CC1
SMC21.T.AAGGTTCGTTCACCTC	CC1
SMC21.T.AAGGTTCTCGTTTGCC	CC1
SMC21.T.AAGTCTGCATATGAGA	CC1
SMC21.T.AAGTCTGGTCTGGAGA	CC1
SMC21.T.AATCCAGAGTATCGAA	CC1
SMC21.T.AATCCAGCACCACGTG	CC2
SMC21.T.AATCCAGCACCTCGGA	CC1
SMC21.T.AATCGGTGTCCATCCT	CC2
SMC21.T.AATCGGTGTCTAAACC	CC1
SMC21.T.ACACCAACAAATACAG	CC1
SMC21.T.ACACCAAGTAAGAGAG	CC1
SMC21.T.ACACCAAGTAGAGGAA	CC1
SMC21.T.ACACCCTAGAGGTTGC	CC1
SMC21.T.ACACCCTAGCTGTTCA	CC1
SMC21.T.ACACCCTAGTACGCCC	CC1
SMC21.T.ACACCCTCACGGCGTT	CC2
SMC21.T.ACACCCTCAGCCAGAA	CC1
SMC21.T.ACACCCTTCAGTTCGA	CC1
SMC21.T.ACACCCTTCTGCGACG	CC1
SMC21.T.ACACCGGCAAGGTGTG	CC1
SMC21.T.ACAGCCGCAGCGATCC	CC1
SMC21.T.ACAGCCGCAGTATCTG	CC1
SMC21.T.ACAGCCGCATCTATGG	CC1
SMC21.T.ACAGCCGGTGGGTATG	CC1
SMC21.T.ACAGCCGGTTGAGGTG	CC1
SMC21.T.ACAGCTAAGTAATCCC	CC1
SMC21.T.ACAGCTACAGCCTTGG	CC1
SMC21.T.ACAGCTACATGTAAGA	CC1
SMC21.T.ACAGCTAGTCCTCTTG	CC1
SMC21.T.ACAGCTAGTGTTCTTT	CC1
SMC21.T.ACATACGCAATCTACG	CC1
SMC21.T.ACATACGCACGCCAGT	CC1
SMC21.T.ACATACGCATGCATGT	CC1
SMC21.T.ACATACGGTGATGTCT	CC1
SMC21.T.ACATCAGAGAAACCAT	CC1
SMC21.T.ACATCAGAGATGTCGG	CC1
SMC21.T.ACATCAGTCTCAAACG	CC1
SMC21.T.ACATGGTCAAAGGCGT	CC1
SMC21.T.ACATGGTCAAATCCGT	CC1
SMC21.T.ACATGGTCAGCTGTGC	CC1
SMC21.T.ACATGGTGTAGCGTCC	CC1
SMC21.T.ACATGGTTCAACGGCC	CC1
SMC21.T.ACATGGTTCCGCTGTT	CC1
SMC21.T.ACCAGTACAGTCTTCC	CC1
SMC21.T.ACCAGTACATATACCG	CC1
SMC21.T.ACCAGTAGTACCGAGA	CC1
SMC21.T.ACCAGTATCGAATGCT	CC1
SMC21.T.ACCCACTAGCTGATAA	CC1
SMC21.T.ACCCACTCAGGGTACA	CC1
SMC21.T.ACCCACTGTATATGAG	CC1
SMC21.T.ACCGTAAAGACCACGA	CC1

SMC21.T.ACCGTAAAGACTAGGC	CC1
SMC21.T.ACCGTAACAAGAGGCT	CC1
SMC21.T.ACCGTAACACATGACT	CC1
SMC21.T.ACCGTAAGTATAAACG	CC1
SMC21.T.ACCGTAATCCCAAGAT	CC1
SMC21.T.ACCTTTACATTTGCCC	CC1
SMC21.T.ACCTTTATCGCAGGCT	CC1
SMC21.T.ACGAGCCAGGCCGAAT	CC1
SMC21.T.ACGAGCCGTATAGGGC	CC1
SMC21.T.ACGAGGAAGCTAACTC	CC1
SMC21.T.ACGAGGAGTCAGAAGC	CC1
SMC21.T.ACGAGGATCACAGGCC	CC1
SMC21.T.ACGATACGTACCAGTT	CC1
SMC21.T.ACGATGTGTACCGAGA	CC1
SMC21.T.ACGATGTGTGGTAACG	CC1
SMC21.T.ACGATGTTCAGTTTGG	CC1
SMC21.T.ACGCAGCAGGAACTGC	CC1
SMC21.T.ACGCAGCGTGACCAAG	CC1
SMC21.T.ACGCAGCTCCGCATAA	CC1
SMC21.T.ACGCCAGAGCTAGTTC	CC1
SMC21.T.ACGCCAGCAAGTTCTG	CC1
SMC21.T.ACGCCAGCACAGGTTT	CC1
SMC21.T.ACGCCAGCATCTCCCA	CC1
SMC21.T.ACGCCAGGTACAAGTA	CC1
SMC21.T.ACGCCGACAATACGCT	CC1
SMC21.T.ACGCCGACACCGAATT	CC1
SMC21.T.ACGCCGACAGGATCGA	CC1
SMC21.T.ACGCCGATCGGTGTCG	CC1
SMC21.T.ACGGAGAGTCTGCAAT	CC2
SMC21.T.ACGGAGATCGCGGATC	CC1
SMC21.T.ACGGAGATCGTCCAGG	CC1
SMC21.T.ACGGCCAAGCGTAATA	CC1
SMC21.T.ACGGCCACAAAGTGCG	CC1
SMC21.T.ACGGGCTAGTGTCCAT	CC1
SMC21.T.ACGGGCTCAATGGTCT	CC1
SMC21.T.ACGGGCTCAGCTGTTA	CC1
SMC21.T.ACGGGCTCAGGATTGG	CC1
SMC21.T.ACGGGTCCAAGCGCTC	CC1
SMC21.T.ACGGGTCCATACAGCT	CC1
SMC21.T.ACGGGTCTCTGGTGTA	CC1
SMC21.T.ACGTCAAAGTGTACGG	CC1
SMC21.T.ACGTCAAGTAAATACG	CC1
SMC21.T.ACGTCAAGTCGCCATG	CC1
SMC21.T.ACGTCAATCCCTTGTG	CC1
SMC21.T.ACTATCTAGGGATACC	CC1
SMC21.T.ACTATCTAGTTCCACA	CC1
SMC21.T.ACTATCTCAATGTAAG	CC1
SMC21.T.ACTATCTTCTTTAGGG	CC1
SMC21.T.ACTGAACAGTAGCGGT	CC1
SMC21.T.ACTGAACAGTTAACGA	CC1
SMC21.T.ACTGAACGTTGGACCC	CC1
SMC21.T.ACTGAGTAGCTCCTCT	CC1
SMC21.T.ACTGAGTCAATTGCTG	CC1
SMC21.T.ACTGAGTCAGACTCGC	CC1
SMC21.T.ACTGAGTCATGACATC	CC1
SMC21.T.ACTGAGTGTCTTGATG	CC1
SMC21.T.ACTGAGTGTGGACGAT	CC1
SMC21.T.ACTGATGAGCTTATCG	CC1

SMC21.T.ACTGATGAGTCGCCGT	CC1
SMC21.T.ACTGATGCACTGTTAG	CC1
SMC21.T.ACTGTCCAGTATCTCG	CC1
SMC21.T.ACTGTCCAGTTACGGG	CC1
SMC21.T.ACTGTCCCAATCCAAC	CC1
SMC21.T.ACTGTCCCAGCTCCGA	CC1
SMC21.T.ACTGTCCGTAAGGGCT	CC1
SMC21.T.ACTGTCCGTAGAGCTG	CC1
SMC21.T.ACTGTCCGTGATAAGT	CC1
SMC21.T.ACTTACTGTACAGACG	CC1
SMC21.T.ACTTACTGTAGCTAAA	CC1
SMC21.T.ACTTACTGTTCTGTTT	CC1
SMC21.T.ACTTACTTCCAGAGGA	CC1
SMC21.T.ACTTGTTAGCCAGTTT	CC1
SMC21.T.ACTTGTTAGCGATATA	CC1
SMC21.T.ACTTGTTCAGCCAGAA	CC1
SMC21.T.ACTTGTTGTACCGCTG	CC1
SMC21.T.ACTTTCAAGATGCGAC	CC1
SMC21.T.ACTTTCACACAGATTC	CC1
SMC21.T.ACTTTCACAGGACCCT	CC1
SMC21.T.ACTTTCACATTTCAGG	CC1
SMC21.T.ACTTTCAGTCCGAATT	CC1
SMC21.T.ACTTTCATCCGAACGC	CC1
SMC21.T.ACTTTCATCCTCAATT	CC1
SMC21.T.ACTTTCATCTTTACAC	CC1
SMC21.T.AGAATAGAGTAGGCCA	CC1
SMC21.T.AGAATAGCATGGATGG	CC1
SMC21.T.AGAATAGCATGGTTGT	CC1
SMC21.T.AGAATAGGTTGTCTTT	CC1
SMC21.T.AGACGTTAGTACATGA	CC1
SMC21.T.AGACGTTCATAAGACA	CC1
SMC21.T.AGACGTTTCCAGGGCT	CC1
SMC21.T.AGAGCGAAGAAGGGTA	CC1
SMC21.T.AGAGCGAAGTGCGTGA	CC1
SMC21.T.AGAGCGACAGAAGCAC	CC1
SMC21.T.AGAGCGACATGATCCA	CC1
SMC21.T.AGAGCGAGTCGAAAGC	CC1
SMC21.T.AGAGCGAGTGTCCTCT	CC1
SMC21.T.AGAGCGATCACCATAG	CC1
SMC21.T.AGAGCTTAGTAGATGT	CC1
SMC21.T.AGAGCTTGTGCTAGCC	CC1
SMC21.T.AGAGCTTTCAGGCAAG	CC1
SMC21.T.AGAGCTTTCTCGTTTA	CC1
SMC21.T.AGAGTGGAGATAGTCA	CC1
SMC21.T.AGAGTGGAGTCGTTTG	CC1
SMC21.T.AGATCTGGTAACGTTC	CC1
SMC21.T.AGATCTGGTCTCCACT	CC1
SMC21.T.AGATCTGTCTCAAGTG	CC1
SMC21.T.AGATTGCCAAATTGCC	CC1
SMC21.T.AGATTGCGTCCAAGTT	CC1
SMC21.T.AGATTGCGTGCATCTA	CC1
SMC21.T.AGCAGCCTCGTGGGAA	CC1
SMC21.T.AGCATACAGAGTACCG	CC1
SMC21.T.AGCATACAGGCATGTG	CC1
SMC21.T.AGCATACGTCATCGGC	CC1
SMC21.T.AGCCTAAAGAACTGTA	CC1
SMC21.T.AGCCTAAGTAAACACA	CC1
SMC21.T.AGCCTAAGTAGGGACT	CC1

SMC21.T.AGCGGTCAGGAGTTTA	CC1
SMC21.T.AGCGGTCCAGATAATG	CC1
SMC21.T.AGCGTATAGCACCGCT	CC2
SMC21.T.AGCGTATAGCCGCCTA	CC1
SMC21.T.AGCGTATAGGACAGAA	CC1
SMC21.T.AGCGTATAGTGTTTGC	CC1
SMC21.T.AGCGTATGTCCAGTAT	CC1
SMC21.T.AGCGTATGTTCTCATT	CC1
SMC21.T.AGCGTCGAGGAGCGTT	CC1
SMC21.T.AGCGTCGAGGCGATAC	CC1
SMC21.T.AGCGTCGGTGACTCAT	CC1
SMC21.T.AGCGTCGGTGTGAAAT	CC1
SMC21.T.AGCGTCGTCTCCGGTT	CC1
SMC21.T.AGCTCCTAGTTCCACA	CC1
SMC21.T.AGCTCCTAGTTCGATC	CC1
SMC21.T.AGCTCCTTCAACCATG	CC1
SMC21.T.AGCTCTCAGAGGTTGC	CC1
SMC21.T.AGCTCTCAGTGTACTC	CC1
SMC21.T.AGCTCTCCAATAAGCA	CC1
SMC21.T.AGCTCTCTCCTAGTGA	CC1
SMC21.T.AGCTTGAAGTGAATTG	CC1
SMC21.T.AGCTTGACAAGGTTCT	CC1
SMC21.T.AGCTTGAGTGAGGCTA	CC1
SMC21.T.AGCTTGAGTGTGACCC	CC1
SMC21.T.AGCTTGATCCGAAGAG	CC1
SMC21.T.AGGCCACAGAACAACT	CC1
SMC21.T.AGGCCACAGTCTTGCA	CC1
SMC21.T.AGGCCACAGTGCCAGA	CC1
SMC21.T.AGGCCACCATGCTAGT	CC1
SMC21.T.AGGCCACGTCATTAGC	CC1
SMC21.T.AGGCCACGTCTTTCAT	CC1
SMC21.T.AGGCCACGTTGATTGC	CC1
SMC21.T.AGGCCACTCCCTAATT	CC1
SMC21.T.AGGCCGTAGCACCGTC	CC1
SMC21.T.AGGCCGTGTCTGCGGT	CC1
SMC21.T.AGGCCGTGTTGGACCC	CC1
SMC21.T.AGGCCGTTCACTTACT	CC1
SMC21.T.AGGGAGTAGGGATGGG	CC1
SMC21.T.AGGGAGTCACAGGAGT	CC1
SMC21.T.AGGGAGTCATGACGGA	CC1
SMC21.T.AGGGAGTGTTCTCATT	CC1
SMC21.T.AGGGATGAGGAGTAGA	CC1
SMC21.T.AGGGATGCAGATGAGC	CC1
SMC21.T.AGGGATGTCCGCTGTT	CC1
SMC21.T.AGGGTGAAGACAATAC	CC1
SMC21.T.AGGGTGAGTAAATGTG	CC1
SMC21.T.AGGGTGATCATGTAGC	CC1
SMC21.T.AGGGTGATCCACTGGG	CC1
SMC21.T.AGGTCATGTTGAACTC	CC1
SMC21.T.AGGTCATTCCCTAATT	CC1
SMC21.T.AGGTCCGGTAAGGGCT	CC1
SMC21.T.AGTAGTCAGAAGAAGC	CC1
SMC21.T.AGTAGTCGTCGCTTTC	CC1
SMC21.T.AGTAGTCGTTCAGACT	CC1
SMC21.T.AGTAGTCTCACGACTA	CC1
SMC21.T.AGTAGTCTCATCATTC	CC1
SMC21.T.AGTCTTTCAATCTGCA	CC1
SMC21.T.AGTCTTTTCCAACCAA	CC1

SMC21.T.AGTGAGGAGGATGCGT	CC1
SMC21.T.AGTGAGGCATTGAGCT	CC1
SMC21.T.AGTGGGAGTCGGCATC	CC1
SMC21.T.AGTGGGATCAACGCTA	CC1
SMC21.T.AGTGGGATCGCGTTTC	CC1
SMC21.T.AGTGGGATCTTGACGA	CC1
SMC21.T.AGTGTCACAACTGGCC	CC1
SMC21.T.AGTGTCACAGCTGTTA	CC1
SMC21.T.AGTGTCAGTGGCAAAC	CC1
SMC21.T.AGTGTCATCAAGATCC	CC1
SMC21.T.AGTGTCATCACCATAG	CC1
SMC21.T.AGTGTCATCACCTTAT	CC1
SMC21.T.AGTTGGTAGGCTAGCA	CC1
SMC21.T.AGTTGGTGTCGAACAG	CC1
SMC21.T.AGTTGGTTCGAGGTAG	CC1
SMC21.T.ATAACGCAGATATGGT	CC1
SMC21.T.ATAACGCAGCTAGGCA	CC1
SMC21.T.ATAACGCCAAGCCATT	CC1
SMC21.T.ATAACGCGTAATCACC	CC1
SMC21.T.ATAAGAGAGAGCCTAG	CC1
SMC21.T.ATAAGAGAGCCCTAAT	CC1
SMC21.T.ATAAGAGCATGTCTCC	CC1
SMC21.T.ATAAGAGTCAAACCGT	CC1
SMC21.T.ATAGACCAGGCTCATT	CC1
SMC21.T.ATAGACCCATGCCTAA	CC1
SMC21.T.ATAGACCTCGGTGTTA	CC1
SMC21.T.ATAGACCTCTGTCTCG	CC1
SMC21.T.ATCACGACACTAAGTC	CC1
SMC21.T.ATCACGACAGTCGATT	CC1
SMC21.T.ATCACGAGTGCAGTAG	CC1
SMC21.T.ATCACGAGTTCTGTTT	CC1
SMC21.T.ATCATCTAGTCATCCA	CC1
SMC21.T.ATCATCTGTCGTGGCT	CC1
SMC21.T.ATCATCTGTTGCCTCT	CC1
SMC21.T.ATCATCTGTTGTACAC	CC1
SMC21.T.ATCATCTTCGATCCCT	CC1
SMC21.T.ATCATGGAGTACCGGA	CC1
SMC21.T.ATCATGGAGTGCGATG	CC1
SMC21.T.ATCATGGCAGACAGGT	CC1
SMC21.T.ATCATGGCAGTCAGCC	CC1
SMC21.T.ATCATGGTCCACGTGG	CC1
SMC21.T.ATCCACCAGACAAGCC	CC1
SMC21.T.ATCCACCAGAGCTGCA	CC1
SMC21.T.ATCCACCCACGGCGTT	CC1
SMC21.T.ATCCACCCATTCACTT	CC1
SMC21.T.ATCCACCTCCCTTGTG	CC1
SMC21.T.ATCCACCTCCGCGGTA	CC1
SMC21.T.ATCCACCTCCTAGAAC	CC1
SMC21.T.ATCCGAAAGATATGCA	CC1
SMC21.T.ATCCGAACAGCCAGAA	CC1
SMC21.T.ATCCGAAGTCTAGTGT	CC1
SMC21.T.ATCGAGTAGACGCAAC	CC1
SMC21.T.ATCGAGTAGATCGATA	CC1
SMC21.T.ATCGAGTAGTACGATA	CC1
SMC21.T.ATCGAGTAGTTAGCGG	CC1
SMC21.T.ATCGAGTCAGTCAGCC	CC1
SMC21.T.ATCGAGTCATGCAACT	CC1
SMC21.T.ATCGAGTGTTCGCGAC	CC1

SMC21.T.ATCTACTAGAGTTGGC	CC1
SMC21.T.ATCTACTCAACGCACC	CC1
SMC21.T.ATCTACTTCCTGCTTG	CC1
SMC21.T.ATCTACTTCCTTTCGG	CC1
SMC21.T.ATCTGCCAGGCTATCT	CC1
SMC21.T.ATCTGCCCACCAGCAC	CC1
SMC21.T.ATCTGCCCACGCATCG	CC1
SMC21.T.ATCTGCCGTCTTGCGG	CC1
SMC21.T.ATCTGCCTCTTCCTTC	CC1
SMC21.T.ATGAGGGAGGCCCGTT	CC1
SMC21.T.ATGAGGGCAAGCCTAT	CC1
SMC21.T.ATGAGGGCACGGTGTC	CC1
SMC21.T.ATGAGGGGTCCTGCTT	CC1
SMC21.T.ATGCGATAGCCCAGCT	CC1
SMC21.T.ATGCGATCAAAGGCGT	CC1
SMC21.T.ATGCGATCAATGGACG	CC1
SMC21.T.ATGCGATGTGCACCAC	CC1
SMC21.T.ATGCGATTCCAAAGTC	CC1
SMC21.T.ATGCGATTCGGCCGAT	CC1
SMC21.T.ATGCGATTCTCTAGGA	CC1
SMC21.T.ATGGGAGAGCGATTCT	CC1
SMC21.T.ATGGGAGCAGGCGATA	CC1
SMC21.T.ATGGGAGTCCGCAAGC	CC1
SMC21.T.ATGGGAGTCGTACCGG	CC1
SMC21.T.ATGTGTGGTATAATGG	CC1
SMC21.T.ATGTGTGGTTGTCGCG	CC1
SMC21.T.ATTACTCGTCACCCAG	CC1
SMC21.T.ATTACTCGTCGTTGTA	CC2
SMC21.T.ATTACTCGTTGCGCAC	CC1
SMC21.T.ATTACTCTCCAAACTG	CC1
SMC21.T.ATTATCCAGCACCGCT	CC1
SMC21.T.ATTATCCAGGATGGTC	CC1
SMC21.T.ATTATCCAGTACGCCC	CC1
SMC21.T.ATTATCCCACATCCGG	CC1
SMC21.T.ATTATCCGTTGTCGCG	CC2
SMC21.T.ATTATCCTCTGCCAGG	CC1
SMC21.T.ATTATCCTCTTTCCTC	CC1
SMC21.T.ATTCTACCACAGACTT	CC1
SMC21.T.ATTCTACCATCGGACC	CC1
SMC21.T.ATTCTACCATGCTGGC	CC1
SMC21.T.ATTCTACGTTACGACT	CC1
SMC21.T.ATTCTACTCTTGTATC	CC1
SMC21.T.ATTGGACAGGGTATCG	CC1
SMC21.T.ATTGGACCAGGCTGAA	CC1
SMC21.T.ATTGGACGTACACCGC	CC1
SMC21.T.ATTGGACGTTATGTGC	CC1
SMC21.T.ATTGGACTCATCGATG	CC1
SMC21.T.ATTGGACTCCCATTAT	CC1
SMC21.T.ATTGGACTCCTTTCGG	CC1
SMC21.T.ATTGGACTCTCTGTCG	CC1
SMC21.T.ATTGGACTCTGCGACG	CC1
SMC21.T.ATTGGTGAGGACACCA	CC1
SMC21.T.ATTGGTGAGGATGTAT	CC1
SMC21.T.ATTGGTGCACGGTAGA	CC1
SMC21.T.ATTGGTGCAGGCTGAA	CC1
SMC21.T.ATTGGTGTCGGCTTGG	CC1
SMC21.T.ATTGGTGTCTCATTCA	CC1
SMC21.T.ATTTCTGAGAGGACGG	CC1

SMC21.T.ATTTCTGCACCTCGTT	CC1
SMC21.T.ATTTCTGCAGCTCCGA	CC1
SMC21.T.ATTTCTGGTGACGCCT	CC1
SMC21.T.ATTTCTGGTTCCCGAG	CC1
SMC21.T.CAACCAAGTATCAGTC	CC1
SMC21.T.CAACCAAGTCTAGGTT	CC1
SMC21.T.CAACCAAGTTGGACCC	CC1
SMC21.T.CAACCAATCAACTCTT	CC1
SMC21.T.CAACCAATCACCACCT	CC1
SMC21.T.CAACCAATCCAAACTG	CC1
SMC21.T.CAACCAATCTGCCCTA	CC1
SMC21.T.CAACCTCAGACCCACC	CC1
SMC21.T.CAACCTCGTAGCGTAG	CC1
SMC21.T.CAACCTCGTATCAGTC	CC1
SMC21.T.CAACCTCTCCAAGTAC	CC1
SMC21.T.CAACCTCTCGCCTGTT	CC1
SMC21.T.CAACCTCTCTCGGACG	CC1
SMC21.T.CAAGAAAAGACCACGA	CC1
SMC21.T.CAAGAAAAGTATTGGA	CC1
SMC21.T.CAAGATCAGCCCAACC	CC1
SMC21.T.CAAGATCGTCTGCGGT	CC1
SMC21.T.CAAGATCTCGAGCCCA	CC1
SMC21.T.CAAGGCCAGTTTAGGA	CC1
SMC21.T.CAAGGCCGTTATTCTC	CC1
SMC21.T.CAAGGCCTCAACCATG	CC1
SMC21.T.CAAGGCCTCAAGCCTA	CC1
SMC21.T.CAAGTTGAGACAAGCC	CC1
SMC21.T.CAAGTTGAGAGTAAGG	CC1
SMC21.T.CAAGTTGAGCCAACAG	CC1
SMC21.T.CAAGTTGGTCCAAGTT	CC1
SMC21.T.CAAGTTGGTGACTACT	CC1
SMC21.T.CAAGTTGTCCTGCCAT	CC1
SMC21.T.CAAGTTGTCGGCCGAT	CC1
SMC21.T.CACAAACTCTGGTATG	CC1
SMC21.T.CACACAAGTCTTGTCC	CC1
SMC21.T.CACACCTCATTCCTGC	CC1
SMC21.T.CACACCTGTGAGTGAC	CC1
SMC21.T.CACACCTTCCAAAGTC	CC1
SMC21.T.CACACCTTCGCAAGCC	CC1
SMC21.T.CACACTCAGAGACGAA	CC1
SMC21.T.CACACTCAGCCAACAG	CC1
SMC21.T.CACACTCCACAGCGTC	CC1
SMC21.T.CACACTCCACCGATAT	CC1
SMC21.T.CACACTCGTTGATTCG	CC1
SMC21.T.CACACTCTCATGTCTT	CC1
SMC21.T.CACAGGCCATTAGGCT	CC1
SMC21.T.CACAGGCGTGTTGGGA	CC1
SMC21.T.CACAGGCTCGTCGTTC	CC1
SMC21.T.CACAGTAAGTGTACGG	CC1
SMC21.T.CACAGTACAAACGCGA	CC1
SMC21.T.CACAGTAGTGTATGGG	CC1
SMC21.T.CACAGTATCACATAGC	CC1
SMC21.T.CACAGTATCTGGCGTG	CC1
SMC21.T.CACATAGAGGCATGGT	CC1
SMC21.T.CACATAGAGGCCCTCA	CC1
SMC21.T.CACATAGTCATTATCC	CC1
SMC21.T.CACATTTAGCTACCTA	CC1
SMC21.T.CACATTTGTCCAGTGC	CC1

SMC21.T.CACATTTTCATCTGCC	CC1
SMC21.T.CACCACTTCTGCGGCA	CC1
SMC21.T.CACCAGGCATTCTTAC	CC1
SMC21.T.CACCAGGGTCTAAAGA	CC1
SMC21.T.CACCTTGAGGCCGAAT	CC1
SMC21.T.CACCTTGCAAAGGAAG	CC1
SMC21.T.CACCTTGCACTGTCGG	CC1
SMC21.T.CACCTTGGTTATCCGA	CC1
SMC21.T.CACTCCAGTCTCTTTA	CC1
SMC21.T.CACTCCAGTTAGGGTG	CC1
SMC21.T.CACTCCAGTTCGCGAC	CC1
SMC21.T.CACTCCATCCATGAAC	CC1
SMC21.T.CACTCCATCCATTCTA	CC1
SMC21.T.CACTCCATCTTCCTTC	CC1
SMC21.T.CAGAATCAGGAATTAC	CC1
SMC21.T.CAGAATCAGTCCCACG	CC1
SMC21.T.CAGAATCCAAGAAAGG	CC1
SMC21.T.CAGAATCCATTGGGCC	CC1
SMC21.T.CAGAATCGTTCCCGAG	CC1
SMC21.T.CAGAATCTCACCACCT	CC2
SMC21.T.CAGAGAGAGGCCCTTG	CC1
SMC21.T.CAGAGAGTCTGGTTCC	CC1
SMC21.T.CAGATCAAGAACTCGG	CC1
SMC21.T.CAGATCAAGACCCACC	CC1
SMC21.T.CAGATCAAGGGTCGAT	CC1
SMC21.T.CAGATCATCGTACGGC	CC1
SMC21.T.CAGATCATCTTGTACT	CC1
SMC21.T.CAGCAGCAGTCGTTTG	CC1
SMC21.T.CAGCATATCGGACAAG	CC1
SMC21.T.CAGCCGACAGATGAGC	CC1
SMC21.T.CAGCGACGTTGTCTTT	CC1
SMC21.T.CAGCGACTCAACACCA	CC1
SMC21.T.CAGCTAAAGACTCGGA	CC1
SMC21.T.CAGCTAACAGTTTACG	CC1
SMC21.T.CAGCTAACATTTCACT	CC1
SMC21.T.CAGCTAAGTTGTTTGG	CC1
SMC21.T.CAGCTAATCCACGACG	CC1
SMC21.T.CAGCTAATCTTGAGAC	CC1
SMC21.T.CAGCTGGAGACAGACC	CC1
SMC21.T.CAGCTGGCAAGTCTAC	CC1
SMC21.T.CAGCTGGGTCCAGTGC	CC1
SMC21.T.CAGCTGGTCGCATGGC	CC1
SMC21.T.CAGGTGCAGGCCCTTG	CC1
SMC21.T.CAGGTGCCACAGAGGT	CC1
SMC21.T.CAGGTGCGTCCGAACC	CC1
SMC21.T.CAGGTGCTCAGAGGTG	CC1
SMC21.T.CAGGTGCTCATTGCGA	CC1
SMC21.T.CAGTAACAGACTGTAA	CC1
SMC21.T.CAGTAACAGAGTTGGC	CC1
SMC21.T.CAGTAACCATGGTTGT	CC1
SMC21.T.CAGTAACGTAGGCTGA	CC1
SMC21.T.CAGTAACTCAGGCGAA	CC1
SMC21.T.CAGTAACTCCCTTGCA	CC1
SMC21.T.CAGTCCTAGAAACGAG	CC1
SMC21.T.CAGTCCTAGCGTTCCG	CC1
SMC21.T.CAGTCCTAGTGTCCAT	CC1
SMC21.T.CATATGGAGAGTGAGA	CC1
SMC21.T.CATATGGCACAAGCCC	CC1

SMC21.T.CATATGGTCCTAGGGC	CC1
SMC21.T.CATATTCAGATGTAAC	CC1
SMC21.T.CATATTCCAAGCTGAG	CC1
SMC21.T.CATATTCCATCCTTGC	CC1
SMC21.T.CATATTCTCAAAGACA	CC2
SMC21.T.CATCAAGAGACCGGAT	CC1
SMC21.T.CATCAAGAGCCAGAAC	CC1
SMC21.T.CATCAAGGTTGGTAAA	CC1
SMC21.T.CATCAAGTCCAGTAGT	CC1
SMC21.T.CATCAAGTCCGAATGT	CC1
SMC21.T.CATCAGACAGCTTCGG	CC1
SMC21.T.CATCAGAGTCTAGCGC	CC1
SMC21.T.CATCAGAGTGATGATA	CC1
SMC21.T.CATCCACAGAATGTTG	CC1
SMC21.T.CATCCACAGAGCTATA	CC1
SMC21.T.CATCCACAGCCCGAAA	CC1
SMC21.T.CATCCACAGTTACCCA	CC1
SMC21.T.CATCCACCACAACGTT	CC1
SMC21.T.CATCCACCACAGACTT	CC1
SMC21.T.CATCGAAAGCGTCAAG	CC1
SMC21.T.CATCGAAAGGCGTACA	CC1
SMC21.T.CATCGAAGTAGGACAC	CC1
SMC21.T.CATCGAATCGCTTGTC	CC1
SMC21.T.CATCGAATCTTTACGT	CC1
SMC21.T.CATCGGGAGTGAACAT	CC1
SMC21.T.CATCGGGTCAATACCG	CC1
SMC21.T.CATCGGGTCCAAGCCG	CC1
SMC21.T.CATCGGGTCTACTATC	CC1
SMC21.T.CATCGGGTCTCGTATT	CC1
SMC21.T.CATGACAAGCAACGGT	CC1
SMC21.T.CATGACAGTCCGTCAG	CC1
SMC21.T.CATGACAGTGGCAAAC	CC1
SMC21.T.CATGACATCCCGGATG	CC1
SMC21.T.CATGCCTAGTACTTGC	CC1
SMC21.T.CATGCCTAGTCTCCTC	CC1
SMC21.T.CATGCCTAGTGACTCT	CC1
SMC21.T.CATGGCGAGAGGACGG	CC1
SMC21.T.CATGGCGGTAAGCACG	CC1
SMC21.T.CATGGCGTCACGCGGT	CC1
SMC21.T.CATGGCGTCCGTCATC	CC1
SMC21.T.CATTATCAGTTTAGGA	CC1
SMC21.T.CATTATCCACCGAATT	CC1
SMC21.T.CATTATCCAGTGAGTG	CC1
SMC21.T.CATTATCCATCTCGCT	CC2
SMC21.T.CATTATCTCAGTTCGA	CC1
SMC21.T.CATTCGCAGCAGATCG	CC1
SMC21.T.CATTCGCGTATAATGG	CC1
SMC21.T.CATTCGCTCGAGAGCA	CC1
SMC21.T.CCAATCCCACTGTTAG	CC1
SMC21.T.CCAATCCGTTGCCTCT	CC1
SMC21.T.CCAATCCTCGTACCGG	CC1
SMC21.T.CCAATCCTCTTCTGGC	CC1
SMC21.T.CCACCTATCATAAAGG	CC1
SMC21.T.CCACGGAGTAGCGTAG	CC1
SMC21.T.CCACGGAGTTAGAACA	CC1
SMC21.T.CCACTACAGCTGGAAC	CC1
SMC21.T.CCACTACCAAATCCGT	CC1
SMC21.T.CCACTACCATTGGTAC	CC1

SMC21.T.CCAGCGATCCATGAAC	CC1
SMC21.T.CCATGTCAGACCTTTG	CC1
SMC21.T.CCATGTCAGGTGCTTT	CC1
SMC21.T.CCATGTCGTGAGCGAT	CC1
SMC21.T.CCATTCGAGCTCCCAG	CC1
SMC21.T.CCATTCGAGTTCGCAT	CC1
SMC21.T.CCATTCGCAATGCCAT	CC1
SMC21.T.CCATTCGCACAGCGTC	CC1
SMC21.T.CCATTCGCAGTAACGG	CC1
SMC21.T.CCATTCGCATCCAACA	CC1
SMC21.T.CCATTCGGTCTTCTCG	CC1
SMC21.T.CCCAATCCACATCTTT	CC1
SMC21.T.CCCAATCTCTGGTGTA	CC1
SMC21.T.CCCAGTTAGCTGCGAA	CC1
SMC21.T.CCCAGTTAGGGCACTA	CC1
SMC21.T.CCCAGTTAGTGTGGCA	CC1
SMC21.T.CCCAGTTCACCATGTA	CC1
SMC21.T.CCCAGTTGTTGCTCCT	CC1
SMC21.T.CCCAGTTTCCCTCAGT	CC1
SMC21.T.CCCAGTTTCCTTCAAT	CC1
SMC21.T.CCCAGTTTCTCCTATA	CC1
SMC21.T.CCCATACAGACTCGGA	CC1
SMC21.T.CCCATACAGTCCGGTC	CC1
SMC21.T.CCCATACGTACCGTTA	CC1
SMC21.T.CCCATACGTCAAAGCG	CC1
SMC21.T.CCCATACTCTCGCTTG	CC1
SMC21.T.CCCTCCTAGACAGAGA	CC1
SMC21.T.CCCTCCTGTCGATTGT	CC1
SMC21.T.CCCTCCTGTGATAAGT	CC1
SMC21.T.CCCTCCTTCAACGCTA	CC1
SMC21.T.CCGGGATAGAGAACAG	CC1
SMC21.T.CCGGGATAGGCTCATT	CC1
SMC21.T.CCGGGATCACCTATCC	CC1
SMC21.T.CCGGGATGTCATACTG	CC1
SMC21.T.CCGGGATGTGTGCCTG	CC1
SMC21.T.CCGGTAGAGCTGCAAG	CC1
SMC21.T.CCGGTAGCAACACCTA	CC1
SMC21.T.CCGGTAGCATGGGACA	CC1
SMC21.T.CCGTACTCACCATCCT	CC1
SMC21.T.CCGTACTGTCGCATCG	CC1
SMC21.T.CCGTGGAAGACAAGCC	CC1
SMC21.T.CCGTGGAAGTATCGAA	CC1
SMC21.T.CCGTGGACAAGGCTCC	CC1
SMC21.T.CCGTGGACATGAAGTA	CC1
SMC21.T.CCGTTCAAGTGGCACA	CC1
SMC21.T.CCGTTCACACGGACAA	CC1
SMC21.T.CCGTTCACATCCGTGG	CC1
SMC21.T.CCGTTCAGTTCACGGC	CC1
SMC21.T.CCTAAAGAGGTCGGAT	CC1
SMC21.T.CCTAAAGGTGCGGTAA	CC1
SMC21.T.CCTAAAGTCGTACCGG	CC1
SMC21.T.CCTACACGTTCTGAAC	CC1
SMC21.T.CCTACACTCACCTCGT	CC1
SMC21.T.CCTACCACATCCCATC	CC1
SMC21.T.CCTACCAGTGGGTATG	CC1
SMC21.T.CCTAGCTAGGACTGGT	CC1
SMC21.T.CCTAGCTAGGATGGAA	CC1
SMC21.T.CCTAGCTCATTCTCAT	CC1

SMC21.T.CCTAGCTGTACACCGC	CC1
SMC21.T.CCTAGCTGTCAGAGGT	CC1
SMC21.T.CCTAGCTGTCCTAGCG	CC1
SMC21.T.CCTAGCTGTCGCGGTT	CC1
SMC21.T.CCTAGCTTCGGCATCG	CC1
SMC21.T.CCTAGCTTCTTACCGC	CC1
SMC21.T.CCTATTAAGCCACTAT	CC1
SMC21.T.CCTATTACACCGGAAA	CC1
SMC21.T.CCTATTACATTCTTAC	CC1
SMC21.T.CCTATTAGTCTTGCGG	CC1
SMC21.T.CCTATTAGTGAACCTT	CC1
SMC21.T.CCTATTATCAAGGCTT	CC1
SMC21.T.CCTCAGTAGAAGGGTA	CC1
SMC21.T.CCTCAGTAGTTGAGAT	CC1
SMC21.T.CCTCTGACACATGACT	CC1
SMC21.T.CCTCTGACAGCGTCCA	CC1
SMC21.T.CCTCTGATCGGATGGA	CC1
SMC21.T.CCTTACGCATCGGACC	CC1
SMC21.T.CCTTCCCCAGCCAATT	CC1
SMC21.T.CCTTCCCCAGCCAGAA	CC1
SMC21.T.CCTTCCCGTGCTGTAT	CC1
SMC21.T.CCTTCCCTCGGGAGTA	CC1
SMC21.T.CCTTCGAAGACCTTTG	CC1
SMC21.T.CCTTCGACAGTAGAGC	CC1
SMC21.T.CCTTCGAGTTATTCTC	CC1
SMC21.T.CCTTCGAGTTTGCATG	CC1
SMC21.T.CCTTTCTCACCCAGTG	CC1
SMC21.T.CCTTTCTCATCGTCGG	CC1
SMC21.T.CCTTTCTGTCATGCCG	CC1
SMC21.T.CCTTTCTTCTTGAGGT	CC1
SMC21.T.CGAACATAGTCCCACG	CC1
SMC21.T.CGAACATGTGCTTCTC	CC1
SMC21.T.CGAACATTCGAATGCT	CC1
SMC21.T.CGAACATTCGTTTAGG	CC1
SMC21.T.CGAATGTCATATACGC	CC1
SMC21.T.CGAATGTCATCGATGT	CC1
SMC21.T.CGAATGTGTGGCAAAC	CC1
SMC21.T.CGAATGTTCGCCAGCA	CC1
SMC21.T.CGACCTTCACGTCAGC	CC1
SMC21.T.CGACTTCCAATGGAGC	CC1
SMC21.T.CGACTTCGTAGCTAAA	CC1
SMC21.T.CGACTTCGTGCATCTA	CC1
SMC21.T.CGACTTCGTTCCACAA	CC1
SMC21.T.CGACTTCGTTTAGGAA	CC2
SMC21.T.CGACTTCTCGCTAGCG	CC1
SMC21.T.CGAGAAGAGACAAAGG	CC1
SMC21.T.CGAGAAGCAGCTGGCT	CC1
SMC21.T.CGAGAAGGTCTAGTCA	CC1
SMC21.T.CGAGAAGGTGTTGAGG	CC1
SMC21.T.CGAGCACAGTGAAGTT	CC1
SMC21.T.CGAGCACCAGGAACGT	CC1
SMC21.T.CGAGCACGTAGTGAAT	CC1
SMC21.T.CGAGCCAAGCTCCCAG	CC1
SMC21.T.CGAGCCATCCACGTGG	CC1
SMC21.T.CGAGCCATCTTATCTG	CC1
SMC21.T.CGATCGGAGGGATACC	CC1
SMC21.T.CGATCGGGTAGGCATG	CC1
SMC21.T.CGATCGGGTATAGTAG	CC1

SMC21.T.CGATCGGGTTACGTCA	CC1
SMC21.T.CGATCGGTCGTACCGG	CC1
SMC21.T.CGATGGCAGTGGTAAT	CC1
SMC21.T.CGATGGCGTAGCGTCC	CC1
SMC21.T.CGATGGCTCTACCAGA	CC1
SMC21.T.CGATGTAAGCTACCGC	CC1
SMC21.T.CGATGTACATGGGACA	CC1
SMC21.T.CGATGTAGTCTAGCGC	CC1
SMC21.T.CGATGTATCCCATTTA	CC1
SMC21.T.CGATTGAAGGACATTA	CC1
SMC21.T.CGATTGACAAATCCGT	CC1
SMC21.T.CGATTGACACCGCTAG	CC1
SMC21.T.CGATTGACAGACGCCT	CC1
SMC21.T.CGATTGATCTTACCGC	CC1
SMC21.T.CGCCAAGAGTACGATA	CC1
SMC21.T.CGCCAAGGTACAGCAG	CC1
SMC21.T.CGCCAAGTCCGATATG	CC1
SMC21.T.CGCCAAGTCTCGCATC	CC1
SMC21.T.CGCGGTACAGCTTAAC	CC1
SMC21.T.CGCGGTATCCCTTGTG	CC1
SMC21.T.CGCGTTTTCAGTTGAC	CC1
SMC21.T.CGCTATCAGATCCCGC	CC1
SMC21.T.CGCTATCGTATCGCAT	CC1
SMC21.T.CGCTATCGTCTAGGTT	CC1
SMC21.T.CGCTGGAAGCGTAATA	CC1
SMC21.T.CGCTGGAGTAGGCATG	CC1
SMC21.T.CGCTGGAGTTACCGAT	CC1
SMC21.T.CGCTTCAAGCGCTTAT	CC1
SMC21.T.CGCTTCAAGCTTATCG	CC1
SMC21.T.CGCTTCAAGGAGTACC	CC1
SMC21.T.CGCTTCACACGGCTAC	CC1
SMC21.T.CGCTTCACATGCAACT	CC1
SMC21.T.CGCTTCAGTGCCTTGG	CC1
SMC21.T.CGGACACTCGTACCGG	CC1
SMC21.T.CGGACGTCAACACCCG	CC1
SMC21.T.CGGACGTCACCCAGTG	CC1
SMC21.T.CGGACGTCATTCTTAC	CC1
SMC21.T.CGGACGTCATTTGCCC	CC1
SMC21.T.CGGACGTGTAGCGCAA	CC1
SMC21.T.CGGACTGAGTCCGGTC	CC1
SMC21.T.CGGACTGCAAATTGCC	CC1
SMC21.T.CGGACTGGTGCACTTA	CC1
SMC21.T.CGGACTGTCAAACAAG	CC1
SMC21.T.CGGAGCTAGTAATCCC	CC1
SMC21.T.CGGAGCTAGTCGTTTG	CC1
SMC21.T.CGGAGCTCAATCCGAT	CC1
SMC21.T.CGGAGCTGTCAAACTC	CC1
SMC21.T.CGGAGCTGTCCCGACA	CC1
SMC21.T.CGGAGCTGTCCGAACC	CC1
SMC21.T.CGGAGCTTCTCCAACC	CC1
SMC21.T.CGGAGTCAGGGATACC	CC1
SMC21.T.CGGAGTCCAAGTACCT	CC1
SMC21.T.CGGAGTCCAGACAAGC	CC1
SMC21.T.CGGAGTCTCACAACGT	CC1
SMC21.T.CGGAGTCTCTCCTATA	CC1
SMC21.T.CGGCTAGAGGACATTA	CC1
SMC21.T.CGGCTAGGTAGCGTAG	CC1
SMC21.T.CGGCTAGTCAGAGCTT	CC1

SMC21.T.CGGGTCAGTAAATGTG	CC1
SMC21.T.CGGGTCAGTCCTGCTT	CC1
SMC21.T.CGGGTCATCACTATTC	CC1
SMC21.T.CGGGTCATCGTCTGCT	CC1
SMC21.T.CGGTTAAAGTTCGATC	CC1
SMC21.T.CGGTTAAAGTTGAGTA	CC1
SMC21.T.CGGTTAACACAGGAGT	CC1
SMC21.T.CGGTTAAGTACAAGTA	CC1
SMC21.T.CGGTTAAGTTTGGGCC	CC1
SMC21.T.CGTAGCGAGAGATGAG	CC1
SMC21.T.CGTAGCGTCAGGCGAA	CC1
SMC21.T.CGTAGGCAGACCACGA	CC1
SMC21.T.CGTAGGCAGACTAAGT	CC1
SMC21.T.CGTAGGCCAGGCAGTA	CC1
SMC21.T.CGTAGGCGTCATGCAT	CC1
SMC21.T.CGTCACTAGGCTACGA	CC1
SMC21.T.CGTCACTCAAGTCTAC	CC1
SMC21.T.CGTCACTGTTATCCGA	CC1
SMC21.T.CGTCACTGTTGACGTT	CC1
SMC21.T.CGTCACTTCAGATAAG	CC1
SMC21.T.CGTCACTTCCACGTGG	CC1
SMC21.T.CGTCAGGGTAAATACG	CC1
SMC21.T.CGTCAGGGTCATACTG	CC1
SMC21.T.CGTCAGGGTGCGCTTG	CC1
SMC21.T.CGTCCATTCCGCAGTG	CC1
SMC21.T.CGTCCATTCTGGTTCC	CC1
SMC21.T.CGTCCATTCTTCATGT	CC1
SMC21.T.CGTCTACCAAGAGTCG	CC1
SMC21.T.CGTCTACCAATCGGTT	CC1
SMC21.T.CGTCTACCACGAAACG	CC1
SMC21.T.CGTCTACGTCAAGCGA	CC1
SMC21.T.CGTCTACGTCAGTGGA	CC1
SMC21.T.CGTCTACGTCCGCTGA	CC1
SMC21.T.CGTGAGCAGCGTGTCC	CC1
SMC21.T.CGTGAGCAGTGCGTGA	CC1
SMC21.T.CGTGAGCCACCTCGTT	CC1
SMC21.T.CGTGAGCTCGGAGGTA	CC1
SMC21.T.CGTGTAAAGCTACCTA	CC1
SMC21.T.CGTGTAAAGGAGTTGC	CC1
SMC21.T.CGTGTAAAGGCTATCT	CC1
SMC21.T.CGTGTAACAACGATCT	CC1
SMC21.T.CGTGTAAGTACATCCA	CC1
SMC21.T.CGTGTCTCAGGGTTAG	CC1
SMC21.T.CGTGTCTTCCACGTTC	CC1
SMC21.T.CGTTAGAAGAGCAATT	CC1
SMC21.T.CGTTCTGAGTCGTTTG	CC1
SMC21.T.CGTTCTGTCACATGCA	CC1
SMC21.T.CGTTCTGTCCCACTTG	CC1
SMC21.T.CGTTCTGTCTGAGTGT	CC1
SMC21.T.CGTTGGGCAAGGTTCT	CC1
SMC21.T.CGTTGGGCAAGGTTTC	CC1
SMC21.T.CGTTGGGTCCTCAATT	CC1
SMC21.T.CTAACTTAGAATGTTG	CC1
SMC21.T.CTAACTTCACGTCAGC	CC1
SMC21.T.CTAACTTTCAGCATGT	CC1
SMC21.T.CTAACTTTCCGCGCAA	CC1
SMC21.T.CTAAGACCAGATCTGT	CC1
SMC21.T.CTAAGACTCCTTTACA	CC1

SMC21.T.CTAATGGAGCCTTGAT	CC1
SMC21.T.CTAATGGAGCGAAGGG	CC1
SMC21.T.CTAATGGCAGGGTATG	CC1
SMC21.T.CTAATGGCATCGGGTC	CC1
SMC21.T.CTAATGGTCGGTGTCG	CC1
SMC21.T.CTAATGGTCTTTACAC	CC1
SMC21.T.CTACACCCAAGCTGGA	CC1
SMC21.T.CTACACCTCCAGATCA	CC1
SMC21.T.CTACACCTCTGTTTGT	CC1
SMC21.T.CTACACCTCTTGCAAG	CC1
SMC21.T.CTACATTAGCCCGAAA	CC1
SMC21.T.CTACATTCAAGTAATG	CC1
SMC21.T.CTACATTCAGGTTTCA	CC1
SMC21.T.CTACATTCATGCATGT	CC1
SMC21.T.CTACATTTCAACACAC	CC1
SMC21.T.CTACATTTCACCCTCA	CC1
SMC21.T.CTACATTTCAGAGCTT	CC1
SMC21.T.CTACATTTCCACGTTC	CC1
SMC21.T.CTACATTTCTATCCTA	CC1
SMC21.T.CTACCCAAGTGTACTC	CC1
SMC21.T.CTACCCACAAGCCATT	CC1
SMC21.T.CTACCCATCCCACTTG	CC1
SMC21.T.CTACCCATCTACTCAT	CC1
SMC21.T.CTACGTCAGTGTACTC	CC1
SMC21.T.CTACGTCGTACAGTGG	CC1
SMC21.T.CTACGTCGTCTCTCTG	CC1
SMC21.T.CTAGAGTAGGCGTACA	CC1
SMC21.T.CTAGAGTAGGTAGCTG	CC1
SMC21.T.CTAGAGTCAGACGCTC	CC1
SMC21.T.CTAGCCTAGAACAACT	CC1
SMC21.T.CTAGCCTCAAGTAGTA	CC1
SMC21.T.CTAGCCTCACAGGCCT	CC1
SMC21.T.CTAGCCTCATTCCTGC	CC1
SMC21.T.CTAGCCTTCGGTCCGA	CC1
SMC21.T.CTAGTGAAGCGTGTCC	CC1
SMC21.T.CTAGTGAAGGGTTCCC	CC1
SMC21.T.CTAGTGACACGTTGGC	CC1
SMC21.T.CTAGTGAGTGACAAAT	CC1
SMC21.T.CTAGTGATCCTAGGGC	CC1
SMC21.T.CTAGTGATCTTGTCAT	CC1
SMC21.T.CTCACACAGGACGAAA	CC1
SMC21.T.CTCACACTCATGTGGT	CC1
SMC21.T.CTCACACTCTAACTTC	CC1
SMC21.T.CTCACACTCTGATACG	CC1
SMC21.T.CTCAGAACACAAGACG	CC1
SMC21.T.CTCAGAACATTTGCTT	CC1
SMC21.T.CTCAGAAGTCTGATCA	CC1
SMC21.T.CTCATTACAAGTAGTA	CC1
SMC21.T.CTCATTACATCGGACC	CC1
SMC21.T.CTCATTATCATGTAGC	CC1
SMC21.T.CTCCTAGAGAGCCTAG	CC1
SMC21.T.CTCCTAGAGCCCAGCT	CC1
SMC21.T.CTCCTAGAGGCTCTTA	CC1
SMC21.T.CTCCTAGTCTGAGTGT	CC1
SMC21.T.CTCGAAAGTACAAGTA	CC1
SMC21.T.CTCGAAATCTTGTATC	CC1
SMC21.T.CTCGAGGGTATATGAG	CC1
SMC21.T.CTCGGAGAGTTATCGC	CC1

SMC21.T.CTCGGAGCAAGCCCAC	CC1
SMC21.T.CTCGGAGGTCCCTACT	CC1
SMC21.T.CTCGGGAAGAGCTTCT	CC1
SMC21.T.CTCGGGACAAGTTGTC	CC1
SMC21.T.CTCGGGAGTCAATGTC	CC1
SMC21.T.CTCGGGAGTCCATCCT	CC1
SMC21.T.CTCGGGATCACGAAGG	CC1
SMC21.T.CTCGGGATCTCAAACG	CC1
SMC21.T.CTCGTACGTCCAGTTA	CC1
SMC21.T.CTCGTCAGTGTGAAAT	CC1
SMC21.T.CTCGTCAGTTCCCGAG	CC1
SMC21.T.CTCTAATAGTTTAGGA	CC1
SMC21.T.CTCTAATCACGAAAGC	CC1
SMC21.T.CTCTAATCAGTCTTCC	CC1
SMC21.T.CTCTACGCAGTGGAGT	CC1
SMC21.T.CTCTACGGTGCGGTAA	CC1
SMC21.T.CTCTACGTCATGGTCA	CC1
SMC21.T.CTCTACGTCCAAACAC	CC1
SMC21.T.CTCTGGTCACCCTATC	CC1
SMC21.T.CTCTGGTGTAGAGCTG	CC1
SMC21.T.CTGAAACAGACATAAC	CC1
SMC21.T.CTGAAACAGGATGGAA	CC1
SMC21.T.CTGAAACGTAGGCTGA	CC1
SMC21.T.CTGAAACGTCTGGTCG	CC2
SMC21.T.CTGAAACGTGGCTCCA	CC1
SMC21.T.CTGAAACGTTACAGAA	CC1
SMC21.T.CTGAAACTCGACAGCC	CC1
SMC21.T.CTGAAACTCGGCCGAT	CC1
SMC21.T.CTGAAACTCTAGCACA	CC1
SMC21.T.CTGAAGTAGACAGAGA	CC1
SMC21.T.CTGAAGTCAATCAGAA	CC1
SMC21.T.CTGAAGTTCAGCTCTC	CC1
SMC21.T.CTGAAGTTCTAAGCCA	CC1
SMC21.T.CTGATAGCAAACAACA	CC1
SMC21.T.CTGATAGCATAACCTG	CC2
SMC21.T.CTGATAGCATCATCCC	CC1
SMC21.T.CTGATAGTCACTCCTG	CC1
SMC21.T.CTGATAGTCCTGTAGA	CC1
SMC21.T.CTGATCCAGAGCTGGT	CC1
SMC21.T.CTGATCCGTAATCGTC	CC1
SMC21.T.CTGCCTAAGGCTCAGA	CC1
SMC21.T.CTGCCTACACCAGGTC	CC1
SMC21.T.CTGCCTATCGTAGGTT	CC1
SMC21.T.CTGCGGACAGACGCCT	CC1
SMC21.T.CTGCGGAGTCCGAACC	CC1
SMC21.T.CTGCGGAGTTGAGTTC	CC1
SMC21.T.CTGCGGATCTCGTTTA	CC1
SMC21.T.CTGCTGTAGTACGCGA	CC1
SMC21.T.CTGCTGTAGTGTCTCA	CC1
SMC21.T.CTGCTGTCAAGAAAGG	CC1
SMC21.T.CTGCTGTCAGCTGCAC	CC1
SMC21.T.CTGCTGTGTTGGTTTG	CC1
SMC21.T.CTGCTGTTCTGTACGA	CC1
SMC21.T.CTGGTCTAGTTATCGC	CC1
SMC21.T.CTGGTCTCAGGACGTA	CC1
SMC21.T.CTGGTCTCATCACAAC	CC1
SMC21.T.CTGTGCTAGGCAATTA	CC1
SMC21.T.CTGTTTAAGGGCTCTC	CC1

SMC21.T.CTGTTTAGTTCGCTAA	CC1
SMC21.T.CTGTTTAGTTTAGCTG	CC1
SMC21.T.CTGTTTATCCTATGTT	CC1
SMC21.T.CTGTTTATCGCCCTTA	CC1
SMC21.T.CTTAACTTCATGCTCC	CC1
SMC21.T.CTTAACTTCCACGACG	CC1
SMC21.T.CTTAACTTCCGAGCCA	CC1
SMC21.T.CTTACCGAGGATGCGT	CC1
SMC21.T.CTTACCGTCGTTACGA	CC1
SMC21.T.CTTAGGAGTTGCCTCT	CC1
SMC21.T.CTTAGGATCAACACGT	CC1
SMC21.T.CTTAGGATCAAGGCTT	CC1
SMC21.T.CTTCTCTCACTCAGGC	CC1
SMC21.T.CTTCTCTCATACGCCG	CC1
SMC21.T.CTTCTCTCATGCCTTC	CC1
SMC21.T.CTTGGCTCAGCCTATA	CC1
SMC21.T.CTTGGCTGTTCTGTTT	CC1
SMC21.T.CTTGGCTTCACTGGGC	CC1
SMC21.T.CTTGGCTTCGAGAGCA	CC1
SMC21.T.CTTTGCGCAGCTGCAC	CC1
SMC21.T.CTTTGCGTCAGCTCTC	CC1
SMC21.T.CTTTGCGTCATGCTCC	CC1
SMC21.T.CTTTGCGTCGGCCGAT	CC1
SMC21.T.CTTTGCGTCTTTACGT	CC1
SMC21.T.GAAACTCAGCCCAATT	CC1
SMC21.T.GAAACTCGTTCGTTGA	CC1
SMC21.T.GAAACTCTCAGCAACT	CC1
SMC21.T.GAAATGAAGAGACTAT	CC1
SMC21.T.GAAATGAAGGCTAGCA	CC1
SMC21.T.GAAATGAGTAGCGCAA	CC1
SMC21.T.GAAATGATCGTGGGAA	CC1
SMC21.T.GAACATCAGCTGCCCA	CC1
SMC21.T.GAACATCGTATTACCG	CC1
SMC21.T.GAACATCTCGCCAGCA	CC1
SMC21.T.GAACATCTCTGGTTCC	CC1
SMC21.T.GAACCTACACCGAAAG	CC1
SMC21.T.GAACCTATCTTAACCT	CC1
SMC21.T.GAACGGAAGGCACATG	CC1
SMC21.T.GAACGGAGTAGAGGAA	CC1
SMC21.T.GAACGGAGTTCCACAA	CC1
SMC21.T.GAACGGATCTGTGCAA	CC1
SMC21.T.GAAGCAGTCCCTGACT	CC1
SMC21.T.GAAGCAGTCTCGAGTA	CC1
SMC21.T.GAATAAGCAGCGATCC	CC1
SMC21.T.GAATAAGGTACCGTTA	CC1
SMC21.T.GAATAAGTCCGCGCAA	CC1
SMC21.T.GAATGAAAGAGCCCAA	CC2
SMC21.T.GAATGAACAATCGAAA	CC1
SMC21.T.GACACGCAGTGGAGAA	CC1
SMC21.T.GACACGCAGTTAGCGG	CC1
SMC21.T.GACACGCCAAACGCGA	CC1
SMC21.T.GACAGAGAGAGACGAA	CC1
SMC21.T.GACAGAGAGGGCTTCC	CC1
SMC21.T.GACAGAGCATGGGACA	CC1
SMC21.T.GACAGAGGTAGTGAAT	CC1
SMC21.T.GACCAATCAGACGCAA	CC1
SMC21.T.GACCAATGTATCACCA	CC1
SMC21.T.GACCTGGAGGGTGTGT	CC1

SMC21.T.GACCTGGAGGTGCACA	CC1
SMC21.T.GACCTGGGTCTTGCGG	CC1
SMC21.T.GACGCGTAGACGACGT	CC1
SMC21.T.GACGCGTAGATCCCGC	CC2
SMC21.T.GACGCGTAGCTGAACG	CC1
SMC21.T.GACGCGTCAAGCGTAG	CC1
SMC21.T.GACGCGTCACAACGTT	CC1
SMC21.T.GACGCGTCAGGCAGTA	CC1
SMC21.T.GACGCGTCATTTCAGG	CC1
SMC21.T.GACGCGTGTAATCGTC	CC1
SMC21.T.GACGGCTCAATGTTGC	CC1
SMC21.T.GACGGCTGTGTGGTTT	CC1
SMC21.T.GACGGCTTCTGTCTAT	CC1
SMC21.T.GACGTGCAGCCGTCGT	CC2
SMC21.T.GACGTGCAGGGCTTGA	CC1
SMC21.T.GACGTGCAGTACGCGA	CC1
SMC21.T.GACGTGCAGTTGAGAT	CC1
SMC21.T.GACGTGCCAAGTCTAC	CC1
SMC21.T.GACGTGCGTTCTCATT	CC1
SMC21.T.GACGTTAAGTTATCGC	CC1
SMC21.T.GACGTTATCATCTGTT	CC1
SMC21.T.GACGTTATCGTTGACA	CC1
SMC21.T.GACTAACGTGTGTGCC	CC1
SMC21.T.GACTACATCCAGATCA	CC1
SMC21.T.GACTACATCTTGAGAC	CC1
SMC21.T.GACTGCGAGAGAGCTC	CC1
SMC21.T.GAGCAGAAGACAATAC	CC1
SMC21.T.GAGCAGAGTTCGGGCT	CC1
SMC21.T.GAGCAGATCAACGAAA	CC1
SMC21.T.GAGCAGATCATACGGT	CC1
SMC21.T.GAGGTGACAATCTGCA	CC1
SMC21.T.GAGGTGAGTGGTACAG	CC1
SMC21.T.GAGGTGATCAACGAAA	CC1
SMC21.T.GAGGTGATCGTTACAG	CC1
SMC21.T.GAGGTGATCTTTAGTC	CC1
SMC21.T.GAGTCCGCAGCCTTTC	CC1
SMC21.T.GAGTCCGGTAAGTTCC	CC1
SMC21.T.GAGTCCGGTTTACTCT	CC1
SMC21.T.GAGTCCGTCACGGTTA	CC1
SMC21.T.GATCAGTAGGTCATCT	CC1
SMC21.T.GATCAGTCAAGGACTG	CC1
SMC21.T.GATCAGTCACCGAATT	CC1
SMC21.T.GATCGATAGATTACCC	CC1
SMC21.T.GATCGATAGTCAAGGC	CC1
SMC21.T.GATCGATCAATACGCT	CC1
SMC21.T.GATCGATCAATCAGAA	CC1
SMC21.T.GATCGATCACAGACTT	CC1
SMC21.T.GATCGATGTCGTTGTA	CC2
SMC21.T.GATCGATGTGTGACCC	CC1
SMC21.T.GATCGCGAGACTAGGC	CC1
SMC21.T.GATCGCGAGGGCTTCC	CC1
SMC21.T.GATCGCGCACTACAGT	CC1
SMC21.T.GATCGCGCATGTAAGA	CC1
SMC21.T.GATCGCGTCTAGCACA	CC1
SMC21.T.GATCGTAAGAATTGTG	CC1
SMC21.T.GATCGTAGTAGGGTAC	CC1
SMC21.T.GATCGTAGTCTGCCAG	CC1
SMC21.T.GATCGTATCCTGCCAT	CC1

SMC21.T.GATCTAGAGAGTGACC	CC1
SMC21.T.GATCTAGAGATAGCAT	CC1
SMC21.T.GATCTAGAGCGTTCCG	CC1
SMC21.T.GATCTAGAGTCGATAA	CC1
SMC21.T.GATCTAGAGTGGTAAT	CC1
SMC21.T.GATCTAGTCAACGGGA	CC1
SMC21.T.GATGAAAAGAGCTATA	CC1
SMC21.T.GATGAAAAGAGTGAGA	CC1
SMC21.T.GATGAAACACCAACCG	CC1
SMC21.T.GATGAAAGTCAATGTC	CC1
SMC21.T.GATGAAAGTTCATGGT	CC1
SMC21.T.GATGAAAGTTTGTTGG	CC1
SMC21.T.GATGAAATCCCTCTTT	CC1
SMC21.T.GATGAAATCGTCCGTT	CC1
SMC21.T.GATGAGGAGACGCAAC	CC1
SMC21.T.GATGAGGAGGACAGCT	CC1
SMC21.T.GATGAGGTCGTCCGTT	CC1
SMC21.T.GATGCTAGTAAACACA	CC1
SMC21.T.GATGCTAGTGATAAAC	CC1
SMC21.T.GATGCTATCACGGTTA	CC1
SMC21.T.GATGCTATCATGTAGC	CC1
SMC21.T.GATGCTATCGTTACGA	CC1
SMC21.T.GATGCTATCTAGAGTC	CC1
SMC21.T.GATTCAGAGTGTCTCA	CC1
SMC21.T.GATTCAGGTAGGACAC	CC1
SMC21.T.GATTCAGGTTACAGAA	CC1
SMC21.T.GATTCAGTCACCCGAG	CC1
SMC21.T.GATTCAGTCATCATTC	CC1
SMC21.T.GATTCAGTCCTTTCTC	CC1
SMC21.T.GCAAACTAGGCAAAGA	CC1
SMC21.T.GCAAACTCATACGCCG	CC1
SMC21.T.GCAAACTGTTCCCGAG	CC1
SMC21.T.GCAAACTTCTCTGAGA	CC1
SMC21.T.GCAATCACACATTTCT	CC1
SMC21.T.GCAATCACAGCGTAAG	CC1
SMC21.T.GCAATCAGTAGAGTGC	CC1
SMC21.T.GCAATCAGTATAGTAG	CC1
SMC21.T.GCAATCAGTCCTGCTT	CC1
SMC21.T.GCAATCAGTTGGTGGA	CC1
SMC21.T.GCAATCATCAAAGACA	CC1
SMC21.T.GCAATCATCAGTTAGC	CC1
SMC21.T.GCAATCATCCTCAATT	CC1
SMC21.T.GCACATAGTAGTACCT	CC2
SMC21.T.GCACATAGTGCGCTTG	CC1
SMC21.T.GCACATATCTGAGTGT	CC1
SMC21.T.GCACTCTCAAGCCCAC	CC1
SMC21.T.GCACTCTGTCCAAGTT	CC1
SMC21.T.GCACTCTTCACCGTAA	CC1
SMC21.T.GCACTCTTCCTAGAAC	CC1
SMC21.T.GCAGCCAAGTCGCCGT	CC1
SMC21.T.GCAGCCAGTGTCTGAT	CC1
SMC21.T.GCAGCCATCTAGCACA	CC1
SMC21.T.GCAGTTAAGTACACCT	CC1
SMC21.T.GCAGTTAGTGCAACTT	CC1
SMC21.T.GCATACAAGCGTTCCG	CC1
SMC21.T.GCATGATCACAGATTC	CC1
SMC21.T.GCATGATCACCATGTA	CC1
SMC21.T.GCATGATGTCCGTCAG	CC1

SMC21.T.GCATGATTCTGCAGTA	CC1
SMC21.T.GCATGCGAGGCAATTA	CC1
SMC21.T.GCATGCGCAAATTGCC	CC1
SMC21.T.GCATGCGTCAGCGATT	CC1
SMC21.T.GCATGTAAGATATACG	CC1
SMC21.T.GCATGTACACCTTGTC	CC1
SMC21.T.GCATGTACAGGGTATG	CC1
SMC21.T.GCCAAATCATGTCGAT	CC1
SMC21.T.GCCAAATGTAACGTTC	CC1
SMC21.T.GCCAAATGTTATGTGC	CC1
SMC21.T.GCCTCTAAGAAAGTGG	CC1
SMC21.T.GCCTCTAAGCGTGAAC	CC1
SMC21.T.GCCTCTACAGTACACT	CC1
SMC21.T.GCCTCTATCAGCTCGG	CC1
SMC21.T.GCGACCACAATGGTCT	CC1
SMC21.T.GCGACCACAGTCAGAG	CC1
SMC21.T.GCGACCAGTATCGCAT	CC1
SMC21.T.GCGAGAAAGGACGAAA	CC1
SMC21.T.GCGAGAAGTTCAGTAC	CC1
SMC21.T.GCGCAACAGCTAAGAT	CC1
SMC21.T.GCGCAACAGTCGAGTG	CC1
SMC21.T.GCGCAACCACAGACAG	CC1
SMC21.T.GCGCAACCAGGCTGAA	CC1
SMC21.T.GCGCAACTCCATGAGT	CC1
SMC21.T.GCGCAGTAGCTGCGAA	CC1
SMC21.T.GCGCAGTCACATAACC	CC1
SMC21.T.GCGCAGTGTTCAACCA	CC1
SMC21.T.GCGCAGTTCCTCAATT	CC1
SMC21.T.GCGCCAAAGCTTTGGT	CC1
SMC21.T.GCGCCAATCACGACTA	CC1
SMC21.T.GCGCCAATCGGCGCAT	CC1
SMC21.T.GCGCGATAGCTGAACG	CC1
SMC21.T.GCGCGATAGGCATGTG	CC1
SMC21.T.GCGCGATTCAACGAAA	CC1
SMC21.T.GCGCGATTCTACTTAC	CC1
SMC21.T.GCGCGATTCTTCTGGC	CC1
SMC21.T.GCGGGTTCAAACGTGG	CC1
SMC21.T.GCGGGTTCAAGACACG	CC1
SMC21.T.GCGGGTTGTCGTCTTC	CC1
SMC21.T.GCGGGTTGTGTTTGTG	CC1
SMC21.T.GCGGGTTTCAGGTAAA	CC1
SMC21.T.GCGGGTTTCGAACGGA	CC1
SMC21.T.GCTCCTAAGACGCAAC	CC1
SMC21.T.GCTCCTACAAGTTAAG	CC1
SMC21.T.GCTCCTAGTTCGTTGA	CC1
SMC21.T.GCTCCTATCACGGTTA	CC1
SMC21.T.GCTCTGTAGCACGCCT	CC1
SMC21.T.GCTCTGTAGCCCAGCT	CC1
SMC21.T.GCTCTGTAGCGATCCC	CC1
SMC21.T.GCTCTGTAGGAGTTGC	CC1
SMC21.T.GCTCTGTAGGCGACAT	CC1
SMC21.T.GCTCTGTCAAGACACG	CC1
SMC21.T.GCTCTGTGTGCCTGGT	CC2
SMC21.T.GCTCTGTGTTGCCTCT	CC1
SMC21.T.GCTCTGTTCTGTGCAA	CC1
SMC21.T.GCTGCAGAGATGTCGG	CC1
SMC21.T.GCTGCAGAGCACCGTC	CC1
SMC21.T.GCTGCAGTCAACGCTA	CC1

SMC21.T.GCTGCAGTCAGATAAG	CC1
SMC21.T.GCTGCAGTCCGCATCT	CC1
SMC21.T.GCTGCAGTCTCATTCA	CC1
SMC21.T.GCTGCGAAGAAGATTC	CC1
SMC21.T.GCTGCGAGTACACCGC	CC1
SMC21.T.GCTGCGAGTTTGTTGG	CC1
SMC21.T.GCTGCGATCAAAGTAG	CC1
SMC21.T.GCTGCGATCGTCACGG	CC1
SMC21.T.GCTGCTTCAAAGCGGT	CC1
SMC21.T.GCTGGGTAGAGTACCG	CC1
SMC21.T.GCTGGGTCAGATAATG	CC1
SMC21.T.GCTGGGTGTCTAAACC	CC1
SMC21.T.GCTGGGTTCCCTTGCA	CC1
SMC21.T.GCTTCCAGTTCCATGA	CC1
SMC21.T.GCTTGAAGTTCCGTCT	CC1
SMC21.T.GGAAAGCAGCCCAGCT	CC1
SMC21.T.GGAAAGCAGTGGTAAT	CC1
SMC21.T.GGAAAGCGTATATCCG	CC1
SMC21.T.GGAACTTCACGTAAGG	CC1
SMC21.T.GGAATAAAGCTACCGC	CC1
SMC21.T.GGAATAAAGGCCATAG	CC1
SMC21.T.GGAATAATCCGCAAGC	CC1
SMC21.T.GGAATAATCTTCGAGA	CC1
SMC21.T.GGACAAGAGATGTTAG	CC2
SMC21.T.GGACAAGAGTACGATA	CC1
SMC21.T.GGACAAGAGTGTACCT	CC1
SMC21.T.GGACAGAAGTCAAGCG	CC1
SMC21.T.GGACAGACAAAGCAAT	CC1
SMC21.T.GGACAGACAAGTCATC	CC1
SMC21.T.GGACAGACATACAGCT	CC1
SMC21.T.GGACAGAGTTGGTGGA	CC1
SMC21.T.GGACAGATCAGCGACC	CC1
SMC21.T.GGACAGATCCTTAATC	CC2
SMC21.T.GGACAGATCTGCTGCT	CC1
SMC21.T.GGACATTAGCAGATCG	CC1
SMC21.T.GGACATTGTGTGTGCC	CC1
SMC21.T.GGACATTTCCGCGGTA	CC1
SMC21.T.GGACGTCCAAACCCAT	CC1
SMC21.T.GGACGTCCACGAAGCA	CC1
SMC21.T.GGACGTCCATGTAAGA	CC1
SMC21.T.GGACGTCTCTGCGACG	CC1
SMC21.T.GGATGTTGTCGAGTTT	CC1
SMC21.T.GGATGTTTCAGCTCGG	CC1
SMC21.T.GGATGTTTCGATAGAA	CC1
SMC21.T.GGATGTTTCGTCCAGG	CC1
SMC21.T.GGATTACAGAGCTATA	CC1
SMC21.T.GGATTACAGGCTAGAC	CC1
SMC21.T.GGATTACCACCAACCG	CC1
SMC21.T.GGATTACCACGAAACG	CC1
SMC21.T.GGATTACTCCAGTATG	CC1
SMC21.T.GGATTACTCGGTCTAA	CC1
SMC21.T.GGATTACTCTGCGACG	CC1
SMC21.T.GGCCGATAGAGCCCAA	CC1
SMC21.T.GGCCGATAGCGCCTCA	CC1
SMC21.T.GGCCGATAGTAATCCC	CC1
SMC21.T.GGCCGATGTCTAGTGT	CC1
SMC21.T.GGCCGATGTCTGCCAG	CC1
SMC21.T.GGCGACTAGAAACCGC	CC1

SMC21.T.GGCGACTCAATCTGCA	CC1
SMC21.T.GGCGACTCACCAGGCT	CC1
SMC21.T.GGCGACTCAGTAAGCG	CC1
SMC21.T.GGCGTGTAGTTAGGTA	CC1
SMC21.T.GGCGTGTGTTGTTTGG	CC1
SMC21.T.GGCTCGAAGGCCCGTT	CC1
SMC21.T.GGCTGGTAGATATACG	CC1
SMC21.T.GGCTGGTAGTACGATA	CC1
SMC21.T.GGCTGGTGTCGCATAT	CC1
SMC21.T.GGGAATGAGCGGATCA	CC1
SMC21.T.GGGACCTCACGTCTCT	CC1
SMC21.T.GGGACCTGTTCCACAA	CC1
SMC21.T.GGGACCTTCAGGATCT	CC1
SMC21.T.GGGAGATAGGGAAACA	CC1
SMC21.T.GGGAGATCAGGCAGTA	CC1
SMC21.T.GGGAGATGTCTCATCC	CC1
SMC21.T.GGGATGAAGAGTACAT	CC1
SMC21.T.GGGATGACAAACCCAT	CC1
SMC21.T.GGGATGACACAGACTT	CC1
SMC21.T.GGGATGAGTCTAGCGC	CC1
SMC21.T.GGGATGAGTGTAATGA	CC1
SMC21.T.GGGCACTCATCGGAAG	CC2
SMC21.T.GGGCACTGTACCGGCT	CC1
SMC21.T.GGGCATCGTAAACCTC	CC1
SMC21.T.GGGCATCTCCTCATTA	CC1
SMC21.T.GGGTCTGAGCGATCCC	CC1
SMC21.T.GGGTCTGAGCTGGAAC	CC1
SMC21.T.GGGTCTGTCTGACCTC	CC1
SMC21.T.GGGTTGCAGGACTGGT	CC1
SMC21.T.GGGTTGCAGGCGATAC	CC1
SMC21.T.GGTATTGCATCCTTGC	CC1
SMC21.T.GGTATTGCATTGGTAC	CC1
SMC21.T.GGTATTGGTGCGGTAA	CC1
SMC21.T.GGTATTGTCAGCCTAA	CC1
SMC21.T.GGTGAAGAGACAATAC	CC1
SMC21.T.GGTGAAGCACCGCTAG	CC1
SMC21.T.GGTGAAGCATATGCTG	CC1
SMC21.T.GGTGAAGGTCGCGTGT	CC1
SMC21.T.GGTGAAGTCATGTAGC	CC1
SMC21.T.GGTGAAGTCGGTGTTA	CC1
SMC21.T.GGTGCGTCACAACTGT	CC1
SMC21.T.GGTGCGTGTACTCTCC	CC1
SMC21.T.GGTGCGTGTAGAAGGA	CC1
SMC21.T.GGTGCGTTCCTAAGTG	CC1
SMC21.T.GGTGTTAAGCTAACTC	CC1
SMC21.T.GGTGTTAAGTAGATGT	CC1
SMC21.T.GGTGTTACATCCTAGA	CC1
SMC21.T.GGTGTTAGTGATAAGT	CC1
SMC21.T.GTAACGTAGGACGAAA	CC1
SMC21.T.GTAACGTCAGTCCTTC	CC1
SMC21.T.GTAACGTTCGGTGTTA	CC1
SMC21.T.GTAACTGAGTGGGTTG	CC1
SMC21.T.GTAACTGCATAGAAAC	CC1
SMC21.T.GTAACTGGTCAAACTC	CC1
SMC21.T.GTAACTGGTCCTCTTG	CC1
SMC21.T.GTACGTATCTACTATC	CC1
SMC21.T.GTACTCCAGTACGACG	CC1
SMC21.T.GTACTCCGTAAGGGCT	CC1

SMC21.T.GTACTCCGTGAGTATA	CC1
SMC21.T.GTACTTTGTACCATCA	CC1
SMC21.T.GTAGGCCAGGGTCTCC	CC1
SMC21.T.GTAGGCCCACAACGTT	CC1
SMC21.T.GTAGGCCGTTCAGTAC	CC1
SMC21.T.GTAGGCCTCTTTCCTC	CC1
SMC21.T.GTAGTCAAGCACCGCT	CC1
SMC21.T.GTAGTCACAAAGTCAA	CC1
SMC21.T.GTAGTCACATCGGTTA	CC1
SMC21.T.GTAGTCAGTCGGCATC	CC1
SMC21.T.GTAGTCATCAGAGGTG	CC1
SMC21.T.GTAGTCATCAGGTTCA	CC1
SMC21.T.GTATCTTAGTTAGGTA	CC1
SMC21.T.GTATCTTCAGAGTGTG	CC1
SMC21.T.GTATCTTTCCACTGGG	CC1
SMC21.T.GTATCTTTCGACCAGC	CC1
SMC21.T.GTATCTTTCTTGTACT	CC1
SMC21.T.GTATTCTAGTGTACGG	CC1
SMC21.T.GTATTCTCACGAAACG	CC1
SMC21.T.GTATTCTGTAGCACGA	CC1
SMC21.T.GTATTCTGTCGCCATG	CC1
SMC21.T.GTATTCTTCGCAAACT	CC1
SMC21.T.GTATTCTTCGGAATCT	CC1
SMC21.T.GTCAAGTGTCTCCATC	CC1
SMC21.T.GTCAAGTGTTTGTGTG	CC1
SMC21.T.GTCAAGTTCCAACCAA	CC1
SMC21.T.GTCACAAAGTGTTGAA	CC1
SMC21.T.GTCACAACAATCACAC	CC1
SMC21.T.GTCACAAGTCCAACTA	CC1
SMC21.T.GTCACGGCAATGACCT	CC1
SMC21.T.GTCACGGCACACTGCG	CC1
SMC21.T.GTCACGGGTAGAGGAA	CC1
SMC21.T.GTCACGGGTCCTCTTG	CC1
SMC21.T.GTCACGGGTCTGATTG	CC2
SMC21.T.GTCACGGGTGACTACT	CC1
SMC21.T.GTCACGGGTGTTTGGT	CC1
SMC21.T.GTCACGGTCTTTAGGG	CC2
SMC21.T.GTCATTTAGTGGGTTG	CC1
SMC21.T.GTCATTTCACCACCAG	CC1
SMC21.T.GTCATTTTCAACACGT	CC1
SMC21.T.GTCATTTTCGCGTAGC	CC1
SMC21.T.GTCCTCAAGATGTCGG	CC1
SMC21.T.GTCCTCACACCAACCG	CC1
SMC21.T.GTCCTCACATGCTGGC	CC1
SMC21.T.GTCCTCAGTTCGTGAT	CC1
SMC21.T.GTCCTCATCATCGCTC	CC2
SMC21.T.GTCCTCATCCGGCACA	CC1
SMC21.T.GTCCTCATCTCTAGGA	CC1
SMC21.T.GTCCTCATCTGCAAGT	CC1
SMC21.T.GTCGGGTGTGTAAGTA	CC1
SMC21.T.GTCGGGTTCAGCTGGC	CC1
SMC21.T.GTCGGGTTCGCCGTGA	CC1
SMC21.T.GTCGTAATCGCCATAA	CC1
SMC21.T.GTCTCGTAGCTGCGAA	CC1
SMC21.T.GTCTCGTAGGCGACAT	CC1
SMC21.T.GTCTCGTAGTGTACGG	CC1
SMC21.T.GTCTCGTGTCAAGCGA	CC1
SMC21.T.GTCTCGTGTCGAATCT	CC1

SMC21.T.GTCTCGTTCCAAGTAC	CC1
SMC21.T.GTCTCGTTCGGATGGA	CC1
SMC21.T.GTCTTCGAGGCTCAGA	CC1
SMC21.T.GTCTTCGCATGACGGA	CC1
SMC21.T.GTCTTCGGTATGCTTG	CC1
SMC21.T.GTCTTCGTCAACACTG	CC1
SMC21.T.GTCTTCGTCGCCATAA	CC1
SMC21.T.GTGAAGGAGAATTGTG	CC1
SMC21.T.GTGAAGGAGAGCCTAG	CC1
SMC21.T.GTGAAGGGTGCCTGCA	CC1
SMC21.T.GTGAAGGGTGTCTGAT	CC1
SMC21.T.GTGCAGCCATTCCTGC	CC1
SMC21.T.GTGCATACATTGGTAC	CC1
SMC21.T.GTGCATAGTACCATCA	CC1
SMC21.T.GTGCGGTAGTATGACA	CC1
SMC21.T.GTGCGGTCAGCGTTCG	CC1
SMC21.T.GTGCGGTTCTACTCAT	CC1
SMC21.T.GTGCTTCAGCCCAACC	CC1
SMC21.T.GTGCTTCAGCGTTTAC	CC1
SMC21.T.GTGCTTCCATTCTTAC	CC1
SMC21.T.GTGCTTCTCACTATTC	CC1
SMC21.T.GTGTGCGAGGGCATGT	CC1
SMC21.T.GTGTGCGAGTAGATGT	CC1
SMC21.T.GTGTGCGTCAAGATCC	CC1
SMC21.T.GTGTGCGTCTGTTTGT	CC1
SMC21.T.GTGTTAGGTCAGTGGA	CC1
SMC21.T.GTTAAGCCAGGACCCT	CC1
SMC21.T.GTTAAGCGTTCGAATC	CC1
SMC21.T.GTTAAGCTCTTTACGT	CC1
SMC21.T.GTTACAGAGAAGGACA	CC1
SMC21.T.GTTACAGGTTCCGGCA	CC1
SMC21.T.GTTACAGGTTCTCATT	CC1
SMC21.T.GTTCATTCAGCCAGAA	CC1
SMC21.T.GTTCATTGTATGAAAC	CC1
SMC21.T.GTTCATTTCAGCGACC	CC1
SMC21.T.GTTCATTTCCACGTTC	CC1
SMC21.T.GTTCGGGAGAGCTATA	CC1
SMC21.T.GTTCGGGAGGTCATCT	CC1
SMC21.T.GTTCGGGGTAGGGTAC	CC1
SMC21.T.GTTCGGGGTCAAAGCG	CC1
SMC21.T.GTTTCTAAGACCACGA	CC1
SMC21.T.GTTTCTAAGCTCCTTC	CC1
SMC21.T.GTTTCTAAGTACGTAA	CC1
SMC21.T.GTTTCTACAAGGGTCA	CC1
SMC21.T.GTTTCTATCGGCTTGG	CC1
SMC21.T.TAAACCGCAACACCTA	CC1
SMC21.T.TAAACCGGTCAGAGGT	CC1
SMC21.T.TAAACCGGTGATGTGG	CC1
SMC21.T.TAAACCGTCCTTGCCA	CC1
SMC21.T.TAAGAGACAAGGTTTC	CC1
SMC21.T.TAAGAGAGTGTAACGG	CC1
SMC21.T.TAAGTGCAGATATACG	CC1
SMC21.T.TAAGTGCAGGCAAAGA	CC1
SMC21.T.TAAGTGCGTCTAGTCA	CC1
SMC21.T.TAAGTGCGTGCGGTAA	CC1
SMC21.T.TACACGACAACTGCTA	CC1
SMC21.T.TACACGACACCCATGG	CC1
SMC21.T.TACACGAGTGGGTATG	CC1

SMC21.T.TACACGAGTTACGTCA	CC1
SMC21.T.TACACGATCCGAACGC	CC1
SMC21.T.TACACGATCTGTTTGT	CC1
SMC21.T.TACAGTGGTATAATGG	CC1
SMC21.T.TACAGTGGTCGCTTCT	CC1
SMC21.T.TACAGTGGTGTATGGG	CC1
SMC21.T.TACCTATAGCTTTGGT	CC1
SMC21.T.TACCTATCACCTATCC	CC1
SMC21.T.TACCTATGTGTTCGAT	CC1
SMC21.T.TACCTTAAGGAATCGC	CC1
SMC21.T.TACCTTAGTGAGTATA	CC1
SMC21.T.TACGGATAGACCTAGG	CC1
SMC21.T.TACGGATCATGTCTCC	CC1
SMC21.T.TACGGGCAGAAGGCCT	CC1
SMC21.T.TACGGGCAGTGTACGG	CC1
SMC21.T.TACGGGCCATACGCTA	CC1
SMC21.T.TACGGGCTCTGCCCTA	CC1
SMC21.T.TACGGTATCCAAGTAC	CC1
SMC21.T.TACTCATCACGGCGTT	CC1
SMC21.T.TACTCATGTAAGTTCC	CC1
SMC21.T.TACTCATGTACCTACA	CC1
SMC21.T.TACTCATGTGACGCCT	CC1
SMC21.T.TACTCGCAGTGTTAGA	CC1
SMC21.T.TACTCGCCAAGCGAGT	CC1
SMC21.T.TACTTACAGAAACCTA	CC1
SMC21.T.TACTTACGTCTCCCTA	CC1
SMC21.T.TACTTACGTTCGTGAT	CC1
SMC21.T.TACTTACTCATACGGT	CC1
SMC21.T.TACTTGTAGGCGCTCT	CC1
SMC21.T.TACTTGTCAGGCAGTA	CC1
SMC21.T.TACTTGTCAGGTCGTC	CC1
SMC21.T.TACTTGTTCCTAGTGA	CC1
SMC21.T.TACTTGTTCTTCTGGC	CC1
SMC21.T.TAGACCACAGACGTAG	CC1
SMC21.T.TAGACCACATCCGGGT	CC1
SMC21.T.TAGACCACATTACGAC	CC1
SMC21.T.TAGACCATCCGTTGTC	CC1
SMC21.T.TAGAGCTCAAACAACA	CC1
SMC21.T.TAGAGCTCAGCTATTG	CC1
SMC21.T.TAGAGCTGTCGAAAGC	CC1
SMC21.T.TAGCCGGAGACCCACC	CC1
SMC21.T.TAGCCGGTCAACACCA	CC1
SMC21.T.TAGCCGGTCTTCGAGA	CC1
SMC21.T.TAGGCATAGCGATGAC	CC1
SMC21.T.TAGGCATAGTGGTAGC	CC1
SMC21.T.TAGGCATGTTAGTGGG	CC1
SMC21.T.TAGGCATTCAGCATGT	CC1
SMC21.T.TAGGCATTCGGAATCT	CC1
SMC21.T.TAGGCATTCGGACAAG	CC1
SMC21.T.TAGGCATTCGGTGTCG	CC1
SMC21.T.TAGTGGTAGGTAAACT	CC1
SMC21.T.TAGTGGTCAAATCCGT	CC1
SMC21.T.TATCAGGAGGTGATTA	CC1
SMC21.T.TATCAGGCACGCTTTC	CC1
SMC21.T.TATCAGGGTCGAACAG	CC1
SMC21.T.TATCAGGTCAATAAGG	CC1
SMC21.T.TATCAGGTCGCATGGC	CC1
SMC21.T.TATGCCCCAGCGTCCA	CC1

SMC21.T.TATTACCCAGCATGAG	CC1
SMC21.T.TATTACCTCTGGCGAC	CC1
SMC21.T.TCAACGACAATACGCT	CC1
SMC21.T.TCAACGAGTACCGAGA	CC1
SMC21.T.TCAATCTAGTGAAGTT	CC1
SMC21.T.TCAATCTTCCAATGGT	CC1
SMC21.T.TCACAAGGTCTGCCAG	CC1
SMC21.T.TCACAAGGTCTTTCAT	CC1
SMC21.T.TCACGAACAGGACCCT	CC1
SMC21.T.TCACGAAGTACCGTAT	CC1
SMC21.T.TCACGAAGTGCAGACA	CC1
SMC21.T.TCACGAATCGATCCCT	CC1
SMC21.T.TCACGAATCGTTTATC	CC1
SMC21.T.TCAGATGCAAACAACA	CC1
SMC21.T.TCAGATGCAAAGGTGC	CC1
SMC21.T.TCAGATGCAGCCTTTC	CC1
SMC21.T.TCAGATGGTAAACCTC	CC1
SMC21.T.TCAGCAATCGCGATCG	CC1
SMC21.T.TCAGCTCAGTCATCCA	CC1
SMC21.T.TCAGCTCCACAGGAGT	CC1
SMC21.T.TCAGCTCGTCATCGGC	CC1
SMC21.T.TCAGCTCTCACCAGGC	CC1
SMC21.T.TCAGGATTCCAGAGGA	CC2
SMC21.T.TCAGGATTCCTACAGA	CC1
SMC21.T.TCAGGTAGTAGTACCT	CC1
SMC21.T.TCATTACGTGACAAAT	CC1
SMC21.T.TCATTACGTGCGAAAC	CC1
SMC21.T.TCATTTGCAGTACACT	CC1
SMC21.T.TCATTTGGTAGCACGA	CC1
SMC21.T.TCATTTGGTGTCAATC	CC1
SMC21.T.TCCACACAGTGAAGAG	CC1
SMC21.T.TCCCGATAGACAGGCT	CC1
SMC21.T.TCCCGATCACCGATAT	CC1
SMC21.T.TCCCGATCATACTCTT	CC1
SMC21.T.TCCCGATGTGAGGGAG	CC1
SMC21.T.TCCCGATGTGCACGAA	CC1
SMC21.T.TCGAGGCCACATCTTT	CC1
SMC21.T.TCGAGGCGTGACTCAT	CC1
SMC21.T.TCGAGGCGTGTCAATC	CC1
SMC21.T.TCGAGGCTCAACCAAC	CC2
SMC21.T.TCGAGGCTCCCTCAGT	CC1
SMC21.T.TCGAGGCTCGGTGTCG	CC1
SMC21.T.TCGCGAGAGAATAGGG	CC1
SMC21.T.TCGCGAGTCCGGCACA	CC1
SMC21.T.TCGCGAGTCGGAAACG	CC1
SMC21.T.TCGCGAGTCGGATGTT	CC1
SMC21.T.TCGCGTTAGACACTAA	CC1
SMC21.T.TCGCGTTGTCCGCTGA	CC1
SMC21.T.TCGGTAAAGCAACGGT	CC1
SMC21.T.TCGGTAACAATAGCGG	CC1
SMC21.T.TCGGTAAGTGATAAAC	CC1
SMC21.T.TCGGTAAGTTCCATGA	CC1
SMC21.T.TCGTACCCAGATCTGT	CC1
SMC21.T.TCGTACCGTAAGTTCC	CC1
SMC21.T.TCGTACCGTGTTCGAT	CC1
SMC21.T.TCGTACCTCCAGGGCT	CC1
SMC21.T.TCGTACCTCGCTAGCG	CC1
SMC21.T.TCGTAGACACCGTTGG	CC1

SMC21.T.TCGTAGAGTGTATGGG	CC1
SMC21.T.TCTATTGAGACTAAGT	CC1
SMC21.T.TCTATTGGTACCATCA	CC1
SMC21.T.TCTATTGGTAGAAAGG	CC1
SMC21.T.TCTCATATCCCAAGAT	CC1
SMC21.T.TCTCTAAAGTGAACGC	CC1
SMC21.T.TCTCTAAGTACCCAAT	CC1
SMC21.T.TCTGAGAAGTGACATA	CC1
SMC21.T.TCTGAGACACCATGTA	CC1
SMC21.T.TCTGAGACACCTTGTC	CC1
SMC21.T.TCTGAGACATAGACTC	CC1
SMC21.T.TCTGGAAGTATTCGTG	CC1
SMC21.T.TCTGGAATCAACACTG	CC1
SMC21.T.TCTTCGGCAAGGTTCT	CC1
SMC21.T.TCTTCGGGTTCGTTGA	CC1
SMC21.T.TCTTCGGTCAACGCTA	CC1
SMC21.T.TCTTTCCAGCCTATGT	CC1
SMC21.T.TCTTTCCAGTGCGATG	CC1
SMC21.T.TCTTTCCCACACTGCG	CC1
SMC21.T.TGAAAGAAGCAACGGT	CC1
SMC21.T.TGAAAGAGTCGCGGTT	CC1
SMC21.T.TGAAAGAGTGCAGACA	CC1
SMC21.T.TGAAAGATCACTGGGC	CC1
SMC21.T.TGAAAGATCTTCAACT	CC1
SMC21.T.TGACAACCATTGGTAC	CC1
SMC21.T.TGACAACTCGAGCCCA	CC1
SMC21.T.TGACGGCAGATGTTAG	CC1
SMC21.T.TGACGGCAGCACAGGT	CC1
SMC21.T.TGACGGCTCTACTCAT	CC1
SMC21.T.TGACTAGCACAGGTTT	CC1
SMC21.T.TGACTAGGTCTTCAAG	CC1
SMC21.T.TGACTAGTCATTTGGG	CC1
SMC21.T.TGACTTTCACGGCCAT	CC1
SMC21.T.TGACTTTGTAGGGACT	CC1
SMC21.T.TGACTTTGTGCCTGGT	CC1
SMC21.T.TGACTTTGTTGCGCAC	CC1
SMC21.T.TGACTTTTCAAGGTAA	CC1
SMC21.T.TGACTTTTCGTGGGAA	CC1
SMC21.T.TGACTTTTCTGACCTC	CC1
SMC21.T.TGAGAGGGTCGGGTCT	CC1
SMC21.T.TGAGAGGGTGACAAAT	CC1
SMC21.T.TGAGAGGTCACATGCA	CC1
SMC21.T.TGAGCATAGCGTTTAC	CC1
SMC21.T.TGAGCATAGGCATTGG	CC1
SMC21.T.TGAGCATAGTGTGAAT	CC1
SMC21.T.TGAGCATGTTACGTCA	CC1
SMC21.T.TGAGCATTCAGCCTAA	CC1
SMC21.T.TGAGCCGAGAGTAATC	CC1
SMC21.T.TGAGCCGAGATGTTAG	CC1
SMC21.T.TGAGCCGCAAAGTGCG	CC1
SMC21.T.TGAGCCGCAATGAAAC	CC1
SMC21.T.TGAGCCGCACCCAGTG	CC1
SMC21.T.TGAGCCGCAGCCTTGG	CC1
SMC21.T.TGAGCCGTCCTCGCAT	CC1
SMC21.T.TGAGGGAAGACTGTAA	CC1
SMC21.T.TGAGGGAAGCAATATG	CC1
SMC21.T.TGAGGGAGTATATCCG	CC1
SMC21.T.TGATTTCCAGTAAGCG	CC1

SMC21.T.TGATTTCGTAAATGTG	CC1
SMC21.T.TGATTTCTCAGAGACG	CC1
SMC21.T.TGATTTCTCTCACATT	CC1
SMC21.T.TGATTTCTCTTGTTTG	CC1
SMC21.T.TGCACCTAGAACTGTA	CC1
SMC21.T.TGCACCTCACAGCGTC	CC1
SMC21.T.TGCACCTGTACCGTTA	CC1
SMC21.T.TGCACCTGTCGACTAT	CC1
SMC21.T.TGCCAAATCTCGTTTA	CC1
SMC21.T.TGCCCATAGACTAGAT	CC1
SMC21.T.TGCCCATAGTACACCT	CC1
SMC21.T.TGCCCATGTCTAGTCA	CC1
SMC21.T.TGCCCATTCTCACATT	CC1
SMC21.T.TGCCCTACAATACGCT	CC1
SMC21.T.TGCCCTACAATTGCTG	CC1
SMC21.T.TGCCCTACACTGTTAG	CC1
SMC21.T.TGCCCTATCAGCATGT	CC1
SMC21.T.TGCCCTATCCTATGTT	CC1
SMC21.T.TGCGCAGAGGCAATTA	CC1
SMC21.T.TGCGCAGCATCACGAT	CC1
SMC21.T.TGCGGGTTCTCTGAGA	CC1
SMC21.T.TGCGTGGAGAAGGACA	CC1
SMC21.T.TGCGTGGAGCCAGAAC	CC1
SMC21.T.TGCGTGGCAAAGGTGC	CC2
SMC21.T.TGCGTGGCACAGCGTC	CC1
SMC21.T.TGCGTGGGTAGCACGA	CC1
SMC21.T.TGCGTGGTCAACACCA	CC1
SMC21.T.TGCGTGGTCCGGGTGT	CC1
SMC21.T.TGCTACCCAGCTGTTA	CC1
SMC21.T.TGCTACCTCATGTCCC	CC1
SMC21.T.TGCTGCTCACAACGCC	CC1
SMC21.T.TGCTGCTCACTGAAGG	CC1
SMC21.T.TGCTGCTCAGGTTTCA	CC1
SMC21.T.TGCTGCTGTTCGTTGA	CC1
SMC21.T.TGCTGCTTCCTCCTAG	CC1
SMC21.T.TGGACGCAGCGATATA	CC1
SMC21.T.TGGACGCGTATAGTAG	CC1
SMC21.T.TGGACGCGTGCAGTAG	CC1
SMC21.T.TGGACGCGTGTATGGG	CC1
SMC21.T.TGGACGCTCAGCTCTC	CC1
SMC21.T.TGGCCAGAGCAACGGT	CC1
SMC21.T.TGGCCAGAGTGACATA	CC1
SMC21.T.TGGCCAGCAGGACCCT	CC1
SMC21.T.TGGCGCAAGACCTTTG	CC1
SMC21.T.TGGCGCAAGCCACTAT	CC1
SMC21.T.TGGCGCATCTTGCCGT	CC1
SMC21.T.TGGCTGGAGGATCGCA	CC1
SMC21.T.TGGCTGGCATCTCCCA	CC1
SMC21.T.TGGCTGGTCCAGGGCT	CC1
SMC21.T.TGGGAAGAGTTCGATC	CC1
SMC21.T.TGGGAAGCAAGAAGAG	CC1
SMC21.T.TGGGAAGTCACGACTA	CC1
SMC21.T.TGGGCGTAGTGTCCCG	CC1
SMC21.T.TGGGCGTGTCTGGTCG	CC1
SMC21.T.TGGGCGTGTTCGAATC	CC1
SMC21.T.TGGGCGTTCACTCTTA	CC1
SMC21.T.TGGGCGTTCAGGCCCA	CC1
SMC21.T.TGGTTAGAGGGATACC	CC2

SMC21.T.TGGTTAGCAATTGCTG	CC1
SMC21.T.TGGTTAGCACACGCTG	CC1
SMC21.T.TGGTTAGGTACAGCAG	CC1
SMC21.T.TGGTTCCAGAGCCCAA	CC1
SMC21.T.TGGTTCCAGATAGGAG	CC1
SMC21.T.TGGTTCCCAAGGACAC	CC2
SMC21.T.TGGTTCCCAAGTAATG	CC1
SMC21.T.TGGTTCCCACCCATTC	CC1
SMC21.T.TGGTTCCGTATGAATG	CC1
SMC21.T.TGGTTCCGTCTAGAGG	CC1
SMC21.T.TGGTTCCGTGAGGGTT	CC1
SMC21.T.TGTATTCCATTCGACA	CC1
SMC21.T.TGTATTCTCGAACTGT	CC1
SMC21.T.TGTCCCACAATGGTCT	CC1
SMC21.T.TGTCCCACAGGGATTG	CC1
SMC21.T.TGTCCCAGTCATGCAT	CC1
SMC21.T.TGTCCCATCGATCCCT	CC1
SMC21.T.TGTGGTAAGCGTTTAC	CC1
SMC21.T.TGTGGTAAGCTAGTCT	CC1
SMC21.T.TGTGGTAAGGCTAGGT	CC1
SMC21.T.TGTGGTATCCTGCCAT	CC1
SMC21.T.TGTGTTTTCCTTGACC	CC1
SMC21.T.TGTGTTTTCGGTCCGA	CC1
SMC21.T.TGTGTTTTCTCCGGTT	CC1
SMC21.T.TGTTCCGAGCCCAATT	CC1
SMC21.T.TGTTCCGAGCTGCCCA	CC1
SMC21.T.TGTTCCGCAAATTGCC	CC1
SMC21.T.TGTTCCGGTTGGGACA	CC1
SMC21.T.TGTTCCGTCCGTAGTA	CC1
SMC21.T.TGTTCCGTCGCATGGC	CC1
SMC21.T.TGTTCCGTCGGAGGTA	CC1
SMC21.T.TGTTCCGTCTACTCAT	CC1
SMC21.T.TTAACTCAGCTATGCT	CC1
SMC21.T.TTAACTCCACTTAACG	CC1
SMC21.T.TTAACTCCATCCCATC	CC1
SMC21.T.TTAACTCGTCACCCAG	CC1
SMC21.T.TTAACTCTCAGAGGTG	CC1
SMC21.T.TTAACTCTCCTCAATT	CC1
SMC21.T.TTAGGCAAGGACGAAA	CC1
SMC21.T.TTAGGCACAATCCAAC	CC2
SMC21.T.TTAGGCAGTAGCGTGA	CC1
SMC21.T.TTAGGCATCCATTCTA	CC1
SMC21.T.TTAGTTCTCTCAAGTG	CC1
SMC21.T.TTAGTTCTCTGTCAAG	CC1
SMC21.T.TTAGTTCTCTGTCTAT	CC1
SMC21.T.TTATGCTAGGCTACGA	CC1
SMC21.T.TTATGCTCAGCTATTG	CC2
SMC21.T.TTATGCTTCCGCGCAA	CC1
SMC21.T.TTCCCAGAGAAGAAGC	CC1
SMC21.T.TTCCCAGTCGCGTAGC	CC1
SMC21.T.TTCGAAGCATCGGAAG	CC1
SMC21.T.TTCGAAGGTTTGCATG	CC1
SMC21.T.TTCGGTCCACATGTGT	CC1
SMC21.T.TTCGGTCCATCCTAGA	CC1
SMC21.T.TTCGGTCGTCCCTACT	CC1
SMC21.T.TTCGGTCTCACATAGC	CC1
SMC21.T.TTCGGTCTCATACGGT	CC1
SMC21.T.TTCGGTCTCTTCAACT	CC1

SMC21.T.TTCTACAGTCATTAGC	CC1
SMC21.T.TTCTACATCTGTCTCG	CC1
SMC21.T.TTCTCAAAGACCGGAT	CC1
SMC21.T.TTCTCAAAGGGTGTTG	CC1
SMC21.T.TTCTCAACAGCGTCCA	CC1
SMC21.T.TTCTCAAGTCGAACAG	CC1
SMC21.T.TTCTCAATCGCGATCG	CC1
SMC21.T.TTCTCCTAGACTTGAA	CC1
SMC21.T.TTCTCCTGTCTCTTAT	CC1
SMC21.T.TTCTCCTTCTGCTGTC	CC1
SMC21.T.TTCTTAGAGCGTAATA	CC1
SMC21.T.TTGAACGGTGATGTCT	CC1
SMC21.T.TTGACTTAGACAGACC	CC1
SMC21.T.TTGACTTAGCGATTCT	CC1
SMC21.T.TTGACTTAGTGGAGTC	CC1
SMC21.T.TTGACTTCAGGCAGTA	CC1
SMC21.T.TTGACTTGTAATAGCA	CC1
SMC21.T.TTGACTTTCATTATCC	CC1
SMC21.T.TTGCCGTAGAAGCCCA	CC1
SMC21.T.TTGCGTCAGCCTTGAT	CC1
SMC21.T.TTGCGTCGTTTGACTG	CC1
SMC21.T.TTGCGTCTCCCTCAGT	CC1
SMC21.T.TTGCGTCTCGTGGACC	CC1
SMC21.T.TTGGAACCACAAGCCC	CC1
SMC21.T.TTGGAACGTACATCCA	CC1
SMC21.T.TTGTAGGAGCGTGAAC	CC1
SMC21.T.TTGTAGGCAATTCCTT	CC1
SMC21.T.TTGTAGGGTCAGAATA	CC1
SMC21.T.TTTACTGGTAGGAGTC	CC1
SMC21.T.TTTACTGGTCGGCACT	CC1
SMC21.T.TTTATGCAGAGTCGGT	CC1
SMC21.T.TTTATGCAGCAATATG	CC1
SMC21.T.TTTATGCAGCCGGTAA	CC1
SMC21.T.TTTATGCCATATGCTG	CC1
SMC21.T.TTTCCTCCATATGAGA	CC1
SMC21.T.TTTGCGCAGGCAATTA	CC1
SMC21.T.TTTGCGCCAGCAGTTT	CC1
SMC21.T.TTTGCGCCATAGACTC	CC1
SMC21.T.TTTGCGCTCTTATCTG	CC1
SMC21.T.TTTGGTTCAAACCCAT	CC1
SMC21.T.TTTGGTTCACGTCAGC	CC1
SMC21.T.TTTGGTTTCTCGTTTA	CC1
SMC21.T.TTTGTCAAGCAATATG	CC1
SMC21.T.TTTGTCAAGCACCGCT	CC1
SMC21.T.TTTGTCAAGTACATGA	CC1
SMC22.T.AAACGGGAGAAACCTA	CC1
SMC22.T.AAAGATGCAATACGCT	CC1
SMC22.T.AAAGATGGTAGTACCT	CC1
SMC22.T.AAAGATGGTGATGATA	CC1
SMC22.T.AAAGATGGTTCAGTAC	CC1
SMC22.T.AAAGATGTCTACTCAT	CC1
SMC22.T.AAAGCAAAGTGTACCT	CC2
SMC22.T.AAAGCAACATTACGAC	CC2
SMC22.T.AAAGCAAGTTCTGAAC	CC1
SMC22.T.AAATGCCAGACAAGCC	CC1
SMC22.T.AAATGCCGTAAGTGGC	CC2
SMC22.T.AACACGTAGTCGAGTG	CC1
SMC22.T.AACACGTCATGCCTAA	CC1

SMC22.T.AACACGTGTCTAACGT	CC1
SMC22.T.AACACGTGTGCGATAG	CC1
SMC22.T.AACACGTTCAAGCCTA	CC1
SMC22.T.AACACGTTCAGTTCGA	CC1
SMC22.T.AACACGTTCCACGACG	CC2
SMC22.T.AACCATGCACATGACT	CC1
SMC22.T.AACCATGGTATTCGTG	CC1
SMC22.T.AACCATGTCATTCACT	CC1
SMC22.T.AACCGCGCAATAGCGG	CC1
SMC22.T.AACCGCGCATCGATGT	CC1
SMC22.T.AACCGCGTCAATCACG	CC1
SMC22.T.AACGTTGAGATGTAAC	CC1
SMC22.T.AACGTTGAGCAATCTC	CC1
SMC22.T.AACGTTGTCGGGAGTA	CC1
SMC22.T.AACTCAGCAGCGATCC	CC1
SMC22.T.AACTCAGTCTTTACGT	CC1
SMC22.T.AACTCCCAGACGCACA	CC1
SMC22.T.AACTCCCAGCAACGGT	CC1
SMC22.T.AACTCCCCACACCGAC	CC2
SMC22.T.AACTCCCCAGTGAGTG	CC1
SMC22.T.AACTCCCGTTACGACT	CC1
SMC22.T.AACTCCCTCGGACAAG	CC1
SMC22.T.AACTCTTCATCCAACA	CC1
SMC22.T.AACTCTTGTCACCTAA	CC1
SMC22.T.AACTCTTTCAGAAATG	CC1
SMC22.T.AACTCTTTCTATCCCG	CC1
SMC22.T.AACTGGTCAATTCCTT	CC1
SMC22.T.AACTGGTGTCGCGAAA	CC1
SMC22.T.AACTGGTTCAAAGTAG	CC1
SMC22.T.AACTTTCAGGACATTA	CC1
SMC22.T.AACTTTCAGTAGGCCA	CC1
SMC22.T.AACTTTCGTAAATGTG	CC1
SMC22.T.AACTTTCTCCTCAACC	CC2
SMC22.T.AAGACCTTCTATCCTA	CC1
SMC22.T.AAGCCGCAGCCAGTTT	CC1
SMC22.T.AAGCCGCCAGGATTGG	CC1
SMC22.T.AAGCCGCCATCCTAGA	CC2
SMC22.T.AAGCCGCGTAGCACGA	CC1
SMC22.T.AAGCCGCGTCCGCTGA	CC2
SMC22.T.AAGCCGCTCGTGGGAA	CC1
SMC22.T.AAGCCGCTCTACTTAC	CC1
SMC22.T.AAGGAGCCACCGAAAG	CC1
SMC22.T.AAGGAGCCATACTCTT	CC2
SMC22.T.AAGGAGCCATCTCGCT	CC1
SMC22.T.AAGGAGCGTTCGCTAA	CC1
SMC22.T.AAGGAGCTCTTCCTTC	CC1
SMC22.T.AAGGCAGAGAGTAAGG	CC2
SMC22.T.AAGGCAGAGGATATAC	CC1
SMC22.T.AAGGCAGAGGGAACGG	CC1
SMC22.T.AAGGCAGCATGGTCTA	CC1
SMC22.T.AAGGCAGGTATGCTTG	CC1
SMC22.T.AAGGCAGGTCTGATCA	CC1
SMC22.T.AAGGCAGTCGATAGAA	CC1
SMC22.T.AAGGTTCAGAGTAAGG	CC1
SMC22.T.AAGGTTCAGATCTGCT	CC2
SMC22.T.AAGGTTCAGCGTAATA	CC2
SMC22.T.AAGGTTCAGTAGCCGA	CC2
SMC22.T.AAGGTTCCAATGTTGC	CC1

SMC22.T.AAGGTTCCATGGTTGT	CC1
SMC22.T.AAGGTTCTCATCGCTC	CC1
SMC22.T.AAGGTTCTCGCGATCG	CC1
SMC22.T.AAGGTTCTCTCTAGGA	CC1
SMC22.T.AAGTCTGAGAGGGATA	CC1
SMC22.T.AAGTCTGGTCTAGTCA	CC1
SMC22.T.AAGTCTGGTCTCTTAT	CC1
SMC22.T.AATCCAGCAAACCTAC	CC2
SMC22.T.AATCCAGTCTAGAGTC	CC1
SMC22.T.AATCGGTAGGATATAC	CC1
SMC22.T.AATCGGTCACGGATAG	CC2
SMC22.T.AATCGGTCAGCTTAAC	CC1
SMC22.T.AATCGGTGTAAACCTC	CC2
SMC22.T.AATCGGTTCCACGAAT	CC1
SMC22.T.AATCGGTTCCCTAACC	CC1
SMC22.T.AATCGGTTCTGTCTCG	CC1
SMC22.T.ACACCAACACCGTTGG	CC2
SMC22.T.ACACCAACACCTTGTC	CC1
SMC22.T.ACACCAAGTAACGACG	CC1
SMC22.T.ACACCAAGTGCAGTAG	CC1
SMC22.T.ACACCAAGTTAGTGGG	CC1
SMC22.T.ACACCAATCGGAAATA	CC2
SMC22.T.ACACCAATCGGTTCGG	CC2
SMC22.T.ACACCCTCAAAGCAAT	CC2
SMC22.T.ACACCCTGTAGCGTAG	CC1
SMC22.T.ACACCCTGTGCGCTTG	CC2
SMC22.T.ACACCGGAGGCAAAGA	CC1
SMC22.T.ACACCGGCAAATACAG	CC1
SMC22.T.ACACCGGGTCGCATCG	CC2
SMC22.T.ACACCGGGTTATTCTC	CC1
SMC22.T.ACACCGGTCAGCATGT	CC1
SMC22.T.ACACCGGTCATACGGT	CC1
SMC22.T.ACACCGGTCTTGTACT	CC1
SMC22.T.ACACTGAAGCGTGTCC	CC1
SMC22.T.ACACTGACAGCTATTG	CC2
SMC22.T.ACACTGAGTCGCTTCT	CC1
SMC22.T.ACACTGAGTGATGTGG	CC1
SMC22.T.ACACTGATCCCACTTG	CC1
SMC22.T.ACAGCCGAGTAAGTAC	CC1
SMC22.T.ACAGCCGTCGGAAATA	CC1
SMC22.T.ACAGCCGTCGTGGGAA	CC1
SMC22.T.ACAGCTAAGCAACGGT	CC1
SMC22.T.ACAGCTAAGCTTCGCG	CC1
SMC22.T.ACAGCTACAAGTAGTA	CC2
SMC22.T.ACAGCTAGTTGAGGTG	CC1
SMC22.T.ACATACGAGGCAGGTT	CC1
SMC22.T.ACATACGCAAGTTCTG	CC2
SMC22.T.ACATACGCACCTTGTC	CC1
SMC22.T.ACATACGGTTCGCGAC	CC2
SMC22.T.ACATACGTCTCCGGTT	CC1
SMC22.T.ACATCAGCAAACGTGG	CC1
SMC22.T.ACATCAGGTACAAGTA	CC2
SMC22.T.ACATGGTCACGCGAAA	CC2
SMC22.T.ACATGGTCAGCTCGCA	CC1
SMC22.T.ACATGGTTCGTCACGG	CC1
SMC22.T.ACCAGTAAGCCGTCGT	CC1
SMC22.T.ACCAGTAAGGATCGCA	CC2
SMC22.T.ACCAGTACAAAGGTGC	CC1

SMC22.T.ACCAGTATCCGAACGC	CC2
SMC22.T.ACCCACTCAATGTTGC	CC1
SMC22.T.ACCCACTCATATACGC	CC2
SMC22.T.ACCCACTGTCAAGCGA	CC1
SMC22.T.ACCCACTGTCTGCAAT	CC1
SMC22.T.ACCCACTGTGTTTGGT	CC2
SMC22.T.ACCCACTTCACGAAGG	CC1
SMC22.T.ACCGTAAAGAGTACAT	CC1
SMC22.T.ACCGTAACACCAGCAC	CC1
SMC22.T.ACCGTAATCCAGTAGT	CC1
SMC22.T.ACCGTAATCCTCCTAG	CC1
SMC22.T.ACCGTAATCTGCTGTC	CC1
SMC22.T.ACCTTTACAATCGAAA	CC2
SMC22.T.ACCTTTAGTAGTGAAT	CC1
SMC22.T.ACCTTTAGTGCAACGA	CC1
SMC22.T.ACCTTTATCACTCTTA	CC2
SMC22.T.ACCTTTATCTCTAGGA	CC2
SMC22.T.ACGAGCCAGCTGATAA	CC1
SMC22.T.ACGAGCCCAATCCAAC	CC2
SMC22.T.ACGAGCCCACAAGTAA	CC1
SMC22.T.ACGAGCCTCGAGCCCA	CC1
SMC22.T.ACGAGGACAAACAACA	CC1
SMC22.T.ACGAGGAGTGCCTGTG	CC1
SMC22.T.ACGAGGATCTGTCTAT	CC2
SMC22.T.ACGATACCATGCTGGC	CC1
SMC22.T.ACGATACTCTTACCTA	CC1
SMC22.T.ACGATGTGTTGAGTTC	CC1
SMC22.T.ACGATGTTCTACGAGT	CC1
SMC22.T.ACGCAGCGTAAATGAC	CC1
SMC22.T.ACGCAGCTCCTGTACC	CC2
SMC22.T.ACGCCAGCAGTCACTA	CC1
SMC22.T.ACGCCAGCATCAGTAC	CC1
SMC22.T.ACGCCGAAGGCCGAAT	CC1
SMC22.T.ACGCCGAAGGGCATGT	CC1
SMC22.T.ACGCCGACAATGTAAG	CC1
SMC22.T.ACGCCGAGTGCGATAG	CC1
SMC22.T.ACGCCGATCTGCAGTA	CC1
SMC22.T.ACGGAGAAGCTACCTA	CC1
SMC22.T.ACGGCCACACATCCAA	CC2
SMC22.T.ACGGCCACACCAGATT	CC1
SMC22.T.ACGGCCATCACGGTTA	CC2
SMC22.T.ACGGGCTGTCGAGATG	CC1
SMC22.T.ACGGGCTGTGCACTTA	CC1
SMC22.T.ACGGGCTTCATCTGCC	CC2
SMC22.T.ACGGGCTTCCGTCAAA	CC1
SMC22.T.ACGGGTCGTATGAATG	CC1
SMC22.T.ACGTCAAAGTCTCGGC	CC1
SMC22.T.ACGTCAAAGTGGCACA	CC2
SMC22.T.ACGTCAACAAGCTGGA	CC2
SMC22.T.ACGTCAAGTAGTACCT	CC1
SMC22.T.ACGTCAAGTTTGTGTG	CC1
SMC22.T.ACGTCAATCAGTCCCT	CC1
SMC22.T.ACTATCTCACAAGACG	CC1
SMC22.T.ACTATCTCACGGCGTT	CC1
SMC22.T.ACTATCTCACTTGGAT	CC1
SMC22.T.ACTATCTCATACAGCT	CC1
SMC22.T.ACTATCTCATACTCTT	CC1
SMC22.T.ACTATCTTCCTTCAAT	CC2

SMC22.T.ACTGAACAGTGTACCT	CC1
SMC22.T.ACTGAACGTACGACCC	CC1
SMC22.T.ACTGAACGTACTCTCC	CC1
SMC22.T.ACTGAGTAGCCCAACC	CC1
SMC22.T.ACTGAGTCAACACGCC	CC1
SMC22.T.ACTGAGTTCGATGAGG	CC1
SMC22.T.ACTGATGAGAGCCCAA	CC1
SMC22.T.ACTGATGAGCGAGAAA	CC1
SMC22.T.ACTGATGCAAGTCTGT	CC1
SMC22.T.ACTGATGGTCAATGTC	CC2
SMC22.T.ACTGATGGTCATCGGC	CC2
SMC22.T.ACTGATGTCCACTGGG	CC1
SMC22.T.ACTGATGTCGCGGATC	CC1
SMC22.T.ACTGCTCAGGCTCTTA	CC1
SMC22.T.ACTGCTCTCCTGTAGA	CC2
SMC22.T.ACTGTCCAGTAATCCC	CC2
SMC22.T.ACTGTCCGTCTTGATG	CC1
SMC22.T.ACTTACTGTACTCAAC	CC1
SMC22.T.ACTTGTTAGGCTAGGT	CC1
SMC22.T.ACTTGTTAGTGACATA	CC2
SMC22.T.ACTTGTTCAACTGCTA	CC1
SMC22.T.ACTTGTTCAATAACGA	CC1
SMC22.T.ACTTGTTCATAACCTG	CC1
SMC22.T.ACTTTCAAGGTGCTAG	CC2
SMC22.T.ACTTTCACATTCACTT	CC1
SMC22.T.ACTTTCAGTAACGTTC	CC1
SMC22.T.ACTTTCAGTTCGGCAC	CC2
SMC22.T.AGAATAGAGTTACGGG	CC1
SMC22.T.AGAATAGTCTCGATGA	CC2
SMC22.T.AGACGTTAGAGATGAG	CC1
SMC22.T.AGACGTTAGCCAGAAC	CC1
SMC22.T.AGACGTTAGCTACCGC	CC1
SMC22.T.AGACGTTAGCTAGCCC	CC1
SMC22.T.AGACGTTCACTGTTAG	CC1
SMC22.T.AGACGTTCATACGCTA	CC1
SMC22.T.AGACGTTTCAGTGCAT	CC1
SMC22.T.AGAGCGACAAATCCGT	CC2
SMC22.T.AGAGCGACACCATGTA	CC1
SMC22.T.AGAGCGACATGCTGGC	CC1
SMC22.T.AGAGCGAGTTCGCTAA	CC1
SMC22.T.AGAGCGATCAATCTCT	CC1
SMC22.T.AGAGCTTAGTCAAGCG	CC1
SMC22.T.AGAGCTTCACGAAACG	CC1
SMC22.T.AGAGCTTCACTGTCGG	CC1
SMC22.T.AGAGCTTCATTGTGCA	CC1
SMC22.T.AGAGCTTGTCTTCGTC	CC1
SMC22.T.AGAGTGGAGTGGTAAT	CC1
SMC22.T.AGAGTGGCATGCCTTC	CC2
SMC22.T.AGAGTGGGTGATAAAC	CC2
SMC22.T.AGATCTGAGCGTTTAC	CC1
SMC22.T.AGATCTGAGTGACATA	CC1
SMC22.T.AGATCTGCACCACCAG	CC1
SMC22.T.AGATCTGCACCGAAAG	CC1
SMC22.T.AGATCTGCACCGGAAA	CC2
SMC22.T.AGATCTGGTACAGACG	CC1
SMC22.T.AGATCTGGTCTGCGGT	CC1
SMC22.T.AGATCTGGTTAAGAAC	CC1
SMC22.T.AGATCTGGTTCCACGG	CC1

SMC22.T.AGATCTGTCACATGCA	CC1
SMC22.T.AGATCTGTCTCCAACC	CC1
SMC22.T.AGATTGCGTACCTACA	CC1
SMC22.T.AGCAGCCAGAACAATC	CC1
SMC22.T.AGCAGCCCACACCGCA	CC1
SMC22.T.AGCATACCAGAGCCAA	CC1
SMC22.T.AGCATACCATGTTGAC	CC1
SMC22.T.AGCATACGTAGGACAC	CC1
SMC22.T.AGCATACGTCCTAGCG	CC2
SMC22.T.AGCATACGTTTGACTG	CC1
SMC22.T.AGCATACTCCACTCCA	CC2
SMC22.T.AGCCTAAAGACGACGT	CC1
SMC22.T.AGCCTAAAGAGTCGGT	CC1
SMC22.T.AGCCTAACAATCAGAA	CC1
SMC22.T.AGCCTAAGTGAAATCA	CC1
SMC22.T.AGCGGTCAGCGATTCT	CC1
SMC22.T.AGCGGTCCAGCGAACA	CC1
SMC22.T.AGCGGTCTCAGCTCGG	CC1
SMC22.T.AGCGTATCAGGGCATA	CC1
SMC22.T.AGCGTATGTGTGACCC	CC2
SMC22.T.AGCGTCGCAACACGCC	CC1
SMC22.T.AGCGTCGGTGGGTCAA	CC1
SMC22.T.AGCGTCGTCCTACAGA	CC2
SMC22.T.AGCTCCTCAAGTACCT	CC1
SMC22.T.AGCTCCTCAATAGAGT	CC2
SMC22.T.AGCTCCTCATATGCTG	CC1
SMC22.T.AGCTCCTGTGACGCCT	CC2
SMC22.T.AGCTCCTTCAGCCTAA	CC1
SMC22.T.AGCTCTCCAAGAGTCG	CC1
SMC22.T.AGCTCTCCACATGGGA	CC1
SMC22.T.AGCTCTCGTTGTACAC	CC1
SMC22.T.AGCTTGACAAACGTGG	CC1
SMC22.T.AGCTTGACATGAACCT	CC1
SMC22.T.AGCTTGATCTAACTGG	CC1
SMC22.T.AGGCCACAGGATTCGG	CC1
SMC22.T.AGGCCACGTCGCATAT	CC2
SMC22.T.AGGCCACGTGTTTGTG	CC1
SMC22.T.AGGCCGTCAATGGAGC	CC2
SMC22.T.AGGCCGTGTCCAAGTT	CC1
SMC22.T.AGGCCGTTCACCGGGT	CC1
SMC22.T.AGGCCGTTCAGTTGAC	CC2
SMC22.T.AGGCCGTTCTAACTTC	CC1
SMC22.T.AGGCCGTTCTCTTATG	CC1
SMC22.T.AGGGAGTAGAGAACAG	CC1
SMC22.T.AGGGAGTGTAGATTAG	CC1
SMC22.T.AGGGAGTTCAAAGTAG	CC1
SMC22.T.AGGGATGCAAGCCGTC	CC1
SMC22.T.AGGGATGCAATGGAAT	CC2
SMC22.T.AGGGATGGTAGAGTGC	CC2
SMC22.T.AGGGATGGTCCAAGTT	CC1
SMC22.T.AGGGTGAAGGTGTTAA	CC1
SMC22.T.AGGGTGAGTGAGTGAC	CC1
SMC22.T.AGGGTGAGTGCGGTAA	CC1
SMC22.T.AGGTCATCAATGAATG	CC1
SMC22.T.AGGTCATGTCGCGAAA	CC1
SMC22.T.AGGTCATGTGCCTGGT	CC1
SMC22.T.AGGTCATGTGCCTGTG	CC1
SMC22.T.AGGTCATGTTAGATGA	CC2

SMC22.T.AGGTCCGAGTGAAGAG	CC1
SMC22.T.AGGTCCGCAAGTAGTA	CC1
SMC22.T.AGGTCCGGTGAAATCA	CC1
SMC22.T.AGGTCCGGTGGGTATG	CC1
SMC22.T.AGGTCCGTCGCAAACT	CC1
SMC22.T.AGTAGTCAGCAGGCTA	CC1
SMC22.T.AGTAGTCAGGAGTTTA	CC2
SMC22.T.AGTAGTCAGTATTGGA	CC2
SMC22.T.AGTAGTCGTTGAACTC	CC2
SMC22.T.AGTAGTCTCAGCTGGC	CC1
SMC22.T.AGTAGTCTCGTCCGTT	CC2
SMC22.T.AGTCTTTGTCGAGATG	CC1
SMC22.T.AGTCTTTGTTAAGTAG	CC1
SMC22.T.AGTCTTTTCGGAAACG	CC2
SMC22.T.AGTGAGGCATCTCCCA	CC1
SMC22.T.AGTGAGGGTATCAGTC	CC1
SMC22.T.AGTGAGGGTTCCATGA	CC2
SMC22.T.AGTGAGGTCGGTTCGG	CC2
SMC22.T.AGTGGGAAGTAGATGT	CC1
SMC22.T.AGTGGGAGTCAGAATA	CC1
SMC22.T.AGTGTCAAGGACATTA	CC1
SMC22.T.AGTGTCAAGTTAACGA	CC1
SMC22.T.AGTGTCACAAACGTGG	CC1
SMC22.T.AGTGTCACACCGATAT	CC1
SMC22.T.AGTGTCACAGGTGGAT	CC1
SMC22.T.AGTGTCACAGTTAACC	CC1
SMC22.T.AGTTGGTAGACTGTAA	CC2
SMC22.T.AGTTGGTAGGGCTTGA	CC1
SMC22.T.AGTTGGTTCAGCTCGG	CC1
SMC22.T.AGTTGGTTCGACCAGC	CC2
SMC22.T.ATAACGCAGGCCCGTT	CC2
SMC22.T.ATAACGCAGTATCGAA	CC1
SMC22.T.ATAACGCGTCAGAAGC	CC2
SMC22.T.ATAAGAGCAGTCTTCC	CC2
SMC22.T.ATAAGAGCATACTCTT	CC1
SMC22.T.ATAAGAGGTAAACCTC	CC1
SMC22.T.ATAAGAGGTCTCACCT	CC2
SMC22.T.ATAAGAGTCATAACCG	CC2
SMC22.T.ATAAGAGTCCAAACTG	CC1
SMC22.T.ATAAGAGTCCGAATGT	CC2
SMC22.T.ATCACGACATATGGTC	CC2
SMC22.T.ATCACGATCGCCTGTT	CC1
SMC22.T.ATCATGGAGACAAGCC	CC1
SMC22.T.ATCATGGAGGGCTTGA	CC1
SMC22.T.ATCATGGGTAAGTGGC	CC1
SMC22.T.ATCATGGGTCCTAGCG	CC1
SMC22.T.ATCATGGGTTCGCTAA	CC2
SMC22.T.ATCATGGTCCGCAGTG	CC1
SMC22.T.ATCCACCTCCTTGGTC	CC1
SMC22.T.ATCCACCTCTAGCACA	CC1
SMC22.T.ATCCACCTCTGTCTCG	CC2
SMC22.T.ATCCGAACATGTTCCC	CC2
SMC22.T.ATCCGAAGTGATAAAC	CC1
SMC22.T.ATCGAGTAGCGTGTCC	CC1
SMC22.T.ATCGAGTAGGCATGGT	CC1
SMC22.T.ATCGAGTCACTTAACG	CC1
SMC22.T.ATCGAGTCAGGGTTAG	CC2
SMC22.T.ATCGAGTCATGCCTAA	CC1

SMC22.T.ATCGAGTGTCCCGACA	CC1
SMC22.T.ATCTACTAGTAGATGT	CC1
SMC22.T.ATCTGCCAGGCTAGCA	CC1
SMC22.T.ATCTGCCCAAGCCATT	CC1
SMC22.T.ATCTGCCCATCGACGC	CC1
SMC22.T.ATCTGCCGTCCGTCAG	CC1
SMC22.T.ATCTGCCGTTCGGCAC	CC1
SMC22.T.ATGAGGGAGATCTGCT	CC1
SMC22.T.ATGAGGGGTGCCTTGG	CC2
SMC22.T.ATGAGGGGTGTGAATA	CC2
SMC22.T.ATGAGGGGTTGGTGGA	CC1
SMC22.T.ATGAGGGTCCATGAAC	CC1
SMC22.T.ATGCGATTCTCGGACG	CC1
SMC22.T.ATGGGAGAGCCTATGT	CC1
SMC22.T.ATGGGAGCACGAAAGC	CC2
SMC22.T.ATGGGAGCATTGAGCT	CC2
SMC22.T.ATGGGAGGTGTTCGAT	CC1
SMC22.T.ATGGGAGTCATGCTCC	CC1
SMC22.T.ATGGGAGTCCCTTGCA	CC1
SMC22.T.ATGTGTGAGATGGGTC	CC1
SMC22.T.ATGTGTGCAAAGTGCG	CC1
SMC22.T.ATGTGTGCAACGATCT	CC1
SMC22.T.ATGTGTGCACCTCGTT	CC2
SMC22.T.ATGTGTGTCCCGGATG	CC1
SMC22.T.ATGTGTGTCTGCCCTA	CC1
SMC22.T.ATTACTCAGCTAGTTC	CC1
SMC22.T.ATTACTCAGTCGTTTG	CC1
SMC22.T.ATTATCCAGCTACCTA	CC2
SMC22.T.ATTATCCCATCGTCGG	CC1
SMC22.T.ATTATCCCATTGTGCA	CC2
SMC22.T.ATTATCCGTTTGACAC	CC1
SMC22.T.ATTATCCTCCGCATAA	CC1
SMC22.T.ATTCTACAGATCTGAA	CC1
SMC22.T.ATTCTACAGTTGCAGG	CC1
SMC22.T.ATTCTACGTTGGTAAA	CC1
SMC22.T.ATTCTACTCGTTGACA	CC1
SMC22.T.ATTCTACTCTGCCAGG	CC1
SMC22.T.ATTGGACAGCGAAGGG	CC1
SMC22.T.ATTGGACAGTTGCAGG	CC1
SMC22.T.ATTGGACGTAATCACC	CC1
SMC22.T.ATTGGACGTATAGGGC	CC1
SMC22.T.ATTGGACTCAACACTG	CC1
SMC22.T.ATTGGACTCCTAGGGC	CC1
SMC22.T.ATTGGACTCCTGCCAT	CC1
SMC22.T.ATTGGACTCTACCTGC	CC1
SMC22.T.ATTGGACTCTTCGAGA	CC1
SMC22.T.ATTGGTGAGCAGGTCA	CC2
SMC22.T.ATTGGTGAGTTACCCA	CC2
SMC22.T.ATTGGTGCAATGCCAT	CC1
SMC22.T.ATTGGTGGTGACTACT	CC1
SMC22.T.CAACCAAAGAGGACGG	CC1
SMC22.T.CAACCAAAGGCTACGA	CC1
SMC22.T.CAACCAACATTGGGCC	CC1
SMC22.T.CAACCAATCCAGATCA	CC2
SMC22.T.CAACCAATCCCGGATG	CC1
SMC22.T.CAACCAATCGGCCGAT	CC1
SMC22.T.CAACCTCAGGGATGGG	CC1
SMC22.T.CAACCTCCAGGTCTCG	CC1

SMC22.T.CAACCTCGTACCGGCT	CC1
SMC22.T.CAACCTCTCCAGAAGG	CC1
SMC22.T.CAACTAGAGGATGCGT	CC1
SMC22.T.CAACTAGTCGTTACAG	CC1
SMC22.T.CAACTAGTCTTGCAAG	CC2
SMC22.T.CAAGAAACAAGGACAC	CC1
SMC22.T.CAAGAAAGTTGATTGC	CC1
SMC22.T.CAAGAAATCAACCAAC	CC1
SMC22.T.CAAGATCAGAAACGCC	CC1
SMC22.T.CAAGATCCAATGACCT	CC2
SMC22.T.CAAGGCCAGGCAGTCA	CC1
SMC22.T.CAAGGCCCAGCCACCA	CC1
SMC22.T.CAAGTTGCAGAGCCAA	CC1
SMC22.T.CAAGTTGGTTTCGCTC	CC2
SMC22.T.CAAGTTGTCAATCACG	CC2
SMC22.T.CACAAACAGCGTTCCG	CC2
SMC22.T.CACAAACCAACTTGAC	CC1
SMC22.T.CACAAACCACTGAAGG	CC1
SMC22.T.CACAAACGTAAATGAC	CC1
SMC22.T.CACAAACTCGCCAAAT	CC1
SMC22.T.CACACAAAGTCGTACT	CC2
SMC22.T.CACACAACACCAGATT	CC1
SMC22.T.CACACAATCAAAGTAG	CC1
SMC22.T.CACACAATCAGGTTCA	CC1
SMC22.T.CACACAATCCACGAAT	CC1
SMC22.T.CACACAATCTGGGCCA	CC1
SMC22.T.CACACCTCAAACTGTC	CC2
SMC22.T.CACACCTGTCTAACGT	CC1
SMC22.T.CACACCTTCGATCCCT	CC2
SMC22.T.CACACTCTCCGCGCAA	CC1
SMC22.T.CACACTCTCCTGCAGG	CC1
SMC22.T.CACACTCTCGTACCGG	CC2
SMC22.T.CACAGGCAGACTAAGT	CC1
SMC22.T.CACAGGCAGAGTAAGG	CC1
SMC22.T.CACAGGCTCATGTAGC	CC2
SMC22.T.CACAGGCTCCCTCAGT	CC1
SMC22.T.CACAGGCTCGTTGACA	CC1
SMC22.T.CACAGTAAGACCTAGG	CC1
SMC22.T.CACAGTAAGCTCCCAG	CC1
SMC22.T.CACAGTATCCTGCTTG	CC1
SMC22.T.CACATAGAGACCTTTG	CC1
SMC22.T.CACATAGAGATGTGTA	CC1
SMC22.T.CACATAGAGGATCGCA	CC1
SMC22.T.CACATAGAGGCACATG	CC1
SMC22.T.CACATAGGTTTCCACC	CC1
SMC22.T.CACATAGTCAACCAAC	CC1
SMC22.T.CACATAGTCAGTGCAT	CC1
SMC22.T.CACATTTAGCAAATCA	CC1
SMC22.T.CACCACTCAAGGCTCC	CC1
SMC22.T.CACCACTGTCTGCGGT	CC1
SMC22.T.CACCACTTCACCATAG	CC1
SMC22.T.CACCACTTCGCTTAGA	CC1
SMC22.T.CACCAGGTCCTTCAAT	CC1
SMC22.T.CACCTTGCAAGCGCTC	CC1
SMC22.T.CACCTTGCACACAGAG	CC1
SMC22.T.CACCTTGCACGGCCAT	CC1
SMC22.T.CACCTTGCATCGGTTA	CC1
SMC22.T.CACCTTGGTATGAATG	CC1

SMC22.T.CACCTTGGTTTGCATG	CC2
SMC22.T.CACCTTGTCTATCCCG	CC1
SMC22.T.CAGAATCAGGACAGCT	CC1
SMC22.T.CAGAATCGTCTACCTC	CC1
SMC22.T.CAGAATCGTCTTCGTC	CC1
SMC22.T.CAGAATCGTGTTCGAT	CC1
SMC22.T.CAGAATCTCCTTTCGG	CC1
SMC22.T.CAGAGAGCACCCATTC	CC1
SMC22.T.CAGAGAGGTCCGTCAG	CC1
SMC22.T.CAGAGAGTCTTGCAAG	CC1
SMC22.T.CAGATCAAGGGCTTCC	CC2
SMC22.T.CAGATCAAGTCTCCTC	CC2
SMC22.T.CAGATCAGTAAATACG	CC1
SMC22.T.CAGATCATCAATCACG	CC1
SMC22.T.CAGATCATCTGTGCAA	CC2
SMC22.T.CAGCAGCAGGACAGAA	CC1
SMC22.T.CAGCAGCGTCTTCTCG	CC1
SMC22.T.CAGCAGCGTGCAACGA	CC2
SMC22.T.CAGCAGCGTTCGCTAA	CC1
SMC22.T.CAGCATAAGCAATATG	CC1
SMC22.T.CAGCATAAGGCCCTTG	CC1
SMC22.T.CAGCATACAAGACGTG	CC1
SMC22.T.CAGCATACAGGCTGAA	CC1
SMC22.T.CAGCATAGTCGAACAG	CC1
SMC22.T.CAGCATATCAGTTCGA	CC1
SMC22.T.CAGCATATCCGGCACA	CC1
SMC22.T.CAGCCGAAGACAAAGG	CC1
SMC22.T.CAGCCGACACGAAAGC	CC1
SMC22.T.CAGCCGAGTATTCGTG	CC1
SMC22.T.CAGCCGAGTCAAAGCG	CC1
SMC22.T.CAGCCGAGTCCGAATT	CC1
SMC22.T.CAGCCGAGTCGACTAT	CC2
SMC22.T.CAGCCGATCCTATTCA	CC1
SMC22.T.CAGCGACAGCTGAACG	CC1
SMC22.T.CAGCGACTCATTCACT	CC2
SMC22.T.CAGCGACTCTTACCTA	CC2
SMC22.T.CAGCTAAGTGACTCAT	CC2
SMC22.T.CAGCTAAGTTCATGGT	CC1
SMC22.T.CAGCTGGAGAGTACAT	CC2
SMC22.T.CAGCTGGAGTTTCCTT	CC2
SMC22.T.CAGCTGGCAGGGAGAG	CC2
SMC22.T.CAGCTGGCAGGTCGTC	CC1
SMC22.T.CAGCTGGCATTCCTGC	CC1
SMC22.T.CAGGTGCAGCCAGGAT	CC2
SMC22.T.CAGGTGCAGGTGCAAC	CC2
SMC22.T.CAGGTGCTCTCCTATA	CC2
SMC22.T.CAGTAACCATCGACGC	CC1
SMC22.T.CAGTAACGTAAATACG	CC1
SMC22.T.CAGTAACTCACGACTA	CC1
SMC22.T.CAGTCCTCATACCATG	CC1
SMC22.T.CAGTCCTGTCGGCATC	CC1
SMC22.T.CAGTCCTGTCTAAAGA	CC1
SMC22.T.CAGTCCTTCAGCATGT	CC1
SMC22.T.CAGTCCTTCATTATCC	CC1
SMC22.T.CATATGGTCGTAGGTT	CC2
SMC22.T.CATATTCGTTCCCGAG	CC1
SMC22.T.CATATTCTCGTCGTTC	CC1
SMC22.T.CATCAAGCAAGGTTTC	CC1

SMC22.T.CATCAAGGTCACCTAA	CC1
SMC22.T.CATCAAGGTCTACCTC	CC1
SMC22.T.CATCAAGTCATTTGGG	CC2
SMC22.T.CATCAGACAGCCACCA	CC1
SMC22.T.CATCAGACAGTCAGAG	CC1
SMC22.T.CATCAGAGTCTCTTAT	CC1
SMC22.T.CATCCACAGCCAGGAT	CC1
SMC22.T.CATCCACCACCATGTA	CC1
SMC22.T.CATCGAACAAGGACAC	CC2
SMC22.T.CATCGAACACACAGAG	CC1
SMC22.T.CATCGAAGTTATGCGT	CC1
SMC22.T.CATCGAATCCGTAGGC	CC1
SMC22.T.CATCGGGAGCGTGAAC	CC1
SMC22.T.CATCGGGCATGCCTAA	CC2
SMC22.T.CATCGGGGTTCCCTTG	CC1
SMC22.T.CATCGGGTCCGCGTTT	CC1
SMC22.T.CATGACACAATGTAAG	CC1
SMC22.T.CATGACACATCCTTGC	CC1
SMC22.T.CATGACACATGAGCGA	CC1
SMC22.T.CATGACAGTGCAGTAG	CC1
SMC22.T.CATGACATCAAACGGG	CC1
SMC22.T.CATGACATCGTACCGG	CC2
SMC22.T.CATGCCTCATGGTCAT	CC1
SMC22.T.CATGGCGAGGGTTCCC	CC1
SMC22.T.CATGGCGGTGAGCGAT	CC1
SMC22.T.CATGGCGTCCAGAAGG	CC1
SMC22.T.CATGGCGTCCGAGCCA	CC1
SMC22.T.CATTATCAGCCCAGCT	CC1
SMC22.T.CATTATCAGCCGGTAA	CC1
SMC22.T.CATTATCAGGAGTCTG	CC1
SMC22.T.CATTATCCAGCTCGCA	CC1
SMC22.T.CATTATCTCGACCAGC	CC1
SMC22.T.CATTCGCAGCTAACTC	CC2
SMC22.T.CATTCGCCAAGTAATG	CC1
SMC22.T.CATTCGCCATCAGTCA	CC1
SMC22.T.CATTCGCCATGTTCCC	CC1
SMC22.T.CATTCGCGTCTTGTCC	CC1
SMC22.T.CATTCGCGTGTGGTTT	CC1
SMC22.T.CATTCGCTCAAGCCTA	CC1
SMC22.T.CATTCGCTCCCGACTT	CC2
SMC22.T.CCAATCCAGGGCTTCC	CC1
SMC22.T.CCAATCCCACCATGTA	CC1
SMC22.T.CCAATCCGTTACGCGC	CC2
SMC22.T.CCAATCCGTTGGACCC	CC1
SMC22.T.CCAATCCTCCGGGTGT	CC2
SMC22.T.CCAATCCTCGGAATCT	CC1
SMC22.T.CCACCTACAACAACCT	CC1
SMC22.T.CCACGGAAGTCATGCT	CC2
SMC22.T.CCACGGAGTCAGTGGA	CC1
SMC22.T.CCACGGAGTTGCTCCT	CC2
SMC22.T.CCACGGATCGTTACAG	CC2
SMC22.T.CCACTACCAGCCACCA	CC2
SMC22.T.CCAGCGACAATTGCTG	CC1
SMC22.T.CCATGTCAGCCTATGT	CC2
SMC22.T.CCATGTCCACGGTTTA	CC2
SMC22.T.CCATGTCTCTAACCGA	CC1
SMC22.T.CCATTCGAGATGTGTA	CC1
SMC22.T.CCATTCGAGCCGGTAA	CC1

SMC22.T.CCATTCGAGGTGCAAC	CC1
SMC22.T.CCATTCGAGTGAAGTT	CC2
SMC22.T.CCATTCGCACAGGAGT	CC1
SMC22.T.CCATTCGGTGAGGGTT	CC1
SMC22.T.CCCAATCGTCCTGCTT	CC1
SMC22.T.CCCAGTTCACGCGAAA	CC1
SMC22.T.CCCAGTTTCGACAGCC	CC2
SMC22.T.CCCATACAGGAATCGC	CC1
SMC22.T.CCCATACCAAGCGATG	CC2
SMC22.T.CCCATACCACTAAGTC	CC1
SMC22.T.CCCTCCTTCAACCAAC	CC1
SMC22.T.CCCTCCTTCATAACCG	CC1
SMC22.T.CCCTCCTTCCGAATGT	CC1
SMC22.T.CCCTCCTTCGTTTATC	CC1
SMC22.T.CCGGGATAGACACGAC	CC1
SMC22.T.CCGGGATAGTACTTGC	CC1
SMC22.T.CCGGGATCATACAGCT	CC1
SMC22.T.CCGGGATCATCGGGTC	CC1
SMC22.T.CCGGGATGTCAGTGGA	CC2
SMC22.T.CCGGGATGTGCAGGTA	CC2
SMC22.T.CCGGGATGTGCGCTTG	CC2
SMC22.T.CCGGGATTCCCTGACT	CC1
SMC22.T.CCGGTAGAGCCTCGTG	CC1
SMC22.T.CCGGTAGCACTAAGTC	CC1
SMC22.T.CCGGTAGCATCCGTGG	CC1
SMC22.T.CCGTACTGTCACAAGG	CC1
SMC22.T.CCGTGGAAGGTAAACT	CC1
SMC22.T.CCGTGGACATGTCTCC	CC1
SMC22.T.CCGTGGAGTACTCTCC	CC2
SMC22.T.CCGTGGAGTGGTACAG	CC1
SMC22.T.CCGTTCAAGGTGTGGT	CC1
SMC22.T.CCGTTCACACCCATGG	CC1
SMC22.T.CCGTTCAGTCATCCCT	CC1
SMC22.T.CCGTTCAGTGACCAAG	CC2
SMC22.T.CCTAAAGCAGTCTTCC	CC2
SMC22.T.CCTAAAGTCCTGTACC	CC1
SMC22.T.CCTACACAGAGGACGG	CC1
SMC22.T.CCTACACAGGACTGGT	CC1
SMC22.T.CCTACACGTCTAAAGA	CC1
SMC22.T.CCTACACTCTGGGCCA	CC1
SMC22.T.CCTACCAAGACAAAGG	CC1
SMC22.T.CCTACCAAGTACTTGC	CC1
SMC22.T.CCTACCAAGTGCAAGC	CC1
SMC22.T.CCTACCACAATGGAGC	CC1
SMC22.T.CCTACCAGTCCGAGTC	CC1
SMC22.T.CCTACCAGTGAAATCA	CC1
SMC22.T.CCTACCAGTGGGTATG	CC1
SMC22.T.CCTACCATCAGAGACG	CC1
SMC22.T.CCTACCATCATCATTC	CC1
SMC22.T.CCTAGCTGTTAAGAAC	CC1
SMC22.T.CCTAGCTTCATAAAGG	CC1
SMC22.T.CCTATTAAGAGCCCAA	CC1
SMC22.T.CCTATTAAGATGCCAG	CC1
SMC22.T.CCTATTACACAGTCGC	CC2
SMC22.T.CCTCAGTAGGAGTAGA	CC1
SMC22.T.CCTCAGTAGGTGATAT	CC1
SMC22.T.CCTCTGAAGTAATCCC	CC1
SMC22.T.CCTCTGACAGTGGAGT	CC1

SMC22.T.CCTCTGAGTTATCGGT	CC1
SMC22.T.CCTCTGAGTTCCACAA	CC1
SMC22.T.CCTCTGAGTTTCGCTC	CC1
SMC22.T.CCTTACGAGCTGAAAT	CC2
SMC22.T.CCTTACGTCCAACCAA	CC1
SMC22.T.CCTTCCCCAACAACCT	CC2
SMC22.T.CCTTCCCCATTACGAC	CC2
SMC22.T.CCTTCCCTCCACTGGG	CC1
SMC22.T.CCTTCCCTCTCTGTCG	CC1
SMC22.T.CCTTCGAAGAAGGTGA	CC1
SMC22.T.CCTTCGAAGAGTACAT	CC1
SMC22.T.CCTTCGACAGTGGAGT	CC1
SMC22.T.CCTTCGACATGCCTAA	CC1
SMC22.T.CCTTCGAGTCGCTTCT	CC1
SMC22.T.CCTTTCTAGACTTGAA	CC2
SMC22.T.CCTTTCTAGTCTCGGC	CC1
SMC22.T.CCTTTCTGTACTCAAC	CC2
SMC22.T.CCTTTCTGTCAGAATA	CC1
SMC22.T.CGAACATTCGCGTAGC	CC1
SMC22.T.CGAATGTGTGCAGACA	CC1
SMC22.T.CGACCTTCAGCTGCTG	CC1
SMC22.T.CGACCTTTCTGAAAGA	CC2
SMC22.T.CGACTTCAGAAGCCCA	CC2
SMC22.T.CGACTTCCATTCTTAC	CC1
SMC22.T.CGACTTCTCGGCTTGG	CC2
SMC22.T.CGAGAAGCATAAGACA	CC1
SMC22.T.CGAGAAGGTGAGGGAG	CC1
SMC22.T.CGAGCACAGCAACGGT	CC1
SMC22.T.CGAGCACAGCAGACTG	CC1
SMC22.T.CGAGCACAGTCCGGTC	CC1
SMC22.T.CGAGCACCACAACGCC	CC2
SMC22.T.CGAGCACCATATGAGA	CC2
SMC22.T.CGAGCACGTGGTACAG	CC1
SMC22.T.CGAGCCAAGCTAACTC	CC2
SMC22.T.CGAGCCAGTTCGCGAC	CC1
SMC22.T.CGAGCCAGTTGGTAAA	CC2
SMC22.T.CGAGCCATCCACGACG	CC1
SMC22.T.CGATCGGAGCGCCTCA	CC2
SMC22.T.CGATCGGCAATCGAAA	CC1
SMC22.T.CGATCGGCACCATCCT	CC1
SMC22.T.CGATCGGCAGATCGGA	CC1
SMC22.T.CGATCGGCATGGTCTA	CC2
SMC22.T.CGATCGGTCAGTTTGG	CC1
SMC22.T.CGATCGGTCCGGGTGT	CC1
SMC22.T.CGATCGGTCCGTTGCT	CC1
SMC22.T.CGATGGCCAGCCAGAA	CC1
SMC22.T.CGATGGCGTAATTGGA	CC2
SMC22.T.CGATGGCGTTGAGGTG	CC1
SMC22.T.CGATGTAAGTTGTAGA	CC2
SMC22.T.CGATGTACAAATCCGT	CC1
SMC22.T.CGATGTACAAGTCTGT	CC1
SMC22.T.CGATGTACATCACAAC	CC1
SMC22.T.CGATGTAGTGTAAGTA	CC2
SMC22.T.CGATGTATCGCCAGCA	CC1
SMC22.T.CGATGTATCTATCCCG	CC2
SMC22.T.CGATGTATCTTTCCTC	CC1
SMC22.T.CGATTGAAGTTGCAGG	CC1
SMC22.T.CGATTGAGTCTAGCGC	CC1

SMC22.T.CGCCAAGAGAGATGAG	CC1
SMC22.T.CGCCAAGAGTGTGAAT	CC2
SMC22.T.CGCCAAGGTCCTAGCG	CC2
SMC22.T.CGCGGTAAGAACTCGG	CC1
SMC22.T.CGCGGTACACAGGTTT	CC2
SMC22.T.CGCGTTTAGCATCATC	CC2
SMC22.T.CGCTATCAGAGCCTAG	CC1
SMC22.T.CGCTATCTCAAACAAG	CC1
SMC22.T.CGCTGGAAGTATGACA	CC1
SMC22.T.CGCTGGACATGTTCCC	CC2
SMC22.T.CGCTGGAGTCAGAATA	CC1
SMC22.T.CGCTGGAGTCGAGTTT	CC1
SMC22.T.CGCTGGAGTTCCAACA	CC1
SMC22.T.CGCTGGATCAGGCCCA	CC1
SMC22.T.CGCTGGATCCCAACGG	CC1
SMC22.T.CGGACACAGTGCGTGA	CC1
SMC22.T.CGGACACAGTGTGGCA	CC1
SMC22.T.CGGACACCACATGGGA	CC1
SMC22.T.CGGACACGTATTCGTG	CC2
SMC22.T.CGGACACTCGTAGGTT	CC1
SMC22.T.CGGACGTAGGCCGAAT	CC1
SMC22.T.CGGACGTAGTGCTGCC	CC1
SMC22.T.CGGACGTTCGGCGCTA	CC1
SMC22.T.CGGACTGAGACTAGAT	CC1
SMC22.T.CGGACTGAGCGAAGGG	CC1
SMC22.T.CGGACTGGTGGAAAGA	CC2
SMC22.T.CGGACTGGTTACCGAT	CC1
SMC22.T.CGGACTGGTTTACTCT	CC1
SMC22.T.CGGAGCTAGGAGCGTT	CC1
SMC22.T.CGGAGCTAGTTTAGGA	CC1
SMC22.T.CGGAGCTCAGCGTCCA	CC1
SMC22.T.CGGAGCTGTAGGACAC	CC1
SMC22.T.CGGAGCTGTCTCTCTG	CC1
SMC22.T.CGGAGCTTCTACCTGC	CC1
SMC22.T.CGGAGTCAGACGACGT	CC1
SMC22.T.CGGAGTCGTCGTTGTA	CC1
SMC22.T.CGGAGTCGTGAAAGAG	CC1
SMC22.T.CGGAGTCTCAGCACAT	CC2
SMC22.T.CGGCTAGAGTCAAGGC	CC1
SMC22.T.CGGCTAGCACGTCTCT	CC1
SMC22.T.CGGCTAGGTCGGCTCA	CC1
SMC22.T.CGGCTAGGTCTGATCA	CC1
SMC22.T.CGGCTAGTCTGGGCCA	CC2
SMC22.T.CGGGTCAAGTTTCCTT	CC1
SMC22.T.CGGGTCACAGGGCATA	CC1
SMC22.T.CGGGTCACATCCTAGA	CC1
SMC22.T.CGGGTCAGTGCCTGGT	CC1
SMC22.T.CGGGTCATCCGTCAAA	CC1
SMC22.T.CGGTTAAAGCACGCCT	CC2
SMC22.T.CGGTTAAAGTGTACTC	CC1
SMC22.T.CGGTTAAGTTATCCGA	CC1
SMC22.T.CGTAGCGAGAGGTTGC	CC2
SMC22.T.CGTAGCGAGATGTGTA	CC1
SMC22.T.CGTAGCGAGCTTATCG	CC1
SMC22.T.CGTAGCGAGTGGTAGC	CC1
SMC22.T.CGTAGCGGTTAAGGGC	CC1
SMC22.T.CGTAGCGGTTATCGGT	CC1
SMC22.T.CGTAGCGTCTACTTAC	CC1

SMC22.T.CGTAGCGTCTGTTGAG	CC1
SMC22.T.CGTAGGCAGGAGTTTA	CC1
SMC22.T.CGTAGGCAGTCACGCC	CC1
SMC22.T.CGTAGGCGTCCCGACA	CC1
SMC22.T.CGTAGGCTCGGTGTCG	CC1
SMC22.T.CGTCACTGTGACGGTA	CC1
SMC22.T.CGTCAGGAGCCACGTC	CC1
SMC22.T.CGTCAGGCAGTCTTCC	CC1
SMC22.T.CGTCAGGTCAGGATCT	CC1
SMC22.T.CGTCCATCAAGAGGCT	CC2
SMC22.T.CGTCCATCACAACGTT	CC1
SMC22.T.CGTCCATCAGCTCCGA	CC1
SMC22.T.CGTCCATCATAGAAAC	CC1
SMC22.T.CGTCCATGTCTTCGTC	CC1
SMC22.T.CGTCCATTCTGCAGTA	CC1
SMC22.T.CGTCCATTCTTTCCTC	CC1
SMC22.T.CGTCTACCAGACAGGT	CC1
SMC22.T.CGTCTACCATTCACTT	CC1
SMC22.T.CGTCTACGTCCGACGT	CC2
SMC22.T.CGTCTACTCAACCATG	CC1
SMC22.T.CGTGAGCAGAAACCGC	CC1
SMC22.T.CGTGAGCCACAACGCC	CC2
SMC22.T.CGTGAGCTCACCTTAT	CC1
SMC22.T.CGTGAGCTCACTCTTA	CC1
SMC22.T.CGTGAGCTCCGCATAA	CC1
SMC22.T.CGTGTAAAGCGTAGTG	CC2
SMC22.T.CGTGTAAAGGGTTCCC	CC1
SMC22.T.CGTGTAAAGTGAACAT	CC1
SMC22.T.CGTGTAACAGAGTGTG	CC1
SMC22.T.CGTGTAACAGATAATG	CC1
SMC22.T.CGTGTCTCAGGGCATA	CC1
SMC22.T.CGTGTCTGTCCAGTGC	CC2
SMC22.T.CGTGTCTTCCTTTCTC	CC1
SMC22.T.CGTTAGAAGAAGCCCA	CC1
SMC22.T.CGTTAGAAGTGTCCCG	CC1
SMC22.T.CGTTAGAAGTTAGCGG	CC1
SMC22.T.CGTTAGACATTATCTC	CC1
SMC22.T.CGTTAGAGTCTTTCAT	CC1
SMC22.T.CGTTAGATCTACTTAC	CC1
SMC22.T.CGTTAGATCTCGCATC	CC1
SMC22.T.CGTTCTGAGTGTTAGA	CC1
SMC22.T.CGTTCTGGTTACGTCA	CC2
SMC22.T.CGTTCTGTCACCACCT	CC1
SMC22.T.CGTTGGGCAATGGACG	CC1
SMC22.T.CGTTGGGGTATATCCG	CC1
SMC22.T.CGTTGGGGTGCATCTA	CC1
SMC22.T.CGTTGGGTCCGTAGTA	CC2
SMC22.T.CTAACTTAGCCAACAG	CC1
SMC22.T.CTAACTTAGCTGTCTA	CC1
SMC22.T.CTAACTTCAACGCACC	CC1
SMC22.T.CTAACTTGTATATCCG	CC1
SMC22.T.CTAACTTTCGTGGGAA	CC2
SMC22.T.CTAAGACGTTCGCGAC	CC1
SMC22.T.CTAAGACTCAACACAC	CC1
SMC22.T.CTAAGACTCCCTGACT	CC2
SMC22.T.CTAAGACTCGATGAGG	CC1
SMC22.T.CTAAGACTCGGAATCT	CC1
SMC22.T.CTAATGGCACTGCCAG	CC2

SMC22.T.CTAATGGCATCCCATC	CC1
SMC22.T.CTAATGGGTAAAGGAG	CC1
SMC22.T.CTAATGGTCTCGTTTA	CC1
SMC22.T.CTACACCAGAATCTCC	CC1
SMC22.T.CTACACCAGCGTAGTG	CC2
SMC22.T.CTACACCAGGCTCATT	CC1
SMC22.T.CTACACCCAAGACGTG	CC2
SMC22.T.CTACACCGTCGGCTCA	CC1
SMC22.T.CTACACCGTGATGTCT	CC1
SMC22.T.CTACACCGTTTGGGCC	CC2
SMC22.T.CTACATTCATTACCTT	CC1
SMC22.T.CTACCCACACATGACT	CC2
SMC22.T.CTACCCAGTGCGAAAC	CC2
SMC22.T.CTACGTCAGAATTGTG	CC1
SMC22.T.CTACGTCAGTACGATA	CC1
SMC22.T.CTACGTCGTAGCCTAT	CC1
SMC22.T.CTACGTCGTGGGTCAA	CC2
SMC22.T.CTACGTCGTGTGACGA	CC1
SMC22.T.CTACGTCGTTTAGGAA	CC1
SMC22.T.CTAGAGTAGATCCCAT	CC1
SMC22.T.CTAGAGTCATGCATGT	CC1
SMC22.T.CTAGAGTGTTGCGCAC	CC1
SMC22.T.CTAGAGTTCACAGTAC	CC1
SMC22.T.CTAGCCTAGCCCGAAA	CC1
SMC22.T.CTAGCCTCATTGGGCC	CC1
SMC22.T.CTAGCCTGTGCCTGGT	CC1
SMC22.T.CTAGCCTTCTGGCGAC	CC1
SMC22.T.CTAGTGAAGAATTGTG	CC1
SMC22.T.CTAGTGAAGATGCCTT	CC2
SMC22.T.CTAGTGAGTCGCGAAA	CC1
SMC22.T.CTAGTGAGTGAGCGAT	CC1
SMC22.T.CTAGTGAGTGGTAACG	CC1
SMC22.T.CTCACACCATATGAGA	CC1
SMC22.T.CTCACACCATTCTTAC	CC1
SMC22.T.CTCACACGTAACGTTC	CC2
SMC22.T.CTCACACGTAAGTAGT	CC2
SMC22.T.CTCACACGTCTGCGGT	CC1
SMC22.T.CTCACACTCCCAACGG	CC1
SMC22.T.CTCACACTCCGTCATC	CC2
SMC22.T.CTCACACTCGGCGCTA	CC1
SMC22.T.CTCACACTCGTCACGG	CC1
SMC22.T.CTCAGAAAGCGTTTAC	CC2
SMC22.T.CTCAGAAAGTGCTGCC	CC1
SMC22.T.CTCAGAACAAGTAATG	CC1
SMC22.T.CTCCTAGAGCGTGAGT	CC1
SMC22.T.CTCCTAGCAGCGATCC	CC2
SMC22.T.CTCCTAGCAGCGTTCG	CC1
SMC22.T.CTCCTAGGTCCCGACA	CC2
SMC22.T.CTCCTAGTCACTATTC	CC2
SMC22.T.CTCCTAGTCGCAGGCT	CC1
SMC22.T.CTCCTAGTCTGGGCCA	CC1
SMC22.T.CTCGAAAAGCGCCTTG	CC1
SMC22.T.CTCGAAAAGCTAACAA	CC1
SMC22.T.CTCGAAAAGTCCAGGA	CC2
SMC22.T.CTCGAAACACACCGCA	CC1
SMC22.T.CTCGAAAGTGTAACGG	CC1
SMC22.T.CTCGAAAGTTCCACAA	CC2
SMC22.T.CTCGAGGAGGCTATCT	CC1

SMC22.T.CTCGAGGAGTGGGCTA	CC1
SMC22.T.CTCGAGGAGTTCCACA	CC1
SMC22.T.CTCGAGGCACATCTTT	CC1
SMC22.T.CTCGGAGGTCCATCCT	CC1
SMC22.T.CTCGGGAAGCTGCAAG	CC2
SMC22.T.CTCGGGAAGTTCGCGC	CC2
SMC22.T.CTCGTACAGACGCTTT	CC1
SMC22.T.CTCGTACCACATCCGG	CC1
SMC22.T.CTCGTACGTCTAACGT	CC1
SMC22.T.CTCGTCAAGCAGCGTA	CC1
SMC22.T.CTCGTCAAGGAGTCTG	CC1
SMC22.T.CTCGTCAAGTACGCGA	CC1
SMC22.T.CTCGTCAAGTCCATAC	CC1
SMC22.T.CTCGTCATCCGTAGTA	CC1
SMC22.T.CTCGTCATCTGTCTAT	CC1
SMC22.T.CTCTAATAGATATACG	CC2
SMC22.T.CTCTAATAGGCGTACA	CC1
SMC22.T.CTCTAATAGTTCGCAT	CC1
SMC22.T.CTCTAATAGTTGTAGA	CC1
SMC22.T.CTCTAATCAGCGTAAG	CC1
SMC22.T.CTCTAATCAGTCACTA	CC1
SMC22.T.CTCTAATCATACCATG	CC1
SMC22.T.CTCTAATGTCCAGTGC	CC1
SMC22.T.CTCTAATTCCTAAGTG	CC1
SMC22.T.CTCTAATTCGAGGTAG	CC1
SMC22.T.CTCTACGAGATGGGTC	CC1
SMC22.T.CTCTACGAGGACAGCT	CC1
SMC22.T.CTCTGGTAGACGCACA	CC2
SMC22.T.CTCTGGTAGAGAGCTC	CC1
SMC22.T.CTCTGGTCATTCTTAC	CC1
SMC22.T.CTCTGGTGTAGCACGA	CC1
SMC22.T.CTCTGGTTCCCAAGAT	CC1
SMC22.T.CTCTGGTTCTGTCTAT	CC2
SMC22.T.CTGAAACAGGACAGCT	CC2
SMC22.T.CTGAAACCAGATGAGC	CC1
SMC22.T.CTGAAACCAGCTGTGC	CC1
SMC22.T.CTGAAACCATCGTCGG	CC1
SMC22.T.CTGAAGTAGTTAAGTG	CC2
SMC22.T.CTGAAGTCAAGACACG	CC1
SMC22.T.CTGAAGTCAGACGCTC	CC1
SMC22.T.CTGAAGTCAGACGTAG	CC1
SMC22.T.CTGAAGTGTATGGTTC	CC1
SMC22.T.CTGATAGAGGGTGTGT	CC2
SMC22.T.CTGATAGGTCCATCCT	CC1
SMC22.T.CTGATCCAGAAGGACA	CC1
SMC22.T.CTGATCCAGGCTCATT	CC1
SMC22.T.CTGATCCAGTGGGTTG	CC2
SMC22.T.CTGATCCCATGCAATC	CC1
SMC22.T.CTGCCTAAGAGACTTA	CC1
SMC22.T.CTGCCTAAGGGAGTAA	CC1
SMC22.T.CTGCCTACAAAGTCAA	CC2
SMC22.T.CTGCCTACAGACAAAT	CC2
SMC22.T.CTGCCTAGTGCACCAC	CC1
SMC22.T.CTGCCTATCACCTTAT	CC2
SMC22.T.CTGCCTATCATCATTC	CC1
SMC22.T.CTGCCTATCTGTCTAT	CC1
SMC22.T.CTGCGGACACCAGGCT	CC1
SMC22.T.CTGCGGAGTACAGTTC	CC1

SMC22.T.CTGCGGAGTCTGCGGT	CC2
SMC22.T.CTGCGGAGTGCAACTT	CC1
SMC22.T.CTGCTGTAGAGGTAGA	CC2
SMC22.T.CTGCTGTAGTGAACAT	CC2
SMC22.T.CTGCTGTCACGAGGTA	CC1
SMC22.T.CTGCTGTCACTAAGTC	CC2
SMC22.T.CTGCTGTTCGCCAGCA	CC1
SMC22.T.CTGCTGTTCTGCGGCA	CC1
SMC22.T.CTGGTCTCAAACCTAC	CC1
SMC22.T.CTGGTCTGTCAACATC	CC1
SMC22.T.CTGGTCTTCGCGTAGC	CC2
SMC22.T.CTGTGCTAGAAACCAT	CC1
SMC22.T.CTGTGCTAGTGTTAGA	CC2
SMC22.T.CTGTGCTAGTTGAGTA	CC2
SMC22.T.CTGTGCTCAAGGTTCT	CC2
SMC22.T.CTGTTTAAGGTGGGTT	CC1
SMC22.T.CTGTTTAAGTCGATAA	CC1
SMC22.T.CTGTTTACACGGATAG	CC1
SMC22.T.CTGTTTACAGGTGGAT	CC1
SMC22.T.CTGTTTAGTCTGCGGT	CC2
SMC22.T.CTTAACTCAGATCTGT	CC1
SMC22.T.CTTAACTCATCGTCGG	CC1
SMC22.T.CTTAACTTCACATACG	CC2
SMC22.T.CTTAACTTCTGATTCT	CC1
SMC22.T.CTTACCGTCACCTCGT	CC1
SMC22.T.CTTACCGTCTTGCATT	CC1
SMC22.T.CTTAGGAAGTAGATGT	CC1
SMC22.T.CTTAGGAAGTGTACTC	CC1
SMC22.T.CTTAGGACAACACGCC	CC1
SMC22.T.CTTAGGACAAGACGTG	CC1
SMC22.T.CTTAGGACATGGGACA	CC1
SMC22.T.CTTAGGATCCTTTCGG	CC1
SMC22.T.CTTCTCTAGACATAAC	CC1
SMC22.T.CTTCTCTAGCTGCAAG	CC1
SMC22.T.CTTCTCTTCAGAAATG	CC1
SMC22.T.CTTGGCTTCACATACG	CC1
SMC22.T.CTTTGCGCATGTTGAC	CC2
SMC22.T.CTTTGCGGTAGCGCTC	CC1
SMC22.T.CTTTGCGTCAAAGACA	CC2
SMC22.T.GAAACTCAGACGACGT	CC1
SMC22.T.GAAACTCAGCTGAAAT	CC2
SMC22.T.GAAACTCCACCAGCAC	CC1
SMC22.T.GAAACTCTCCACTGGG	CC1
SMC22.T.GAAATGAAGATCCGAG	CC1
SMC22.T.GAAATGAAGCTGATAA	CC1
SMC22.T.GAAATGACAATAACGA	CC2
SMC22.T.GAAATGACACGGTGTC	CC1
SMC22.T.GAAATGATCTTCTGGC	CC1
SMC22.T.GAACATCAGCCCAACC	CC1
SMC22.T.GAACCTAAGCGACGTA	CC1
SMC22.T.GAACCTAAGCTCCTTC	CC1
SMC22.T.GAACCTACATGGGACA	CC1
SMC22.T.GAACCTAGTCAAACTC	CC1
SMC22.T.GAACCTATCCCATTTA	CC2
SMC22.T.GAACCTATCCTTGGTC	CC1
SMC22.T.GAACGGACATATGCTG	CC1
SMC22.T.GAAGCAGAGCACAGGT	CC1
SMC22.T.GAAGCAGCACATAACC	CC2

SMC22.T.GAAGCAGTCTGGCGAC	CC1
SMC22.T.GAATAAGAGAGTACCG	CC1
SMC22.T.GAATAAGGTAGCGATG	CC2
SMC22.T.GAATAAGGTCGTCTTC	CC2
SMC22.T.GAATAAGTCTGTCCGT	CC1
SMC22.T.GAATGAAAGATATGCA	CC1
SMC22.T.GAATGAAAGCATCATC	CC1
SMC22.T.GAATGAAAGGTGCACA	CC1
SMC22.T.GAATGAATCCGATATG	CC1
SMC22.T.GACACGCAGGTCATCT	CC1
SMC22.T.GACACGCCAAGCTGTT	CC1
SMC22.T.GACACGCGTAAGTTCC	CC1
SMC22.T.GACACGCGTCATGCCG	CC1
SMC22.T.GACACGCGTTCCAACA	CC1
SMC22.T.GACAGAGCATGCAACT	CC2
SMC22.T.GACAGAGCATGTCTCC	CC1
SMC22.T.GACCAATGTCTAGGTT	CC1
SMC22.T.GACCTGGAGGCTAGGT	CC1
SMC22.T.GACCTGGAGTCGAGTG	CC1
SMC22.T.GACCTGGGTGTAACGG	CC1
SMC22.T.GACGCGTAGAAGAAGC	CC2
SMC22.T.GACGCGTAGCGCCTCA	CC2
SMC22.T.GACGCGTAGCTAAGAT	CC1
SMC22.T.GACGCGTCACAGATTC	CC1
SMC22.T.GACGCGTGTCGACTGC	CC1
SMC22.T.GACGCGTTCAGGATCT	CC2
SMC22.T.GACGCGTTCTCCCTGA	CC1
SMC22.T.GACGCGTTCTCGAGTA	CC1
SMC22.T.GACGCGTTCTGATTCT	CC1
SMC22.T.GACGGCTAGATAGCAT	CC1
SMC22.T.GACGGCTCACCATCCT	CC1
SMC22.T.GACGGCTGTCGAATCT	CC2
SMC22.T.GACGGCTTCGGCGCTA	CC1
SMC22.T.GACGGCTTCTACTATC	CC1
SMC22.T.GACGTGCAGCCGATTT	CC2
SMC22.T.GACGTGCGTACATGTC	CC1
SMC22.T.GACGTGCTCCTGCCAT	CC1
SMC22.T.GACGTTACAAAGGCGT	CC1
SMC22.T.GACGTTACAGCGAACA	CC2
SMC22.T.GACGTTATCAGCATGT	CC1
SMC22.T.GACTAACCAATTGCTG	CC2
SMC22.T.GACTAACCACAGATTC	CC1
SMC22.T.GACTACACATGGGACA	CC1
SMC22.T.GACTACAGTCAGCTAT	CC1
SMC22.T.GACTACATCCAACCAA	CC2
SMC22.T.GACTGCGCAGACGTAG	CC1
SMC22.T.GACTGCGGTATCAGTC	CC1
SMC22.T.GACTGCGTCAGGCGAA	CC1
SMC22.T.GAGCAGAAGGGATACC	CC2
SMC22.T.GAGCAGAAGTGTGAAT	CC1
SMC22.T.GAGCAGACACGTCAGC	CC1
SMC22.T.GAGCAGACAGGTTTCA	CC1
SMC22.T.GAGCAGATCACAACGT	CC1
SMC22.T.GAGCAGATCACCTTAT	CC1
SMC22.T.GAGCAGATCGACGGAA	CC1
SMC22.T.GAGCAGATCGTAGGAG	CC1
SMC22.T.GAGGTGAAGATAGCAT	CC1
SMC22.T.GAGGTGAGTTCGGCAC	CC2

SMC22.T.GAGTCCGAGAGCCCAA	CC1
SMC22.T.GAGTCCGGTGCTCTTC	CC1
SMC22.T.GAGTCCGGTTTAGGAA	CC2
SMC22.T.GAGTCCGTCTTACCGC	CC2
SMC22.T.GATCAGTCACGCGAAA	CC1
SMC22.T.GATCAGTGTAGCGTCC	CC1
SMC22.T.GATCGATAGGCACATG	CC1
SMC22.T.GATCGATAGGCTATCT	CC1
SMC22.T.GATCGATAGGGATGGG	CC1
SMC22.T.GATCGATAGTGGTAAT	CC1
SMC22.T.GATCGATCAACGATCT	CC2
SMC22.T.GATCGATCAAGCCCAC	CC1
SMC22.T.GATCGATTCCGTTGTC	CC1
SMC22.T.GATCGCGAGCAGCGTA	CC1
SMC22.T.GATCGCGCAGCAGTTT	CC1
SMC22.T.GATCGTAGTCGCGTGT	CC2
SMC22.T.GATCTAGAGATATGGT	CC1
SMC22.T.GATCTAGGTCTTCGTC	CC1
SMC22.T.GATCTAGTCGTGACAT	CC2
SMC22.T.GATGAAAAGGATGCGT	CC2
SMC22.T.GATGAAATCAGCCTAA	CC1
SMC22.T.GATGAGGAGATGCCAG	CC1
SMC22.T.GATGAGGAGCGCCTCA	CC1
SMC22.T.GATGAGGAGGGTGTGT	CC1
SMC22.T.GATGAGGCAAGGCTCC	CC1
SMC22.T.GATGAGGCATACTCTT	CC1
SMC22.T.GATGCTACAACTGCGC	CC1
SMC22.T.GATGCTAGTCACACGC	CC1
SMC22.T.GATTCAGAGCGCCTTG	CC1
SMC22.T.GATTCAGCATGGGACA	CC1
SMC22.T.GATTCAGGTACAGTGG	CC1
SMC22.T.GATTCAGGTTAGTGGG	CC1
SMC22.T.GATTCAGTCCCAAGTA	CC1
SMC22.T.GCAAACTAGTTCGCAT	CC1
SMC22.T.GCAAACTCATACGCCG	CC2
SMC22.T.GCAAACTTCAGCTCGG	CC1
SMC22.T.GCAATCACAATGGATA	CC1
SMC22.T.GCAATCACACCGTTGG	CC2
SMC22.T.GCACATAAGTGGTAGC	CC1
SMC22.T.GCACATAAGTTCCACA	CC2
SMC22.T.GCACATAGTCGATTGT	CC1
SMC22.T.GCACATAGTTGTTTGG	CC1
SMC22.T.GCACTCTAGCCCAACC	CC1
SMC22.T.GCACTCTGTAAGGGCT	CC1
SMC22.T.GCAGCCAAGCCTCGTG	CC1
SMC22.T.GCAGCCACATACTCTT	CC2
SMC22.T.GCAGCCAGTAAGGGAA	CC1
SMC22.T.GCAGCCAGTACCGCTG	CC1
SMC22.T.GCAGCCAGTCCAGTAT	CC2
SMC22.T.GCAGCCAGTGGTAACG	CC1
SMC22.T.GCAGCCATCACCACCT	CC1
SMC22.T.GCAGCCATCTACTATC	CC1
SMC22.T.GCAGTTAAGCAGGCTA	CC1
SMC22.T.GCAGTTAGTTCGTGAT	CC1
SMC22.T.GCATACAAGTGTCCCG	CC2
SMC22.T.GCATACACAACGATGG	CC1
SMC22.T.GCATACAGTACTTAGC	CC2
SMC22.T.GCATACAGTCTAGTGT	CC1

SMC22.T.GCATACAGTTCTGGTA	CC1
SMC22.T.GCATACATCCTCAATT	CC1
SMC22.T.GCATACATCCTGCCAT	CC1
SMC22.T.GCATGATAGCTCCTTC	CC1
SMC22.T.GCATGATAGGCCCTCA	CC1
SMC22.T.GCATGATGTGACAAAT	CC1
SMC22.T.GCATGATTCAGCGACC	CC1
SMC22.T.GCATGATTCCTTCAAT	CC1
SMC22.T.GCATGCGAGAAACCAT	CC2
SMC22.T.GCATGCGAGTAAGTAC	CC1
SMC22.T.GCATGCGCATGTTCCC	CC1
SMC22.T.GCATGCGGTACCGTTA	CC1
SMC22.T.GCATGCGTCACGCATA	CC2
SMC22.T.GCATGTAAGGCCCTCA	CC1
SMC22.T.GCATGTACAGGCAGTA	CC1
SMC22.T.GCATGTATCGTTTGCC	CC1
SMC22.T.GCATGTATCTCAAACG	CC1
SMC22.T.GCCAAATAGGTGTTAA	CC1
SMC22.T.GCCAAATCAATCGGTT	CC1
SMC22.T.GCCAAATCAGGATTGG	CC1
SMC22.T.GCCAAATCATACTCTT	CC1
SMC22.T.GCCAAATTCCGTCATC	CC2
SMC22.T.GCCTCTAAGCCCAATT	CC1
SMC22.T.GCCTCTATCGTGGACC	CC2
SMC22.T.GCGACCACATATGGTC	CC1
SMC22.T.GCGACCAGTTGCCTCT	CC2
SMC22.T.GCGACCATCAACCAAC	CC2
SMC22.T.GCGAGAAAGGACAGCT	CC2
SMC22.T.GCGAGAAAGTCCCACG	CC1
SMC22.T.GCGAGAACATCACGTA	CC1
SMC22.T.GCGCAACAGTGGGTTG	CC2
SMC22.T.GCGCAACCAAAGTGCG	CC1
SMC22.T.GCGCAACGTATAAACG	CC1
SMC22.T.GCGCAACGTGCAGTAG	CC1
SMC22.T.GCGCAGTAGTGCCATT	CC1
SMC22.T.GCGCCAAAGATCCGAG	CC1
SMC22.T.GCGCCAACATAAAGGT	CC1
SMC22.T.GCGCCAAGTAATTGGA	CC1
SMC22.T.GCGCGATGTGTTGAGG	CC2
SMC22.T.GCGCGATTCAGCTCGG	CC2
SMC22.T.GCGGGTTAGAATGTGT	CC1
SMC22.T.GCGGGTTAGGTAGCTG	CC2
SMC22.T.GCGGGTTAGTTGAGTA	CC1
SMC22.T.GCGGGTTCACAAGACG	CC1
SMC22.T.GCGGGTTCATTGGCGC	CC1
SMC22.T.GCGGGTTGTAAGAGGA	CC1
SMC22.T.GCGGGTTTCAGCGACC	CC2
SMC22.T.GCTCTGTAGCAGACTG	CC1
SMC22.T.GCTCTGTAGGACGAAA	CC1
SMC22.T.GCTCTGTAGTGTCCAT	CC1
SMC22.T.GCTCTGTCACAGCGTC	CC1
SMC22.T.GCTCTGTGTATCTGCA	CC2
SMC22.T.GCTCTGTGTGTGCGTC	CC2
SMC22.T.GCTGCAGAGAGAGCTC	CC2
SMC22.T.GCTGCAGAGATCCTGT	CC1
SMC22.T.GCTGCAGAGGAGTCTG	CC2
SMC22.T.GCTGCAGCAAGGTTTC	CC2
SMC22.T.GCTGCAGCAGGACGTA	CC1

SMC22.T.GCTGCAGGTTGCCTCT	CC1
SMC22.T.GCTGCAGGTTGGACCC	CC2
SMC22.T.GCTGCAGTCAGTCCCT	CC2
SMC22.T.GCTGCGAAGCTAGCCC	CC2
SMC22.T.GCTGCGACAATCACAC	CC2
SMC22.T.GCTGCGACAATCGGTT	CC1
SMC22.T.GCTGCGATCTGTGCAA	CC1
SMC22.T.GCTGCTTAGGAGTCTG	CC1
SMC22.T.GCTGCTTAGTACGCGA	CC1
SMC22.T.GCTGCTTCATCGTCGG	CC1
SMC22.T.GCTGCTTGTAATCACC	CC1
SMC22.T.GCTGCTTGTACGCACC	CC1
SMC22.T.GCTGCTTGTGTTAAGA	CC2
SMC22.T.GCTGGGTTCCAAACTG	CC1
SMC22.T.GCTGGGTTCTATCCTA	CC2
SMC22.T.GCTTCCAAGAAACCGC	CC2
SMC22.T.GCTTCCAGTCAAACTC	CC1
SMC22.T.GCTTCCAGTGTGACCC	CC1
SMC22.T.GCTTCCATCATATCGG	CC1
SMC22.T.GCTTCCATCGTTACAG	CC1
SMC22.T.GCTTCCATCTGCCAGG	CC1
SMC22.T.GCTTGAAAGCCTATGT	CC1
SMC22.T.GCTTGAATCCAAGTAC	CC1
SMC22.T.GCTTGAATCCGCGTTT	CC2
SMC22.T.GCTTGAATCTCGTATT	CC2
SMC22.T.GGAAAGCAGCCCTAAT	CC1
SMC22.T.GGAAAGCCATCACCCT	CC2
SMC22.T.GGAAAGCCATGTCTCC	CC1
SMC22.T.GGAAAGCTCAGTTTGG	CC2
SMC22.T.GGAAAGCTCGAGAGCA	CC1
SMC22.T.GGAACTTCATCCGGGT	CC1
SMC22.T.GGAACTTGTACCGTAT	CC1
SMC22.T.GGAACTTTCAACACCA	CC2
SMC22.T.GGAATAAGTCTGGAGA	CC1
SMC22.T.GGAATAAGTTCCACAA	CC2
SMC22.T.GGAATAATCTTATCTG	CC1
SMC22.T.GGACAAGAGCCTTGAT	CC1
SMC22.T.GGACAAGCATACAGCT	CC1
SMC22.T.GGACAAGCATTCCTCG	CC1
SMC22.T.GGACAAGGTACTCTCC	CC1
SMC22.T.GGACAAGGTCAAACTC	CC1
SMC22.T.GGACAAGGTGCCTGTG	CC2
SMC22.T.GGACAGAAGTGCCAGA	CC2
SMC22.T.GGACAGAAGTGGGATC	CC1
SMC22.T.GGACAGACAGCTGCAC	CC2
SMC22.T.GGACAGAGTCCTCCAT	CC1
SMC22.T.GGACAGATCAAGATCC	CC2
SMC22.T.GGACAGATCCAGTAGT	CC2
SMC22.T.GGACAGATCCCAAGAT	CC1
SMC22.T.GGACAGATCGAATGCT	CC1
SMC22.T.GGACATTAGTGCTGCC	CC2
SMC22.T.GGACATTTCTCTGCTG	CC1
SMC22.T.GGACGTCAGTGCTGCC	CC1
SMC22.T.GGACGTCCACGACGAA	CC2
SMC22.T.GGACGTCGTGCAGTAG	CC1
SMC22.T.GGACGTCGTTACGCGC	CC1
SMC22.T.GGACGTCTCAACGGGA	CC1
SMC22.T.GGACGTCTCCAAAGTC	CC1

SMC22.T.GGAGCAAAGATATACG	CC1
SMC22.T.GGAGCAAAGGGTTTCT	CC1
SMC22.T.GGAGCAAAGTACGTTC	CC2
SMC22.T.GGAGCAACACCTGGTG	CC1
SMC22.T.GGAGCAACACTGCCAG	CC1
SMC22.T.GGAGCAACATCCAACA	CC2
SMC22.T.GGAGCAAGTAGCGATG	CC1
SMC22.T.GGAGCAAGTCAGCTAT	CC1
SMC22.T.GGAGCAAGTGTTCGAT	CC1
SMC22.T.GGAGCAAGTTCAGACT	CC1
SMC22.T.GGATGTTAGAGTACCG	CC1
SMC22.T.GGATGTTAGTTTGCGT	CC1
SMC22.T.GGATGTTCAAGAAGAG	CC2
SMC22.T.GGATGTTGTACAGACG	CC2
SMC22.T.GGATGTTGTATATCCG	CC1
SMC22.T.GGATTACAGAGTGAGA	CC2
SMC22.T.GGATTACAGATGAGAG	CC2
SMC22.T.GGATTACAGCTGCAAG	CC1
SMC22.T.GGCAATTAGTGCTGCC	CC1
SMC22.T.GGCAATTTCACGACTA	CC1
SMC22.T.GGCCGATAGCTAGTTC	CC1
SMC22.T.GGCCGATAGTCCCACG	CC1
SMC22.T.GGCCGATCAGACAAGC	CC1
SMC22.T.GGCCGATGTTGCCTCT	CC1
SMC22.T.GGCCGATTCGGATGTT	CC1
SMC22.T.GGCGACTAGCTGCGAA	CC1
SMC22.T.GGCGACTGTTAGATGA	CC2
SMC22.T.GGCGACTTCCTGCAGG	CC1
SMC22.T.GGCGTGTAGTCCGTAT	CC2
SMC22.T.GGCGTGTCATGGGAAC	CC1
SMC22.T.GGCGTGTTCCAAACTG	CC1
SMC22.T.GGCGTGTTCGACAGCC	CC1
SMC22.T.GGCTCGAAGTACTTGC	CC1
SMC22.T.GGCTCGAAGTGGTCCC	CC1
SMC22.T.GGCTCGAGTGCAGGTA	CC2
SMC22.T.GGCTGGTAGCCACGTC	CC2
SMC22.T.GGCTGGTAGCGGATCA	CC1
SMC22.T.GGCTGGTCATATACGC	CC2
SMC22.T.GGCTGGTCATCATCCC	CC1
SMC22.T.GGCTGGTCATTACCTT	CC2
SMC22.T.GGCTGGTGTTACGTCA	CC1
SMC22.T.GGGAATGAGTGCGATG	CC1
SMC22.T.GGGAATGCAAGCGCTC	CC2
SMC22.T.GGGAATGGTAATTGGA	CC1
SMC22.T.GGGAATGTCAGTTTGG	CC1
SMC22.T.GGGAATGTCGATAGAA	CC1
SMC22.T.GGGACCTAGACAGACC	CC1
SMC22.T.GGGACCTTCAATACCG	CC1
SMC22.T.GGGACCTTCGATCCCT	CC2
SMC22.T.GGGACCTTCTGGCGTG	CC1
SMC22.T.GGGACCTTCTTAGCCC	CC1
SMC22.T.GGGAGATAGCTCTCGG	CC1
SMC22.T.GGGAGATCATCCGTGG	CC2
SMC22.T.GGGAGATTCTCTGTCG	CC1
SMC22.T.GGGATGAAGAAACCAT	CC2
SMC22.T.GGGATGACAAGCCATT	CC1
SMC22.T.GGGATGACATGTTCCC	CC1
SMC22.T.GGGATGAGTAATCGTC	CC1

SMC22.T.GGGCACTAGATCGGGT	CC1
SMC22.T.GGGCACTGTACTTAGC	CC1
SMC22.T.GGGCACTGTGTGAAAT	CC1
SMC22.T.GGGCACTTCATCGATG	CC1
SMC22.T.GGGCATCGTAGAAGGA	CC2
SMC22.T.GGGCATCTCACTTACT	CC1
SMC22.T.GGGCATCTCAGCATGT	CC1
SMC22.T.GGGCATCTCTACCTGC	CC1
SMC22.T.GGGTTGCAGCGCCTTG	CC1
SMC22.T.GGTATTGAGCCCGAAA	CC1
SMC22.T.GGTATTGAGTTAAGTG	CC1
SMC22.T.GGTATTGCACCACGTG	CC1
SMC22.T.GGTATTGCATGAACCT	CC1
SMC22.T.GGTGAAGAGCGTGTCC	CC1
SMC22.T.GGTGAAGCAGGTCTCG	CC2
SMC22.T.GGTGAAGCATCTGGTA	CC1
SMC22.T.GGTGAAGCATGTTCCC	CC2
SMC22.T.GGTGAAGGTCCAAGTT	CC2
SMC22.T.GGTGAAGTCTTCGAGA	CC2
SMC22.T.GGTGCGTCACCAGTTA	CC1
SMC22.T.GGTGCGTGTAGCTGCC	CC2
SMC22.T.GGTGCGTGTCAAAGAT	CC2
SMC22.T.GGTGCGTTCTAGCACA	CC1
SMC22.T.GGTGCGTTCTGTCCGT	CC1
SMC22.T.GGTGTTACACTGTCGG	CC1
SMC22.T.GGTGTTAGTTTGACAC	CC1
SMC22.T.GGTGTTATCACAACGT	CC1
SMC22.T.GGTGTTATCGACAGCC	CC1
SMC22.T.GGTGTTATCTGGTATG	CC1
SMC22.T.GTAACGTCATTATCTC	CC1
SMC22.T.GTAACTGAGCCAGTAG	CC2
SMC22.T.GTAACTGAGTAACCCT	CC1
SMC22.T.GTAACTGGTTGTGGAG	CC2
SMC22.T.GTAACTGTCAACCATG	CC2
SMC22.T.GTAACTGTCATGGTCA	CC1
SMC22.T.GTACGTAGTTTAGCTG	CC1
SMC22.T.GTACGTATCGCGTAGC	CC1
SMC22.T.GTACGTATCGTAGGAG	CC1
SMC22.T.GTACGTATCTTGTACT	CC2
SMC22.T.GTACTCCAGGAATGGA	CC2
SMC22.T.GTACTCCCAGGTTTCA	CC1
SMC22.T.GTACTCCGTTGACGTT	CC2
SMC22.T.GTACTTTAGAGACTAT	CC1
SMC22.T.GTACTTTAGCAATCTC	CC2
SMC22.T.GTACTTTCAACACCCG	CC2
SMC22.T.GTACTTTCAAGTAGTA	CC2
SMC22.T.GTACTTTTCACTTATC	CC1
SMC22.T.GTACTTTTCATGCATG	CC1
SMC22.T.GTAGGCCAGAAACCAT	CC1
SMC22.T.GTAGGCCCAATAACGA	CC1
SMC22.T.GTAGGCCCACGGTTTA	CC1
SMC22.T.GTAGGCCTCCATGCTC	CC1
SMC22.T.GTAGGCCTCTGTCTAT	CC1
SMC22.T.GTAGTCAAGATAGTCA	CC1
SMC22.T.GTAGTCACAAAGGTGC	CC1
SMC22.T.GTAGTCAGTACGCACC	CC1
SMC22.T.GTAGTCAGTCCTGCTT	CC1
SMC22.T.GTAGTCAGTGCGCTTG	CC1

SMC22.T.GTAGTCAGTGTTTGTG	CC2
SMC22.T.GTAGTCAGTTAGTGGG	CC1
SMC22.T.GTAGTCAGTTCAACCA	CC1
SMC22.T.GTATCTTAGGACACCA	CC1
SMC22.T.GTATCTTAGGCCGAAT	CC1
SMC22.T.GTATCTTCAGCTGGCT	CC1
SMC22.T.GTATTCTAGAACAATC	CC1
SMC22.T.GTATTCTAGGATATAC	CC1
SMC22.T.GTATTCTCAGCCAATT	CC1
SMC22.T.GTATTCTCATGACATC	CC1
SMC22.T.GTATTCTGTGCGAAAC	CC1
SMC22.T.GTATTCTGTTCGTCTC	CC1
SMC22.T.GTATTCTGTTTCCACC	CC1
SMC22.T.GTATTCTTCCGTTGCT	CC1
SMC22.T.GTCAAGTAGAAAGTGG	CC1
SMC22.T.GTCAAGTAGCTAACTC	CC1
SMC22.T.GTCACAAAGGAATGGA	CC2
SMC22.T.GTCACAAAGGCATTGG	CC1
SMC22.T.GTCACAACAGCTATTG	CC2
SMC22.T.GTCACAACATGGTCTA	CC1
SMC22.T.GTCACAAGTACACCGC	CC2
SMC22.T.GTCACAATCAGCTTAG	CC2
SMC22.T.GTCACGGGTCGCGTGT	CC2
SMC22.T.GTCACGGTCCATGCTC	CC1
SMC22.T.GTCATTTAGGATGGAA	CC1
SMC22.T.GTCATTTGTTTACTCT	CC1
SMC22.T.GTCATTTTCTGGTGTA	CC2
SMC22.T.GTCCTCAAGGATTCGG	CC1
SMC22.T.GTCCTCACAGACAAGC	CC2
SMC22.T.GTCCTCATCTAACTGG	CC1
SMC22.T.GTCGGGTCAAACCTAC	CC1
SMC22.T.GTCGGGTCATTCACTT	CC2
SMC22.T.GTCGGGTGTGCAACTT	CC1
SMC22.T.GTCGGGTTCGAGAGCA	CC1
SMC22.T.GTCGTAAGTGTTGGGA	CC1
SMC22.T.GTCGTAAGTTGAGTTC	CC2
SMC22.T.GTCGTAATCACGAAGG	CC1
SMC22.T.GTCGTAATCATAACCG	CC1
SMC22.T.GTCGTAATCATGCAAC	CC1
SMC22.T.GTCGTAATCCTAGTGA	CC1
SMC22.T.GTCTCGTAGATGCCAG	CC1
SMC22.T.GTCTCGTCATGGGACA	CC1
SMC22.T.GTCTTCGGTAGGCTGA	CC1
SMC22.T.GTCTTCGGTGGGTCAA	CC1
SMC22.T.GTCTTCGTCCAAACAC	CC1
SMC22.T.GTGAAGGAGAAGAAGC	CC1
SMC22.T.GTGAAGGAGTCCATAC	CC2
SMC22.T.GTGAAGGCAGGGATTG	CC1
SMC22.T.GTGAAGGGTGCCTGGT	CC1
SMC22.T.GTGAAGGGTGTAATGA	CC1
SMC22.T.GTGAAGGTCACATAGC	CC1
SMC22.T.GTGCAGCAGACTAAGT	CC1
SMC22.T.GTGCAGCAGCGTAGTG	CC2
SMC22.T.GTGCAGCAGCGTGAGT	CC1
SMC22.T.GTGCAGCCAATCTGCA	CC1
SMC22.T.GTGCAGCCACACATGT	CC1
SMC22.T.GTGCAGCCACAGAGGT	CC1
SMC22.T.GTGCAGCCATGCAACT	CC1

SMC22.T.GTGCAGCGTATATGGA	CC1
SMC22.T.GTGCAGCGTCGCGGTT	CC1
SMC22.T.GTGCAGCTCTGATTCT	CC1
SMC22.T.GTGCATACATATGGTC	CC1
SMC22.T.GTGCATAGTGCGAAAC	CC2
SMC22.T.GTGCATATCGAACGGA	CC1
SMC22.T.GTGCATATCGTAGGTT	CC1
SMC22.T.GTGCGGTAGCCAGGAT	CC1
SMC22.T.GTGCGGTAGTCAATAG	CC1
SMC22.T.GTGCGGTAGTCGATAA	CC1
SMC22.T.GTGCGGTAGTGAACGC	CC1
SMC22.T.GTGCGGTGTATAATGG	CC1
SMC22.T.GTGCGGTGTGCATCTA	CC1
SMC22.T.GTGCTTCAGTGAATTG	CC1
SMC22.T.GTGCTTCCAGACGCCT	CC1
SMC22.T.GTGCTTCCAGCCACCA	CC1
SMC22.T.GTGCTTCGTCGAATCT	CC1
SMC22.T.GTGCTTCGTGCTCTTC	CC1
SMC22.T.GTGCTTCTCCTGTAGA	CC1
SMC22.T.GTGCTTCTCGCGGATC	CC2
SMC22.T.GTGCTTCTCGGCCGAT	CC2
SMC22.T.GTGCTTCTCTACTCAT	CC1
SMC22.T.GTGGGTCAGACTAAGT	CC2
SMC22.T.GTGGGTCAGCATCATC	CC2
SMC22.T.GTGGGTCGTAGCCTAT	CC2
SMC22.T.GTGGGTCTCCTAGAAC	CC2
SMC22.T.GTGTGCGCACATGTGT	CC1
SMC22.T.GTGTGCGCAGCATGAG	CC1
SMC22.T.GTGTGCGTCAAACGGG	CC1
SMC22.T.GTGTTAGAGGTGACCA	CC1
SMC22.T.GTGTTAGAGTTAACGA	CC1
SMC22.T.GTGTTAGCAATGAATG	CC1
SMC22.T.GTGTTAGCAGTGGGAT	CC1
SMC22.T.GTGTTAGCAGTTCCCT	CC1
SMC22.T.GTGTTAGGTTGGAGGT	CC2
SMC22.T.GTGTTAGTCATCGATG	CC1
SMC22.T.GTGTTAGTCGAGAACG	CC2
SMC22.T.GTTAAGCCACCACGTG	CC2
SMC22.T.GTTAAGCCATGGAATA	CC1
SMC22.T.GTTAAGCTCCCTAACC	CC1
SMC22.T.GTTAAGCTCCTCAACC	CC1
SMC22.T.GTTACAGGTCATCGGC	CC2
SMC22.T.GTTACAGGTGTGACGA	CC2
SMC22.T.GTTCATTAGCTGTCTA	CC2
SMC22.T.GTTCATTAGTACTTGC	CC1
SMC22.T.GTTCATTAGTTGTAGA	CC2
SMC22.T.GTTCATTCAAGCGAGT	CC1
SMC22.T.GTTCATTCATCCTTGC	CC1
SMC22.T.GTTCATTGTCTCTCGT	CC2
SMC22.T.GTTCATTGTTAGGGTG	CC2
SMC22.T.GTTCATTTCATGGTCA	CC1
SMC22.T.GTTCGGGGTCTTGCGG	CC1
SMC22.T.GTTCGGGTCGCGATCG	CC1
SMC22.T.GTTCTCGGTCTTCTCG	CC1
SMC22.T.GTTCTCGGTTTGACAC	CC1
SMC22.T.GTTCTCGTCCATTCTA	CC1
SMC22.T.GTTTCTAAGATGTCGG	CC2
SMC22.T.GTTTCTACATGCATGT	CC1

SMC22.T.GTTTCTAGTCAGATAA	CC1
SMC22.T.GTTTCTAGTCTAGCGC	CC1
SMC22.T.GTTTCTAGTCTTGATG	CC1
SMC22.T.GTTTCTAGTGTCGCTG	CC2
SMC22.T.GTTTCTATCCAAAGTC	CC1
SMC22.T.GTTTCTATCCCAGGTG	CC1
SMC22.T.GTTTCTATCTGTCCGT	CC1
SMC22.T.TAAGAGAAGTGAAGAG	CC1
SMC22.T.TAAGAGATCGCCCTTA	CC1
SMC22.T.TAAGCGTAGAAACCAT	CC1
SMC22.T.TAAGCGTAGATCCGAG	CC1
SMC22.T.TAAGCGTCAGTTCATG	CC1
SMC22.T.TAAGCGTGTCATGCCG	CC2
SMC22.T.TAAGTGCAGCGATGAC	CC1
SMC22.T.TAAGTGCCACTAAGTC	CC1
SMC22.T.TAAGTGCGTACAGTGG	CC1
SMC22.T.TAAGTGCGTGTGAATA	CC1
SMC22.T.TAAGTGCTCCATTCTA	CC1
SMC22.T.TACACGACAACACCTA	CC1
SMC22.T.TACACGACAGTAAGAT	CC2
SMC22.T.TACACGATCTCAACTT	CC1
SMC22.T.TACACGATCTTACCGC	CC2
SMC22.T.TACAGTGAGGATGGTC	CC1
SMC22.T.TACAGTGAGTTAAGTG	CC1
SMC22.T.TACCTATAGAATTGTG	CC1
SMC22.T.TACCTATAGCCCGAAA	CC1
SMC22.T.TACCTATAGCTAGTCT	CC1
SMC22.T.TACCTATCAGATCTGT	CC1
SMC22.T.TACCTATGTGACGCCT	CC1
SMC22.T.TACCTTAAGAGTTGGC	CC2
SMC22.T.TACCTTAAGCAGCGTA	CC1
SMC22.T.TACGGATCATGATCCA	CC2
SMC22.T.TACGGATGTTCGCTAA	CC2
SMC22.T.TACGGGCAGCACACAG	CC2
SMC22.T.TACGGGCTCAAGAAGT	CC1
SMC22.T.TACGGTACACAGCCCA	CC1
SMC22.T.TACGGTAGTACCGTTA	CC1
SMC22.T.TACGGTAGTCAGATAA	CC1
SMC22.T.TACGGTAGTCTCAACA	CC1
SMC22.T.TACGGTAGTTTCGCTC	CC1
SMC22.T.TACTCATAGAGTCTGG	CC2
SMC22.T.TACTCATTCGAGCCCA	CC1
SMC22.T.TACTCGCAGACTAGGC	CC1
SMC22.T.TACTCGCAGGCCCTCA	CC1
SMC22.T.TACTCGCGTGACTCAT	CC1
SMC22.T.TACTTACAGAATTGTG	CC1
SMC22.T.TACTTACAGTCCATAC	CC2
SMC22.T.TACTTACTCTGTTTGT	CC1
SMC22.T.TACTTGTAGAGTAATC	CC1
SMC22.T.TACTTGTCAACGCACC	CC1
SMC22.T.TACTTGTCACAGACTT	CC2
SMC22.T.TACTTGTCAGCCACCA	CC1
SMC22.T.TACTTGTTCACGCGGT	CC1
SMC22.T.TAGACCACATACAGCT	CC1
SMC22.T.TAGACCAGTTACGTCA	CC2
SMC22.T.TAGACCATCGCGCCAA	CC1
SMC22.T.TAGAGCTCAAGTTCTG	CC2
SMC22.T.TAGAGCTGTAGCTCCG	CC1

SMC22.T.TAGAGCTTCCAGGGCT	CC2
SMC22.T.TAGCCGGAGCTAAGAT	CC1
SMC22.T.TAGCCGGCAGCCACCA	CC1
SMC22.T.TAGCCGGGTCTCAACA	CC1
SMC22.T.TAGCCGGGTGTCTGAT	CC1
SMC22.T.TAGCCGGTCCAGAAGG	CC1
SMC22.T.TAGGCATAGGTAGCTG	CC1
SMC22.T.TAGGCATAGTGCGTGA	CC1
SMC22.T.TAGGCATTCGGCTTGG	CC2
SMC22.T.TAGGCATTCTGCTGCT	CC1
SMC22.T.TAGTGGTAGGTAGCTG	CC1
SMC22.T.TAGTGGTGTACTCAAC	CC1
SMC22.T.TAGTGGTGTTGGTTTG	CC1
SMC22.T.TAGTGGTTCAGTTGAC	CC1
SMC22.T.TAGTTGGAGGATATAC	CC1
SMC22.T.TAGTTGGCACAGTCGC	CC1
SMC22.T.TAGTTGGTCGAATGCT	CC1
SMC22.T.TATCAGGAGCCACGTC	CC1
SMC22.T.TATCAGGAGCTAGTTC	CC1
SMC22.T.TATCAGGAGGTAGCTG	CC2
SMC22.T.TATCAGGCAGGATTGG	CC1
SMC22.T.TATCAGGGTAAGGGCT	CC1
SMC22.T.TATCAGGTCGAGAGCA	CC1
SMC22.T.TATCTCACAAGTTGTC	CC1
SMC22.T.TATCTCACAGCGATCC	CC2
SMC22.T.TATCTCATCAAAGACA	CC1
SMC22.T.TATCTCATCAACCAAC	CC2
SMC22.T.TATCTCATCCGATATG	CC1
SMC22.T.TATCTCATCCTTGGTC	CC1
SMC22.T.TATGCCCAGTTGCAGG	CC1
SMC22.T.TATGCCCTCCGCATAA	CC1
SMC22.T.TATTACCCAGCTGTTA	CC1
SMC22.T.TATTACCGTCGCGAAA	CC1
SMC22.T.TATTACCTCGGACAAG	CC1
SMC22.T.TCAACGAAGACTCGGA	CC1
SMC22.T.TCAACGACAATAGAGT	CC1
SMC22.T.TCAACGACAGCGTAAG	CC1
SMC22.T.TCAACGAGTAGCGATG	CC1
SMC22.T.TCAACGAGTCCTCTTG	CC1
SMC22.T.TCAACGATCGGTGTCG	CC1
SMC22.T.TCAATCTAGAAGAAGC	CC1
SMC22.T.TCAATCTGTCGGCACT	CC2
SMC22.T.TCAATCTGTCGTGGCT	CC1
SMC22.T.TCAATCTGTTGCGCAC	CC1
SMC22.T.TCAATCTTCCGTAGTA	CC2
SMC22.T.TCAATCTTCTGCGTAA	CC1
SMC22.T.TCACAAGAGGCTAGAC	CC1
SMC22.T.TCACAAGGTAGCGTGA	CC1
SMC22.T.TCACAAGTCACATACG	CC2
SMC22.T.TCACGAAAGGATCGCA	CC1
SMC22.T.TCACGAACAATGTAAG	CC1
SMC22.T.TCAGATGAGACAAGCC	CC2
SMC22.T.TCAGATGCATTGGGCC	CC1
SMC22.T.TCAGATGGTAGCAAAT	CC1
SMC22.T.TCAGATGGTTGCCTCT	CC1
SMC22.T.TCAGATGTCTCAAGTG	CC2
SMC22.T.TCAGCAAAGTATCTCG	CC1
SMC22.T.TCAGCAACATGTCGAT	CC1

SMC22.T.TCAGCAAGTGGGTATG	CC1
SMC22.T.TCAGCTCAGCTGTCTA	CC1
SMC22.T.TCAGCTCCACCTCGTT	CC1
SMC22.T.TCAGCTCCAGCAGTTT	CC1
SMC22.T.TCAGCTCCATACGCCG	CC1
SMC22.T.TCAGCTCGTCTTTCAT	CC1
SMC22.T.TCAGCTCGTGCGAAAC	CC1
SMC22.T.TCAGCTCGTTAAAGAC	CC2
SMC22.T.TCAGGATAGGTAAACT	CC1
SMC22.T.TCAGGATAGTCCGTAT	CC1
SMC22.T.TCAGGATAGTCTTGCA	CC1
SMC22.T.TCAGGATCACCAGGCT	CC1
SMC22.T.TCAGGATGTTAGTGGG	CC1
SMC22.T.TCAGGATGTTTCCACC	CC1
SMC22.T.TCAGGTACAGGATTGG	CC1
SMC22.T.TCAGGTACAGTTTACG	CC1
SMC22.T.TCAGGTAGTTCCGTCT	CC2
SMC22.T.TCAGGTATCGGATGGA	CC2
SMC22.T.TCATTACAGAGAGCTC	CC2
SMC22.T.TCATTACCAAATACAG	CC1
SMC22.T.TCATTACTCGATGAGG	CC1
SMC22.T.TCATTTGAGGGCTTGA	CC2
SMC22.T.TCATTTGCAAGCTGAG	CC2
SMC22.T.TCATTTGGTCCTGCTT	CC1
SMC22.T.TCATTTGGTGTTCTTT	CC1
SMC22.T.TCATTTGTCAGAGGTG	CC2
SMC22.T.TCCACACAGAAGATTC	CC1
SMC22.T.TCCACACAGCACCGTC	CC1
SMC22.T.TCCACACAGTGCGATG	CC1
SMC22.T.TCCACACCAAAGCGGT	CC1
SMC22.T.TCCACACGTATAGGTA	CC2
SMC22.T.TCCCGATAGATATGCA	CC1
SMC22.T.TCCCGATAGCAAATCA	CC2
SMC22.T.TCCCGATCAAGGTTTC	CC1
SMC22.T.TCCCGATGTATCGCAT	CC1
SMC22.T.TCCCGATGTATGAATG	CC1
SMC22.T.TCCCGATGTCTCACCT	CC1
SMC22.T.TCCCGATGTGCACGAA	CC1
SMC22.T.TCCCGATGTGGCAAAC	CC1
SMC22.T.TCCCGATTCACCACCT	CC1
SMC22.T.TCCCGATTCCAGTAGT	CC1
SMC22.T.TCCCGATTCTGCAAGT	CC1
SMC22.T.TCGAGGCAGGCTATCT	CC1
SMC22.T.TCGAGGCAGTCAAGCG	CC1
SMC22.T.TCGAGGCTCAGCAACT	CC2
SMC22.T.TCGCGAGAGAAGGCCT	CC1
SMC22.T.TCGCGAGAGTCCCACG	CC2
SMC22.T.TCGCGAGCAATAGCGG	CC1
SMC22.T.TCGCGAGCATAGACTC	CC2
SMC22.T.TCGCGAGTCATCATTC	CC1
SMC22.T.TCGCGAGTCCGTACAA	CC1
SMC22.T.TCGCGTTAGGAACTGC	CC1
SMC22.T.TCGCGTTGTTCGAATC	CC1
SMC22.T.TCGCGTTTCCCAAGAT	CC1
SMC22.T.TCGCGTTTCCGCATAA	CC1
SMC22.T.TCGCGTTTCCTTAATC	CC1
SMC22.T.TCGGGACAGACTCGGA	CC1
SMC22.T.TCGGGACAGCAGACTG	CC1

SMC22.T.TCGGGACAGCCGTCGT	CC1
SMC22.T.TCGGGACTCAGGCCCA	CC1
SMC22.T.TCGGGACTCCAGAGGA	CC1
SMC22.T.TCGGTAAAGTGGGCTA	CC1
SMC22.T.TCGGTAAAGTTCGCGC	CC1
SMC22.T.TCGGTAAGTCACTTCC	CC1
SMC22.T.TCGTACCAGGCACATG	CC1
SMC22.T.TCGTACCCACTAAGTC	CC1
SMC22.T.TCGTACCCAGGCAGTA	CC1
SMC22.T.TCGTACCTCCTCAACC	CC1
SMC22.T.TCGTAGAAGCCATCGC	CC2
SMC22.T.TCGTAGACAGGTTTCA	CC2
SMC22.T.TCGTAGACATGGATGG	CC1
SMC22.T.TCGTAGATCAGAGCTT	CC2
SMC22.T.TCTATTGAGTACTTGC	CC1
SMC22.T.TCTATTGGTCACTGGC	CC2
SMC22.T.TCTATTGGTCATGCCG	CC1
SMC22.T.TCTATTGTCGGCATCG	CC1
SMC22.T.TCTATTGTCTCATTCA	CC1
SMC22.T.TCTCATAGTAGAGTGC	CC1
SMC22.T.TCTCATATCATCACCC	CC1
SMC22.T.TCTCTAAAGACGCTTT	CC2
SMC22.T.TCTCTAAAGACTTGAA	CC2
SMC22.T.TCTCTAAAGCTAGCCC	CC1
SMC22.T.TCTCTAACATGTAGTC	CC1
SMC22.T.TCTCTAAGTCCGAACC	CC1
SMC22.T.TCTCTAATCCAGAAGG	CC2
SMC22.T.TCTCTAATCTCGCATC	CC1
SMC22.T.TCTCTAATCTGTCAAG	CC1
SMC22.T.TCTGAGAAGCTAGTTC	CC1
SMC22.T.TCTGAGAAGTCATCCA	CC1
SMC22.T.TCTGAGAGTATTACCG	CC1
SMC22.T.TCTGAGATCACAGGCC	CC1
SMC22.T.TCTGAGATCACCATAG	CC1
SMC22.T.TCTGAGATCTTTCCTC	CC1
SMC22.T.TCTGGAACAACACCTA	CC1
SMC22.T.TCTGGAACAGTATCTG	CC2
SMC22.T.TCTGGAAGTTTGTTTC	CC1
SMC22.T.TCTGGAATCGGTGTTA	CC1
SMC22.T.TCTTCGGTCCGTCAAA	CC2
SMC22.T.TCTTTCCAGCTTTGGT	CC1
SMC22.T.TCTTTCCCATATACGC	CC1
SMC22.T.TCTTTCCCATCCGCGA	CC1
SMC22.T.TCTTTCCGTAAAGTCA	CC1
SMC22.T.TGAAAGAAGCGCCTTG	CC1
SMC22.T.TGAAAGAAGGATGTAT	CC1
SMC22.T.TGAAAGAGTGAGTATA	CC1
SMC22.T.TGAAAGAGTGGACGAT	CC1
SMC22.T.TGAAAGATCACCGGGT	CC1
SMC22.T.TGACAACGTCTAAACC	CC1
SMC22.T.TGACAACTCGCCAAAT	CC1
SMC22.T.TGACAACTCGTGACAT	CC1
SMC22.T.TGACAACTCTCCAGGG	CC1
SMC22.T.TGACGGCAGCCCAGCT	CC1
SMC22.T.TGACGGCCAAGCCGCT	CC2
SMC22.T.TGACGGCCAGCGTAAG	CC2
SMC22.T.TGACGGCGTATATGAG	CC2
SMC22.T.TGACGGCGTCGCGTGT	CC2

SMC22.T.TGACGGCTCCACGTGG	CC1
SMC22.T.TGACGGCTCTATCCCG	CC1
SMC22.T.TGACTAGAGAAGAAGC	CC1
SMC22.T.TGACTAGCACATGGGA	CC1
SMC22.T.TGACTAGGTACATCCA	CC2
SMC22.T.TGACTAGGTAGCACGA	CC1
SMC22.T.TGACTAGGTTCATGGT	CC2
SMC22.T.TGACTAGGTTCGCGAC	CC1
SMC22.T.TGACTTTCAAAGGTGC	CC1
SMC22.T.TGACTTTCACTTAAGC	CC1
SMC22.T.TGACTTTGTCCGCTGA	CC1
SMC22.T.TGACTTTTCATCGATG	CC2
SMC22.T.TGAGAGGAGCCGGTAA	CC1
SMC22.T.TGAGAGGAGGAATCGC	CC2
SMC22.T.TGAGAGGAGTACGCGA	CC1
SMC22.T.TGAGAGGCATCGGACC	CC2
SMC22.T.TGAGAGGGTCTAGAGG	CC2
SMC22.T.TGAGAGGTCACAGTAC	CC1
SMC22.T.TGAGCATAGTGTGAAT	CC1
SMC22.T.TGAGCATGTATTCTCT	CC1
SMC22.T.TGAGCATGTTCAGACT	CC1
SMC22.T.TGAGCCGCATCTGGTA	CC2
SMC22.T.TGAGCCGCATTCTCAT	CC1
SMC22.T.TGAGCCGGTTCCGTCT	CC1
SMC22.T.TGAGCCGGTTCTGAAC	CC1
SMC22.T.TGAGCCGGTTCTGGTA	CC1
SMC22.T.TGAGCCGTCCGTCAAA	CC1
SMC22.T.TGAGCCGTCGTACGGC	CC1
SMC22.T.TGAGGGAAGCTGCAAG	CC1
SMC22.T.TGAGGGACACTCAGGC	CC1
SMC22.T.TGAGGGACAGCTTCGG	CC1
SMC22.T.TGAGGGACATCTATGG	CC1
SMC22.T.TGAGGGAGTCGTGGCT	CC1
SMC22.T.TGAGGGATCCAAATGC	CC2
SMC22.T.TGAGGGATCCCTTGTG	CC1
SMC22.T.TGAGGGATCTGGTATG	CC1
SMC22.T.TGATTTCAGCCACTAT	CC2
SMC22.T.TGATTTCAGCGTCTAT	CC2
SMC22.T.TGATTTCCACGAAAGC	CC1
SMC22.T.TGATTTCTCACCGGGT	CC1
SMC22.T.TGATTTCTCCGTAGGC	CC2
SMC22.T.TGCACCTAGATAGGAG	CC1
SMC22.T.TGCACCTAGGACACCA	CC1
SMC22.T.TGCACCTAGTGAACGC	CC1
SMC22.T.TGCACCTCAATCCAAC	CC1
SMC22.T.TGCACCTCACCAGCAC	CC1
SMC22.T.TGCACCTGTCTGATTG	CC1
SMC22.T.TGCACCTTCACCTTAT	CC2
SMC22.T.TGCACCTTCTGATACG	CC1
SMC22.T.TGCCAAACATTCCTGC	CC1
SMC22.T.TGCCAAAGTCCCTTGT	CC1
SMC22.T.TGCCAAAGTTAAGTAG	CC1
SMC22.T.TGCCAAATCCACGCAG	CC1
SMC22.T.TGCCAAATCGTTGACA	CC1
SMC22.T.TGCCCATCAACACCTA	CC2
SMC22.T.TGCCCATCACAAGACG	CC1
SMC22.T.TGCCCATCATACTCTT	CC1
SMC22.T.TGCCCATGTAACGTTC	CC2

SMC22.T.TGCCCATGTGCCTGTG	CC1
SMC22.T.TGCCCATGTTATGCGT	CC1
SMC22.T.TGCCCATTCCTCATTA	CC1
SMC22.T.TGCCCTAAGCAAATCA	CC1
SMC22.T.TGCCCTAAGCGGATCA	CC1
SMC22.T.TGCCCTACATGTCGAT	CC1
SMC22.T.TGCGCAGAGCCGTCGT	CC2
SMC22.T.TGCGCAGAGCTAAGAT	CC2
SMC22.T.TGCGCAGGTACGCACC	CC2
SMC22.T.TGCGGGTAGTACATGA	CC1
SMC22.T.TGCGGGTGTATCACCA	CC1
SMC22.T.TGCGTGGCAACGATCT	CC1
SMC22.T.TGCGTGGCACTCGACG	CC1
SMC22.T.TGCGTGGGTTCAACCA	CC1
SMC22.T.TGCGTGGTCAGGTTCA	CC2
SMC22.T.TGCGTGGTCTGGCGAC	CC1
SMC22.T.TGCTACCCACGGTTTA	CC1
SMC22.T.TGCTACCCAGATCGGA	CC1
SMC22.T.TGCTGCTCAAACTGTC	CC1
SMC22.T.TGCTGCTCAATCTACG	CC2
SMC22.T.TGCTGCTCAGCTTCGG	CC1
SMC22.T.TGCTGCTCATGGTAGG	CC1
SMC22.T.TGCTGCTGTAAGAGGA	CC1
SMC22.T.TGCTGCTTCGGATGTT	CC1
SMC22.T.TGGACGCAGATCCGAG	CC1
SMC22.T.TGGACGCGTACAGTGG	CC1
SMC22.T.TGGACGCTCAAGCCTA	CC1
SMC22.T.TGGACGCTCACCTTAT	CC1
SMC22.T.TGGACGCTCTGGAGCC	CC1
SMC22.T.TGGCCAGCAGACAAAT	CC2
SMC22.T.TGGCCAGCAGACACTT	CC1
SMC22.T.TGGCCAGCATTGTGCA	CC1
SMC22.T.TGGCCAGGTAGTAGTA	CC1
SMC22.T.TGGCGCACACACGCTG	CC2
SMC22.T.TGGCGCATCGCACTCT	CC1
SMC22.T.TGGCTGGAGTTCGATC	CC2
SMC22.T.TGGCTGGGTCTCACCT	CC2
SMC22.T.TGGGCGTCATTGCGGC	CC1
SMC22.T.TGGTTAGAGTGCGTGA	CC1
SMC22.T.TGGTTAGTCAGTTTGG	CC1
SMC22.T.TGGTTCCAGTTAGGTA	CC1
SMC22.T.TGGTTCCCACGTAAGG	CC1
SMC22.T.TGGTTCCGTAGGACAC	CC1
SMC22.T.TGGTTCCGTCGAAAGC	CC1
SMC22.T.TGTATTCCATGCCACG	CC1
SMC22.T.TGTATTCGTCTGGAGA	CC1
SMC22.T.TGTATTCTCGCAAGCC	CC2
SMC22.T.TGTCCCAAGCGATTCT	CC1
SMC22.T.TGTCCCAAGGATCGCA	CC1
SMC22.T.TGTCCCACACACAGAG	CC1
SMC22.T.TGTCCCACAGTCGTGC	CC1
SMC22.T.TGTCCCAGTCATATGC	CC1
SMC22.T.TGTCCCAGTTAAGAAC	CC1
SMC22.T.TGTGGTAGTGATGTCT	CC2
SMC22.T.TGTGGTATCTCACATT	CC1
SMC22.T.TGTGTTTTCCACGTGG	CC2
SMC22.T.TGTTCCGTCCTTAATC	CC1
SMC22.T.TGTTCCGTCGCTAGCG	CC1

SMC22.T.TGTTCCGTCGGTGTCG	CC1
SMC22.T.TTAACTCCAATCGGTT	CC1
SMC22.T.TTAACTCCAGCATGAG	CC1
SMC22.T.TTAACTCCATGTCTCC	CC1
SMC22.T.TTAACTCGTTGAGTTC	CC1
SMC22.T.TTAGGACAGTGAATTG	CC1
SMC22.T.TTAGGACTCAGCGACC	CC2
SMC22.T.TTAGGACTCCTGTACC	CC1
SMC22.T.TTAGGCAAGCAGATCG	CC1
SMC22.T.TTAGGCAGTGATGTGG	CC1
SMC22.T.TTAGGCAGTGCAGTAG	CC1
SMC22.T.TTAGGCATCTGTCAAG	CC1
SMC22.T.TTAGTTCAGCCTTGAT	CC1
SMC22.T.TTAGTTCCATTCTTAC	CC1
SMC22.T.TTAGTTCTCATTTGGG	CC1
SMC22.T.TTAGTTCTCCACTGGG	CC1
SMC22.T.TTAGTTCTCCCTCAGT	CC1
SMC22.T.TTAGTTCTCGTTGACA	CC2
SMC22.T.TTATGCTAGAGACTTA	CC2
SMC22.T.TTCCCAGCAACGCACC	CC2
SMC22.T.TTCCCAGGTAAACGCG	CC1
SMC22.T.TTCCCAGGTCATCGGC	CC1
SMC22.T.TTCCCAGGTCGCGTGT	CC1
SMC22.T.TTCGAAGAGATGTAAC	CC2
SMC22.T.TTCGAAGGTGATAAAC	CC1
SMC22.T.TTCGAAGGTTCCAACA	CC2
SMC22.T.TTCGGTCAGATGTGTA	CC2
SMC22.T.TTCGGTCGTGGTACAG	CC1
SMC22.T.TTCTACAAGGGTGTGT	CC1
SMC22.T.TTCTACAGTTATCGGT	CC1
SMC22.T.TTCTCAAAGAACAACT	CC1
SMC22.T.TTCTCAAAGACAAGCC	CC1
SMC22.T.TTCTCAATCTTCCTTC	CC1
SMC22.T.TTCTCCTAGAAACGAG	CC1
SMC22.T.TTCTCCTAGCTGGAAC	CC1
SMC22.T.TTCTCCTGTCAAGCGA	CC1
SMC22.T.TTCTCCTTCTGTGCAA	CC2
SMC22.T.TTCTTAGAGACAGAGA	CC1
SMC22.T.TTCTTAGAGACCTTTG	CC1
SMC22.T.TTCTTAGCAATAAGCA	CC2
SMC22.T.TTCTTAGGTAAACACA	CC1
SMC22.T.TTCTTAGTCTTTACAC	CC1
SMC22.T.TTGAACGCATCAGTCA	CC1
SMC22.T.TTGAACGGTCATTAGC	CC2
SMC22.T.TTGAACGTCCCAAGAT	CC1
SMC22.T.TTGAACGTCGAATGGG	CC2
SMC22.T.TTGAACGTCTTGTTTG	CC1
SMC22.T.TTGACTTAGCTAGGCA	CC2
SMC22.T.TTGACTTAGGACCACA	CC1
SMC22.T.TTGCCGTCACCAGTTA	CC2
SMC22.T.TTGCCGTCATAACCTG	CC2
SMC22.T.TTGCGTCAGGCTCAGA	CC2
SMC22.T.TTGCGTCCATGTTCCC	CC1
SMC22.T.TTGGAACCAAACGCGA	CC2
SMC22.T.TTGGAACCAGTCACTA	CC2
SMC22.T.TTGGCAAAGACATAAC	CC1
SMC22.T.TTGGCAAAGCTTTGGT	CC2
SMC22.T.TTGGCAAAGGTCGGAT	CC1

SMC22.T.TTGGCAAGTGTGGTTT	CC1
SMC22.T.TTGGCAAGTTTAGCTG	CC1
SMC22.T.TTGGCAATCAGCACAT	CC1
SMC22.T.TTGGCAATCTAGCACA	CC2
SMC22.T.TTGTAGGAGCTGTCTA	CC1
SMC22.T.TTGTAGGGTTACTGAC	CC1
SMC22.T.TTGTAGGTCGCCCTTA	CC1
SMC22.T.TTTACTGGTCGTCTTC	CC2
SMC22.T.TTTACTGTCCCAAGTA	CC1
SMC22.T.TTTACTGTCTGTGCAA	CC1
SMC22.T.TTTATGCAGCAGCCTC	CC1
SMC22.T.TTTATGCCACCAGCAC	CC1
SMC22.T.TTTATGCCATTGGGCC	CC1
SMC22.T.TTTATGCGTCGACTGC	CC1
SMC22.T.TTTATGCGTTTCGCTC	CC1
SMC22.T.TTTATGCTCCCACTTG	CC1
SMC22.T.TTTATGCTCCTGTACC	CC1
SMC22.T.TTTATGCTCGTCCGTT	CC2
SMC22.T.TTTCCTCAGGCGACAT	CC2
SMC22.T.TTTCCTCGTGTGGCTC	CC1
SMC22.T.TTTCCTCTCACAATGC	CC1
SMC22.T.TTTGCGCAGAGAACAG	CC1
SMC22.T.TTTGCGCAGATCTGAA	CC1
SMC22.T.TTTGCGCCATCGGGTC	CC1
SMC22.T.TTTGCGCGTCATTAGC	CC1
SMC22.T.TTTGCGCGTTACGGAG	CC1
SMC22.T.TTTGCGCGTTGGGACA	CC1
SMC22.T.TTTGGTTAGACAGGCT	CC1
SMC22.T.TTTGGTTAGTACTTGC	CC1
SMC22.T.TTTGGTTCAACACCCG	CC1
SMC22.T.TTTGGTTTCTGTGCAA	CC1
SMC22.T.TTTGGTTTCTTGCCGT	CC1
SMC22.T.TTTGTCATCCTCAATT	CC1
SMC23.T.AAACCTGTCTTAGCCC	CC1
SMC23.T.AAACGGGCATTCCTGC	CC1
SMC23.T.AAACGGGGTAGGAGTC	CC1
SMC23.T.AAAGATGCACCAGGCT	CC1
SMC23.T.AAAGCAAAGATAGCAT	CC1
SMC23.T.AAAGCAACATGATCCA	CC1
SMC23.T.AAAGCAAGTCCGCTGA	CC1
SMC23.T.AAATGCCCAGGAATCG	CC1
SMC23.T.AACACGTGTTAAGTAG	CC1
SMC23.T.AACCATGCAATCCGAT	CC1
SMC23.T.AACCGCGTCGCACTCT	CC1
SMC23.T.AACGTTGTCAGCACAT	CC1
SMC23.T.AACTCAGTCCGAAGAG	CC1
SMC23.T.AACTCTTCATGCAACT	CC2
SMC23.T.AACTCTTGTGAGTATA	CC1
SMC23.T.AAGGAGCAGAGGGCTT	CC1
SMC23.T.AAGGAGCCAAGGACAC	CC1
SMC23.T.AAGGAGCCACCACGTG	CC1
SMC23.T.AAGGAGCCATCCAACA	CC2
SMC23.T.AAGGAGCGTGTGCCTG	CC1
SMC23.T.AAGGCAGCACAGACTT	CC1
SMC23.T.AAGGTTCTCAAACCAC	CC1
SMC23.T.AAGTCTGCAGTCTTCC	CC1
SMC23.T.AAGTCTGCATACCATG	CC1
SMC23.T.AATCCAGAGACTAGGC	CC1

SMC23.T.AATCGGTAGGAGTAGA	CC2
SMC23.T.ACACCCTCAGGTCTCG	CC1
SMC23.T.ACACCCTTCATCATTC	CC1
SMC23.T.ACACTGAAGTGCGTGA	CC1
SMC23.T.ACAGCTATCCGAGCCA	CC1
SMC23.T.ACATACGGTCAACATC	CC1
SMC23.T.ACATGGTCAAAGCAAT	CC1
SMC23.T.ACATGGTTCGGAGGTA	CC1
SMC23.T.ACCAGTACACGGACAA	CC1
SMC23.T.ACCAGTAGTTTAGGAA	CC1
SMC23.T.ACCCACTAGGTCATCT	CC1
SMC23.T.ACCCACTCATAAGACA	CC1
SMC23.T.ACCCACTTCGATGAGG	CC1
SMC23.T.ACCGTAAAGCAATATG	CC1
SMC23.T.ACCGTAACAGGTCGTC	CC1
SMC23.T.ACGAGCCCACTGTTAG	CC1
SMC23.T.ACGAGCCCATATGAGA	CC1
SMC23.T.ACGAGGAGTACGCTGC	CC2
SMC23.T.ACGAGGAGTGATGTGG	CC1
SMC23.T.ACGAGGATCCGGGTGT	CC1
SMC23.T.ACGAGGATCTACCAGA	CC1
SMC23.T.ACGATACAGCCAACAG	CC1
SMC23.T.ACGATACTCCGCAGTG	CC1
SMC23.T.ACGATGTAGAACAACT	CC1
SMC23.T.ACGCAGCAGCTGAAAT	CC1
SMC23.T.ACGCAGCCACAGGAGT	CC1
SMC23.T.ACGCCGAAGTCCCACG	CC1
SMC23.T.ACGCCGATCCATTCTA	CC1
SMC23.T.ACGCCGATCTCCTATA	CC1
SMC23.T.ACGGAGAGTGTGGCTC	CC1
SMC23.T.ACGGAGATCGGCGCTA	CC1
SMC23.T.ACGGCCACAGCCTTGG	CC1
SMC23.T.ACGGGTCAGGGAAACA	CC1
SMC23.T.ACGGGTCGTTGTCGCG	CC1
SMC23.T.ACGGGTCTCCTCAATT	CC1
SMC23.T.ACTGAACGTGCAGTAG	CC1
SMC23.T.ACTGAGTAGGCGATAC	CC1
SMC23.T.ACTGAGTAGTACCGGA	CC1
SMC23.T.ACTGATGCATTCCTGC	CC1
SMC23.T.ACTGATGGTAACGACG	CC1
SMC23.T.ACTGTCCCATAGAAAC	CC1
SMC23.T.ACTTACTCAATAGCGG	CC1
SMC23.T.ACTTACTGTCCAAGTT	CC1
SMC23.T.ACTTACTGTGGCGAAT	CC1
SMC23.T.ACTTACTTCTCGATGA	CC1
SMC23.T.ACTTTCACACCGTTGG	CC2
SMC23.T.AGAATAGTCTCAAGTG	CC1
SMC23.T.AGACGTTAGCGATAGC	CC1
SMC23.T.AGACGTTAGGGATGGG	CC1
SMC23.T.AGACGTTCAAGTCTAC	CC1
SMC23.T.AGACGTTCAATCGGTT	CC1
SMC23.T.AGAGCGAAGTGGTCCC	CC1
SMC23.T.AGAGCGACAATAGCAA	CC1
SMC23.T.AGAGCGATCACTATTC	CC2
SMC23.T.AGAGCGATCTACCAGA	CC1
SMC23.T.AGAGCTTGTTTGACAC	CC1
SMC23.T.AGAGTGGTCGCCAAAT	CC1
SMC23.T.AGAGTGGTCTACCTGC	CC2

SMC23.T.AGATTGCCAAGCCCAC	CC1
SMC23.T.AGATTGCCACTGAAGG	CC1
SMC23.T.AGCAGCCAGACTGGGT	CC1
SMC23.T.AGCATACGTCCTGCTT	CC1
SMC23.T.AGCCTAAGTCCGACGT	CC1
SMC23.T.AGGCCACCAATGTTGC	CC1
SMC23.T.AGGCCACTCTGTCTCG	CC1
SMC23.T.AGGCCGTCACCTGGTG	CC1
SMC23.T.AGGCCGTTCATTGCGA	CC2
SMC23.T.AGGGAGTTCGGCTACG	CC1
SMC23.T.AGGGATGCAGTGAGTG	CC1
SMC23.T.AGGGATGCATTAACCG	CC1
SMC23.T.AGGGATGGTACATCCA	CC1
SMC23.T.AGGGTGAAGCACCGTC	CC1
SMC23.T.AGGGTGACAGCCTATA	CC1
SMC23.T.AGGGTGATCAAACCGT	CC1
SMC23.T.AGGTCCGAGCACAGGT	CC1
SMC23.T.AGGTCCGCAGACAAGC	CC1
SMC23.T.AGGTCCGCATCGACGC	CC1
SMC23.T.AGGTCCGTCGCTAGCG	CC1
SMC23.T.AGTCTTTAGCTTCGCG	CC1
SMC23.T.AGTCTTTCATCACGAT	CC1
SMC23.T.AGTGAGGAGCAATCTC	CC1
SMC23.T.AGTGAGGGTGCAGTAG	CC1
SMC23.T.AGTGGGAGTAAGTGTA	CC1
SMC23.T.AGTGTCAAGTTCGCGC	CC1
SMC23.T.AGTGTCATCTTAGAGC	CC1
SMC23.T.AGTTGGTTCCGTCAAA	CC1
SMC23.T.ATAACGCTCGTTTAGG	CC1
SMC23.T.ATAAGAGGTCCGAAGA	CC1
SMC23.T.ATAAGAGTCAGTACGT	CC1
SMC23.T.ATCACGATCCCAAGAT	CC1
SMC23.T.ATCATCTGTTCCACAA	CC1
SMC23.T.ATCCACCAGACTAAGT	CC1
SMC23.T.ATCGAGTAGCCAGAAC	CC2
SMC23.T.ATCGAGTAGCGTTTAC	CC1
SMC23.T.ATCGAGTCAGCTGTGC	CC1
SMC23.T.ATCGAGTGTTATGCGT	CC2
SMC23.T.ATCGAGTTCGCCATAA	CC1
SMC23.T.ATCTACTGTGAGCGAT	CC1
SMC23.T.ATCTGCCAGGTGACCA	CC1
SMC23.T.ATCTGCCTCAGCTGGC	CC1
SMC23.T.ATCTGCCTCGCAAGCC	CC2
SMC23.T.ATGAGGGAGAGGGATA	CC1
SMC23.T.ATGTGTGCAAGCTGAG	CC1
SMC23.T.ATTACTCGTGTGACCC	CC2
SMC23.T.ATTATCCGTACCGGCT	CC1
SMC23.T.ATTCTACGTAGCCTCG	CC2
SMC23.T.ATTCTACTCCAAACTG	CC1
SMC23.T.ATTGGACAGTCTCAAC	CC1
SMC23.T.ATTGGACCACTTAAGC	CC1
SMC23.T.ATTGGACCATGGTCTA	CC1
SMC23.T.ATTTCTGAGACAGGCT	CC1
SMC23.T.ATTTCTGGTTGGAGGT	CC2
SMC23.T.ATTTCTGGTTTAAGCC	CC1
SMC23.T.ATTTCTGTCACGCGGT	CC2
SMC23.T.CAACCAACATGCCTAA	CC1
SMC23.T.CAACCAAGTCCGAACC	CC1

SMC23.T.CAACCAATCAGCCTAA	CC1
SMC23.T.CAACCTCAGAGTCTGG	CC1
SMC23.T.CAACCTCCACGTCAGC	CC1
SMC23.T.CAACTAGAGCTAACAA	CC2
SMC23.T.CAAGAAAGTCTTCTCG	CC1
SMC23.T.CAAGAAATCCCATTTA	CC1
SMC23.T.CAAGATCCATTACCTT	CC1
SMC23.T.CAAGTTGGTAAGGATT	CC1
SMC23.T.CACAAACAGCCTCGTG	CC1
SMC23.T.CACAAACCAGGCTGAA	CC1
SMC23.T.CACAAACGTACGAAAT	CC1
SMC23.T.CACAAACGTCTGGAGA	CC1
SMC23.T.CACACCTCATTCCTCG	CC1
SMC23.T.CACACCTTCATAGCAC	CC1
SMC23.T.CACACTCCACCCATTC	CC1
SMC23.T.CACACTCGTAATCACC	CC1
SMC23.T.CACACTCTCTCAACTT	CC2
SMC23.T.CACACTCTCTGCTTGC	CC1
SMC23.T.CACAGGCTCCGTTGTC	CC2
SMC23.T.CACAGTATCGGTCTAA	CC2
SMC23.T.CACATAGAGCAGATCG	CC1
SMC23.T.CACATTTAGCGATATA	CC1
SMC23.T.CACATTTGTAAATACG	CC1
SMC23.T.CACCACTCAAATCCGT	CC1
SMC23.T.CACCACTTCGGCGCTA	CC1
SMC23.T.CACTCCAAGGCTCAGA	CC1
SMC23.T.CAGAGAGCATCAGTAC	CC1
SMC23.T.CAGAGAGTCATAGCAC	CC1
SMC23.T.CAGATCAGTAAAGGAG	CC1
SMC23.T.CAGCATAAGGATCGCA	CC2
SMC23.T.CAGCCGAGTCGCGGTT	CC1
SMC23.T.CAGCTAACATAGGATA	CC1
SMC23.T.CAGCTAAGTCCAGTAT	CC1
SMC23.T.CAGCTGGTCTCGTATT	CC1
SMC23.T.CAGTAACAGAAAGTGG	CC1
SMC23.T.CAGTCCTTCTCAACTT	CC2
SMC23.T.CATATTCTCTGATTCT	CC1
SMC23.T.CATCAAGTCGCCTGAG	CC1
SMC23.T.CATCAGAAGTCACGCC	CC1
SMC23.T.CATCAGATCCCAAGAT	CC1
SMC23.T.CATCAGATCCGTCATC	CC1
SMC23.T.CATCAGATCCTTCAAT	CC1
SMC23.T.CATCAGATCTTGTACT	CC2
SMC23.T.CATCCACAGTGACTCT	CC1
SMC23.T.CATCGAAAGCGGCTTC	CC1
SMC23.T.CATCGAAGTATCGCAT	CC1
SMC23.T.CATCGGGAGCGATAGC	CC1
SMC23.T.CATCGGGCAGTCTTCC	CC1
SMC23.T.CATGACACATGAGCGA	CC1
SMC23.T.CATGACAGTTATGCGT	CC1
SMC23.T.CATGACATCTTCGAGA	CC1
SMC23.T.CATGCCTAGGTGACCA	CC1
SMC23.T.CATGCCTCATTTCAGG	CC1
SMC23.T.CATGCCTGTCTCTCGT	CC1
SMC23.T.CATGCCTGTTCGGCAC	CC1
SMC23.T.CATGCCTGTTTGTTTC	CC1
SMC23.T.CATGGCGTCTCGCATC	CC1
SMC23.T.CATTATCAGACCCACC	CC1

SMC23.T.CATTCGCTCTTGCCGT	CC1
SMC23.T.CCAATCCAGACTACAA	CC1
SMC23.T.CCAATCCCAGTTCATG	CC1
SMC23.T.CCACCTAAGAGCTGGT	CC1
SMC23.T.CCACCTAAGCTCCTTC	CC2
SMC23.T.CCATTCGAGAGTGACC	CC1
SMC23.T.CCATTCGGTCATGCCG	CC1
SMC23.T.CCCAATCCACCGTTGG	CC1
SMC23.T.CCCAGTTCAGCCAGAA	CC1
SMC23.T.CCCAGTTGTCAAAGCG	CC1
SMC23.T.CCCATACAGGCTACGA	CC1
SMC23.T.CCGGGATGTCTCAACA	CC1
SMC23.T.CCGGGATTCGGAGGTA	CC1
SMC23.T.CCGGTAGCAAGGACAC	CC1
SMC23.T.CCGGTAGCAGTAAGAT	CC2
SMC23.T.CCGTGGATCGTCTGAA	CC1
SMC23.T.CCGTTCAGTTATTCTC	CC1
SMC23.T.CCTAAAGCACTTGGAT	CC1
SMC23.T.CCTAAAGGTCTCTCGT	CC1
SMC23.T.CCTAAAGTCTCTTATG	CC1
SMC23.T.CCTACACTCACAATGC	CC1
SMC23.T.CCTACCAAGCTTCGCG	CC1
SMC23.T.CCTACCATCCTTGGTC	CC1
SMC23.T.CCTACCATCTGAGTGT	CC1
SMC23.T.CCTAGCTCACCCATTC	CC1
SMC23.T.CCTCAGTAGCAGCCTC	CC1
SMC23.T.CCTCAGTCAATGTTGC	CC1
SMC23.T.CCTTACGAGGGAGTAA	CC1
SMC23.T.CCTTCCCGTCACACGC	CC1
SMC23.T.CCTTCGAGTCTCTTTA	CC1
SMC23.T.CCTTCGAGTGCCTGTG	CC2
SMC23.T.CCTTCGATCTATGTGG	CC1
SMC23.T.CCTTCGATCTGCAGTA	CC1
SMC23.T.CCTTTCTAGTAAGTAC	CC1
SMC23.T.CGAACATTCATGCTCC	CC1
SMC23.T.CGAACATTCTTTACAC	CC1
SMC23.T.CGAATGTGTCGAATCT	CC1
SMC23.T.CGACCTTGTAGAAAGG	CC1
SMC23.T.CGACTTCGTAAACACA	CC1
SMC23.T.CGACTTCTCTGTTTGT	CC1
SMC23.T.CGAGAAGCACGCCAGT	CC1
SMC23.T.CGAGCACAGGCGACAT	CC1
SMC23.T.CGAGCACGTCTGGTCG	CC1
SMC23.T.CGAGCCAGTTTAGGAA	CC1
SMC23.T.CGATGTAAGAGTAAGG	CC1
SMC23.T.CGATGTAAGGTACTCT	CC1
SMC23.T.CGCCAAGGTAGGAGTC	CC1
SMC23.T.CGCCAAGGTCGGGTCT	CC1
SMC23.T.CGCGGTAGTTCTGGTA	CC1
SMC23.T.CGCGTTTCACACCGAC	CC1
SMC23.T.CGCGTTTGTAGCTCCG	CC1
SMC23.T.CGCGTTTTCGGCATCG	CC2
SMC23.T.CGCTGGAAGTCCCACG	CC1
SMC23.T.CGCTGGACACGGCCAT	CC1
SMC23.T.CGCTGGATCAACTCTT	CC1
SMC23.T.CGCTTCACATATACGC	CC1
SMC23.T.CGCTTCAGTCTCCATC	CC1
SMC23.T.CGCTTCATCTGTGCAA	CC1

SMC23.T.CGGACGTAGGAGTAGA	CC2
SMC23.T.CGGACGTTCCCAAGAT	CC1
SMC23.T.CGGACTGAGTGTGGCA	CC1
SMC23.T.CGGAGTCGTTCAGCGC	CC2
SMC23.T.CGGAGTCTCGGCCGAT	CC1
SMC23.T.CGGCTAGCATACCATG	CC1
SMC23.T.CGGCTAGGTTCTGAAC	CC1
SMC23.T.CGGGTCAAGAGAACAG	CC1
SMC23.T.CGGGTCAGTCCTGCTT	CC1
SMC23.T.CGGTTAATCGGAATCT	CC1
SMC23.T.CGGTTAATCTGTCAAG	CC2
SMC23.T.CGTAGCGGTCTAAACC	CC1
SMC23.T.CGTCAGGAGTGAAGAG	CC1
SMC23.T.CGTCAGGTCAAACAAG	CC1
SMC23.T.CGTCAGGTCCGAATGT	CC1
SMC23.T.CGTCCATCATGCTAGT	CC1
SMC23.T.CGTCCATTCCTATGTT	CC1
SMC23.T.CGTCTACAGCTGCCCA	CC1
SMC23.T.CGTCTACAGTCCGGTC	CC2
SMC23.T.CGTCTACCATCCGGGT	CC1
SMC23.T.CGTGAGCAGCCGGTAA	CC1
SMC23.T.CGTGAGCAGTTACCCA	CC1
SMC23.T.CGTGAGCCAGGGTTAG	CC1
SMC23.T.CGTGTAAAGGCGCTCT	CC2
SMC23.T.CGTGTCTAGAATAGGG	CC1
SMC23.T.CGTGTCTAGCGTAGTG	CC1
SMC23.T.CGTGTCTCAAGGTTTC	CC1
SMC23.T.CGTGTCTGTTCCGTCT	CC1
SMC23.T.CGTTAGATCGAGAGCA	CC2
SMC23.T.CGTTCTGGTTGAGGTG	CC1
SMC23.T.CGTTGGGTCCATTCTA	CC1
SMC23.T.CTAACTTTCCACTGGG	CC1
SMC23.T.CTAATGGCACGCCAGT	CC1
SMC23.T.CTACACCAGCTTATCG	CC1
SMC23.T.CTACACCCATCCGGGT	CC1
SMC23.T.CTACACCCATGTCCTC	CC1
SMC23.T.CTACACCTCATCGATG	CC1
SMC23.T.CTACATTTCTGCCCTA	CC1
SMC23.T.CTACCCAGTCCAGTGC	CC1
SMC23.T.CTACCCAGTGCACGAA	CC2
SMC23.T.CTAGAGTGTCGCCATG	CC2
SMC23.T.CTCACACCAGGTCGTC	CC1
SMC23.T.CTCAGAACATCGGAAG	CC1
SMC23.T.CTCCTAGCAGGCTCAC	CC1
SMC23.T.CTCCTAGTCCTAAGTG	CC1
SMC23.T.CTCGAAACAGCGAACA	CC1
SMC23.T.CTCGAAAGTTGTGGCC	CC1
SMC23.T.CTCGAGGCAAGTAGTA	CC1
SMC23.T.CTCGGAGGTCATATCG	CC1
SMC23.T.CTCGGGAAGTGAATTG	CC1
SMC23.T.CTCGGGACACTGCCAG	CC1
SMC23.T.CTCGGGAGTTACCGAT	CC1
SMC23.T.CTCGTACGTTTCGCTC	CC1
SMC23.T.CTCGTACTCACCGTAA	CC1
SMC23.T.CTCGTACTCCACTCCA	CC1
SMC23.T.CTCGTACTCTGCAGTA	CC1
SMC23.T.CTCTAATTCAGTACGT	CC2
SMC23.T.CTCTAATTCCCAGGTG	CC1

SMC23.T.CTCTGGTAGGAGTTTA	CC1
SMC23.T.CTCTGGTCAACTGCGC	CC2
SMC23.T.CTCTGGTTCAGTTAGC	CC1
SMC23.T.CTGAAACAGCTGAAAT	CC1
SMC23.T.CTGAAACAGCTGCAAG	CC1
SMC23.T.CTGAAACCAGCCTGTG	CC1
SMC23.T.CTGAAACGTCGGGTCT	CC1
SMC23.T.CTGAAGTGTTCGCTAA	CC1
SMC23.T.CTGATAGAGGGATCTG	CC1
SMC23.T.CTGATCCTCGGCGCTA	CC2
SMC23.T.CTGCCTAAGATTACCC	CC2
SMC23.T.CTGCCTACATGATCCA	CC2
SMC23.T.CTGCCTATCCTACAGA	CC1
SMC23.T.CTGCTGTGTCTGATTG	CC1
SMC23.T.CTGCTGTGTGATGTGG	CC1
SMC23.T.CTGGTCTAGTAGCCGA	CC1
SMC23.T.CTGGTCTTCTCGTTTA	CC1
SMC23.T.CTGTGCTAGCACGCCT	CC1
SMC23.T.CTGTGCTGTACGAAAT	CC1
SMC23.T.CTTACCGGTTACGTCA	CC1
SMC23.T.CTTGGCTGTTTGCATG	CC2
SMC23.T.CTTTGCGGTCGGGTCT	CC1
SMC23.T.CTTTGCGTCTCCGGTT	CC1
SMC23.T.GAAATGACAGTTTACG	CC1
SMC23.T.GAACATCCAAGTAGTA	CC1
SMC23.T.GAACATCCAATAAGCA	CC1
SMC23.T.GAACATCTCTCCCTGA	CC1
SMC23.T.GAACATCTCTGACCTC	CC1
SMC23.T.GAACCTAAGTGTCCAT	CC2
SMC23.T.GAACGGACAGGGTACA	CC1
SMC23.T.GAACGGAGTCCATCCT	CC1
SMC23.T.GAACGGAGTTACGCGC	CC1
SMC23.T.GAAGCAGAGGTGACCA	CC1
SMC23.T.GAAGCAGGTAGTGAAT	CC1
SMC23.T.GAAGCAGTCCTTAATC	CC1
SMC23.T.GACAGAGAGAAGGCCT	CC1
SMC23.T.GACCAATAGAGCCCAA	CC1
SMC23.T.GACCAATAGCATCATC	CC1
SMC23.T.GACCTGGTCCAAGTAC	CC1
SMC23.T.GACGCGTGTTCCACTC	CC1
SMC23.T.GACGGCTAGACCTAGG	CC2
SMC23.T.GACGGCTCACCAGGTC	CC1
SMC23.T.GACGGCTTCTGTCTAT	CC1
SMC23.T.GACGTGCGTTCGAATC	CC1
SMC23.T.GACGTGCTCAGCTTAG	CC1
SMC23.T.GACGTTAAGTCTCCTC	CC1
SMC23.T.GACGTTAAGTGTTGAA	CC1
SMC23.T.GACTACAGTCTCTCTG	CC1
SMC23.T.GAGCAGACACCTCGTT	CC1
SMC23.T.GAGCAGATCCCTTGTG	CC1
SMC23.T.GAGGTGAAGATCCTGT	CC1
SMC23.T.GAGGTGATCGGTCTAA	CC1
SMC23.T.GAGTCCGAGCAGGCTA	CC1
SMC23.T.GAGTCCGAGTTTGCGT	CC1
SMC23.T.GAGTCCGGTCTGCCAG	CC1
SMC23.T.GAGTCCGTCAGTTGAC	CC1
SMC23.T.GATCGCGAGCTCCCAG	CC1
SMC23.T.GATGAAAAGTCAATAG	CC1

SMC23.T.GATGAGGCATGTCGAT	CC1
SMC23.T.GATGCTACAAACAACA	CC1
SMC23.T.GATTCAGAGACTAGGC	CC1
SMC23.T.GCAAACTCAGCTGCAC	CC1
SMC23.T.GCAATCATCGGATGTT	CC1
SMC23.T.GCACATACATACGCCG	CC1
SMC23.T.GCACATATCCAACCAA	CC1
SMC23.T.GCACATATCCACGCAG	CC1
SMC23.T.GCACATATCGCGATCG	CC1
SMC23.T.GCACTCTCAAATACAG	CC1
SMC23.T.GCACTCTGTTCTCATT	CC1
SMC23.T.GCACTCTTCTCAAGTG	CC1
SMC23.T.GCACTCTTCTGTGCAA	CC1
SMC23.T.GCAGCCAAGAATCTCC	CC1
SMC23.T.GCAGCCACACCTTGTC	CC1
SMC23.T.GCAGCCACAGCTCGAC	CC1
SMC23.T.GCATACACAATCAGAA	CC1
SMC23.T.GCATACAGTCGAATCT	CC1
SMC23.T.GCATGATCAATGGAAT	CC1
SMC23.T.GCATGATTCTTGAGAC	CC1
SMC23.T.GCATGCGTCAAGATCC	CC1
SMC23.T.GCCTCTAAGATGCCTT	CC1
SMC23.T.GCCTCTAAGGGCTTCC	CC1
SMC23.T.GCCTCTAGTCTCGTTC	CC1
SMC23.T.GCGAGAAGTCATGCAT	CC1
SMC23.T.GCGCCAAAGCCGTCGT	CC2
SMC23.T.GCGCGATTCTGACCTC	CC1
SMC23.T.GCTCCTATCACCCTCA	CC1
SMC23.T.GCTCTGTGTTAGGGTG	CC1
SMC23.T.GCTGCAGGTTGGAGGT	CC1
SMC23.T.GCTGCAGTCTACTATC	CC2
SMC23.T.GCTGCGAGTTGAACTC	CC1
SMC23.T.GCTGCTTTCTACGAGT	CC1
SMC23.T.GCTTCCACACGTGAGA	CC1
SMC23.T.GCTTCCACAGACAGGT	CC1
SMC23.T.GGAAAGCCAGGTTTCA	CC1
SMC23.T.GGAAAGCTCTCGCATC	CC2
SMC23.T.GGAACTTCAGTCCTTC	CC1
SMC23.T.GGAACTTGTCTAAACC	CC1
SMC23.T.GGAATAATCCTCTAGC	CC1
SMC23.T.GGACAAGAGGAGTAGA	CC1
SMC23.T.GGACAAGCAGCTTAAC	CC1
SMC23.T.GGACAGAGTAAGCACG	CC1
SMC23.T.GGACATTTCATAACCG	CC1
SMC23.T.GGACGTCAGCTGAAAT	CC1
SMC23.T.GGATGTTCACACCGCA	CC1
SMC23.T.GGATGTTGTAGAGCTG	CC1
SMC23.T.GGATGTTTCTTCGAGA	CC1
SMC23.T.GGATTACAGCCACGCT	CC1
SMC23.T.GGCAATTTCTTGTATC	CC1
SMC23.T.GGCCGATCAGGTGCCT	CC1
SMC23.T.GGCCGATGTGACTCAT	CC2
SMC23.T.GGCGACTAGGAGTTTA	CC1
SMC23.T.GGCGACTCAGTCAGAG	CC1
SMC23.T.GGCGACTCAGTTCCCT	CC1
SMC23.T.GGCGTGTAGTTAGGTA	CC1
SMC23.T.GGCTCGAGTTTGACTG	CC1
SMC23.T.GGCTGGTGTAAGGGAA	CC1

SMC23.T.GGGACCTCAATCGGTT	CC1
SMC23.T.GGGACCTCATACGCTA	CC1
SMC23.T.GGGAGATCAATGGAAT	CC1
SMC23.T.GGGTCTGTCTGGGCCA	CC1
SMC23.T.GGGTTGCGTTGGTAAA	CC1
SMC23.T.GGTATTGAGTACGACG	CC1
SMC23.T.GGTGAAGCATACTCTT	CC1
SMC23.T.GGTGAAGCATCACCCT	CC1
SMC23.T.GGTGCGTAGAATAGGG	CC1
SMC23.T.GGTGCGTAGATCTGCT	CC2
SMC23.T.GGTGCGTAGGACGAAA	CC1
SMC23.T.GGTGCGTAGTATTGGA	CC1
SMC23.T.GGTGCGTTCCCAACGG	CC1
SMC23.T.GGTGTTAGTGGCAAAC	CC1
SMC23.T.GTAACGTAGGGTGTTG	CC2
SMC23.T.GTAACGTCAAGCGAGT	CC1
SMC23.T.GTAACGTCACAGGCCT	CC1
SMC23.T.GTAACGTTCGGACAAG	CC1
SMC23.T.GTAACTGCAGTGGGAT	CC1
SMC23.T.GTACTCCCATCCCATC	CC1
SMC23.T.GTACTCCCATTAGGCT	CC1
SMC23.T.GTACTCCTCAACACTG	CC1
SMC23.T.GTATCTTCACGTCAGC	CC1
SMC23.T.GTATCTTGTTCCACTC	CC1
SMC23.T.GTCAAGTCACCTATCC	CC1
SMC23.T.GTCAAGTGTCCGTTAA	CC1
SMC23.T.GTCACAACAGACACTT	CC1
SMC23.T.GTCACAAGTCCCTTGT	CC1
SMC23.T.GTCACGGAGGTGCTAG	CC2
SMC23.T.GTCACGGTCGAGAACG	CC1
SMC23.T.GTCCTCAAGGCATTGG	CC1
SMC23.T.GTCCTCAGTGAAGGCT	CC1
SMC23.T.GTCGGGTCATTACCTT	CC1
SMC23.T.GTCTCGTTCAGCCTAA	CC1
SMC23.T.GTCTTCGGTCGTGGCT	CC1
SMC23.T.GTGAAGGAGGAATGGA	CC1
SMC23.T.GTGCAGCAGATGTAAC	CC2
SMC23.T.GTGCAGCCACCTCGTT	CC1
SMC23.T.GTGCATAAGAGAGCTC	CC1
SMC23.T.GTGCATACACATCCGG	CC1
SMC23.T.GTGCGGTAGAGATGAG	CC1
SMC23.T.GTGCTTCCAGTAACGG	CC1
SMC23.T.GTGCTTCCATTCGACA	CC1
SMC23.T.GTGCTTCTCCCAACGG	CC1
SMC23.T.GTGGGTCTCCAAACAC	CC2
SMC23.T.GTGTGCGGTTCGAATC	CC1
SMC23.T.GTTAAGCCATGAAGTA	CC1
SMC23.T.GTTACAGTCCGTCATC	CC1
SMC23.T.GTTCATTGTCCGAGTC	CC1
SMC23.T.GTTCTCGAGGCACATG	CC1
SMC23.T.GTTTCTATCAGTGTTG	CC1
SMC23.T.TAAACCGGTCACCTAA	CC1
SMC23.T.TAAGAGACAAGCCCAC	CC1
SMC23.T.TAAGAGACAGATGAGC	CC1
SMC23.T.TAAGAGACATTCCTGC	CC1
SMC23.T.TAAGTGCAGAAGGACA	CC1
SMC23.T.TAAGTGCAGCTCAACT	CC2
SMC23.T.TACACGAAGTACACCT	CC1

SMC23.T.TACACGAGTTAGGGTG	CC1
SMC23.T.TACACGATCTTGACGA	CC1
SMC23.T.TACAGTGAGCGTGAGT	CC1
SMC23.T.TACAGTGCAATCCAAC	CC1
SMC23.T.TACCTATCATCACAAC	CC1
SMC23.T.TACGGGCGTGTTGGGA	CC1
SMC23.T.TACGGTAAGGGTATCG	CC1
SMC23.T.TACGGTAGTCCCGACA	CC1
SMC23.T.TACGGTAGTGCGATAG	CC1
SMC23.T.TACTCATCAGTCACTA	CC1
SMC23.T.TACTCATGTACTTCTT	CC1
SMC23.T.TACTTACTCATCACCC	CC1
SMC23.T.TAGAGCTCAGTTAACC	CC1
SMC23.T.TAGCCGGAGACTCGGA	CC1
SMC23.T.TAGGCATAGATGCGAC	CC1
SMC23.T.TAGGCATAGATGTGTA	CC1
SMC23.T.TAGTGGTCAATGTAAG	CC1
SMC23.T.TAGTGGTGTCCTCTTG	CC1
SMC23.T.TAGTTGGCAGATGAGC	CC1
SMC23.T.TAGTTGGCATATACGC	CC1
SMC23.T.TAGTTGGTCGATAGAA	CC1
SMC23.T.TATCAGGAGAAGGTTT	CC1
SMC23.T.TATCAGGCAGGGTACA	CC1
SMC23.T.TATCTCATCCAGTATG	CC1
SMC23.T.TATCTCATCCCATTAT	CC1
SMC23.T.TATGCCCGTCGTTGTA	CC1
SMC23.T.TATGCCCTCCTCAATT	CC1
SMC23.T.TATTACCAGTATCTCG	CC1
SMC23.T.TCAACGAAGCAGACTG	CC1
SMC23.T.TCAACGATCGGTTCGG	CC1
SMC23.T.TCAATCTCAGATCGGA	CC1
SMC23.T.TCACAAGAGGTGCTTT	CC2
SMC23.T.TCACAAGTCTTTACAC	CC1
SMC23.T.TCACGAAGTAAGTTCC	CC1
SMC23.T.TCAGATGAGAAACCGC	CC1
SMC23.T.TCAGATGGTGTGACGA	CC1
SMC23.T.TCAGCTCAGCTGCCCA	CC1
SMC23.T.TCAGGATGTCGAACAG	CC1
SMC23.T.TCAGGATTCGAACTGT	CC1
SMC23.T.TCATTACCACGGTTTA	CC1
SMC23.T.TCATTACCATCCTTGC	CC1
SMC23.T.TCATTACGTCTTCAAG	CC1
SMC23.T.TCATTTGGTCCGAACC	CC1
SMC23.T.TCATTTGTCAAGGCTT	CC1
SMC23.T.TCCACACGTCTCCACT	CC1
SMC23.T.TCCCGATGTTTGACAC	CC1
SMC23.T.TCGCGAGAGGTACTCT	CC1
SMC23.T.TCGCGAGCACACCGCA	CC1
SMC23.T.TCGCGAGCATGATCCA	CC1
SMC23.T.TCGCGTTGTATAAACG	CC1
SMC23.T.TCGGGACCAATGACCT	CC1
SMC23.T.TCGGGACTCGTCCAGG	CC1
SMC23.T.TCGGTAATCACCTTAT	CC1
SMC23.T.TCGTACCAGCGTGAAC	CC1
SMC23.T.TCTATTGGTGGTCCGT	CC2
SMC23.T.TCTATTGGTTCACGGC	CC1
SMC23.T.TCTATTGTCATCATTC	CC1
SMC23.T.TCTCATAAGATATGGT	CC1

SMC23.T.TCTCTAATCTACCTGC	CC1
SMC23.T.TCTGAGAAGAGGGATA	CC2
SMC23.T.TCTGAGATCAAAGTAG	CC1
SMC23.T.TCTGGAAAGCGTGTCC	CC1
SMC23.T.TCTGGAAGTAAATGTG	CC1
SMC23.T.TCTTTCCCATTTGCTT	CC1
SMC23.T.TGAAAGAGTGCAGACA	CC1
SMC23.T.TGAAAGATCCTGCTTG	CC1
SMC23.T.TGACAACAGTGAAGAG	CC1
SMC23.T.TGACAACCACGGCCAT	CC1
SMC23.T.TGACGGCTCGGAGGTA	CC1
SMC23.T.TGACTAGTCCTTTCTC	CC1
SMC23.T.TGACTTTCAGGCAGTA	CC1
SMC23.T.TGACTTTTCGCCCTTA	CC1
SMC23.T.TGAGAGGTCGGGAGTA	CC1
SMC23.T.TGAGCATCACACCGCA	CC1
SMC23.T.TGAGCATCACACGCTG	CC1
SMC23.T.TGAGCATGTGCATCTA	CC1
SMC23.T.TGAGCCGAGCAGACTG	CC1
SMC23.T.TGAGCCGAGCCAGTAG	CC1
SMC23.T.TGAGCCGGTTCAACCA	CC1
SMC23.T.TGAGCCGTCAGCACAT	CC1
SMC23.T.TGAGGGAGTTATGCGT	CC1
SMC23.T.TGAGGGAGTTCCGTCT	CC2
SMC23.T.TGATTTCCAATACGCT	CC1
SMC23.T.TGATTTCGTGATGTGG	CC1
SMC23.T.TGCCCATCATATGAGA	CC1
SMC23.T.TGCGCAGGTACCGGCT	CC1
SMC23.T.TGCGGGTAGAGAGCTC	CC1
SMC23.T.TGCGGGTAGTCGCCGT	CC1
SMC23.T.TGCGGGTGTAGCTAAA	CC1
SMC23.T.TGCTACCCAAGACACG	CC1
SMC23.T.TGCTACCTCAGCTTAG	CC2
SMC23.T.TGCTGCTAGCACCGTC	CC1
SMC23.T.TGGCCAGGTTCAACCA	CC1
SMC23.T.TGGCCAGTCAGTTGAC	CC1
SMC23.T.TGGCGCAAGTGATCGG	CC1
SMC23.T.TGGCGCACACTAGTAC	CC1
SMC23.T.TGGCTGGTCATGTAGC	CC2
SMC23.T.TGGCTGGTCGGTTAAC	CC2
SMC23.T.TGGGAAGAGGCCCTTG	CC1
SMC23.T.TGGGAAGCACAGCGTC	CC1
SMC23.T.TGGGAAGGTTGCGTTA	CC1
SMC23.T.TGGGCGTTCGTATCAG	CC1
SMC23.T.TGGTTCCCAATAGAGT	CC1
SMC23.T.TGGTTCCGTGGTGTAG	CC1
SMC23.T.TGGTTCCTCTTTACAC	CC1
SMC23.T.TGTATTCCAGATGAGC	CC1
SMC23.T.TGTGGTAAGCTCTCGG	CC1
SMC23.T.TGTGGTAGTCGCTTTC	CC2
SMC23.T.TGTGTTTCAGTATGCT	CC1
SMC23.T.TGTTCCGCAAGTAATG	CC1
SMC23.T.TGTTCCGCAATTCCTT	CC1
SMC23.T.TTAGGACAGTCATCCA	CC2
SMC23.T.TTAGGCACAATCTACG	CC1
SMC23.T.TTAGGCACATGAAGTA	CC1
SMC23.T.TTATGCTGTGTTGAGG	CC1
SMC23.T.TTATGCTTCTCGAGTA	CC1

SMC23.T.TTCGAAGAGGCATGGT	CC1
SMC23.T.TTCGAAGCAGCAGTTT	CC1
SMC23.T.TTCGGTCTCGAGCCCA	CC1
SMC23.T.TTCTACAAGAGTCGGT	CC1
SMC23.T.TTCTACAAGGGCACTA	CC1
SMC23.T.TTCTACACAGGGTACA	CC1
SMC23.T.TTCTACATCCGCTGTT	CC1
SMC23.T.TTCTCAACACACCGCA	CC1
SMC23.T.TTCTCAAGTTCCGTCT	CC1
SMC23.T.TTGAACGAGAGCTGCA	CC1
SMC23.T.TTGAACGGTATAATGG	CC1
SMC23.T.TTGAACGGTCGACTGC	CC1
SMC23.T.TTGACTTAGCACGCCT	CC1
SMC23.T.TTGCCGTCAGGCGATA	CC1
SMC23.T.TTGCCGTGTTTCGCTC	CC1
SMC23.T.TTGCGTCAGGTCGGAT	CC1
SMC23.T.TTGGAACAGATGTAAC	CC1
SMC23.T.TTGGAACCACATCCGG	CC1
SMC23.T.TTGGAACGTTGGTAAA	CC1
SMC23.T.TTGTAGGAGACTAGGC	CC1
SMC23.T.TTGTAGGAGTTGCAGG	CC1
SMC23.T.TTGTAGGCATTAACCG	CC1
SMC23.T.TTGTAGGGTGGCCCTA	CC1
SMC23.T.TTTACTGAGATGAGAG	CC1
SMC23.T.TTTACTGGTCACTTCC	CC1
SMC23.T.TTTATGCGTAGCGTAG	CC1
SMC23.T.TTTCCTCGTCTAGCCG	CC1
SMC23.T.TTTCCTCTCTCGCATC	CC1
SMC23.T.TTTGCGCAGAGTTGGC	CC1
SMC23.T.TTTGCGCAGGCGATAC	CC1
SMC23.T.TTTGCGCCAGACGCTC	CC1
SMC23.T.TTTGTCAAGCTACCGC	CC1
SMC23.T.TTTGTCACAGCTGCTG	CC1
SMC23.T.TTTGTCATCAATCTCT	CC1
SMC24.T.AAAGCAAAGACTGGGT	CC2
SMC24.T.AAAGTAGAGCTAAGAT	CC2
SMC24.T.AAAGTAGTCTTGTACT	CC2
SMC24.T.AACACGTAGGTGCTAG	CC2
SMC24.T.AACGTTGCATCACGAT	CC2
SMC24.T.AACTCAGTCCCTCTTT	CC2
SMC24.T.AACTCCCCACCAACCG	CC2
SMC24.T.AACTCCCTCATCACCC	CC2
SMC24.T.AACTTTCCAGATGGGT	CC2
SMC24.T.AAGACCTCAGTTTACG	CC2
SMC24.T.AAGACCTTCTCCGGTT	CC2
SMC24.T.AAGCCGCAGAGCAATT	CC2
SMC24.T.AAGGCAGCATTCGACA	CC2
SMC24.T.AAGGCAGTCTCAAGTG	CC2
SMC24.T.AAGTCTGTCTCCGGTT	CC2
SMC24.T.AATCCAGTCCGCATCT	CC2
SMC24.T.AATCCAGTCGAACTGT	CC2
SMC24.T.ACACCCTCATTTGCCC	CC2
SMC24.T.ACAGCCGAGAAGCCCA	CC2
SMC24.T.ACAGCCGGTGTAACGG	CC2
SMC24.T.ACATCAGAGGCTATCT	CC1
SMC24.T.ACATCAGTCCGCATAA	CC2
SMC24.T.ACCCACTAGAGCAATT	CC2
SMC24.T.ACCCACTAGGACCACA	CC2

SMC24.T.ACCGTAATCTATGTGG	CC2
SMC24.T.ACCTTTACAAATCCGT	CC2
SMC24.T.ACCTTTATCTTCGAGA	CC2
SMC24.T.ACGAGCCCACTAGTAC	CC2
SMC24.T.ACGAGGAAGTCCATAC	CC2
SMC24.T.ACGATACTCTGCGACG	CC2
SMC24.T.ACGATGTAGTCTTGCA	CC2
SMC24.T.ACGATGTGTCTAACGT	CC2
SMC24.T.ACGCAGCAGTTGCAGG	CC2
SMC24.T.ACGCAGCGTACTCAAC	CC2
SMC24.T.ACGCAGCGTTCGTGAT	CC2
SMC24.T.ACGCAGCTCAGTTAGC	CC2
SMC24.T.ACGCAGCTCTGATTCT	CC2
SMC24.T.ACGGGCTAGAGAGCTC	CC2
SMC24.T.ACGTCAAGTCTAGGTT	CC1
SMC24.T.ACTATCTTCTATCCTA	CC2
SMC24.T.ACTGATGTCACCCTCA	CC2
SMC24.T.ACTTACTAGGGCTCTC	CC2
SMC24.T.ACTTACTCAGTTAACC	CC2
SMC24.T.ACTTGTTTCGAATGCT	CC2
SMC24.T.AGAGCGAGTGTGCGTC	CC2
SMC24.T.AGATCTGTCGCTTGTC	CC2
SMC24.T.AGCAGCCAGGGTTCCC	CC2
SMC24.T.AGCAGCCCAAGCGTAG	CC2
SMC24.T.AGCTCCTTCAGTCAGT	CC2
SMC24.T.AGCTCTCAGGCTCATT	CC2
SMC24.T.AGCTTGAAGATGCCTT	CC2
SMC24.T.AGGGATGTCGATGAGG	CC2
SMC24.T.AGTTGGTGTTAGAACA	CC2
SMC24.T.ATAACGCGTACGAAAT	CC2
SMC24.T.ATAGACCAGTTTAGGA	CC2
SMC24.T.ATAGACCCAATCAGAA	CC2
SMC24.T.ATCACGACACGGTAGA	CC2
SMC24.T.ATCCACCAGAATTCCC	CC2
SMC24.T.ATCCACCTCCTGTAGA	CC2
SMC24.T.ATCCGAATCGTCCGTT	CC2
SMC24.T.ATCCGAATCGTGACAT	CC2
SMC24.T.ATCGAGTTCCTCAATT	CC2
SMC24.T.ATCTACTGTTAAAGTG	CC2
SMC24.T.ATCTACTGTTCCACTC	CC2
SMC24.T.ATGAGGGTCTGTCAAG	CC2
SMC24.T.ATGCGATGTCTCAACA	CC2
SMC24.T.ATGTGTGTCAGAGACG	CC2
SMC24.T.ATTATCCAGATGTAAC	CC2
SMC24.T.ATTATCCCAAGGACAC	CC2
SMC24.T.ATTGGACGTCATATGC	CC2
SMC24.T.ATTGGTGAGTTACGGG	CC2
SMC24.T.ATTGGTGGTCTGATTG	CC2
SMC24.T.ATTTCTGAGATGGGTC	CC2
SMC24.T.CAACCAAAGCCAACAG	CC2
SMC24.T.CAACCTCAGCCAGGAT	CC2
SMC24.T.CAAGGCCGTTGAACTC	CC2
SMC24.T.CACACCTCAGGTGCCT	CC2
SMC24.T.CACAGGCGTTACCAGT	CC2
SMC24.T.CACCACTGTATTACCG	CC2
SMC24.T.CACTCCACACCCATGG	CC2
SMC24.T.CAGAATCCAATGAAAC	CC2
SMC24.T.CAGATCAAGTAAGTAC	CC2

SMC24.T.CAGATCACAGCTATTG	CC2
SMC24.T.CAGCATAGTATAGGTA	CC2
SMC24.T.CAGCATAGTCCGTGAC	CC2
SMC24.T.CAGCGACCAACAACCT	CC2
SMC24.T.CAGCTAACACATTTCT	CC2
SMC24.T.CAGCTAATCCTTAATC	CC2
SMC24.T.CAGCTGGCATAGTAAG	CC2
SMC24.T.CAGGTGCAGTGTTAGA	CC2
SMC24.T.CAGGTGCGTCGCATAT	CC1
SMC24.T.CAGTCCTCATGTCCTC	CC2
SMC24.T.CATATGGCAATGGTCT	CC2
SMC24.T.CATATGGCAGGGTACA	CC2
SMC24.T.CATATTCTCAAGATCC	CC2
SMC24.T.CATCAAGAGATGGGTC	CC2
SMC24.T.CATCAGATCTTCATGT	CC2
SMC24.T.CATCCACAGCAAATCA	CC2
SMC24.T.CATTATCTCGGCGCAT	CC2
SMC24.T.CCACCTAGTGCTGTAT	CC2
SMC24.T.CCAGCGACAGCTGTTA	CC2
SMC24.T.CCATGTCTCATTTGGG	CC2
SMC24.T.CCCAGTTAGGCTCTTA	CC2
SMC24.T.CCCTCCTGTTTGGGCC	CC2
SMC24.T.CCGTACTTCATGCTCC	CC2
SMC24.T.CCGTGGAGTTATTCTC	CC2
SMC24.T.CCGTTCACAAAGGTGC	CC2
SMC24.T.CCTAGCTTCCGATATG	CC2
SMC24.T.CCTCAGTCACAGGTTT	CC2
SMC24.T.CCTCAGTGTACTTAGC	CC2
SMC24.T.CCTTCCCAGTTACCCA	CC2
SMC24.T.CCTTCCCCATCCCATC	CC2
SMC24.T.CCTTCCCTCTGGCGAC	CC2
SMC24.T.CCTTCGACACCAACCG	CC2
SMC24.T.CCTTCGATCTCACATT	CC2
SMC24.T.CCTTTCTGTAGTGAAT	CC2
SMC24.T.CGAACATCAGCTGCAC	CC2
SMC24.T.CGAACATCAGTAAGAT	CC2
SMC24.T.CGAATGTCATGAGCGA	CC1
SMC24.T.CGACCTTCAATGCCAT	CC2
SMC24.T.CGACTTCTCTGGTTCC	CC2
SMC24.T.CGACTTCTCTTGCCGT	CC2
SMC24.T.CGAGAAGGTAGCGTCC	CC2
SMC24.T.CGAGAAGTCGGATGTT	CC2
SMC24.T.CGAGCACTCTACTATC	CC2
SMC24.T.CGAGCCAGTTCCCTTG	CC2
SMC24.T.CGATTGAAGCCCGAAA	CC2
SMC24.T.CGATTGAGTTCAACCA	CC2
SMC24.T.CGCCAAGAGATCTGCT	CC2
SMC24.T.CGCGTTTCAAAGGAAG	CC2
SMC24.T.CGCGTTTGTAACGCGA	CC2
SMC24.T.CGCGTTTTCCCTTGTG	CC2
SMC24.T.CGCTATCAGCGGCTTC	CC2
SMC24.T.CGCTATCTCAGATAAG	CC2
SMC24.T.CGCTTCACAAGACGTG	CC2
SMC24.T.CGGACACGTACATCCA	CC2
SMC24.T.CGGACACGTACGAAAT	CC2
SMC24.T.CGGACACTCAAGAAGT	CC2
SMC24.T.CGGACGTGTGTTAAGA	CC2
SMC24.T.CGGACTGAGAACAACT	CC2

SMC24.T.CGGACTGTCTTTAGTC	CC2
SMC24.T.CGGTTAACACCCAGTG	CC2
SMC24.T.CGGTTAATCCGAACGC	CC2
SMC24.T.CGTCACTGTAGCTGCC	CC2
SMC24.T.CGTCAGGTCCGCTGTT	CC2
SMC24.T.CGTCTACGTCCAGTTA	CC2
SMC24.T.CGTGAGCCATCACAAC	CC2
SMC24.T.CGTGAGCTCGTTTGCC	CC2
SMC24.T.CTAATGGCATTGGTAC	CC2
SMC24.T.CTACATTCAGGGCATA	CC2
SMC24.T.CTACCCACACGAAGCA	CC1
SMC24.T.CTACCCATCGCTTAGA	CC2
SMC24.T.CTACGTCCATATACCG	CC2
SMC24.T.CTACGTCGTTACGGAG	CC2
SMC24.T.CTACGTCTCCAGATCA	CC2
SMC24.T.CTAGTGAAGCGCTTAT	CC2
SMC24.T.CTAGTGATCACATGCA	CC2
SMC24.T.CTCACACCACGACGAA	CC2
SMC24.T.CTCACACGTCAGAAGC	CC2
SMC24.T.CTCGAAACAGGGCATA	CC2
SMC24.T.CTCGGAGGTCGTCTTC	CC2
SMC24.T.CTCTAATAGGTAGCCA	CC2
SMC24.T.CTCTAATTCGTCGTTC	CC2
SMC24.T.CTCTAATTCGTCTGAA	CC2
SMC24.T.CTCTACGAGACAAAGG	CC2
SMC24.T.CTCTACGAGATGAGAG	CC2
SMC24.T.CTGATCCAGATCCCGC	CC2
SMC24.T.CTTAACTCAATAGCGG	CC2
SMC24.T.CTTACCGAGATTACCC	CC2
SMC24.T.CTTCTCTCATTTCAGG	CC2
SMC24.T.CTTCTCTTCCTCATTA	CC2
SMC24.T.CTTTGCGTCGCTTGTC	CC2
SMC24.T.GAACATCGTAGAGTGC	CC2
SMC24.T.GAACATCTCTGAGGGA	CC2
SMC24.T.GAACGGAAGTTGAGAT	CC2
SMC24.T.GAATGAACATCCTAGA	CC2
SMC24.T.GAATGAATCAACCATG	CC2
SMC24.T.GACACGCGTGACGCCT	CC2
SMC24.T.GACAGAGAGACAGAGA	CC2
SMC24.T.GACGCGTAGTTGTCGT	CC2
SMC24.T.GACGGCTAGGTGCTAG	CC2
SMC24.T.GACGGCTCAGTCAGCC	CC2
SMC24.T.GACTACATCGCATGGC	CC2
SMC24.T.GACTGCGCACCGGAAA	CC2
SMC24.T.GAGGTGACAATCAGAA	CC2
SMC24.T.GAGGTGACATGGATGG	CC2
SMC24.T.GAGGTGATCCACTCCA	CC2
SMC24.T.GATCAGTCATGGGAAC	CC2
SMC24.T.GATCGCGGTCAGAAGC	CC2
SMC24.T.GATCTAGGTTGAGGTG	CC2
SMC24.T.GATGAGGAGTGCTGCC	CC2
SMC24.T.GATGCTAGTGCCTGGT	CC2
SMC24.T.GCAATCACAAAGCAAT	CC2
SMC24.T.GCAATCACAGCCAATT	CC2
SMC24.T.GCACTCTGTGACCAAG	CC2
SMC24.T.GCAGCCAGTAGCTAAA	CC2
SMC24.T.GCAGCCATCCAAACAC	CC2
SMC24.T.GCAGTTAGTGAGTATA	CC2

SMC24.T.GCATACAAGCGCCTCA	CC2
SMC24.T.GCATGATTCCGCTGTT	CC2
SMC24.T.GCCAAATTCCACGCAG	CC2
SMC24.T.GCGACCAAGTACGACG	CC2
SMC24.T.GCGCAACTCGGGAGTA	CC2
SMC24.T.GCTGCAGAGCTTATCG	CC2
SMC24.T.GCTGGGTCACTTCTGC	CC2
SMC24.T.GCTGGGTTCGAGAGCA	CC2
SMC24.T.GCTTCCATCTATCCCG	CC2
SMC24.T.GCTTCCATCTGCAAGT	CC2
SMC24.T.GGAAAGCAGACAAGCC	CC2
SMC24.T.GGACAGAGTACGACCC	CC2
SMC24.T.GGACATTCAAACGCGA	CC2
SMC24.T.GGATGTTGTACCATCA	CC2
SMC24.T.GGATTACCATTCGACA	CC2
SMC24.T.GGCAATTAGACGCACA	CC2
SMC24.T.GGCGACTGTCAAGCGA	CC2
SMC24.T.GGCTGGTAGTTTAGGA	CC2
SMC24.T.GGGACCTAGGAGTCTG	CC2
SMC24.T.GGGATGAGTATATCCG	CC2
SMC24.T.GGGCACTCAGTGGGAT	CC2
SMC24.T.GGGCACTGTAATAGCA	CC2
SMC24.T.GGGCACTGTCTACCTC	CC2
SMC24.T.GGGCACTTCAAAGACA	CC2
SMC24.T.GGGCATCAGCGATCCC	CC2
SMC24.T.GGGCATCTCTCCAGGG	CC2
SMC24.T.GGGTCTGAGGCTCAGA	CC2
SMC24.T.GGGTTGCTCTCCCTGA	CC2
SMC24.T.GTAACGTGTGCCTGGT	CC2
SMC24.T.GTAACTGTCTCAACTT	CC2
SMC24.T.GTACTCCCACCATGTA	CC2
SMC24.T.GTACTCCTCTAGCACA	CC2
SMC24.T.GTAGGCCCAAGAGGCT	CC2
SMC24.T.GTCATTTGTTAGGGTG	CC2
SMC24.T.GTCTTCGGTTCCGGCA	CC2
SMC24.T.GTGCATATCTTGCCGT	CC2
SMC24.T.GTGGGTCAGCGACGTA	CC2
SMC24.T.GTGGGTCTCCGGGTGT	CC2
SMC24.T.GTTACAGAGAGCAATT	CC2
SMC24.T.GTTACAGAGAGCCTAG	CC1
SMC24.T.GTTCATTCACTCTGTC	CC2
SMC24.T.GTTCGGGGTTCGGGCT	CC2
SMC24.T.TAAGAGAAGTTAAGTG	CC2
SMC24.T.TAAGTGCGTCTACCTC	CC2
SMC24.T.TAAGTGCTCAGAGCTT	CC2
SMC24.T.TACAGTGTCACGAAGG	CC2
SMC24.T.TACCTTACACGTGAGA	CC2
SMC24.T.TACGGGCTCCAGTAGT	CC2
SMC24.T.TACTCATCAAAGTCAA	CC2
SMC24.T.TACTCGCCATCACAAC	CC2
SMC24.T.TACTTACAGTTAACGA	CC2
SMC24.T.TACTTGTGTTCCACAA	CC1
SMC24.T.TAGAGCTAGTGTACGG	CC2
SMC24.T.TAGAGCTGTTCAACCA	CC2
SMC24.T.TAGCCGGGTCTCCCTA	CC2
SMC24.T.TAGGCATGTGATGTGG	CC2
SMC24.T.TAGGCATGTGGGTCAA	CC2
SMC24.T.TAGTGGTGTTCCGTCT	CC2

SMC24.T.TATCAGGAGGCGTACA	CC2
SMC24.T.TATTACCAGTCTCCTC	CC2
SMC24.T.TCAACGAGTACCGTAT	CC2
SMC24.T.TCAACGATCCCTCAGT	CC2
SMC24.T.TCAATCTAGCCCAGCT	CC2
SMC24.T.TCAATCTGTGATGTGG	CC2
SMC24.T.TCACAAGAGAGACGAA	CC2
SMC24.T.TCACAAGCACGGTGTC	CC2
SMC24.T.TCACAAGTCGTTTGCC	CC2
SMC24.T.TCACGAATCGGGAGTA	CC2
SMC24.T.TCAGATGAGGACACCA	CC2
SMC24.T.TCAGATGCAGCCACCA	CC2
SMC24.T.TCAGCTCTCCAAACAC	CC2
SMC24.T.TCAGGATCATGCTGGC	CC2
SMC24.T.TCAGGTATCAATCTCT	CC2
SMC24.T.TCATTTGAGTACATGA	CC2
SMC24.T.TCGGTAACACAGGAGT	CC1
SMC24.T.TCGGTAACAGAGTGTG	CC2
SMC24.T.TCGGTAATCACTTATC	CC2
SMC24.T.TCGTACCAGTGTACGG	CC2
SMC24.T.TCGTACCTCCATGAAC	CC2
SMC24.T.TCGTAGAGTGTGAAAT	CC2
SMC24.T.TCGTAGATCGGCGCTA	CC2
SMC24.T.TCTATTGTCGTTGCCT	CC2
SMC24.T.TCTATTGTCTCATTCA	CC2
SMC24.T.TCTCTAACATGAAGTA	CC2
SMC24.T.TCTTCGGGTAACGACG	CC2
SMC24.T.TCTTTCCCACAGAGGT	CC2
SMC24.T.TGACTAGGTCATGCCG	CC2
SMC24.T.TGACTTTAGTTCGCGC	CC2
SMC24.T.TGACTTTCAGGTCCAC	CC2
SMC24.T.TGACTTTTCTTAGAGC	CC2
SMC24.T.TGAGCCGGTAGTAGTA	CC2
SMC24.T.TGAGGGACACGTTGGC	CC2
SMC24.T.TGATTTCGTGACTCAT	CC2
SMC24.T.TGCCCTATCTGGCGTG	CC2
SMC24.T.TGCGCAGGTCTGCCAG	CC2
SMC24.T.TGCGCAGGTTACGACT	CC2
SMC24.T.TGGCTGGGTCTTCGTC	CC2
SMC24.T.TGGGAAGGTTGATTGC	CC2
SMC24.T.TGGGCGTCATGGATGG	CC2
SMC24.T.TGTATTCAGTGTTTGC	CC2
SMC24.T.TGTGTTTGTAAGGGAA	CC2
SMC24.T.TGTTCCGAGACTACAA	CC2
SMC24.T.TTAACTCAGTCCGGTC	CC2
SMC24.T.TTAACTCTCAAACCGT	CC1
SMC24.T.TTAGTTCAGCTGAAAT	CC2
SMC24.T.TTCCCAGAGGTGCAAC	CC2
SMC24.T.TTCCCAGTCTACGAGT	CC2
SMC24.T.TTCGGTCCAAGTTCTG	CC2
SMC24.T.TTCGGTCGTGACCAAG	CC2
SMC24.T.TTCTTAGGTCTAGAGG	CC2
SMC24.T.TTCTTAGTCGCCTGTT	CC2
SMC24.T.TTGAACGAGAGACTTA	CC2
SMC24.T.TTGGCAAAGTCTTGCA	CC2
SMC24.T.TTGTAGGCAGCCAATT	CC2
SMC24.T.TTTATGCGTAGCAAAT	CC2
SMC24.T.TTTCCTCGTTGTCTTT	CC2

SMC24.T.TTTGGTTAGATGGCGT	CC2
SMC24.T.TTTGTCAGTTCCCTTG	CC2
SMC24.T.TTTGTCAGTTGCGTTA	CC2
SMC25.T.AAACGGGGTTATGTGC	CC1
SMC25.T.AAACGGGTCAACACAC	CC1
SMC25.T.AAAGATGGTAACGTTC	CC1
SMC25.T.AAATGCCAGTGATCGG	CC1
SMC25.T.AAATGCCTCAAACGGG	CC1
SMC25.T.AACCATGTCCTAAGTG	CC2
SMC25.T.AACTCAGCACCGAAAG	CC1
SMC25.T.AACTCAGGTAGAGTGC	CC1
SMC25.T.AACTCAGGTCCGAAGA	CC1
SMC25.T.AACTCAGGTTGGTAAA	CC1
SMC25.T.AACTCTTGTAGGGACT	CC1
SMC25.T.AACTCTTGTTCGCGAC	CC1
SMC25.T.AACTGGTTCAGGCCCA	CC1
SMC25.T.AACTTTCAGCCCAATT	CC1
SMC25.T.AAGACCTCAGGAATCG	CC1
SMC25.T.AAGACCTTCAGTCCCT	CC1
SMC25.T.AAGCCGCCAGGTGCCT	CC1
SMC25.T.AAGGCAGAGCAAATCA	CC1
SMC25.T.AAGGCAGCAAGCCATT	CC1
SMC25.T.AAGGCAGCACTTAACG	CC1
SMC25.T.AAGGCAGGTCCAGTGC	CC1
SMC25.T.AAGGTTCTCTGTCTAT	CC1
SMC25.T.AAGTCTGAGCTACCGC	CC1
SMC25.T.AAGTCTGGTGGTGTAG	CC1
SMC25.T.AATCCAGGTGTAATGA	CC1
SMC25.T.AATCCAGTCCGCATAA	CC1
SMC25.T.AATCGGTCAGTACACT	CC1
SMC25.T.AATCGGTTCCGTCATC	CC1
SMC25.T.ACACCAATCGAATCCA	CC1
SMC25.T.ACACCCTAGTATCGAA	CC2
SMC25.T.ACACCGGTCCTGTACC	CC1
SMC25.T.ACACTGACAGTCACTA	CC1
SMC25.T.ACAGCTATCACTCCTG	CC1
SMC25.T.ACATACGAGTCAATAG	CC1
SMC25.T.ACATACGCACCACGTG	CC1
SMC25.T.ACATCAGAGGGCTCTC	CC1
SMC25.T.ACATGGTAGCTCCTTC	CC1
SMC25.T.ACCCACTAGAATGTTG	CC2
SMC25.T.ACCCACTAGCAGCGTA	CC1
SMC25.T.ACCTTTACACCAACCG	CC1
SMC25.T.ACCTTTAGTGACCAAG	CC1
SMC25.T.ACCTTTATCACCACCT	CC1
SMC25.T.ACGAGCCGTAATAGCA	CC1
SMC25.T.ACGAGGAAGTGAAGTT	CC1
SMC25.T.ACGAGGACACGGCCAT	CC1
SMC25.T.ACGATGTTCAGTTTGG	CC1
SMC25.T.ACGCCAGGTCGTGGCT	CC1
SMC25.T.ACGCCGACACAGATTC	CC1
SMC25.T.ACGCCGAGTCTAGTGT	CC1
SMC25.T.ACGGGCTTCATACGGT	CC1
SMC25.T.ACGGGTCAGGCATTGG	CC1
SMC25.T.ACGGGTCAGGCTATCT	CC2
SMC25.T.ACGGGTCAGTTGTCGT	CC1
SMC25.T.ACGTCAACACAGCCCA	CC1
SMC25.T.ACGTCAAGTCGCTTCT	CC1

SMC25.T.ACGTCAATCTACTCAT	CC1
SMC25.T.ACTATCTCACTGAAGG	CC1
SMC25.T.ACTGAACGTCGCTTTC	CC1
SMC25.T.ACTGAGTGTCAAGCGA	CC1
SMC25.T.ACTGATGTCTACTCAT	CC1
SMC25.T.ACTGATGTCTTAGCCC	CC1
SMC25.T.ACTGCTCAGTCGATAA	CC1
SMC25.T.ACTGCTCGTGAGTGAC	CC2
SMC25.T.ACTGTCCGTTATTCTC	CC1
SMC25.T.ACTGTCCTCTGGCGTG	CC1
SMC25.T.ACTTGTTGTTGTGGAG	CC1
SMC25.T.AGAGCTTCAGACAGGT	CC1
SMC25.T.AGAGTGGCAAATCCGT	CC2
SMC25.T.AGATTGCGTATGCTTG	CC1
SMC25.T.AGCAGCCAGTGCAAGC	CC1
SMC25.T.AGCAGCCGTATATGAG	CC1
SMC25.T.AGCCTAATCACTTCAT	CC1
SMC25.T.AGCGTATAGATAGTCA	CC1
SMC25.T.AGCGTCGAGTGGGATC	CC1
SMC25.T.AGCTCTCCAGCTGTGC	CC1
SMC25.T.AGCTCTCGTAGCACGA	CC1
SMC25.T.AGGCCACGTCCAGTAT	CC1
SMC25.T.AGGGAGTTCCGTACAA	CC1
SMC25.T.AGGTCCGAGTGGGATC	CC1
SMC25.T.AGTCTTTCACCGTTGG	CC1
SMC25.T.AGTCTTTGTTCTGTTT	CC1
SMC25.T.AGTGTCAAGTAATCCC	CC1
SMC25.T.ATAACGCAGGTGACCA	CC1
SMC25.T.ATAACGCTCAATCTCT	CC1
SMC25.T.ATCATCTAGACAAAGG	CC1
SMC25.T.ATCATGGCAGACGCCT	CC1
SMC25.T.ATCATGGTCGGTGTCG	CC1
SMC25.T.ATCATGGTCTGGAGCC	CC1
SMC25.T.ATCGAGTCATTGGGCC	CC1
SMC25.T.ATCTACTCAACTGGCC	CC1
SMC25.T.ATCTACTCACTGTCGG	CC1
SMC25.T.ATCTACTCAGCTCGCA	CC1
SMC25.T.ATGAGGGCAGACAAGC	CC1
SMC25.T.ATGCGATCATAGAAAC	CC1
SMC25.T.ATGGGAGCATCACGTA	CC1
SMC25.T.ATGTGTGTCTTGTATC	CC1
SMC25.T.ATTACTCGTCAGAAGC	CC1
SMC25.T.ATTATCCTCCGCATAA	CC1
SMC25.T.ATTGGACCATAAAGGT	CC1
SMC25.T.CAACCTCCAGCTGCAC	CC1
SMC25.T.CAAGATCGTTGCGCAC	CC1
SMC25.T.CAAGATCTCACCATAG	CC1
SMC25.T.CAAGTTGCATAGACTC	CC1
SMC25.T.CACAAACCATTTCACT	CC1
SMC25.T.CACAAACGTATAGGGC	CC1
SMC25.T.CACACAAAGAACAATC	CC1
SMC25.T.CACACCTGTCCGTGAC	CC1
SMC25.T.CACACCTGTGAAAGAG	CC1
SMC25.T.CACACTCGTCACTTCC	CC1
SMC25.T.CACAGGCAGGGTGTGT	CC1
SMC25.T.CACCTTGAGATCTGCT	CC1
SMC25.T.CACCTTGCAATCCGAT	CC1
SMC25.T.CAGAATCCAATAGCGG	CC1

SMC25.T.CAGATCACATGTAGTC	CC1
SMC25.T.CAGCATAAGTGAATTG	CC2
SMC25.T.CAGCATACAAGGTTTC	CC1
SMC25.T.CAGCATACAGTGAGTG	CC1
SMC25.T.CAGCCGAGTCGCATAT	CC1
SMC25.T.CAGCCGAGTTCGTCTC	CC1
SMC25.T.CAGCCGATCGCAAACT	CC1
SMC25.T.CAGCCGATCTCTTGAT	CC1
SMC25.T.CAGCTAAAGCCCAGCT	CC1
SMC25.T.CAGCTGGCAGCTGCTG	CC1
SMC25.T.CAGGTGCAGGTAAACT	CC2
SMC25.T.CAGGTGCGTCGCATAT	CC1
SMC25.T.CATATTCTCTTGAGAC	CC1
SMC25.T.CATCAGACAATGTAAG	CC1
SMC25.T.CATCCACAGATCGGGT	CC2
SMC25.T.CATCCACGTCTCAACA	CC1
SMC25.T.CATCGAAGTTTGTTGG	CC1
SMC25.T.CATGACACATATACCG	CC1
SMC25.T.CATGGCGGTTCGCTAA	CC1
SMC25.T.CATTATCAGGTAGCCA	CC1
SMC25.T.CATTATCGTACCCAAT	CC1
SMC25.T.CCAATCCGTCGAATCT	CC1
SMC25.T.CCATGTCTCGAATGGG	CC1
SMC25.T.CCATGTCTCGACAGCC	CC1
SMC25.T.CCATTCGCACAAGACG	CC1
SMC25.T.CCCAATCTCTTATCTG	CC1
SMC25.T.CCCTCCTTCATGGTCA	CC1
SMC25.T.CCCTCCTTCTCGCTTG	CC1
SMC25.T.CCGGGATAGCGGCTTC	CC1
SMC25.T.CCGGTAGAGTATGACA	CC1
SMC25.T.CCGTTCAGTAGAGCTG	CC1
SMC25.T.CCGTTCATCCGTACAA	CC1
SMC25.T.CCTAAAGGTAAACCTC	CC1
SMC25.T.CCTACACCATCTATGG	CC1
SMC25.T.CCTACCACAAAGTGCG	CC1
SMC25.T.CCTCTGAAGAACAACT	CC1
SMC25.T.CCTTCCCGTTAAAGAC	CC1
SMC25.T.CCTTTCTAGGCCCTCA	CC1
SMC25.T.CCTTTCTGTCCGAGTC	CC1
SMC25.T.CGAACATAGTCCGTAT	CC1
SMC25.T.CGAATGTCAACGATGG	CC1
SMC25.T.CGAATGTCATGAGCGA	CC1
SMC25.T.CGACCTTTCTTCCTTC	CC1
SMC25.T.CGATGGCGTACCGGCT	CC1
SMC25.T.CGATTGATCGAATCCA	CC1
SMC25.T.CGATTGATCGACCAGC	CC1
SMC25.T.CGCCAAGTCCAACCAA	CC1
SMC25.T.CGCTATCTCTCCAACC	CC1
SMC25.T.CGCTGGATCATCTGTT	CC1
SMC25.T.CGCTTCAGTAGCGATG	CC1
SMC25.T.CGGACACAGGCAGTCA	CC1
SMC25.T.CGGACGTTCTTCCTTC	CC1
SMC25.T.CGGACTGGTGGTAACG	CC1
SMC25.T.CGGAGTCGTAAAGTCA	CC1
SMC25.T.CGGAGTCTCTCAAACG	CC1
SMC25.T.CGGAGTCTCTTAGCCC	CC1
SMC25.T.CGGCTAGAGACTACAA	CC1
SMC25.T.CGGCTAGGTCCGAATT	CC1

SMC25.T.CGGTTAACATGCCTAA	CC1
SMC25.T.CGGTTAAGTCTAAAGA	CC2
SMC25.T.CGTAGGCAGTATCGAA	CC2
SMC25.T.CGTCCATTCACATAGC	CC1
SMC25.T.CGTCTACAGTAGATGT	CC1
SMC25.T.CGTTAGATCGAACTGT	CC1
SMC25.T.CGTTGGGCAGGCAGTA	CC1
SMC25.T.CTAACTTAGAGGTAGA	CC1
SMC25.T.CTAATGGAGCTAGCCC	CC1
SMC25.T.CTAATGGGTTATGTGC	CC1
SMC25.T.CTAGTGATCTTGTTTG	CC2
SMC25.T.CTCACACGTATTCGTG	CC1
SMC25.T.CTCAGAACAAGGCTCC	CC1
SMC25.T.CTCCTAGCAAGCTGAG	CC1
SMC25.T.CTCGAAACACCGAAAG	CC1
SMC25.T.CTCGAGGCATCTACGA	CC1
SMC25.T.CTCGGAGAGCGTAATA	CC1
SMC25.T.CTCGGGAGTAGGACAC	CC1
SMC25.T.CTCGTACCATGCTGGC	CC1
SMC25.T.CTCGTACGTTAAGGGC	CC1
SMC25.T.CTCGTCAGTATATGAG	CC1
SMC25.T.CTCTACGGTCAATGTC	CC1
SMC25.T.CTCTGGTAGTCATCCA	CC1
SMC25.T.CTGATAGGTCCCTACT	CC1
SMC25.T.CTGATAGGTTGCGTTA	CC1
SMC25.T.CTGCTGTGTATTACCG	CC1
SMC25.T.CTGGTCTAGCCACCTG	CC1
SMC25.T.CTGGTCTAGGAGCGAG	CC1
SMC25.T.CTGTTTACAGCTCCGA	CC1
SMC25.T.CTTACCGGTCATCGGC	CC1
SMC25.T.CTTCTCTCAATTCCTT	CC1
SMC25.T.CTTCTCTCATCGATGT	CC2
SMC25.T.CTTCTCTTCTGCTGCT	CC1
SMC25.T.GAAACTCAGAATGTGT	CC1
SMC25.T.GAAACTCAGACTAAGT	CC1
SMC25.T.GAAACTCCATGCCTAA	CC1
SMC25.T.GAAATGAGTTTGGCGC	CC1
SMC25.T.GAAATGATCGATCCCT	CC1
SMC25.T.GAACATCTCAAACCAC	CC1
SMC25.T.GAACCTAAGGTGACCA	CC1
SMC25.T.GAACGGACAGTTTACG	CC1
SMC25.T.GAAGCAGGTAAATGAC	CC1
SMC25.T.GAAGCAGTCAGAGCTT	CC1
SMC25.T.GAAGCAGTCGCTTAGA	CC1
SMC25.T.GAATAAGAGAGTCGGT	CC1
SMC25.T.GAATAAGTCGTACGGC	CC1
SMC25.T.GAATGAACATGCATGT	CC1
SMC25.T.GAATGAATCATATCGG	CC1
SMC25.T.GACACGCGTAAACGCG	CC1
SMC25.T.GACACGCGTTCGCGAC	CC1
SMC25.T.GACAGAGGTTCACGGC	CC1
SMC25.T.GACCAATCAAGCCATT	CC1
SMC25.T.GACCAATTCCTAGGGC	CC1
SMC25.T.GACGTGCAGCTAAGAT	CC1
SMC25.T.GACGTGCCAGCCTTGG	CC1
SMC25.T.GACGTTACACTGCCAG	CC1
SMC25.T.GACTACAAGCAAATCA	CC1
SMC25.T.GACTACACAGGCGATA	CC1

SMC25.T.GACTGCGAGACGCTTT	CC1
SMC25.T.GACTGCGCACGGATAG	CC1
SMC25.T.GACTGCGCAGCGTCCA	CC1
SMC25.T.GAGCAGAGTCTTCGTC	CC1
SMC25.T.GAGGTGATCTGGCGTG	CC1
SMC25.T.GATCAGTCATGGTCAT	CC1
SMC25.T.GATCAGTGTTACGACT	CC1
SMC25.T.GATCTAGCAGTATGCT	CC2
SMC25.T.GATGAAACATGTCCTC	CC1
SMC25.T.GATGAAATCTGTCTCG	CC1
SMC25.T.GATGAGGAGCCGGTAA	CC1
SMC25.T.GATGAGGTCTGGTTCC	CC1
SMC25.T.GATTCAGGTCGGCACT	CC1
SMC25.T.GATTCAGGTTCCATGA	CC1
SMC25.T.GCAAACTCACGGTGTC	CC1
SMC25.T.GCAATCAAGAGTACCG	CC1
SMC25.T.GCACTCTCACGACTCG	CC1
SMC25.T.GCAGCCAGTTCCTCCA	CC1
SMC25.T.GCAGCCATCAAGGCTT	CC1
SMC25.T.GCAGTTACAGACAAGC	CC1
SMC25.T.GCATACATCAATACCG	CC1
SMC25.T.GCATGATAGGAATTAC	CC1
SMC25.T.GCATGATGTCTGGAGA	CC1
SMC25.T.GCATGATGTGTCGCTG	CC1
SMC25.T.GCATGTAGTAGCTTGT	CC2
SMC25.T.GCATGTATCACTTCAT	CC1
SMC25.T.GCATGTATCTTCGAGA	CC1
SMC25.T.GCCAAATCATTTGCCC	CC1
SMC25.T.GCGAGAATCTGGAGCC	CC1
SMC25.T.GCGCAACAGCTTCGCG	CC1
SMC25.T.GCGCAACAGTTCCACA	CC1
SMC25.T.GCGCAGTAGTCACGCC	CC2
SMC25.T.GCGGGTTAGCGTCTAT	CC1
SMC25.T.GCGGGTTAGTTATCGC	CC1
SMC25.T.GCGGGTTCATCGGTTA	CC1
SMC25.T.GCTCCTAGTAACGCGA	CC1
SMC25.T.GCTGCGATCGCAAGCC	CC1
SMC25.T.GCTGCTTAGTCCCACG	CC1
SMC25.T.GCTGCTTCAAGTCATC	CC1
SMC25.T.GCTGGGTGTACTTGAC	CC1
SMC25.T.GCTTCCACATGCAACT	CC1
SMC25.T.GGAAAGCAGATCCTGT	CC1
SMC25.T.GGAAAGCTCAGGCAAG	CC1
SMC25.T.GGAATAAAGATATGCA	CC1
SMC25.T.GGACAAGTCTACTATC	CC1
SMC25.T.GGACATTAGAACTGTA	CC1
SMC25.T.GGACATTCAGCTCCGA	CC1
SMC25.T.GGACATTCATGCGCAC	CC1
SMC25.T.GGACGTCCAGGTGCCT	CC1
SMC25.T.GGAGCAAAGGCTAGCA	CC1
SMC25.T.GGAGCAAGTTAAGGGC	CC1
SMC25.T.GGAGCAATCCCTCAGT	CC2
SMC25.T.GGATGTTAGCAATATG	CC1
SMC25.T.GGATTACAGTGTACTC	CC1
SMC25.T.GGCAATTCACGCATCG	CC1
SMC25.T.GGCGACTGTGAGCGAT	CC1
SMC25.T.GGCGACTGTTGTGGAG	CC1
SMC25.T.GGCGTGTTCTGAGTGT	CC1

SMC25.T.GGCTGGTTCGCCATAA	CC1
SMC25.T.GGGAATGTCAAACAAG	CC1
SMC25.T.GGGACCTCATCCCATC	CC1
SMC25.T.GGGAGATGTCTACCTC	CC1
SMC25.T.GGGAGATGTGTGCGTC	CC1
SMC25.T.GGGATGATCTGTCTAT	CC1
SMC25.T.GGGTCTGAGAAAGTGG	CC1
SMC25.T.GGGTCTGCAACACGCC	CC1
SMC25.T.GGTATTGAGCTCCTTC	CC1
SMC25.T.GGTGCGTTCGCGTTTC	CC1
SMC25.T.GTAACGTAGTGGGATC	CC1
SMC25.T.GTAACGTTCAGTCAGT	CC1
SMC25.T.GTAACTGGTGATGCCC	CC1
SMC25.T.GTACGTATCCAAACAC	CC1
SMC25.T.GTATCTTAGTGAACAT	CC1
SMC25.T.GTATCTTGTGCAACTT	CC1
SMC25.T.GTATCTTTCAGTCAGT	CC1
SMC25.T.GTCAAGTAGACATAAC	CC1
SMC25.T.GTCAAGTCACCAGGCT	CC1
SMC25.T.GTCAAGTCATTAGGCT	CC1
SMC25.T.GTCACAACACGGCGTT	CC1
SMC25.T.GTCCTCAGTAAACACA	CC1
SMC25.T.GTCCTCAGTGCCTGCA	CC1
SMC25.T.GTCGTAATCACTTCAT	CC1
SMC25.T.GTCTCGTGTTAAGGGC	CC1
SMC25.T.GTGAAGGCACTAAGTC	CC1
SMC25.T.GTGAAGGTCGATGAGG	CC1
SMC25.T.GTGCATACAGCTCCGA	CC1
SMC25.T.GTGCGGTGTAGGCATG	CC2
SMC25.T.GTGCGGTGTTGTGGAG	CC1
SMC25.T.GTGCTTCAGAGTAATC	CC1
SMC25.T.GTGCTTCCAGTATCTG	CC1
SMC25.T.GTGGGTCAGCGCTTAT	CC1
SMC25.T.GTGTTAGAGGGTTCCC	CC1
SMC25.T.GTGTTAGCAGACACTT	CC1
SMC25.T.GTTAAGCGTCTCTTAT	CC1
SMC25.T.GTTACAGAGAGCCTAG	CC1
SMC25.T.GTTACAGAGGGATCTG	CC1
SMC25.T.GTTACAGTCGCAAACT	CC1
SMC25.T.GTTACAGTCGCCAGCA	CC1
SMC25.T.GTTCGGGAGTACGCGA	CC1
SMC25.T.GTTCTCGAGAGTAATC	CC1
SMC25.T.GTTCTCGCATTACGAC	CC1
SMC25.T.GTTTCTAGTGACAAAT	CC1
SMC25.T.TAAACCGAGTGGGATC	CC1
SMC25.T.TAAGAGAGTTGTGGAG	CC1
SMC25.T.TACACGATCCACGCAG	CC1
SMC25.T.TACAGTGAGAGCTGGT	CC1
SMC25.T.TACCTTAGTAATCGTC	CC1
SMC25.T.TACCTTATCATTTGGG	CC1
SMC25.T.TACGGATTCTCGAGTA	CC1
SMC25.T.TACGGTAAGGGAGTAA	CC1
SMC25.T.TAGACCATCCTGTACC	CC1
SMC25.T.TAGAGCTCACAGGCCT	CC1
SMC25.T.TAGAGCTTCAGTCAGT	CC1
SMC25.T.TAGAGCTTCTCACATT	CC1
SMC25.T.TAGCCGGCAAGAGTCG	CC1
SMC25.T.TAGGCATAGAGTCGGT	CC1

SMC25.T.TAGGCATAGGATGTAT	CC2
SMC25.T.TAGGCATGTTCCACTC	CC1
SMC25.T.TAGTGGTAGATCGGGT	CC1
SMC25.T.TAGTTGGTCAAGAAGT	CC1
SMC25.T.TATGCCCAGTTACGGG	CC1
SMC25.T.TATTACCGTGTTTGTG	CC1
SMC25.T.TATTACCTCACCCGAG	CC1
SMC25.T.TATTACCTCTGGTGTA	CC1
SMC25.T.TCAACGAAGGCAGTCA	CC1
SMC25.T.TCACGAACATCCTTGC	CC1
SMC25.T.TCACGAAGTAAATGAC	CC1
SMC25.T.TCAGCTCCATTAACCG	CC2
SMC25.T.TCATTTGAGATGTTAG	CC1
SMC25.T.TCCCGATGTAGCGTGA	CC1
SMC25.T.TCGAGGCAGAACTCGG	CC1
SMC25.T.TCGCGAGCAAGAGTCG	CC1
SMC25.T.TCGCGAGGTCGAATCT	CC1
SMC25.T.TCGCGTTTCCGAGCCA	CC1
SMC25.T.TCGGGACCATATACGC	CC2
SMC25.T.TCGTACCCACAGACAG	CC1
SMC25.T.TCGTAGACAGCCTGTG	CC1
SMC25.T.TCGTAGATCTTCGAGA	CC1
SMC25.T.TCTATTGTCATTATCC	CC1
SMC25.T.TCTCATAAGGCTCAGA	CC1
SMC25.T.TCTCTAAAGTACGTAA	CC1
SMC25.T.TCTCTAACATCGTCGG	CC1
SMC25.T.TCTGAGACACGTCTCT	CC1
SMC25.T.TCTGAGACATCACGAT	CC1
SMC25.T.TCTGGAACAGTGGAGT	CC1
SMC25.T.TCTTCGGGTCTCCCTA	CC1
SMC25.T.TCTTTCCAGCGTCTAT	CC2
SMC25.T.TCTTTCCGTAGCGTCC	CC1
SMC25.T.TCTTTCCTCTACCAGA	CC1
SMC25.T.TCTTTCCTCTGGCGTG	CC1
SMC25.T.TGACAACAGCGTGAGT	CC1
SMC25.T.TGACAACCACTGTGTA	CC1
SMC25.T.TGACAACGTTTACTCT	CC1
SMC25.T.TGACTTTTCAACTCTT	CC1
SMC25.T.TGAGAGGAGAATTCCC	CC1
SMC25.T.TGAGAGGAGTGGAGTC	CC1
SMC25.T.TGAGAGGTCAACTCTT	CC1
SMC25.T.TGAGCATTCTCTGTCG	CC1
SMC25.T.TGAGCCGTCTTGTTTG	CC1
SMC25.T.TGCGCAGTCTGGTATG	CC1
SMC25.T.TGCGTGGGTCAATACC	CC1
SMC25.T.TGCTACCGTATAATGG	CC1
SMC25.T.TGGACGCGTGGTGTAG	CC1
SMC25.T.TGGCTGGAGATCCTGT	CC1
SMC25.T.TGGGAAGAGACCCACC	CC1
SMC25.T.TGGGAAGCAGGCTCAC	CC1
SMC25.T.TGGTTAGGTTACAGAA	CC1
SMC25.T.TGGTTCCGTTCAGGCC	CC1
SMC25.T.TGTGGTAAGTGGTCCC	CC1
SMC25.T.TGTTCCGTCCTTGCCA	CC1
SMC25.T.TTAACTCTCAAACCGT	CC1
SMC25.T.TTAGGCACACAGAGGT	CC1
SMC25.T.TTAGGCAGTTTGTGTG	CC1
SMC25.T.TTAGTTCCAATAACGA	CC1

SMC25.T.TTCTACATCTTAGAGC	CC1
SMC25.T.TTCTCAAAGGTTCCTA	CC1
SMC25.T.TTCTCAACATAGACTC	CC1
SMC25.T.TTCTCCTGTTAGATGA	CC1
SMC25.T.TTGAACGAGGCGACAT	CC1
SMC25.T.TTGAACGGTATGCTTG	CC1
SMC25.T.TTGGAACCAGGGTATG	CC1
SMC25.T.TTGGAACGTCAACTGT	CC1
SMC25.T.TTGTAGGGTCATATGC	CC1
SMC25.T.TTTGCGCAGACACGAC	CC1
SMC25.T.TTTGCGCCATGGAATA	CC1
SMC25.T.TTTGGTTGTAGGGTAC	CC2
SMC25.T.TTTGTCAAGAGGGATA	CC2

Table S4. The annotation of single cells

cell name	annotation
KUL01.T_AAACCTGGTCTTTCAT	CC2
KUL01.T_AAACGGGTCGGTTAAC	CC2
KUL01.T_AAAGATGGTATAGGGC	CC1
KUL01.T_AAAGATGGTGGCCCTA	CC2
KUL01.T_AAAGCAAGTAAACACA	CC2
KUL01.T_AAAGCAATCACATGCA	CC2
KUL01.T_AAAGTAGTCCGTCATC	CC2
KUL01.T_AAATGCCAGTCGTTTG	CC2
KUL01.T_AAATGCCAGTGACTCT	CC2
KUL01.T_AAATGCCCACAGTCGC	CC2
KUL01.T_AAATGCCTCAAACCAC	CC2
KUL01.T_AAATGCCTCAACCATG	CC2
KUL01.T_AAATGCCTCCCTCAGT	CC2
KUL01.T_AACACGTCACGTCTCT	CC2
KUL01.T_AACACGTCAGGTTTCA	CC2
KUL01.T_AACACGTGTACAGTTC	CC2
KUL01.T_AACACGTGTCATGCAT	CC1
KUL01.T_AACCATGAGCAGCCTC	CC2
KUL01.T_AACCATGGTAAATACG	CC2
KUL01.T_AACCGCGCAATGAATG	CC2
KUL01.T_AACGTTGGTCAAAGAT	CC2
KUL01.T_AACTCAGAGCTAGTTC	CC2
KUL01.T_AACTCTTTCACAAACC	CC2
KUL01.T_AACTGGTCAGTGACAG	CC2
KUL01.T_AACTTTCCACTTACGA	CC2
KUL01.T_AACTTTCCATACCATG	CC2
KUL01.T_AACTTTCGTGTGAAAT	CC2
KUL01.T_AAGACCTAGAGGGATA	CC2
KUL01.T_AAGACCTAGGAGCGTT	CC2
KUL01.T_AAGACCTAGTCATCCA	CC1
KUL01.T_AAGACCTGTACTCTCC	CC2
KUL01.T_AAGGCAGAGAGTAATC	CC2
KUL01.T_AAGGCAGTCAACACAC	CC2
KUL01.T_AAGGCAGTCTGAAAGA	CC2
KUL01.T_AAGTCTGAGGGCATGT	CC1
KUL01.T_AAGTCTGCACTTGGAT	CC2
KUL01.T_AATCCAGAGATGGGTC	CC2
KUL01.T_AATCCAGGTTCTCATT	CC2
KUL01.T_AATCGGTTCACCACCT	CC1
KUL01.T_ACACCAAAGAACAACT	CC2
KUL01.T_ACACCGGGTGCAGTAG	CC2
KUL01.T_ACACCGGTCCCATTAT	CC2
KUL01.T_ACACTGAGTAATCACC	CC2
KUL01.T_ACAGCCGCACCAACCG	CC2
KUL01.T_ACAGCCGCAGCTCCGA	CC1
KUL01.T_ACAGCCGTCTACCTGC	CC1
KUL01.T_ACAGCTAAGGAGTAGA	CC2
KUL01.T_ACAGCTAGTTGTCTTT	CC2
KUL01.T_ACATACGAGAAGGCCT	CC2
KUL01.T_ACATACGAGTCCCACG	CC2
KUL01.T_ACATACGCAGCTGTTA	CC2
KUL01.T_ACATACGTCCACGTTC	CC1
KUL01.T_ACATCAGTCCTATTCA	CC2
KUL01.T_ACATGGTTCGCGGATC	CC1
KUL01.T_ACCCACTGTAGGCATG	CC2
KUL01.T_ACCGTAAGTCAAACTC	CC2

KUL01.T_ACCGTAAGTTACTGAC	CC2
KUL01.T_ACCTTTACACTACAGT	CC1
KUL01.T_ACGAGGACACGGATAG	CC2
KUL01.T_ACGATGTAGTCCAGGA	CC2
KUL01.T_ACGATGTGTCTAGTGT	CC2
KUL01.T_ACGCAGCAGGTGTTAA	CC2
KUL01.T_ACGCAGCTCAGGTTCA	CC2
KUL01.T_ACGCCAGGTTAGTGGG	CC2
KUL01.T_ACGCCAGTCACATACG	CC2
KUL01.T_ACGCCGAAGCCCGAAA	CC1
KUL01.T_ACGGAGAGTGCACCAC	CC2
KUL01.T_ACGGCCAGTTCTGAAC	CC2
KUL01.T_ACGGGCTTCGATAGAA	CC2
KUL01.T_ACGTCAAAGCGAGAAA	CC2
KUL01.T_ACTATCTCACGCATCG	CC2
KUL01.T_ACTATCTCATCACAAC	CC2
KUL01.T_ACTATCTTCTTCTGGC	CC2
KUL01.T_ACTGAACAGGCTACGA	CC2
KUL01.T_ACTGAACGTGACTACT	CC2
KUL01.T_ACTGAGTAGTAAGTAC	CC2
KUL01.T_ACTGAGTCAATGGAAT	CC2
KUL01.T_ACTGAGTCACCGAATT	CC2
KUL01.T_ACTGAGTTCACAACGT	CC2
KUL01.T_ACTGAGTTCGCCATAA	CC2
KUL01.T_ACTGATGGTAAACGCG	CC2
KUL01.T_ACTGATGGTATAAACG	CC2
KUL01.T_ACTGATGGTCTAGCGC	CC2
KUL01.T_ACTGCTCCAAATACAG	CC2
KUL01.T_ACTGCTCCAATCTGCA	CC2
KUL01.T_ACTGCTCCACGCGAAA	CC2
KUL01.T_ACTGCTCGTGACAAAT	CC2
KUL01.T_ACTGCTCGTTCGTGAT	CC2
KUL01.T_ACTGTCCCACTGTTAG	CC2
KUL01.T_ACTGTCCCAGGACGTA	CC2
KUL01.T_ACTTACTCAATCTGCA	CC1
KUL01.T_ACTTACTCACCGTTGG	CC2
KUL01.T_ACTTACTGTGAGGGAG	CC2
KUL01.T_ACTTGTTAGCTATGCT	CC2
KUL01.T_ACTTTCAAGACAAGCC	CC2
KUL01.T_AGAATAGAGACTCGGA	CC2
KUL01.T_AGAATAGCAATACGCT	CC2
KUL01.T_AGAATAGCACAGGAGT	CC2
KUL01.T_AGAATAGGTATAAACG	CC2
KUL01.T_AGAATAGGTCATCCCT	CC2
KUL01.T_AGACGTTGTTGACGTT	CC1
KUL01.T_AGAGCGACAGGATCGA	CC2
KUL01.T_AGAGCGAGTCGAACAG	CC2
KUL01.T_AGAGTGGTCAGTGCAT	CC1
KUL01.T_AGATCTGCAATGGAGC	CC2
KUL01.T_AGATTGCCACGAAACG	CC2
KUL01.T_AGATTGCGTACCGTAT	CC2
KUL01.T_AGATTGCTCACTCCTG	CC1
KUL01.T_AGCAGCCAGAGAGCTC	CC2
KUL01.T_AGCCTAACACACAGAG	CC2
KUL01.T_AGCCTAAGTCGCGGTT	CC2
KUL01.T_AGCGGTCCATAAAGGT	CC2
KUL01.T_AGCGGTCTCTTGAGAC	CC2
KUL01.T_AGCGTATCATATACCG	CC2

KUL01.T_AGCGTCGAGAACTGTA	CC2
KUL01.T_AGCTCTCAGACTGGGT	CC2
KUL01.T_AGCTCTCCACACGCTG	CC1
KUL01.T_AGCTCTCGTCGGATCC	CC1
KUL01.T_AGCTCTCTCGCGTTTC	CC2
KUL01.T_AGCTTGACATTTCACT	CC2
KUL01.T_AGCTTGAGTTCTGAAC	CC1
KUL01.T_AGCTTGATCTCACATT	CC2
KUL01.T_AGGCCACTCAGAGGTG	CC2
KUL01.T_AGGCCACTCCACTCCA	CC2
KUL01.T_AGGGAGTGTTCCGGCA	CC2
KUL01.T_AGGGATGGTGGCTCCA	CC2
KUL01.T_AGGGATGTCTACTCAT	CC2
KUL01.T_AGGGTGATCTCACATT	CC2
KUL01.T_AGGTCCGAGACAGGCT	CC2
KUL01.T_AGGTCCGAGACTTTCG	CC1
KUL01.T_AGGTCCGAGGCTCAGA	CC2
KUL01.T_AGGTCCGTCGGAGCAA	CC2
KUL01.T_AGTAGTCGTGCAACTT	CC2
KUL01.T_AGTAGTCTCAAAGACA	CC2
KUL01.T_AGTCTTTGTCCGCTGA	CC2
KUL01.T_AGTCTTTGTGCATCTA	CC2
KUL01.T_AGTCTTTTCGTGACAT	CC1
KUL01.T_AGTGAGGAGCGATAGC	CC1
KUL01.T_AGTGAGGGTAGCCTAT	CC2
KUL01.T_AGTGAGGTCGGAAATA	CC2
KUL01.T_AGTGTCAGTCTCCACT	CC1
KUL01.T_AGTGTCATCTTGCATT	CC2
KUL01.T_AGTTGGTCAGGGATTG	CC2
KUL01.T_AGTTGGTGTTACGTCA	CC2
KUL01.T_ATAACGCCAGAGTGTG	CC2
KUL01.T_ATAAGAGGTACGCTGC	CC2
KUL01.T_ATAAGAGGTTATCCGA	CC1
KUL01.T_ATAAGAGGTTTGTTGG	CC2
KUL01.T_ATAAGAGTCAAACCGT	CC2
KUL01.T_ATAAGAGTCTCCAACC	CC2
KUL01.T_ATCACGACATTTGCTT	CC2
KUL01.T_ATCACGATCAGTTCGA	CC1
KUL01.T_ATCATCTTCGTGACAT	CC2
KUL01.T_ATCATGGCACGTCAGC	CC2
KUL01.T_ATCATGGGTACAGTGG	CC2
KUL01.T_ATCATGGTCCTAGTGA	CC1
KUL01.T_ATCATGGTCCTGCTTG	CC2
KUL01.T_ATCCACCGTATTCTCT	CC1
KUL01.T_ATCCGAATCACATACG	CC2
KUL01.T_ATCGAGTTCAAAGACA	CC2
KUL01.T_ATCTACTAGCTCCCAG	CC2
KUL01.T_ATCTACTCAAACCTAC	CC2
KUL01.T_ATGCGATAGTGTACTC	CC1
KUL01.T_ATGCGATTCCCGGATG	CC2
KUL01.T_ATGGGAGTCACCTCGT	CC1
KUL01.T_ATGGGAGTCTACTTAC	CC2
KUL01.T_ATGTGTGAGGACAGAA	CC2
KUL01.T_ATTACTCAGTCGATAA	CC2
KUL01.T_ATTATCCCACCGCTAG	CC2
KUL01.T_ATTATCCGTGGCCCTA	CC2
KUL01.T_ATTCTACAGCTGCGAA	CC2
KUL01.T_ATTCTACCACCTGGTG	CC2

KUL01.T_ATTGGACAGATAGCAT	CC2
KUL01.T_ATTGGTGCAAAGGCGT	CC2
KUL01.T_ATTGGTGGTTAGAACA	CC2
KUL01.T_ATTGGTGTCGCATGAT	CC2
KUL01.T_CAACCTCTCAGGTAAA	CC2
KUL01.T_CAACCTCTCTCGAGTA	CC2
KUL01.T_CACACAACAGTTCATG	CC2
KUL01.T_CACACAAGTAAGTTCC	CC1
KUL01.T_CACACAAGTGTGAATA	CC2
KUL01.T_CACACCTCAATGGTCT	CC2
KUL01.T_CACACTCTCCGCAAGC	CC2
KUL01.T_CACAGTAGTCCTCTTG	CC2
KUL01.T_CACATTTCAATGGAGC	CC2
KUL01.T_CACCACTGTAGCGCAA	CC2
KUL01.T_CACCACTGTGGTACAG	CC2
KUL01.T_CACCAGGAGCGATCCC	CC2
KUL01.T_CACCTTGTCTATGTGG	CC2
KUL01.T_CACTCCACACGTAAGG	CC2
KUL01.T_CAGAGAGGTTTACTCT	CC2
KUL01.T_CAGATCAGTCATATCG	CC2
KUL01.T_CAGATCATCTCTTATG	CC2
KUL01.T_CAGCAGCCATGGGAAC	CC2
KUL01.T_CAGCAGCTCCTGCAGG	CC2
KUL01.T_CAGCATAGTCTCTTAT	CC2
KUL01.T_CAGCCGAAGAAACGCC	CC2
KUL01.T_CAGCCGAGTTATGCGT	CC1
KUL01.T_CAGCCGATCGTAGGTT	CC2
KUL01.T_CAGCGACAGAGCTATA	CC1
KUL01.T_CAGCGACGTAGCACGA	CC2
KUL01.T_CAGCGACGTCTCTTAT	CC2
KUL01.T_CAGCGACTCACCACCT	CC1
KUL01.T_CAGCGACTCGATGAGG	CC2
KUL01.T_CAGCGACTCTCAACTT	CC2
KUL01.T_CAGCTAAGTCTAACGT	CC2
KUL01.T_CAGCTAAGTGTTCGAT	CC2
KUL01.T_CAGCTAATCTACCTGC	CC2
KUL01.T_CAGCTGGCAAGGGTCA	CC2
KUL01.T_CAGCTGGCAGAGTGTG	CC2
KUL01.T_CAGTAACAGCGGCTTC	CC2
KUL01.T_CAGTAACAGGCGATAC	CC2
KUL01.T_CATATGGGTGTTCTTT	CC2
KUL01.T_CATATGGTCCGTTGTC	CC2
KUL01.T_CATATGGTCGCCAGCA	CC2
KUL01.T_CATATGGTCTTTAGTC	CC2
KUL01.T_CATATTCGTTACTGAC	CC2
KUL01.T_CATCAAGCAGGTCGTC	CC2
KUL01.T_CATCAAGCATCGGAAG	CC2
KUL01.T_CATCAGAGTAAGAGGA	CC2
KUL01.T_CATCCACGTGTTCTTT	CC2
KUL01.T_CATCCACGTTCATGGT	CC2
KUL01.T_CATCGAAGTATAGGGC	CC2
KUL01.T_CATGACAAGGCTAGGT	CC2
KUL01.T_CATGACAAGTCGTACT	CC2
KUL01.T_CATGACAAGTGTACCT	CC2
KUL01.T_CATGCCTGTTATTCTC	CC2
KUL01.T_CATGGCGCACCATCCT	CC2
KUL01.T_CATTATCCAGTAAGCG	CC2
KUL01.T_CCAATCCAGTGCCAGA	CC2

KUL01.T_CCACCTAAGCGTAATA	CC1
KUL01.T_CCACTACAGACGACGT	CC2
KUL01.T_CCACTACAGCGCCTCA	CC2
KUL01.T_CCACTACGTATGAAAC	CC2
KUL01.T_CCACTACTCGTCACGG	CC2
KUL01.T_CCAGCGAAGAAGCCCA	CC1
KUL01.T_CCAGCGAGTAGCCTCG	CC2
KUL01.T_CCATGTCTCGGTCCGA	CC2
KUL01.T_CCATTCGAGCTAACTC	CC2
KUL01.T_CCATTCGCACCGATAT	CC2
KUL01.T_CCATTCGTCACTGGGC	CC2
KUL01.T_CCCAGTTAGAATAGGG	CC1
KUL01.T_CCCAGTTAGCTAGTCT	CC2
KUL01.T_CCCAGTTGTCCGTCAG	CC2
KUL01.T_CCCATACTCTCACATT	CC2
KUL01.T_CCCTCCTAGGCTAGGT	CC2
KUL01.T_CCCTCCTTCTTGTTTG	CC2
KUL01.T_CCGGGATAGTGATCGG	CC2
KUL01.T_CCGGGATAGTTTCCTT	CC2
KUL01.T_CCGGTAGCATGGGAAC	CC2
KUL01.T_CCGGTAGTCCACGAAT	CC2
KUL01.T_CCGTACTCACATCCGG	CC2
KUL01.T_CCGTTCAAGAGGACGG	CC2
KUL01.T_CCTAAAGGTCGACTGC	CC2
KUL01.T_CCTAAAGGTTCGTGAT	CC2
KUL01.T_CCTAAAGTCTTAACCT	CC2
KUL01.T_CCTACACCATCGGGTC	CC1
KUL01.T_CCTACCAGTCGGCTCA	CC2
KUL01.T_CCTACCAGTTCAGACT	CC2
KUL01.T_CCTAGCTAGTGAATTG	CC2
KUL01.T_CCTAGCTAGTGTACTC	CC2
KUL01.T_CCTAGCTGTATATCCG	CC2
KUL01.T_CCTAGCTTCATAGCAC	CC2
KUL01.T_CCTCTGAAGAGTTGGC	CC2
KUL01.T_CCTCTGATCCTAGAAC	CC2
KUL01.T_CCTTACGCAGGGTATG	CC2
KUL01.T_CCTTACGCATACTACG	CC2
KUL01.T_CCTTACGGTAGAAGGA	CC2
KUL01.T_CCTTACGTCGGCCGAT	CC2
KUL01.T_CCTTCCCTCTAACTCT	CC2
KUL01.T_CCTTCGAAGGACTGGT	CC2
KUL01.T_CCTTCGAGTGAGTATA	CC2
KUL01.T_CCTTCGAGTTCGAATC	CC1
KUL01.T_CCTTTCTCAGCGTCCA	CC2
KUL01.T_CCTTTCTGTCCGAAGA	CC2
KUL01.T_CCTTTCTGTGTGGCTC	CC2
KUL01.T_CGAACATAGTCAAGCG	CC2
KUL01.T_CGAACATGTGCATCTA	CC2
KUL01.T_CGAATGTCAATACGCT	CC2
KUL01.T_CGACTTCAGATATGCA	CC2
KUL01.T_CGACTTCAGGCTAGAC	CC2
KUL01.T_CGACTTCTCTATGTGG	CC2
KUL01.T_CGAGAAGCAGGAATCG	CC2
KUL01.T_CGAGCACAGCCACGCT	CC2
KUL01.T_CGAGCACTCGTTTAGG	CC2
KUL01.T_CGAGCCACAGCAGTTT	CC2
KUL01.T_CGAGCCACAGTATGCT	CC2
KUL01.T_CGAGCCATCGACAGCC	CC2

KUL01.T_CGATCGGCAATCAGAA	CC2
KUL01.T_CGATCGGTCCTTGCCA	CC2
KUL01.T_CGATGGCAGATCGGGT	CC2
KUL01.T_CGATTGAAGCTCCCAG	CC2
KUL01.T_CGATTGATCCACGAAT	CC2
KUL01.T_CGATTGATCTGGCGAC	CC2
KUL01.T_CGCCAAGAGTCCTCCT	CC2
KUL01.T_CGCGGTATCAACTCTT	CC2
KUL01.T_CGCGTTTAGCGCTTAT	CC2
KUL01.T_CGCTATCTCACCCTCA	CC1
KUL01.T_CGCTGGACATAGTAAG	CC2
KUL01.T_CGCTGGAGTGGTCTCG	CC2
KUL01.T_CGCTGGATCAGCCTAA	CC2
KUL01.T_CGCTTCAGTCGAGTTT	CC2
KUL01.T_CGGACACCAAGGACAC	CC2
KUL01.T_CGGACACGTTATGCGT	CC1
KUL01.T_CGGACACTCAGCAACT	CC1
KUL01.T_CGGACGTAGTGACTCT	CC2
KUL01.T_CGGACGTCAGTACACT	CC2
KUL01.T_CGGACGTTCGCTTAGA	CC2
KUL01.T_CGGAGCTCAATGCCAT	CC2
KUL01.T_CGGAGCTGTAATCGTC	CC2
KUL01.T_CGGAGCTGTTGGGACA	CC2
KUL01.T_CGGAGCTTCGCCATAA	CC1
KUL01.T_CGGAGTCGTAATCGTC	CC2
KUL01.T_CGGCTAGTCCTCATTA	CC2
KUL01.T_CGGGTCACAAGAAAGG	CC2
KUL01.T_CGGGTCATCGGTCCGA	CC1
KUL01.T_CGGTTAAAGATGTAAC	CC2
KUL01.T_CGGTTAATCTGACCTC	CC2
KUL01.T_CGTAGCGGTAGCGATG	CC2
KUL01.T_CGTAGCGTCAGGCCCA	CC2
KUL01.T_CGTAGGCGTCGACTAT	CC2
KUL01.T_CGTAGGCGTCGACTGC	CC2
KUL01.T_CGTAGGCTCAGTTTGG	CC2
KUL01.T_CGTCACTCATCCTTGC	CC2
KUL01.T_CGTCTACAGATGAGAG	CC2
KUL01.T_CGTGAGCAGACCGGAT	CC2
KUL01.T_CGTGTAAAGCGAAGGG	CC2
KUL01.T_CGTGTCTCACGGACAA	CC2
KUL01.T_CGTTAGATCAAGATCC	CC2
KUL01.T_CGTTCTGGTATAATGG	CC2
KUL01.T_CGTTGGGAGCCGTCGT	CC2
KUL01.T_CGTTGGGGTCGCTTTC	CC2
KUL01.T_CTAACTTAGCTCTCGG	CC2
KUL01.T_CTAAGACAGTGGAGTC	CC2
KUL01.T_CTAATGGAGGTGCACA	CC1
KUL01.T_CTACATTCAATCTACG	CC2
KUL01.T_CTACATTCAGTCACTA	CC2
KUL01.T_CTACCCATCGACCAGC	CC1
KUL01.T_CTACGTCCAGGATCGA	CC2
KUL01.T_CTAGAGTCATACGCTA	CC2
KUL01.T_CTAGAGTGTACTCAAC	CC2
KUL01.T_CTAGTGAAGCTACCTA	CC1
KUL01.T_CTAGTGAGTATGAATG	CC2
KUL01.T_CTCACACGTAGCCTAT	CC2
KUL01.T_CTCACACGTCCTGCTT	CC2
KUL01.T_CTCACACGTGCACCAC	CC2

KUL01.T_CTCACACGTGTATGGG	CC2
KUL01.T_CTCAGAAAGTGGTAAT	CC1
KUL01.T_CTCATTACATACCATG	CC2
KUL01.T_CTCGAAACAGTAACGG	CC2
KUL01.T_CTCGAAAGTGCAGGTA	CC2
KUL01.T_CTCGAAATCAACTCTT	CC2
KUL01.T_CTCGAAATCTAACTGG	CC2
KUL01.T_CTCGAGGGTAGCTCCG	CC2
KUL01.T_CTCGAGGGTTAAGATG	CC2
KUL01.T_CTCGAGGTCATGGTCA	CC2
KUL01.T_CTCGGAGAGCGATGAC	CC2
KUL01.T_CTCGGAGCACTAGTAC	CC2
KUL01.T_CTCGGAGTCCCGGATG	CC2
KUL01.T_CTCGGGACAATCGGTT	CC1
KUL01.T_CTCGGGAGTACGAAAT	CC2
KUL01.T_CTCGTACCAATCCGAT	CC2
KUL01.T_CTCGTCAAGGCTAGGT	CC2
KUL01.T_CTCTAATAGGCGATAC	CC2
KUL01.T_CTCTACGCAGACAAAT	CC2
KUL01.T_CTCTACGGTTCCCTTG	CC2
KUL01.T_CTCTACGTCATTTGGG	CC2
KUL01.T_CTGAAACGTATATCCG	CC2
KUL01.T_CTGAAACTCCAGTAGT	CC2
KUL01.T_CTGAAGTAGCGGCTTC	CC1
KUL01.T_CTGATAGCAGTACACT	CC1
KUL01.T_CTGATCCCATTGAGCT	CC2
KUL01.T_CTGATCCTCTTGTACT	CC2
KUL01.T_CTGCCTACATCGGGTC	CC2
KUL01.T_CTGCGGAAGTGGTAAT	CC2
KUL01.T_CTGCGGAGTGGTCCGT	CC2
KUL01.T_CTGCGGAGTTGATTGC	CC2
KUL01.T_CTGCGGATCCCTTGTG	CC1
KUL01.T_CTGTTTAAGCGCCTCA	CC2
KUL01.T_CTGTTTAAGTGGCACA	CC2
KUL01.T_CTGTTTACAGGACGTA	CC1
KUL01.T_CTTAACTCAGCGTAAG	CC2
KUL01.T_CTTACCGCAAGCGATG	CC2
KUL01.T_CTTACCGTCAACGGCC	CC2
KUL01.T_CTTACCGTCCCGACTT	CC2
KUL01.T_CTTACCGTCTACCTGC	CC2
KUL01.T_CTTAGGAAGAGTAAGG	CC2
KUL01.T_CTTCTCTAGTGTACCT	CC1
KUL01.T_CTTCTCTGTGCCTGGT	CC2
KUL01.T_CTTCTCTGTGTAAGTA	CC2
KUL01.T_CTTCTCTTCATCGATG	CC2
KUL01.T_CTTCTCTTCCGTACAA	CC2
KUL01.T_CTTGGCTAGACTGGGT	CC2
KUL01.T_CTTTGCGCACGAAAGC	CC2
KUL01.T_CTTTGCGGTCTTGATG	CC2
KUL01.T_GAAACTCAGGGATGGG	CC2
KUL01.T_GAAACTCCACCAGGCT	CC2
KUL01.T_GAAATGAAGGTACTCT	CC2
KUL01.T_GAACATCGTCCCGACA	CC2
KUL01.T_GAACCTAAGTAAGTAC	CC2
KUL01.T_GAACGGACAGCGTAAG	CC2
KUL01.T_GAACGGATCTGATTCT	CC1
KUL01.T_GAAGCAGAGAGTAAGG	CC2
KUL01.T_GAATAAGCAGTAGAGC	CC2

KUL01.T_GAATAAGGTGAGGCTA	CC2
KUL01.T_GAATGAAAGGTGCACA	CC2
KUL01.T_GAATGAACATGTCGAT	CC2
KUL01.T_GACACGCAGATGCCTT	CC2
KUL01.T_GACACGCAGTTAACGA	CC2
KUL01.T_GACACGCTCGATCCCT	CC2
KUL01.T_GACAGAGAGCTGATAA	CC2
KUL01.T_GACAGAGCAGGGTATG	CC2
KUL01.T_GACAGAGGTAGTAGTA	CC2
KUL01.T_GACAGAGTCACGCGGT	CC2
KUL01.T_GACCTGGAGGCAATTA	CC2
KUL01.T_GACGCGTAGAGGTTGC	CC2
KUL01.T_GACGCGTCACTACAGT	CC2
KUL01.T_GACGCGTGTGTCGCTG	CC2
KUL01.T_GACGCGTTCGCGATCG	CC2
KUL01.T_GACGTGCCATCGATGT	CC2
KUL01.T_GACGTTATCCTGCCAT	CC2
KUL01.T_GACTACACATCCAACA	CC2
KUL01.T_GACTACAGTACATGTC	CC2
KUL01.T_GACTGCGGTCGAAAGC	CC2
KUL01.T_GACTGCGTCTTATCTG	CC1
KUL01.T_GAGCAGACAGACACTT	CC1
KUL01.T_GAGCAGATCTGTTGAG	CC2
KUL01.T_GAGGTGAGTACATGTC	CC2
KUL01.T_GAGTCCGAGGGTGTTG	CC2
KUL01.T_GAGTCCGGTGAAATCA	CC2
KUL01.T_GAGTCCGGTTGCGTTA	CC2
KUL01.T_GATCGATGTCAGAGGT	CC2
KUL01.T_GATCGCGAGTGGAGAA	CC2
KUL01.T_GATCGCGTCTCTAGGA	CC2
KUL01.T_GATCGTAAGGTGATTA	CC1
KUL01.T_GATCGTATCCAACCAA	CC2
KUL01.T_GATCTAGAGCGTCTAT	CC2
KUL01.T_GATGAGGGTAGCTAAA	CC2
KUL01.T_GATGAGGTCAGTGTTG	CC2
KUL01.T_GATGCTAAGATGCCAG	CC2
KUL01.T_GATGCTACAAGCTGGA	CC2
KUL01.T_GATTCAGAGTGTCTCA	CC2
KUL01.T_GCAATCACAGCATGAG	CC1
KUL01.T_GCAATCAGTACTTGAC	CC2
KUL01.T_GCAATCATCCAAGTAC	CC2
KUL01.T_GCACATAAGCTGGAAC	CC2
KUL01.T_GCACATACAAACAACA	CC2
KUL01.T_GCACATAGTAGCCTAT	CC2
KUL01.T_GCACATAGTCTTGCGG	CC2
KUL01.T_GCACATATCGAACTGT	CC2
KUL01.T_GCACTCTAGTGCCATT	CC2
KUL01.T_GCAGCCAGTAGAGTGC	CC2
KUL01.T_GCATACACAGGTGGAT	CC1
KUL01.T_GCATACATCCGCATCT	CC2
KUL01.T_GCATGATAGATGTAAC	CC2
KUL01.T_GCATGCGAGCTGAACG	CC2
KUL01.T_GCATGCGGTTTCGCTC	CC2
KUL01.T_GCATGTAGTCGAACAG	CC2
KUL01.T_GCATGTAGTGTCAATC	CC2
KUL01.T_GCCAAATAGCAAATCA	CC2
KUL01.T_GCCAAATAGCTGAACG	CC2
KUL01.T_GCCAAATCACTAGTAC	CC2

KUL01.T_GCGACCAGTAAGAGGA	CC2
KUL01.T_GCGAGAACATCGGACC	CC2
KUL01.T_GCGCAACAGTAGATGT	CC1
KUL01.T_GCGCAACTCGGCATCG	CC2
KUL01.T_GCGCCAAAGTTACCCA	CC2
KUL01.T_GCGCCAACAGAGCCAA	CC1
KUL01.T_GCGCGATAGAGCTGGT	CC2
KUL01.T_GCGCGATTCGAATGGG	CC2
KUL01.T_GCGGGTTAGCTGAACG	CC2
KUL01.T_GCGGGTTTCAGCGATT	CC2
KUL01.T_GCTCCTAAGTGAACAT	CC1
KUL01.T_GCTCCTAGTTTCCACC	CC2
KUL01.T_GCTCTGTGTCATTAGC	CC1
KUL01.T_GCTCTGTGTGCACTTA	CC1
KUL01.T_GCTGCAGCAGGAATGC	CC2
KUL01.T_GCTGCAGGTCGACTAT	CC2
KUL01.T_GCTGCAGGTCTTTCAT	CC2
KUL01.T_GCTGCAGGTTATGCGT	CC2
KUL01.T_GCTGCGACACTGAAGG	CC2
KUL01.T_GCTGCGAGTCTCGTTC	CC2
KUL01.T_GCTGCGAGTGAAGGCT	CC2
KUL01.T_GCTGCGATCGCACTCT	CC2
KUL01.T_GCTGCTTCAGCATGAG	CC2
KUL01.T_GCTGCTTTCTTCCTTC	CC2
KUL01.T_GCTTCCAAGTATCTCG	CC2
KUL01.T_GCTTCCATCCACGTTC	CC2
KUL01.T_GCTTGAAAGTGAAGAG	CC2
KUL01.T_GCTTGAACACCACCAG	CC2
KUL01.T_GCTTGAACAGATCTGT	CC2
KUL01.T_GCTTGAAGTAGCGCAA	CC1
KUL01.T_GGAACTTCATAAAGGT	CC2
KUL01.T_GGAACTTTCTGTCAAG	CC2
KUL01.T_GGAATAACAGCGATCC	CC2
KUL01.T_GGAATAAGTATTAGCC	CC2
KUL01.T_GGAATAATCTTGCCGT	CC2
KUL01.T_GGACAAGCATCCTTGC	CC2
KUL01.T_GGACAGACAAAGGAAG	CC2
KUL01.T_GGACAGATCGGGAGTA	CC1
KUL01.T_GGACATTAGTGTCTCA	CC2
KUL01.T_GGACGTCCACGGCCAT	CC2
KUL01.T_GGACGTCCAGTCACTA	CC2
KUL01.T_GGAGCAAAGATCGGGT	CC1
KUL01.T_GGAGCAATCGTGTAGT	CC2
KUL01.T_GGATGTTAGATGTGTA	CC2
KUL01.T_GGATGTTTCCTTCAAT	CC1
KUL01.T_GGATTACGTTGATTGC	CC2
KUL01.T_GGCAATTGTGACAAAT	CC2
KUL01.T_GGCAATTTCTAACTTC	CC2
KUL01.T_GGCGACTAGGGCACTA	CC1
KUL01.T_GGCGTGTCAGACGCAA	CC1
KUL01.T_GGCTCGATCCTCAATT	CC2
KUL01.T_GGCTGGTCACCAGCAC	CC2
KUL01.T_GGCTGGTCACGTCTCT	CC2
KUL01.T_GGGAATGAGTCCAGGA	CC1
KUL01.T_GGGAATGGTGTCAATC	CC2
KUL01.T_GGGACCTGTGTAATGA	CC2
KUL01.T_GGGAGATTCGTGTAGT	CC1
KUL01.T_GGGATGAAGCAGGCTA	CC2

KUL01.T_GGGATGACAGACGCTC	CC2
KUL01.T_GGGATGATCAGGTTCA	CC2
KUL01.T_GGGATGATCCGCGCAA	CC2
KUL01.T_GGGCACTAGGTGTTAA	CC2
KUL01.T_GGGCACTAGGTTCCTA	CC2
KUL01.T_GGGCACTAGTTCCACA	CC2
KUL01.T_GGGCATCGTTAGGGTG	CC2
KUL01.T_GGGTCTGGTTTGCATG	CC2
KUL01.T_GGGTCTGTCTTGAGGT	CC2
KUL01.T_GGGTCTGTCTTTAGGG	CC2
KUL01.T_GGGTTGCGTTATTCTC	CC2
KUL01.T_GGTATTGAGACTTGAA	CC2
KUL01.T_GGTATTGGTATTAGCC	CC2
KUL01.T_GGTGAAGGTCGCGAAA	CC2
KUL01.T_GGTGCGTAGACTAGGC	CC2
KUL01.T_GGTGCGTAGAGAGCTC	CC2
KUL01.T_GGTGCGTAGGTGGGTT	CC2
KUL01.T_GGTGTTAAGATGTGGC	CC2
KUL01.T_GTAACTGGTAACGCGA	CC2
KUL01.T_GTACGTAAGGTGATTA	CC2
KUL01.T_GTACGTACAGCTGTGC	CC2
KUL01.T_GTACTCCAGCGAGAAA	CC2
KUL01.T_GTACTTTTCTCGGACG	CC2
KUL01.T_GTAGGCCAGGTGATTA	CC2
KUL01.T_GTATTCTTCATCGCTC	CC2
KUL01.T_GTCAAGTGTATTAGCC	CC1
KUL01.T_GTCAAGTGTTATGCGT	CC2
KUL01.T_GTCAAGTTCACGCATA	CC2
KUL01.T_GTCACAAAGAGAGCTC	CC2
KUL01.T_GTCACGGAGGAGCGAG	CC2
KUL01.T_GTCACGGGTAAACCTC	CC2
KUL01.T_GTCACGGGTAGCTAAA	CC2
KUL01.T_GTCATTTGTTGATTGC	CC2
KUL01.T_GTCCTCAAGGCTCATT	CC2
KUL01.T_GTCCTCAAGTACGTAA	CC1
KUL01.T_GTCCTCAGTATAGGGC	CC2
KUL01.T_GTCCTCATCATAAAGG	CC2
KUL01.T_GTCCTCATCGCAAGCC	CC2
KUL01.T_GTCGGGTCAAACTGCT	CC2
KUL01.T_GTCGGGTTCGTTACAG	CC2
KUL01.T_GTCGTAACAGGTGGAT	CC2
KUL01.T_GTCGTAAGTGTTGGGA	CC2
KUL01.T_GTGAAGGAGTATGACA	CC2
KUL01.T_GTGAAGGCAGTCAGAG	CC2
KUL01.T_GTGAAGGTCCGCGTTT	CC2
KUL01.T_GTGCAGCCACGAGGTA	CC2
KUL01.T_GTGCATAAGAGACTAT	CC2
KUL01.T_GTGCGGTAGCGTAGTG	CC2
KUL01.T_GTGTGCGAGGATCGCA	CC2
KUL01.T_GTGTGCGGTCTCATCC	CC2
KUL01.T_GTGTGCGGTTGATTGC	CC2
KUL01.T_GTGTGCGGTTGCGTTA	CC2
KUL01.T_GTGTTAGGTCAAAGAT	CC2
KUL01.T_GTGTTAGGTTAAAGAC	CC1
KUL01.T_GTTAAGCTCGGCATCG	CC2
KUL01.T_GTTACAGTCGTCACGG	CC2
KUL01.T_GTTACAGTCTTCATGT	CC2
KUL01.T_GTTCTCGCAAGCGCTC	CC2

KUL01.T_TAAACCGCATGAACCT	CC2
KUL01.T_TAAACCGGTCTACCTC	CC1
KUL01.T_TAAACCGGTTGCGTTA	CC2
KUL01.T_TAAGAGAAGGTGATTA	CC2
KUL01.T_TAAGAGAGTAAATGAC	CC2
KUL01.T_TAAGCGTAGCGATATA	CC1
KUL01.T_TAAGCGTCAGTGAGTG	CC2
KUL01.T_TAAGCGTGTTTGACTG	CC2
KUL01.T_TAAGTGCCATTAACCG	CC2
KUL01.T_TAAGTGCTCTCGATGA	CC2
KUL01.T_TACAGTGCAAGTTCTG	CC1
KUL01.T_TACCTATTCATCACCC	CC2
KUL01.T_TACCTTAAGATCGGGT	CC2
KUL01.T_TACCTTACAGACAAGC	CC2
KUL01.T_TACCTTATCCATGAGT	CC2
KUL01.T_TACCTTATCTTTAGTC	CC1
KUL01.T_TACGGATCAACTGCTA	CC2
KUL01.T_TACGGATCAAGACGTG	CC2
KUL01.T_TACGGATGTACAGCAG	CC2
KUL01.T_TACGGGCCACGGTGTC	CC2
KUL01.T_TACGGTAAGAAACGAG	CC2
KUL01.T_TACTCGCTCCGCGCAA	CC2
KUL01.T_TACTTACCAACGATCT	CC2
KUL01.T_TAGACCATCGCACTCT	CC2
KUL01.T_TAGCCGGGTAAATACG	CC2
KUL01.T_TAGCCGGGTCGCGAAA	CC2
KUL01.T_TAGGCATAGACTACAA	CC2
KUL01.T_TAGGCATGTCGCTTTC	CC2
KUL01.T_TAGTGGTCAAGGACAC	CC2
KUL01.T_TAGTTGGAGCCTCGTG	CC2
KUL01.T_TAGTTGGTCAGTTGAC	CC2
KUL01.T_TATCAGGAGGCAAAGA	CC2
KUL01.T_TATCAGGTCACGCATA	CC2
KUL01.T_TATGCCCGTGCACGAA	CC2
KUL01.T_TATTACCAGGCATGTG	CC2
KUL01.T_TATTACCCAGGAATGC	CC2
KUL01.T_TCAACGAGTGACTACT	CC2
KUL01.T_TCAACGAGTTATTCTC	CC2
KUL01.T_TCAATCTGTTAAAGAC	CC2
KUL01.T_TCAATCTGTTTCGCTC	CC2
KUL01.T_TCACGAAAGCAGATCG	CC2
KUL01.T_TCAGCAACAGCCAGAA	CC2
KUL01.T_TCAGCTCAGACCTTTG	CC2
KUL01.T_TCAGCTCAGCTGAACG	CC2
KUL01.T_TCAGCTCGTATCTGCA	CC2
KUL01.T_TCAGCTCTCTAGAGTC	CC2
KUL01.T_TCAGGATCAGCATACT	CC2
KUL01.T_TCAGGTACATAAAGGT	CC2
KUL01.T_TCAGGTATCAAGATCC	CC2
KUL01.T_TCATTACCAGCCTTTC	CC2
KUL01.T_TCATTACTCTGGAGCC	CC2
KUL01.T_TCATTTGCAGGATTGG	CC2
KUL01.T_TCCACACAGTACACCT	CC2
KUL01.T_TCCACACGTAGGGTAC	CC2
KUL01.T_TCCCGATTCTGCCCTA	CC2
KUL01.T_TCGAGGCAGGCGATAC	CC2
KUL01.T_TCGCGAGAGATGTTAG	CC2
KUL01.T_TCGCGAGAGTAGATGT	CC2

KUL01.T_TCGCGAGTCACTCCTG	CC2
KUL01.T_TCGCGTTAGAACAATC	CC2
KUL01.T_TCGCGTTAGTGGACGT	CC1
KUL01.T_TCGGTAACAGTCACTA	CC2
KUL01.T_TCGTACCCACCACGTG	CC2
KUL01.T_TCGTAGAAGCTCAACT	CC2
KUL01.T_TCGTAGACACCAGTTA	CC2
KUL01.T_TCTCATACAATCTGCA	CC2
KUL01.T_TCTCATAGTCATCCCT	CC2
KUL01.T_TCTCATATCTGGCGAC	CC2
KUL01.T_TCTCTAAAGAAGGGTA	CC2
KUL01.T_TCTCTAAAGACAAGCC	CC1
KUL01.T_TCTCTAAAGATGGGTC	CC2
KUL01.T_TCTCTAATCTTGACGA	CC2
KUL01.T_TCTGAGATCAAAGTAG	CC2
KUL01.T_TCTTTCCGTCGAACAG	CC2
KUL01.T_TGACAACCAGCTGTTA	CC2
KUL01.T_TGACTAGTCGACCAGC	CC2
KUL01.T_TGACTTTGTTCCATGA	CC2
KUL01.T_TGACTTTTCGCCCTTA	CC2
KUL01.T_TGAGAGGAGGACGAAA	CC2
KUL01.T_TGAGAGGCAAGACGTG	CC2
KUL01.T_TGAGCATAGATACACA	CC2
KUL01.T_TGAGCATAGTGACATA	CC2
KUL01.T_TGAGCATCATCATCCC	CC2
KUL01.T_TGAGCCGCATTGGGCC	CC2
KUL01.T_TGAGCCGGTACTTCTT	CC2
KUL01.T_TGAGGGAAGTACTTGC	CC1
KUL01.T_TGAGGGATCACCGTAA	CC2
KUL01.T_TGATTTCAGGGATCTG	CC2
KUL01.T_TGATTTCGTTCGTTGA	CC1
KUL01.T_TGCACCTAGCCCGAAA	CC2
KUL01.T_TGCCCATTCCGCATAA	CC2
KUL01.T_TGCCCATTCCTTTACA	CC2
KUL01.T_TGCCCTACACAAGCCC	CC1
KUL01.T_TGCGCAGAGTAGGTGC	CC2
KUL01.T_TGCGCAGTCATAACCG	CC2
KUL01.T_TGCGCAGTCGGTCCGA	CC2
KUL01.T_TGCGGGTGTCCGAGTC	CC2
KUL01.T_TGCGTGGTCGGTTAAC	CC1
KUL01.T_TGCTACCAGCCAGTAG	CC2
KUL01.T_TGCTACCGTGTCTGAT	CC2
KUL01.T_TGCTACCTCCATGAGT	CC2
KUL01.T_TGCTGCTAGGTGATAT	CC2
KUL01.T_TGCTGCTGTGAAGGCT	CC1
KUL01.T_TGCTGCTGTGTTTGTG	CC2
KUL01.T_TGGACGCGTGACGGTA	CC2
KUL01.T_TGGACGCGTTACCAGT	CC2
KUL01.T_TGGCCAGGTGCGCTTG	CC2
KUL01.T_TGGCTGGCACATCCGG	CC2
KUL01.T_TGGCTGGGTTACGGAG	CC2
KUL01.T_TGGGAAGCAAGAAGAG	CC2
KUL01.T_TGGGAAGGTCCAAGTT	CC2
KUL01.T_TGGGCGTAGAGTCGGT	CC2
KUL01.T_TGGGCGTCACGGCCAT	CC2
KUL01.T_TGGGCGTTCTTTCCTC	CC2
KUL01.T_TGGTTAGGTTCATGGT	CC1
KUL01.T_TGGTTCCGTACTCGCG	CC2

KUL01.T_TGTCCCACAAGTAGTA	CC2
KUL01.T_TGTCCCAGTGGTACAG	CC2
KUL01.T_TGTCCCATCCGTCATC	CC2
KUL01.T_TGTGTTTGTATCTGCA	CC2
KUL01.T_TGTTCCGAGATCCCAT	CC2
KUL01.T_TGTTCCGCAGACAGGT	CC2
KUL01.T_TGTTCCGGTTACGGAG	CC2
KUL01.T_TTAACTCCAGGTCCAC	CC1
KUL01.T_TTAGTTCAGTGCCATT	CC2
KUL01.T_TTCGAAGAGTGAAGTT	CC2
KUL01.T_TTCGAAGGTCCAAGTT	CC2
KUL01.T_TTCGAAGTCTTCGGTC	CC2
KUL01.T_TTCTCCTAGCACCGCT	CC2
KUL01.T_TTCTCCTCATTGCGGC	CC2
KUL01.T_TTGAACGGTCGACTGC	CC2
KUL01.T_TTGACTTAGATATACG	CC2
KUL01.T_TTGACTTTCAACACTG	CC2
KUL01.T_TTGCGTCTCTTGTTTG	CC2
KUL01.T_TTGGCAATCACGACTA	CC2
KUL01.T_TTGGCAATCATGTAGC	CC2
KUL01.T_TTGTAGGAGAGTAATC	CC1
KUL01.T_TTGTAGGAGCGTCTAT	CC2
KUL01.T_TTGTAGGCAGGACCCT	CC2
KUL01.T_TTTACTGCACCAGATT	CC2
KUL01.T_TTTACTGTCTCGTATT	CC2
KUL01.T_TTTATGCCAGCGTAAG	CC2
KUL01.T_TTTATGCTCCACTCCA	CC2
KUL01.T_TTTCCTCAGAGAGCTC	CC2
KUL01.T_TTTCCTCAGCGTTGCC	CC2
KUL01.T_TTTCCTCCATCAGTCA	CC1
KUL01.T_TTTCCTCTCACCGTAA	CC2
KUL01.T_TTTCCTCTCAGTGCAT	CC2
KUL01.T_TTTGCGCGTGTGGTTT	CC2
KUL01.T_TTTGGTTAGTTGCAGG	CC2
KUL01.T_TTTGTCAAGGAGTAGA	CC2
KUL01.T_TTTGTCATCCGAAGAG	CC2
KUL19.T_AAACCTGGTTCAACCA	CC1
KUL19.T_AAACGGGAGCTAGTGG	CC1
KUL19.T_AAACGGGAGGCACATG	CC1
KUL19.T_AAACGGGTCTTGCATT	CC1
KUL19.T_AAAGATGAGCTGAAAT	CC1
KUL19.T_AAAGCAAAGAGCTGGT	CC1
KUL19.T_AAAGCAACATGCCACG	CC1
KUL19.T_AAATGCCCACCCTATC	CC1
KUL19.T_AACCATGCAAGCTGGA	CC1
KUL19.T_AACCATGCATGACATC	CC1
KUL19.T_AACCGCGGTCTTCTCG	CC1
KUL19.T_AACGTTGTCCGTAGTA	CC1
KUL19.T_AACTCCCGTATATGGA	CC1
KUL19.T_AACTCCCTCGCGATCG	CC1
KUL19.T_AACTCCCTCTCGTTTA	CC1
KUL19.T_AACTCTTTCGTCCAGG	CC1
KUL19.T_AACTGGTCATCCTAGA	CC1
KUL19.T_AACTGGTCATGCCCGA	CC1
KUL19.T_AACTGGTCATTTGCTT	CC1
KUL19.T_AACTGGTGTCTTGCGG	CC1
KUL19.T_AAGACCTGTACCGCTG	CC1
KUL19.T_AAGACCTGTCTAGTCA	CC1

KUL19.T_AAGCCGCAGATATGCA	CC1
KUL19.T_AAGCCGCAGGAGCGTT	CC1
KUL19.T_AAGCCGCAGGCACATG	CC1
KUL19.T_AAGCCGCGTAAATACG	CC1
KUL19.T_AAGCCGCGTAGATTAG	CC1
KUL19.T_AAGGCAGCAGCATGAG	CC1
KUL19.T_AAGGTTCAGCCGGTAA	CC1
KUL19.T_AAGGTTCTCCATGCTC	CC1
KUL19.T_AAGTCTGGTTGGTTTG	CC1
KUL19.T_AATCCAGAGCTACCGC	CC1
KUL19.T_AATCGGTCAAATTGCC	CC1
KUL19.T_ACACCAAAGTCAAGGC	CC1
KUL19.T_ACACCAAGTTTAGCTG	CC1
KUL19.T_ACACCGGAGATGCCTT	CC1
KUL19.T_ACACCGGAGGGCACTA	CC1
KUL19.T_ACACCGGGTACCATCA	CC1
KUL19.T_ACACTGAAGCCAGTTT	CC1
KUL19.T_ACACTGATCCGCGGTA	CC1
KUL19.T_ACATACGGTACTTGAC	CC1
KUL19.T_ACATACGGTTCGTTGA	CC2
KUL19.T_ACATCAGAGACTTGAA	CC1
KUL19.T_ACATCAGCACTTACGA	CC1
KUL19.T_ACATCAGGTGAAAGAG	CC1
KUL19.T_ACATGGTCACCGATAT	CC1
KUL19.T_ACATGGTGTCTCCCTA	CC1
KUL19.T_ACCAGTAGTAAGGGAA	CC1
KUL19.T_ACCCACTCAAGCTGTT	CC1
KUL19.T_ACCCACTGTAGCAAAT	CC1
KUL19.T_ACCCACTTCAGATAAG	CC1
KUL19.T_ACCGTAAAGTTAGCGG	CC1
KUL19.T_ACCGTAATCCGAAGAG	CC1
KUL19.T_ACCTTTAAGACTCGGA	CC1
KUL19.T_ACCTTTAGTGGCAAAC	CC1
KUL19.T_ACCTTTAGTTCAGGCC	CC1
KUL19.T_ACCTTTAGTTCGTGAT	CC1
KUL19.T_ACGAGGAAGGGTTCCC	CC1
KUL19.T_ACGAGGATCACCGTAA	CC1
KUL19.T_ACGATGTAGCTGCAAG	CC1
KUL19.T_ACGATGTCAGTCCTTC	CC1
KUL19.T_ACGCCGAAGCGCCTCA	CC1
KUL19.T_ACGCCGAGTGCCTGTG	CC1
KUL19.T_ACGGAGAAGAACTGTA	CC1
KUL19.T_ACGGAGACACTTCGAA	CC1
KUL19.T_ACGGCCATCCGGGTGT	CC1
KUL19.T_ACGGCCATCTCTTATG	CC1
KUL19.T_ACGGGCTGTCGATTGT	CC1
KUL19.T_ACGGGCTGTGTGGTTT	CC1
KUL19.T_ACGTCAATCTGTCTAT	CC1
KUL19.T_ACTATCTAGTGGCACA	CC1
KUL19.T_ACTATCTGTCCGAACC	CC1
KUL19.T_ACTGAGTCACAGTCGC	CC1
KUL19.T_ACTGCTCAGACTGTAA	CC1
KUL19.T_ACTGCTCAGAGCCTAG	CC1
KUL19.T_ACTGCTCAGCTTCGCG	CC1
KUL19.T_ACTGTCCAGACCTTTG	CC1
KUL19.T_ACTGTCCCAGCCACCA	CC2
KUL19.T_ACTTACTGTACACCGC	CC1
KUL19.T_ACTTACTTCTGCTGCT	CC1

KUL19.T_ACTTGTTAGTAAGTAC	CC1
KUL19.T_ACTTGTTCATATGAGA	CC1
KUL19.T_ACTTTCATCCTACAGA	CC1
KUL19.T_AGAATAGCAGGTCGTC	CC1
KUL19.T_AGACGTTAGCCACTAT	CC1
KUL19.T_AGAGCGAAGGCTATCT	CC1
KUL19.T_AGAGTGGCAGCCTATA	CC1
KUL19.T_AGAGTGGGTCGCGTGT	CC1
KUL19.T_AGATCTGCACTTCGAA	CC1
KUL19.T_AGATCTGCATTTCAGG	CC1
KUL19.T_AGATTGCAGCCCAACC	CC1
KUL19.T_AGCAGCCGTTACGGAG	CC1
KUL19.T_AGCATACGTCATGCAT	CC1
KUL19.T_AGCCTAAGTTATCCGA	CC1
KUL19.T_AGCGGTCGTACTTAGC	CC1
KUL19.T_AGCGGTCGTCACTTCC	CC1
KUL19.T_AGCGGTCGTTAAAGTG	CC1
KUL19.T_AGCGGTCTCCTTTCTC	CC1
KUL19.T_AGCGTATAGGCTAGCA	CC1
KUL19.T_AGCGTATTCCGTTGCT	CC1
KUL19.T_AGCGTCGTCGAGGTAG	CC1
KUL19.T_AGCGTCGTCTGATACG	CC1
KUL19.T_AGCTTGACACCTGGTG	CC1
KUL19.T_AGGCCACAGGAGTTTA	CC1
KUL19.T_AGGCCACCAGCCTATA	CC1
KUL19.T_AGGCCGTCACATTAGC	CC1
KUL19.T_AGGGATGAGGAATGGA	CC1
KUL19.T_AGGGATGCAGATGGCA	CC1
KUL19.T_AGGGTGATCAGTTGAC	CC1
KUL19.T_AGGTCATAGAGCTGCA	CC1
KUL19.T_AGGTCATCAAGTAGTA	CC1
KUL19.T_AGGTCATTCATCTGCC	CC1
KUL19.T_AGGTCCGGTTGTCGCG	CC1
KUL19.T_AGTAGTCCATCGGTTA	CC1
KUL19.T_AGTCTTTAGTCCGTAT	CC1
KUL19.T_AGTGGGACACCATCCT	CC1
KUL19.T_AGTGTCAGTAAGTTCC	CC1
KUL19.T_AGTTGGTAGAATCTCC	CC1
KUL19.T_AGTTGGTTCCTATGTT	CC1
KUL19.T_ATAACGCAGGGCTTGA	CC1
KUL19.T_ATAACGCTCAAGCCTA	CC2
KUL19.T_ATAACGCTCGACCAGC	CC1
KUL19.T_ATAAGAGAGTTCCACA	CC1
KUL19.T_ATAAGAGGTTATCGGT	CC1
KUL19.T_ATAGACCAGAAACCAT	CC1
KUL19.T_ATAGACCCATCCTTGC	CC1
KUL19.T_ATCACGAGTCTTGTCC	CC1
KUL19.T_ATCACGATCACCATAG	CC1
KUL19.T_ATCATCTCATGGGAAC	CC1
KUL19.T_ATCATCTGTACCGGCT	CC1
KUL19.T_ATCCGAACACGGTAAG	CC1
KUL19.T_ATCTGCCAGAAGATTC	CC1
KUL19.T_ATCTGCCCAACTGGCC	CC1
KUL19.T_ATCTGCCTCTGGAGCC	CC1
KUL19.T_ATGAGGGCAGCAGTTT	CC1
KUL19.T_ATGAGGGCAGGAATGC	CC1
KUL19.T_ATGTGTGGTTAAGGGC	CC1
KUL19.T_ATTCTACGTGAGTATA	CC1

KUL19.T_ATTCTACGTTTGTTTC	CC1
KUL19.T_ATTGGACAGACATAAC	CC1
KUL19.T_ATTGGACAGCCAGGAT	CC1
KUL19.T_ATTGGACAGGCCCTTG	CC1
KUL19.T_ATTGGACTCCTAGAAC	CC1
KUL19.T_ATTGGTGAGTGTGGCA	CC1
KUL19.T_ATTTCTGAGACTGTAA	CC1
KUL19.T_ATTTCTGAGCGTTGCC	CC1
KUL19.T_ATTTCTGCAAGTACCT	CC1
KUL19.T_CAACCTCAGCGTAATA	CC1
KUL19.T_CAACCTCCATTGAGCT	CC1
KUL19.T_CAAGAAAAGAGACTAT	CC2
KUL19.T_CAAGAAATCAGGTTCA	CC1
KUL19.T_CAAGATCTCTGTCAAG	CC1
KUL19.T_CAAGGCCCAGCTTAAC	CC1
KUL19.T_CAAGTTGAGATATACG	CC1
KUL19.T_CACACCTAGTGGACGT	CC1
KUL19.T_CACACCTCAATAGCAA	CC1
KUL19.T_CACACCTTCTTAACCT	CC1
KUL19.T_CACACTCCATCCGCGA	CC1
KUL19.T_CACAGGCCACACATGT	CC1
KUL19.T_CACAGTAAGACTTGAA	CC1
KUL19.T_CACAGTAAGTGAACAT	CC1
KUL19.T_CACATAGAGTAGCGGT	CC2
KUL19.T_CACATAGAGTTAGCGG	CC1
KUL19.T_CACATAGCAAAGCGGT	CC1
KUL19.T_CACATAGCACCCTATC	CC1
KUL19.T_CACCTTGTCACTGGGC	CC1
KUL19.T_CACTCCAAGACCCACC	CC1
KUL19.T_CAGAATCAGATGCGAC	CC1
KUL19.T_CAGCATAGTAAATGTG	CC1
KUL19.T_CAGCATAGTCCATCCT	CC1
KUL19.T_CAGCCGATCTAACTCT	CC1
KUL19.T_CAGCGACGTAGCTAAA	CC1
KUL19.T_CAGCGACTCAGGCAAG	CC1
KUL19.T_CAGCGACTCAGTGCAT	CC1
KUL19.T_CAGCGACTCTGATACG	CC1
KUL19.T_CAGCTAACAGTACACT	CC1
KUL19.T_CAGCTGGCATGTAGTC	CC1
KUL19.T_CAGCTGGGTACCGGCT	CC1
KUL19.T_CAGGTGCAGAATTCCC	CC1
KUL19.T_CAGGTGCTCAAACAAG	CC1
KUL19.T_CAGTAACGTAAGTTCC	CC1
KUL19.T_CAGTCCTGTCTAGCGC	CC1
KUL19.T_CATATTCAGTGTGAAT	CC1
KUL19.T_CATCAAGTCTTGCATT	CC1
KUL19.T_CATCAGAAGGGTGTTG	CC1
KUL19.T_CATCAGAGTTACCGAT	CC1
KUL19.T_CATCAGAGTTCCCTTG	CC1
KUL19.T_CATCCACCAATACGCT	CC1
KUL19.T_CATCCACCACATAACC	CC1
KUL19.T_CATCCACCATTCCTCG	CC1
KUL19.T_CATCGAACACCGAAAG	CC1
KUL19.T_CATCGGGCAACACGCC	CC1
KUL19.T_CATGACATCGTACGGC	CC1
KUL19.T_CATGCCTGTATGCTTG	CC1
KUL19.T_CATGGCGAGTTACGGG	CC1
KUL19.T_CATTATCAGACATAAC	CC1

KUL19.T_CATTCGCGTCGACTAT	CC1
KUL19.T_CCACCTATCAATAAGG	CC1
KUL19.T_CCACGGAGTTATTCTC	CC1
KUL19.T_CCACTACAGTGGGATC	CC1
KUL19.T_CCACTACCAATGCCAT	CC1
KUL19.T_CCACTACGTACTCAAC	CC1
KUL19.T_CCAGCGAAGCAGCCTC	CC1
KUL19.T_CCAGCGAAGCGTCAAG	CC1
KUL19.T_CCATGTCTCCGCATAA	CC1
KUL19.T_CCATGTCTCGCTTGTC	CC1
KUL19.T_CCCAATCTCCCTCTTT	CC1
KUL19.T_CCCAGTTCAGCCTTTC	CC1
KUL19.T_CCCAGTTGTAGAAAGG	CC1
KUL19.T_CCCTCCTAGTGCGATG	CC1
KUL19.T_CCGTACTGTCATTAGC	CC1
KUL19.T_CCGTACTTCAAACCGT	CC1
KUL19.T_CCGTGGATCCTCAACC	CC1
KUL19.T_CCGTTCACACATTAGC	CC1
KUL19.T_CCTAAAGAGTCCATAC	CC1
KUL19.T_CCTAAAGCATTCTTAC	CC1
KUL19.T_CCTAAAGTCAGGCAAG	CC1
KUL19.T_CCTACACCACACCGAC	CC1
KUL19.T_CCTACACTCAGCACAT	CC1
KUL19.T_CCTACACTCGGCTTGG	CC1
KUL19.T_CCTACCAGTTATCACG	CC1
KUL19.T_CCTACCATCATCTGCC	CC1
KUL19.T_CCTCAGTAGTTAACGA	CC1
KUL19.T_CCTCTGATCTAACCGA	CC2
KUL19.T_CCTTACGCACAGGCCT	CC1
KUL19.T_CCTTCCCAGAAACCGC	CC1
KUL19.T_CCTTCGAAGATGCCTT	CC1
KUL19.T_CCTTTCTGTAGAGTGC	CC1
KUL19.T_CCTTTCTTCGACAGCC	CC1
KUL19.T_CGAACATGTAAGTGTA	CC1
KUL19.T_CGAATGTCACAACGTT	CC1
KUL19.T_CGACCTTGTAGCCTCG	CC1
KUL19.T_CGACTTCAGGCCCTCA	CC1
KUL19.T_CGACTTCGTCTTCTCG	CC1
KUL19.T_CGAGAAGCAGGGCATA	CC1
KUL19.T_CGAGAAGTCCGTAGGC	CC1
KUL19.T_CGAGAAGTCTGCTTGC	CC1
KUL19.T_CGAGCACAGACATAAC	CC1
KUL19.T_CGAGCACCAGCGTTCG	CC1
KUL19.T_CGAGCCAAGAATTCCC	CC1
KUL19.T_CGAGCCACAATGTAAG	CC1
KUL19.T_CGATCGGAGGCACATG	CC1
KUL19.T_CGATCGGAGTATTGGA	CC1
KUL19.T_CGATTGAAGCGACGTA	CC1
KUL19.T_CGATTGAAGTCAAGGC	CC1
KUL19.T_CGATTGACAGTGACAG	CC1
KUL19.T_CGATTGAGTCGCCATG	CC1
KUL19.T_CGCCAAGTCATACGGT	CC1
KUL19.T_CGCGTTTAGAGCTATA	CC1
KUL19.T_CGCGTTTCACTGTTAG	CC1
KUL19.T_CGCGTTTTCACCGTAA	CC1
KUL19.T_CGCTATCCAAATTGCC	CC1
KUL19.T_CGCTTCATCGTGTAGT	CC1
KUL19.T_CGCTTCATCTCCAACC	CC1

KUL19.T_CGGACACCACAACTGT	CC1
KUL19.T_CGGACACGTCTAAACC	CC1
KUL19.T_CGGACACTCTGGCGAC	CC1
KUL19.T_CGGACGTCACTTACGA	CC1
KUL19.T_CGGACTGCAGCCTATA	CC1
KUL19.T_CGGACTGGTTGTTTGG	CC1
KUL19.T_CGGAGCTCACAGAGGT	CC1
KUL19.T_CGGAGTCCACGTCTCT	CC1
KUL19.T_CGGAGTCTCACTTCAT	CC1
KUL19.T_CGGGTCACAACACCCG	CC1
KUL19.T_CGTAGCGGTCAGTGGA	CC1
KUL19.T_CGTCAGGTCGGTTCGG	CC1
KUL19.T_CGTCCATGTAGAGGAA	CC1
KUL19.T_CGTCTACAGCGTAATA	CC1
KUL19.T_CGTGTAACAAGGACAC	CC1
KUL19.T_CGTGTAACACATGTGT	CC1
KUL19.T_CGTGTAACACCTATCC	CC1
KUL19.T_CGTTAGACACGAAACG	CC1
KUL19.T_CGTTCTGCAATGGAAT	CC1
KUL19.T_CGTTCTGCACGACTCG	CC1
KUL19.T_CGTTCTGTCTCCCTGA	CC1
KUL19.T_CGTTGGGAGCGTCTAT	CC1
KUL19.T_CGTTGGGCAAGTAGTA	CC1
KUL19.T_CGTTGGGTCACGACTA	CC1
KUL19.T_CGTTGGGTCTGCAAGT	CC1
KUL19.T_CTAACTTGTTTGACAC	CC1
KUL19.T_CTAAGACCATTGGTAC	CC1
KUL19.T_CTAATGGAGCCACCTG	CC1
KUL19.T_CTAATGGCACTGAAGG	CC1
KUL19.T_CTACATTAGAGGGCTT	CC1
KUL19.T_CTACATTCAGTAGAGC	CC1
KUL19.T_CTACGTCGTAGCGCTC	CC1
KUL19.T_CTAGAGTCACCTCGTT	CC1
KUL19.T_CTAGAGTGTAATCACC	CC1
KUL19.T_CTAGAGTTCTCGCATC	CC1
KUL19.T_CTAGCCTGTGATAAGT	CC1
KUL19.T_CTCACACGTTGTACAC	CC1
KUL19.T_CTCAGAACACAACGTT	CC1
KUL19.T_CTCATTAGTATAATGG	CC1
KUL19.T_CTCGAAACAACACGCC	CC1
KUL19.T_CTCGAGGCAAGGCTCC	CC1
KUL19.T_CTCGGAGCAGCAGTTT	CC1
KUL19.T_CTCGTACAGGAGTCTG	CC1
KUL19.T_CTCGTACAGTTTAGGA	CC1
KUL19.T_CTCTAATAGCGCCTCA	CC1
KUL19.T_CTCTAATTCGCAGGCT	CC1
KUL19.T_CTCTACGAGCTAAACA	CC1
KUL19.T_CTCTACGTCAAAGACA	CC1
KUL19.T_CTGAAACGTAAGTGGC	CC1
KUL19.T_CTGATAGGTCGAATCT	CC1
KUL19.T_CTGATCCAGGGTTCCC	CC1
KUL19.T_CTGATCCGTGAGGGAG	CC1
KUL19.T_CTGATCCTCGGAAACG	CC1
KUL19.T_CTGCCTATCAGATAAG	CC1
KUL19.T_CTGCCTATCCTCAACC	CC2
KUL19.T_CTGCCTATCGCGCCAA	CC1
KUL19.T_CTGCGGAGTACGCACC	CC1
KUL19.T_CTGCGGATCTTCAACT	CC1

KUL19.T_CTGTGCTGTCCTCTTG	CC1
KUL19.T_CTGTGCTGTTGCTCCT	CC1
KUL19.T_CTGTGCTTCAACACAC	CC1
KUL19.T_CTGTTTACAAGTAGTA	CC1
KUL19.T_CTTAACTAGTAGTGCG	CC1
KUL19.T_CTTAACTGTGGTAACG	CC1
KUL19.T_CTTCTCTCAAGCGTAG	CC1
KUL19.T_CTTGGCTAGGACCACA	CC1
KUL19.T_CTTGGCTGTTCCAACA	CC1
KUL19.T_CTTTGCGTCATAAAGG	CC1
KUL19.T_GAAACTCCAAGGTGTG	CC1
KUL19.T_GAAACTCGTTATGTGC	CC1
KUL19.T_GAAACTCTCTCTTATG	CC1
KUL19.T_GAAACTCTCTTGTTTG	CC1
KUL19.T_GAAATGAGTCCTCTTG	CC1
KUL19.T_GAACATCAGTTCGCGC	CC1
KUL19.T_GAACCTATCAAGCCTA	CC1
KUL19.T_GAACGGAGTCACAAGG	CC1
KUL19.T_GAAGCAGGTCAACATC	CC1
KUL19.T_GAATGAAGTACCGAGA	CC1
KUL19.T_GACACGCAGGGTTTCT	CC1
KUL19.T_GACAGAGCAAGTTCTG	CC1
KUL19.T_GACAGAGGTCGATTGT	CC1
KUL19.T_GACAGAGTCCAATGGT	CC1
KUL19.T_GACCAATCATCCGCGA	CC1
KUL19.T_GACCAATGTGTAACGG	CC1
KUL19.T_GACCAATTCAAACGGG	CC1
KUL19.T_GACGGCTTCACATAGC	CC1
KUL19.T_GACGGCTTCGGAGGTA	CC1
KUL19.T_GACGTGCTCCTAGGGC	CC1
KUL19.T_GACGTTACACGGCTAC	CC1
KUL19.T_GACGTTACATCCAACA	CC1
KUL19.T_GACTGCGCATATACGC	CC1
KUL19.T_GACTGCGTCAGAGACG	CC1
KUL19.T_GAGCAGAAGCGTTTAC	CC1
KUL19.T_GAGCAGAGTACAGACG	CC1
KUL19.T_GAGCAGAGTGTTGGGA	CC1
KUL19.T_GAGGTGACAGTACACT	CC1
KUL19.T_GAGTCCGGTTTGGCGC	CC1
KUL19.T_GATCAGTAGGCGATAC	CC1
KUL19.T_GATCAGTCACAGGCCT	CC1
KUL19.T_GATCGATAGGACCACA	CC1
KUL19.T_GATCGATCACTGAAGG	CC1
KUL19.T_GATCGATTCCAAAGTC	CC1
KUL19.T_GATCGTAAGGCGTACA	CC1
KUL19.T_GATCTAGTCTTAGCCC	CC1
KUL19.T_GATGAGGCATCACGAT	CC1
KUL19.T_GATGCTACATGCGCAC	CC1
KUL19.T_GATTCAGAGATGGGTC	CC1
KUL19.T_GATTCAGAGCGGATCA	CC1
KUL19.T_GATTCAGAGGTGCTAG	CC1
KUL19.T_GATTCAGGTAAGAGAG	CC1
KUL19.T_GCAATCAAGGCGCTCT	CC1
KUL19.T_GCACTCTTCATCGGAT	CC1
KUL19.T_GCATACACAAATCCGT	CC1
KUL19.T_GCATGATCATTAGCCA	CC1
KUL19.T_GCATGATTCGCGTTTC	CC1
KUL19.T_GCATGCGAGTGGGTTG	CC1

KUL19.T_GCATGCGCAGATGGCA	CC1
KUL19.T_GCATGTACAATAACGA	CC1
KUL19.T_GCATGTAGTAAACCTC	CC1
KUL19.T_GCGACCACACAGACTT	CC1
KUL19.T_GCGAGAAAGTTGTAGA	CC1
KUL19.T_GCGCAACAGCTGTTCA	CC1
KUL19.T_GCGCAACTCCGCGTTT	CC1
KUL19.T_GCGCAACTCGGCCGAT	CC1
KUL19.T_GCGCCAACACTTCTGC	CC1
KUL19.T_GCGCCAATCTGTCAAG	CC1
KUL19.T_GCGCGATGTCTCTTAT	CC1
KUL19.T_GCGCGATGTGTGAAAT	CC1
KUL19.T_GCGGGTTAGAATAGGG	CC1
KUL19.T_GCTCTGTAGGACTGGT	CC1
KUL19.T_GCTGCGAAGGCATGTG	CC1
KUL19.T_GCTGCGAAGGCCGAAT	CC1
KUL19.T_GCTGGGTTCAAGGCTT	CC1
KUL19.T_GCTTGAAGTGATGTGG	CC1
KUL19.T_GCTTGAAGTTTGGCGC	CC1
KUL19.T_GGAAAGCAGGATGTAT	CC1
KUL19.T_GGACAAGCATAACCTG	CC1
KUL19.T_GGACAGATCGCGTTTC	CC1
KUL19.T_GGACATTAGCGCTCCA	CC1
KUL19.T_GGACATTTCAATACCG	CC1
KUL19.T_GGACGTCTCCGCATCT	CC1
KUL19.T_GGAGCAACAACGATCT	CC1
KUL19.T_GGATGTTCAACACCTA	CC1
KUL19.T_GGATGTTCACTTGGAT	CC1
KUL19.T_GGCCGATCAAGACGTG	CC1
KUL19.T_GGCGTGTAGATATGCA	CC1
KUL19.T_GGCGTGTAGCGCCTTG	CC1
KUL19.T_GGCTGGTGTTAGTGGG	CC1
KUL19.T_GGCTGGTTCATAGCAC	CC1
KUL19.T_GGGAGATTCACTGGGC	CC1
KUL19.T_GGGATGAAGATAGGAG	CC1
KUL19.T_GGGATGAAGATGGCGT	CC1
KUL19.T_GGGATGACAAGTAATG	CC1
KUL19.T_GGGCACTCACCATGTA	CC1
KUL19.T_GGGCACTCATCCAACA	CC1
KUL19.T_GGGTCTGCATGCCTTC	CC1
KUL19.T_GGGTTGCAGTCAAGCG	CC1
KUL19.T_GGTATTGGTCGCGTGT	CC1
KUL19.T_GGTGAAGAGCCCTAAT	CC1
KUL19.T_GGTGCGTAGGGATACC	CC1
KUL19.T_GGTGCGTTCCTTGGTC	CC1
KUL19.T_GGTGTTACACGTCAGC	CC1
KUL19.T_GGTGTTATCAATCTCT	CC1
KUL19.T_GTAACGTAGTCGCCGT	CC1
KUL19.T_GTAACGTCAATAACGA	CC1
KUL19.T_GTAACTGAGAAACCGC	CC1
KUL19.T_GTACGTAAGGCATGGT	CC1
KUL19.T_GTACGTACAACAACCT	CC1
KUL19.T_GTACGTAGTGGCCCTA	CC1
KUL19.T_GTACGTATCGAATGCT	CC1
KUL19.T_GTACTCCTCAACACAC	CC1
KUL19.T_GTACTTTGTAAGGATT	CC1
KUL19.T_GTAGGCCAGTACGCGA	CC1
KUL19.T_GTAGGCCTCATAGCAC	CC1

KUL19.T_GTAGTCAAGCTCAACT	CC1
KUL19.T_GTATCTTGTATAGTAG	CC1
KUL19.T_GTATTCTCATTGAGCT	CC1
KUL19.T_GTCACGGCATGGGACA	CC1
KUL19.T_GTCATTTTCATATCGG	CC1
KUL19.T_GTCATTTTCATTGCCC	CC1
KUL19.T_GTCCTCAAGACTAGAT	CC1
KUL19.T_GTCCTCAAGCGAGAAA	CC1
KUL19.T_GTCGGGTCAGATGAGC	CC1
KUL19.T_GTCGGGTCAGCTTCGG	CC1
KUL19.T_GTCGTAAAGAATGTTG	CC1
KUL19.T_GTCTCGTAGTTGAGAT	CC1
KUL19.T_GTCTCGTTCAGCTCGG	CC1
KUL19.T_GTGAAGGGTAGCTCCG	CC1
KUL19.T_GTGAAGGGTGTCCTCT	CC1
KUL19.T_GTGCAGCAGAGTTGGC	CC1
KUL19.T_GTGCAGCGTTCTGTTT	CC1
KUL19.T_GTGCGGTGTCGCATCG	CC1
KUL19.T_GTGCGGTGTGTGCCTG	CC1
KUL19.T_GTGCTTCGTCCGAGTC	CC1
KUL19.T_GTGGGTCCATTATCTC	CC1
KUL19.T_GTGTGCGCAAGTTAAG	CC1
KUL19.T_GTTAAGCAGCTGTTCA	CC1
KUL19.T_GTTAAGCCAGGTTTCA	CC1
KUL19.T_GTTAAGCCAGTCGATT	CC1
KUL19.T_GTTAAGCTCCTCTAGC	CC1
KUL19.T_GTTAAGCTCTCGATGA	CC1
KUL19.T_GTTACAGTCCTCTAGC	CC1
KUL19.T_GTTCATTCACGTCAGC	CC1
KUL19.T_GTTCGGGAGCTATGCT	CC1
KUL19.T_GTTCGGGAGTCTCAAC	CC1
KUL19.T_GTTCTCGAGTAGCCGA	CC1
KUL19.T_GTTCTCGGTATATGAG	CC2
KUL19.T_TAAACCGGTGCTAGCC	CC1
KUL19.T_TAAGCGTTCGCCCTTA	CC1
KUL19.T_TACACGAGTTGGTGGA	CC1
KUL19.T_TACAGTGAGCATGGCA	CC1
KUL19.T_TACCTTAAGCTCCTTC	CC1
KUL19.T_TACGGATTCGCGCCAA	CC1
KUL19.T_TACGGGCAGTACTTGC	CC1
KUL19.T_TACGGTAAGCCGGTAA	CC1
KUL19.T_TACTTACAGTACTTGC	CC1
KUL19.T_TACTTACCAAGCGATG	CC1
KUL19.T_TACTTGTTCTTGCAAG	CC1
KUL19.T_TAGACCACACCAACCG	CC1
KUL19.T_TAGACCATCCGTACAA	CC1
KUL19.T_TAGAGCTGTGCTTCTC	CC1
KUL19.T_TAGCCGGAGAGCTATA	CC1
KUL19.T_TAGCCGGTCTACTATC	CC1
KUL19.T_TAGGCATAGGTAGCCA	CC1
KUL19.T_TAGTGGTAGATAGTCA	CC1
KUL19.T_TAGTGGTGTTGCGTTA	CC1
KUL19.T_TATGCCCGTCCGTCAG	CC1
KUL19.T_TATGCCCGTGACAAAT	CC1
KUL19.T_TCACGAAGTATGGTTC	CC1
KUL19.T_TCACGAATCGCGTAGC	CC1
KUL19.T_TCAGATGTCCGTACAA	CC1
KUL19.T_TCAGGATAGGTAGCCA	CC1

KUL19.T_TCAGGATGTCCGACGT	CC1
KUL19.T_TCAGGTAGTGTGTGCC	CC1
KUL19.T_TCATTACAGGCTCAGA	CC1
KUL19.T_TCATTACCATGAACCT	CC1
KUL19.T_TCATTTGGTAGCTCCG	CC1
KUL19.T_TCATTTGTCTAGAGTC	CC1
KUL19.T_TCCCGATTCATCTGTT	CC1
KUL19.T_TCGAGGCAGTTATCGC	CC1
KUL19.T_TCGCGAGGTACTTCTT	CC1
KUL19.T_TCGCGTTAGTTATCGC	CC1
KUL19.T_TCGCGTTCATATGAGA	CC1
KUL19.T_TCGGGACAGATCTGAA	CC1
KUL19.T_TCGGGACCATGCCACG	CC1
KUL19.T_TCGGGACTCCTGCCAT	CC1
KUL19.T_TCGTACCCAAGCCGCT	CC1
KUL19.T_TCGTAGAGTTCGGGCT	CC1
KUL19.T_TCTATTGTCAACACTG	CC1
KUL19.T_TCTCTAAGTATCTGCA	CC1
KUL19.T_TCTCTAAGTCCGAAGA	CC1
KUL19.T_TCTCTAAGTTGGACCC	CC1
KUL19.T_TCTGGAATCAGCTCTC	CC1
KUL19.T_TCTTTCCCAGATTGCT	CC1
KUL19.T_TCTTTCCCATGAGCGA	CC1
KUL19.T_TCTTTCCTCACTTATC	CC1
KUL19.T_TGACAACCACTATCTT	CC1
KUL19.T_TGACGGCTCTTGCAAG	CC1
KUL19.T_TGACTAGAGCCACGTC	CC1
KUL19.T_TGACTAGCAGCTGTTA	CC1
KUL19.T_TGACTAGGTTGACGTT	CC1
KUL19.T_TGACTAGTCCGTAGGC	CC1
KUL19.T_TGACTTTCAAGGTGTG	CC1
KUL19.T_TGACTTTCACAACGCC	CC1
KUL19.T_TGACTTTGTGTCCTCT	CC1
KUL19.T_TGAGAGGTCTTGCAAG	CC1
KUL19.T_TGAGCATGTCTAGGTT	CC1
KUL19.T_TGAGCATTCGTCCGTT	CC1
KUL19.T_TGAGCCGTCAGTCAGT	CC1
KUL19.T_TGAGGGAAGGTGTTAA	CC1
KUL19.T_TGAGGGAGTCTTGATG	CC1
KUL19.T_TGATTTCTCCTTTACA	CC1
KUL19.T_TGCACCTCAGTAACGG	CC1
KUL19.T_TGCACCTGTACGAAAT	CC1
KUL19.T_TGCACCTTCGTTTATC	CC1
KUL19.T_TGCACCTTCTTGCAAG	CC1
KUL19.T_TGCCCATGTCTTGATG	CC1
KUL19.T_TGCCCTAAGGAGTAGA	CC1
KUL19.T_TGCCCTAGTCGTGGCT	CC1
KUL19.T_TGCGGGTCATCACCCT	CC1
KUL19.T_TGCGGGTTCAACACTG	CC1
KUL19.T_TGCTGCTCAATCACAC	CC1
KUL19.T_TGCTGCTCATTAGCCA	CC1
KUL19.T_TGGCGCATCCTCGCAT	CC1
KUL19.T_TGGCTGGTCAACACGT	CC1
KUL19.T_TGGCTGGTCTGACCTC	CC1
KUL19.T_TGGGCGTCATTGTGCA	CC1
KUL19.T_TGGTTAGTCACGCGGT	CC1
KUL19.T_TGGTTAGTCGTACGGC	CC1
KUL19.T_TGGTTCCCACCGATAT	CC1

KUL19.T_TGTATTCAGGACAGCT	CC1
KUL19.T_TGTGGTATCAAGGTAA	CC1
KUL19.T_TGTGTTTAGCCATCGC	CC1
KUL19.T_TGTGTTTAGGATATAC	CC1
KUL19.T_TGTGTTTTCTTTACGT	CC1
KUL19.T_TGTTCCGTCTGGCGAC	CC1
KUL19.T_TTAACTCCACACAGAG	CC1
KUL19.T_TTAACTCCATTCTCAT	CC1
KUL19.T_TTAACTCGTCGTTGTA	CC1
KUL19.T_TTAGGACAGATCTGCT	CC1
KUL19.T_TTAGGACCAAGTAATG	CC1
KUL19.T_TTAGGACCACGAGGTA	CC1
KUL19.T_TTCCCAGAGTGTCTCA	CC1
KUL19.T_TTCGAAGCACGCCAGT	CC1
KUL19.T_TTCTACATCGTCTGAA	CC1
KUL19.T_TTCTACATCTGGCGAC	CC1
KUL19.T_TTCTACATCTTTACGT	CC1
KUL19.T_TTCTCAAAGAATTCCC	CC1
KUL19.T_TTCTCCTGTACTTGAC	CC1
KUL19.T_TTCTTAGCAGGGATTG	CC1
KUL19.T_TTCTTAGCATCGGAAG	CC1
KUL19.T_TTCTTAGGTCCTGCTT	CC1
KUL19.T_TTGCCGTCATTATCTC	CC1
KUL19.T_TTGCCGTGTCCGTCAG	CC1
KUL19.T_TTGGCAATCCGCATCT	CC1
KUL19.T_TTTATGCCATCACCCT	CC2
KUL19.T_TTTATGCGTTCAACCA	CC1
KUL19.T_TTTCCTCCACAGATTC	CC1
KUL19.T_TTTGTCACACGGCTAC	CC1
KUL19.T_TTTGTCATCGGCTACG	CC1
KUL21.T_AAACGGGGTCTTCAAG	CC1
KUL21.T_AAAGATGCATCAGTCA	CC1
KUL21.T_AAAGATGTCTCTGCTG	CC2
KUL21.T_AAAGCAAAGCCGTCGT	CC2
KUL21.T_AAAGCAAAGGATATAC	CC2
KUL21.T_AAAGCAAAGTGTCCAT	CC2
KUL21.T_AAAGCAACAGACGCTC	CC2
KUL21.T_AAAGCAACATTCTTAC	CC2
KUL21.T_AAAGTAGGTAAGTGGC	CC2
KUL21.T_AAAGTAGGTGATGTCT	CC1
KUL21.T_AACACGTAGGAATCGC	CC2
KUL21.T_AACCATGCATGACGGA	CC2
KUL21.T_AACCGCGAGTTCGCGC	CC2
KUL21.T_AACCGCGCACATGTGT	CC2
KUL21.T_AACCGCGCAGATTGCT	CC1
KUL21.T_AACGTTGCAATGACCT	CC2
KUL21.T_AACGTTGGTCTCTCTG	CC1
KUL21.T_AACGTTGTCGCTTGTC	CC2
KUL21.T_AACGTTGTCGTACGGC	CC2
KUL21.T_AACTCAGTCAGTCAGT	CC2
KUL21.T_AACTCCCCAAGTTAAG	CC2
KUL21.T_AACTCTTGTAAACACA	CC2
KUL21.T_AACTCTTGTGTGACGA	CC1
KUL21.T_AACTCTTTCTGGCGTG	CC1
KUL21.T_AACTGGTCAGACAAAT	CC2
KUL21.T_AACTGGTGTAATCGTC	CC1
KUL21.T_AACTTTCCAACAACCT	CC2
KUL21.T_AACTTTCGTCTTCGTC	CC1

KUL21.T_AAGACCTCATGCCCGA	CC1
KUL21.T_AAGACCTGTATATCCG	CC2
KUL21.T_AAGGAGCAGTGGAGTC	CC2
KUL21.T_AAGGCAGCACCTTGTC	CC2
KUL21.T_AAGGCAGCAGACGCAA	CC1
KUL21.T_AAGTCTGCAGATCTGT	CC1
KUL21.T_AATCCAGGTTATTCTC	CC2
KUL21.T_AATCCAGGTTTGACTG	CC2
KUL21.T_AATCGGTGTATAATGG	CC2
KUL21.T_AATCGGTGTTAAAGTG	CC2
KUL21.T_ACACCAAAGCAGCCTC	CC2
KUL21.T_ACACCCTAGTAGCCGA	CC1
KUL21.T_ACACCCTCAATGGATA	CC2
KUL21.T_ACACCGGAGAGTACAT	CC2
KUL21.T_ACACTGAAGGCAATTA	CC1
KUL21.T_ACACTGATCTGGTTCC	CC1
KUL21.T_ACAGCCGCAGCATGAG	CC1
KUL21.T_ACAGCCGTCCACGAAT	CC2
KUL21.T_ACAGCCGTCTTTAGGG	CC2
KUL21.T_ACAGCTAAGGCATTGG	CC2
KUL21.T_ACATCAGTCCTAGAAC	CC1
KUL21.T_ACATGGTGTTTGCATG	CC1
KUL21.T_ACCAGTAAGAGACTTA	CC2
KUL21.T_ACCAGTAAGTTGTAGA	CC1
KUL21.T_ACCAGTAGTAGAGTGC	CC2
KUL21.T_ACCAGTATCCAGAAGG	CC2
KUL21.T_ACCCACTAGGTAGCTG	CC1
KUL21.T_ACCGTAAGTAAGTTCC	CC2
KUL21.T_ACCTTTACAAGTCTAC	CC1
KUL21.T_ACCTTTATCAACGGGA	CC1
KUL21.T_ACCTTTATCGACAGCC	CC1
KUL21.T_ACCTTTATCGTGTAGT	CC2
KUL21.T_ACGAGCCCAGCTGCTG	CC2
KUL21.T_ACGAGCCTCAGAGGTG	CC2
KUL21.T_ACGAGGAAGAGCTATA	CC2
KUL21.T_ACGAGGATCATCTGTT	CC2
KUL21.T_ACGAGGATCCCGACTT	CC1
KUL21.T_ACGATACAGAGGGCTT	CC1
KUL21.T_ACGATACGTGTTAAGA	CC2
KUL21.T_ACGCAGCCACATCCAA	CC1
KUL21.T_ACGCAGCGTCAAAGAT	CC2
KUL21.T_ACGCAGCGTGGTACAG	CC1
KUL21.T_ACGCAGCTCAATACCG	CC2
KUL21.T_ACGCCGAAGCAATCTC	CC1
KUL21.T_ACGCCGACAAATCCGT	CC1
KUL21.T_ACGCCGACAATCTACG	CC1
KUL21.T_ACGCCGACACAGGCCT	CC1
KUL21.T_ACGGGCTAGAGGGCTT	CC1
KUL21.T_ACGGGCTGTTTAGCTG	CC1
KUL21.T_ACGGGTCCATTGCGGC	CC2
KUL21.T_ACGTCAAAGAACTGTA	CC2
KUL21.T_ACTGAACAGACCCACC	CC2
KUL21.T_ACTGAACGTGTTTGGT	CC1
KUL21.T_ACTGAGTAGCGATGAC	CC1
KUL21.T_ACTGAGTCAGCGTAAG	CC2
KUL21.T_ACTGATGGTCATCCCT	CC1
KUL21.T_ACTGCTCCAAGACGTG	CC2
KUL21.T_ACTGTCCAGACTGGGT	CC2

KUL21.T_ACTGTCCCATGCAATC	CC2
KUL21.T_ACTGTCCTCACTTACT	CC1
KUL21.T_ACTGTCCTCTCTTATG	CC2
KUL21.T_ACTTACTAGTGCGTGA	CC2
KUL21.T_ACTTACTCAATGGAGC	CC2
KUL21.T_ACTTGTTGTCGGATCC	CC1
KUL21.T_ACTTGTTTCTTTAGTC	CC2
KUL21.T_ACTTTCATCACAACGT	CC1
KUL21.T_ACTTTCATCACTCTTA	CC2
KUL21.T_AGACGTTAGATGTGTA	CC2
KUL21.T_AGACGTTCAGTGGGAT	CC2
KUL21.T_AGACGTTTCTCGTATT	CC2
KUL21.T_AGAGCGAGTTATGTGC	CC2
KUL21.T_AGAGCTTCAGCTATTG	CC2
KUL21.T_AGAGCTTCATGGTTGT	CC1
KUL21.T_AGAGCTTTCCGTTGCT	CC2
KUL21.T_AGAGTGGAGCGATATA	CC1
KUL21.T_AGATCTGAGAAACGAG	CC2
KUL21.T_AGATCTGAGGCCCGTT	CC1
KUL21.T_AGATCTGTCATGTAGC	CC1
KUL21.T_AGATTGCGTGTTAAGA	CC1
KUL21.T_AGCAGCCAGTCTCCTC	CC1
KUL21.T_AGCATACGTGGGTCAA	CC2
KUL21.T_AGCCTAAAGTAGATGT	CC1
KUL21.T_AGCCTAATCCTTGACC	CC1
KUL21.T_AGCGGTCCAGTCACTA	CC2
KUL21.T_AGCGGTCCATCCAACA	CC2
KUL21.T_AGCGGTCTCTGCGACG	CC1
KUL21.T_AGCGTATCATGCGCAC	CC2
KUL21.T_AGCGTATGTCCAGTTA	CC1
KUL21.T_AGCGTCGCACGCCAGT	CC2
KUL21.T_AGCTCCTAGGACATTA	CC1
KUL21.T_AGCTCTCTCGCGTTTC	CC1
KUL21.T_AGCTTGAGTGCAACGA	CC1
KUL21.T_AGGCCACAGAAGCCCA	CC2
KUL21.T_AGGCCGTGTGCTCTTC	CC2
KUL21.T_AGGCCGTTCTATCCTA	CC1
KUL21.T_AGGGAGTAGTCTCAAC	CC2
KUL21.T_AGGGATGCAGACGCCT	CC2
KUL21.T_AGGGATGTCGACGGAA	CC1
KUL21.T_AGGGATGTCGTCCGTT	CC2
KUL21.T_AGGGTGACAAGCCCAC	CC2
KUL21.T_AGGGTGACACCAGGCT	CC2
KUL21.T_AGGTCCGGTTCCCTTG	CC2
KUL21.T_AGTAGTCAGGAGTTGC	CC2
KUL21.T_AGTAGTCAGGCTCATT	CC1
KUL21.T_AGTAGTCCATGTCCTC	CC2
KUL21.T_AGTGAGGAGGTGCTTT	CC2
KUL21.T_AGTGAGGGTCCGAGTC	CC2
KUL21.T_AGTGAGGTCATCATTC	CC2
KUL21.T_AGTGAGGTCTGGCGAC	CC2
KUL21.T_AGTGGGAGTGTGACGA	CC1
KUL21.T_AGTGGGATCCACGAAT	CC1
KUL21.T_AGTTGGTGTAGGCATG	CC2
KUL21.T_ATAACGCAGGCATTGG	CC2
KUL21.T_ATAGACCAGAGAACAG	CC2
KUL21.T_ATCACGATCTCGCATC	CC1
KUL21.T_ATCATCTCAATGGAAT	CC1

KUL21.T_ATCATCTCACAGGCCT	CC2
KUL21.T_ATCATCTGTACAGACG	CC2
KUL21.T_ATCATCTGTTAAGATG	CC2
KUL21.T_ATCATCTTCAGAGACG	CC2
KUL21.T_ATCATCTTCTACGAGT	CC2
KUL21.T_ATCATGGCAGGAATGC	CC2
KUL21.T_ATCCACCAGTTCCACA	CC1
KUL21.T_ATCCACCGTCCGAAGA	CC1
KUL21.T_ATCCGAACATTAGCCA	CC1
KUL21.T_ATCCGAAGTCTAGTGT	CC2
KUL21.T_ATCGAGTAGTCATCCA	CC2
KUL21.T_ATCGAGTAGTCCCACG	CC2
KUL21.T_ATCTACTTCACTATTC	CC1
KUL21.T_ATGAGGGCAGACAGGT	CC2
KUL21.T_ATGAGGGCAGTTCCCT	CC1
KUL21.T_ATGAGGGGTTAAGATG	CC1
KUL21.T_ATGCGATCAGCGTCCA	CC1
KUL21.T_ATGCGATTCTTCATGT	CC1
KUL21.T_ATGGGAGCATCACCCT	CC2
KUL21.T_ATGGGAGGTTCCAACA	CC2
KUL21.T_ATGTGTGAGCGTAATA	CC2
KUL21.T_ATGTGTGGTCTCTCTG	CC1
KUL21.T_ATTACTCCATATACCG	CC2
KUL21.T_ATTACTCTCCGCGGTA	CC1
KUL21.T_ATTCTACAGCCTCGTG	CC2
KUL21.T_ATTCTACCACCAGTTA	CC1
KUL21.T_ATTCTACCACGTTGGC	CC1
KUL21.T_ATTCTACGTCATGCCG	CC2
KUL21.T_ATTGGTGCACACGCTG	CC1
KUL21.T_ATTGGTGCACAGGCCT	CC2
KUL21.T_ATTGGTGCACTCTGTC	CC2
KUL21.T_ATTGGTGTCCACGACG	CC2
KUL21.T_ATTTCTGAGGGATACC	CC1
KUL21.T_CAACCTCTCCACGTTC	CC1
KUL21.T_CAACTAGCACGGCGTT	CC2
KUL21.T_CAAGATCGTAGAGCTG	CC1
KUL21.T_CAAGATCGTTGATTGC	CC2
KUL21.T_CAAGATCTCGGAAATA	CC1
KUL21.T_CAAGGCCAGATCCTGT	CC2
KUL21.T_CAAGTTGCACACATGT	CC1
KUL21.T_CAAGTTGGTAGCCTCG	CC2
KUL21.T_CAAGTTGGTTATGTGC	CC1
KUL21.T_CAAGTTGTCCTTGGTC	CC1
KUL21.T_CACAAACCAGTATGCT	CC2
KUL21.T_CACAAACTCAACGGGA	CC2
KUL21.T_CACACAAAGCCACGTC	CC1
KUL21.T_CACACAATCATCGGAT	CC1
KUL21.T_CACACAATCTCTGAGA	CC1
KUL21.T_CACACCTCACGGCGTT	CC1
KUL21.T_CACACCTTCCGCGCAA	CC2
KUL21.T_CACACCTTCTCAAGTG	CC1
KUL21.T_CACAGGCAGACTACAA	CC2
KUL21.T_CACAGGCAGGTTACCT	CC2
KUL21.T_CACAGTAGTCGATTGT	CC2
KUL21.T_CACATTTCACCGCTAG	CC2
KUL21.T_CACATTTGTCCGTGAC	CC1
KUL21.T_CACATTTGTTGGACCC	CC2
KUL21.T_CACCACTGTAGGAGTC	CC2

KUL21.T_CACCAGGAGAATAGGG	CC2
KUL21.T_CACCTTGAGACTAAGT	CC2
KUL21.T_CACCTTGAGGAACTGC	CC2
KUL21.T_CACCTTGCATCATCCC	CC1
KUL21.T_CACTCCAGTCCGTTAA	CC1
KUL21.T_CACTCCATCACCAGGC	CC1
KUL21.T_CAGAATCCAGCCAGAA	CC2
KUL21.T_CAGAATCGTCTCGTTC	CC1
KUL21.T_CAGAGAGAGGCTAGCA	CC2
KUL21.T_CAGAGAGTCGGCCGAT	CC1
KUL21.T_CAGCATATCGCGCCAA	CC1
KUL21.T_CAGCCGAGTTCGTTGA	CC1
KUL21.T_CAGCTGGCACCACGTG	CC2
KUL21.T_CAGCTGGGTAAAGGAG	CC1
KUL21.T_CAGCTGGTCACTTACT	CC1
KUL21.T_CAGCTGGTCGGATGTT	CC1
KUL21.T_CAGTCCTCACAAGACG	CC2
KUL21.T_CAGTCCTTCGGTGTCG	CC1
KUL21.T_CAGTCCTTCTCGTATT	CC2
KUL21.T_CATATGGAGCGTAGTG	CC2
KUL21.T_CATATGGCACTCAGGC	CC1
KUL21.T_CATATGGGTCATTAGC	CC2
KUL21.T_CATATGGGTGATGCCC	CC2
KUL21.T_CATATGGTCCACGCAG	CC2
KUL21.T_CATATTCCAGGGTACA	CC2
KUL21.T_CATATTCTCAGTTAGC	CC1
KUL21.T_CATCAAGTCTTGACGA	CC2
KUL21.T_CATCAGACAGGAATGC	CC1
KUL21.T_CATCAGACATGTTGAC	CC2
KUL21.T_CATCAGATCACCGGGT	CC2
KUL21.T_CATCCACGTAGCTGCC	CC2
KUL21.T_CATCGAAAGCCCGAAA	CC2
KUL21.T_CATCGAAAGTGCCATT	CC1
KUL21.T_CATCGAACAACACCCG	CC2
KUL21.T_CATCGAATCACTTACT	CC2
KUL21.T_CATCGAATCTTAGAGC	CC2
KUL21.T_CATGACAGTGGTTTCA	CC1
KUL21.T_CATGACATCCTCCTAG	CC1
KUL21.T_CATGCCTCAACGCACC	CC1
KUL21.T_CATGGCGCACTCAGGC	CC2
KUL21.T_CATGGCGGTGCGCTTG	CC1
KUL21.T_CATGGCGGTTGAACTC	CC2
KUL21.T_CATGGCGTCTTAGAGC	CC2
KUL21.T_CATTATCAGGCGCTCT	CC2
KUL21.T_CATTATCAGTGAAGAG	CC1
KUL21.T_CATTATCTCAACACAC	CC1
KUL21.T_CATTCGCGTCGCCATG	CC2
KUL21.T_CCACCTACACAGCCCA	CC2
KUL21.T_CCACGGAAGTGAATTG	CC1
KUL21.T_CCACTACGTAGGGTAC	CC2
KUL21.T_CCAGCGACAAAGGCGT	CC2
KUL21.T_CCATGTCGTACTTCTT	CC2
KUL21.T_CCATGTCGTTCCCTTG	CC1
KUL21.T_CCATGTCTCTTCGGTC	CC2
KUL21.T_CCATTCGAGTGAATTG	CC1
KUL21.T_CCATTCGGTGTGGCTC	CC1
KUL21.T_CCCAATCGTCTACCTC	CC2
KUL21.T_CCCAGTTCAAGCCGCT	CC2

KUL21.T_CCCATACAGGTGTGGT	CC2
KUL21.T_CCCTCCTCAGTGGAGT	CC1
KUL21.T_CCGGTAGCACCAGCAC	CC2
KUL21.T_CCGGTAGGTCTAGTGT	CC2
KUL21.T_CCGTACTGTCCGAATT	CC2
KUL21.T_CCGTGGATCGCCATAA	CC2
KUL21.T_CCGTTCACACGGTTTA	CC1
KUL21.T_CCGTTCACATACTCTT	CC2
KUL21.T_CCGTTCAGTAGTGAAT	CC2
KUL21.T_CCGTTCATCGGTTAAC	CC2
KUL21.T_CCTAAAGCAGCGTAAG	CC1
KUL21.T_CCTAAAGCAGGGAGAG	CC1
KUL21.T_CCTACCAAGCCCAATT	CC2
KUL21.T_CCTACCAGTCACTGGC	CC1
KUL21.T_CCTAGCTCAGCCTGTG	CC2
KUL21.T_CCTATTACATCGTCGG	CC1
KUL21.T_CCTCAGTAGCACCGCT	CC2
KUL21.T_CCTCAGTCACTCTGTC	CC2
KUL21.T_CCTCAGTTCAACGGGA	CC2
KUL21.T_CCTCTGACAAAGGTGC	CC1
KUL21.T_CCTTACGGTTCAGTAC	CC2
KUL21.T_CCTTACGGTTGCGCAC	CC1
KUL21.T_CCTTACGTCCGGGTGT	CC2
KUL21.T_CCTTCCCAGGCGATAC	CC1
KUL21.T_CCTTCCCAGGTGTGGT	CC2
KUL21.T_CCTTCCCGTATATGGA	CC1
KUL21.T_CCTTCGAGTCGCATAT	CC2
KUL21.T_CCTTCGATCGTCCAGG	CC1
KUL21.T_CGAACATGTCAAAGCG	CC2
KUL21.T_CGAACATGTTTGGGCC	CC1
KUL21.T_CGAATGTCACATCTTT	CC2
KUL21.T_CGAATGTCACGTCAGC	CC1
KUL21.T_CGAATGTTCGGCTACG	CC2
KUL21.T_CGAGAAGAGAAGGTGA	CC2
KUL21.T_CGAGAAGGTTGAGGTG	CC1
KUL21.T_CGAGCACCATGCCTTC	CC1
KUL21.T_CGAGCCAAGAAACGAG	CC1
KUL21.T_CGATCGGTCATGGTCA	CC2
KUL21.T_CGATGGCAGCCACCTG	CC1
KUL21.T_CGATGGCTCACATGCA	CC2
KUL21.T_CGATGTAAGCCATCGC	CC2
KUL21.T_CGCCAAGTCGGACAAG	CC2
KUL21.T_CGCGGTACAAGGACTG	CC1
KUL21.T_CGCGTTTCAAGAAGAG	CC2
KUL21.T_CGCTATCGTCGGCACT	CC2
KUL21.T_CGCTATCGTGGTACAG	CC1
KUL21.T_CGCTGGACACGGTTTA	CC1
KUL21.T_CGCTGGACATGCAACT	CC1
KUL21.T_CGCTGGATCAACTCTT	CC2
KUL21.T_CGCTGGATCTATCCTA	CC1
KUL21.T_CGCTTCAGTTATGCGT	CC2
KUL21.T_CGGACACCAATAAGCA	CC2
KUL21.T_CGGACGTCAATCGGTT	CC2
KUL21.T_CGGACGTTCCTCCTAG	CC2
KUL21.T_CGGACTGTCAGCTCGG	CC1
KUL21.T_CGGAGTCAGATGTGTA	CC2
KUL21.T_CGGAGTCTCGTTTATC	CC2
KUL21.T_CGGCTAGGTGTAAGTA	CC1

KUL21.T_CGGGTCAAGACAATAC	CC2
KUL21.T_CGGGTCAAGCGAAGGG	CC2
KUL21.T_CGGGTCACACTGTTAG	CC2
KUL21.T_CGGGTCATCCGTCAAA	CC1
KUL21.T_CGGTTAACAATGTAAG	CC1
KUL21.T_CGTAGCGGTGAGTGAC	CC1
KUL21.T_CGTAGGCCATGCGCAC	CC1
KUL21.T_CGTCAGGGTGCAACGA	CC1
KUL21.T_CGTCAGGTCCACGACG	CC1
KUL21.T_CGTCCATGTATAGTAG	CC2
KUL21.T_CGTGAGCTCGATCCCT	CC2
KUL21.T_CGTGTAAAGCGATCCC	CC2
KUL21.T_CGTGTCTTCCCTCTTT	CC2
KUL21.T_CGTTAGACATTTCACT	CC2
KUL21.T_CGTTCTGAGTCGATAA	CC1
KUL21.T_CGTTGGGTCGGGAGTA	CC2
KUL21.T_CTAACTTAGGCCGAAT	CC1
KUL21.T_CTAACTTGTTCTGAAC	CC2
KUL21.T_CTAACTTTCCAGAGGA	CC2
KUL21.T_CTAACTTTCTTTACAC	CC1
KUL21.T_CTAAGACAGGGCATGT	CC1
KUL21.T_CTAAGACCATTCTTAC	CC2
KUL21.T_CTAAGACGTCAGAGGT	CC2
KUL21.T_CTAAGACTCGTAGGTT	CC1
KUL21.T_CTAATGGAGCAAATCA	CC2
KUL21.T_CTACACCGTGCCTTGG	CC1
KUL21.T_CTACCCAAGAATGTGT	CC2
KUL21.T_CTACCCACACCTTGTC	CC2
KUL21.T_CTACCCAGTTTAGCTG	CC1
KUL21.T_CTACCCATCCTGCAGG	CC1
KUL21.T_CTACGTCCACTAGTAC	CC2
KUL21.T_CTACGTCGTTCAGACT	CC2
KUL21.T_CTACGTCGTTTGACAC	CC2
KUL21.T_CTACGTCTCGGCCGAT	CC2
KUL21.T_CTAGAGTAGACGCTTT	CC1
KUL21.T_CTAGAGTCAATGCCAT	CC1
KUL21.T_CTAGAGTCAGCTTCGG	CC2
KUL21.T_CTAGAGTCATGCCTTC	CC1
KUL21.T_CTAGAGTGTGGCCCTA	CC1
KUL21.T_CTAGAGTTCAGCGACC	CC1
KUL21.T_CTAGAGTTCCAGATCA	CC2
KUL21.T_CTAGCCTAGGTGCAAC	CC2
KUL21.T_CTAGCCTAGTACGCCC	CC2
KUL21.T_CTAGCCTCAGGGTACA	CC1
KUL21.T_CTAGCCTTCCCTAATT	CC1
KUL21.T_CTAGTGAAGGCGATAC	CC2
KUL21.T_CTAGTGAGTTCGTGAT	CC1
KUL21.T_CTAGTGATCGCCAAAT	CC1
KUL21.T_CTCACACAGTCGTTTG	CC2
KUL21.T_CTCACACGTCTGCCAG	CC2
KUL21.T_CTCACACGTTGAACTC	CC2
KUL21.T_CTCATTACACAGGCCT	CC1
KUL21.T_CTCCTAGCAATCTGCA	CC1
KUL21.T_CTCCTAGCAGCCTTTC	CC1
KUL21.T_CTCGAAAGTACGACCC	CC2
KUL21.T_CTCGAAATCACTCCTG	CC1
KUL21.T_CTCGGGACAATAAGCA	CC1
KUL21.T_CTCGGGATCGGATGTT	CC2

KUL21.T_CTCGTCAGTCAAACTC	CC1
KUL21.T_CTGAAACAGTTAGGTA	CC1
KUL21.T_CTGAAACGTATGGTTC	CC2
KUL21.T_CTGAAGTTCTCTTGAT	CC2
KUL21.T_CTGATAGGTCTCCATC	CC2
KUL21.T_CTGATCCAGTCTTGCA	CC1
KUL21.T_CTGATCCTCCATGCTC	CC2
KUL21.T_CTGCGGAAGCTACCGC	CC1
KUL21.T_CTGCGGAAGGGTCTCC	CC2
KUL21.T_CTGCTGTGTGTGACCC	CC1
KUL21.T_CTGCTGTTCAGTGTTG	CC1
KUL21.T_CTGGTCTAGATCTGAA	CC1
KUL21.T_CTGGTCTCACGAAATA	CC2
KUL21.T_CTGGTCTGTCTACCTC	CC2
KUL21.T_CTGTGCTCAGCTGCTG	CC2
KUL21.T_CTGTGCTCATCGGGTC	CC2
KUL21.T_CTTAACTCATCCGTGG	CC1
KUL21.T_CTTAACTGTAAGGGAA	CC2
KUL21.T_CTTAACTGTCCTCTTG	CC2
KUL21.T_CTTACCGAGCGTTGCC	CC1
KUL21.T_CTTACCGAGTAGCGGT	CC1
KUL21.T_CTTACCGGTTTAGGAA	CC1
KUL21.T_CTTACCGTCTCTGCTG	CC2
KUL21.T_CTTAGGACAGCCTATA	CC1
KUL21.T_CTTAGGACAGTTCCCT	CC1
KUL21.T_CTTAGGAGTTAAGAAC	CC1
KUL21.T_CTTCTCTAGATGTGGC	CC1
KUL21.T_CTTCTCTGTAGCGCAA	CC2
KUL21.T_CTTCTCTTCATCGATG	CC2
KUL21.T_CTTGGCTCATTCCTGC	CC1
KUL21.T_CTTGGCTTCCTAGAAC	CC2
KUL21.T_CTTTGCGAGAATCTCC	CC1
KUL21.T_CTTTGCGCAATCCAAC	CC1
KUL21.T_GAAACTCAGATCTGAA	CC2
KUL21.T_GAAACTCAGTTACGGG	CC1
KUL21.T_GAAACTCGTAAGCACG	CC2
KUL21.T_GAAATGAGTGACTACT	CC1
KUL21.T_GAACATCGTATGAATG	CC2
KUL21.T_GAACCTAAGGCTAGGT	CC2
KUL21.T_GAACCTATCCAAACTG	CC2
KUL21.T_GAACGGATCCACGAAT	CC1
KUL21.T_GAACGGATCTGCTGCT	CC1
KUL21.T_GAATAAGGTCGAAAGC	CC1
KUL21.T_GAATAAGTCGCGATCG	CC1
KUL21.T_GAATGAAAGTGTACCT	CC2
KUL21.T_GAATGAACAGCGATCC	CC2
KUL21.T_GAATGAACAGCTATTG	CC2
KUL21.T_GACACGCGTGATGTCT	CC1
KUL21.T_GACAGAGAGGATCGCA	CC1
KUL21.T_GACAGAGCAGACAGGT	CC2
KUL21.T_GACAGAGTCAGCTCGG	CC2
KUL21.T_GACCAATAGCTACCGC	CC1
KUL21.T_GACCAATCAGGATTGG	CC2
KUL21.T_GACCAATTCAAGGCTT	CC1
KUL21.T_GACCTGGGTTACCGAT	CC1
KUL21.T_GACGCGTAGGCATTGG	CC2
KUL21.T_GACGGCTCATGAAGTA	CC2
KUL21.T_GACGGCTGTCCGAGTC	CC2

KUL21.T_GACGTGCTCCTACAGA	CC2
KUL21.T_GACGTTAAGGCAATTA	CC2
KUL21.T_GACGTTAGTCTAGTCA	CC1
KUL21.T_GACGTTATCGCAAGCC	CC1
KUL21.T_GACGTTATCGCGCCAA	CC1
KUL21.T_GACTAACAGCTGTTCA	CC2
KUL21.T_GACTACAGTCAGAAGC	CC2
KUL21.T_GACTGCGTCTTCAACT	CC1
KUL21.T_GAGCAGAAGAAACGAG	CC2
KUL21.T_GAGTCCGGTAGCTTGT	CC1
KUL21.T_GATCGATGTGACTACT	CC1
KUL21.T_GATCGCGGTATTCGTG	CC1
KUL21.T_GATCGTAAGATCGATA	CC1
KUL21.T_GATCTAGAGTGACTCT	CC1
KUL21.T_GATGAAAAGATGGCGT	CC2
KUL21.T_GATGAAACAGGAATGC	CC2
KUL21.T_GATGAAAGTACCGCTG	CC1
KUL21.T_GATGAAAGTCGCCATG	CC2
KUL21.T_GATGAAAGTCGTGGCT	CC2
KUL21.T_GATGAGGCATGAGCGA	CC2
KUL21.T_GATGAGGGTTTGGCGC	CC2
KUL21.T_GATGCTATCATAAAGG	CC2
KUL21.T_GCAAACTAGTGCCATT	CC2
KUL21.T_GCAATCACAATAAGCA	CC1
KUL21.T_GCACATACACCTGGTG	CC1
KUL21.T_GCACATAGTACGCACC	CC2
KUL21.T_GCACTCTGTCAAACTC	CC1
KUL21.T_GCACTCTGTCTAGCCG	CC1
KUL21.T_GCAGCCAAGCCTATGT	CC2
KUL21.T_GCATGATCACCAGATT	CC2
KUL21.T_GCATGCGAGTGTCTCA	CC1
KUL21.T_GCATGCGCAATAACGA	CC1
KUL21.T_GCATGCGCAGATGAGC	CC1
KUL21.T_GCATGCGGTTAAGGGC	CC2
KUL21.T_GCATGTAAGTACGTTC	CC2
KUL21.T_GCCAAATGTAATAGCA	CC2
KUL21.T_GCCTCTATCTCGATGA	CC2
KUL21.T_GCGACCAAGACGCTTT	CC2
KUL21.T_GCGACCACAGCAGTTT	CC2
KUL21.T_GCGACCAGTCCAGTGC	CC2
KUL21.T_GCGACCATCTGTTGAG	CC2
KUL21.T_GCGAGAACAATGACCT	CC2
KUL21.T_GCGAGAAGTCAGGACA	CC2
KUL21.T_GCGAGAAGTTGACGTT	CC2
KUL21.T_GCGAGAATCCAATGGT	CC2
KUL21.T_GCGCAACAGGGTCGAT	CC1
KUL21.T_GCGCAACCAGCGAACA	CC2
KUL21.T_GCGCAGTCAGACTCGC	CC1
KUL21.T_GCGCAGTTCCCTTGCA	CC2
KUL21.T_GCGCCAACAAGACGTG	CC1
KUL21.T_GCGCCAAGTTACCGAT	CC2
KUL21.T_GCGCGATCAAACGTGG	CC1
KUL21.T_GCTCCTAAGCCCTAAT	CC2
KUL21.T_GCTCCTAGTAGATTAG	CC1
KUL21.T_GCTCCTATCCCAGGTG	CC1
KUL21.T_GCTCTGTCATGTTCCC	CC1
KUL21.T_GCTCTGTGTGTAAGTA	CC2
KUL21.T_GCTGCAGAGGCCATAG	CC2

KUL21.T_GCTGCGAAGATGAGAG	CC2
KUL21.T_GCTGCGAGTCACCCAG	CC1
KUL21.T_GCTGCTTAGAATGTTG	CC1
KUL21.T_GCTGCTTAGAGGGATA	CC1
KUL21.T_GCTGCTTCAATGCCAT	CC2
KUL21.T_GCTGCTTCACTGTTAG	CC2
KUL21.T_GCTGGGTAGTAGATGT	CC2
KUL21.T_GCTGGGTCATGGAATA	CC2
KUL21.T_GCTGGGTCATTCTTAC	CC2
KUL21.T_GCTGGGTTCCGTTGTC	CC1
KUL21.T_GCTTGAATCTTAGCCC	CC2
KUL21.T_GGAAAGCAGGAGTTGC	CC1
KUL21.T_GGAACTTAGGCGTACA	CC2
KUL21.T_GGAATAACAGGCTCAC	CC1
KUL21.T_GGAATAACATGCGCAC	CC2
KUL21.T_GGAATAAGTCTCATCC	CC1
KUL21.T_GGACAGAAGCTTATCG	CC1
KUL21.T_GGACAGACAGAGCCAA	CC2
KUL21.T_GGACAGAGTCTCTCTG	CC2
KUL21.T_GGACAGATCAGGTTCA	CC2
KUL21.T_GGAGCAAAGGCTAGGT	CC2
KUL21.T_GGAGCAAAGTTATCGC	CC2
KUL21.T_GGAGCAACAGATGGCA	CC1
KUL21.T_GGATGTTAGTACGTAA	CC2
KUL21.T_GGATTACCACAGACTT	CC1
KUL21.T_GGCCGATAGCGCCTCA	CC1
KUL21.T_GGCCGATGTCCATGAT	CC1
KUL21.T_GGCGTGTAGGTACTCT	CC2
KUL21.T_GGCGTGTCATGTTCCC	CC1
KUL21.T_GGCTCGAAGGCCGAAT	CC2
KUL21.T_GGCTCGACACCAGGTC	CC2
KUL21.T_GGCTGGTAGTACGTAA	CC1
KUL21.T_GGCTGGTTCAATACCG	CC2
KUL21.T_GGGAATGTCTGTGCAA	CC1
KUL21.T_GGGACCTTCCAAACAC	CC2
KUL21.T_GGGAGATAGAAGGACA	CC1
KUL21.T_GGGAGATAGCTGTTCA	CC1
KUL21.T_GGGAGATTCCTACAGA	CC1
KUL21.T_GGGAGATTCTGGTTCC	CC2
KUL21.T_GGGATGAAGTCGATAA	CC1
KUL21.T_GGGATGACACCAACCG	CC1
KUL21.T_GGGATGAGTAAGTTCC	CC2
KUL21.T_GGGCACTGTCTCAACA	CC1
KUL21.T_GGGCATCAGAGTCTGG	CC2
KUL21.T_GGGTCTGAGCTCCTCT	CC2
KUL21.T_GGGTCTGGTCCATCCT	CC2
KUL21.T_GGGTTGCCATTGGTAC	CC2
KUL21.T_GGTGAAGCAGTAAGAT	CC2
KUL21.T_GGTGAAGGTGTGCGTC	CC2
KUL21.T_GGTGAAGTCGGCGGTT	CC1
KUL21.T_GGTGCGTCACAACGCC	CC2
KUL21.T_GGTGTTAAGTCCGTAT	CC2
KUL21.T_GGTGTTAGTGGTCCGT	CC2
KUL21.T_GGTGTTATCATCATTC	CC1
KUL21.T_GTAACGTCACTGCCAG	CC2
KUL21.T_GTAACGTCACTTAAGC	CC1
KUL21.T_GTAACGTGTGTTGGGA	CC1
KUL21.T_GTAACTGTCAAACAAG	CC2

KUL21.T_GTACGTACAGCGTCCA	CC2
KUL21.T_GTACGTAGTTCCACAA	CC2
KUL21.T_GTACGTATCCCTCTTT	CC2
KUL21.T_GTACTCCAGTCGAGTG	CC1
KUL21.T_GTACTCCGTTCTGTTT	CC1
KUL21.T_GTACTTTAGTGTCCCG	CC1
KUL21.T_GTACTTTCAAACAACA	CC1
KUL21.T_GTACTTTTCACGATGT	CC2
KUL21.T_GTAGGCCGTTCGCGAC	CC1
KUL21.T_GTATTCTCATTACGAC	CC2
KUL21.T_GTATTCTTCCATGAAC	CC1
KUL21.T_GTCAAGTAGCGTTCCG	CC2
KUL21.T_GTCAAGTTCGGTCTAA	CC2
KUL21.T_GTCACAATCTGATACG	CC1
KUL21.T_GTCATTTTCTCGCATC	CC2
KUL21.T_GTCCTCAGTTCCCTTG	CC1
KUL21.T_GTCCTCATCACAACGT	CC1
KUL21.T_GTCGGGTCATGGTCAT	CC1
KUL21.T_GTCGGGTGTGCCTGTG	CC2
KUL21.T_GTCGGGTTCCCTCTTT	CC2
KUL21.T_GTCGTAAAGAGGGCTT	CC1
KUL21.T_GTCGTAATCCAGTAGT	CC1
KUL21.T_GTCTCGTAGGTACTCT	CC2
KUL21.T_GTCTTCGTCCAGAGGA	CC2
KUL21.T_GTGAAGGTCTCTTGAT	CC2
KUL21.T_GTGCAGCAGGCTCAGA	CC2
KUL21.T_GTGCAGCGTCAAACTC	CC2
KUL21.T_GTGCGGTTCGTTTGCC	CC1
KUL21.T_GTGCTTCAGACGCAAC	CC1
KUL21.T_GTGCTTCCATACTACG	CC1
KUL21.T_GTGCTTCTCCCAGGTG	CC2
KUL21.T_GTGTGCGAGAGTACAT	CC2
KUL21.T_GTTAAGCGTCTCACCT	CC2
KUL21.T_GTTACAGTCCCTAATT	CC1
KUL21.T_GTTCATTCAGGAACGT	CC1
KUL21.T_GTTCGGGAGGCCCGTT	CC2
KUL21.T_GTTCGGGGTGCAGACA	CC2
KUL21.T_GTTCGGGGTTCCCTTG	CC1
KUL21.T_GTTCTCGGTATAAACG	CC2
KUL21.T_GTTCTCGGTCCTAGCG	CC1
KUL21.T_GTTCTCGGTTTGGCGC	CC2
KUL21.T_GTTCTCGTCCACTCCA	CC2
KUL21.T_GTTCTCGTCCAGGGCT	CC1
KUL21.T_TAAACCGCACCCATTC	CC1
KUL21.T_TAAACCGTCACCAGGC	CC2
KUL21.T_TAAACCGTCTGCTGCT	CC1
KUL21.T_TAAGAGAGTCCAGTTA	CC2
KUL21.T_TAAGCGTCACTAGTAC	CC1
KUL21.T_TAAGCGTGTATAATGG	CC1
KUL21.T_TAAGCGTTCCCTCAGT	CC1
KUL21.T_TAAGCGTTCTGAAAGA	CC2
KUL21.T_TAAGTGCAGACAGAGA	CC2
KUL21.T_TAAGTGCTCCATGCTC	CC2
KUL21.T_TACAGTGTCATGTGGT	CC1
KUL21.T_TACCTATTCCTAGTGA	CC2
KUL21.T_TACCTTACACGAAATA	CC1
KUL21.T_TACGGATCAGCTATTG	CC1
KUL21.T_TACGGATCATCGATGT	CC1

KUL21.T_TACGGTACATTGAGCT	CC2
KUL21.T_TACTCATAGGAGTTTA	CC1
KUL21.T_TACTCGCAGCCCAACC	CC2
KUL21.T_TACTCGCAGTAATCCC	CC2
KUL21.T_TACTTACAGGCCGAAT	CC1
KUL21.T_TACTTGTAGGTCGGAT	CC2
KUL21.T_TAGACCACAAGGTTTC	CC1
KUL21.T_TAGCCGGAGTGGAGAA	CC2
KUL21.T_TAGGCATGTGACGCCT	CC2
KUL21.T_TAGTGGTAGCTAGTGG	CC1
KUL21.T_TAGTGGTGTCCGTCAG	CC1
KUL21.T_TAGTTGGAGAGTCTGG	CC1
KUL21.T_TAGTTGGAGATGCCTT	CC2
KUL21.T_TAGTTGGAGTCTCCTC	CC1
KUL21.T_TATCAGGCAAAGGCGT	CC2
KUL21.T_TATCTCACACCAGCAC	CC2
KUL21.T_TATCTCACAGCAGTTT	CC2
KUL21.T_TATGCCCAGCCACGCT	CC1
KUL21.T_TATTACCAGGCACATG	CC1
KUL21.T_TATTACCGTCGACTGC	CC2
KUL21.T_TCAATCTAGAGCTATA	CC2
KUL21.T_TCACAAGGTATCACCA	CC1
KUL21.T_TCACAAGTCATGTCTT	CC2
KUL21.T_TCACAAGTCCGCGTTT	CC2
KUL21.T_TCACGAAGTCCGTTAA	CC2
KUL21.T_TCACGAATCACTCTTA	CC1
KUL21.T_TCAGCAAAGAGGTACC	CC1
KUL21.T_TCAGCTCAGGGTCGAT	CC1
KUL21.T_TCAGCTCCACGAGAGT	CC2
KUL21.T_TCAGCTCTCCCATTAT	CC1
KUL21.T_TCATTACCACGTAAGG	CC1
KUL21.T_TCATTACGTAGGCTGA	CC2
KUL21.T_TCATTACTCAACGGCC	CC1
KUL21.T_TCATTTGGTAGCTCCG	CC2
KUL21.T_TCATTTGTCCGCGCAA	CC2
KUL21.T_TCCACACGTGCACGAA	CC1
KUL21.T_TCCACACGTGGTACAG	CC1
KUL21.T_TCGAGGCAGTATCGAA	CC1
KUL21.T_TCGCGAGAGACTGTAA	CC1
KUL21.T_TCGCGAGCAGGGAGAG	CC2
KUL21.T_TCGGGACGTCCGTGAC	CC2
KUL21.T_TCGTACCAGCGATGAC	CC1
KUL21.T_TCGTACCGTCGAATCT	CC1
KUL21.T_TCGTACCTCATGGTCA	CC2
KUL21.T_TCGTAGATCGGACAAG	CC1
KUL21.T_TCTATTGGTGTTCTTT	CC1
KUL21.T_TCTATTGTCTGGCGTG	CC2
KUL21.T_TCTCATAGTGGTACAG	CC2
KUL21.T_TCTCTAACAGACGCTC	CC2
KUL21.T_TCTCTAAGTACAGTGG	CC1
KUL21.T_TCTCTAAGTGCAGACA	CC2
KUL21.T_TCTGAGAAGGGTCGAT	CC2
KUL21.T_TCTGAGATCAGGTTCA	CC2
KUL21.T_TCTGGAAGTCACCCAG	CC2
KUL21.T_TCTTCGGTCGTTACGA	CC1
KUL21.T_TCTTTCCAGGTGACCA	CC2
KUL21.T_TGAAAGAAGCGAGAAA	CC2
KUL21.T_TGAAAGAAGTACACCT	CC1

KUL21.T_TGACGGCAGAGCCCAA	CC2
KUL21.T_TGACGGCAGGGTTCCC	CC2
KUL21.T_TGACGGCTCTGATACG	CC2
KUL21.T_TGACTAGAGAACAACT	CC2
KUL21.T_TGACTAGCACCCAGTG	CC2
KUL21.T_TGAGAGGAGGACTGGT	CC1
KUL21.T_TGAGAGGCACGCATCG	CC2
KUL21.T_TGAGCCGAGAGTGACC	CC1
KUL21.T_TGAGCCGGTCAGAATA	CC1
KUL21.T_TGAGGGAGTCCATCCT	CC1
KUL21.T_TGATTTCTCCTCAATT	CC2
KUL21.T_TGCACCTAGGAGCGAG	CC2
KUL21.T_TGCCAAAAGGACAGAA	CC1
KUL21.T_TGCCAAAAGTGTCCCG	CC2
KUL21.T_TGCCAAACAGCGATCC	CC1
KUL21.T_TGCCAAATCTGCTGTC	CC1
KUL21.T_TGCCCATAGCAACGGT	CC2
KUL21.T_TGCCCATGTGAAGGCT	CC1
KUL21.T_TGCCCATTCTTAGCCC	CC2
KUL21.T_TGCCCTACACGGCCAT	CC2
KUL21.T_TGCCCTAGTTCCACAA	CC1
KUL21.T_TGCGGGTCAATGGTCT	CC1
KUL21.T_TGCGGGTTCCTAGTGA	CC1
KUL21.T_TGCGGGTTCTGTCTCG	CC2
KUL21.T_TGCTACCGTATCTGCA	CC2
KUL21.T_TGCTGCTAGCAGATCG	CC1
KUL21.T_TGCTGCTAGGCATGGT	CC2
KUL21.T_TGCTGCTGTCTTGATG	CC1
KUL21.T_TGCTGCTGTGCAGGTA	CC2
KUL21.T_TGCTGCTTCCTGCAGG	CC2
KUL21.T_TGGACGCCAGGAATCG	CC1
KUL21.T_TGGACGCCATACCATG	CC2
KUL21.T_TGGACGCGTAAACACA	CC1
KUL21.T_TGGACGCGTAGAAGGA	CC1
KUL21.T_TGGACGCGTTGGACCC	CC2
KUL21.T_TGGCCAGCATACAGCT	CC1
KUL21.T_TGGCGCAGTGGCAAAC	CC2
KUL21.T_TGGGAAGCAAAGAATC	CC2
KUL21.T_TGGGCGTCATGAGCGA	CC1
KUL21.T_TGGGCGTTCAACGGCC	CC2
KUL21.T_TGGTTAGCACTTCGAA	CC2
KUL21.T_TGGTTAGGTTCGTGAT	CC2
KUL21.T_TGGTTAGTCAGAGGTG	CC2
KUL21.T_TGGTTCCGTAATTGGA	CC1
KUL21.T_TGTATTCCAGGAATGC	CC2
KUL21.T_TGTATTCGTTGACGTT	CC1
KUL21.T_TGTGTTTCAAGAGGCT	CC2
KUL21.T_TGTGTTTCAAGCCCAC	CC2
KUL21.T_TGTGTTTCATGCGCAC	CC2
KUL21.T_TGTGTTTGTGGTTTCA	CC1
KUL21.T_TGTTCCGCAAGCTGGA	CC2
KUL21.T_TGTTCCGGTACAAGTA	CC1
KUL21.T_TTAACTCCATGTCTCC	CC2
KUL21.T_TTAGGACAGTCCATAC	CC1
KUL21.T_TTAGGACTCAGGATCT	CC1
KUL21.T_TTAGGCAGTGGTAACG	CC1
KUL21.T_TTAGGCAGTTCCCTTG	CC1
KUL21.T_TTAGTTCGTCTCAACA	CC2

KUL21.T_TTATGCTCACGCGAAA	CC1
KUL21.T_TTCCCAGAGGGTGTGT	CC2
KUL21.T_TTCCCAGAGGTAGCCA	CC2
KUL21.T_TTCCCAGCAAGGACAC	CC1
KUL21.T_TTCGAAGTCAGGCGAA	CC2
KUL21.T_TTCGGTCAGACCTAGG	CC1
KUL21.T_TTCGGTCCAGGTGCCT	CC1
KUL21.T_TTCTACACACTACAGT	CC1
KUL21.T_TTCTACAGTGGGTATG	CC2
KUL21.T_TTCTCAACAGTAACGG	CC1
KUL21.T_TTCTCAATCCAGAAGG	CC2
KUL21.T_TTCTCAATCGTCTGAA	CC2
KUL21.T_TTCTCCTTCTGGAGCC	CC1
KUL21.T_TTCTTAGAGGCCCTCA	CC2
KUL21.T_TTGAACGCAAGCTGAG	CC2
KUL21.T_TTGCCGTTCTGCTTGC	CC1
KUL21.T_TTGCGTCTCAGCGATT	CC2
KUL21.T_TTGGAACAGTGTTAGA	CC2
KUL21.T_TTGGAACCAGGGAGAG	CC2
KUL21.T_TTGGCAAAGACTCGGA	CC2
KUL21.T_TTGGCAAAGGCAGTCA	CC2
KUL21.T_TTGGCAAAGTGCTGCC	CC2
KUL21.T_TTGGCAAAGTTGCAGG	CC2
KUL21.T_TTGGCAACAATCCAAC	CC1
KUL21.T_TTGTAGGGTTCGCTAA	CC1
KUL21.T_TTTCCTCAGCGGATCA	CC2
KUL21.T_TTTCCTCCATTCTCAT	CC1
KUL21.T_TTTCCTCGTATGGTTC	CC2
KUL21.T_TTTGCGCCACCCATTC	CC1
KUL21.T_TTTGGTTGTCCTGCTT	CC2
KUL21.T_TTTGGTTTCAGCCTAA	CC2
KUL21.T_TTTGGTTTCCTATTCA	CC2
KUL21.T_TTTGTCAAGCCTATGT	CC1
KUL21.T_TTTGTCACACTCGACG	CC1
KUL21.T_TTTGTCATCTTACCTA	CC2
KUL28.T_AAGGAGCGTGAGTATA	CC1
KUL28.T_ACATGGTAGCAGATCG	CC1
KUL28.T_ACGATACGTCTCTCGT	CC1
KUL28.T_ACGTCAAGTGTTGAGG	CC1
KUL28.T_AGCCTAAAGAGTACAT	CC1
KUL28.T_AGCGTATTCCTAGTGA	CC1
KUL28.T_AGCTCTCAGCGTTGCC	CC1
KUL28.T_AGTGTCATCAACACAC	CC1
KUL28.T_AGTTGGTAGCGCTCCA	CC1
KUL28.T_AGTTGGTTCAATAAGG	CC1
KUL28.T_ATCTGCCTCCCGACTT	CC1
KUL28.T_CACAGGCGTCTCTCTG	CC1
KUL28.T_CAGATCAGTACAGTGG	CC1
KUL28.T_CCATGTCTCTGTTGAG	CC1
KUL28.T_CGAGAAGCACGTAAGG	CC1
KUL28.T_CGGGTCATCGAACTGT	CC1
KUL28.T_CGTCAGGTCCATGAGT	CC1
KUL28.T_CTGAAGTAGATGTCGG	CC1
KUL28.T_CTGCTGTCATTAACCG	CC1
KUL28.T_CTGGTCTTCTAACTCT	CC1
KUL28.T_GCTGGGTAGAAGGGTA	CC1
KUL28.T_GGAACTTCAAGAGGCT	CC1
KUL28.T_GGACAAGTCCACTCCA	CC1

KUL28.T_GTAACGTCACATTCGA	CC1
KUL28.T_GTCGGGTCACTTGGAT	CC1
KUL28.T_TAGCCGGCAACCGCCA	CC1
KUL28.T_TCACAAGAGAGGTTGC	CC1
KUL28.T_TGAAAGACATCCGTGG	CC1
KUL28.T_TGACTAGCAGCCAATT	CC1
KUL28.T_TGAGAGGTCATTCACT	CC1
KUL28.T_TGAGCCGAGACAGGCT	CC1
KUL28.T_TGATTTCCAAGCGATG	CC1
KUL28.T_TGCCAAAGTAGGGACT	CC1
KUL28.T_TGGTTAGGTGATGTGG	CC1
KUL28.T_TGTTCCGAGAAGGGTA	CC1
KUL28.T_TTAACTCGTATATGAG	CC1
KUL28.T_TTAGGCAAGACACTAA	CC1
KUL28.T_TTCTCAACAGCTGCTG	CC1
KUL28.T_TTGCCGTAGAGATGAG	CC1
KUL28.T_TTGCCGTTCAGGTAAA	CC1
KUL30.T_AAACCTGCACAAGACG	CC1
KUL30.T_AAAGATGCAGTGACAG	CC1
KUL30.T_AACCGCGAGCGGATCA	CC2
KUL30.T_AACTCAGGTCAGAATA	CC1
KUL30.T_AAGTCTGGTTCAACCA	CC2
KUL30.T_ACATGGTAGTTATCGC	CC2
KUL30.T_ACGCCAGAGTAAGTAC	CC1
KUL30.T_ACGTCAAGTGCCTGCA	CC1
KUL30.T_ACTGATGTCAGGCAAG	CC1
KUL30.T_AGCTTGACAGGGATTG	CC1
KUL30.T_AGGGTGAGTGAGCGAT	CC1
KUL30.T_AGGGTGATCATTGCGA	CC1
KUL30.T_AGTGAGGTCTCCGGTT	CC1
KUL30.T_ATCCACCAGACTACAA	CC2
KUL30.T_ATTTCTGGTTCTGAAC	CC2
KUL30.T_CAAGAAAGTCAAAGCG	CC1
KUL30.T_CAAGTTGTCGGATGTT	CC1
KUL30.T_CAGATCAAGTTCGATC	CC1
KUL30.T_CAGATCAGTACTCTCC	CC1
KUL30.T_CAGCTGGGTTTCGCTC	CC1
KUL30.T_CATTATCGTGAGCGAT	CC1
KUL30.T_CCACGGACATTTCAGG	CC1
KUL30.T_CCACTACCAAGCGCTC	CC1
KUL30.T_CCGTGGAAGCAGCGTA	CC1
KUL30.T_CCTACACAGGCGCTCT	CC1
KUL30.T_CCTACACTCACTTCAT	CC2
KUL30.T_CCTAGCTGTAGCCTCG	CC1
KUL30.T_CCTTACGGTCCATCCT	CC2
KUL30.T_CCTTACGGTCCTCTTG	CC2
KUL30.T_CGAACATGTGGTCTCG	CC1
KUL30.T_CGCGGTAGTAGGGTAC	CC2
KUL30.T_CGCTTCATCTAACTGG	CC2
KUL30.T_CGGAGTCGTGTCGCTG	CC1
KUL30.T_CGTAGGCAGATCCCGC	CC2
KUL30.T_CTACCCAGTGATAAAC	CC1
KUL30.T_CTCGAGGAGGCTAGCA	CC1
KUL30.T_CTCGTCAGTTCTCATT	CC1
KUL30.T_CTCTACGTCGGAAACG	CC1
KUL30.T_CTGCTGTGTGATGCCC	CC1
KUL30.T_GAGTCCGCACGACGAA	CC1
KUL30.T_GCACATAAGAGGTACC	CC1

KUL30.T_GCCTCTAAGCTCCCAG	CC1
KUL30.T_GCGCGATTCGTTACGA	CC2
KUL30.T_GGACGTCAGTAGTGCG	CC1
KUL30.T_GGGAATGTCACGATGT	CC2
KUL30.T_GGGCACTTCTCGGACG	CC2
KUL30.T_GGGTTGCTCTCGGACG	CC2
KUL30.T_GGTGTTAGTCTGGTCG	CC1
KUL30.T_GTCAAGTGTCCGCTGA	CC1
KUL30.T_GTCTTCGTCAAGGTAA	CC1
KUL30.T_GTGGGTCGTACCAGTT	CC2
KUL30.T_TAAACCGCAAACTGCT	CC1
KUL30.T_TAGCCGGCATCGGACC	CC2
KUL30.T_TATCAGGGTCGAACAG	CC1
KUL30.T_TATCTCATCATTTGGG	CC1
KUL30.T_TCAACGAGTCCAGTAT	CC1
KUL30.T_TCGAGGCTCGGATGTT	CC1
KUL30.T_TGAGCCGTCTCGATGA	CC2
KUL30.T_TGAGCCGTCTTACCTA	CC1
KUL30.T_TGCCAAACAGGCGATA	CC1
KUL30.T_TGGACGCCAAGTTCTG	CC1
KUL30.T_TGGCTGGAGTATCTCG	CC1
KUL30.T_TGGTTAGTCCGTACAA	CC1
KUL30.T_TTCTCAATCATAACCG	CC2
KUL30.T_TTGCCGTTCGATAGAA	CC1
KUL30.T_TTGCGTCAGCACGCCT	CC2
KUL30.T_TTGGAACCAGTAGAGC	CC1
KUL30.T_TTTGGTTAGGACGAAA	CC1
KUL31.T_AACACGTGTCATCCCT	CC1
KUL31.T_AACTCAGGTCAACATC	CC1
KUL31.T_AAGCCGCAGTGTCCAT	CC1
KUL31.T_AATCGGTAGTCCCACG	CC1
KUL31.T_ACGCCGAGTAAACACA	CC1
KUL31.T_ACTTTCACATGCATGT	CC2
KUL31.T_AGAGCGAGTCGACTGC	CC1
KUL31.T_AGAGCTTTCCACTCCA	CC1
KUL31.T_ATCACGATCTACCAGA	CC1
KUL31.T_ATCCACCTCGGAGCAA	CC1
KUL31.T_CACAGTACAAGCCTAT	CC1
KUL31.T_CACCACTGTGCAACGA	CC1
KUL31.T_CACTCCAGTACATCCA	CC1
KUL31.T_CAGGTGCAGGTGCACA	CC1
KUL31.T_CATCCACTCTATCCCG	CC2
KUL31.T_CCATTCGGTAGCGTGA	CC1
KUL31.T_CCTCAGTAGACATAAC	CC1
KUL31.T_CGCCAAGTCCAGGGCT	CC1
KUL31.T_CGGAGCTCACTGTCGG	CC1
KUL31.T_CGTGAGCAGGAGTAGA	CC1
KUL31.T_CTAATGGAGCTTTGGT	CC1
KUL31.T_CTCGGAGCATTTGCCC	CC1
KUL31.T_CTGAAACAGTGTACGG	CC1
KUL31.T_GAAATGATCCTTGGTC	CC1
KUL31.T_GAACGGATCCTGCTTG	CC1
KUL31.T_GATCGTAGTAAGTAGT	CC1
KUL31.T_GCATGCGTCAGTTCGA	CC1
KUL31.T_GTAGTCAAGTCTCGGC	CC1
KUL31.T_GTATCTTCAAGAGTCG	CC1
KUL31.T_TAAACCGAGGTGATAT	CC1
KUL31.T_TAGCCGGTCGAACTGT	CC1

KUL31.T_TATCAGGCACGAAAGC	CC1
KUL31.T_TCAGATGTCCTTTACA	CC1
KUL31.T_TCTTTCCCAGCGTTCG	CC1
KUL31.T_TGACAACTCACTCTTA	CC1
KUL31.T_TGCTGCTCATCCAACA	CC2
KUL31.T_TGTTCCGCATGCTAGT	CC1
KUL31.T_TTCTTAGCATTCTTAC	CC1
KUL31.T_TTGAACGTCGGGAGTA	CC1
KUL31.T_TTGACTTGTAAACACA	CC1
KUL31.T_TTGACTTGTTATCACG	CC1

Table S5. TCGA gene clusters and genes A-C

samples	NMF clusters	Gene clusters
TCGA.A6.5656.01	CC1	Gene cluster A
TCGA.A6.5659.01	CC1	Gene cluster A
TCGA.A6.5662.01	CC1	Gene cluster A
TCGA.A6.5667.01	CC1	Gene cluster A
TCGA.A6.A56B.01	CC1	Gene cluster A
TCGA.AA.A01X.01	CC1	Gene cluster A
TCGA.AF.3911.01	CC1	Gene cluster A
TCGA.AF.4110.01	CC1	Gene cluster A
TCGA.AH.6643.01	CC1	Gene cluster A
TCGA.AM.5820.01	CC1	Gene cluster A
TCGA.CA.5797.01	CC1	Gene cluster A
TCGA.CA.6716.01	CC1	Gene cluster A
TCGA.CM.4747.01	CC1	Gene cluster A
TCGA.CM.5344.01	CC1	Gene cluster A
TCGA.CM.6164.01	CC1	Gene cluster A
TCGA.CM.6165.01	CC1	Gene cluster A
TCGA.CM.6678.01	CC1	Gene cluster A
TCGA.D5.6922.01	CC1	Gene cluster A
TCGA.D5.6924.01	CC1	Gene cluster A
TCGA.D5.6926.01	CC1	Gene cluster A
TCGA.DC.6681.01	CC1	Gene cluster A
TCGA.DC.6683.01	CC1	Gene cluster A
TCGA.DY.A1DD.01	CC1	Gene cluster A
TCGA.DY.A1DF.01	CC1	Gene cluster A
TCGA.EI.6514.01	CC1	Gene cluster A
TCGA.EI.6883.01	CC1	Gene cluster A
TCGA.EI.7002.01	CC1	Gene cluster A
TCGA.F4.6463.01	CC1	Gene cluster A
TCGA.F4.6854.01	CC1	Gene cluster A
TCGA.F5.6571.01	CC1	Gene cluster A
TCGA.F5.6814.01	CC1	Gene cluster A
TCGA.F5.6861.01	CC1	Gene cluster A
TCGA.F5.6863.01	CC1	Gene cluster A
TCGA.F5.6864.01	CC1	Gene cluster A
TCGA.G4.6303.01	CC1	Gene cluster A
TCGA.G4.6310.01	CC1	Gene cluster A
TCGA.NH.A8F8.01	CC1	Gene cluster A
TCGA.QG.A5Z1.01	CC1	Gene cluster A
TCGA.A6.6649.01	CC2	Gene cluster A
TCGA.AD.5900.01	CC2	Gene cluster A
TCGA.A6.2684.01	CC3	Gene cluster A
TCGA.A6.2685.01	CC3	Gene cluster A
TCGA.A6.5657.01	CC3	Gene cluster A
TCGA.A6.5664.01	CC3	Gene cluster A
TCGA.A6.6651.01	CC3	Gene cluster A
TCGA.AF.2687.01	CC3	Gene cluster A
TCGA.AF.2690.01	CC3	Gene cluster A
TCGA.AG.4022.01	CC3	Gene cluster A
TCGA.AH.6644.01	CC3	Gene cluster A
TCGA.CA.6717.01	CC3	Gene cluster A
TCGA.CA.6719.01	CC3	Gene cluster A
TCGA.CK.6748.01	CC3	Gene cluster A
TCGA.CM.5348.01	CC3	Gene cluster A
TCGA.CM.5349.01	CC3	Gene cluster A
TCGA.CM.6167.01	CC3	Gene cluster A

TCGA.CM.6168.01	CC3	Gene cluster A
TCGA.CM.6679.01	CC3	Gene cluster A
TCGA.D5.6898.01	CC3	Gene cluster A
TCGA.DT.5265.01	CC3	Gene cluster A
TCGA.DY.A1DE.01	CC3	Gene cluster A
TCGA.EI.6885.01	CC3	Gene cluster A
TCGA.EI.7004.01	CC3	Gene cluster A
TCGA.F4.6459.01	CC3	Gene cluster A
TCGA.F4.6460.01	CC3	Gene cluster A
TCGA.F4.6461.01	CC3	Gene cluster A
TCGA.F4.6704.01	CC3	Gene cluster A
TCGA.F4.6805.01	CC3	Gene cluster A
TCGA.F4.6807.01	CC3	Gene cluster A
TCGA.F4.6809.01	CC3	Gene cluster A
TCGA.F5.6464.01	CC3	Gene cluster A
TCGA.F5.6465.01	CC3	Gene cluster A
TCGA.F5.6702.01	CC3	Gene cluster A
TCGA.F5.6812.01	CC3	Gene cluster A
TCGA.F5.6813.01	CC3	Gene cluster A
TCGA.G4.6302.01	CC3	Gene cluster A
TCGA.G4.6314.01	CC3	Gene cluster A
TCGA.G4.6627.01	CC3	Gene cluster A
TCGA.A6.5660.01	CC1	Gene cluster B
TCGA.AA.3496.01	CC1	Gene cluster B
TCGA.AA.3662.01	CC1	Gene cluster B
TCGA.AZ.4684.01	CC1	Gene cluster B
TCGA.CM.5868.01	CC1	Gene cluster B
TCGA.CM.6170.01	CC1	Gene cluster B
TCGA.D5.5541.01	CC1	Gene cluster B
TCGA.D5.6929.01	CC1	Gene cluster B
TCGA.D5.6932.01	CC1	Gene cluster B
TCGA.DC.6154.01	CC1	Gene cluster B
TCGA.DM.A1HA.01	CC1	Gene cluster B
TCGA.DM.A28A.01	CC1	Gene cluster B
TCGA.EI.6509.01	CC1	Gene cluster B
TCGA.G4.6304.01	CC1	Gene cluster B
TCGA.NH.A6GA.01	CC1	Gene cluster B
TCGA.NH.A6GC.01	CC1	Gene cluster B
TCGA.A6.2686.01	CC2	Gene cluster B
TCGA.A6.5661.01	CC2	Gene cluster B
TCGA.A6.5665.01	CC2	Gene cluster B
TCGA.A6.6138.01	CC2	Gene cluster B
TCGA.A6.6653.01	CC2	Gene cluster B
TCGA.A6.6780.01	CC2	Gene cluster B
TCGA.AA.3492.01	CC2	Gene cluster B
TCGA.AA.3506.01	CC2	Gene cluster B
TCGA.AA.3663.01	CC2	Gene cluster B
TCGA.AA.3713.01	CC2	Gene cluster B
TCGA.AA.A01P.01	CC2	Gene cluster B
TCGA.AA.A02K.01	CC2	Gene cluster B
TCGA.AD.6548.01	CC2	Gene cluster B
TCGA.AD.6889.01	CC2	Gene cluster B
TCGA.AD.6895.01	CC2	Gene cluster B
TCGA.AD.A5EJ.01	CC2	Gene cluster B
TCGA.AF.6655.01	CC2	Gene cluster B
TCGA.AG.3902.01	CC2	Gene cluster B
TCGA.AM.5821.01	CC2	Gene cluster B
TCGA.AU.6004.01	CC2	Gene cluster B

TCGA.AZ.4315.01	CC2	Gene cluster B
TCGA.AZ.4615.01	CC2	Gene cluster B
TCGA.BM.6198.01	CC2	Gene cluster B
TCGA.CA.5254.01	CC2	Gene cluster B
TCGA.CA.6718.01	CC2	Gene cluster B
TCGA.CK.4948.01	CC2	Gene cluster B
TCGA.CK.4951.01	CC2	Gene cluster B
TCGA.CK.4952.01	CC2	Gene cluster B
TCGA.CK.5913.01	CC2	Gene cluster B
TCGA.CK.6751.01	CC2	Gene cluster B
TCGA.CM.4743.01	CC2	Gene cluster B
TCGA.CM.4751.01	CC2	Gene cluster B
TCGA.CM.5860.01	CC2	Gene cluster B
TCGA.CM.5861.01	CC2	Gene cluster B
TCGA.CM.5863.01	CC2	Gene cluster B
TCGA.CM.6162.01	CC2	Gene cluster B
TCGA.CM.6169.01	CC2	Gene cluster B
TCGA.CM.6171.01	CC2	Gene cluster B
TCGA.CM.6674.01	CC2	Gene cluster B
TCGA.CM.6675.01	CC2	Gene cluster B
TCGA.CM.6680.01	CC2	Gene cluster B
TCGA.D5.5538.01	CC2	Gene cluster B
TCGA.D5.5539.01	CC2	Gene cluster B
TCGA.D5.6529.01	CC2	Gene cluster B
TCGA.D5.6530.01	CC2	Gene cluster B
TCGA.D5.6531.01	CC2	Gene cluster B
TCGA.D5.6535.01	CC2	Gene cluster B
TCGA.D5.6536.01	CC2	Gene cluster B
TCGA.D5.6540.01	CC2	Gene cluster B
TCGA.D5.6541.01	CC2	Gene cluster B
TCGA.D5.6928.01	CC2	Gene cluster B
TCGA.D5.6930.01	CC2	Gene cluster B
TCGA.D5.6931.01	CC2	Gene cluster B
TCGA.D5.7000.01	CC2	Gene cluster B
TCGA.DC.6158.01	CC2	Gene cluster B
TCGA.DM.A1HB.01	CC2	Gene cluster B
TCGA.DM.A280.01	CC2	Gene cluster B
TCGA.DM.A28K.01	CC2	Gene cluster B
TCGA.EI.6511.01	CC2	Gene cluster B
TCGA.EI.6917.01	CC2	Gene cluster B
TCGA.F4.6570.01	CC2	Gene cluster B
TCGA.F5.6811.01	CC2	Gene cluster B
TCGA.G4.6297.01	CC2	Gene cluster B
TCGA.G4.6299.01	CC2	Gene cluster B
TCGA.G4.6311.01	CC2	Gene cluster B
TCGA.G4.6586.01	CC2	Gene cluster B
TCGA.G4.6588.01	CC2	Gene cluster B
TCGA.G4.6628.01	CC2	Gene cluster B
TCGA.NH.A50V.01	CC2	Gene cluster B
TCGA.NH.A5IV.01	CC2	Gene cluster B
TCGA.A6.2675.01	CC3	Gene cluster B
TCGA.A6.6142.01	CC3	Gene cluster B
TCGA.A6.6654.01	CC3	Gene cluster B
TCGA.A6.6781.01	CC3	Gene cluster B
TCGA.A6.6782.01	CC3	Gene cluster B
TCGA.A6.A5ZU.01	CC3	Gene cluster B
TCGA.AA.3489.01	CC3	Gene cluster B
TCGA.AD.6899.01	CC3	Gene cluster B

TCGA.AD.6901.01	CC3	Gene cluster B
TCGA.AD.6964.01	CC3	Gene cluster B
TCGA.AG.3731.01	CC3	Gene cluster B
TCGA.AG.4021.01	CC3	Gene cluster B
TCGA.AH.6547.01	CC3	Gene cluster B
TCGA.D5.6534.01	CC3	Gene cluster B
TCGA.DC.6156.01	CC3	Gene cluster B
TCGA.EI.6507.01	CC3	Gene cluster B
TCGA.F4.6569.01	CC3	Gene cluster B
TCGA.F4.6703.01	CC3	Gene cluster B
TCGA.F4.6855.01	CC3	Gene cluster B
TCGA.G4.6625.01	CC3	Gene cluster B
TCGA.WS.AB45.01	CC3	Gene cluster B
TCGA.3L.AA1B.01	CC1	Gene cluster C
TCGA.4N.A93T.01	CC1	Gene cluster C
TCGA.4T.AA8H.01	CC1	Gene cluster C
TCGA.5M.AATE.01	CC1	Gene cluster C
TCGA.A6.5666.01	CC1	Gene cluster C
TCGA.A6.6140.01	CC1	Gene cluster C
TCGA.A6.6648.01	CC1	Gene cluster C
TCGA.A6.6652.01	CC1	Gene cluster C
TCGA.AA.3509.01	CC1	Gene cluster C
TCGA.AA.3511.01	CC1	Gene cluster C
TCGA.AA.3660.01	CC1	Gene cluster C
TCGA.AA.3675.01	CC1	Gene cluster C
TCGA.AD.6888.01	CC1	Gene cluster C
TCGA.AD.6890.01	CC1	Gene cluster C
TCGA.AD.6965.01	CC1	Gene cluster C
TCGA.AD.A5EK.01	CC1	Gene cluster C
TCGA.AF.6136.01	CC1	Gene cluster C
TCGA.AF.6672.01	CC1	Gene cluster C
TCGA.AF.A56L.01	CC1	Gene cluster C
TCGA.AF.A56N.01	CC1	Gene cluster C
TCGA.AG.3732.01	CC1	Gene cluster C
TCGA.AH.6544.01	CC1	Gene cluster C
TCGA.AH.6549.01	CC1	Gene cluster C
TCGA.AH.6897.01	CC1	Gene cluster C
TCGA.AH.6903.01	CC1	Gene cluster C
TCGA.AY.A54L.01	CC1	Gene cluster C
TCGA.AY.A69D.01	CC1	Gene cluster C
TCGA.AY.A71X.01	CC1	Gene cluster C
TCGA.AY.A8YK.01	CC1	Gene cluster C
TCGA.CA.5255.01	CC1	Gene cluster C
TCGA.CA.5256.01	CC1	Gene cluster C
TCGA.CA.6715.01	CC1	Gene cluster C
TCGA.CI.6619.01	CC1	Gene cluster C
TCGA.CI.6620.01	CC1	Gene cluster C
TCGA.CI.6622.01	CC1	Gene cluster C
TCGA.CK.5912.01	CC1	Gene cluster C
TCGA.CK.5915.01	CC1	Gene cluster C
TCGA.CL.4957.01	CC1	Gene cluster C
TCGA.CL.5917.01	CC1	Gene cluster C
TCGA.CL.5918.01	CC1	Gene cluster C
TCGA.CM.5864.01	CC1	Gene cluster C
TCGA.CM.6161.01	CC1	Gene cluster C
TCGA.CM.6163.01	CC1	Gene cluster C
TCGA.CM.6166.01	CC1	Gene cluster C
TCGA.CM.6172.01	CC1	Gene cluster C

TCGA.CM.6676.01	CC1	Gene cluster C
TCGA.D5.5537.01	CC1	Gene cluster C
TCGA.D5.5540.01	CC1	Gene cluster C
TCGA.D5.6532.01	CC1	Gene cluster C
TCGA.D5.6533.01	CC1	Gene cluster C
TCGA.D5.6538.01	CC1	Gene cluster C
TCGA.D5.6923.01	CC1	Gene cluster C
TCGA.DC.4745.01	CC1	Gene cluster C
TCGA.DC.4749.01	CC1	Gene cluster C
TCGA.DC.5869.01	CC1	Gene cluster C
TCGA.DC.6155.01	CC1	Gene cluster C
TCGA.DC.6157.01	CC1	Gene cluster C
TCGA.DC.6682.01	CC1	Gene cluster C
TCGA.DM.A1D0.01	CC1	Gene cluster C
TCGA.DM.A1D4.01	CC1	Gene cluster C
TCGA.DM.A1D6.01	CC1	Gene cluster C
TCGA.DM.A1D7.01	CC1	Gene cluster C
TCGA.DM.A1D8.01	CC1	Gene cluster C
TCGA.DM.A1D9.01	CC1	Gene cluster C
TCGA.DM.A282.01	CC1	Gene cluster C
TCGA.DM.A288.01	CC1	Gene cluster C
TCGA.DM.A28C.01	CC1	Gene cluster C
TCGA.DM.A28E.01	CC1	Gene cluster C
TCGA.DM.A28F.01	CC1	Gene cluster C
TCGA.DM.A28G.01	CC1	Gene cluster C
TCGA.DM.A28H.01	CC1	Gene cluster C
TCGA.DY.A0XA.01	CC1	Gene cluster C
TCGA.DY.A1H8.01	CC1	Gene cluster C
TCGA.EF.5830.01	CC1	Gene cluster C
TCGA.EF.5831.01	CC1	Gene cluster C
TCGA.EI.6508.01	CC1	Gene cluster C
TCGA.EI.6512.01	CC1	Gene cluster C
TCGA.EI.6513.01	CC1	Gene cluster C
TCGA.EI.6881.01	CC1	Gene cluster C
TCGA.F4.6806.01	CC1	Gene cluster C
TCGA.F4.6808.01	CC1	Gene cluster C
TCGA.G4.6307.01	CC1	Gene cluster C
TCGA.G4.6315.01	CC1	Gene cluster C
TCGA.G4.6317.01	CC1	Gene cluster C
TCGA.G5.6235.01	CC1	Gene cluster C
TCGA.G5.6641.01	CC1	Gene cluster C
TCGA.NH.A50T.01	CC1	Gene cluster C
TCGA.NH.A8F7.01	CC1	Gene cluster C
TCGA.QG.A5YV.01	CC1	Gene cluster C
TCGA.QG.A5YX.01	CC1	Gene cluster C
TCGA.QL.A97D.01	CC1	Gene cluster C
TCGA.RU.A8FL.01	CC1	Gene cluster C
TCGA.SS.A7HO.01	CC1	Gene cluster C
TCGA.T9.A92H.01	CC1	Gene cluster C
TCGA.A6.6137.01	CC2	Gene cluster C
TCGA.A6.6141.01	CC2	Gene cluster C
TCGA.A6.6650.01	CC2	Gene cluster C
TCGA.AA.3495.01	CC2	Gene cluster C
TCGA.AA.3502.01	CC2	Gene cluster C
TCGA.AA.3526.01	CC2	Gene cluster C
TCGA.AA.3655.01	CC2	Gene cluster C
TCGA.AA.3685.01	CC2	Gene cluster C
TCGA.AA.3697.01	CC2	Gene cluster C

TCGA.AA.3712.01	CC2	Gene cluster C
TCGA.AA.A01Z.01	CC2	Gene cluster C
TCGA.AA.A02Y.01	CC2	Gene cluster C
TCGA.AD.6963.01	CC2	Gene cluster C
TCGA.AF.2693.01	CC2	Gene cluster C
TCGA.AF.5654.01	CC2	Gene cluster C
TCGA.AF.A56K.01	CC2	Gene cluster C
TCGA.AG.3591.01	CC2	Gene cluster C
TCGA.AG.3592.01	CC2	Gene cluster C
TCGA.AG.3725.01	CC2	Gene cluster C
TCGA.AG.3742.01	CC2	Gene cluster C
TCGA.AY.5543.01	CC2	Gene cluster C
TCGA.AY.6197.01	CC2	Gene cluster C
TCGA.AY.6386.01	CC2	Gene cluster C
TCGA.AZ.4313.01	CC2	Gene cluster C
TCGA.AZ.5407.01	CC2	Gene cluster C
TCGA.CA.5796.01	CC2	Gene cluster C
TCGA.CI.6621.01	CC2	Gene cluster C
TCGA.CI.6623.01	CC2	Gene cluster C
TCGA.CI.6624.01	CC2	Gene cluster C
TCGA.CK.4947.01	CC2	Gene cluster C
TCGA.CK.4950.01	CC2	Gene cluster C
TCGA.CK.5914.01	CC2	Gene cluster C
TCGA.CK.6747.01	CC2	Gene cluster C
TCGA.CM.4744.01	CC2	Gene cluster C
TCGA.CM.6677.01	CC2	Gene cluster C
TCGA.D5.6537.01	CC2	Gene cluster C
TCGA.D5.6539.01	CC2	Gene cluster C
TCGA.D5.6920.01	CC2	Gene cluster C
TCGA.DC.5337.01	CC2	Gene cluster C
TCGA.DC.6160.01	CC2	Gene cluster C
TCGA.DM.A0X9.01	CC2	Gene cluster C
TCGA.DM.A1DA.01	CC2	Gene cluster C
TCGA.DM.A1DB.01	CC2	Gene cluster C
TCGA.DM.A28M.01	CC2	Gene cluster C
TCGA.EI.6506.01	CC2	Gene cluster C
TCGA.EI.6510.01	CC2	Gene cluster C
TCGA.EI.6882.01	CC2	Gene cluster C
TCGA.EI.6884.01	CC2	Gene cluster C
TCGA.F4.6856.01	CC2	Gene cluster C
TCGA.G4.6293.01	CC2	Gene cluster C
TCGA.G4.6295.01	CC2	Gene cluster C
TCGA.G4.6306.01	CC2	Gene cluster C
TCGA.G4.6309.01	CC2	Gene cluster C
TCGA.G4.6320.01	CC2	Gene cluster C
TCGA.G4.6321.01	CC2	Gene cluster C
TCGA.G4.6322.01	CC2	Gene cluster C
TCGA.G4.6323.01	CC2	Gene cluster C
TCGA.NH.A6GB.01	CC2	Gene cluster C
TCGA.QG.A5YW.01	CC2	Gene cluster C
TCGA.QG.A5Z2.01	CC2	Gene cluster C

Table S5. meta-GEO gene clusters a

meta-GEO samples	Gene clusters
GSM358341	C
GSM358342	C
GSM358343	B
GSM358344	C
GSM358345	C
GSM358346	A
GSM358347	C
GSM358348	C
GSM358349	C
GSM358350	C
GSM358351	C
GSM358352	C
GSM358353	B
GSM358354	B
GSM358355	B
GSM358356	C
GSM358357	C
GSM358358	B
GSM358359	A
GSM358360	B
GSM358361	B
GSM358362	B
GSM358363	C
GSM358364	B
GSM358365	C
GSM358366	B
GSM358367	B
GSM358368	C
GSM358370	C
GSM358371	C
GSM358373	B
GSM358374	B
GSM358375	B
GSM358376	C
GSM358377	B
GSM358378	C
GSM358380	C
GSM358381	C
GSM358382	A
GSM358383	C
GSM358384	A
GSM358385	A
GSM358386	A
GSM358387	B
GSM358388	B
GSM358389	A
GSM358390	A
GSM358391	B
GSM358392	A
GSM358393	A
GSM358394	B
GSM358395	B
GSM358396	C
GSM358397	A
GSM358398	B

GSM358399	A
GSM358400	C
GSM358401	C
GSM358402	B
GSM358403	C
GSM358404	B
GSM358405	A
GSM358406	B
GSM358407	A
GSM358408	A
GSM358409	A
GSM358410	C
GSM358411	C
GSM358412	C
GSM358413	C
GSM358414	B
GSM358415	B
GSM358416	A
GSM358417	A
GSM358418	B
GSM358419	A
GSM358420	C
GSM358421	A
GSM358422	B
GSM358423	B
GSM358424	C
GSM358425	B
GSM358426	C
GSM358427	B
GSM358428	B
GSM358429	B
GSM358430	C
GSM358431	A
GSM358432	C
GSM358433	B
GSM358434	B
GSM358435	B
GSM358436	B
GSM358437	B
GSM358438	B
GSM358439	A
GSM358440	A
GSM358441	A
GSM358442	A
GSM358443	C
GSM358444	B
GSM358445	B
GSM358446	A
GSM358447	B
GSM358448	B
GSM358449	C
GSM358450	C
GSM358451	C
GSM358452	C
GSM358453	B
GSM358454	B
GSM358455	C
GSM358456	C

GSM358457	B
GSM358458	C
GSM358459	B
GSM358460	C
GSM358461	C
GSM358462	A
GSM358463	C
GSM358464	B
GSM358465	C
GSM358466	C
GSM358467	A
GSM358468	A
GSM358469	B
GSM358470	B
GSM358471	C
GSM358472	A
GSM358473	C
GSM358474	A
GSM358475	A
GSM358476	A
GSM358477	B
GSM358478	A
GSM358479	A
GSM358480	B
GSM358481	B
GSM358482	C
GSM358483	C
GSM358484	B
GSM358485	B
GSM358486	B
GSM358487	B
GSM358488	A
GSM358489	B
GSM358490	C
GSM358491	A
GSM358492	A
GSM358493	A
GSM358494	A
GSM358495	A
GSM358496	C
GSM358497	B
GSM358498	B
GSM358499	C
GSM358500	B
GSM358501	A
GSM358502	B
GSM358503	B
GSM358504	C
GSM358505	A
GSM358506	C
GSM358507	A
GSM358508	C
GSM358509	C
GSM358510	C
GSM358511	C
GSM358512	C
GSM358513	C
GSM358514	B

GSM358515	C
GSM358516	C
GSM358517	A
GSM358518	B
GSM358519	B
GSM358520	B
GSM358521	B
GSM358522	A
GSM358523	C
GSM358524	C
GSM358525	C
GSM358526	C
GSM358527	B
GSM358528	C
GSM358529	A
GSM358530	C
GSM358531	C
GSM358532	C
GSM358533	A
GSM358534	C
GSM358535	A
GSM358536	A
GSM358537	C
GSM358538	A
GSM358539	C
GSM358540	C
GSM358541	A
GSM358542	C
GSM358543	C
GSM358544	A
GSM358545	C
GSM358546	C
GSM358547	C
GSM358548	B
GSM358549	C
GSM358550	C
GSM358551	B
GSM358552	C
GSM358553	A
GSM358554	B
GSM358555	A
GSM358556	B
GSM358557	C
GSM358558	B
GSM358559	B
GSM358560	A
GSM358561	C
GSM358562	B
GSM358563	C
GSM358564	B
GSM358565	A
GSM358566	C
GSM358567	A
GSM358568	A
GSM358569	B
GSM929508	A
GSM929511	B
GSM929622	A

GSM929546	B
GSM929623	C
GSM929539	A
GSM929614	B
GSM929496	C
GSM929574	C
GSM929590	C
GSM929586	C
GSM929585	C
GSM929612	C
GSM929609	A
GSM929497	C
GSM929593	B
GSM929591	B
GSM929551	B
GSM929587	A
GSM929523	C
GSM929519	C
GSM929524	C
GSM929525	B
GSM929599	A
GSM929617	B
GSM929576	B
GSM929572	B
GSM929499	A
GSM929504	A
GSM929610	A
GSM929498	B
GSM929618	B
GSM929575	A
GSM929577	C
GSM929502	B
GSM929503	A
GSM929500	B
GSM929494	C
GSM929495	C
GSM929501	A
GSM929505	C
GSM929506	B
GSM929507	A
GSM929509	C
GSM929510	C
GSM929512	A
GSM929513	A
GSM929514	C
GSM929515	C
GSM929516	C
GSM929517	B
GSM929518	C
GSM929520	B
GSM929521	C
GSM929522	B
GSM929526	C
GSM929527	B
GSM929528	B
GSM929529	C
GSM929530	A
GSM929531	B

GSM929532	C
GSM929533	C
GSM929534	C
GSM929535	C
GSM929536	C
GSM929537	C
GSM929538	B
GSM929540	A
GSM929541	B
GSM929542	B
GSM929543	B
GSM929544	B
GSM929545	A
GSM929547	C
GSM929548	A
GSM929549	A
GSM929550	C
GSM929552	A
GSM929553	A
GSM929554	C
GSM929555	A
GSM929556	A
GSM929557	C
GSM929558	B
GSM929559	B
GSM929560	B
GSM929561	B
GSM929562	A
GSM929563	B
GSM929564	C
GSM929565	C
GSM929566	B
GSM929567	B
GSM929568	B
GSM929569	B
GSM929570	C
GSM929571	C
GSM929573	C
GSM929578	B
GSM929579	C
GSM929580	B
GSM929581	C
GSM929582	A
GSM929583	C
GSM929584	C
GSM929588	A
GSM929589	C
GSM929592	B
GSM929594	A
GSM929595	C
GSM929596	C
GSM929597	C
GSM929598	C
GSM929600	A
GSM929601	C
GSM929602	B
GSM929603	B
GSM929604	B

GSM929605	B
GSM929606	A
GSM929607	C
GSM929608	A
GSM929611	A
GSM929613	C
GSM929615	B
GSM929616	C
GSM929619	A
GSM929620	A
GSM929621	B
GSM971957	A
GSM971958	A
GSM971959	B
GSM971960	C
GSM971961	C
GSM971962	A
GSM971963	A
GSM971964	B
GSM971965	A
GSM971966	C
GSM971968	A
GSM971969	B
GSM971970	B
GSM971971	B
GSM971972	C
GSM971973	B
GSM971974	B
GSM971975	B
GSM971976	C
GSM971977	A
GSM971978	B
GSM971979	B
GSM971980	B
GSM971981	B
GSM971982	B
GSM971983	B
GSM971984	B
GSM971985	C
GSM971986	B
GSM971987	A
GSM971988	B
GSM971989	B
GSM971990	B
GSM971991	C
GSM971992	B
GSM971993	B
GSM971994	A
GSM971995	B
GSM971996	A
GSM971997	B
GSM971998	C
GSM971999	A
GSM972000	A
GSM972001	C
GSM972002	B
GSM972003	B
GSM972004	C

GSM972005	B
GSM972006	C
GSM972007	A
GSM972008	A
GSM972009	B
GSM972010	A
GSM972011	A
GSM972012	B
GSM972013	A
GSM972014	C
GSM972015	B
GSM972016	B
GSM972017	A
GSM972018	A
GSM972019	A
GSM972020	C
GSM972021	C
GSM972022	A
GSM972023	A
GSM972024	A
GSM972025	C
GSM972026	A
GSM972027	A
GSM972028	C
GSM972029	A
GSM972030	A
GSM972031	C
GSM972032	A
GSM972033	C
GSM972034	C
GSM972035	C
GSM972036	C
GSM972037	B
GSM972038	B
GSM972039	A
GSM972040	A
GSM972041	A
GSM972042	B
GSM972043	C
GSM972044	B
GSM972045	B
GSM972046	B
GSM972047	B
GSM972048	B
GSM972049	B
GSM972050	C
GSM972051	A
GSM972052	A
GSM972053	C
GSM972054	A
GSM972055	B
GSM972056	B
GSM972057	A
GSM972058	B
GSM972059	A
GSM972060	A
GSM972061	B
GSM972062	A

GSM972063	A
GSM972064	B
GSM972065	C
GSM972066	B
GSM972067	A
GSM972068	B
GSM972069	A
GSM972070	C
GSM972071	C
GSM972072	B
GSM972073	A
GSM972074	C
GSM972075	C
GSM972076	A
GSM972077	B
GSM972078	B
GSM972079	B
GSM972080	B
GSM972081	B
GSM972082	A
GSM972083	C
GSM972084	A
GSM972085	C
GSM972086	A
GSM972087	C
GSM972088	A
GSM972089	A
GSM972090	C
GSM972091	C
GSM972092	A
GSM972093	A
GSM972094	C
GSM972095	A
GSM972096	B
GSM972097	B
GSM972098	C
GSM972099	C
GSM972100	B
GSM972101	C
GSM972102	C
GSM972103	B
GSM972104	A
GSM972105	A
GSM972106	C
GSM972107	C
GSM972108	C
GSM972109	B
GSM972110	C
GSM972111	C
GSM972112	B
GSM972113	A
GSM972114	B
GSM972115	C
GSM972116	A
GSM972117	B
GSM972118	B
GSM972119	A
GSM972120	C

GSM972121	B
GSM972122	C
GSM972123	C
GSM972124	C
GSM972125	A
GSM972126	C
GSM972127	A
GSM972128	B
GSM972129	C
GSM972130	C
GSM972131	A
GSM972132	A
GSM972133	A
GSM972134	A
GSM972135	C
GSM972136	C
GSM972137	C
GSM972138	C
GSM972139	C
GSM972140	A
GSM972141	C
GSM972142	C
GSM972143	A
GSM972144	C
GSM972145	C
GSM972146	A
GSM972147	A
GSM972148	A
GSM972149	C
GSM972150	C
GSM972151	C
GSM972152	C
GSM972153	C
GSM972154	B
GSM972155	B
GSM972156	C
GSM972157	A
GSM972158	C
GSM972159	B
GSM972160	C
GSM972161	C
GSM972162	A
GSM972163	C
GSM972164	C
GSM972165	B
GSM972166	A
GSM972167	C
GSM972168	B
GSM972169	C
GSM972170	A
GSM972171	A
GSM972172	B
GSM972173	C
GSM972174	C
GSM972175	B
GSM972176	A
GSM972177	C
GSM972178	C

GSM972179	A
GSM972180	C
GSM972181	B
GSM972182	B
GSM972183	B
GSM972184	B
GSM972185	C
GSM972186	A
GSM972187	C
GSM972188	B
GSM972189	B
GSM972190	C
GSM972191	A
GSM972192	B
GSM972193	C
GSM972194	B
GSM972195	B
GSM972196	B
GSM972197	B
GSM972198	B
GSM972199	C
GSM972200	C
GSM972201	A
GSM972202	C
GSM972203	C
GSM972204	A
GSM972205	B
GSM972206	B
GSM972207	A
GSM972208	B
GSM972209	B
GSM972210	A
GSM972211	A
GSM972212	B
GSM972213	C
GSM972214	B
GSM972215	C
GSM972216	C
GSM972217	A
GSM972218	B
GSM972219	A
GSM972220	B
GSM972221	B
GSM972222	B
GSM972223	B
GSM972224	B
GSM972225	C
GSM972226	B
GSM972227	B
GSM972228	B
GSM972229	B
GSM972230	C
GSM972231	A
GSM972232	A
GSM972233	C
GSM972234	A
GSM972235	C
GSM972236	C

GSM972237	C
GSM972238	C
GSM972239	A
GSM972240	A
GSM972241	C
GSM972242	B
GSM972243	C
GSM972244	C
GSM972245	B
GSM972246	C
GSM972247	B
GSM972248	B
GSM972249	C
GSM972250	C
GSM972251	C
GSM972252	A
GSM972253	C
GSM972254	A
GSM972255	A
GSM972256	C
GSM972257	C
GSM972258	C
GSM972259	C
GSM972260	C
GSM972261	C
GSM972262	B
GSM972263	B
GSM972264	C
GSM972265	B
GSM972266	C
GSM972267	C
GSM972268	C
GSM972269	C
GSM972270	B
GSM972271	A
GSM972272	C
GSM972273	B
GSM972274	A
GSM972275	B
GSM972276	B
GSM972277	B
GSM972278	B
GSM972279	C
GSM972280	B
GSM972281	C
GSM972282	B
GSM972283	B
GSM972284	C
GSM972285	A
GSM972286	B
GSM972287	B
GSM972288	C
GSM972289	C
GSM972290	C
GSM972291	B
GSM972292	B
GSM972293	B
GSM972294	C

GSM972295	C
GSM972296	C
GSM972297	B
GSM972298	B
GSM972299	C
GSM972300	B
GSM972301	C
GSM972302	C
GSM972303	B
GSM972304	B
GSM972305	B
GSM972306	C
GSM972307	C
GSM972308	C
GSM972309	C
GSM972310	B
GSM972311	C
GSM972312	B
GSM972313	C
GSM972314	C
GSM972315	C
GSM972316	B
GSM972317	B
GSM972318	B
GSM972319	C
GSM972320	C
GSM972321	C
GSM972322	C
GSM972323	C
GSM972324	B
GSM972325	B
GSM972326	B
GSM972327	B
GSM972328	A
GSM972329	C
GSM972330	B
GSM972331	C
GSM972332	C
GSM972333	C
GSM972334	B
GSM972335	C
GSM972336	C
GSM972337	B
GSM972338	C
GSM972339	C
GSM972340	C
GSM972341	C
GSM972342	C
GSM972343	B
GSM972344	B
GSM972345	A
GSM972346	B
GSM972347	C
GSM972348	C
GSM972349	B
GSM972351	A
GSM972352	A
GSM972353	A

GSM972354	A
GSM972355	C
GSM972357	A
GSM972358	C
GSM972359	A
GSM972360	C
GSM972361	B
GSM972362	C
GSM972363	C
GSM972364	C
GSM972365	B
GSM972366	C
GSM972367	A
GSM972368	B
GSM972369	C
GSM972370	C
GSM972371	A
GSM972372	A
GSM972373	A
GSM972374	C
GSM972375	A
GSM972376	A
GSM972377	A
GSM972378	B
GSM972379	C
GSM972380	A
GSM972381	C
GSM972382	A
GSM972383	C
GSM972384	A
GSM972385	A
GSM972386	C
GSM972387	C
GSM972388	C
GSM972389	B
GSM972390	B
GSM972391	B
GSM972392	C
GSM972393	B
GSM972394	A
GSM972395	C
GSM972396	B
GSM972397	B
GSM972398	C
GSM972399	A
GSM972400	C
GSM972401	B
GSM972402	B
GSM972403	B
GSM972404	A
GSM972405	C
GSM972406	A
GSM972407	A
GSM972408	B
GSM972409	C
GSM972410	A
GSM972411	C
GSM972412	B

GSM972413	A
GSM972414	C
GSM972415	C
GSM972416	B
GSM972417	A
GSM972418	A
GSM972419	A
GSM972420	B
GSM972421	A
GSM972422	A
GSM972423	B
GSM972424	B
GSM972425	B
GSM972426	A
GSM972427	A
GSM972428	A
GSM972429	B
GSM972430	B
GSM972431	B
GSM972432	A
GSM972433	A
GSM972434	B
GSM972435	C
GSM972436	B
GSM972437	A
GSM972438	B
GSM972439	A
GSM972440	C
GSM972441	A
GSM972442	C
GSM972443	B
GSM972444	B
GSM972445	A
GSM972447	B
GSM972449	B
GSM972450	C
GSM972451	C
GSM972452	B
GSM972453	B
GSM972454	C
GSM972455	B
GSM972456	C
GSM972457	C
GSM972458	B
GSM972459	B
GSM972460	C
GSM972461	C
GSM972462	C
GSM972464	A
GSM972465	B
GSM972466	C
GSM972467	A
GSM972468	A
GSM972469	B
GSM972470	C
GSM972472	B
GSM972473	B
GSM972474	C

GSM972475	B
GSM972476	A
GSM972477	B
GSM972478	C
GSM972479	B
GSM972480	B
GSM972481	C
GSM972482	A
GSM972483	A
GSM972484	B
GSM972485	B
GSM972486	A
GSM972487	C
GSM972488	B
GSM972489	B
GSM972490	C
GSM972491	C
GSM972492	B
GSM972493	A
GSM972494	B
GSM972495	B
GSM972496	C
GSM972497	C
GSM972498	C
GSM972499	B
GSM972501	B
GSM972502	C
GSM972503	A
GSM972504	C
GSM972505	B
GSM972506	B
GSM972507	C
GSM972508	C
GSM972509	C
GSM972510	C
GSM972511	C
GSM972512	B
GSM972513	C
GSM972515	C
GSM972516	C
GSM972517	C
GSM972518	B
GSM972519	B
GSM972520	B
GSM972521	A
GSM972522	C

Table S5. TCGA gene clusters and genes A

DEGs overlapped	Genes A-C
TET1	genes A
VLDLR	genes A
DIRAS2	genes A
MUM1L1	genes A
RASL11B	genes A
TRAM1L1	genes A
DTNA	genes A
CC2D2A	genes A
TNFAIP8L3	genes A
GHR	genes A
ST6GAL2	genes A
CLSTN2	genes A
SNORD116-28	genes A
IPW	genes A
PAR-SN	genes A
ZNF415	genes A
ZNF134	genes A
ZNF471	genes A
ZSCAN18	genes A
FGF13	genes A
KL	genes A
TXLNB	genes A
DAB2	genes A
TGFBR2	genes A
KLF7	genes A
KIAA0355	genes A
MBD5	genes A
KIDINS220	genes A
BMPR2	genes A
RNF146	genes A
TSPYL4	genes A
NIPSNAP3B	genes A
RCBTB2	genes A
ODZ2	genes A
CGNL1	genes A
GPRASP2	genes A
NFASC	genes A
RAI2	genes A
ZNF167	genes A
ZNF569	genes A
ZNF287	genes A
GBGT1	genes A
MAGEE1	genes A
ZNF853	genes A
SMO	genes A
ZNF423	genes A
HDGFRP3	genes A
LOC285548	genes A
RAB9B	genes A
C11orf63	genes A
SEMA4C	genes A
CRY2	genes A
ZFHX3	genes A
SMTN	genes A
ZNF362	genes A

AKAP6		genes A
RBM20		genes A
	5-Sep	genes A
BRSK1		genes A
PCDHGC3		genes A
CILP2		genes A
KIF7		genes A
TSPYL2		genes A
MECP2		genes A
PCP4L1		genes A
HRNBP3		genes A
C6orf168		genes A
CCDC136		genes A
LOC100128239		genes A
NCRNA00085		genes A
TF		genes A
SLC7A10		genes A
FNDC5		genes A
TERF2IP		genes A
CDH8		genes A
	7-Sep	genes A
TMEM43		genes A
NXPH3		genes A
NPTXR		genes A
SPEG		genes A
AGTR1		genes A
GPRASP1		genes A
LIMS2		genes A
TCF7L1		genes A
MAP6		genes A
PDZD4		genes A
MPPED2		genes A
HSPB6		genes A
OGN		genes A
CDON		genes A
PCYT1B		genes A
DCHS2		genes A
C1orf95		genes A
ADCY5		genes A
ATP1A2		genes A
CASQ2		genes A
SYNM		genes A
MYH11		genes A
LMO3		genes A
HSPB7		genes A
CARTPT		genes A
ASB5		genes A
HAND1		genes A
CHRM2		genes A
NRSN1		genes A
KIF1A		genes A
FMN2		genes A
PGM5P2		genes A
PGM5		genes A
DDIT4L		genes A
DYNC1I1		genes A
WASF3		genes A
SORBS1		genes A

RBMS1	genes A
NRXN2	genes A
LGI4	genes A
PPP1R14A	genes A
HRC	genes A
SLC35F1	genes A
RGS5	genes A
FLJ22536	genes A
RPRM	genes A
TMEM35	genes A
ISLR2	genes A
ABCB4	genes A
ORAI2	genes A
MAPK8IP1	genes A
BCAM	genes A
RND2	genes A
WFIKKN2	genes A
LEFTY2	genes A
PTPRT	genes A
HLF	genes A
LOC643763	genes A
ABL2	genes A
STAT5B	genes A
NNAT	genes A
NRIP2	genes A
AASS	genes A
KCTD7	genes A
ZNF333	genes B
IGFL2	genes B
KLF2	genes B
ACTB	genes B
PLOD1	genes B
TREML3	genes B
DCBLD1	genes B
ANXA5	genes B
GUCA1A	genes B
IKBIP	genes B
TMEM45A	genes B
COL6A6	genes B
ALOX15B	genes B
ADAM8	genes B
VMO1	genes B
NFATC1	genes B
TGFB1	genes B
HAPLN3	genes B
SPHK1	genes B
AMZ1	genes B
CLEC4E	genes B
CCL18	genes B
C19orf59	genes B
KCNJ15	genes B
BCL2A1	genes B
TREM1	genes B
AQP9	genes B
CAMK1G	genes B
CHST11	genes B
FCGR2B	genes B
FCGR2C	genes B

CD14	genes B
FCGR2A	genes B
PILRA	genes B
C5AR1	genes B
LILRA6	genes B
LILRB3	genes B
HK3	genes B
NCF2	genes B
ITGAM	genes B
FGR	genes B
ITGAX	genes B
ALOX5AP	genes B
SIGLEC5	genes B
LILRA2	genes B
FPR1	genes B
CSF3R	genes B
MCHR1	genes B
MMP9	genes B
SPP1	genes B
MARCO	genes B
SLC11A1	genes B
CLEC5A	genes B
RGS16	genes B
SOCS3	genes B
OSM	genes B
CHSY1	genes B
STC1	genes B
SLC2A3	genes B
EMP3	genes B
LGALS1	genes B
NNMT	genes B
TSPAN4	genes B
PLEKHO1	genes B
GYPC	genes B
A4GALT	genes B
PTGIR	genes B
ALPK2	genes B
BCAT1	genes B
ADAM12	genes B
TNFAIP6	genes B
LOX	genes B
PRRX1	genes B
KCND2	genes B
MDGA1	genes B
HS3ST3A1	genes B
PDPN	genes B
PRR16	genes B
WISP1	genes B
GPR176	genes B
XIRP1	genes B
PLAU	genes B
NID2	genes B
ADAMTS2	genes B
ZNF469	genes B
COL5A3	genes B
ADAMTS4	genes B
SLC2A6	genes B
ZNF467	genes B

FAM167B	genes B
ESAM	genes B
SH2D3C	genes B
STARD8	genes B
CD93	genes B
CLEC1A	genes B
PECAM1	genes B
SCARF1	genes B
SEMA6B	genes B
ENG	genes B
DEGS1	genes B
TFPI	genes B
AIF1L	genes B
HOXC4	genes B
HOXC9	genes B
ANXA8L2	genes B
UNC5A	genes B
IRF1	genes B
NFIL3	genes B
FGFBP2	genes B
HRASLS5	genes B
VIT	genes B
PDE4DIP	genes B
FGF11	genes B
VAMP2	genes B
SMG6	genes B
WDR81	genes B
ABHD4	genes B
KLHL22	genes B
ADRB2	genes B
ARHGDIB	genes B
RNASE6	genes B
PDE6B	genes B
MAL	genes B
LTA	genes B
LTB	genes B
NAPSB	genes B
AMICA1	genes B
CLEC10A	genes B
RAB33A	genes B
ITM2A	genes B
CLIC2	genes B
GIMAP1	genes B
GIMAP5	genes B
GIMAP7	genes B
ARHGAP15	genes B
GGTA1	genes B
SLC9A9	genes B
BIN2	genes B
SIGLEC8	genes B
GZMK	genes B
PLA2G2D	genes B
TNFSF14	genes B
SPOCK2	genes B
ZNF831	genes B
GVIN1	genes B
KIAA0748	genes B
PPP1R16B	genes B

PRKCB	genes B
IL16	genes B
LY9	genes B
P2RY8	genes B
ABCD2	genes B
LOC100233209	genes B
TRAF3IP3	genes B
ARHGAP9	genes B
RASAL3	genes B
IL21R	genes B
CCR5	genes B
WDFY4	genes B
SELL	genes B
CR1	genes B
HLA-DQA1	genes B
FGD2	genes B
EVI2B	genes B
CD180	genes B
FYB	genes B
APBB1IP	genes B
DOCK10	genes B
ARHGAP25	genes B
KLHL6	genes B
RCSD1	genes B
C17orf87	genes B
STX11	genes B
IL10RA	genes B
NCKAP1L	genes B
SLA	genes B
PIK3R5	genes B
CD53	genes B
BTK	genes B
LILRB1	genes B
SIGLEC10	genes B
FAM78A	genes B
MYO1G	genes B
CYTH4	genes B
FMNL1	genes B
MYO1F	genes B
LCP1	genes B
WAS	genes B
HCLS1	genes B
ARHGAP30	genes B
DOCK2	genes B
ITGAL	genes B
SASH3	genes B
PTPRC	genes B
IKZF1	genes B
NCF1	genes B
LSP1	genes B
CD37	genes B
RHOH	genes B
TAGAP	genes B
TNFSF8	genes B
NCF1C	genes B
NCF1B	genes B
KLRG1	genes B
LOC254559	genes B

PTCRA	genes B
C17orf60	genes B
LILRA4	genes B
HLA-DQA2	genes B
HLA-DQB2	genes B
HLA-DPB2	genes B
CD52	genes B
CD48	genes B
GNGT2	genes B
HCST	genes B
GMFG	genes B
TNFAIP8L2	genes B
LST1	genes B
AIF1	genes B
PSTPIP1	genes B
LIMD2	genes B
FCRL6	genes B
LOC400759	genes B
IL18BP	genes B
ATP6V0D2	genes B
LIPA	genes B
SLC2A5	genes B
GM2A	genes B
HS3ST2	genes B
TREM2	genes B
APOE	genes B
APOC1	genes B
OSCAR	genes B
SLC1A3	genes B
OLR1	genes B
MSR1	genes B
GPNMB	genes B
CTSL1	genes B
C1orf162	genes B
FCGR1C	genes B
FCGR3A	genes B
FCGR1B	genes B
FCGR1A	genes B
PLD3	genes B
PSAP	genes B
KCNE1	genes B
CD68	genes B
CTSB	genes B
MRO	genes B
CPVL	genes B
TNNI2	genes B
BAI2	genes B
PLEKHF1	genes B
FLT3LG	genes B
NAGK	genes B
PPM1M	genes B
CACNA1A	genes B
MX2	genes B
RSAD2	genes B
DDX58	genes B
DRAM1	genes B
PPM1K	genes B
HOXB2	genes B

ERMN	genes B
RNF122	genes B
PDE10A	genes B
CD109	genes B
FBLN7	genes B
CALHM2	genes B
LOC400043	genes B
PDGFRL	genes B
DZIP1L	genes B
ARHGAP24	genes B
ARHGAP20	genes B
PLXDC2	genes B
PALLD	genes B
FYN	genes B
PLXND1	genes B
CHST15	genes B
PEAR1	genes B
C6orf204	genes B
DOCK8	genes B
TLR6	genes B
FZD2	genes B
ARHGAP22	genes B
MOBKL2A	genes B
S1PR2	genes B
RIMBP3	genes B
MAMLD1	genes B
EID3	genes B
NCRNA00181	genes B
NTRK1	genes B
CCDC88A	genes B
OPRL1	genes B
HSD17B14	genes B
HVCN1	genes B
LYL1	genes B
SPATC1	genes B
PLCB2	genes B
STAT2	genes B
NLRP1	genes B
C9orf139	genes B
HSPA7	genes B
CORO1A	genes B
SYTL3	genes B
CD72	genes B
GPSM3	genes B
MFNG	genes B
DOK3	genes B
PIK3CD	genes B
POU2F2	genes B
FERMT3	genes B
GPR132	genes B
SUCNR1	genes B
PLA2G7	genes B
CLEC4A	genes B
EVI2A	genes B
SRGN	genes B
SAMSN1	genes B
PDCD1LG2	genes B
MNDA	genes B

LCP2		genes B
PLEK		genes B
GPR65		genes B
C10orf128		genes B
KCNK13		genes B
GPR34		genes B
MS4A6A		genes B
MS4A4A		genes B
MS4A7		genes B
TLR8		genes B
CYBB		genes B
CD84		genes B
TFEC		genes B
CLEC7A		genes B
IGSF6		genes B
TLR1		genes B
PLXNC1		genes B
TLR7		genes B
MPEG1		genes B
CMKLR1		genes B
CD4		genes B
CSF1R		genes B
GPR141		genes B
	1-Mar	genes B
EMILIN2		genes B
PTAFR		genes B
CD300C		genes B
NLRP3		genes B
CASS4		genes B
FCN1		genes B
RASGRP4		genes B
HRH2		genes B
C1orf38		genes B
EMR2		genes B
MS4A14		genes B
SDS		genes B
CD300LB		genes B
DPEP2		genes B
LY86		genes B
PIK3R6		genes B
PRAM1		genes B
PARVG		genes B
EVL		genes B
ESR1		genes B
CECR1		genes B
SLC37A2		genes B
ZNF804A		genes B
SLC31A2		genes B
PDE6G		genes B
CLEC12A		genes B
KMO		genes B
RAB42		genes B
ADORA3		genes B
P2RX7		genes B
FAM20A		genes B
PLEKHO2		genes B
MRC1		genes B
VSIG4		genes B

CD33	genes B
CCR1	genes B
SIRPB2	genes B
LILRB2	genes B
SIGLEC9	genes B
LRRC25	genes B
SIGLEC7	genes B
CD163	genes B
C3AR1	genes B
FPR3	genes B
SIGLEC1	genes B
NLRC4	genes B
TNFSF13B	genes B
HLA-DPB1	genes B
HLA-DOA	genes B
SLC15A3	genes B
NFAM1	genes B
ADAP2	genes B
CD300A	genes B
CD300LF	genes B
HCK	genes B
SLAMF8	genes B
LAPTM5	genes B
ITGB2	genes B
SPI1	genes B
LAIR1	genes B
TYROBP	genes B
FCER1G	genes B
LILRB4	genes B
CD86	genes B
HAVCR2	genes B
TRPV2	genes B
ABI3	genes B
DOK2	genes B
C1QA	genes B
C1QB	genes B
C1QC	genes B
MATK	genes B
ACP5	genes B
DNAJC5B	genes B
STEAP4	genes B
LOC339524	genes B
CD36	genes B
SSTR2	genes B
CYTL1	genes B
NLRP12	genes B
STAC	genes B
CCL8	genes B
CCL7	genes B
DSE	genes B
RNASE2	genes B
GLIPR2	genes B
VAMP5	genes B
C1orf54	genes B
CLEC2B	genes B
LY96	genes B
NCRNA00189	genes B
IL4I1	genes B

ICAM1	genes B
JAK3	genes B
TNFRSF4	genes B
ADORA2A	genes B
TNFRSF8	genes B
SIRPB1	genes B
P2RY6	genes B
LILRA5	genes B
GPR84	genes B
ZNF385A	genes B
RGS19	genes B
MCTP1	genes B
LAT2	genes B
KIAA1949	genes B
ARL4C	genes B
RASGRF2	genes B
SPON1	genes B
ANXA1	genes B
RGS2	genes B
PNMA1	genes B
ATP8B3	genes B
ADCY7	genes B
PMP22	genes B
C10orf10	genes B
LOXL1	genes B
PRKCDBP	genes B
ABLIM3	genes B
MAPK11	genes B
KIFC3	genes B
GNAI2	genes B
MAP7D1	genes B
ANKRD34A	genes B
BCL6	genes B
SLC43A3	genes B
ITPRIPL2	genes B
TOX2	genes B
NFIC	genes B
C14orf49	genes B
CCL23	genes B
MFSD7	genes B
MCOLN1	genes B
ACP2	genes B
C10orf54	genes B
MGAT1	genes B
KLF9	genes B
AGPAT4	genes B
GLIS3	genes B
MFSD1	genes B
RNF144B	genes B
GPR137B	genes B
TCL1A	genes B
CLEC17A	genes B
FAIM3	genes B
TNFRSF13B	genes B
FCRL3	genes B
CD22	genes B
FCRLA	genes B
BLK	genes B

CXCR5	genes B
MS4A1	genes B
CD79B	genes B
TLR10	genes B
AFF2	genes B
TREML1	genes B
PER1	genes B
NR4A3	genes B
ITPRIP	genes B
SERPINE1	genes B
EGR3	genes B
SGK1	genes B
TNFAIP3	genes B
DUSP1	genes B
GPR183	genes B
RGS1	genes B
B4GALT1	genes B
KCTD11	genes B
EMP1	genes B
SEMA7A	genes B
PLEKHG1	genes B
KCTD12	genes B
PDE5A	genes B
ARSB	genes B
SLC16A7	genes B
TRAM2	genes B
ST5	genes B
AHNAK	genes B
AHDC1	genes B
GSN	genes B
C17orf107	genes B
SIDT2	genes B
ASAP3	genes B
RNF24	genes B
PPFIBP1	genes B
HTR1B	genes B
IFNAR2	genes B
TCN2	genes B
SLCO2B1	genes B
IL1RL1	genes B
CTSG	genes B
SIGLEC6	genes B
SIGLECP3	genes B
HDC	genes B
TPSAB1	genes B
TPSB2	genes B
CPA3	genes B
MS4A2	genes B
C1orf150	genes B
C1orf186	genes B
BEND4	genes B
CAMK4	genes B
CLEC9A	genes B
FLT3	genes B
KCND3	genes B
CCDC69	genes B
FAM65B	genes B
FAM55C	genes B

PIP4K2A	genes B
CCR7	genes B
CCL22	genes B
TNFRSF9	genes B
FOXP3	genes B
CCR8	genes B
SPN	genes B
CD28	genes B
CCR4	genes B
PRKAR2B	genes B
S100B	genes B
CCDC141	genes B
EPB41L3	genes B
SASH1	genes B
TIAM1	genes B
ROR1	genes B
STK10	genes B
TMEM140	genes B
RUNDC2A	genes B
LYST	genes B
KIAA0247	genes B
CYLD	genes B
GIT2	genes B
LOC257358	genes B
EMR4P	genes B
EMR1	genes B
CEBPE	genes B
CEACAM4	genes B
CFP	genes B
CLEC4G	genes B
CCL13	genes B
LILRB5	genes B
FOLR2	genes B
PDE4B	genes B
IL10	genes B
SLC24A4	genes B
CXCR4	genes B
SELPLG	genes B
RASSF5	genes B
CHI3L2	genes B
PLCG2	genes B
C16orf54	genes B
LRMP	genes B
STOM	genes B
ITGA1	genes B
AVPR1A	genes B
TRPC6	genes B
CAV1	genes B
STK32B	genes B
COL6A1	genes B
ITGA5	genes B
IRAK3	genes B
HHEX	genes B
CITED2	genes B
LHFPL2	genes B
TCP11L1	genes B
SWAP70	genes B
RAB8B	genes B

SNRK	genes B
CHST2	genes B
KCNN3	genes B
ZNF366	genes B
SFMBT2	genes B
STAB1	genes B
P2RY13	genes B
RGS18	genes B
ST8SIA4	genes B
ITGA4	genes B
CYSLTR1	genes B
CSF2RB	genes B
PIK3CG	genes B
ATP8B4	genes B
GAB3	genes B
CXorf21	genes B
CCR2	genes B
FGL2	genes B
CYSLTR2	genes B
GJD3	genes B
TMEM26	genes B
DOCK4	genes B
RASGRP3	genes B
NR3C1	genes B
GNG2	genes B
VCAM1	genes B
FAM49A	genes B
GIMAP4	genes B
GIMAP6	genes B
FLI1	genes B
GIMAP8	genes B
RASSF2	genes B
ARHGEF6	genes B
MYO5A	genes B
WIPF1	genes B
GPR77	genes B
CLEC4D	genes B
FCAR	genes B
CD300E	genes B
GPR97	genes B
GLT1D1	genes B
EMR3	genes B
LILRA1	genes B
FAM196B	genes B
PAPPA	genes B
APBA2	genes B
C3	genes B
BASP1	genes B
GPR68	genes B
MSC	genes B
SDK1	genes B
ETS1	genes B
ARHGAP31	genes B
SLFN11	genes B
HSD11B1	genes B
CCL2	genes B
CSF1	genes B
NRP1	genes B

DPYD	genes B
LRRC8C	genes B
MSN	genes B
IL1R1	genes B
ATP8B2	genes B
ASAM	genes B
OSMR	genes B
COLEC12	genes B
VIM	genes B
RAB31	genes B
CFH	genes B
GNB4	genes B
ZEB2	genes B
MAFB	genes B
MAF	genes B
FUT11	genes B
LIMS1	genes B
SH2B3	genes B
PLBD2	genes B
MTIF2	genes C
OLA1	genes C
HSPE1	genes C
CCDC58	genes C
GPR35	genes C
DUS1L	genes C
TBRG4	genes C
LY6G6D	genes C
EBPL	genes C
KIF11	genes C
HNRNPF	genes C
PBK	genes C
RRM2	genes C
H2AFZ	genes C
CENPM	genes C
ORC1L	genes C
CDC20	genes C
PPA2	genes C
NDUFA9	genes C
PSMA5	genes C
PGAM5	genes C
SORD	genes C
CCNO	genes C
TMC5	genes C
CASP5	genes C
F2RL1	genes C

Table S6. Fersig score of different databases

samples	TCGA PC1 of genes A	TCGA PC1 of genes B
TCGA.3L.AA1B.01	6.106741023	0.533374706
TCGA.4N.A93T.01	-4.003593467	-5.882265235
TCGA.4T.AA8H.01	-5.088839373	-11.04406171
TCGA.5M.AATE.01	-0.88023619	-6.21739114
TCGA.A6.5656.01	2.252418895	-4.092000021
TCGA.A6.5659.01	4.681027345	-3.63126201
TCGA.A6.5660.01	-0.921547602	-1.05371654
TCGA.A6.5662.01	5.032663199	0.021882242
TCGA.A6.5666.01	-4.44594474	-7.244400768
TCGA.A6.5667.01	7.104003138	-1.800366602
TCGA.A6.6140.01	-2.860856999	-6.459034155
TCGA.A6.6648.01	-3.39725703	-7.94652783
TCGA.A6.6652.01	0.0194015	-3.948648302
TCGA.A6.A56B.01	3.554120817	0.09027404
TCGA.AA.3496.01	0.213615277	4.261033879
TCGA.AA.3509.01	-0.973032235	-1.452295053
TCGA.AA.3511.01	0.114176832	-0.977031466
TCGA.AA.3660.01	-0.443400259	-0.598829175
TCGA.AA.3662.01	0.666778463	3.491179044
TCGA.AA.3675.01	-3.948033571	-2.768542773
TCGA.AA.A01X.01	7.271196211	0.046633991
TCGA.AD.6888.01	-3.763744025	-6.71520917
TCGA.AD.6890.01	-1.792776229	-0.928164178
TCGA.AD.6965.01	-1.069447019	-1.858086372
TCGA.AD.A5EK.01	0.611075789	-4.633043268
TCGA.AF.3911.01	2.906688555	-0.369744072
TCGA.AF.4110.01	4.758278296	3.568795192
TCGA.AF.6136.01	-2.00597497	-6.311284676
TCGA.AF.6672.01	-0.166197322	-3.61045943
TCGA.AF.A56L.01	1.318026665	-0.239484987
TCGA.AF.A56N.01	0.589778126	-1.364676293
TCGA.AG.3732.01	2.683045617	-0.683470644
TCGA.AH.6544.01	-4.574626663	-9.11945896
TCGA.AH.6549.01	0.40364921	-0.808589357
TCGA.AH.6643.01	2.276623768	1.35370854
TCGA.AH.6897.01	-6.719275678	-10.45582888
TCGA.AH.6903.01	-0.251306905	-8.59831181
TCGA.AM.5820.01	4.247883091	-1.539574333
TCGA.AY.A54L.01	-4.877384839	-10.03260249
TCGA.AY.A69D.01	-2.370839207	-4.597192035
TCGA.AY.A71X.01	-4.744542655	-5.89066046
TCGA.AY.A8YK.01	-1.002426935	-5.06904886
TCGA.AZ.4684.01	-2.391119509	1.413205018
TCGA.CA.5255.01	-3.626841184	-2.596260521
TCGA.CA.5256.01	-5.066904353	-4.925858381
TCGA.CA.5797.01	3.161237437	-1.618276822
TCGA.CA.6715.01	-0.301610154	-6.578457066
TCGA.CA.6716.01	5.73933306	-3.732349026
TCGA.CI.6619.01	0.859733284	1.841168667
TCGA.CI.6620.01	0.171798414	-2.367265541
TCGA.CI.6622.01	-3.878679451	-6.121340271
TCGA.CK.5912.01	-0.463601179	-6.311605643
TCGA.CK.5915.01	-4.43651113	-10.87521785
TCGA.CL.4957.01	-2.035585154	-4.590217721
TCGA.CL.5917.01	2.796556509	-3.022237556

TCGA.CL.5918.01	-4.081078176	-6.377236846
TCGA.CM.4747.01	2.954262705	-0.487979847
TCGA.CM.5344.01	3.04988584	1.636199627
TCGA.CM.5864.01	-2.722207575	-4.824768607
TCGA.CM.5868.01	0.892339004	0.727116234
TCGA.CM.6161.01	0.214456657	-0.761702501
TCGA.CM.6163.01	0.067228065	0.128579629
TCGA.CM.6164.01	5.276615509	-0.875323542
TCGA.CM.6165.01	3.684613692	-0.237323149
TCGA.CM.6166.01	2.980372817	-5.609978774
TCGA.CM.6170.01	-0.248324353	-0.034327402
TCGA.CM.6172.01	0.032855894	-2.719250779
TCGA.CM.6676.01	4.663828882	-2.149020374
TCGA.CM.6678.01	2.911152199	-2.545613689
TCGA.D5.5537.01	-0.362006647	-0.657571902
TCGA.D5.5540.01	-5.826025153	-5.612780112
TCGA.D5.5541.01	2.562308283	2.800192101
TCGA.D5.6532.01	-3.434206796	-7.940648002
TCGA.D5.6533.01	-2.532055155	-2.948190328
TCGA.D5.6538.01	2.028129148	-7.441430489
TCGA.D5.6922.01	8.708201828	1.065629384
TCGA.D5.6923.01	0.363412518	2.090976781
TCGA.D5.6924.01	5.235267931	4.105890878
TCGA.D5.6926.01	5.042039418	4.451609014
TCGA.D5.6929.01	0.048752229	3.355905052
TCGA.D5.6932.01	1.535238798	2.035321213
TCGA.DC.4745.01	-0.606299397	-3.864471382
TCGA.DC.4749.01	-3.69424284	-6.964755107
TCGA.DC.5869.01	-2.834777495	-3.725632938
TCGA.DC.6154.01	0.798217709	0.067032734
TCGA.DC.6155.01	-4.440253025	0.496270604
TCGA.DC.6157.01	0.431743062	-4.359996769
TCGA.DC.6681.01	9.099296742	-1.033910713
TCGA.DC.6682.01	0.188388727	-5.855982917
TCGA.DC.6683.01	1.969410331	-2.97140644
TCGA.DM.A1D0.01	-3.586059832	-6.81404498
TCGA.DM.A1D4.01	-6.458430046	-7.312130059
TCGA.DM.A1D6.01	-5.485881254	-9.13036791
TCGA.DM.A1D7.01	-2.432946468	-4.60018109
TCGA.DM.A1D8.01	-1.757671388	-5.946725555
TCGA.DM.A1D9.01	-2.893615544	-5.206052875
TCGA.DM.A1HA.01	-2.972324905	-0.73343662
TCGA.DM.A282.01	0.2206868	-5.57492534
TCGA.DM.A288.01	-6.322859464	-3.878999736
TCGA.DM.A28A.01	-0.383901767	0.724523418
TCGA.DM.A28C.01	-2.352105986	-8.787414
TCGA.DM.A28E.01	-6.043655429	-11.94813216
TCGA.DM.A28F.01	-4.391994742	-5.628105274
TCGA.DM.A28G.01	0.220658788	-5.509678773
TCGA.DM.A28H.01	-4.616375594	-5.396141461
TCGA.DY.A0XA.01	-1.00008412	-4.637225877
TCGA.DY.A1DD.01	10.56051893	-0.360316804
TCGA.DY.A1DF.01	8.627181408	1.700845055
TCGA.DY.A1H8.01	-2.06709282	-5.190427466
TCGA.EF.5830.01	-2.155012108	-4.779546277
TCGA.EF.5831.01	-1.212166209	-1.044365571
TCGA.EI.6508.01	-0.320985231	-4.85197741
TCGA.EI.6509.01	1.737121259	1.331630087

TCGA.EI.6512.01	-3.580759258	-2.418205052
TCGA.EI.6513.01	-0.697201051	-1.361352201
TCGA.EI.6514.01	8.593928882	-1.641429829
TCGA.EI.6881.01	-5.075063758	-4.031314064
TCGA.EI.6883.01	2.467208903	-0.807828439
TCGA.EI.7002.01	5.738719076	0.218801304
TCGA.F4.6463.01	8.137410277	2.443547921
TCGA.F4.6806.01	-0.824437517	-1.04199009
TCGA.F4.6808.01	-2.104467868	-6.336782614
TCGA.F4.6854.01	5.634880681	-0.521982106
TCGA.F5.6571.01	5.720454978	2.935925658
TCGA.F5.6814.01	2.299032732	1.011692776
TCGA.F5.6861.01	1.754232638	-2.599406638
TCGA.F5.6863.01	12.86622397	-0.982149743
TCGA.F5.6864.01	10.13357699	5.761267028
TCGA.G4.6303.01	4.539538516	-1.093410042
TCGA.G4.6304.01	-5.119533669	-2.7096416
TCGA.G4.6307.01	-1.459304506	-6.435366623
TCGA.G4.6310.01	14.21934446	-1.901579358
TCGA.G4.6315.01	-4.065871246	-8.83754768
TCGA.G4.6317.01	-4.509837738	-11.32063121
TCGA.G5.6235.01	-4.958032098	-9.606530551
TCGA.G5.6641.01	-2.057868528	-10.20465004
TCGA.NH.A50T.01	-5.303156382	-8.694073264
TCGA.NH.A6GA.01	1.144681783	0.680672928
TCGA.NH.A6GC.01	2.566077187	0.450244421
TCGA.NH.A8F7.01	2.666228303	-8.00667852
TCGA.NH.A8F8.01	6.140757921	3.24751845
TCGA.QG.A5YV.01	-3.03732007	-5.99663526
TCGA.QG.A5YX.01	-7.006493887	-9.201690573
TCGA.QG.A5Z1.01	7.582116955	-0.447474465
TCGA.QL.A97D.01	-4.972592134	-5.073920585
TCGA.RU.A8FL.01	-6.758484568	-11.10095491
TCGA.SS.A7HO.01	-0.761891001	-5.18874806
TCGA.T9.A92H.01	-5.010459322	-7.83416194
TCGA.A6.2686.01	1.040603864	9.441053502
TCGA.A6.5661.01	-9.506975596	-4.060668791
TCGA.A6.5665.01	-9.464842106	-1.14534931
TCGA.A6.6137.01	-1.262271552	-1.649672434
TCGA.A6.6138.01	0.282595087	4.278763048
TCGA.A6.6141.01	-0.742653242	0.886346127
TCGA.A6.6649.01	5.611932741	3.355123838
TCGA.A6.6650.01	-4.967262279	-8.37001939
TCGA.A6.6653.01	-7.279819502	-1.200663552
TCGA.A6.6780.01	-7.841642845	6.820240481
TCGA.AA.3492.01	-6.349978263	1.098694639
TCGA.AA.3495.01	-3.928745673	-1.859546903
TCGA.AA.3502.01	-5.763001709	-3.017621948
TCGA.AA.3506.01	-0.157524188	1.786740185
TCGA.AA.3526.01	-4.244569313	0.091774751
TCGA.AA.3655.01	-3.187986006	-0.797406264
TCGA.AA.3663.01	-9.331447565	-1.57161305
TCGA.AA.3685.01	-4.715360885	1.470293906
TCGA.AA.3697.01	-3.20055838	-2.576293904
TCGA.AA.3712.01	-3.600872758	-1.369496867
TCGA.AA.3713.01	-4.155415645	2.845217208
TCGA.AA.A01P.01	3.399271076	10.0743339
TCGA.AA.A01Z.01	-7.432216089	-8.723489042

TCGA.AA.A02K.01	-5.969638268	0.590454206
TCGA.AA.A02Y.01	-9.487358253	-7.32374664
TCGA.AD.5900.01	2.941652946	4.087608588
TCGA.AD.6548.01	-0.213745037	3.62393615
TCGA.AD.6889.01	-6.301792772	-0.709129668
TCGA.AD.6895.01	-0.19822768	4.295770657
TCGA.AD.6963.01	-4.922518638	-3.031125666
TCGA.AD.A5EJ.01	-6.101579303	0.211032781
TCGA.AF.2693.01	-1.950588029	-1.018697146
TCGA.AF.5654.01	-3.649798745	-7.707200909
TCGA.AF.6655.01	0.339009865	0.521511743
TCGA.AF.A56K.01	1.823302135	3.4829721
TCGA.AG.3591.01	-4.565467914	-3.831494734
TCGA.AG.3592.01	-3.093010902	-2.51191205
TCGA.AG.3725.01	-4.990524	-6.371845207
TCGA.AG.3742.01	-0.909838558	-2.295670456
TCGA.AG.3902.01	-1.926110989	4.190348659
TCGA.AM.5821.01	-8.896727846	5.856545364
TCGA.AU.6004.01	-0.651795748	4.240304509
TCGA.AY.5543.01	-7.600311846	-4.636715956
TCGA.AY.6197.01	-7.025722647	-2.51766548
TCGA.AY.6386.01	-6.572878881	-2.940612291
TCGA.AZ.4313.01	-10.58589182	-4.953636434
TCGA.AZ.4315.01	-8.470607034	-1.357203778
TCGA.AZ.4615.01	-4.800493692	5.780176037
TCGA.AZ.5407.01	-7.520028611	-4.887722486
TCGA.BM.6198.01	0.27684305	4.74567035
TCGA.CA.5254.01	-3.623904278	0.312446355
TCGA.CA.5796.01	-7.546918318	-5.506234967
TCGA.CA.6718.01	1.859502357	7.556643542
TCGA.CI.6621.01	-0.508965563	0.812555503
TCGA.CI.6623.01	-5.64334245	-4.310401498
TCGA.CI.6624.01	1.806431164	0.849778367
TCGA.CK.4947.01	-3.100147781	-0.924782876
TCGA.CK.4948.01	-1.549791548	3.11855672
TCGA.CK.4950.01	-3.443488918	0.922146724
TCGA.CK.4951.01	-3.02566894	6.084577749
TCGA.CK.4952.01	-7.896197574	-3.691690057
TCGA.CK.5913.01	-6.305016138	0.827980969
TCGA.CK.5914.01	-5.665693825	-5.356695363
TCGA.CK.6747.01	-3.193003932	-3.64044603
TCGA.CK.6751.01	-4.732254382	-0.899995501
TCGA.CM.4743.01	-8.234011741	2.260767517
TCGA.CM.4744.01	-8.34963052	-2.834021381
TCGA.CM.4751.01	-0.424686967	2.565004763
TCGA.CM.5860.01	-2.916488957	3.251657009
TCGA.CM.5861.01	-4.190685195	0.348527861
TCGA.CM.5863.01	1.770041784	4.094688277
TCGA.CM.6162.01	5.311522398	7.864634129
TCGA.CM.6169.01	3.327736028	5.433606476
TCGA.CM.6171.01	-5.747488293	0.951188102
TCGA.CM.6674.01	0.869687865	3.265480617
TCGA.CM.6675.01	-5.614674997	-0.068764197
TCGA.CM.6677.01	-0.679205157	-0.89411105
TCGA.CM.6680.01	2.201899149	3.090034954
TCGA.D5.5538.01	-0.780562684	6.870975202
TCGA.D5.5539.01	-0.26731125	3.369410288
TCGA.D5.6529.01	-0.391534083	6.475664122

TCGA.D5.6530.01	-6.249404446	2.239767384
TCGA.D5.6531.01	0.501315057	3.764799
TCGA.D5.6535.01	-4.603002313	0.518256696
TCGA.D5.6536.01	1.406404555	4.705156473
TCGA.D5.6537.01	-5.007716258	-4.411315718
TCGA.D5.6539.01	-3.42271904	-3.628352887
TCGA.D5.6540.01	-5.806892507	3.969525988
TCGA.D5.6541.01	0.072990803	6.189108266
TCGA.D5.6920.01	-4.59735771	-1.014273597
TCGA.D5.6928.01	3.563190675	12.65800118
TCGA.D5.6930.01	1.060988739	4.80941736
TCGA.D5.6931.01	-3.594888851	1.913681339
TCGA.D5.7000.01	-0.826518	0.708341005
TCGA.DC.5337.01	-6.944730072	-3.798008728
TCGA.DC.6158.01	1.16800075	4.447859047
TCGA.DC.6160.01	-5.78049281	-4.903627601
TCGA.DM.A0X9.01	-4.766581046	-4.242163752
TCGA.DM.A1DA.01	-6.906054628	-4.943787948
TCGA.DM.A1DB.01	-1.743110609	-4.706969599
TCGA.DM.A1HB.01	-7.161393864	-0.900751106
TCGA.DM.A280.01	-5.500949419	-1.263697129
TCGA.DM.A28K.01	-6.687747767	-3.600355215
TCGA.DM.A28M.01	-7.272614746	-8.616244613
TCGA.EI.6506.01	-2.632108685	-0.491321472
TCGA.EI.6510.01	-4.817717325	-7.482049794
TCGA.EI.6511.01	-1.015000075	4.409721057
TCGA.EI.6882.01	-7.248721141	-3.314160796
TCGA.EI.6884.01	-0.321016845	0.977743863
TCGA.EI.6917.01	0.35074434	8.883732993
TCGA.F4.6570.01	0.773207683	11.41208202
TCGA.F4.6856.01	-1.646442683	-1.043966362
TCGA.F5.6811.01	1.300114141	2.591875977
TCGA.G4.6293.01	-3.481846631	-0.477570138
TCGA.G4.6295.01	-3.829822983	-1.578685383
TCGA.G4.6297.01	1.668527932	4.725096492
TCGA.G4.6299.01	0.241193492	7.700300472
TCGA.G4.6306.01	-7.267926189	-8.30012664
TCGA.G4.6309.01	-7.465295121	-1.602710945
TCGA.G4.6311.01	-1.924880848	3.766843161
TCGA.G4.6320.01	-3.994634752	-2.654839009
TCGA.G4.6321.01	-5.622788639	-2.377813452
TCGA.G4.6322.01	-3.642989702	-1.738842925
TCGA.G4.6323.01	-2.319084707	-1.530278835
TCGA.G4.6586.01	-8.943206824	0.800993733
TCGA.G4.6588.01	-6.992413388	1.642689332
TCGA.G4.6628.01	-1.68987258	5.834935572
TCGA.NH.A50V.01	-0.35502048	3.644638728
TCGA.NH.A5IV.01	-2.908992864	3.928378991
TCGA.NH.A6GB.01	-7.034646009	-1.595268379
TCGA.QG.A5YW.01	-1.744884367	0.495377244
TCGA.QG.A5Z2.01	-5.640445552	-2.158199725
TCGA.A6.2675.01	3.361697178	4.88398105
TCGA.A6.2684.01	6.760026343	6.610613056
TCGA.A6.2685.01	11.48981462	9.05011345
TCGA.A6.5657.01	8.42434241	4.404055795
TCGA.A6.5664.01	7.011761778	5.923034998
TCGA.A6.6142.01	4.401528828	6.450400988
TCGA.A6.6651.01	15.72283919	10.99207651

TCGA.A6.6654.01	7.274137046	11.48416726
TCGA.A6.6781.01	4.232819474	12.07492863
TCGA.A6.6782.01	4.859953505	5.960906391
TCGA.A6.A5ZU.01	0.989490391	4.645079177
TCGA.AA.3489.01	6.436500116	10.15533489
TCGA.AD.6899.01	4.39500818	6.387497351
TCGA.AD.6901.01	5.990541715	8.787979223
TCGA.AD.6964.01	2.113843236	9.663704245
TCGA.AF.2687.01	10.93908263	8.979900343
TCGA.AF.2690.01	10.79885501	11.16273241
TCGA.AG.3731.01	3.259988309	5.205676931
TCGA.AG.4021.01	2.754047275	5.262928808
TCGA.AG.4022.01	5.211213917	2.121218383
TCGA.AH.6547.01	2.471349543	5.987599066
TCGA.AH.6644.01	4.748890978	1.819736616
TCGA.CA.6717.01	8.807642289	6.823871783
TCGA.CA.6719.01	8.273559786	4.185092669
TCGA.CK.6748.01	6.032982324	4.11338817
TCGA.CM.5348.01	9.250311696	10.30417511
TCGA.CM.5349.01	6.425604149	4.838985192
TCGA.CM.6167.01	15.98868497	10.00454205
TCGA.CM.6168.01	7.573552903	6.937548229
TCGA.CM.6679.01	6.44740701	3.607256673
TCGA.D5.6534.01	7.401545214	14.08175841
TCGA.D5.6898.01	6.231590763	2.050573968
TCGA.DC.6156.01	3.811238228	9.410915627
TCGA.DT.5265.01	10.75346714	9.937244547
TCGA.DY.A1DE.01	4.776184254	1.975300279
TCGA.EI.6507.01	-0.848757043	8.592787685
TCGA.EI.6885.01	6.747309155	4.451038751
TCGA.EI.7004.01	8.577877432	10.45317886
TCGA.F4.6459.01	11.67105574	4.451128356
TCGA.F4.6460.01	12.36288397	2.834763785
TCGA.F4.6461.01	5.318422465	5.575933087
TCGA.F4.6569.01	5.846503035	9.156126485
TCGA.F4.6703.01	5.305187627	14.99402296
TCGA.F4.6704.01	10.36056256	6.642276603
TCGA.F4.6805.01	7.688367011	6.203013471
TCGA.F4.6807.01	8.538313206	5.728614435
TCGA.F4.6809.01	11.57035791	6.925163496
TCGA.F4.6855.01	2.301461094	5.556455031
TCGA.F5.6464.01	11.83021194	10.38806979
TCGA.F5.6465.01	5.256024367	3.947375001
TCGA.F5.6702.01	8.740060788	7.563354364
TCGA.F5.6812.01	12.58596618	5.23910996
TCGA.F5.6813.01	6.506760519	2.845762577
TCGA.G4.6302.01	18.51110043	11.06526433
TCGA.G4.6314.01	10.25675486	3.782415273
TCGA.G4.6625.01	2.736153601	4.455358478
TCGA.G4.6627.01	8.535697381	5.152899908
TCGA.WS.AB45.01	5.183020692	13.12808786

TCGA Fersig score
6.640115729
-9.885858702
-16.13290109
-7.09762733
-1.839581126
1.049765335
-1.975264142
5.054545441
-11.69034551
5.303636536
-9.319891155
-11.34378486
-3.929246802
3.644394857
4.474649156
-2.425327288
-0.862854633
-1.042229434
4.157957507
-6.716576345
7.317830202
-10.47895319
-2.720940407
-2.927533391
-4.021967479
2.536944482
8.327073488
-8.317259646
-3.776656752
1.078541678
-0.774898167
1.999574973
-13.69408562
-0.404940147
3.630332308
-17.17510456
-8.849618715
2.708308757
-14.90998733
-6.968031241
-10.63520312
-6.071475795
-0.977914491
-6.223101705
-9.992762734
1.542960615
-6.880067219
2.006984034
2.700901951
-2.195467128
-10.00001972
-6.775206823
-15.31172898
-6.625802875
-0.225681047

-10.45831502
2.466282858
4.686085467
-7.546976181
1.619455238
-0.547245843
0.195807694
4.401291967
3.447290543
-2.629605957
-0.282651755
-2.686394885
2.514808508
0.36553851
-1.019578549
-11.43880526
5.362500384
-11.3748548
-5.480245482
-5.413301341
9.773831213
2.454389299
9.341158809
9.493648432
3.40465728
3.570560011
-4.470770779
-10.65899795
-6.560410433
0.865250443
-3.943982422
-3.928253707
8.06538603
-5.66759419
-1.001996109
-10.40010481
-13.77056011
-14.61624916
-7.033127558
-7.704396942
-8.099668419
-3.705761525
-5.35423854
-10.2018592
0.340621651
-11.13951999
-17.99178758
-10.02010002
-5.289019985
-10.01251705
-5.637309996
10.20020212
10.32802646
-7.257520286
-6.934558384
-2.25653178
-5.172962641
3.068751346

-5.998964311
-2.058553252
6.952499053
-9.106377822
1.659380464
5.95752038
10.5809582
-1.866427607
-8.441250481
5.112898575
8.656380636
3.310725508
-0.845174
11.88407422
15.89484401
3.446128474
-7.829175269
-7.894671129
12.3177651
-12.90341893
-15.83046895
-14.56456265
-12.26251857
-13.99722965
1.825354711
3.016321607
-5.340450217
9.388276371
-9.03395533
-16.20818446
7.13464249
-10.04651272
-17.85943948
-5.950639061
-12.84462126
10.48165737
-13.56764439
-10.61019142
-2.911943987
4.561358135
0.143692885
8.967056579
-13.33728167
-8.480483053
-1.021402363
-5.251283624
-5.788292576
-8.780623657
1.629215997
-4.152794562
-3.98539227
-10.90306061
-3.245066979
-5.776852284
-4.970369625
-1.310198437
13.47360498
-16.15570513

-5.379184062
-16.81110489
7.029261533
3.410191113
-7.010922439
4.097542977
-7.953644303
-5.890546521
-2.969285175
-11.35699965
0.860521608
5.306274235
-8.396962647
-5.604922952
-11.36236921
-3.205509014
2.264237671
-3.040182482
3.588508762
-12.2370278
-9.543388126
-9.513491172
-15.53952825
-9.827810812
0.979682345
-12.4077511
5.022513399
-3.311457923
-13.05315329
9.416145899
0.30358994
-9.953743947
2.656209531
-4.024930657
1.568765171
-2.521342194
3.058908808
-11.58788763
-5.477035169
-11.02238919
-6.833449962
-5.632249883
-5.973244224
-11.1836519
2.140317796
0.335168052
-3.842157334
5.864730062
13.17615653
8.761342504
-4.796300191
4.135168483
-5.683439194
-1.573316207
5.291934103
6.090412518
3.102099038
6.084130039

-4.009637061
4.266114057
-4.084745617
6.111561029
-9.419031977
-7.051071927
-1.837366519
6.262099069
-5.611631307
16.22119186
5.870406099
-1.681207512
-0.118176995
-10.7427388
5.615859797
-10.68412041
-9.008744798
-11.84984258
-6.450080208
-8.06214497
-6.764646548
-10.28810298
-15.88885936
-3.123430156
-12.29976712
3.394720982
-10.56288194
0.656727018
9.234477333
12.1852897
-2.690409045
3.891990118
-3.95941677
-5.408508366
6.393624424
7.941493963
-15.56805283
-9.068006066
1.841962314
-6.649473761
-8.000602091
-5.381832626
-3.849363542
-8.142213091
-5.349724056
4.145062992
3.289618248
1.019386128
-8.629914388
-1.249507123
-7.798645276
8.245678228
13.3706394
20.53992807
12.8283982
12.93479678
10.85192982
26.7149157

18.75830431
16.30774811
10.8208599
5.634569568
16.59183501
10.78250553
14.77852094
11.77754748
19.91898297
21.96158742
8.46566524
8.016976084
7.3324323
8.45894861
6.568627594
15.63151407
12.45865245
10.14637049
19.5544868
11.26458934
25.99322702
14.51110113
10.05466368
21.48330363
8.282164731
13.22215385
20.69071169
6.751484534
7.744030641
11.19834791
19.03105629
16.1221841
15.19764775
10.89435555
15.00262952
20.29921059
17.00283917
13.89138048
14.26692764
18.49552141
7.857916125
22.21828172
9.203399369
16.30341515
17.82507614
9.352523096
29.57636476
14.03917013
7.191512078
13.68859729
18.31110855

Table S6. Fersig score of different databases

samples	GEO PC1 of genes A	GEO PC1 of genes B
GSM929508	8.85130345	-7.295086787
GSM929511	-0.957958463	-6.285103184
GSM929622	10.79007285	-1.560711018
GSM929546	-1.634714291	3.770913664
GSM929623	-0.308297316	1.974421849
GSM929539	15.07758073	-10.21946797
GSM929614	0.13553872	-20.61409952
GSM929496	-2.703542177	0.184081895
GSM929574	1.543691318	-3.253491998
GSM929590	-0.465568017	-15.3144753
GSM929586	-2.279737211	7.414028219
GSM929585	0.119065243	-1.957691103
GSM929612	1.046043718	-0.936528876
GSM929609	4.049664033	-7.109561602
GSM929497	-3.605444797	4.789586521
GSM929593	-0.690672322	-6.818756241
GSM929591	0.881532088	-1.341325536
GSM929551	-4.343781449	8.574747245
GSM929587	-0.010980216	3.374354188
GSM929523	-1.096703117	1.680318164
GSM929519	0.602493063	3.718900479
GSM929524	-0.145079439	7.953714309
GSM929525	2.092532149	7.487982467
GSM929599	-0.717271071	-2.739983058
GSM929617	-2.023578634	-8.832199327
GSM929576	-0.468334373	-1.557499297
GSM929572	6.632231507	-14.13124325
GSM929499	-0.263725886	-9.613940855
GSM929504	5.880390964	-2.939265854
GSM929610	0.646231871	8.190159859
GSM929498	-3.387332469	3.142340935
GSM929618	-0.189039517	4.249693533
GSM929575	6.185546865	-14.65495758
GSM929577	0.217391682	-4.561913489
GSM929502	-2.433816523	-2.589629328
GSM929503	6.987976528	-10.1339663
GSM929500	-2.233183827	-0.72654717
GSM929494	-3.086915645	10.29784978
GSM929495	-2.12587946	2.851842114
GSM929501	4.684325081	-26.59148471
GSM929505	0.941750354	-5.649258686
GSM929506	-3.180355649	7.856462242
GSM929507	3.48367521	-4.347272376
GSM929509	-0.548123706	-1.63648108
GSM929510	-2.08308992	-3.080411563
GSM929512	7.827081356	-3.574888977
GSM929513	3.814492775	-8.812499148
GSM929514	-0.621095737	4.84105847
GSM929515	-2.101350158	11.31165439
GSM929516	0.287983609	7.553912493
GSM929517	-3.408969027	8.681052039
GSM929518	0.557235929	6.001798516
GSM929520	-2.764880197	0.328417291
GSM929521	-2.333239466	-5.330863401

GSM929522	0.60682118	-0.147320797
GSM929526	1.088307856	5.487813498
GSM929527	0.594974391	-0.153321128
GSM929528	-2.872936738	3.931701468
GSM929529	-1.021551492	5.716432848
GSM929530	3.713636299	0.192631114
GSM929531	-2.256210924	-4.615771516
GSM929532	-4.101014382	3.373197063
GSM929533	-2.557510596	6.709718173
GSM929534	-1.933118248	0.344396292
GSM929535	-2.404272238	9.48165198
GSM929536	-2.624729133	7.383644145
GSM929537	-3.940637677	5.304448838
GSM929538	-3.840573269	6.572572589
GSM929540	-1.892740771	6.65356679
GSM929541	0.048518208	3.709091052
GSM929542	-4.51719246	5.673147088
GSM929543	-3.990497493	9.492229002
GSM929544	-1.012032938	2.827367047
GSM929545	-0.918020748	6.183016758
GSM929547	-2.847864821	13.5468785
GSM929548	8.569583154	-5.547372256
GSM929549	16.43717585	-10.106919
GSM929550	-2.253525729	10.52953145
GSM929552	-0.530772207	-5.076095788
GSM929553	1.51314483	-1.411151268
GSM929554	-2.37556405	-3.566792585
GSM929555	-0.134319485	-0.366571018
GSM929556	-2.222630419	4.845770461
GSM929557	-2.135306143	10.53875464
GSM929558	-4.318599743	6.106825201
GSM929559	-1.452800014	-26.47739241
GSM929560	-0.624732059	2.678821717
GSM929561	-0.779272194	5.467208493
GSM929562	5.359104978	-8.901188824
GSM929563	-1.477779036	5.809469504
GSM929564	-2.074385803	5.9357603
GSM929565	-1.010278997	4.299636816
GSM929566	-3.310830651	6.070836103
GSM929567	-0.233391024	8.78108443
GSM929568	2.516358908	5.47553824
GSM929569	1.352202646	3.089827372
GSM929570	-2.415784027	9.976747791
GSM929571	0.695648048	3.714888386
GSM929573	3.938911183	-13.78708769
GSM929578	0.143440473	-16.77963814
GSM929579	0.119055068	4.132329794
GSM929580	-5.438165058	7.823482296
GSM929581	-2.695937803	7.473257862
GSM929582	7.412088945	-10.64694254
GSM929583	-2.196166805	4.178467382
GSM929584	-1.443753838	3.092562668
GSM929588	2.513776398	1.088218977
GSM929589	-3.117654609	3.576938313
GSM929592	-0.422904783	-12.53845409
GSM929594	5.703049665	-11.14981727
GSM929595	-1.984123114	3.522845328
GSM929596	-2.045162751	4.680220254

GSM929597	-1.511671464	2.445333357
GSM929598	-2.270192471	-1.490206664
GSM929600	2.288481232	-2.300527195
GSM929601	1.602522013	-6.80235271
GSM929602	-3.213998773	6.529653434
GSM929603	0.109467747	-0.99522796
GSM929604	-1.760777215	-0.63629
GSM929605	-2.264767129	0.018790091
GSM929606	2.347125278	-6.705604835
GSM929607	-3.664276167	-2.958449035
GSM929608	0.304123257	3.680275335
GSM929611	0.608439793	-0.210500987
GSM929613	-3.050047111	9.338903169
GSM929615	-2.17337021	5.865577984
GSM929616	-1.266264072	-9.200899775
GSM929619	-0.196447396	1.113638683
GSM929620	1.690082784	-10.13455542
GSM929621	-1.32090569	-5.854905164
GSM358341	-2.837193064	4.821438457
GSM358342	1.290314483	-10.39401924
GSM358343	-2.235066499	-2.773462184
GSM358344	6.636713993	-3.582498586
GSM358345	0.472669594	-6.325859041
GSM358346	2.923900817	3.98397742
GSM358347	-1.119573016	-0.584895435
GSM358348	0.496556335	5.316590229
GSM358349	0.508230718	-0.231309633
GSM358350	-1.930165205	3.016098964
GSM358351	-0.076217003	-5.402553352
GSM358352	-0.018471049	1.610286397
GSM358353	-2.845576786	4.438115813
GSM358354	-5.677842101	6.78063542
GSM358355	1.048722554	1.321955158
GSM358356	-1.133365067	7.68098893
GSM358357	-2.545485889	8.615413457
GSM358358	-2.958563176	3.08765266
GSM358359	1.598853841	-16.60368316
GSM358360	-3.224315327	-0.695149373
GSM358361	-2.200150148	5.360723022
GSM358362	-2.418072307	7.011837932
GSM358363	0.40214042	5.087054783
GSM358364	0.52759159	-9.965996509
GSM358365	-0.626524558	-2.432052254
GSM358366	-2.568133491	6.288561542
GSM358367	0.631710924	4.846341504
GSM358368	-0.288490142	-1.019876407
GSM358370	1.54921888	7.710320737
GSM358371	-3.588927546	10.10372349
GSM358373	-1.503527559	5.692022981
GSM358374	-2.371092432	-4.693440527
GSM358375	-1.945418517	7.918287618
GSM358376	-3.447633029	6.076712044
GSM358377	-5.76835928	8.011347593
GSM358378	-0.525284525	8.878849995
GSM358380	-1.166367981	9.285249359
GSM358381	-1.085446818	0.771002702
GSM358382	-2.029999201	0.808274508
GSM358383	2.139009404	0.172164581

GSM358384	9.699112702	-5.628306983
GSM358385	-2.257306396	-6.918073291
GSM358386	6.182953975	2.448142631
GSM358387	2.276276176	-1.503627427
GSM358388	-0.861395445	-4.049296132
GSM358389	5.895097293	-2.023365933
GSM358390	2.889727994	0.141571593
GSM358391	-1.912981352	-1.953744058
GSM358392	2.460873834	-6.875239063
GSM358393	-0.593757317	3.193944255
GSM358394	-1.82224874	1.34220182
GSM358395	-0.802400136	-7.004091206
GSM358396	-0.034113012	1.44229063
GSM358397	4.029963434	1.660847933
GSM358398	-4.851974726	0.159226459
GSM358399	-2.492507569	2.309790211
GSM358400	0.18490116	-3.498637838
GSM358401	-0.979489684	4.549616394
GSM358402	-1.138422844	7.499121007
GSM358403	0.909689809	0.937671549
GSM358404	-1.553051207	-6.569287049
GSM358405	-0.12011304	-1.929760069
GSM358406	0.013422346	0.427410628
GSM358407	5.108850094	2.640277337
GSM358408	-1.249568566	2.497671724
GSM358409	7.351621542	-9.37960611
GSM358410	-2.210363035	-0.117373537
GSM358411	1.595945365	-6.153553513
GSM358412	-1.686840724	-1.424123052
GSM358413	-2.632268068	-8.920923217
GSM358414	-2.520760582	3.670047288
GSM358415	-1.812455255	-6.497723988
GSM358416	0.392115739	0.735237221
GSM358417	3.640739705	-8.851722836
GSM358418	-3.028980928	5.497529523
GSM358419	1.958115627	-5.754610196
GSM358420	-0.115423663	3.342535762
GSM358421	5.433476978	-0.037887754
GSM358422	-6.183805433	3.61227778
GSM358423	-1.933385442	0.980501951
GSM358424	-0.62047284	-6.008111397
GSM358425	3.452564899	-7.622480592
GSM358426	2.619566245	-4.683492954
GSM358427	-1.567728611	-3.938560427
GSM358428	4.916865614	-3.365933117
GSM358429	-1.239578577	1.933226947
GSM358430	-1.591357158	4.368937284
GSM358431	3.046229454	3.742784064
GSM358432	-1.278339275	2.428473589
GSM358433	-3.387187018	2.979675079
GSM358434	-1.350576618	7.033696787
GSM358435	4.374590814	-0.490015631
GSM358436	4.0978544	-5.884934455
GSM358437	-0.800015228	6.805094468
GSM358438	0.558282542	-8.105092703
GSM358439	-0.749779561	6.198372661
GSM358440	2.892669922	0.425454484
GSM358441	-0.290136454	-0.205049343

GSM358442	1.902963073	0.417517326
GSM358443	5.325539906	-0.179804952
GSM358444	-1.090178314	4.719029527
GSM358445	-5.176879915	10.7489861
GSM358446	1.55180018	-6.67402246
GSM358447	-3.230840112	-13.11519247
GSM358448	2.83987759	7.557427747
GSM358449	0.613512536	7.236317776
GSM358450	0.521155552	-1.197260059
GSM358451	-3.12448579	6.163718838
GSM358452	-1.102526003	0.738331395
GSM358453	-4.971468265	2.70346932
GSM358454	-2.590159516	3.017239549
GSM358455	-2.197918493	11.80786721
GSM358456	-2.531940482	6.207029283
GSM358457	-6.289454656	2.695635513
GSM358458	-3.594717333	-3.059023286
GSM358459	-6.570956032	7.898088796
GSM358460	-1.686035968	10.24822502
GSM358461	-3.336013565	9.059800652
GSM358462	10.40106569	-11.78404674
GSM358463	-0.946785691	-0.962003859
GSM358464	2.963563596	2.611269573
GSM358465	-2.84139344	1.088215112
GSM358466	4.985133811	-1.656916326
GSM358467	5.882202584	-5.442046783
GSM358468	7.037259143	0.139336544
GSM358469	0.019691543	4.320224014
GSM358470	0.011033689	-5.333937684
GSM358471	-0.773014941	1.131259782
GSM358472	9.896502306	-3.197982326
GSM358473	-0.561582055	4.250143202
GSM358474	0.620774202	4.820100995
GSM358475	13.57344844	-2.295072992
GSM358476	0.392395964	2.129153476
GSM358477	-2.649481618	-16.4779029
GSM358478	10.61472866	-4.579097704
GSM358479	-1.880791328	-0.973081428
GSM358480	-1.223675881	2.325004952
GSM358481	-2.256381199	-0.981146432
GSM358482	-1.199327516	4.285808875
GSM358483	2.114218288	8.18421782
GSM358484	-2.828738337	-0.521474766
GSM358485	-3.129410999	2.419662407
GSM358486	1.542187268	-1.977001879
GSM358487	-1.583414151	-3.509463821
GSM358488	0.324791685	0.260848578
GSM358489	-0.168495064	-2.311762219
GSM358490	-0.434021871	2.717601923
GSM358491	-0.637334878	-7.507961644
GSM358492	3.107829328	-8.626670325
GSM358493	3.772185434	-18.83758042
GSM358494	2.142647737	-9.12116231
GSM358495	-2.123416095	7.026120917
GSM358496	2.573905182	-2.152902386
GSM358497	-1.796599855	-3.008587411
GSM358498	-5.876104876	1.966300335
GSM358499	0.625906297	-4.904084116

GSM358500	-4.38716387	-9.779928337
GSM358501	7.025366156	-8.509842463
GSM358502	-3.200282681	-3.157733639
GSM358503	-3.906355988	-3.016058799
GSM358504	-0.683879644	-4.267269563
GSM358505	0.787617392	1.253002006
GSM358506	0.372645622	3.442499683
GSM358507	1.912907032	-9.263180606
GSM358508	0.249632804	2.654597575
GSM358509	-0.943361501	-9.114249319
GSM358510	-0.037581317	-14.87982117
GSM358511	-2.874821964	10.45702925
GSM358512	0.61760737	-1.778985111
GSM358513	4.291528172	-7.816081207
GSM358514	-3.362491464	-3.719941087
GSM358515	2.786062951	-8.640919292
GSM358516	-0.442883671	-0.642879182
GSM358517	-1.197078531	-0.798475688
GSM358518	0.860640362	2.782418147
GSM358519	-0.766412892	4.185043349
GSM358520	-3.126612486	-10.10822743
GSM358521	-1.824862812	6.296714774
GSM358522	-0.415269243	7.070992914
GSM358523	-2.54144055	6.459868541
GSM358524	-0.479321858	-0.53278447
GSM358525	-1.210960008	-11.10176847
GSM358526	-3.105230419	1.892009155
GSM358527	-3.357624954	-1.964317789
GSM358528	-4.081532849	2.964118759
GSM358529	-1.358256677	-2.451995683
GSM358530	1.521474968	-2.357924512
GSM358531	-0.805571277	5.623755304
GSM358532	-1.340115865	-9.86691897
GSM358533	1.663846242	0.663957255
GSM358534	-0.481845623	-2.886654276
GSM358535	0.306553892	-6.192759891
GSM358536	-0.445977636	-4.104001288
GSM358537	2.162645881	4.472660634
GSM358538	0.34111549	-7.485541457
GSM358539	1.591533891	5.356995836
GSM358540	1.807073245	7.393199844
GSM358541	12.31719798	-17.23082522
GSM358542	2.844311913	2.977044762
GSM358543	-0.899892659	6.578598237
GSM358544	4.145275368	5.44772501
GSM358545	-2.283061934	5.496713808
GSM358546	-4.589099265	0.658226787
GSM358547	0.506267523	0.267992948
GSM358548	0.60260036	3.349618577
GSM358549	-0.231803326	3.770489515
GSM358550	-3.208817252	11.67385282
GSM358551	-3.202457981	-3.876167713
GSM358552	-1.622491069	4.25128525
GSM358553	3.130988056	-5.735572002
GSM358554	-5.8049936	-4.066054088
GSM358555	-0.704534427	2.082218777
GSM358556	-4.226099087	10.84187646
GSM358557	-0.218520717	3.88415527

GSM358558	4.470424863	-4.894558674
GSM358559	-2.77503434	5.139565893
GSM358560	0.86541943	8.999209466
GSM358561	-1.902773765	3.156893449
GSM358562	-1.617457236	4.339522994
GSM358563	-3.922202854	-4.49929164
GSM358564	1.689210221	1.008421115
GSM358565	3.709462225	4.084939204
GSM358566	-2.894958847	5.53239597
GSM358567	6.048858519	-5.106412278
GSM358568	11.91597054	-6.530905701
GSM358569	-1.153548746	3.165586262
GSM971957	0.359194938	-2.150756119
GSM971958	6.495958423	-5.93221995
GSM971959	-2.768626854	2.12755566
GSM971960	-1.484508334	4.226776824
GSM971961	-0.787818869	-19.08679045
GSM971962	5.926134363	-10.46171763
GSM971963	-2.10453109	-16.83170873
GSM971964	-3.212480033	2.251966546
GSM971965	0.506004669	0.240252605
GSM971966	-1.621631278	-0.076328255
GSM971968	-0.787141645	-11.31262727
GSM971969	-2.570642086	-6.18413531
GSM971970	-4.52631332	4.414958831
GSM971971	-2.768993035	-3.11853235
GSM971972	-1.621817237	-0.688055575
GSM971973	-3.020822036	1.771833876
GSM971974	-0.646459842	0.137573843
GSM971975	-1.692027996	1.179875344
GSM971976	-2.063497469	6.661056218
GSM971977	-2.354327617	-7.787640353
GSM971978	-4.507626905	-1.400620661
GSM971979	-3.729040694	-6.05226234
GSM971980	-3.615128548	-4.96272379
GSM971981	-3.682473796	-9.509518238
GSM971982	-3.079500216	-10.21443005
GSM971983	-3.072759545	-9.275710235
GSM971984	-4.053127119	-5.869605245
GSM971985	-3.215762181	5.992014912
GSM971986	2.980451194	-7.575504304
GSM971987	1.671582951	-5.983392125
GSM971988	-1.826391476	1.955221657
GSM971989	-3.818382184	6.688753769
GSM971990	-4.612778581	-0.613417651
GSM971991	-0.591752483	-1.245160609
GSM971992	-2.072719124	4.362160427
GSM971993	-1.387513937	-4.770169596
GSM971994	-3.154374731	3.933007053
GSM971995	-1.92821742	-1.224913444
GSM971996	2.590042911	-16.06823626
GSM971997	-2.342729525	-9.572882859
GSM971998	-2.201540184	-0.86085936
GSM971999	-1.271915961	-5.242871279
GSM972000	1.682719916	0.298889729
GSM972001	-3.312893716	3.240876259
GSM972002	-3.996211465	3.024793867
GSM972003	0.098988542	3.917984416

GSM972004	-1.559095585	0.810154034
GSM972005	-2.338479578	9.737905701
GSM972006	-1.667924	-1.794188863
GSM972007	6.142564957	-9.747251698
GSM972008	-0.297172775	-15.2941879
GSM972009	-0.139005731	-3.79069325
GSM972010	1.673309271	-2.112046842
GSM972011	3.44893794	-9.154178002
GSM972012	-2.108523205	4.363054364
GSM972013	-0.568775504	5.182416264
GSM972014	-3.411727618	6.796129784
GSM972015	-3.989102244	0.563953215
GSM972016	-3.027399828	-4.373311982
GSM972017	4.132902587	-2.219107422
GSM972018	2.201485972	-5.120410846
GSM972019	4.141994902	-9.014456561
GSM972020	-0.467893838	-0.171437873
GSM972021	-0.776699545	1.190131155
GSM972022	1.210240574	-0.219671191
GSM972023	1.067214572	-0.557196538
GSM972024	-0.627602108	5.332387779
GSM972025	-1.344947383	2.412070832
GSM972026	9.575500341	-11.70467319
GSM972027	5.363052893	2.234313466
GSM972028	3.873357823	-5.991687076
GSM972029	9.338773388	-5.101067454
GSM972030	8.315733783	-10.18186878
GSM972031	-1.162704525	5.013361186
GSM972032	-3.734187894	7.134481862
GSM972033	0.109087027	9.663440431
GSM972034	2.681401231	6.394440935
GSM972035	-1.711183532	11.19873596
GSM972036	1.167827372	4.125562204
GSM972037	-0.229408422	3.888973438
GSM972038	-0.139966932	6.371568925
GSM972039	16.66187859	-13.03009033
GSM972040	-1.794246662	7.564667887
GSM972041	0.53998553	4.260174272
GSM972042	-4.139379891	6.887551088
GSM972043	-3.693468734	8.138299901
GSM972044	-3.822516497	10.98649641
GSM972045	-3.002180005	7.805433155
GSM972046	1.929590944	-9.565682895
GSM972047	-1.00263579	3.973511159
GSM972048	-0.371501067	4.551827786
GSM972049	-1.784632822	6.720766785
GSM972050	-1.590294982	6.620728101
GSM972051	15.33170811	-7.823624788
GSM972052	-2.038704933	5.993006116
GSM972053	-2.036605572	11.69890945
GSM972054	6.462483725	-7.685874977
GSM972055	0.309903597	-6.082690832
GSM972056	-1.175059028	-1.158345714
GSM972057	3.440316993	-0.635358951
GSM972058	-2.659941709	-12.09311698
GSM972059	5.790483262	-21.1239496
GSM972060	-0.105437953	-1.939522558
GSM972061	3.906702874	-4.278418202

GSM972062	0.51720295	-1.759983293
GSM972063	-1.504285743	-3.078267347
GSM972064	0.897816868	-13.56546663
GSM972065	-0.338604527	-2.397921764
GSM972066	0.299351912	-1.480551467
GSM972067	0.636438289	-6.44280152
GSM972068	0.515121057	-20.32077186
GSM972069	-1.410514764	1.868890633
GSM972070	-0.895144794	-1.324584502
GSM972071	-1.486849843	1.803285399
GSM972072	-3.662833734	-8.457321145
GSM972073	-0.43242762	-9.502884287
GSM972074	1.171239754	-2.526639067
GSM972075	-2.365117357	-2.274812723
GSM972076	-0.168829681	-5.960540144
GSM972077	4.804778721	-18.14001781
GSM972078	-0.157559704	-4.243058174
GSM972079	5.346366709	-19.57531038
GSM972080	-2.708036844	-3.114564388
GSM972081	0.304564617	1.001670902
GSM972082	3.528851003	-11.30136496
GSM972083	1.577092557	-3.790873892
GSM972084	7.255102486	-6.57358766
GSM972085	-0.43485446	-3.950442922
GSM972086	-0.485394647	7.464701428
GSM972087	-1.278205832	5.011996653
GSM972088	10.23854593	-4.874158638
GSM972089	18.35582199	-9.474181654
GSM972090	-2.179792715	11.32445852
GSM972091	-3.723293519	9.66468854
GSM972092	-0.127007641	-3.564660938
GSM972093	1.871993706	-0.364155249
GSM972094	-1.846107866	-2.766470652
GSM972095	0.090084691	0.82581327
GSM972096	2.763906732	8.686092087
GSM972097	7.325533365	-13.36876817
GSM972098	0.659679477	7.100879942
GSM972099	1.154091552	7.105245394
GSM972100	0.915407252	0.96636639
GSM972101	1.970242534	-2.240698576
GSM972102	4.476740594	-13.42228302
GSM972103	-0.519605165	-5.6419045
GSM972104	5.195519446	-8.744508418
GSM972105	8.303160515	-2.034182288
GSM972106	-0.601013454	2.672682542
GSM972107	1.419553927	-5.983550363
GSM972108	-1.58758468	5.387533157
GSM972109	-2.563640686	1.071124455
GSM972110	-0.649611219	-0.464957198
GSM972111	0.463166544	-3.141134658
GSM972112	0.094703456	-15.80003225
GSM972113	6.782982322	-14.34314428
GSM972114	-2.086447886	0.983641197
GSM972115	0.423553746	2.392607061
GSM972116	12.00362389	-0.676951463
GSM972117	-2.437558693	-8.283424696
GSM972118	-0.159063572	5.9084813
GSM972119	0.3859443	5.24344962

GSM972120	-2.57734353	-5.064035495
GSM972121	0.142338945	1.047242088
GSM972122	-0.083687447	9.746989636
GSM972123	-0.725727515	6.099875855
GSM972124	0.044288155	-1.328504867
GSM972125	11.0734715	-7.245447327
GSM972126	-2.128936867	12.55108394
GSM972127	4.276846606	-3.770907613
GSM972128	-0.918138189	-5.632392241
GSM972129	-2.135769516	-2.723533126
GSM972130	0.628555943	8.962904837
GSM972131	2.428425845	-10.26927911
GSM972132	1.288719802	1.394457372
GSM972133	4.467623428	-9.002099903
GSM972134	1.860332544	-0.255515758
GSM972135	1.585594812	6.124587553
GSM972136	-0.257501914	3.507186182
GSM972137	1.790220849	4.094405463
GSM972138	0.428311741	3.616463246
GSM972139	1.83671213	3.905238451
GSM972140	3.295672993	-11.08725713
GSM972141	-0.793178274	-4.620769436
GSM972142	-0.010931536	4.987463747
GSM972143	1.93123337	-1.138684915
GSM972144	2.208614538	2.69451532
GSM972145	1.464988527	0.373072167
GSM972146	2.59004838	-2.623992178
GSM972147	-1.346285014	3.408131198
GSM972148	2.976240624	0.854580005
GSM972149	5.94303258	-3.421400645
GSM972150	1.168568746	-1.648888662
GSM972151	0.419969732	3.25292786
GSM972152	-0.657331333	5.068544465
GSM972153	-0.097584753	-0.167609962
GSM972154	0.231995558	2.472388892
GSM972155	0.457789542	4.312472006
GSM972156	0.52258119	-0.303901432
GSM972157	3.710616791	0.698502327
GSM972158	0.79175063	-11.67645609
GSM972159	0.416882037	-0.200925885
GSM972160	3.204636664	-1.852291922
GSM972161	2.328534062	0.043638647
GSM972162	3.356288508	-6.206654915
GSM972163	1.757625875	-1.757191694
GSM972164	0.355248198	-2.355223706
GSM972165	0.598565798	-3.216476583
GSM972166	0.094318623	0.096407951
GSM972167	0.126981686	-0.945631462
GSM972168	1.179184403	-7.873066751
GSM972169	0.749868734	1.855551129
GSM972170	2.18678802	-4.035453795
GSM972171	1.776816755	-14.20095211
GSM972172	5.840512299	-2.22516333
GSM972173	2.776970891	-3.537170065
GSM972174	3.382852683	-2.093309009
GSM972175	4.140101434	-2.436386542
GSM972176	3.64369315	-11.60733813
GSM972177	4.949740061	-6.402423993

GSM972178	0.895989544	-2.689544735
GSM972179	6.035671798	-16.13666115
GSM972180	-0.094269488	-9.630673408
GSM972181	6.83382358	-21.45081103
GSM972182	3.231597476	-3.023338781
GSM972183	3.917297732	1.972128752
GSM972184	3.530092445	-12.56403347
GSM972185	6.499751776	-2.825537831
GSM972186	12.91383881	-18.41057614
GSM972187	1.7474049	-10.17723726
GSM972188	5.052489525	-3.849591922
GSM972189	-0.6916955	-5.571138202
GSM972190	4.01148519	-0.386331593
GSM972191	1.172624805	2.115840723
GSM972192	1.974082344	-8.567998543
GSM972193	2.391036726	-0.472803342
GSM972194	3.138942306	-4.142873257
GSM972195	0.196433193	-3.109632803
GSM972196	-0.377903124	-3.60697477
GSM972197	1.319861381	-1.464800568
GSM972198	0.450855713	2.283599227
GSM972199	1.442189158	1.675552603
GSM972200	-0.625902517	-2.035308453
GSM972201	3.019929904	-10.67703129
GSM972202	-0.525872015	0.528755311
GSM972203	-0.231350102	-4.373070814
GSM972204	1.074796828	-6.182300397
GSM972205	3.781129821	-6.328325495
GSM972206	1.307272484	-4.040088493
GSM972207	0.216592827	-0.424783192
GSM972208	0.198917706	-11.40687853
GSM972209	-1.509735483	-9.041510074
GSM972210	8.034867627	-3.882253466
GSM972211	5.500592657	1.658606669
GSM972212	-0.575523162	2.560171171
GSM972213	-0.141684333	-1.450145537
GSM972214	-1.567913322	1.572139892
GSM972215	-0.191464165	-6.712040985
GSM972216	1.562416553	-0.148989848
GSM972217	2.825577554	-2.216088858
GSM972218	-1.4952423	3.558793282
GSM972219	2.163118792	3.745741778
GSM972220	-4.184801951	0.17723612
GSM972221	-5.485324354	9.042328266
GSM972222	-3.926387935	6.696995318
GSM972223	-2.305951585	1.858696002
GSM972224	-2.645227429	-5.459739725
GSM972225	-1.716800636	5.285990409
GSM972226	-1.127487789	4.699600381
GSM972227	-1.600824105	8.805873172
GSM972228	-2.145969453	8.156613834
GSM972229	-5.813352594	1.834750628
GSM972230	-1.795296985	10.40046697
GSM972231	3.489242074	-2.056888013
GSM972232	0.157605079	0.291257004
GSM972233	-1.214464352	0.762555709
GSM972234	2.897988329	-0.579469348
GSM972235	-2.265483823	6.021022481

GSM972236	-0.606079189	7.523997357
GSM972237	-1.801680467	8.545886211
GSM972238	-2.546473535	5.986549414
GSM972239	-1.517624793	3.360894989
GSM972240	-0.982178363	-1.961594899
GSM972241	-1.112164625	6.175689087
GSM972242	-0.629969858	0.685246036
GSM972243	-1.21380137	5.127405044
GSM972244	3.915163399	-0.609719186
GSM972245	-0.492691827	5.513874367
GSM972246	1.190355969	7.30370913
GSM972247	0.152551	7.269705316
GSM972248	-0.756335203	6.770783941
GSM972249	-2.021955289	7.06473833
GSM972250	-0.903146629	7.471904003
GSM972251	1.293691828	0.678230976
GSM972252	-1.451306854	2.657858321
GSM972253	0.020530247	8.502742039
GSM972254	5.355793728	-0.374920571
GSM972255	-1.131793291	4.054266225
GSM972256	1.148714265	6.455400944
GSM972257	1.364471428	3.407721738
GSM972258	2.51469035	5.420682582
GSM972259	0.416428096	3.049104714
GSM972260	-0.888157084	4.043130177
GSM972261	-2.451574687	4.527932092
GSM972262	-1.753553547	-12.15343101
GSM972263	-2.256802081	1.839237271
GSM972264	-4.861463759	6.100434417
GSM972265	-1.994365401	3.12813033
GSM972266	-4.589587858	13.66618605
GSM972267	1.863468282	5.896719937
GSM972268	-0.465195629	5.942456509
GSM972269	2.50628618	1.708887203
GSM972270	-3.286885424	2.416806415
GSM972271	-0.711601928	8.692757119
GSM972272	-1.573475363	9.182404309
GSM972273	0.030786161	7.015105315
GSM972274	-3.296883755	-0.868930032
GSM972275	0.491782545	-13.3892347
GSM972276	-1.201016447	4.858873642
GSM972277	-2.561042295	6.151940612
GSM972278	-4.129819166	6.494411192
GSM972279	-3.033607135	-0.362369258
GSM972280	-1.481822882	-0.936152324
GSM972281	-3.257623185	5.72050733
GSM972282	-3.649747294	7.35196915
GSM972283	-3.03319955	1.382646004
GSM972284	-1.535531984	11.22031225
GSM972285	-0.465159838	5.092366212
GSM972286	-2.446511907	2.98077482
GSM972287	-1.84460145	-8.01624883
GSM972288	2.418761835	6.352595768
GSM972289	-3.713724052	11.37874603
GSM972290	-0.682072385	-1.987667498
GSM972291	0.966689768	2.304988179
GSM972292	-2.856702674	4.085293817
GSM972293	-1.39852854	1.94976875

GSM972294	-2.577817469	-3.914368909
GSM972295	2.324154969	6.81182479
GSM972296	-3.292736544	8.018303814
GSM972297	-0.620467265	-2.629906317
GSM972298	-2.851634418	5.165029271
GSM972299	0.492937535	4.897693113
GSM972300	-1.542346021	6.061667134
GSM972301	-0.538256544	-3.024216238
GSM972302	-3.986119901	11.30953795
GSM972303	-3.175023178	11.99755356
GSM972304	-3.744800278	11.95378612
GSM972305	-2.277815316	-3.737958107
GSM972306	-2.756673008	11.36557568
GSM972307	-2.203960565	8.697426064
GSM972308	-5.542381597	2.549433353
GSM972309	-3.189653596	14.57350597
GSM972310	-4.703633029	7.760986387
GSM972311	1.825407688	3.88666348
GSM972312	-3.230086346	10.19253586
GSM972313	-3.150171373	12.75690507
GSM972314	-3.947911459	0.27313072
GSM972315	-3.60113185	9.502632324
GSM972316	-4.224595372	5.303315173
GSM972317	-5.974712189	3.047016322
GSM972318	-2.88853663	7.562908252
GSM972319	-3.079717526	10.29788099
GSM972320	-1.45189718	7.489498446
GSM972321	-2.871406762	7.066834485
GSM972322	-1.812210773	3.918473323
GSM972323	-2.938314168	11.12723973
GSM972324	-3.516815643	8.970972256
GSM972325	-4.618771674	3.232448117
GSM972326	-3.494653732	9.762061102
GSM972327	-2.250673269	-3.685265656
GSM972328	-0.418285656	8.549648115
GSM972329	-3.886904154	4.593877867
GSM972330	-4.915132087	0.865235591
GSM972331	-2.860294209	7.91216594
GSM972332	-4.347236643	4.125530424
GSM972333	-2.266190587	10.9414374
GSM972334	-4.374998383	-5.2854569
GSM972335	-2.373969127	8.428796035
GSM972336	-3.653820148	9.339002177
GSM972337	-3.627593573	-3.559547088
GSM972338	-2.838503451	8.56732778
GSM972339	-4.152196373	9.080618576
GSM972340	-3.895031119	6.305656244
GSM972341	-4.592919641	5.486413747
GSM972342	-2.157083287	7.249884404
GSM972343	-3.53256137	7.831132815
GSM972344	-2.74926006	9.115838205
GSM972345	3.835661926	1.965751665
GSM972346	-3.985289109	8.681416899
GSM972347	-4.589058368	10.17002837
GSM972348	-5.445218453	9.888599906
GSM972349	1.745369404	-7.649052446
GSM972351	1.354418965	5.816178289
GSM972352	-0.925887189	-5.322869269

GSM972353	-2.203289385	-0.53855489
GSM972354	-2.130189978	4.227272043
GSM972355	1.161076512	-0.936462688
GSM972357	4.508571454	0.64267834
GSM972358	-1.843036517	1.504954282
GSM972359	16.98009792	-9.685989061
GSM972360	-1.640200768	-0.12336099
GSM972361	1.369430741	-20.55404688
GSM972362	-1.662485276	4.216266879
GSM972363	-3.370117298	-1.981799717
GSM972364	-3.196622284	4.5073015
GSM972365	0.084526922	-12.27430012
GSM972366	-0.005649517	-6.194129506
GSM972367	6.585718069	-11.23679967
GSM972368	-2.302768735	7.258996581
GSM972369	-0.100485655	-15.53663642
GSM972370	1.172227873	0.018820554
GSM972371	1.50452359	4.119553247
GSM972372	-0.065263872	-2.182307136
GSM972373	0.896744965	9.098224147
GSM972374	-1.782752457	-0.767912063
GSM972375	3.724414094	-1.949812198
GSM972376	1.516585812	0.141745087
GSM972377	2.561222819	-6.030710999
GSM972378	0.453889188	-0.536797824
GSM972379	-1.589356947	3.594800929
GSM972380	5.402430434	-7.002991206
GSM972381	0.757406934	-0.974383111
GSM972382	0.040693825	4.396750536
GSM972383	-1.179245436	3.656026884
GSM972384	3.761003034	2.204496597
GSM972385	8.783465584	-10.50806862
GSM972386	-1.984300321	5.466956681
GSM972387	-2.227604313	7.94595112
GSM972388	-2.376970332	8.48754707
GSM972389	-6.028778895	9.115550948
GSM972390	5.137100108	-6.713773831
GSM972391	2.318983379	-8.709658369
GSM972392	1.145325608	-4.099279867
GSM972393	2.882692675	-4.728344918
GSM972394	3.781245793	-8.942008789
GSM972395	0.926261187	-0.524742781
GSM972396	1.187957662	8.913470285
GSM972397	1.935227286	-0.099761834
GSM972398	2.413699095	0.038775236
GSM972399	2.315374576	0.472838535
GSM972400	-2.735579008	-2.690334166
GSM972401	-0.841047461	-25.13782597
GSM972402	-0.199407571	-5.832705465
GSM972403	-0.950591403	-5.437885337
GSM972404	0.524275897	0.733220969
GSM972405	-1.474665377	-4.656703462
GSM972406	-1.315656622	-9.901446896
GSM972407	2.122038613	-1.543160384
GSM972408	-0.687957478	-3.853661237
GSM972409	-0.768671921	-22.95283813
GSM972410	3.444498344	-12.96255929
GSM972411	-1.276413587	4.260873745

GSM972412	3.612327789	-3.095262104
GSM972413	2.006137132	-15.2446519
GSM972414	0.324502536	1.492001808
GSM972415	0.74485616	4.909675506
GSM972416	2.178053961	-5.263387169
GSM972417	6.431759485	-5.506704715
GSM972418	2.794936193	-0.968818195
GSM972419	3.307613827	-6.602272923
GSM972420	4.571550432	-18.19131785
GSM972421	9.957813598	-9.287443299
GSM972422	4.823275358	-22.53445799
GSM972423	1.451248146	-4.492288766
GSM972424	3.123073841	-0.777196868
GSM972425	1.159144409	-10.0759351
GSM972426	7.426052572	-12.56478875
GSM972427	2.528897034	-3.125144392
GSM972428	7.020990495	-12.33793448
GSM972429	2.603536848	-6.749848941
GSM972430	0.10452757	-8.695537157
GSM972431	1.468210017	-6.85384653
GSM972432	2.71979665	-5.545696614
GSM972433	7.855496426	-15.2341135
GSM972434	-0.685708946	3.645892127
GSM972435	2.979333022	-0.505567493
GSM972436	1.41225417	-4.336925453
GSM972437	3.024874429	-7.340060605
GSM972438	2.126497652	-15.27019145
GSM972439	9.071545503	0.88214784
GSM972440	-0.4979263	-10.19305846
GSM972441	6.333958359	-13.13357266
GSM972442	0.624464043	-8.090189108
GSM972443	-2.592350873	6.315262272
GSM972444	-3.18837447	-6.184387947
GSM972445	-2.98569968	3.087749644
GSM972447	-0.473074774	-0.608640604
GSM972449	-1.364739647	2.768051914
GSM972450	-1.882630842	8.34785056
GSM972451	-3.462560073	12.49143265
GSM972452	-3.999726339	-7.128579412
GSM972453	-3.755015896	8.805680307
GSM972454	-2.910030209	6.76227354
GSM972455	-3.668917128	-8.827858292
GSM972456	-1.758423686	7.514592798
GSM972457	-3.879508547	9.339033589
GSM972458	-4.379975231	-2.449513973
GSM972459	-3.399285593	8.98817413
GSM972460	-3.871192122	6.96584478
GSM972461	-4.380810823	11.92840963
GSM972462	-3.013955897	8.855187992
GSM972464	2.002979929	2.435932456
GSM972465	-5.041407163	7.601835195
GSM972466	-5.213368032	10.54184118
GSM972467	13.46567833	-4.06622179
GSM972468	-1.390137569	6.625966473
GSM972469	-2.406667266	6.346084772
GSM972470	-4.44010975	-13.72546805
GSM972472	-4.536866838	6.010257557
GSM972473	-5.459357213	5.984594653

GSM972474	-3.223991275	5.385549758
GSM972475	-3.548173634	8.17369266
GSM972476	4.821975096	-5.061889825
GSM972477	-3.004551783	10.04752892
GSM972478	-3.570769749	7.739461146
GSM972479	-1.55126582	-13.49282283
GSM972480	-4.496229368	9.948228534
GSM972481	-2.08735878	2.395875864
GSM972482	-0.029669407	4.900494035
GSM972483	-0.744131712	9.588527229
GSM972484	-3.490515863	5.182663856
GSM972485	-2.665304202	5.528869862
GSM972486	-2.526983225	6.982745775
GSM972487	-4.608306802	7.973098294
GSM972488	-5.37573149	7.46532073
GSM972489	-3.618532011	7.39560376
GSM972490	-2.990933649	11.1406136
GSM972491	-2.398455792	8.693895918
GSM972492	-3.969436787	8.370392805
GSM972493	0.888706648	6.91043744
GSM972494	-2.930076861	3.387501295
GSM972495	-0.094259103	5.327628742
GSM972496	-1.228826718	1.081619889
GSM972497	-1.265430642	5.125157887
GSM972498	-1.830728803	7.712672748
GSM972499	-5.641601973	3.174478934
GSM972501	0.769464155	-2.470140739
GSM972502	-1.306329178	8.156098862
GSM972503	5.361271879	-5.864707656
GSM972504	-0.444453291	-4.063330915
GSM972505	1.589750275	-1.931816645
GSM972506	2.475044142	-2.852304416
GSM972507	-4.074563375	5.165883285
GSM972508	-3.140444809	7.711036255
GSM972509	-2.897206315	11.57627607
GSM972510	-3.21591044	11.78112554
GSM972511	-4.496681115	10.01857351
GSM972512	-3.471098693	7.631568769
GSM972513	-1.736138727	8.81780476
GSM972515	-4.47878399	12.60826251
GSM972516	-3.176623982	13.57769842
GSM972517	-4.432041916	7.849051534
GSM972518	1.577396734	-9.17121223
GSM972519	3.705244391	-6.98206707
GSM972520	-2.675088972	-1.641932706
GSM972521	3.062941312	-5.030657946
GSM972522	2.09143885	-12.02610408

GEO Fersig score
16.14639024
5.327144722
12.35078387
-5.405627956
-2.282719165
25.2970487
20.74963824
-2.887624072
4.797183316
14.84890728
-9.69376543
2.076756346
1.982572594
11.15922564
-8.395031318
6.128083919
2.222857624
-12.91852869
-3.385334404
-2.77702128
-3.116407415
-8.098793748
-5.395450318
2.022711986
6.808620693
1.089164923
20.76347476
9.350214968
8.819656818
-7.543927988
-6.529673404
-4.43873305
20.84050444
4.779305171
0.155812805
17.12194283
-1.506636657
-13.38476542
-4.977721574
31.27580979
6.59100904
-11.03681789
7.830947586
1.088357375
0.997321643
11.40197033
12.62699192
-5.462154207
-13.41300454
-7.265928884
-12.09002107
-5.444562587
-3.093297488
2.997623935

0.754141977
-4.399505642
0.748295519
-6.804638206
-6.737984339
3.521005185
2.359560592
-7.474211445
-9.26722877
-2.27751454
-11.88592422
-10.00837328
-9.245086514
-10.41314586
-8.546307561
-3.660572845
-10.19033955
-13.48272649
-3.839399985
-7.101037507
-16.39474333
14.11695541
26.54409485
-12.78305717
4.545323581
2.924296099
1.191228535
0.232251533
-7.06840088
-12.67406078
-10.42542494
25.0245924
-3.303553776
-6.246480687
14.2602938
-7.28724854
-8.010146103
-5.309915813
-9.381666754
-9.014475453
-2.959179332
-1.737624727
-12.39253182
-3.019240338
17.72599887
16.92307861
-4.013274726
-13.26164735
-10.16919567
18.05903149
-6.374634187
-4.536316506
1.425557421
-6.694592921
12.11554931
16.85286693
-5.506968442
-6.725383006

-3.957004821
-0.779985806
4.589008427
8.404874723
-9.743652207
1.104695707
-1.124487215
-2.28355722
9.052730113
-0.705827132
-3.376152078
0.818940779
-12.38895028
-8.038948194
7.934635702
-1.310086079
11.8246382
4.533999474
-7.658631521
11.68433372
0.538395685
10.21921258
6.798528635
-1.060076603
-0.534677581
-4.820033894
0.739540351
-4.946264169
5.326336349
-1.628757446
-7.283692599
-12.45847752
-0.273232604
-8.814353997
-11.16089935
-6.046215835
18.202537
-2.529165954
-7.560873169
-9.429910239
-4.684914362
10.4935881
1.805527697
-8.856695032
-4.21463058
0.731386265
-6.161101857
-13.69265104
-7.19555054
2.322348096
-9.863706134
-9.524345073
-13.77970687
-9.40413452
-10.45161734
-1.856449519
-2.838273709
1.966844822

15.32741968
4.660766896
3.734811345
3.779903603
3.187900687
7.918463226
2.748156401
0.040762707
9.336112897
-3.787701572
-3.16445056
6.20169107
-1.476403642
2.369115501
-5.011201185
-4.80229778
3.683538998
-5.529106078
-8.637543851
-0.02798174
5.016235843
1.809647029
-0.413988282
2.468572758
-3.74724029
16.73122765
-2.092989498
7.749498878
-0.262717672
6.288655149
-6.19080787
4.685268733
-0.343121482
12.49246254
-8.526510451
7.712725823
-3.457959425
5.471364732
-9.796083213
-2.913887392
5.387638557
11.07504549
7.303059199
2.370831816
8.282798731
-3.172805524
-5.960294442
-0.69655461
-3.706812864
-6.366862096
-8.384273405
4.864606446
9.982788855
-7.605109696
8.663375245
-6.948152222
2.467215438
-0.08508711

1.485445747
5.505344857
-5.809207842
-15.92586602
8.22582264
9.884352359
-4.717550157
-6.62280524
1.718415612
-9.288204628
-1.840857398
-7.674937585
-5.607399065
-14.00578571
-8.738969765
-8.985090168
-0.535694047
-14.46904483
-11.93426099
-12.39581422
22.18511243
0.015218168
0.352294023
-3.929608552
6.642050137
11.32424937
6.897922599
-4.300532471
5.344971372
-1.904274723
13.09448463
-4.811725257
-4.199326793
15.86852143
-1.736757512
13.82842128
15.19382637
-0.9077099
-3.548680833
-1.275234767
-5.485136391
-6.069999531
-2.307263571
-5.549073406
3.519189147
1.92604967
0.063943106
2.143267155
-3.151623795
6.870626767
11.73449965
22.60976585
11.26381005
-9.149537012
4.726807567
1.211987556
-7.842405212
5.529990413

5.392764466
15.53520862
-0.042549042
-0.890297189
3.583389919
-0.465384615
-3.069854061
11.17608764
-2.404964771
8.170887818
14.84223986
-13.33185122
2.396592481
12.10760938
0.357449623
11.42698224
0.199995511
-0.398602843
-1.921777785
-4.951456241
6.981614949
-8.121577586
-7.486262157
-9.001309091
0.053462612
9.890808466
-4.997239574
-1.393307164
-7.045651609
1.093739006
3.87939948
-6.429326581
8.526803105
0.999888987
2.404808653
6.499313783
3.658023652
-2.310014753
7.826656947
-3.765461945
-5.586126599
29.5480232
-0.132732849
-7.478490896
-1.302449642
-7.779775742
-5.247326052
0.238274575
-2.747018217
-4.002292841
-14.88267007
0.673709731
-5.873776319
8.866560058
-1.738939513
-2.786753204
-15.06797555
-4.102675987

9.364983537
-7.914600233
-8.133790036
-5.059667214
-5.95698023
0.577088786
0.680789106
-0.375476979
-8.427354817
11.1552708
18.44687624
-4.319135008
2.509951057
12.42817837
-4.896182514
-5.711285158
18.29897158
16.387852
14.72717764
-5.464446579
0.265752064
-1.545303023
10.52548562
3.613493224
-8.941272151
0.349539314
-0.933761662
-4.792655912
-0.784033685
-2.87190334
-8.724553687
5.433312736
-3.107006244
2.323221647
1.347595242
5.827044442
7.134929832
6.20295069
1.816478126
-9.207777093
10.5559555
7.654975076
-3.781613133
-10.50713595
-3.99936093
0.653408126
-6.434879551
3.382655659
-7.087381784
-0.703303975
18.65827917
7.230153334
-1.340680825
3.970955317
1.383830187
-6.553769975
-7.021005332
-3.818995874

-2.369249619
-12.07638528
0.126264863
15.88981665
14.99701512
3.651687519
3.785356114
12.60311594
-6.471577569
-5.751191768
-10.2078574
-4.553055459
1.345912155
6.35201001
7.321896818
13.15645146
-0.296455965
-1.9668307
1.429911765
1.62441111
-5.959989887
-3.757018215
21.28017353
3.128739428
9.865044899
14.43984084
18.49760257
-6.176065711
-10.86866976
-9.554353403
-3.713039704
-12.90991949
-2.957734831
-4.118381859
-6.511535858
29.69196892
-9.358914549
-3.720188742
-11.02693098
-11.83176863
-14.80901291
-10.80761316
11.49527384
-4.976146948
-4.923328854
-8.505399607
-8.211023084
23.1553329
-8.031711049
-13.73551503
14.1483587
6.392594429
-0.016713314
4.075675944
9.433175272
26.91443286
1.834084604
8.185121076

2.277186243
1.573981605
14.4632835
2.059317237
1.779903379
7.079239809
20.83589292
-3.279405397
0.429439708
-3.290135243
4.794487411
9.070456666
3.697878821
-0.090304634
5.791710464
22.94479653
4.08549847
24.92167709
0.406527545
-0.697106285
14.83021597
5.36796645
13.82869015
3.515588463
-7.950096075
-6.290202485
15.11270457
27.83000364
-13.50425124
-13.38798206
3.437653297
2.236148955
0.920362787
-0.735728579
-5.922185354
20.69430154
-6.441200465
-5.951153841
-0.050959139
4.21094111
17.89902362
5.122299335
13.94002786
10.3373428
-3.273695996
7.403104291
-6.975117837
-3.634765141
-0.184654021
3.604301203
15.8947357
21.1261266
-3.070089083
-1.969053315
12.68057535
5.845866003
-6.067544872
-4.85750532

2.486691965
-0.904903143
-9.830677083
-6.82560337
1.372793022
18.31891883
-14.68002081
8.047754219
4.714254052
0.587763611
-8.334348893
12.69770496
-0.10573757
13.46972333
2.115848303
-4.538992741
-3.764688097
-2.304184614
-3.188151506
-2.068526321
14.38293012
3.827591162
-4.998395283
3.069918285
-0.485900782
1.09191636
5.214040558
-4.754416213
2.121660618
9.364433225
2.817457408
-2.832958128
-5.725875798
0.070025209
-2.240393333
-3.854682464
0.826482622
3.012114464
12.46820672
0.617807922
5.056928585
2.284895415
9.562943422
3.514817569
2.710471905
3.815042382
-0.002089329
1.072613149
9.052251154
-1.105682396
6.222241816
15.97776887
8.06567563
6.314140955
5.476161692
6.576487975
15.25103128
11.35216405

3.585534279
22.17233295
9.53640392
28.28463461
6.254936258
1.94516898
16.09412592
9.325289607
31.32441495
11.92464216
8.902081447
4.879442702
4.397816783
-0.943215917
10.54208089
2.863840069
7.281815563
3.306065996
3.229071646
2.784661949
-1.832743514
-0.233363445
1.409405936
13.6969612
-1.054627325
4.141720712
7.257097224
10.10945532
5.347360977
0.641376019
11.60579624
7.531774591
11.91712109
3.841985988
-3.135694333
1.308461204
-3.140053214
6.52057682
1.711406401
5.041666411
-5.054035582
-1.582622986
-4.362038071
-14.52765262
-10.62338325
-4.164647587
2.814512296
-7.002791045
-5.82708817
-10.40669728
-10.30258329
-7.648103222
-12.19576396
5.546130086
-0.133651924
-1.977020061
3.477457676
-8.286506304

-8.130076546
-10.34756668
-8.533022949
-4.878519782
0.979416536
-7.287853712
-1.315215894
-6.341206414
4.524882585
-6.006566194
-6.113353161
-7.117154316
-7.527119144
-9.086693619
-8.375050632
0.615460852
-4.109165176
-8.482211793
5.730714299
-5.186059516
-5.306686679
-2.043250311
-2.905992231
-2.632676618
-4.931287261
-6.979506779
10.39987746
-4.096039352
-10.96189818
-5.122495732
-18.25577391
-4.033251655
-6.407652138
0.797398977
-5.703691839
-9.404359047
-10.75587967
-6.984319153
-2.427953723
13.88101724
-6.059890089
-8.712982907
-10.62423036
-2.671237877
-0.545670558
-8.978130516
-11.00171644
-4.415845554
-12.75584423
-5.55752605
-5.427286728
6.17164738
-3.933833933
-15.09247008
1.305595113
-1.338298411
-6.941996491
-3.34829729

1.33655144
-4.487669821
-11.31104036
2.009439052
-8.016663689
-4.404755578
-7.604013155
2.485959694
-15.29565785
-15.17257674
-15.6985864
1.460142791
-14.12224869
-10.90138663
-8.09181495
-17.76315957
-12.46461942
-2.061255792
-13.42262221
-15.90707644
-4.221042179
-13.10376417
-9.527910545
-9.021728511
-10.45144488
-13.37759852
-8.941395626
-9.938241247
-5.730684097
-14.0655539
-12.4877879
-7.85121979
-13.25671483
1.434592387
-8.967933771
-8.480782021
-5.780367678
-10.77246015
-8.472767066
-13.20762798
0.910458517
-10.80276516
-12.99282233
-0.068046485
-11.40583123
-13.23281495
-10.20068736
-10.07933339
-9.406967691
-11.36369419
-11.86509826
1.869910261
-12.66670601
-14.75908674
-15.33381836
9.39442185
-4.461759324
4.39698208

-1.664734495
-6.357462021
2.0975392
3.865893115
-3.347990799
26.66608699
-1.516839778
21.92347762
-5.878752155
-1.388317581
-7.703923784
12.35882705
6.18847999
17.82251774
-9.561765316
15.43615077
1.153407319
-2.615029657
2.117043264
-8.201479182
-1.014840394
5.674226292
1.374840725
8.591933818
0.990687012
-5.184157876
12.40542164
1.731790045
-4.356056711
-4.83527232
1.556506437
19.29153421
-7.451257002
-10.17355543
-10.8645174
-15.14432984
11.85087394
11.02864175
5.244605475
7.611037593
12.72325458
1.451003968
-7.725512623
2.03498912
2.374923859
1.842536041
-0.045244842
24.29677851
5.633297894
4.487293934
-0.208945072
3.182038086
8.585790274
3.665198997
3.16570376
22.18416621
16.40705763
-5.537287333

6.707589893
17.25078903
-1.167499272
-4.164819346
7.44144113
11.9384642
3.763754389
9.90988675
22.76286828
19.2452569
27.35773334
5.943536912
3.900270709
11.23507951
19.99084132
5.654041426
19.35892497
9.353385789
8.800064727
8.322056547
8.265493264
23.08960993
-4.331601073
3.484900514
5.749179623
10.36493503
17.3966891
8.189397663
9.695132162
19.46753102
8.71465315
-8.907613145
2.996013477
-6.073449325
0.13556583
-4.132791561
-10.2304814
-15.95399272
3.128853073
-12.5606962
-9.672303748
5.158941164
-9.273016484
-13.21854214
-1.930461258
-12.38745972
-10.8370369
-16.30922046
-11.86914389
-0.432952527
-12.64324236
-15.75520921
17.53190012
-8.016104043
-8.752752038
9.285358303
-10.54712439
-11.44395187

-8.609541033
-11.72186629
9.883864921
-13.0520807
-11.31023089
11.94155701
-14.4444579
-4.483234643
-4.930163442
-10.33265894
-8.673179718
-8.194174064
-9.509729
-12.5814051
-12.84105222
-11.01413577
-14.13154725
-11.09235171
-12.33982959
-6.021730792
-6.317578156
-5.421887844
-2.310446607
-6.390588529
-9.543401551
-8.816080907
3.239604894
-9.46242804
11.22597953
3.618877624
3.52156692
5.327348558
-9.24044666
-10.85148106
-14.47348239
-14.99703598
-14.51525462
-11.10266746
-10.55394349
-17.0870465
-16.7543224
-12.28109345
10.74860896
10.68731146
-1.033156266
8.093599258
14.11754293

Table S6. Fersig score of different databases

samples	Liu PC1 of genes A	Liu PC1 of genes B	Liu Fersig score
Patient107	-23.80745186	-21.47483195	-2.332619905
Patient163	4.603320171	0.428653169	4.174667002
Patient96	0.933685419	0.687551842	0.246133577
Patient83	0.41275053	-2.641835437	3.054585966
Patient121	2.792776258	2.815565443	-0.022789185
Patient48	-2.645320039	-2.653624975	0.008304935
Patient94	1.60283282	1.136807624	0.466025196
Patient117	3.074452009	3.835144862	-0.760692854
Patient146	1.053291252	1.406463501	-0.353172249
Patient99	0.303442657	3.587045674	-3.283603017
Patient185	1.382480485	1.755067352	-0.372586867
Patient78	1.789925535	3.474724229	-1.684798694
Patient201	1.943360436	-0.442985813	2.386346249
Patient112	1.783808945	2.267832144	-0.484023199
Patient24	1.61770294	1.247336656	0.370366284
Patient187	1.071112201	0.894927489	0.176184712
Patient79	3.041668211	3.066675126	-0.025006915
Patient181	2.196966207	1.849659937	0.34730627
Patient8	0.444599993	-0.256364939	0.700964933
Patient40	-0.971389054	-1.580215117	0.608826063
Patient11	-0.2528136	0.531041628	-0.783855228
Patient34	5.062675306	-0.261274943	5.323950249
Patient72	1.913935463	0.732770315	1.181165148
Patient27	0.406591344	0.100316383	0.306274961
Patient38	-0.038110319	-0.426205025	0.388094706
Patient167	2.758938477	2.851642142	-0.092703665
Patient67	-1.450615964	-3.245409576	1.794793612
Patient193	2.553249606	2.24702318	0.306226427
Patient170	0.829382499	0.964034115	-0.134651616
Patient140	-0.98296165	-0.696718896	-0.286242753
Patient191	-0.097097601	2.372174314	-2.469271914
Patient98	1.794997348	1.93847633	-0.143478982
Patient42	-0.603488467	0.1744312	-0.777919667
Patient183	-2.47838733	-4.088553122	1.610165793
Patient205	0.391474326	2.760480186	-2.369005861
Patient82	0.132335029	-2.231214101	2.36354913
Patient45	0.071840621	3.097589103	-3.025748481
Patient116	3.655870399	0.925544728	2.730325671
Patient206	3.864585585	2.971417947	0.893167638
Patient35	1.674020015	1.328567067	0.345452948
Patient7	1.216125365	0.566764125	0.64936124
Patient144	-1.65378019	-3.528023601	1.87424341
Patient100	1.19096903	4.351230409	-3.160261379
Patient141	-2.418873163	-1.805382651	-0.613490512
Patient47	1.845747249	-1.054179787	2.899927037
Patient105	-3.977244541	-3.614320486	-0.362924056
Patient162	-1.140134543	-0.298694532	-0.841440011
Patient33	1.806144436	2.685720564	-0.879576128
Patient62	1.454475477	3.238365037	-1.78388956
Patient155	0.266507263	0.646653726	-0.380146464
Patient165	-0.163396392	0.591365087	-0.754761479
Patient56	3.156974918	2.538536897	0.618438021
Patient126	1.032414905	1.928625853	-0.896210949
Patient37	0.885131038	2.06995457	-1.184823532
Patient63	0.074084714	-1.782649768	1.856734481

Patient142	-1.996396708	-0.285794148	-1.71060256
Patient169	0.116358406	0.573725974	-0.457367568
Patient18	0.809596044	-0.463951832	1.273547876
Patient61	0.989425751	-6.157680499	7.14710625
Patient58	-2.373672222	-1.769546122	-0.604126101
Patient4	0.244497144	0.343162139	-0.098664995
Patient189	2.303382586	1.249142973	1.054239613
Patient196	1.062229884	0.424820022	0.637409862
Patient31	1.315893821	0.412302491	0.903591329
Patient10	1.341338084	-0.155986767	1.497324851
Patient197	0.751217163	0.123655566	0.627561598
Patient20	-1.366353705	2.659680346	-4.026034051
Patient166	-0.705025849	-0.603426611	-0.101599238
Patient149	0.535824092	4.064162713	-3.52833862
Patient200	4.26668939	2.580767068	1.685922322
Patient23	1.87754961	0.542128147	1.335421463
Patient87	0.016382122	0.607227189	-0.590845067
Patient44	-3.844833739	-1.036468702	-2.808365036
Patient145	0.98245577	1.56928548	-0.586829711
Patient135	-1.847966361	0.104534324	-1.952500684
Patient132	0.764867513	2.667458786	-1.902591274
Patient204	2.772685234	1.613781357	1.158903877
Patient134	0.877819315	0.949226027	-0.071406713
Patient77	0.314650879	2.010746607	-1.696095728
Patient184	-0.774963192	0.522513402	-1.297476594
Patient32	-0.34642393	-0.616998341	0.270574411
Patient108	-13.38330596	-14.28659754	0.903291588
Patient137	-2.162367978	2.292834442	-4.45520242
Patient51	-6.820141305	-2.217801681	-4.602339624
Patient133	-1.939638529	0.509636183	-2.449274712
Patient143	-32.73298265	-32.64521923	-0.087763423
Patient159	1.721914672	1.682767075	0.039147597
Patient195	1.296482828	1.308847624	-0.012364797
Patient25	3.208899635	1.823223897	1.385675738
Patient131	-4.473734865	-2.282169814	-2.191565051
Patient15	1.339210945	-0.397970588	1.737181532
Patient154	4.644832971	0.51381743	4.131015541
Patient106	0.061858344	1.047281109	-0.985422766
Patient17	0.657101761	1.958406443	-1.301304682
Patient130	1.591998423	1.47439849	0.117599934
Patient156	2.239646316	1.957140312	0.282506005
Patient172	2.542570493	2.519602529	0.022967964
Patient150	0.530366057	0.285899604	0.244466453
Patient173	1.835108623	1.937728116	-0.102619493
Patient203	2.652659229	1.99624396	0.656415269
Patient21	-0.45584902	0.190779717	-0.646628737
Patient125	-2.2709535	0.972462635	-3.243416135
Patient6	0.494403283	0.770109375	-0.275706093
Patient158	1.748657638	1.109975492	0.638682146
Patient188	1.563543324	-0.620622468	2.184165792
Patient147	-1.358950435	-1.159199734	-0.199750701
Patient36	0.869128135	-0.935914417	1.805042552
Patient1	-0.307810205	-1.991628532	1.683818327
Patient102	1.751691564	1.893929239	-0.142237676
Patient75	-2.681846211	2.572965989	-5.254812199
Patient168	-0.183834071	0.54772504	-0.731559111
Patient30	-1.190009603	0.015312335	-1.205321938
Patient148	0.732437992	0.435434977	0.297003014

Patient14	2.179404815	1.953417323	0.225987492
Patient9	1.762107117	-0.15093356	1.913040677
Patient73	1.551758102	0.21521774	1.336540362
Patient86	0.059454268	0.968457244	-0.909002977
Patient127	3.005069489	1.434361698	1.570707791
Patient13	2.629219031	0.808248371	1.820970659
Patient179	-6.80245096	-7.997613106	1.195162146
Patient22	0.601541363	-0.422716544	1.024257906

OS	Dead	BRgroup
47	1	CR/PR
173	1	PD/SD
913	0	CR/PR
71	1	PD/SD
1306	0	CR/PR
53	1	PD/SD
40	1	PD/SD
295	1	PD/SD
612	0	CR/PR
921	0	CR/PR
203	0	PD/SD
356	1	PD/SD
167	1	PD/SD
521	1	PD/SD
137	1	PD/SD
617	0	CR/PR
849	0	PD/SD
476	0	CR/PR
1005	1	PD/SD
468	1	PD/SD
160	1	PD/SD
1691	0	CR/PR
395	0	PD/SD
431	1	PD/SD
343	1	PD/SD
282	1	PD/SD
136	1	PD/SD
659	0	PD/SD
347	0	PD/SD
351	1	PD/SD
658	0	CR/PR
683	1	PD/SD
1027	0	CR/PR
572	1	PD/SD
392	1	PD/SD
131	1	PD/SD
1666	0	CR/PR
118	1	PD/SD
90	1	PD/SD
968	1	PD/SD
269	1	PD/SD
662	0	CR/PR
938	1	CR/PR
875	0	CR/PR
937	0	PD/SD
521	1	CR/PR
201	1	PD/SD
1679	0	CR/PR
1001	0	CR/PR
475	0	PD/SD
75	0	PD/SD
122	1	PD/SD
992	0	CR/PR
275	1	PD/SD
178	1	PD/SD

996	0	PD/SD
543	0	CR/PR
130	1	PD/SD
987	0	PD/SD
303	1	PD/SD
600	1	CR/PR
718	0	CR/PR
81	1	PD/SD
426	1	PD/SD
1139	1	CR/PR
960	0	CR/PR
242	1	PD/SD
205	1	CR/PR
497	1	PD/SD
512	1	PD/SD
975	0	PD/SD
1263	0	CR/PR
922	0	CR/PR
833	0	CR/PR
628	0	CR/PR
739	0	CR/PR
819	0	CR/PR
590	1	PD/SD
444	1	PD/SD
513	0	CR/PR
53	1	PD/SD
165	1	PD/SD
772	0	CR/PR
1547	0	CR/PR
370	1	PD/SD
630	0	PD/SD
288	0	PD/SD
895	0	CR/PR
1096	0	CR/PR
833	0	CR/PR
149	1	CR/PR
635	0	PD/SD
780	0	CR/PR
686	1	PD/SD
192	1	PD/SD
87	1	PD/SD
272	0	PD/SD
685	0	CR/PR
42	1	PD/SD
626	1	PD/SD
906	0	CR/PR
978	0	CR/PR
687	1	PD/SD
194	0	CR/PR
74	1	PD/SD
960	0	CR/PR
451	1	PD/SD
283	1	PD/SD
785	0	CR/PR
891	0	CR/PR
623	0	CR/PR
213	1	PD/SD
792	0	CR/PR

186	1	PD/SD
963	0	PD/SD
846	1	PD/SD
1327	0	PD/SD
866	0	CR/PR
58	1	PD/SD
653	0	CR/PR
253	1	PD/SD

Table S6. Fersig score of different databases

samples	Vanallen PC1 of genes A	Vanallen PC1 of genes B
Pt1.baseline	-0.361057658	-0.99009198
Pt2.baseline	-0.070806538	-0.703963262
Pt4.baseline	0.941912746	1.025178075
Pt5.baseline	2.832124426	-4.772701248
Pt6.baseline	-1.043321579	0.62301097
Pt7.baseline	-1.333143924	2.390269156
Pt8.baseline	-1.416292151	1.088585952
Pt9.baseline	0.635362935	-0.290027741
Pt10.baseline	0.468505343	2.149579516
Pt12.baseline	-1.921825859	1.979360996
Pt13.baseline	0.612200365	0.286403325
Pt14.baseline	-0.35186344	1.67918879
Pt15.baseline	-1.869728786	0.632739384
Pt19.baseline	-1.334422477	-0.053248537
Pt20.baseline	-3.756047795	2.026191197
Pt22.baseline	0.915378015	-0.654642908
Pt23.baseline	-3.136455353	2.979018214
Pt25.baseline	-3.591950585	3.947589642
Pt28.baseline	-2.571244128	4.599635325
Pt29.baseline	-1.754614963	2.899474904
Pt31.baseline	1.653638057	-3.608233836
Pt32.baseline	3.293402175	-0.264777681
Pt35.baseline	3.45491312	-4.540842878
Pt37.baseline	2.299128939	-3.606734371
Pt38.baseline	2.483781707	-3.876667891

Vanallen Fersig score	Overall.Survival	Dead	BRgroup
-0.629034323	607	1	PD
-0.633156723	927	0	CR/PR
0.083265329	948	0	CR/PR
-7.604825674	439	0	CR/PR
1.666332548	882	0	CR/PR
3.72341308	662	1	PD
2.504878103	NA	0	CR/PR
-0.925390676	1054	0	CR/PR
1.681074173	387	0	PD
3.901186854	327	1	PD
-0.32579704	917	0	CR/PR
2.03105223	54	0	PD
2.50246817	980	1	CR/PR
1.28117394	1060	0	CR/PR
5.782238992	337	1	PD
-1.570020923	182	1	PD
6.115473567	103	1	PD
7.539540227	262	1	PD
7.170879452	439	1	CR/PR
4.654089867	269	1	PD
-5.261871893	704	0	PD
-3.558179856	171	1	PD
-7.995755998	427	0	CR/PR
-5.90586331	364	0	CR/PR
-6.360449598	448	0	CR/PR
